# Causal relationship between serum metabolites and juvenile idiopathic arthritis: a mendelian randomization study

**DOI:** 10.1186/s12969-024-00986-0

**Published:** 2024-05-09

**Authors:** Han Zhang, Xiao Ma, Wanlu Liu, Ze Wang, Zian Zhang, GuanHong Chen, Yingze Zhang, Tianrui Wang, Tengbo Yu, Yongtao Zhang

**Affiliations:** 1 Department of Orthopedics, Affiliated Hospital of Qingdao University, Qingdao, China; 2 Shanxian Central Hospital, Heze, Shandong Province China; 3 Department of Neurology, Qingdao Haici Hospital, Qingdao, China; 4 Department of Orthopedics, The Third Hospital of Hebei Medical University, Shijiazhuang, China; 5 Qingdao Municipal Hospital, Qingdao, China

**Keywords:** Serum, Metabolites, Juvenile idiopathic arthritis, Mendelian randomization, Causality

## Abstract

**Background:**

Juvenile Idiopathic Arthritis (JIA) is a condition that occurs when individuals under the age of 16 develop arthritis that lasts for more than six weeks, and the cause is unknown. The development of JIA may be linked to serum metabolites. Nevertheless, the association between JIA pathogenesis and serum metabolites is unclear, and there are discrepancies in the findings across studies.

**Methods:**

In this research, the association between JIA in humans and 486 serum metabolites was assessed using genetic variation data and genome-wide association study. The identification of causal relationships was accomplished through the application of univariate Mendelian randomization (MR) analysis. Various statistical methods, including inverse variance weighted and MR-Egger, were applied to achieve this objective. To ensure that the findings from the MR analysis were trustworthy, a number of assessments were carried out. To ensure the accuracy of the obtained results, a range of techniques were utilised including the Cochran Q test, examination of the MR-Egger intercept, implementation of the leave-one-out strategy, and regression analysis of linkage disequilibrium scores. In order to identify the specific metabolic pathways associated with JIA, our primary objective was to perform pathway enrichment analysis using the Kyoto Encyclopedia of Genes and Genomes.

**Results:**

Two-sample summary data MR analyses and sensitivity analyses showed that five metabolites were significantly causally associated with JIA, including two risk factors—kynurenine (odds ratio [OR]: 16.39, 95% confidence interval [CI]: 2.07-129.63, *p* = 5.11 × 10^− 6^) and linolenate (OR: 16.48, 95% CI: 1.32-206.22, *p* = 0.030)—and three protective factors—3-dehydrocarnitine (OR: 0.32, 95% CI: 0.14–0.72, *p* = 0.007), levulinate (4-oxovalerate) (OR: 0.40, 95% CI: 0.20–0.80, *p* = 0.010), and X-14,208 (phenylalanylserine) (OR: 0.68, 95% CI: 0.51–0.92, *p* = 0.010). Furthermore, seven metabolic pathways, including α-linolenic acid metabolism and pantothenate and CoA biosynthesis, are potentially associated with the onset and progression of JIA.

**Conclusion:**

Five serum metabolites, including kynurenine and 3-dehydrocarnitine, may be causally associated with JIA. These results provide a theoretical framework for developing effective JIA prevention and screening strategies.

**Supplementary Information:**

The online version contains supplementary material available at 10.1186/s12969-024-00986-0.

## Introduction

Juvenile idiopathic arthritis (JIA) encompasses a variety of intricate and diverse disorders characterized by persistent inflammation, mainly observed in the synovial membranes and this chronic inflammatory process significantly heightens the risk of degenerative changes occurring in the osteocartilaginous tissues [1]. The prevalence of JIA is 3.8–400 per 100,000 proportion [1]. JIA, a condition primarily affecting children under the age of 16, is characterized by symptoms such as swelling, pain, and restricted joint movement that persists for a minimum of 6 weeks, according to research conducted by the International League of Associations for Rheumatology [2, 3]. In the early stage, there may be severe symptom, including macrophage activation syndrome and synovitis, potentially leading to multi-organ damage [4]. Cartilage damage and bone erosion may occur as the disease progresses, leading to joint deformities and functional impairment, affecting the quality of life and increasing morbidity.JIA is a condition whose development is thought to be influenced by various factors, such as genetic and environmental factors, as well as infections and it is believed that these factors can trigger inflammatory responses and lead to the onset of autoimmune disorders [5–7]. Nonetheless, the influence of serum metabolites on disease pathogenesis is unclear [8]. Therefore, the early identification of changes in serum metabolites can help prevent JIA.

Metabolomics has emerged as a burgeoning field that focuses on the detection, analysis, and measurement of naturally occurring small-molecule metabolites in biological samples and this field holds the potential to enhance diagnoses by identifying biomarkers and pathway components, as well as analyzing changes in serum metabolite levels [9–11]. JIA is associated with changes in serum metabolites. Currently, only a limited number of molecular, immune, and clinical markers with JIA have been suggested in these studies [12, 13]. For instance, calprotectin (also known as MRP8/14 and S100A8/A9) is useful for diagnosing JIA [14]. In turn, circulating levels of 25-(OH)D appear to have no detectable effect on the incidence of JIA [15]. In particular, the occurrence of JIA is linked to the activation of endothelial cells, the activation of macrophages, heightened levels of pro-inflammatory cytokines (such as IL-6, IL-10, and IFN-γ), and increased levels of adipokines [16]. These findings highlight the close connection between lipid profiles, inflammatory responses, and the pathogenesis and progression of autoimmune diseases [17]. High-mobility group box 1 and matrix metalloproteinase 3 are markers of JIA [18]. Population-based observational studies have identified various metabolites associated with JIA but are influenced by potential confounding factors or limited by sample size. Therefore, large studies are needed to identify and characterize serum biomarkers that are clinically useful for the early diagnosis of JIA.

The Mendelian randomization method utilizes genetic variants, particularly single nucleotide polymorphisms (SNPs), that are strongly linked to exposure factors, serving as instrumental variables (IVs) and these instrumental variables are then used to estimate the causal effects of exposure factors on health outcomes [19]. Moreover, MR eliminates potential confounders and reverses causation effects, making it somewhat similar to randomized controlled trials and capable of evaluating genetic correlations across complex diseases [20]. MR studies based on genome-wide association study (GWAS) datasets utilize genetic variation data as IVs to estimate causal effects. This MR study inferred the causal relationships of 486 serum metabolites (exposure factors) with JIA (outcome), thus providing a basis for identifying JIA biomarkers and metabolic pathways.

## Materials and methods

### Experimental design

**Fig. 1:**
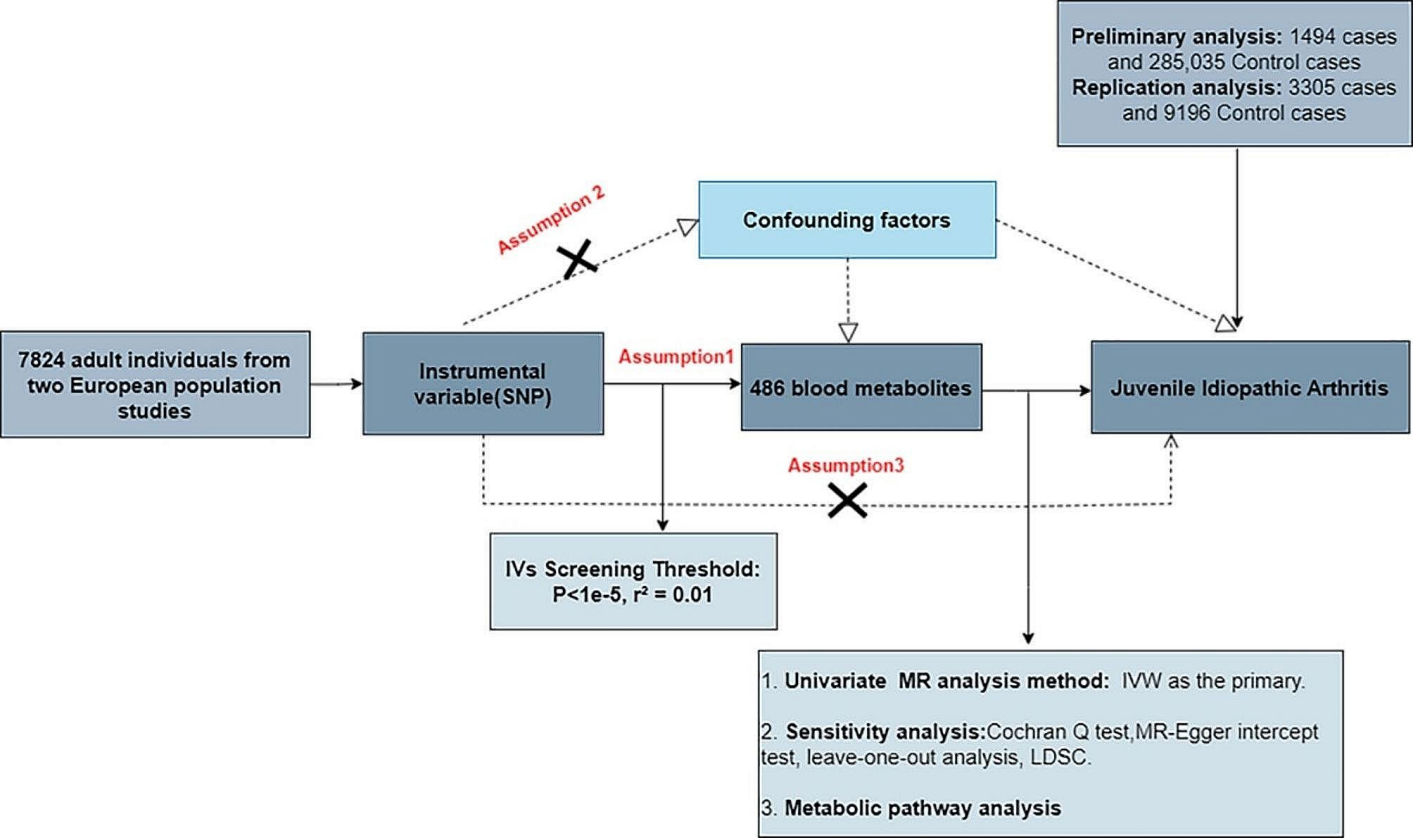
Flowchart of the study design

MR analysis was performed using publicly available GWAS catalog and FinnGen datasets to investigate the causal relationship of 486 serum metabolites (exposure factors) with JIA (outcome). Three fundamental assumptions were met to ensure the reliability of inferences [21, 22]: (1) IVs were strongly correlated with serum metabolites; (2) IVs were not affected by confounders of JIA, such as body mass index (BMI), body fat percentage, body weight, smoking, and inflammatory bowel disease; (3) there was no genetic pleiotropy, i.e., the effects of IVs on JIA were mediated solely by the exposure. Therefore, it is currently impossible to completely exclude SNPs related to outcomes. The extent of bias can be assessed by evaluating the magnitude of horizontal pleiotropy. Additionally, considering the possibility of non-reproducibility of GWAS results [23], two sets of JIA genetic variation data were used in reproducibility analysis. Combining the results of two MR studies increases confidence in causal estimates (Fig. 1).

### Source of GWAS data on JIA

Data on JIA were obtained from the FinnGen consortium release 9 [24], which includes 286,529 participants of European descent (1,494 JIA patients and 285,035 healthy controls). The phenotype of interest is JIA. The genetic variation data for JIA used in the replication analysis were sourced from the GWAS catalog dataset [25] containing 12,501 participants of European ancestry (3,305 JIA patients and 9,196 healthy controls) (Accession Code GCST90010715). The JIA diagnostic criteria used in this study adhere to the International Classification of Diseases, 10th Edition (ICD-10) (https://icd.who.int/browse10/2016/en#). Ethical approval and informed consent were waived because this study was based on previously published articles and open-source databases.

We used genome-wide association summary datasets containing 486 human serum metabolites [26], Among these metabolites, 177 had undisclosed biochemical characteristics, while 309 were categorized into eight distinct biochemical groups, namely amino acids, peptides, energy, cofactors and vitamins, lipids, xenobiotics, carbohydrates, and nucleotides. Ultimately, the GWAS dataset consisted of roughly 2.1 million SNPs, collected from the KORA and TwinsUK datasets, which encompassed a total of 7,824 adult subjects.

### Identifying appropriate IVs for screening

To ensure the validity and reliability of the results, we met three key assumptions that served as the basis for selecting IVs. We established a threshold of *p* < 1 × 10^− 5^ for selecting SNPs significantly associated with the exposure. The strength of SNPs was evaluated by calculating the F statistic and in order to avoid any potential bias resulting from weak instruments, SNPs with an F statistic value below 10 were excluded from the analysis [27, 28]. F was calculated using the following equations:

$${R}^{2}=\frac{2\times (1-\text{M}\text{A}\text{F})\times \text{M}\text{A}\text{F}\times {\beta }}{SD}$$

The minor allele frequency (MAF), effect size (β), and standard error (SD) of β play important roles in the analysis.

$$F=\frac{{R}^{2}\times (N-1-K)}{(1-{R}^{2})\times K}$$

The exposure GWAS study’s sample size, denoted by N, the number of IVs, denoted by K, and the degree to which the IVs explain the exposure (as measured by R², the coefficient of determination in the regression equation) are considered for analysis.

### MR analysis

Genetic variant data were sourced from the FinnGen dataset. Given the advantages of the inverse variance weighted (IVW) method in testing efficiency and statistical power, we selected it as the primary method for establishing causal relationships. Other methods were employed, including simple mode, MR-Egger, weighted median, and weighted model. The IVW method calculates the Wald ratio for each IV using inverse variance weights and combines the results through meta-analysis. The weight of each IV was determined by the inverse of its effect variance, i.e., larger studies with smaller standard errors have more weight than smaller studies. This weight allocation approach reduces inaccuracies in estimating combined effects. The slope corresponds to the causal impact of the exposure factor on the outcome. The variance of the effect can be estimated using a fixed or random effects model.

The desired objective of establishing a reliable association between exposure and outcome can be accomplished by utilizing different SNPs and assuming that each genetic variant satisfies the instrumental variable assumptions, thus enabling the combination of Wald ratio estimates [29]. Nevertheless, the implementation of the IVW approach may introduce estimation bias in assessing causal effects if the instrumental variables exhibit pleiotropy and the estimation of causal effects is represented by the slope of MR-Egger regression, while the intercept corresponds to the average pleiotropic effect of a genetic variant [30]. Through the utilization of weighted models, causal effect estimations can be obtained by giving weights to each SNP, with the largest weight being considered [31].

### Sensitivity analysis

In MR studies, the accuracy of causal estimates is increased using IVs. However, this approach may also introduce IVs with pleiotropy and heterogeneity, resulting in bias. Various techniques are employed to enhance the dependability of results, which encompass the application of the MR-Egger procedure [32], adoption of the Cochran Q test [33], and implementation of the leave-one-out strategy [34]. The MR-Egger intercept test evaluates the impact of pleiotropy on the causal effect of interest; a nonzero intercept is evidence of pleiotropy. The Cochran Q test assesses heterogeneity among IVs.The approach of leaving one out investigates the impact of each IV and examines the robustness of the results by consecutively excluding each IV and determining the collective effect of the remaining IVs.

### Analysis of metabolic pathways

Metabolic pathways associated with JIA were identified using the KEGG database (https://new.metaboanalyst.ca/MetaboAnalyst/). Differentially expressed metabolites were selected based on a significance level of *p* < 0.05. This analysis was performed using MetaboAnalyst version 5.0. The metabolic pathways that showed a significance level of *p* < 0.1 are listed in Supplementary Table 2.

### Exclusion of confounding factors

In spite of the exclusion of inadequate independent variables (F < 10) and the execution of sensitivity analyses to evaluate the dependability of Mendelian randomization (MR) findings, certain independent variables can breach assumptions (2) and (3).IVs associated with potential confounders of JIA, including BMI, body fat percentage, body weight, smoking, and inflammatory bowel disease, were identified using PhenoScanner version 2 (http://www.phenoscanner.medschl.cam.ac.uk/).

### Replication analysis and meta-analysis

To increase the reliability of MR estimates, we performed a replication analysis using a second set of JIA genetic variation data obtained from the GWAS dataset. Then, we merged the findings of two MR studies to identify metabolites causally associated with JIA.

### Linkage disequilibrium score regression (LDSC) and reverse causality analysis

Once the cause-and-effect relationship between the exposure and outcome is established, LDSC becomes a valuable tool for examining the genetic association among intricate characteristics.This type of regression helps avoid overestimations and confounding from polygenicity. LDSC involves measuring the linkage disequilibrium (LD) score for each SNP to evaluate its degree of association with complex traits. This score quantifies the strength of LD between a SNP and neighboring SNPs. Further, LD analysis reduces the potential confounding effects of shared genetic factors on MR results [35].

To prevent endogeneity resulting from reverse causation and improve the reliability of MR findings, we performed a reverse causation analysis on the identified group of metabolites.

## Results

Following the screening process, a total of 8,845 SNPs directly related to 486 metabolites were identified. The range of SNPs associated with each metabolite varied from a minimum of 3 to a maximum of 413. Moreover, it is noteworthy that all of these SNPs exhibited F values exceeding the threshold of 10, thereby confirming their potential as reliable instruments for MR investigations (Supplementary Tables 1 and 2).

### MR analysis

The MR findings are presented in (Supplementary Table 3). The IVW results indicated that JIA was causally related to 24 serum metabolites. It emphasize the presence of unknown metabolites with unknown properties. Additionally, It highlights the classification of 18 metabolites into eight distinct categories. These categories include amino acids, peptides, energy, cofactors and vitamins, lipids, xenobiotics, carbohydrates, and nucleotides. The study found several notable findings regarding specific metabolites. Firstly, the metabolite kynurenine (Kyn) demonstrated a strong association with the studied condition (odds ratio [OR]: 16.39, 95% confidence interval [CI]: 2.07-129.63, *p* = 5.11 × 10 − 6). On the other hand, the metabolites 3-dehydrocarnitine (OR:0.32, 95%CI: 0.14–0.72, *p* = 0.0068), levulinate (4-oxovalerate) (OR: 0.40, 95%CI: 0.20–0.80, *p* = 0.0098), X-14,208 (phenylalanylserine) (OR: 0.68, 95%CI: 0.51–0.92, *p* = 0.010), and linolenate (OR: 16.48, 95% CI: 1.32-206.22, *p* = 0.030) were found to have significant associations as well. These findings suggest the potential involvement of these metabolites in the studied condition.

There was evidence of heterogeneity in Kyn, acetylcarnitine, and cholate; thus, their causal relationship with JIA was assessed using the IVW random effects model. IVs related to acetylcarnitine showed horizontal pleiotropy, while IVs related to other metabolites did not exhibit heterogeneity or horizontal pleiotropy (Supplementary Table 4) (Supplementary file 1).

### Confounding analysis, replicate analysis, and meta-analysis

Although 17 metabolites (excluding acetylcarnitine because of horizontal pleiotropy) passed tests for heterogeneity and horizontal pleiotropy, we further investigated the associations of IVs with other phenotypes. Based on the PhenoScanner results, we found that SNPs related to stearoylcarnitine, linolenate, 3-carboxy-4-methyl-5-propyl-2-furanpropanoate (CMPF), ursodeoxycholate, levulinate, and X-14,208 (phenylalanylserine) were not associated with confounders of JIA. However, SNPs related to Kyn, 3-dehydrocarnitine, cysteine, pantothenate, phenylalanine, N-acetylglycine, tryptophan betaine, and cholate were associated with confounding factors such as BMI, body fat percentage, body weight, smoking, and inflammatory bowel disease (Supplementary Table 5).

**Fig. 2:**
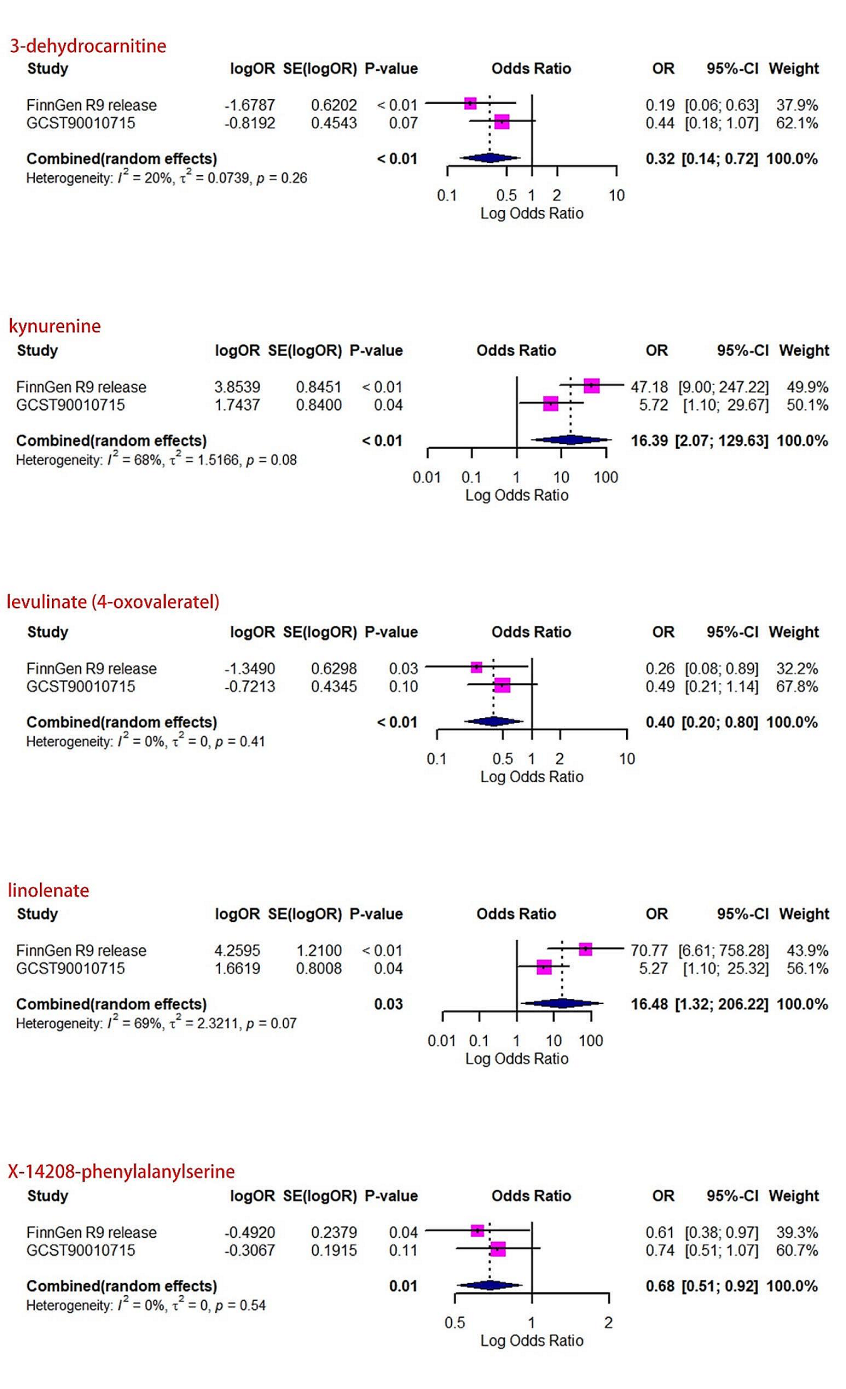
Meta-analysis of the causal associations of serum metabolites with juvenile idiopathic arthritis. OR, odds ratio; CI, confidence interval

**Fig. 3:**
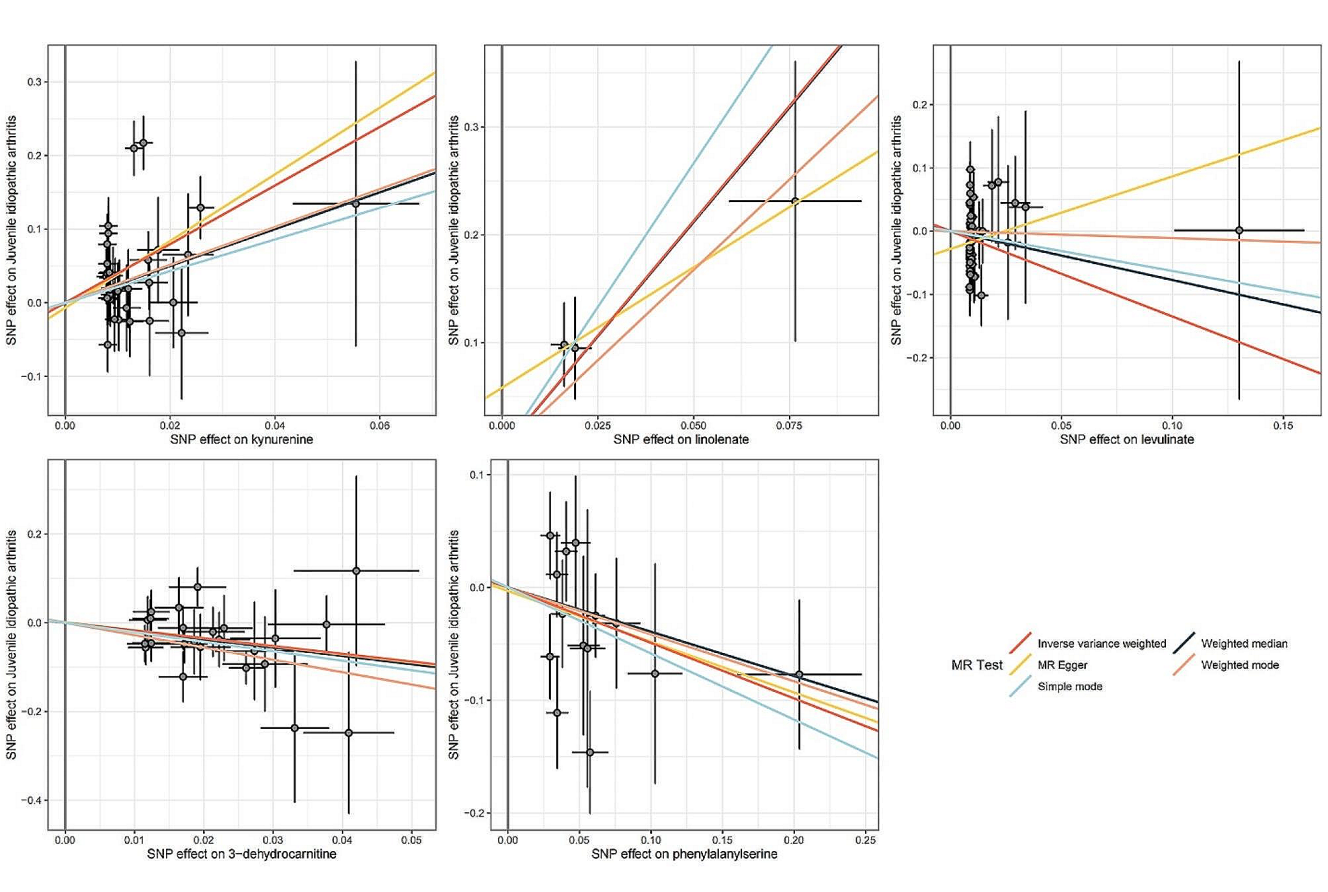
Scatter plot of five metabolites( Kyn, 3-dehydrocarnitine, levulinate, X−14,208 (phenylalanylserine), and linolenate )

After excluding SNPs associated with these confounding factors, we discovered that Kyn, 3-dehydrocarnitine, cysteine, and pantothenate were causally related to JIA, while phenylalanine, N-acetylglycine, tryptophan betaine, and cholate were not causally associated with JIA. To validate the MR analysis, we obtained independent JIA genetic data from the FinnGen dataset for replication analysis, which revealed that five metabolites had characteristics consistent with the MR analysis (Fig. 2) (Supplementary Tables 6, 7 and 8). Then, a meta-analysis was conducted to combine the results of two studies, confirming that Kyn, 3-dehydrocarnitine, levulinate, X-14,208 (phenylalanylserine), and linolenate were causally associated with JIA (Fig. 3). CMPF and ursodeoxycholate were excluded because of inconsistent directions in the two meta-analyses.

### Metabolic pathway analysis

JIA was found to have significant associations with seven metabolic pathways. These include the biosynthesis of pantothenate and Coenzyme A (CoA) (*p* = 0.002, KEGG), the biosynthesis of aminoacyl-tRNA (*p* = 0.013, KEGG), the biosynthesis of phenylalanine, tyrosine, and tryptophan (Trp) (*p* = 0.015, KEGG), the metabolism of thiamine (*p* = 0.027, KEGG), the metabolism of taurine and hypotaurine (*p* = 0.030, KEGG), the metabolism of phenylalanine (*p* = 0.038, KEGG), and the metabolism of α-linolenic acid (*p* = 0.049, KEGG).

### LDSC and reverse causality analyses

Based on LDSC analysis, there was no notable genetic correlation observed between JIA and Kyn (rg: 0.148, SE: 0.118, *p* = 0.210), levulinate (rg: 0.041, SE: 0.092, *p* = 0.654), and 3-dehydrocarnitine (rg: 0.041, SE: 0.092, *p* = 0.654). These findings indicate that the MR outcomes were not impacted by common genetic elements.

The reverse causality analysis revealed no significant genetic evidence supporting a cause-and-effect relationship between JIA and Kyn, 3-dehydrocarnitine, levulinate, X-14,208 (phenylalanylserine), and linolenate (Supplementary Table 9). This analysis helps mitigate the potential influence of environmental endogeneity on these factors.

## Discussion

To investigate the causality between JIA and 486 serum metabolites in humans, this research utilized FinnGen datasets and the GWAS catalog through a two-sample MR approach.The findings suggest that elevated levels of Kyn and linolenate increase the risk of JIA, whereas increased levels of 3-dehydrocarnitine, levulinate, and X-14,208 (phenylalanylserine) protect against this condition. Furthermore, the LDSC analysis indicated no genetic correlation of these metabolites with JIA, demonstrating that the MR analysis was reliable and unaffected by pleiotropy. Moreover, seven metabolic pathways were significantly associated with JIA, including pantothenate and CoA biosynthesis and α-linolenic acid metabolism. JIA is a condition resulting from a combination of genetic and environmental factors that subsequently induces systemic immune reactions, which means timely and precise identification and intervention play a crucial role in enhancing patient outcome [4]. In the United States, JIA affects about one in a thousand children, being the most prevalent pediatric rheumatic disease and a leading cause of disability acquired in childhood [36, 37]. This MR research has deepened our understanding of the mechanisms behind JIA, playing a significant role in the disease’s prevention and treatment.

The causal relationship between Kyn and JIA remains unclear.However, previous research has shown that kyn is a significant byproduct of Trp catabolism through tryptophan 2,3-dioxygenase (TDO) or indoleamine 2,3-dioxygenase (IDO) [38]. The ultimate metabolic product of Kyn is NAD+, which plays a crucial role in immune regulation [39]. Additionally, under certain physiological conditions, Kyn can be converted into kynurenic acid and xanthurenic acid, both of which are involved in inflammation and immunity in mammals [40, 41]. The three rate-limiting enzymes of the Trp-Kyn pathway are IDO1, IDO2, and TDO2, with IDO1 promoting inflammation in rheumatoid arthritis (RA) [42]. Because of its high homology with IDO1, IDO2 may also be implicated in the onset and progression of autoimmune arthritis [43]. Moreover, a significant increase in serum Kyn levels is associated with chronic low-grade inflammation [44].JIA is a multifactorial disease with heterogeneous manifestations, including many forms of chronic arthritis [2]. The levels of Trp-Kyn pathway metabolites are elevated in the serum, urine, and synovial fluid of RA patients [45]. Moreover, RA is correlated with increased Trp catabolism, increased Kyn concentrations, and immune cell activation in patients and animal models [46]. These data suggest that the Trp-Kyn metabolic pathway is involved in the pathogenesis of RA. Moreover, there is increased evidence of the role of Kyn metabolites in physiological and disease states. Therefore, increased Kyn levels may be implicated in the pathophysiology of JIA, as demonstrated in this study.

Although the causal relationship between linolenate and JIA is unclear, the findings suggest that linolenate increases the risk of JIA via alpha-linolenic acid metabolism. Research demonstrates that Wuwei Shexiang pill treatment has been linked to a reduction in γ-linolenic acid and other components of the linoleic acid metabolic pathway, suggesting its anti-inflammatory properties by inhibiting linoleic acid metabolism and affecting arachidonic acid metabolism [47]. Alpha-linolenic acid has multiple biological functions and is involved in endoplasmic reticulum (ER) stress and lipid metabolism. Linolenate induces ER stress by inhibiting the expression of fatty acid synthase, thereby affecting fatty acid synthesis and inflammatory immune responses [48]. Some drugs can influence the production of inflammatory mediators, including PGE2 and leukotrienes, by regulating alpha-linolenic acid and arachidonic acid metabolism [49, 50]. These data and our findings suggest that linolenate is implicated in the development and progression of JIA.

We found that three serum metabolites—3-dehydrocarnitine, levulinate, and X-14,208 (phenylalanylserine)—protected against JIA. Nonetheless, little is known about the causal relationship of these metabolites with JIA. Specifically, 3-dehydrocarnitine affects fatty acid metabolism in gout arthritis [51]. Levulinate has a genetic causal relationship with RA [52]. Additionally, 5-aminolevulinic acid (5-ALA) has anti-inflammatory and immunomodulatory properties and 5-ALA combined with sodium ferrous citrate (SFC) increases the expression and release of heme oxygenase 1 (HO-1) and its metabolites in macrophages and has been utilized in the treatment of inflammatory diseases [53–55]. These data suggest that the interaction between levulinate and JIA may be mediated by the upregulation of HO-1 by 5-ALA/SFC in macrophages. However, this hypothesis needs to be validated by experimental research. We found that the increased expression of X-14,208 (phenylalanylserine) was associated with a reduced risk of JIA. A study indicates that serum levels of threonine, phenylalanine, and leucine exhibit a positive correlation with the expression of synovial IL-1β and IL-8 in RA patients [56]. Wine-processed Curculigo orchioides (pCO)’s potential anti-inflammatory actions might be due to its modulation of the phenylalanine metabolic pathway [57]. Additionally, another study aimed at correlating serum metabolic profiles of RA patients undergoing methotrexate treatment with synovial gene expression discovered associations between serine/glycine/phenylalanine metabolism and aminoacyl-tRNA biosynthesis with TNF-α/CD3E and B cell/plasma related signatures, suggesting a role in lymphocyte regulation within the RA synovium [58].

Metabolomics, an advanced technology, provides a comprehensive means to explore variations in metabolite levels within biological frameworks, offering invaluable insights into the disruption of metabolic pathways across various diseases [59]. For instances, a study highlights the pivotal role of metabolomics in revealing that exclusive enteral nutrition can effectively trigger remission in JIA by significantly altering the microbiome and metabolome [60]. This approach has pinpointed seven critical metabolic pathways associated with JIA, encompassing the biosynthesis of pantothenate and CoA, aminoacyl-tRNA, phenylalanine, tyrosine, and tryptophan, as well as the metabolism of thiamine, taurine and hypotaurine, phenylalanine, and α-linolenic acid. These pathways play essential roles in cellular energy metabolism and the activation of inflammatory cells, highlighting their importance in the pathogenesis of diseases. Employing Ultra Performance Liquid Chromatography-Tandem Mass Spectrometry technology, research has shown that Munziq Balgam, a herbal medicine, can modulate collagen-induced arthritis (CIA) in rat models by affecting these specific pathways, including linoleic acid, alpha-linolenic acid, and the biosynthesis of pantothenate and CoA [61]. Additionally, Wuwei Shexiang pills have been observed to indirectly affect mitochondrial function and the tricarboxylic acid cycle by altering the synthesis of pantothenic acid and CoA, influencing phenylalanine metabolism [47]. Further investigations into blood metabolomics of RA rats revealed the impact of Phellodendri Amurensis Cortex, berberine, and palmatine on aminoacyl-tRNA biosynthesis, phenylalanine metabolism, tryptophan metabolism, and the biosynthesis of pantothenic acid and coenzyme A, showcasing anti-RA effect [62]. Thiamine enhances neurotransmission, muscle function, and immune response in CIA by adjusting metabolism to meet increased energy needs and reduce cellular stress, highlighting the importance of thiamine and arachidonic acid levels in CIA treatment [63]. Furthermore, research indicates that pCO treatment targeting taurine metabolism can mitigate RA inflammation and bone degradation by modulating anti-inflammatory responses and protecting against oxidative stress [57]. These studies indicate that targeted regulation of specific metabolic pathways provides a meaningful pathway for the treatment and comprehension of JIA.

This study has strengths. First, IVs and exposure factors were strongly correlated (F > 10). Second, we obtained JIA genetic variation data from multiple sources, conducted several MR analyses, and combined the results of two MR studies to enhance the confidence of MR estimates. Third, MR can eliminate confounding factors and is unaffected by reverse causality. Fourth, the results of MR studies are more robust than those of traditional observational studies. Fifth, we addressed the problem of endogeneity due to reverse causation. To address multiple comparisons, we implemented the Bonferroni correction. The significance level was adjusted to *p* < 0.00024 (0.05/486) for the analysis.

This study also has limitations. First, factors such as the Beavis effect, compensatory mechanisms (e.g., canalization), low statistical power, and genetic complexity can limit the application of MR studies [35]. Second, causal inferences were drawn from the results of MR studies and should be further validated through molecular experiment and real-world clinical studies. Third, the SNP data were obtained from European populations, limiting the generalizability of the findings. Fourth, data on age, gender, and other demographic characteristics were unavailable. Fifth, there was heterogeneity in the MR analysis of serum metabolites and JIA. Although heterogeneity was reduced to acceptable levels after removing outliers, the results should be interpreted with caution.

## Conclusion

This study utilized GWAS datasets and MR to assess the causal relationships of circulating metabolites with JIA and found that several metabolites, including Kyn, linolenate, and 3-dehydrocarnitine, were genetically associated with JIA. Thus, the study provides theoretical support for the development of early screening and prevention strategies for JIA and has significant clinical implications.

### Electronic supplementary material

Below is the link to the electronic supplementary material.

Supplementary Material 1

Supplementary Material 2

Supplementary Material 3

Supplementary Material 4

Supplementary Material 5

Supplementary Material 6

Supplementary Material 7

Supplementary Material 8

Supplementary Material 9

Supplementary Material 10

Supplementary Material 11

Supplementary Material 12

Supplementary Material 13

## Data Availability

The original contributions presented in the study are included in the article(https://www.finngen.fi/en/access_results, https://www.ebi.ac.uk/gwas/) and supplementary material, further inquiries can be directed to the corresponding authors.

## Abbreviations

JIA: Juvenile Idiopathic Arthritis
MR: Mendelian randomization
OR: odds ratio
CI: confidence interval
SNPs: single nucleotide polymorphisms
GWAS: genome-wide association study
BMI: body mass index
IVW: inverse variance weighted
KEGG: the Kyoto Encyclopedia of Genes and Genomes
LDSC: linkage disequilibrium score regression
LD: linkage disequilibrium
CoA: Coenzyme A
Kyn: kynurenine
CMPF: 3-carboxy-4-methyl-5-propyl-2-furanpropanoate
TDO: tryptophan 2,3-dioxygenase
IDO: indoleamine 2,3-dioxygenase
RA: rheumatoid arthritis
ER: endoplasmic reticulum
5- ALA: 6- 5-aminolevulinic acid
SFC: sodium ferrous citrate
HO-1: oxygenase 1
CIA: collagen-induced arthritis
pCO: wine-processed Curculigo orchioides
