# Supplementary material for: Causal relationship between serum metabolites and juvenile idiopathic arthritis: a mendelian randomization study
Source: Pediatr Rheumatol Online J. 2024 May 9;22:51. doi: 10.1186/s12969-024-00986-0 (PMC11080266; doi:10.1186/s12969-024-00986-0)
Supplement: Supplementary file 7 — Supplementary Material 7 [file 12969_2024_986_MOESM7_ESM.docx]

Dear Reviewer #1,

First and foremost, I would like to express our sincere gratitude for the meticulous review and valuable comments you provided on our manuscript, "The Causal Relationship Between Serum Metabolites and Juvenile Idiopathic Arthritis: A Mendelian Randomization Study." We are delighted to hear that you found our research interesting and believe it offers new insights into the pathophysiology of Juvenile Idiopathic Arthritis (JIA). Your suggestions are highly appreciated and have been instrumental in refining both our study and manuscript. In response to your key comments, we have made the following revisions and responses:

**1. Comment:** The authors should describe more precisely how can these changes lead to arthritis pathogenesis.

**Response:** You rightly pointed out that we needed to more precisely describe how changes in serum metabolites lead to the onset of arthritis. In the revised manuscript, we have added detailed sections (paragraphs 269-272, 289-297, and 299-324) exploring how α-linolenic acid metabolism, as well as pantothenate and CoA biosynthesis, participate in the pathogenesis of JIA by regulating immune responses, promoting or inhibiting inflammatory processes, and affecting cellular energy metabolism.

**2.Comment:** There are only a few metabolomic studies in the relevant literature, it would be better to discuss them in the paper.

**Response:** Following your suggestion, we have included a new section (paragraphs 299-324) that elaborates on metabolomics research related to JIA. This addition aims to deepen the understanding of how our results contribute to unraveling the pathogenesis of JIA.

**3.Comment:** In literature, there are studies investigating if metabolomics affects JIA symptoms, treatment success etc… I would rather to see more description about the study sample, patient characteristics and disease manifestations, disease activity and remission states.

**Response:** We acknowledge the importance of thoroughly describing the patient cohort for a comprehensive understanding of the study context and findings. Thus, in the revised draft, we have expanded on the significance of metabolomics in predicting clinical remission in JIA patients (paragraphs 301-303).

**4.Comment:** The results are noteworthy, however, interpretation of the results with the real-world would be better, and more valuable.

**Response:** We fully agree with your perspective that connecting our findings with practical clinical applications significantly enhances the value of our research. Although our current study is confined to a Mendelian randomization approach, we have addressed the importance of linking research outcomes to real-world implications in the limitations section. In future studies, we plan to conduct prospective, large-scale real-world research to increase the reliability of our findings.

We are deeply grateful for your invaluable feedback and hope that our efforts will make the manuscript more rigorous and profound, thereby contributing meaningfully to the field. We look forward to your further comments and guidance on the revised manuscript.

Warmest regards,

Yongtao Zhang.

Dear Reviewer #2,

**Comment:** Dear Authors, This is a good and interesting manuscript.

**Response:** Thank you very much for your encouraging comments on our manuscript. We are thrilled to learn that you find our work both interesting and valuable. Your positive feedback greatly motivates us to further our research in this field. We are dedicated to advancing the understanding and treatment of polyarticular Juvenile Idiopathic Arthritis (JIA) and hope our findings will indeed establish a new standard of care. We deeply appreciate your recognition of our efforts and aspire to make a significant impact in the field.

Warmest regards,

Yongtao Zhang.
