# Supplementary material for: Causal relationship between serum metabolites and juvenile idiopathic arthritis: a mendelian randomization study
Source: Pediatr Rheumatol Online J. 2024 May 9;22:51. doi: 10.1186/s12969-024-00986-0 (PMC11080266; doi:10.1186/s12969-024-00986-0)
Supplement: Supplementary file 12 — Supplementary Material 12 [file 12969_2024_986_MOESM12_ESM.pdf]

TOPEDIT

TOPEDIT SCIENTIFIC EDITING

CERTIFICATE OF ENGLISH DEVELOPMENTAL EDITING

This is to certify that the manuscript detailed below has been edited by multiple native English-speaking academic editors in TopEdit. Neither the research content nor the authors' intentions were altered in any way during the editing process. TopEdit guarantees the quality of English grammar, spelling, punctuation, syntax, technical consistency, expression continuity, phrasing, presentation coherence, logic, argument, and organization in this manuscript, provided that our editors' corrections and suggestions are accepted and further changes made by the authors are checked by our editors.

Manuscript title

Causal Relationship between Serum Metabolites and Juven ile Idiopathic Arthritis: A Mendelian Randomization Study

Date Issued

11/28/2023

Certificate Number

CN 14786-01-1116-03 R2

TopEdit specializes in comprehensive evaluation and academic editing of technical manuscripts, book chapters, grant proposals, and other types of scientific materials. Aiming at breaking language barriers and advancing research publication, we offer four levels of English editing: Proofreading, Copyediting, Line editing, and Developmental editing. Our editorial team comprises professional native English-speaking experts in various academic fields with advanced Ph.D. degrees.

info@topeditsci.com
https://www.topeditsci.com
https://en.topeditsci.com/
