# Supplementary material for: Causal relationship between serum metabolites and juvenile idiopathic arthritis: a mendelian randomization study
Source: Pediatr Rheumatol Online J. 2024 May 9;22:51. doi: 10.1186/s12969-024-00986-0 (PMC11080266; doi:10.1186/s12969-024-00986-0)
Supplement: Supplementary file 13 — Supplementary Material 13 [file 12969_2024_986_MOESM13_ESM.pdf]

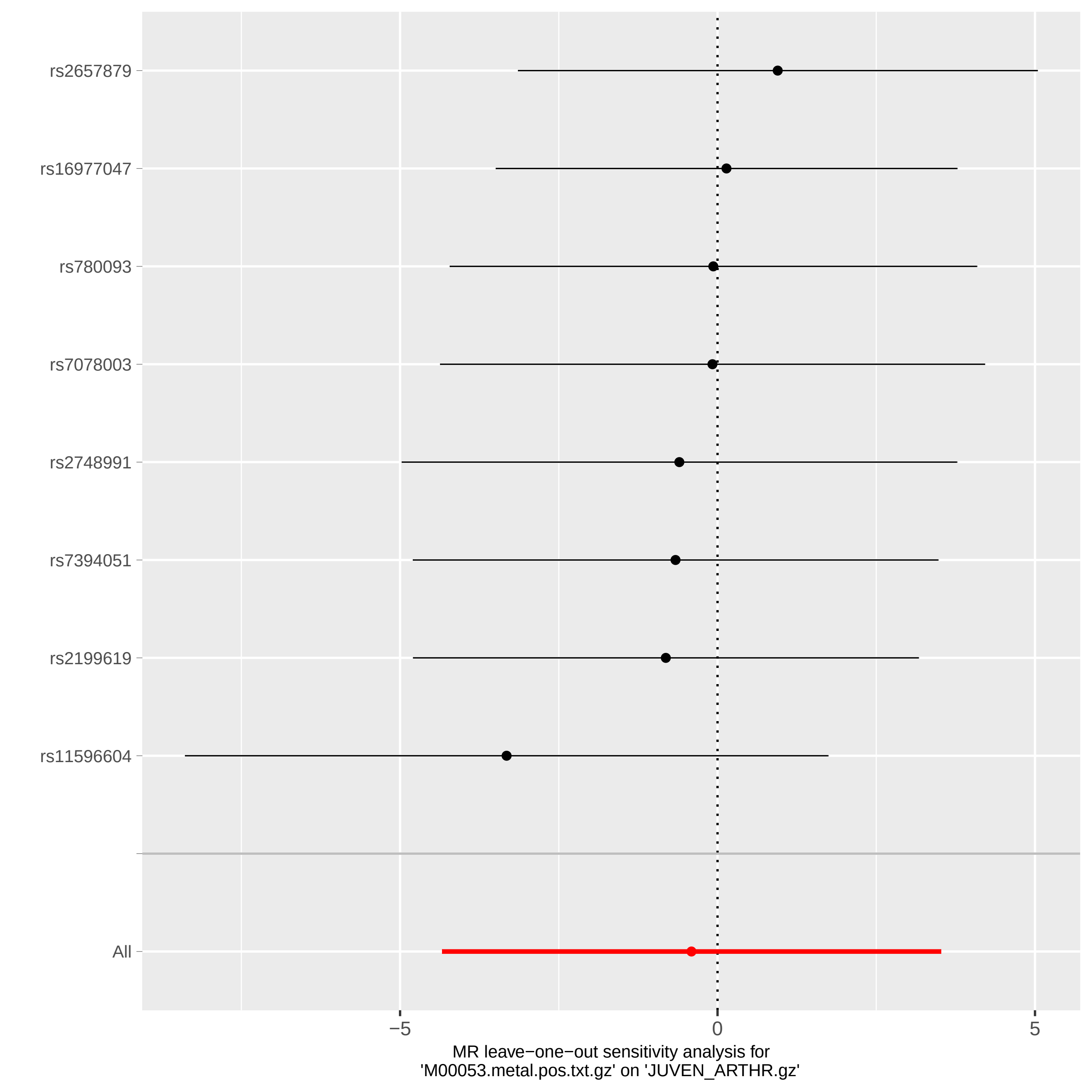

rs2657879
rs16977047
rs780093
rs7078003
rs2748991
rs7394051
rs2199619
rs11596604
All
−5
0
5
MR leave−one−out sensitivity analysis for
'M00053.metal.pos.txt.gz' on 'JUVEN_ARTHR.gz'

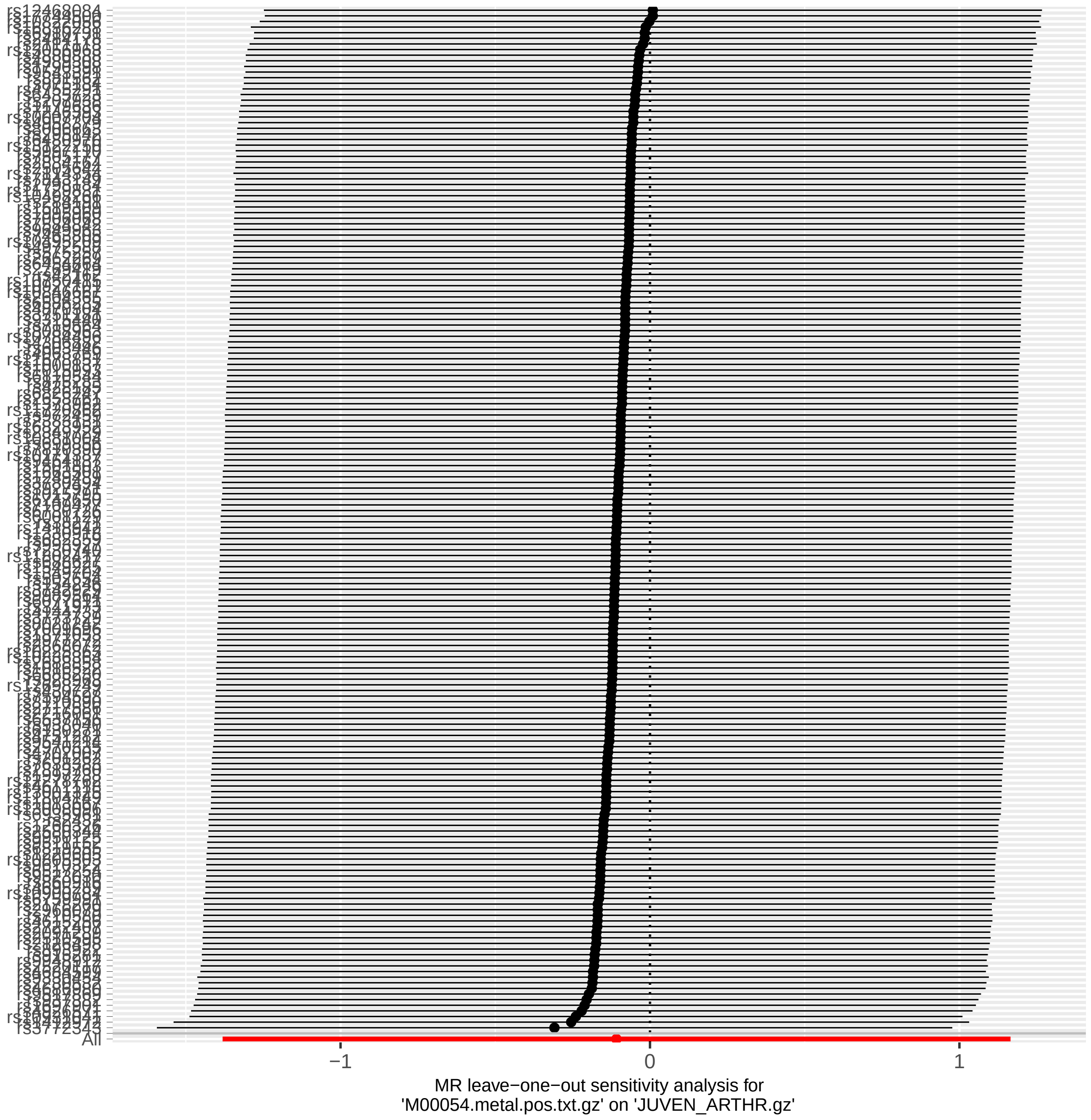

rs12468084
rs3772343
All
−1
0
1
MR leave−one−out sensitivity analysis for
'M00054.metal.pos.txt.gz' on 'JUVEN_ARTHR.gz'

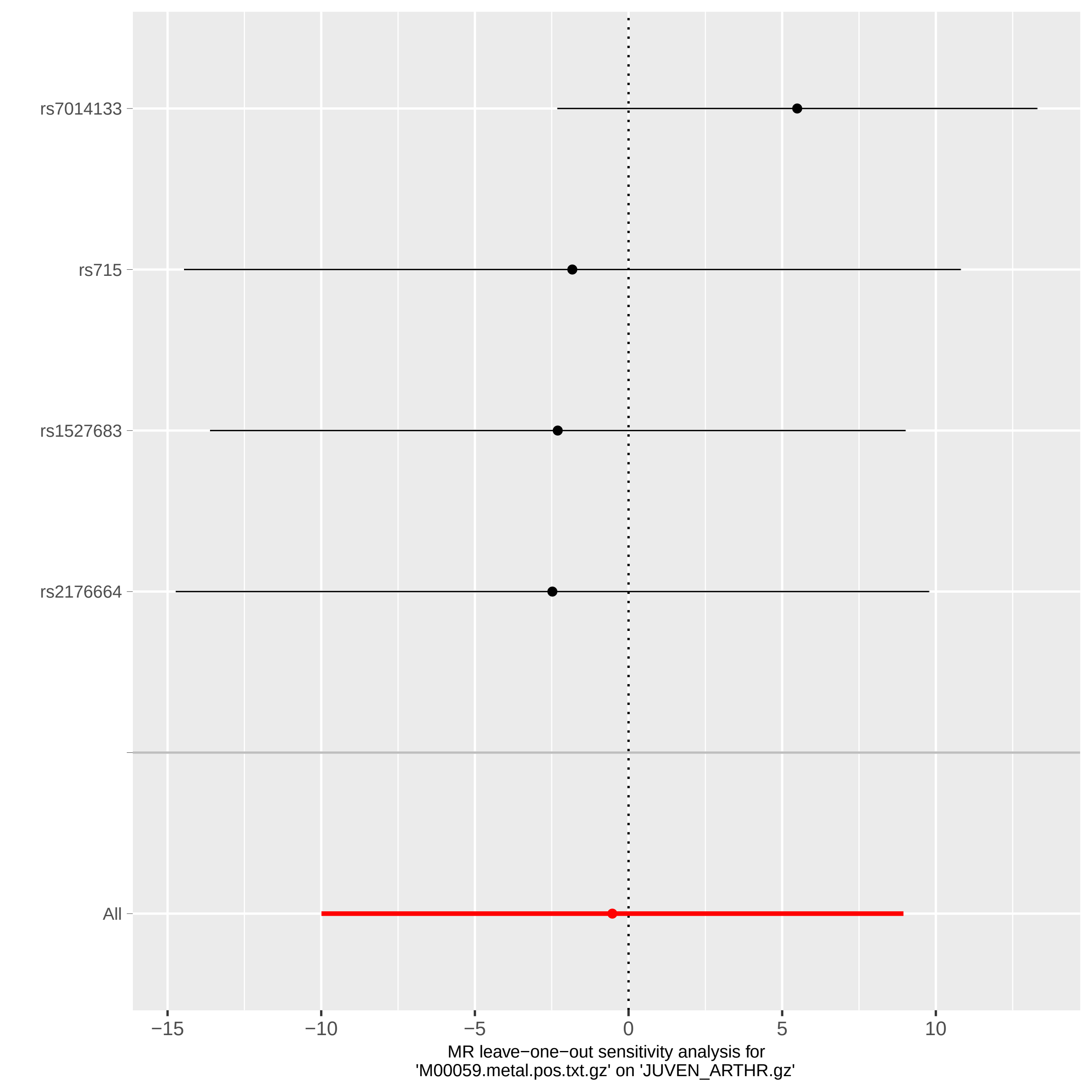
rs7014133
rs715
rs1527683
rs2176664
All
−15
−10
−5
0
5
10
MR leave−one−out sensitivity analysis for
'M00059.metal.pos.txt.gz' on 'JUVEN_ARTHR.gz'

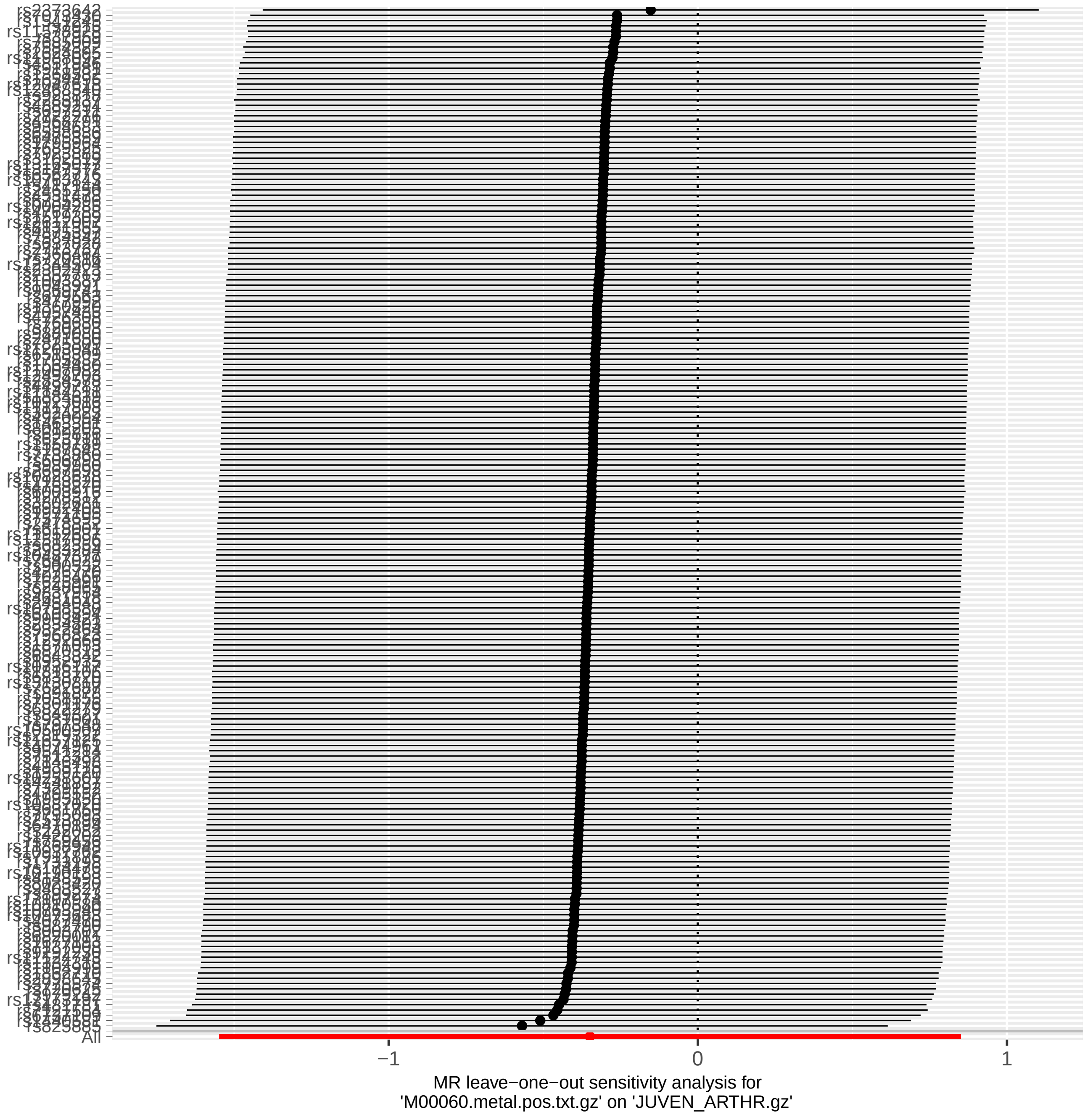
rs2373642
All
−1
0
1
MR leave−one−out sensitivity analysis for
'M00060.metal.pos.txt.gz' on 'JUVEN_ARTHR.gz'

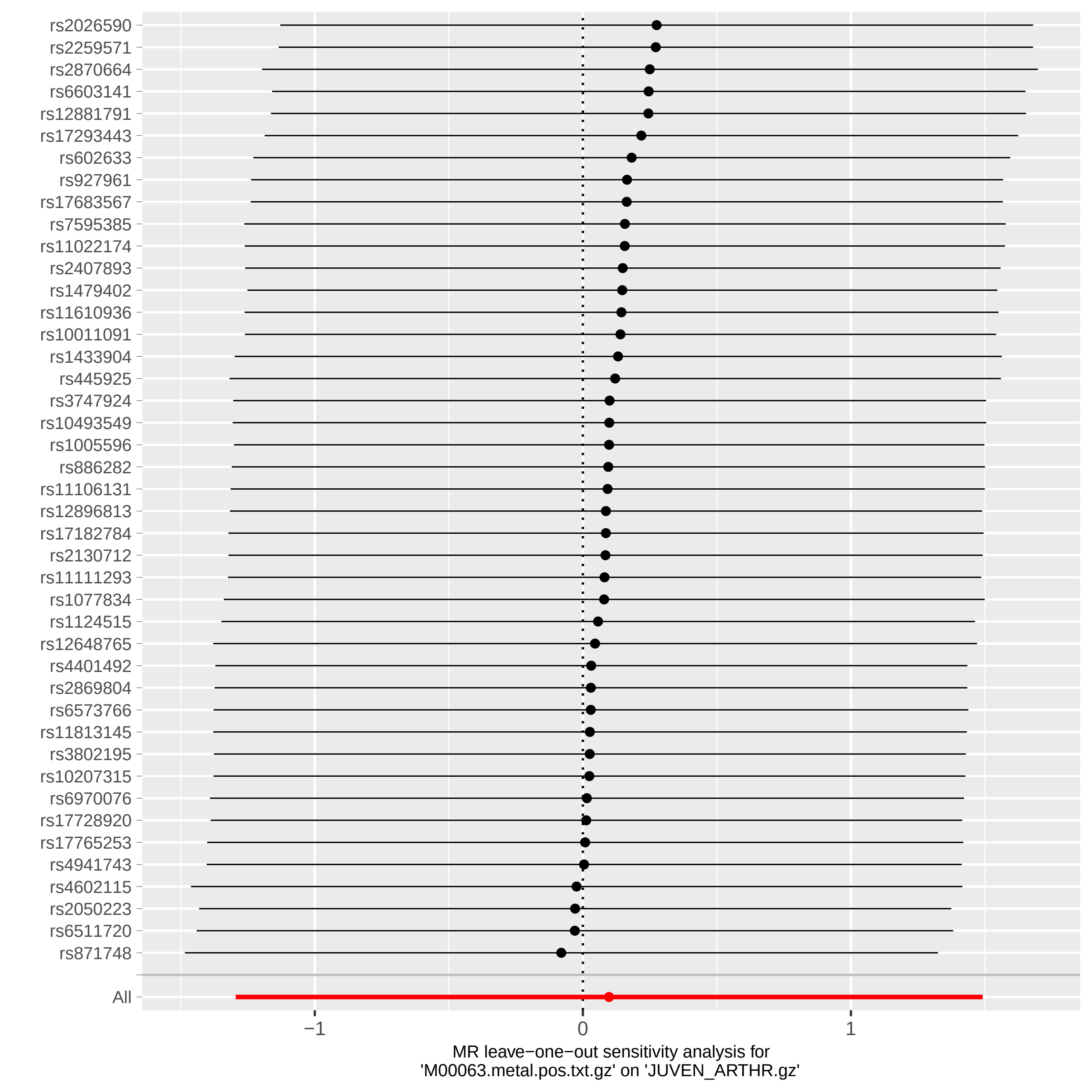
rs2026590
rs2259571
rs2870664
rs6603141
rs12881791
rs17293443
rs602633
rs927961
rs17683567
rs7595385
rs11022174
rs2407893
rs1479402
rs11610936
rs10011091
rs1433904
rs445925
rs3747924
rs10493549
rs1005596
rs886282
rs11106131
rs12896813
rs17182784
rs2130712
rs11111293
rs1077834
rs1124515
rs12648765
rs4401492
rs2869804
rs6573766
rs11813145
rs3802195
rs10207315
rs6970076
rs17728920
rs17765253
rs4941743
rs4602115
rs2050223
rs6511720
rs871748
All
−1
0
1
MR leave−one−out sensitivity analysis for
'M00063.metal.pos.txt.gz' on 'JUVEN_ARTHR.gz'

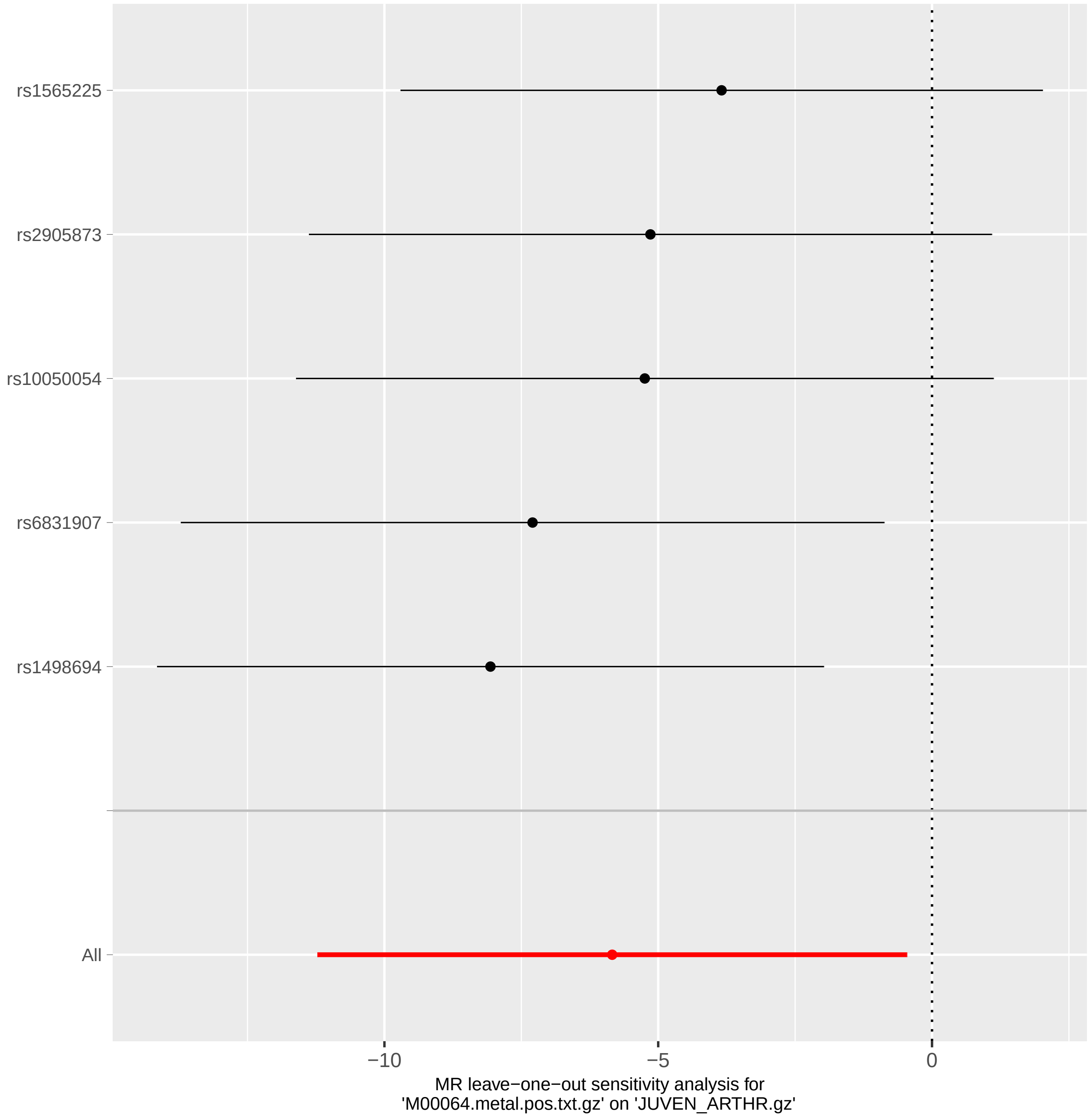
rs1565225
rs2905873
rs10050054
rs6831907
rs1498694
All
−10
−5
0
MR leave−one−out sensitivity analysis for
'M00064.metal.pos.txt.gz' on 'JUVEN_ARTHR.gz'

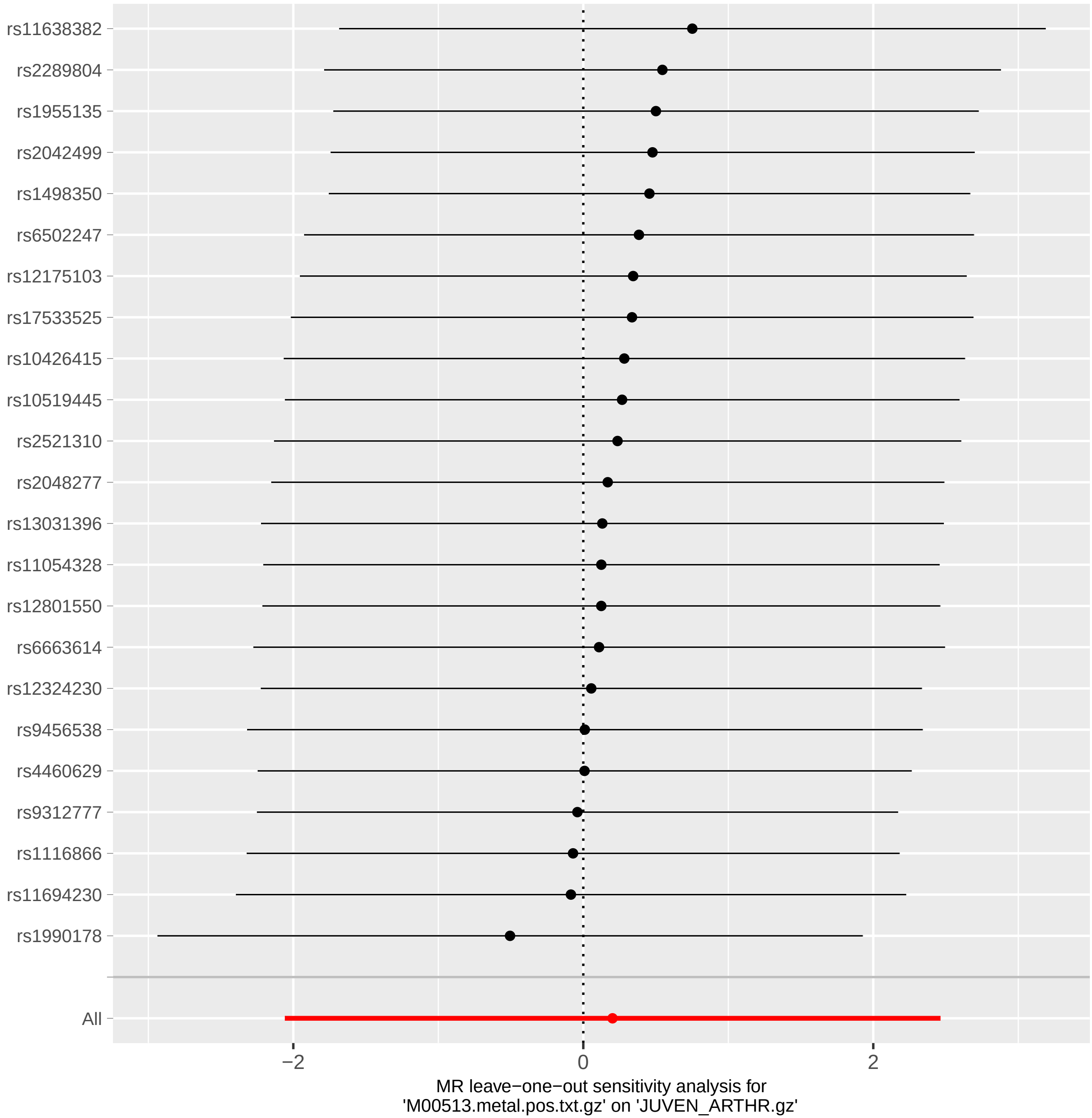
rs11638382
rs2289804
rs1955135
rs2042499
rs1498350
rs6502247
rs12175103
rs17533525
rs10426415
rs10519445
rs2521310
rs2048277
rs13031396
rs11054328
rs12801550
rs6663614
rs12324230
rs9456538
rs4460629
rs9312777
rs1116866
rs11694230
rs1990178
All
−2
0
2
MR leave−one−out sensitivity analysis for
'M00513.metal.pos.txt.gz' on 'JUVEN_ARTHR.gz'

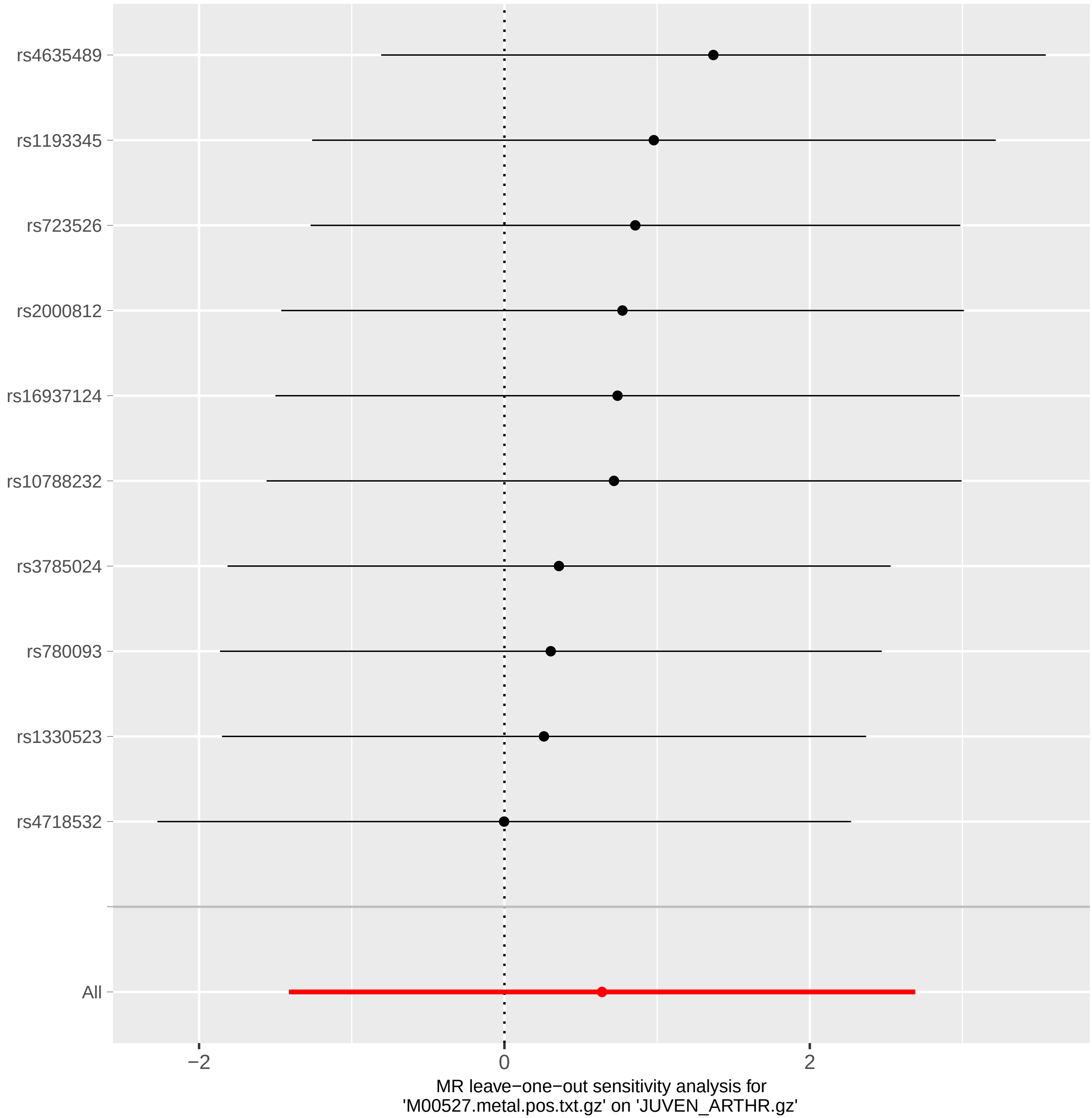
rs4635489
rs1193345
rs723526
rs2000812
rs16937124
rs10788232
rs3785024
rs780093
rs1330523
rs4718532
All
−2
0
2
MR leave−one−out sensitivity analysis for
'M00527.metal.pos.txt.gz' on 'JUVEN_ARTHR.gz'

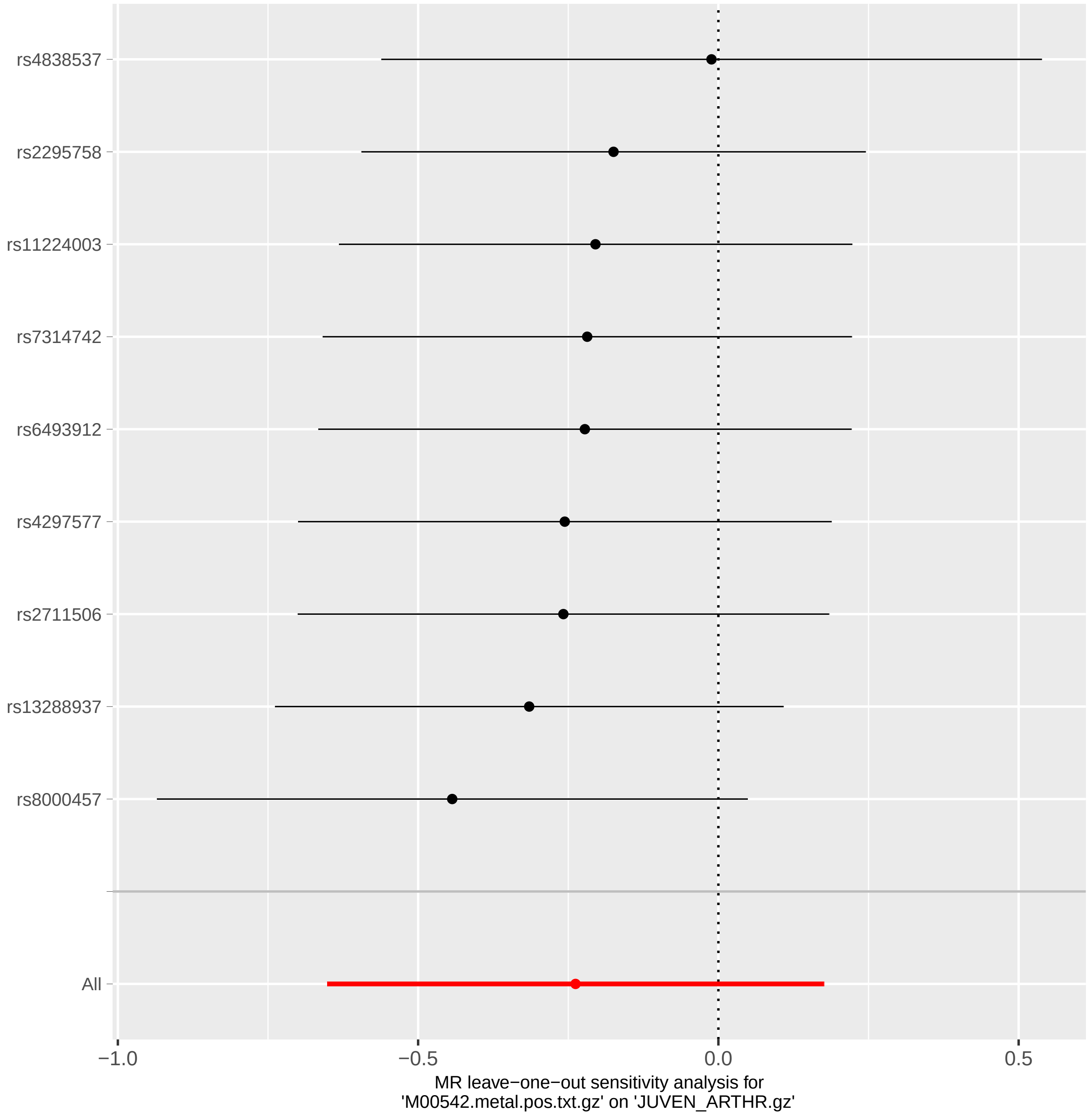
rs4838537
rs2295758
rs11224003
rs7314742
rs6493912
rs4297577
rs2711506
rs13288937
rs8000457
All
−1.0
−0.5
0.0
0.5
MR leave−one−out sensitivity analysis for
'M00542.metal.pos.txt.gz' on 'JUVEN_ARTHR.gz'

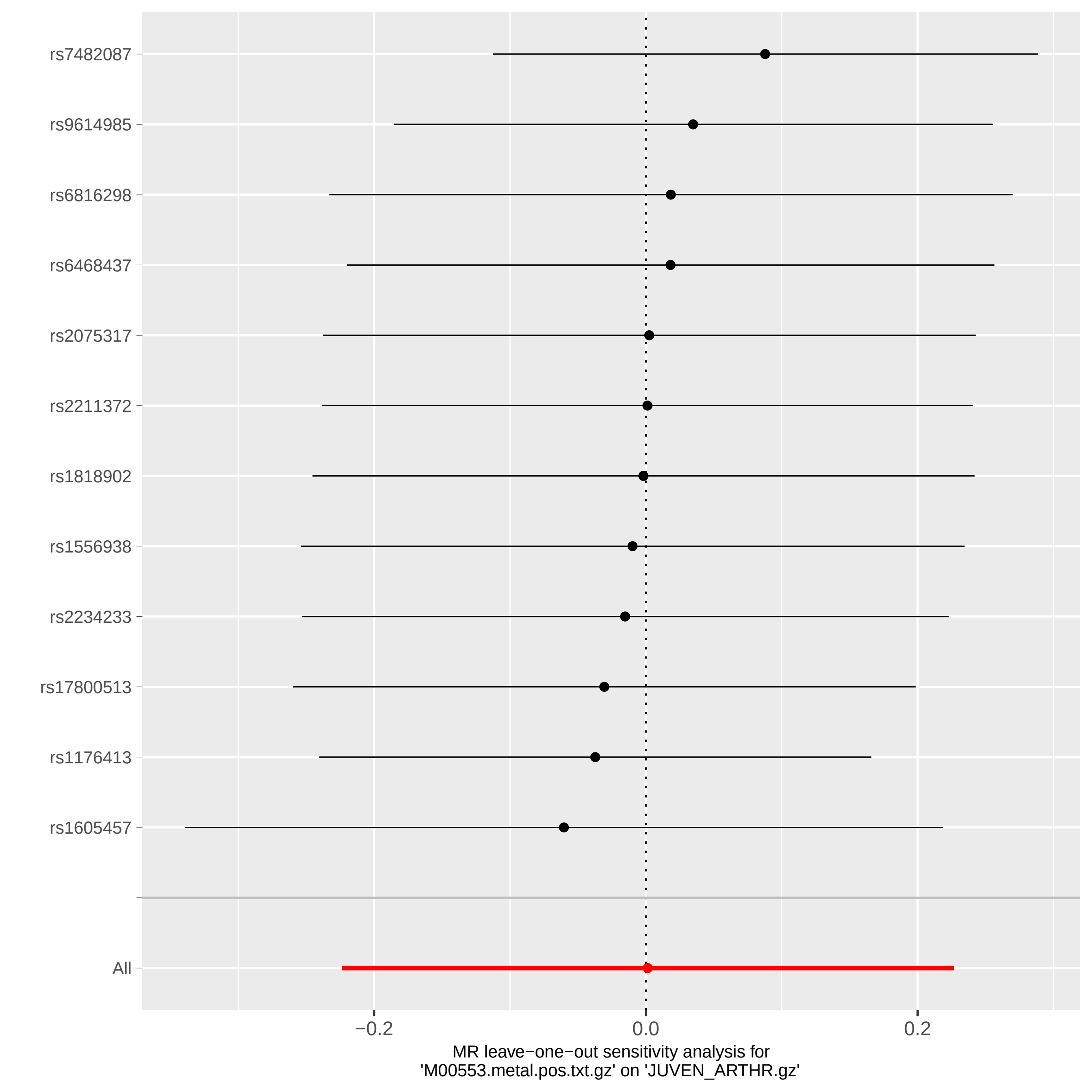
rs7482087
rs9614985
rs6816298
rs6468437
rs2075317
rs2211372
rs1818902
rs1556938
rs2234233
rs17800513
rs1176413
rs1605457
All
−0.2
0.0
0.2
MR leave−one−out sensitivity analysis for
'M00553.metal.pos.txt.gz' on 'JUVEN_ARTHR.gz'

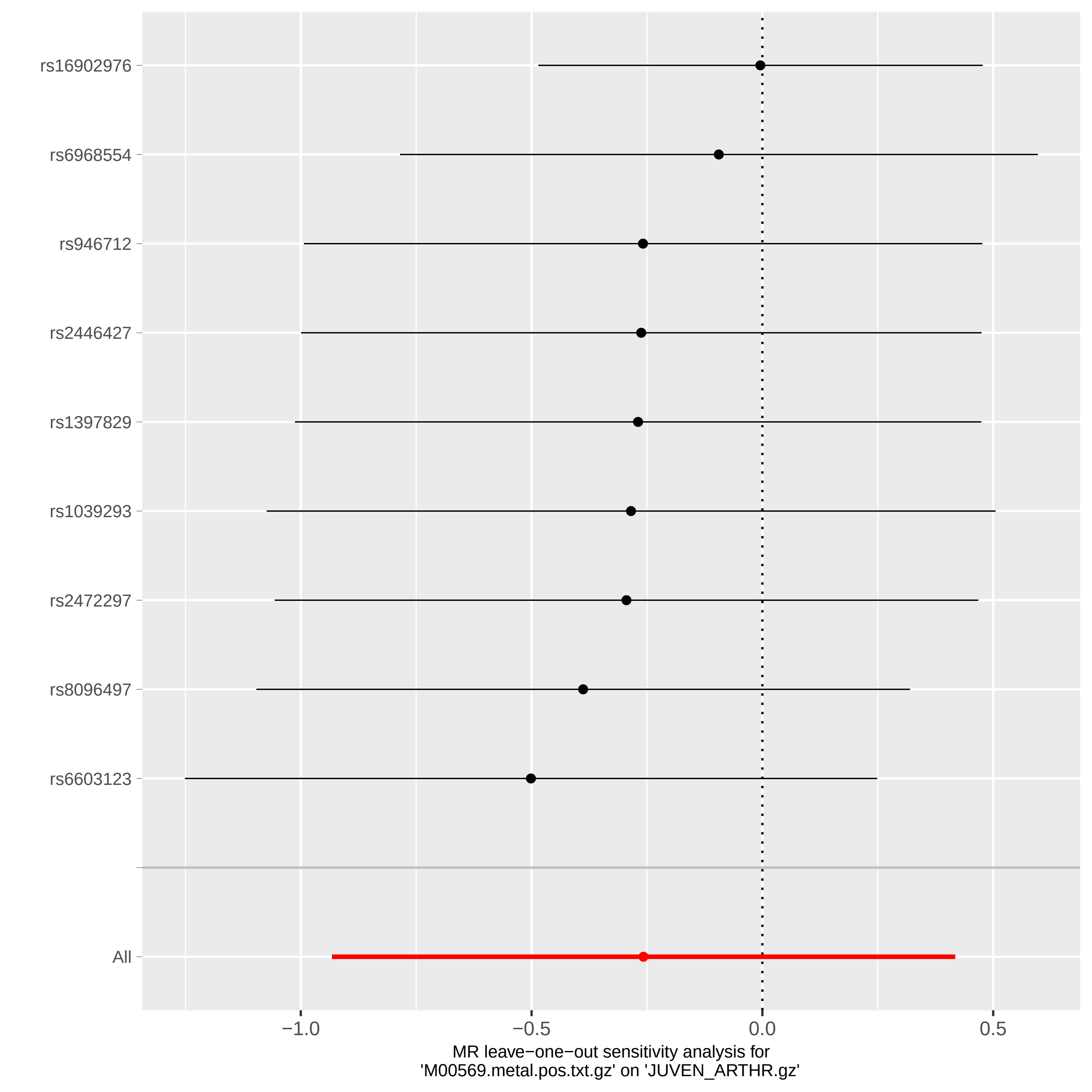
rs16902976
rs6968554
rs946712
rs2446427
rs1397829
rs1039293
rs2472297
rs8096497
rs6603123
All
−1.0
−0.5
0.0
0.5
MR leave−one−out sensitivity analysis for
'M00569.metal.pos.txt.gz' on 'JUVEN_ARTHR.gz'

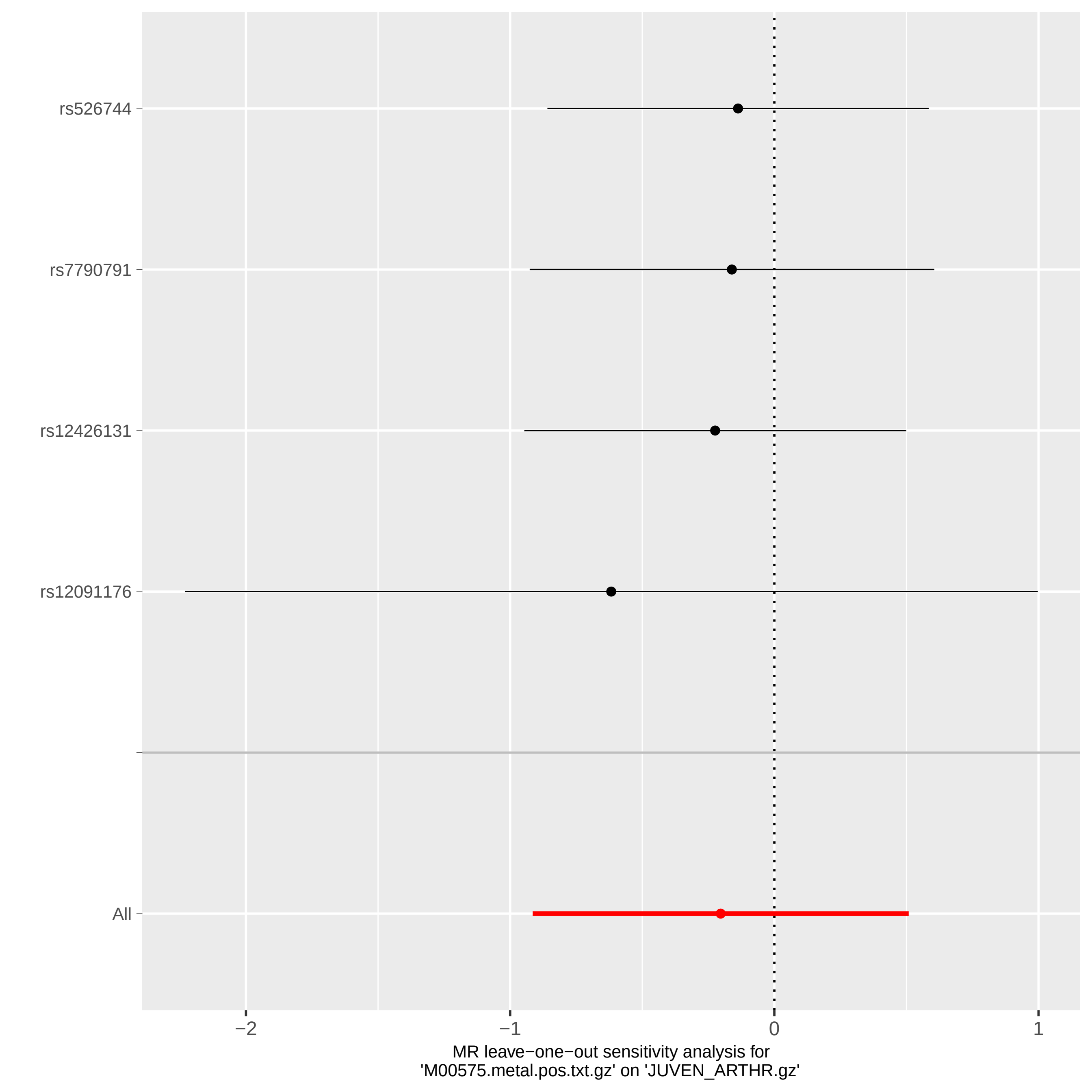

rs526744
rs7790791
rs12426131
rs12091176
All
−2
−1
0
1
MR leave−one−out sensitivity analysis for
'M00575.metal.pos.txt.gz' on 'JUVEN_ARTHR.gz'

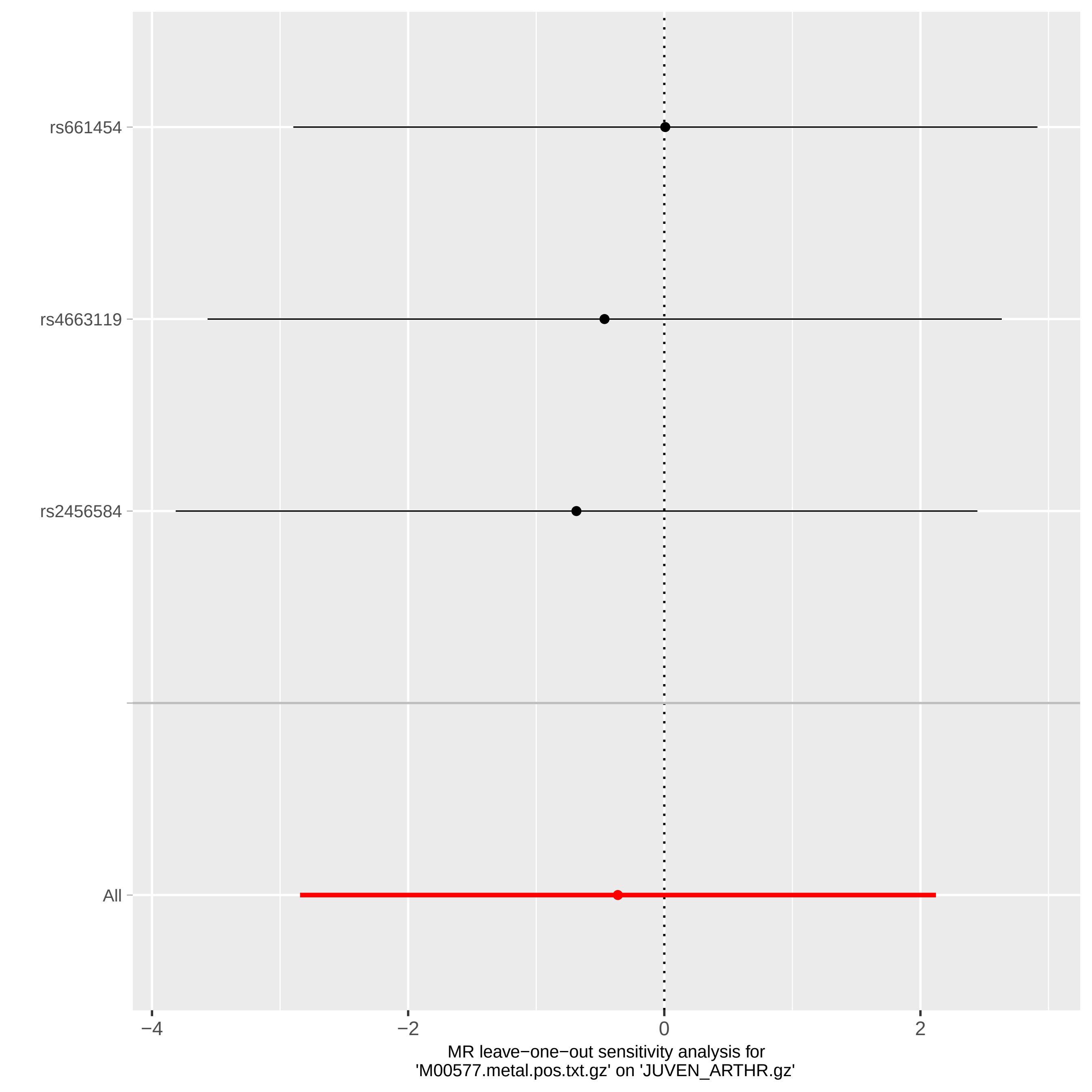

rs661454
rs4663119
rs2456584
All
−4
−2
0
2
MR leave−one−out sensitivity analysis for
'M00577.metal.pos.txt.gz' on 'JUVEN_ARTHR.gz'

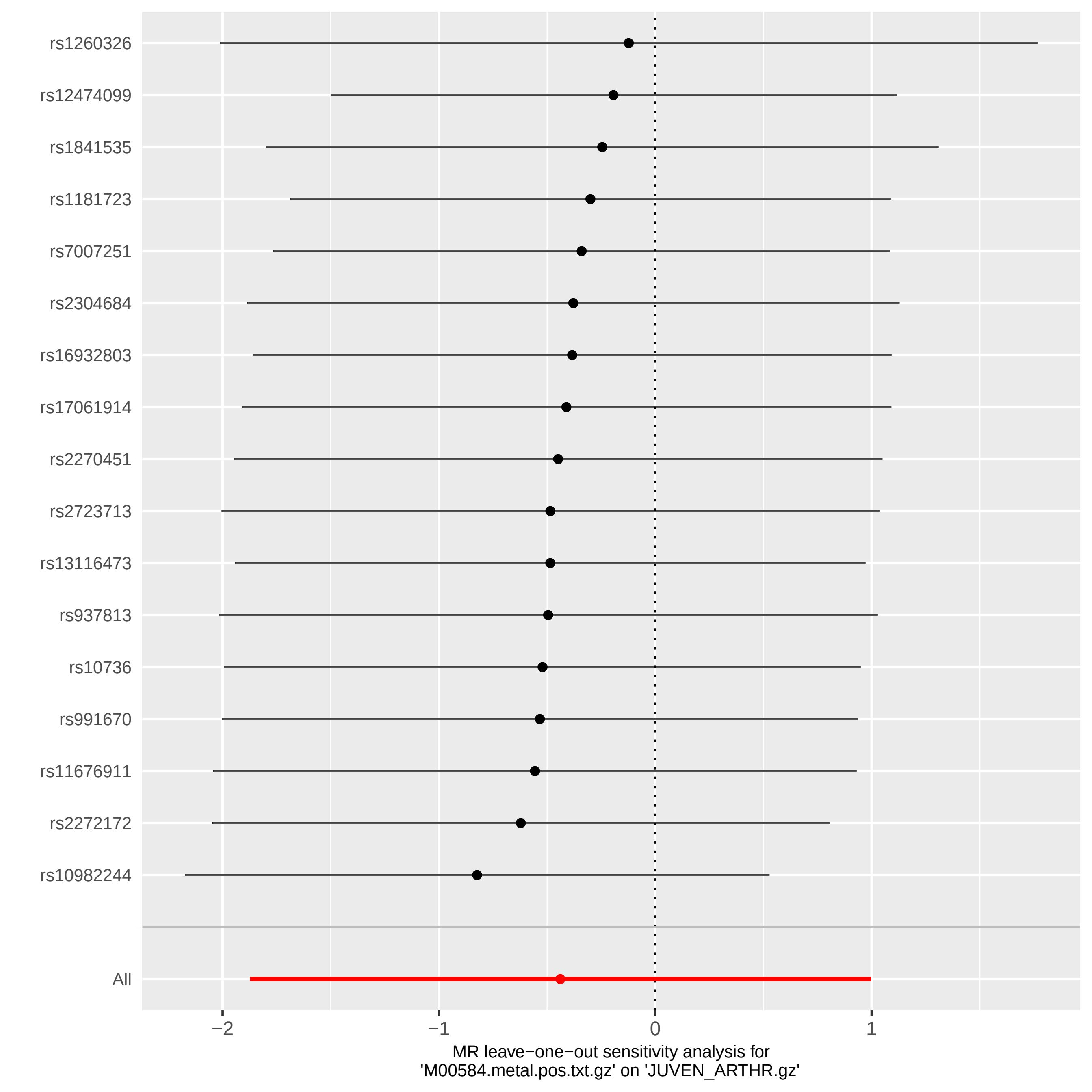

rs1260326
rs12474099
rs1841535
rs1181723
rs7007251
rs2304684
rs16932803
rs17061914
rs2270451
rs2723713
rs13116473
rs937813
rs10736
rs991670
rs11676911
rs2272172
rs10982244
All
−2
−1
0
1
MR leave−one−out sensitivity analysis for
'M00584.metal.pos.txt.gz' on 'JUVEN_ARTHR.gz'

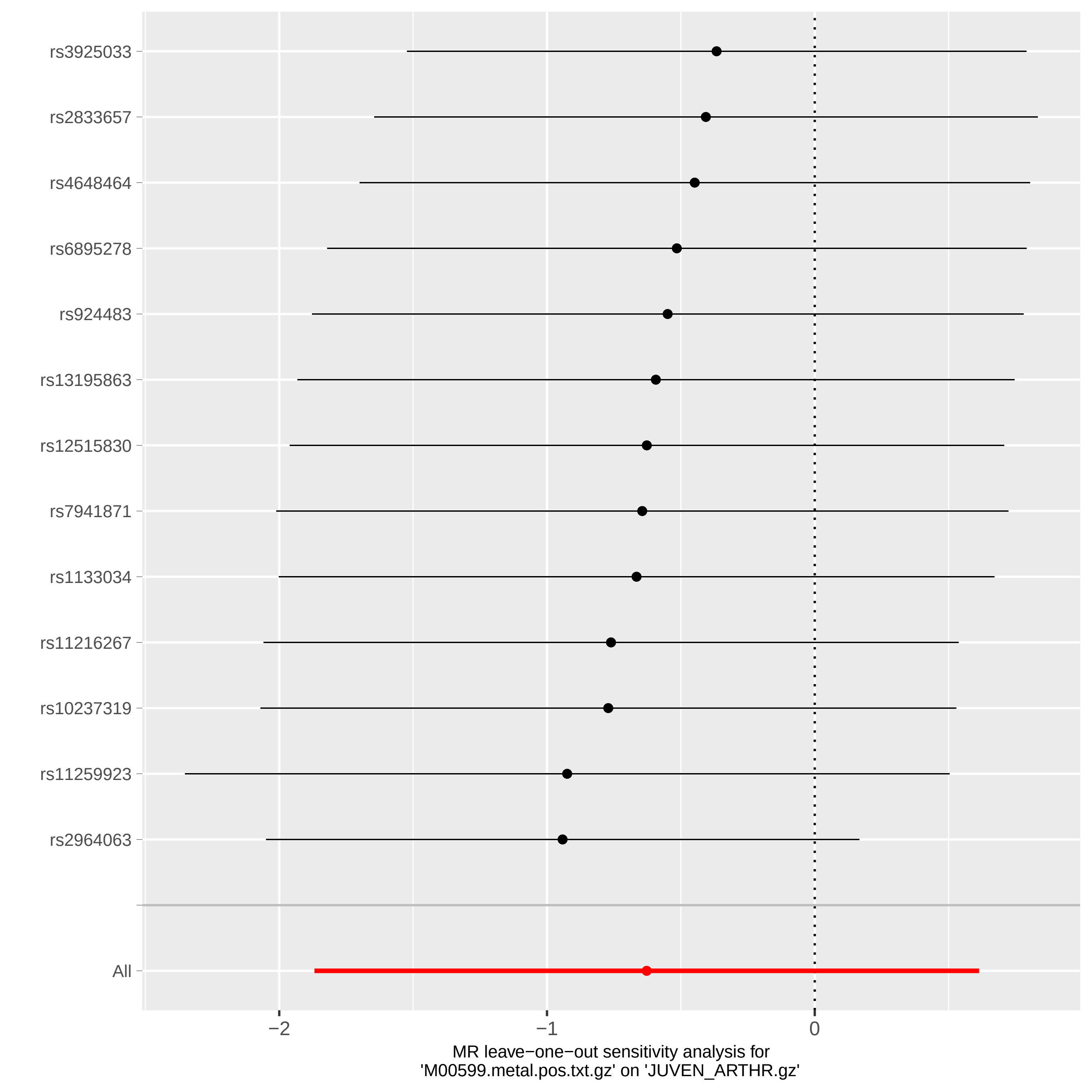
rs3925033
rs2833657
rs4648464
rs6895278
rs924483
rs13195863
rs12515830
rs7941871
rs1133034
rs11216267
rs10237319
rs11259923
rs2964063
All
−2
−1
0
MR leave−one−out sensitivity analysis for
'M00599.metal.pos.txt.gz' on 'JUVEN_ARTHR.gz'

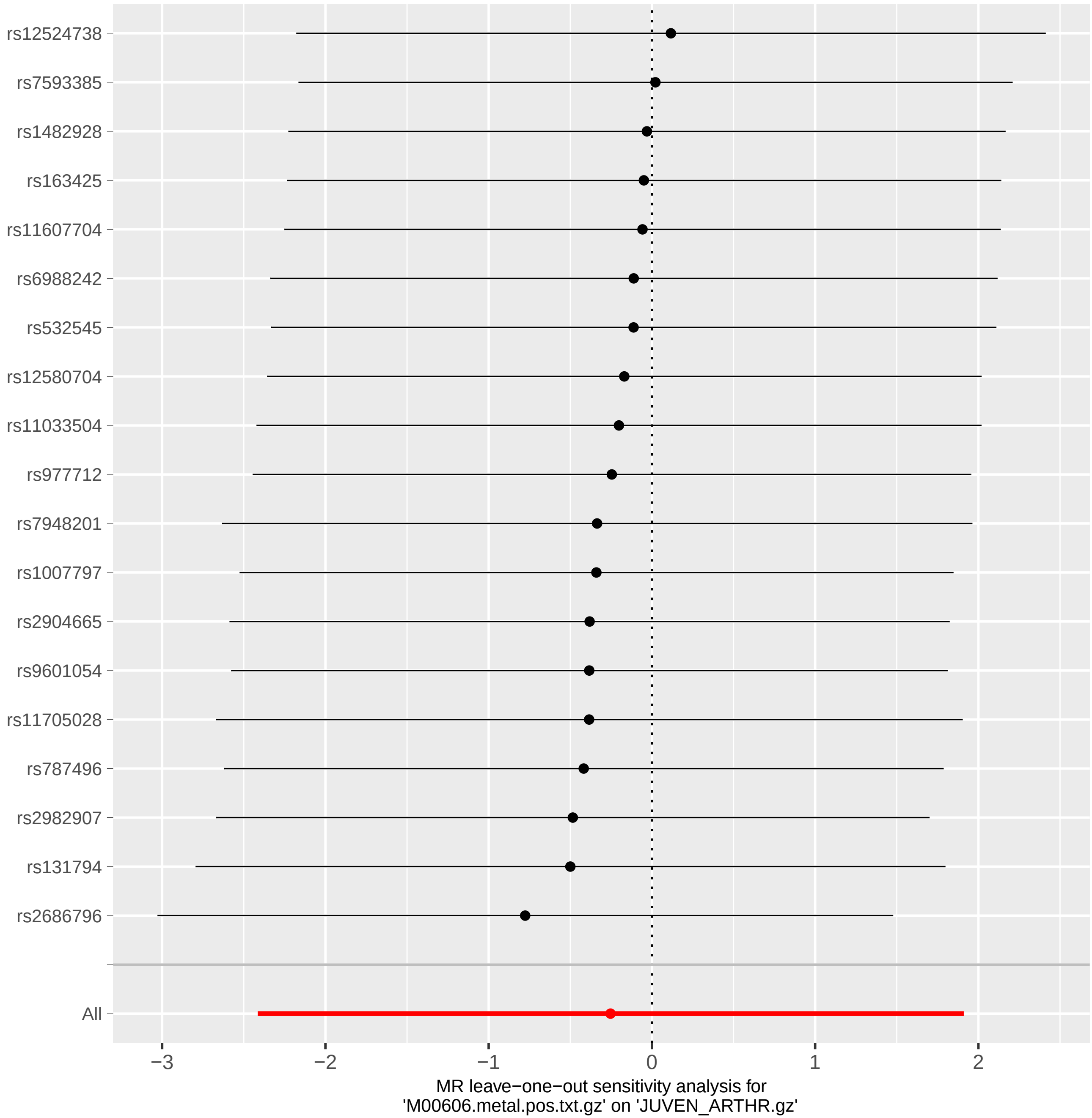
rs12524738
rs7593385
rs1482928
rs163425
rs11607704
rs6988242
rs532545
rs12580704
rs11033504
rs977712
rs7948201
rs1007797
rs2904665
rs9601054
rs11705028
rs787496
rs2982907
rs131794
rs2686796
All
−3
−2
−1
0
1
2
MR leave−one−out sensitivity analysis for
'M00606.metal.pos.txt.gz' on 'JUVEN_ARTHR.gz'

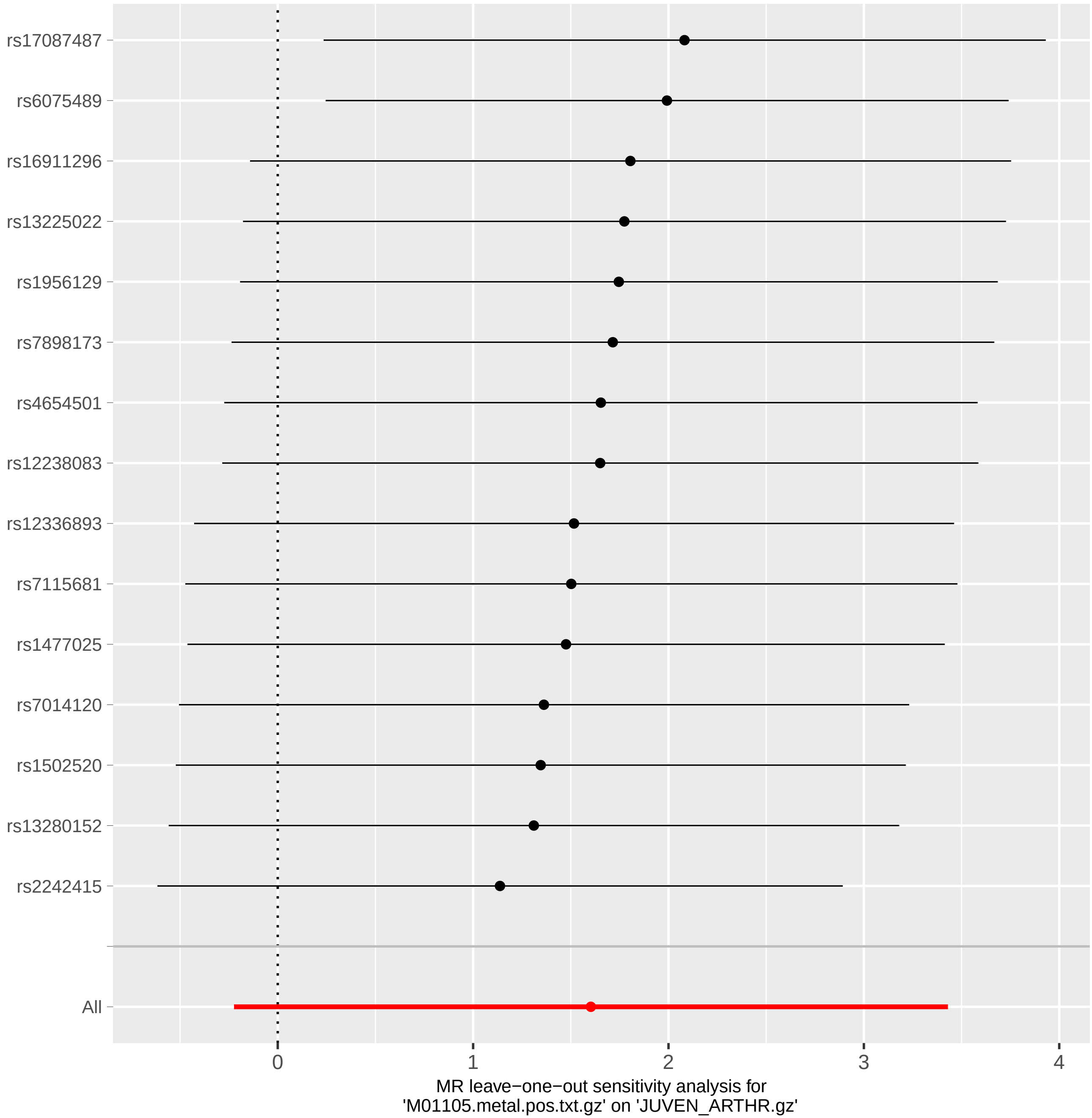
rs17087487
rs6075489
rs16911296
rs13225022
rs1956129
rs7898173
rs4654501
rs12238083
rs12336893
rs7115681
rs1477025
rs7014120
rs1502520
rs13280152
rs2242415
All
0
1
2
3
4
MR leave−one−out sensitivity analysis for
'M01105.metal.pos.txt.gz' on 'JUVEN_ARTHR.gz'

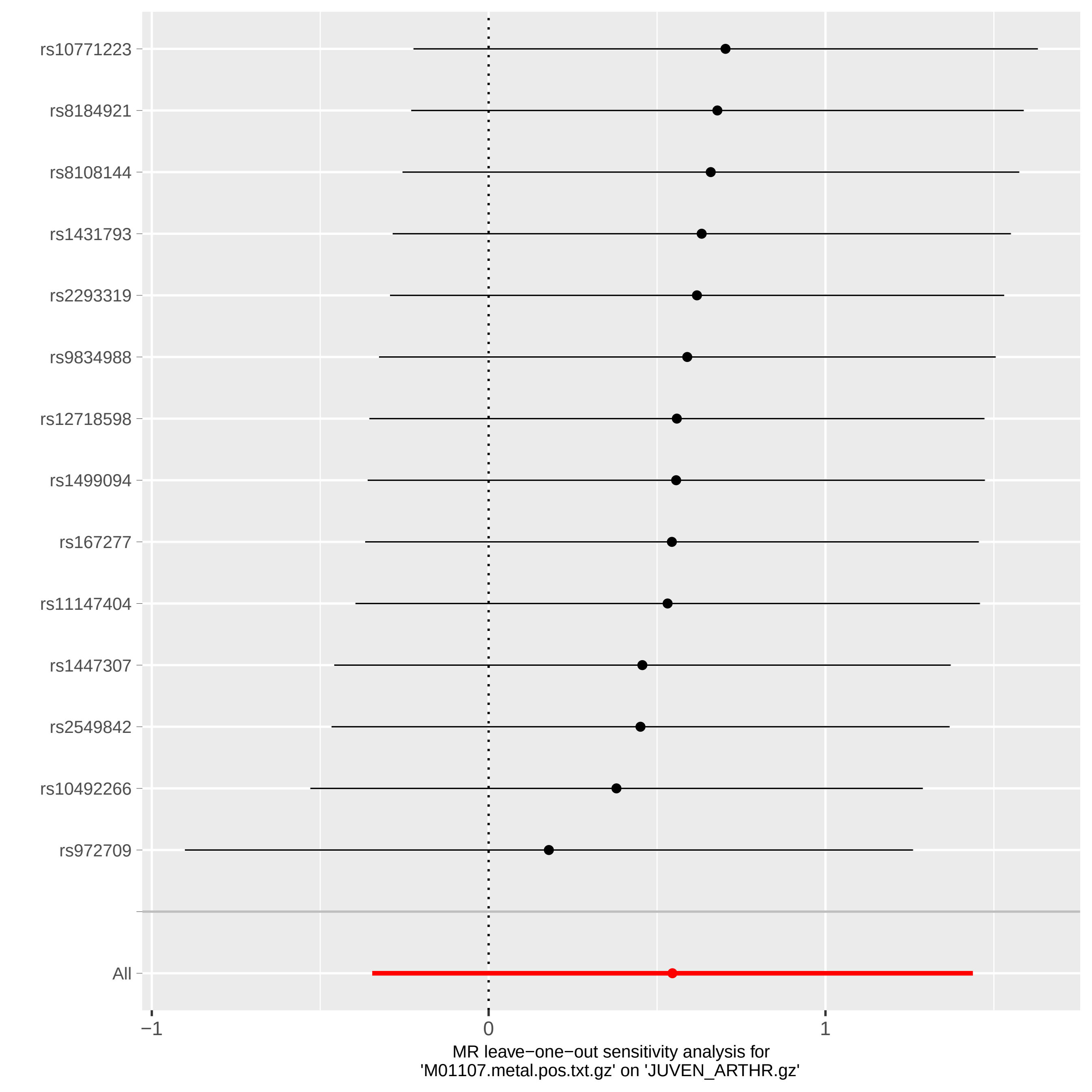
rs10771223
rs8184921
rs8108144
rs1431793
rs2293319
rs9834988
rs12718598
rs1499094
rs167277
rs11147404
rs1447307
rs2549842
rs10492266
rs972709
All
−1
0
1
MR leave−one−out sensitivity analysis for
'M01107.metal.pos.txt.gz' on 'JUVEN_ARTHR.gz'

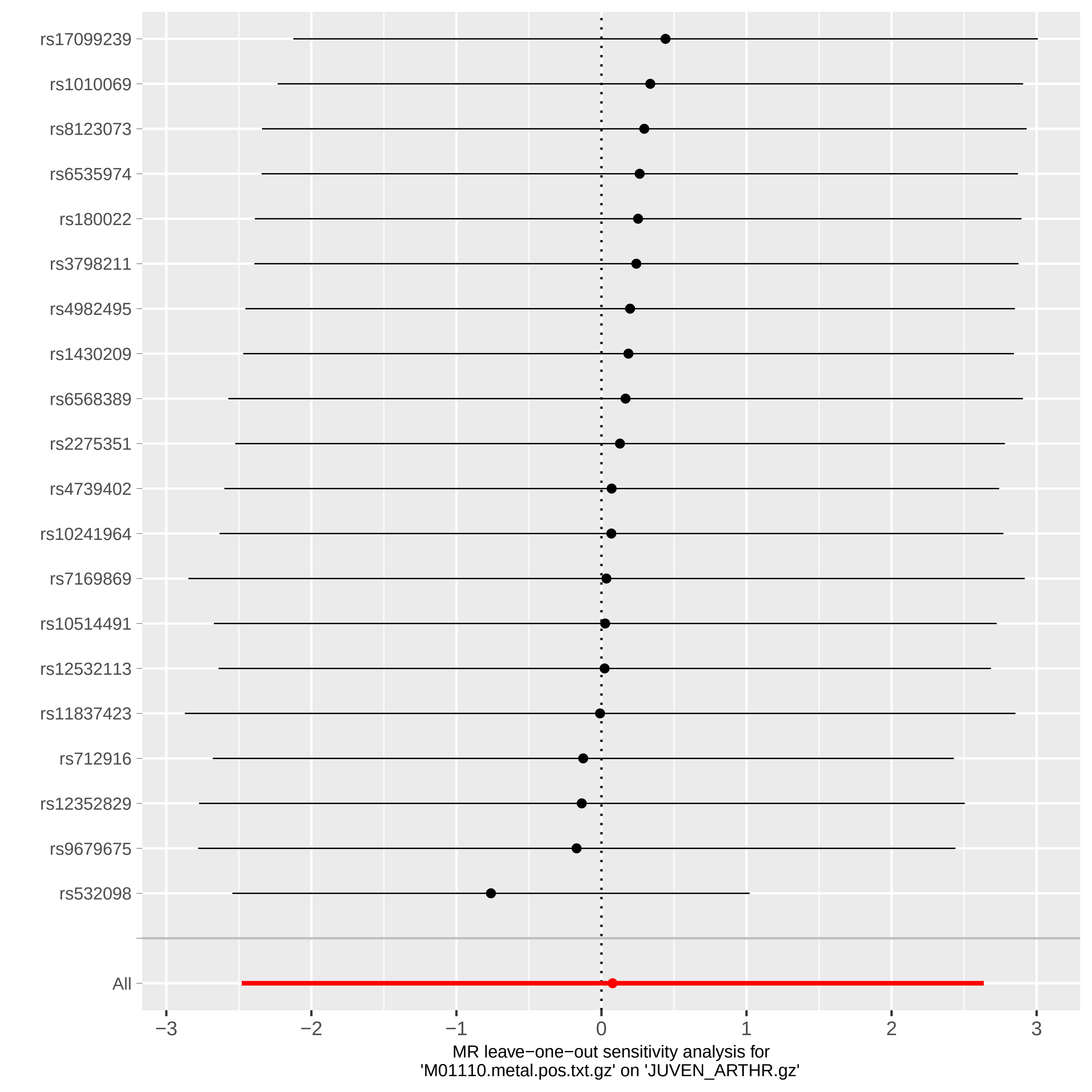
rs17099239
rs1010069
rs8123073
rs6535974
rs180022
rs3798211
rs4982495
rs1430209
rs6568389
rs2275351
rs4739402
rs10241964
rs7169869
rs10514491
rs12532113
rs11837423
rs712916
rs12352829
rs9679675
rs532098
All
−3
−2
−1
0
1
2
3
MR leave−one−out sensitivity analysis for
'M01110.metal.pos.txt.gz' on 'JUVEN_ARTHR.gz'

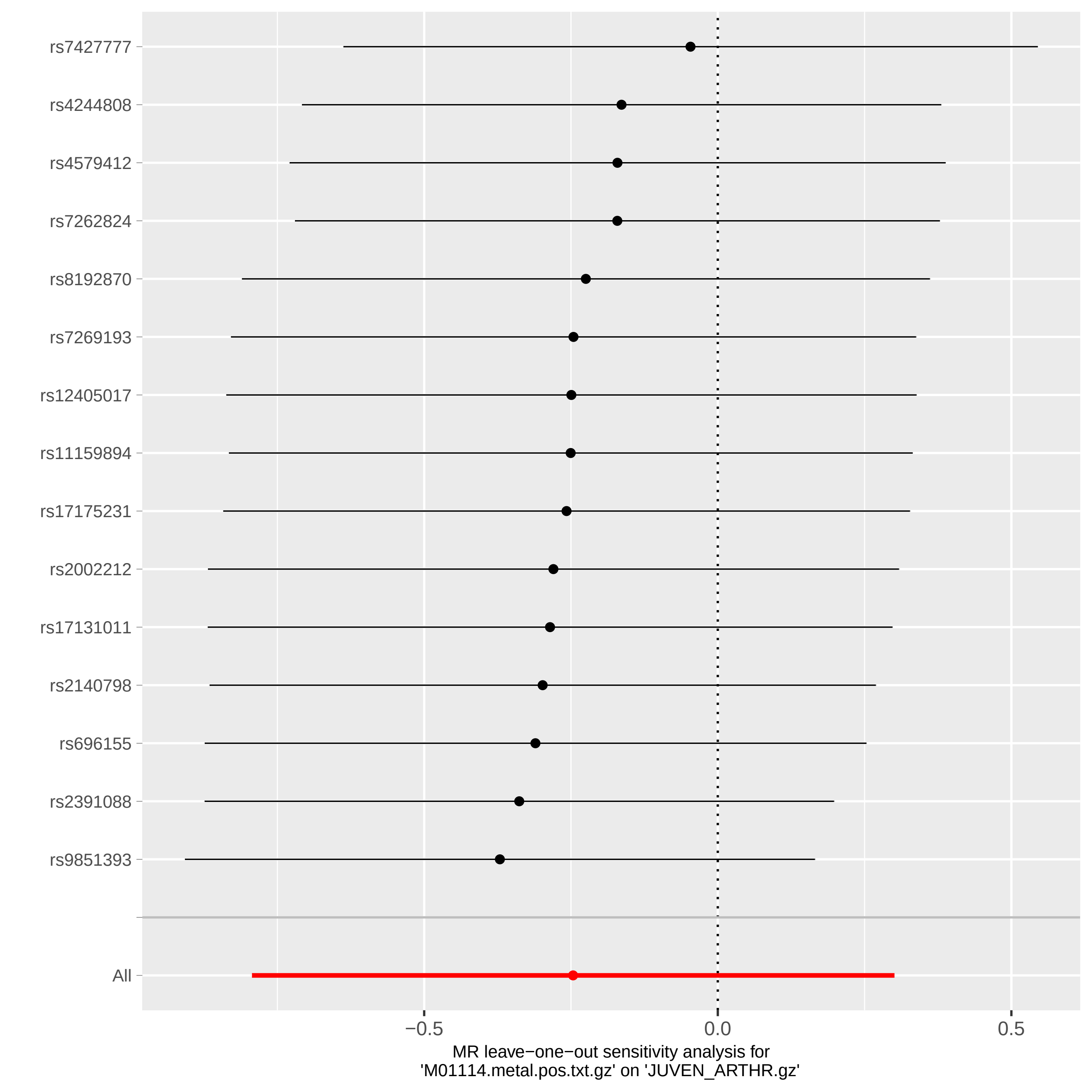
rs7427777
rs4244808
rs4579412
rs7262824
rs8192870
rs7269193
rs12405017
rs11159894
rs17175231
rs2002212
rs17131011
rs2140798
rs696155
rs2391088
rs9851393
All
−0.5
0.0
0.5
MR leave−one−out sensitivity analysis for
'M01114.metal.pos.txt.gz' on 'JUVEN_ARTHR.gz'

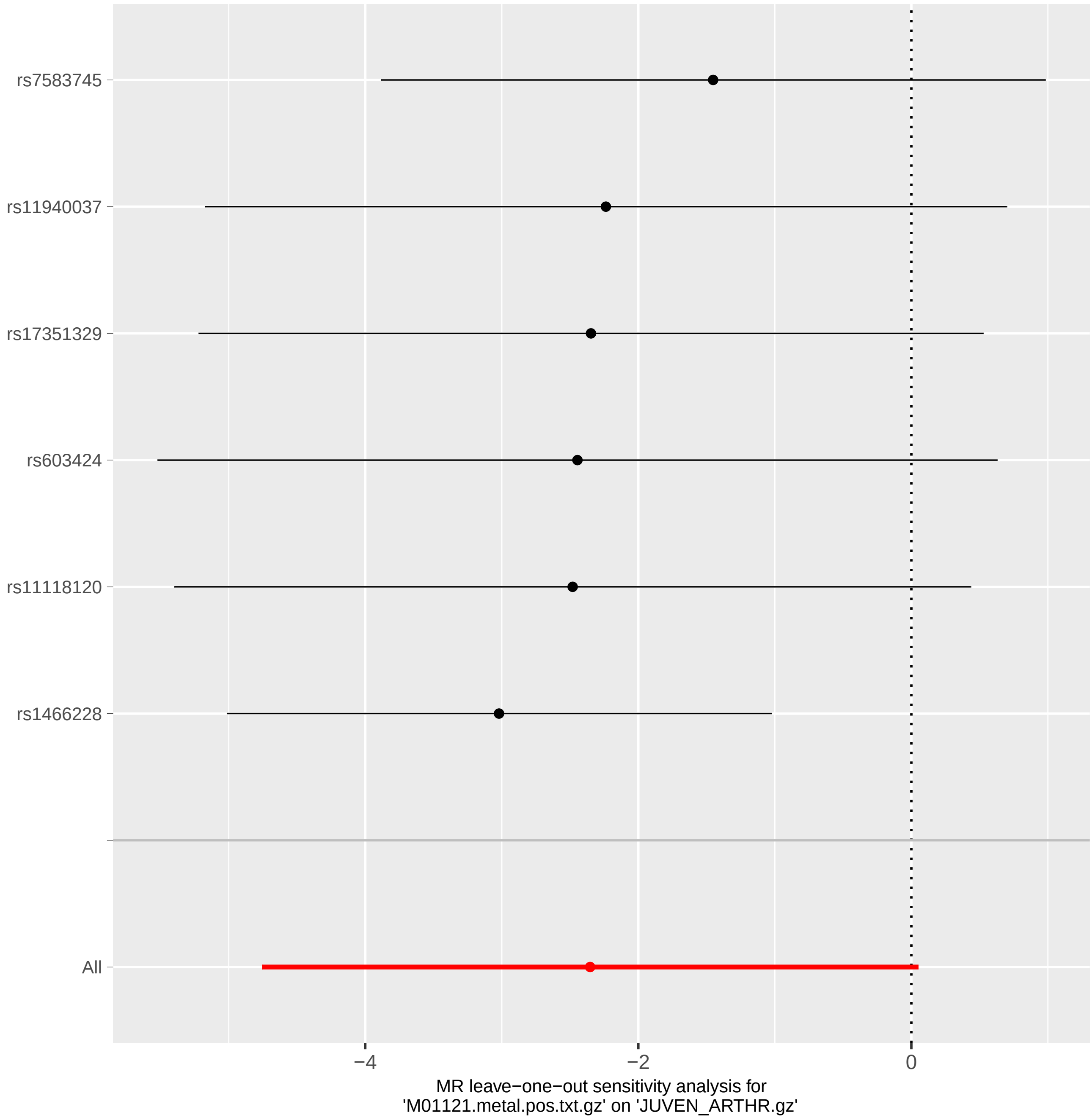

rs7583745
rs11940037
rs17351329
rs603424
rs11118120
rs1466228
All
−4
−2
0
MR leave−one−out sensitivity analysis for
'M01121.metal.pos.txt.gz' on 'JUVEN_ARTHR.gz'

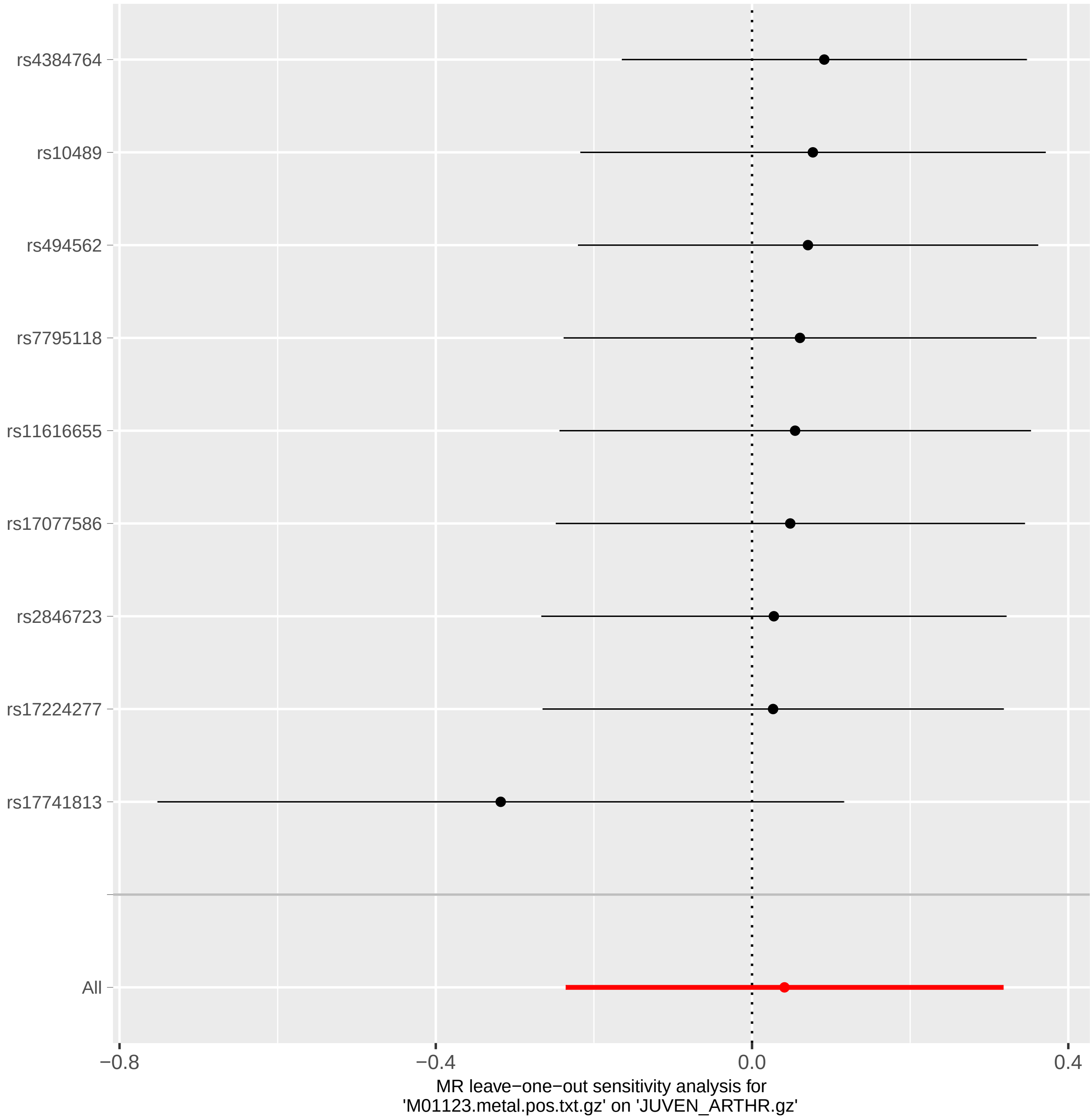
rs4384764
rs10489
rs494562
rs7795118
rs11616655
rs17077586
rs2846723
rs17224277
rs17741813
All
−0.8
−0.4
0.0
0.4
MR leave−one−out sensitivity analysis for
'M01123.metal.pos.txt.gz' on 'JUVEN_ARTHR.gz'

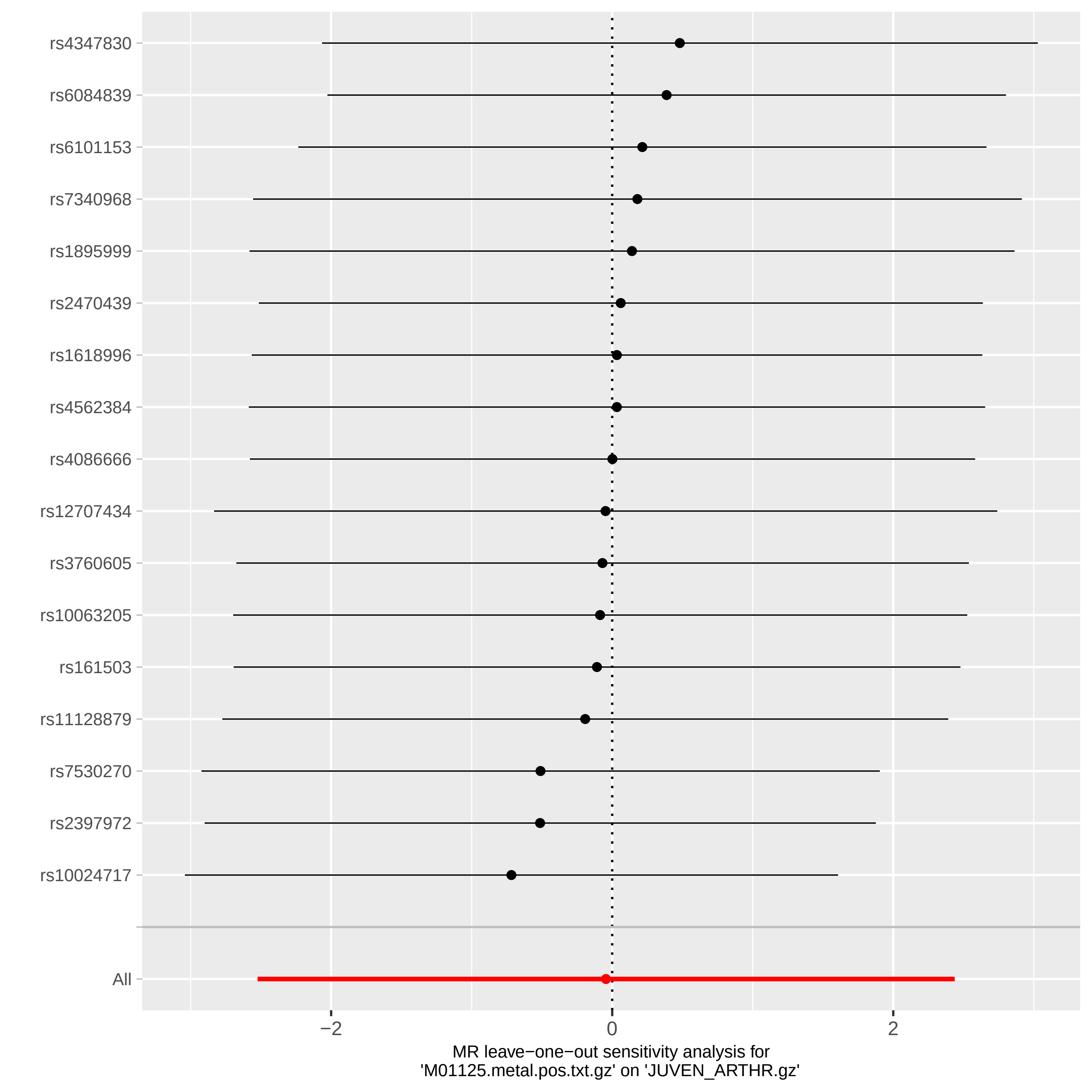

rs4347830
rs6084839
rs6101153
rs7340968
rs1895999
rs2470439
rs1618996
rs4562384
rs4086666
rs12707434
rs3760605
rs10063205
rs161503
rs11128879
rs7530270
rs2397972
rs10024717
All
−2
0
2
MR leave−one−out sensitivity analysis for
'M01125.metal.pos.txt.gz' on 'JUVEN_ARTHR.gz'

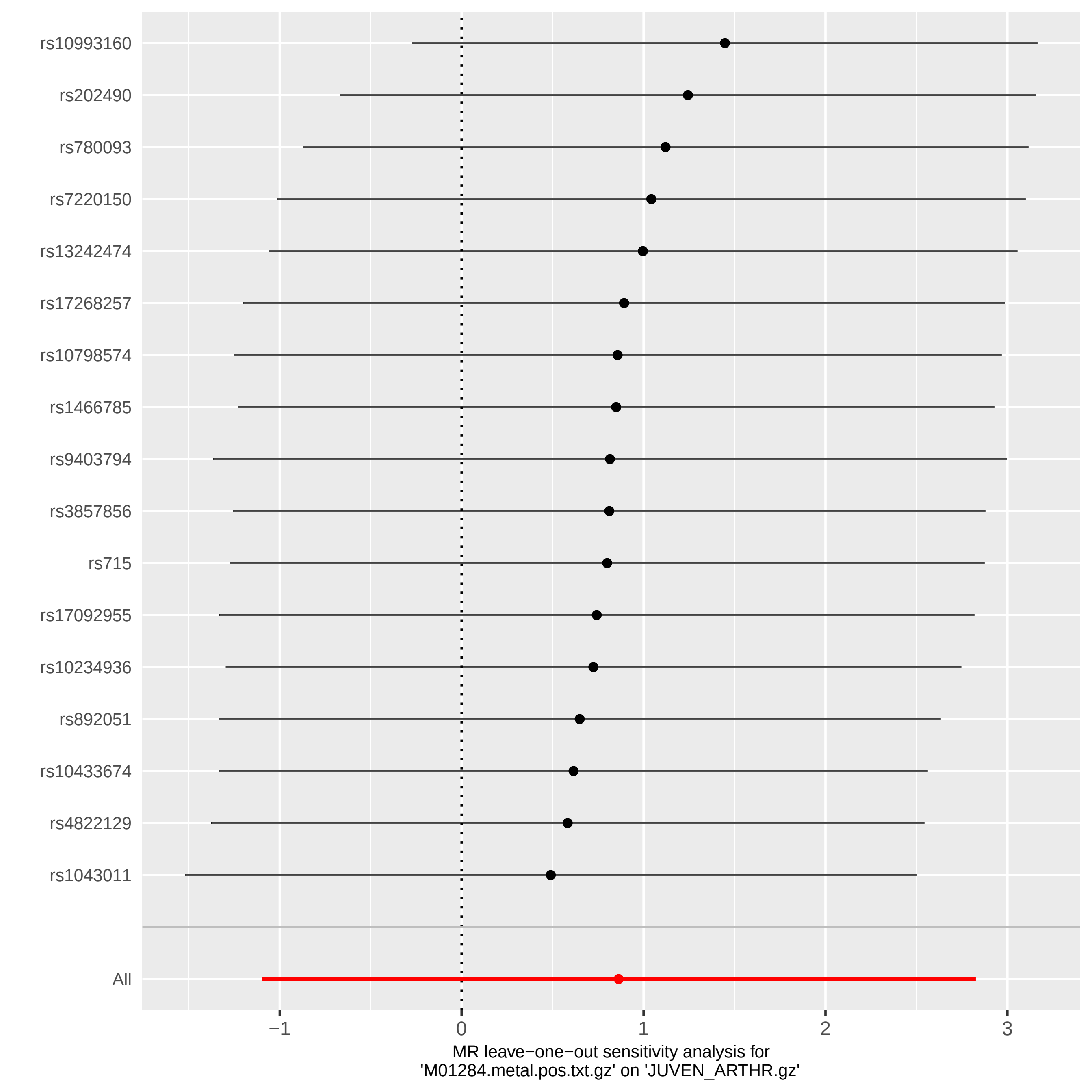
rs10993160
rs202490
rs780093
rs7220150
rs13242474
rs17268257
rs10798574
rs1466785
rs9403794
rs3857856
rs715
rs17092955
rs10234936
rs892051
rs10433674
rs4822129
rs1043011
All
−1
0
1
2
3
MR leave−one−out sensitivity analysis for
'M01284.metal.pos.txt.gz' on 'JUVEN_ARTHR.gz'

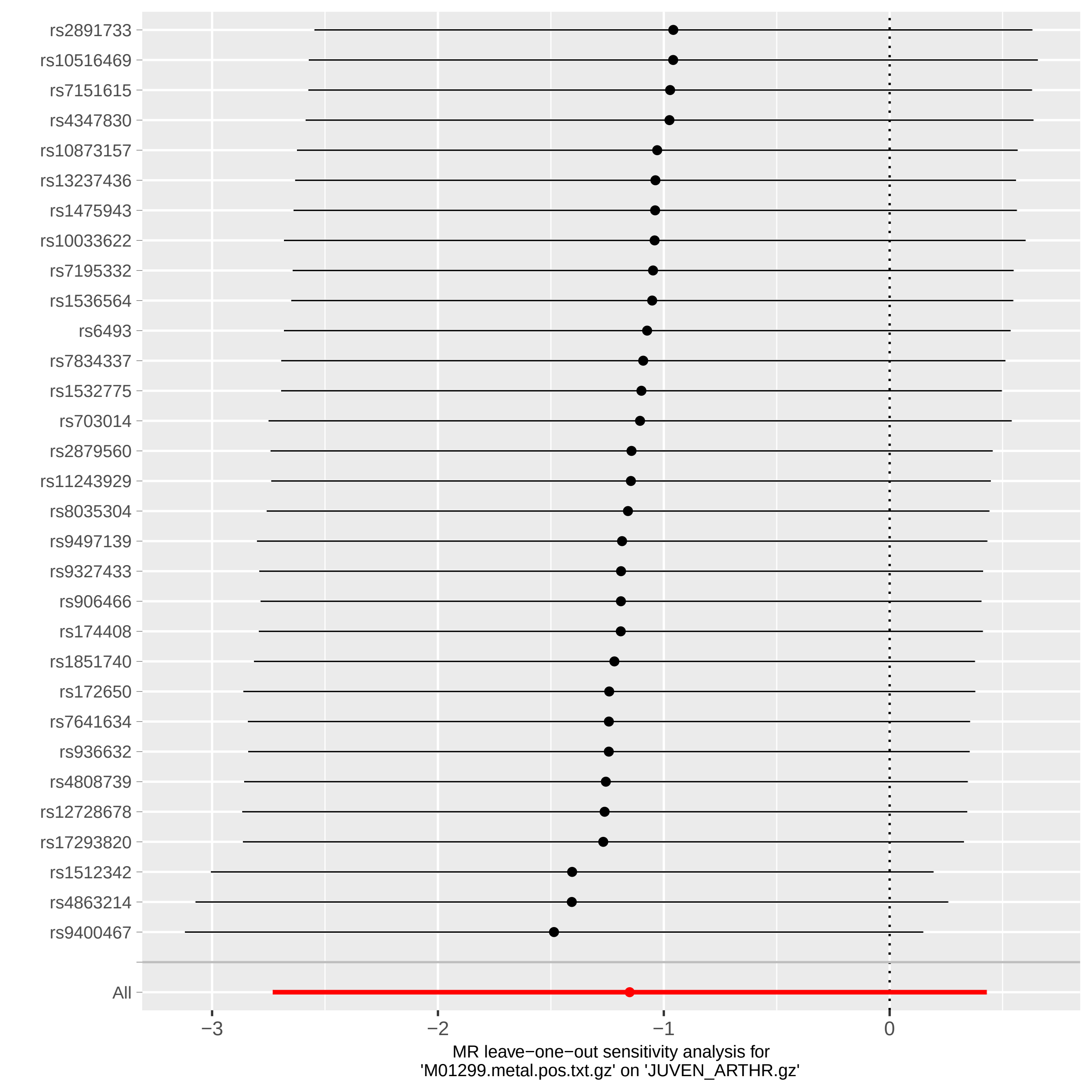
rs2891733
rs10516469
rs7151615
rs4347830
rs10873157
rs13237436
rs1475943
rs10033622
rs7195332
rs1536564
rs6493
rs7834337
rs1532775
rs703014
rs2879560
rs11243929
rs8035304
rs9497139
rs9327433
rs906466
rs174408
rs1851740
rs172650
rs7641634
rs936632
rs4808739
rs12728678
rs17293820
rs1512342
rs4863214
rs9400467
All
−3
−2
−1
0
MR leave−one−out sensitivity analysis for
'M01299.metal.pos.txt.gz' on 'JUVEN_ARTHR.gz'

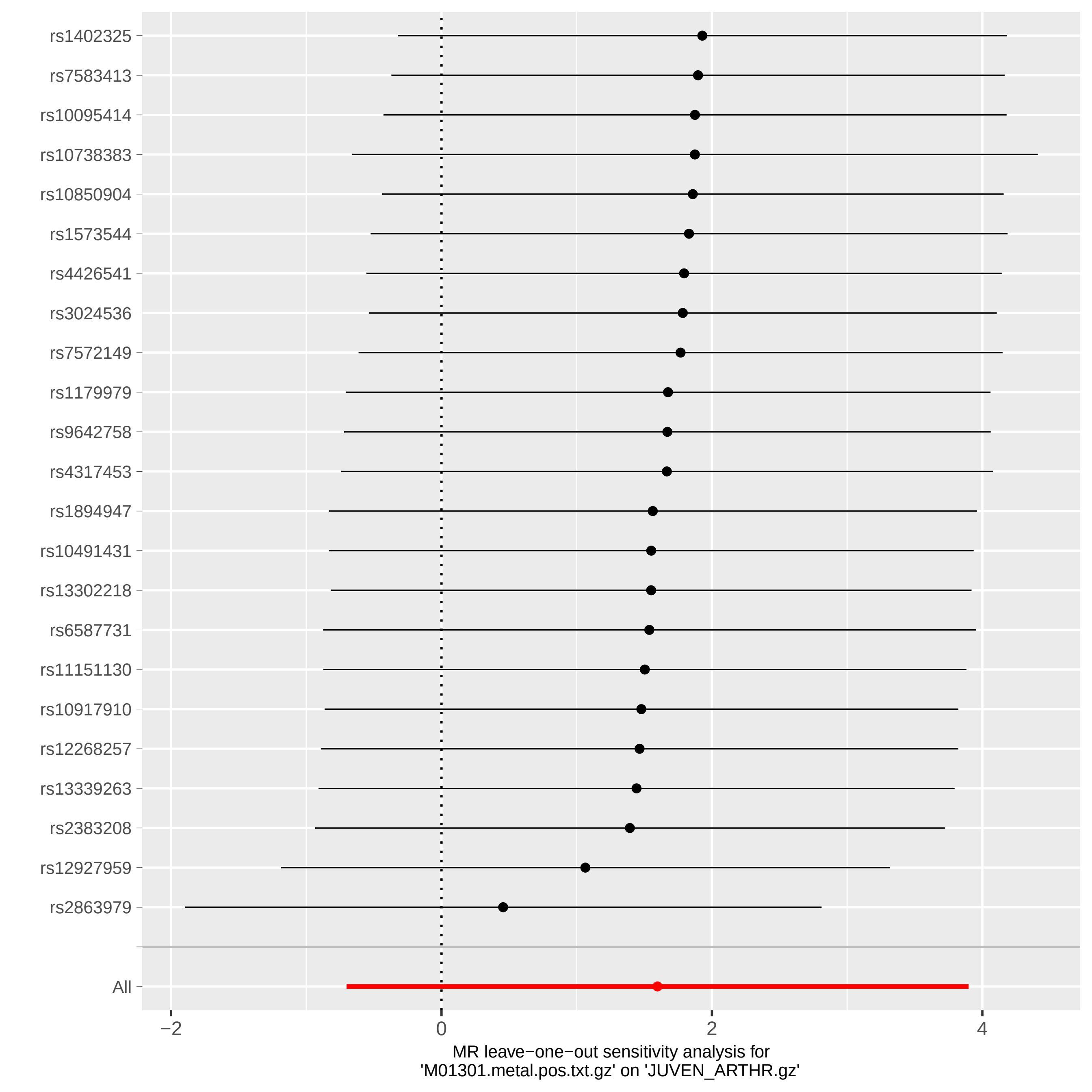
rs1402325
rs7583413
rs10095414
rs10738383
rs10850904
rs1573544
rs4426541
rs3024536
rs7572149
rs1179979
rs9642758
rs4317453
rs1894947
rs10491431
rs13302218
rs6587731
rs11151130
rs10917910
rs12268257
rs13339263
rs2383208
rs12927959
rs2863979
All
−2
0
2
4
MR leave−one−out sensitivity analysis for
'M01301.metal.pos.txt.gz' on 'JUVEN_ARTHR.gz'

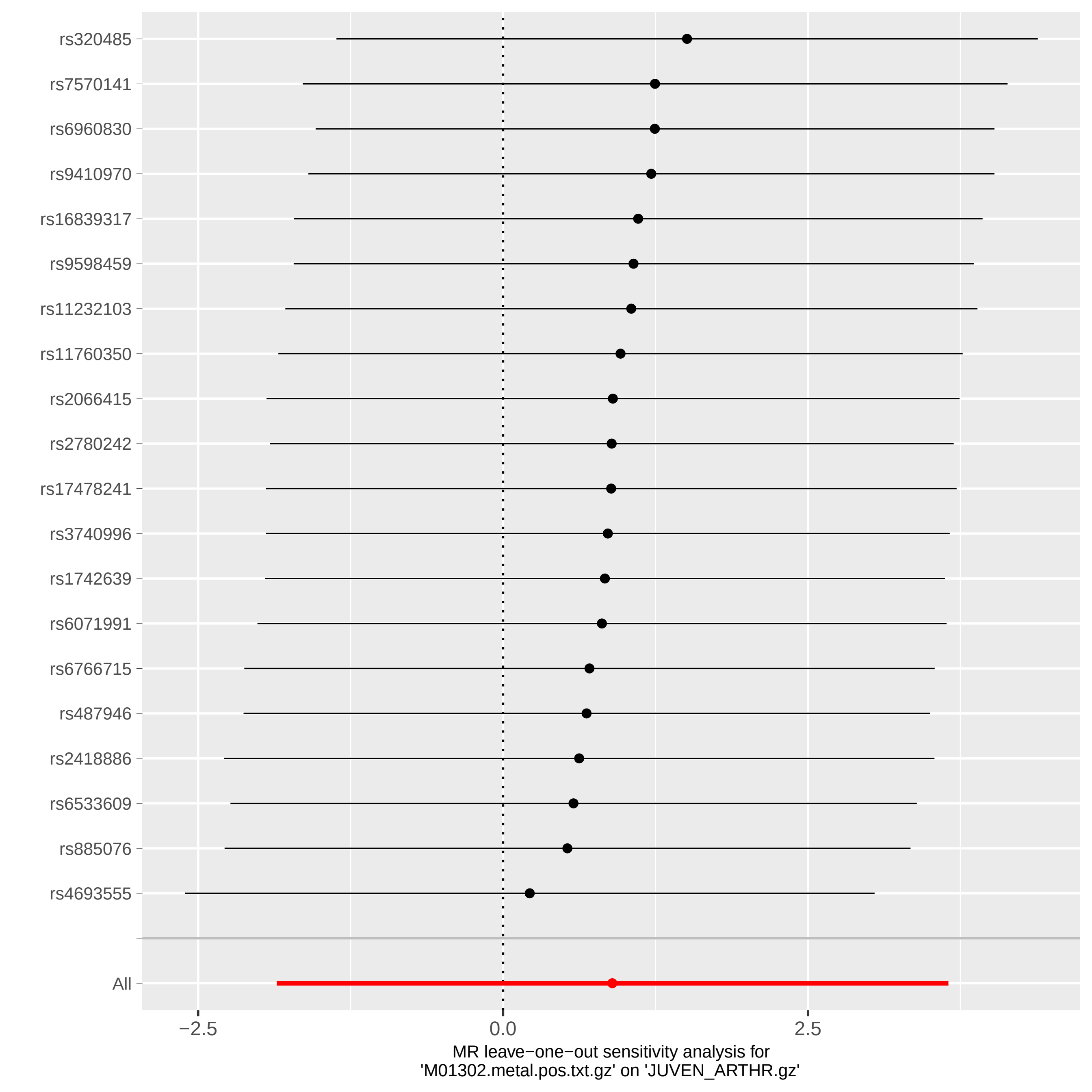

rs320485
rs7570141
rs6960830
rs9410970
rs16839317
rs9598459
rs11232103
rs11760350
rs2066415
rs2780242
rs17478241
rs3740996
rs1742639
rs6071991
rs6766715
rs487946
rs2418886
rs6533609
rs885076
rs4693555
All
−2.5
0.0
2.5
MR leave−one−out sensitivity analysis for
'M01302.metal.pos.txt.gz' on 'JUVEN_ARTHR.gz'

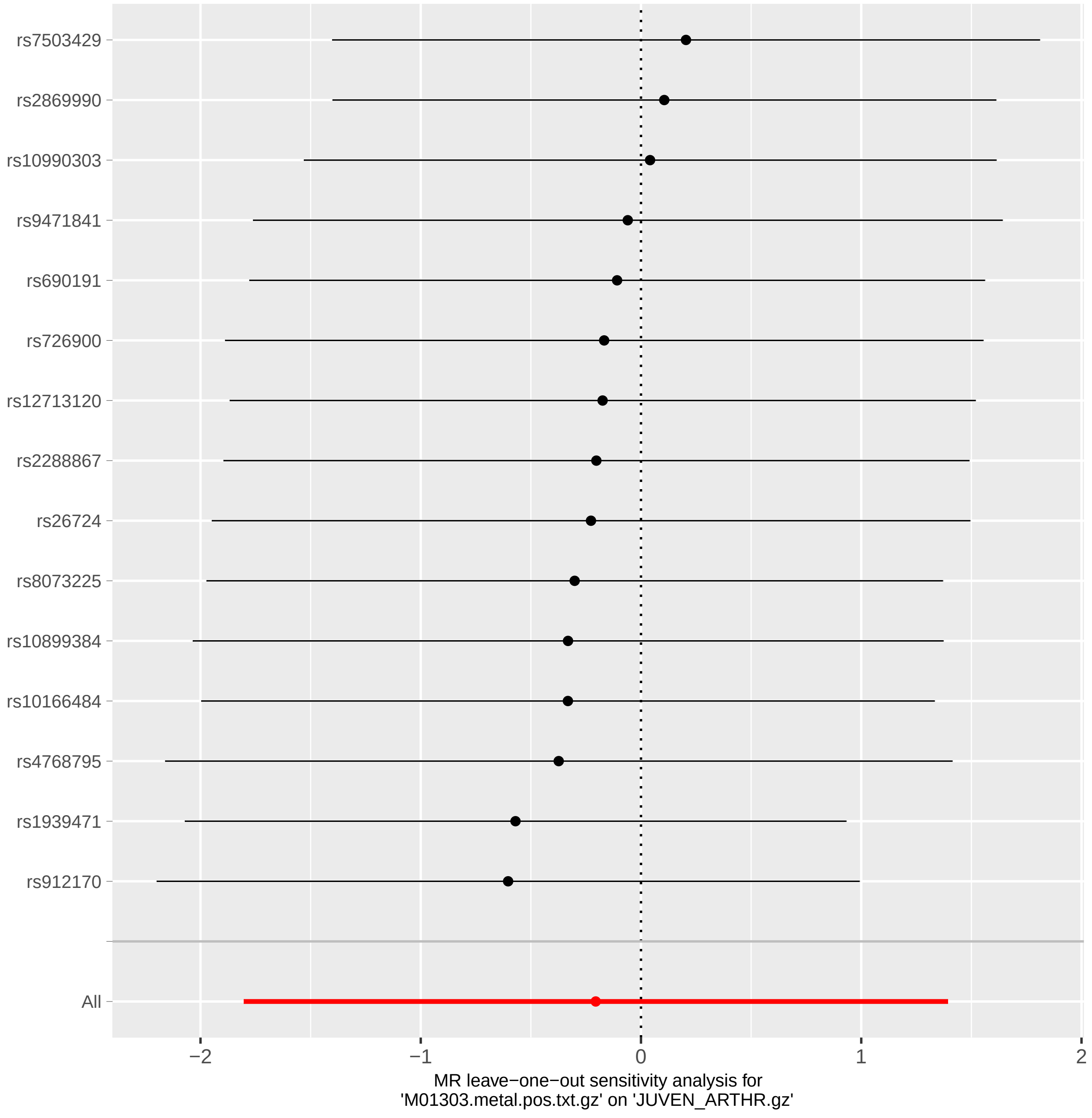
rs7503429
rs2869990
rs10990303
rs9471841
rs690191
rs726900
rs12713120
rs2288867
rs26724
rs8073225
rs10899384
rs10166484
rs4768795
rs1939471
rs912170
All
−2
−1
0
1
2
MR leave−one−out sensitivity analysis for
'M01303.metal.pos.txt.gz' on 'JUVEN_ARTHR.gz'

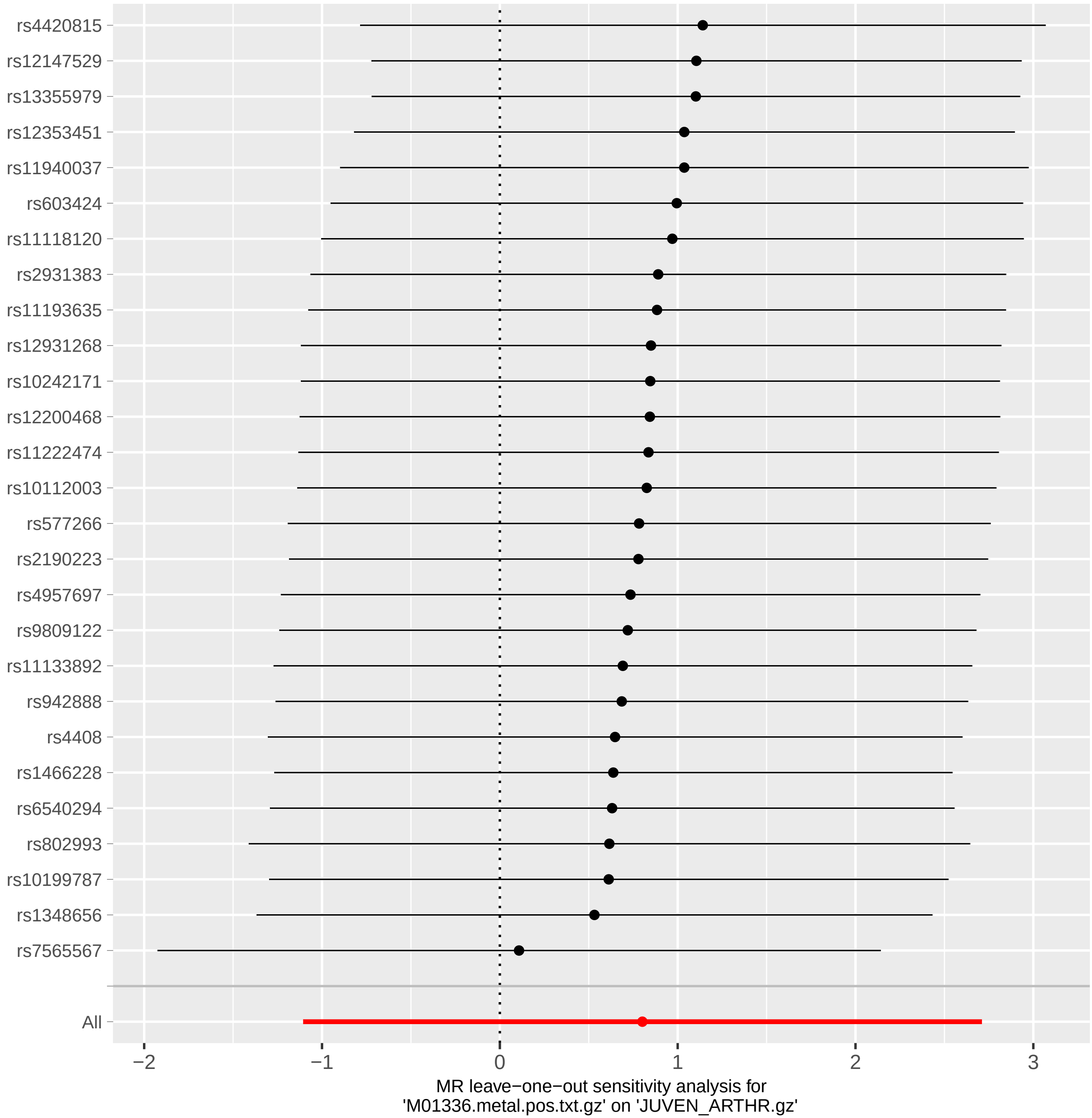
rs4420815
rs12147529
rs13355979
rs12353451
rs11940037
rs603424
rs11118120
rs2931383
rs11193635
rs12931268
rs10242171
rs12200468
rs11222474
rs10112003
rs577266
rs2190223
rs4957697
rs9809122
rs11133892
rs942888
rs4408
rs1466228
rs6540294
rs802993
rs10199787
rs1348656
rs7565567
All
−2
−1
0
1
2
3
MR leave−one−out sensitivity analysis for
'M01336.metal.pos.txt.gz' on 'JUVEN_ARTHR.gz'

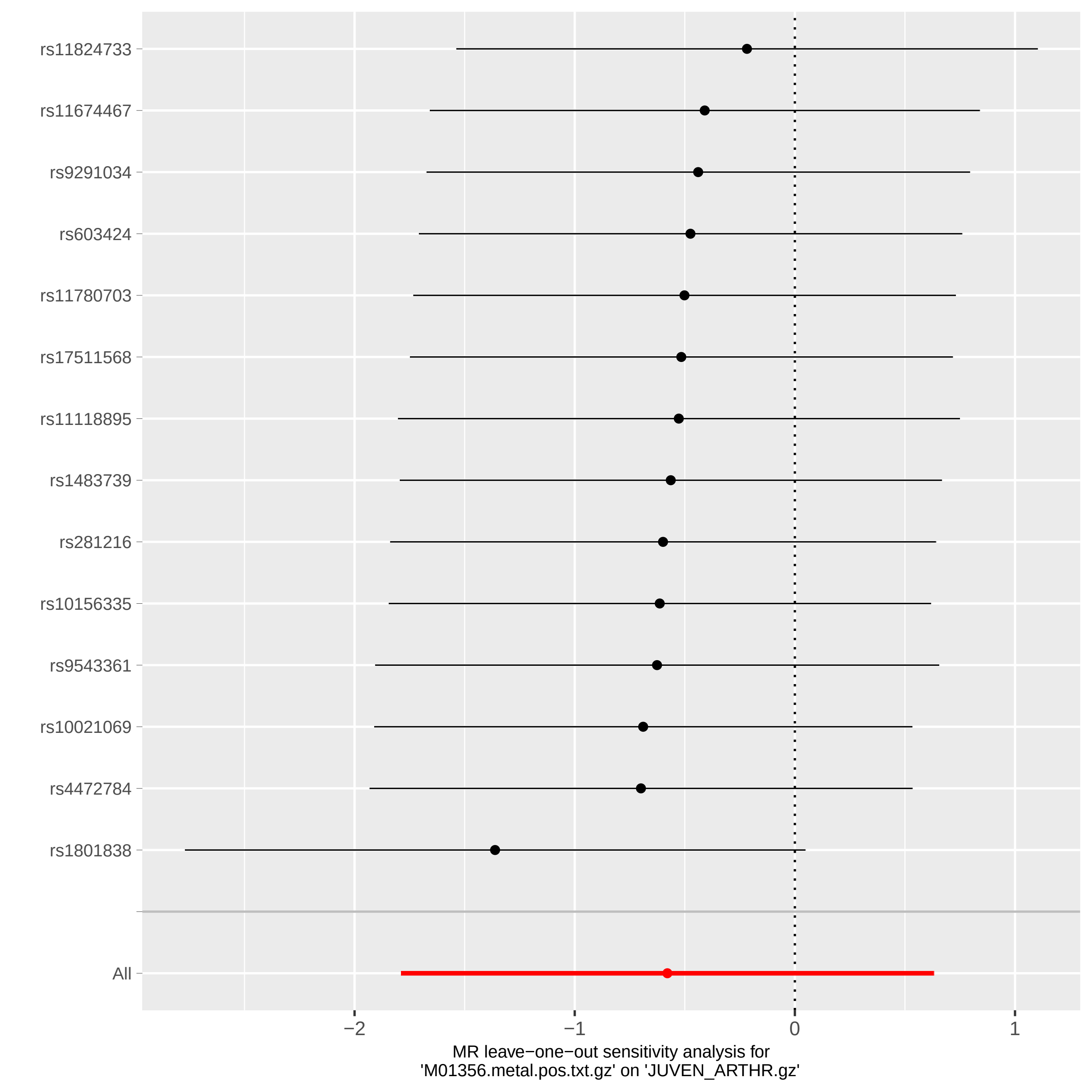

rs11824733
rs11674467
rs9291034
rs603424
rs11780703
rs17511568
rs11118895
rs1483739
rs281216
rs10156335
rs9543361
rs10021069
rs4472784
rs1801838
All
−2
−1
0
1
MR leave−one−out sensitivity analysis for
'M01356.metal.pos.txt.gz' on 'JUVEN_ARTHR.gz'

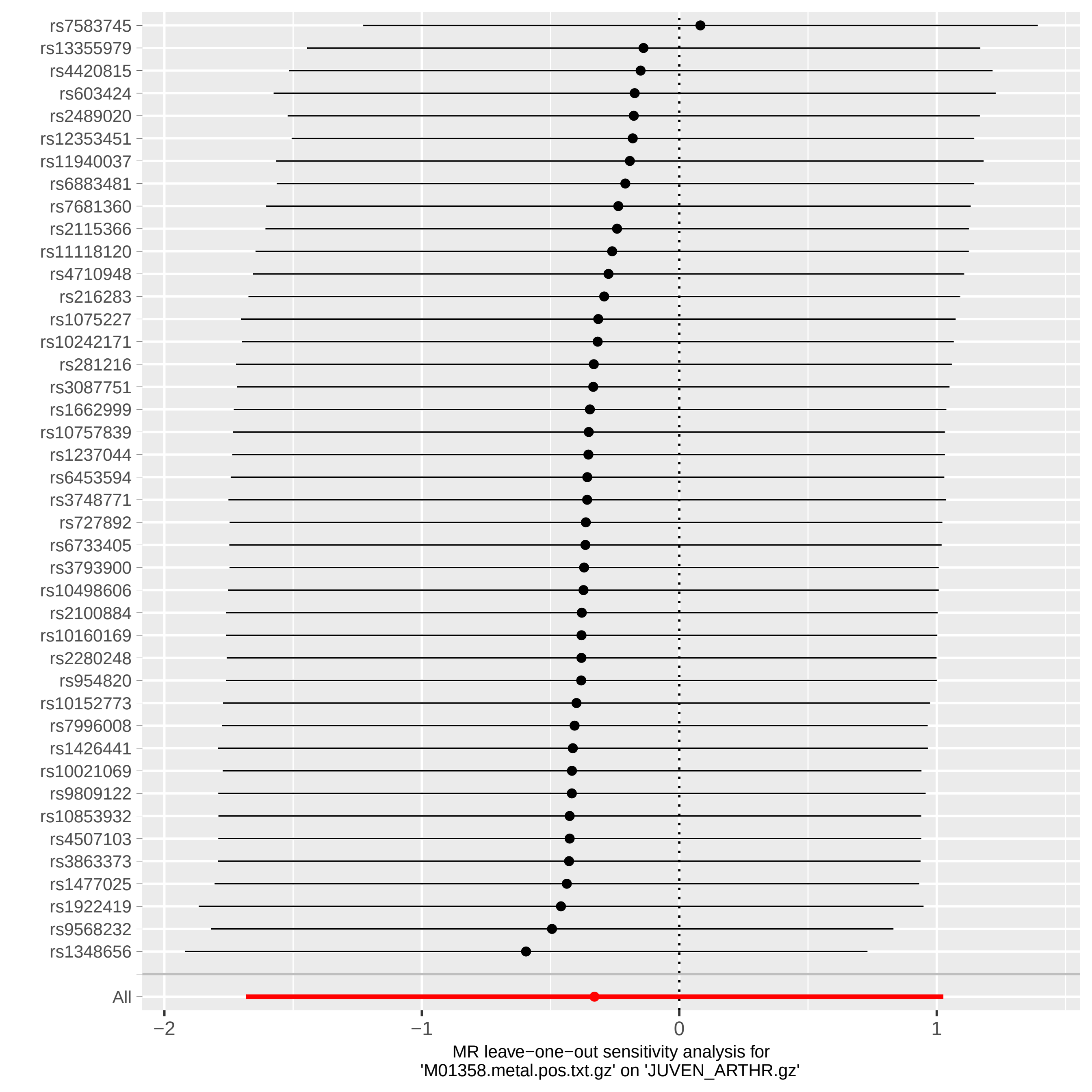

rs7583745
rs13355979
rs4420815
rs603424
rs2489020
rs12353451
rs11940037
rs6883481
rs7681360
rs2115366
rs11118120
rs4710948
rs216283
rs1075227
rs10242171
rs281216
rs3087751
rs1662999
rs10757839
rs1237044
rs6453594
rs3748771
rs727892
rs6733405
rs3793900
rs10498606
rs2100884
rs10160169
rs2280248
rs954820
rs10152773
rs7996008
rs1426441
rs10021069
rs9809122
rs10853932
rs4507103
rs3863373
rs1477025
rs1922419
rs9568232
rs1348656
All
−2
−1
0
1
MR leave−one−out sensitivity analysis for
'M01358.metal.pos.txt.gz' on 'JUVEN_ARTHR.gz'

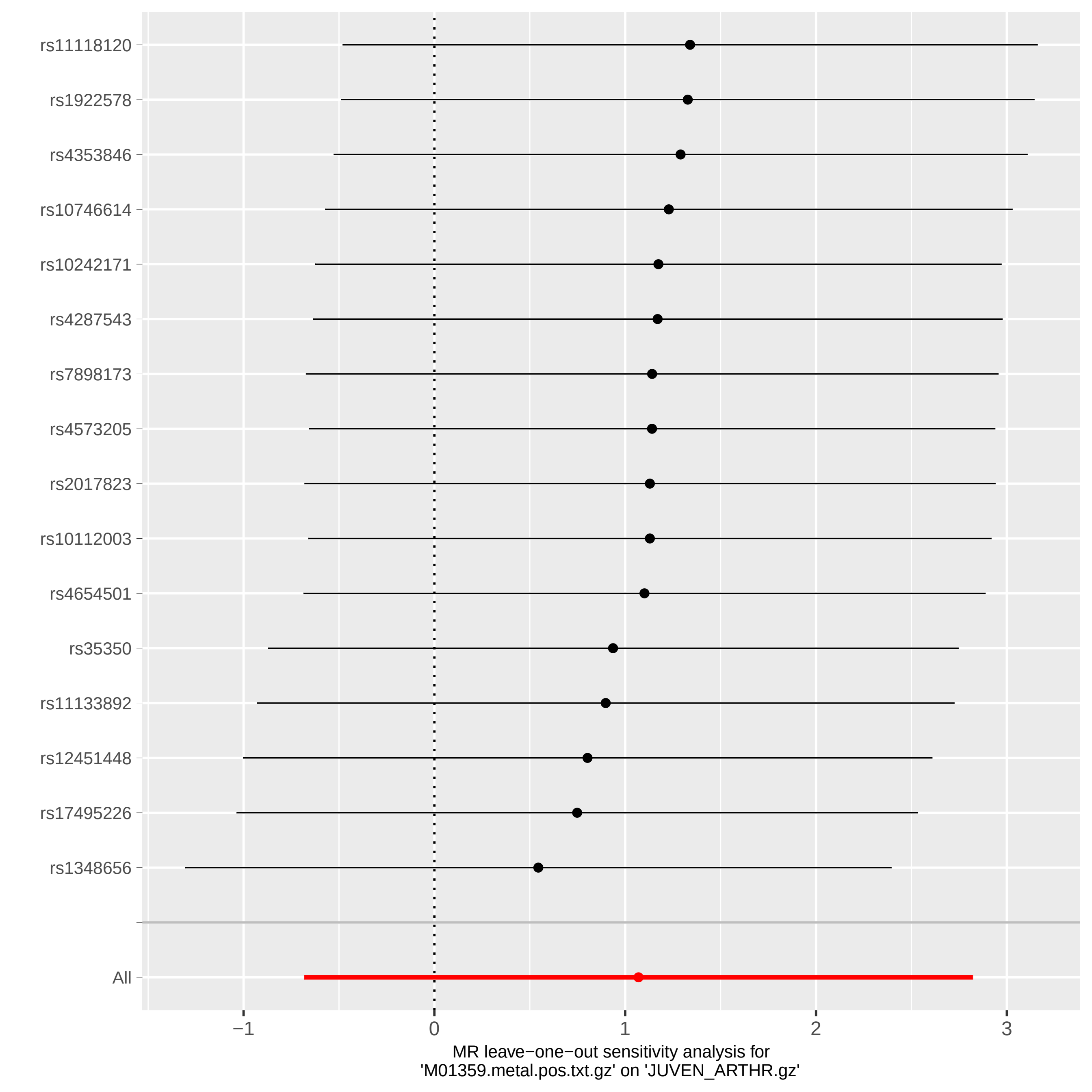
rs11118120
rs1922578
rs4353846
rs10746614
rs10242171
rs4287543
rs7898173
rs4573205
rs2017823
rs10112003
rs4654501
rs35350
rs11133892
rs12451448
rs17495226
rs1348656
All
−1
0
1
2
3
MR leave−one−out sensitivity analysis for
'M01359.metal.pos.txt.gz' on 'JUVEN_ARTHR.gz'

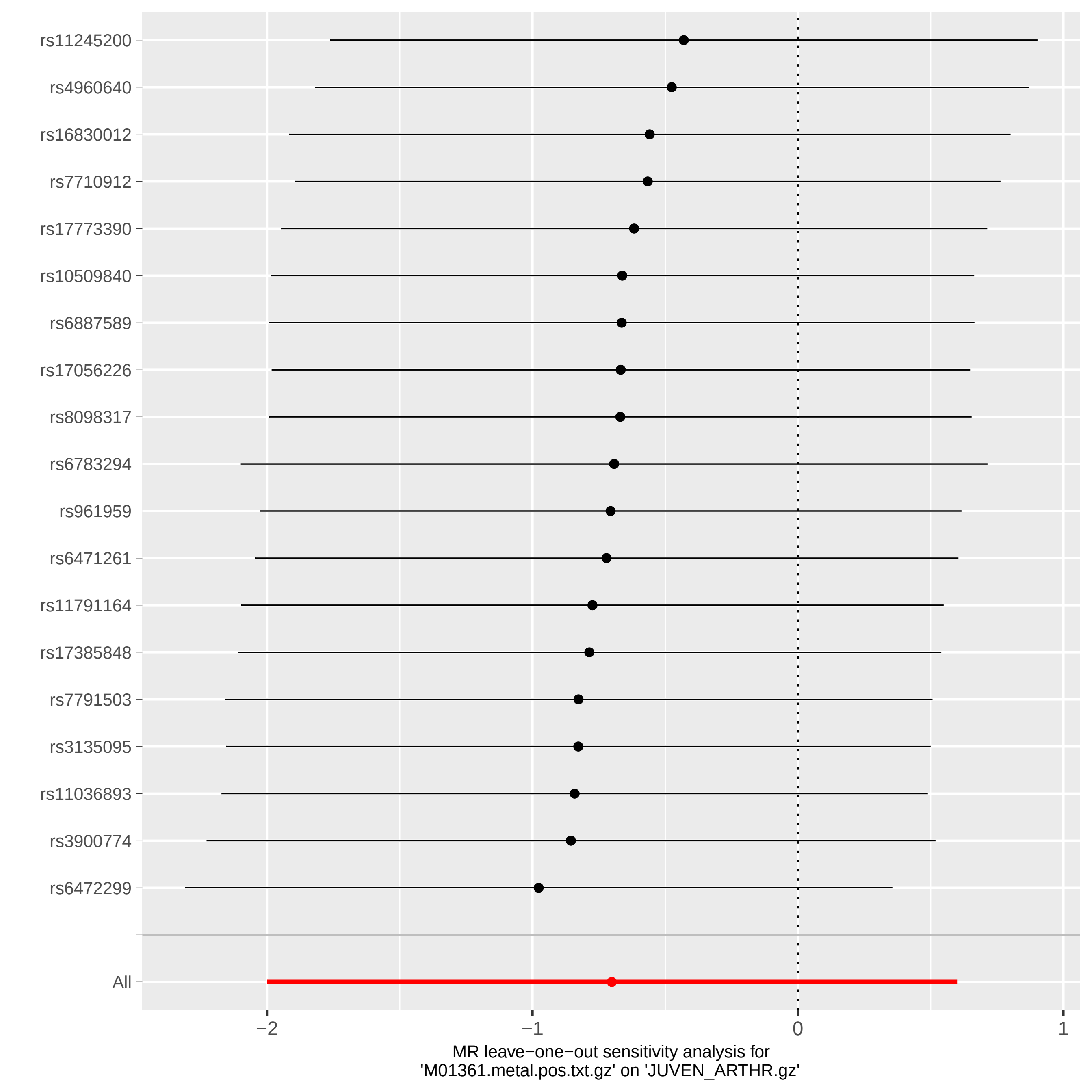
rs11245200
rs4960640
rs16830012
rs7710912
rs17773390
rs10509840
rs6887589
rs17056226
rs8098317
rs6783294
rs961959
rs6471261
rs11791164
rs17385848
rs7791503
rs3135095
rs11036893
rs3900774
rs6472299
All
−2
−1
0
1
MR leave−one−out sensitivity analysis for
'M01361.metal.pos.txt.gz' on 'JUVEN_ARTHR.gz'

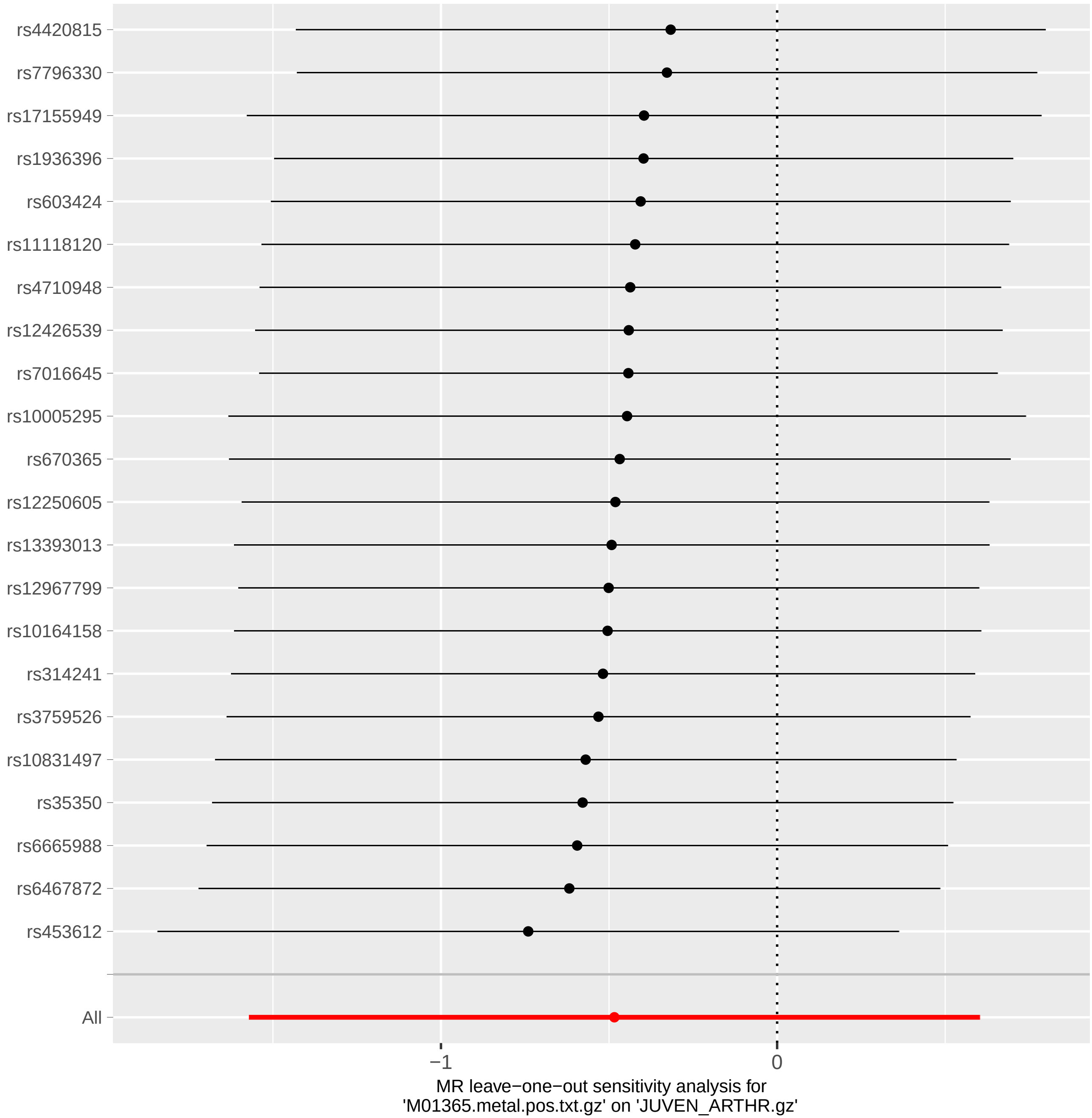

rs4420815
rs7796330
rs17155949
rs1936396
rs603424
rs11118120
rs4710948
rs12426539
rs7016645
rs10005295
rs670365
rs12250605
rs13393013
rs12967799
rs10164158
rs314241
rs3759526
rs10831497
rs35350
rs6665988
rs6467872
rs453612
All
−1
0
MR leave−one−out sensitivity analysis for
'M01365.metal.pos.txt.gz' on 'JUVEN_ARTHR.gz'

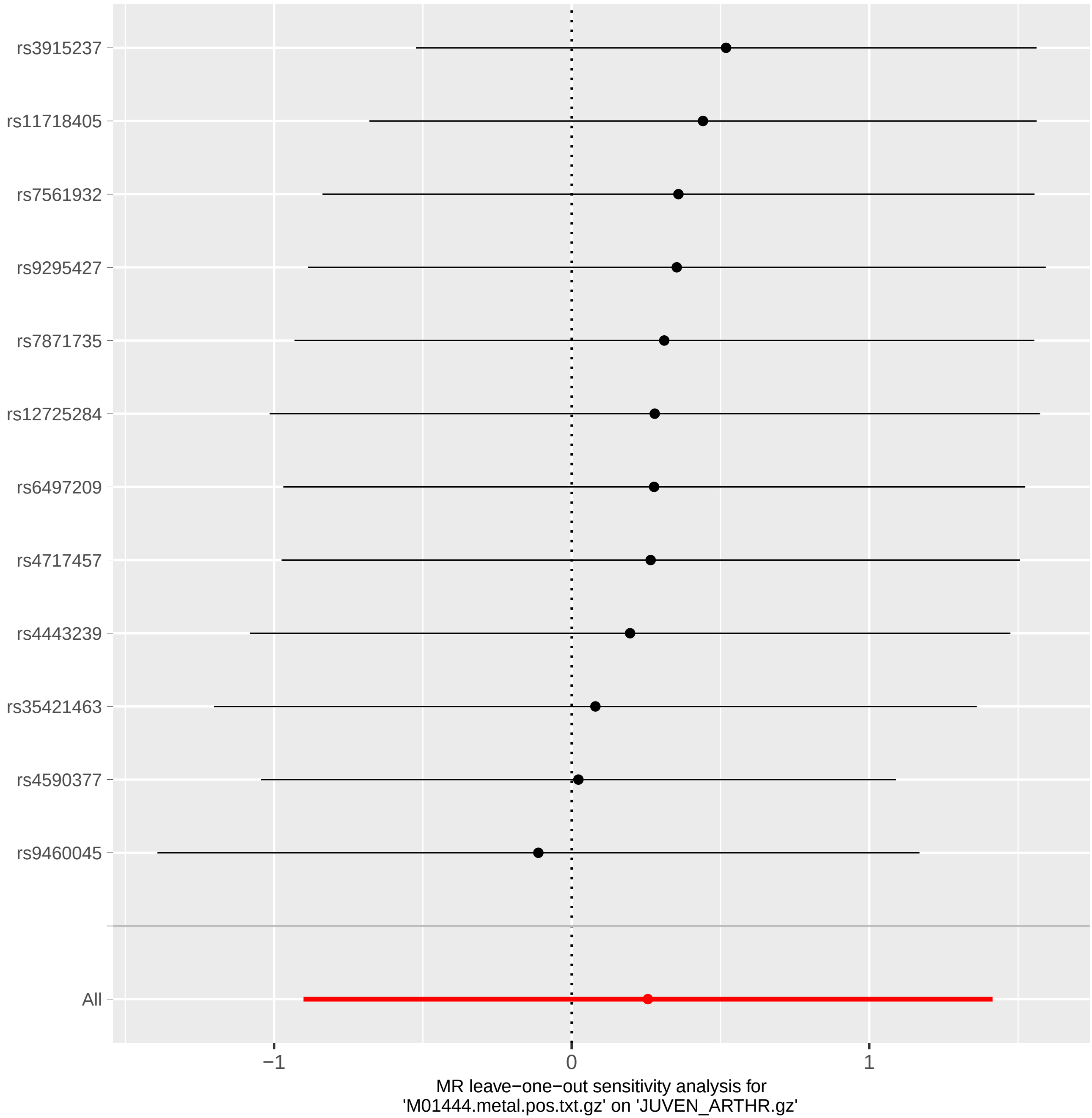
rs3915237
rs11718405
rs7561932
rs9295427
rs7871735
rs12725284
rs6497209
rs4717457
rs4443239
rs35421463
rs4590377
rs9460045
All
−1
0
1
MR leave−one−out sensitivity analysis for
'M01444.metal.pos.txt.gz' on 'JUVEN_ARTHR.gz'

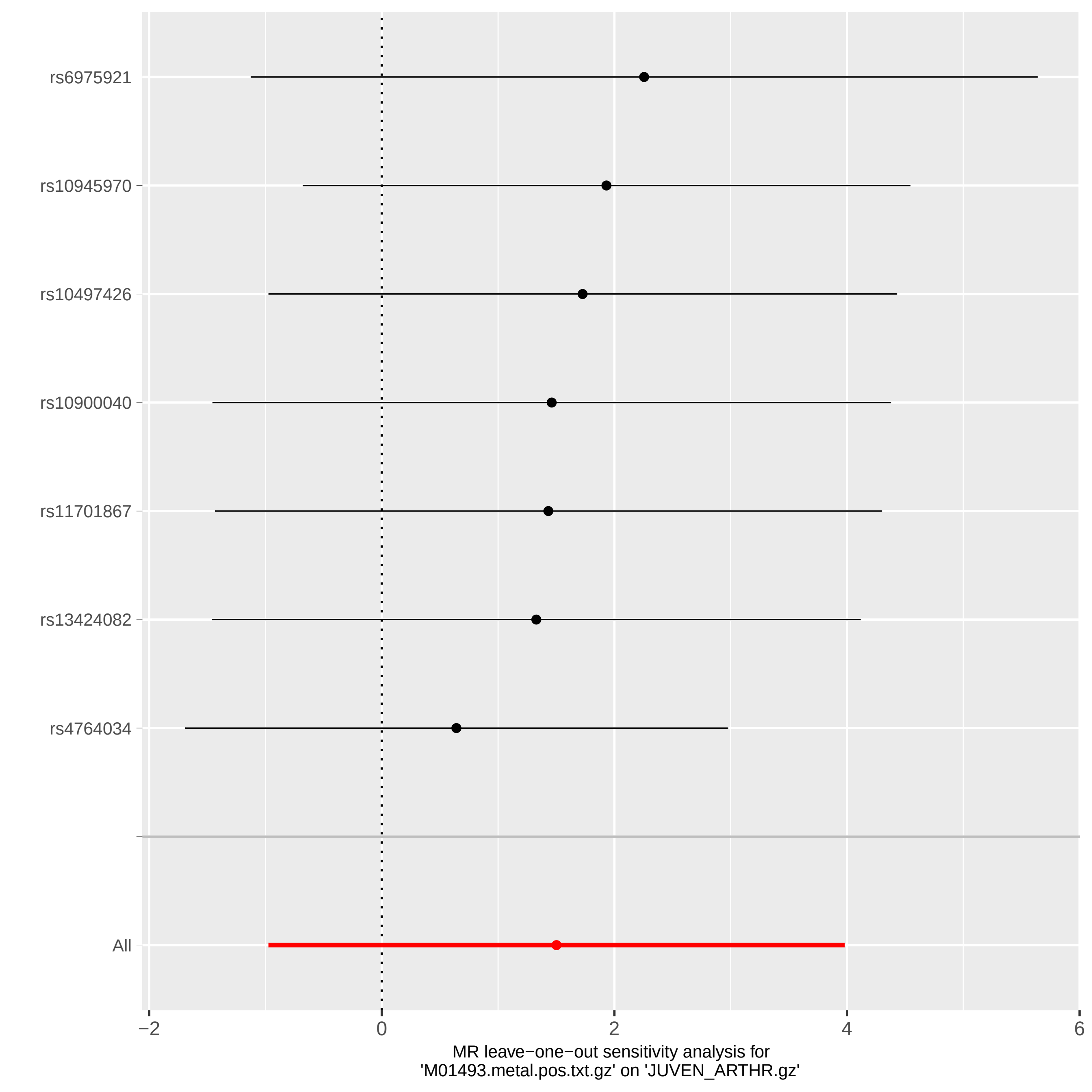
rs6975921
rs10945970
rs10497426
rs10900040
rs11701867
rs13424082
rs4764034
All
−2
0
2
4
6
MR leave−one−out sensitivity analysis for
'M01493.metal.pos.txt.gz' on 'JUVEN_ARTHR.gz'

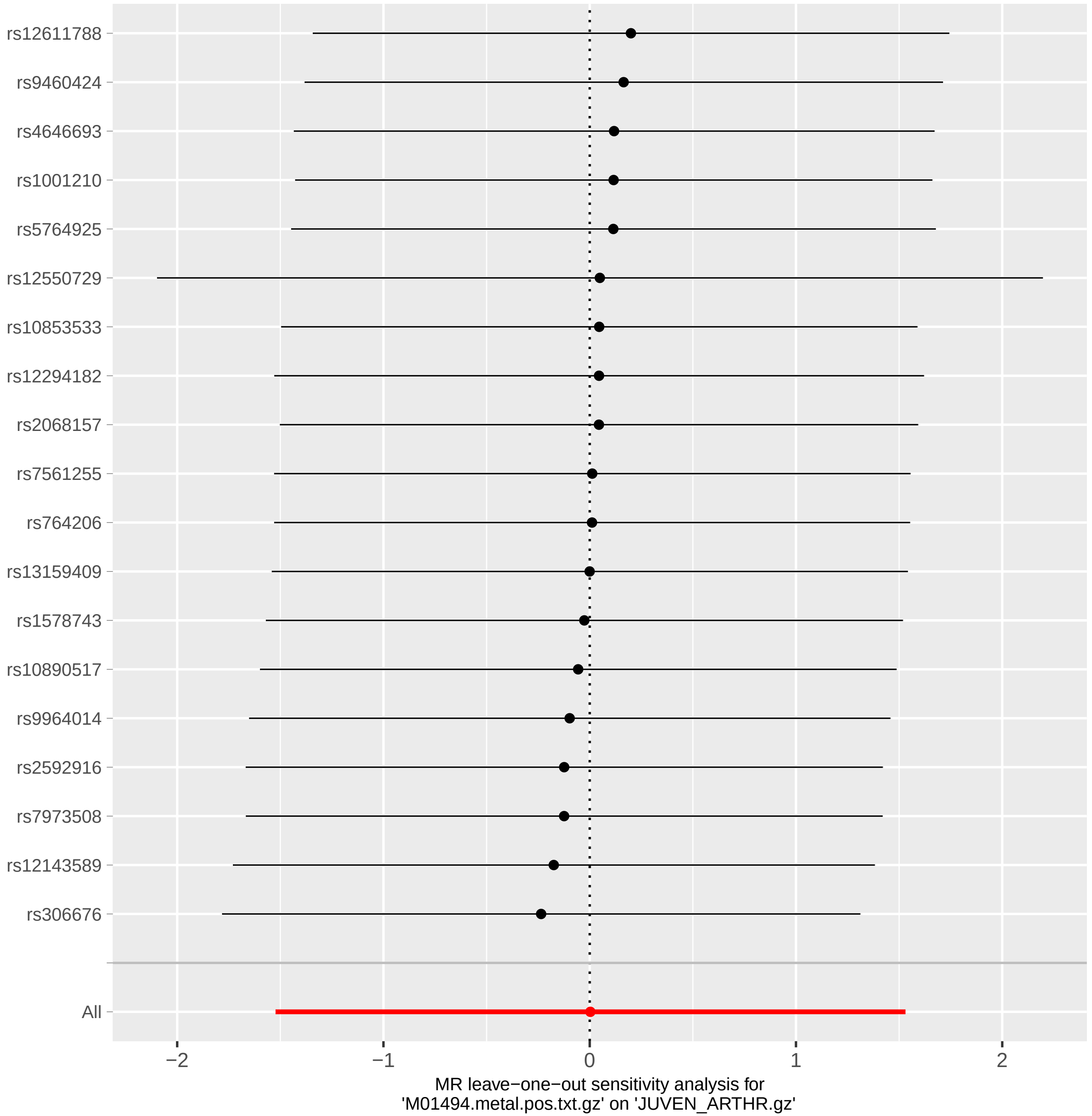
rs12611788
rs9460424
rs4646693
rs1001210
rs5764925
rs12550729
rs10853533
rs12294182
rs2068157
rs7561255
rs764206
rs13159409
rs1578743
rs10890517
rs9964014
rs2592916
rs7973508
rs12143589
rs306676
All
−2
−1
0
1
2
MR leave−one−out sensitivity analysis for
'M01494.metal.pos.txt.gz' on 'JUVEN_ARTHR.gz'

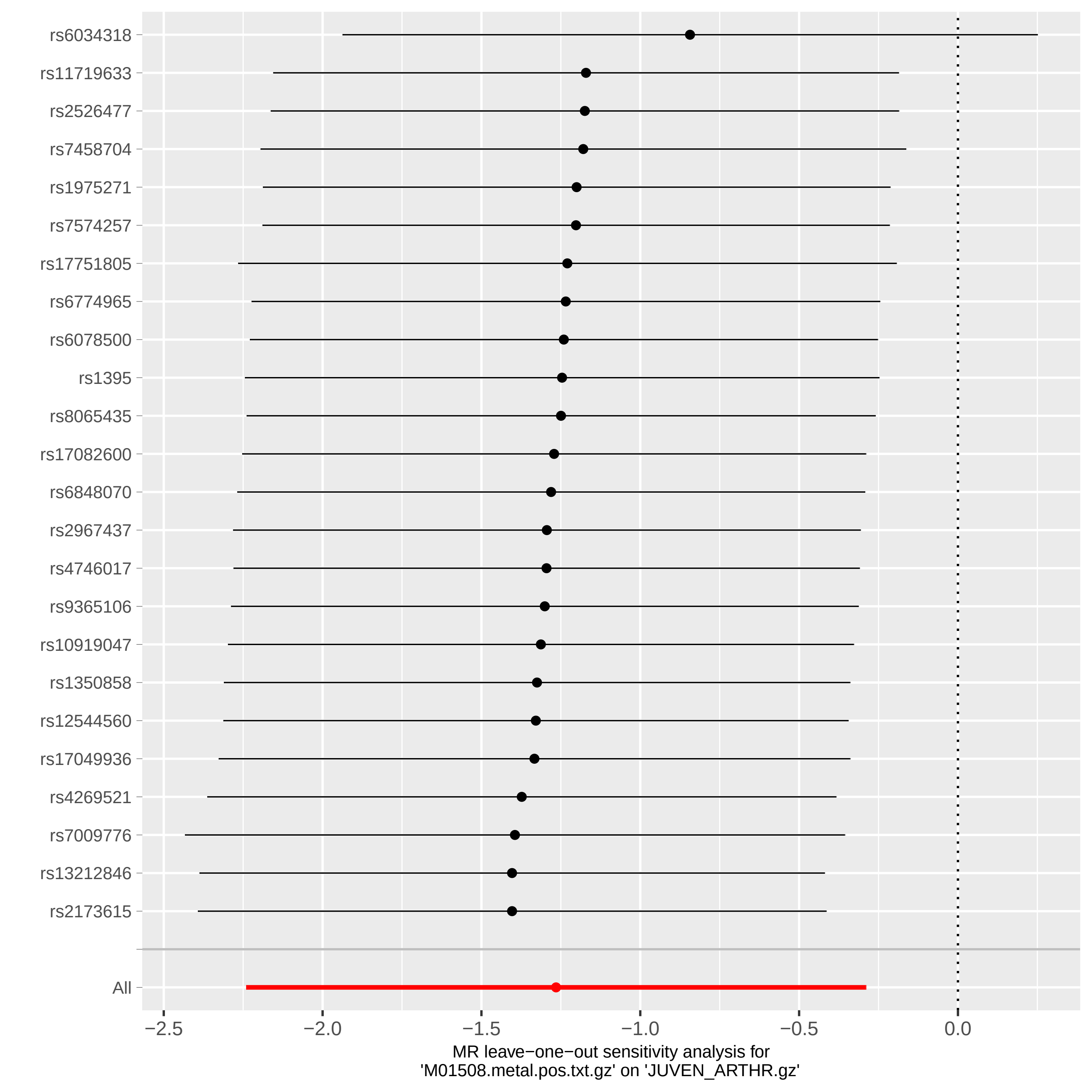
rs6034318
rs11719633
rs2526477
rs7458704
rs1975271
rs7574257
rs17751805
rs6774965
rs6078500
rs1395
rs8065435
rs17082600
rs6848070
rs2967437
rs4746017
rs9365106
rs10919047
rs1350858
rs12544560
rs17049936
rs4269521
rs7009776
rs13212846
rs2173615
All
−2.5
−2.0
−1.5
−1.0
−0.5
0.0
MR leave−one−out sensitivity analysis for
'M01508.metal.pos.txt.gz' on 'JUVEN_ARTHR.gz'

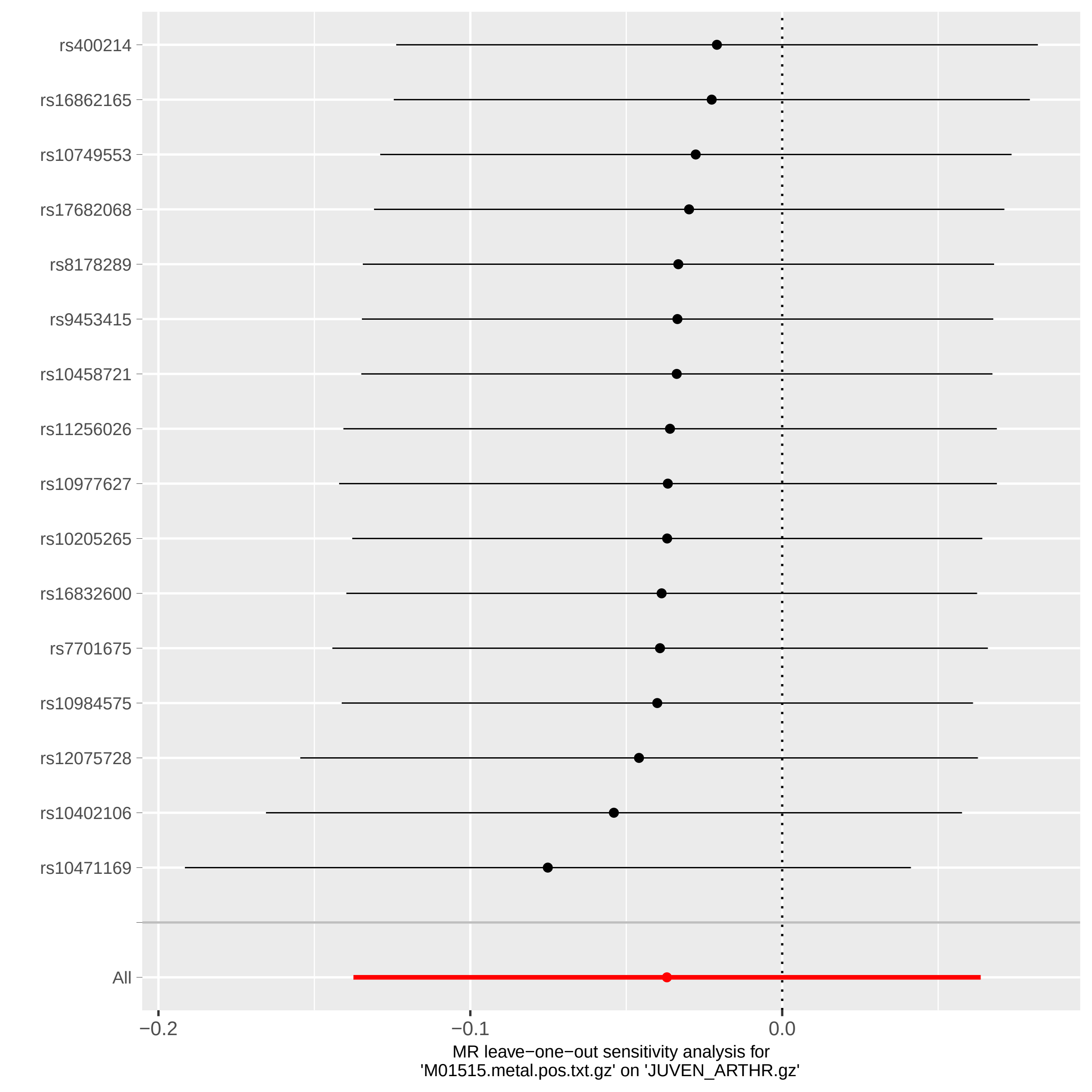
rs400214
rs16862165
rs10749553
rs17682068
rs8178289
rs9453415
rs10458721
rs11256026
rs10977627
rs10205265
rs16832600
rs7701675
rs10984575
rs12075728
rs10402106
rs10471169
All
−0.2
−0.1
0.0
MR leave−one−out sensitivity analysis for
'M01515.metal.pos.txt.gz' on 'JUVEN_ARTHR.gz'

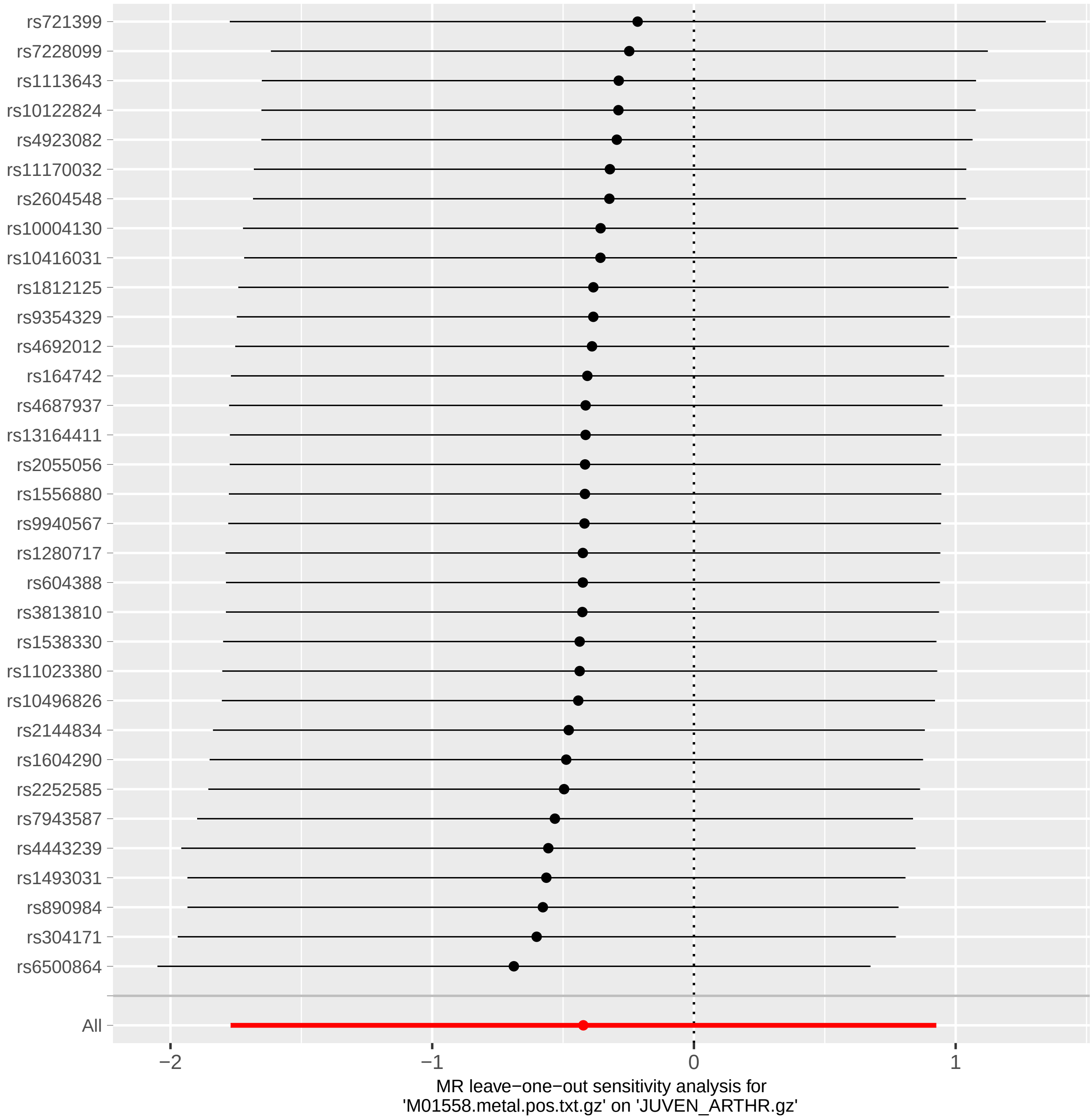
rs721399
rs7228099
rs1113643
rs10122824
rs4923082
rs11170032
rs2604548
rs10004130
rs10416031
rs1812125
rs9354329
rs4692012
rs164742
rs4687937
rs13164411
rs2055056
rs1556880
rs9940567
rs1280717
rs604388
rs3813810
rs1538330
rs11023380
rs10496826
rs2144834
rs1604290
rs2252585
rs7943587
rs4443239
rs1493031
rs890984
rs304171
rs6500864
All
−2
−1
0
1
MR leave−one−out sensitivity analysis for
'M01558.metal.pos.txt.gz' on 'JUVEN_ARTHR.gz'

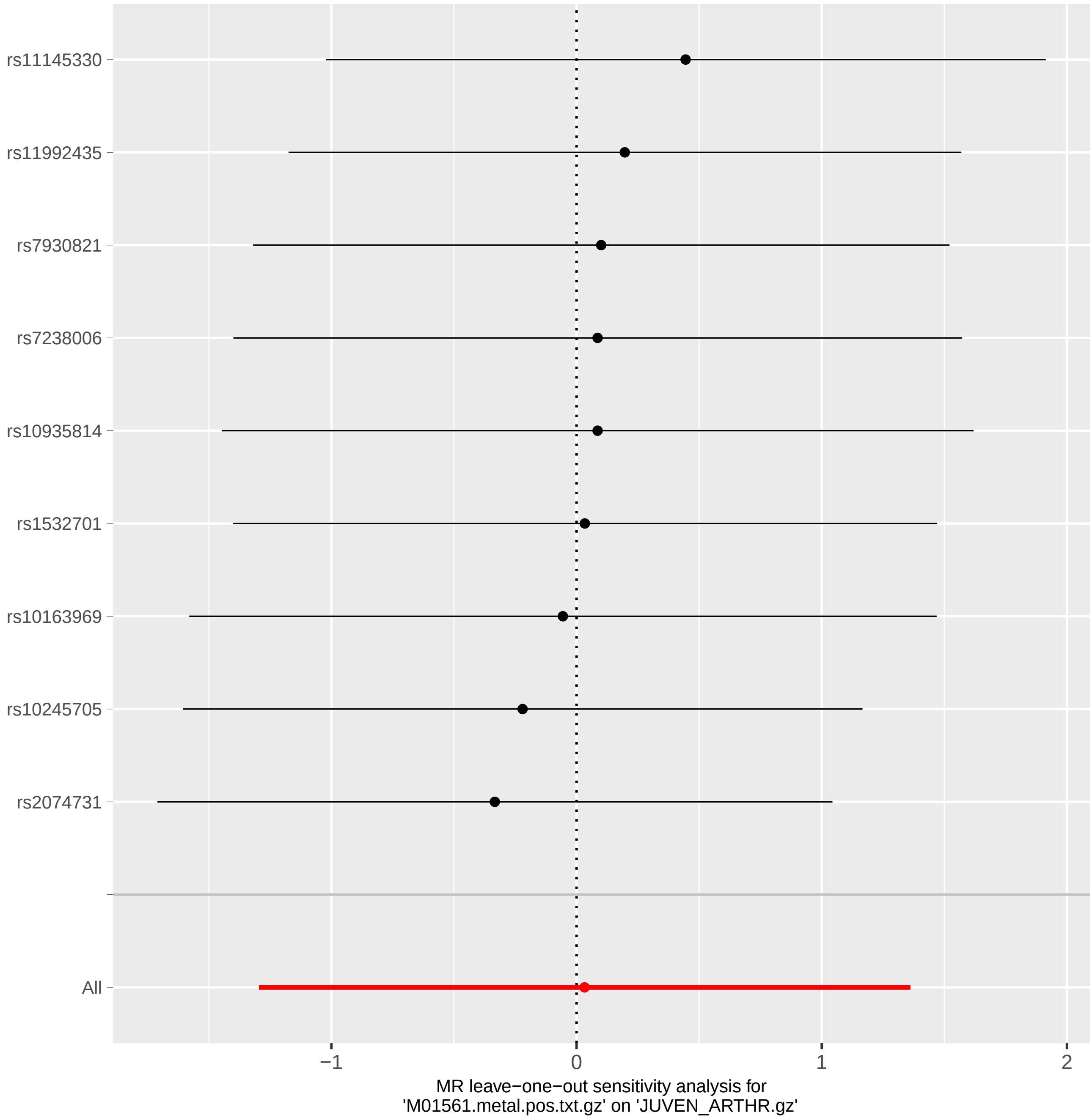
rs11145330
rs11992435
rs7930821
rs7238006
rs10935814
rs1532701
rs10163969
rs10245705
rs2074731
All
−1
0
1
2
MR leave−one−out sensitivity analysis for
'M01561.metal.pos.txt.gz' on 'JUVEN_ARTHR.gz'

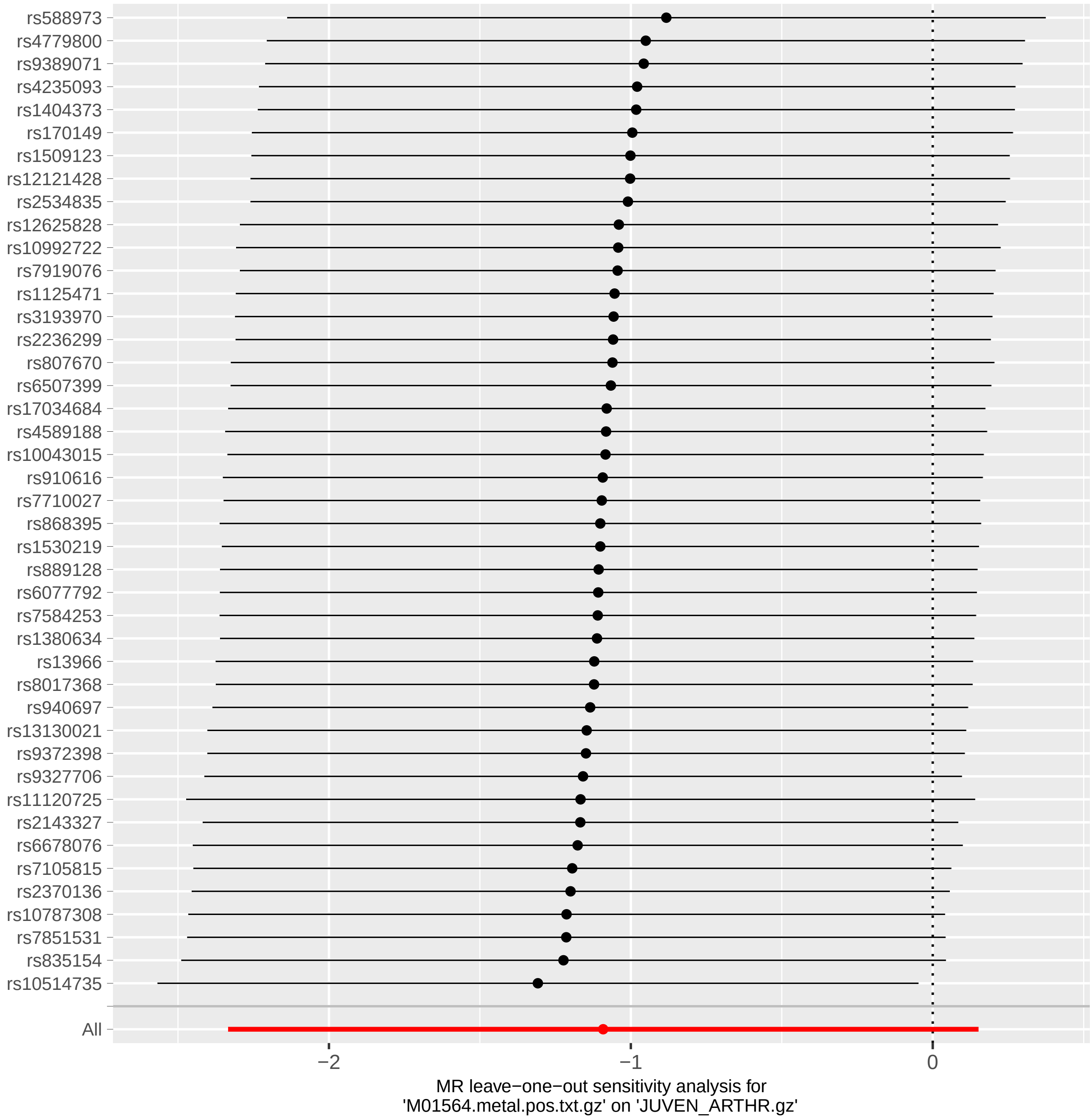
rs588973
rs4779800
rs9389071
rs4235093
rs1404373
rs170149
rs1509123
rs12121428
rs2534835
rs12625828
rs10992722
rs7919076
rs1125471
rs3193970
rs2236299
rs807670
rs6507399
rs17034684
rs4589188
rs10043015
rs910616
rs7710027
rs868395
rs1530219
rs889128
rs6077792
rs7584253
rs1380634
rs13966
rs8017368
rs940697
rs13130021
rs9372398
rs9327706
rs11120725
rs2143327
rs6678076
rs7105815
rs2370136
rs10787308
rs7851531
rs835154
rs10514735
All
−2
−1
0
MR leave−one−out sensitivity analysis for
'M01564.metal.pos.txt.gz' on 'JUVEN_ARTHR.gz'

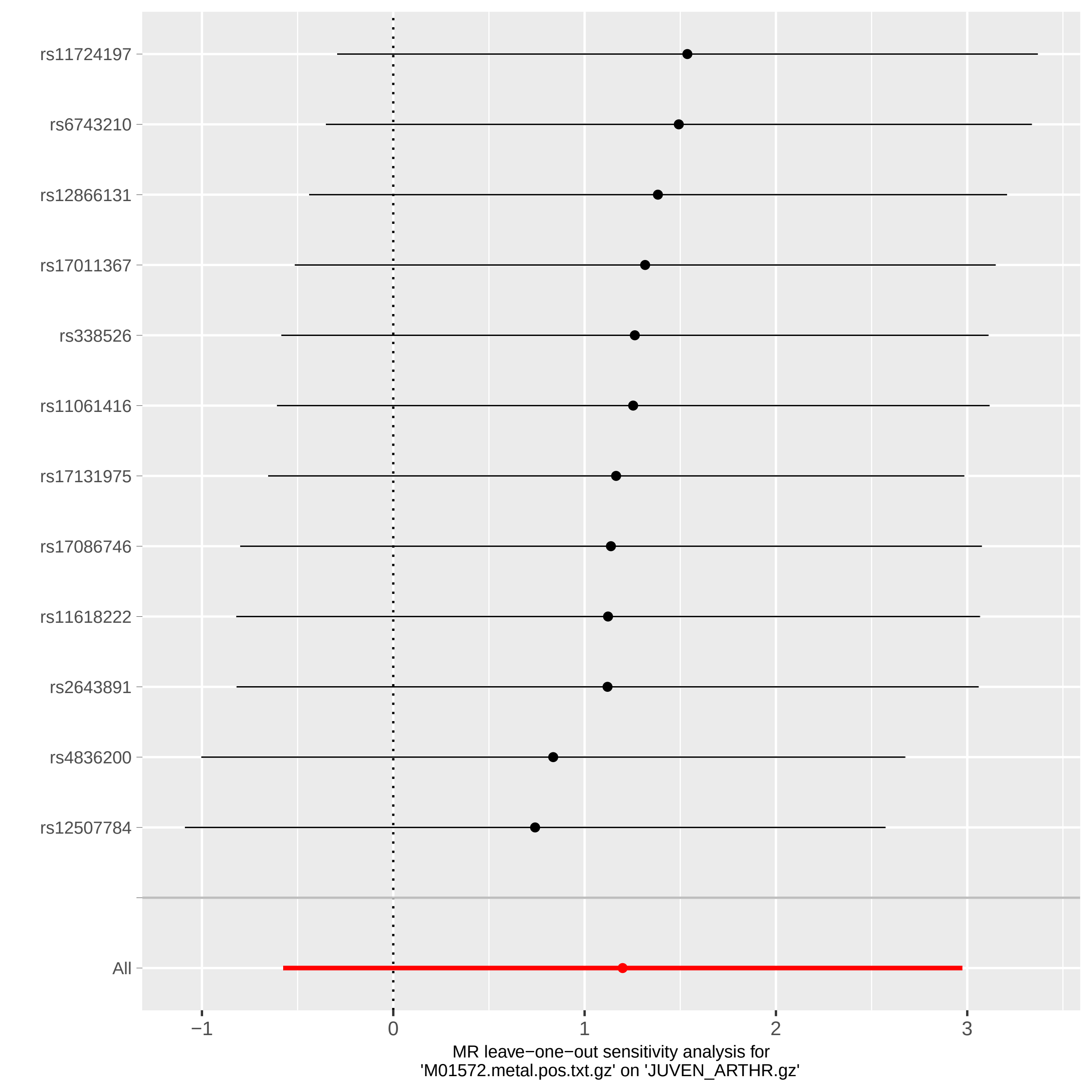
rs11724197
rs6743210
rs12866131
rs17011367
rs338526
rs11061416
rs17131975
rs17086746
rs11618222
rs2643891
rs4836200
rs12507784
All
−1
0
1
2
3
MR leave−one−out sensitivity analysis for
'M01572.metal.pos.txt.gz' on 'JUVEN_ARTHR.gz'

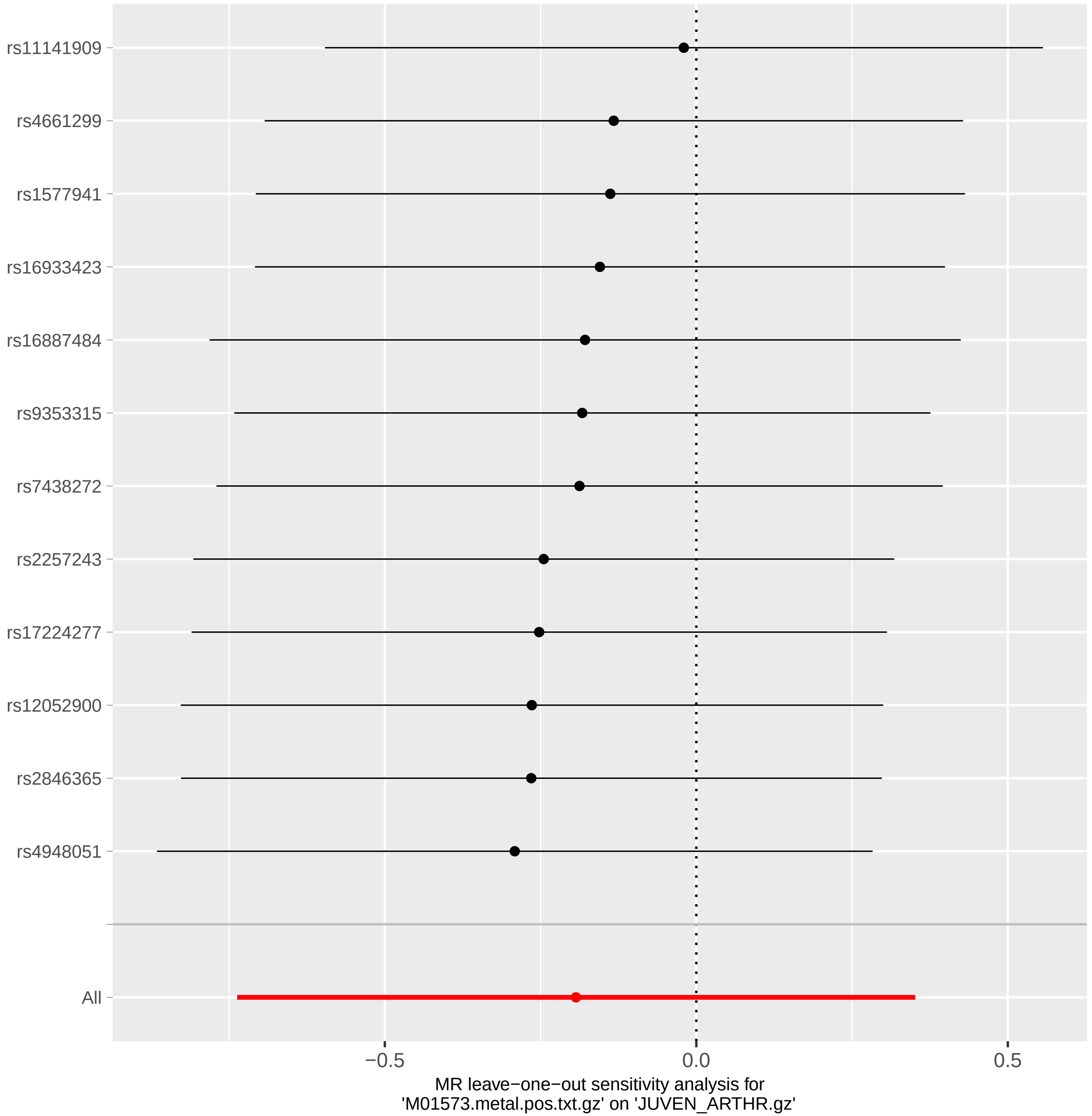
rs11141909
rs4661299
rs1577941
rs16933423
rs16887484
rs9353315
rs7438272
rs2257243
rs17224277
rs12052900
rs2846365
rs4948051
All
−0.5
0.0
0.5
MR leave−one−out sensitivity analysis for
'M01573.metal.pos.txt.gz' on 'JUVEN_ARTHR.gz'

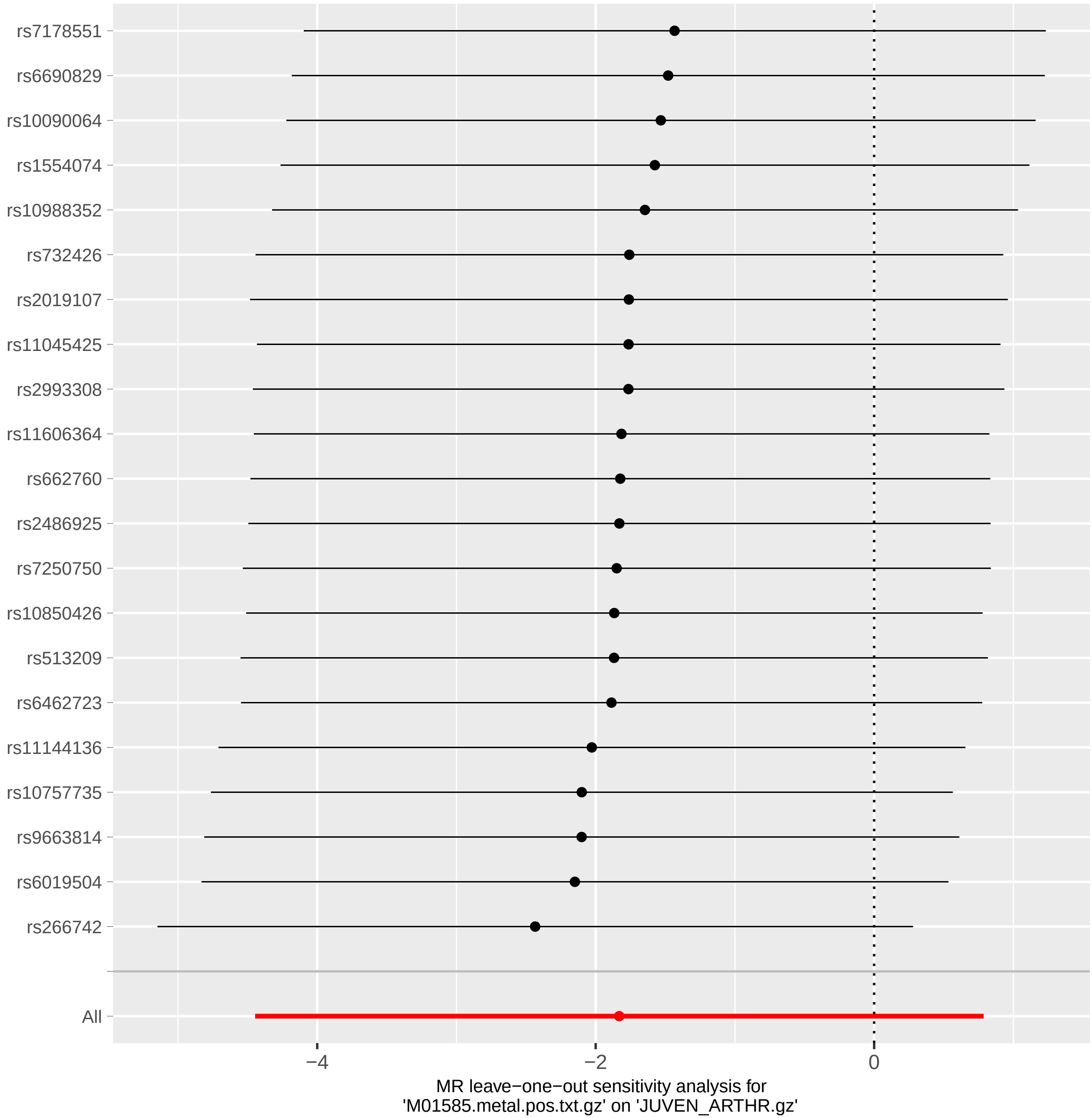

rs7178551
rs6690829
rs10090064
rs1554074
rs10988352
rs732426
rs2019107
rs11045425
rs2993308
rs11606364
rs662760
rs2486925
rs7250750
rs10850426
rs513209
rs6462723
rs11144136
rs10757735
rs9663814
rs6019504
rs266742
All
−4
−2
0
MR leave−one−out sensitivity analysis for
'M01585.metal.pos.txt.gz' on 'JUVEN_ARTHR.gz'

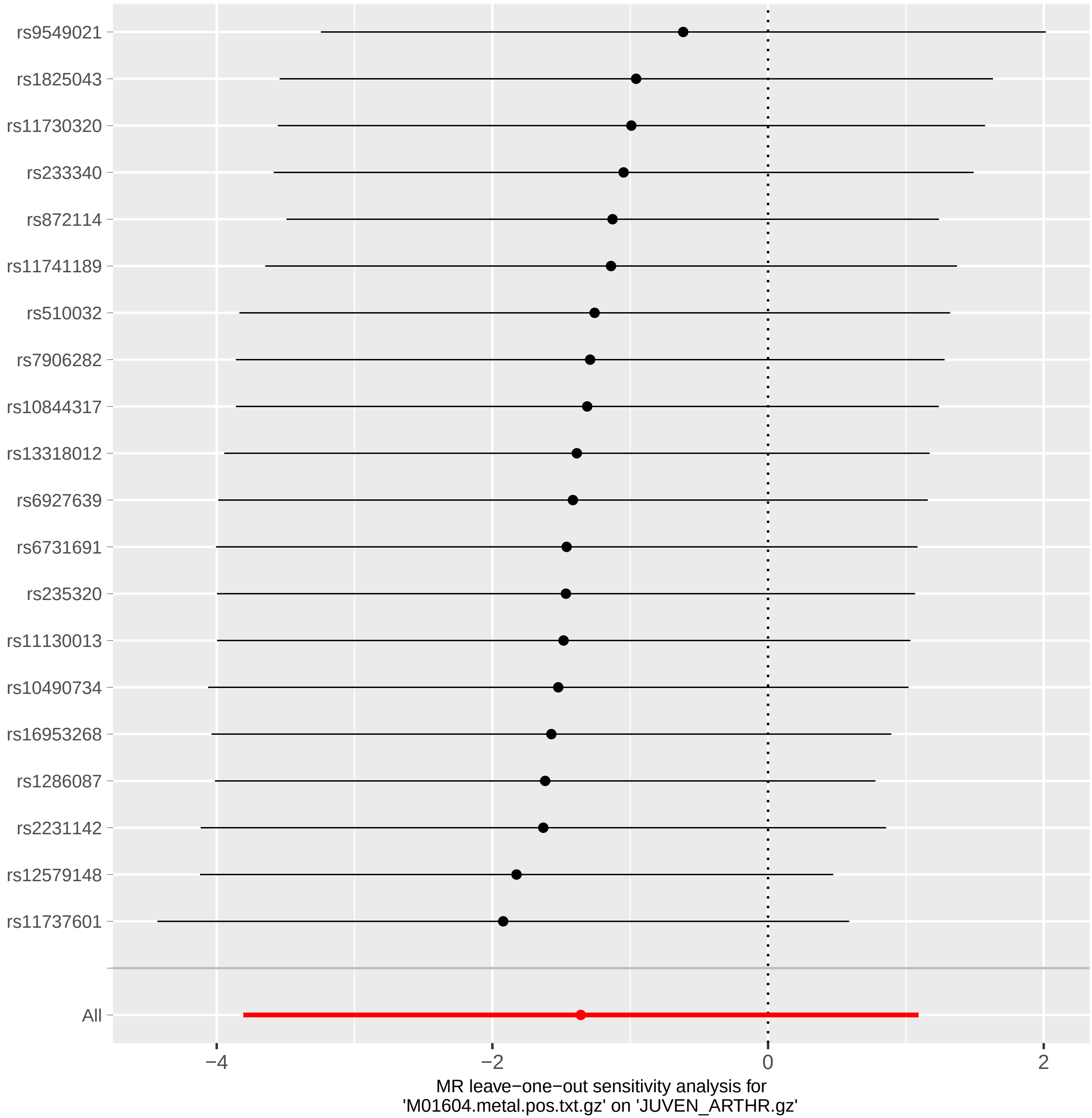

rs9549021
rs1825043
rs11730320
rs233340
rs872114
rs11741189
rs510032
rs7906282
rs10844317
rs13318012
rs6927639
rs6731691
rs235320
rs11130013
rs10490734
rs16953268
rs1286087
rs2231142
rs12579148
rs11737601
All
−4
−2
0
2
MR leave−one−out sensitivity analysis for
'M01604.metal.pos.txt.gz' on 'JUVEN_ARTHR.gz'

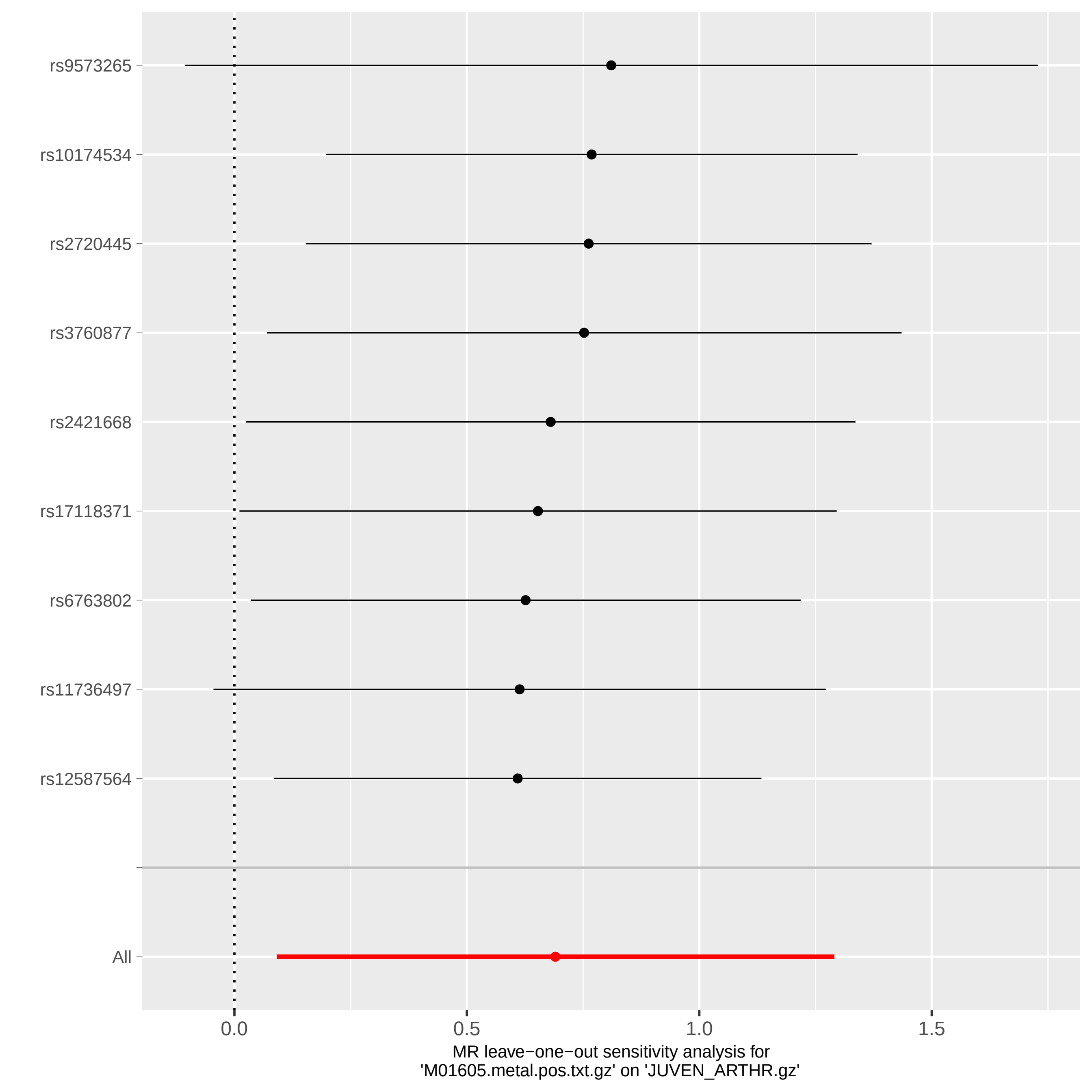
rs9573265
rs10174534
rs2720445
rs3760877
rs2421668
rs17118371
rs6763802
rs11736497
rs12587564
All
0.0
0.5
1.0
1.5
MR leave−one−out sensitivity analysis for
'M01605.metal.pos.txt.gz' on 'JUVEN_ARTHR.gz'

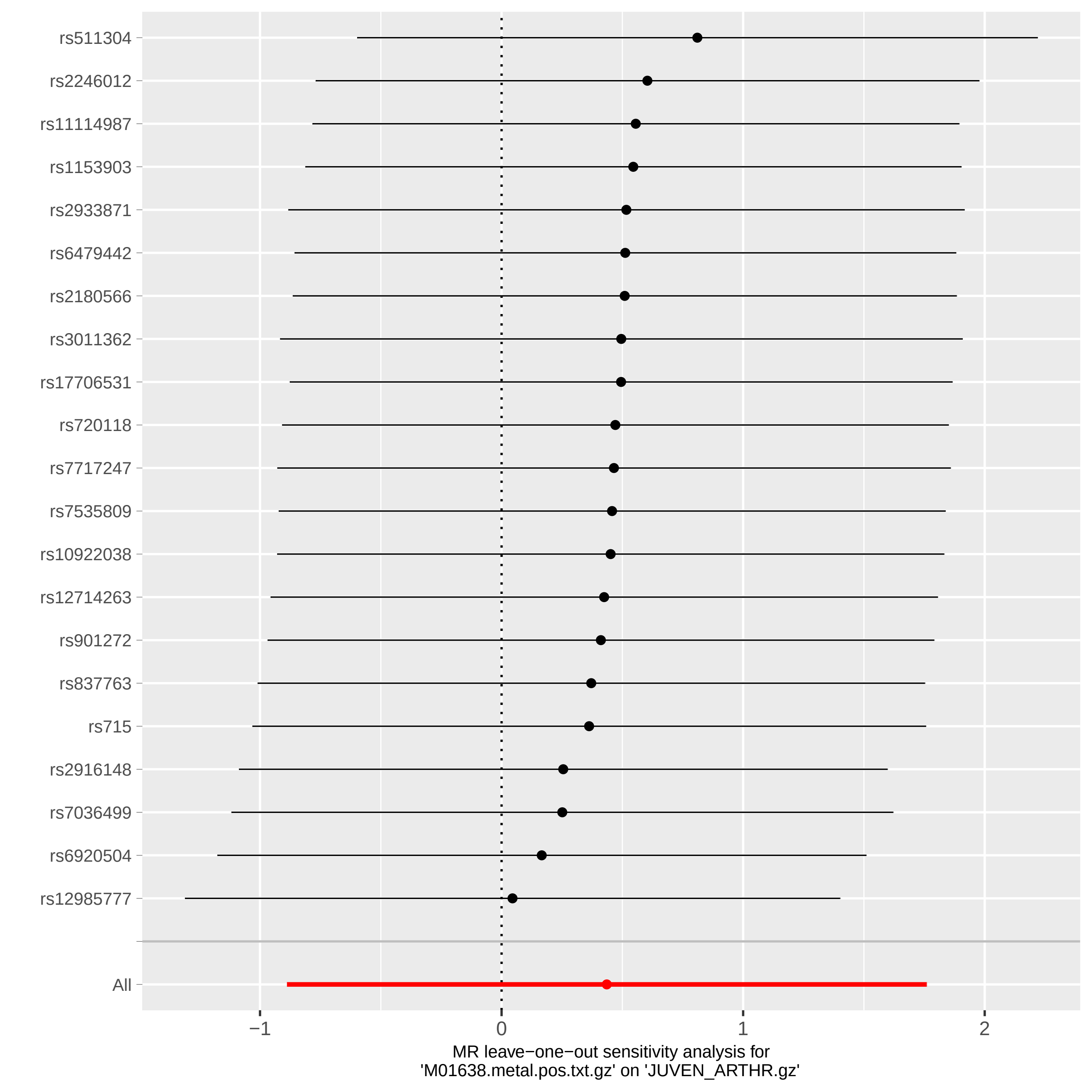
rs511304
rs2246012
rs11114987
rs1153903
rs2933871
rs6479442
rs2180566
rs3011362
rs17706531
rs720118
rs7717247
rs7535809
rs10922038
rs12714263
rs901272
rs837763
rs715
rs2916148
rs7036499
rs6920504
rs12985777
All
−1
0
1
2
MR leave−one−out sensitivity analysis for
'M01638.metal.pos.txt.gz' on 'JUVEN_ARTHR.gz'

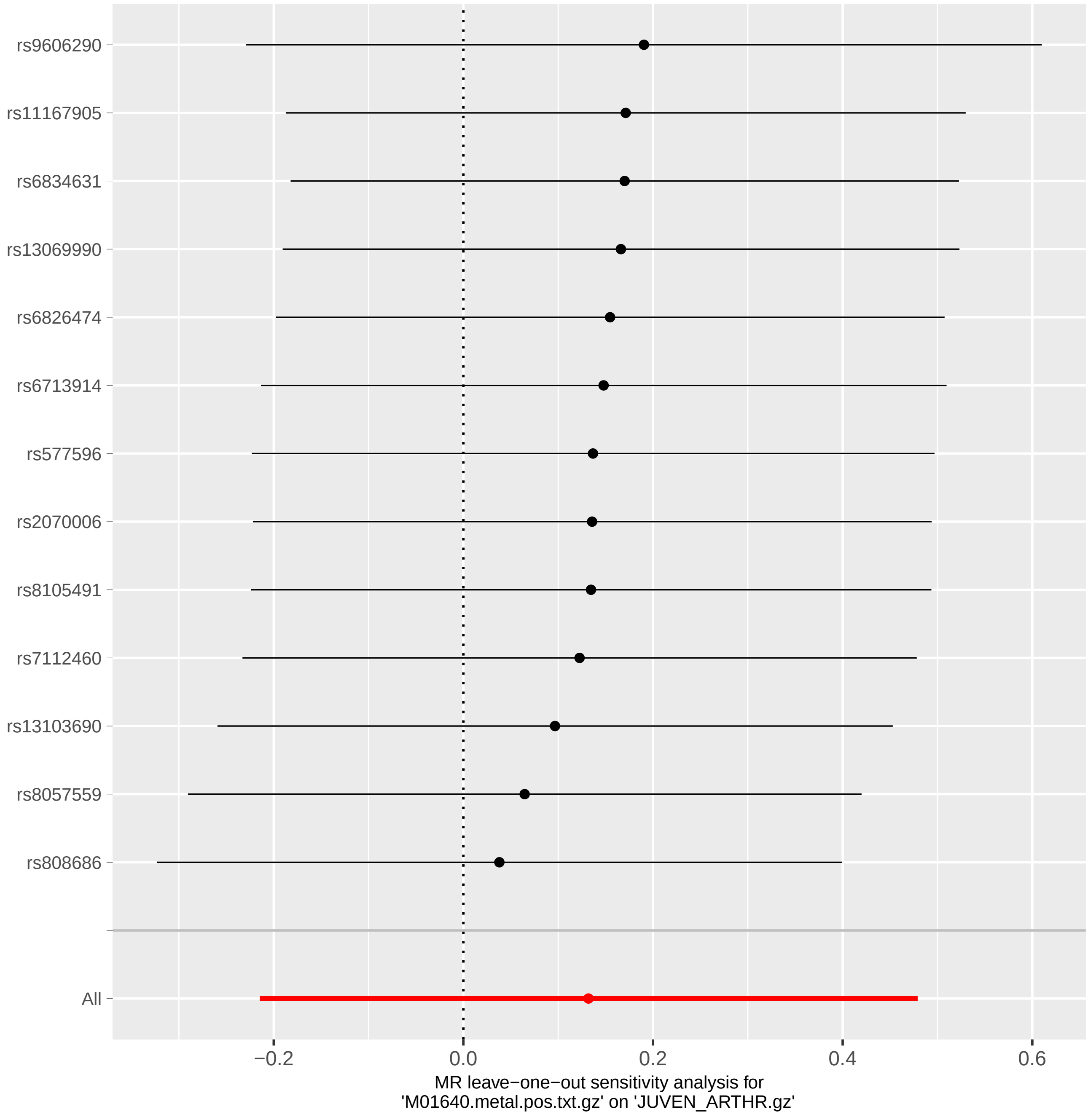
rs9606290
rs11167905
rs6834631
rs13069990
rs6826474
rs6713914
rs577596
rs2070006
rs8105491
rs7112460
rs13103690
rs8057559
rs808686
All
−0.2
0.0
0.2
0.4
0.6
MR leave−one−out sensitivity analysis for
'M01640.metal.pos.txt.gz' on 'JUVEN_ARTHR.gz'

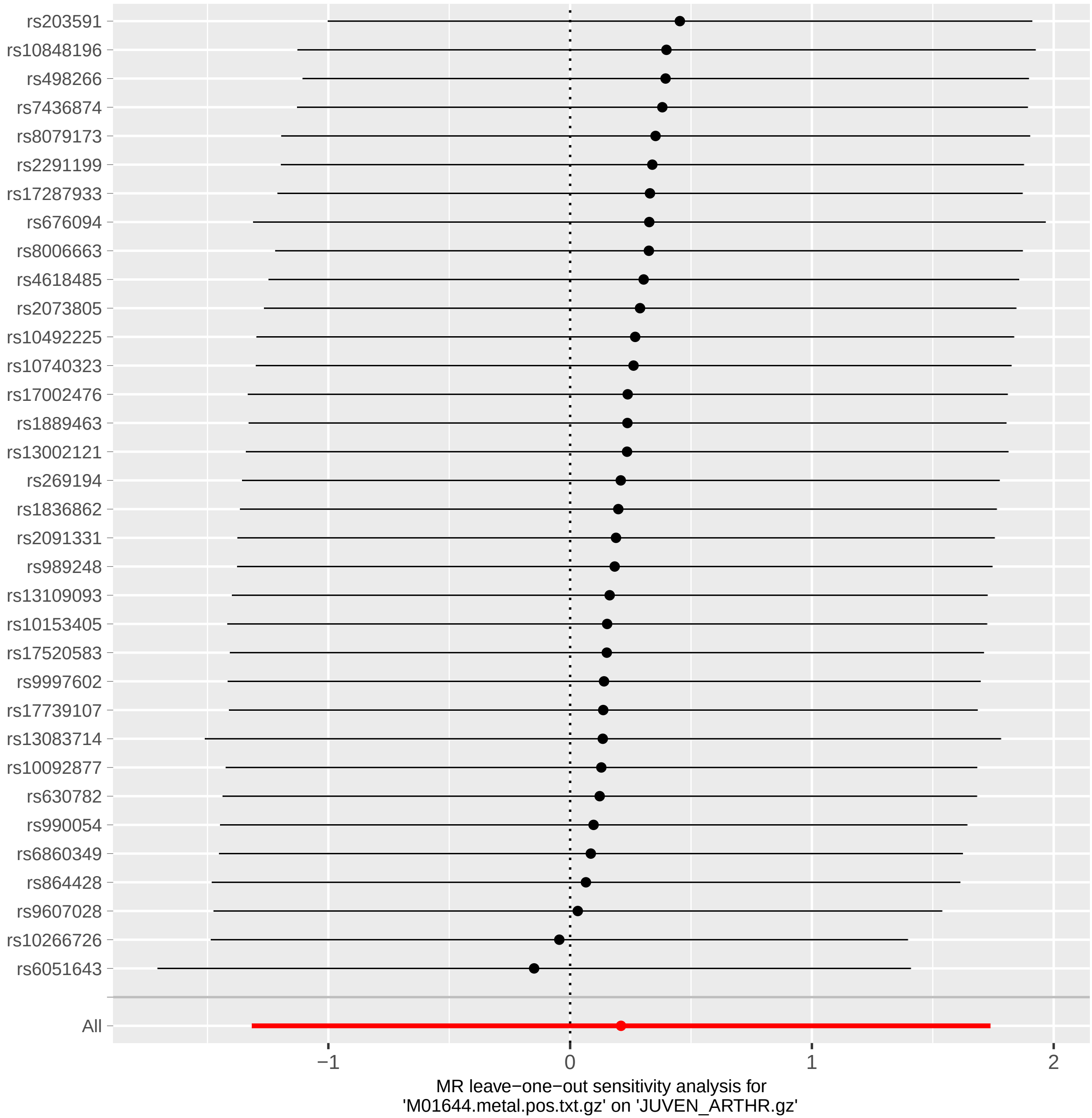
rs203591
rs10848196
rs498266
rs7436874
rs8079173
rs2291199
rs17287933
rs676094
rs8006663
rs4618485
rs2073805
rs10492225
rs10740323
rs17002476
rs1889463
rs13002121
rs269194
rs1836862
rs2091331
rs989248
rs13109093
rs10153405
rs17520583
rs9997602
rs17739107
rs13083714
rs10092877
rs630782
rs990054
rs6860349
rs864428
rs9607028
rs10266726
rs6051643
All
−1
0
1
2
MR leave−one−out sensitivity analysis for
'M01644.metal.pos.txt.gz' on 'JUVEN_ARTHR.gz'

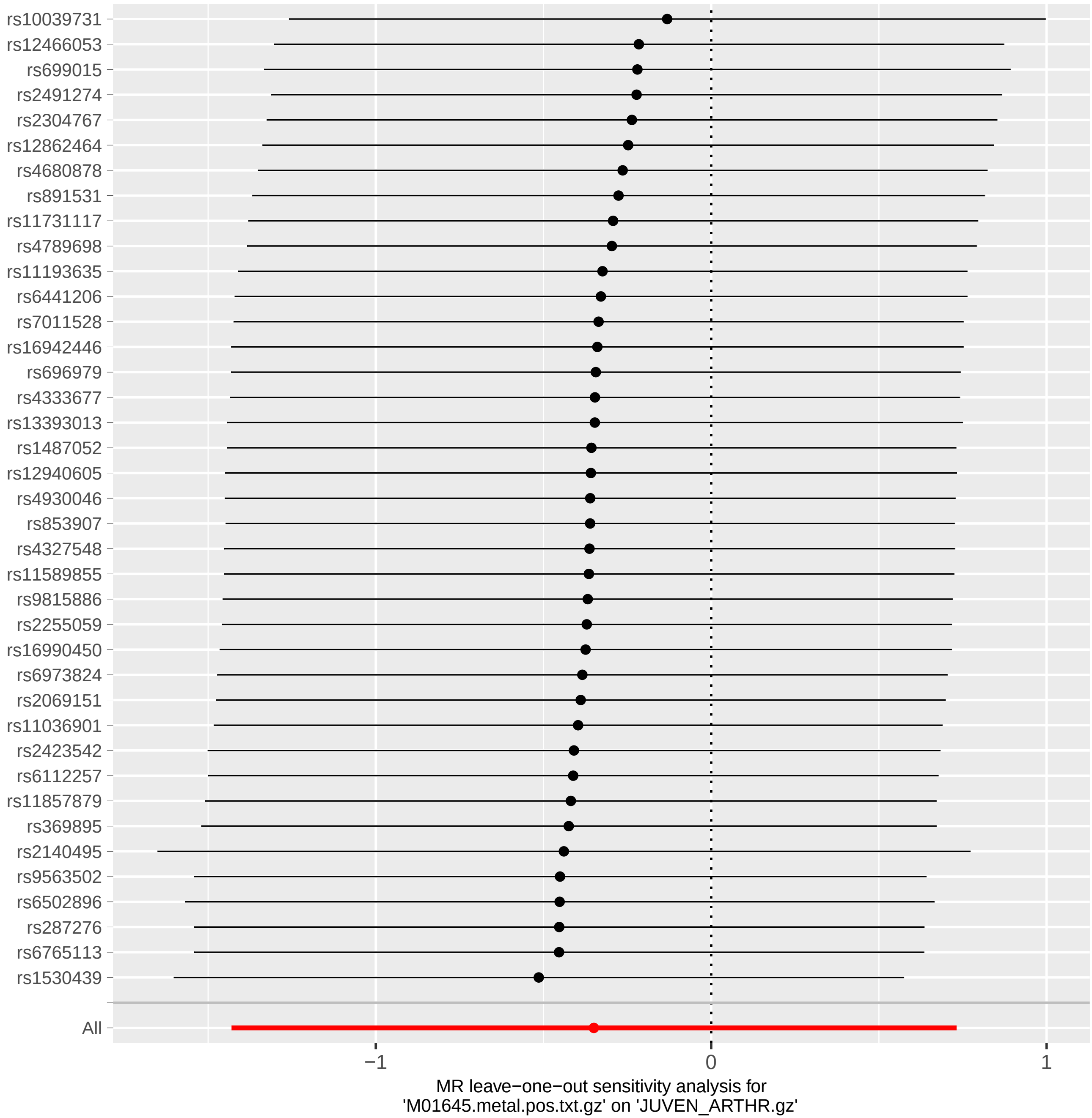
rs10039731
rs12466053
rs699015
rs2491274
rs2304767
rs12862464
rs4680878
rs891531
rs11731117
rs4789698
rs11193635
rs6441206
rs7011528
rs16942446
rs696979
rs4333677
rs13393013
rs1487052
rs12940605
rs4930046
rs853907
rs4327548
rs11589855
rs9815886
rs2255059
rs16990450
rs6973824
rs2069151
rs11036901
rs2423542
rs6112257
rs11857879
rs369895
rs2140495
rs9563502
rs6502896
rs287276
rs6765113
rs1530439
All
−1
0
1
MR leave−one−out sensitivity analysis for
'M01645.metal.pos.txt.gz' on 'JUVEN_ARTHR.gz'

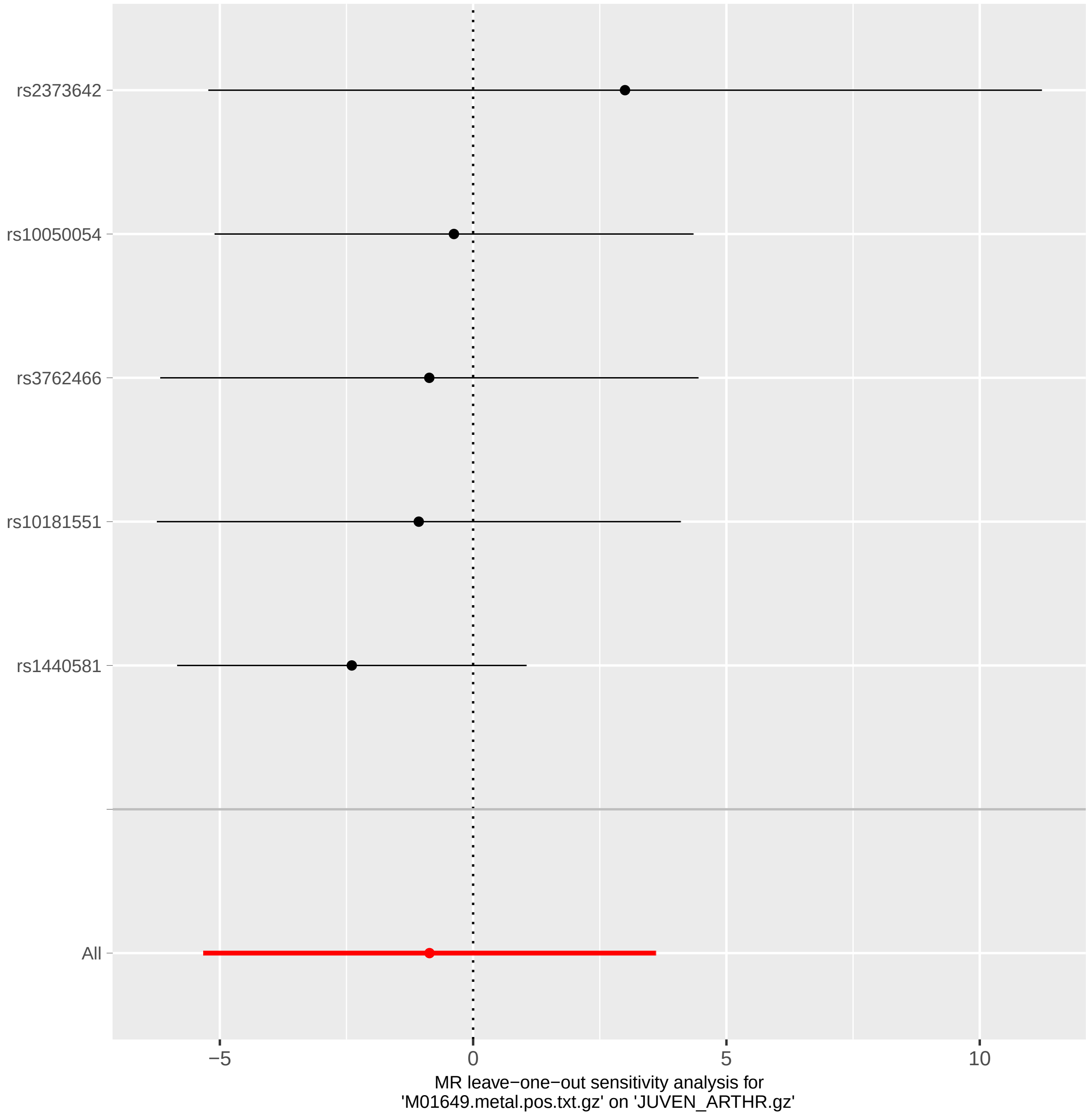
rs2373642
rs10050054
rs3762466
rs10181551
rs1440581
All
−5
0
5
10
MR leave−one−out sensitivity analysis for
'M01649.metal.pos.txt.gz' on 'JUVEN_ARTHR.gz'

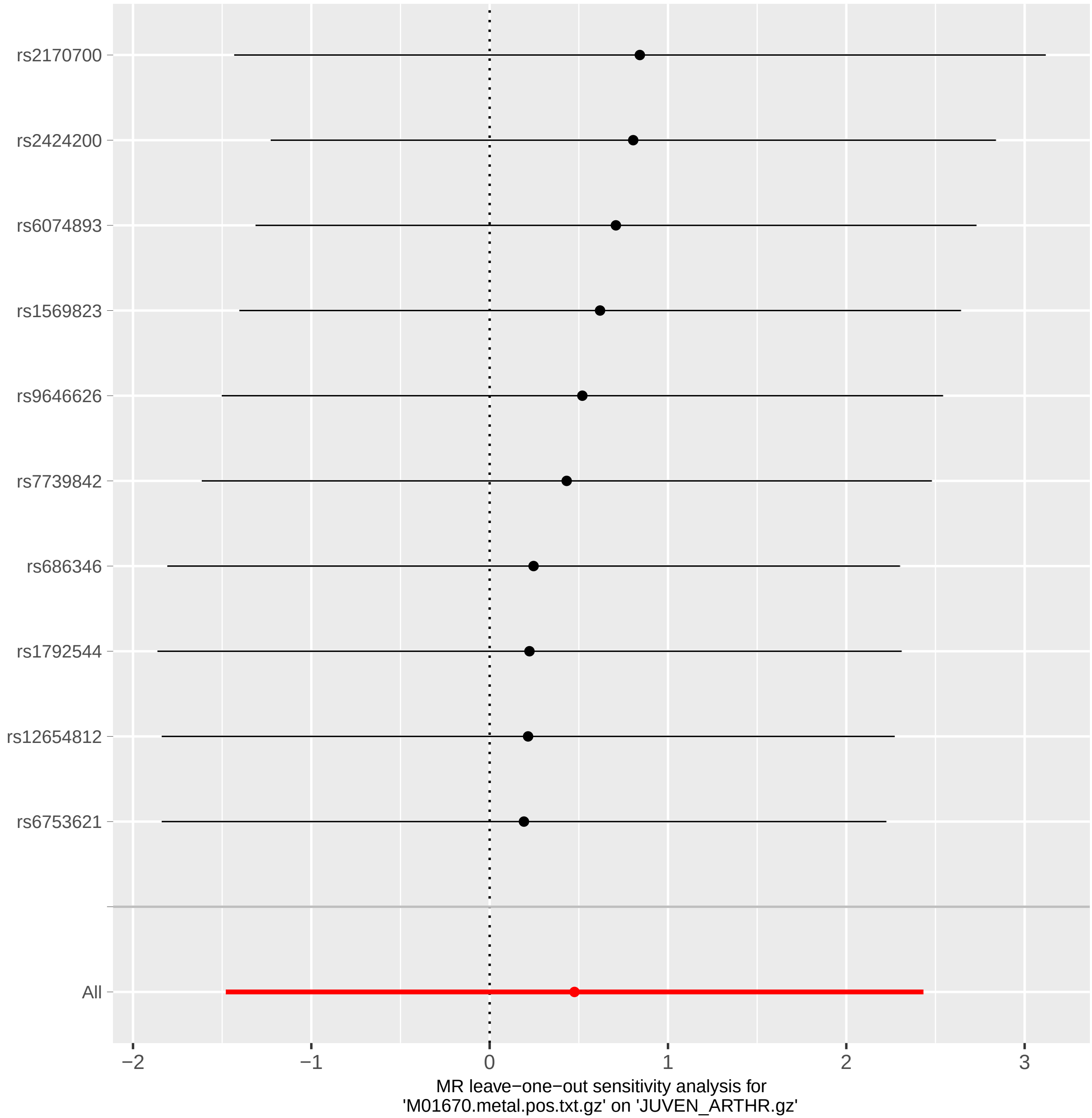
rs2170700
rs2424200
rs6074893
rs1569823
rs9646626
rs7739842
rs686346
rs1792544
rs12654812
rs6753621
All
−2
−1
0
1
2
3
MR leave−one−out sensitivity analysis for
'M01670.metal.pos.txt.gz' on 'JUVEN_ARTHR.gz'

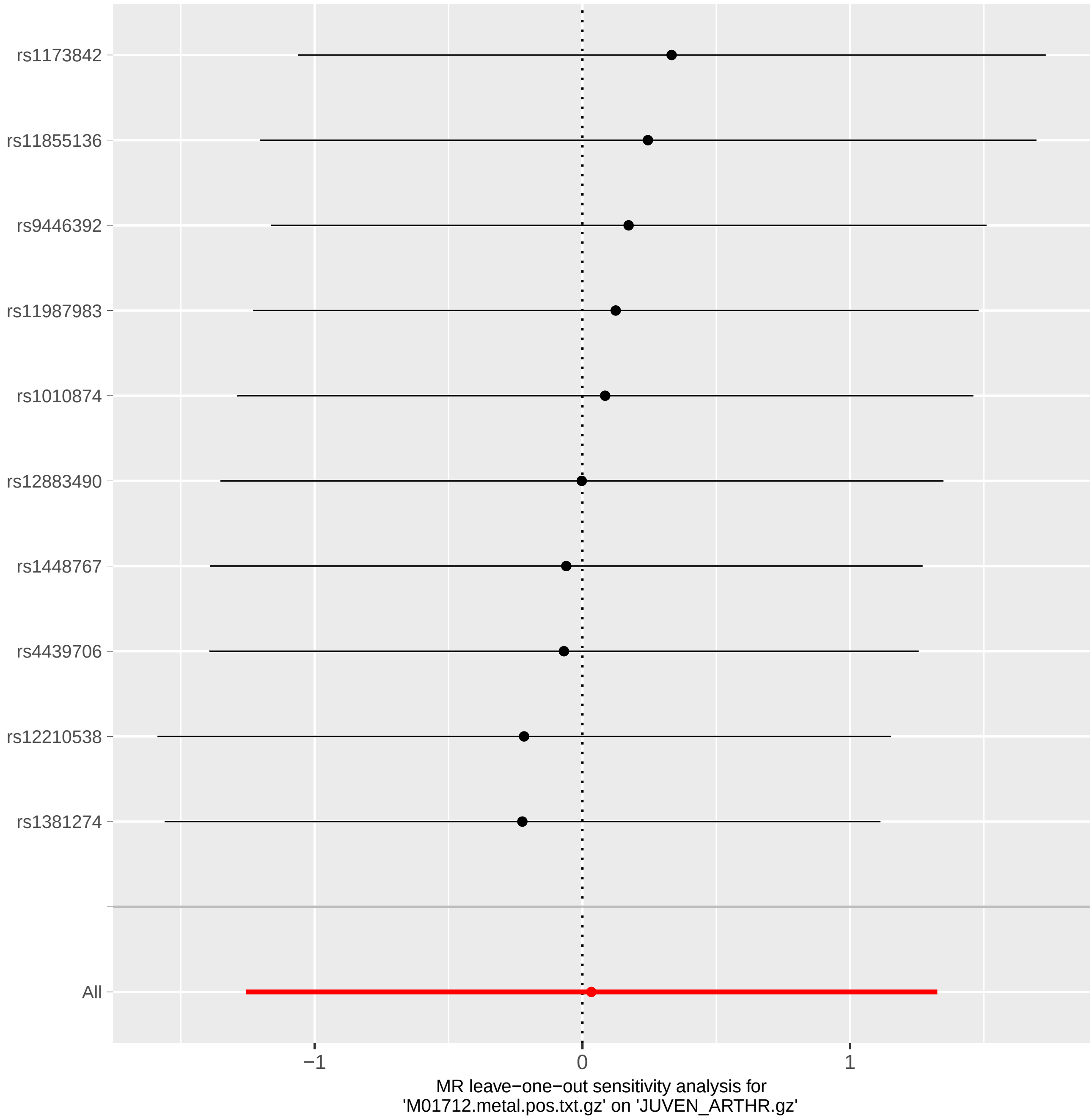
rs1173842
rs11855136
rs9446392
rs11987983
rs1010874
rs12883490
rs1448767
rs4439706
rs12210538
rs1381274
All
−1
0
1
MR leave−one−out sensitivity analysis for
'M01712.metal.pos.txt.gz' on 'JUVEN_ARTHR.gz'

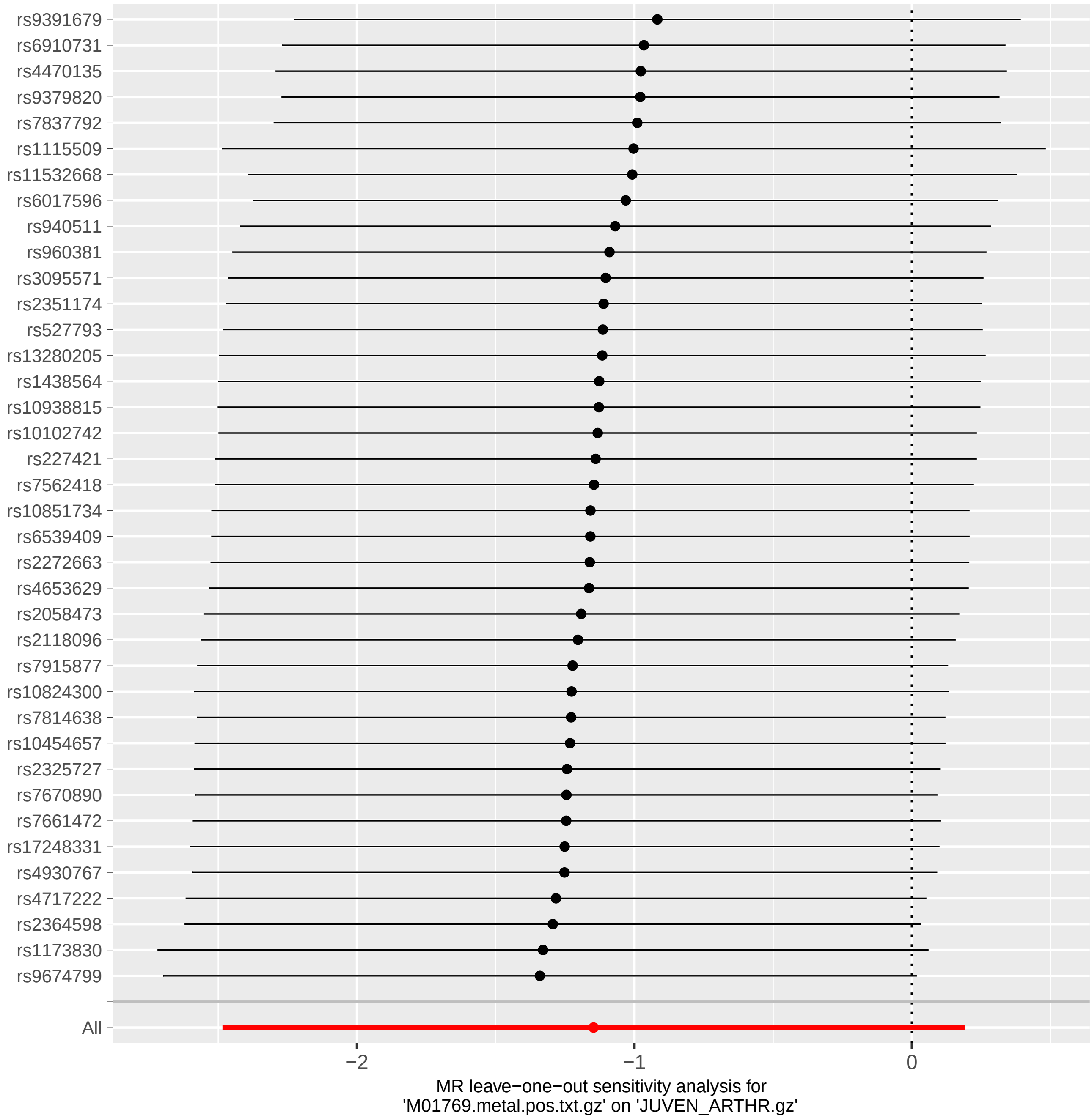
rs9391679
rs6910731
rs4470135
rs9379820
rs7837792
rs1115509
rs11532668
rs6017596
rs940511
rs960381
rs3095571
rs2351174
rs527793
rs13280205
rs1438564
rs10938815
rs10102742
rs227421
rs7562418
rs10851734
rs6539409
rs2272663
rs4653629
rs2058473
rs2118096
rs7915877
rs10824300
rs7814638
rs10454657
rs2325727
rs7670890
rs7661472
rs17248331
rs4930767
rs4717222
rs2364598
rs1173830
rs9674799
All
−2
−1
0
MR leave−one−out sensitivity analysis for
'M01769.metal.pos.txt.gz' on 'JUVEN_ARTHR.gz'

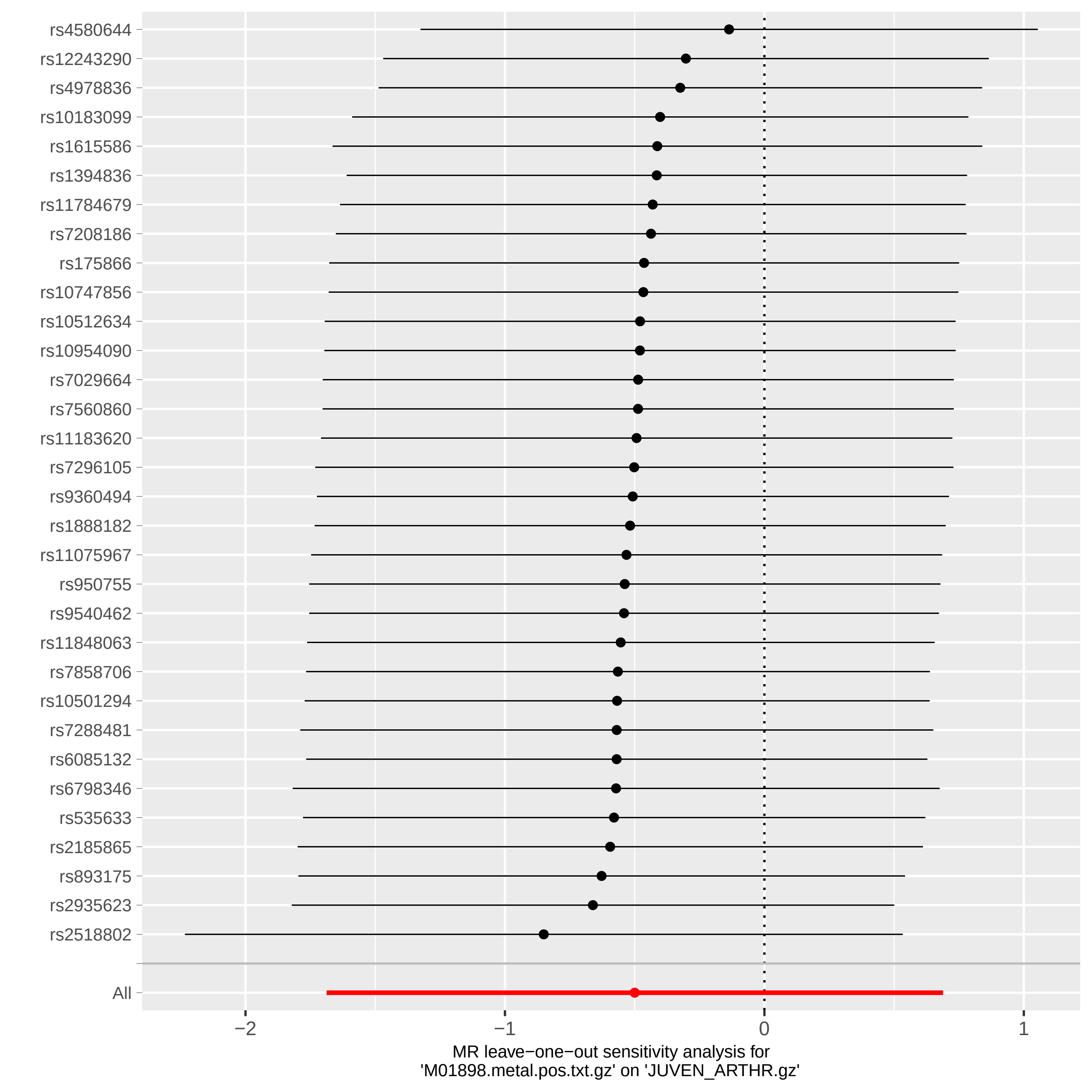
rs4580644
rs12243290
rs4978836
rs10183099
rs1615586
rs1394836
rs11784679
rs7208186
rs175866
rs10747856
rs10512634
rs10954090
rs7029664
rs7560860
rs11183620
rs7296105
rs9360494
rs1888182
rs11075967
rs950755
rs9540462
rs11848063
rs7858706
rs10501294
rs7288481
rs6085132
rs6798346
rs535633
rs2185865
rs893175
rs2935623
rs2518802
All
−2
−1
0
1
MR leave−one−out sensitivity analysis for
'M01898.metal.pos.txt.gz' on 'JUVEN_ARTHR.gz'

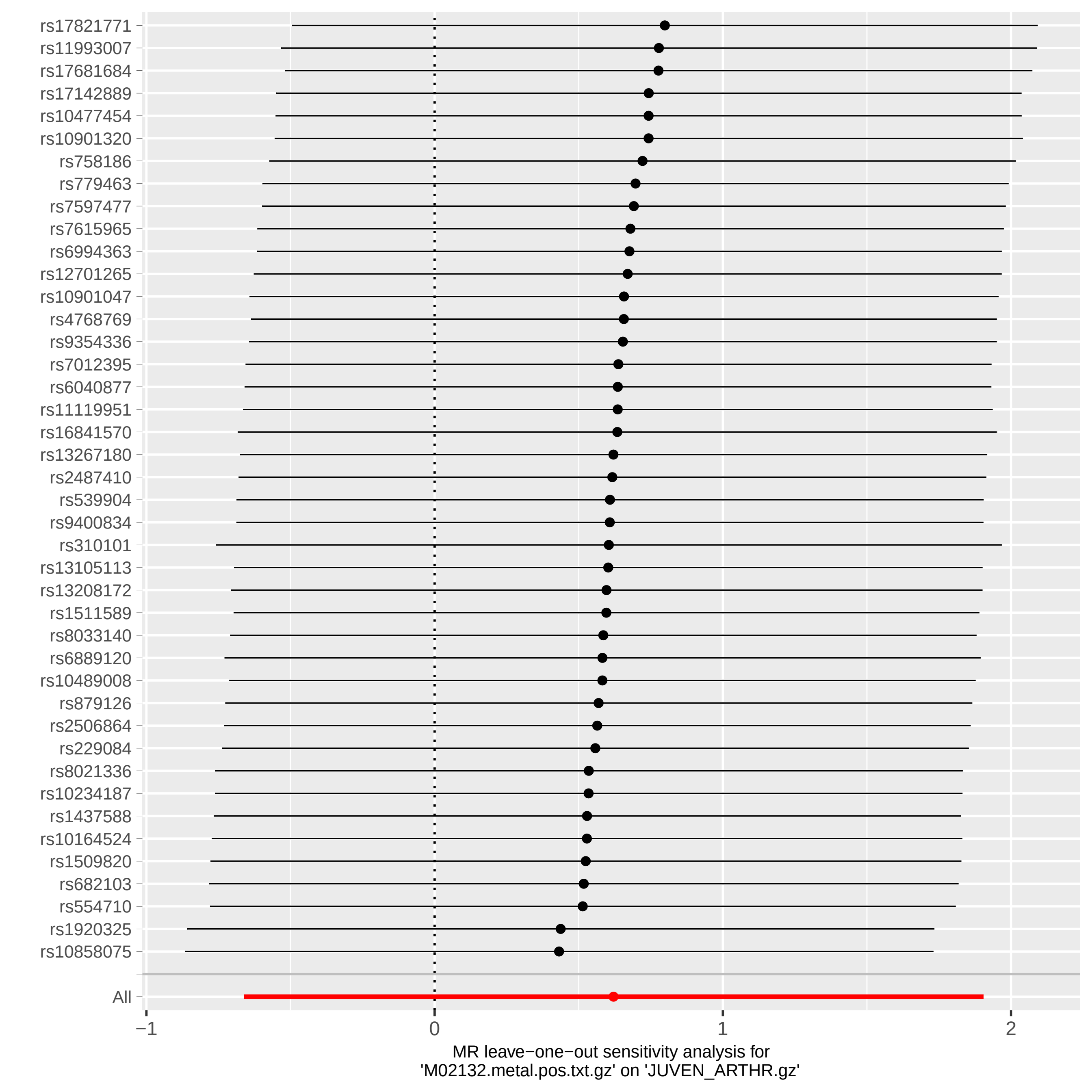

rs17821771
rs11993007
rs17681684
rs17142889
rs10477454
rs10901320
rs758186
rs779463
rs7597477
rs7615965
rs6994363
rs12701265
rs10901047
rs4768769
rs9354336
rs7012395
rs6040877
rs11119951
rs16841570
rs13267180
rs2487410
rs539904
rs9400834
rs310101
rs13105113
rs13208172
rs1511589
rs8033140
rs6889120
rs10489008
rs879126
rs2506864
rs229084
rs8021336
rs10234187
rs1437588
rs10164524
rs1509820
rs682103
rs554710
rs1920325
rs10858075
All
−1
0
1
2
MR leave−one−out sensitivity analysis for
'M02132.metal.pos.txt.gz' on 'JUVEN_ARTHR.gz'

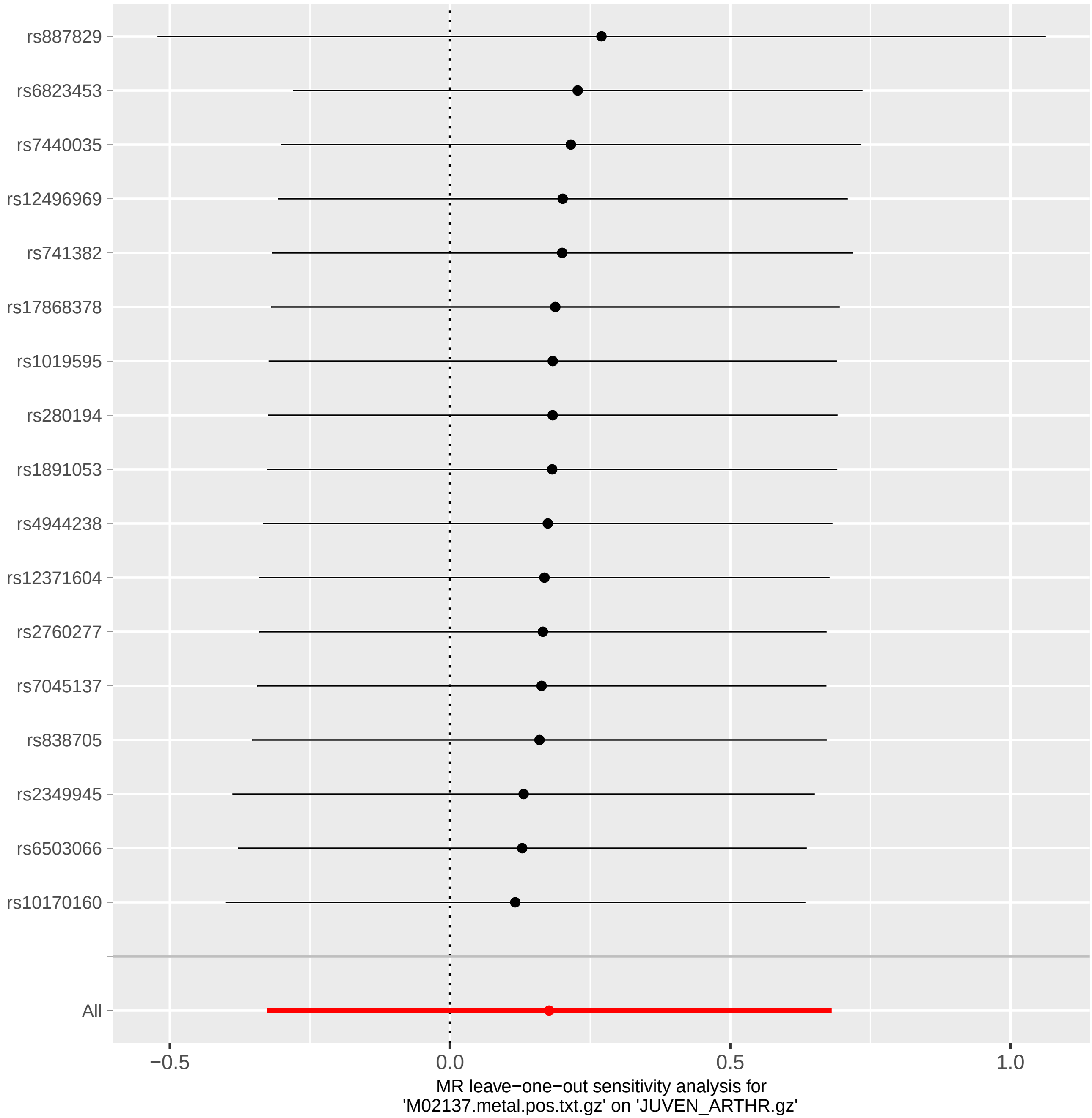
rs887829
rs6823453
rs7440035
rs12496969
rs741382
rs17868378
rs1019595
rs280194
rs1891053
rs4944238
rs12371604
rs2760277
rs7045137
rs838705
rs2349945
rs6503066
rs10170160
All
−0.5
0.0
0.5
1.0
MR leave−one−out sensitivity analysis for
'M02137.metal.pos.txt.gz' on 'JUVEN_ARTHR.gz'

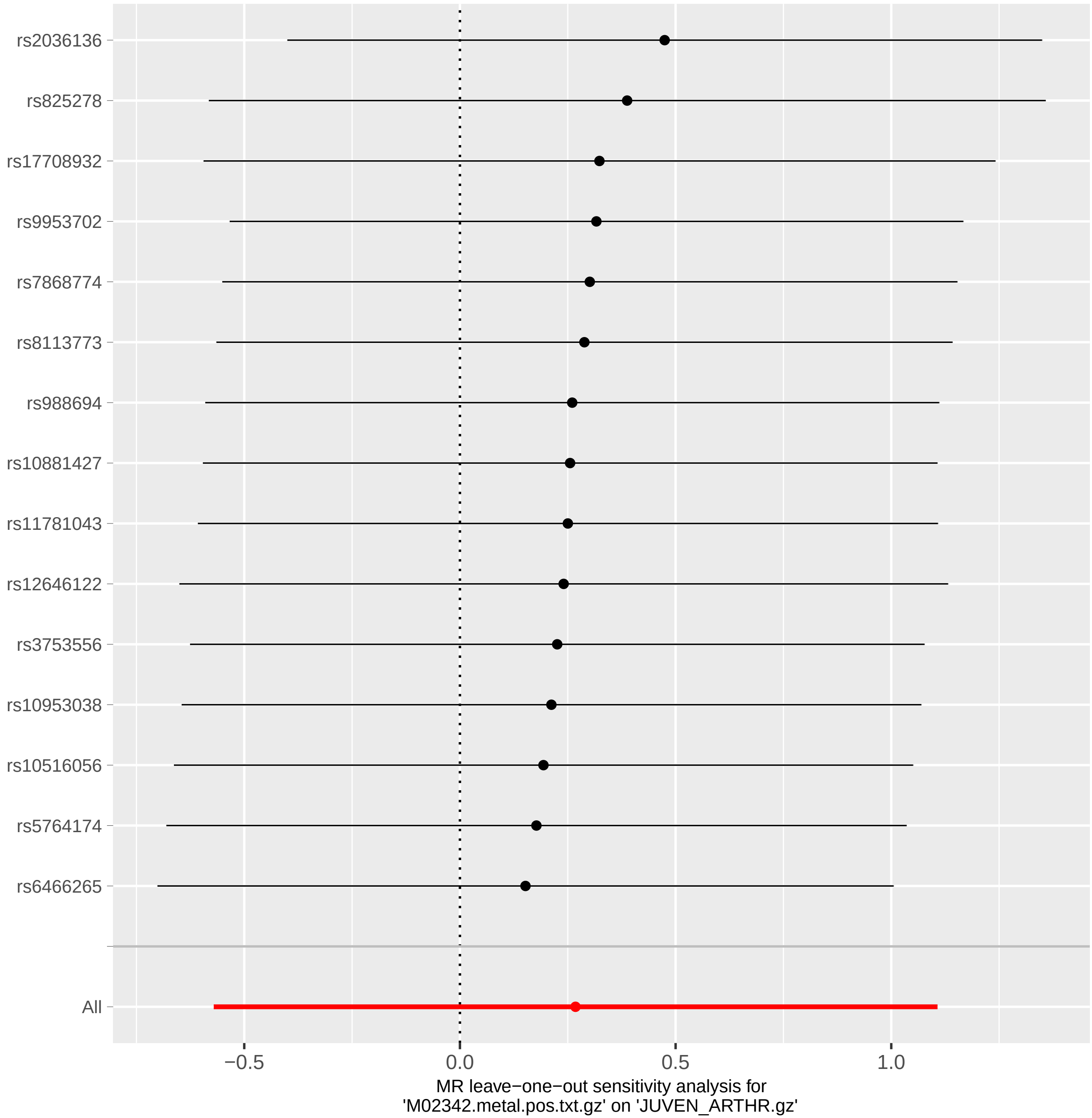
rs2036136
rs825278
rs17708932
rs9953702
rs7868774
rs8113773
rs988694
rs10881427
rs11781043
rs12646122
rs3753556
rs10953038
rs10516056
rs5764174
rs6466265
All
−0.5
0.0
0.5
1.0
MR leave−one−out sensitivity analysis for
'M02342.metal.pos.txt.gz' on 'JUVEN_ARTHR.gz'

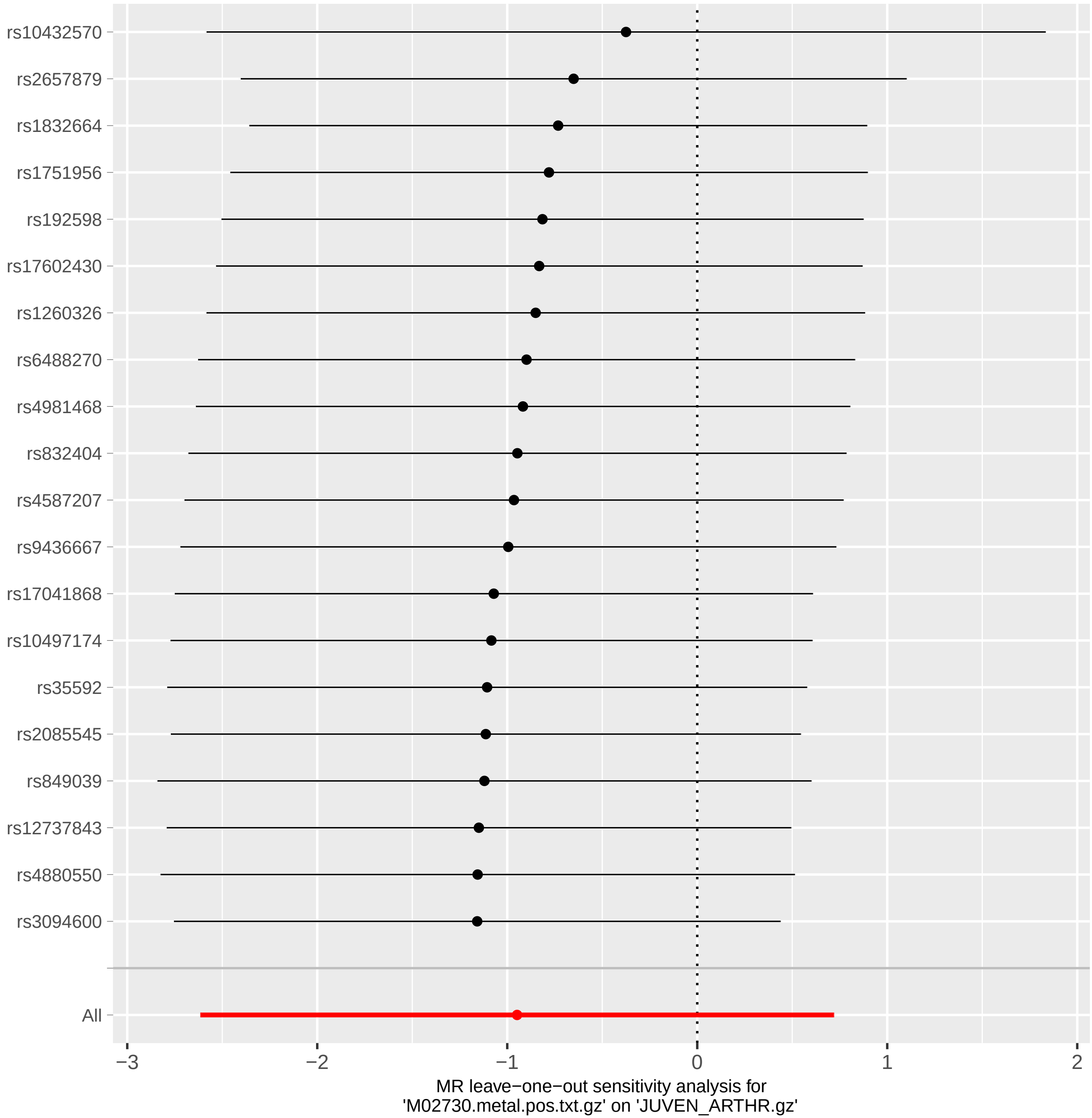
rs10432570
rs2657879
rs1832664
rs1751956
rs192598
rs17602430
rs1260326
rs6488270
rs4981468
rs832404
rs4587207
rs9436667
rs17041868
rs10497174
rs35592
rs2085545
rs849039
rs12737843
rs4880550
rs3094600
All
−3
−2
−1
0
1
2
MR leave−one−out sensitivity analysis for
'M02730.metal.pos.txt.gz' on 'JUVEN_ARTHR.gz'

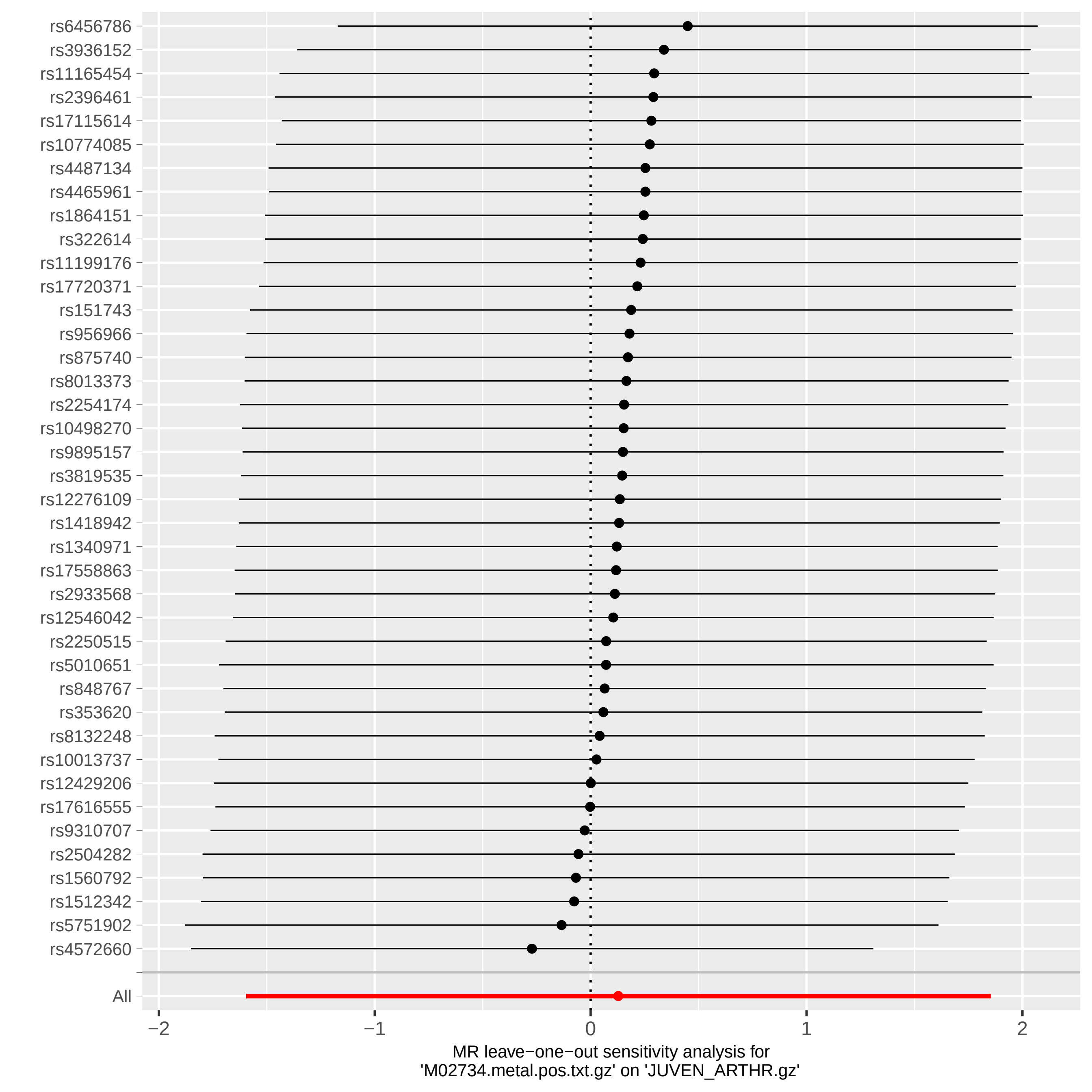

rs6456786
rs3936152
rs11165454
rs2396461
rs17115614
rs10774085
rs4487134
rs4465961
rs1864151
rs322614
rs11199176
rs17720371
rs151743
rs956966
rs875740
rs8013373
rs2254174
rs10498270
rs9895157
rs3819535
rs12276109
rs1418942
rs1340971
rs17558863
rs2933568
rs12546042
rs2250515
rs5010651
rs848767
rs353620
rs8132248
rs10013737
rs12429206
rs17616555
rs9310707
rs2504282
rs1560792
rs1512342
rs5751902
rs4572660
All
−2
−1
0
1
2
MR leave−one−out sensitivity analysis for
'M02734.metal.pos.txt.gz' on 'JUVEN_ARTHR.gz'

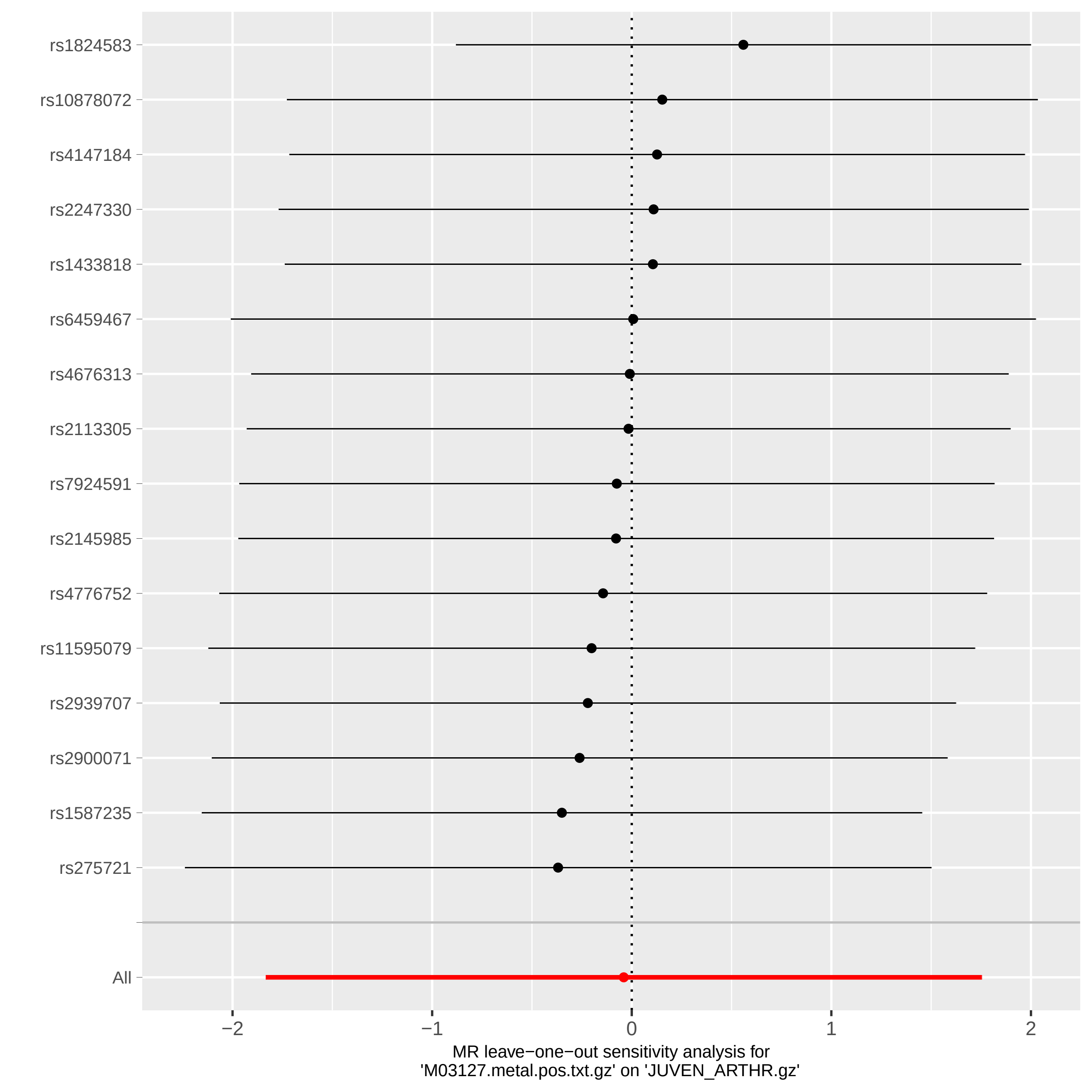

rs1824583
rs10878072
rs4147184
rs2247330
rs1433818
rs6459467
rs4676313
rs2113305
rs7924591
rs2145985
rs4776752
rs11595079
rs2939707
rs2900071
rs1587235
rs275721
All
−2
−1
0
1
2
MR leave−one−out sensitivity analysis for
'M03127.metal.pos.txt.gz' on 'JUVEN_ARTHR.gz'

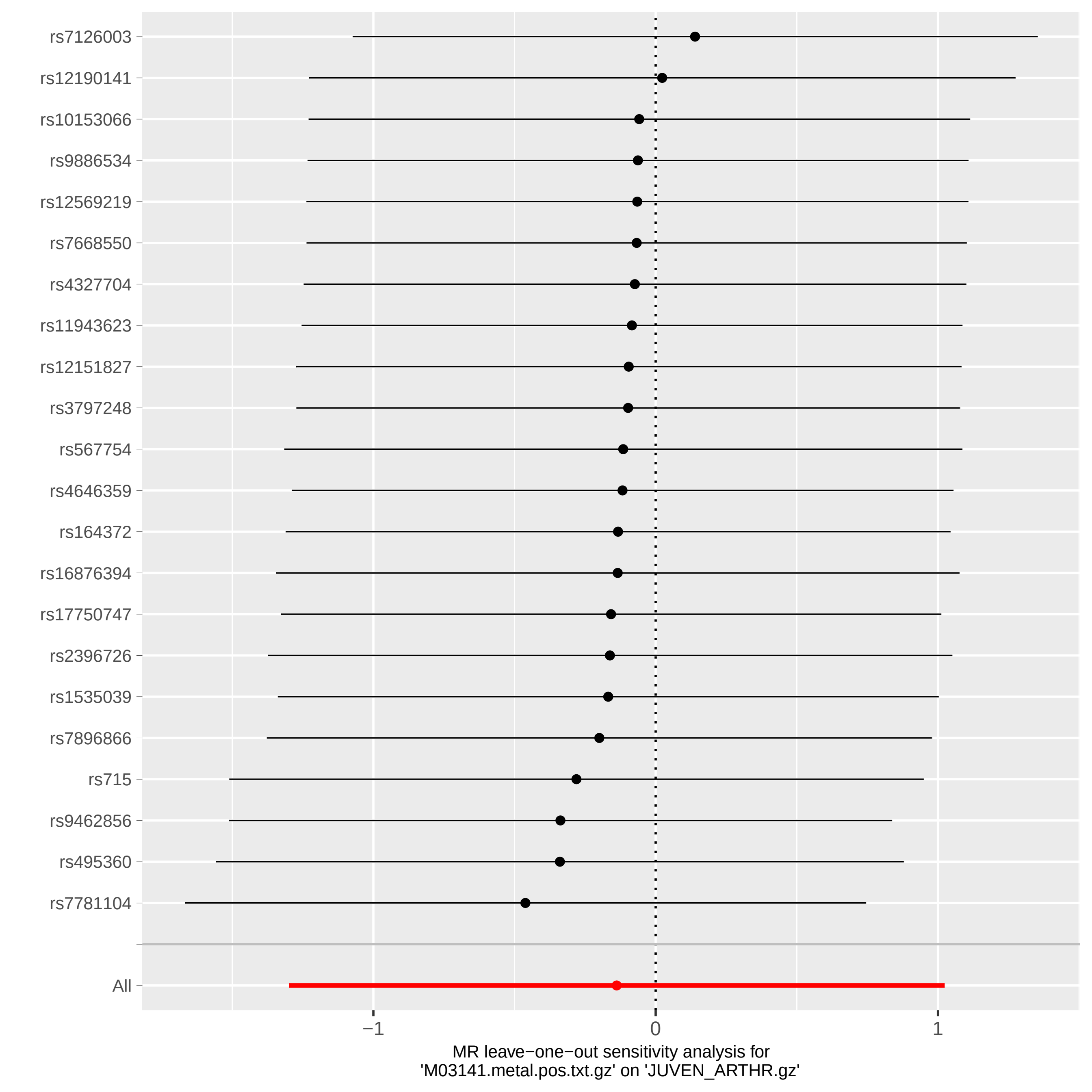
rs7126003
rs12190141
rs10153066
rs9886534
rs12569219
rs7668550
rs4327704
rs11943623
rs12151827
rs3797248
rs567754
rs4646359
rs164372
rs16876394
rs17750747
rs2396726
rs1535039
rs7896866
rs715
rs9462856
rs495360
rs7781104
All
−1
0
1
MR leave−one−out sensitivity analysis for
'M03141.metal.pos.txt.gz' on 'JUVEN_ARTHR.gz'

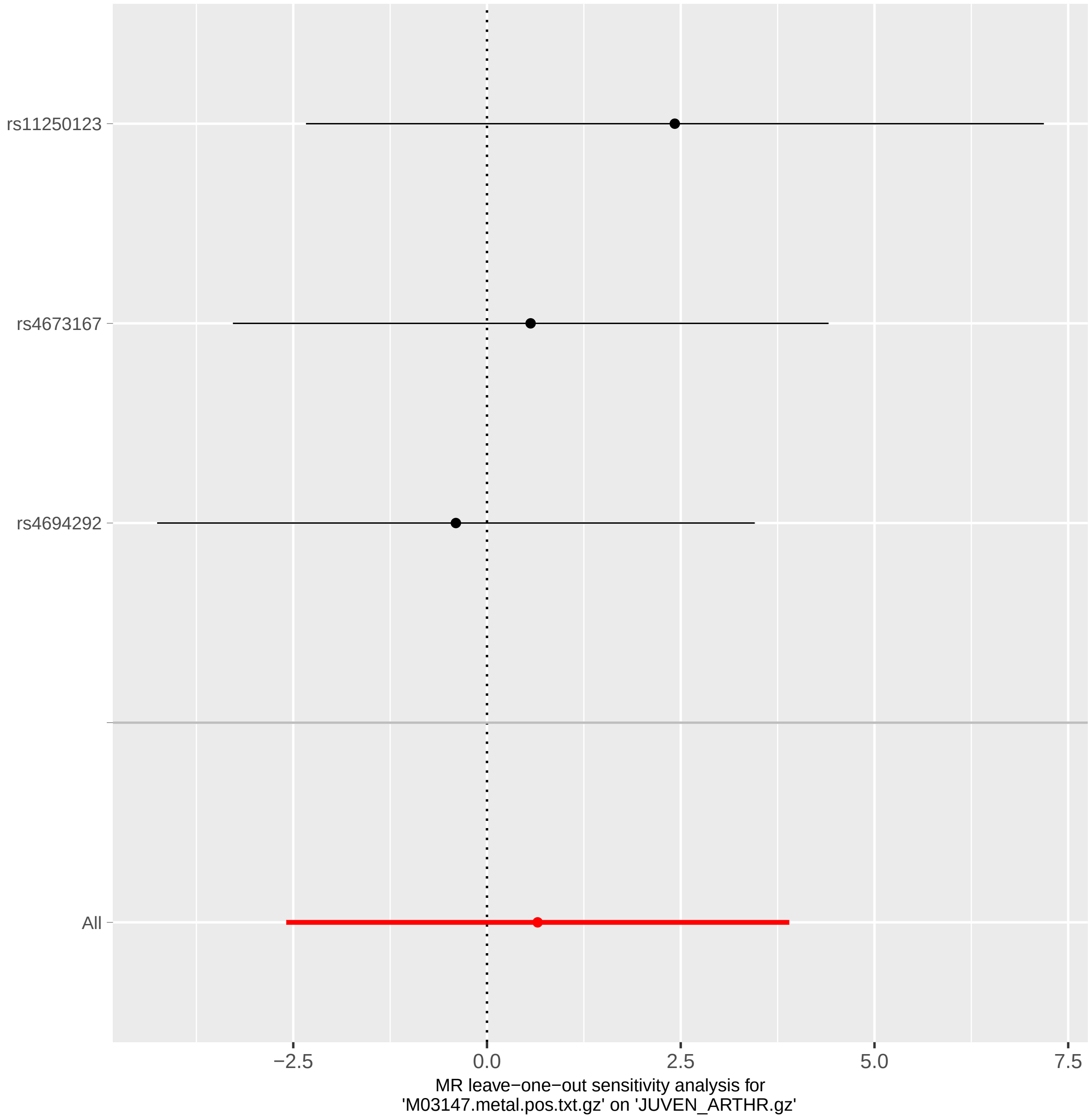

rs11250123
rs4673167
rs4694292
All
−2.5
0.0
2.5
5.0
7.5
MR leave−one−out sensitivity analysis for
'M03147.metal.pos.txt.gz' on 'JUVEN_ARTHR.gz'

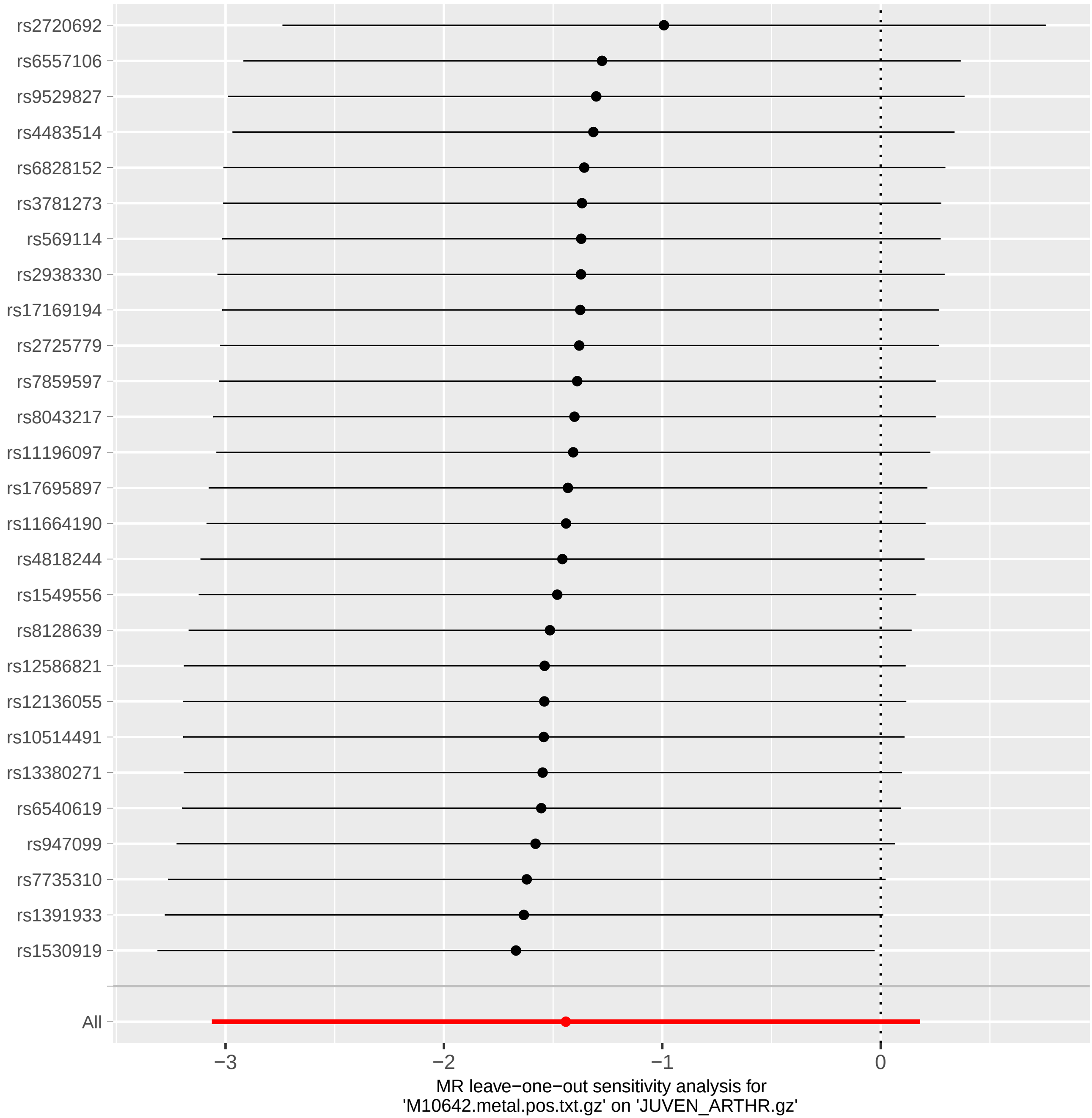

rs2720692
rs6557106
rs9529827
rs4483514
rs6828152
rs3781273
rs569114
rs2938330
rs17169194
rs2725779
rs7859597
rs8043217
rs11196097
rs17695897
rs11664190
rs4818244
rs1549556
rs8128639
rs12586821
rs12136055
rs10514491
rs13380271
rs6540619
rs947099
rs7735310
rs1391933
rs1530919
All
−3
−2
−1
0
MR leave−one−out sensitivity analysis for
'M10642.metal.pos.txt.gz' on 'JUVEN_ARTHR.gz'

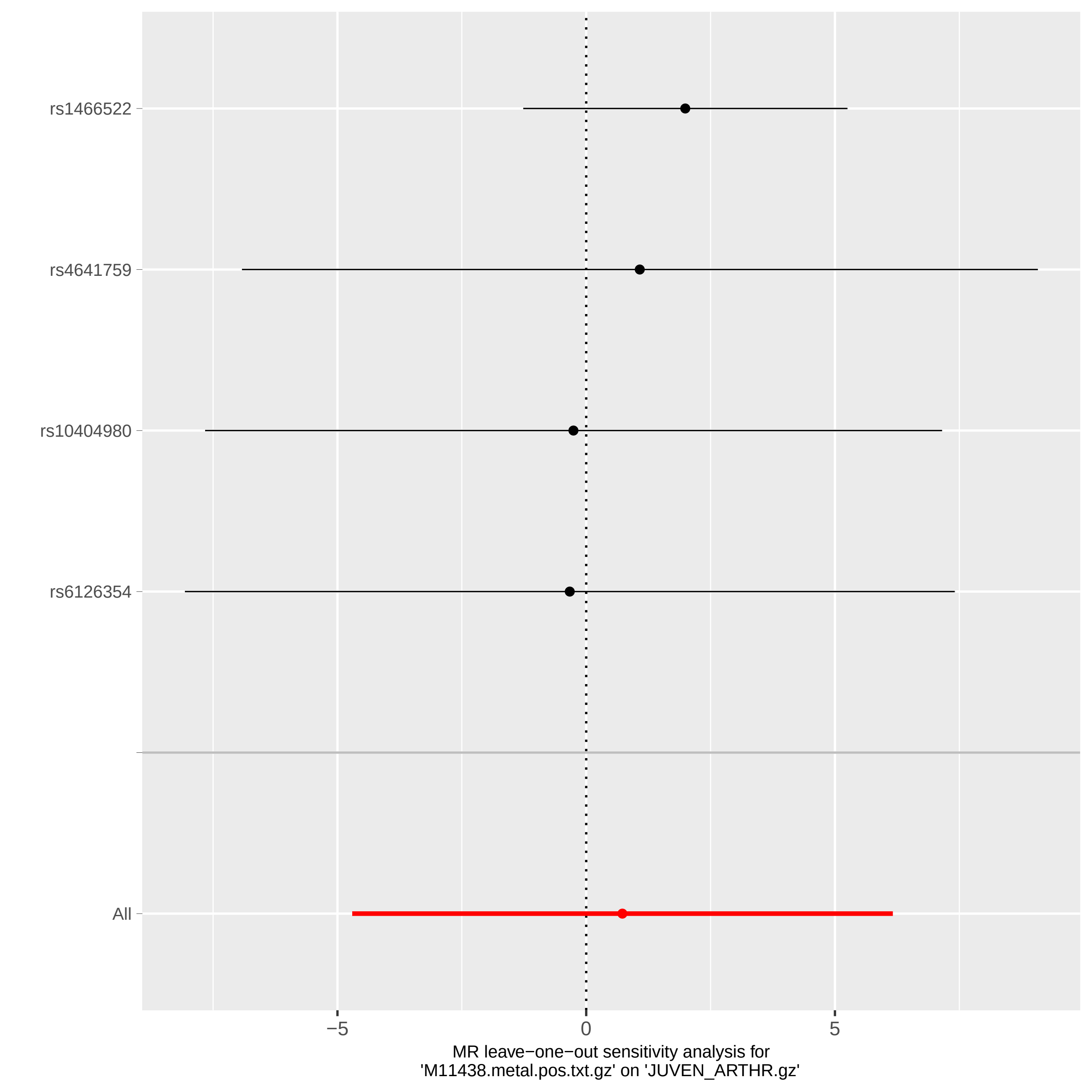
rs1466522
rs4641759
rs10404980
rs6126354
All
−5
0
5
MR leave−one−out sensitivity analysis for
'M11438.metal.pos.txt.gz' on 'JUVEN_ARTHR.gz'

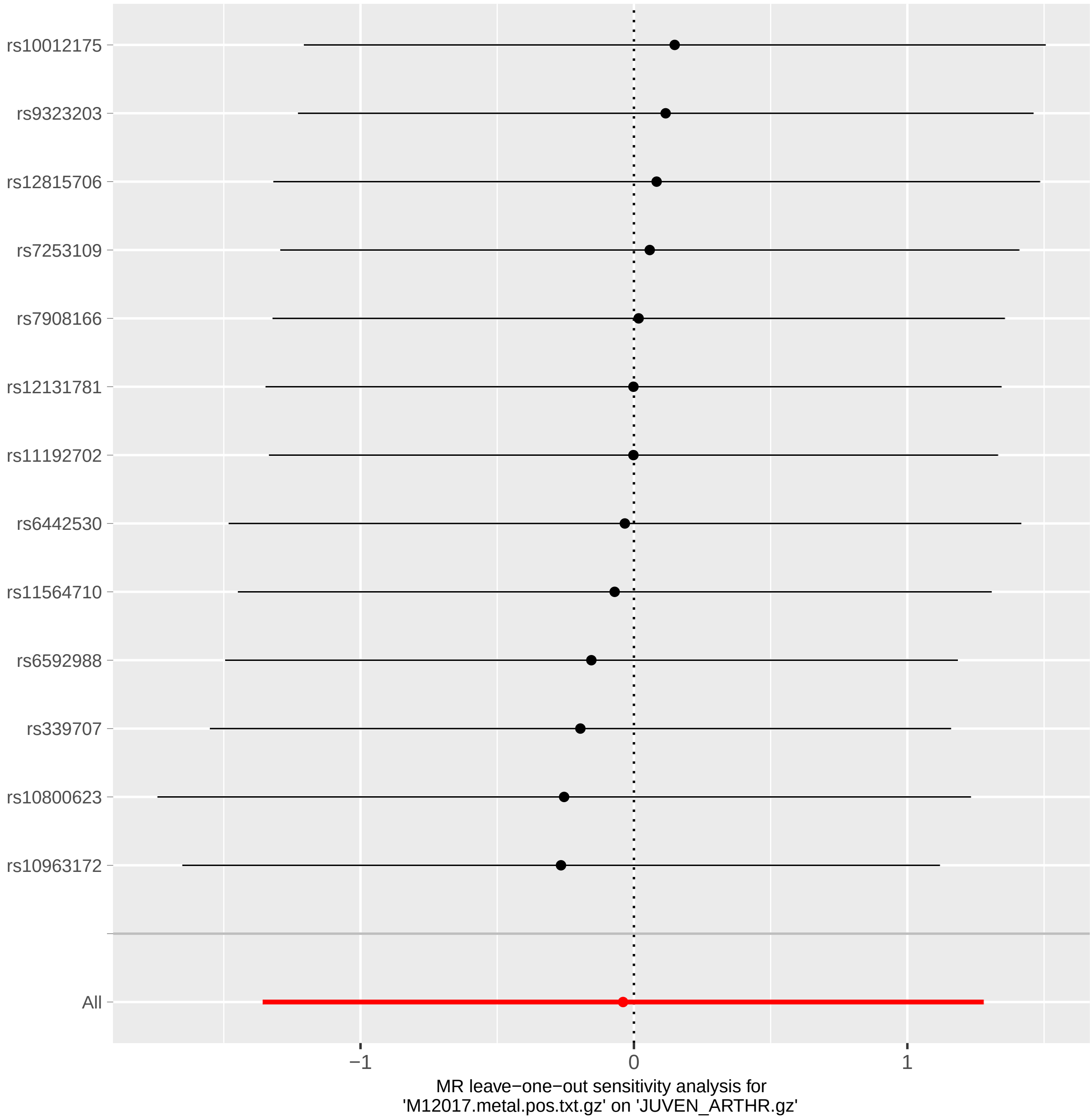
rs10012175
rs9323203
rs12815706
rs7253109
rs7908166
rs12131781
rs11192702
rs6442530
rs11564710
rs6592988
rs339707
rs10800623
rs10963172
All
−1
0
1
MR leave−one−out sensitivity analysis for
'M12017.metal.pos.txt.gz' on 'JUVEN_ARTHR.gz'

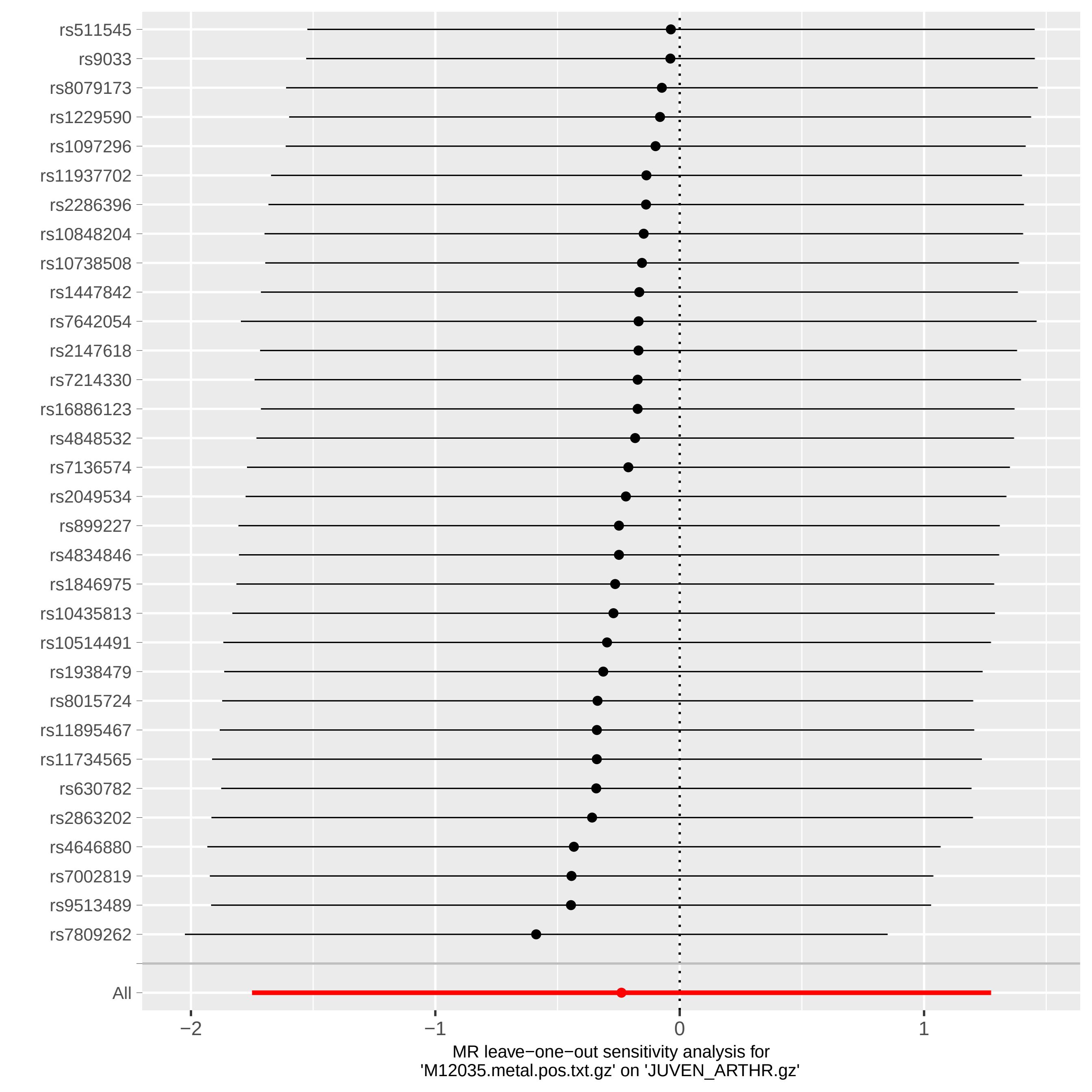
rs511545
rs9033
rs8079173
rs1229590
rs1097296
rs11937702
rs2286396
rs10848204
rs10738508
rs1447842
rs7642054
rs2147618
rs7214330
rs16886123
rs4848532
rs7136574
rs2049534
rs899227
rs4834846
rs1846975
rs10435813
rs10514491
rs1938479
rs8015724
rs11895467
rs11734565
rs630782
rs2863202
rs4646880
rs7002819
rs9513489
rs7809262
All
−2
−1
0
1
MR leave−one−out sensitivity analysis for
'M12035.metal.pos.txt.gz' on 'JUVEN_ARTHR.gz'

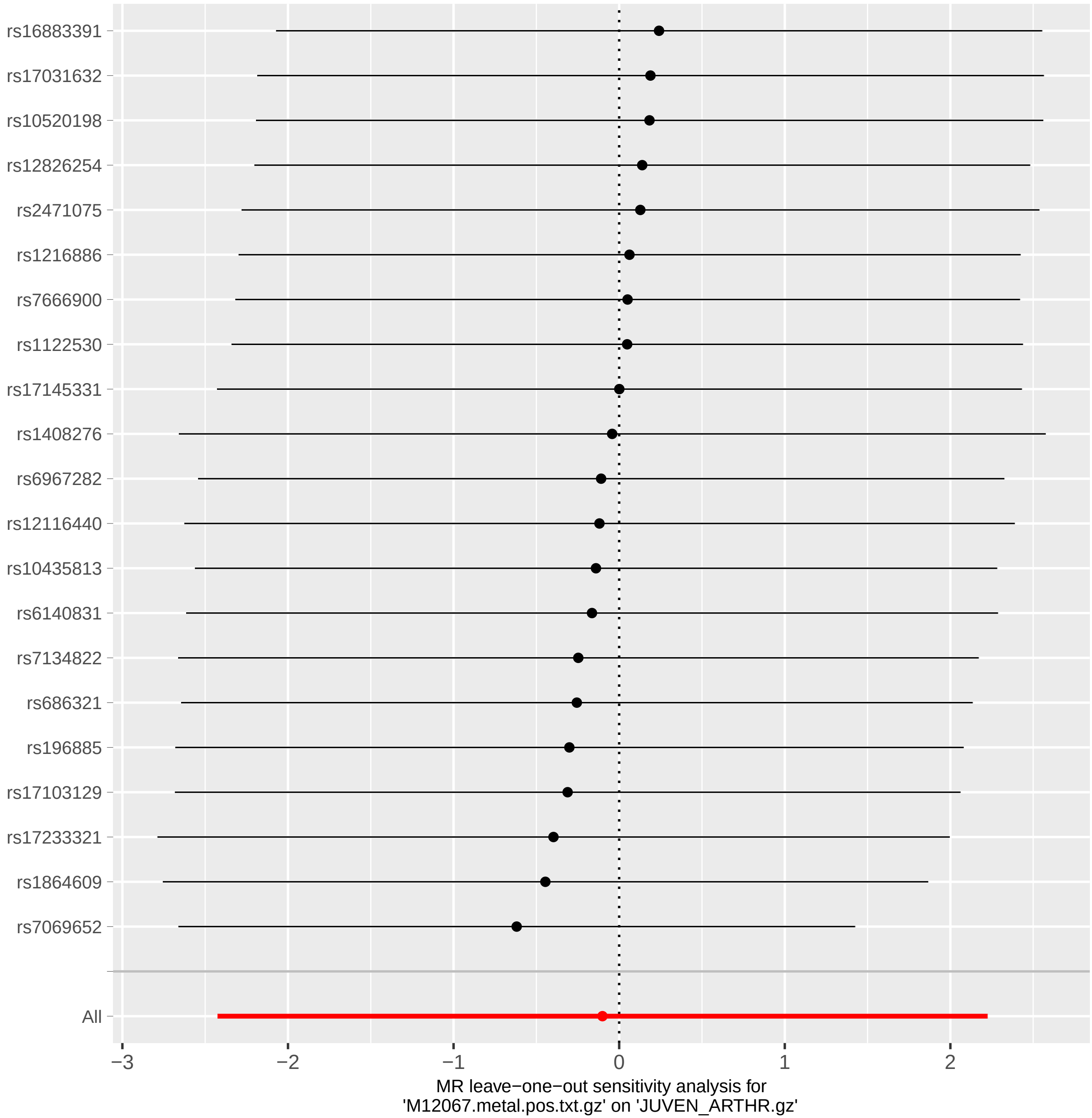
rs16883391
rs17031632
rs10520198
rs12826254
rs2471075
rs1216886
rs7666900
rs1122530
rs17145331
rs1408276
rs6967282
rs12116440
rs10435813
rs6140831
rs7134822
rs686321
rs196885
rs17103129
rs17233321
rs1864609
rs7069652
All
−3
−2
−1
0
1
2
MR leave−one−out sensitivity analysis for
'M12067.metal.pos.txt.gz' on 'JUVEN_ARTHR.gz'

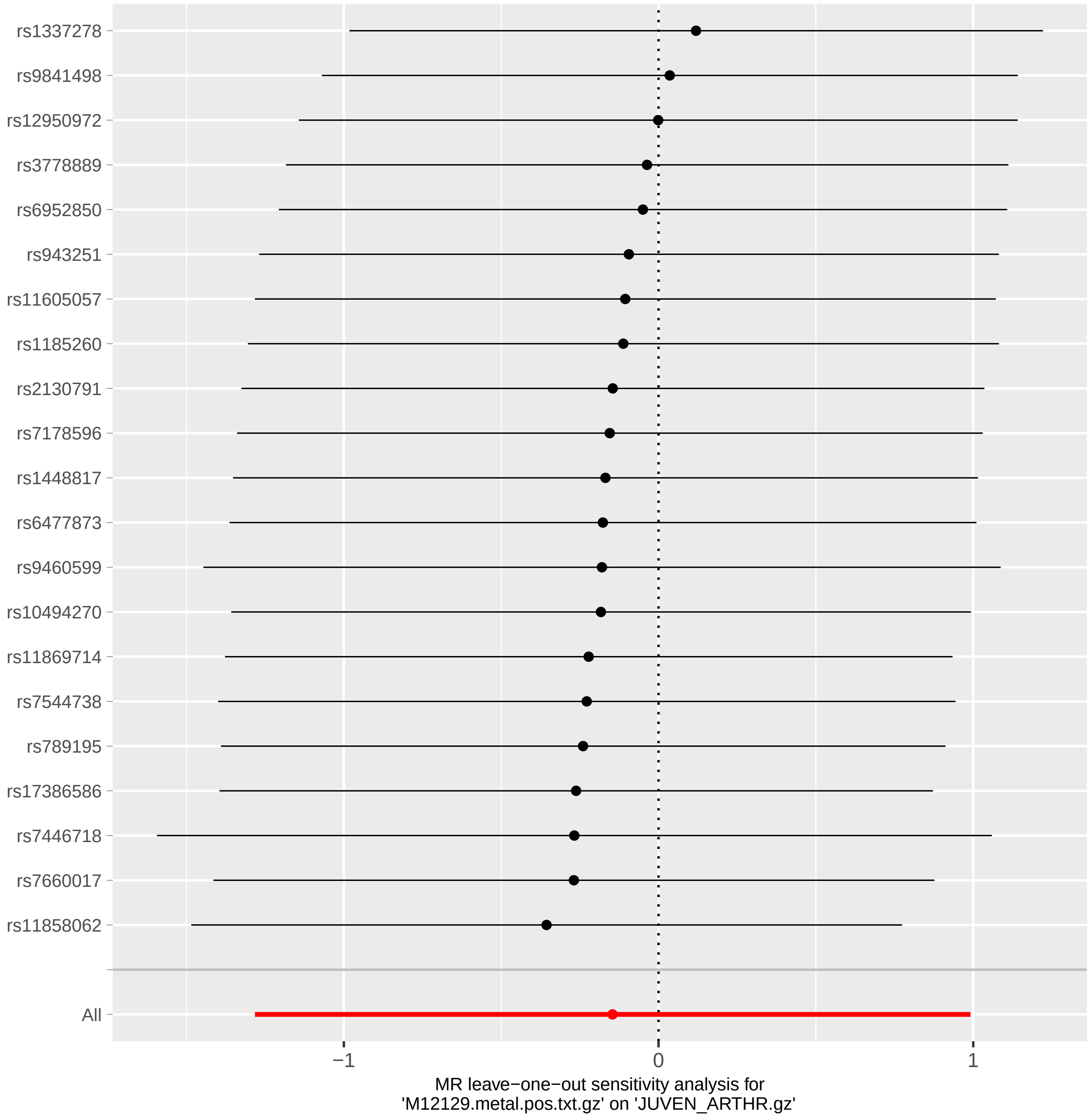
rs1337278
rs9841498
rs12950972
rs3778889
rs6952850
rs943251
rs11605057
rs1185260
rs2130791
rs7178596
rs1448817
rs6477873
rs9460599
rs10494270
rs11869714
rs7544738
rs789195
rs17386586
rs7446718
rs7660017
rs11858062
All
−1
0
1
MR leave−one−out sensitivity analysis for
'M12129.metal.pos.txt.gz' on 'JUVEN_ARTHR.gz'

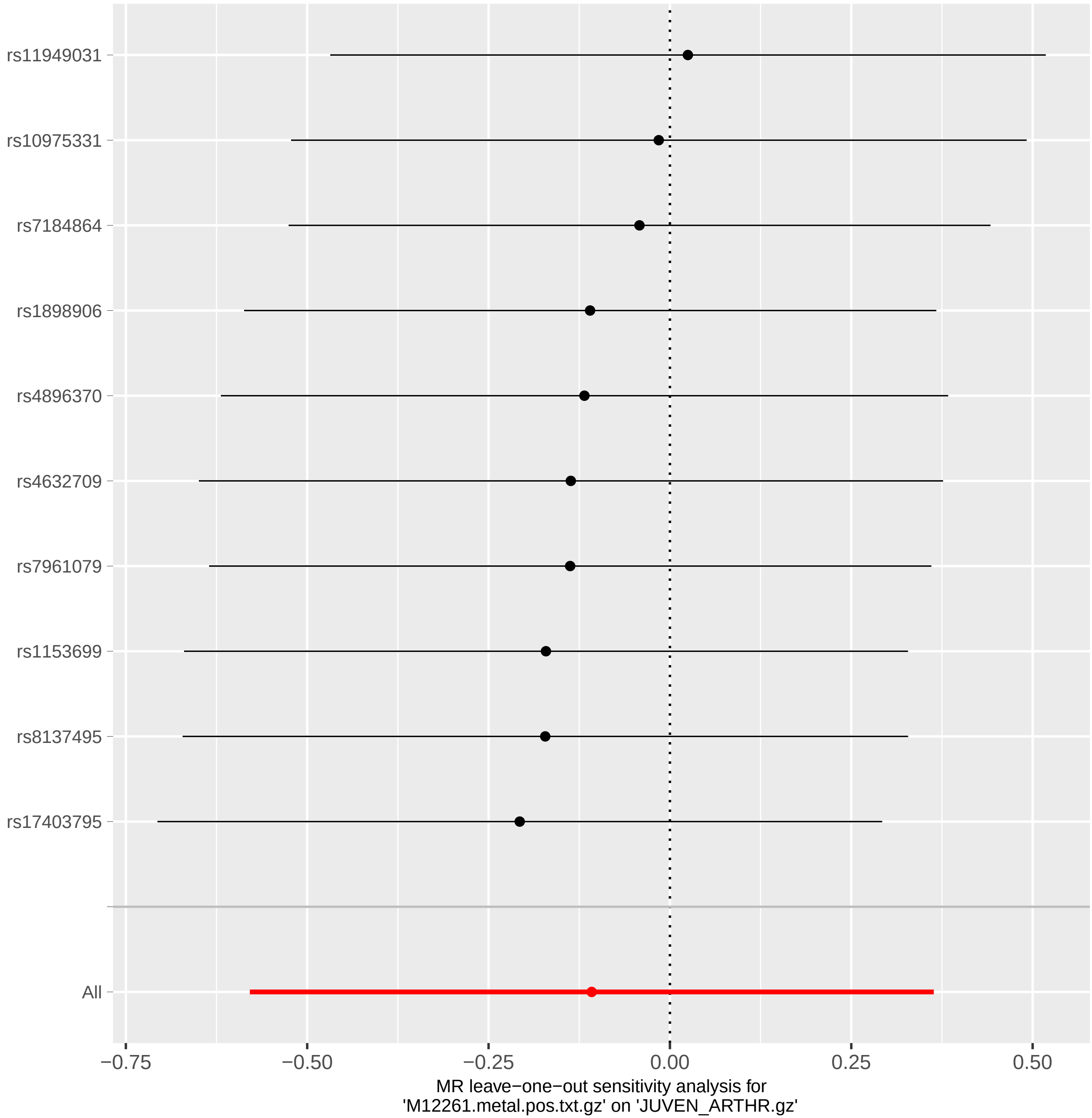
rs11949031
rs10975331
rs7184864
rs1898906
rs4896370
rs4632709
rs7961079
rs1153699
rs8137495
rs17403795
All
−0.75
−0.50
−0.25
0.00
0.25
0.50
MR leave−one−out sensitivity analysis for
'M12261.metal.pos.txt.gz' on 'JUVEN_ARTHR.gz'

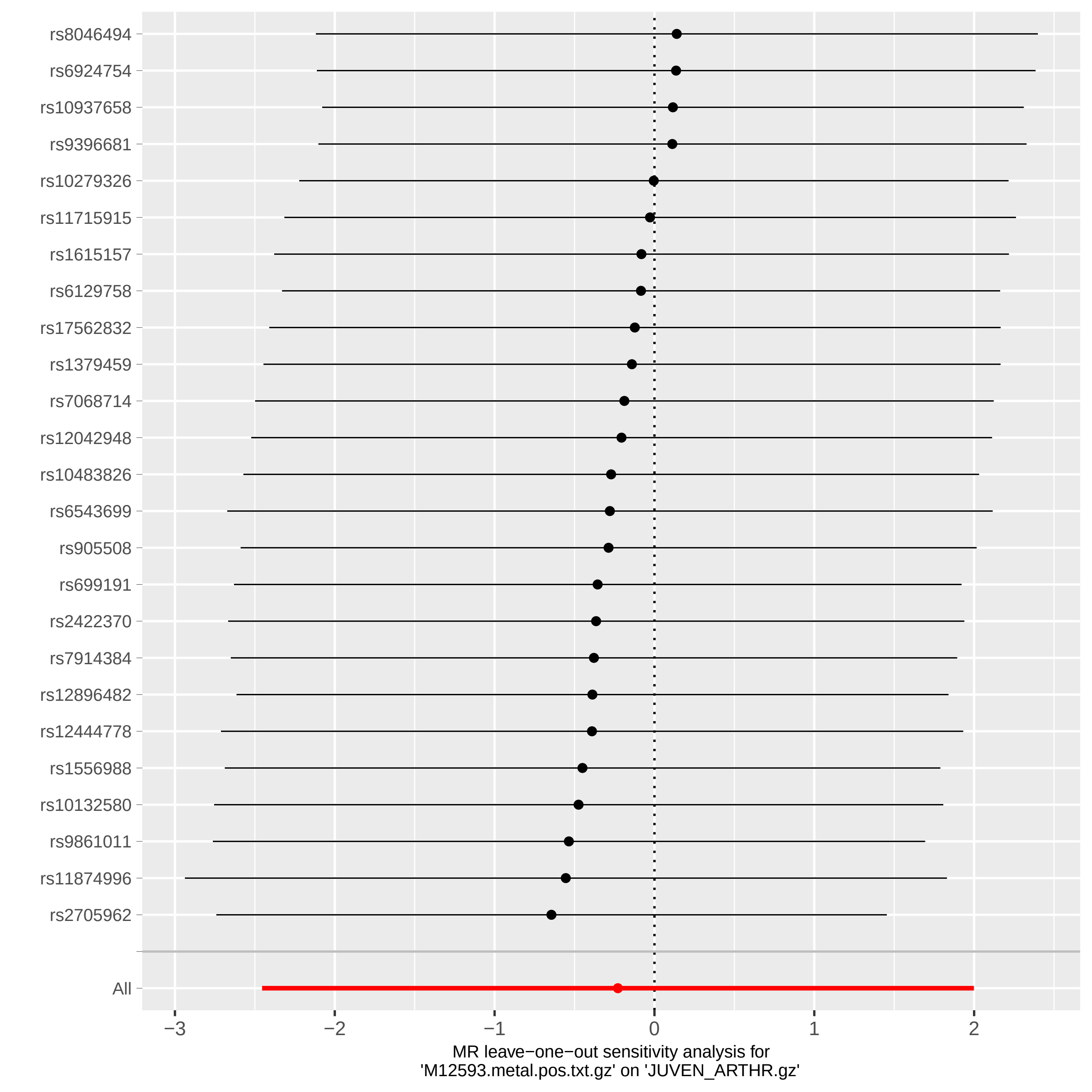
rs8046494
rs6924754
rs10937658
rs9396681
rs10279326
rs11715915
rs1615157
rs6129758
rs17562832
rs1379459
rs7068714
rs12042948
rs10483826
rs6543699
rs905508
rs699191
rs2422370
rs7914384
rs12896482
rs12444778
rs1556988
rs10132580
rs9861011
rs11874996
rs2705962
All
−3
−2
−1
0
1
2
MR leave−one−out sensitivity analysis for
'M12593.metal.pos.txt.gz' on 'JUVEN_ARTHR.gz'

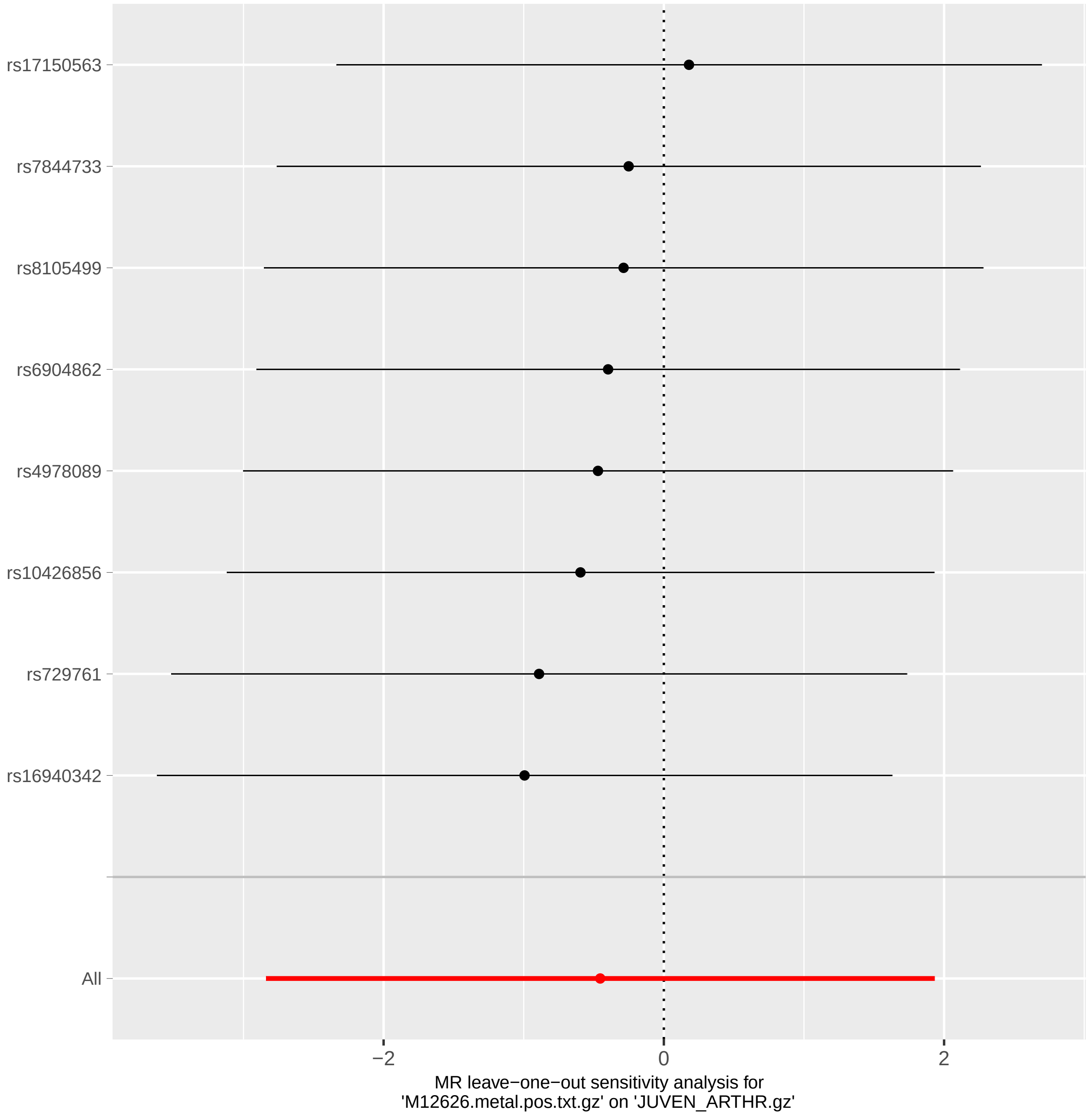
rs17150563
rs7844733
rs8105499
rs6904862
rs4978089
rs10426856
rs729761
rs16940342
All
−2
0
2
MR leave−one−out sensitivity analysis for
'M12626.metal.pos.txt.gz' on 'JUVEN_ARTHR.gz'

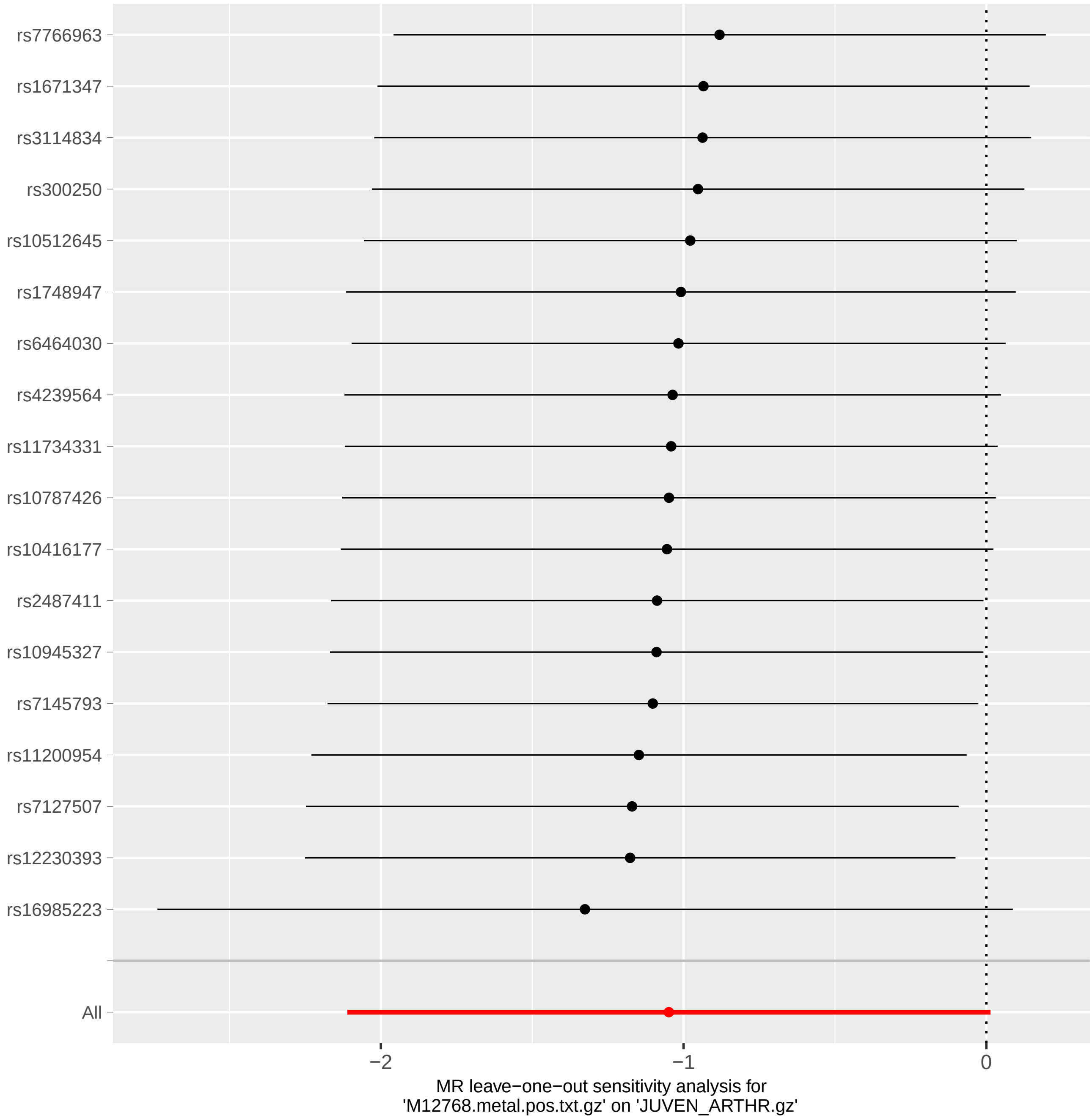
rs7766963
rs1671347
rs3114834
rs300250
rs10512645
rs1748947
rs6464030
rs4239564
rs11734331
rs10787426
rs10416177
rs2487411
rs10945327
rs7145793
rs11200954
rs7127507
rs12230393
rs16985223
All
−2
−1
0
MR leave−one−out sensitivity analysis for
'M12768.metal.pos.txt.gz' on 'JUVEN_ARTHR.gz'

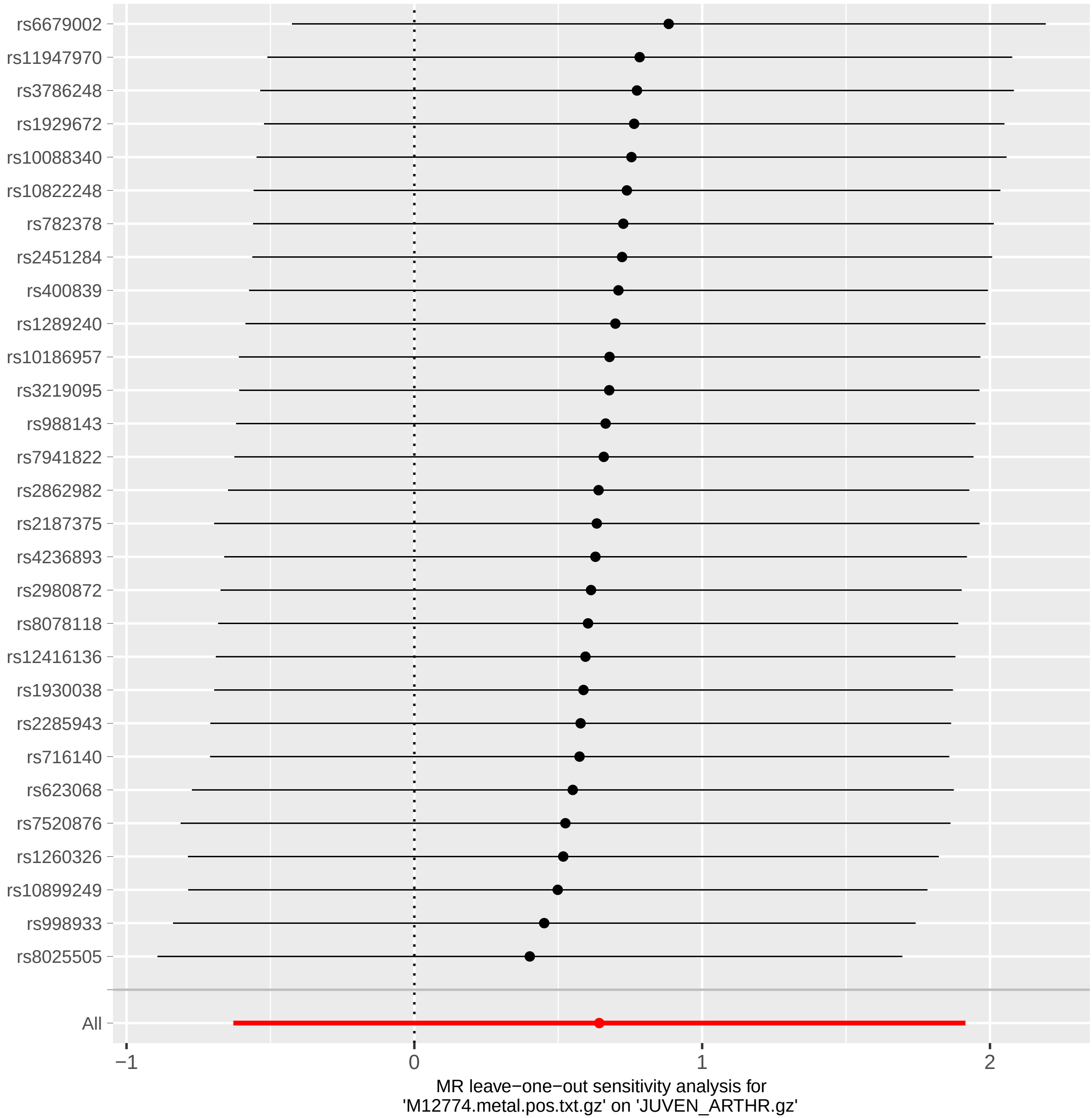
rs6679002
rs11947970
rs3786248
rs1929672
rs10088340
rs10822248
rs782378
rs2451284
rs400839
rs1289240
rs10186957
rs3219095
rs988143
rs7941822
rs2862982
rs2187375
rs4236893
rs2980872
rs8078118
rs12416136
rs1930038
rs2285943
rs716140
rs623068
rs7520876
rs1260326
rs10899249
rs998933
rs8025505
All
−1
0
1
2
MR leave−one−out sensitivity analysis for
'M12774.metal.pos.txt.gz' on 'JUVEN_ARTHR.gz'

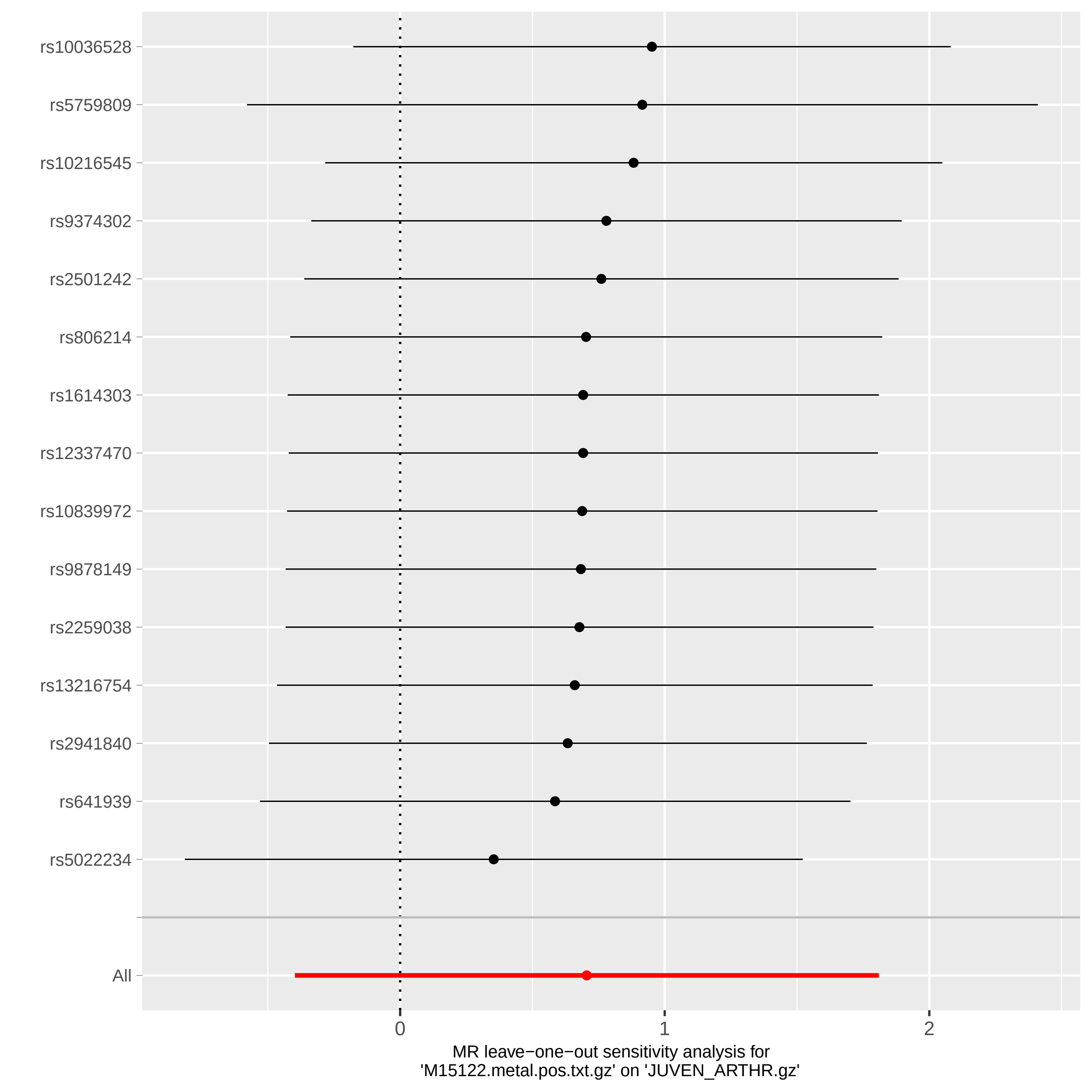
rs10036528
rs5759809
rs10216545
rs9374302
rs2501242
rs806214
rs1614303
rs12337470
rs10839972
rs9878149
rs2259038
rs13216754
rs2941840
rs641939
rs5022234
All
0
1
2
MR leave−one−out sensitivity analysis for
'M15122.metal.pos.txt.gz' on 'JUVEN_ARTHR.gz'

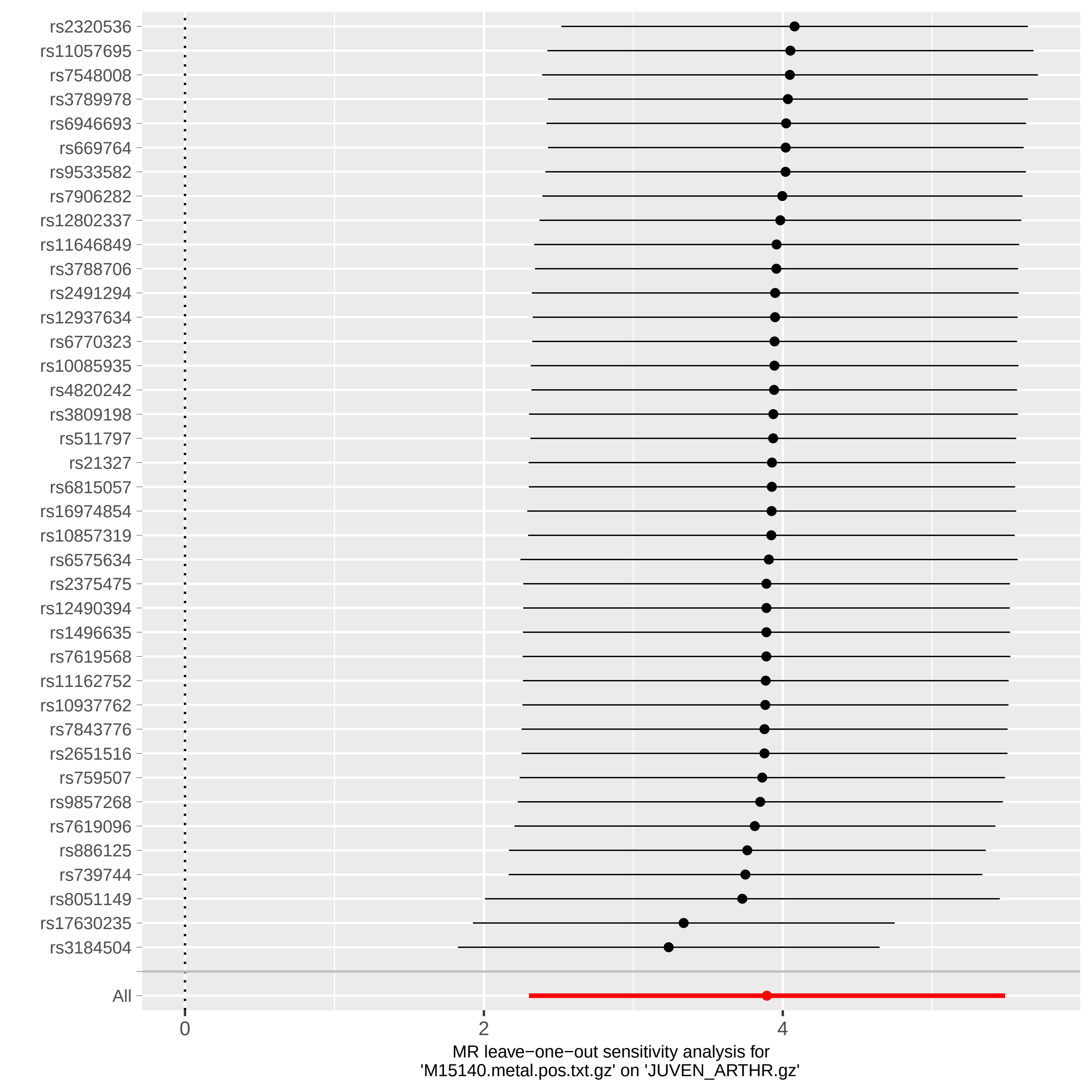
rs2320536
rs11057695
rs7548008
rs3789978
rs6946693
rs669764
rs9533582
rs7906282
rs12802337
rs11646849
rs3788706
rs2491294
rs12937634
rs6770323
rs10085935
rs4820242
rs3809198
rs511797
rs21327
rs6815057
rs16974854
rs10857319
rs6575634
rs2375475
rs12490394
rs1496635
rs7619568
rs11162752
rs10937762
rs7843776
rs2651516
rs759507
rs9857268
rs7619096
rs886125
rs739744
rs8051149
rs17630235
rs3184504
All
0
2
4
MR leave−one−out sensitivity analysis for
'M15140.metal.pos.txt.gz' on 'JUVEN_ARTHR.gz'

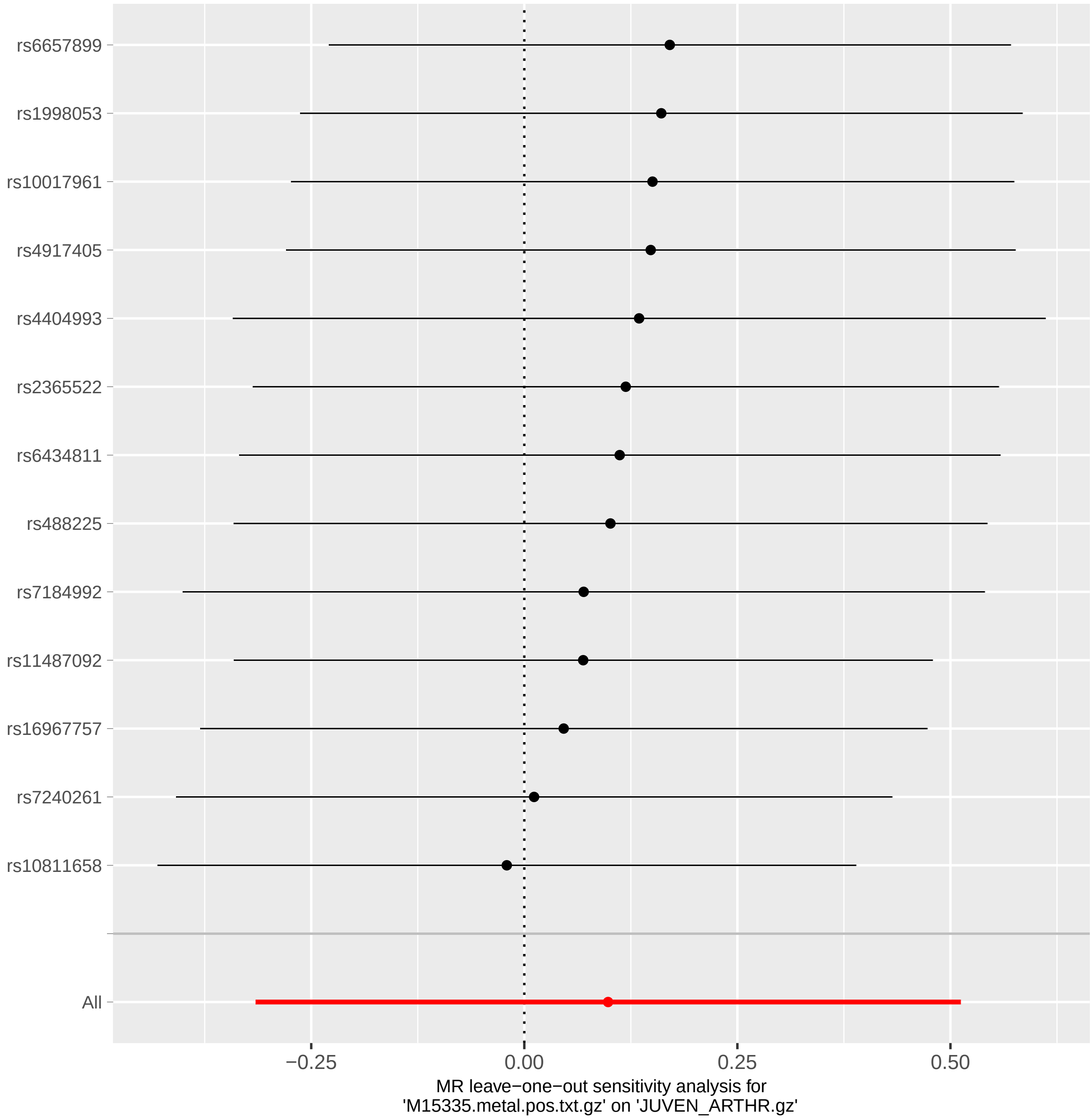
rs6657899
rs1998053
rs10017961
rs4917405
rs4404993
rs2365522
rs6434811
rs488225
rs7184992
rs11487092
rs16967757
rs7240261
rs10811658
All
−0.25
0.00
0.25
0.50
MR leave−one−out sensitivity analysis for
'M15335.metal.pos.txt.gz' on 'JUVEN_ARTHR.gz'

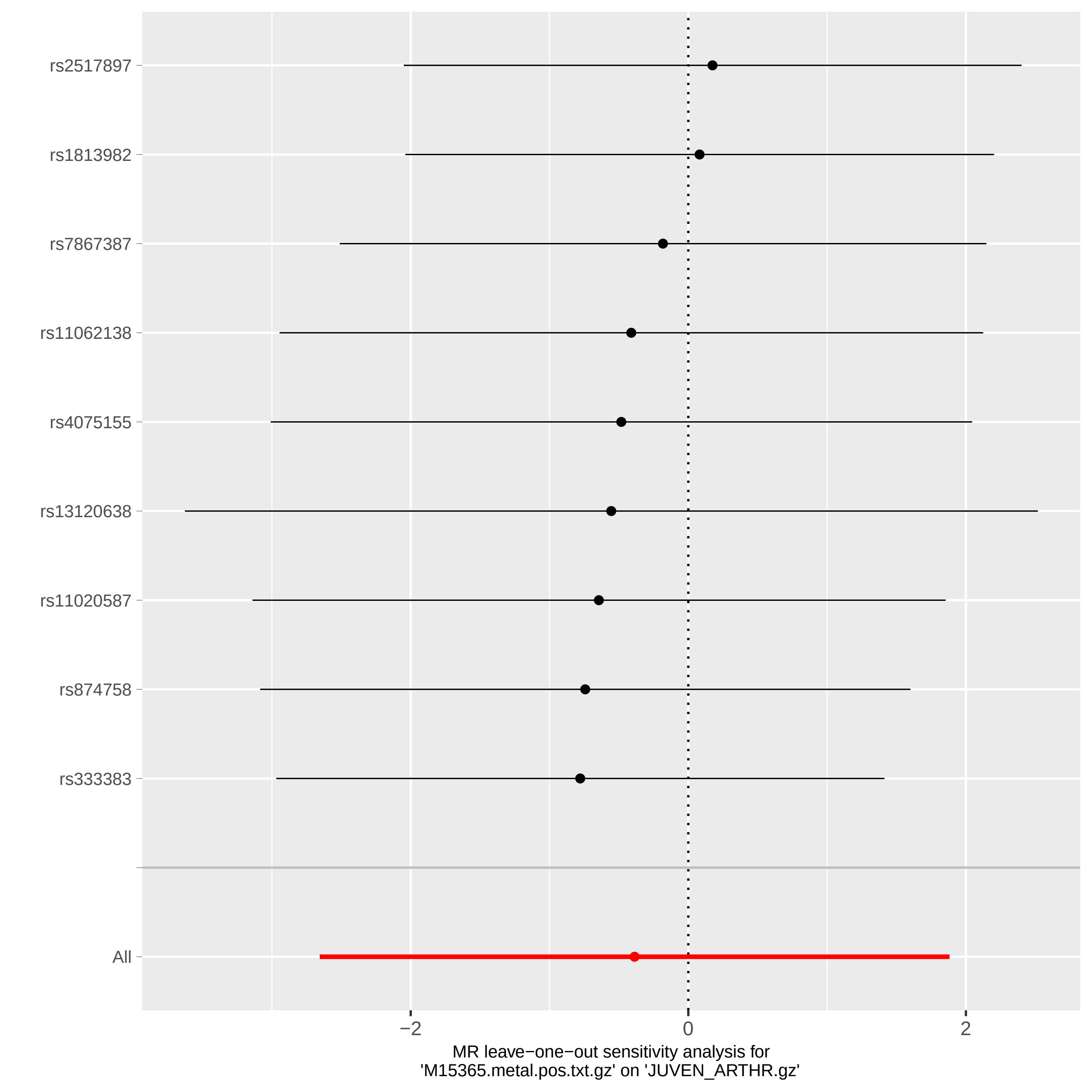
rs2517897
rs1813982
rs7867387
rs11062138
rs4075155
rs13120638
rs11020587
rs874758
rs333383
All
−2
0
2
MR leave−one−out sensitivity analysis for
'M15365.metal.pos.txt.gz' on 'JUVEN_ARTHR.gz'

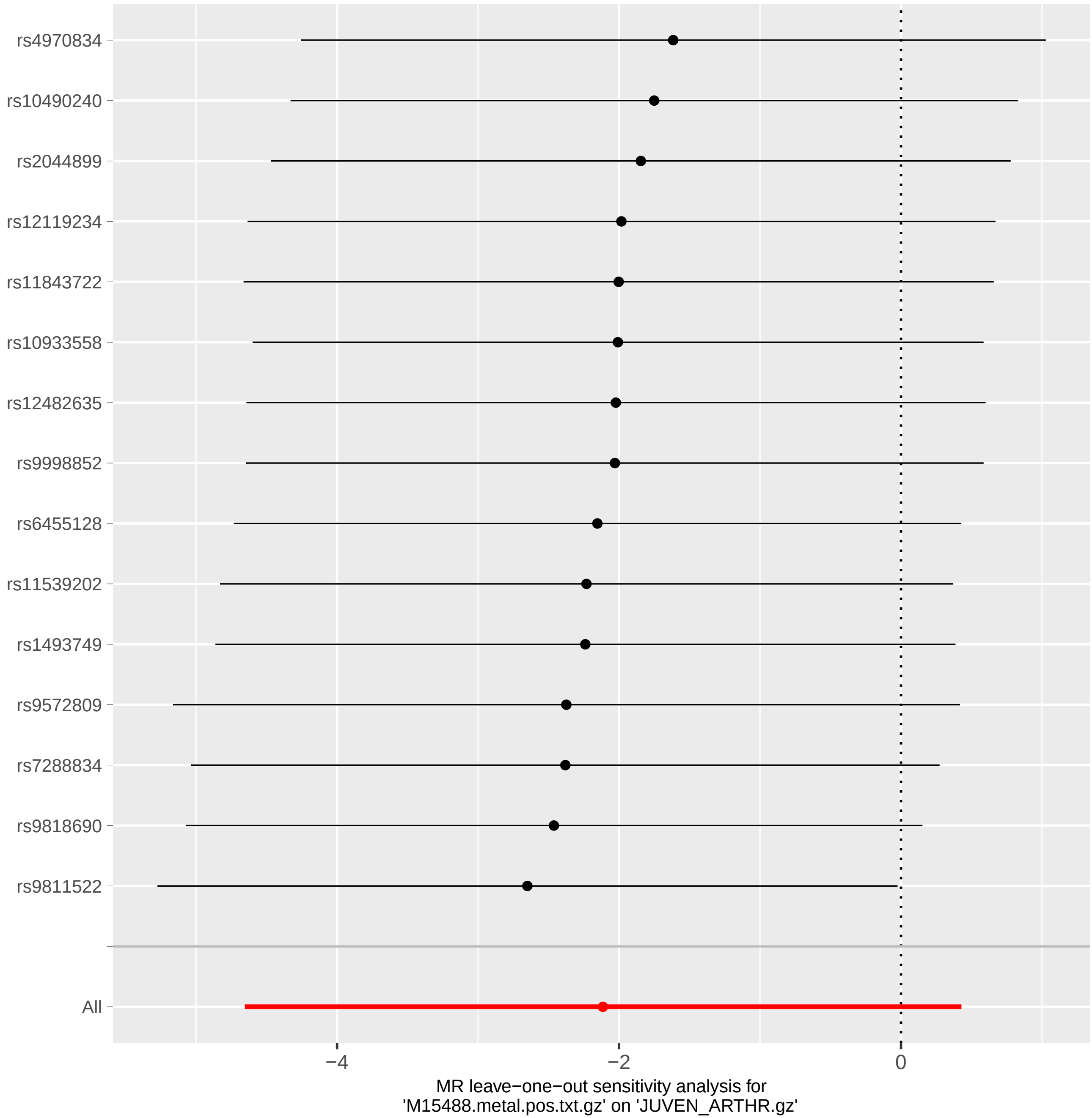
rs4970834
rs10490240
rs2044899
rs12119234
rs11843722
rs10933558
rs12482635
rs9998852
rs6455128
rs11539202
rs1493749
rs9572809
rs7288834
rs9818690
rs9811522
All
−4
−2
0
MR leave−one−out sensitivity analysis for
'M15488.metal.pos.txt.gz' on 'JUVEN_ARTHR.gz'

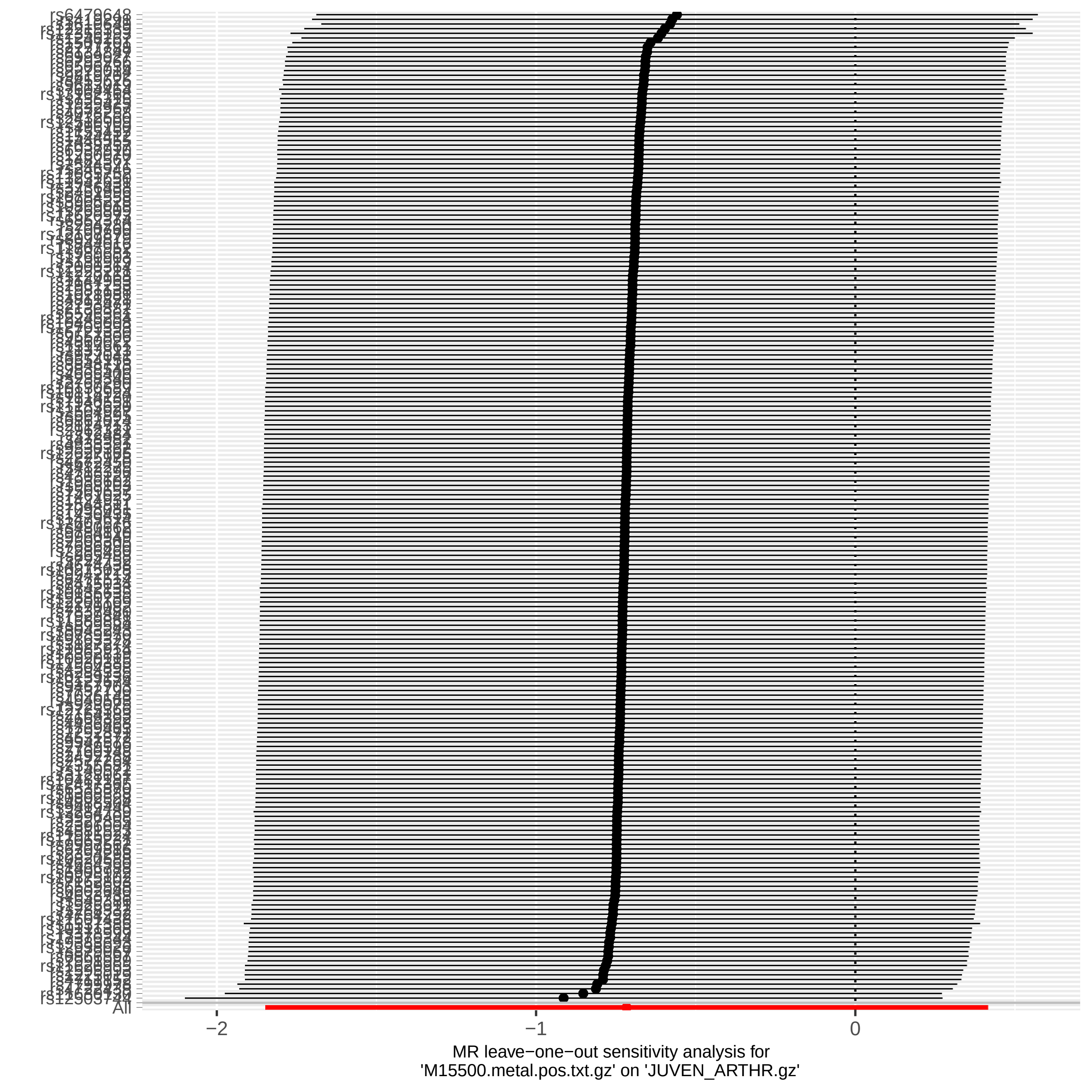

rs6470648
rs12903744
All
−2
−1
0
MR leave−one−out sensitivity analysis for
'M15500.metal.pos.txt.gz' on 'JUVEN_ARTHR.gz'

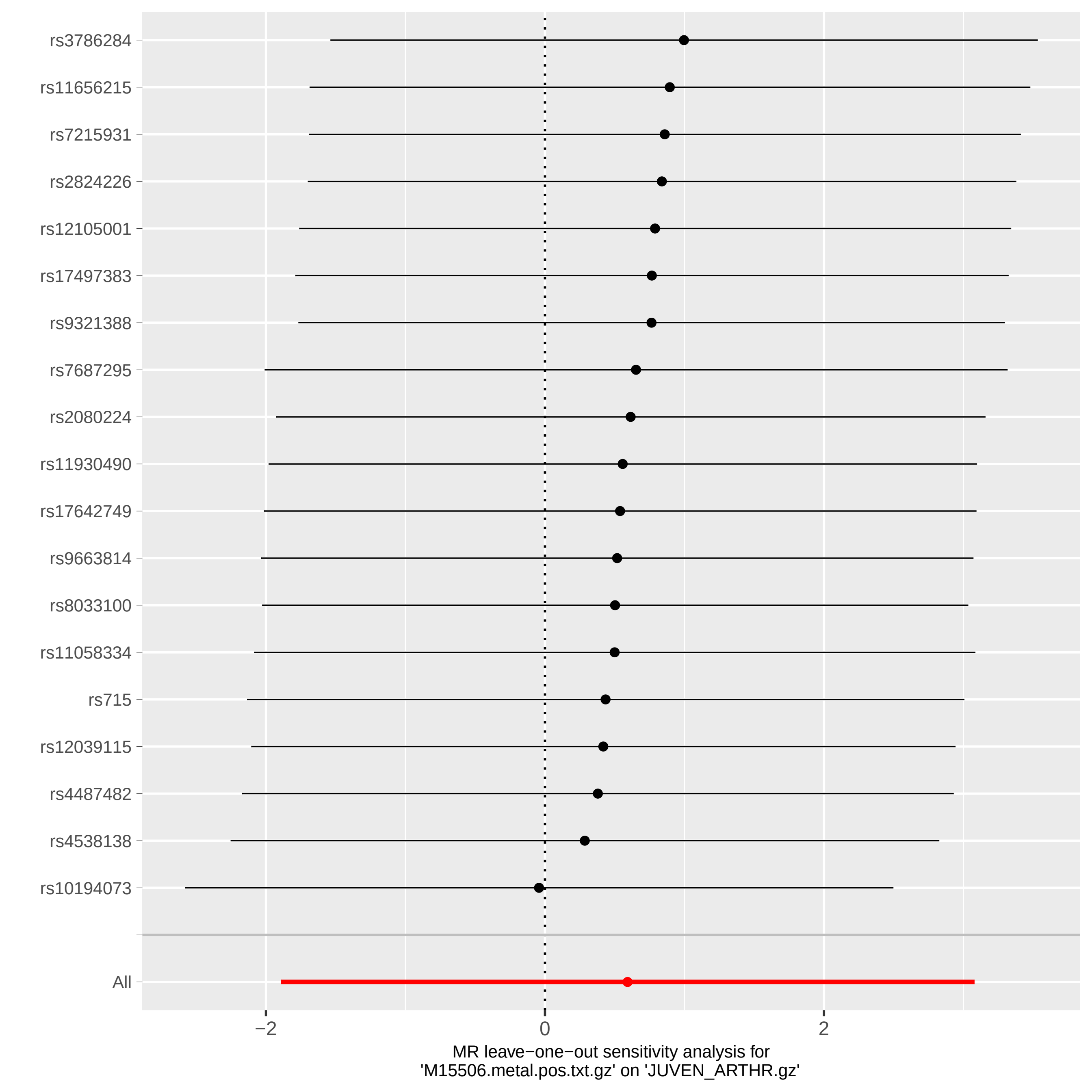

rs3786284
rs11656215
rs7215931
rs2824226
rs12105001
rs17497383
rs9321388
rs7687295
rs2080224
rs11930490
rs17642749
rs9663814
rs8033100
rs11058334
rs715
rs12039115
rs4487482
rs4538138
rs10194073
All
−2
0
2
MR leave−one−out sensitivity analysis for
'M15506.metal.pos.txt.gz' on 'JUVEN_ARTHR.gz'

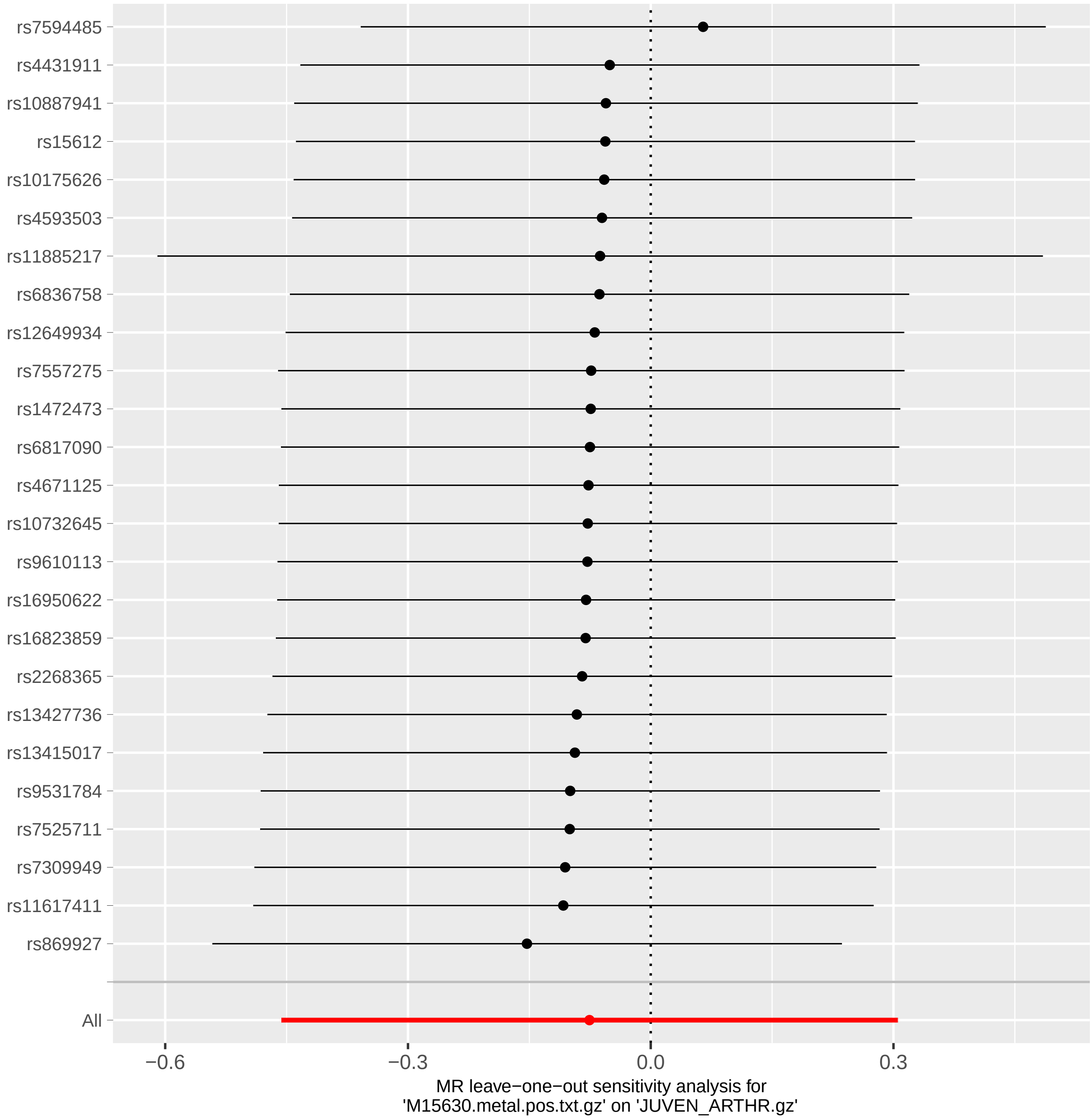
rs7594485
rs4431911
rs10887941
rs15612
rs10175626
rs4593503
rs11885217
rs6836758
rs12649934
rs7557275
rs1472473
rs6817090
rs4671125
rs10732645
rs9610113
rs16950622
rs16823859
rs2268365
rs13427736
rs13415017
rs9531784
rs7525711
rs7309949
rs11617411
rs869927
All
−0.6
−0.3
0.0
0.3
MR leave−one−out sensitivity analysis for
'M15630.metal.pos.txt.gz' on 'JUVEN_ARTHR.gz'

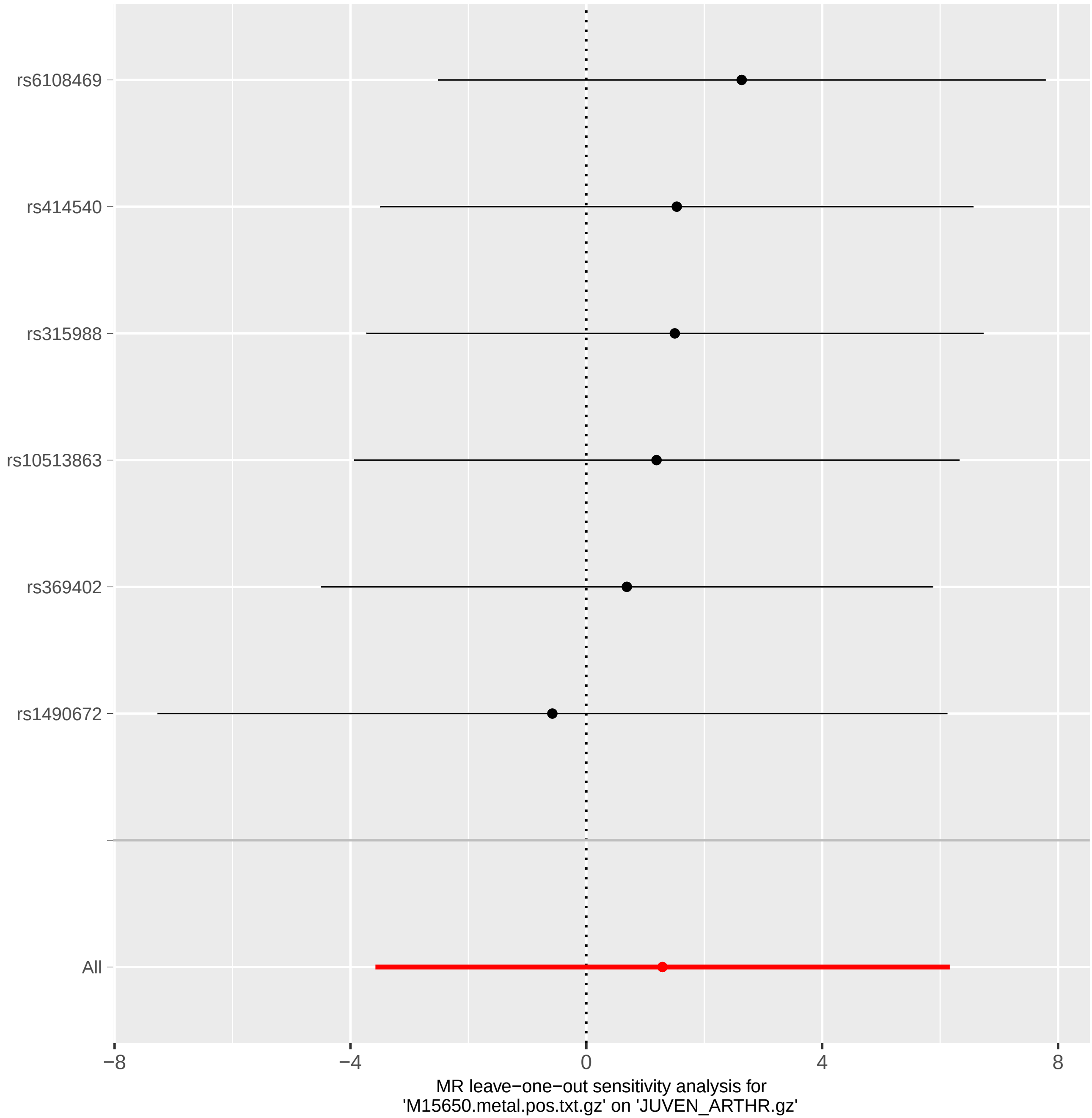
rs6108469
rs414540
rs315988
rs10513863
rs369402
rs1490672
All
−8
−4
0
4
8
MR leave−one−out sensitivity analysis for
'M15650.metal.pos.txt.gz' on 'JUVEN_ARTHR.gz'

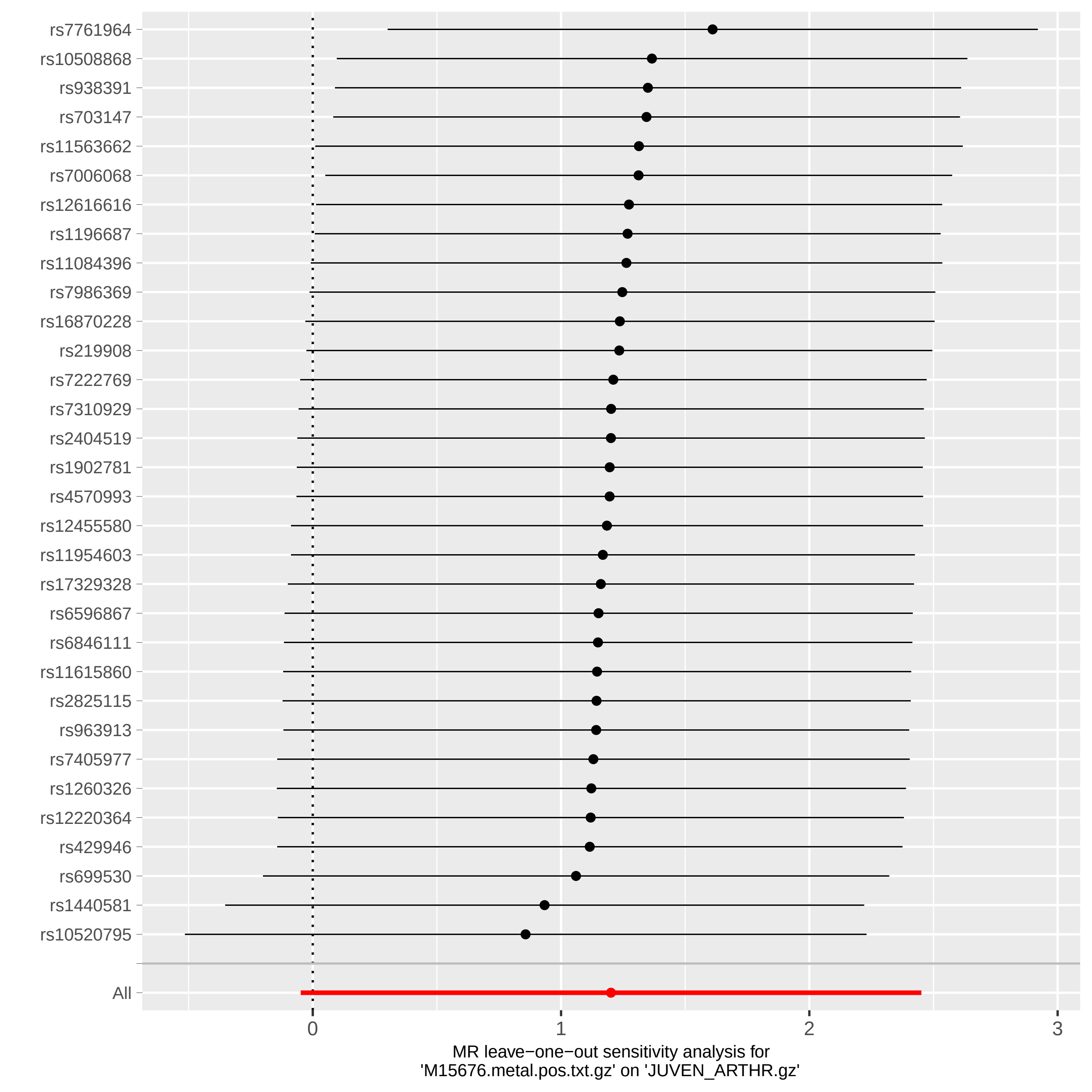

rs7761964
rs10508868
rs938391
rs703147
rs11563662
rs7006068
rs12616616
rs1196687
rs11084396
rs7986369
rs16870228
rs219908
rs7222769
rs7310929
rs2404519
rs1902781
rs4570993
rs12455580
rs11954603
rs17329328
rs6596867
rs6846111
rs11615860
rs2825115
rs963913
rs7405977
rs1260326
rs12220364
rs429946
rs699530
rs1440581
rs10520795
All
0
1
2
3
MR leave−one−out sensitivity analysis for
'M15676.metal.pos.txt.gz' on 'JUVEN_ARTHR.gz'

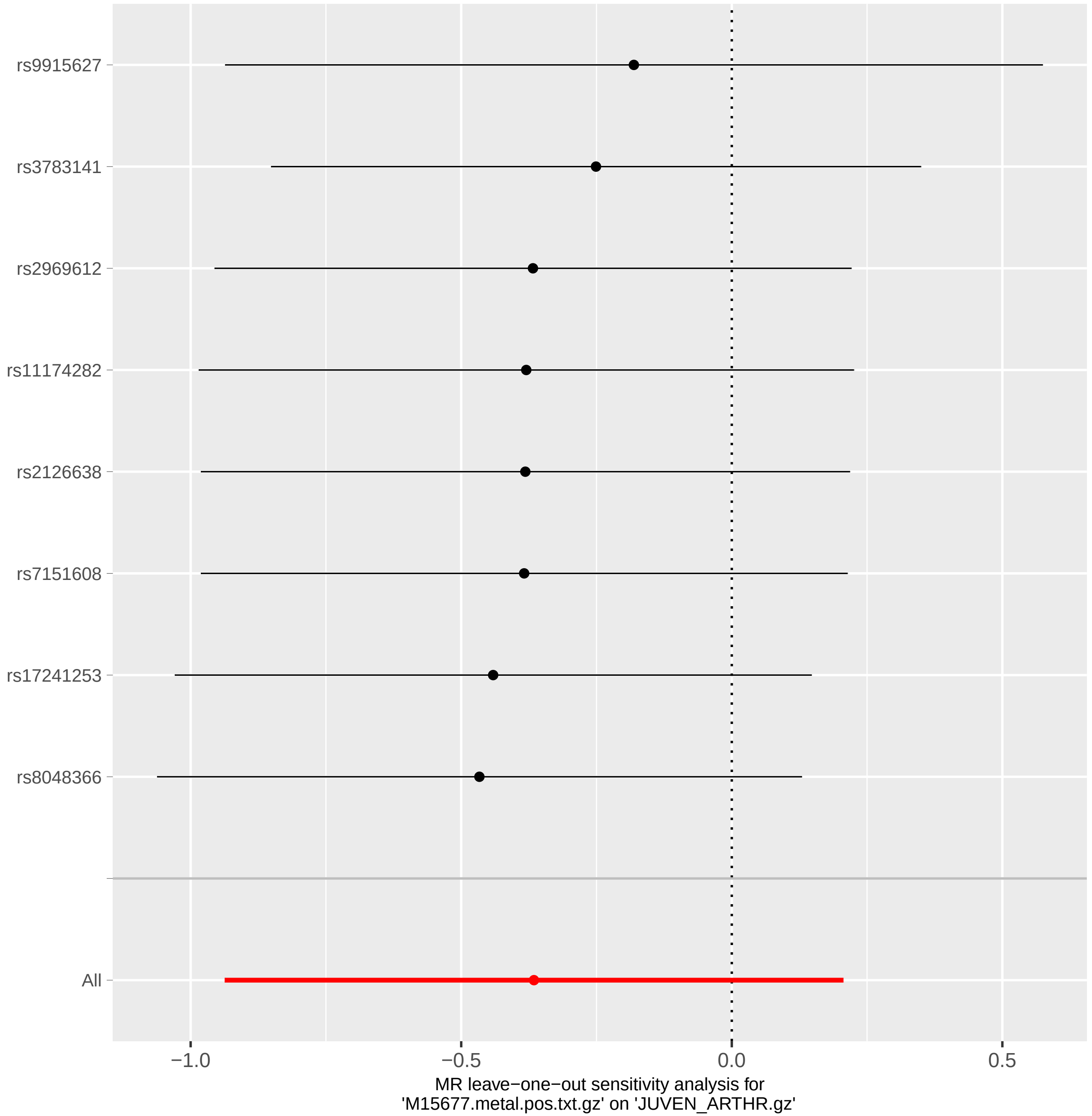
rs9915627
rs3783141
rs2969612
rs11174282
rs2126638
rs7151608
rs17241253
rs8048366
All
−1.0
−0.5
0.0
0.5
MR leave−one−out sensitivity analysis for
'M15677.metal.pos.txt.gz' on 'JUVEN_ARTHR.gz'

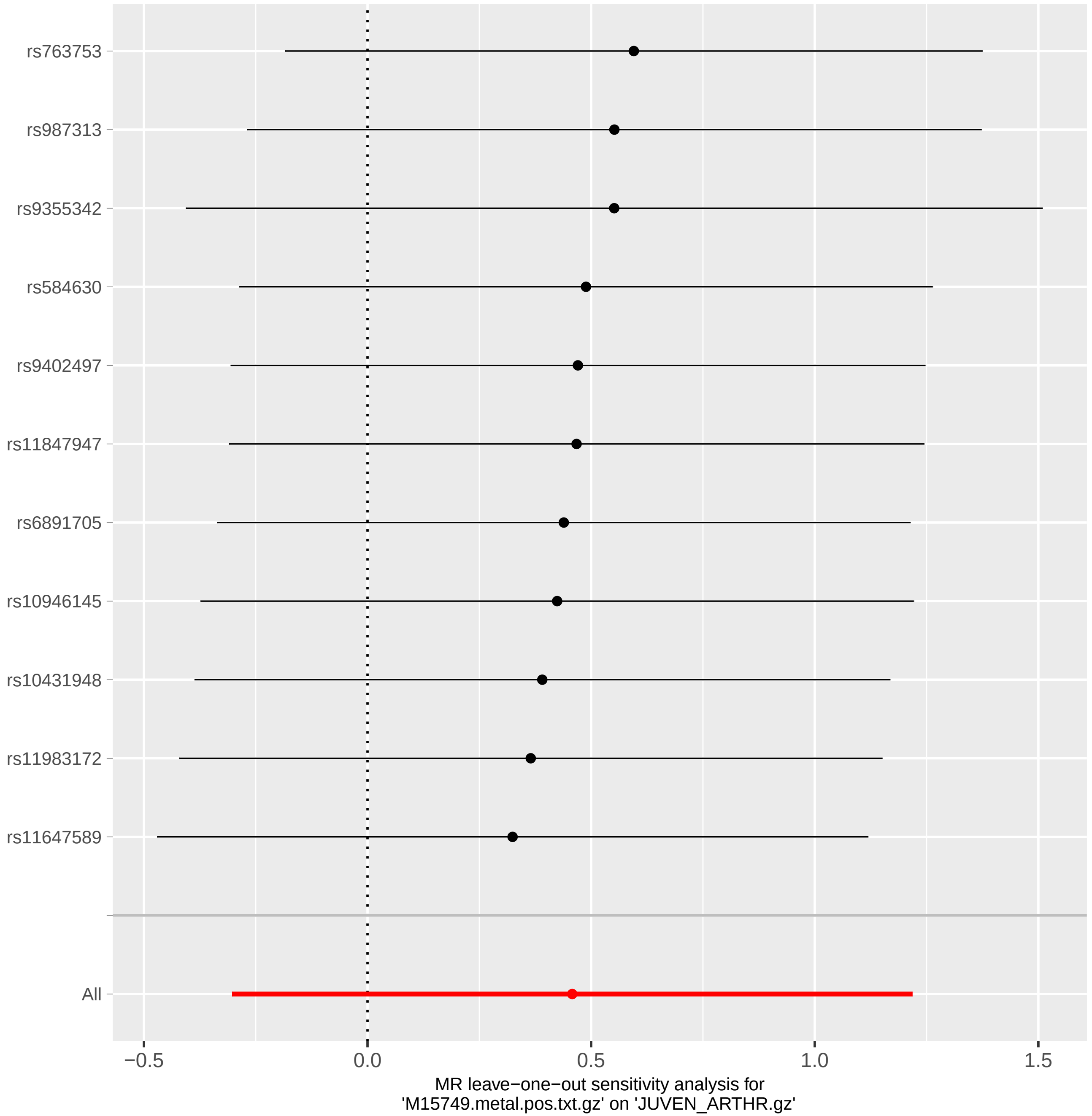
rs763753
rs987313
rs9355342
rs584630
rs9402497
rs11847947
rs6891705
rs10946145
rs10431948
rs11983172
rs11647589
All
−0.5
0.0
0.5
1.0
1.5
MR leave−one−out sensitivity analysis for
'M15749.metal.pos.txt.gz' on 'JUVEN_ARTHR.gz'

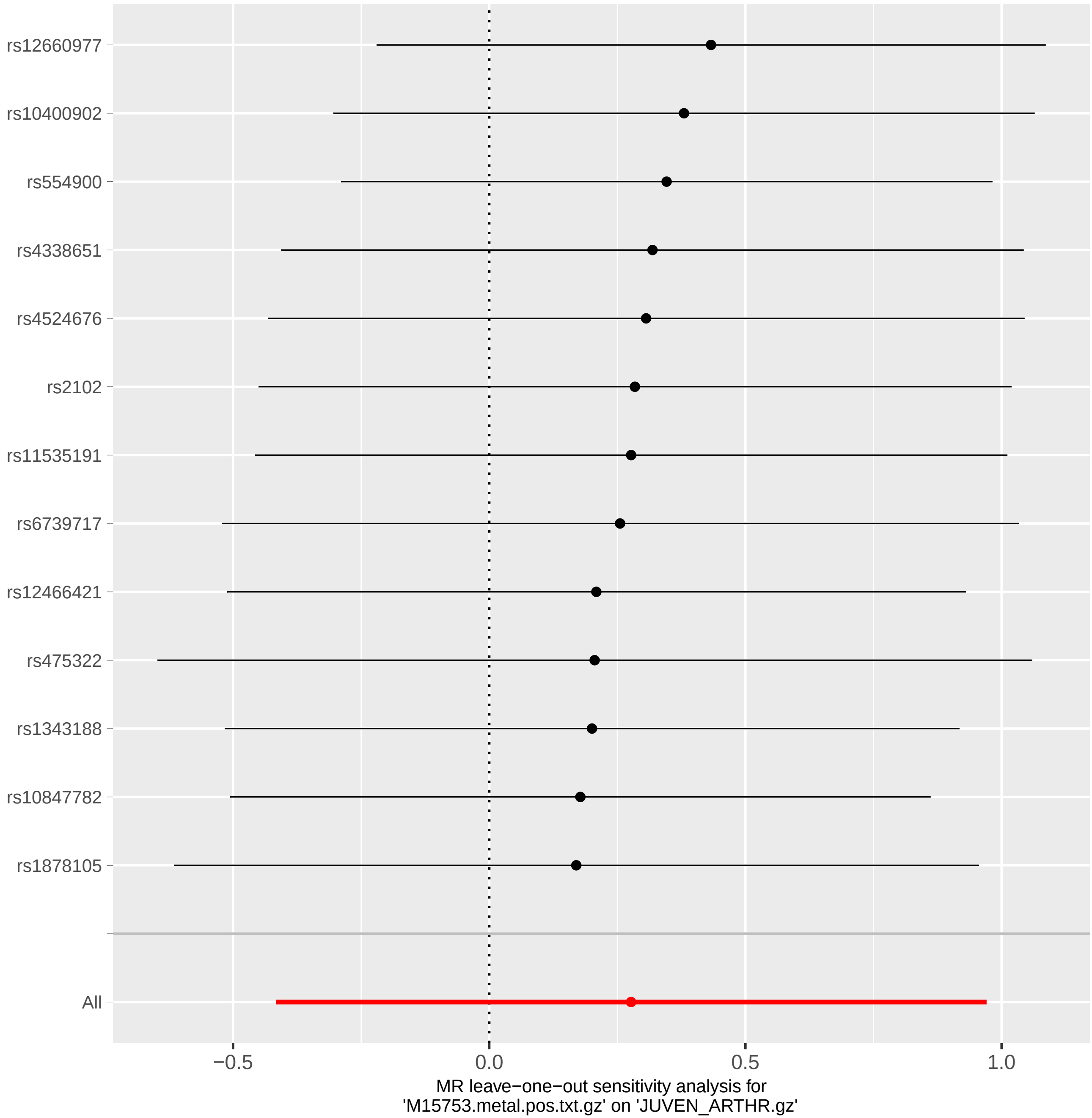

rs12660977
rs10400902
rs554900
rs4338651
rs4524676
rs2102
rs11535191
rs6739717
rs12466421
rs475322
rs1343188
rs10847782
rs1878105
All
−0.5
0.0
0.5
1.0
MR leave−one−out sensitivity analysis for
'M15753.metal.pos.txt.gz' on 'JUVEN_ARTHR.gz'

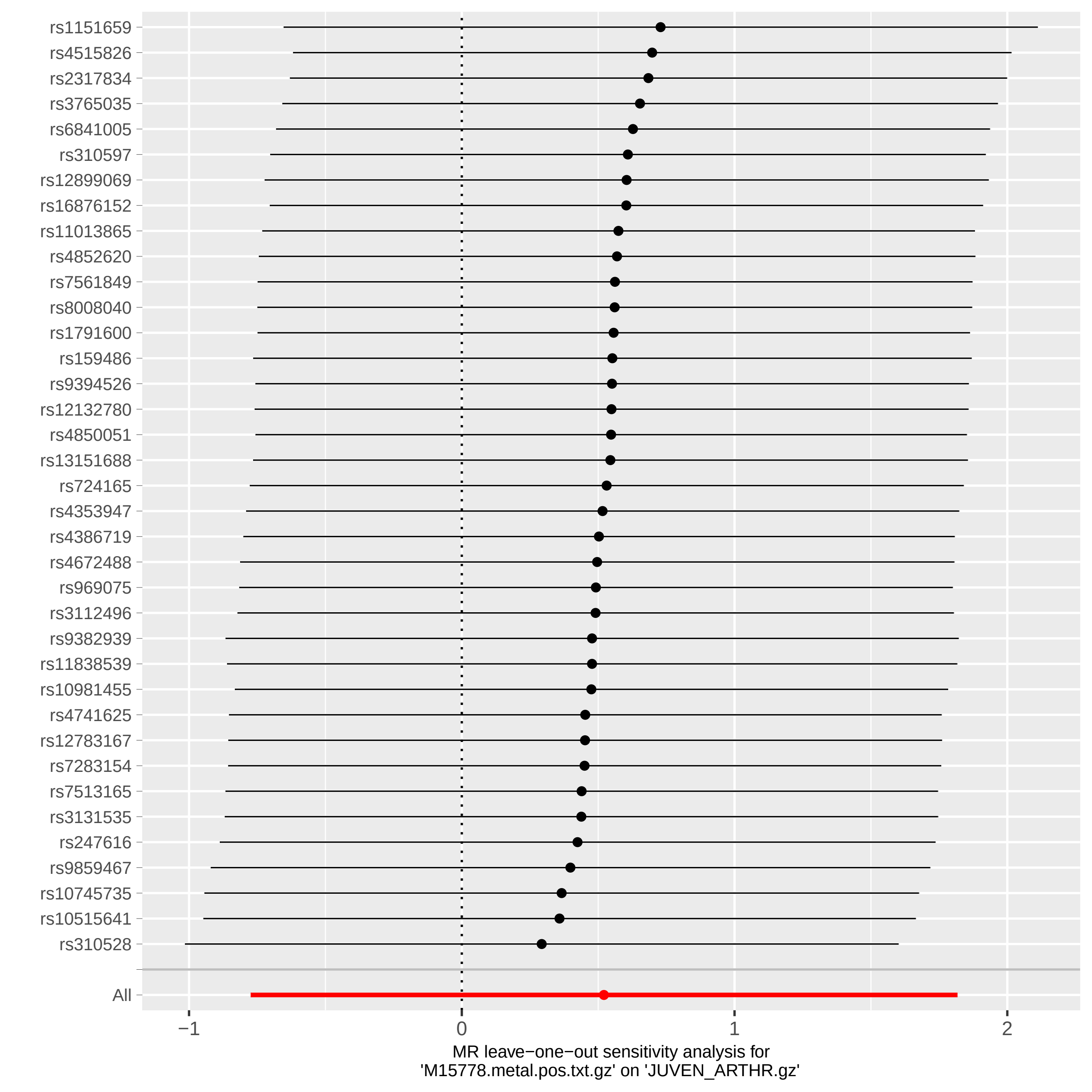
rs1151659
rs4515826
rs2317834
rs3765035
rs6841005
rs310597
rs12899069
rs16876152
rs11013865
rs4852620
rs7561849
rs8008040
rs1791600
rs159486
rs9394526
rs12132780
rs4850051
rs13151688
rs724165
rs4353947
rs4386719
rs4672488
rs969075
rs3112496
rs9382939
rs11838539
rs10981455
rs4741625
rs12783167
rs7283154
rs7513165
rs3131535
rs247616
rs9859467
rs10745735
rs10515641
rs310528
All
−1
0
1
2
MR leave−one−out sensitivity analysis for
'M15778.metal.pos.txt.gz' on 'JUVEN_ARTHR.gz'

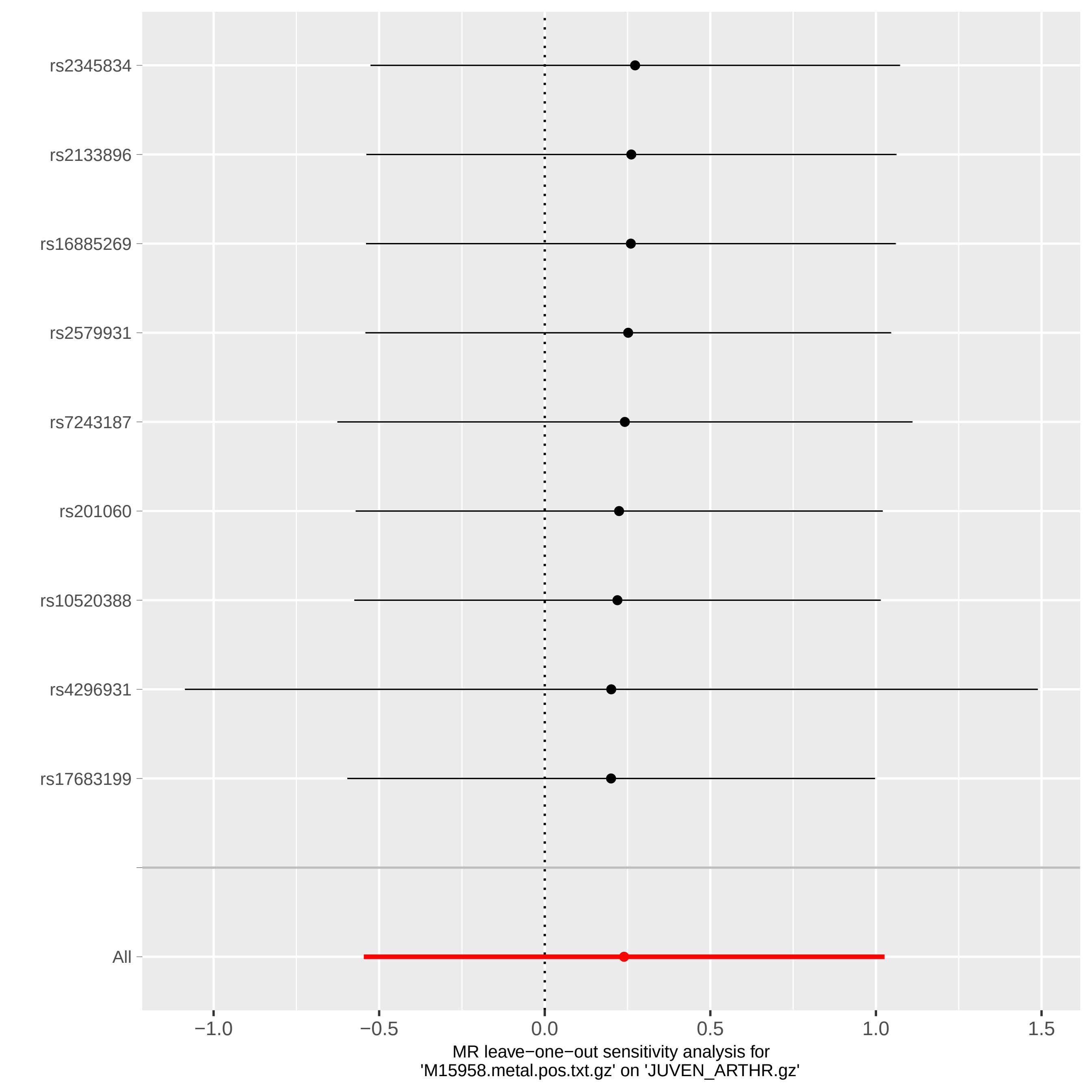
rs2345834
rs2133896
rs16885269
rs2579931
rs7243187
rs201060
rs10520388
rs4296931
rs17683199
All
−1.0
−0.5
0.0
0.5
1.0
1.5
MR leave−one−out sensitivity analysis for
'M15958.metal.pos.txt.gz' on 'JUVEN_ARTHR.gz'

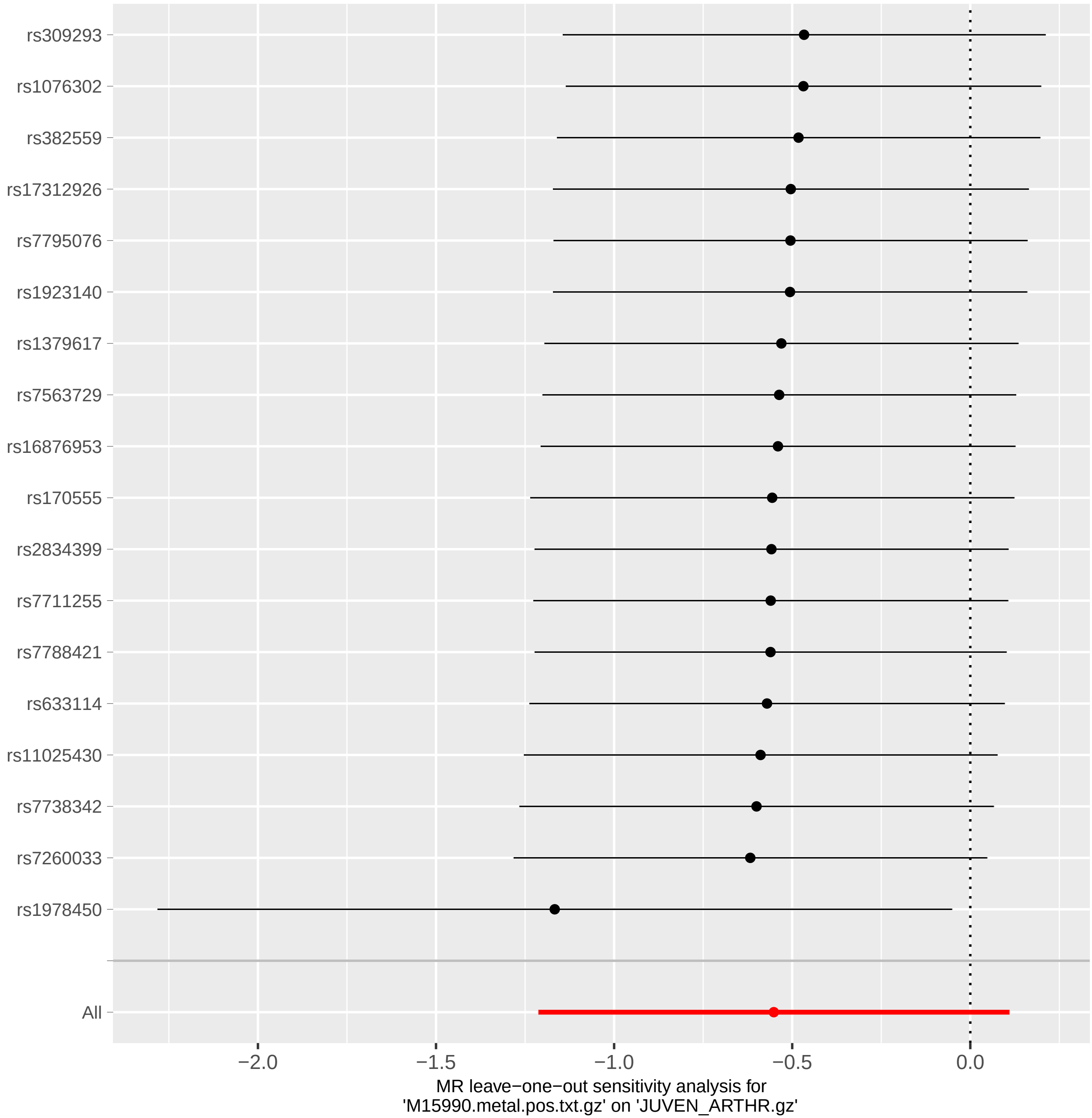

rs309293
rs1076302
rs382559
rs17312926
rs7795076
rs1923140
rs1379617
rs7563729
rs16876953
rs170555
rs2834399
rs7711255
rs7788421
rs633114
rs11025430
rs7738342
rs7260033
rs1978450
All
−2.0
−1.5
−1.0
−0.5
0.0
MR leave−one−out sensitivity analysis for
'M15990.metal.pos.txt.gz' on 'JUVEN_ARTHR.gz'

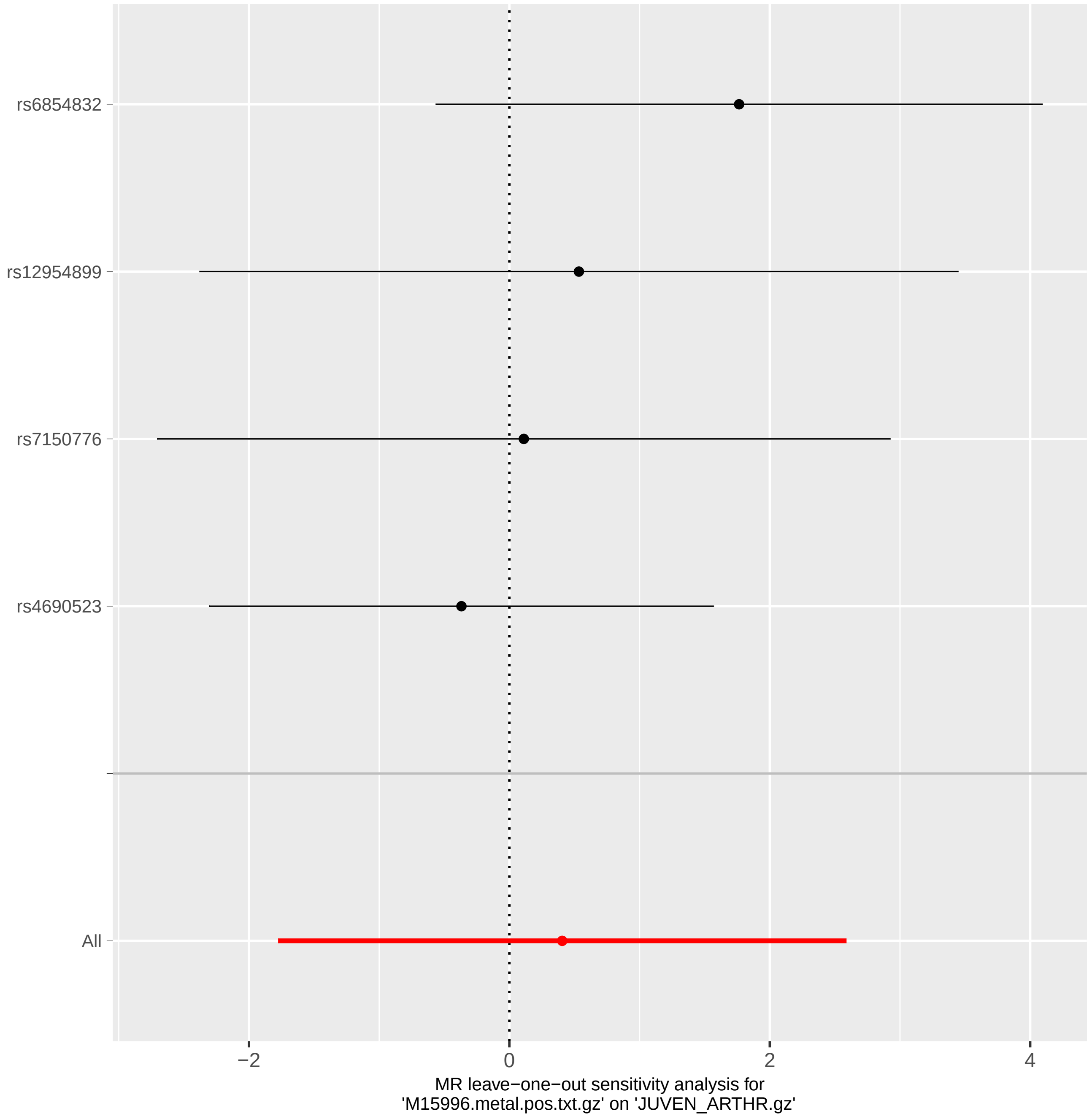
rs6854832
rs12954899
rs7150776
rs4690523
All
−2
0
2
4
MR leave−one−out sensitivity analysis for
'M15996.metal.pos.txt.gz' on 'JUVEN_ARTHR.gz'

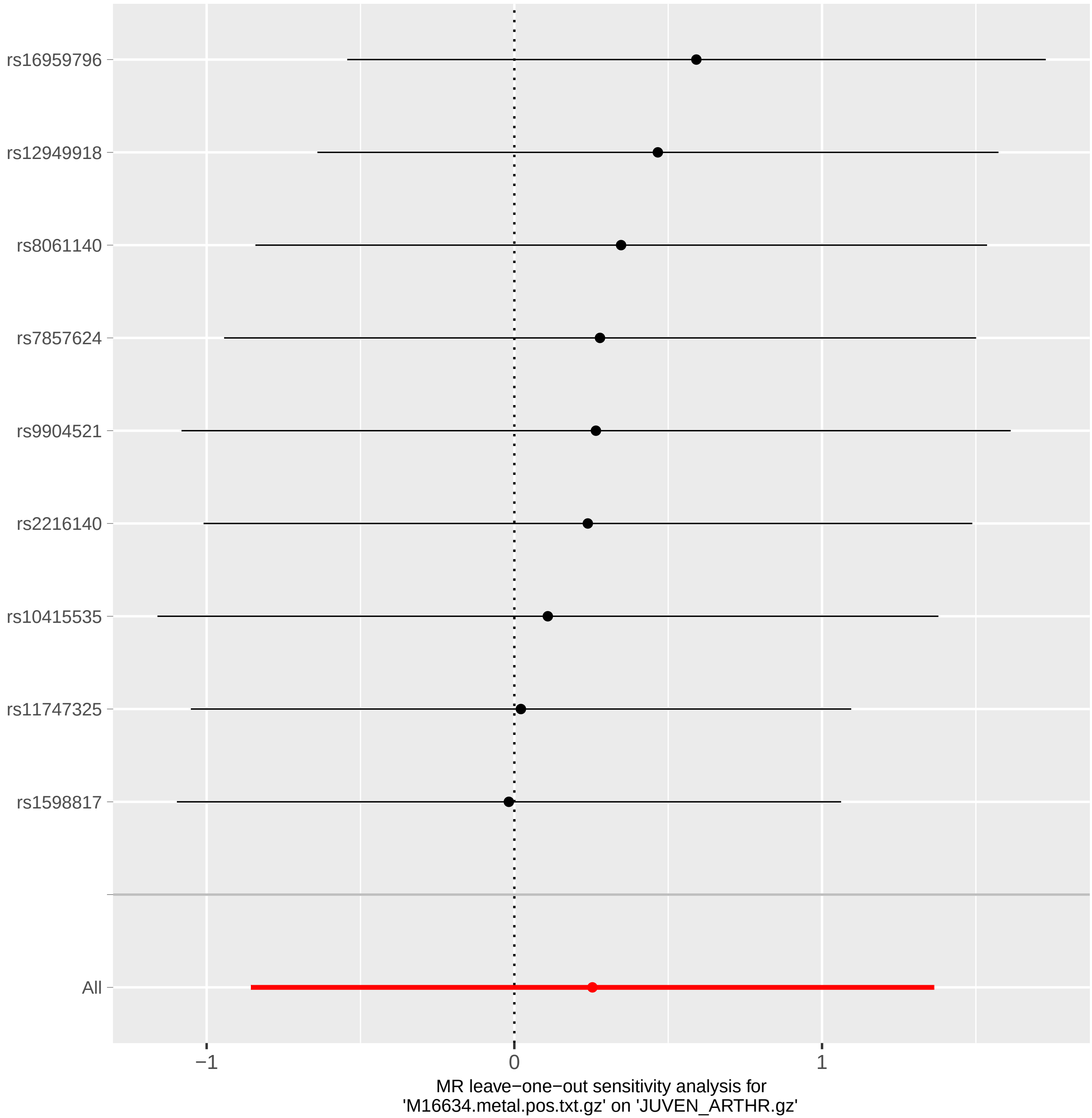
rs16959796
rs12949918
rs8061140
rs7857624
rs9904521
rs2216140
rs10415535
rs11747325
rs1598817
All
−1
0
1
MR leave−one−out sensitivity analysis for
'M16634.metal.pos.txt.gz' on 'JUVEN_ARTHR.gz'

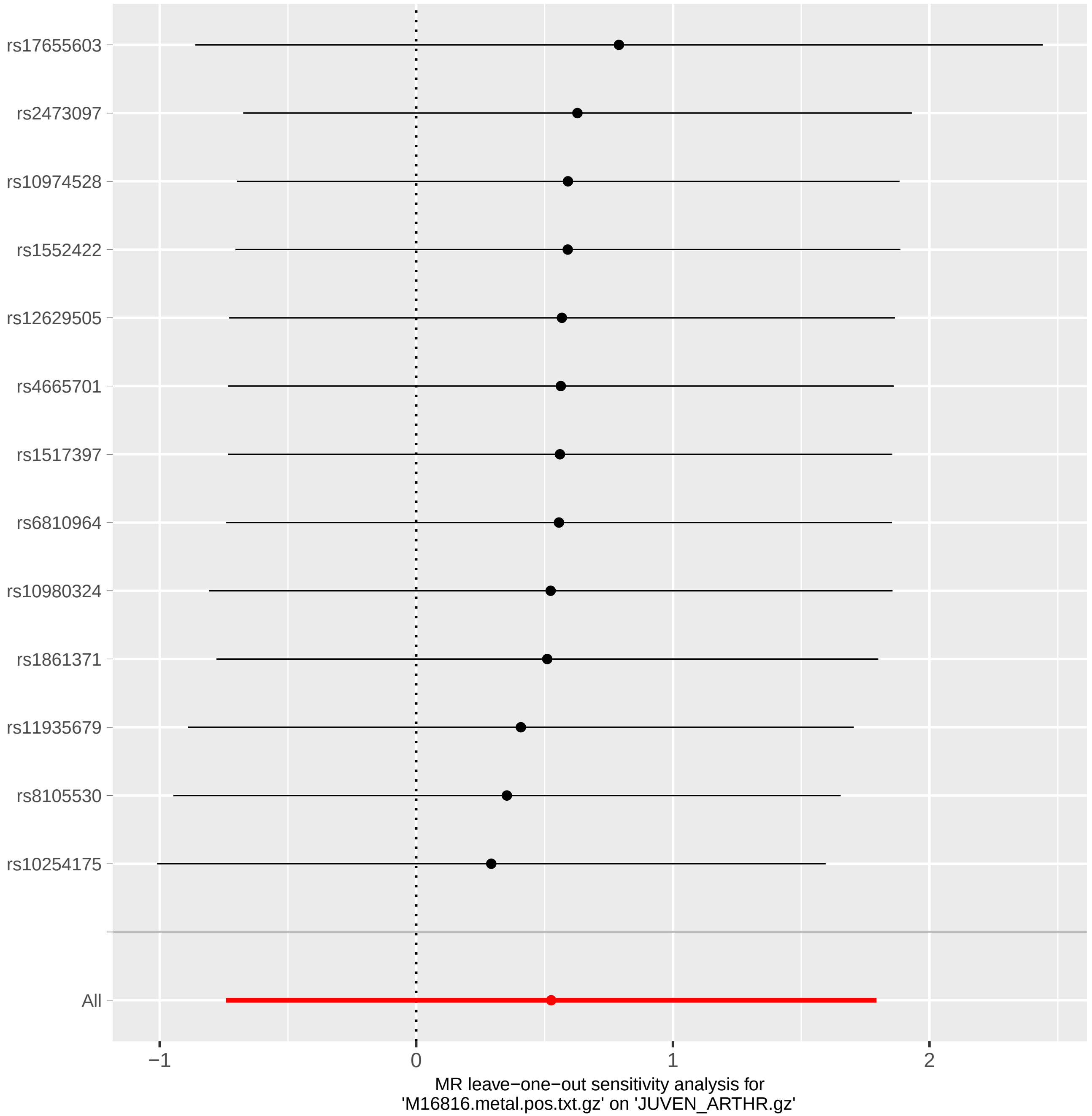
rs17655603
rs2473097
rs10974528
rs1552422
rs12629505
rs4665701
rs1517397
rs6810964
rs10980324
rs1861371
rs11935679
rs8105530
rs10254175
All
−1
0
1
2
MR leave−one−out sensitivity analysis for
'M16816.metal.pos.txt.gz' on 'JUVEN_ARTHR.gz'

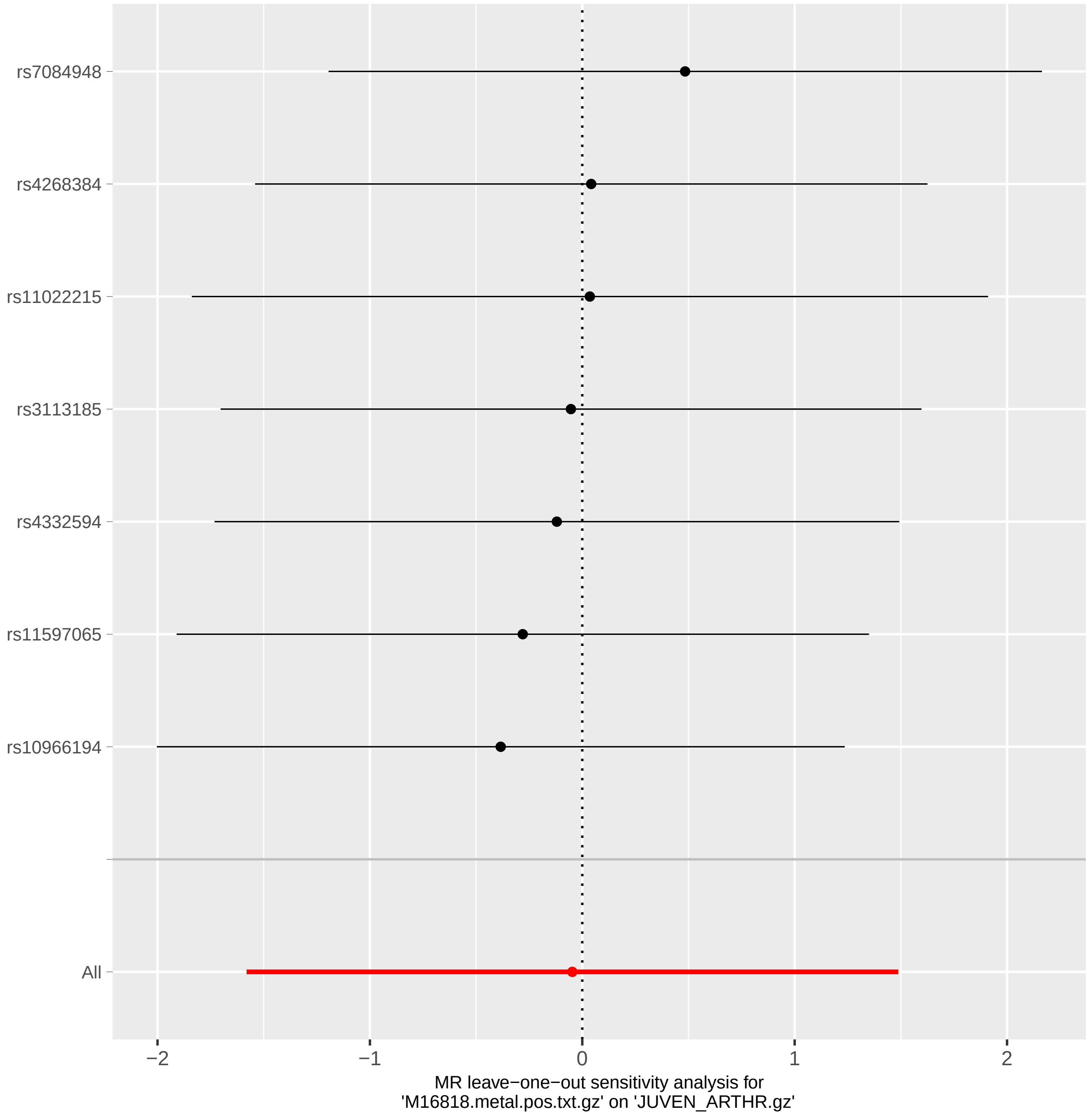

rs7084948
rs4268384
rs11022215
rs3113185
rs4332594
rs11597065
rs10966194
All
−2
−1
0
1
2
MR leave−one−out sensitivity analysis for
'M16818.metal.pos.txt.gz' on 'JUVEN_ARTHR.gz'

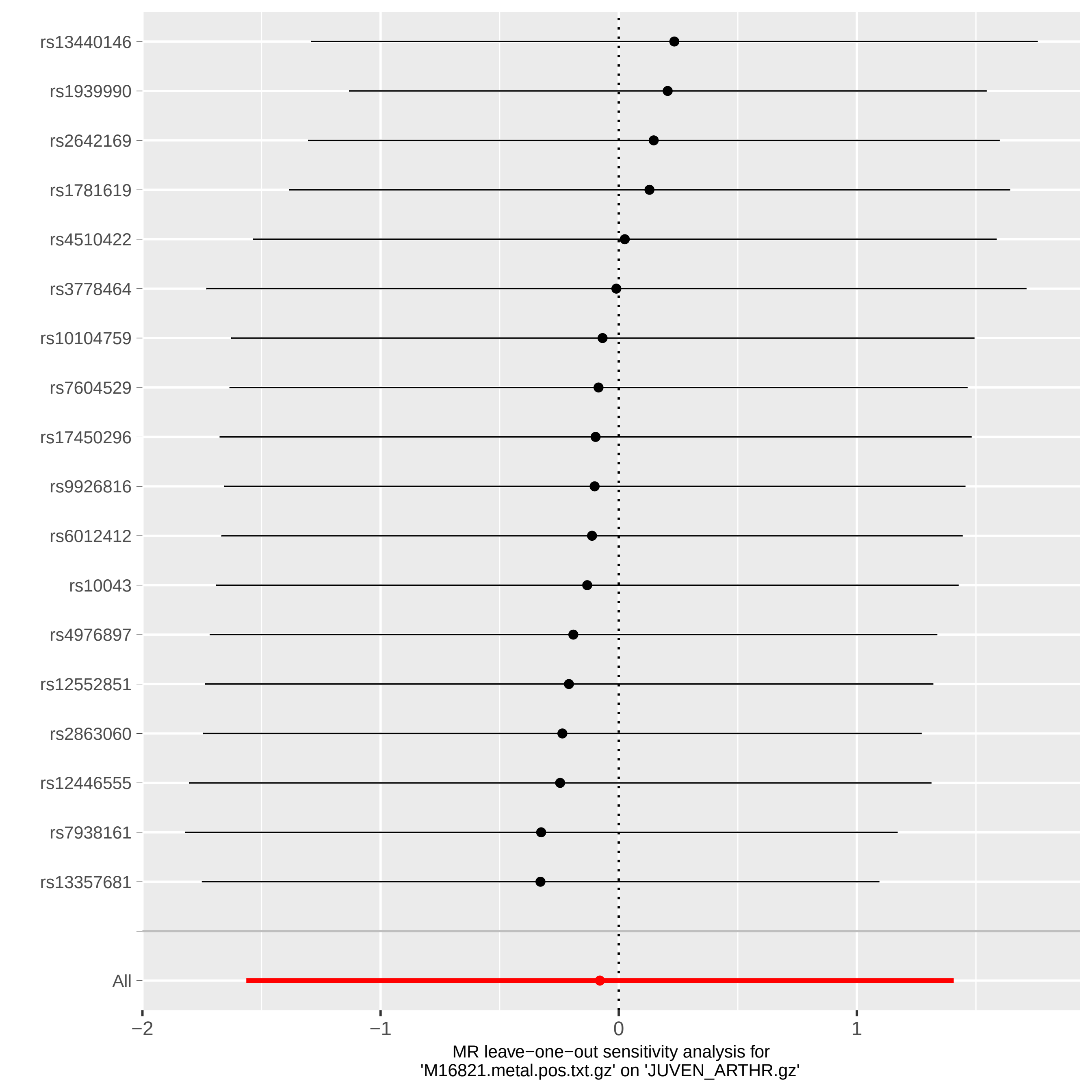
rs13440146
rs1939990
rs2642169
rs1781619
rs4510422
rs3778464
rs10104759
rs7604529
rs17450296
rs9926816
rs6012412
rs10043
rs4976897
rs12552851
rs2863060
rs12446555
rs7938161
rs13357681
All
−2
−1
0
1
MR leave−one−out sensitivity analysis for
'M16821.metal.pos.txt.gz' on 'JUVEN_ARTHR.gz'

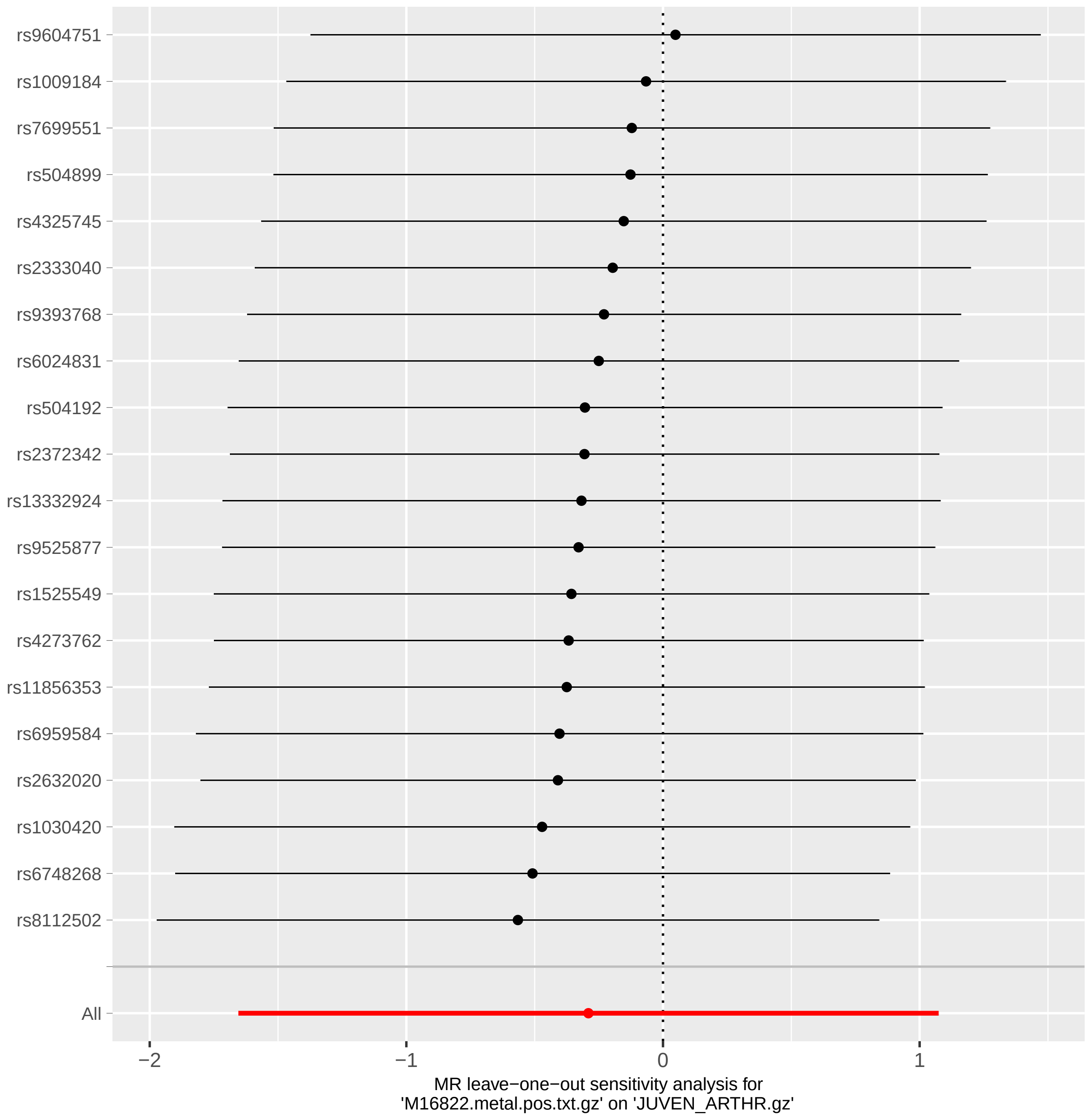

rs9604751
rs1009184
rs7699551
rs504899
rs4325745
rs2333040
rs9393768
rs6024831
rs504192
rs2372342
rs13332924
rs9525877
rs1525549
rs4273762
rs11856353
rs6959584
rs2632020
rs1030420
rs6748268
rs8112502
All
−2
−1
0
1
MR leave−one−out sensitivity analysis for
'M16822.metal.pos.txt.gz' on 'JUVEN_ARTHR.gz'

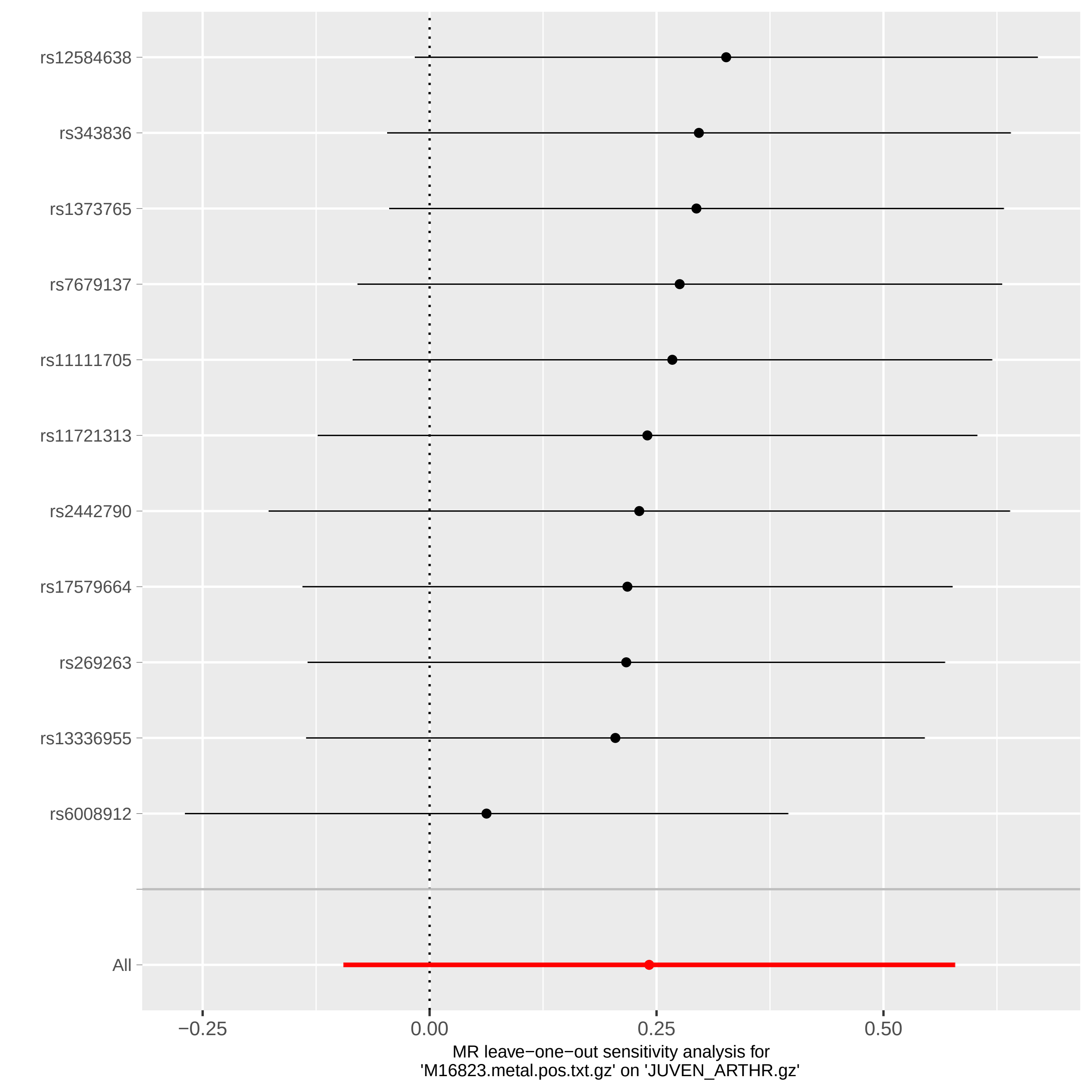
rs12584638
rs343836
rs1373765
rs7679137
rs11111705
rs11721313
rs2442790
rs17579664
rs269263
rs13336955
rs6008912
All
−0.25
0.00
0.25
0.50
MR leave−one−out sensitivity analysis for
'M16823.metal.pos.txt.gz' on 'JUVEN_ARTHR.gz'

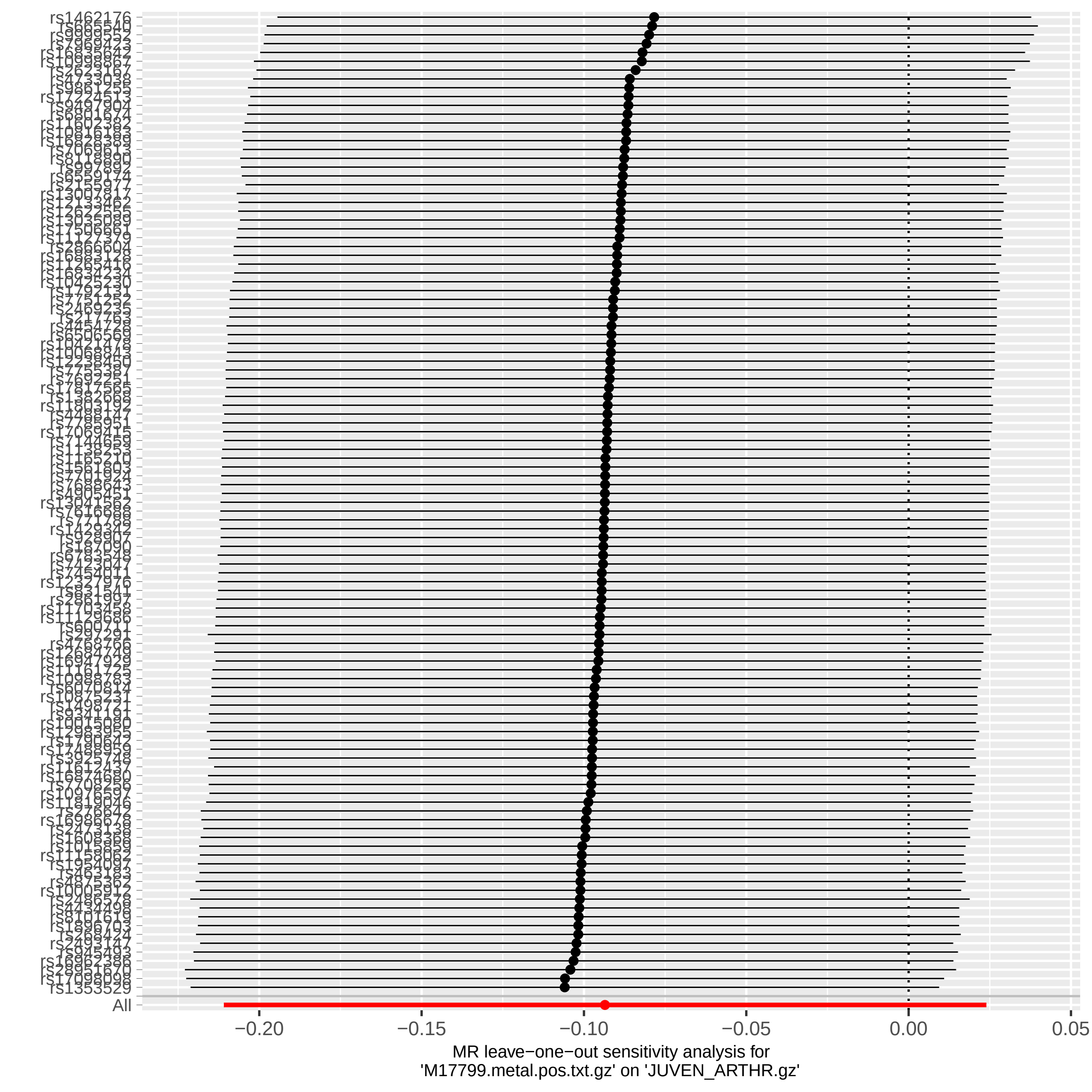
rs1462176
rs1353529
All
−0.20
−0.15
−0.10
−0.05
0.00
0.05
MR leave−one−out sensitivity analysis for
'M17799.metal.pos.txt.gz' on 'JUVEN_ARTHR.gz'

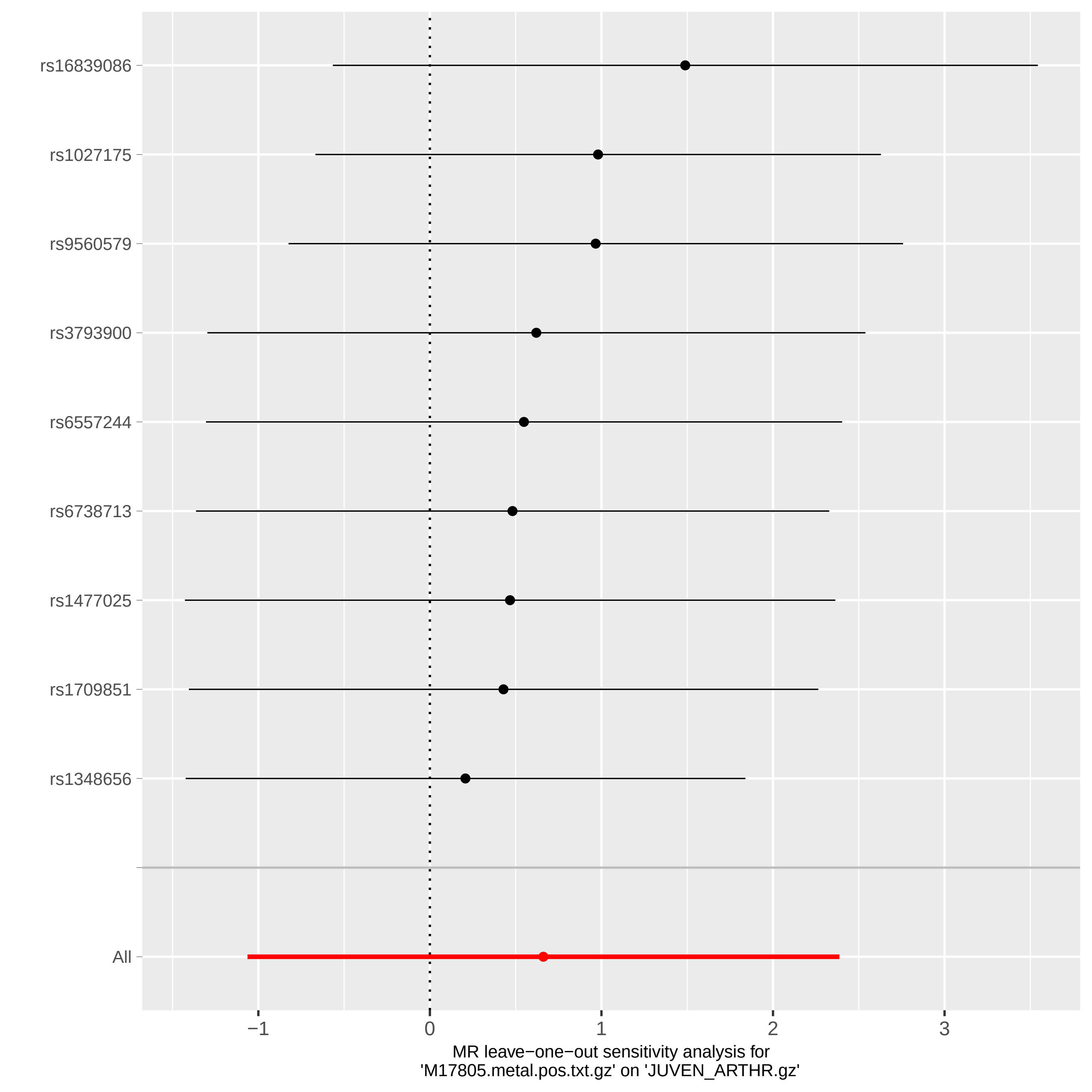
rs16839086
rs1027175
rs9560579
rs3793900
rs6557244
rs6738713
rs1477025
rs1709851
rs1348656
All
−1
0
1
2
3
MR leave−one−out sensitivity analysis for
'M17805.metal.pos.txt.gz' on 'JUVEN_ARTHR.gz'

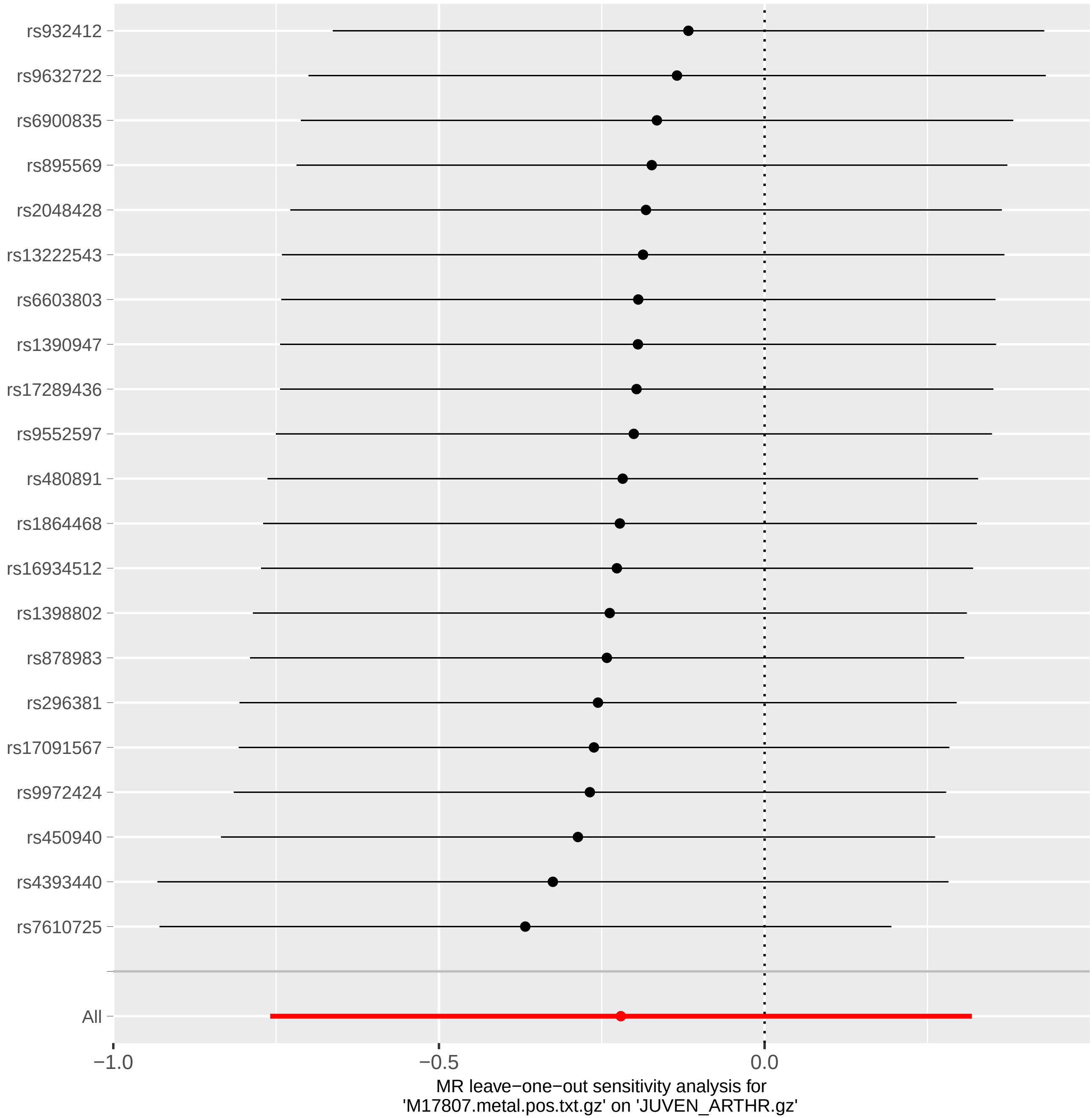
rs932412
rs9632722
rs6900835
rs895569
rs2048428
rs13222543
rs6603803
rs1390947
rs17289436
rs9552597
rs480891
rs1864468
rs16934512
rs1398802
rs878983
rs296381
rs17091567
rs9972424
rs450940
rs4393440
rs7610725
All
−1.0
−0.5
0.0
MR leave−one−out sensitivity analysis for
'M17807.metal.pos.txt.gz' on 'JUVEN_ARTHR.gz'

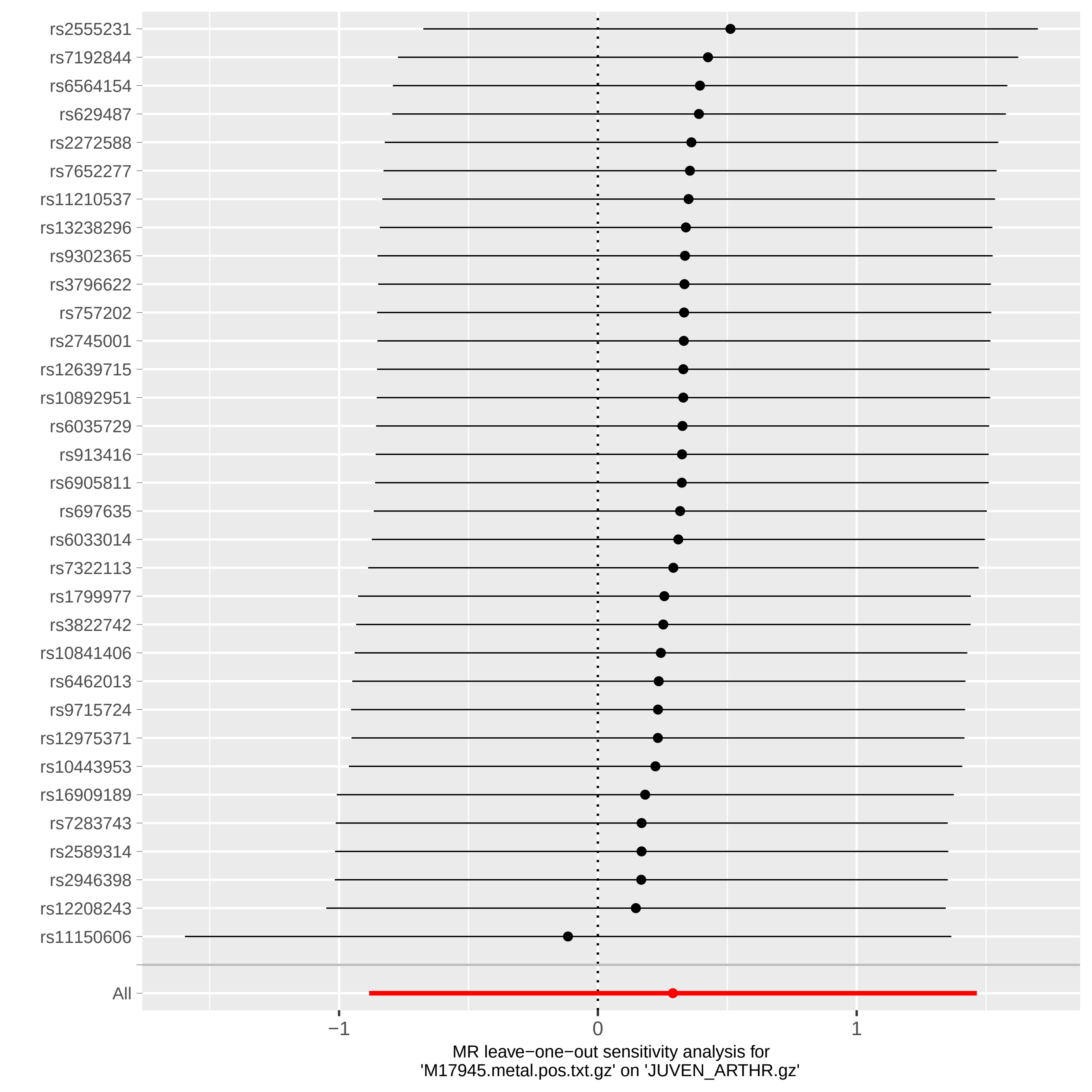

rs2555231
rs7192844
rs6564154
rs629487
rs2272588
rs7652277
rs11210537
rs13238296
rs9302365
rs3796622
rs757202
rs2745001
rs12639715
rs10892951
rs6035729
rs913416
rs6905811
rs697635
rs6033014
rs7322113
rs1799977
rs3822742
rs10841406
rs6462013
rs9715724
rs12975371
rs10443953
rs16909189
rs7283743
rs2589314
rs2946398
rs12208243
rs11150606
All
−1
0
1
MR leave−one−out sensitivity analysis for
'M17945.metal.pos.txt.gz' on 'JUVEN_ARTHR.gz'

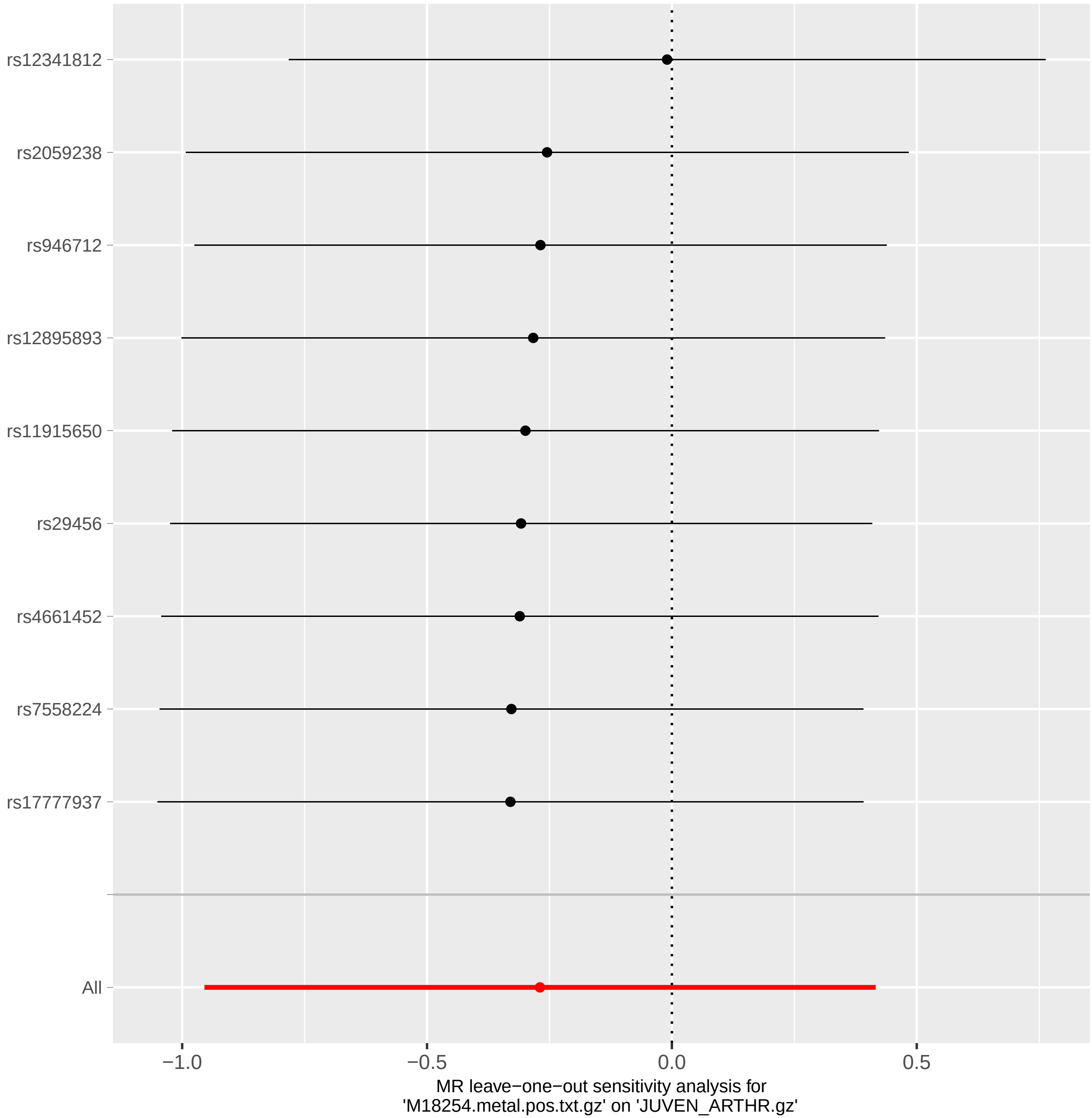
rs12341812
rs2059238
rs946712
rs12895893
rs11915650
rs29456
rs4661452
rs7558224
rs17777937
All
−1.0
−0.5
0.0
0.5
MR leave−one−out sensitivity analysis for
'M18254.metal.pos.txt.gz' on 'JUVEN_ARTHR.gz'

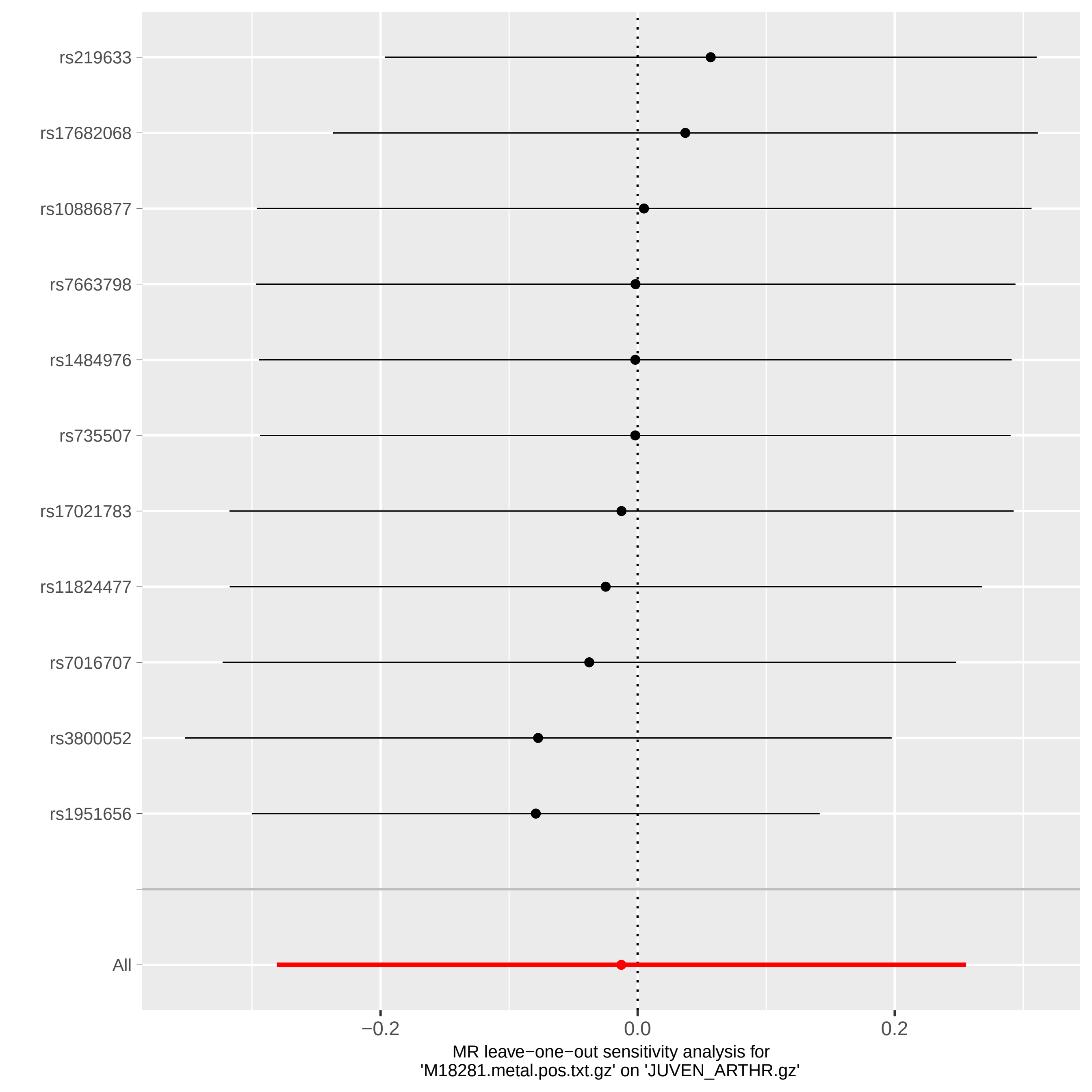
rs219633
rs17682068
rs10886877
rs7663798
rs1484976
rs735507
rs17021783
rs11824477
rs7016707
rs3800052
rs1951656
All
−0.2
0.0
0.2
MR leave−one−out sensitivity analysis for
'M18281.metal.pos.txt.gz' on 'JUVEN_ARTHR.gz'

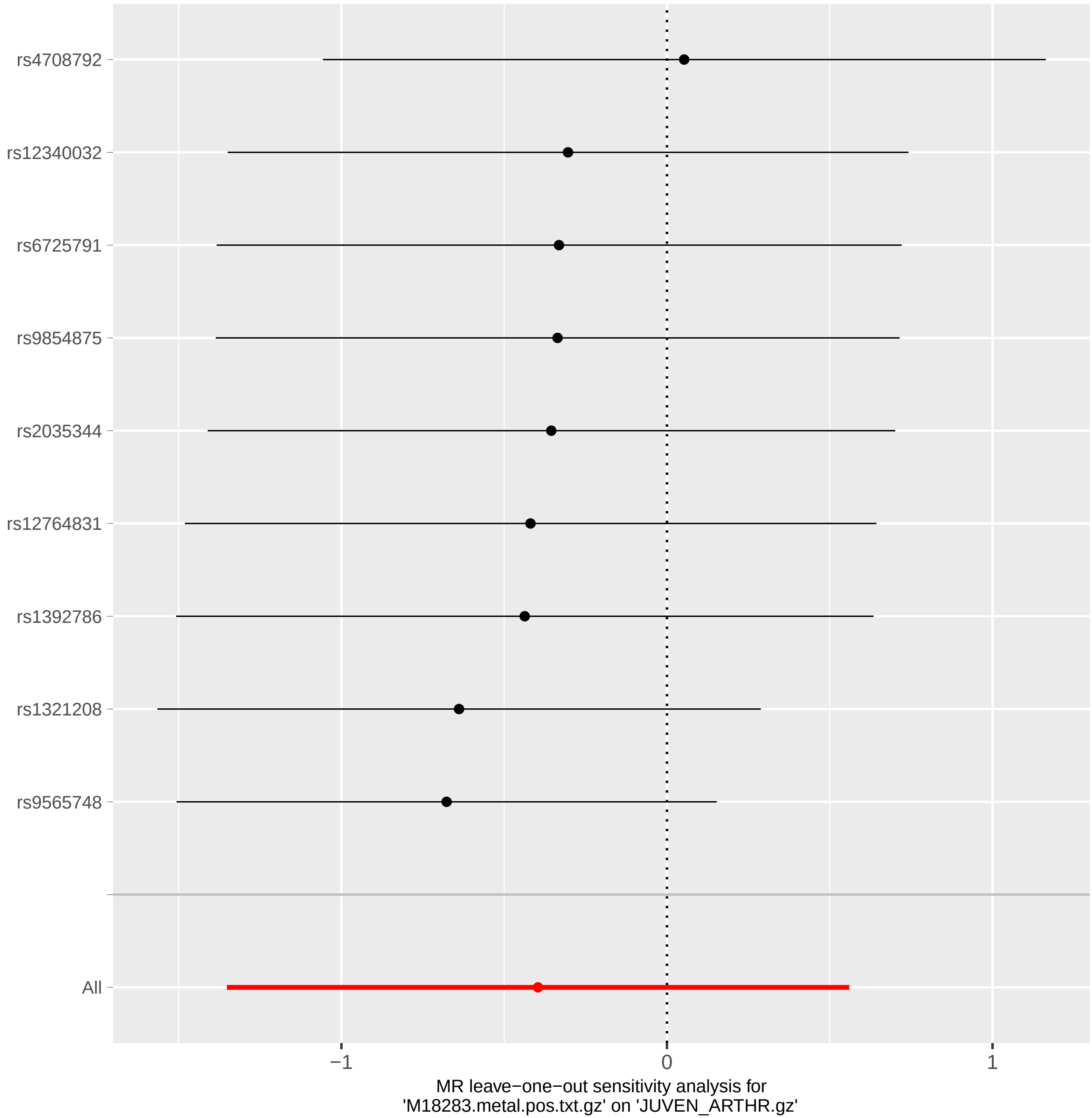
rs4708792
rs12340032
rs6725791
rs9854875
rs2035344
rs12764831
rs1392786
rs1321208
rs9565748
All
−1
0
1
MR leave−one−out sensitivity analysis for
'M18283.metal.pos.txt.gz' on 'JUVEN_ARTHR.gz'

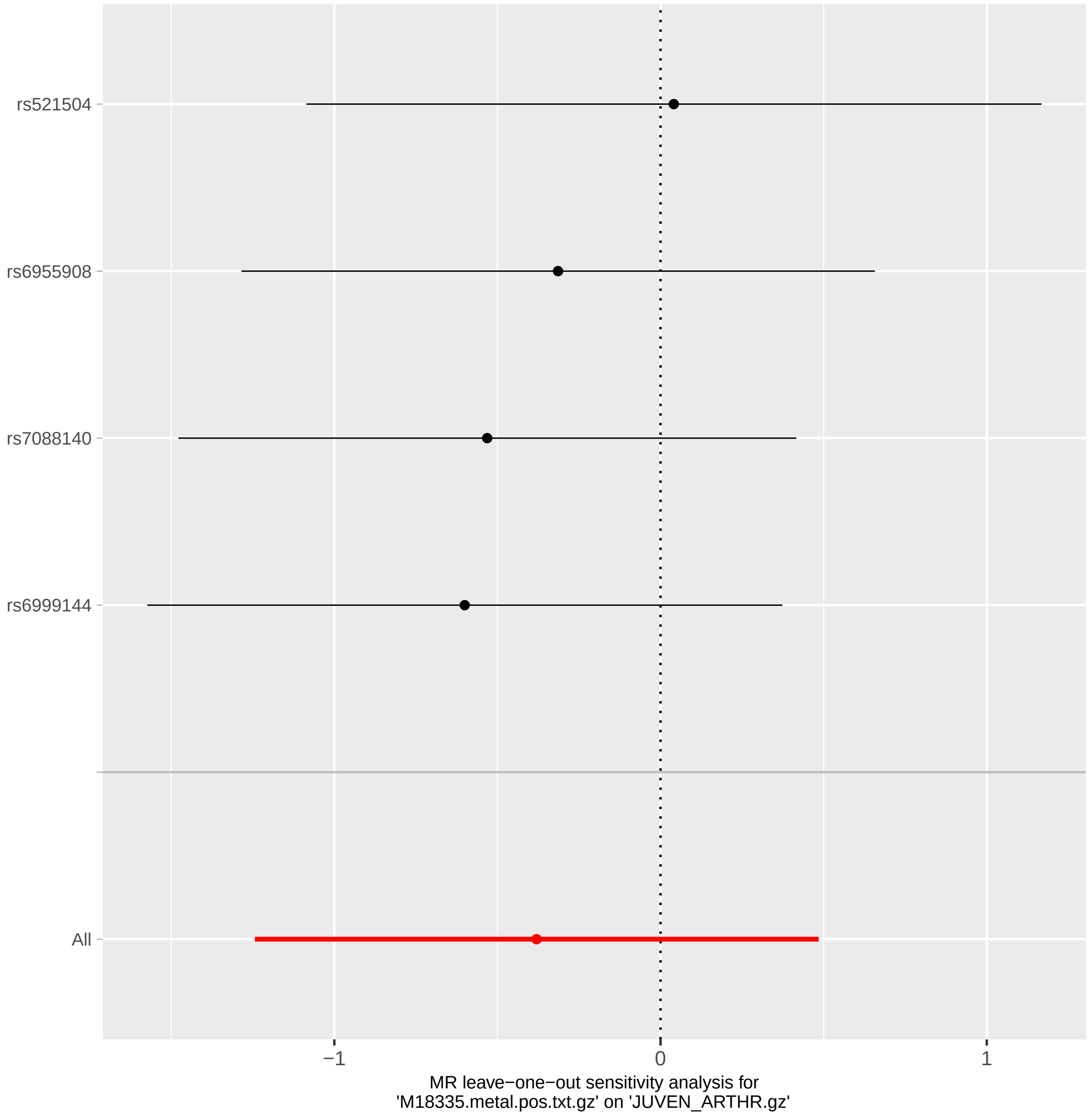

rs521504
rs6955908
rs7088140
rs6999144
All
−1
0
1
MR leave−one−out sensitivity analysis for
'M18335.metal.pos.txt.gz' on 'JUVEN_ARTHR.gz'

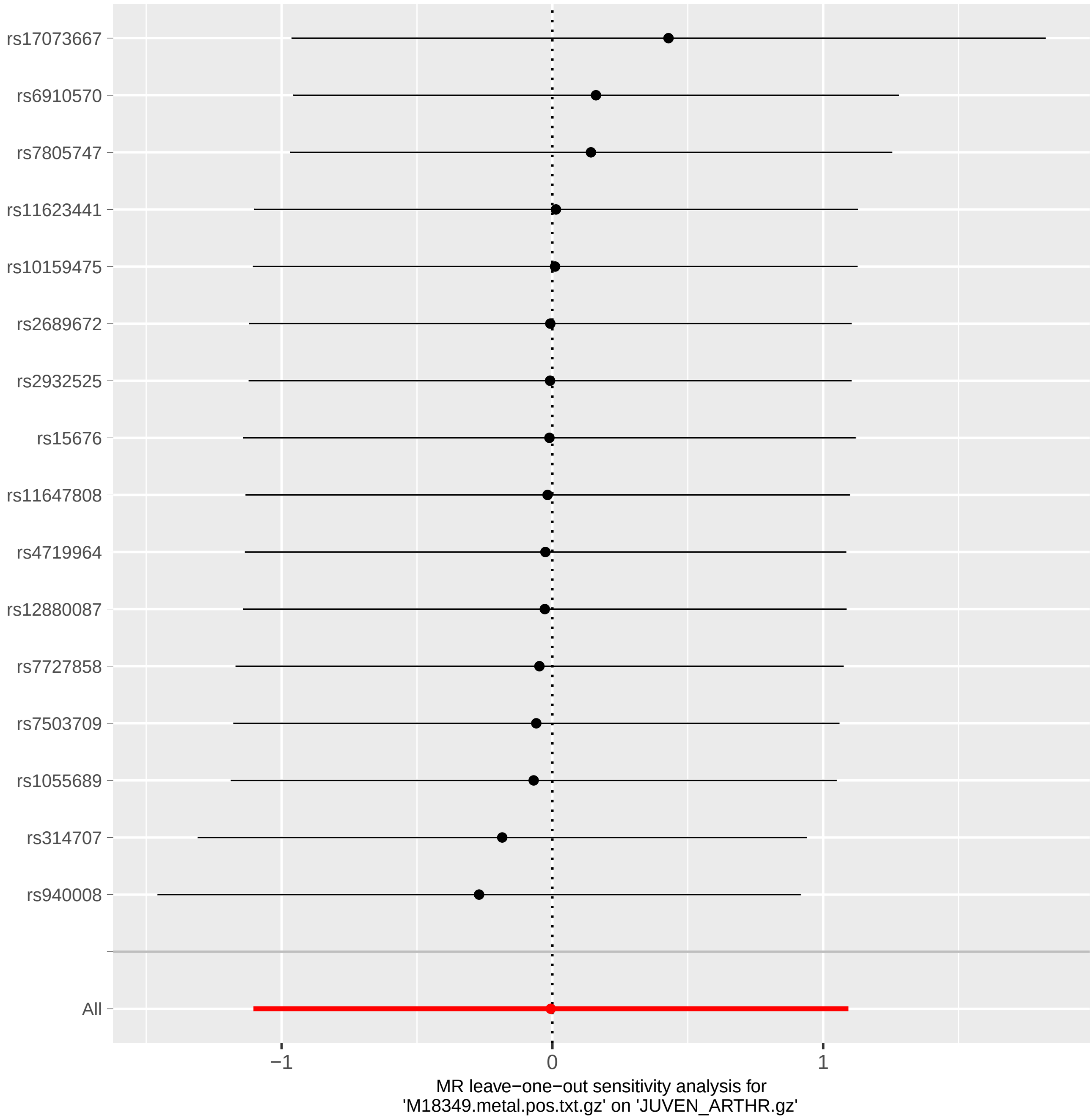

rs17073667
rs6910570
rs7805747
rs11623441
rs10159475
rs2689672
rs2932525
rs15676
rs11647808
rs4719964
rs12880087
rs7727858
rs7503709
rs1055689
rs314707
rs940008
All
−1
0
1
MR leave−one−out sensitivity analysis for
'M18349.metal.pos.txt.gz' on 'JUVEN_ARTHR.gz'

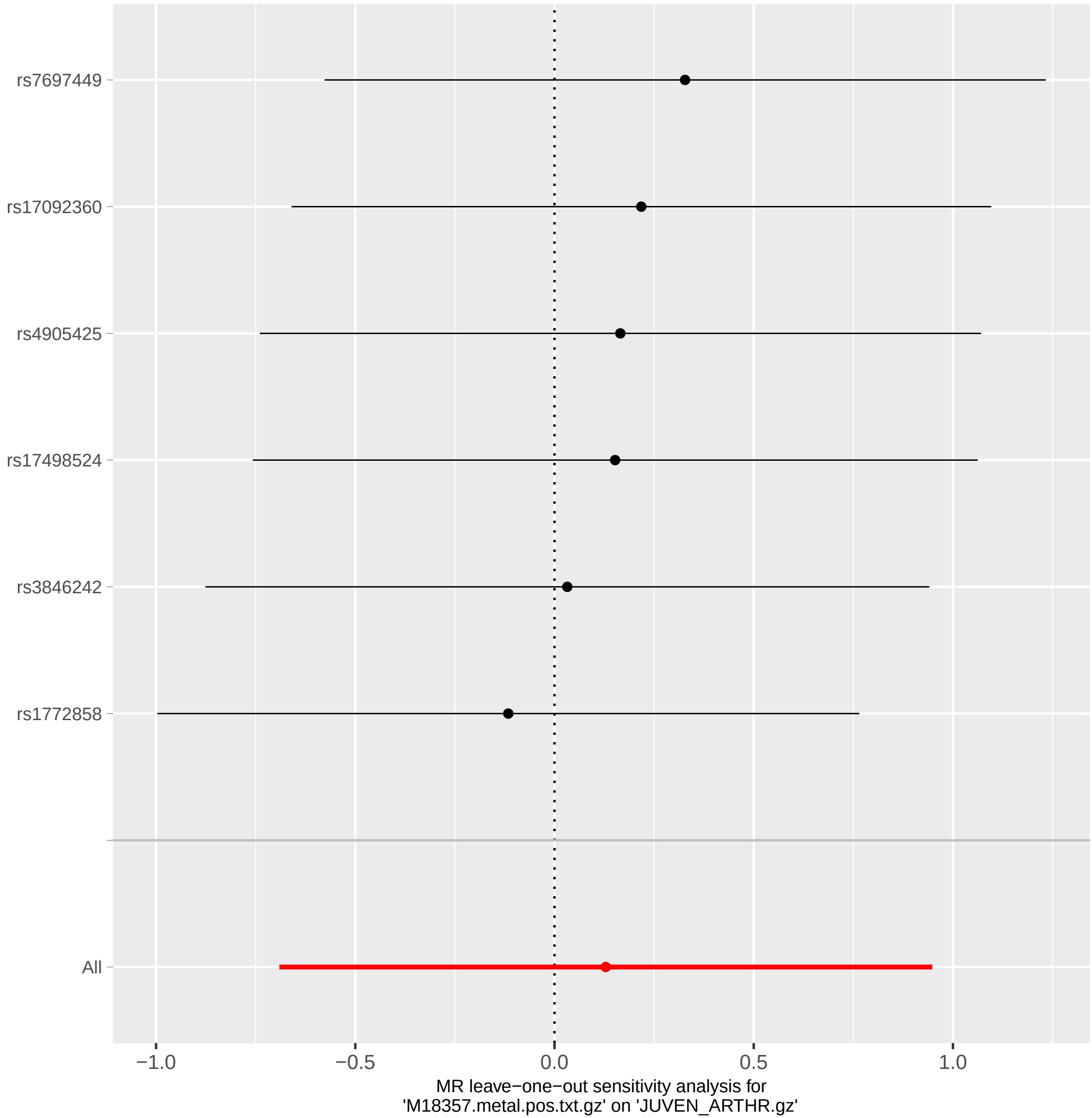

rs7697449
rs17092360
rs4905425
rs17498524
rs3846242
rs1772858
All
−1.0
−0.5
0.0
0.5
1.0
MR leave−one−out sensitivity analysis for
'M18357.metal.pos.txt.gz' on 'JUVEN_ARTHR.gz'

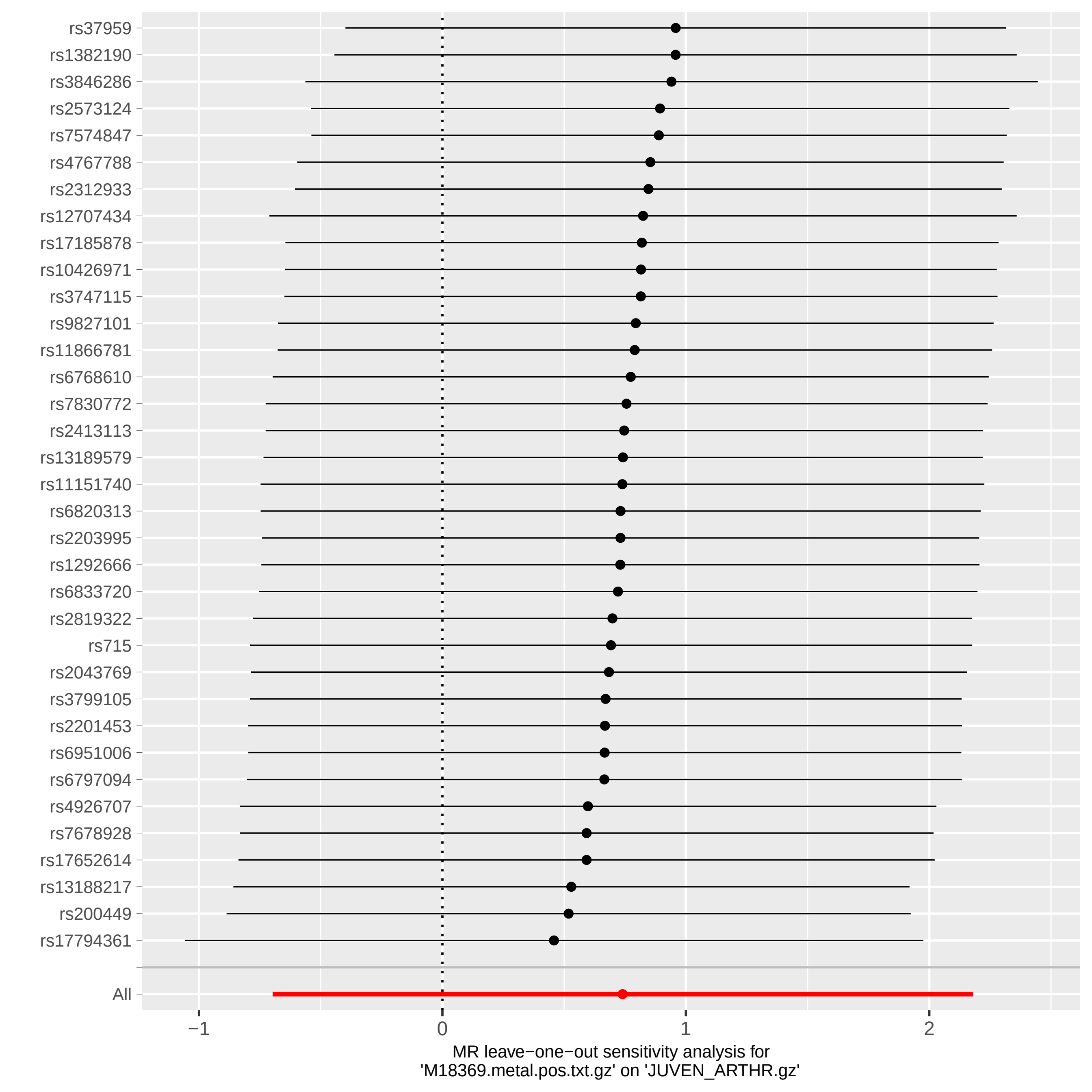
rs37959
rs1382190
rs3846286
rs2573124
rs7574847
rs4767788
rs2312933
rs12707434
rs17185878
rs10426971
rs3747115
rs9827101
rs11866781
rs6768610
rs7830772
rs2413113
rs13189579
rs11151740
rs6820313
rs2203995
rs1292666
rs6833720
rs2819322
rs715
rs2043769
rs3799105
rs2201453
rs6951006
rs6797094
rs4926707
rs7678928
rs17652614
rs13188217
rs200449
rs17794361
All
−1
0
1
2
MR leave−one−out sensitivity analysis for
'M18369.metal.pos.txt.gz' on 'JUVEN_ARTHR.gz'

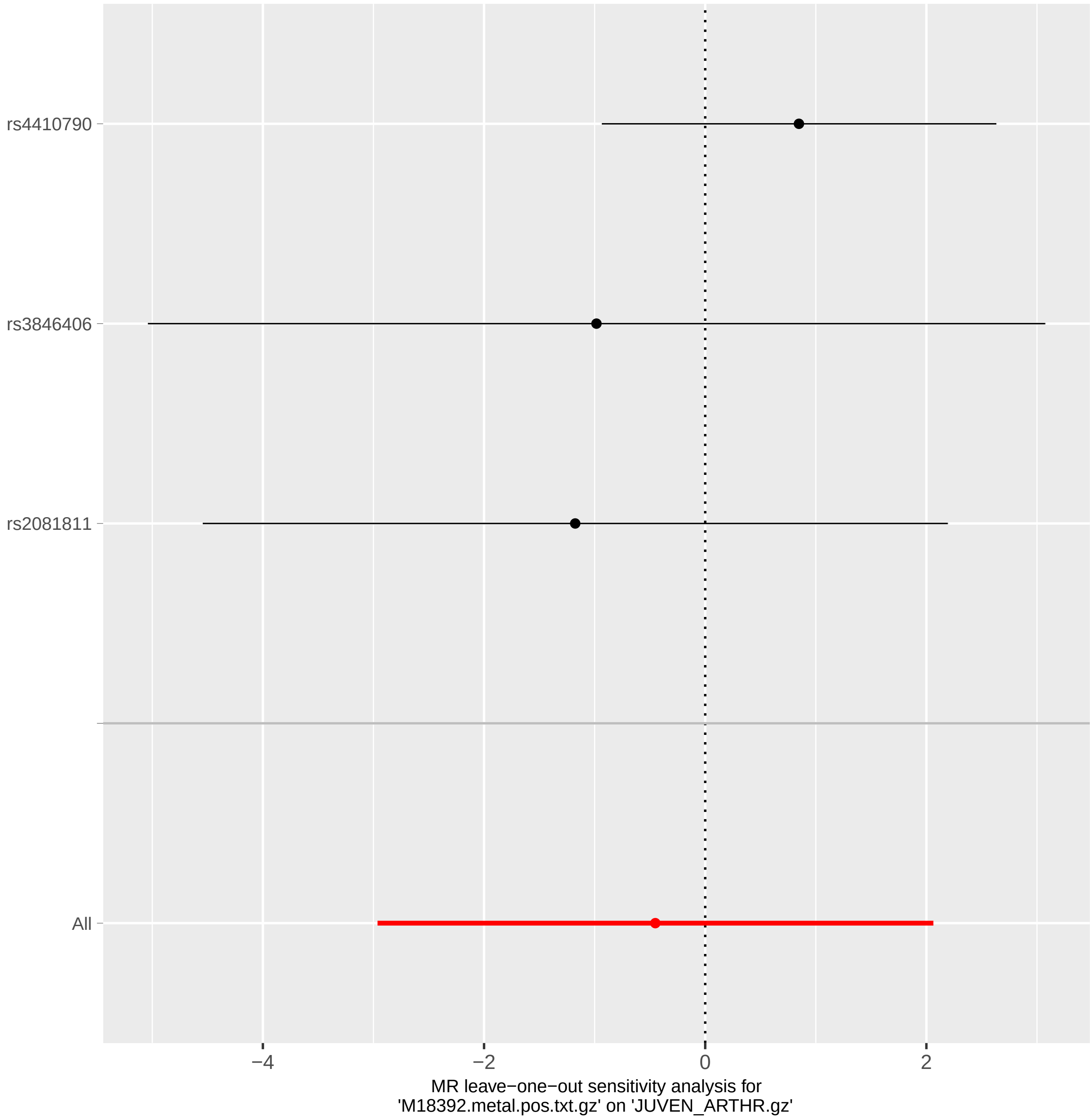
rs4410790
rs3846406
rs2081811
All
−4
−2
0
2
MR leave−one−out sensitivity analysis for
'M18392.metal.pos.txt.gz' on 'JUVEN_ARTHR.gz'

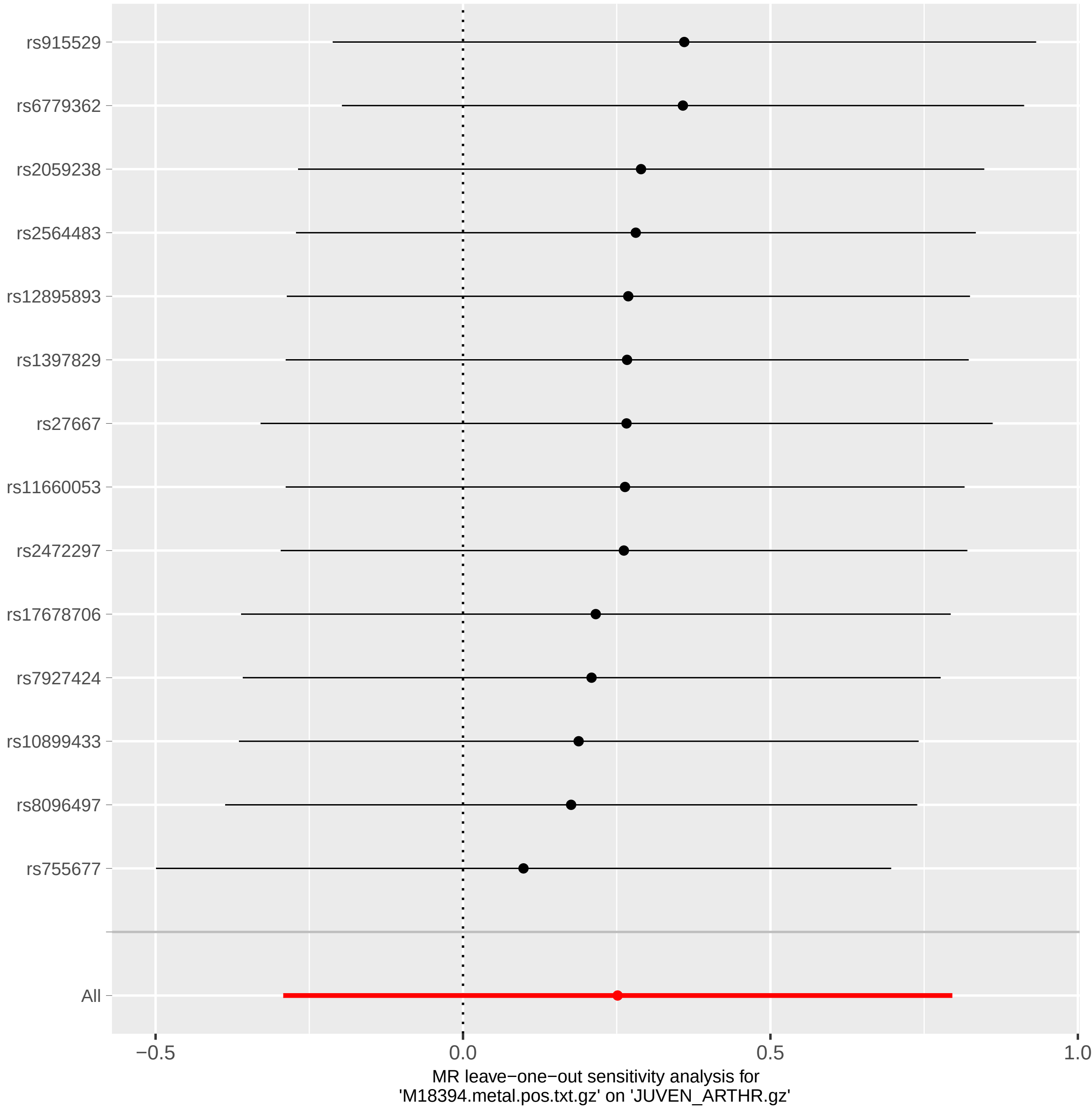

rs915529
rs6779362
rs2059238
rs2564483
rs12895893
rs1397829
rs27667
rs11660053
rs2472297
rs17678706
rs7927424
rs10899433
rs8096497
rs755677
All
−0.5
0.0
0.5
1.0
MR leave−one−out sensitivity analysis for
'M18394.metal.pos.txt.gz' on 'JUVEN_ARTHR.gz'

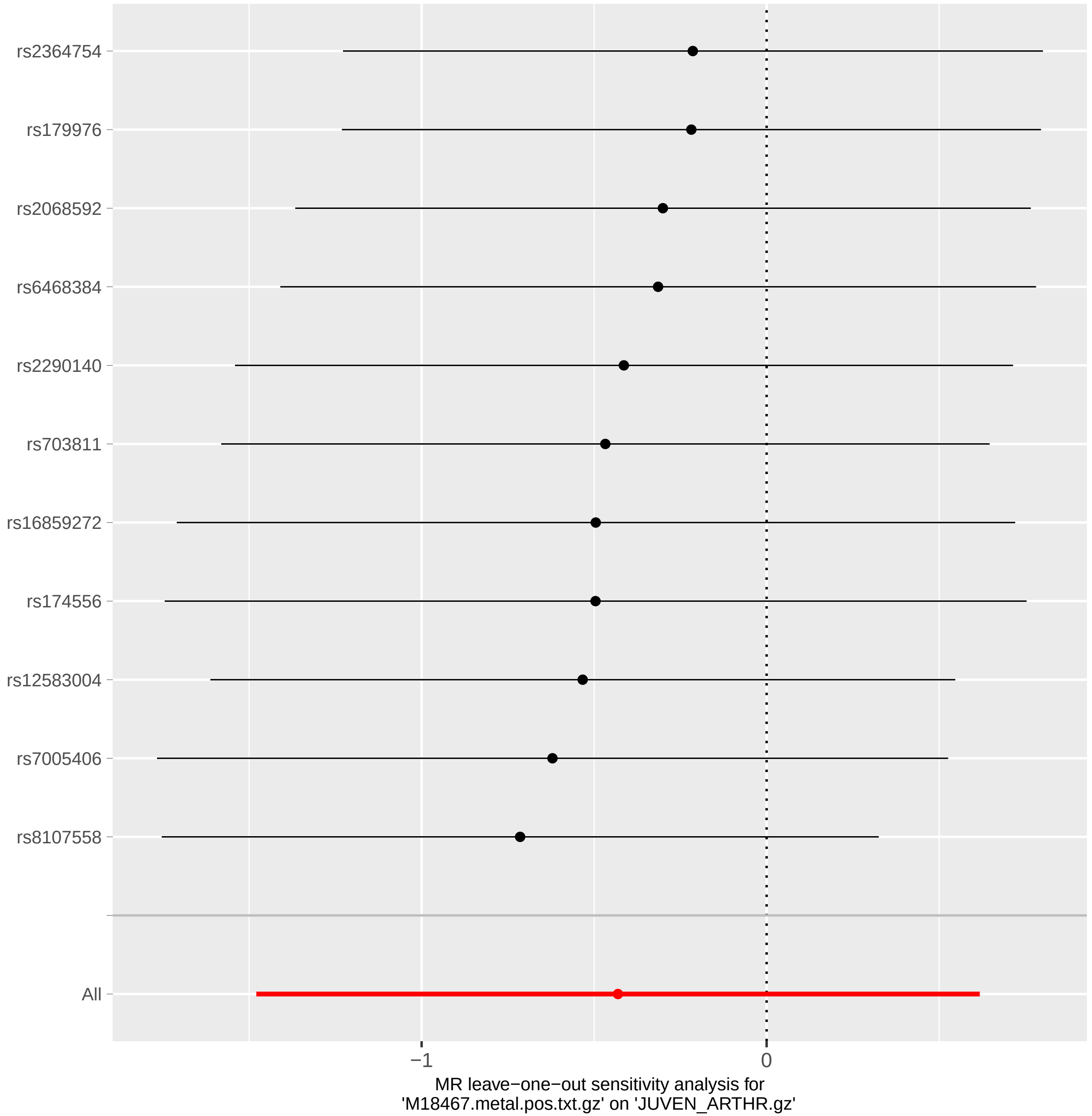
rs2364754
rs179976
rs2068592
rs6468384
rs2290140
rs703811
rs16859272
rs174556
rs12583004
rs7005406
rs8107558
All
−1
0
MR leave−one−out sensitivity analysis for
'M18467.metal.pos.txt.gz' on 'JUVEN_ARTHR.gz'

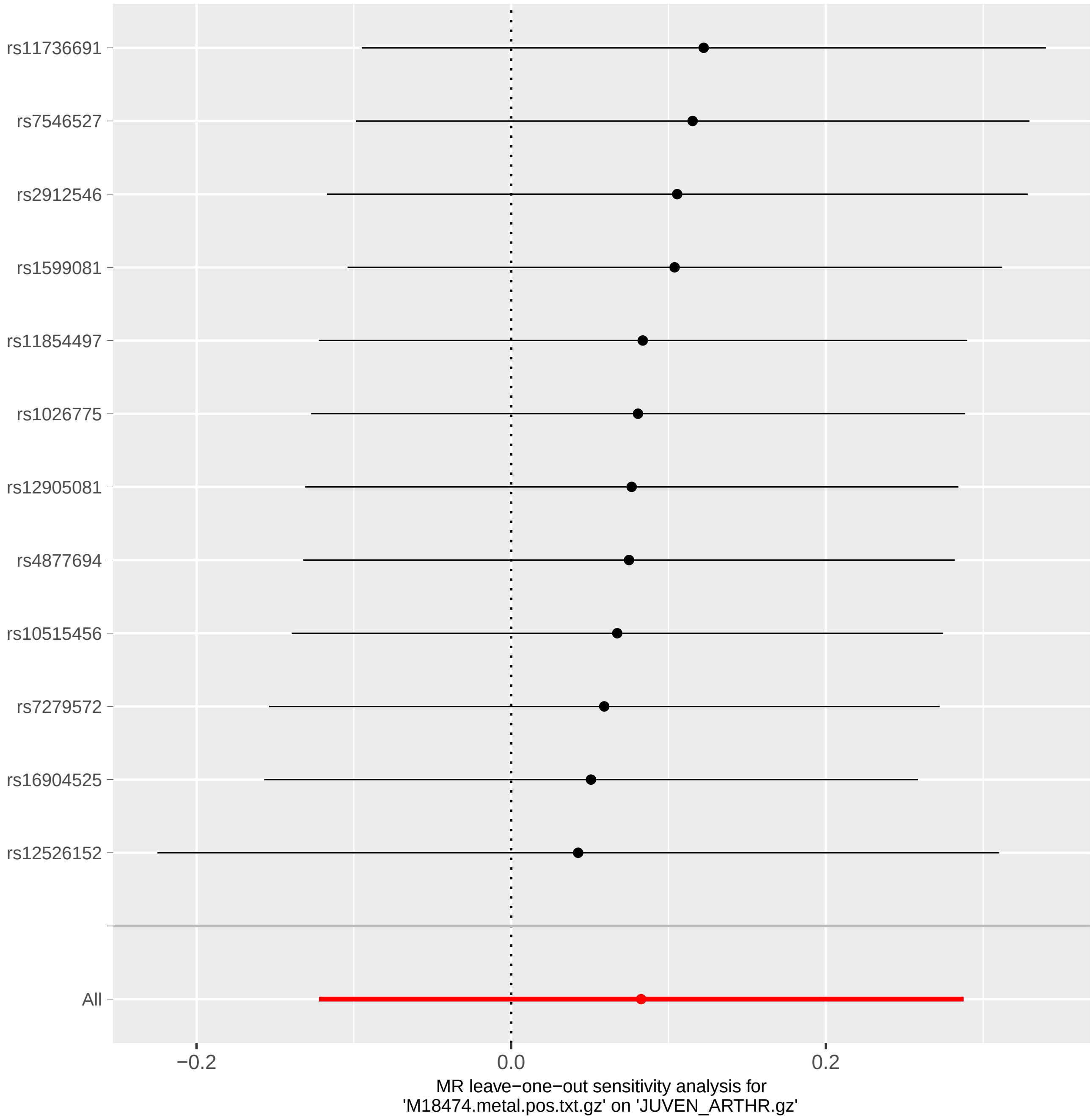
rs11736691
rs7546527
rs2912546
rs1599081
rs11854497
rs1026775
rs12905081
rs4877694
rs10515456
rs7279572
rs16904525
rs12526152
All
−0.2
0.0
0.2
MR leave−one−out sensitivity analysis for
'M18474.metal.pos.txt.gz' on 'JUVEN_ARTHR.gz'

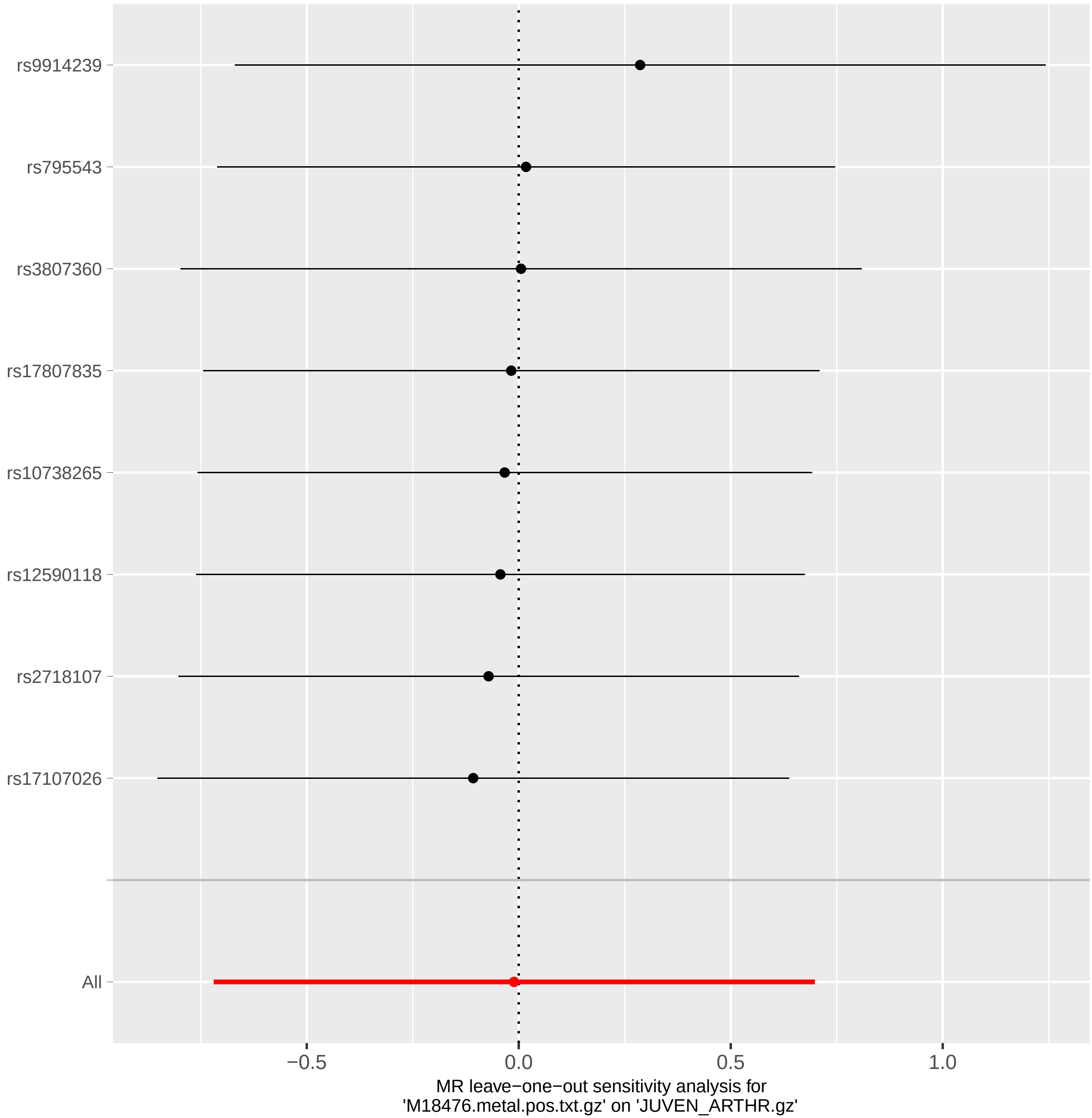
rs9914239
rs795543
rs3807360
rs17807835
rs10738265
rs12590118
rs2718107
rs17107026
All
−0.5
0.0
0.5
1.0
MR leave−one−out sensitivity analysis for
'M18476.metal.pos.txt.gz' on 'JUVEN_ARTHR.gz'

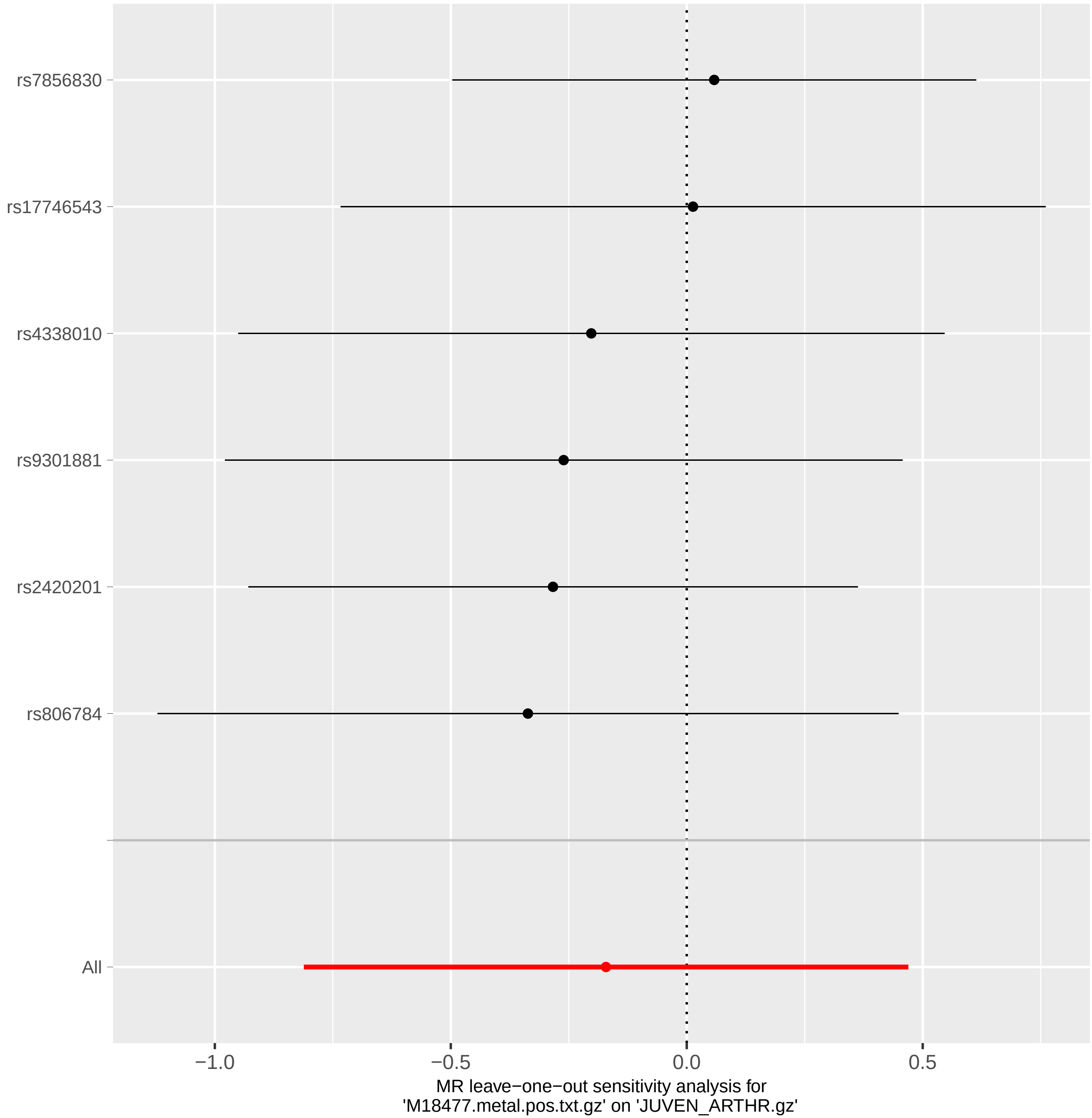
rs7856830
rs17746543
rs4338010
rs9301881
rs2420201
rs806784
All
−1.0
−0.5
0.0
0.5
MR leave−one−out sensitivity analysis for
'M18477.metal.pos.txt.gz' on 'JUVEN_ARTHR.gz'

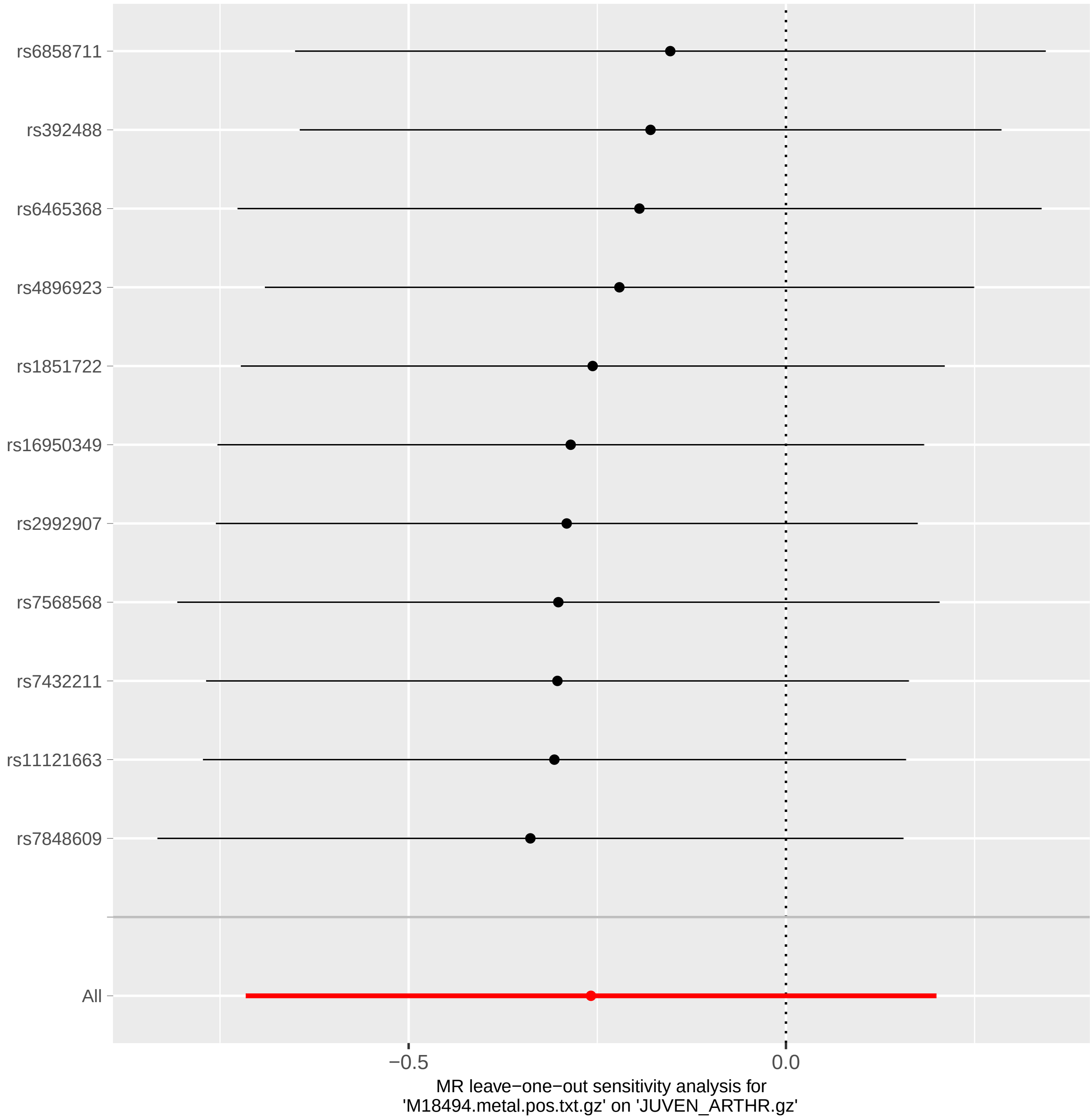
rs6858711
rs392488
rs6465368
rs4896923
rs1851722
rs16950349
rs2992907
rs7568568
rs7432211
rs11121663
rs7848609
All
−0.5
0.0
MR leave−one−out sensitivity analysis for
'M18494.metal.pos.txt.gz' on 'JUVEN_ARTHR.gz'

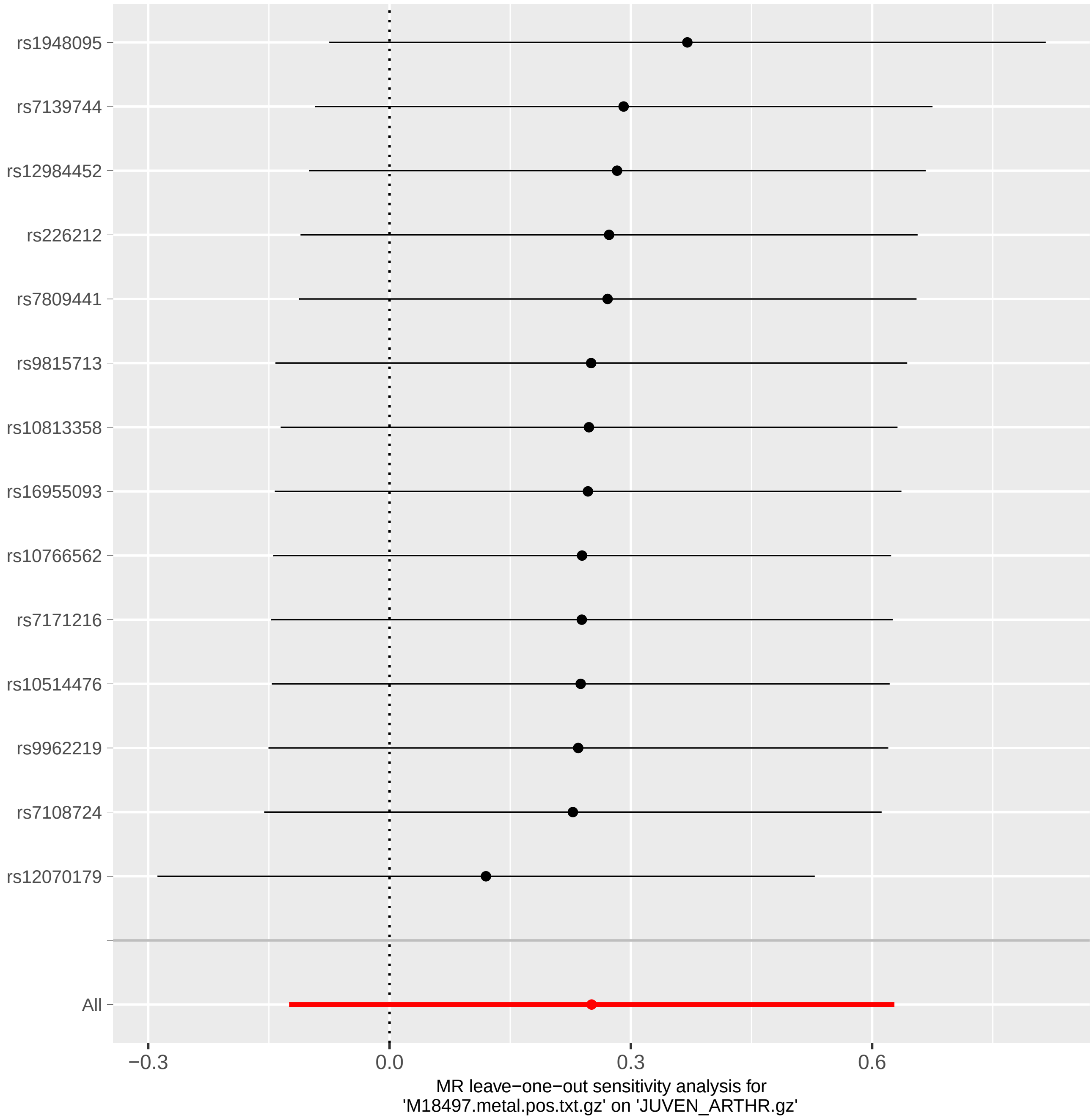
rs1948095
rs7139744
rs12984452
rs226212
rs7809441
rs9815713
rs10813358
rs16955093
rs10766562
rs7171216
rs10514476
rs9962219
rs7108724
rs12070179
All
−0.3
0.0
0.3
0.6
MR leave−one−out sensitivity analysis for
'M18497.metal.pos.txt.gz' on 'JUVEN_ARTHR.gz'

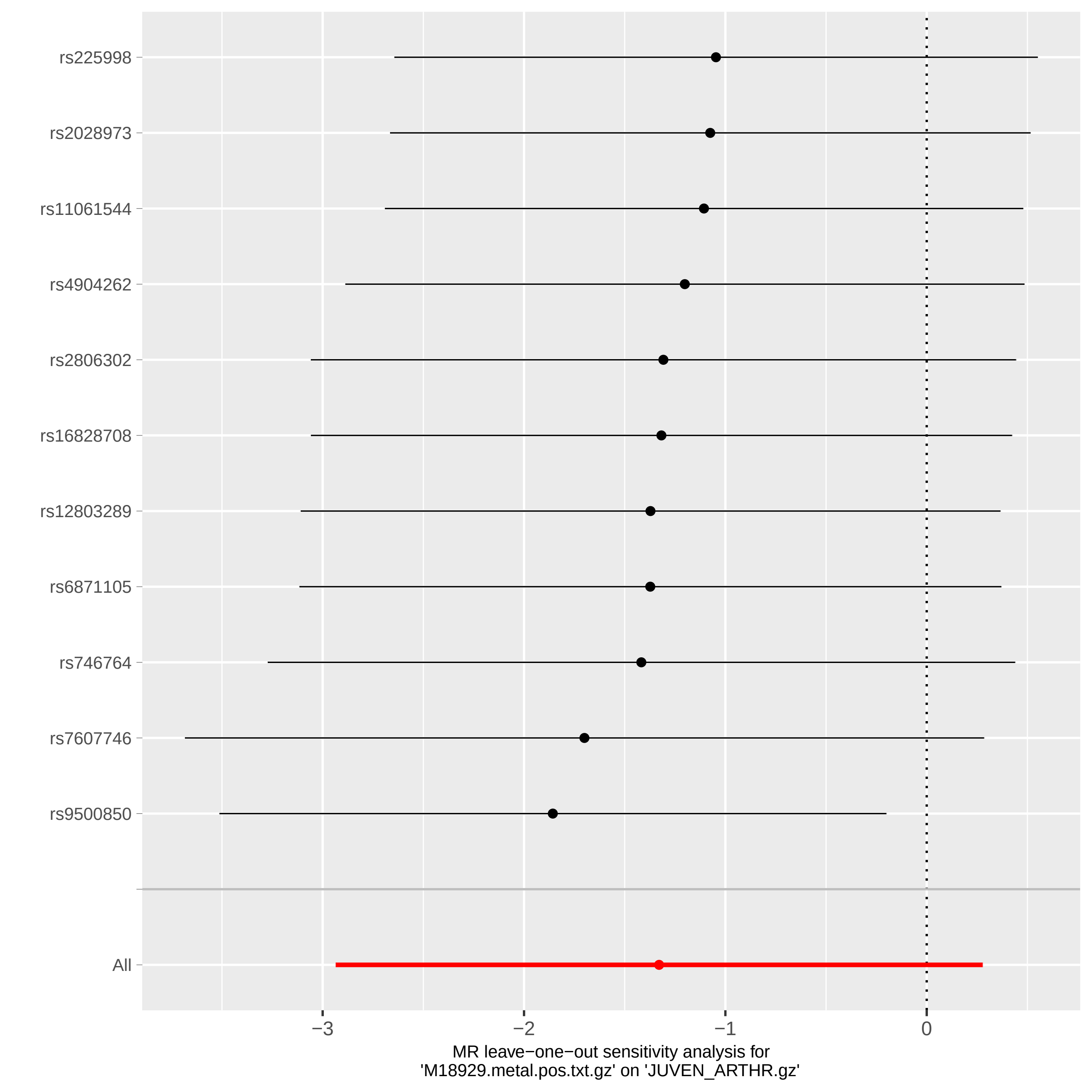
rs225998
rs2028973
rs11061544
rs4904262
rs2806302
rs16828708
rs12803289
rs6871105
rs746764
rs7607746
rs9500850
All
−3
−2
−1
0
MR leave−one−out sensitivity analysis for
'M18929.metal.pos.txt.gz' on 'JUVEN_ARTHR.gz'

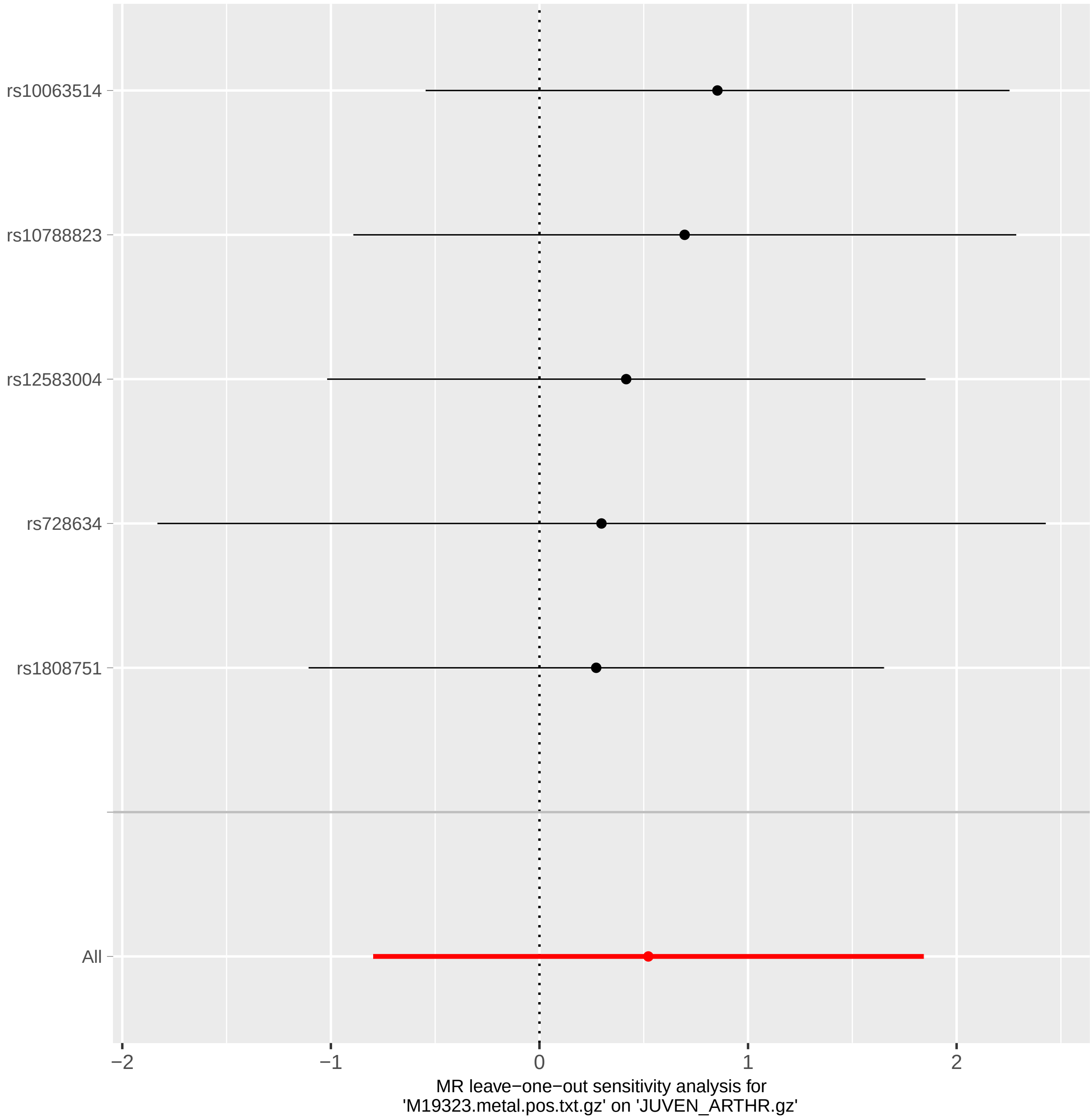
rs10063514
rs10788823
rs12583004
rs728634
rs1808751
All
−2
−1
0
1
2
MR leave−one−out sensitivity analysis for
'M19323.metal.pos.txt.gz' on 'JUVEN_ARTHR.gz'

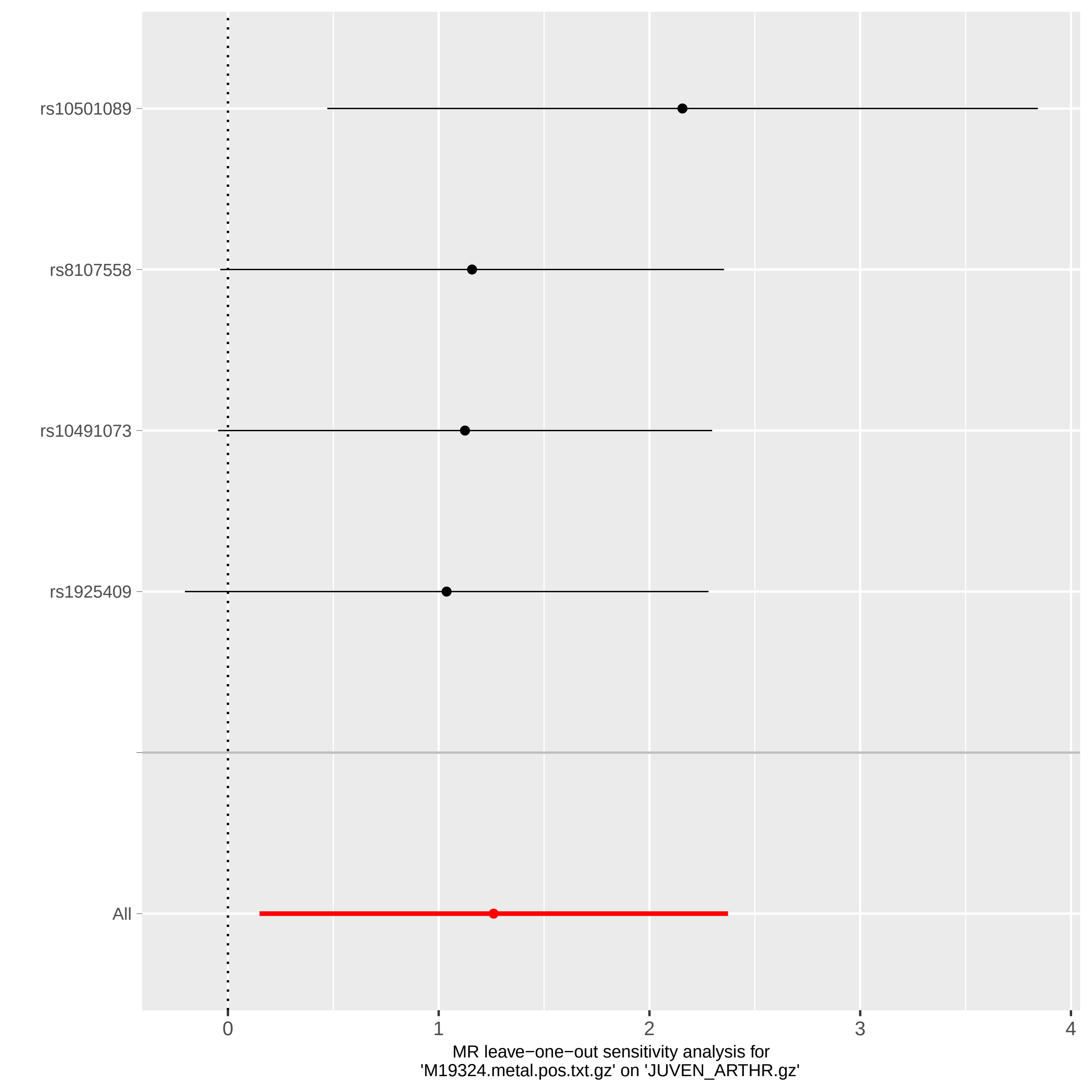
rs10501089
rs8107558
rs10491073
rs1925409
All
0
1
2
3
4
MR leave−one−out sensitivity analysis for
'M19324.metal.pos.txt.gz' on 'JUVEN_ARTHR.gz'

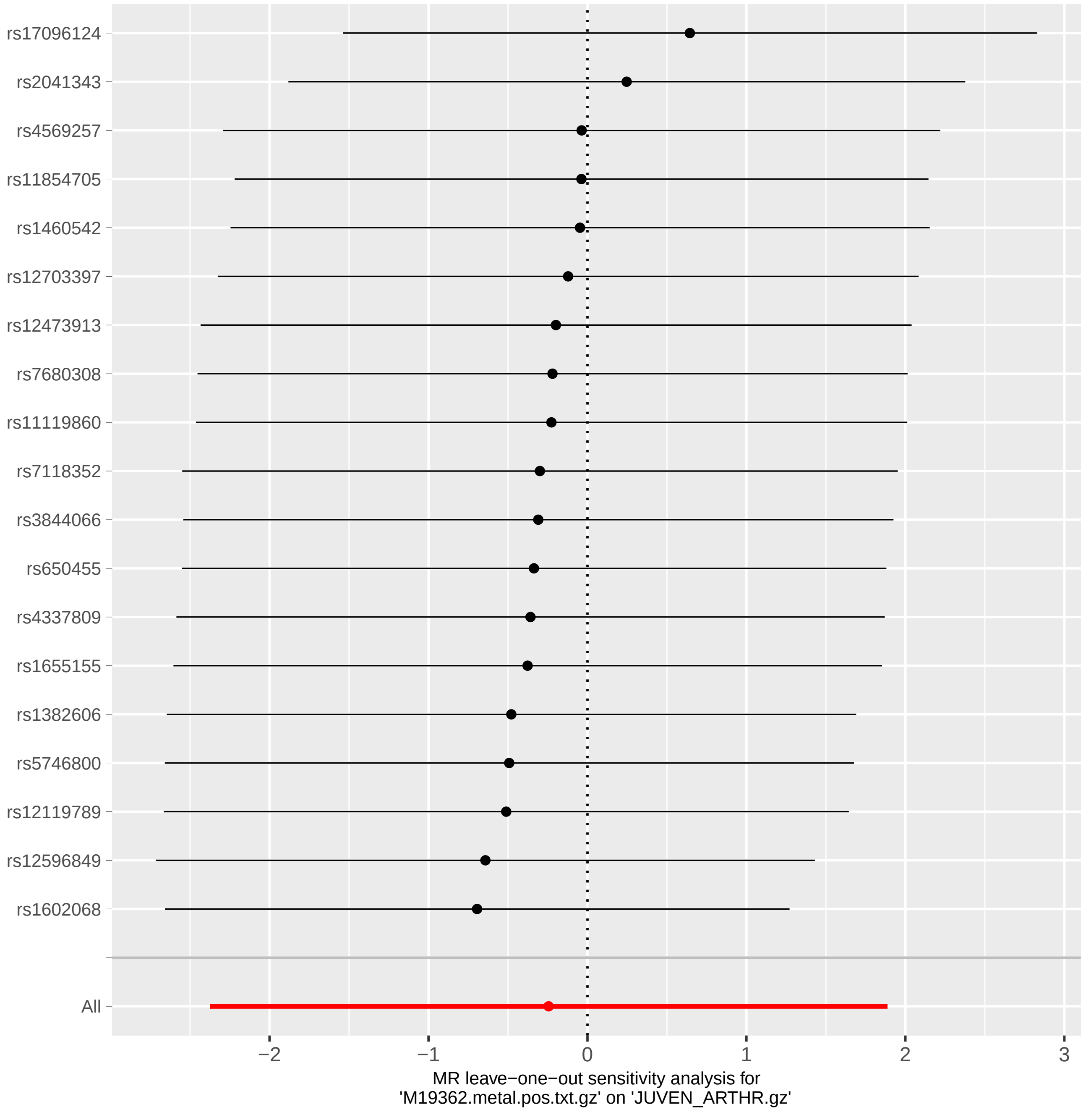

rs17096124
rs2041343
rs4569257
rs11854705
rs1460542
rs12703397
rs12473913
rs7680308
rs11119860
rs7118352
rs3844066
rs650455
rs4337809
rs1655155
rs1382606
rs5746800
rs12119789
rs12596849
rs1602068
All
−2
−1
0
1
2
3
MR leave−one−out sensitivity analysis for
'M19362.metal.pos.txt.gz' on 'JUVEN_ARTHR.gz'

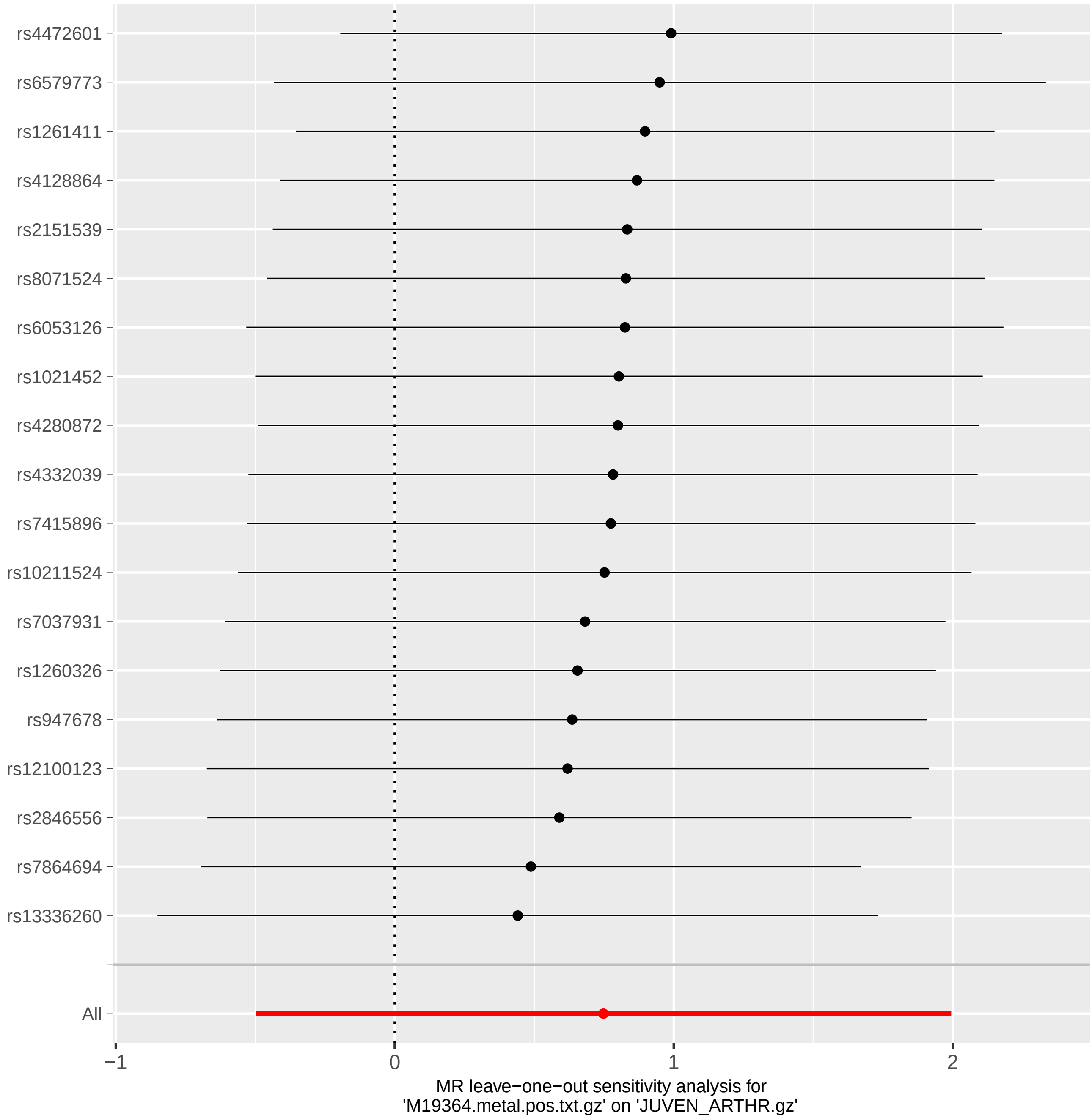
rs4472601
rs6579773
rs1261411
rs4128864
rs2151539
rs8071524
rs6053126
rs1021452
rs4280872
rs4332039
rs7415896
rs10211524
rs7037931
rs1260326
rs947678
rs12100123
rs2846556
rs7864694
rs13336260
All
−1
0
1
2
MR leave−one−out sensitivity analysis for
'M19364.metal.pos.txt.gz' on 'JUVEN_ARTHR.gz'

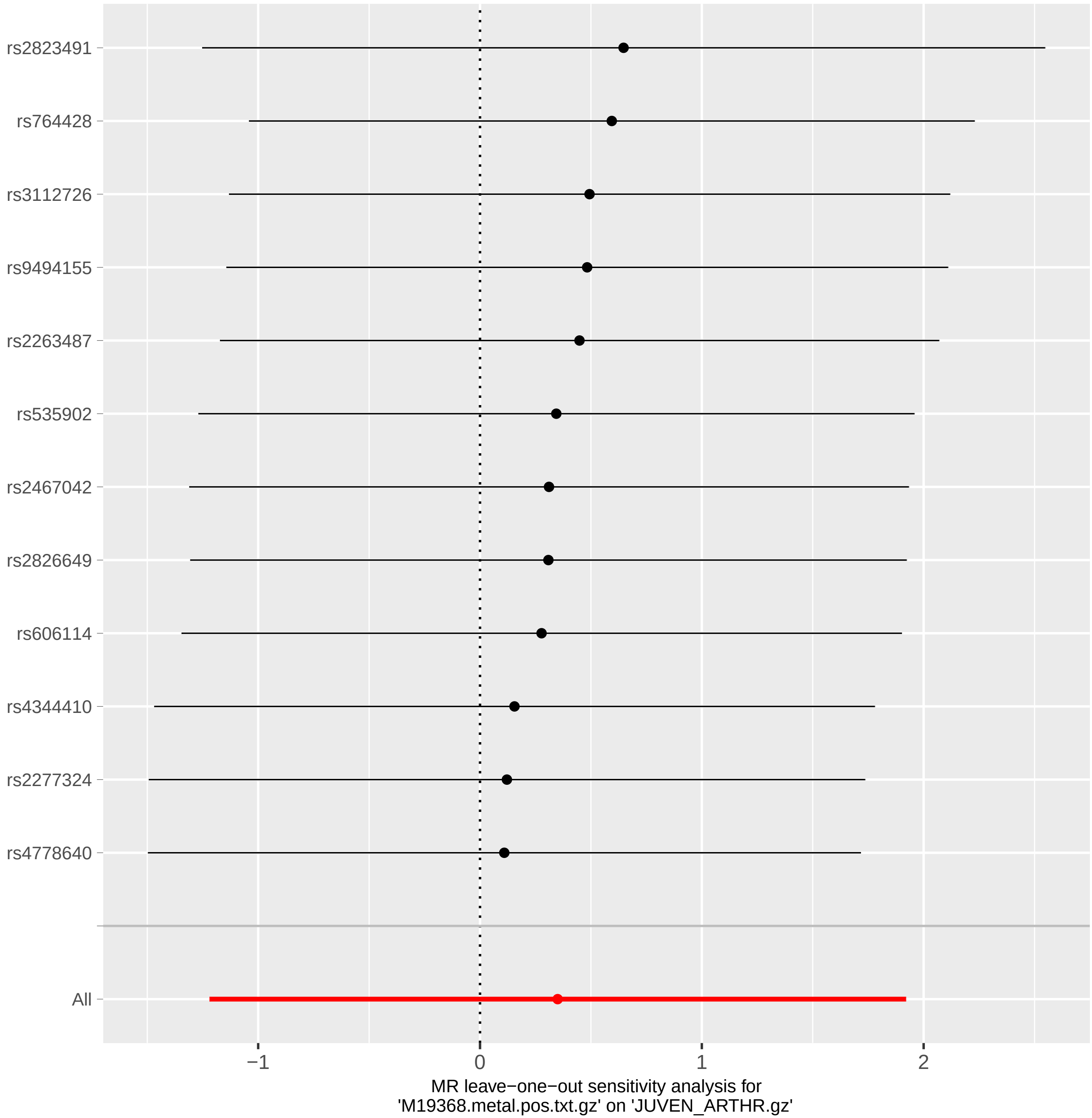

rs2823491
rs764428
rs3112726
rs9494155
rs2263487
rs535902
rs2467042
rs2826649
rs606114
rs4344410
rs2277324
rs4778640
All
−1
0
1
2
MR leave−one−out sensitivity analysis for
'M19368.metal.pos.txt.gz' on 'JUVEN_ARTHR.gz'

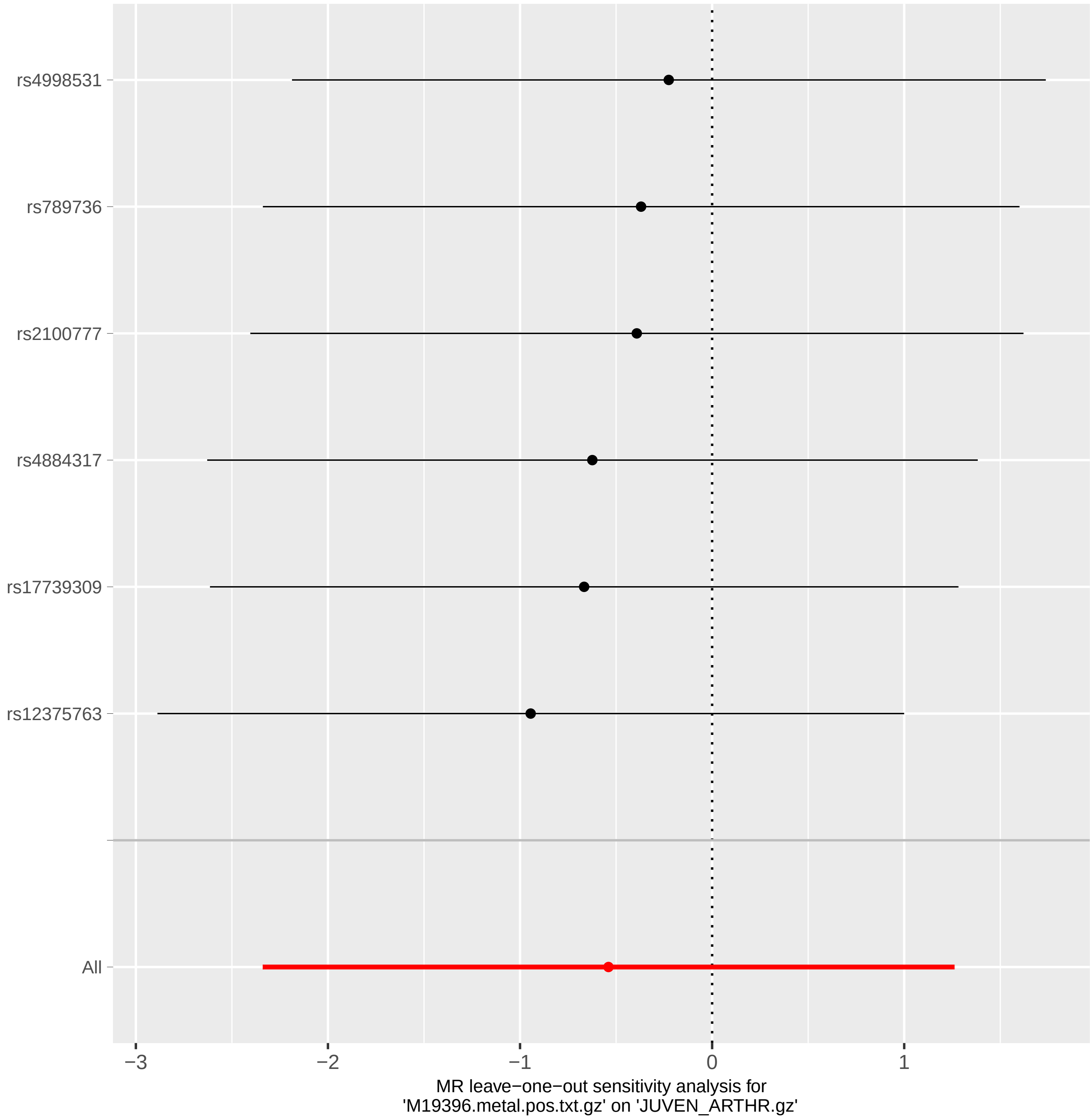
rs4998531
rs789736
rs2100777
rs4884317
rs17739309
rs12375763
All
−3
−2
−1
0
1
MR leave−one−out sensitivity analysis for
'M19396.metal.pos.txt.gz' on 'JUVEN_ARTHR.gz'

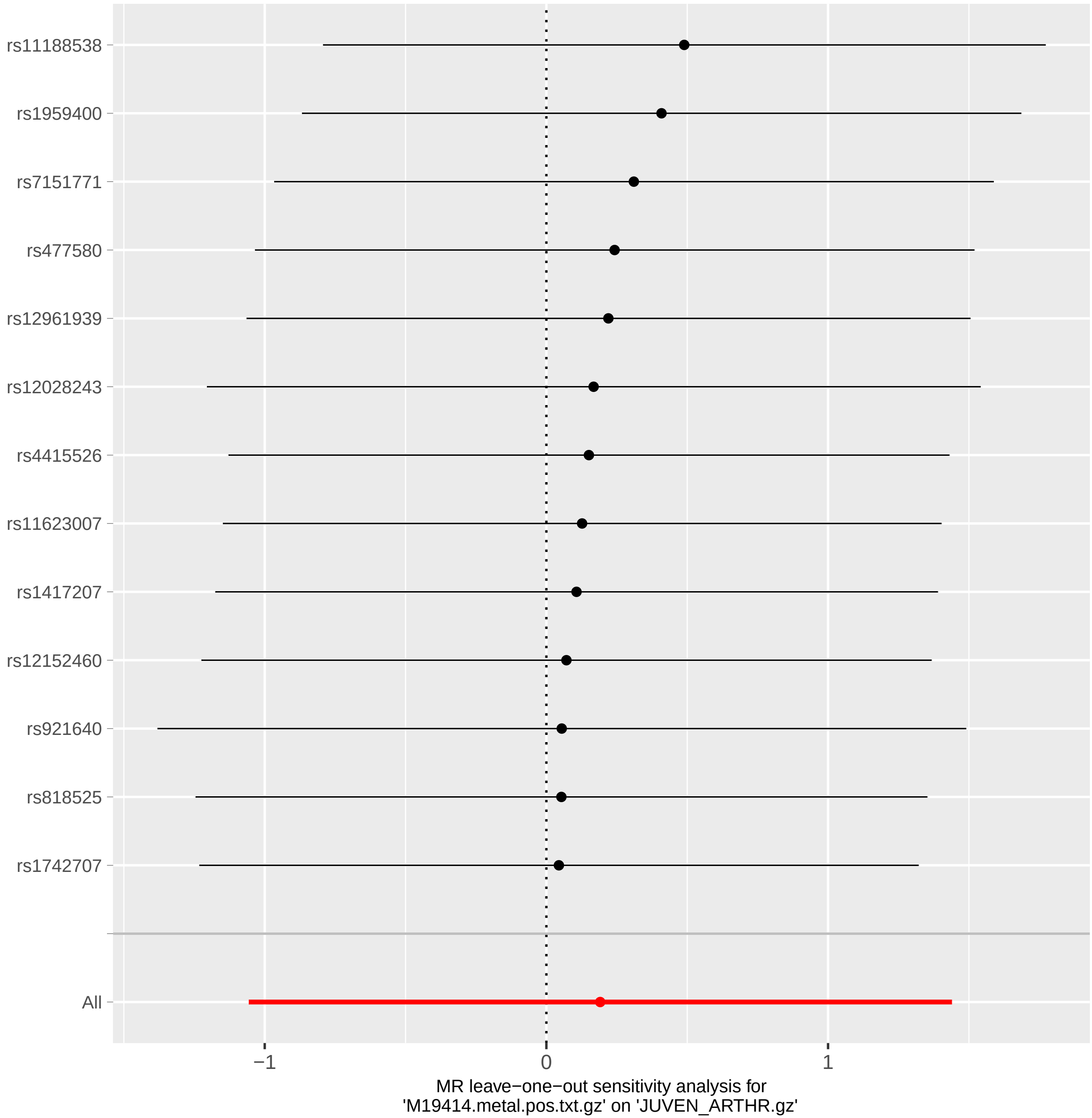

rs11188538
rs1959400
rs7151771
rs477580
rs12961939
rs12028243
rs4415526
rs11623007
rs1417207
rs12152460
rs921640
rs818525
rs1742707
All
−1
0
1
MR leave−one−out sensitivity analysis for
'M19414.metal.pos.txt.gz' on 'JUVEN_ARTHR.gz'

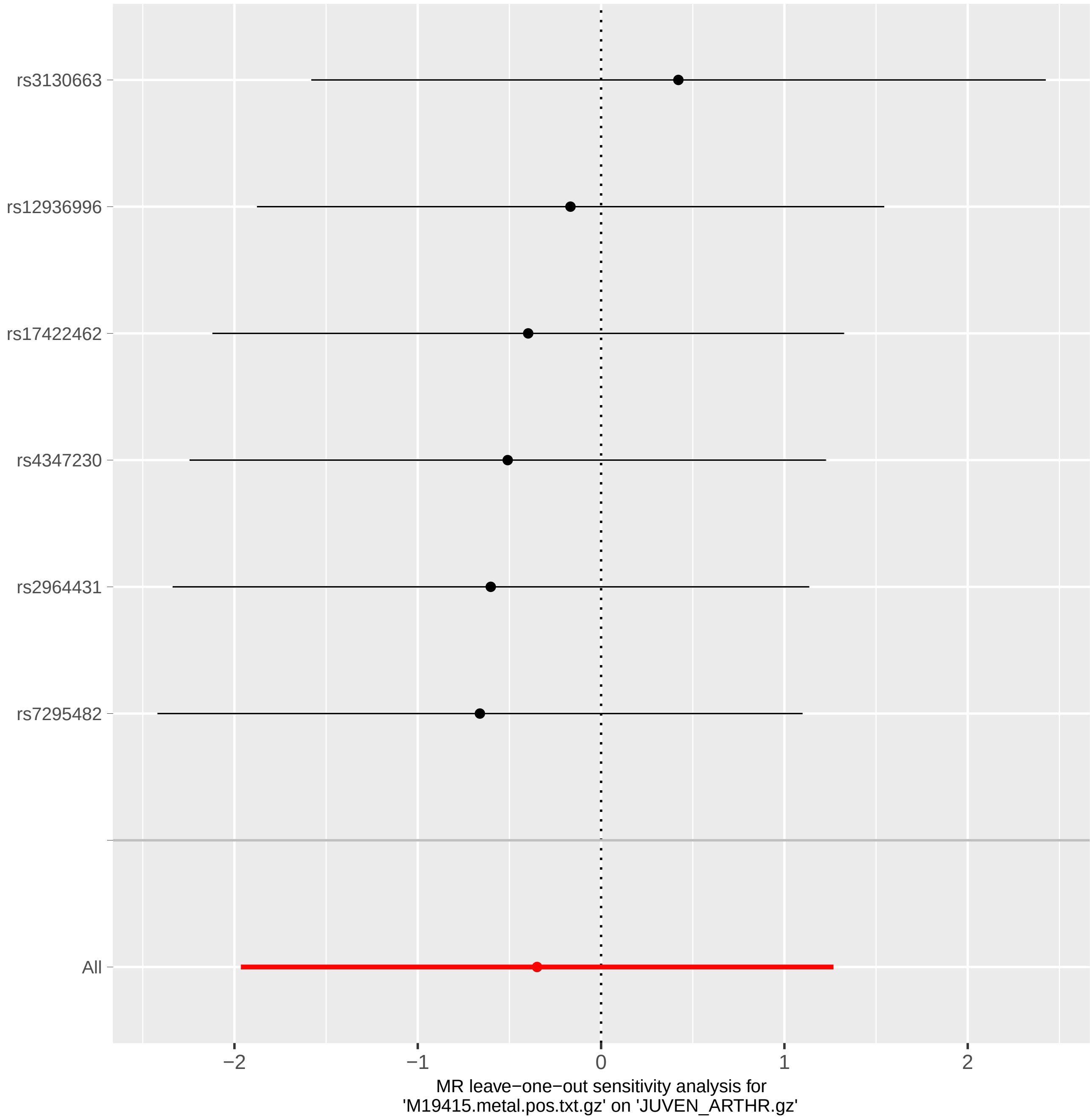
rs3130663
rs12936996
rs17422462
rs4347230
rs2964431
rs7295482
All
−2
−1
0
1
2
MR leave−one−out sensitivity analysis for
'M19415.metal.pos.txt.gz' on 'JUVEN_ARTHR.gz'

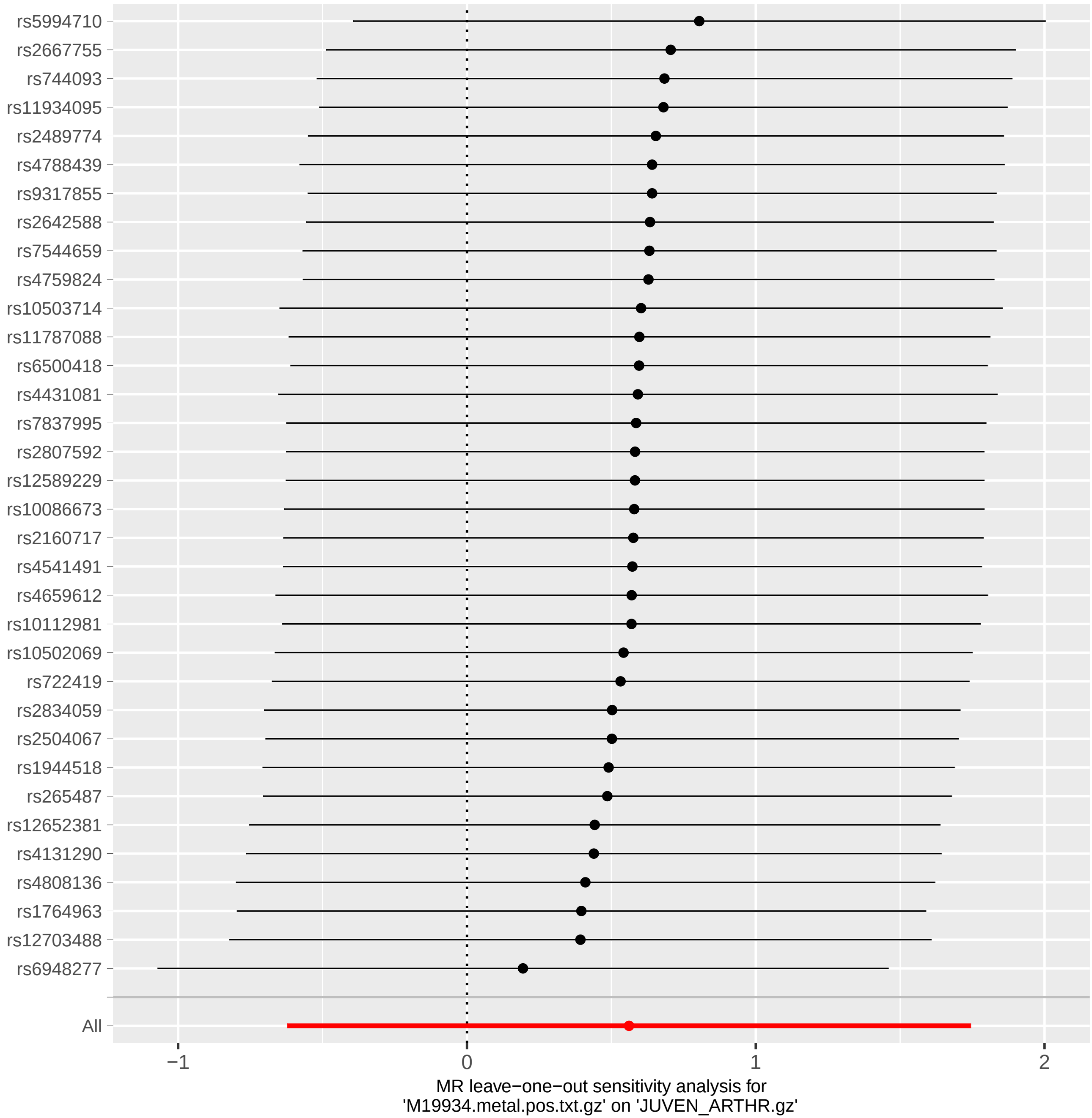
rs5994710
rs2667755
rs744093
rs11934095
rs2489774
rs4788439
rs9317855
rs2642588
rs7544659
rs4759824
rs10503714
rs11787088
rs6500418
rs4431081
rs7837995
rs2807592
rs12589229
rs10086673
rs2160717
rs4541491
rs4659612
rs10112981
rs10502069
rs722419
rs2834059
rs2504067
rs1944518
rs265487
rs12652381
rs4131290
rs4808136
rs1764963
rs12703488
rs6948277
All
−1
0
1
2
MR leave−one−out sensitivity analysis for
'M19934.metal.pos.txt.gz' on 'JUVEN_ARTHR.gz'

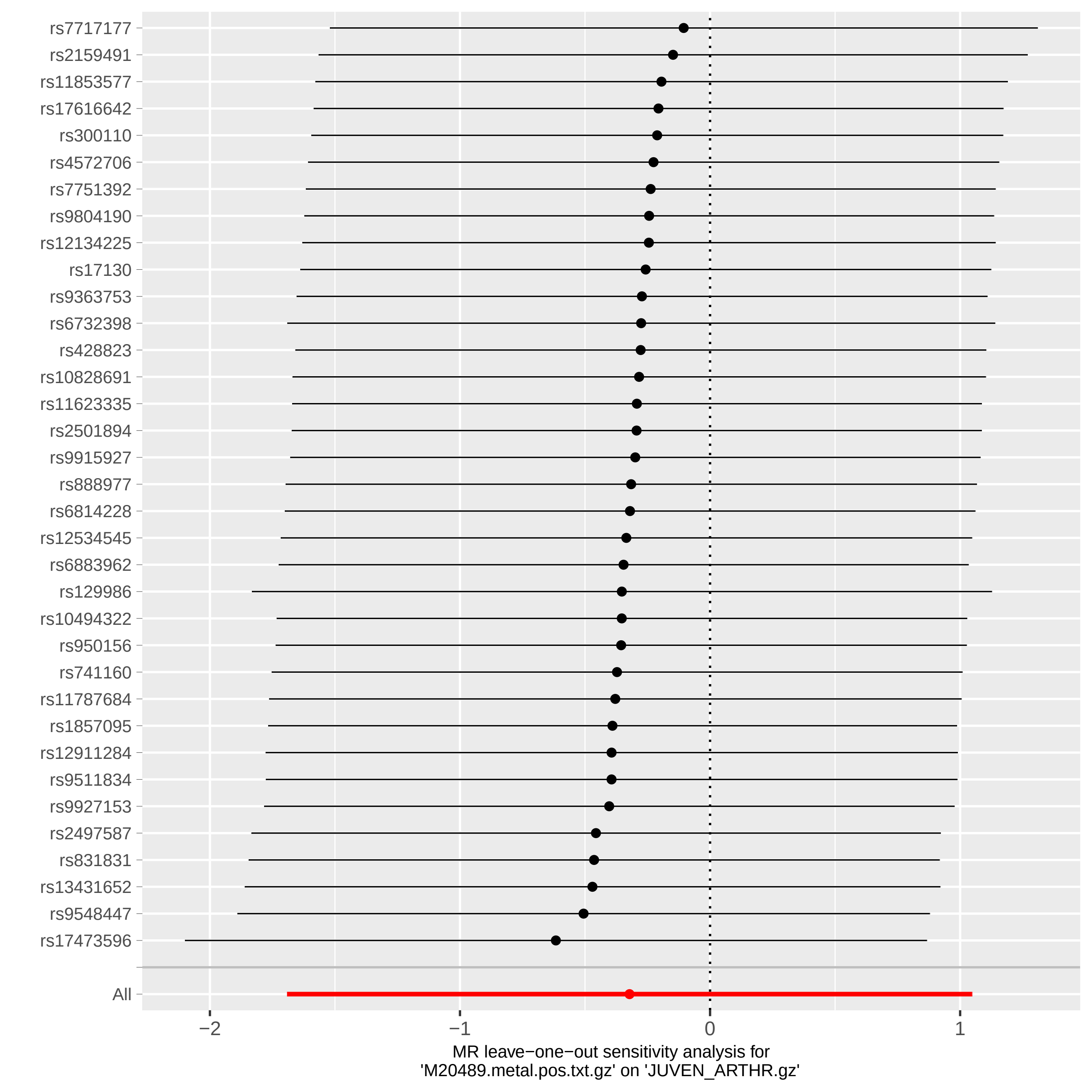

rs7717177
rs2159491
rs11853577
rs17616642
rs300110
rs4572706
rs7751392
rs9804190
rs12134225
rs17130
rs9363753
rs6732398
rs428823
rs10828691
rs11623335
rs2501894
rs9915927
rs888977
rs6814228
rs12534545
rs6883962
rs129986
rs10494322
rs950156
rs741160
rs11787684
rs1857095
rs12911284
rs9511834
rs9927153
rs2497587
rs831831
rs13431652
rs9548447
rs17473596
All
−2
−1
0
1
MR leave−one−out sensitivity analysis for
'M20489.metal.pos.txt.gz' on 'JUVEN_ARTHR.gz'

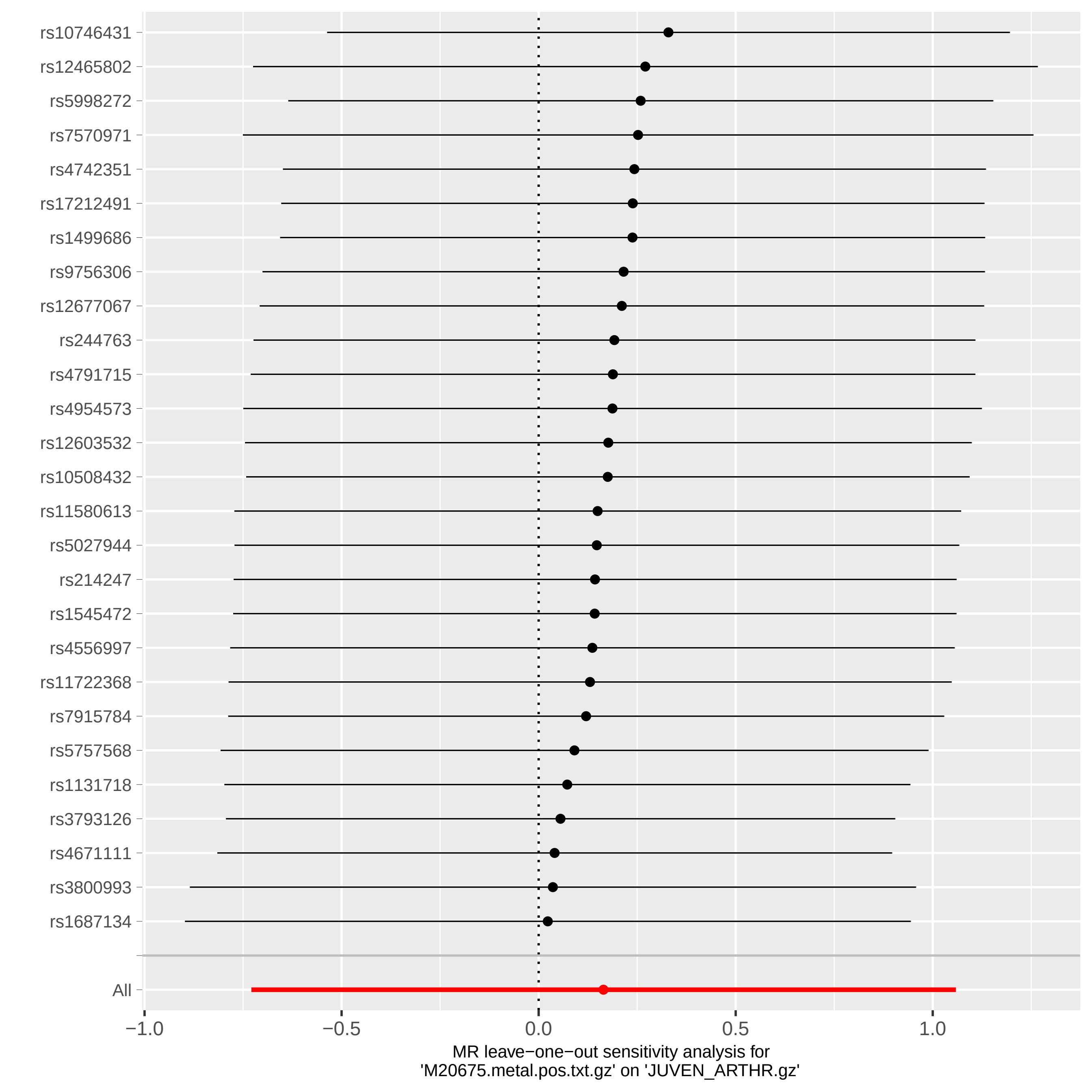
rs10746431
rs12465802
rs5998272
rs7570971
rs4742351
rs17212491
rs1499686
rs9756306
rs12677067
rs244763
rs4791715
rs4954573
rs12603532
rs10508432
rs11580613
rs5027944
rs214247
rs1545472
rs4556997
rs11722368
rs7915784
rs5757568
rs1131718
rs3793126
rs4671111
rs3800993
rs1687134
All
−1.0
−0.5
0.0
0.5
1.0
MR leave−one−out sensitivity analysis for
'M20675.metal.pos.txt.gz' on 'JUVEN_ARTHR.gz'

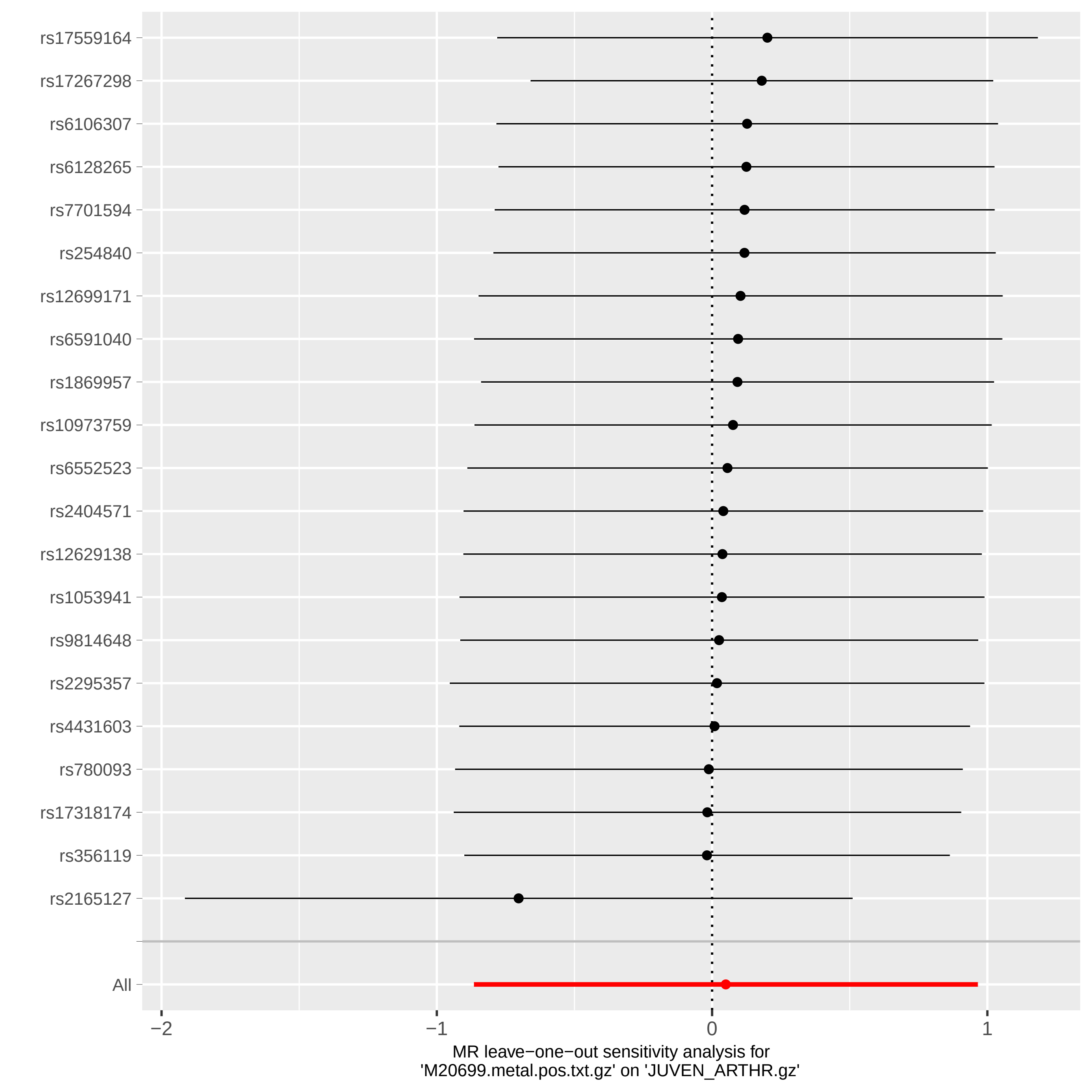
rs17559164
rs17267298
rs6106307
rs6128265
rs7701594
rs254840
rs12699171
rs6591040
rs1869957
rs10973759
rs6552523
rs2404571
rs12629138
rs1053941
rs9814648
rs2295357
rs4431603
rs780093
rs17318174
rs356119
rs2165127
All
−2
−1
0
1
MR leave−one−out sensitivity analysis for
'M20699.metal.pos.txt.gz' on 'JUVEN_ARTHR.gz'

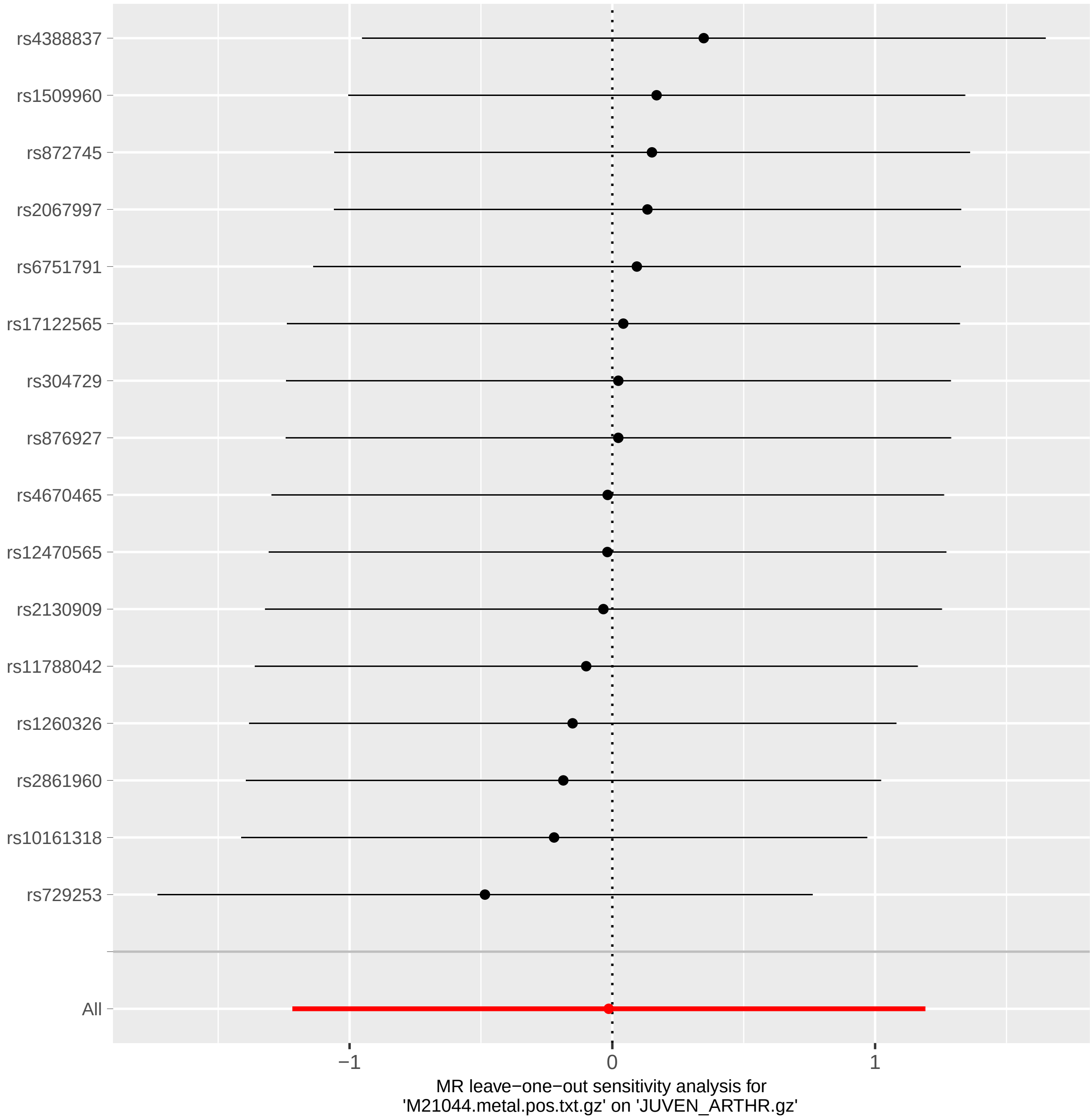
rs4388837
rs1509960
rs872745
rs2067997
rs6751791
rs17122565
rs304729
rs876927
rs4670465
rs12470565
rs2130909
rs11788042
rs1260326
rs2861960
rs10161318
rs729253
All
−1
0
1
MR leave−one−out sensitivity analysis for
'M21044.metal.pos.txt.gz' on 'JUVEN_ARTHR.gz'

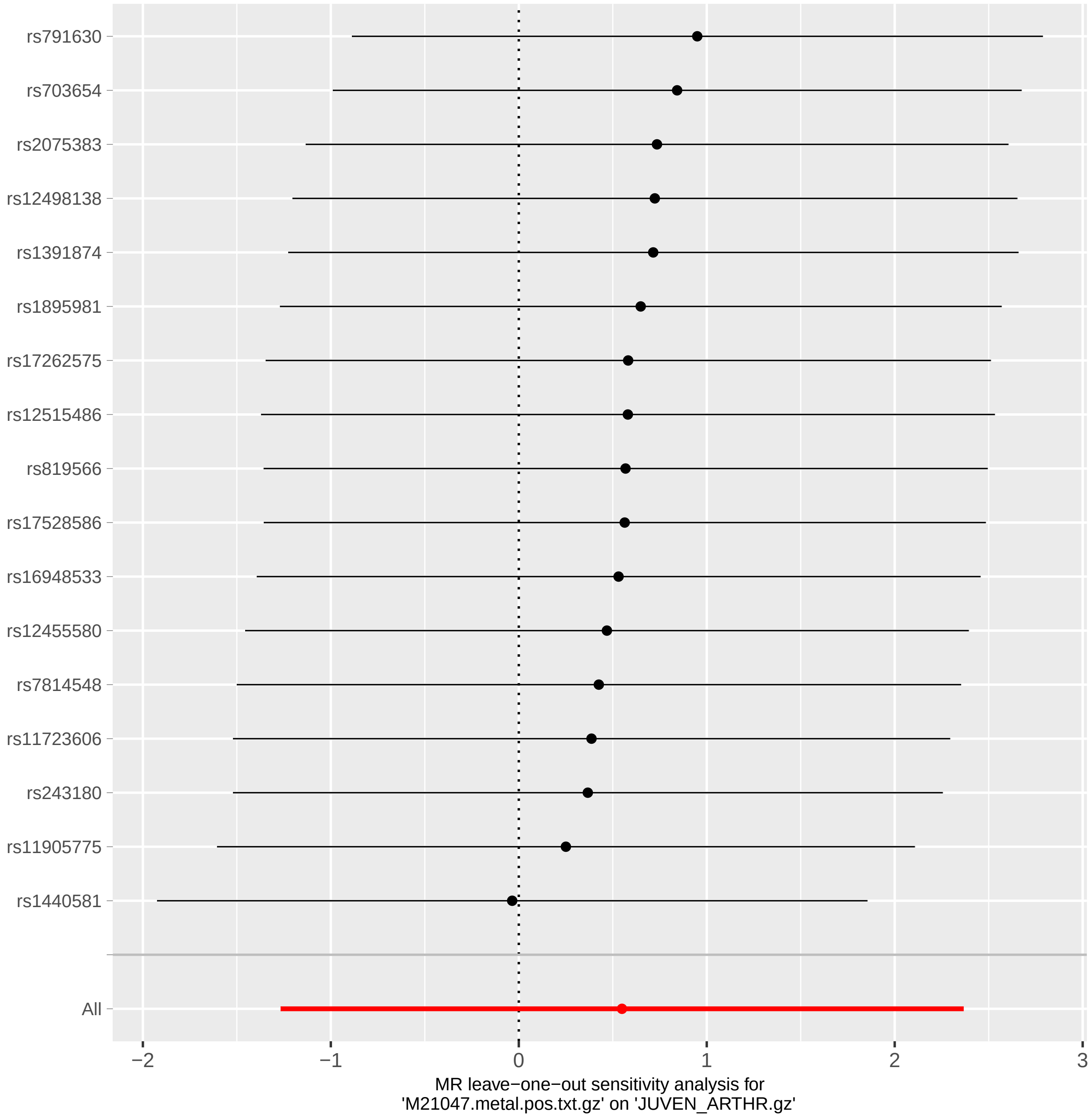
rs791630
rs703654
rs2075383
rs12498138
rs1391874
rs1895981
rs17262575
rs12515486
rs819566
rs17528586
rs16948533
rs12455580
rs7814548
rs11723606
rs243180
rs11905775
rs1440581
All
−2
−1
0
1
2
3
MR leave−one−out sensitivity analysis for
'M21047.metal.pos.txt.gz' on 'JUVEN_ARTHR.gz'

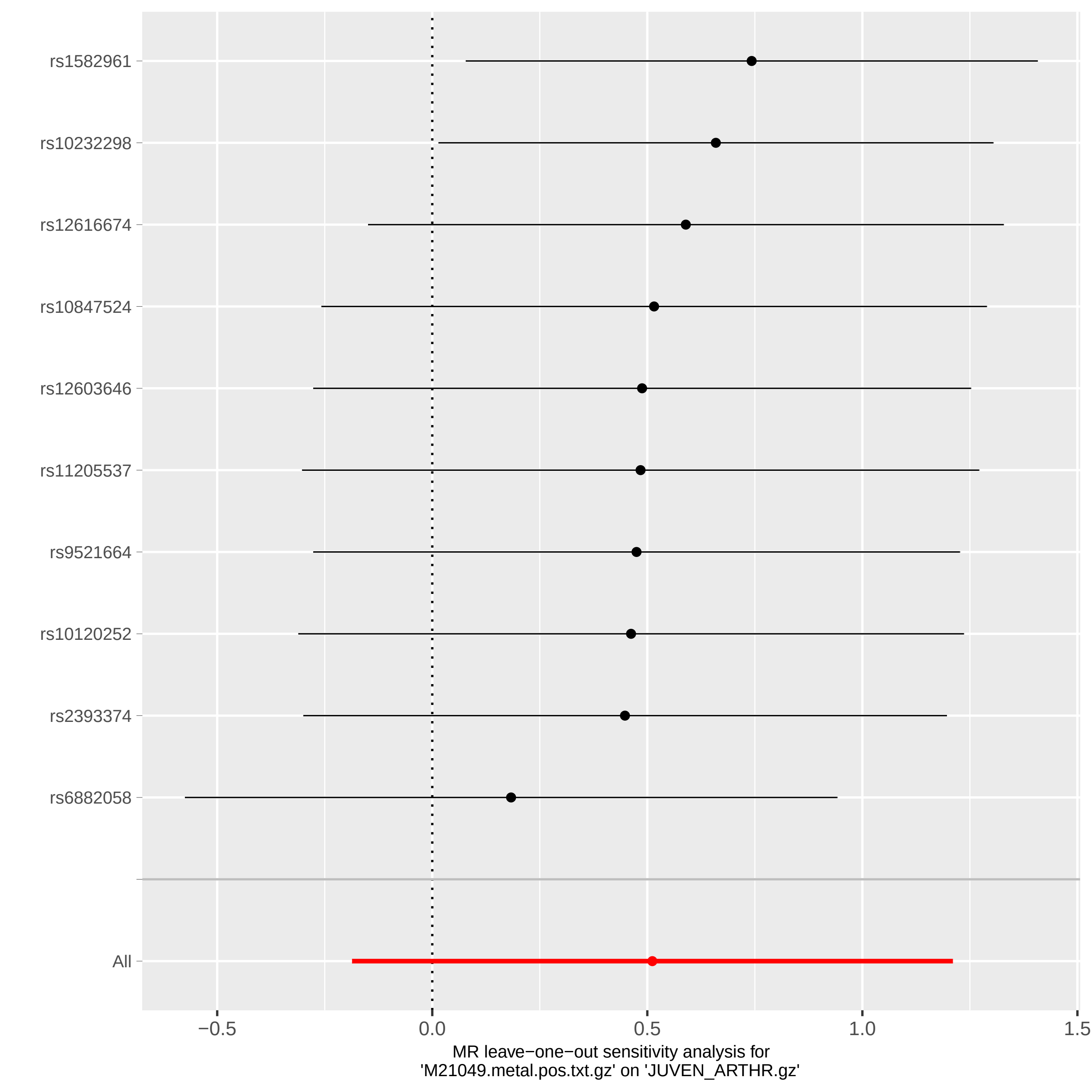
rs1582961
rs10232298
rs12616674
rs10847524
rs12603646
rs11205537
rs9521664
rs10120252
rs2393374
rs6882058
All
−0.5
0.0
0.5
1.0
1.5
MR leave−one−out sensitivity analysis for
'M21049.metal.pos.txt.gz' on 'JUVEN_ARTHR.gz'

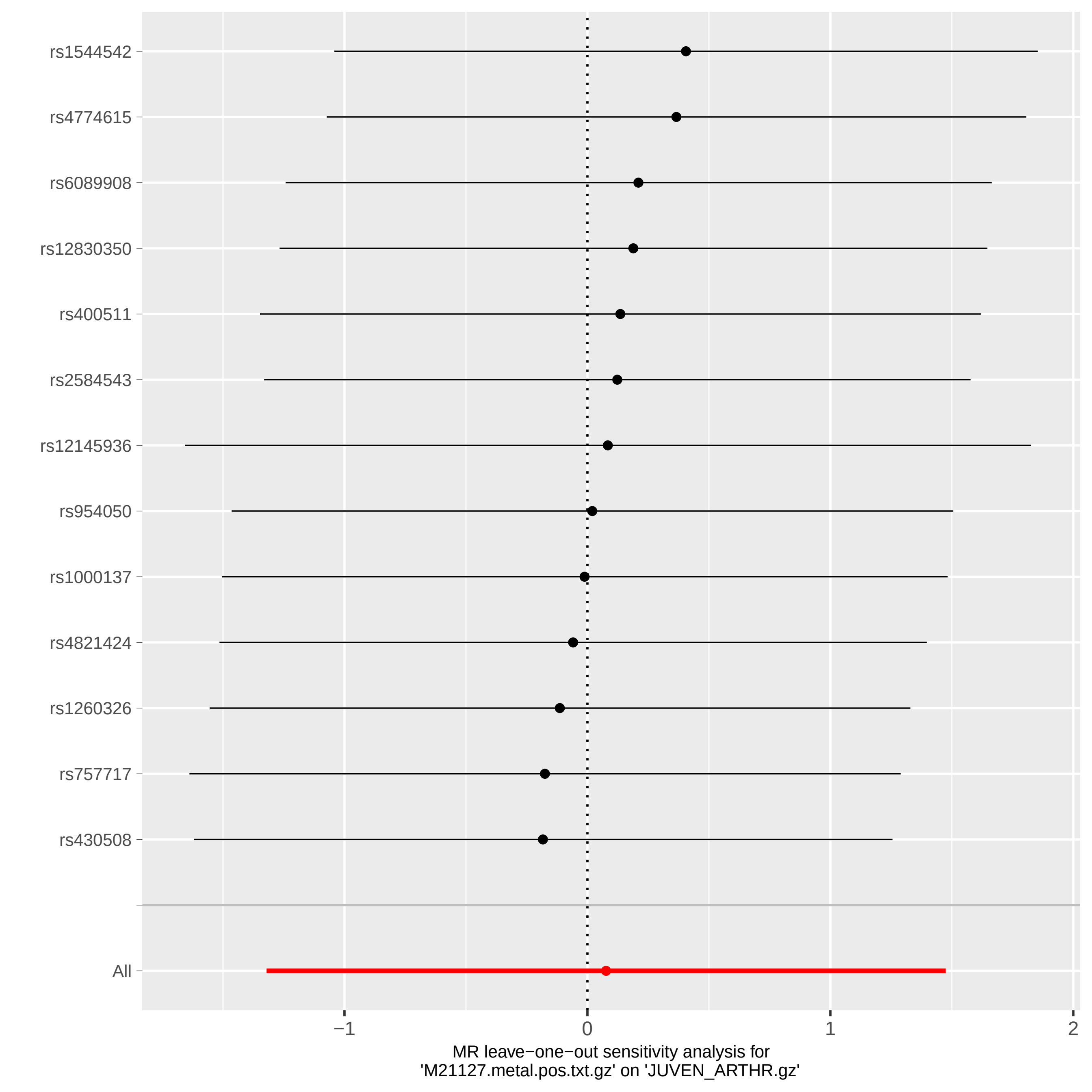
rs1544542
rs4774615
rs6089908
rs12830350
rs400511
rs2584543
rs12145936
rs954050
rs1000137
rs4821424
rs1260326
rs757717
rs430508
All
−1
0
1
2
MR leave−one−out sensitivity analysis for
'M21127.metal.pos.txt.gz' on 'JUVEN_ARTHR.gz'

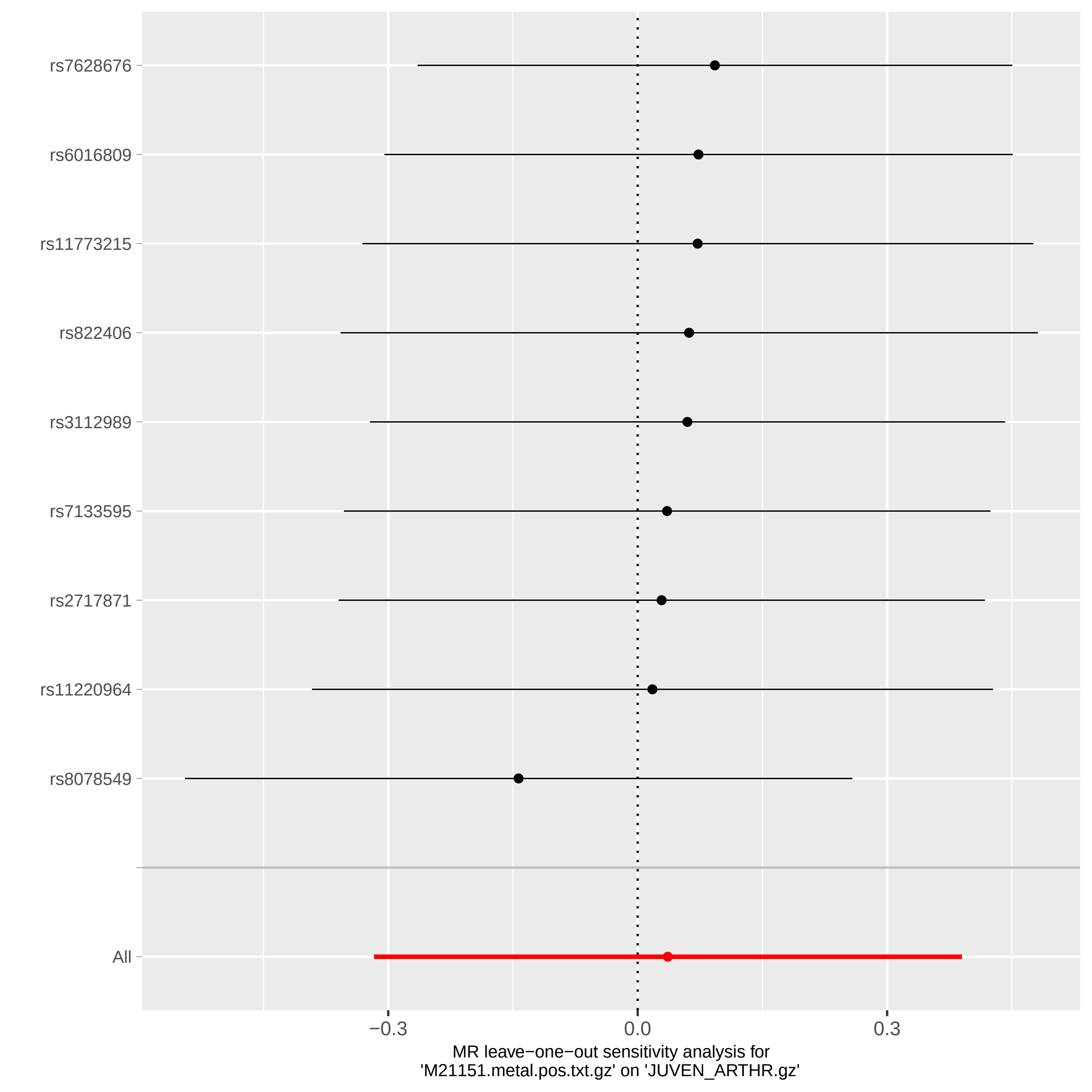
rs7628676
rs6016809
rs11773215
rs822406
rs3112989
rs7133595
rs2717871
rs11220964
rs8078549
All
−0.3
0.0
0.3
MR leave−one−out sensitivity analysis for
'M21151.metal.pos.txt.gz' on 'JUVEN_ARTHR.gz'

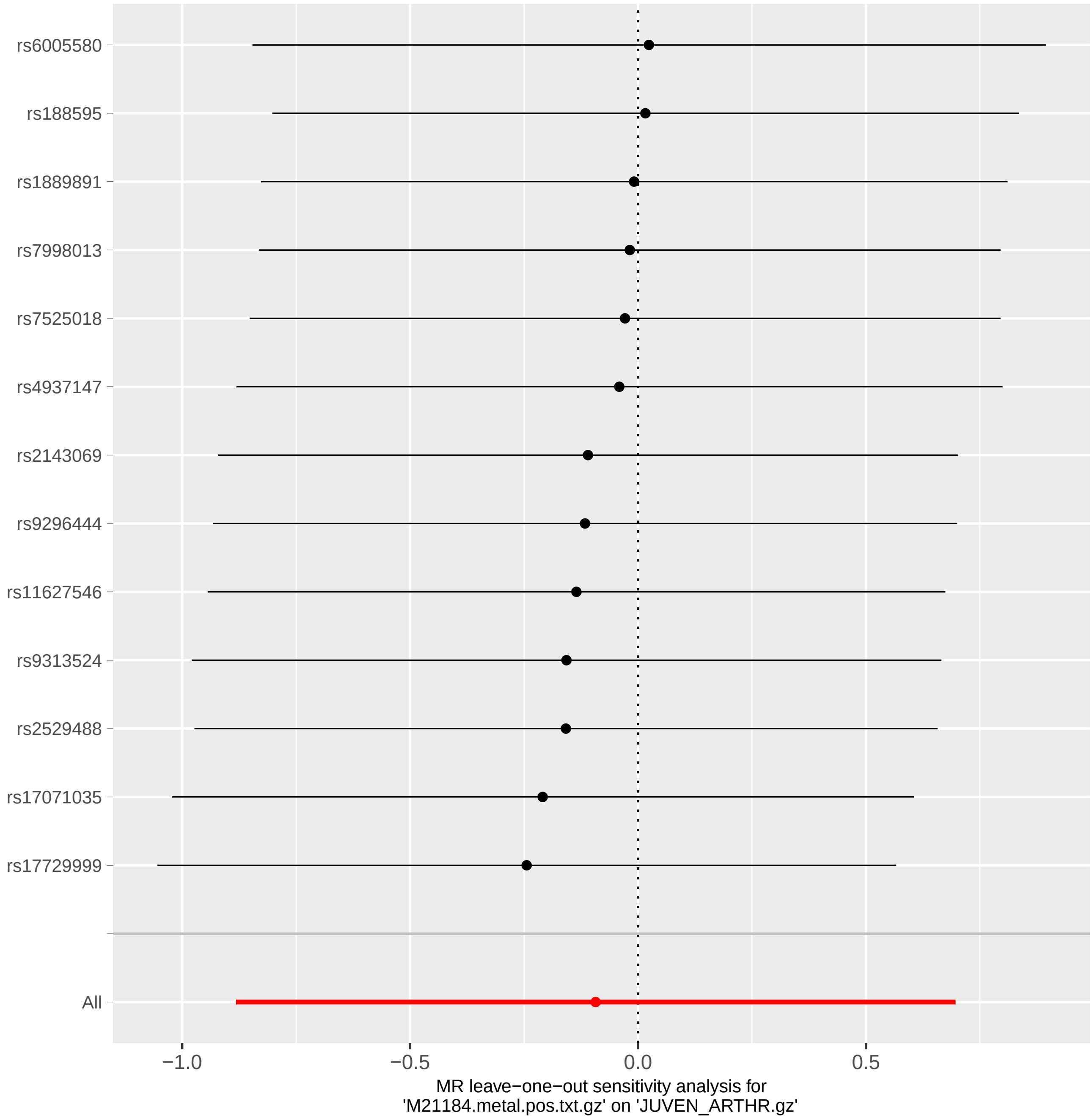

rs6005580
rs188595
rs1889891
rs7998013
rs7525018
rs4937147
rs2143069
rs9296444
rs11627546
rs9313524
rs2529488
rs17071035
rs17729999
All
−1.0
−0.5
0.0
0.5
MR leave−one−out sensitivity analysis for
'M21184.metal.pos.txt.gz' on 'JUVEN_ARTHR.gz'

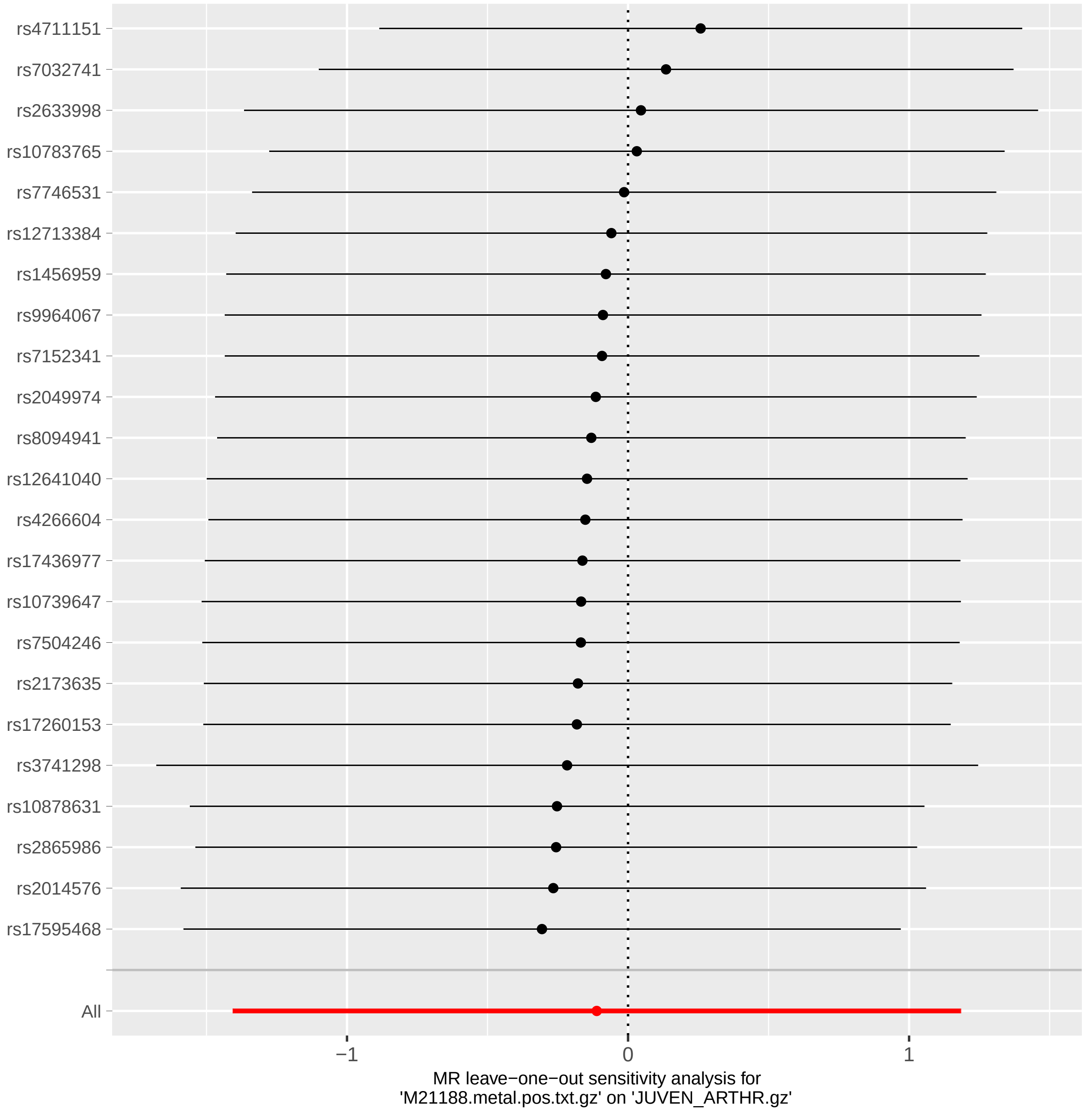

rs4711151
rs7032741
rs2633998
rs10783765
rs7746531
rs12713384
rs1456959
rs9964067
rs7152341
rs2049974
rs8094941
rs12641040
rs4266604
rs17436977
rs10739647
rs7504246
rs2173635
rs17260153
rs3741298
rs10878631
rs2865986
rs2014576
rs17595468
All
−1
0
1
MR leave−one−out sensitivity analysis for
'M21188.metal.pos.txt.gz' on 'JUVEN_ARTHR.gz'

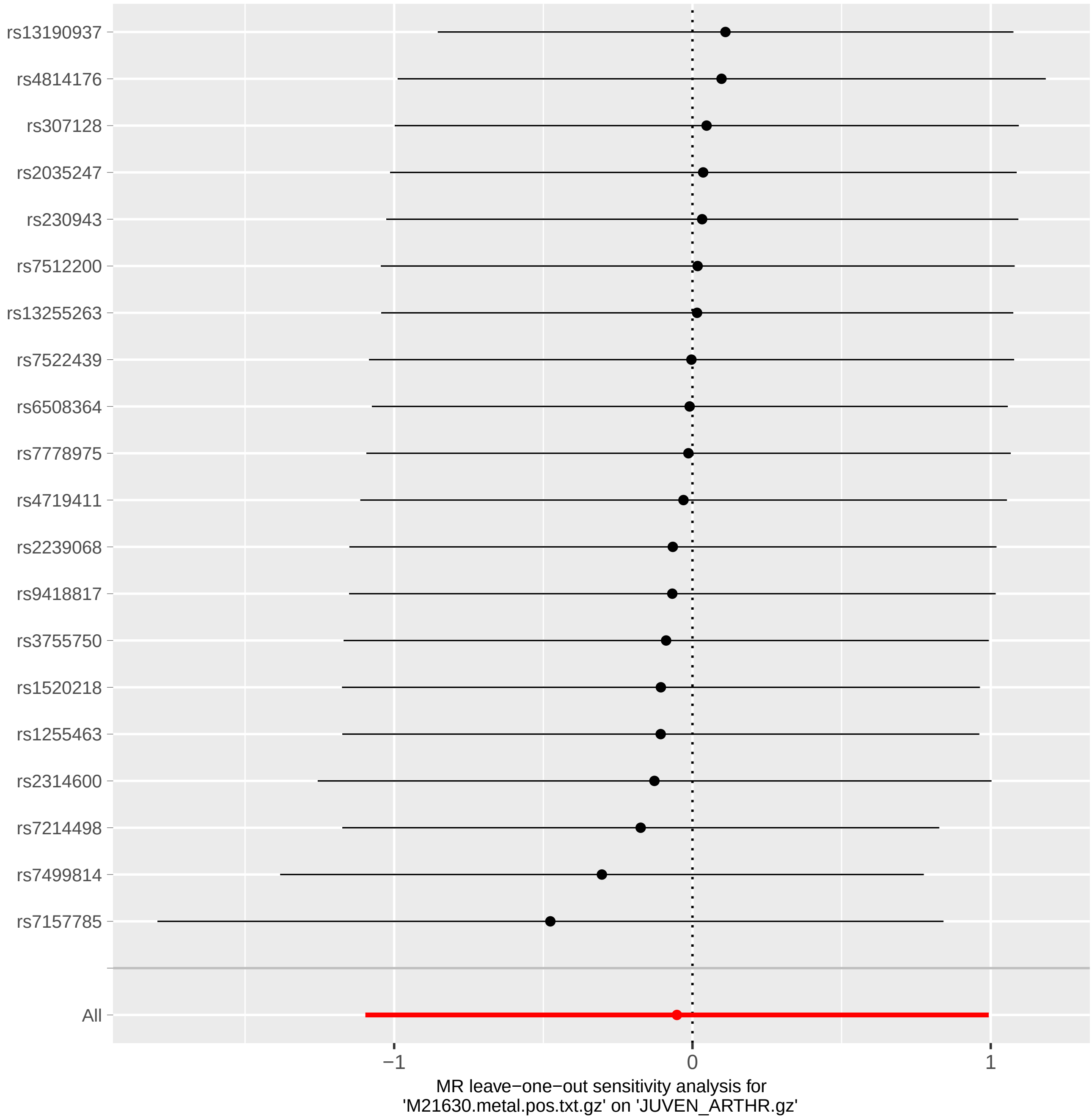
rs13190937
rs4814176
rs307128
rs2035247
rs230943
rs7512200
rs13255263
rs7522439
rs6508364
rs7778975
rs4719411
rs2239068
rs9418817
rs3755750
rs1520218
rs1255463
rs2314600
rs7214498
rs7499814
rs7157785
All
−1
0
1
MR leave−one−out sensitivity analysis for
'M21630.metal.pos.txt.gz' on 'JUVEN_ARTHR.gz'

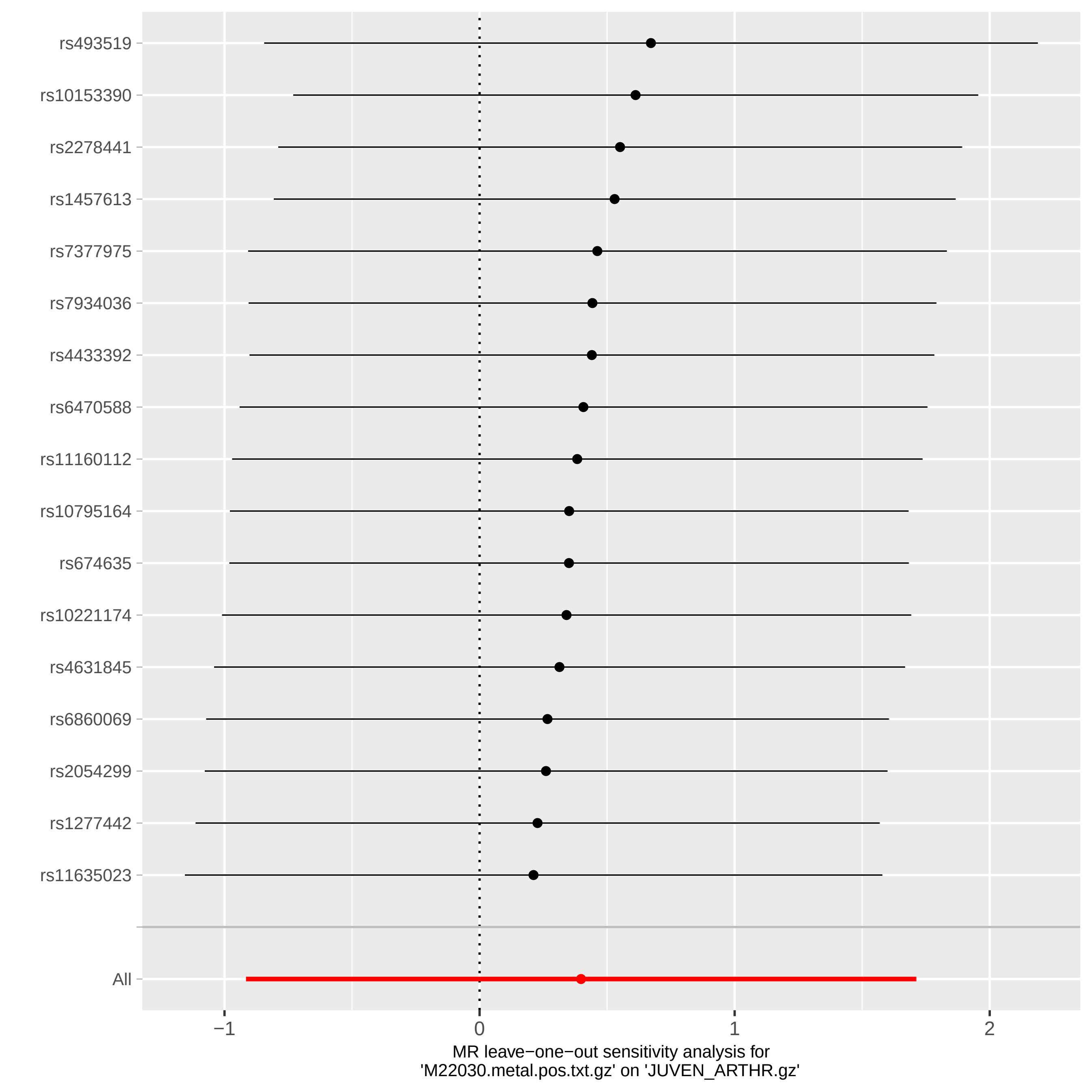
rs493519
rs10153390
rs2278441
rs1457613
rs7377975
rs7934036
rs4433392
rs6470588
rs11160112
rs10795164
rs674635
rs10221174
rs4631845
rs6860069
rs2054299
rs1277442
rs11635023
All
−1
0
1
2
MR leave−one−out sensitivity analysis for
'M22030.metal.pos.txt.gz' on 'JUVEN_ARTHR.gz'

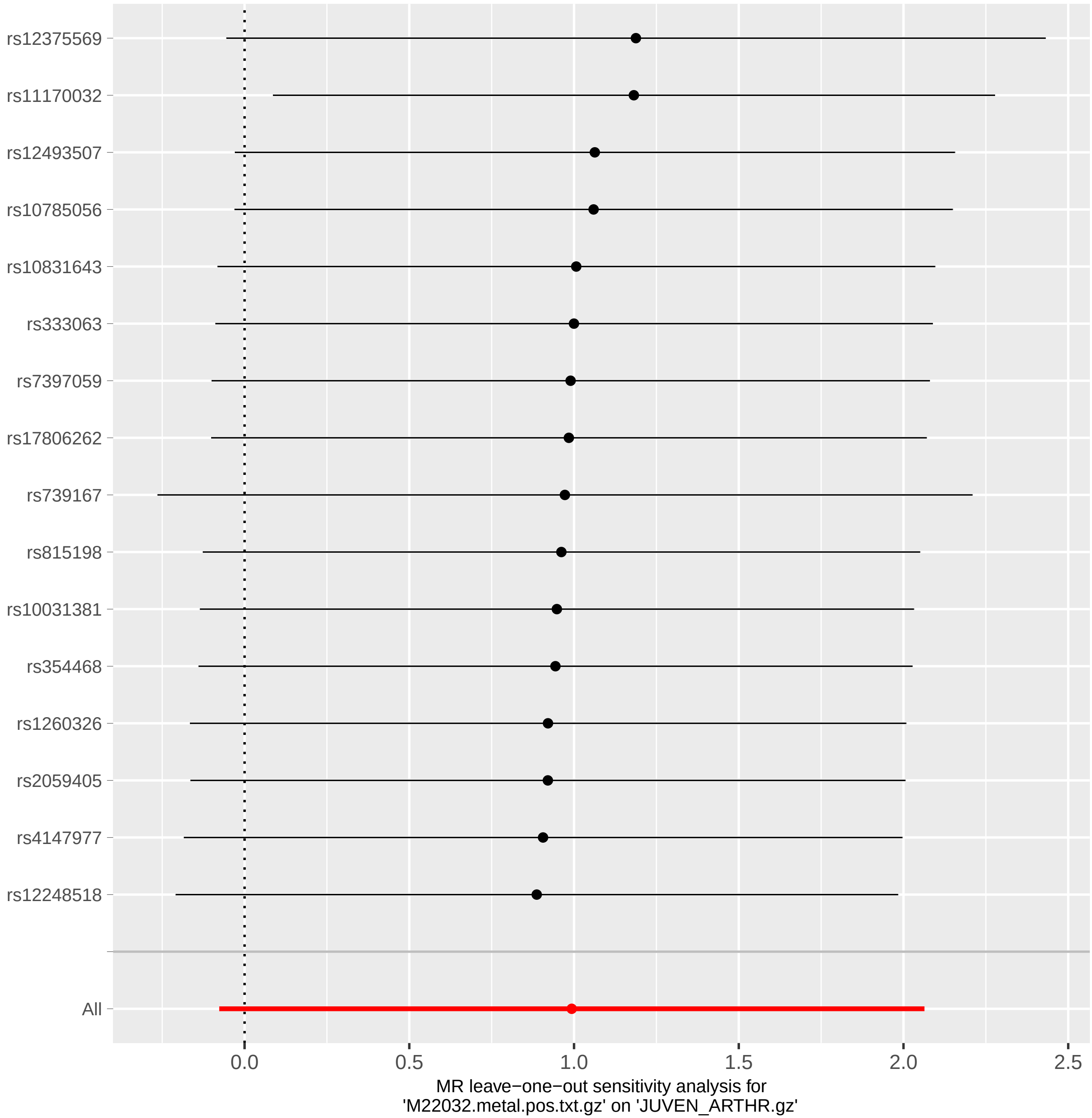
rs12375569
rs11170032
rs12493507
rs10785056
rs10831643
rs333063
rs7397059
rs17806262
rs739167
rs815198
rs10031381
rs354468
rs1260326
rs2059405
rs4147977
rs12248518
All
0.0
0.5
1.0
1.5
2.0
2.5
MR leave−one−out sensitivity analysis for
'M22032.metal.pos.txt.gz' on 'JUVEN_ARTHR.gz'

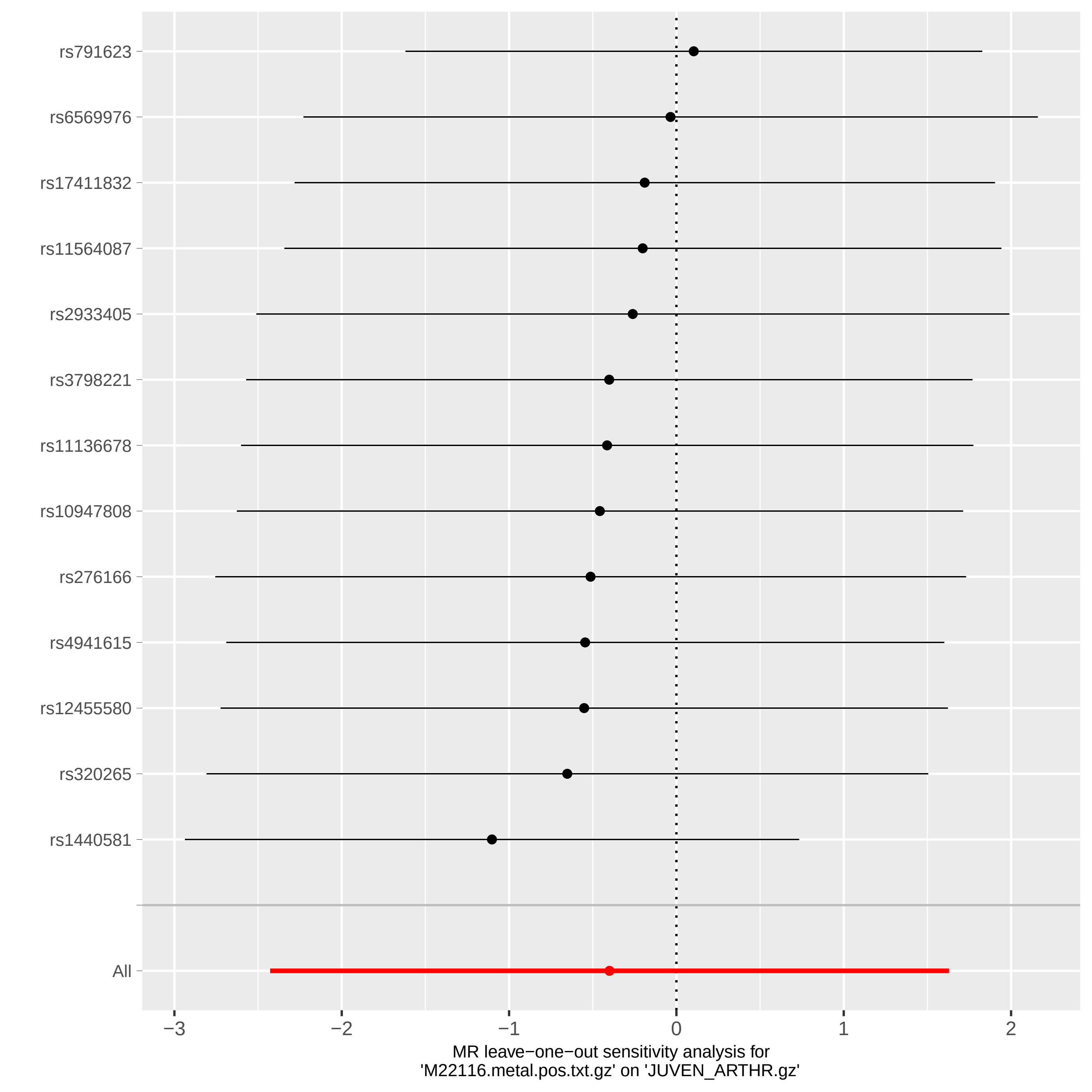
rs791623
rs6569976
rs17411832
rs11564087
rs2933405
rs3798221
rs11136678
rs10947808
rs276166
rs4941615
rs12455580
rs320265
rs1440581
All
−3
−2
−1
0
1
2
MR leave−one−out sensitivity analysis for
'M22116.metal.pos.txt.gz' on 'JUVEN_ARTHR.gz'

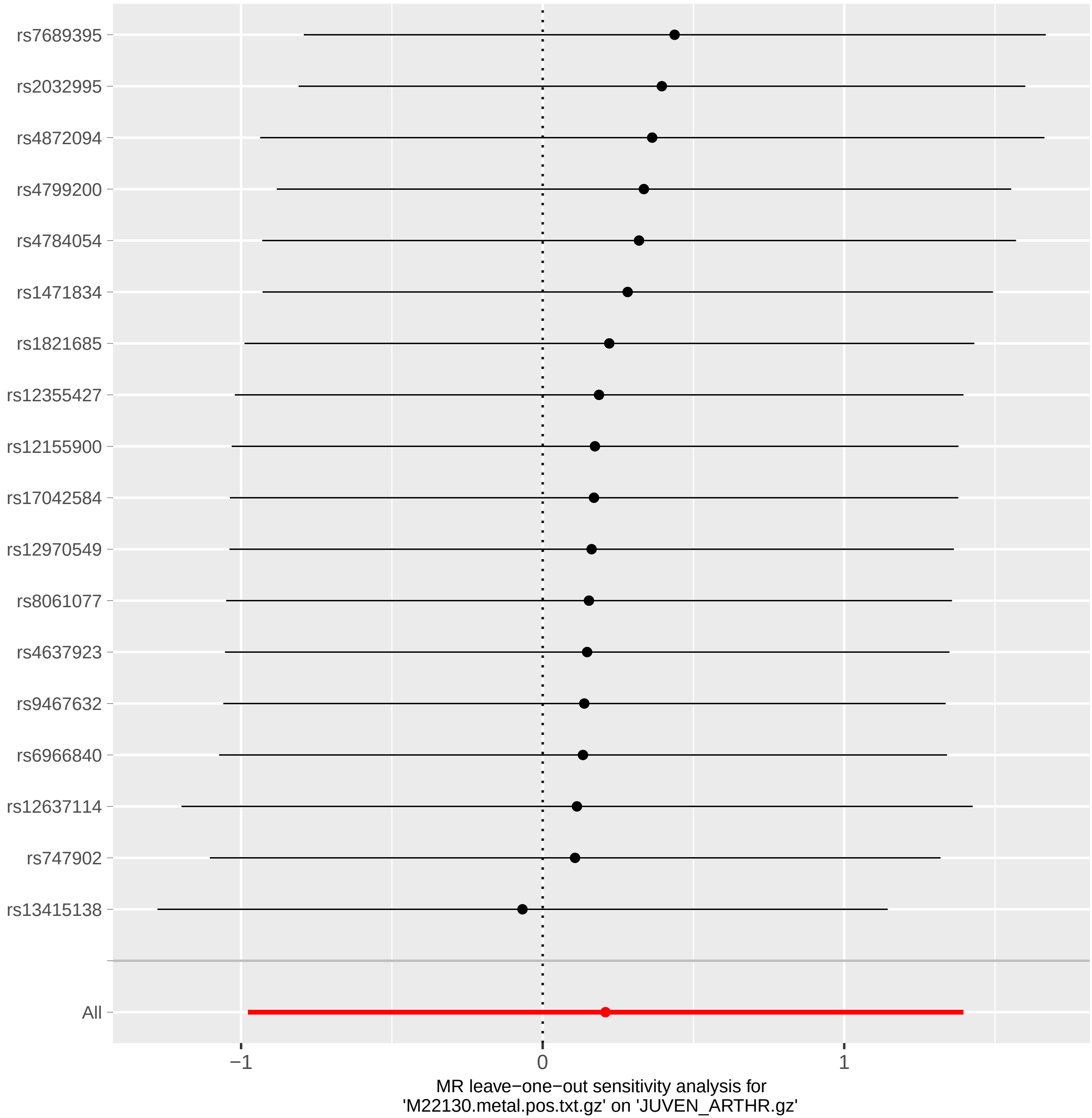
rs7689395
rs2032995
rs4872094
rs4799200
rs4784054
rs1471834
rs1821685
rs12355427
rs12155900
rs17042584
rs12970549
rs8061077
rs4637923
rs9467632
rs6966840
rs12637114
rs747902
rs13415138
All
−1
0
1
MR leave−one−out sensitivity analysis for
'M22130.metal.pos.txt.gz' on 'JUVEN_ARTHR.gz'

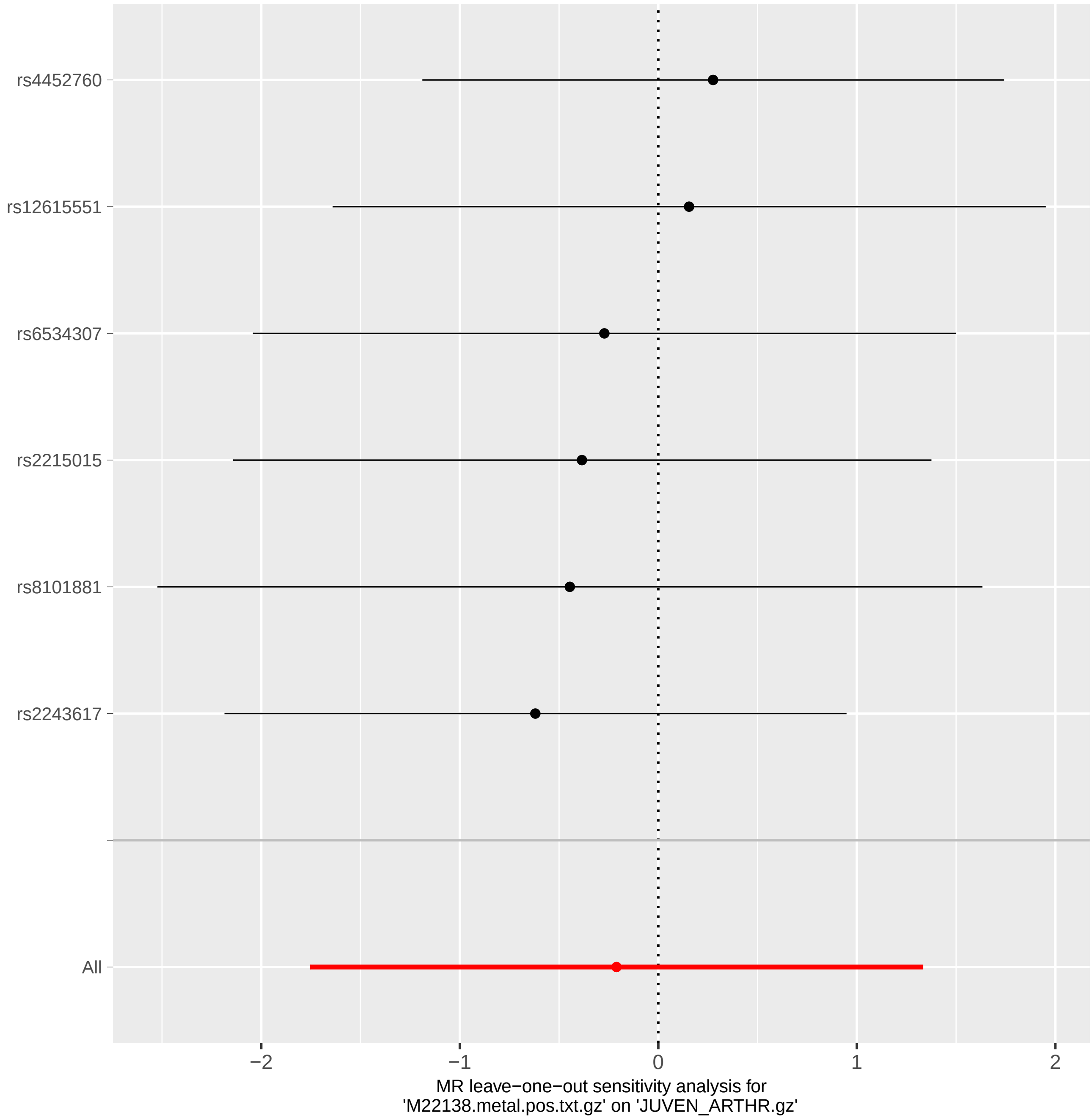

rs4452760
rs12615551
rs6534307
rs2215015
rs8101881
rs2243617
All
−2
−1
0
1
2
MR leave−one−out sensitivity analysis for
'M22138.metal.pos.txt.gz' on 'JUVEN_ARTHR.gz'

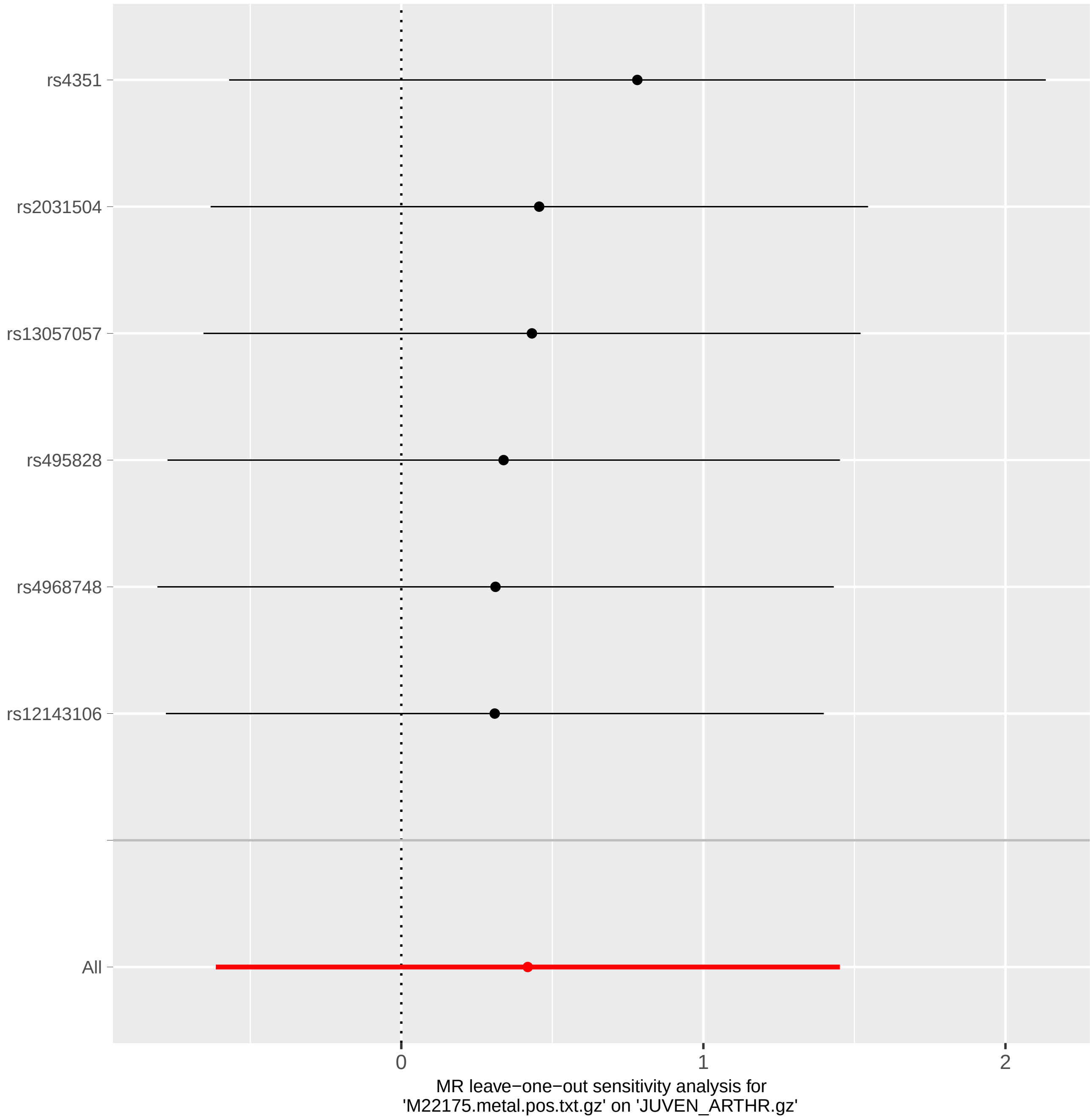
rs4351
rs2031504
rs13057057
rs495828
rs4968748
rs12143106
All
0
1
2
MR leave−one−out sensitivity analysis for
'M22175.metal.pos.txt.gz' on 'JUVEN_ARTHR.gz'

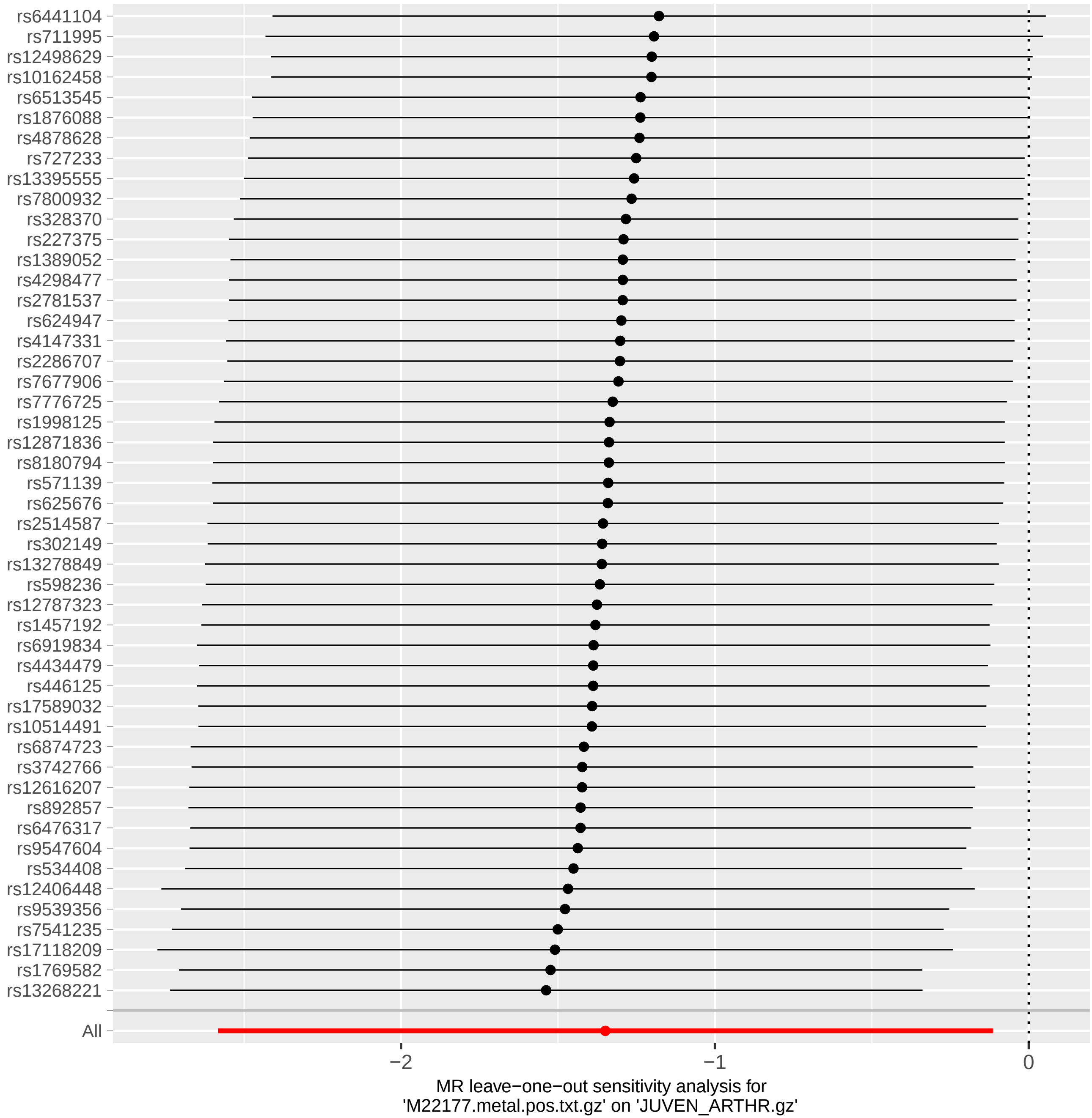

rs6441104
rs711995
rs12498629
rs10162458
rs6513545
rs1876088
rs4878628
rs727233
rs13395555
rs7800932
rs328370
rs227375
rs1389052
rs4298477
rs2781537
rs624947
rs4147331
rs2286707
rs7677906
rs7776725
rs1998125
rs12871836
rs8180794
rs571139
rs625676
rs2514587
rs302149
rs13278849
rs598236
rs12787323
rs1457192
rs6919834
rs4434479
rs446125
rs17589032
rs10514491
rs6874723
rs3742766
rs12616207
rs892857
rs6476317
rs9547604
rs534408
rs12406448
rs9539356
rs7541235
rs17118209
rs1769582
rs13268221
All
−2
−1
0
MR leave−one−out sensitivity analysis for
'M22177.metal.pos.txt.gz' on 'JUVEN_ARTHR.gz'

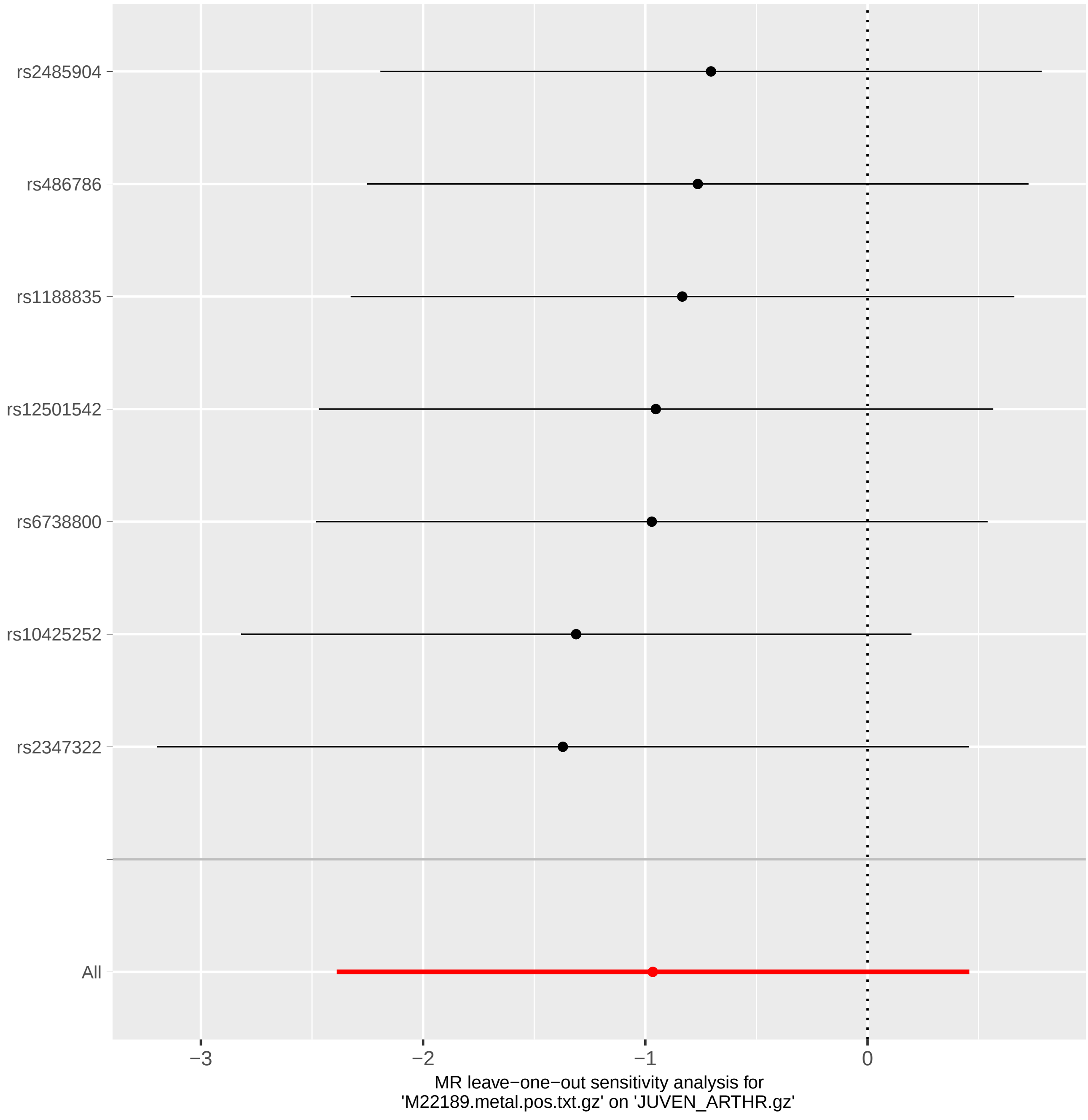
rs2485904
rs486786
rs1188835
rs12501542
rs6738800
rs10425252
rs2347322
All
−3
−2
−1
0
MR leave−one−out sensitivity analysis for
'M22189.metal.pos.txt.gz' on 'JUVEN_ARTHR.gz'

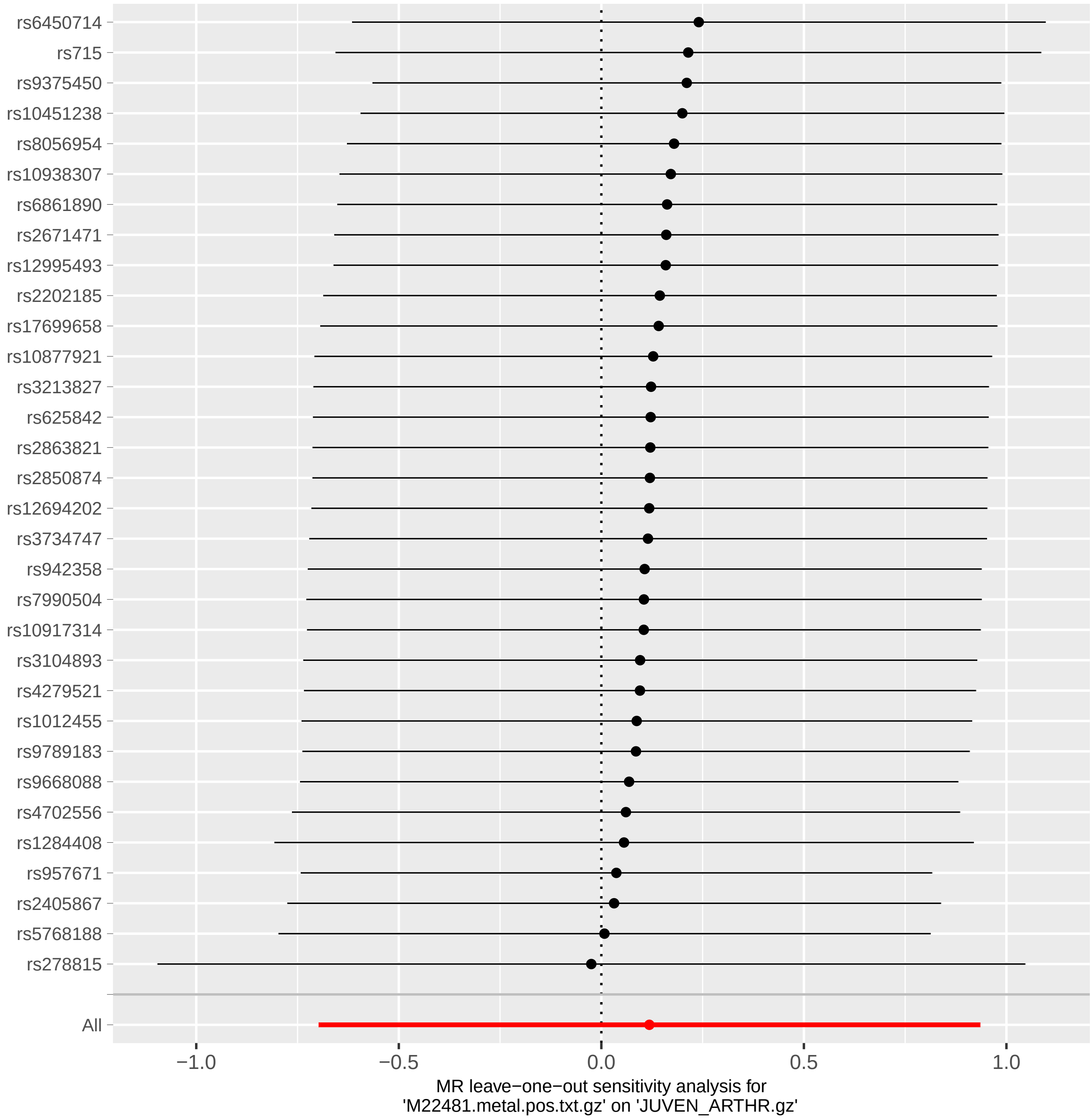
rs6450714
rs715
rs9375450
rs10451238
rs8056954
rs10938307
rs6861890
rs2671471
rs12995493
rs2202185
rs17699658
rs10877921
rs3213827
rs625842
rs2863821
rs2850874
rs12694202
rs3734747
rs942358
rs7990504
rs10917314
rs3104893
rs4279521
rs1012455
rs9789183
rs9668088
rs4702556
rs1284408
rs957671
rs2405867
rs5768188
rs278815
All
−1.0
−0.5
0.0
0.5
1.0
MR leave−one−out sensitivity analysis for
'M22481.metal.pos.txt.gz' on 'JUVEN_ARTHR.gz'

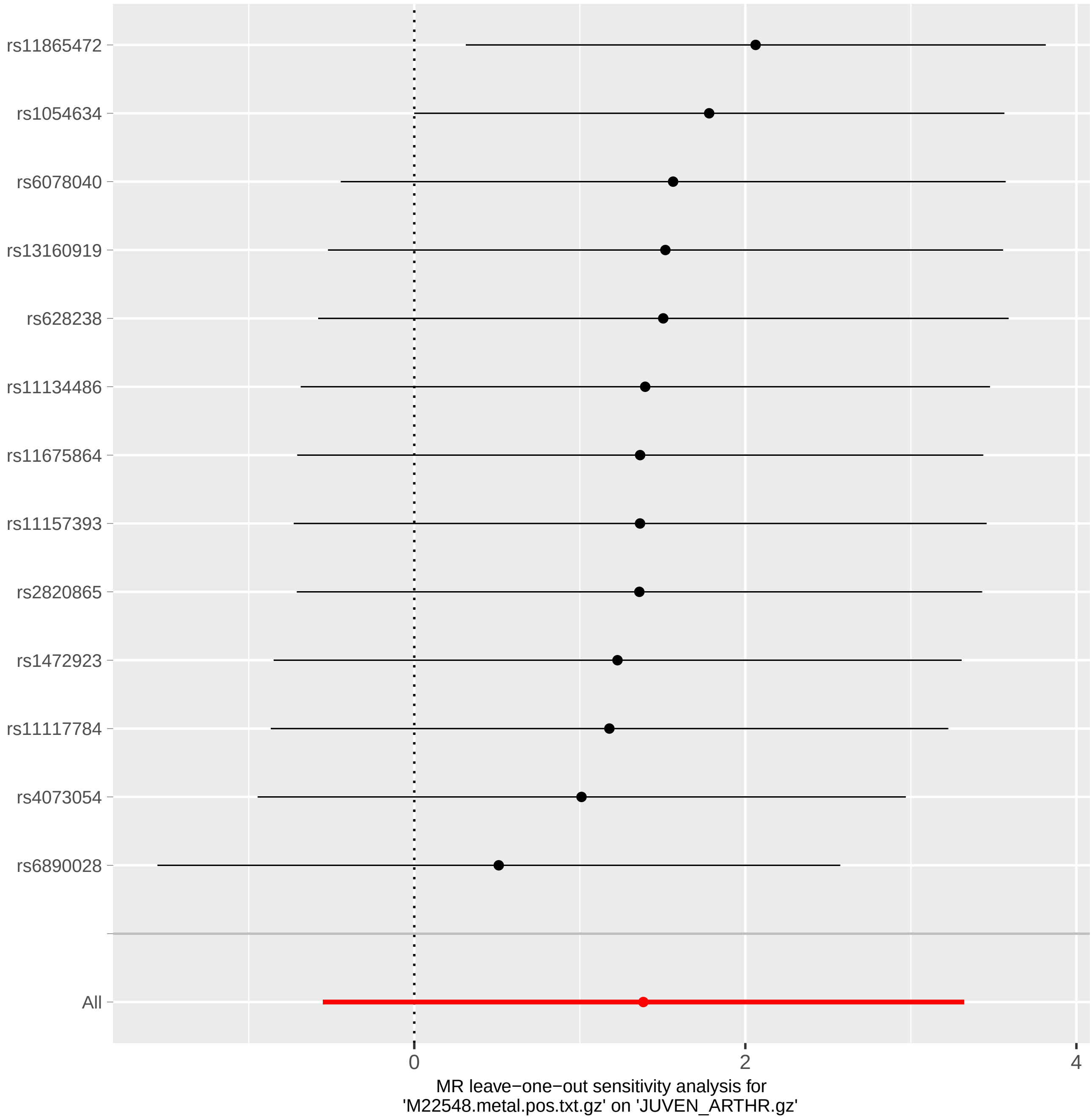
rs11865472
rs1054634
rs6078040
rs13160919
rs628238
rs11134486
rs11675864
rs11157393
rs2820865
rs1472923
rs11117784
rs4073054
rs6890028
All
0
2
4
MR leave−one−out sensitivity analysis for
'M22548.metal.pos.txt.gz' on 'JUVEN_ARTHR.gz'

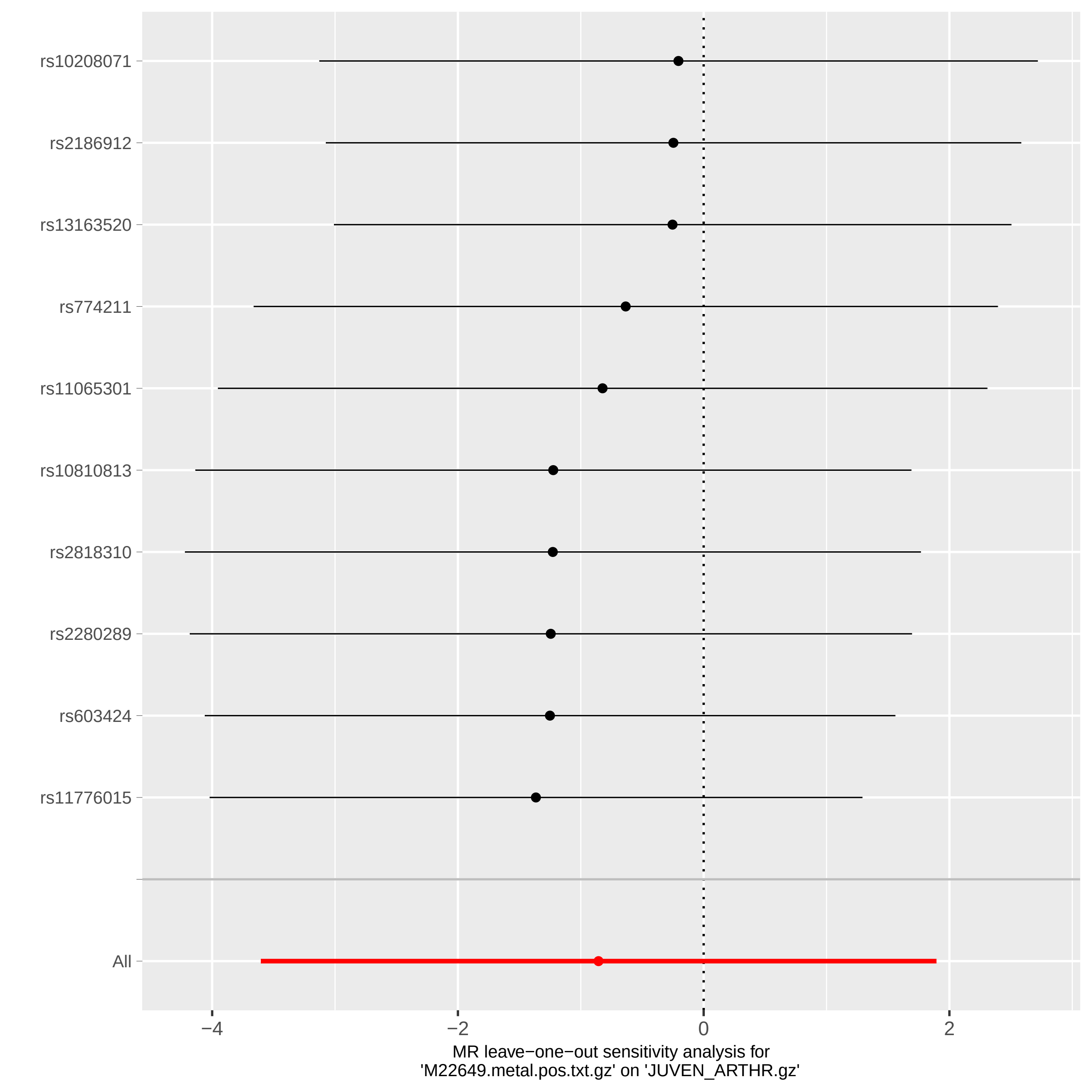
rs10208071
rs2186912
rs13163520
rs774211
rs11065301
rs10810813
rs2818310
rs2280289
rs603424
rs11776015
All
−4
−2
0
2
MR leave−one−out sensitivity analysis for
'M22649.metal.pos.txt.gz' on 'JUVEN_ARTHR.gz'

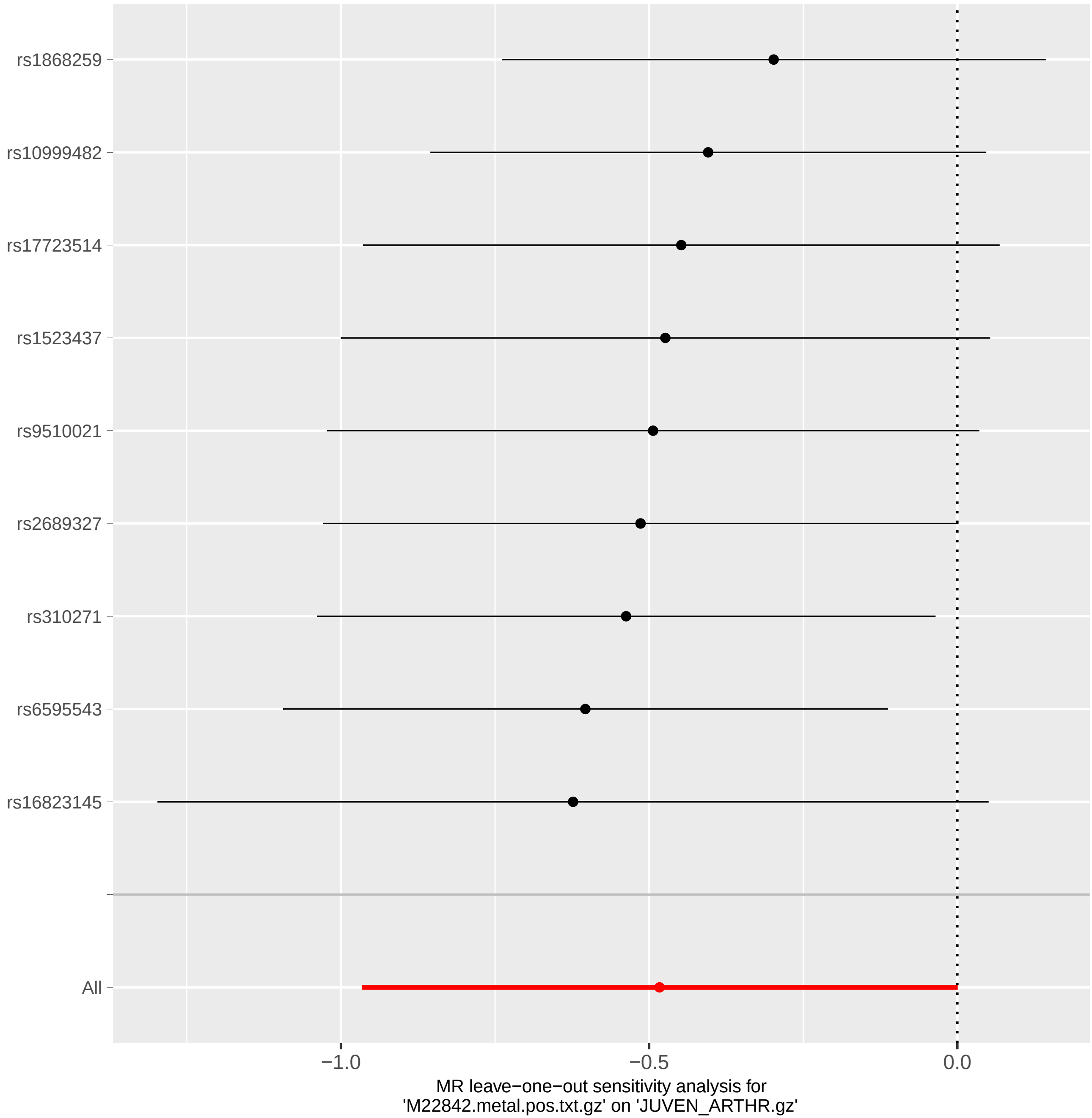

rs1868259
rs10999482
rs17723514
rs1523437
rs9510021
rs2689327
rs310271
rs6595543
rs16823145
All
−1.0
−0.5
0.0
MR leave−one−out sensitivity analysis for
'M22842.metal.pos.txt.gz' on 'JUVEN_ARTHR.gz'

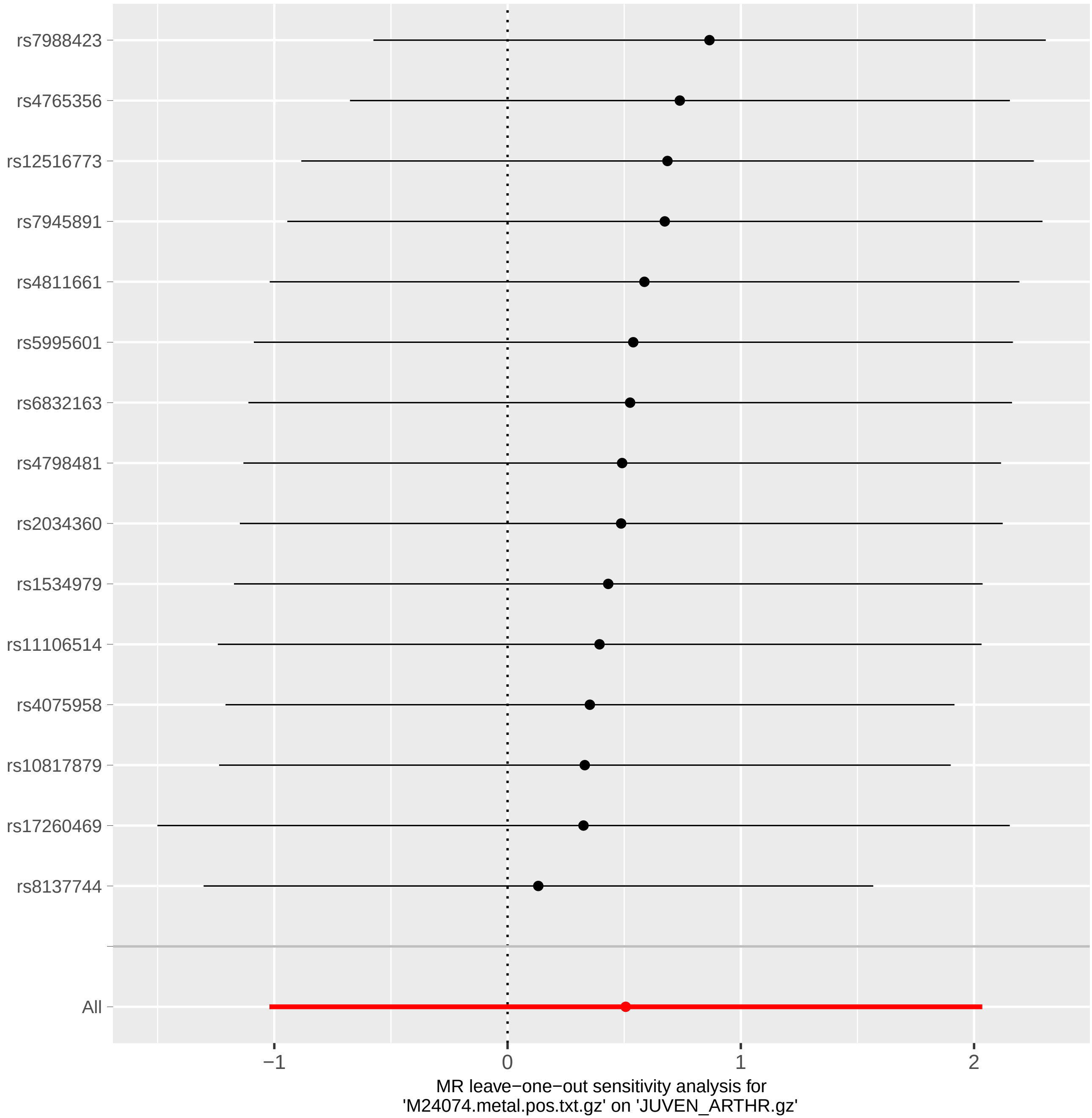

rs7988423
rs4765356
rs12516773
rs7945891
rs4811661
rs5995601
rs6832163
rs4798481
rs2034360
rs1534979
rs11106514
rs4075958
rs10817879
rs17260469
rs8137744
All
−1
0
1
2
MR leave−one−out sensitivity analysis for
'M24074.metal.pos.txt.gz' on 'JUVEN_ARTHR.gz'

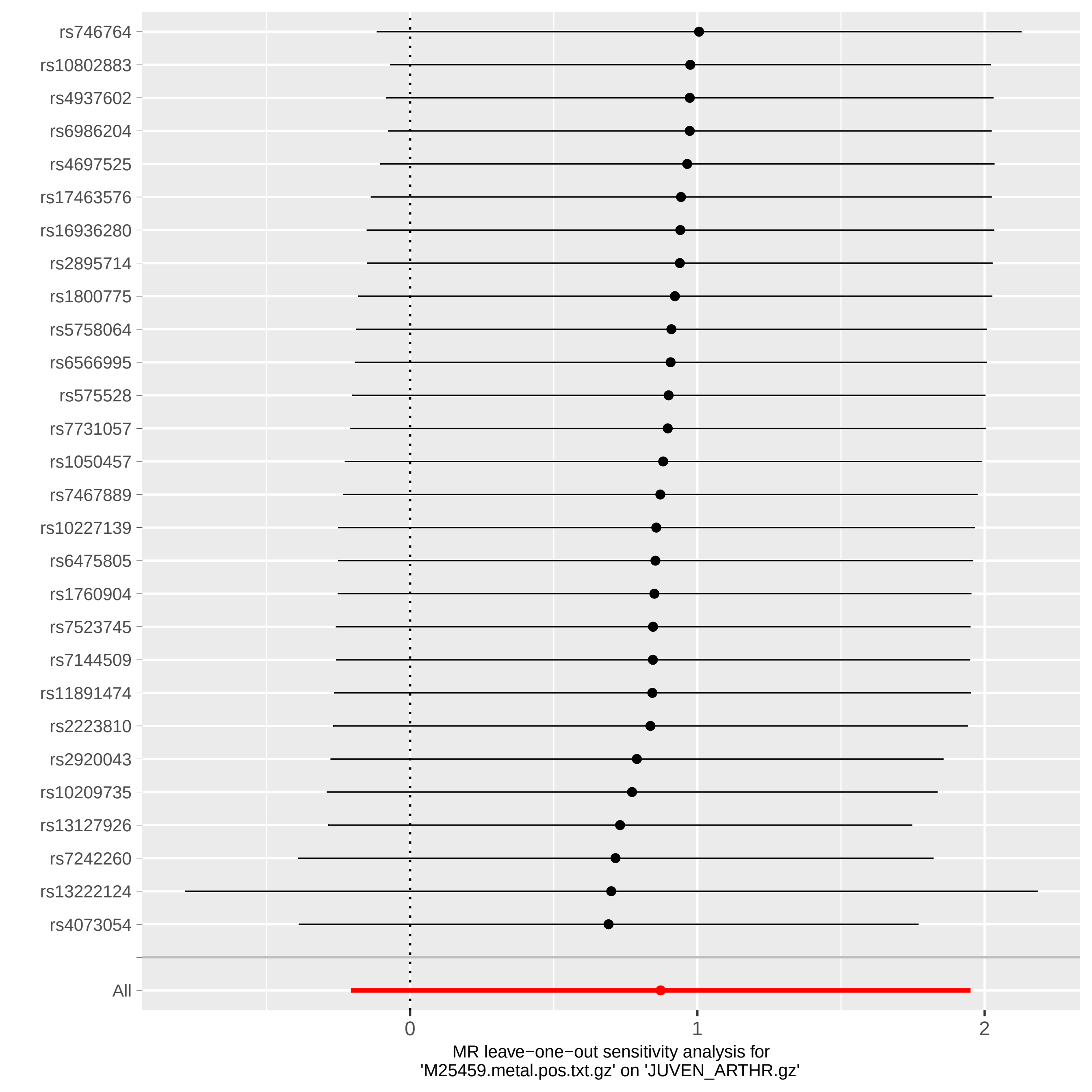

rs746764
rs10802883
rs4937602
rs6986204
rs4697525
rs17463576
rs16936280
rs2895714
rs1800775
rs5758064
rs6566995
rs575528
rs7731057
rs1050457
rs7467889
rs10227139
rs6475805
rs1760904
rs7523745
rs7144509
rs11891474
rs2223810
rs2920043
rs10209735
rs13127926
rs7242260
rs13222124
rs4073054
All
0
1
2
MR leave−one−out sensitivity analysis for
'M25459.metal.pos.txt.gz' on 'JUVEN_ARTHR.gz'

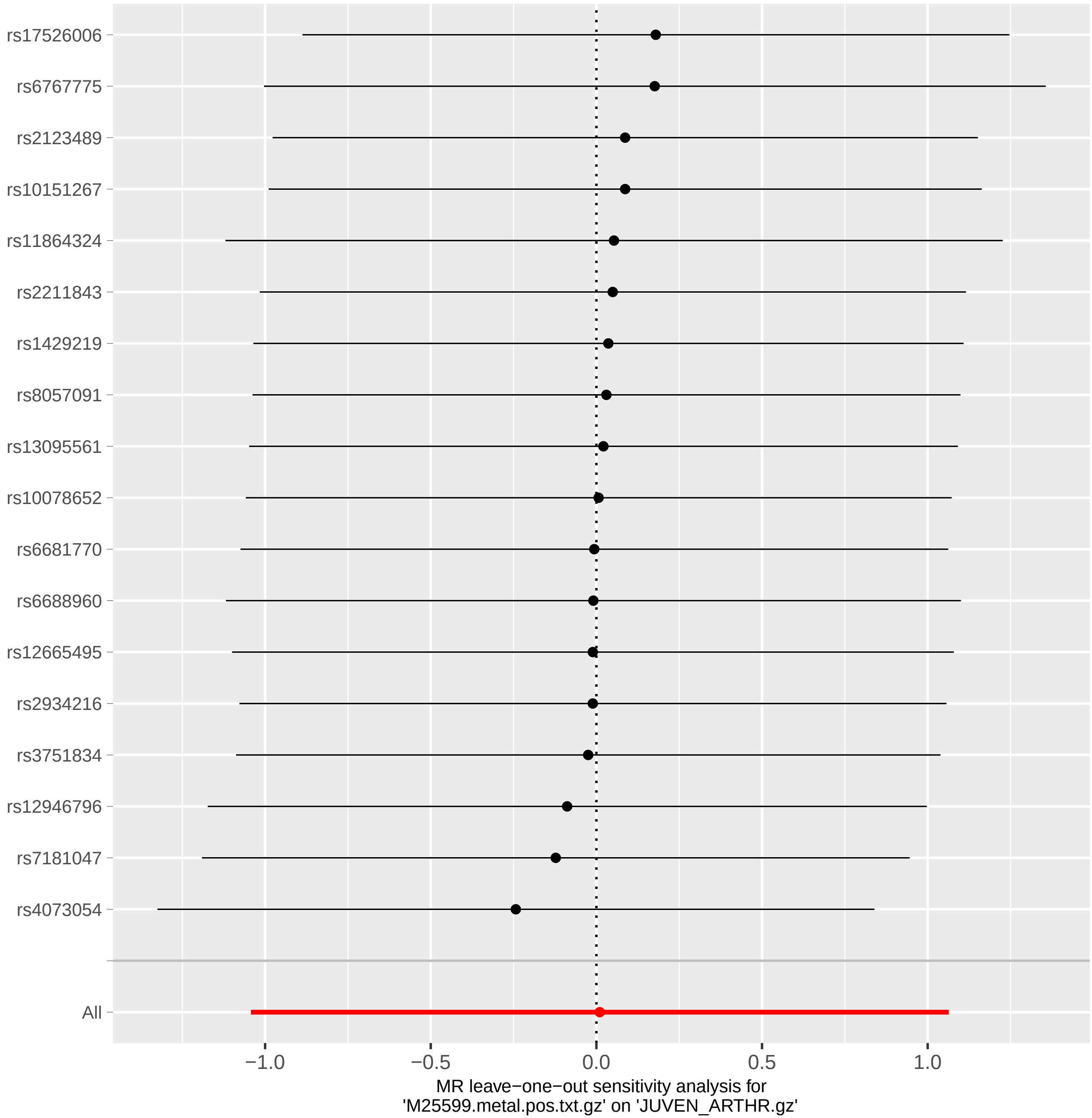

rs17526006
rs6767775
rs2123489
rs10151267
rs11864324
rs2211843
rs1429219
rs8057091
rs13095561
rs10078652
rs6681770
rs6688960
rs12665495
rs2934216
rs3751834
rs12946796
rs7181047
rs4073054
All
−1.0
−0.5
0.0
0.5
1.0
MR leave−one−out sensitivity analysis for
'M25599.metal.pos.txt.gz' on 'JUVEN_ARTHR.gz'

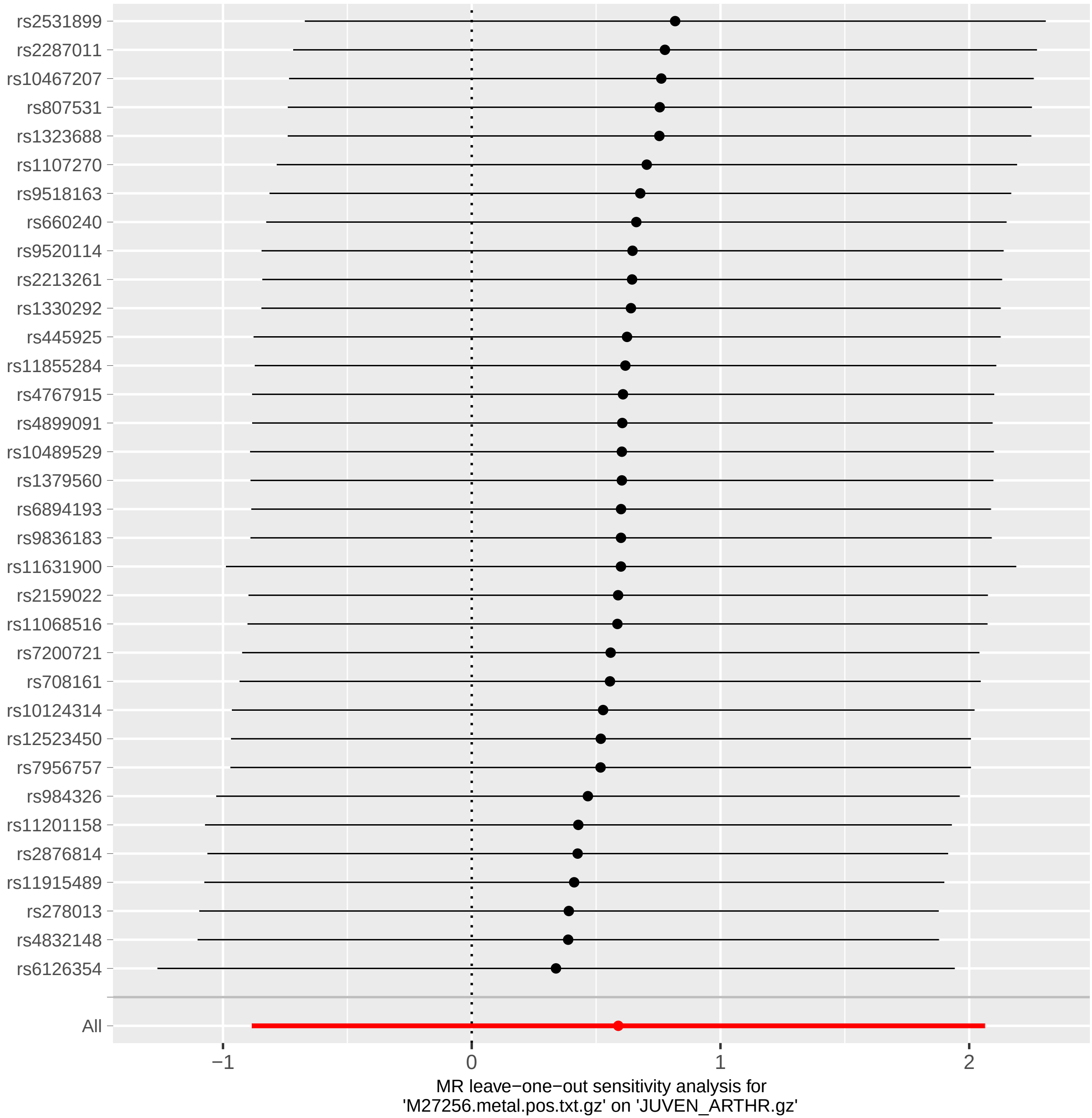

rs2531899
rs2287011
rs10467207
rs807531
rs1323688
rs1107270
rs9518163
rs660240
rs9520114
rs2213261
rs1330292
rs445925
rs11855284
rs4767915
rs4899091
rs10489529
rs1379560
rs6894193
rs9836183
rs11631900
rs2159022
rs11068516
rs7200721
rs708161
rs10124314
rs12523450
rs7956757
rs984326
rs11201158
rs2876814
rs11915489
rs278013
rs4832148
rs6126354
All
−1
0
1
2
MR leave−one−out sensitivity analysis for
'M27256.metal.pos.txt.gz' on 'JUVEN_ARTHR.gz'

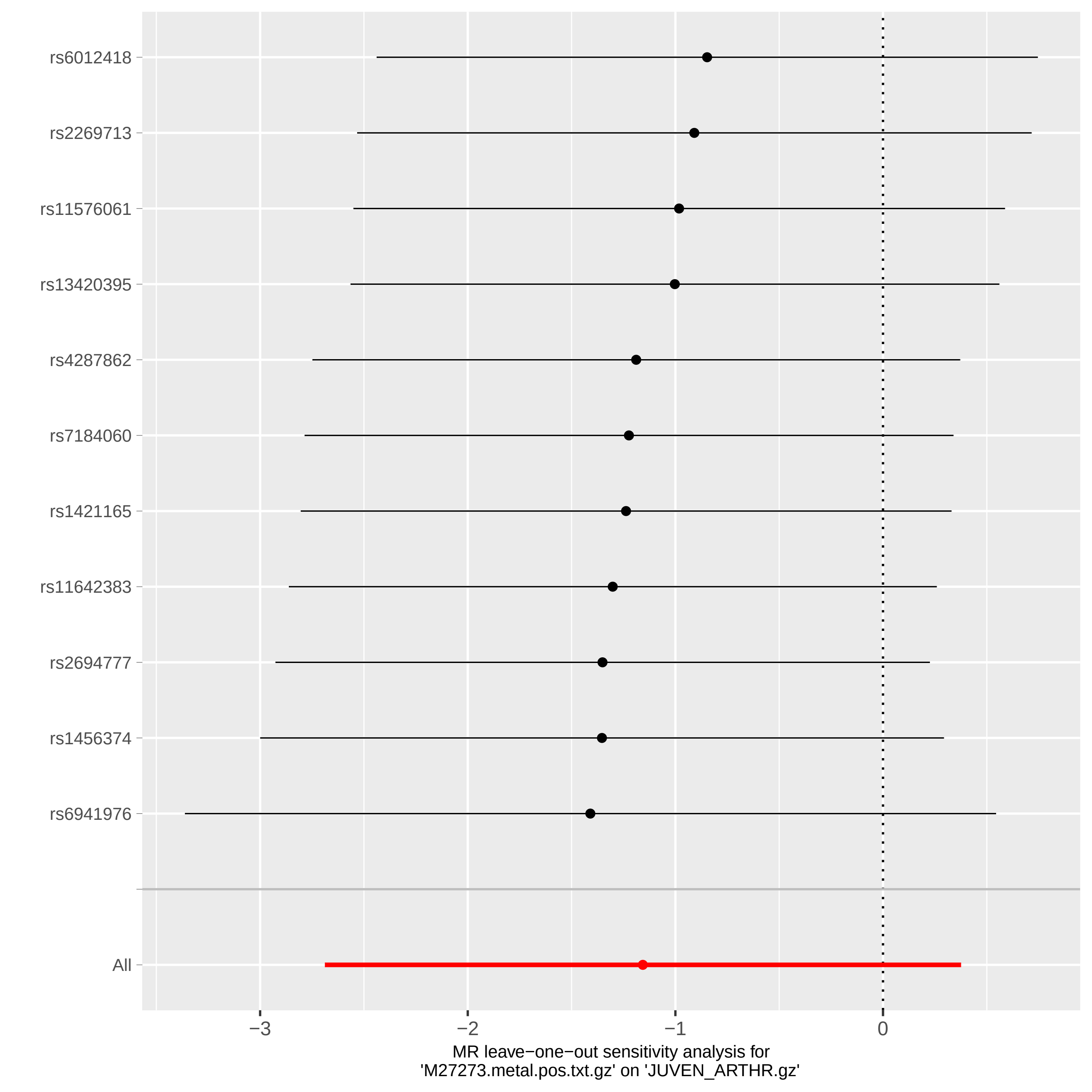
rs6012418
rs2269713
rs11576061
rs13420395
rs4287862
rs7184060
rs1421165
rs11642383
rs2694777
rs1456374
rs6941976
All
−3
−2
−1
0
MR leave−one−out sensitivity analysis for
'M27273.metal.pos.txt.gz' on 'JUVEN_ARTHR.gz'

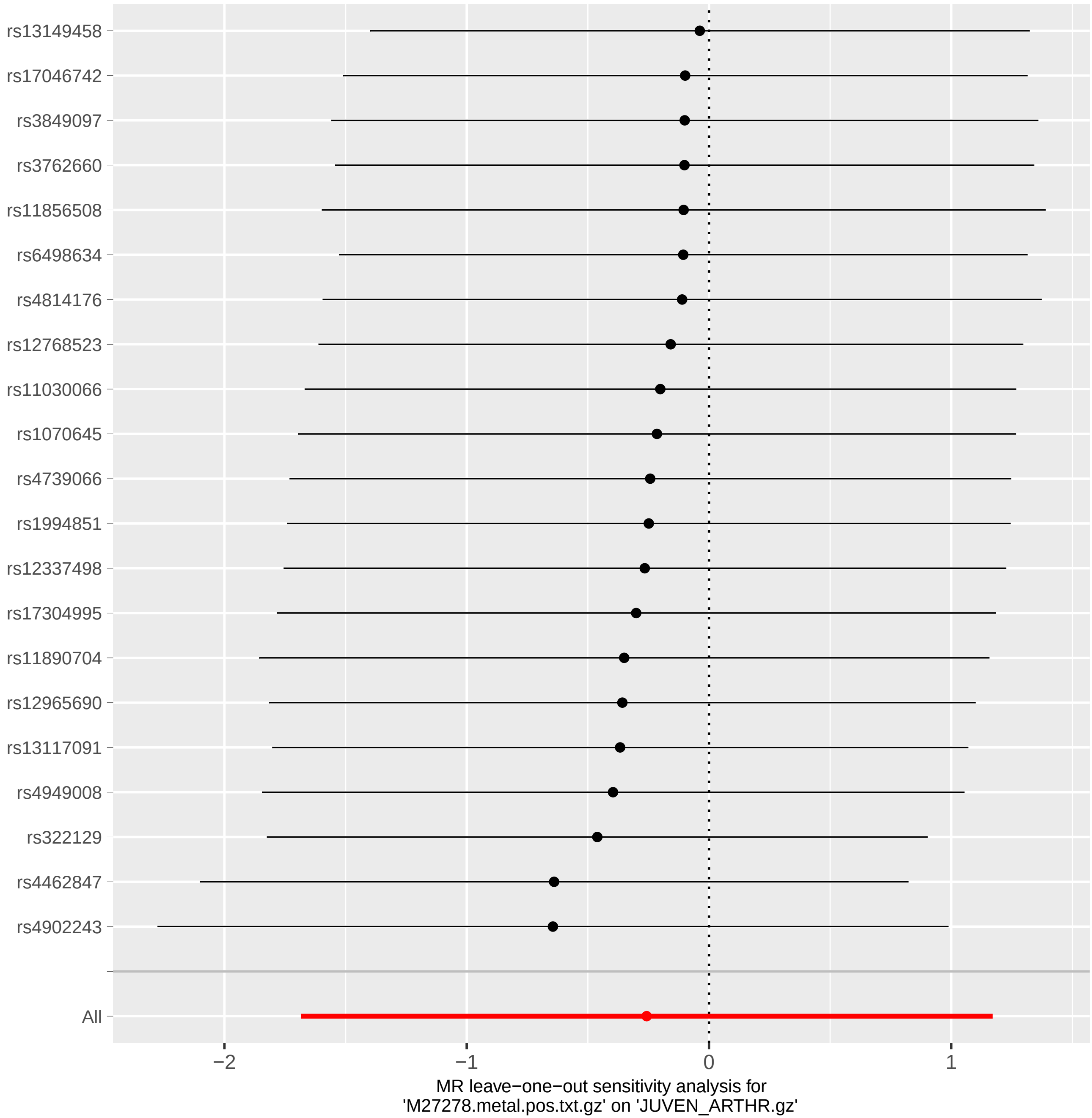
rs13149458
rs17046742
rs3849097
rs3762660
rs11856508
rs6498634
rs4814176
rs12768523
rs11030066
rs1070645
rs4739066
rs1994851
rs12337498
rs17304995
rs11890704
rs12965690
rs13117091
rs4949008
rs322129
rs4462847
rs4902243
All
−2
−1
0
1
MR leave−one−out sensitivity analysis for
'M27278.metal.pos.txt.gz' on 'JUVEN_ARTHR.gz'

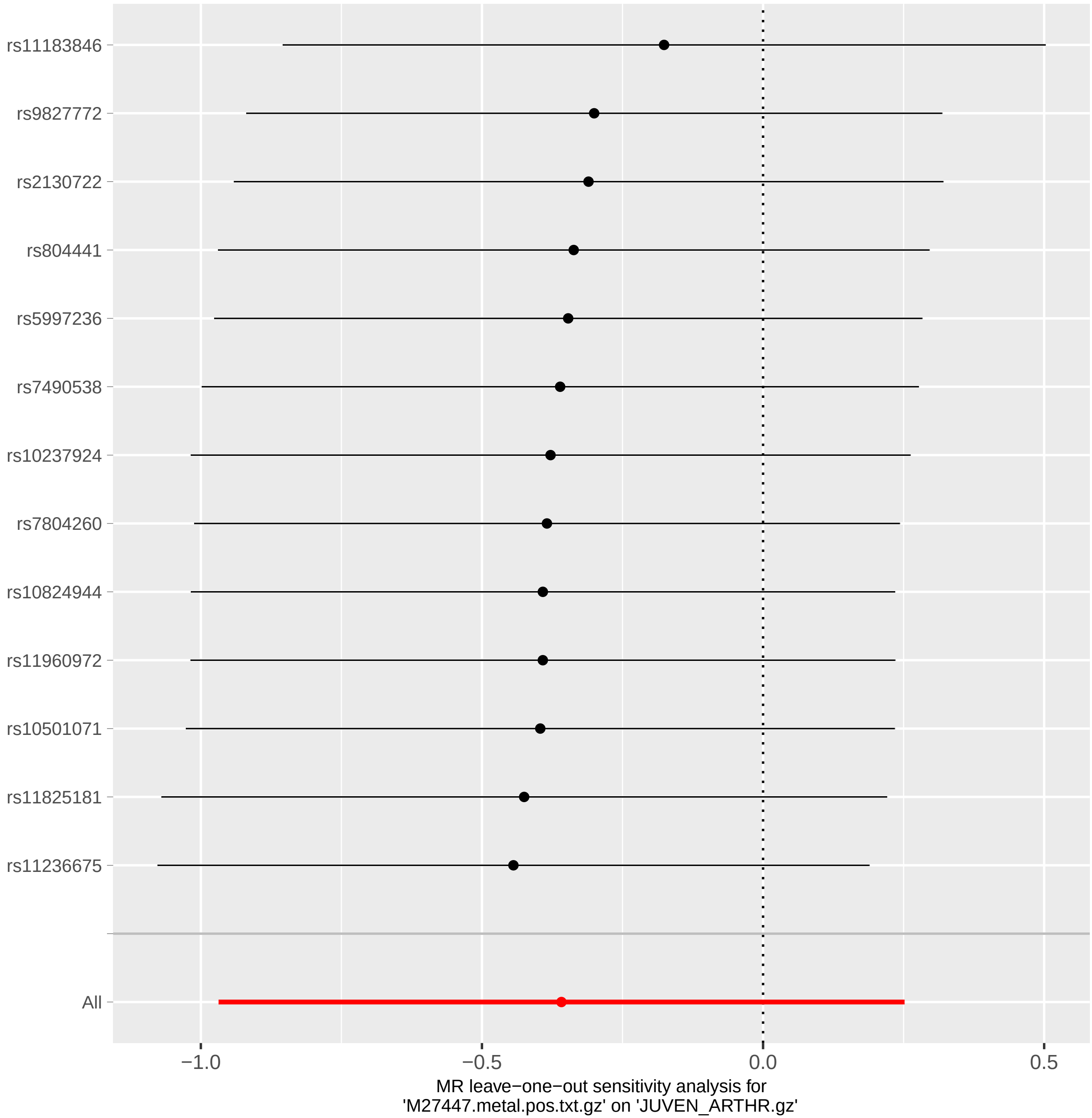
rs11183846
rs9827772
rs2130722
rs804441
rs5997236
rs7490538
rs10237924
rs7804260
rs10824944
rs11960972
rs10501071
rs11825181
rs11236675
All
−1.0
−0.5
0.0
0.5
MR leave−one−out sensitivity analysis for
'M27447.metal.pos.txt.gz' on 'JUVEN_ARTHR.gz'

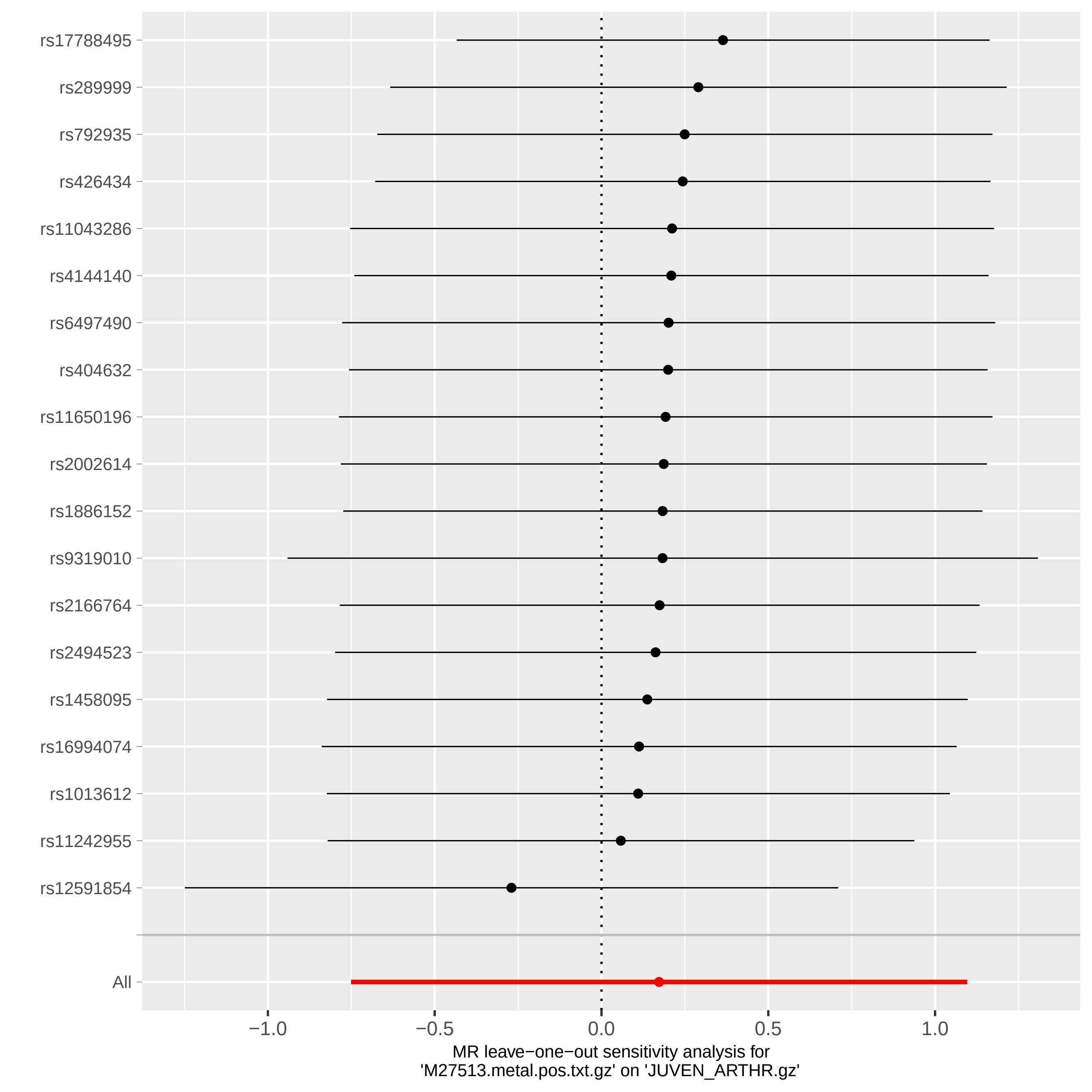

rs17788495
rs289999
rs792935
rs426434
rs11043286
rs4144140
rs6497490
rs404632
rs11650196
rs2002614
rs1886152
rs9319010
rs2166764
rs2494523
rs1458095
rs16994074
rs1013612
rs11242955
rs12591854
All
−1.0
−0.5
0.0
0.5
1.0
MR leave−one−out sensitivity analysis for
'M27513.metal.pos.txt.gz' on 'JUVEN_ARTHR.gz'

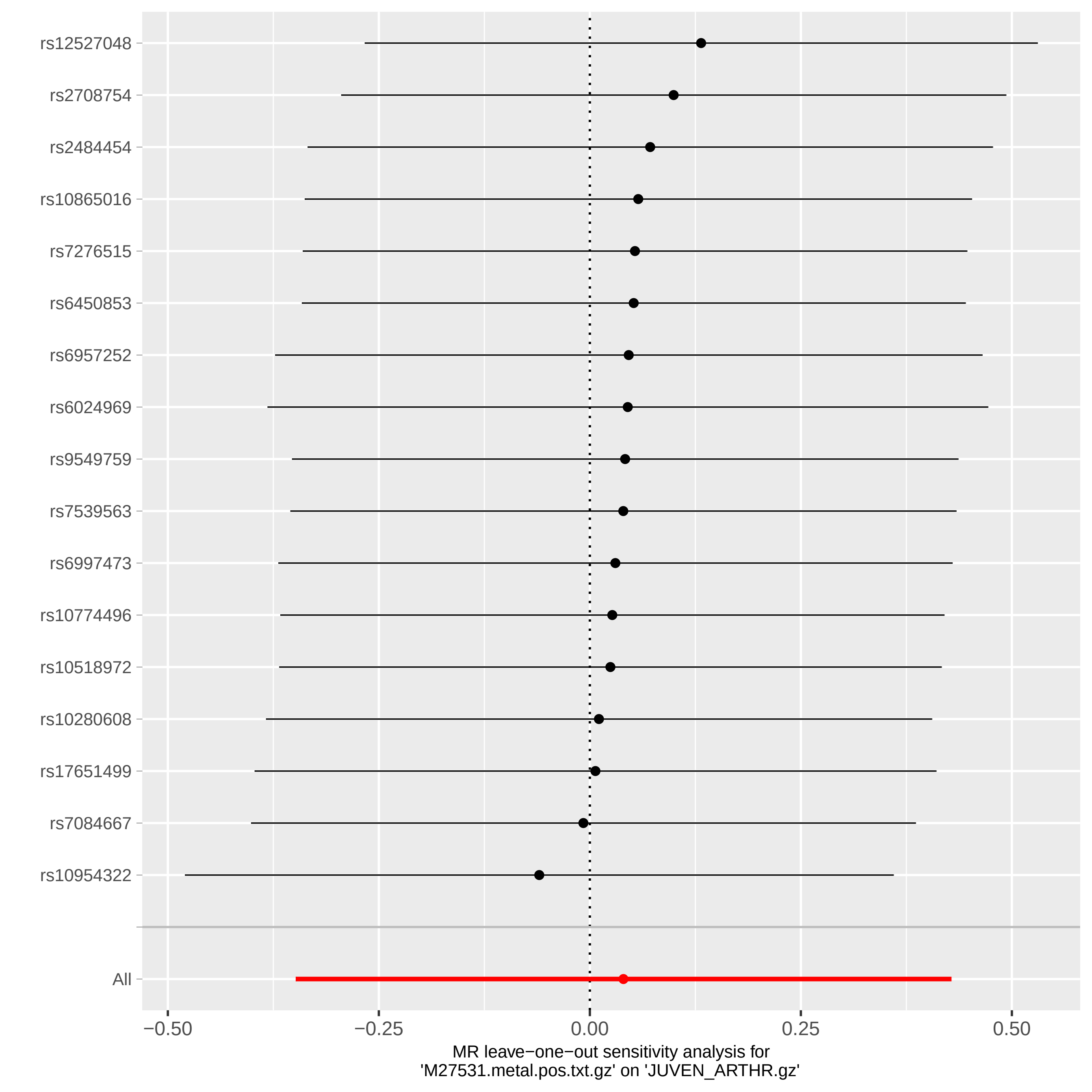

rs12527048
rs2708754
rs2484454
rs10865016
rs7276515
rs6450853
rs6957252
rs6024969
rs9549759
rs7539563
rs6997473
rs10774496
rs10518972
rs10280608
rs17651499
rs7084667
rs10954322
All
−0.50
−0.25
0.00
0.25
0.50
MR leave−one−out sensitivity analysis for
'M27531.metal.pos.txt.gz' on 'JUVEN_ARTHR.gz'

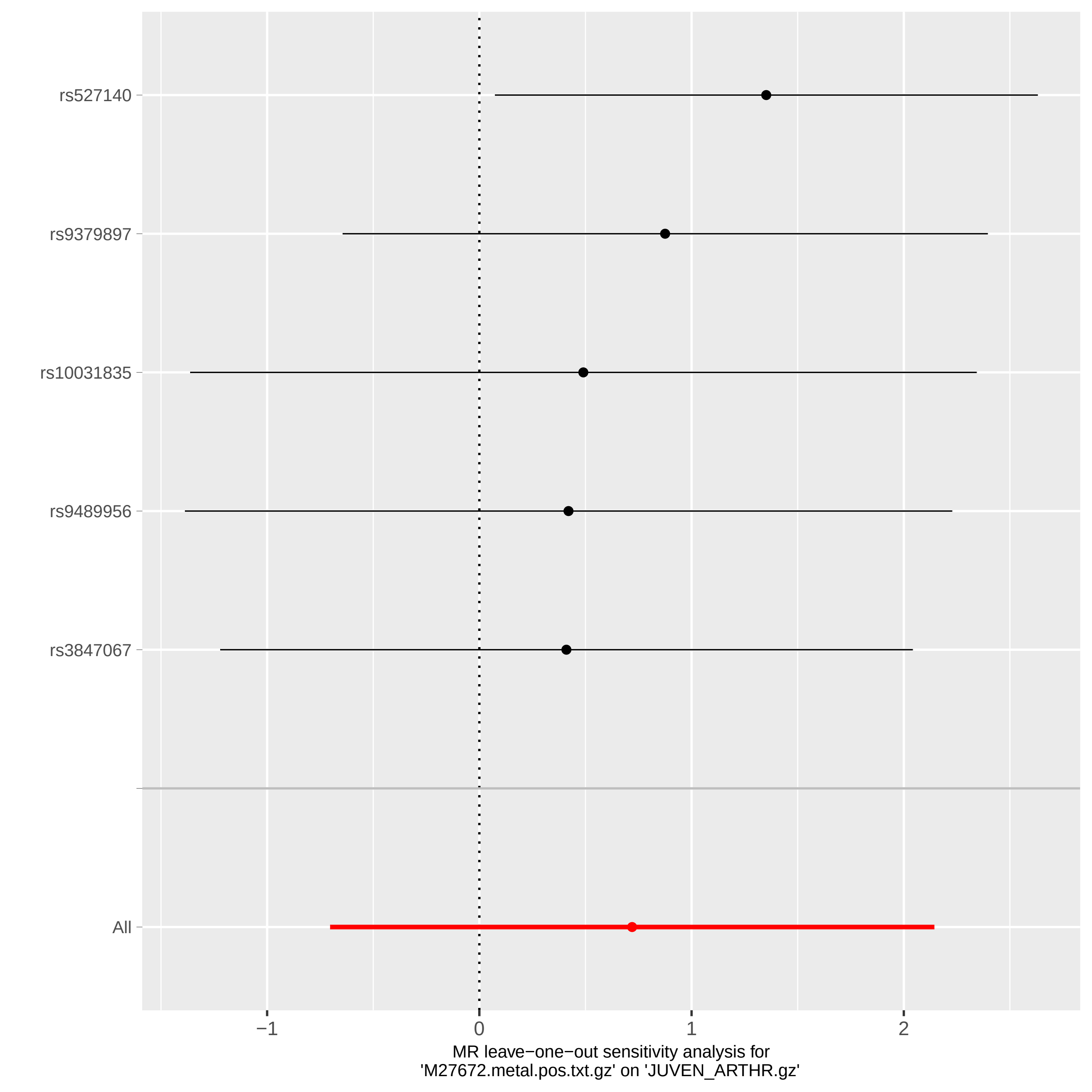
rs527140
rs9379897
rs10031835
rs9489956
rs3847067
All
−1
0
1
2
MR leave−one−out sensitivity analysis for
'M27672.metal.pos.txt.gz' on 'JUVEN_ARTHR.gz'

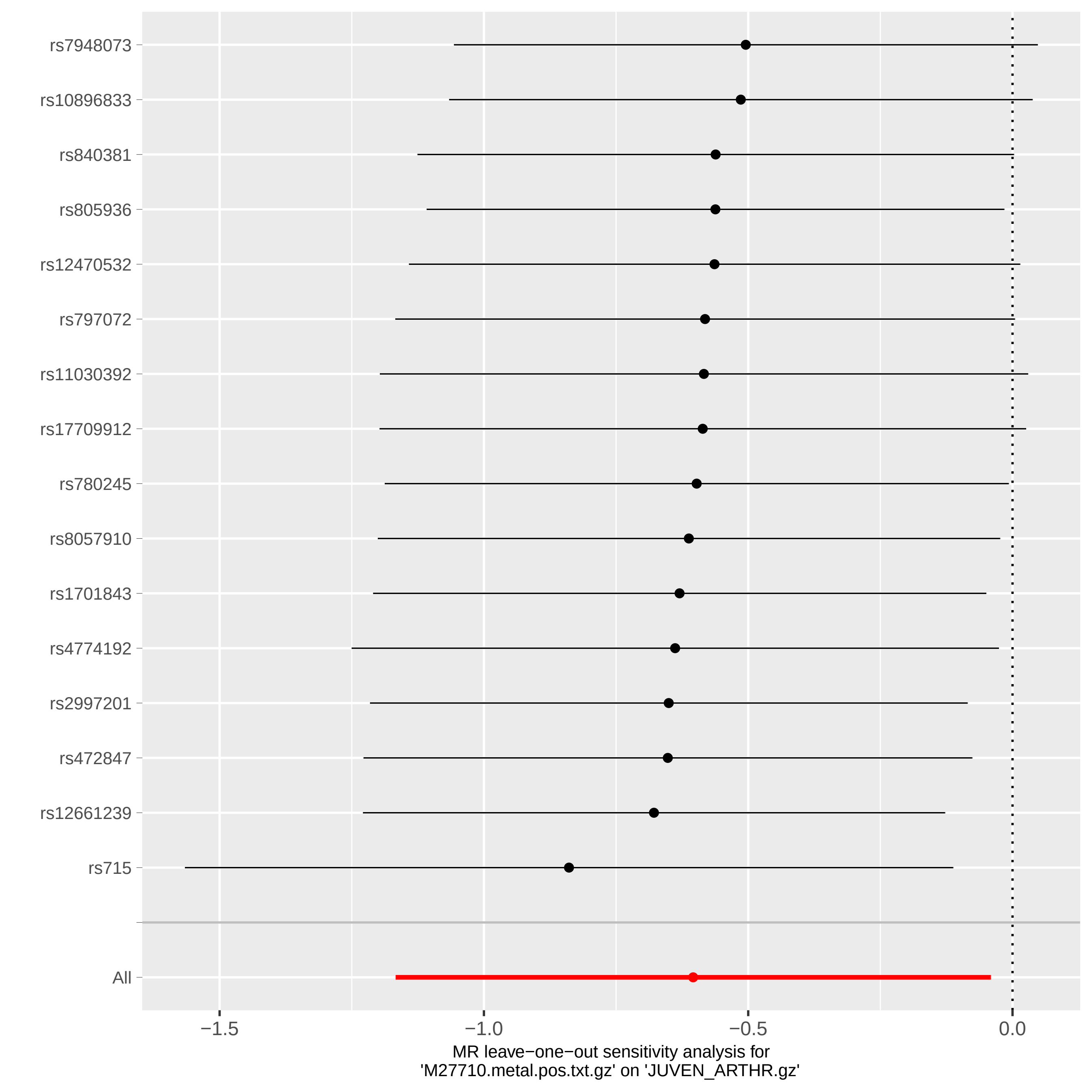
rs7948073
rs10896833
rs840381
rs805936
rs12470532
rs797072
rs11030392
rs17709912
rs780245
rs8057910
rs1701843
rs4774192
rs2997201
rs472847
rs12661239
rs715
All
−1.5
−1.0
−0.5
0.0
MR leave−one−out sensitivity analysis for
'M27710.metal.pos.txt.gz' on 'JUVEN_ARTHR.gz'

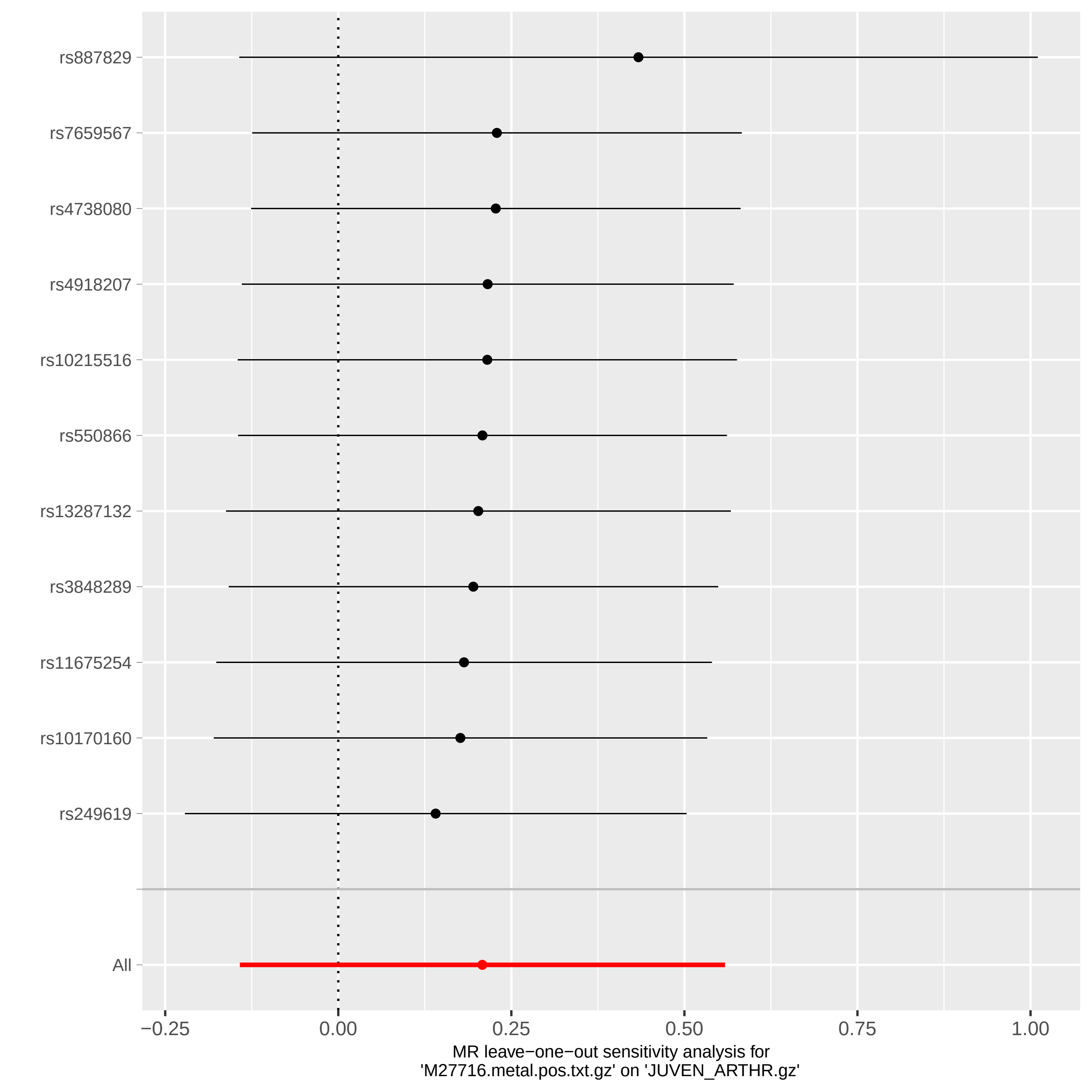
rs887829
rs7659567
rs4738080
rs4918207
rs10215516
rs550866
rs13287132
rs3848289
rs11675254
rs10170160
rs249619
All
−0.25
0.00
0.25
0.50
0.75
1.00
MR leave−one−out sensitivity analysis for
'M27716.metal.pos.txt.gz' on 'JUVEN_ARTHR.gz'

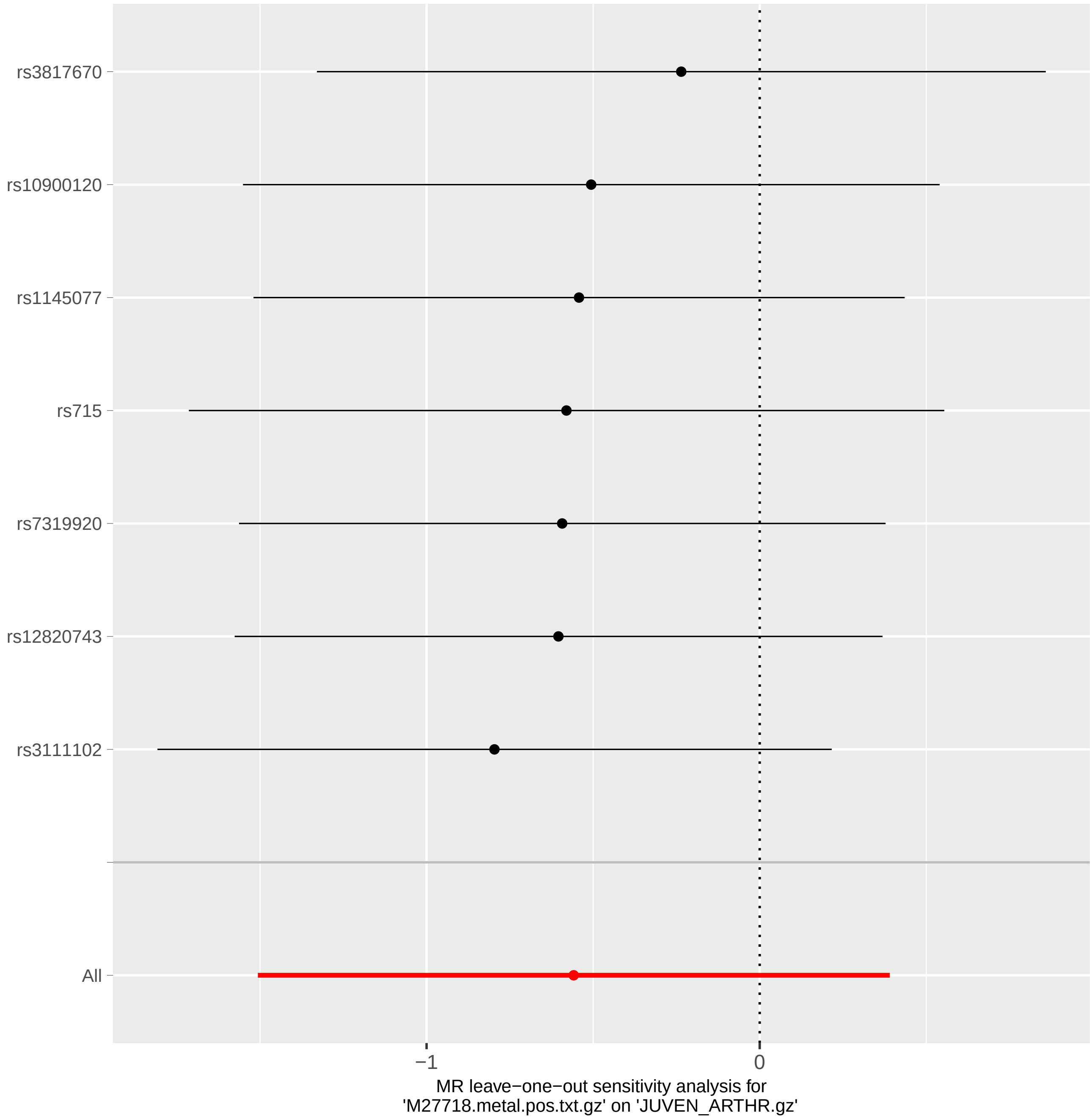
rs3817670
rs10900120
rs1145077
rs715
rs7319920
rs12820743
rs3111102
All
−1
0
MR leave−one−out sensitivity analysis for
'M27718.metal.pos.txt.gz' on 'JUVEN_ARTHR.gz'

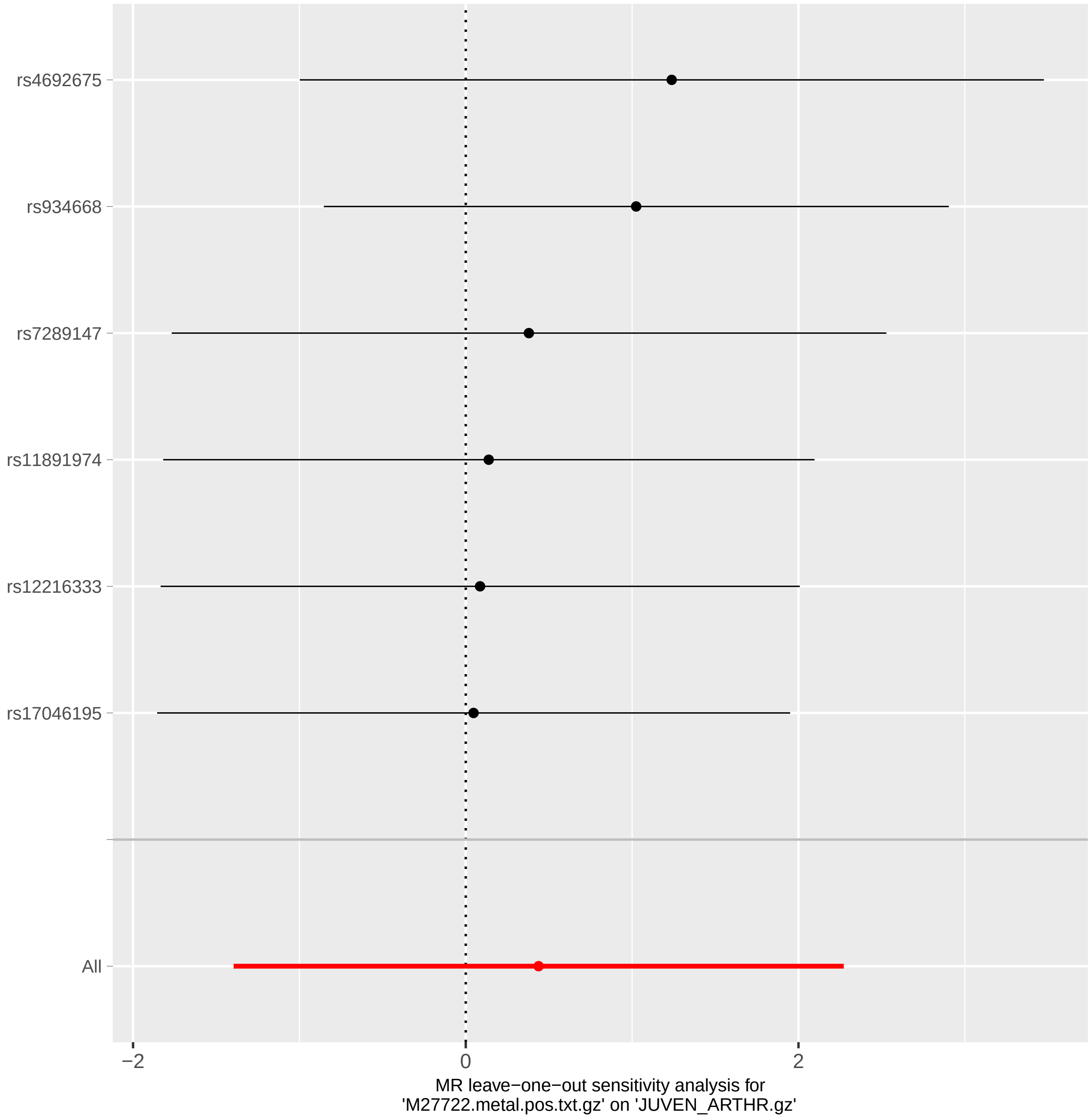
rs4692675
rs934668
rs7289147
rs11891974
rs12216333
rs17046195
All
−2
0
2
MR leave−one−out sensitivity analysis for
'M27722.metal.pos.txt.gz' on 'JUVEN_ARTHR.gz'

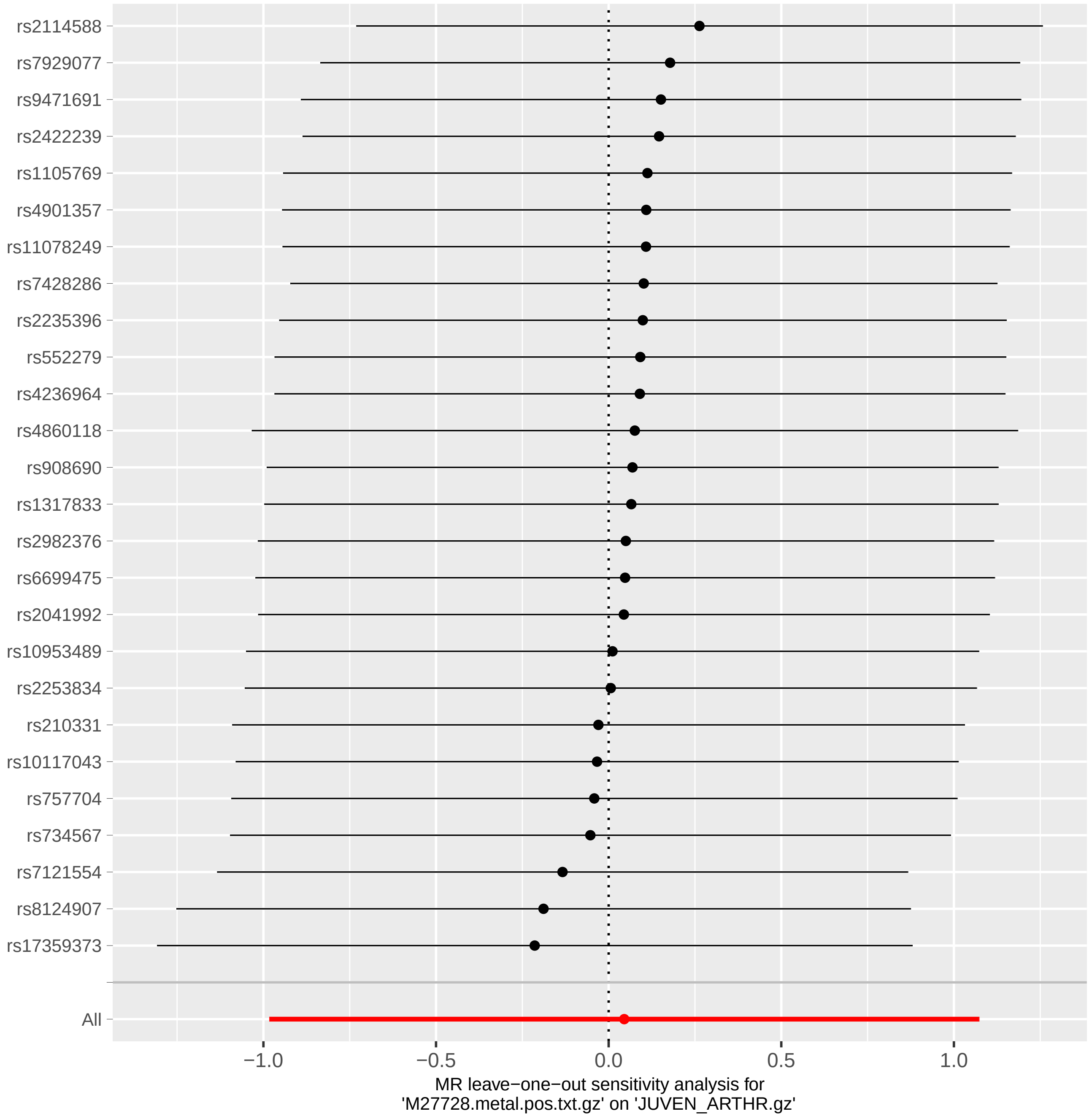
rs2114588
rs7929077
rs9471691
rs2422239
rs1105769
rs4901357
rs11078249
rs7428286
rs2235396
rs552279
rs4236964
rs4860118
rs908690
rs1317833
rs2982376
rs6699475
rs2041992
rs10953489
rs2253834
rs210331
rs10117043
rs757704
rs734567
rs7121554
rs8124907
rs17359373
All
−1.0
−0.5
0.0
0.5
1.0
MR leave−one−out sensitivity analysis for
'M27728.metal.pos.txt.gz' on 'JUVEN_ARTHR.gz'

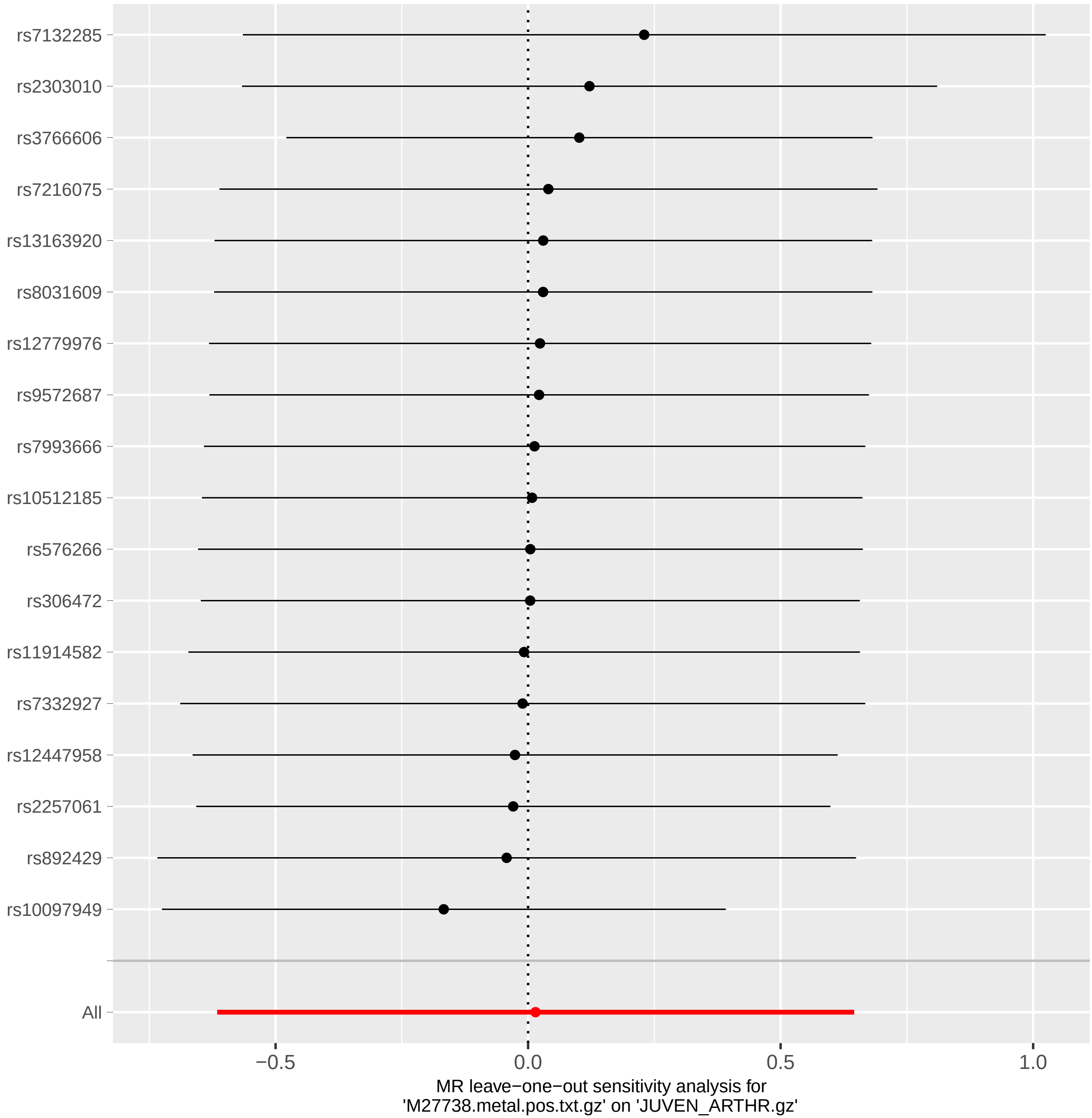

rs7132285
rs2303010
rs3766606
rs7216075
rs13163920
rs8031609
rs12779976
rs9572687
rs7993666
rs10512185
rs576266
rs306472
rs11914582
rs7332927
rs12447958
rs2257061
rs892429
rs10097949
All
−0.5
0.0
0.5
1.0
MR leave−one−out sensitivity analysis for
'M27738.metal.pos.txt.gz' on 'JUVEN_ARTHR.gz'

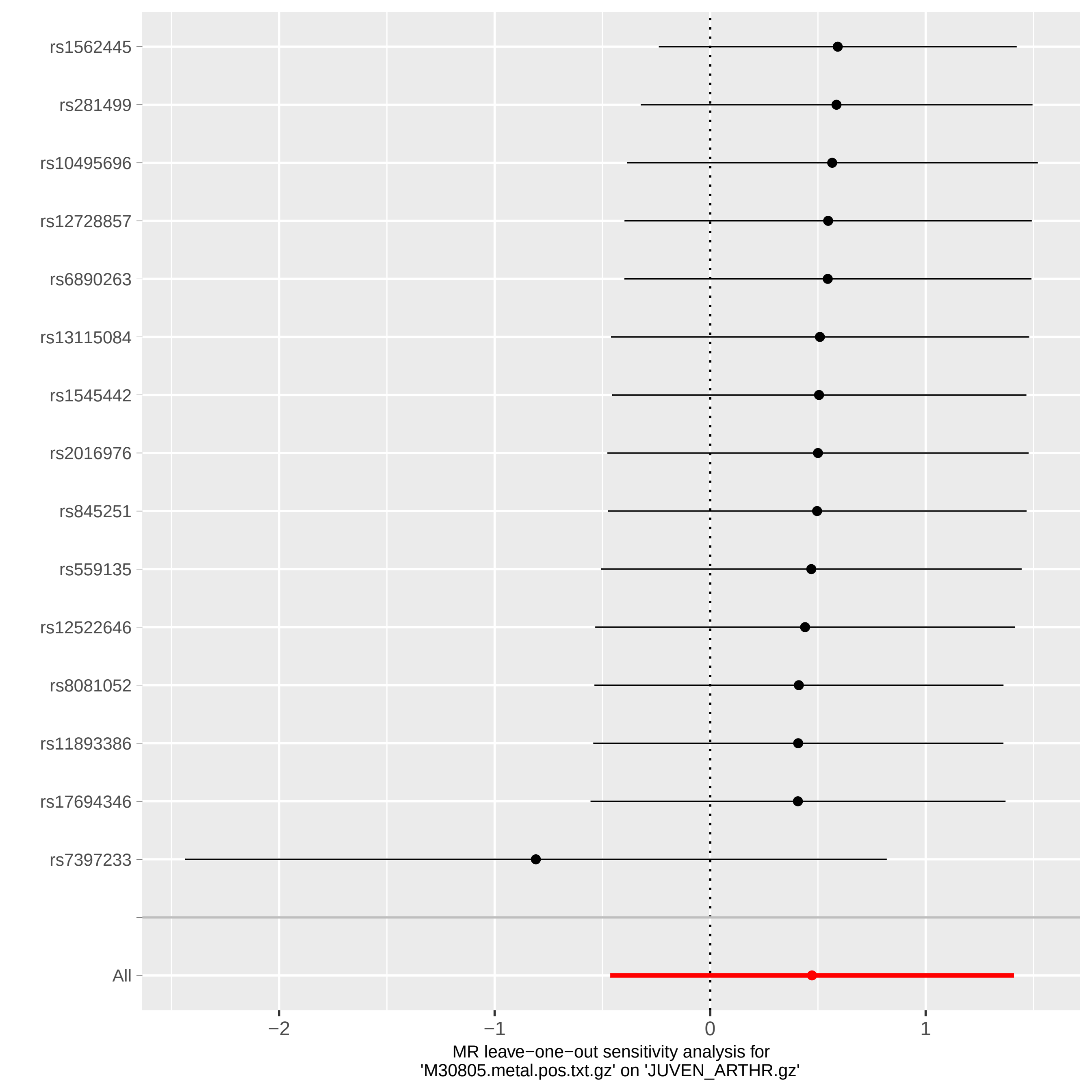
rs1562445
rs281499
rs10495696
rs12728857
rs6890263
rs13115084
rs1545442
rs2016976
rs845251
rs559135
rs12522646
rs8081052
rs11893386
rs17694346
rs7397233
All
−2
−1
0
1
MR leave−one−out sensitivity analysis for
'M30805.metal.pos.txt.gz' on 'JUVEN_ARTHR.gz'

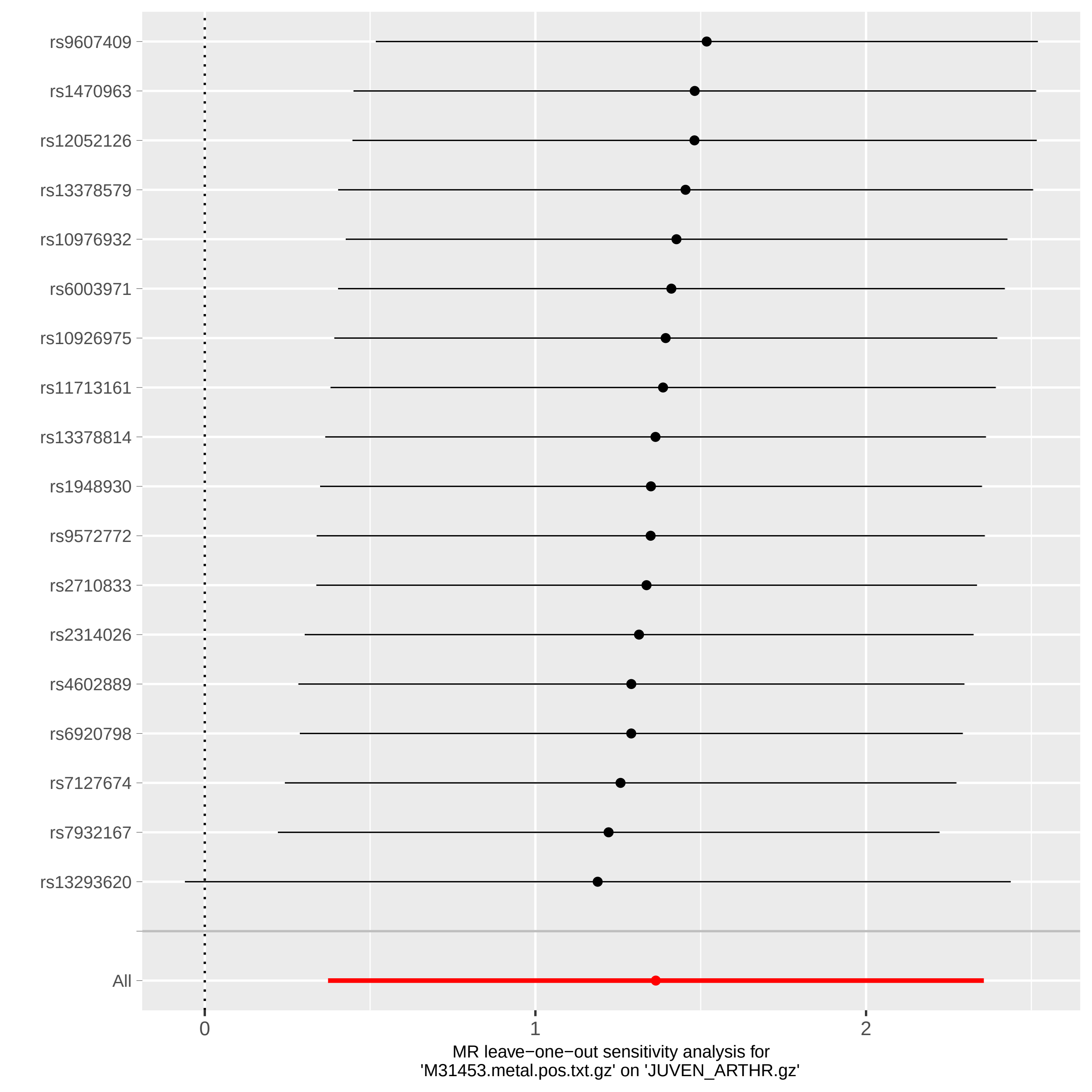
rs9607409
rs1470963
rs12052126
rs13378579
rs10976932
rs6003971
rs10926975
rs11713161
rs13378814
rs1948930
rs9572772
rs2710833
rs2314026
rs4602889
rs6920798
rs7127674
rs7932167
rs13293620
All
0
1
2
MR leave−one−out sensitivity analysis for
'M31453.metal.pos.txt.gz' on 'JUVEN_ARTHR.gz'

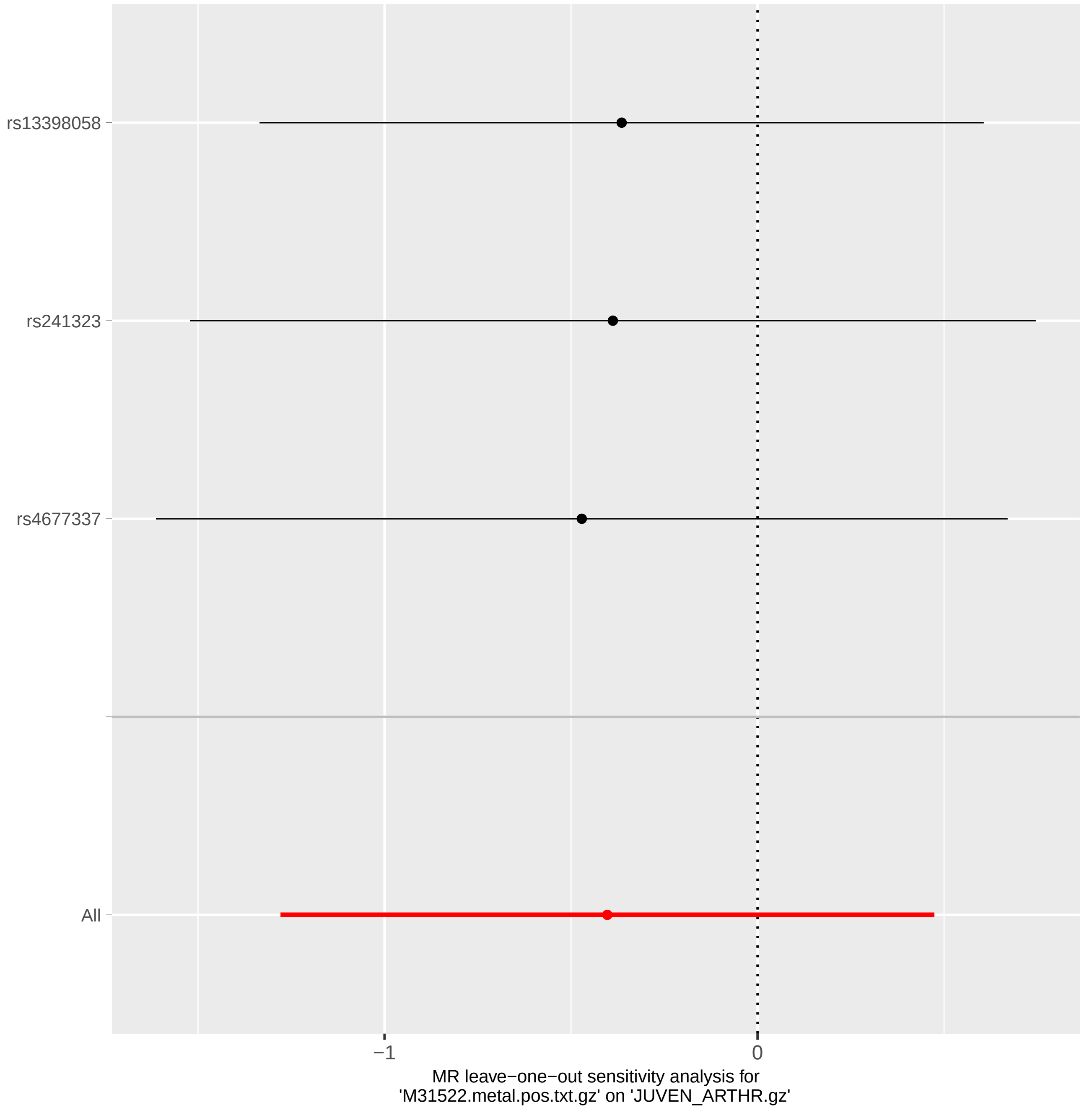
rs13398058
rs241323
rs4677337
All
−1
0
MR leave−one−out sensitivity analysis for
'M31522.metal.pos.txt.gz' on 'JUVEN_ARTHR.gz'

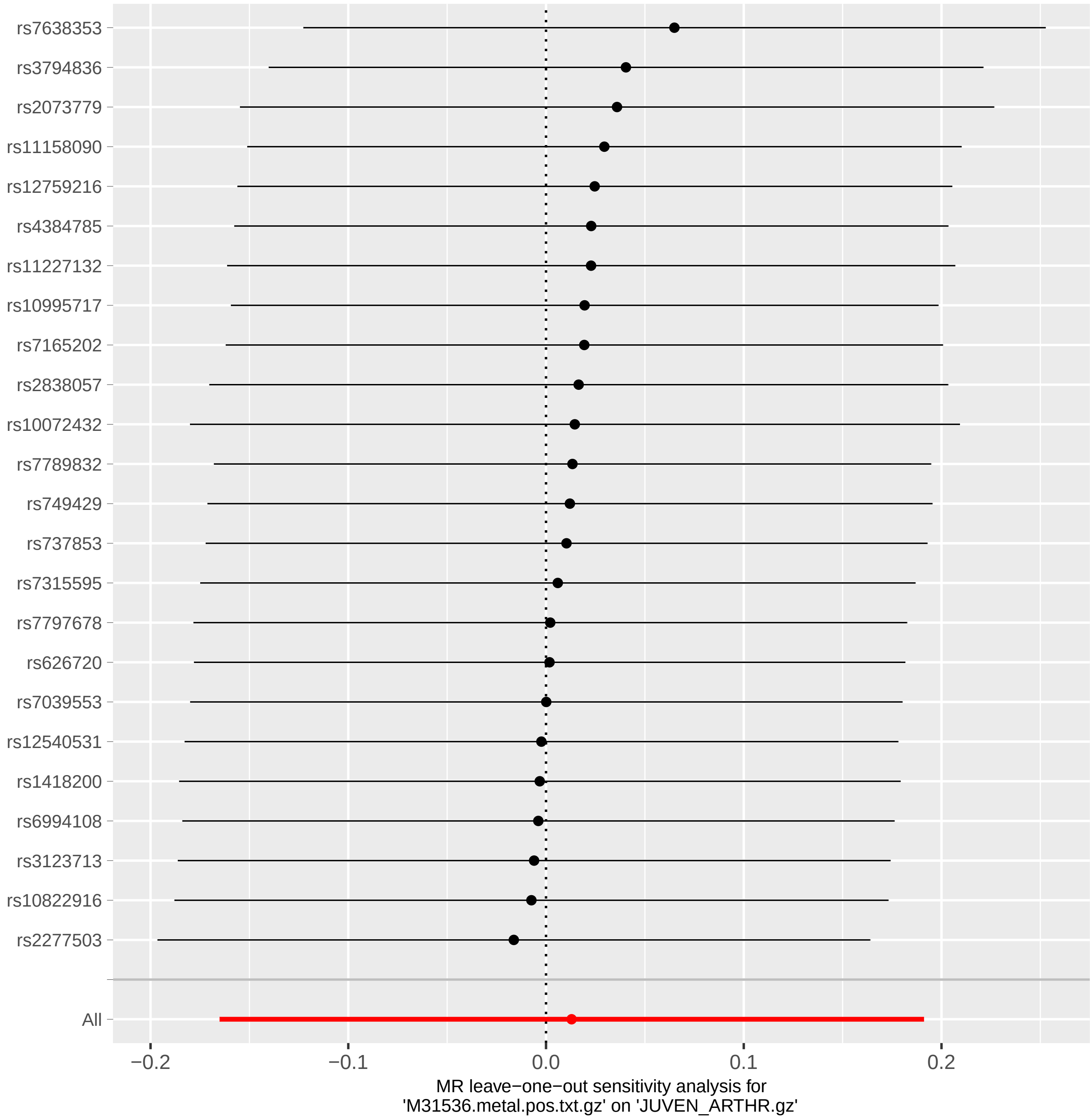

rs7638353
rs3794836
rs2073779
rs11158090
rs12759216
rs4384785
rs11227132
rs10995717
rs7165202
rs2838057
rs10072432
rs7789832
rs749429
rs737853
rs7315595
rs7797678
rs626720
rs7039553
rs12540531
rs1418200
rs6994108
rs3123713
rs10822916
rs2277503
All
−0.2
−0.1
0.0
0.1
0.2
MR leave−one−out sensitivity analysis for
'M31536.metal.pos.txt.gz' on 'JUVEN_ARTHR.gz'

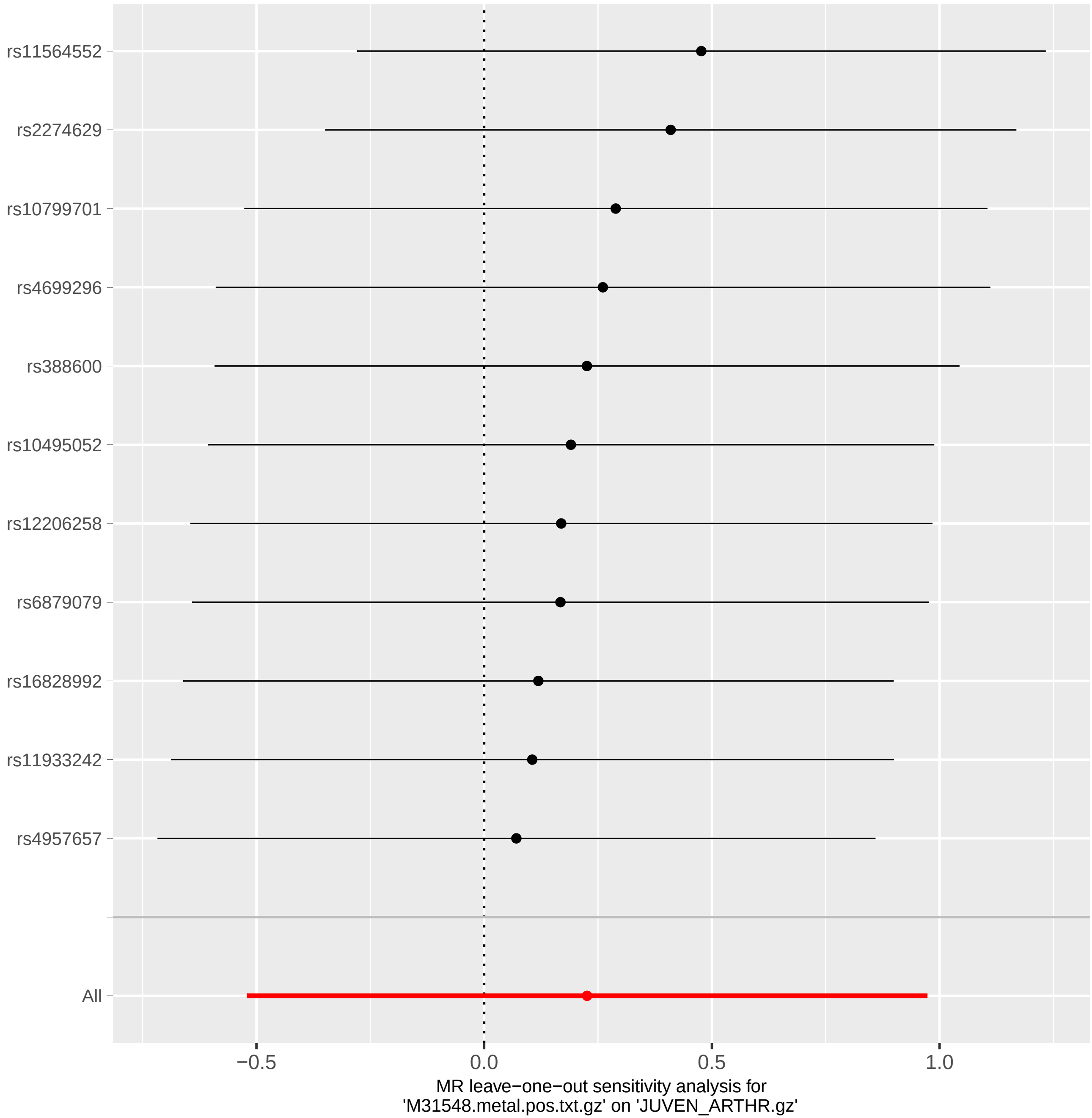
rs11564552
rs2274629
rs10799701
rs4699296
rs388600
rs10495052
rs12206258
rs6879079
rs16828992
rs11933242
rs4957657
All
−0.5
0.0
0.5
1.0
MR leave−one−out sensitivity analysis for
'M31548.metal.pos.txt.gz' on 'JUVEN_ARTHR.gz'

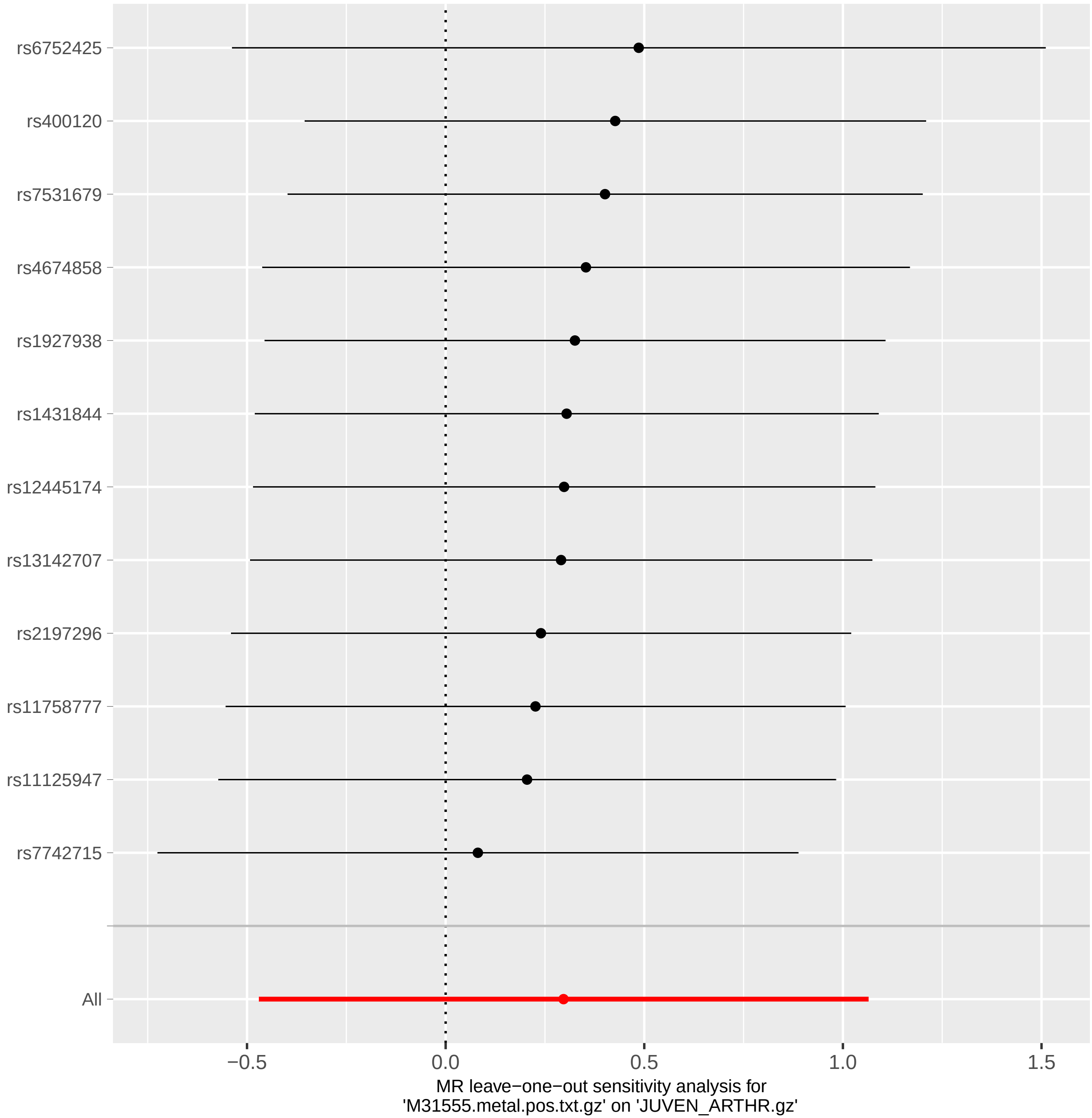

rs6752425
rs400120
rs7531679
rs4674858
rs1927938
rs1431844
rs12445174
rs13142707
rs2197296
rs11758777
rs11125947
rs7742715
All
−0.5
0.0
0.5
1.0
1.5
MR leave−one−out sensitivity analysis for
'M31555.metal.pos.txt.gz' on 'JUVEN_ARTHR.gz'

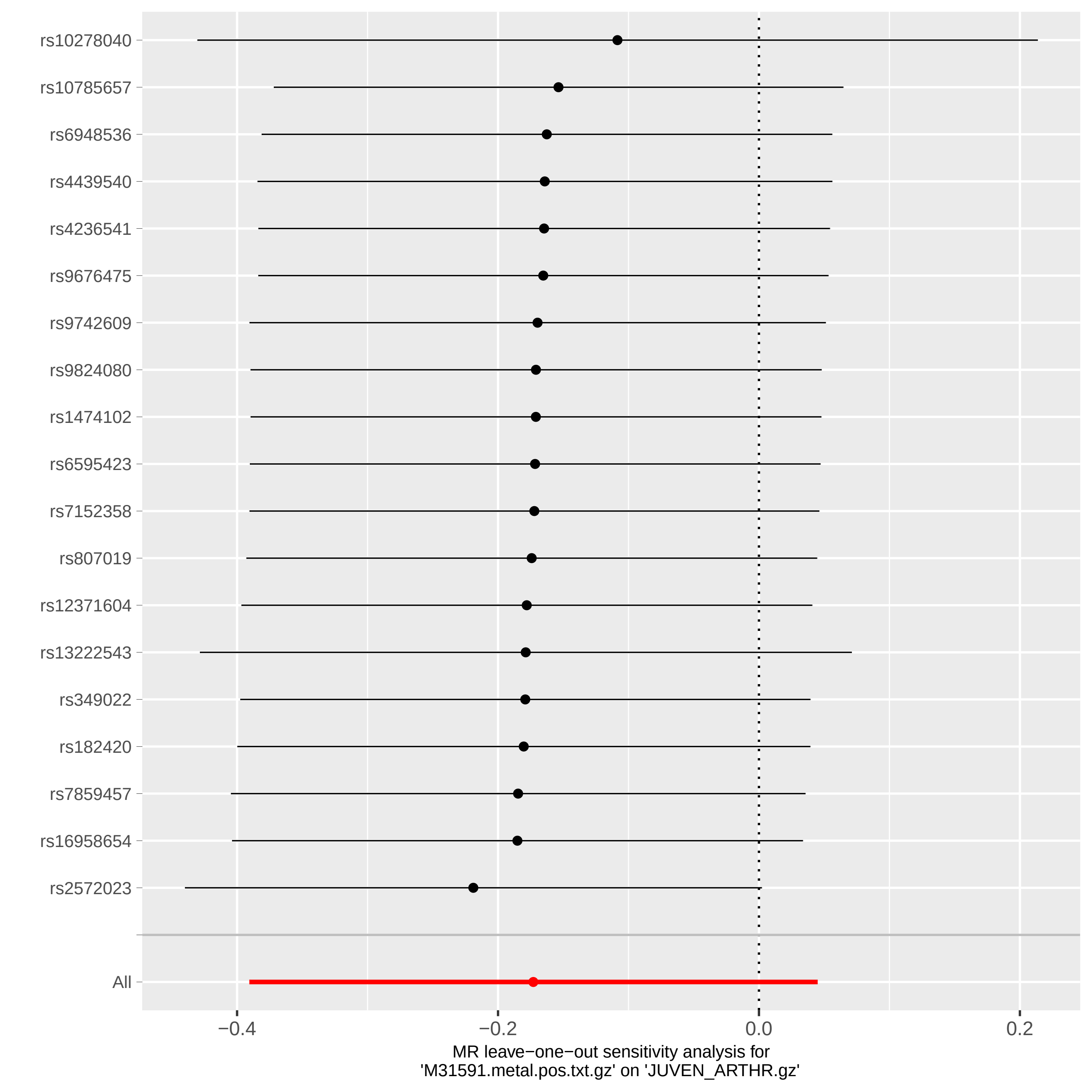

rs10278040
rs10785657
rs6948536
rs4439540
rs4236541
rs9676475
rs9742609
rs9824080
rs1474102
rs6595423
rs7152358
rs807019
rs12371604
rs13222543
rs349022
rs182420
rs7859457
rs16958654
rs2572023
All
−0.4
−0.2
0.0
0.2
MR leave−one−out sensitivity analysis for
'M31591.metal.pos.txt.gz' on 'JUVEN_ARTHR.gz'

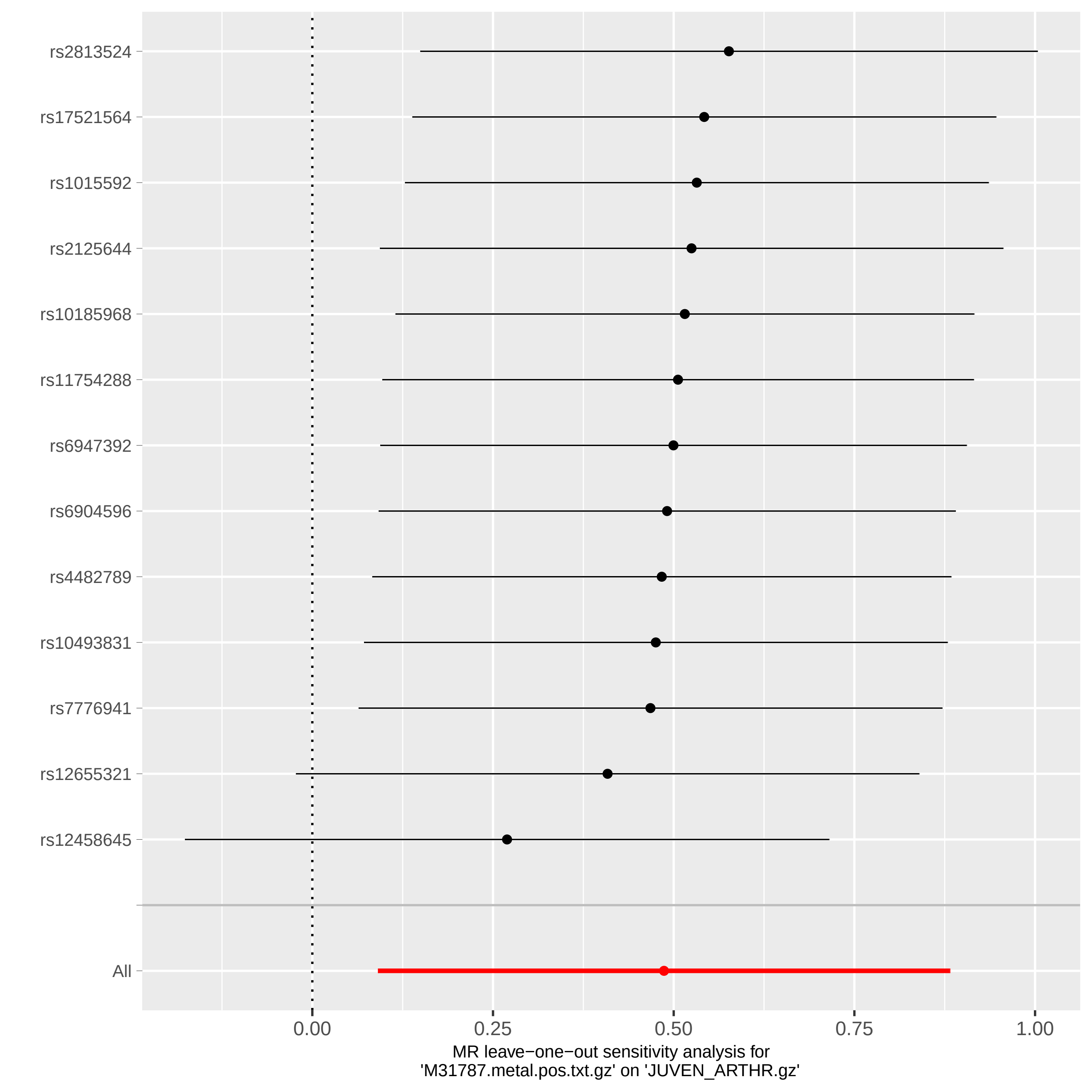
rs2813524
rs17521564
rs1015592
rs2125644
rs10185968
rs11754288
rs6947392
rs6904596
rs4482789
rs10493831
rs7776941
rs12655321
rs12458645
All
0.00
0.25
0.50
0.75
1.00
MR leave−one−out sensitivity analysis for
'M31787.metal.pos.txt.gz' on 'JUVEN_ARTHR.gz'

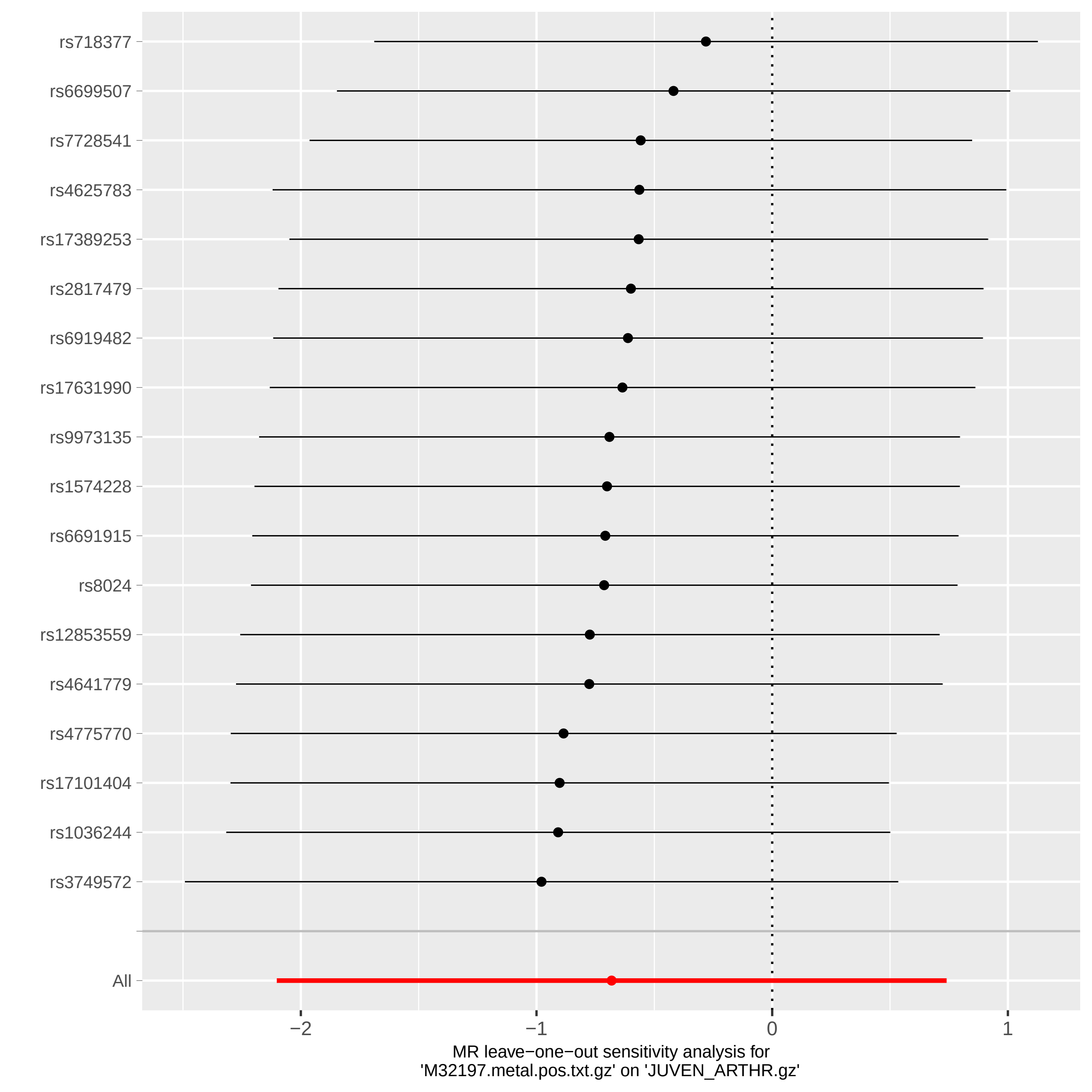
rs718377
rs6699507
rs7728541
rs4625783
rs17389253
rs2817479
rs6919482
rs17631990
rs9973135
rs1574228
rs6691915
rs8024
rs12853559
rs4641779
rs4775770
rs17101404
rs1036244
rs3749572
All
−2
−1
0
1
MR leave−one−out sensitivity analysis for
'M32197.metal.pos.txt.gz' on 'JUVEN_ARTHR.gz'

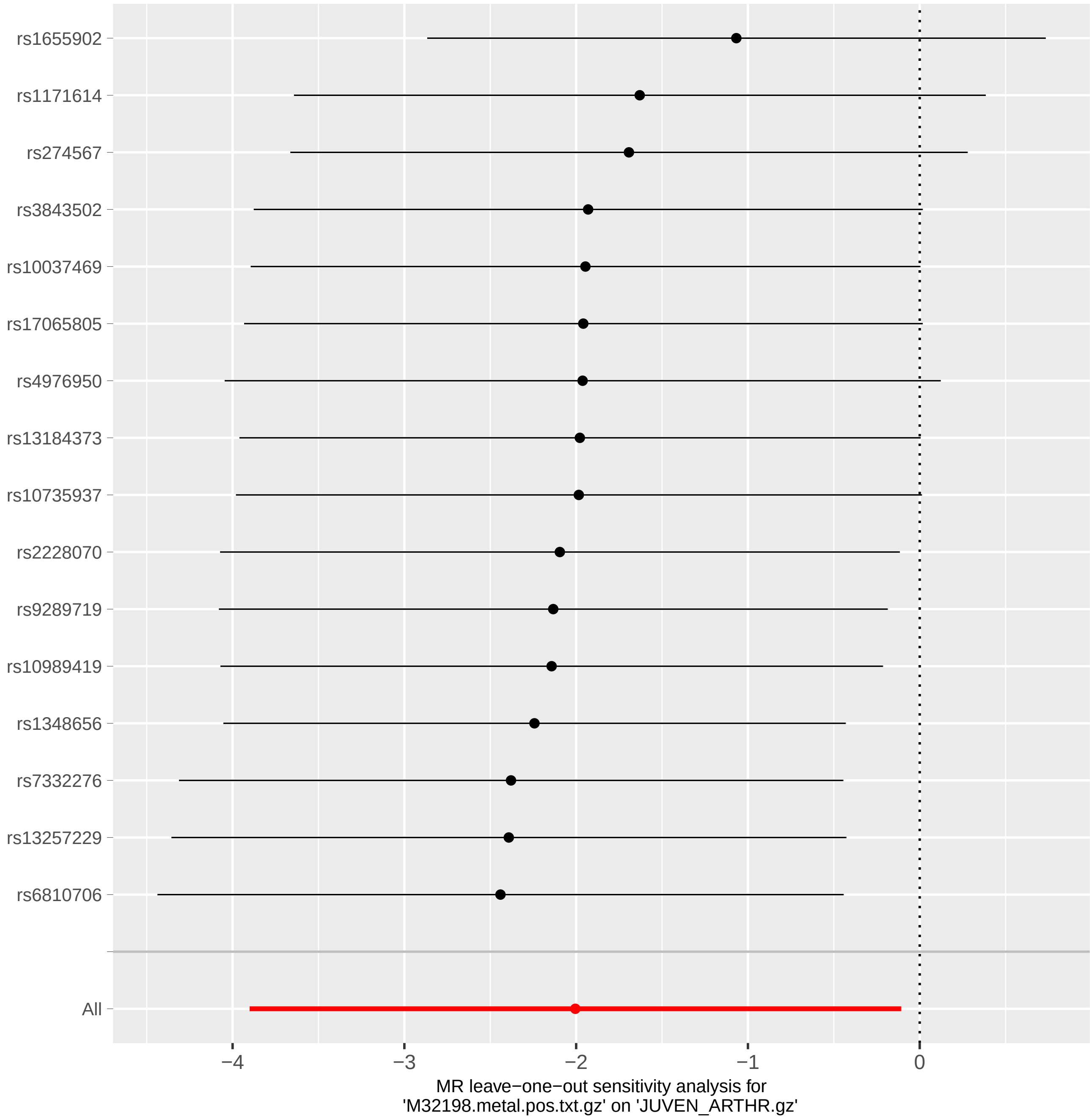
rs1655902
rs1171614
rs274567
rs3843502
rs10037469
rs17065805
rs4976950
rs13184373
rs10735937
rs2228070
rs9289719
rs10989419
rs1348656
rs7332276
rs13257229
rs6810706
All
−4
−3
−2
−1
0
MR leave−one−out sensitivity analysis for
'M32198.metal.pos.txt.gz' on 'JUVEN_ARTHR.gz'

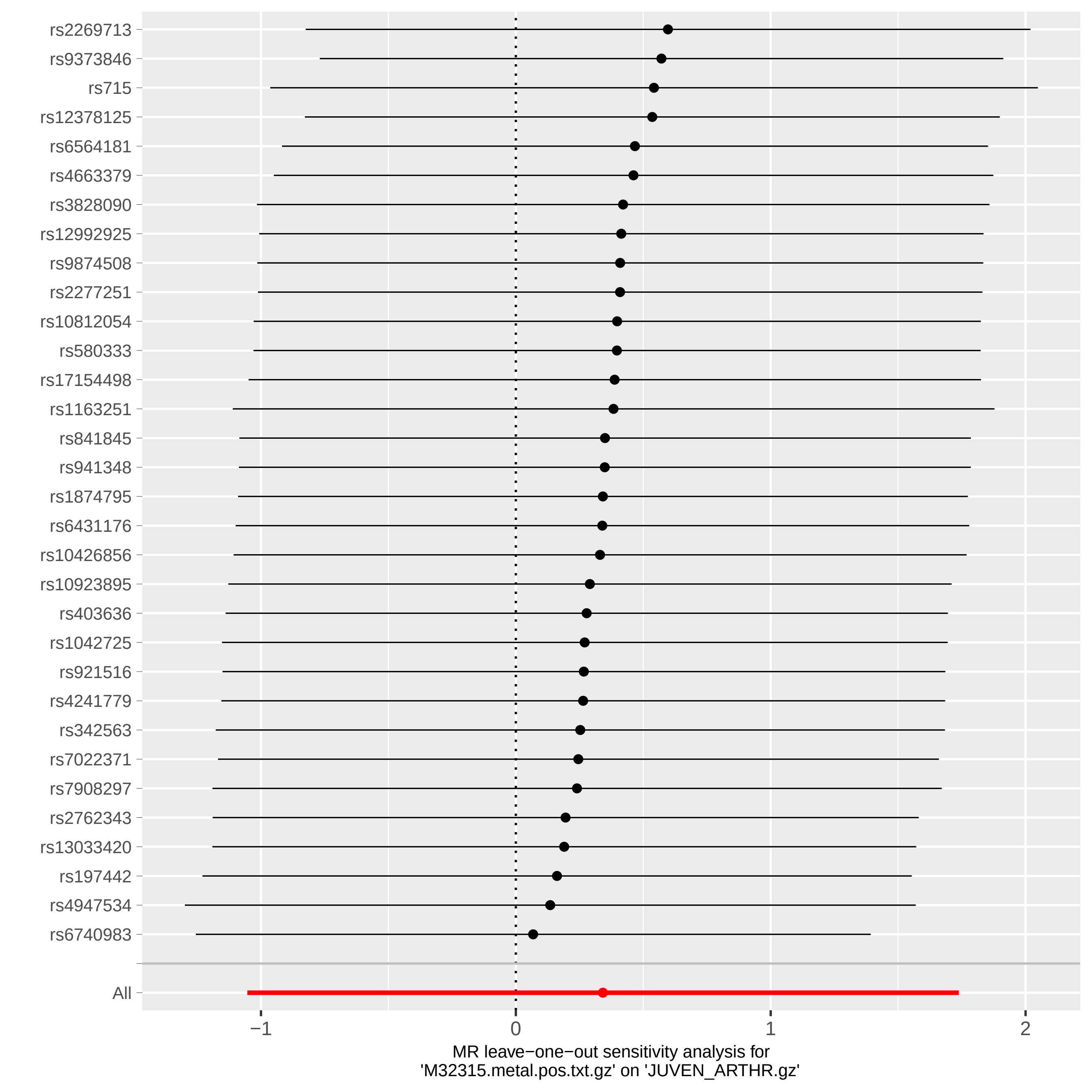
rs2269713
rs9373846
rs715
rs12378125
rs6564181
rs4663379
rs3828090
rs12992925
rs9874508
rs2277251
rs10812054
rs580333
rs17154498
rs1163251
rs841845
rs941348
rs1874795
rs6431176
rs10426856
rs10923895
rs403636
rs1042725
rs921516
rs4241779
rs342563
rs7022371
rs7908297
rs2762343
rs13033420
rs197442
rs4947534
rs6740983
All
−1
0
1
2
MR leave−one−out sensitivity analysis for
'M32315.metal.pos.txt.gz' on 'JUVEN_ARTHR.gz'

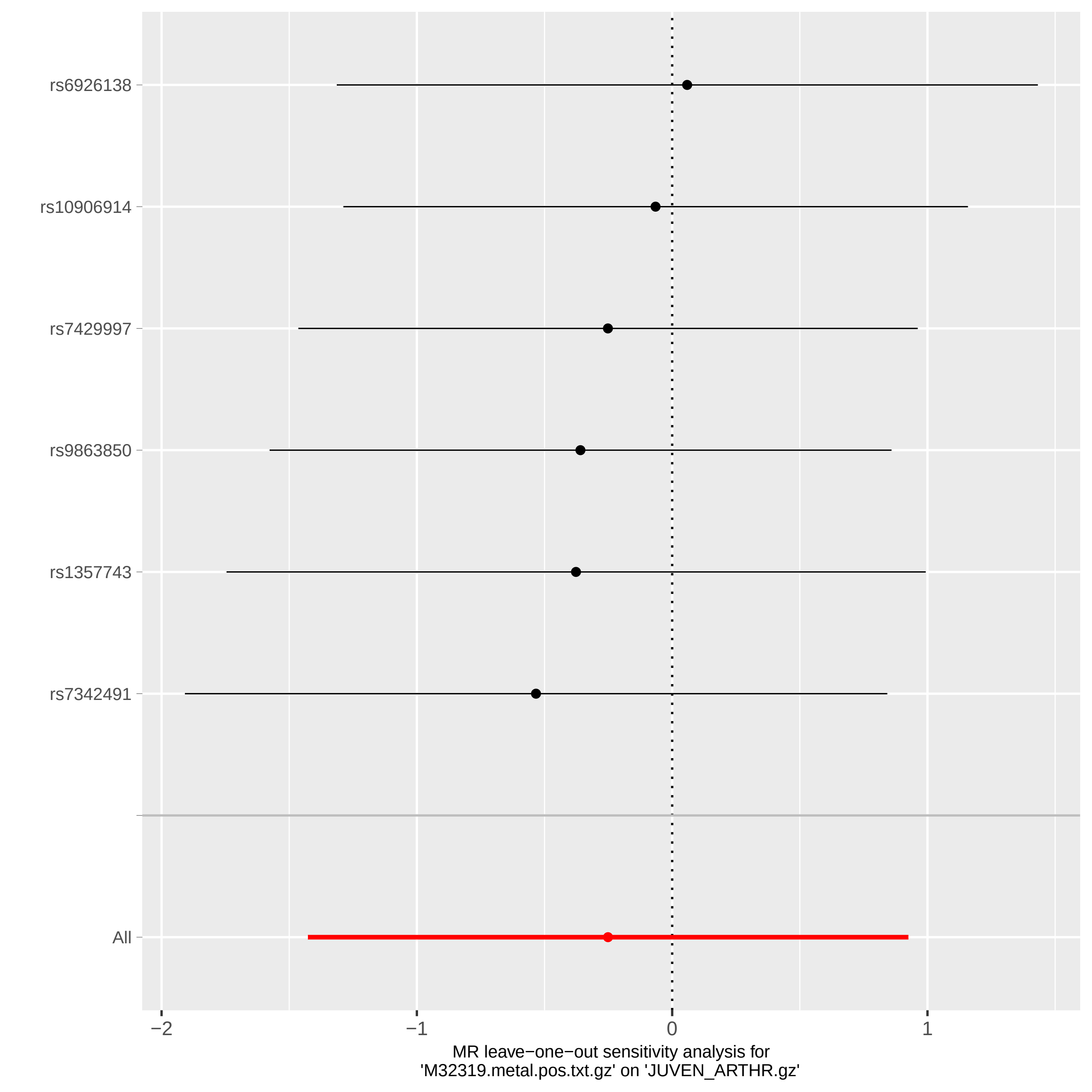
rs6926138
rs10906914
rs7429997
rs9863850
rs1357743
rs7342491
All
−2
−1
0
1
MR leave−one−out sensitivity analysis for
'M32319.metal.pos.txt.gz' on 'JUVEN_ARTHR.gz'

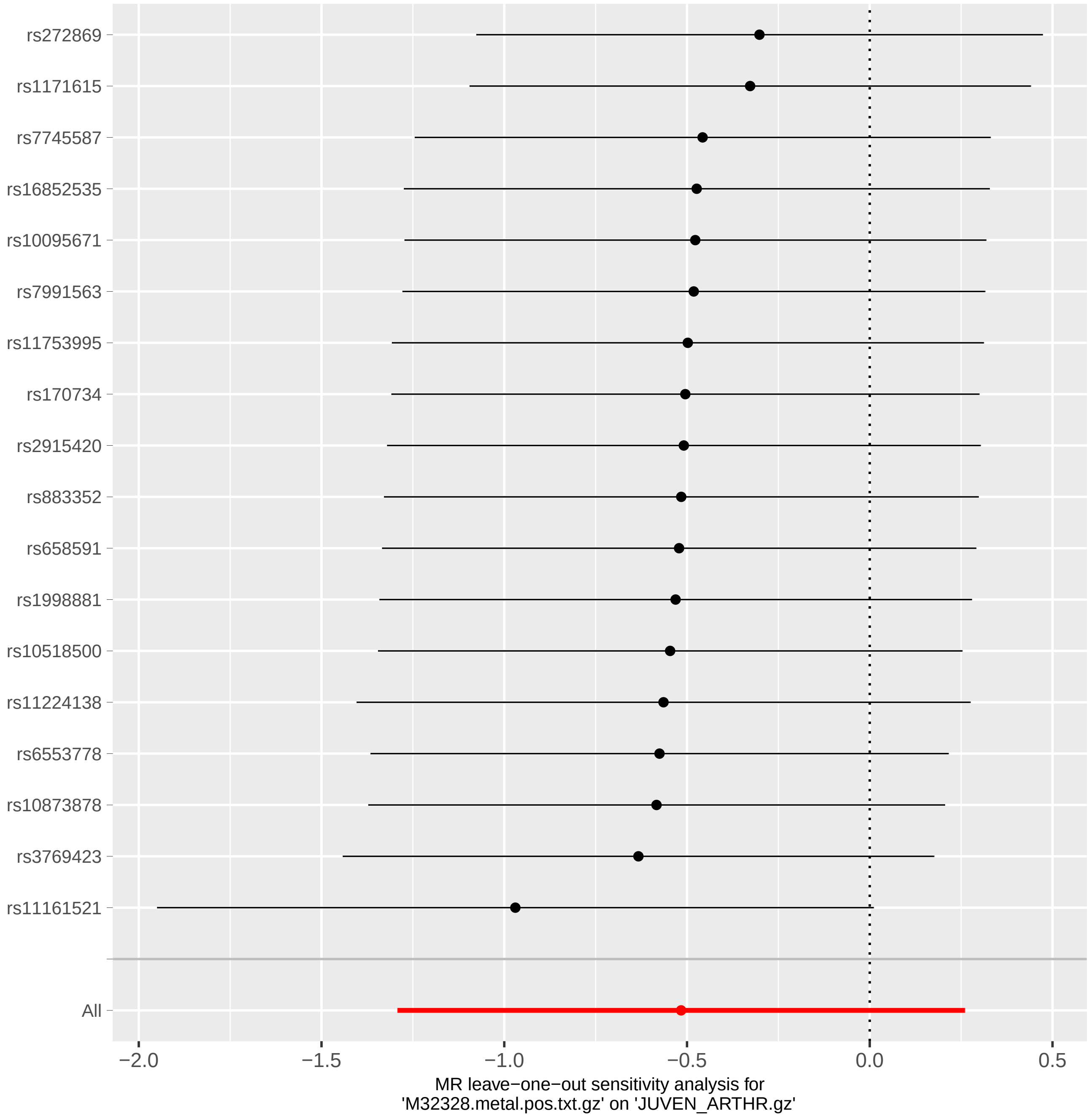
rs272869
rs1171615
rs7745587
rs16852535
rs10095671
rs7991563
rs11753995
rs170734
rs2915420
rs883352
rs658591
rs1998881
rs10518500
rs11224138
rs6553778
rs10873878
rs3769423
rs11161521
All
−2.0
−1.5
−1.0
−0.5
0.0
0.5
MR leave−one−out sensitivity analysis for
'M32328.metal.pos.txt.gz' on 'JUVEN_ARTHR.gz'

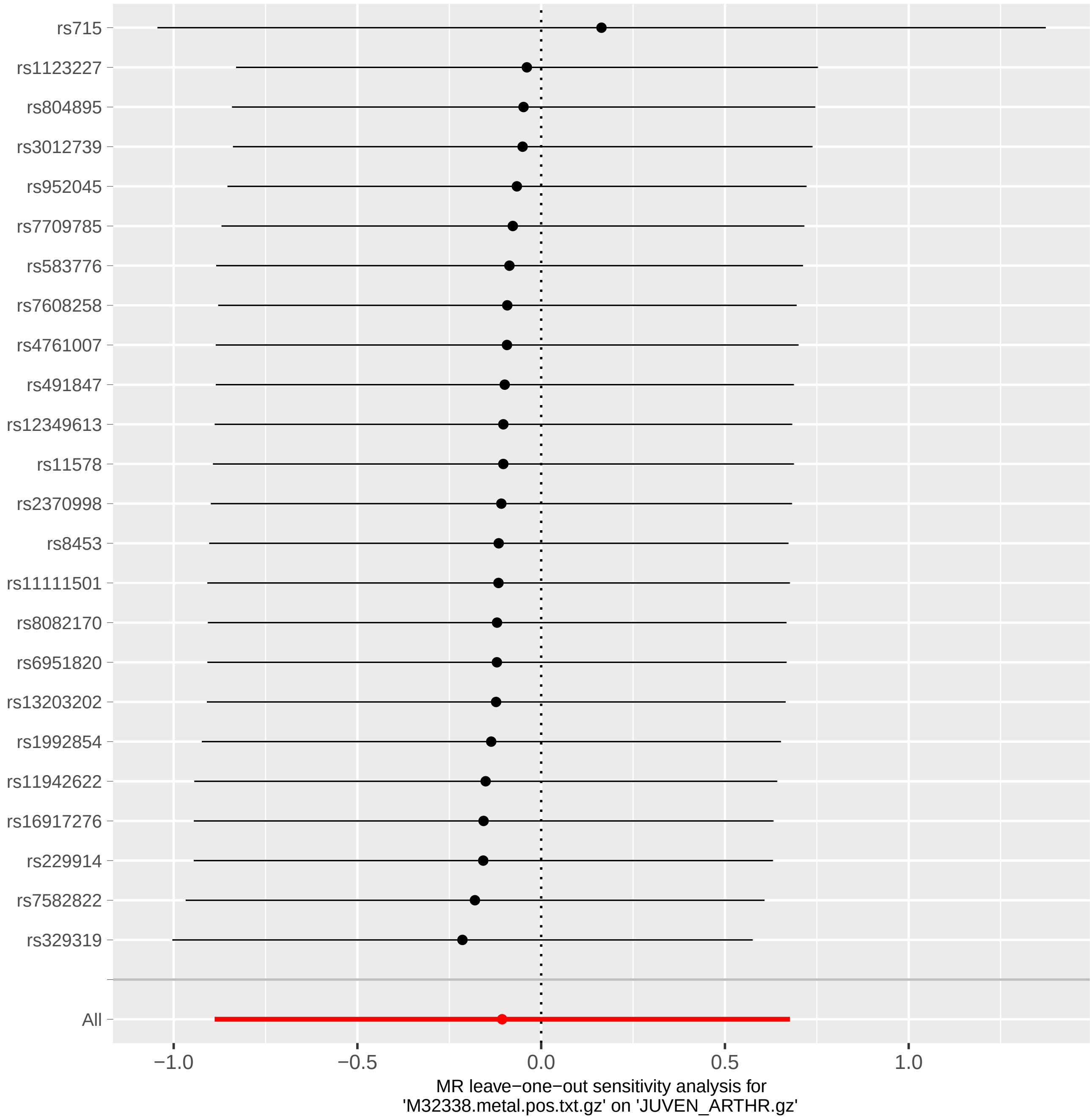
rs715
rs1123227
rs804895
rs3012739
rs952045
rs7709785
rs583776
rs7608258
rs4761007
rs491847
rs12349613
rs11578
rs2370998
rs8453
rs11111501
rs8082170
rs6951820
rs13203202
rs1992854
rs11942622
rs16917276
rs229914
rs7582822
rs329319
All
−1.0
−0.5
0.0
0.5
1.0
MR leave−one−out sensitivity analysis for
'M32338.metal.pos.txt.gz' on 'JUVEN_ARTHR.gz'

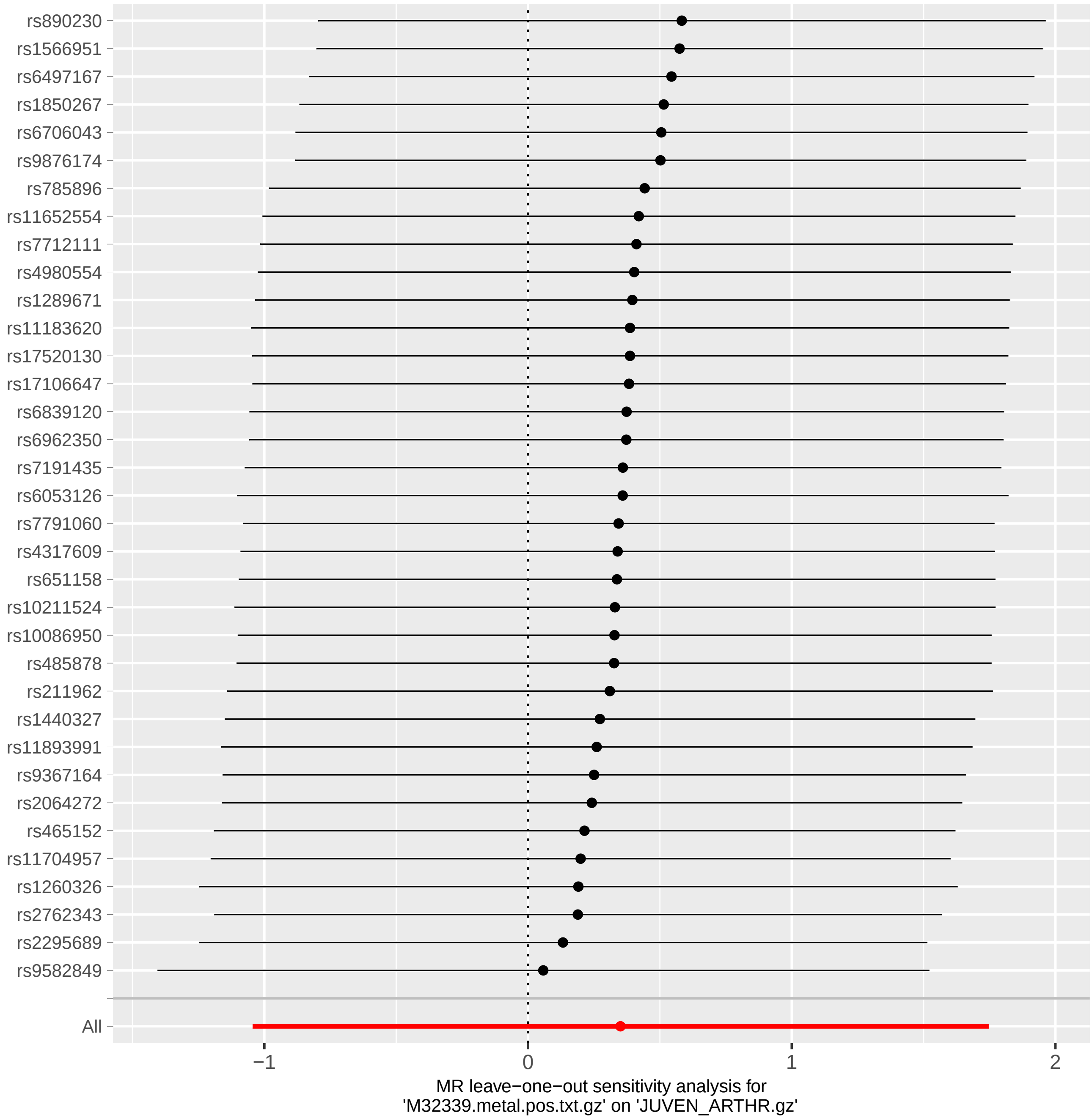
rs890230
rs1566951
rs6497167
rs1850267
rs6706043
rs9876174
rs785896
rs11652554
rs7712111
rs4980554
rs1289671
rs11183620
rs17520130
rs17106647
rs6839120
rs6962350
rs7191435
rs6053126
rs7791060
rs4317609
rs651158
rs10211524
rs10086950
rs485878
rs211962
rs1440327
rs11893991
rs9367164
rs2064272
rs465152
rs11704957
rs1260326
rs2762343
rs2295689
rs9582849
All
−1
0
1
2
MR leave−one−out sensitivity analysis for
'M32339.metal.pos.txt.gz' on 'JUVEN_ARTHR.gz'

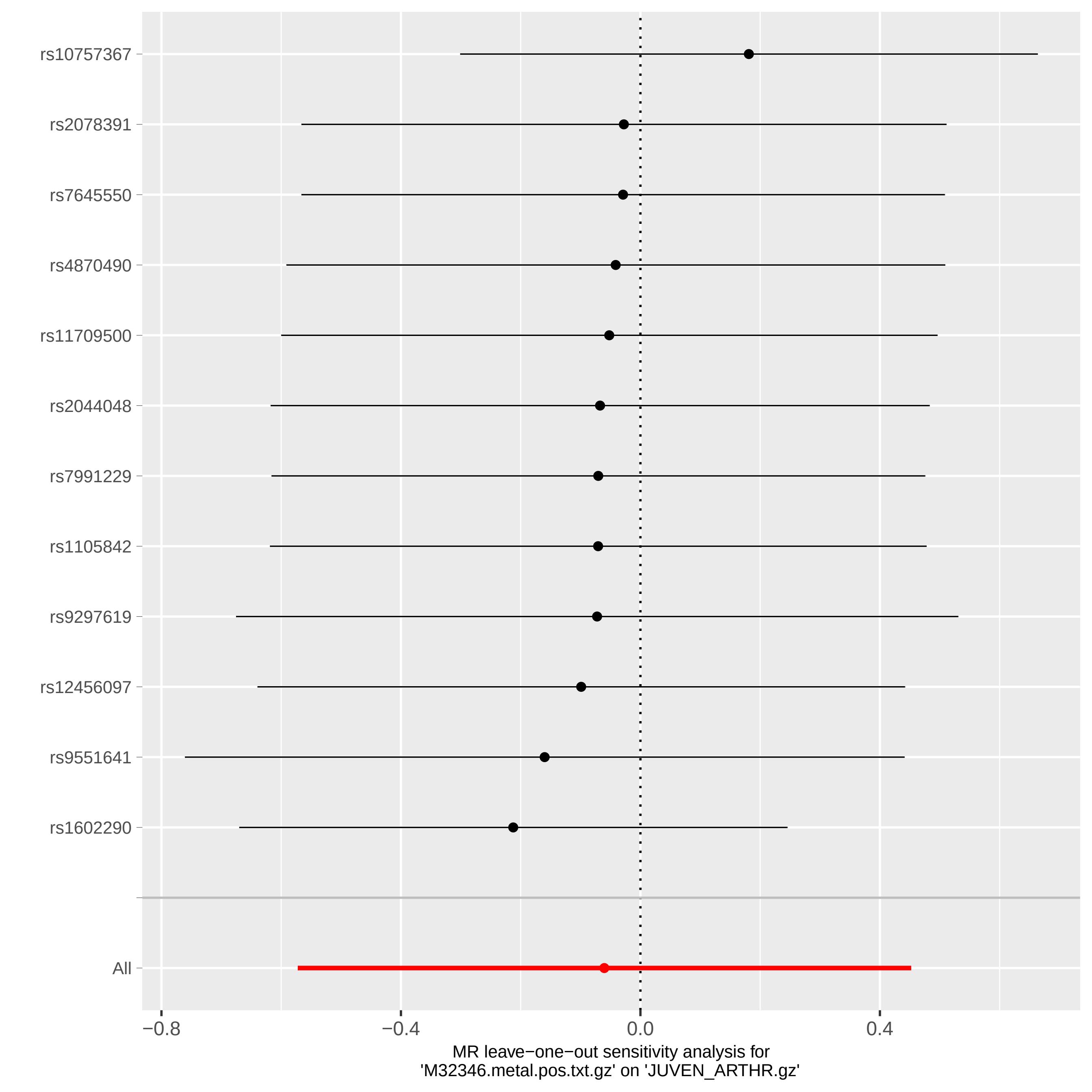

rs10757367
rs2078391
rs7645550
rs4870490
rs11709500
rs2044048
rs7991229
rs1105842
rs9297619
rs12456097
rs9551641
rs1602290
All
−0.8
−0.4
0.0
0.4
MR leave−one−out sensitivity analysis for
'M32346.metal.pos.txt.gz' on 'JUVEN_ARTHR.gz'

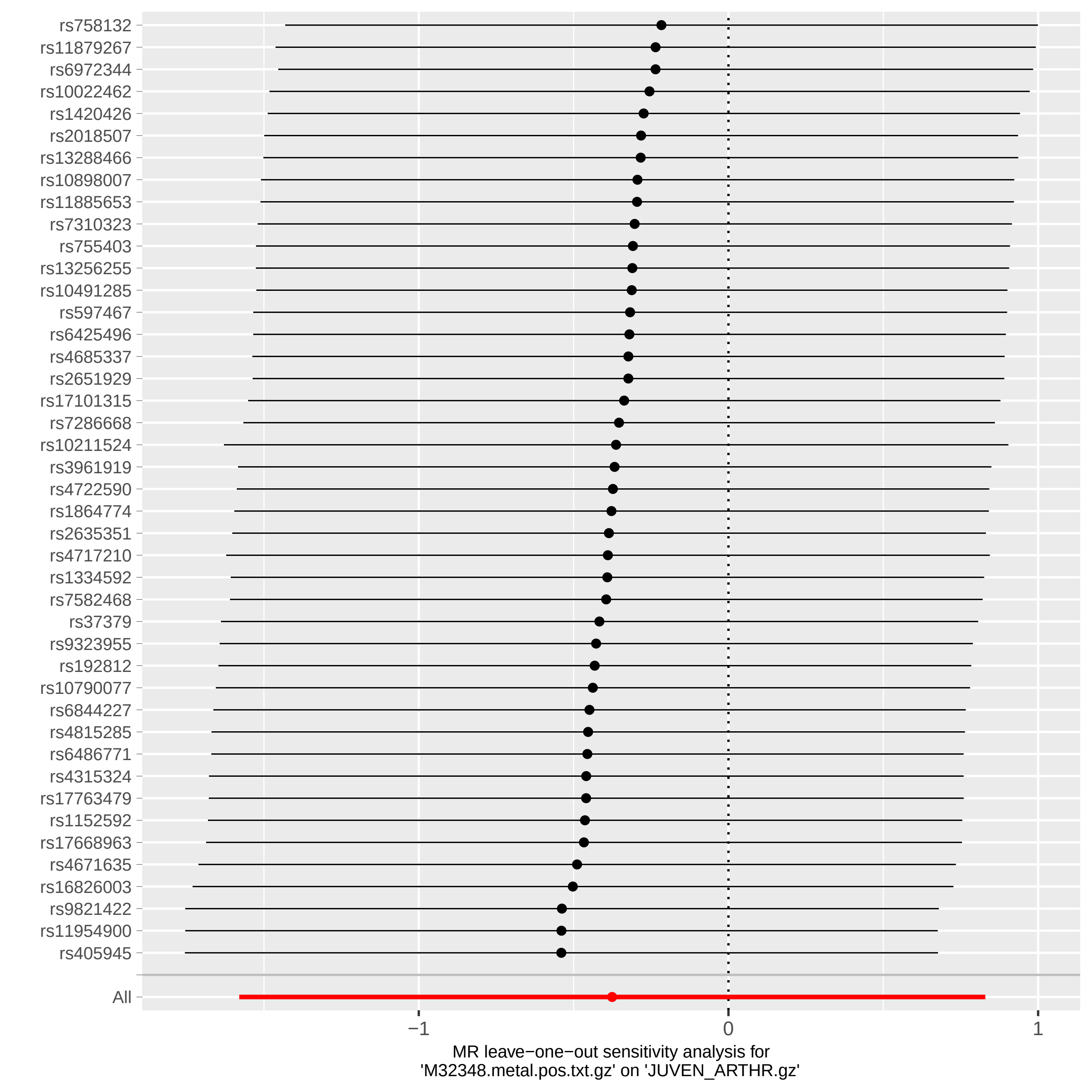

rs758132
rs11879267
rs6972344
rs10022462
rs1420426
rs2018507
rs13288466
rs10898007
rs11885653
rs7310323
rs755403
rs13256255
rs10491285
rs597467
rs6425496
rs4685337
rs2651929
rs17101315
rs7286668
rs10211524
rs3961919
rs4722590
rs1864774
rs2635351
rs4717210
rs1334592
rs7582468
rs37379
rs9323955
rs192812
rs10790077
rs6844227
rs4815285
rs6486771
rs4315324
rs17763479
rs1152592
rs17668963
rs4671635
rs16826003
rs9821422
rs11954900
rs405945
All
−1
0
1
MR leave−one−out sensitivity analysis for
'M32348.metal.pos.txt.gz' on 'JUVEN_ARTHR.gz'

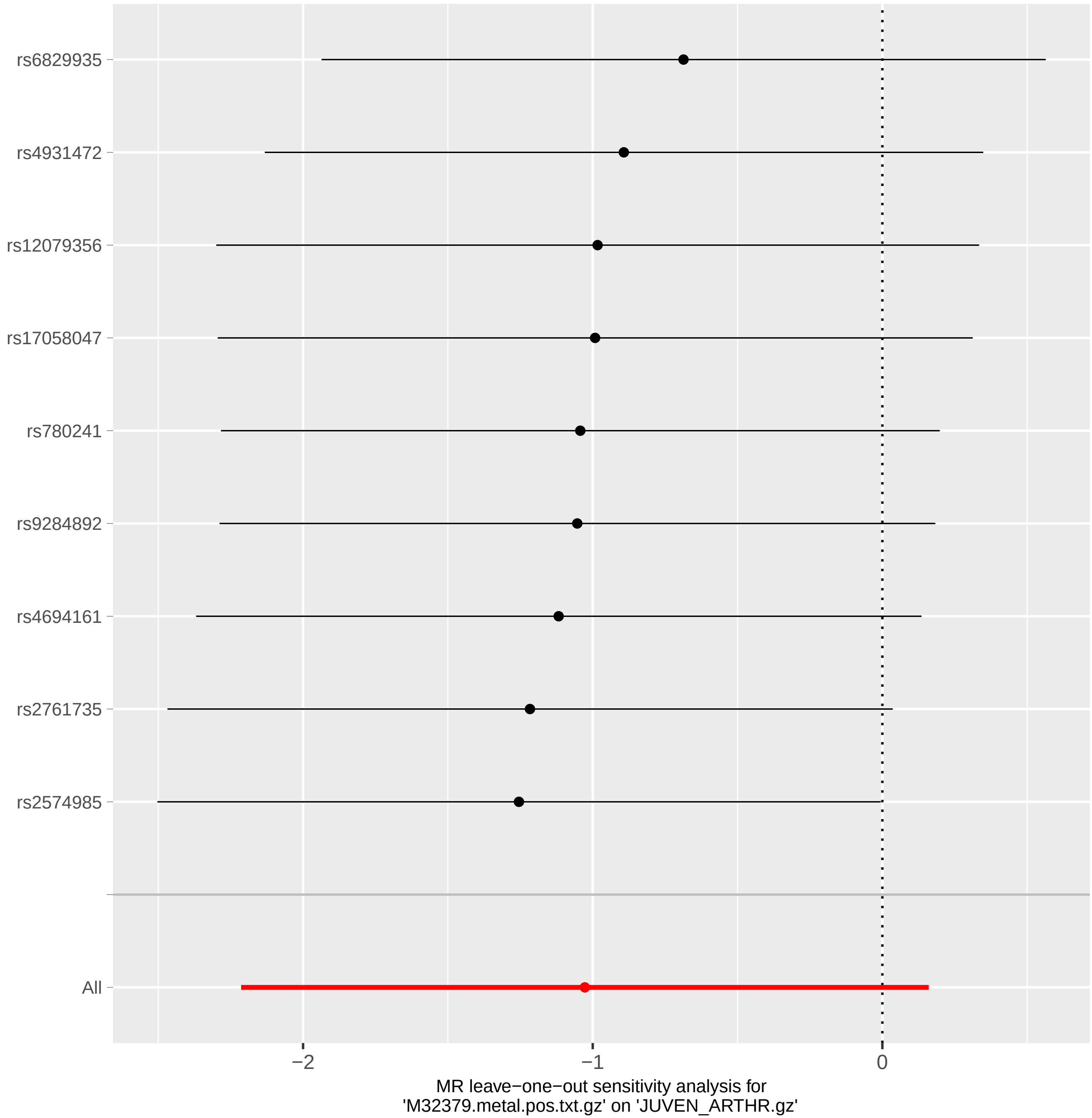

rs6829935
rs4931472
rs12079356
rs17058047
rs780241
rs9284892
rs4694161
rs2761735
rs2574985
All
−2
−1
0
MR leave−one−out sensitivity analysis for
'M32379.metal.pos.txt.gz' on 'JUVEN_ARTHR.gz'

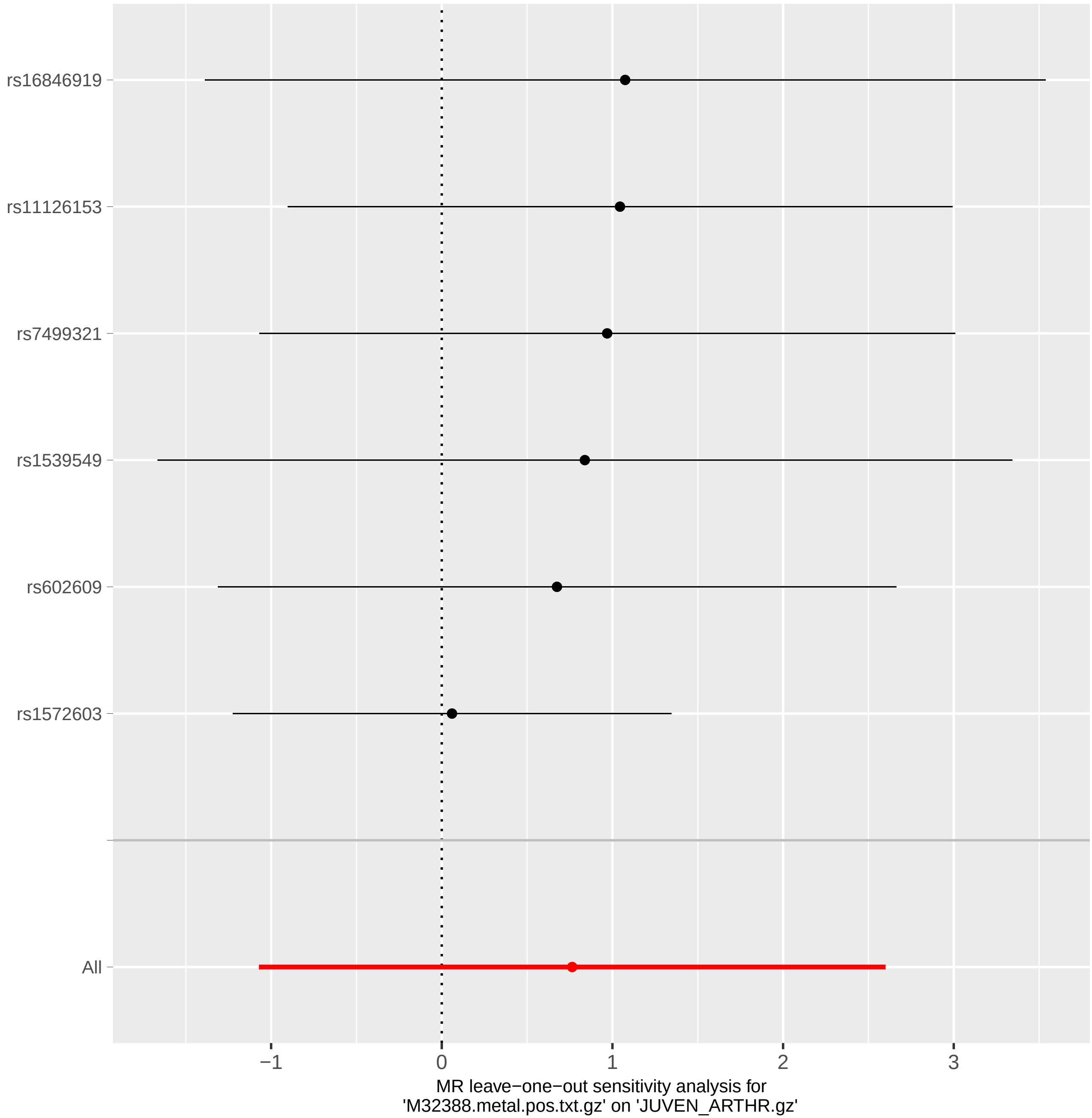
rs16846919
rs11126153
rs7499321
rs1539549
rs602609
rs1572603
All
−1
0
1
2
3
MR leave−one−out sensitivity analysis for
'M32388.metal.pos.txt.gz' on 'JUVEN_ARTHR.gz'

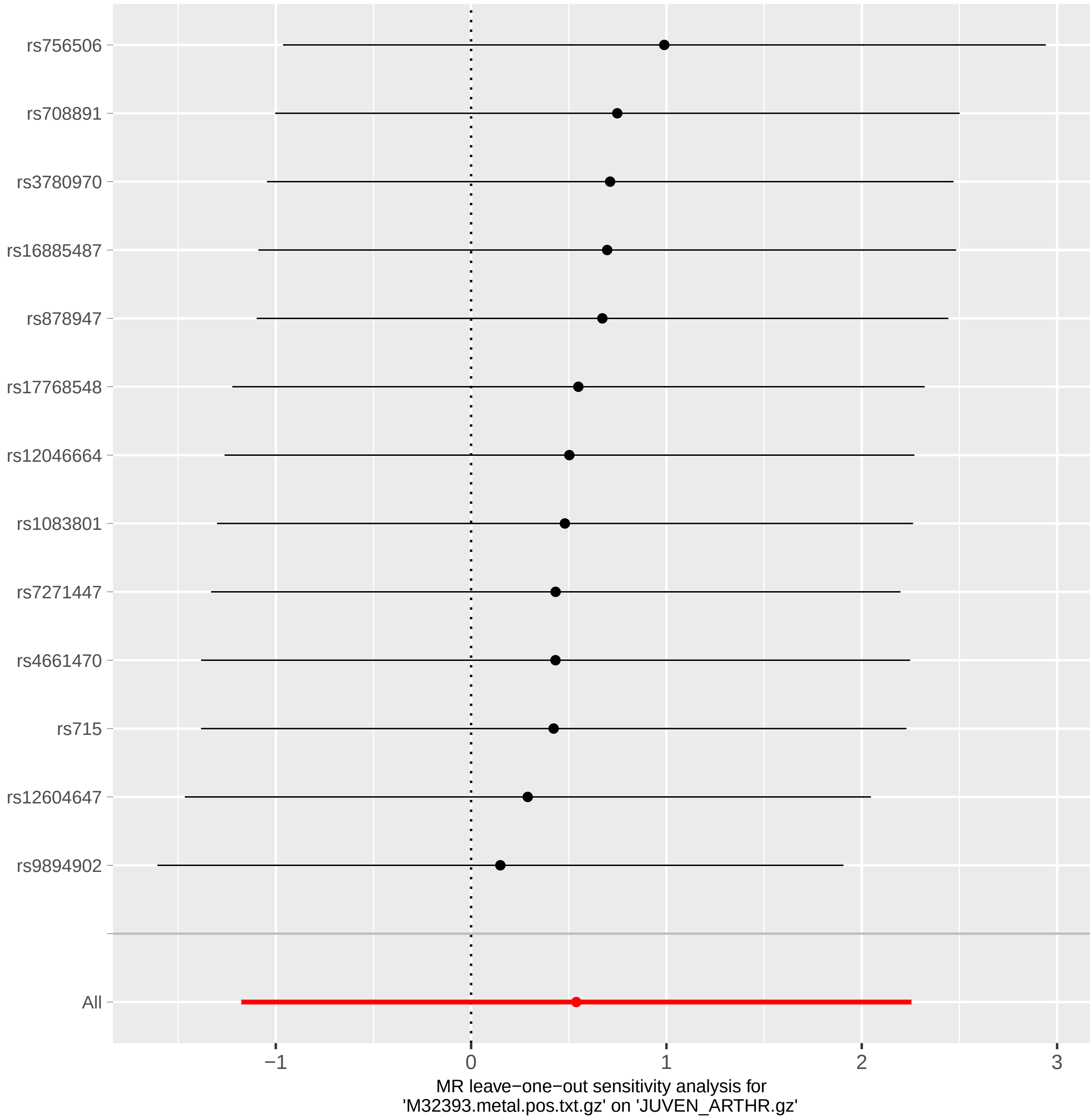

rs756506
rs708891
rs3780970
rs16885487
rs878947
rs17768548
rs12046664
rs1083801
rs7271447
rs4661470
rs715
rs12604647
rs9894902
All
−1
0
1
2
3
MR leave−one−out sensitivity analysis for
'M32393.metal.pos.txt.gz' on 'JUVEN_ARTHR.gz'

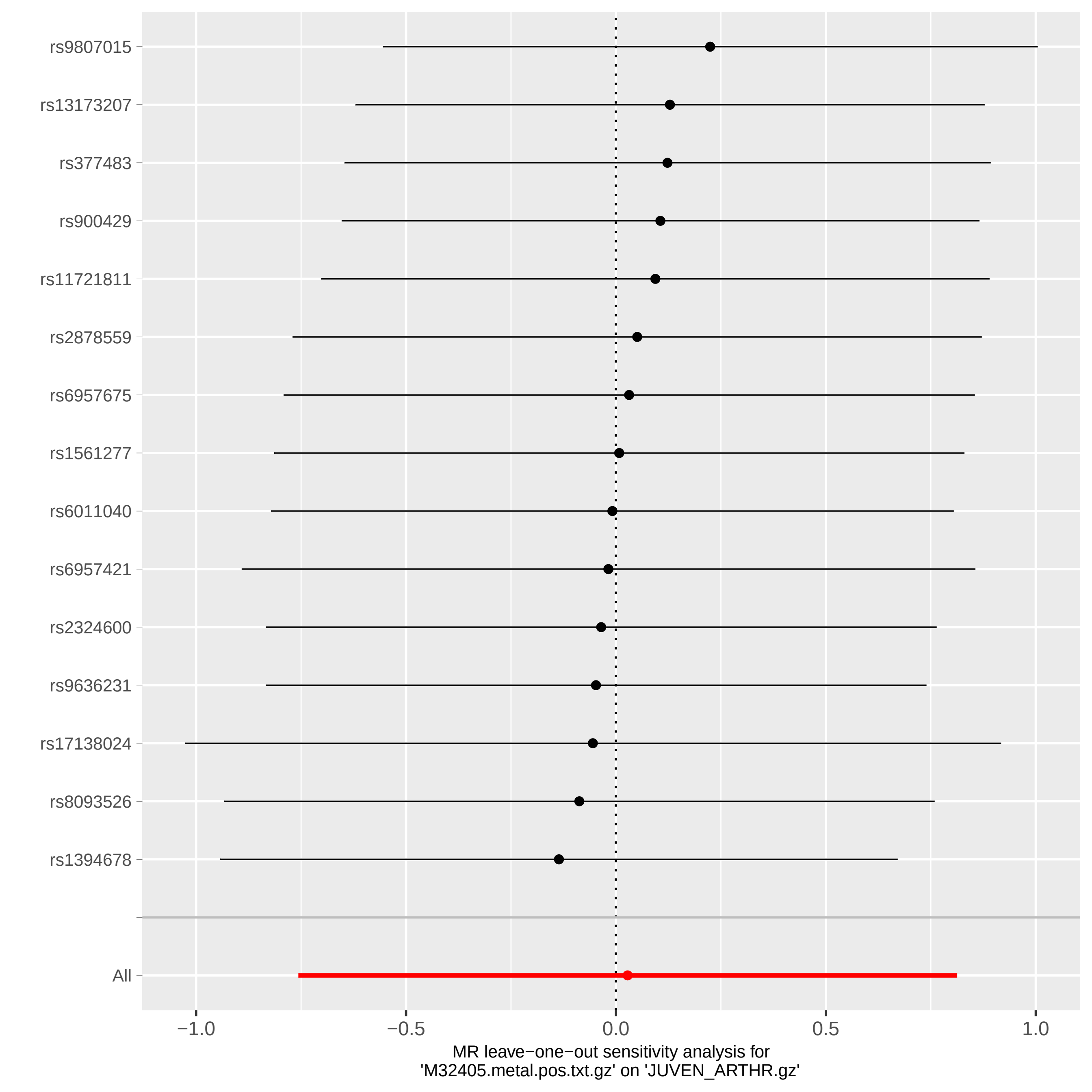

rs9807015
rs13173207
rs377483
rs900429
rs11721811
rs2878559
rs6957675
rs1561277
rs6011040
rs6957421
rs2324600
rs9636231
rs17138024
rs8093526
rs1394678
All
−1.0
−0.5
0.0
0.5
1.0
MR leave−one−out sensitivity analysis for
'M32405.metal.pos.txt.gz' on 'JUVEN_ARTHR.gz'

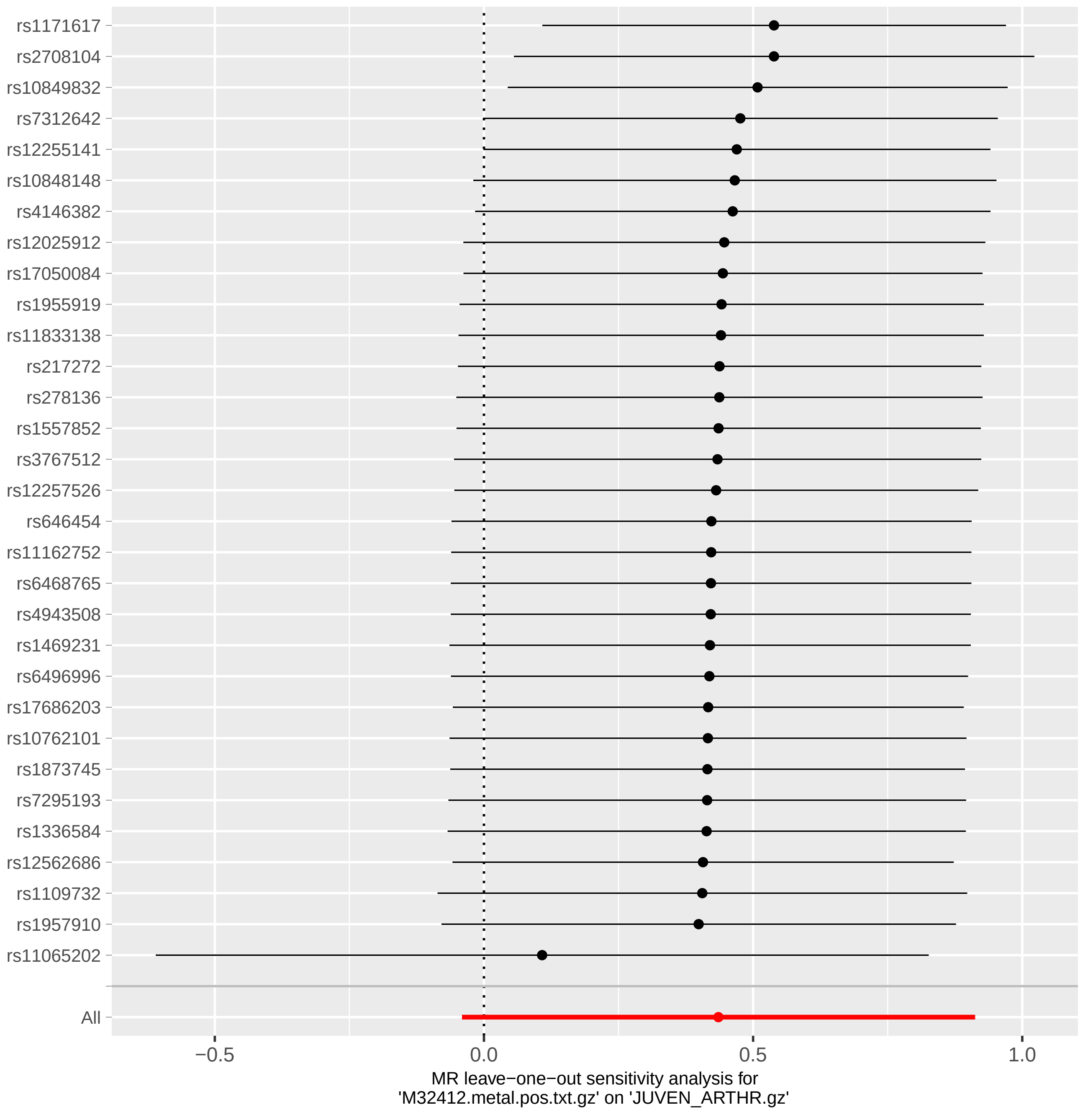

rs1171617
rs2708104
rs10849832
rs7312642
rs12255141
rs10848148
rs4146382
rs12025912
rs17050084
rs1955919
rs11833138
rs217272
rs278136
rs1557852
rs3767512
rs12257526
rs646454
rs11162752
rs6468765
rs4943508
rs1469231
rs6496996
rs17686203
rs10762101
rs1873745
rs7295193
rs1336584
rs12562686
rs1109732
rs1957910
rs11065202
All
−0.5
0.0
0.5
1.0
MR leave−one−out sensitivity analysis for
'M32412.metal.pos.txt.gz' on 'JUVEN_ARTHR.gz'

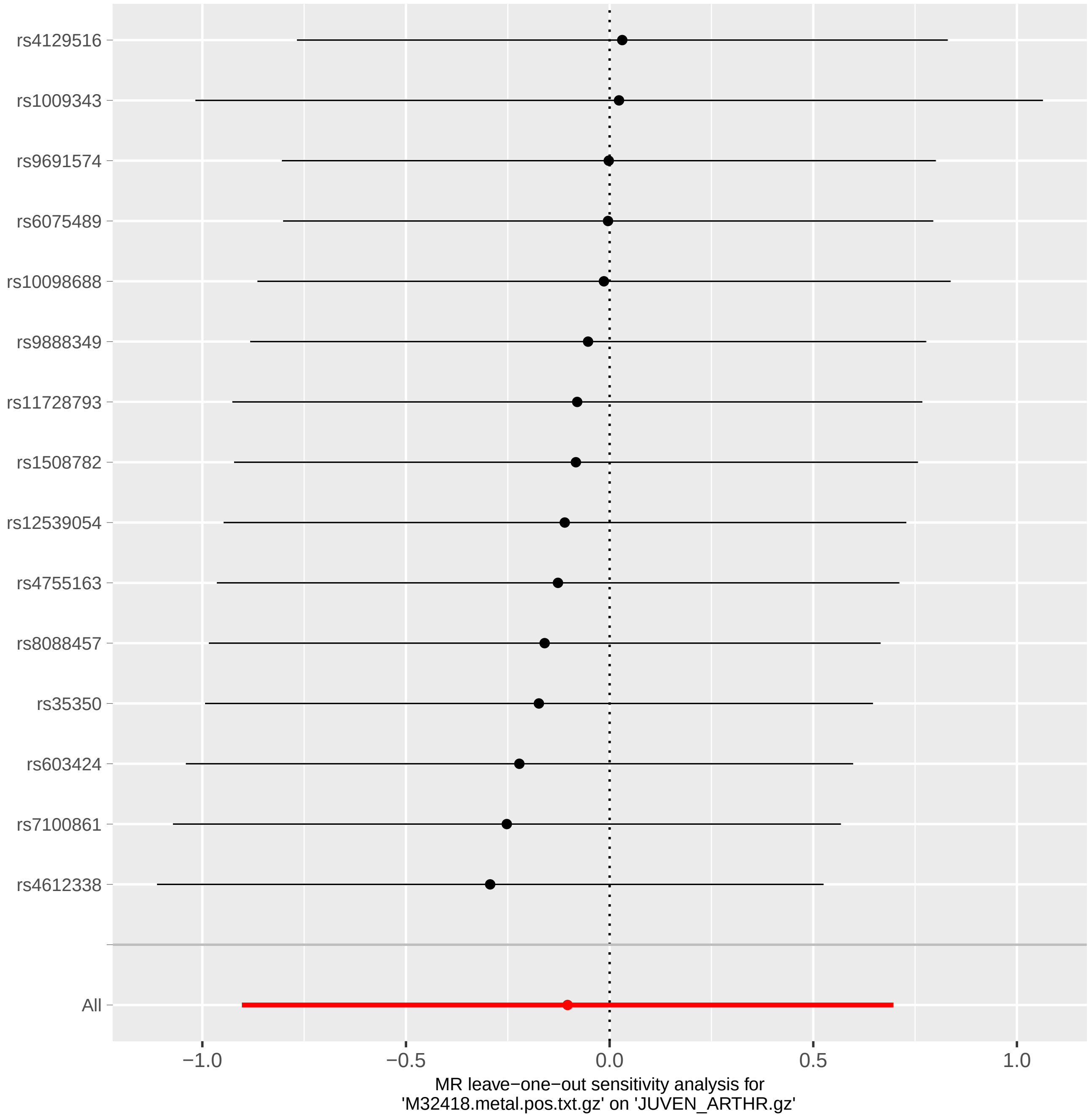
rs4129516
rs1009343
rs9691574
rs6075489
rs10098688
rs9888349
rs11728793
rs1508782
rs12539054
rs4755163
rs8088457
rs35350
rs603424
rs7100861
rs4612338
All
−1.0
−0.5
0.0
0.5
1.0
MR leave−one−out sensitivity analysis for
'M32418.metal.pos.txt.gz' on 'JUVEN_ARTHR.gz'

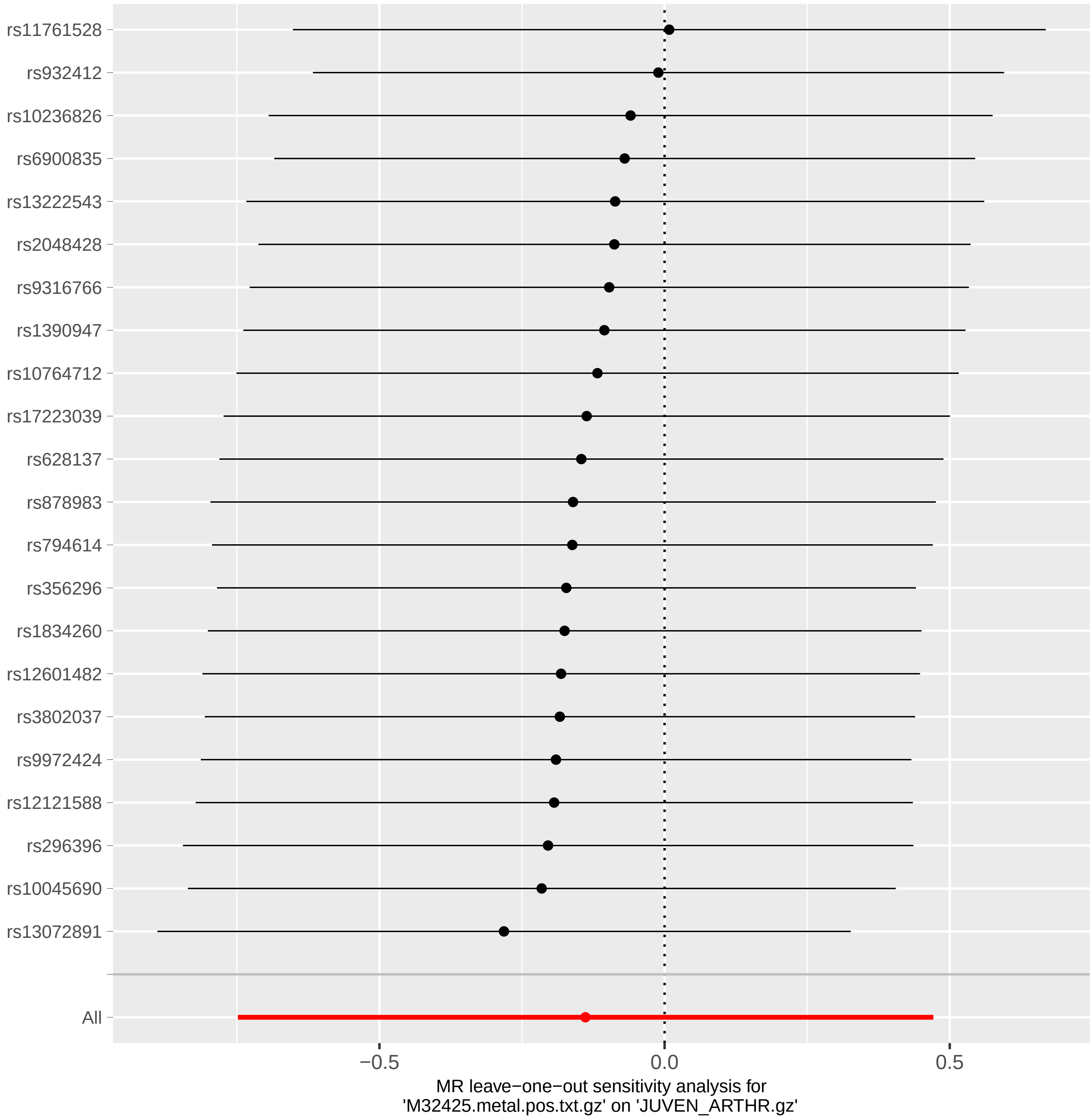

rs11761528
rs932412
rs10236826
rs6900835
rs13222543
rs2048428
rs9316766
rs1390947
rs10764712
rs17223039
rs628137
rs878983
rs794614
rs356296
rs1834260
rs12601482
rs3802037
rs9972424
rs12121588
rs296396
rs10045690
rs13072891
All
−0.5
0.0
0.5
MR leave−one−out sensitivity analysis for
'M32425.metal.pos.txt.gz' on 'JUVEN_ARTHR.gz'

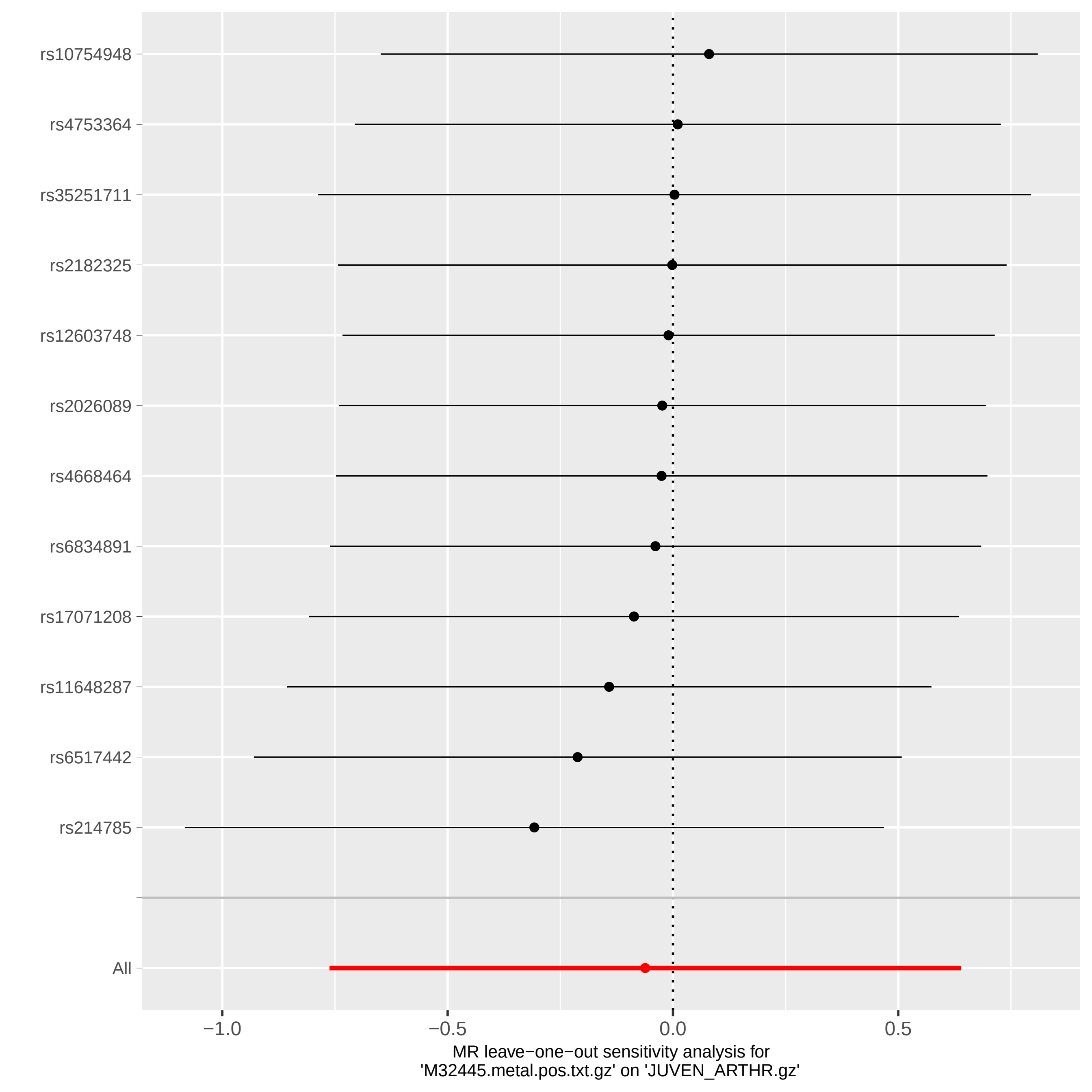
rs10754948
rs4753364
rs35251711
rs2182325
rs12603748
rs2026089
rs4668464
rs6834891
rs17071208
rs11648287
rs6517442
rs214785
All
−1.0
−0.5
0.0
0.5
MR leave−one−out sensitivity analysis for
'M32445.metal.pos.txt.gz' on 'JUVEN_ARTHR.gz'

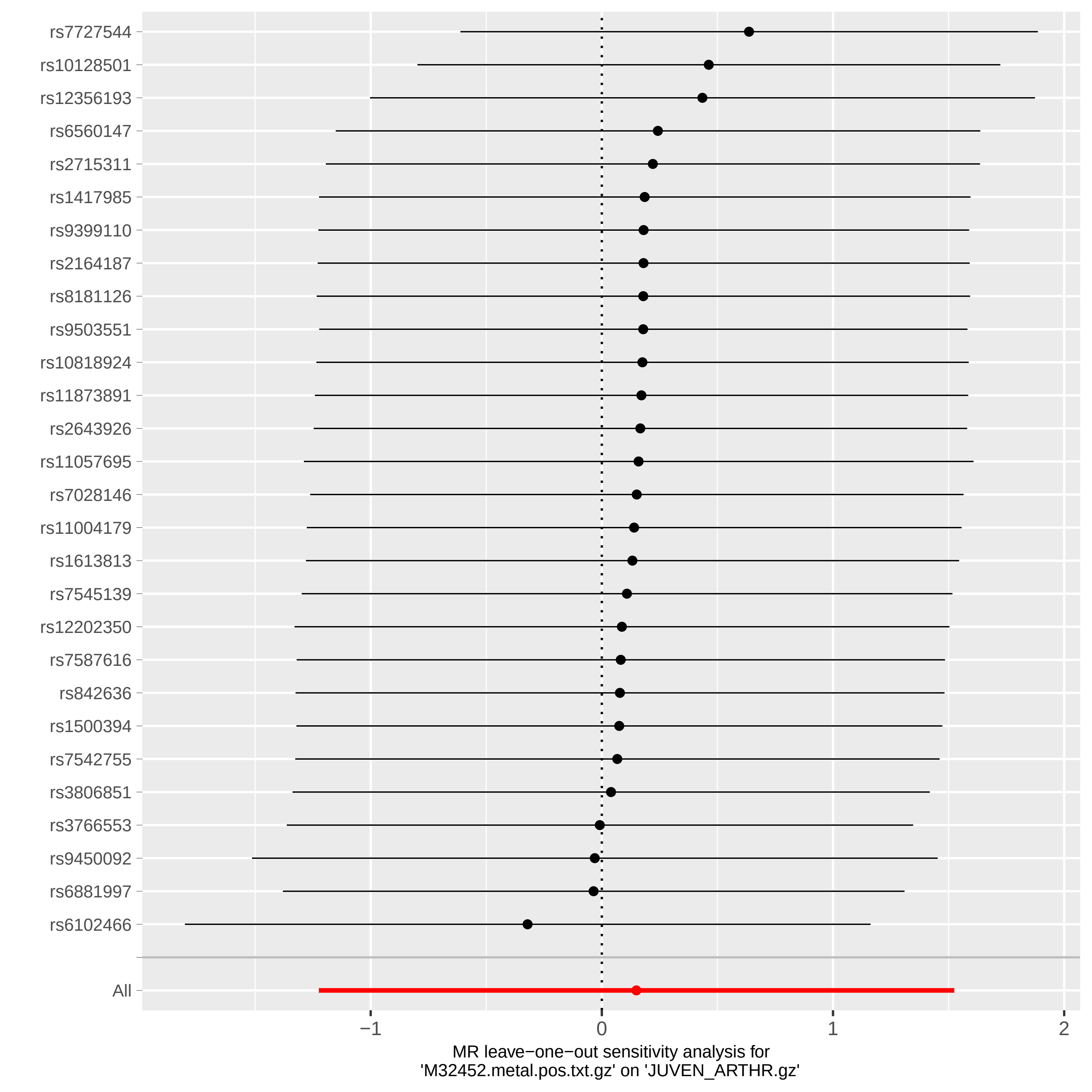

rs7727544
rs10128501
rs12356193
rs6560147
rs2715311
rs1417985
rs9399110
rs2164187
rs8181126
rs9503551
rs10818924
rs11873891
rs2643926
rs11057695
rs7028146
rs11004179
rs1613813
rs7545139
rs12202350
rs7587616
rs842636
rs1500394
rs7542755
rs3806851
rs3766553
rs9450092
rs6881997
rs6102466
All
−1
0
1
2
MR leave−one−out sensitivity analysis for
'M32452.metal.pos.txt.gz' on 'JUVEN_ARTHR.gz'

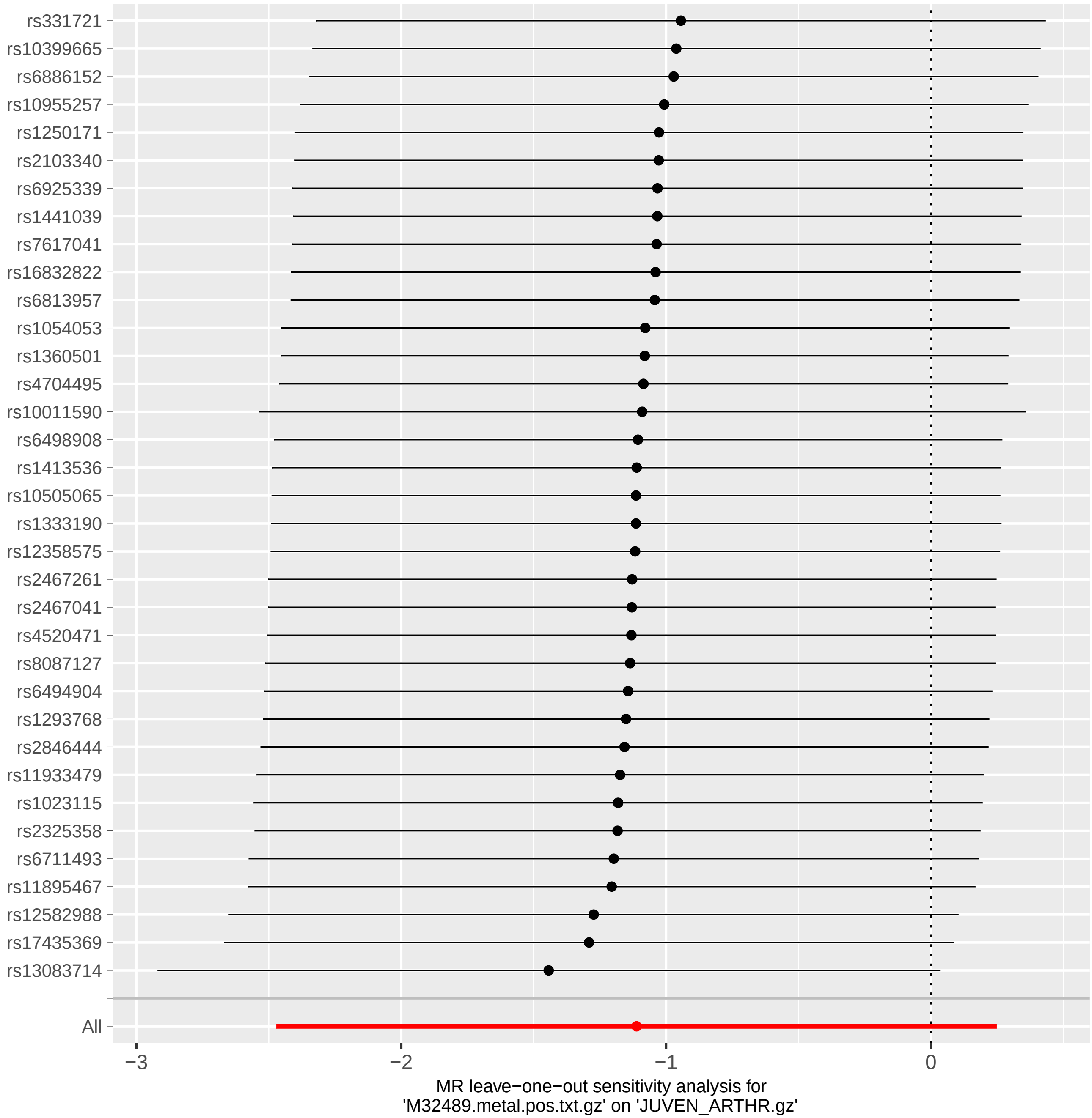

rs331721
rs10399665
rs6886152
rs10955257
rs1250171
rs2103340
rs6925339
rs1441039
rs7617041
rs16832822
rs6813957
rs1054053
rs1360501
rs4704495
rs10011590
rs6498908
rs1413536
rs10505065
rs1333190
rs12358575
rs2467261
rs2467041
rs4520471
rs8087127
rs6494904
rs1293768
rs2846444
rs11933479
rs1023115
rs2325358
rs6711493
rs11895467
rs12582988
rs17435369
rs13083714
All
−3
−2
−1
0
MR leave−one−out sensitivity analysis for
'M32489.metal.pos.txt.gz' on 'JUVEN_ARTHR.gz'

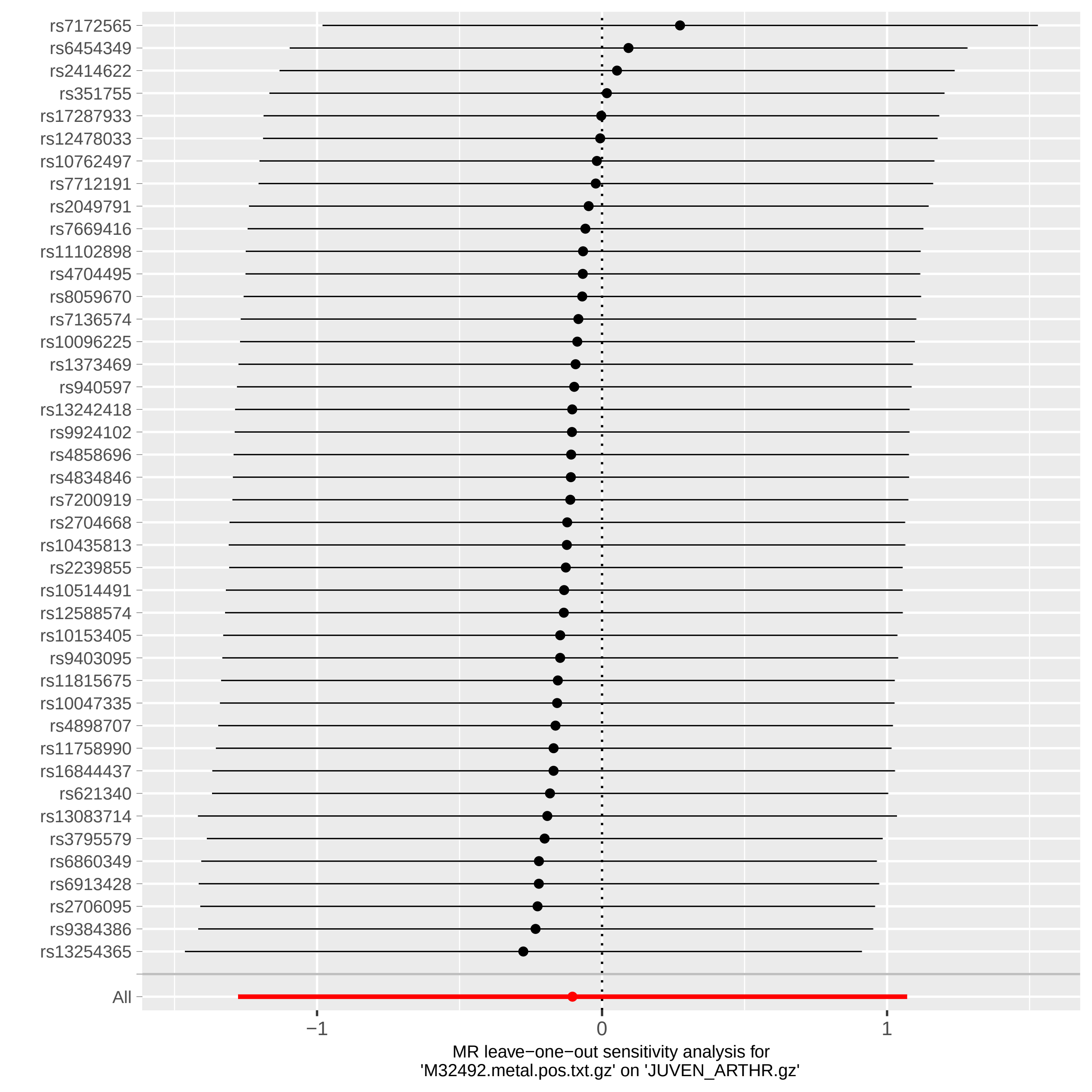
rs7172565
rs6454349
rs2414622
rs351755
rs17287933
rs12478033
rs10762497
rs7712191
rs2049791
rs7669416
rs11102898
rs4704495
rs8059670
rs7136574
rs10096225
rs1373469
rs940597
rs13242418
rs9924102
rs4858696
rs4834846
rs7200919
rs2704668
rs10435813
rs2239855
rs10514491
rs12588574
rs10153405
rs9403095
rs11815675
rs10047335
rs4898707
rs11758990
rs16844437
rs621340
rs13083714
rs3795579
rs6860349
rs6913428
rs2706095
rs9384386
rs13254365
All
−1
0
1
MR leave−one−out sensitivity analysis for
'M32492.metal.pos.txt.gz' on 'JUVEN_ARTHR.gz'

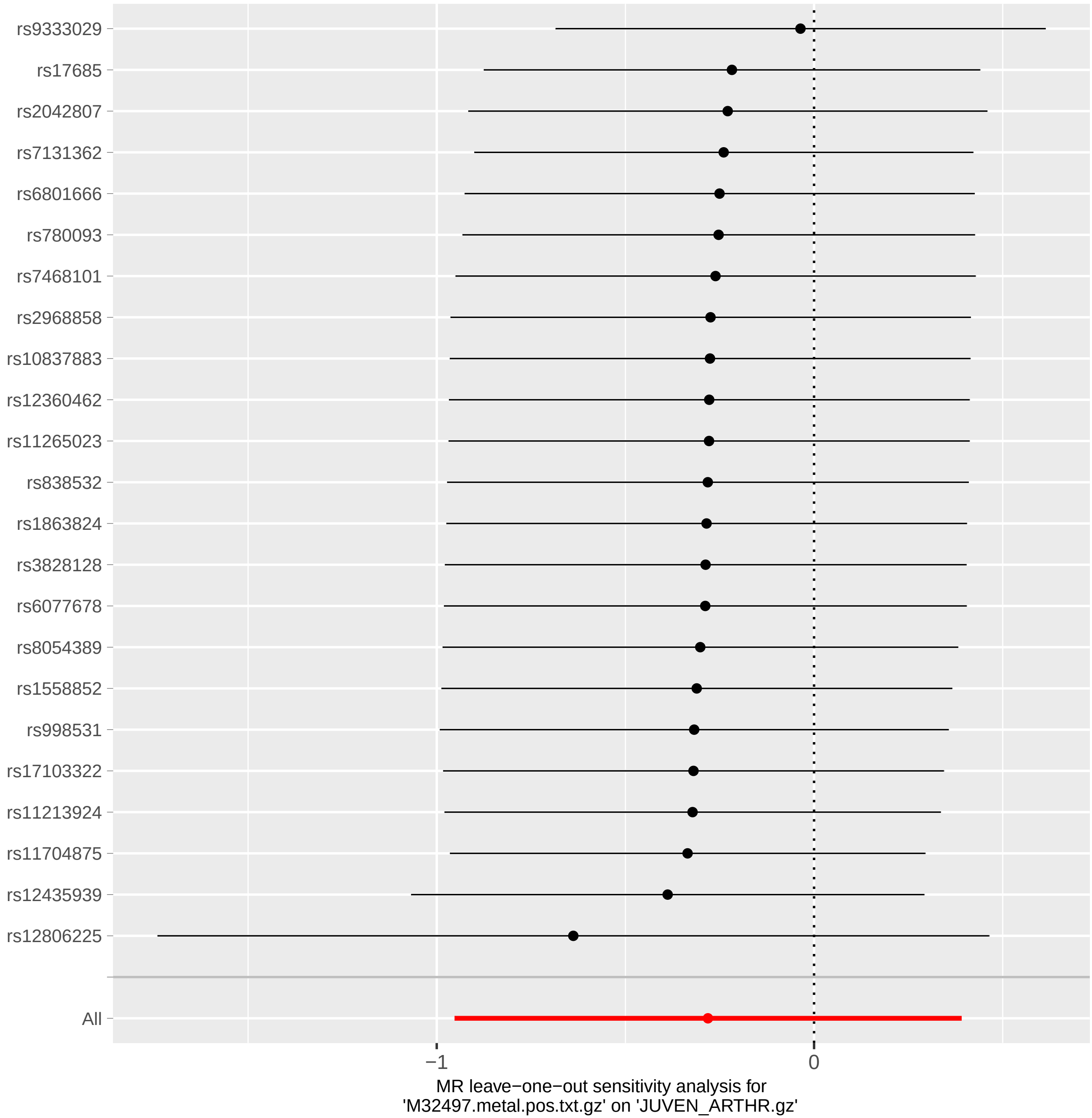
rs9333029
rs17685
rs2042807
rs7131362
rs6801666
rs780093
rs7468101
rs2968858
rs10837883
rs12360462
rs11265023
rs838532
rs1863824
rs3828128
rs6077678
rs8054389
rs1558852
rs998531
rs17103322
rs11213924
rs11704875
rs12435939
rs12806225
All
−1
0
MR leave−one−out sensitivity analysis for
'M32497.metal.pos.txt.gz' on 'JUVEN_ARTHR.gz'

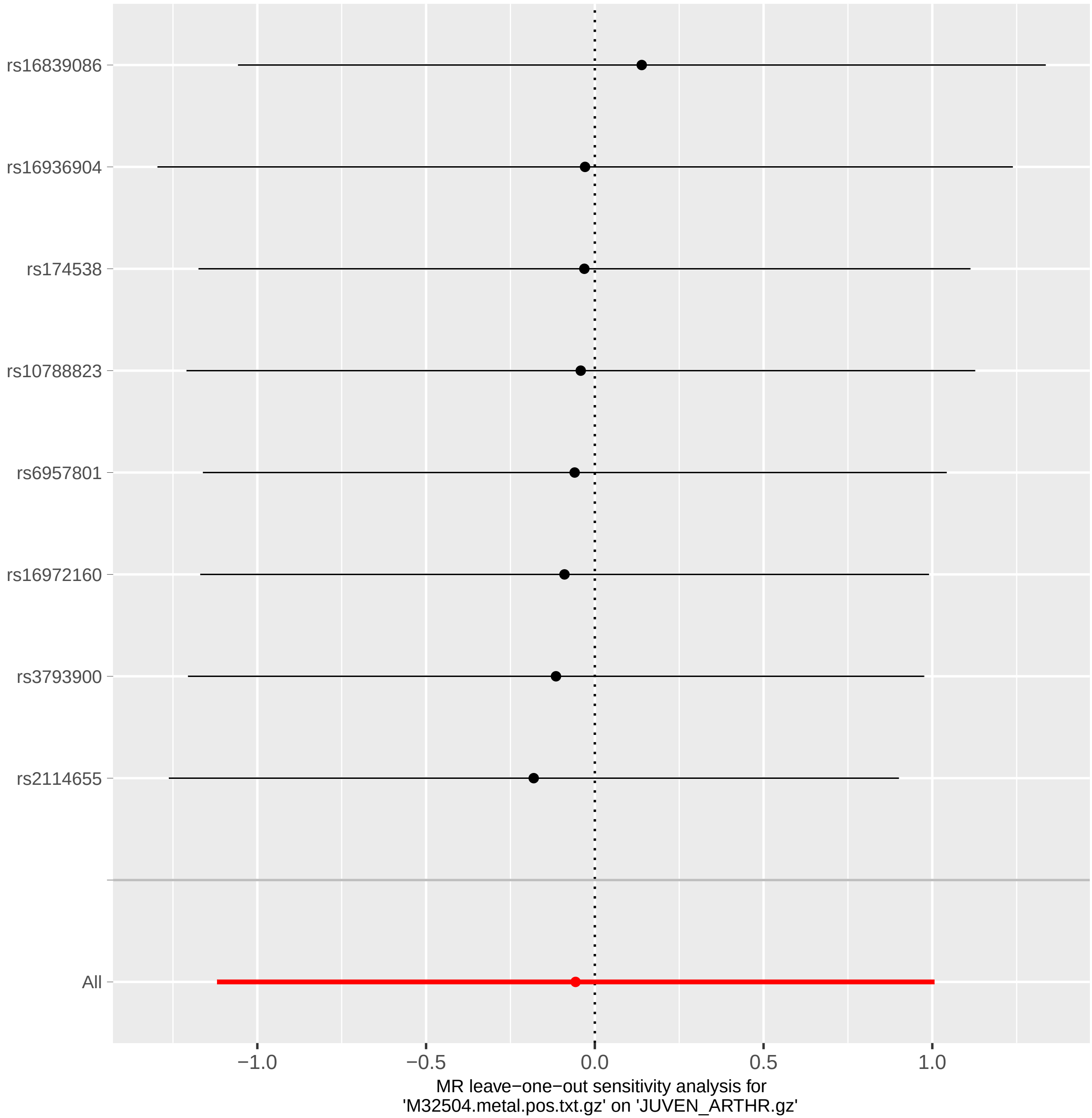

rs16839086
rs16936904
rs174538
rs10788823
rs6957801
rs16972160
rs3793900
rs2114655
All
−1.0
−0.5
0.0
0.5
1.0
MR leave−one−out sensitivity analysis for
'M32504.metal.pos.txt.gz' on 'JUVEN_ARTHR.gz'

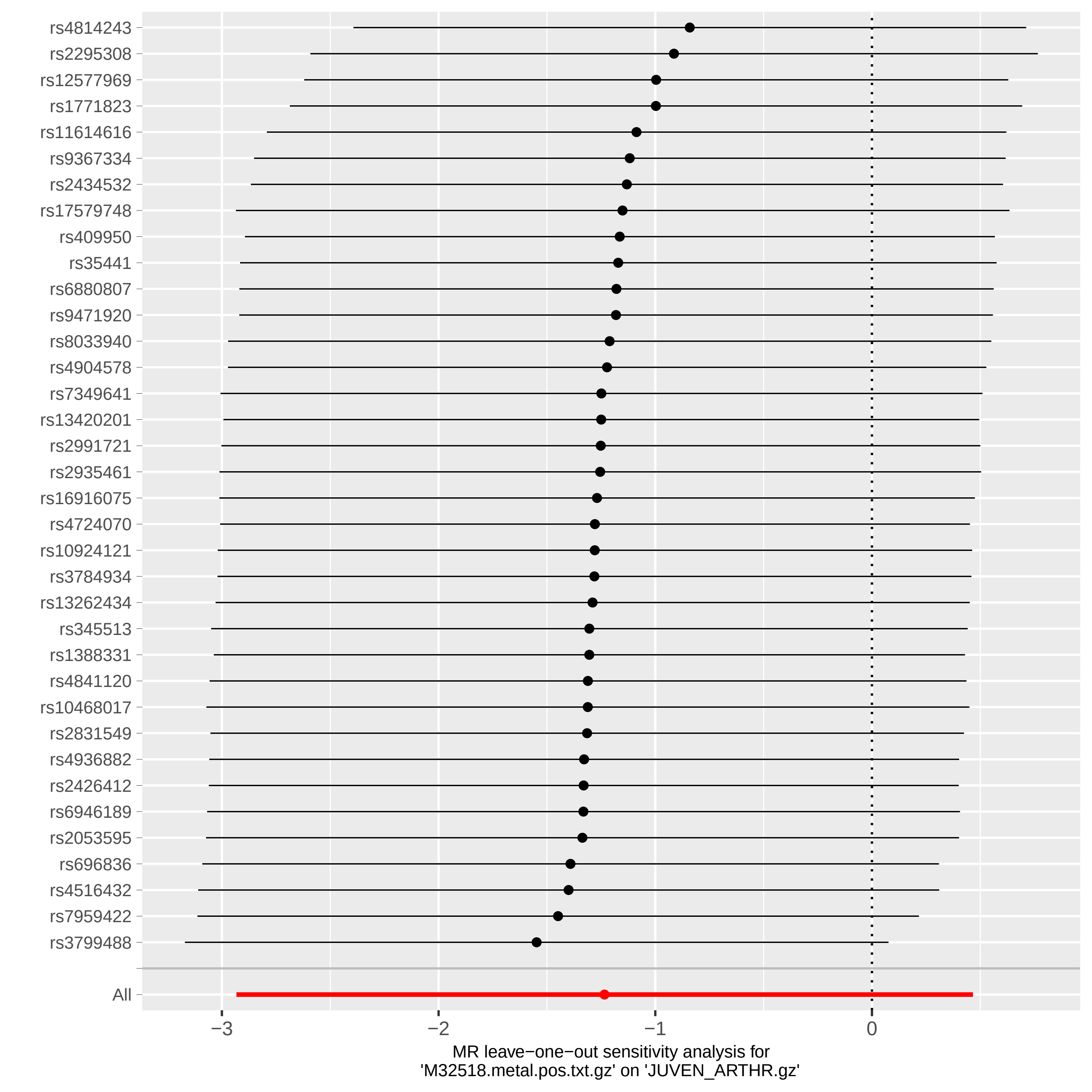

rs4814243
rs2295308
rs12577969
rs1771823
rs11614616
rs9367334
rs2434532
rs17579748
rs409950
rs35441
rs6880807
rs9471920
rs8033940
rs4904578
rs7349641
rs13420201
rs2991721
rs2935461
rs16916075
rs4724070
rs10924121
rs3784934
rs13262434
rs345513
rs1388331
rs4841120
rs10468017
rs2831549
rs4936882
rs2426412
rs6946189
rs2053595
rs696836
rs4516432
rs7959422
rs3799488
All
−3
−2
−1
0
MR leave−one−out sensitivity analysis for
'M32518.metal.pos.txt.gz' on 'JUVEN_ARTHR.gz'

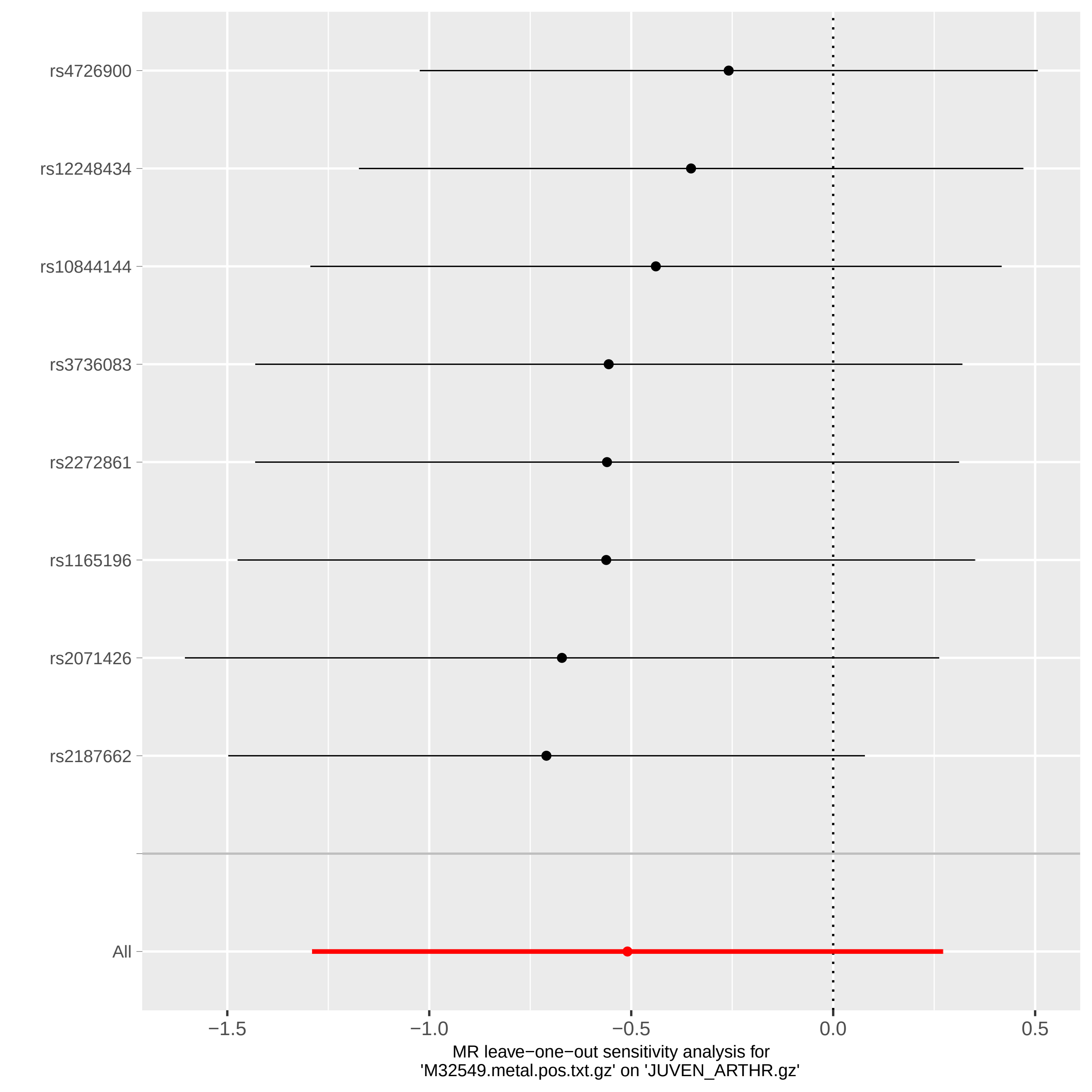
rs4726900
rs12248434
rs10844144
rs3736083
rs2272861
rs1165196
rs2071426
rs2187662
All
−1.5
−1.0
−0.5
0.0
0.5
MR leave−one−out sensitivity analysis for
'M32549.metal.pos.txt.gz' on 'JUVEN_ARTHR.gz'

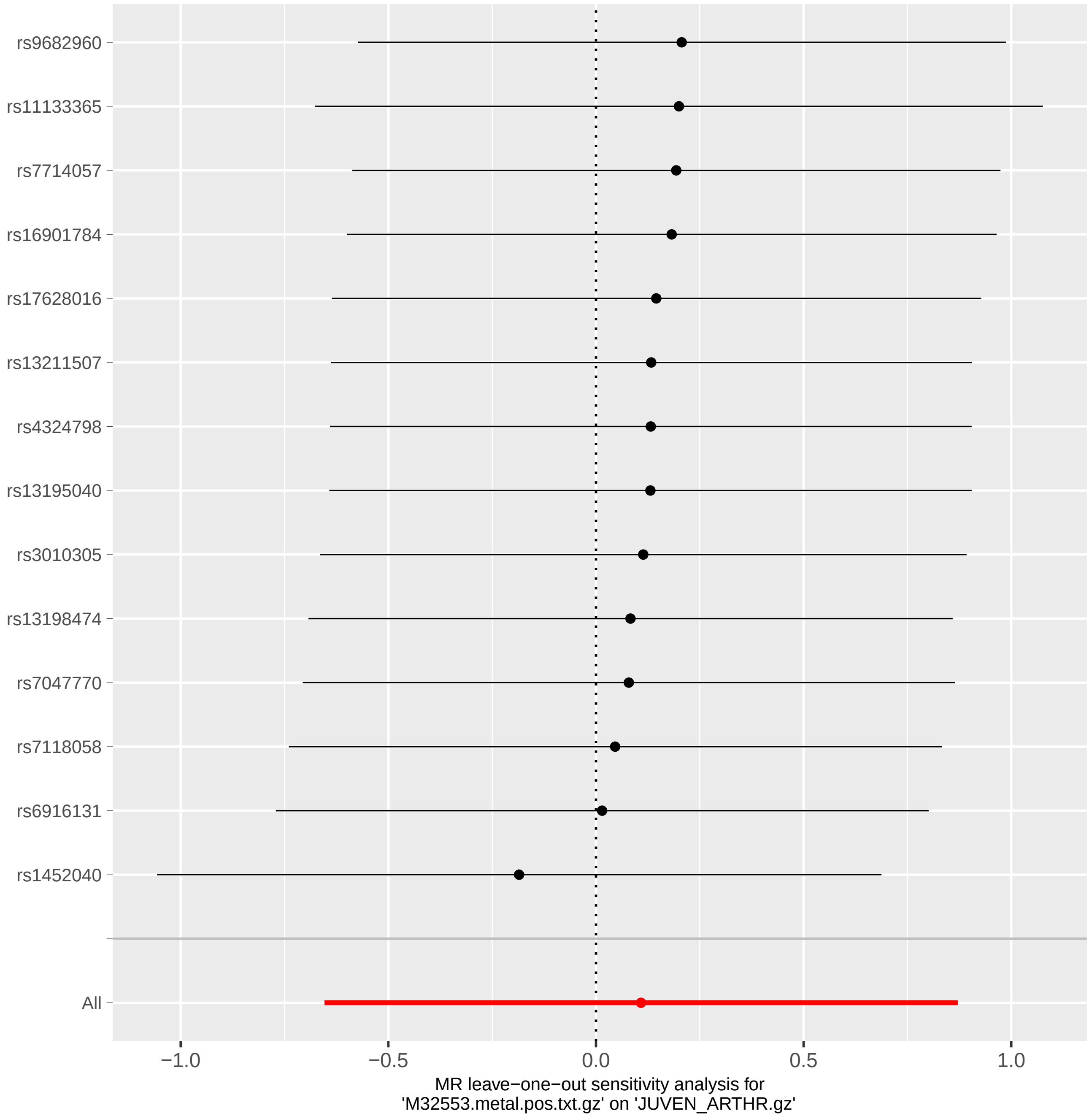
rs9682960
rs11133365
rs7714057
rs16901784
rs17628016
rs13211507
rs4324798
rs13195040
rs3010305
rs13198474
rs7047770
rs7118058
rs6916131
rs1452040
All
−1.0
−0.5
0.0
0.5
1.0
MR leave−one−out sensitivity analysis for
'M32553.metal.pos.txt.gz' on 'JUVEN_ARTHR.gz'

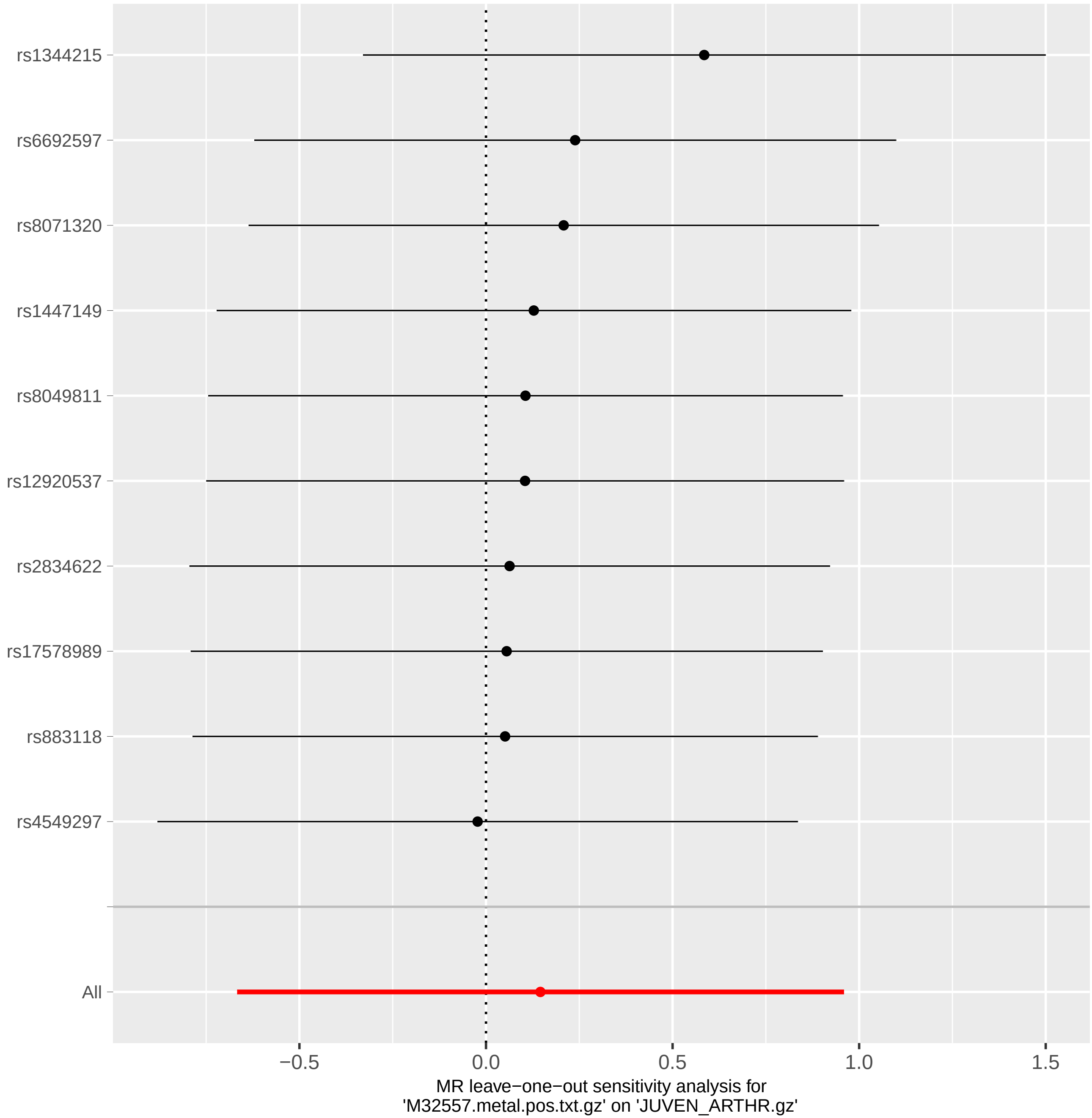

rs1344215
rs6692597
rs8071320
rs1447149
rs8049811
rs12920537
rs2834622
rs17578989
rs883118
rs4549297
All
−0.5
0.0
0.5
1.0
1.5
MR leave−one−out sensitivity analysis for
'M32557.metal.pos.txt.gz' on 'JUVEN_ARTHR.gz'

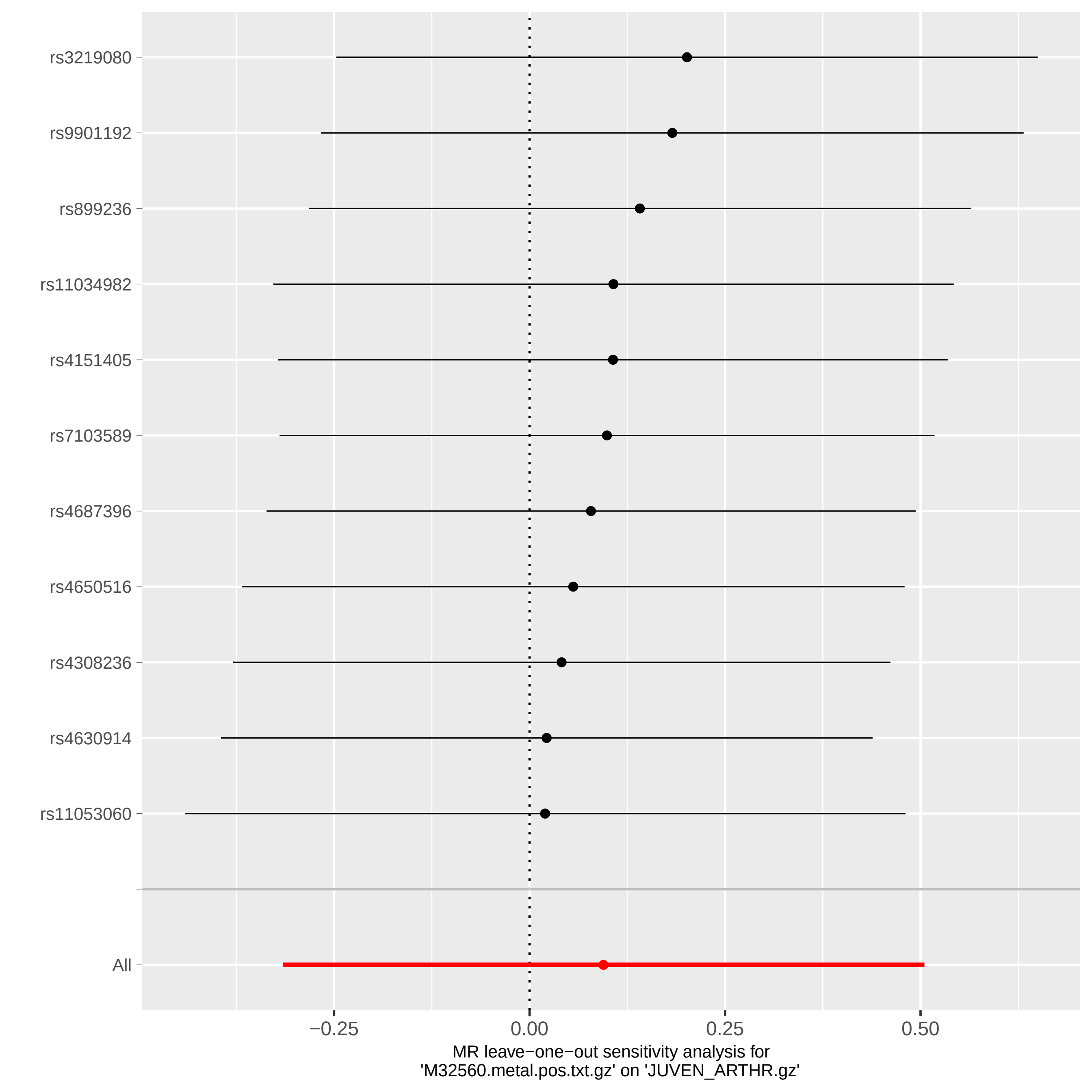

rs3219080
rs9901192
rs899236
rs11034982
rs4151405
rs7103589
rs4687396
rs4650516
rs4308236
rs4630914
rs11053060
All
−0.25
0.00
0.25
0.50
MR leave−one−out sensitivity analysis for
'M32560.metal.pos.txt.gz' on 'JUVEN_ARTHR.gz'

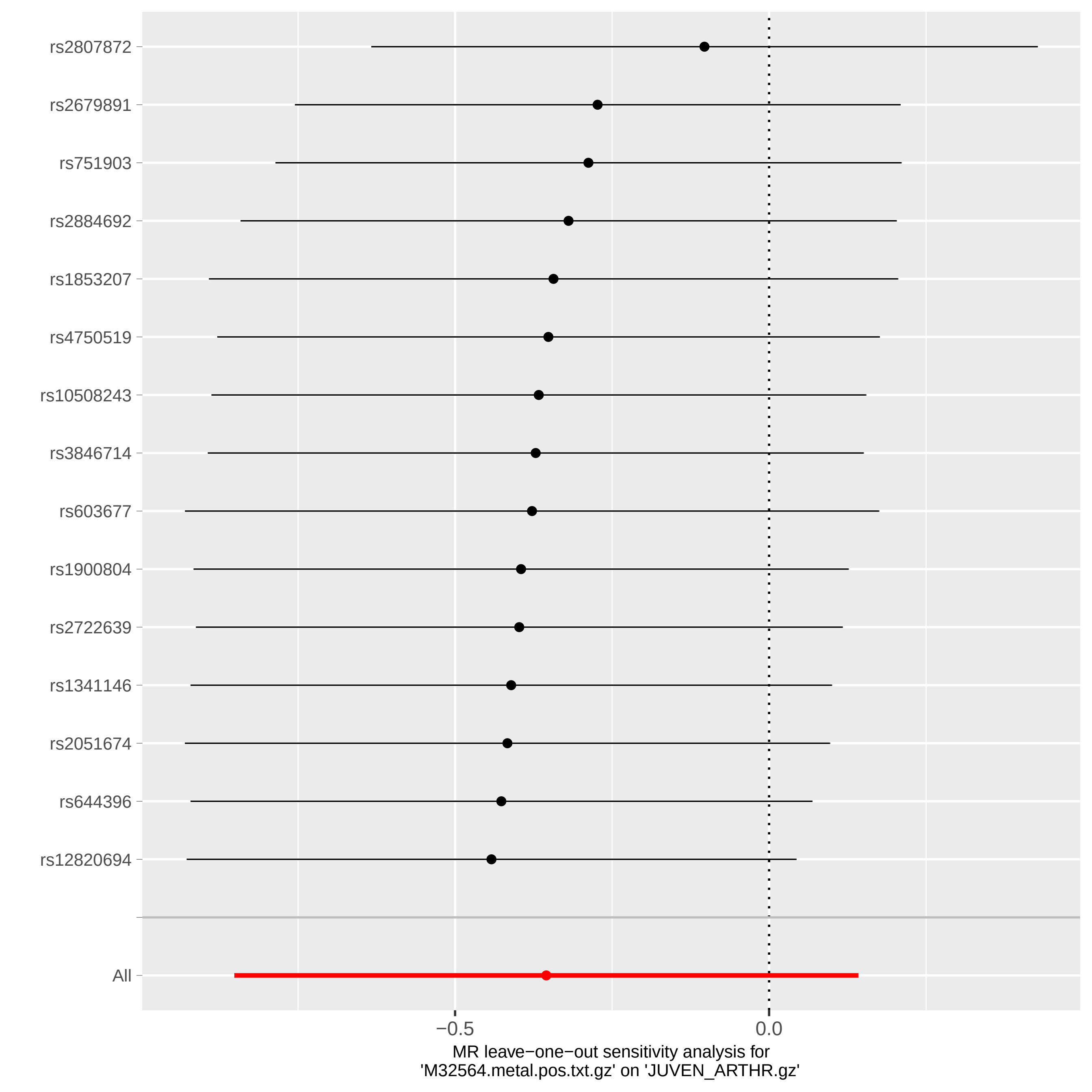

rs2807872
rs2679891
rs751903
rs2884692
rs1853207
rs4750519
rs10508243
rs3846714
rs603677
rs1900804
rs2722639
rs1341146
rs2051674
rs644396
rs12820694
All
−0.5
0.0
MR leave−one−out sensitivity analysis for
'M32564.metal.pos.txt.gz' on 'JUVEN_ARTHR.gz'

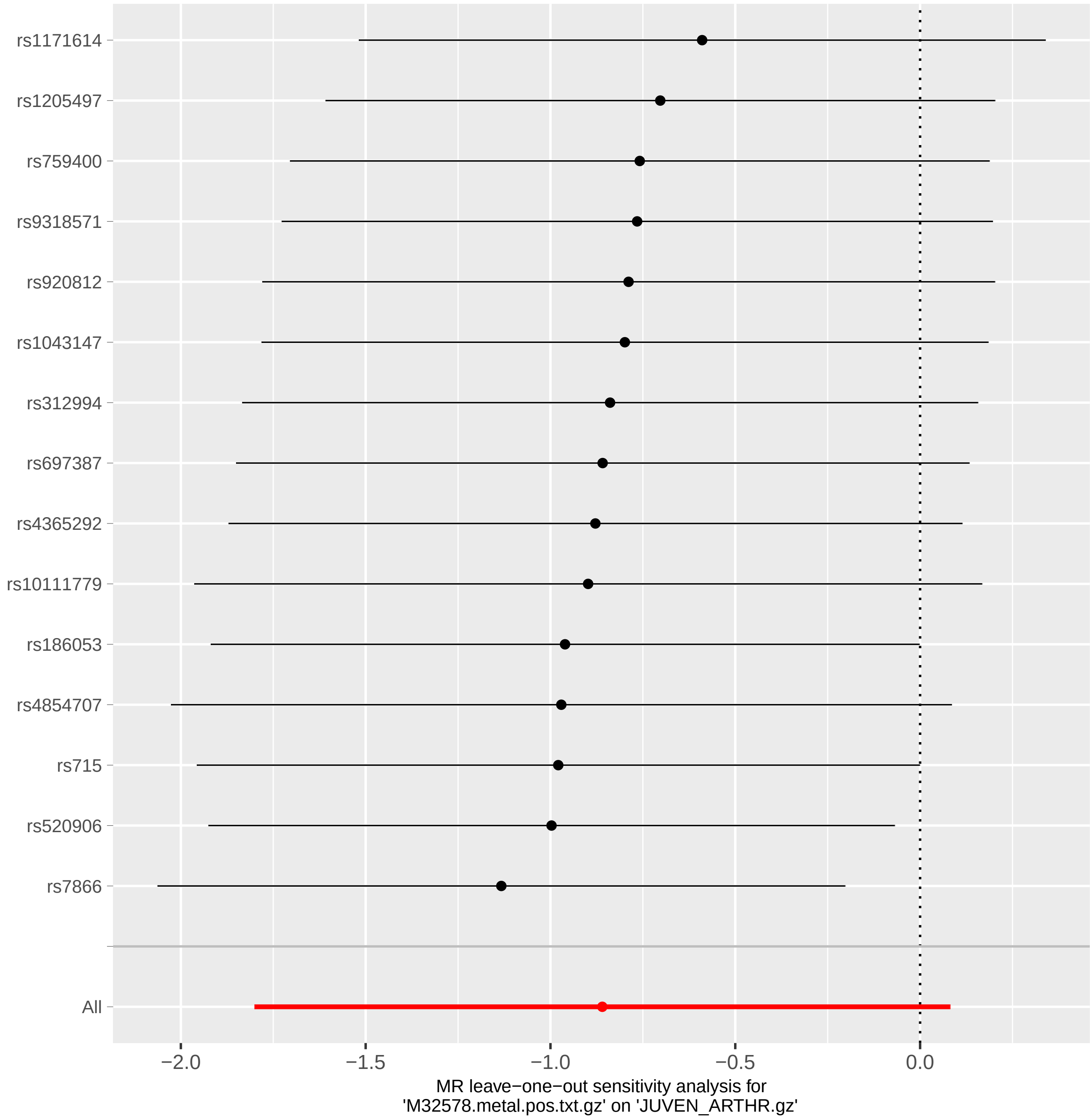
rs1171614
rs1205497
rs759400
rs9318571
rs920812
rs1043147
rs312994
rs697387
rs4365292
rs10111779
rs186053
rs4854707
rs715
rs520906
rs7866
All
−2.0
−1.5
−1.0
−0.5
0.0
MR leave−one−out sensitivity analysis for
'M32578.metal.pos.txt.gz' on 'JUVEN_ARTHR.gz'

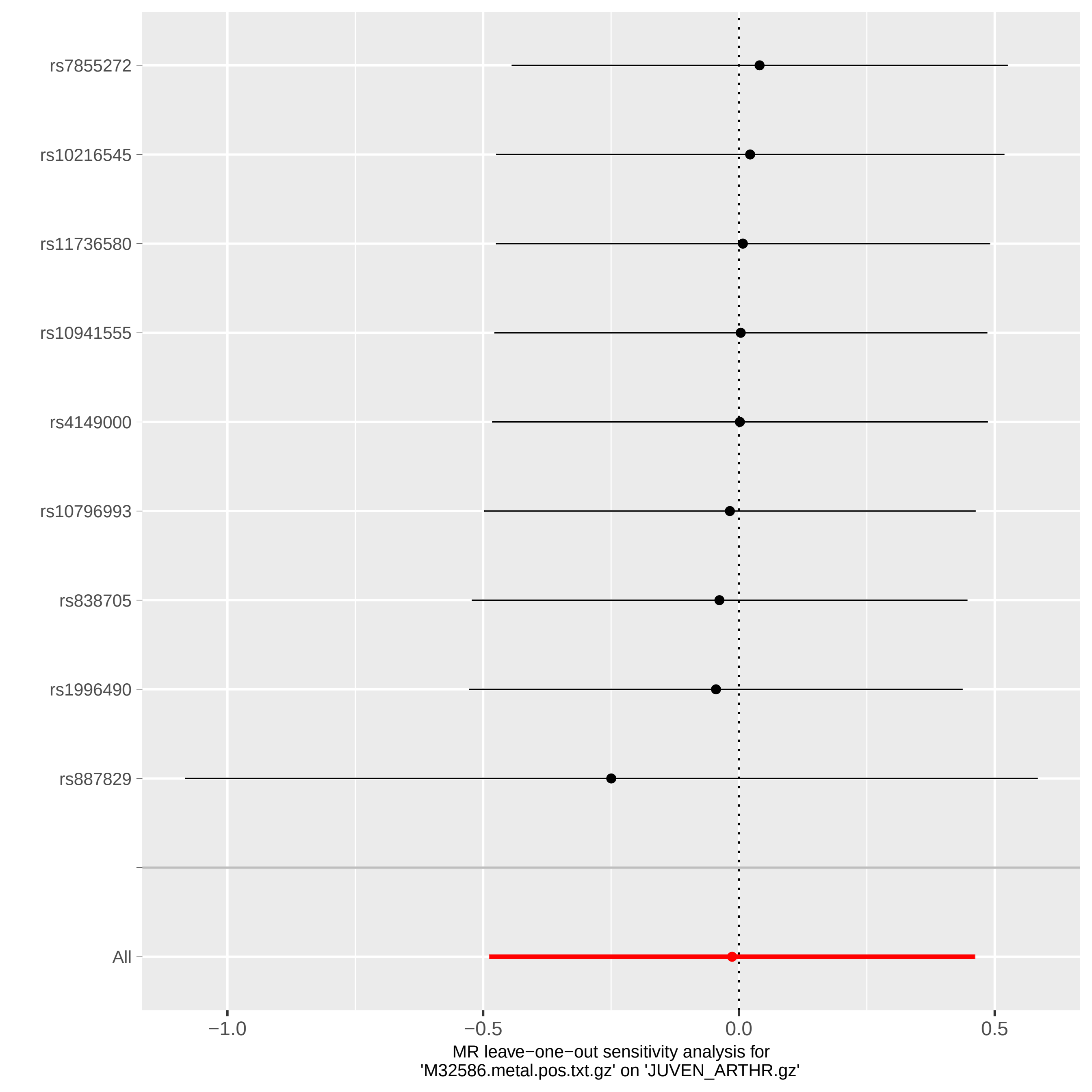
rs7855272
rs10216545
rs11736580
rs10941555
rs4149000
rs10796993
rs838705
rs1996490
rs887829
All
−1.0
−0.5
0.0
0.5
MR leave−one−out sensitivity analysis for
'M32586.metal.pos.txt.gz' on 'JUVEN_ARTHR.gz'

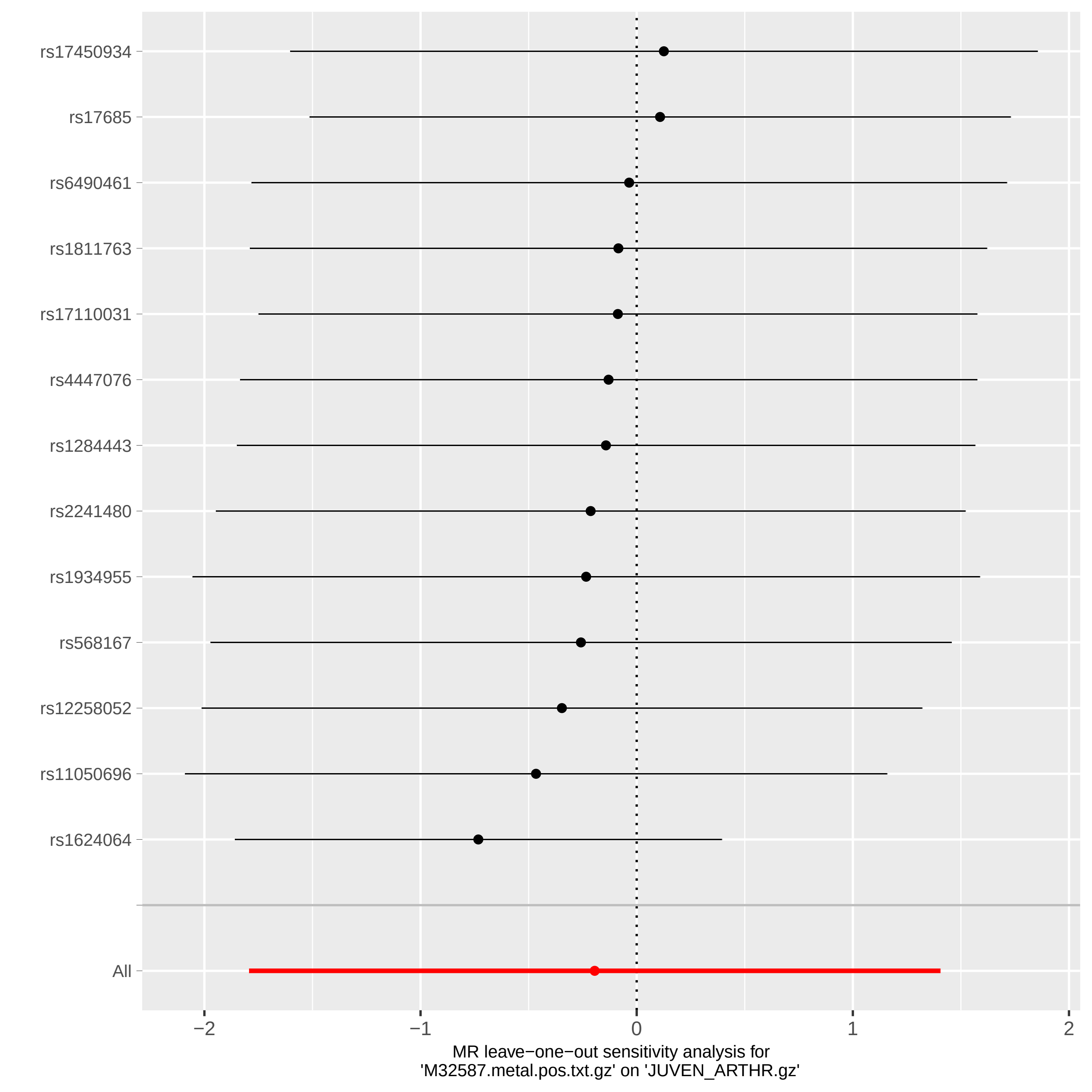

rs17450934
rs17685
rs6490461
rs1811763
rs17110031
rs4447076
rs1284443
rs2241480
rs1934955
rs568167
rs12258052
rs11050696
rs1624064
All
−2
−1
0
1
2
MR leave−one−out sensitivity analysis for
'M32587.metal.pos.txt.gz' on 'JUVEN_ARTHR.gz'

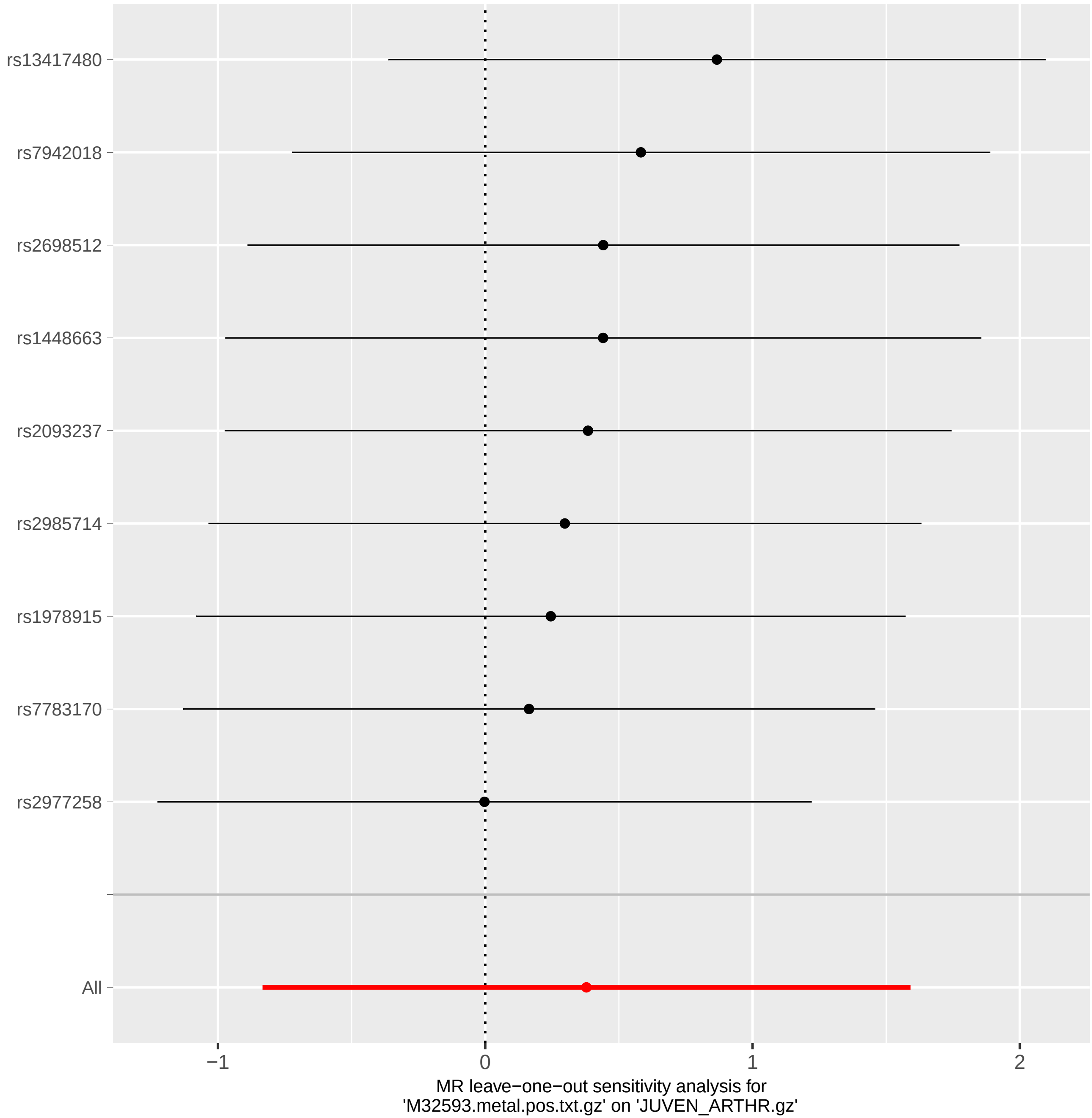
rs13417480
rs7942018
rs2698512
rs1448663
rs2093237
rs2985714
rs1978915
rs7783170
rs2977258
All
−1
0
1
2
MR leave−one−out sensitivity analysis for
'M32593.metal.pos.txt.gz' on 'JUVEN_ARTHR.gz'

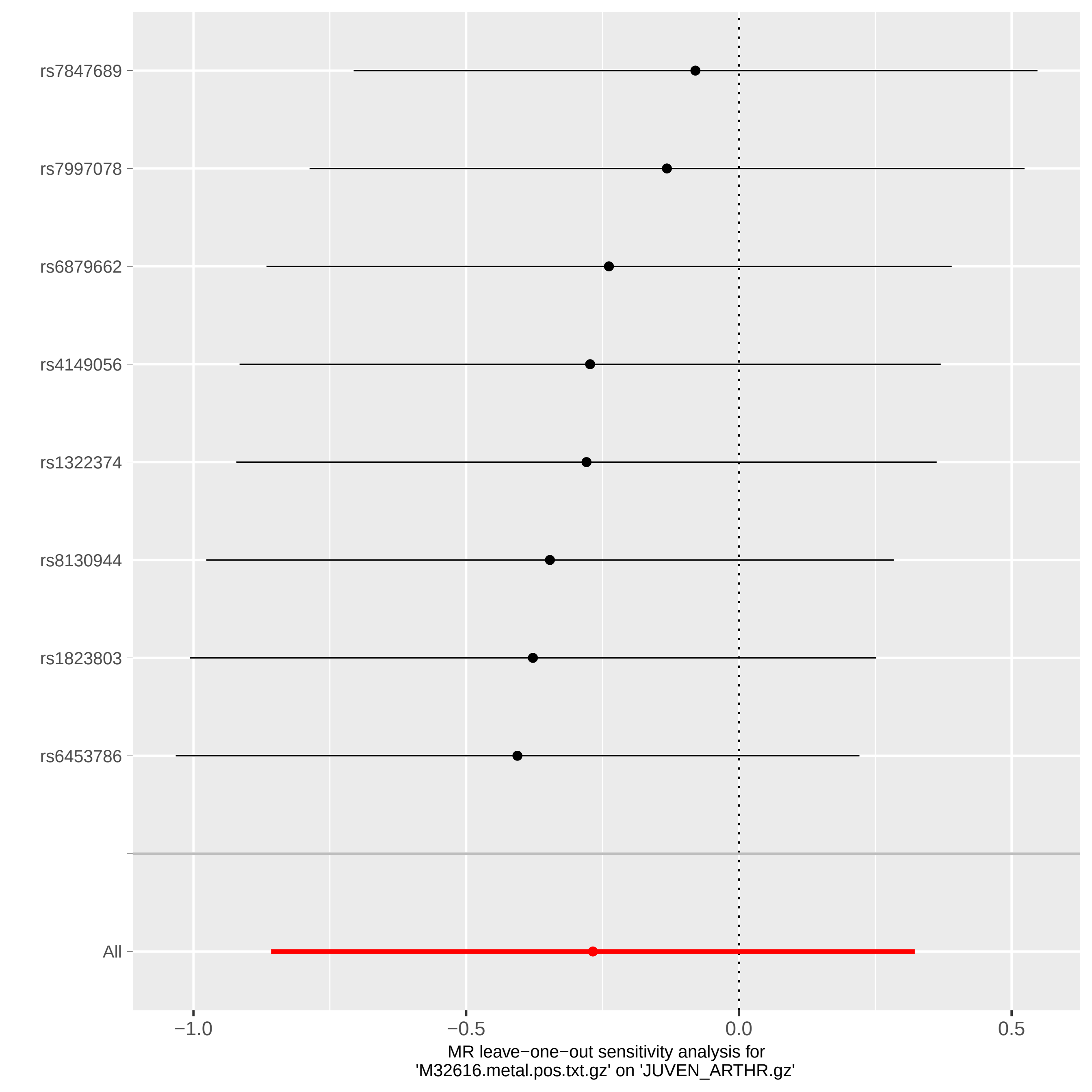
rs7847689
rs7997078
rs6879662
rs4149056
rs1322374
rs8130944
rs1823803
rs6453786
All
−1.0
−0.5
0.0
0.5
MR leave−one−out sensitivity analysis for
'M32616.metal.pos.txt.gz' on 'JUVEN_ARTHR.gz'

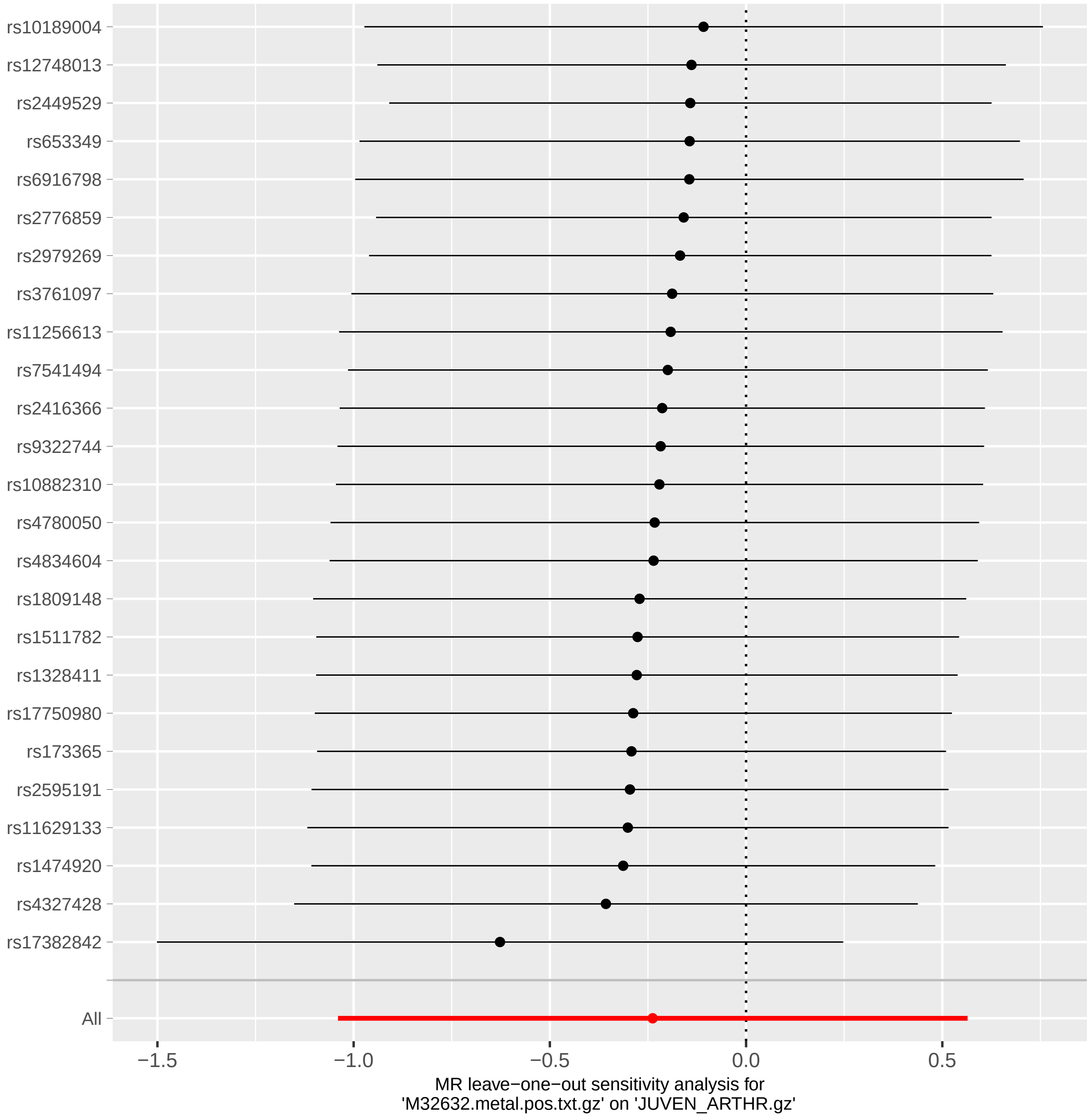

rs10189004
rs12748013
rs2449529
rs653349
rs6916798
rs2776859
rs2979269
rs3761097
rs11256613
rs7541494
rs2416366
rs9322744
rs10882310
rs4780050
rs4834604
rs1809148
rs1511782
rs1328411
rs17750980
rs173365
rs2595191
rs11629133
rs1474920
rs4327428
rs17382842
All
−1.5
−1.0
−0.5
0.0
0.5
MR leave−one−out sensitivity analysis for
'M32632.metal.pos.txt.gz' on 'JUVEN_ARTHR.gz'

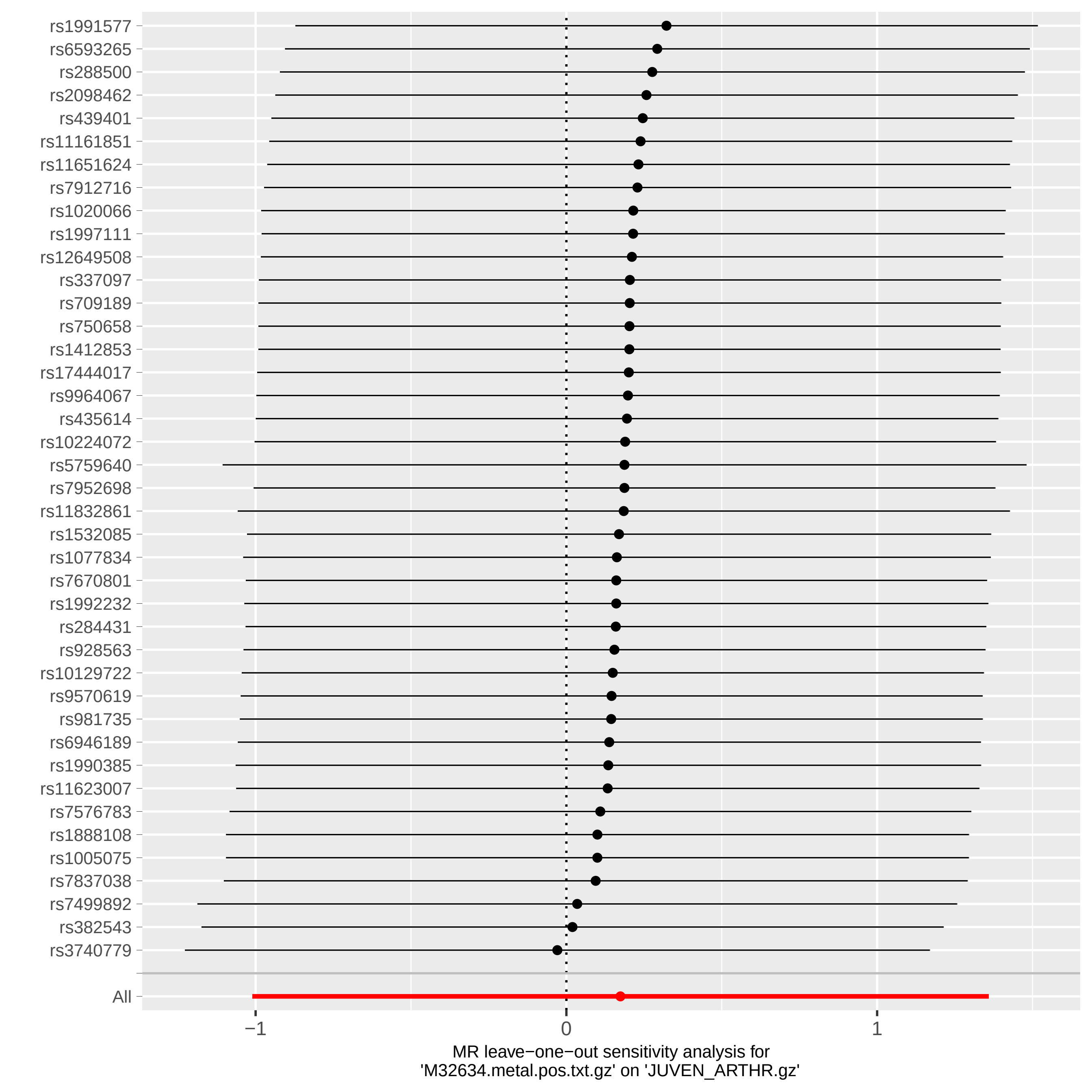

rs1991577
rs6593265
rs288500
rs2098462
rs439401
rs11161851
rs11651624
rs7912716
rs1020066
rs1997111
rs12649508
rs337097
rs709189
rs750658
rs1412853
rs17444017
rs9964067
rs435614
rs10224072
rs5759640
rs7952698
rs11832861
rs1532085
rs1077834
rs7670801
rs1992232
rs284431
rs928563
rs10129722
rs9570619
rs981735
rs6946189
rs1990385
rs11623007
rs7576783
rs1888108
rs1005075
rs7837038
rs7499892
rs382543
rs3740779
All
−1
0
1
MR leave−one−out sensitivity analysis for
'M32634.metal.pos.txt.gz' on 'JUVEN_ARTHR.gz'

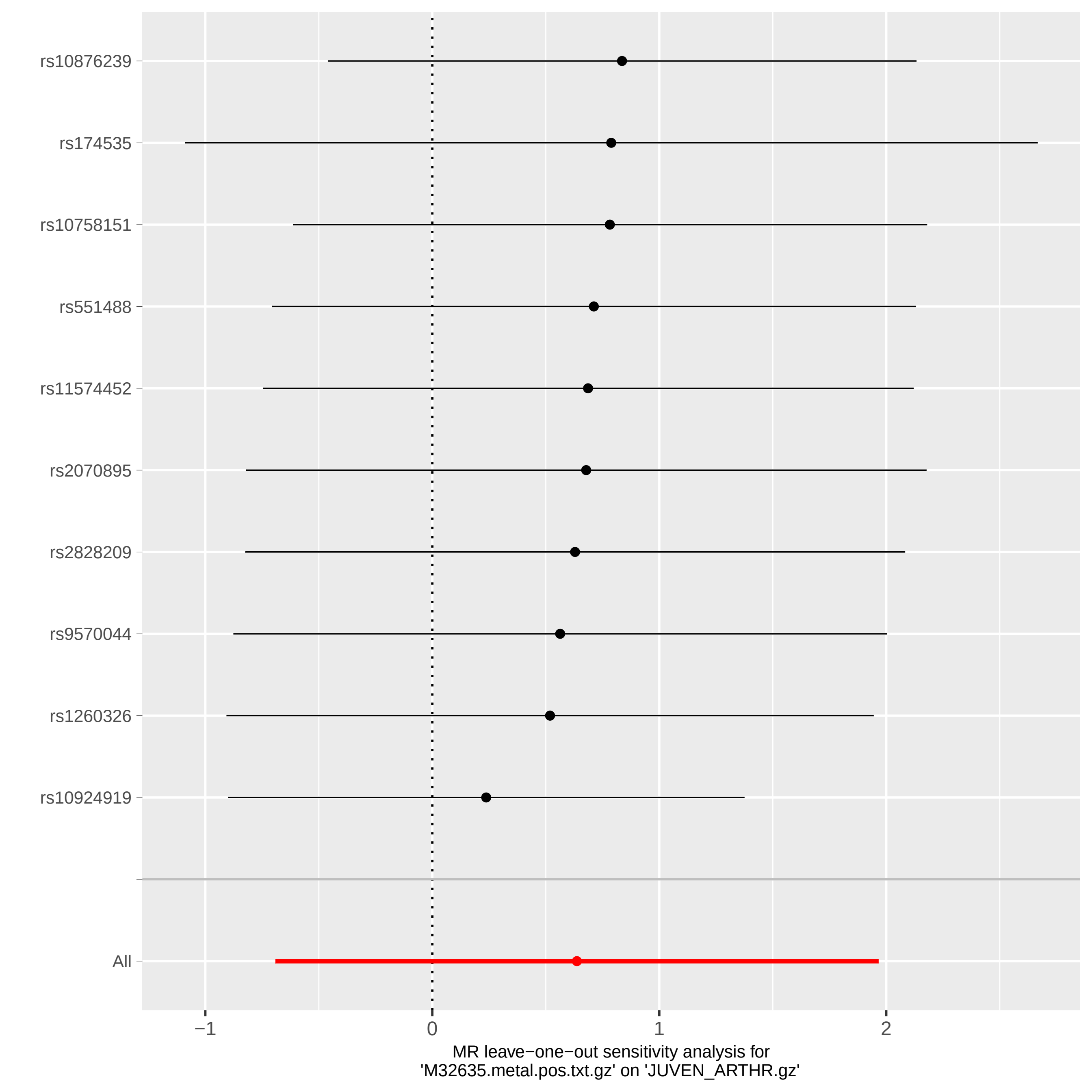

rs10876239
rs174535
rs10758151
rs551488
rs11574452
rs2070895
rs2828209
rs9570044
rs1260326
rs10924919
All
−1
0
1
2
MR leave−one−out sensitivity analysis for
'M32635.metal.pos.txt.gz' on 'JUVEN_ARTHR.gz'

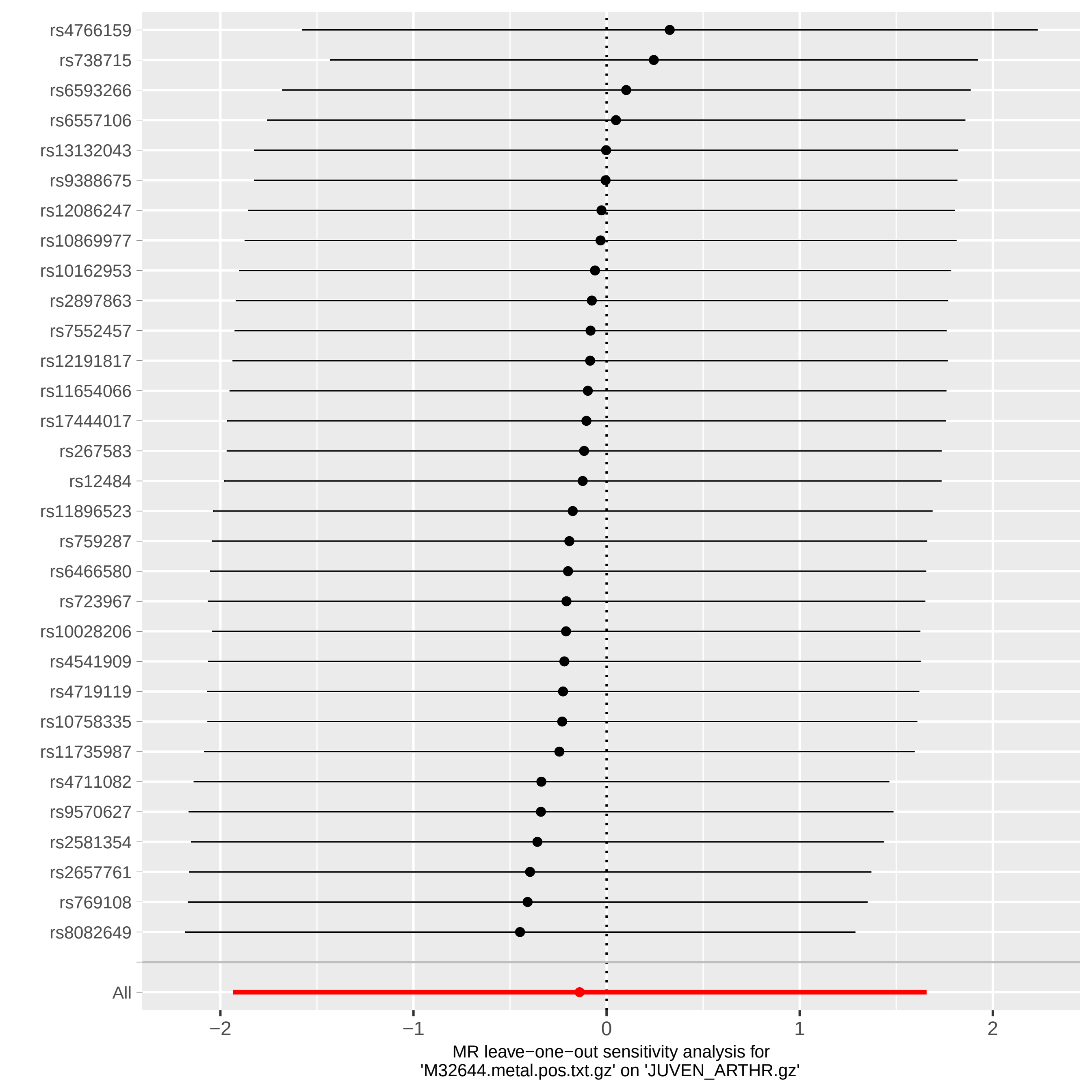
rs4766159
rs738715
rs6593266
rs6557106
rs13132043
rs9388675
rs12086247
rs10869977
rs10162953
rs2897863
rs7552457
rs12191817
rs11654066
rs17444017
rs267583
rs12484
rs11896523
rs759287
rs6466580
rs723967
rs10028206
rs4541909
rs4719119
rs10758335
rs11735987
rs4711082
rs9570627
rs2581354
rs2657761
rs769108
rs8082649
All
−2
−1
0
1
2
MR leave−one−out sensitivity analysis for
'M32644.metal.pos.txt.gz' on 'JUVEN_ARTHR.gz'

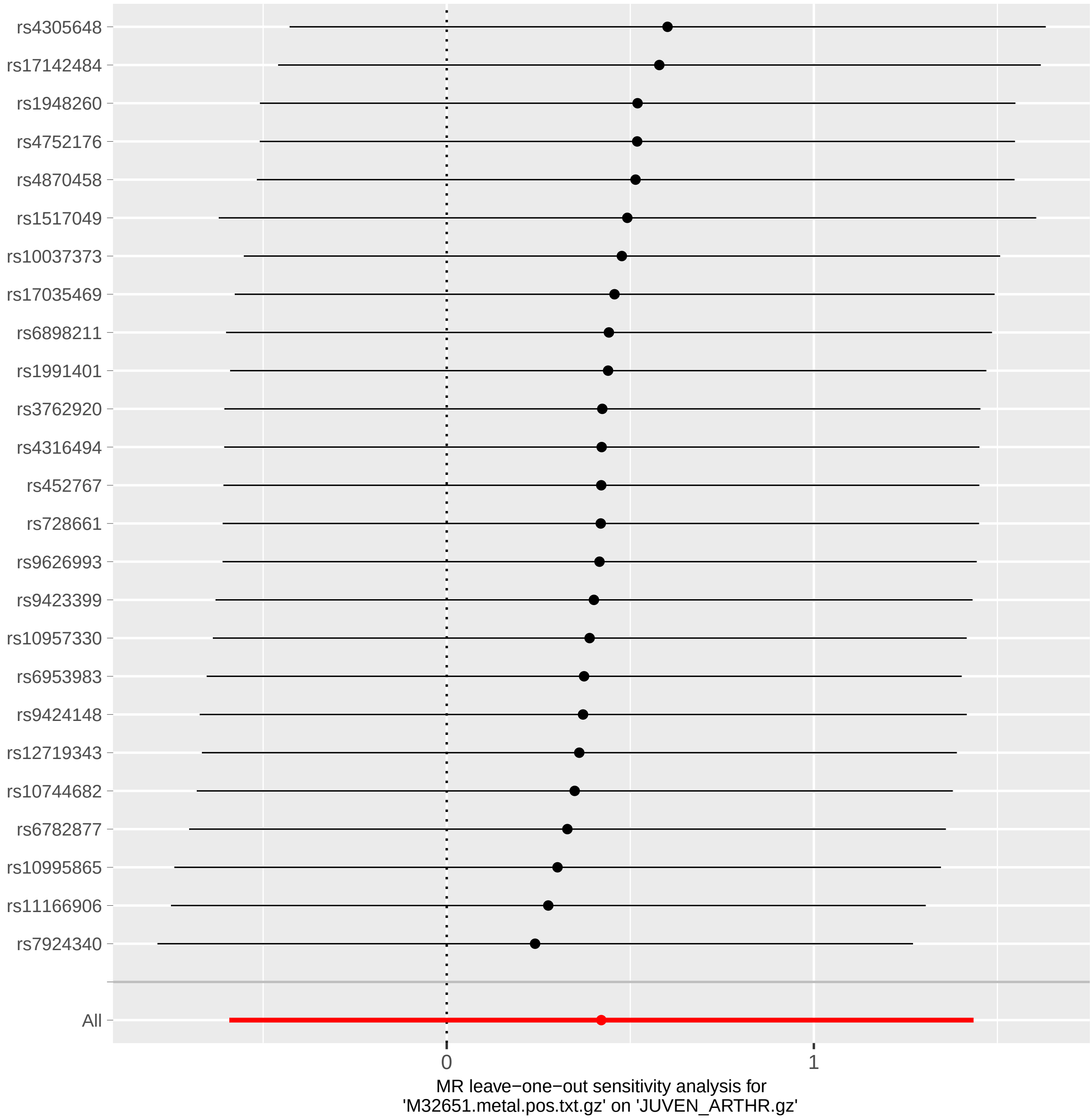

rs4305648
rs17142484
rs1948260
rs4752176
rs4870458
rs1517049
rs10037373
rs17035469
rs6898211
rs1991401
rs3762920
rs4316494
rs452767
rs728661
rs9626993
rs9423399
rs10957330
rs6953983
rs9424148
rs12719343
rs10744682
rs6782877
rs10995865
rs11166906
rs7924340
All
0
1
MR leave−one−out sensitivity analysis for
'M32651.metal.pos.txt.gz' on 'JUVEN_ARTHR.gz'

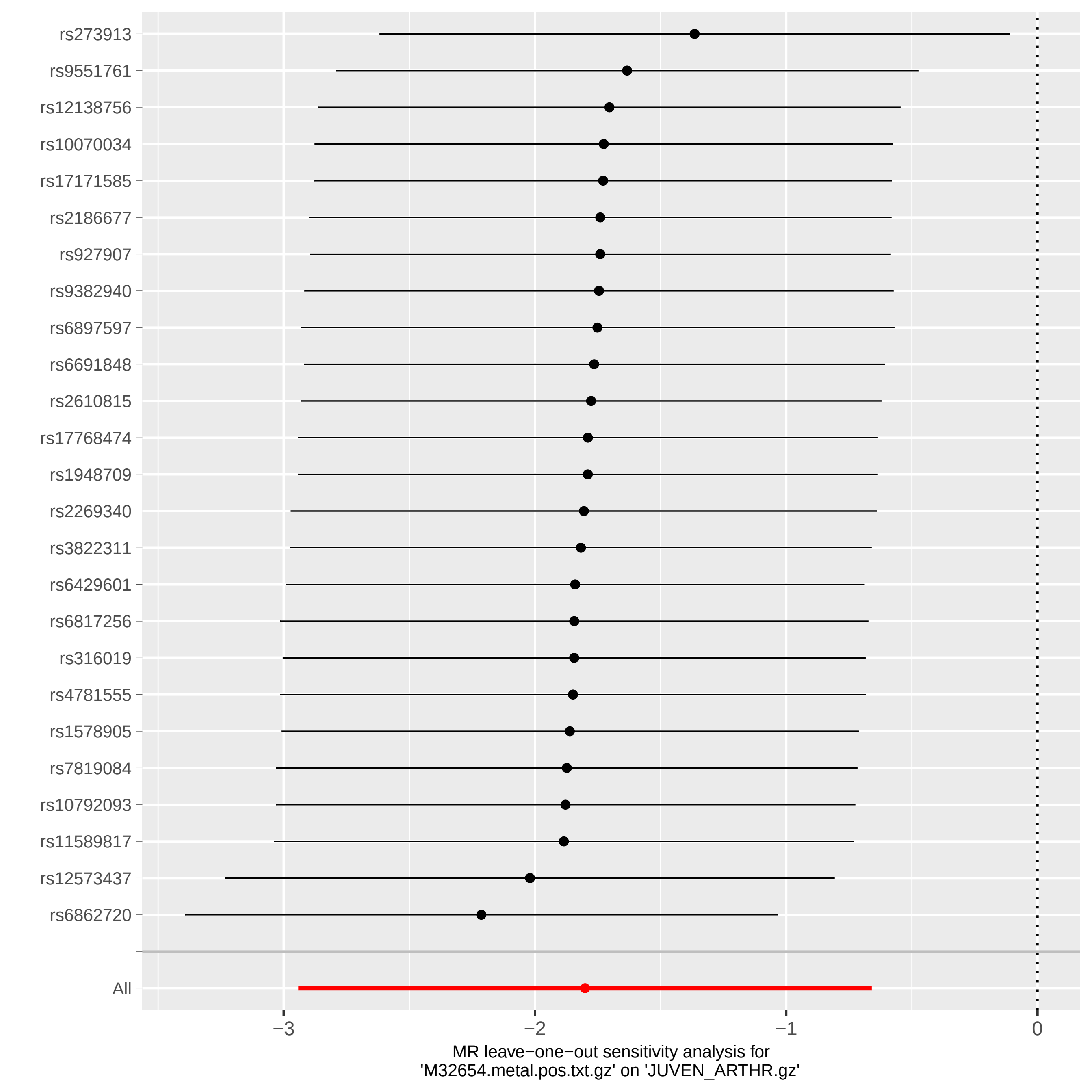

rs273913
rs9551761
rs12138756
rs10070034
rs17171585
rs2186677
rs927907
rs9382940
rs6897597
rs6691848
rs2610815
rs17768474
rs1948709
rs2269340
rs3822311
rs6429601
rs6817256
rs316019
rs4781555
rs1578905
rs7819084
rs10792093
rs11589817
rs12573437
rs6862720
All
−3
−2
−1
0
MR leave−one−out sensitivity analysis for
'M32654.metal.pos.txt.gz' on 'JUVEN_ARTHR.gz'

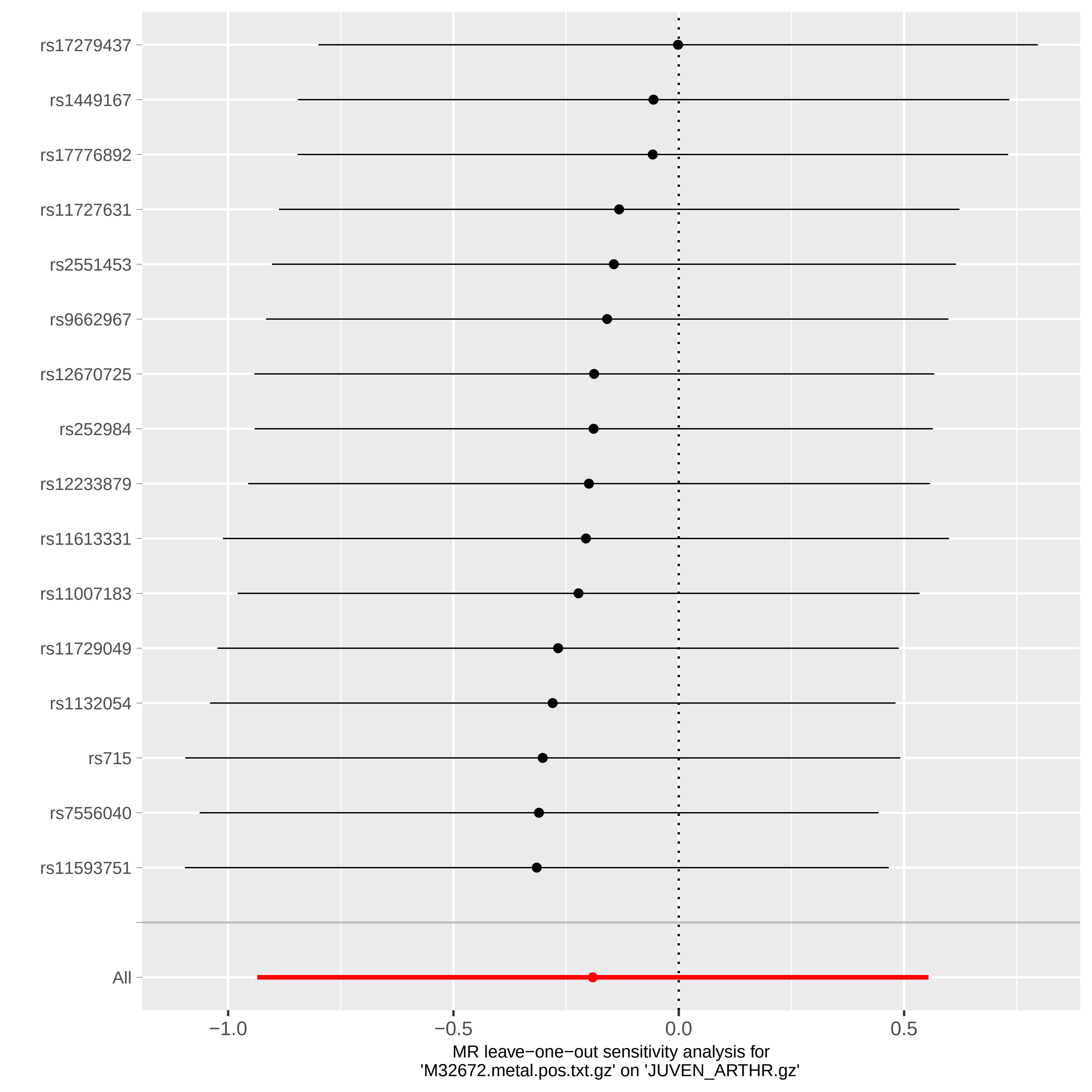
rs17279437
rs1449167
rs17776892
rs11727631
rs2551453
rs9662967
rs12670725
rs252984
rs12233879
rs11613331
rs11007183
rs11729049
rs1132054
rs715
rs7556040
rs11593751
All
−1.0
−0.5
0.0
0.5
MR leave−one−out sensitivity analysis for
'M32672.metal.pos.txt.gz' on 'JUVEN_ARTHR.gz'

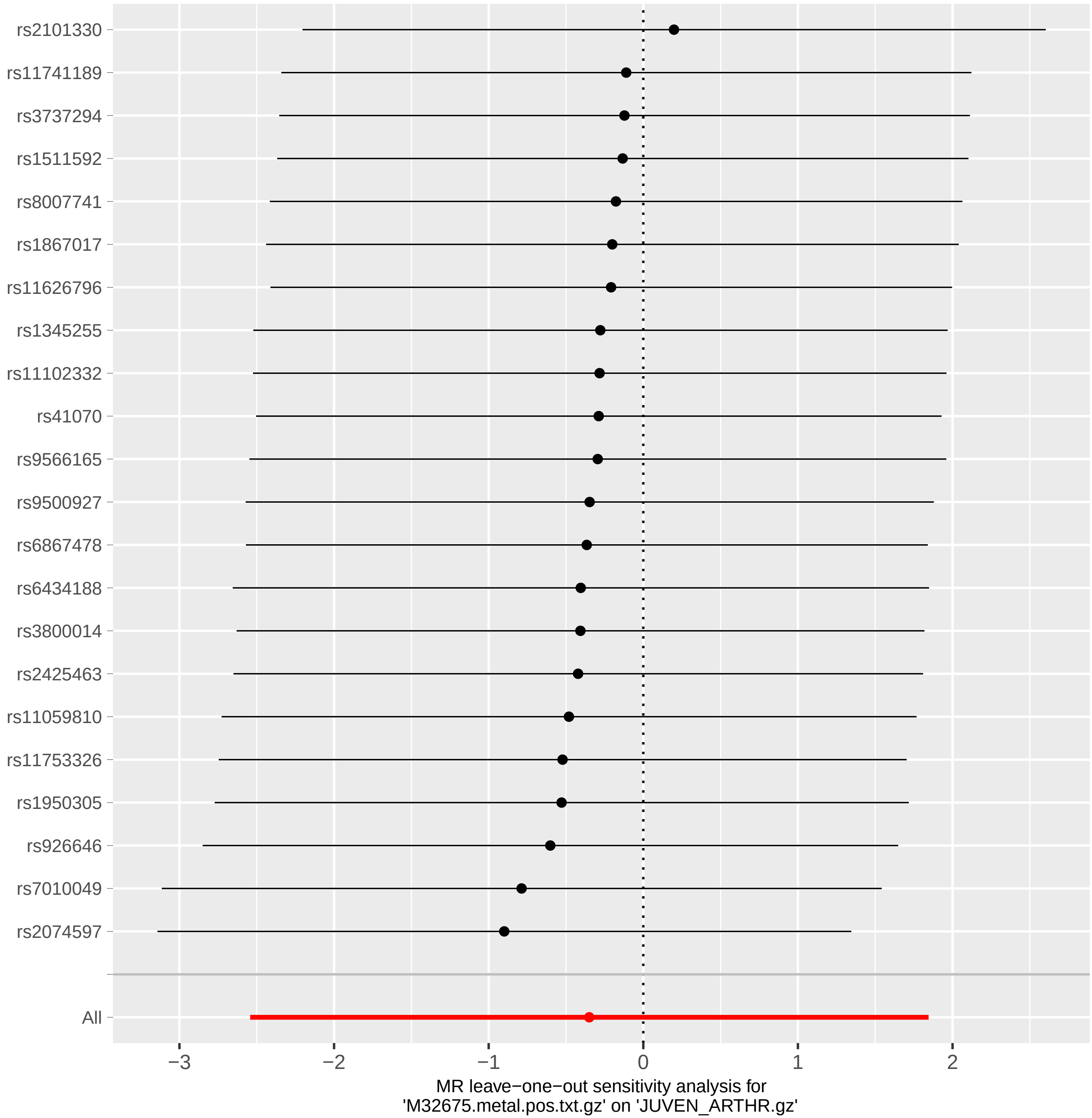

rs2101330
rs11741189
rs3737294
rs1511592
rs8007741
rs1867017
rs11626796
rs1345255
rs11102332
rs41070
rs9566165
rs9500927
rs6867478
rs6434188
rs3800014
rs2425463
rs11059810
rs11753326
rs1950305
rs926646
rs7010049
rs2074597
All
−3
−2
−1
0
1
2
MR leave−one−out sensitivity analysis for
'M32675.metal.pos.txt.gz' on 'JUVEN_ARTHR.gz'

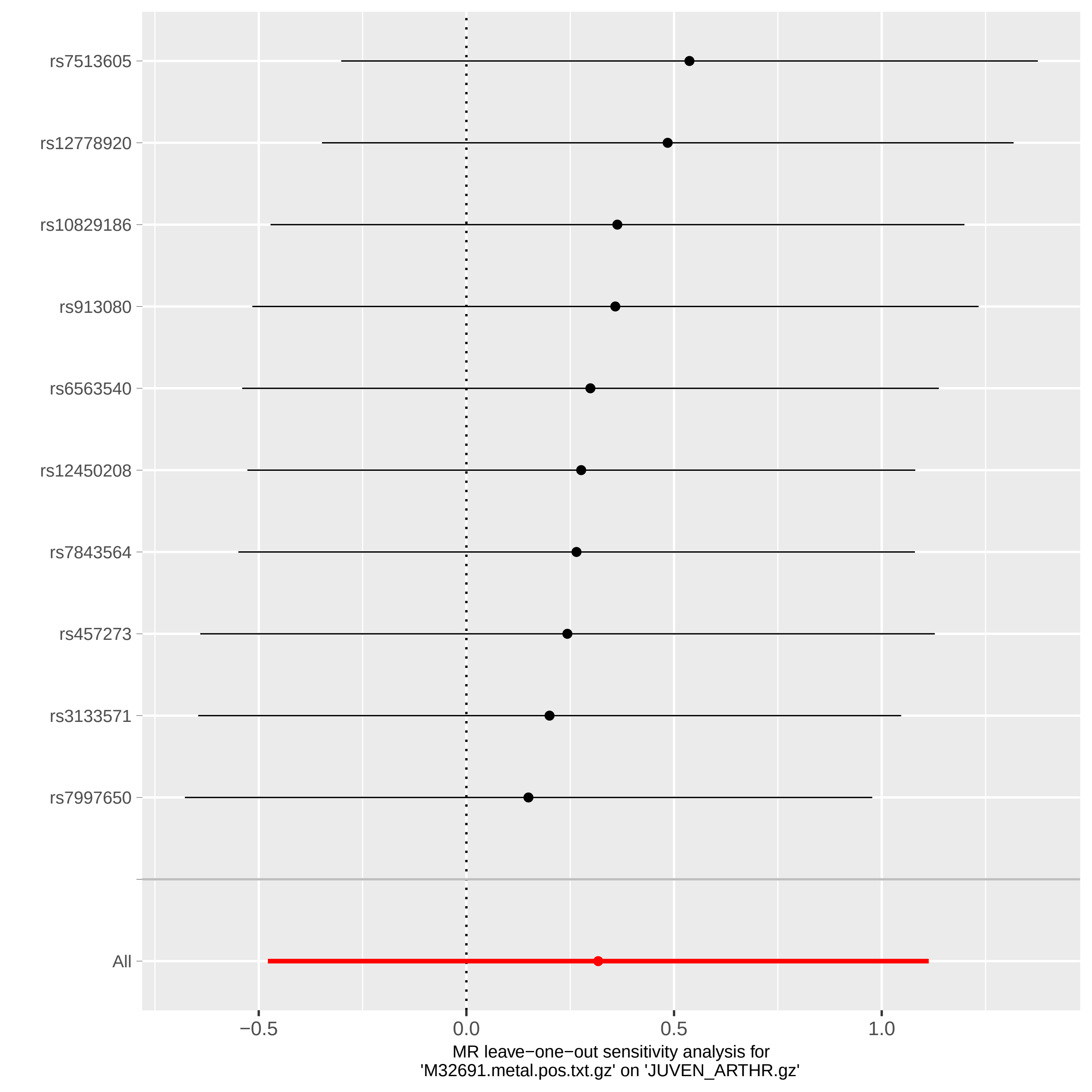

rs7513605
rs12778920
rs10829186
rs913080
rs6563540
rs12450208
rs7843564
rs457273
rs3133571
rs7997650
All
−0.5
0.0
0.5
1.0
MR leave−one−out sensitivity analysis for
'M32691.metal.pos.txt.gz' on 'JUVEN_ARTHR.gz'

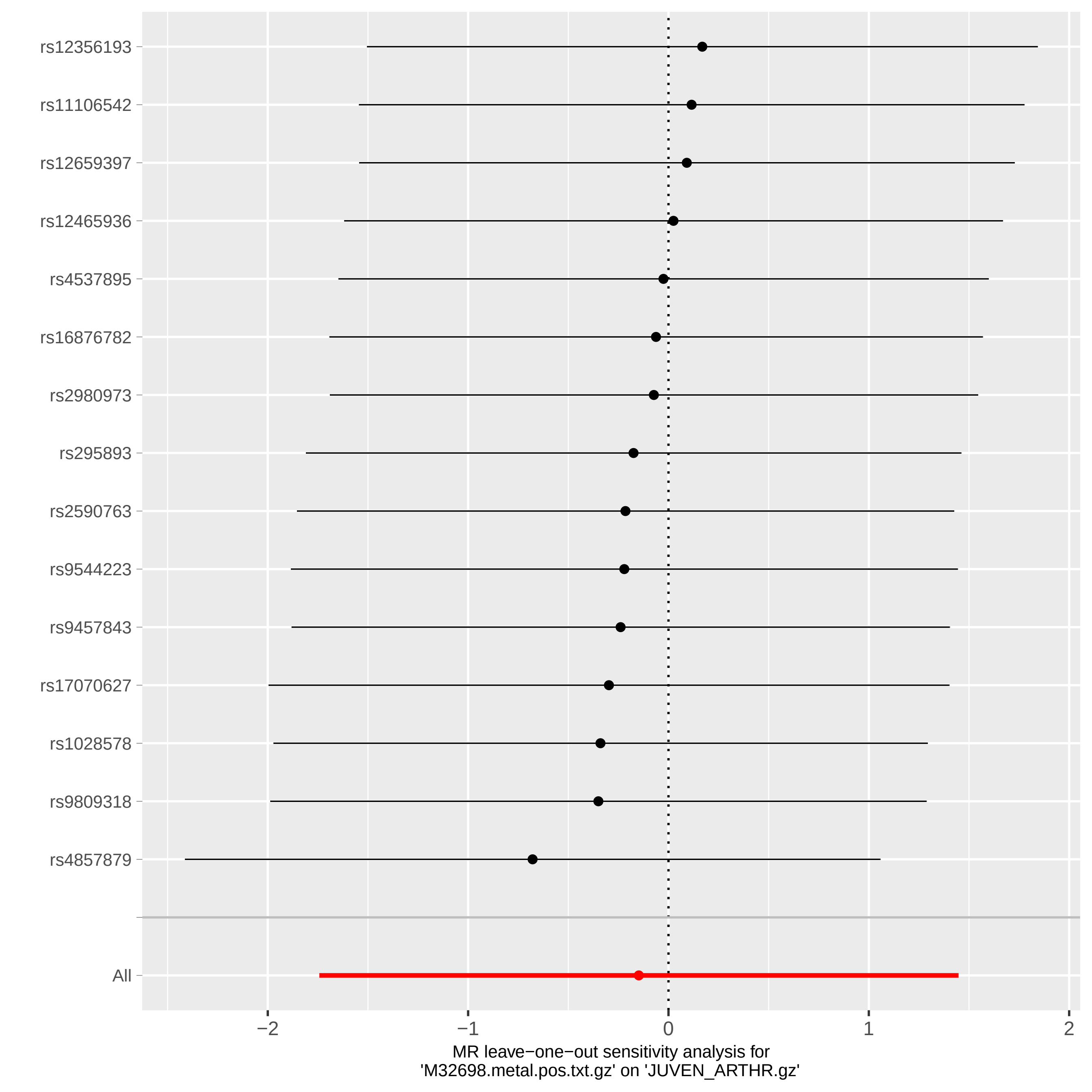
rs12356193
rs11106542
rs12659397
rs12465936
rs4537895
rs16876782
rs2980973
rs295893
rs2590763
rs9544223
rs9457843
rs17070627
rs1028578
rs9809318
rs4857879
All
−2
−1
0
1
2
MR leave−one−out sensitivity analysis for
'M32698.metal.pos.txt.gz' on 'JUVEN_ARTHR.gz'

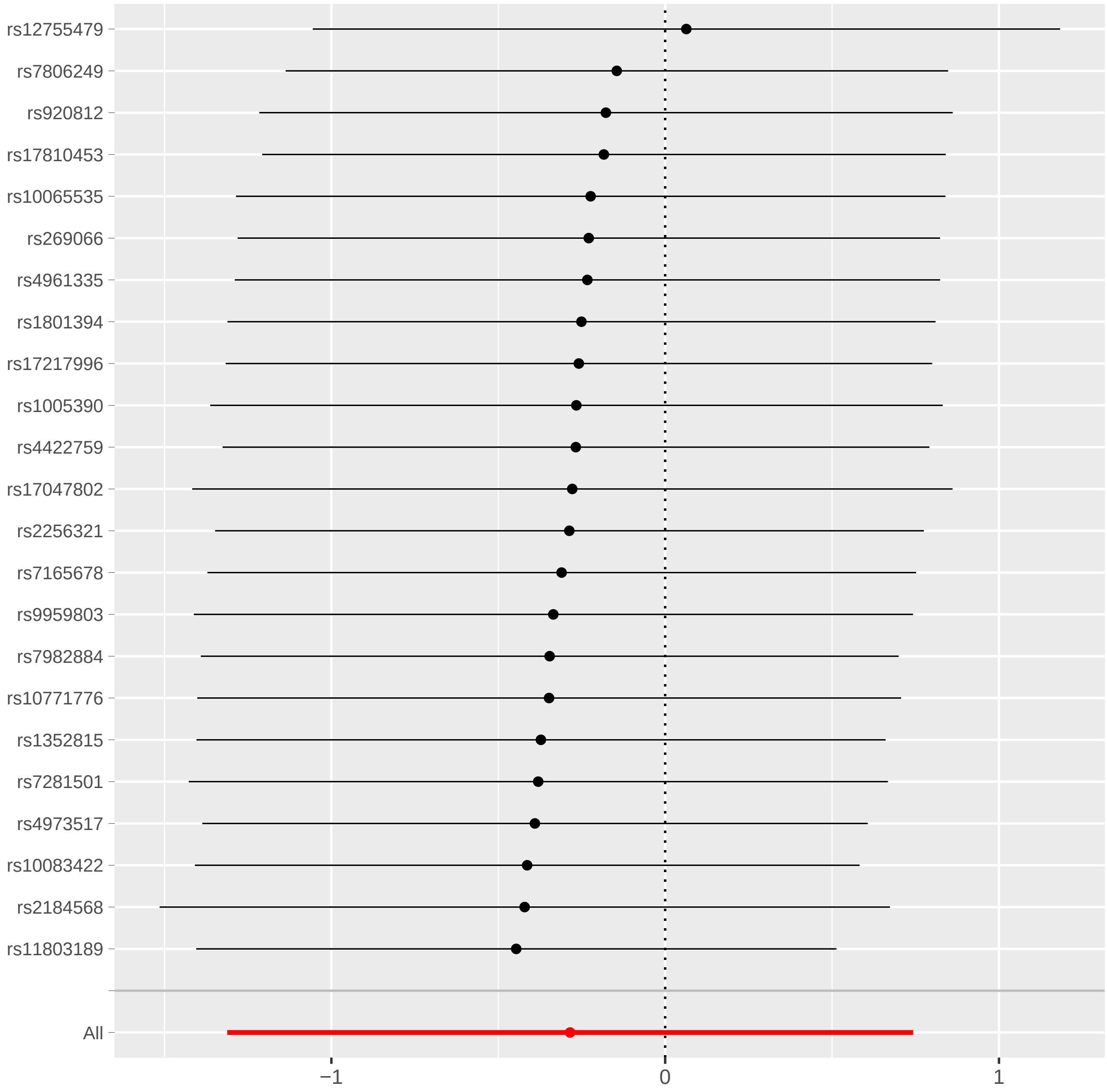

MR leave−one−out sensitivity analysis for
'M32709.metal.pos.txt.gz' on 'JUVEN_ARTHR.gz'

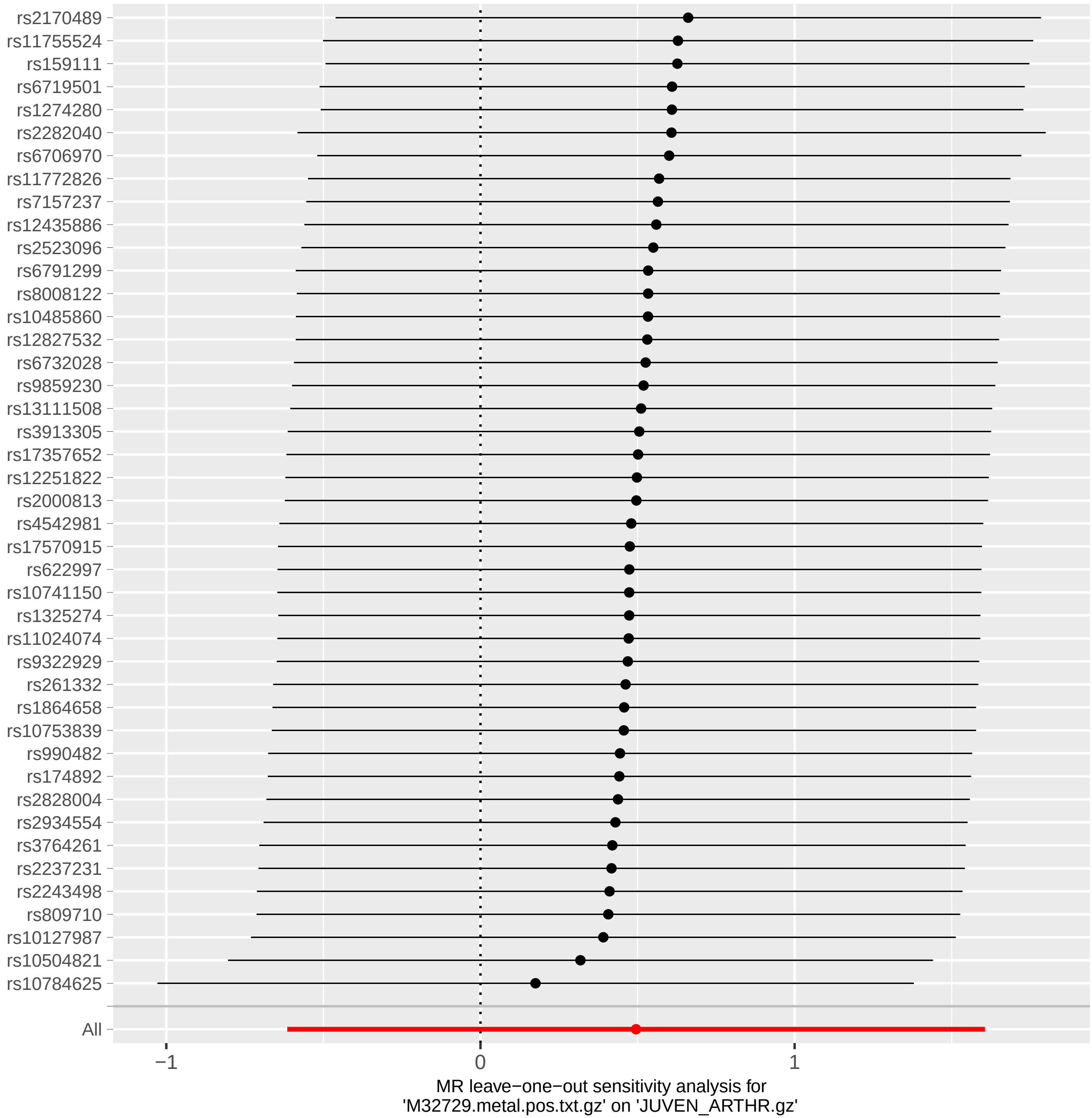

rs2170489
rs11755524
rs159111
rs6719501
rs1274280
rs2282040
rs6706970
rs11772826
rs7157237
rs12435886
rs2523096
rs6791299
rs8008122
rs10485860
rs12827532
rs6732028
rs9859230
rs13111508
rs3913305
rs17357652
rs12251822
rs2000813
rs4542981
rs17570915
rs622997
rs10741150
rs1325274
rs11024074
rs9322929
rs261332
rs1864658
rs10753839
rs990482
rs174892
rs2828004
rs2934554
rs3764261
rs2237231
rs2243498
rs809710
rs10127987
rs10504821
rs10784625
All
−1
0
1
MR leave−one−out sensitivity analysis for
'M32729.metal.pos.txt.gz' on 'JUVEN_ARTHR.gz'

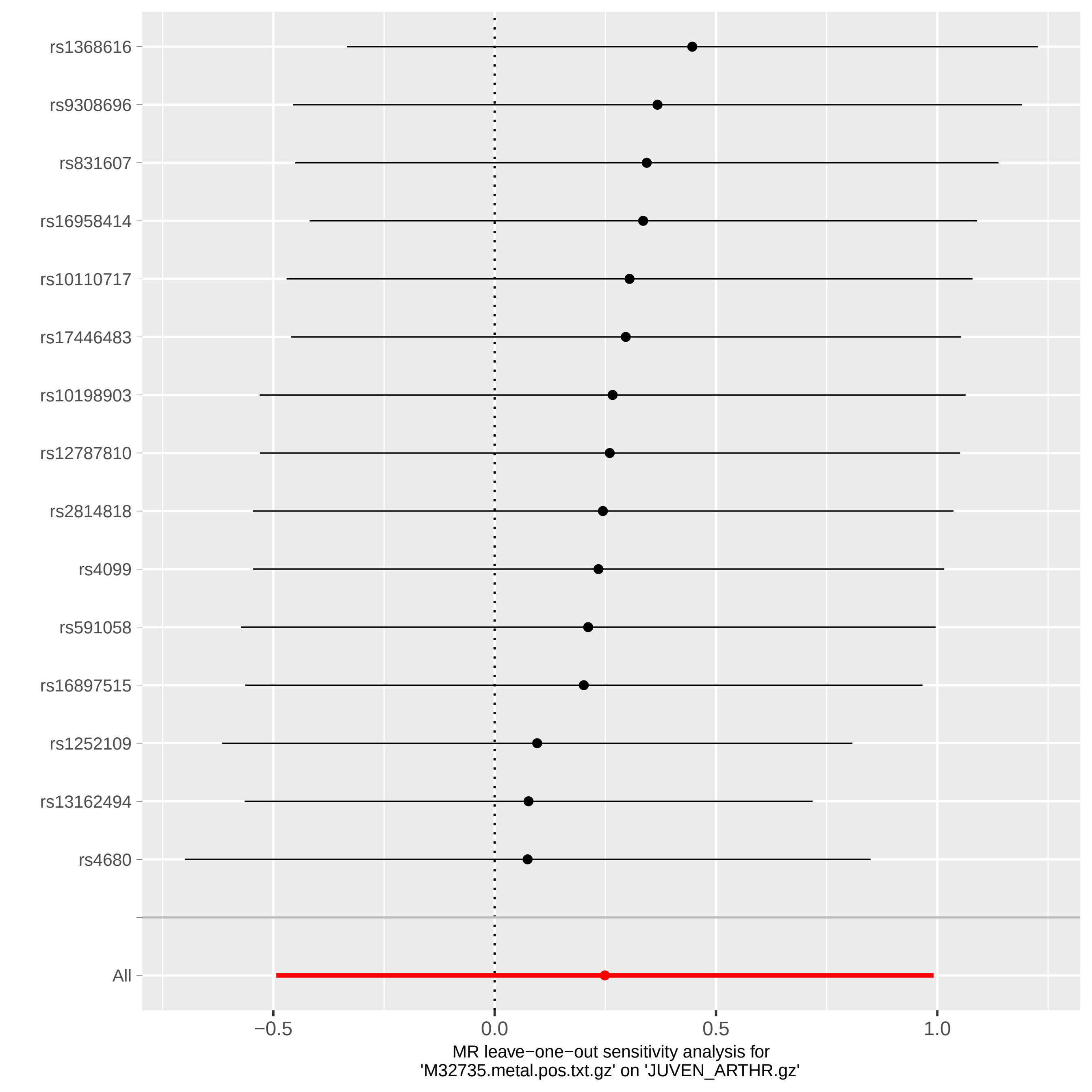
rs1368616
rs9308696
rs831607
rs16958414
rs10110717
rs17446483
rs10198903
rs12787810
rs2814818
rs4099
rs591058
rs16897515
rs1252109
rs13162494
rs4680
All
−0.5
0.0
0.5
1.0
MR leave−one−out sensitivity analysis for
'M32735.metal.pos.txt.gz' on 'JUVEN_ARTHR.gz'

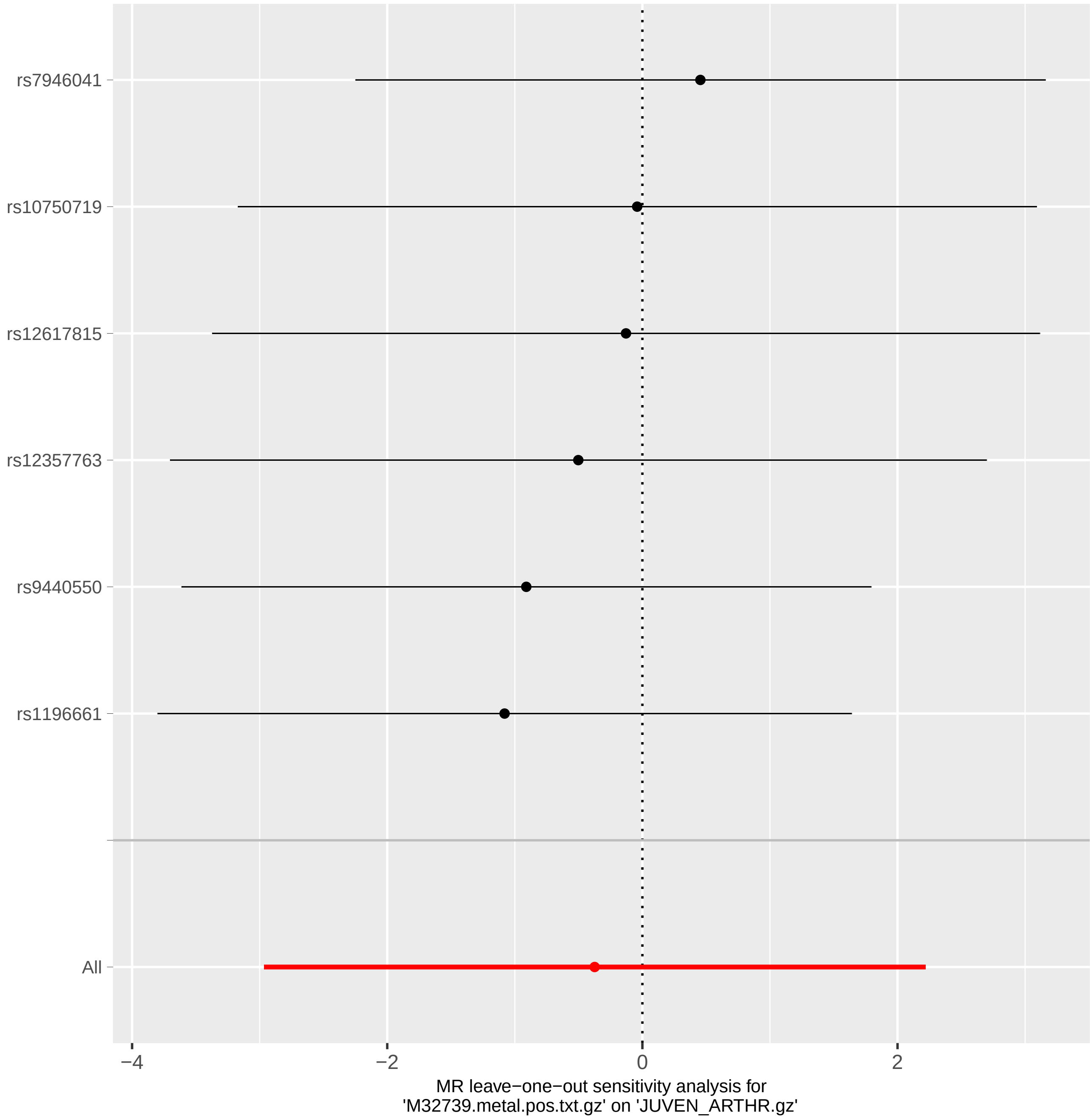
rs7946041
rs10750719
rs12617815
rs12357763
rs9440550
rs1196661
All
−4
−2
0
2
MR leave−one−out sensitivity analysis for
'M32739.metal.pos.txt.gz' on 'JUVEN_ARTHR.gz'

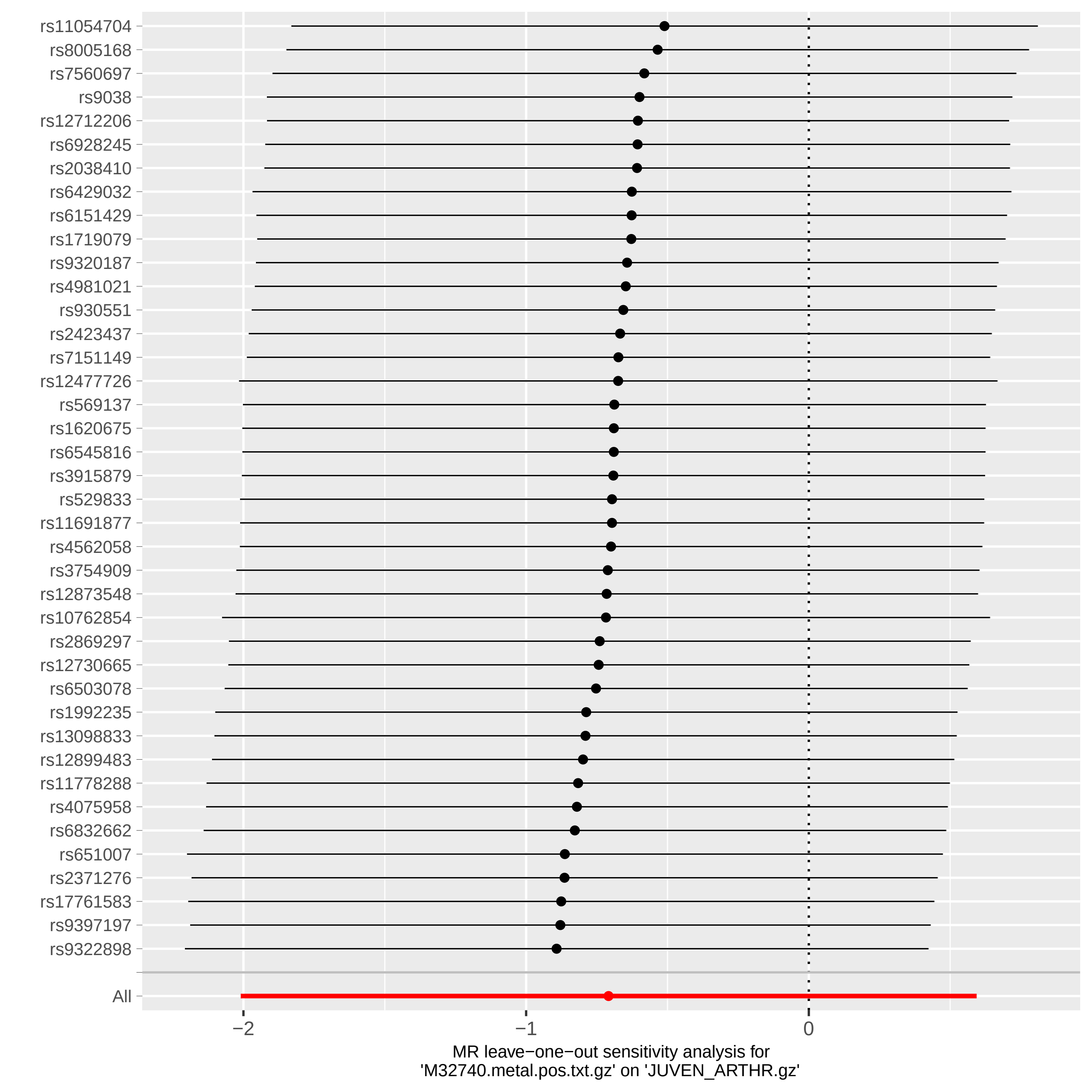

rs11054704
rs8005168
rs7560697
rs9038
rs12712206
rs6928245
rs2038410
rs6429032
rs6151429
rs1719079
rs9320187
rs4981021
rs930551
rs2423437
rs7151149
rs12477726
rs569137
rs1620675
rs6545816
rs3915879
rs529833
rs11691877
rs4562058
rs3754909
rs12873548
rs10762854
rs2869297
rs12730665
rs6503078
rs1992235
rs13098833
rs12899483
rs11778288
rs4075958
rs6832662
rs651007
rs2371276
rs17761583
rs9397197
rs9322898
All
−2
−1
0
MR leave−one−out sensitivity analysis for
'M32740.metal.pos.txt.gz' on 'JUVEN_ARTHR.gz'

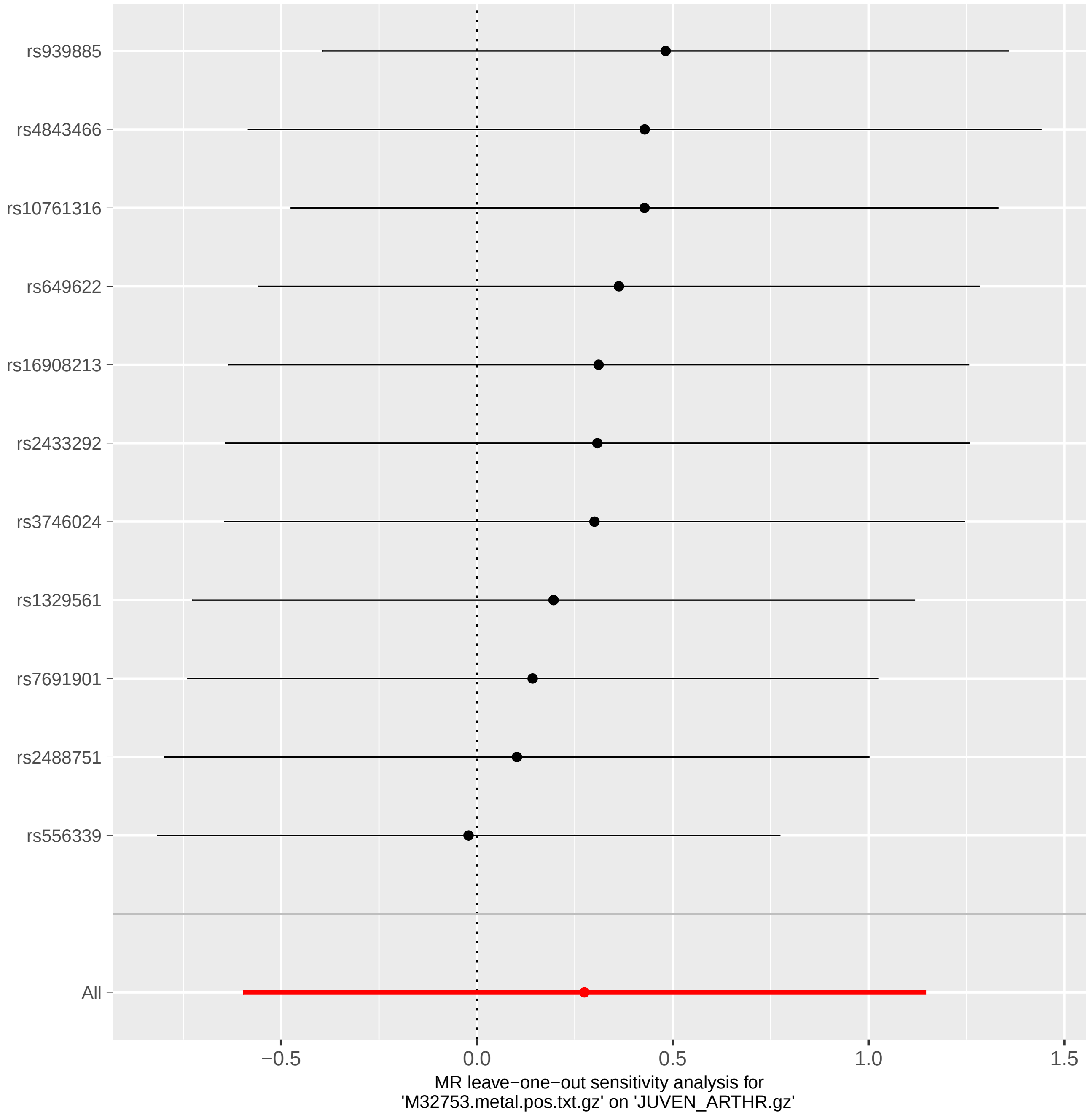
rs939885
rs4843466
rs10761316
rs649622
rs16908213
rs2433292
rs3746024
rs1329561
rs7691901
rs2488751
rs556339
All
−0.5
0.0
0.5
1.0
1.5
MR leave−one−out sensitivity analysis for
'M32753.metal.pos.txt.gz' on 'JUVEN_ARTHR.gz'

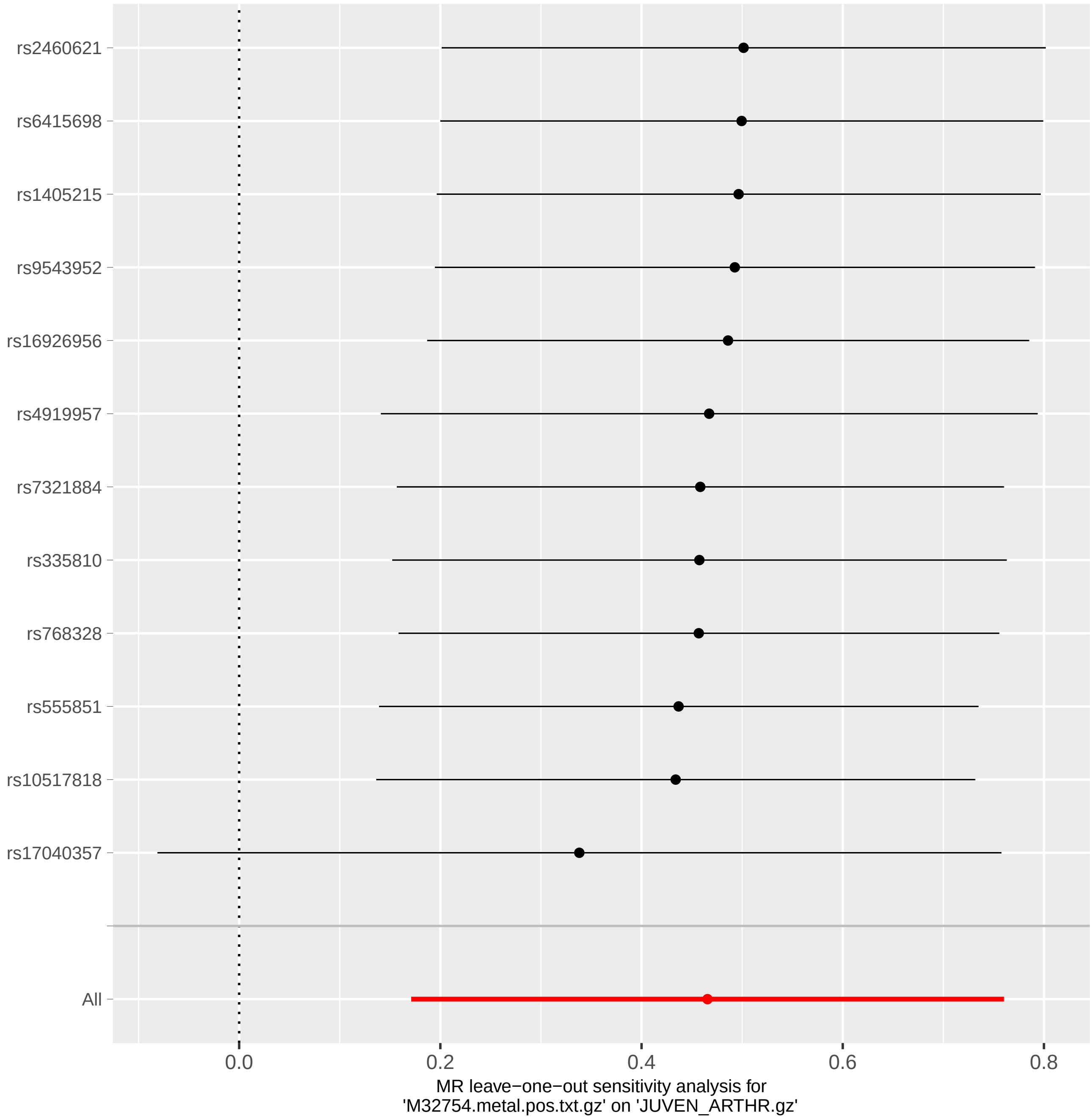
rs2460621
rs6415698
rs1405215
rs9543952
rs16926956
rs4919957
rs7321884
rs335810
rs768328
rs555851
rs10517818
rs17040357
All
0.0
0.2
0.4
0.6
0.8
MR leave−one−out sensitivity analysis for
'M32754.metal.pos.txt.gz' on 'JUVEN_ARTHR.gz'

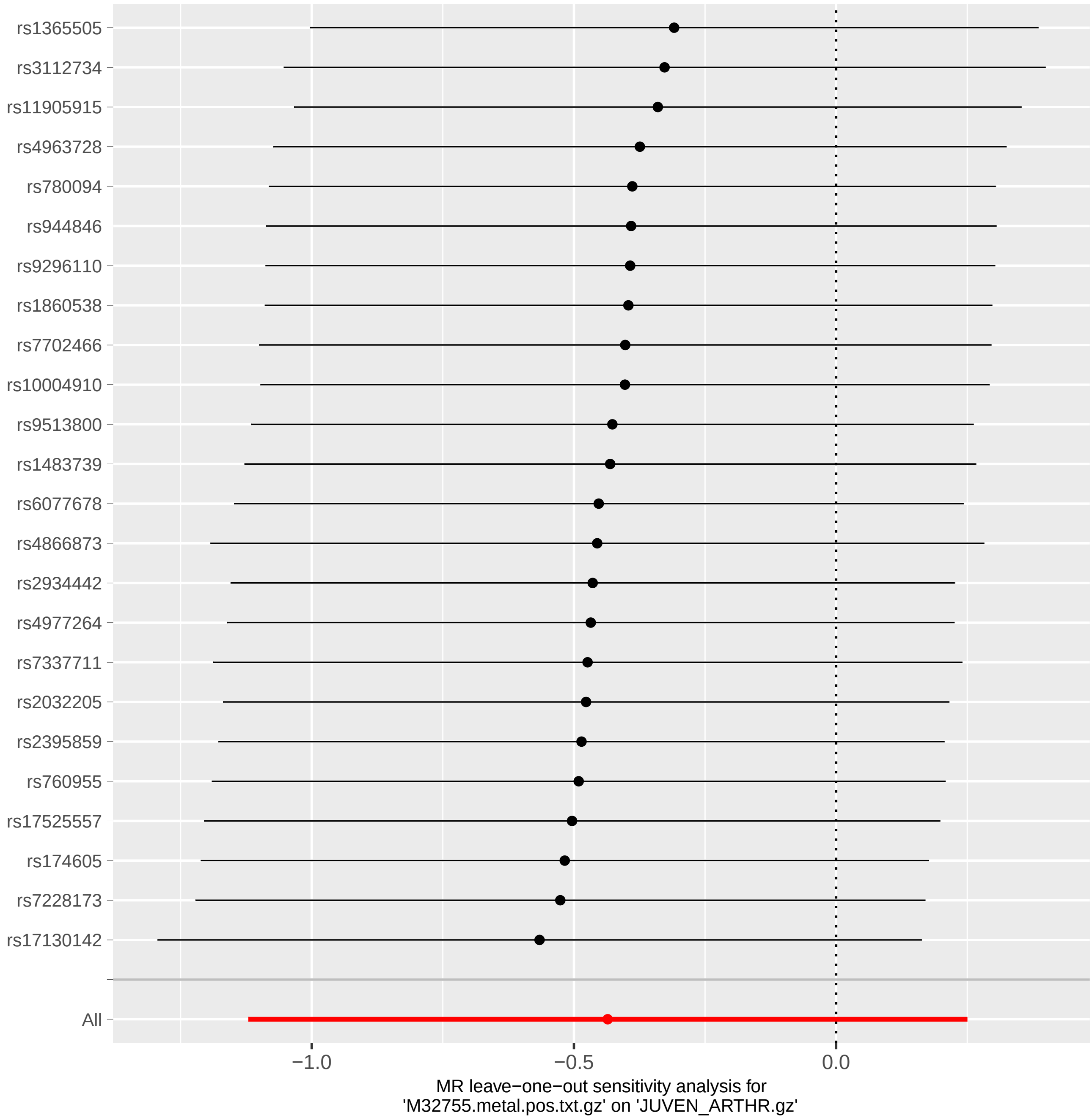

rs1365505
rs3112734
rs11905915
rs4963728
rs780094
rs944846
rs9296110
rs1860538
rs7702466
rs10004910
rs9513800
rs1483739
rs6077678
rs4866873
rs2934442
rs4977264
rs7337711
rs2032205
rs2395859
rs760955
rs17525557
rs174605
rs7228173
rs17130142
All
−1.0
−0.5
0.0
MR leave−one−out sensitivity analysis for
'M32755.metal.pos.txt.gz' on 'JUVEN_ARTHR.gz'

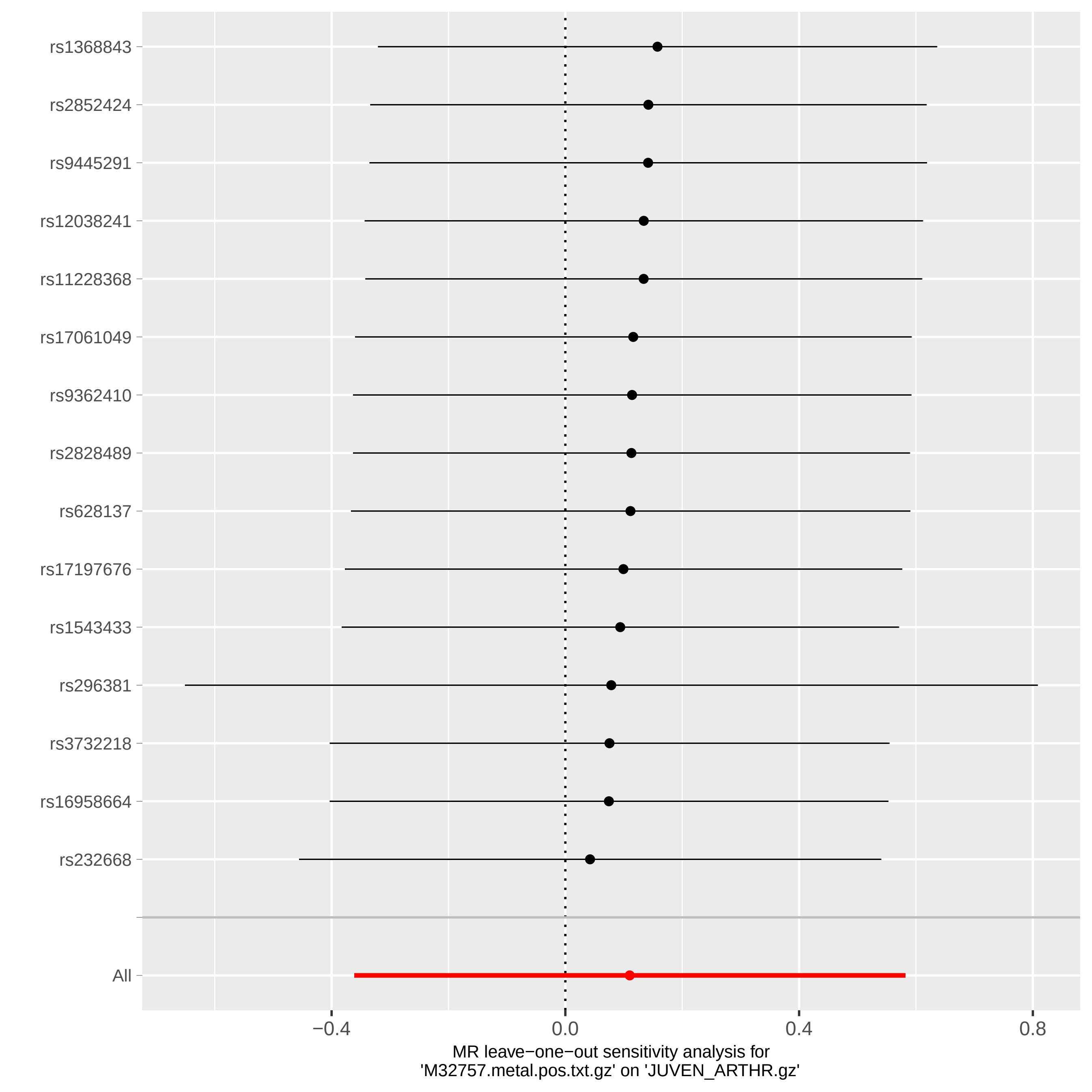
rs1368843
rs2852424
rs9445291
rs12038241
rs11228368
rs17061049
rs9362410
rs2828489
rs628137
rs17197676
rs1543433
rs296381
rs3732218
rs16958664
rs232668
All
−0.4
0.0
0.4
0.8
MR leave−one−out sensitivity analysis for
'M32757.metal.pos.txt.gz' on 'JUVEN_ARTHR.gz'

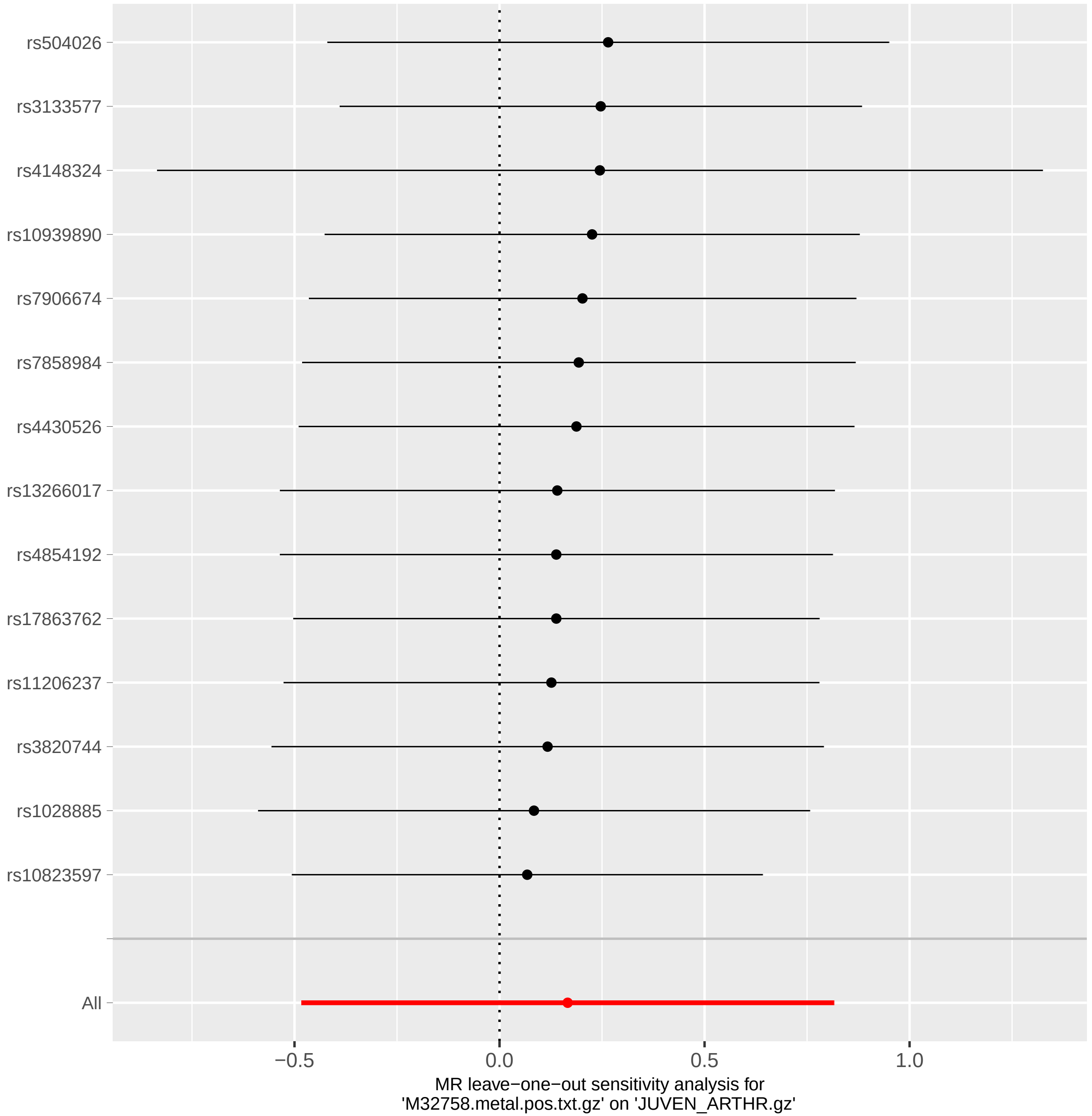
rs504026
rs3133577
rs4148324
rs10939890
rs7906674
rs7858984
rs4430526
rs13266017
rs4854192
rs17863762
rs11206237
rs3820744
rs1028885
rs10823597
All
−0.5
0.0
0.5
1.0
MR leave−one−out sensitivity analysis for
'M32758.metal.pos.txt.gz' on 'JUVEN_ARTHR.gz'

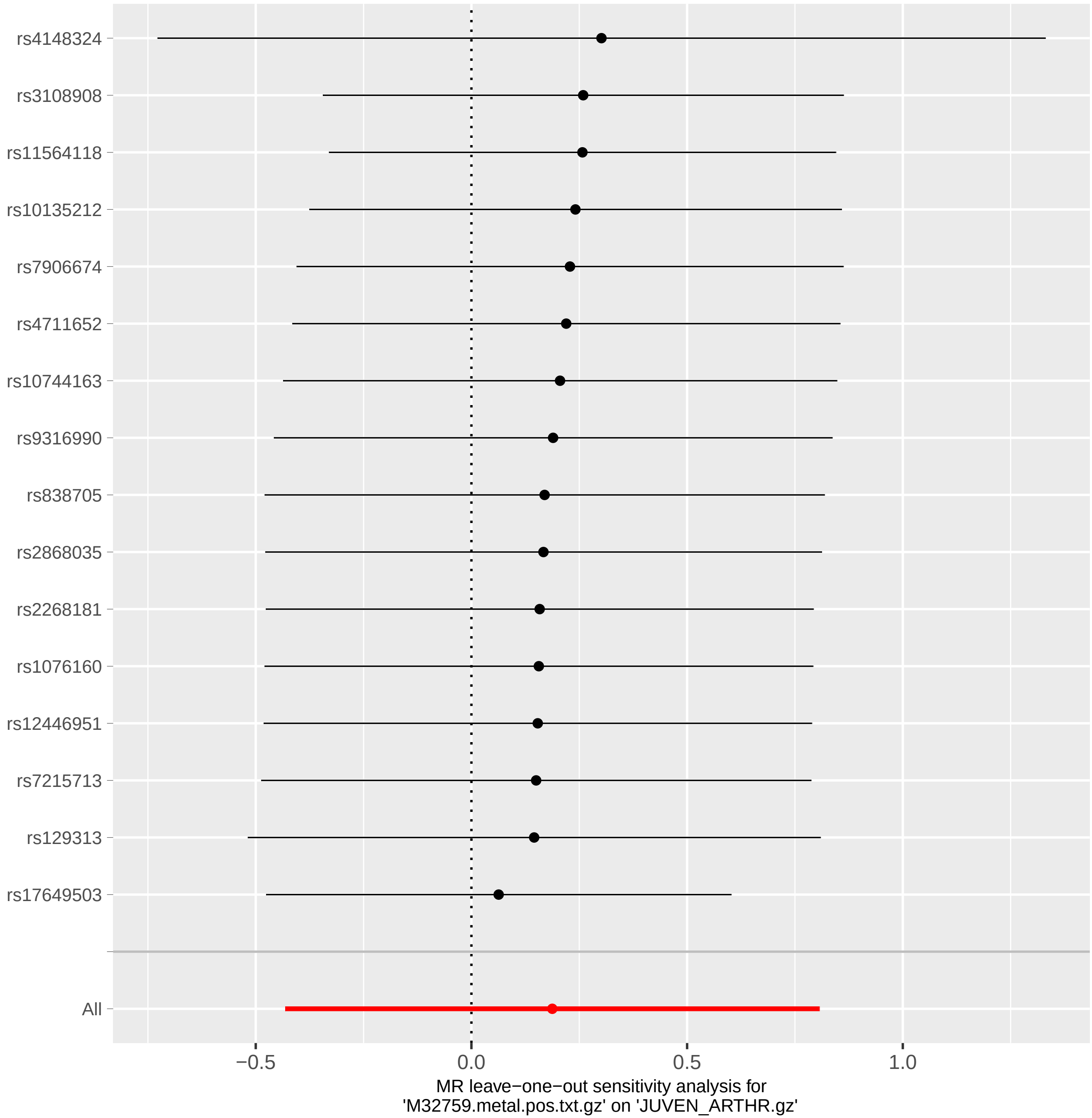
rs4148324
rs3108908
rs11564118
rs10135212
rs7906674
rs4711652
rs10744163
rs9316990
rs838705
rs2868035
rs2268181
rs1076160
rs12446951
rs7215713
rs129313
rs17649503
All
−0.5
0.0
0.5
1.0
MR leave−one−out sensitivity analysis for
'M32759.metal.pos.txt.gz' on 'JUVEN_ARTHR.gz'

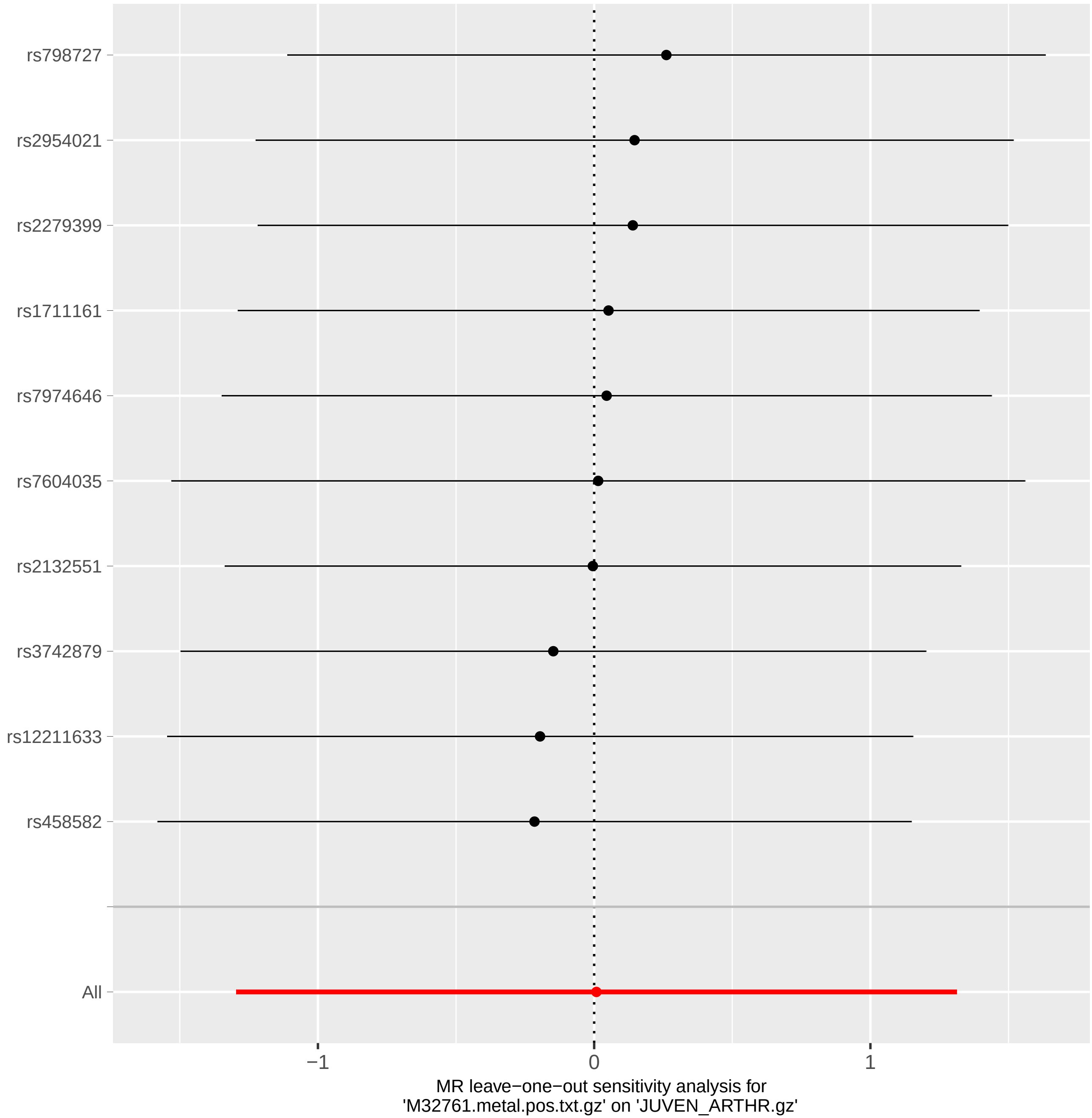
rs798727
rs2954021
rs2279399
rs1711161
rs7974646
rs7604035
rs2132551
rs3742879
rs12211633
rs458582
All
−1
0
1
MR leave−one−out sensitivity analysis for
'M32761.metal.pos.txt.gz' on 'JUVEN_ARTHR.gz'

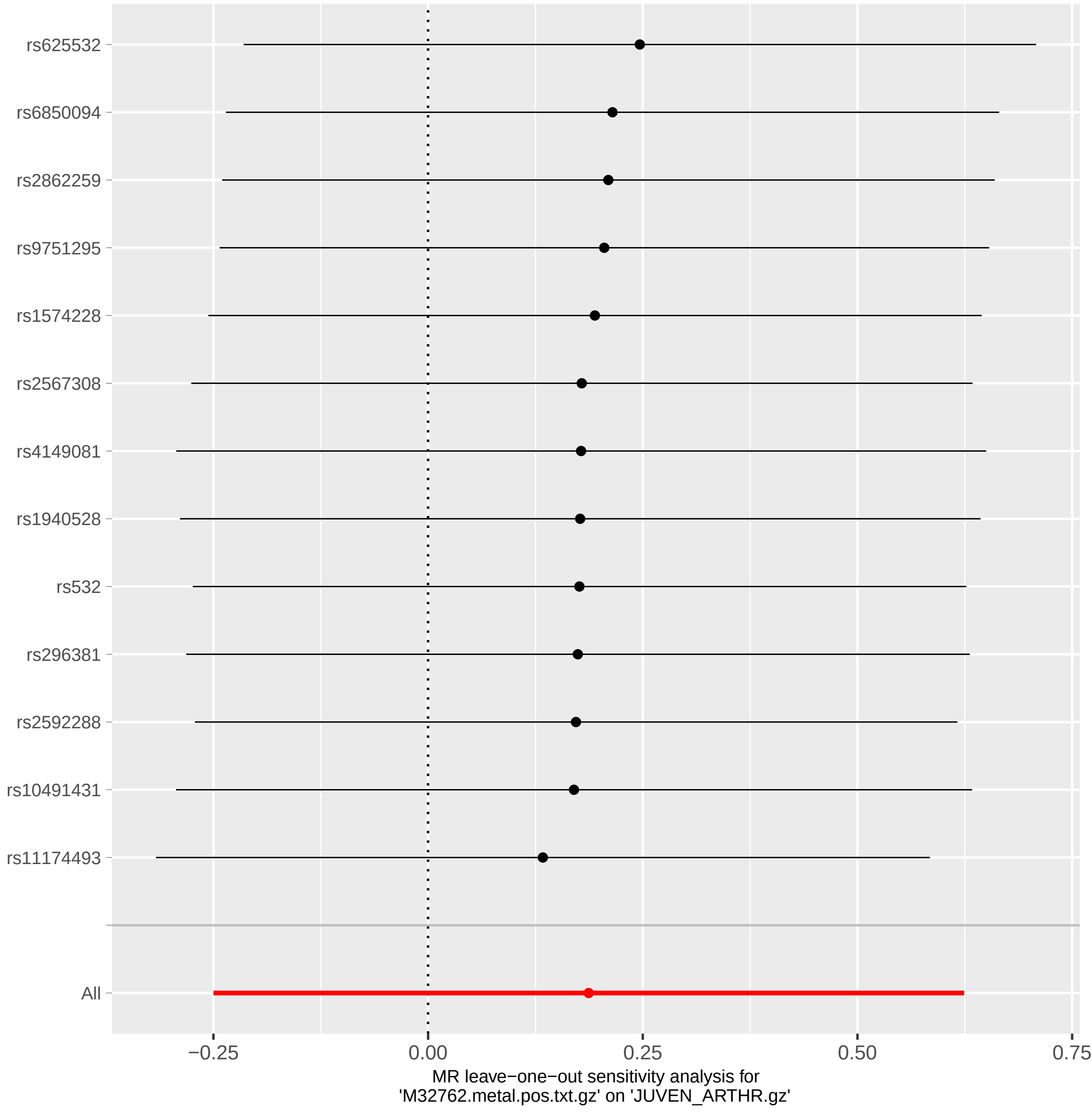
rs625532
rs6850094
rs2862259
rs9751295
rs1574228
rs2567308
rs4149081
rs1940528
rs532
rs296381
rs2592288
rs10491431
rs11174493
All
−0.25
0.00
0.25
0.50
0.75
MR leave−one−out sensitivity analysis for
'M32762.metal.pos.txt.gz' on 'JUVEN_ARTHR.gz'

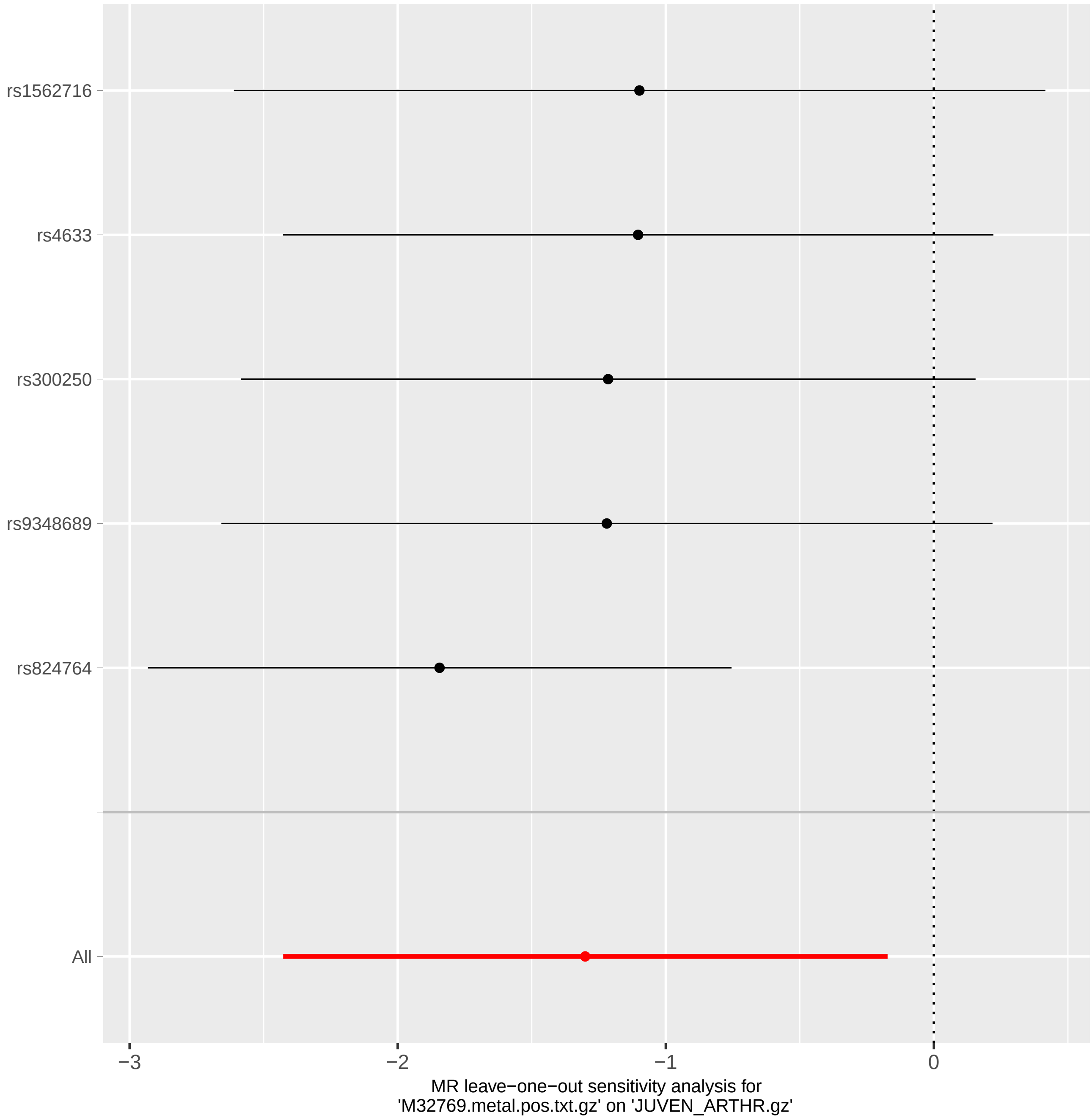
rs1562716
rs4633
rs300250
rs9348689
rs824764
All
−3
−2
−1
0
MR leave−one−out sensitivity analysis for
'M32769.metal.pos.txt.gz' on 'JUVEN_ARTHR.gz'

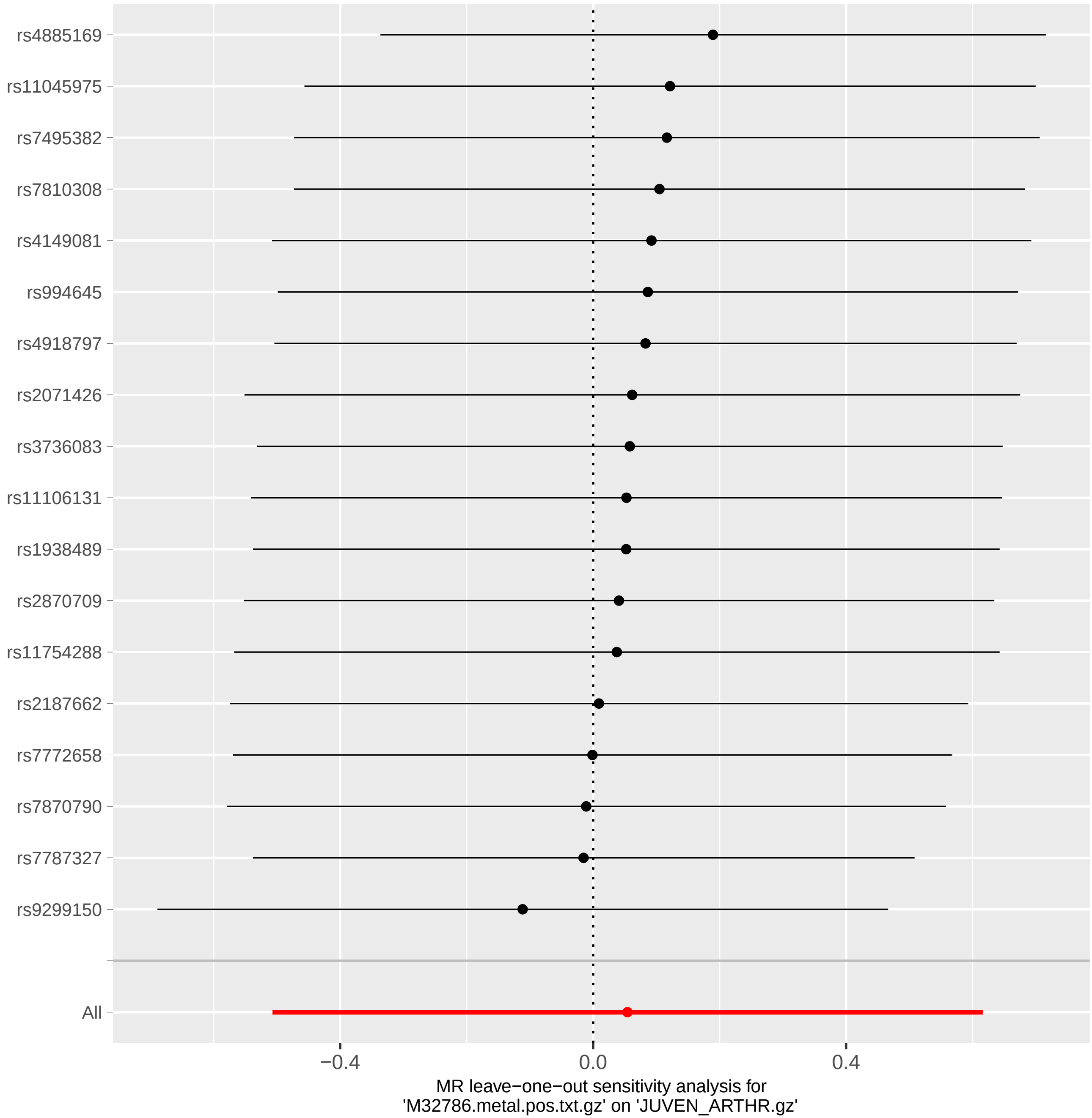
rs4885169
rs11045975
rs7495382
rs7810308
rs4149081
rs994645
rs4918797
rs2071426
rs3736083
rs11106131
rs1938489
rs2870709
rs11754288
rs2187662
rs7772658
rs7870790
rs7787327
rs9299150
All
−0.4
0.0
0.4
MR leave−one−out sensitivity analysis for
'M32786.metal.pos.txt.gz' on 'JUVEN_ARTHR.gz'

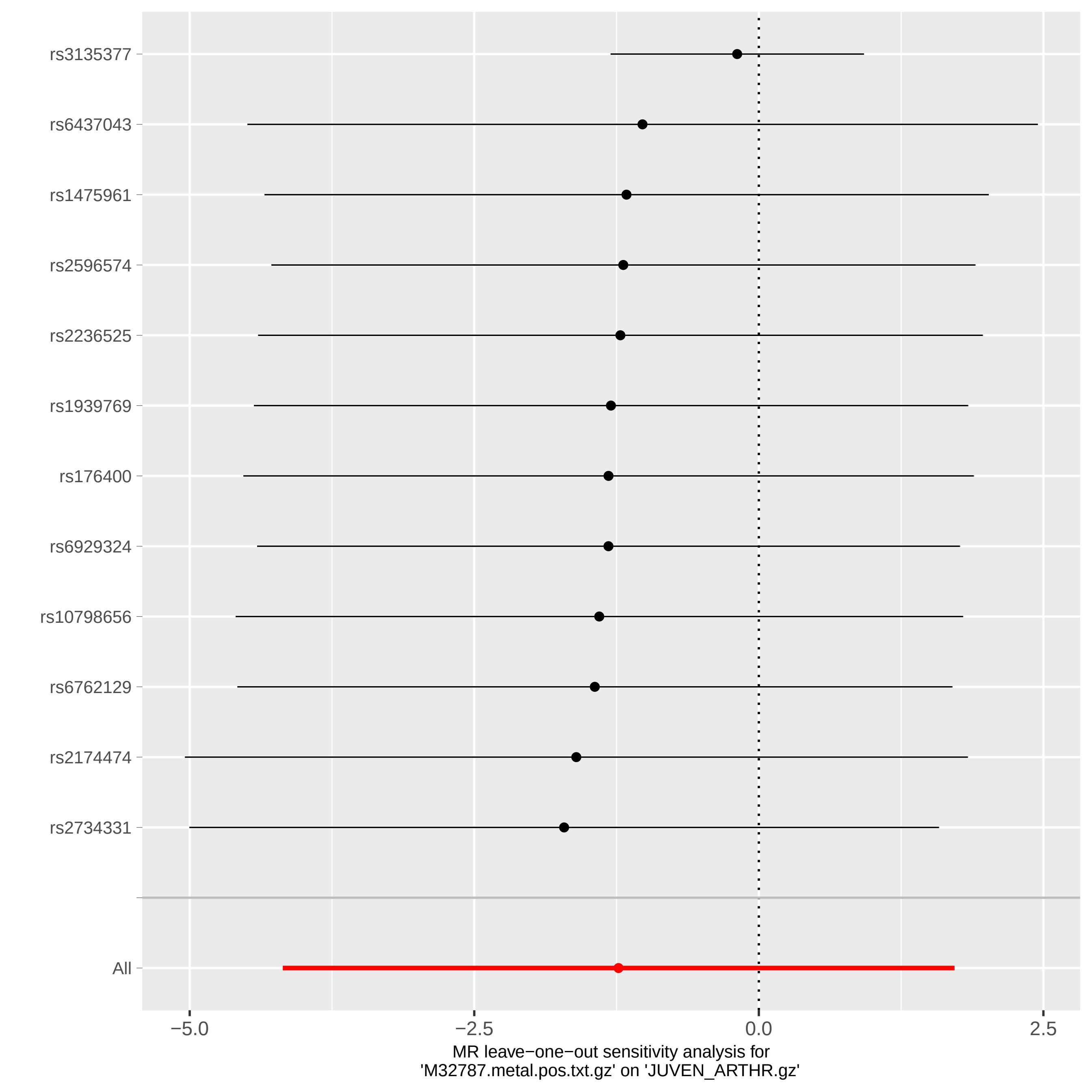

rs3135377
rs6437043
rs1475961
rs2596574
rs2236525
rs1939769
rs176400
rs6929324
rs10798656
rs6762129
rs2174474
rs2734331
All
−5.0
−2.5
0.0
2.5
MR leave−one−out sensitivity analysis for
'M32787.metal.pos.txt.gz' on 'JUVEN_ARTHR.gz'

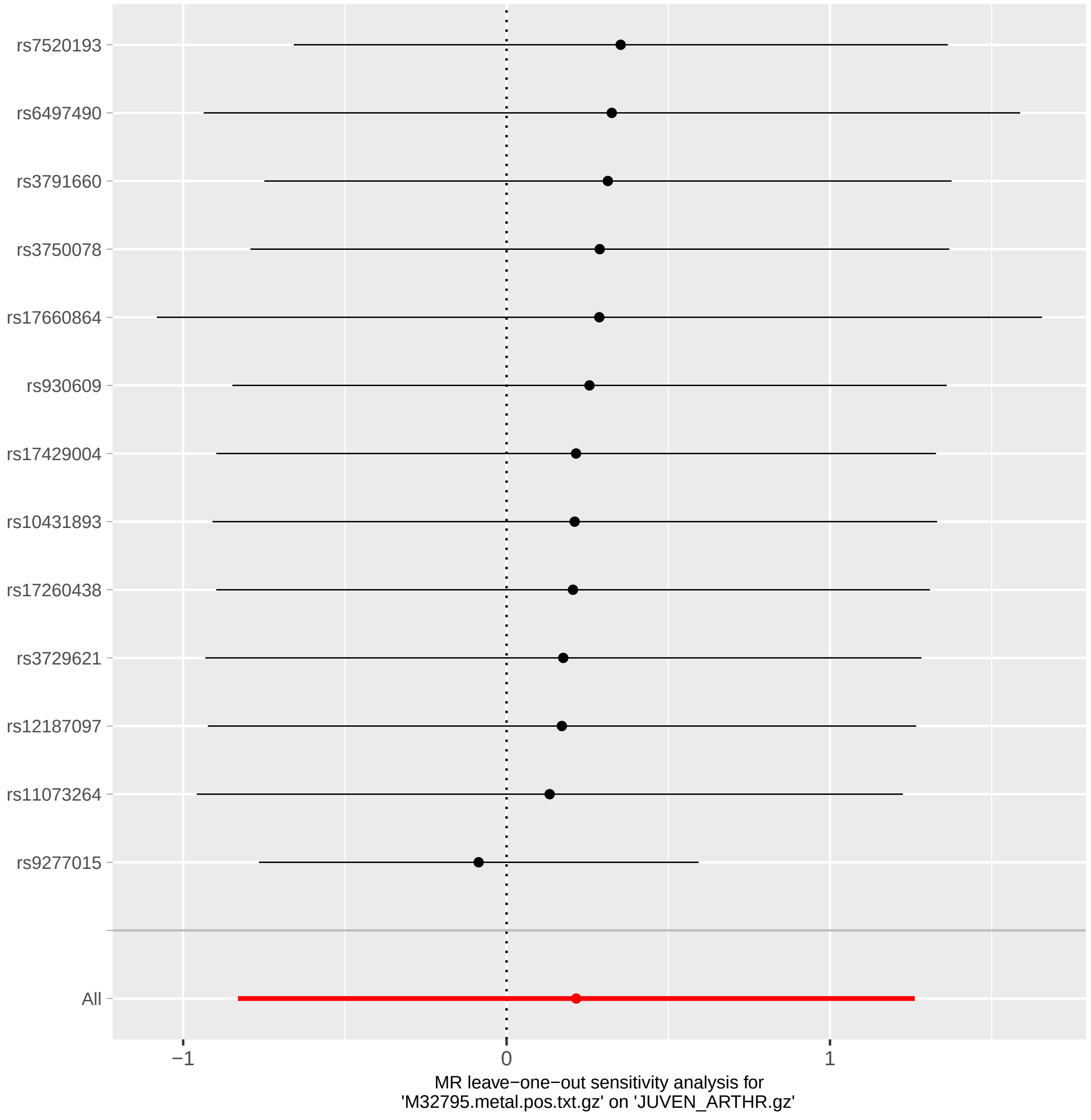
rs7520193
rs6497490
rs3791660
rs3750078
rs17660864
rs930609
rs17429004
rs10431893
rs17260438
rs3729621
rs12187097
rs11073264
rs9277015
All
−1
0
1
MR leave−one−out sensitivity analysis for
'M32795.metal.pos.txt.gz' on 'JUVEN_ARTHR.gz'

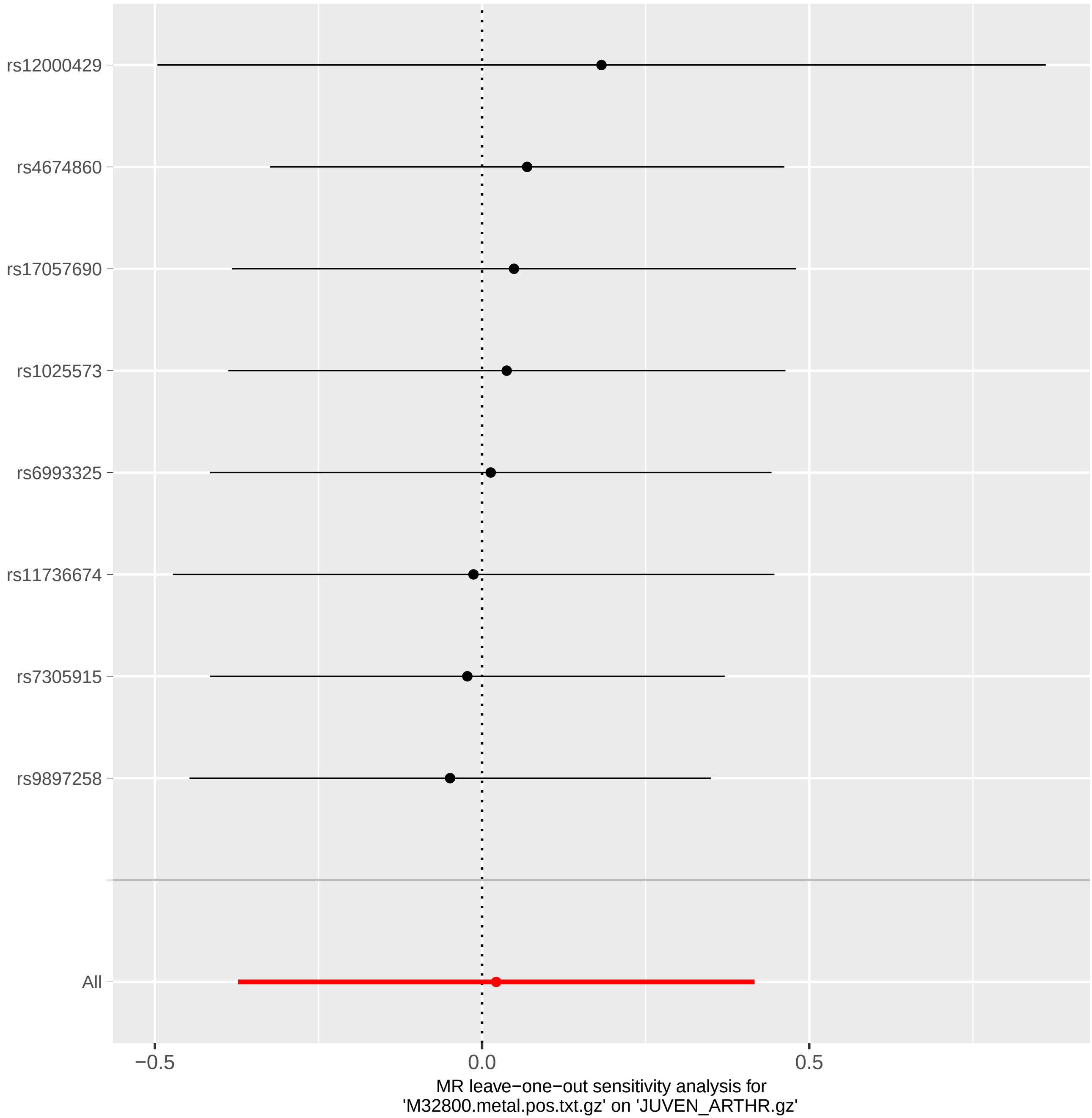
rs12000429
rs4674860
rs17057690
rs1025573
rs6993325
rs11736674
rs7305915
rs9897258
All
−0.5
0.0
0.5
MR leave−one−out sensitivity analysis for
'M32800.metal.pos.txt.gz' on 'JUVEN_ARTHR.gz'

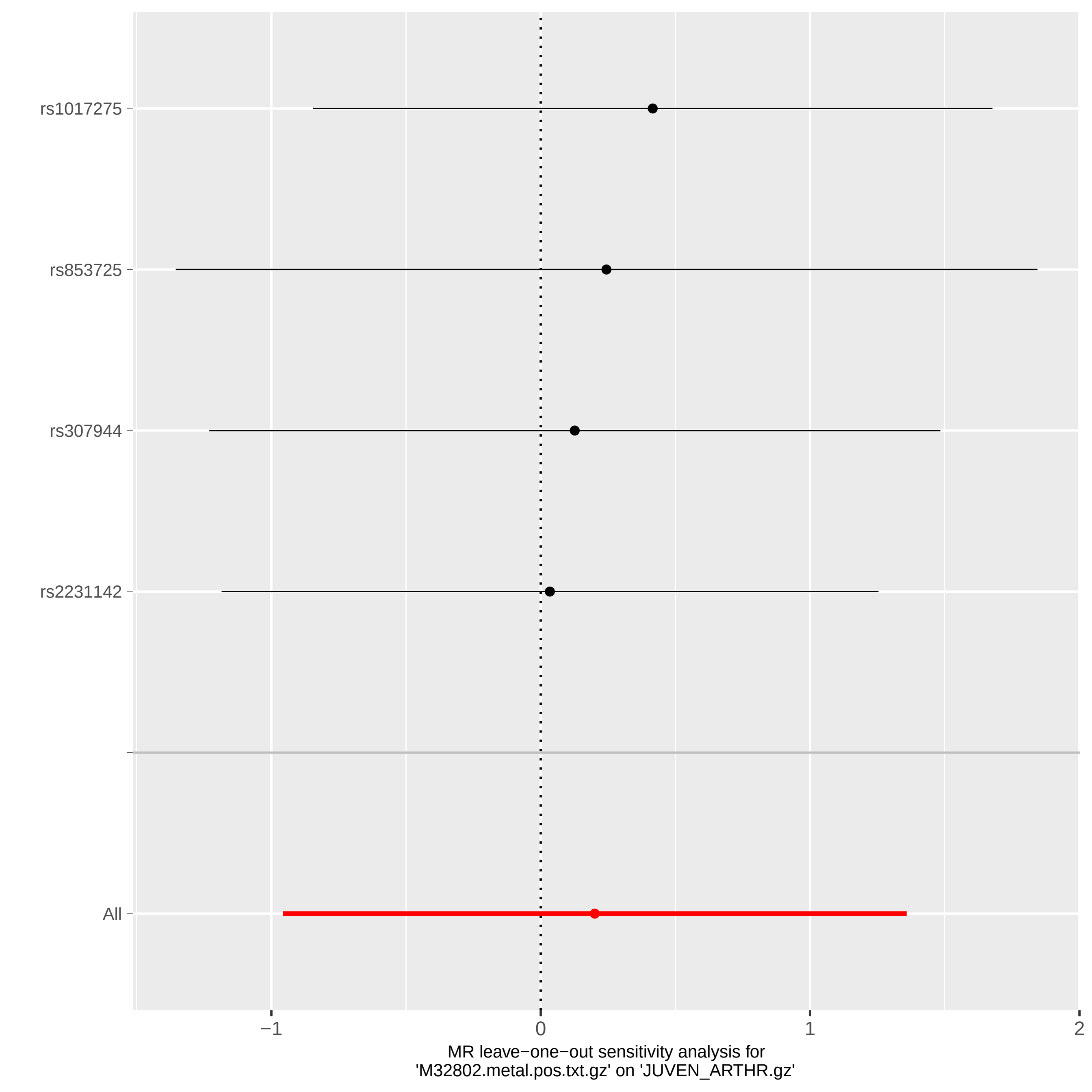

rs1017275
rs853725
rs307944
rs2231142
All
−1
0
1
2
MR leave−one−out sensitivity analysis for
'M32802.metal.pos.txt.gz' on 'JUVEN_ARTHR.gz'

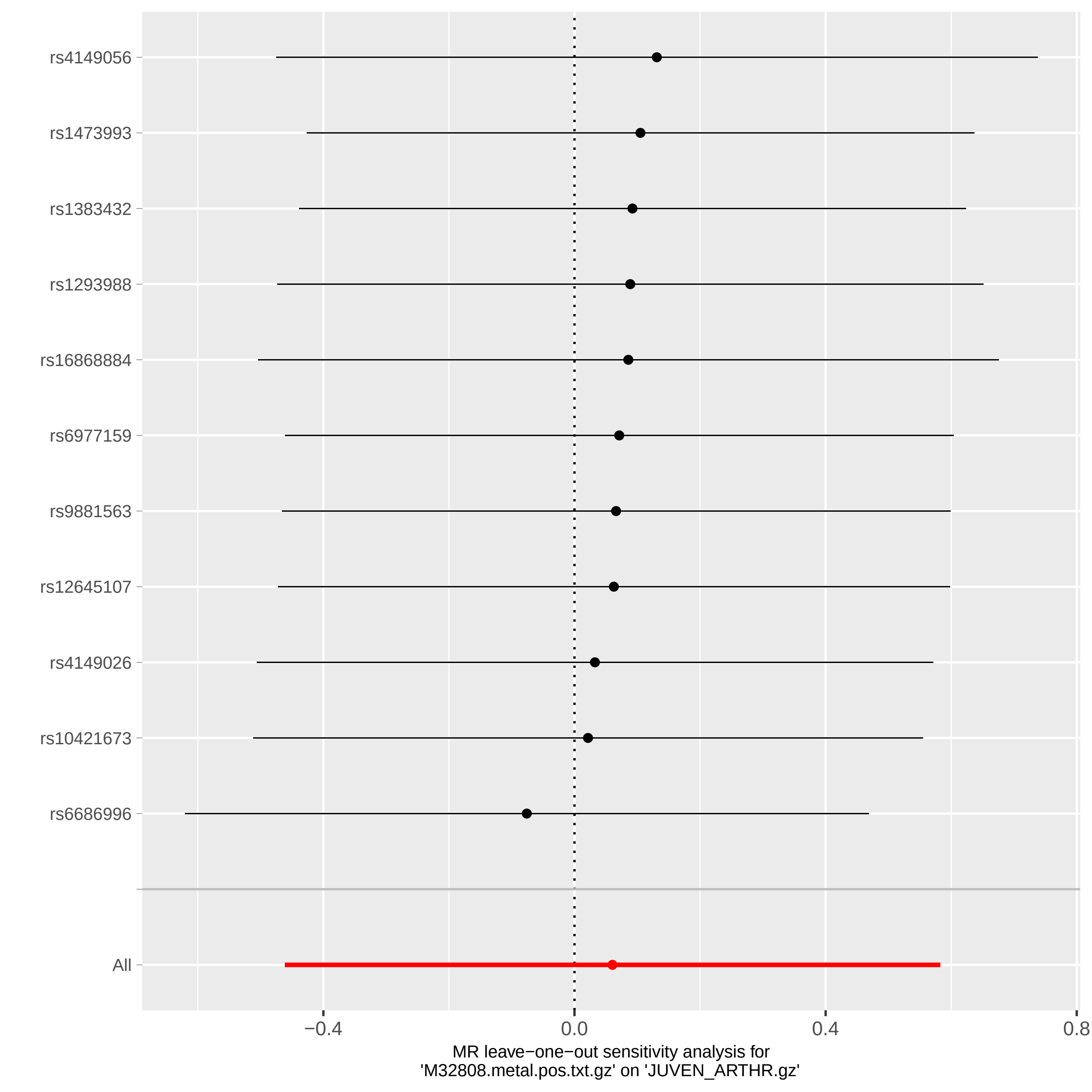

rs4149056
rs1473993
rs1383432
rs1293988
rs16868884
rs6977159
rs9881563
rs12645107
rs4149026
rs10421673
rs6686996
All
−0.4
0.0
0.4
0.8
MR leave−one−out sensitivity analysis for
'M32808.metal.pos.txt.gz' on 'JUVEN_ARTHR.gz'

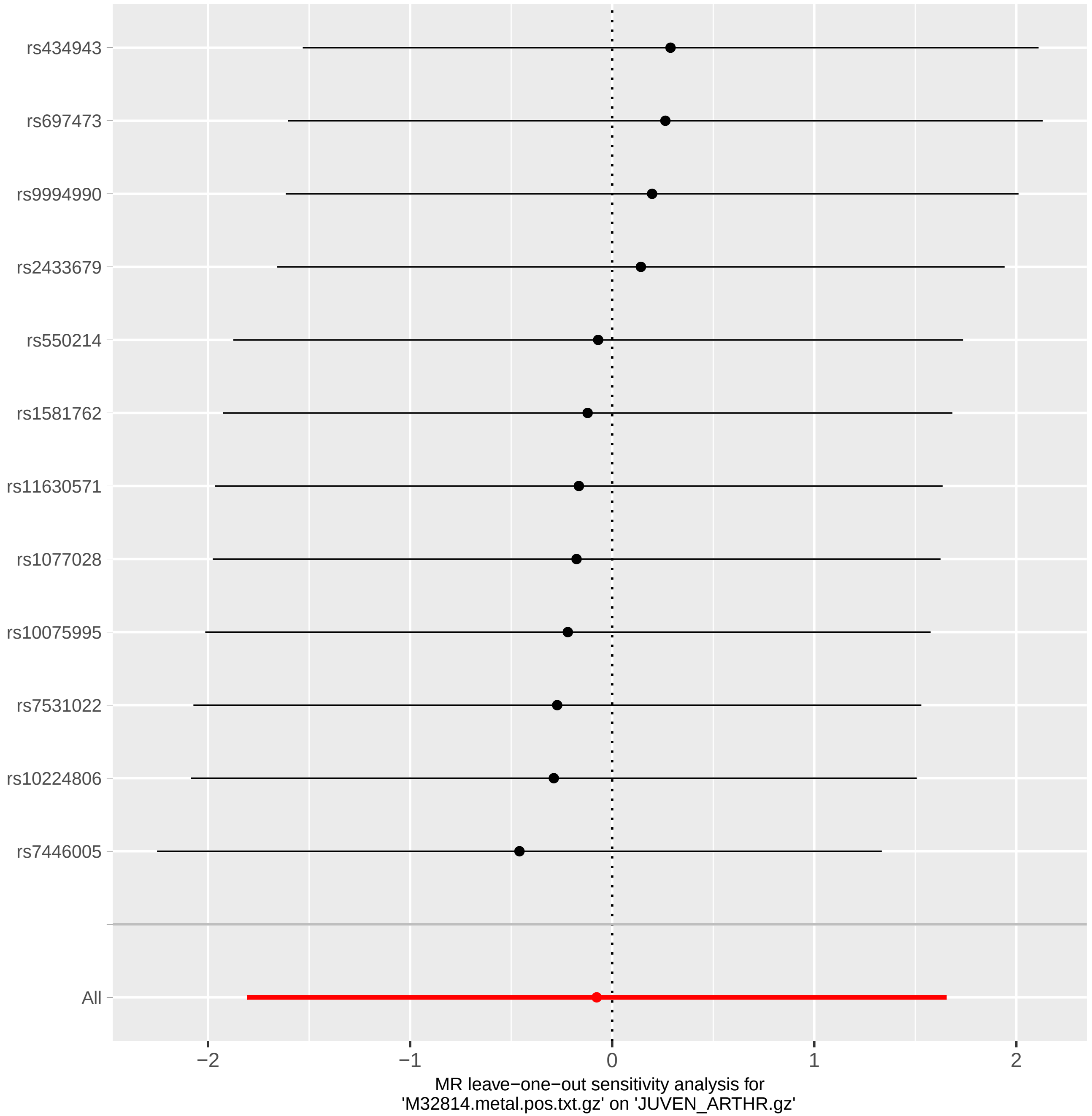
rs434943
rs697473
rs9994990
rs2433679
rs550214
rs1581762
rs11630571
rs1077028
rs10075995
rs7531022
rs10224806
rs7446005
All
−2
−1
0
1
2
MR leave−one−out sensitivity analysis for
'M32814.metal.pos.txt.gz' on 'JUVEN_ARTHR.gz'

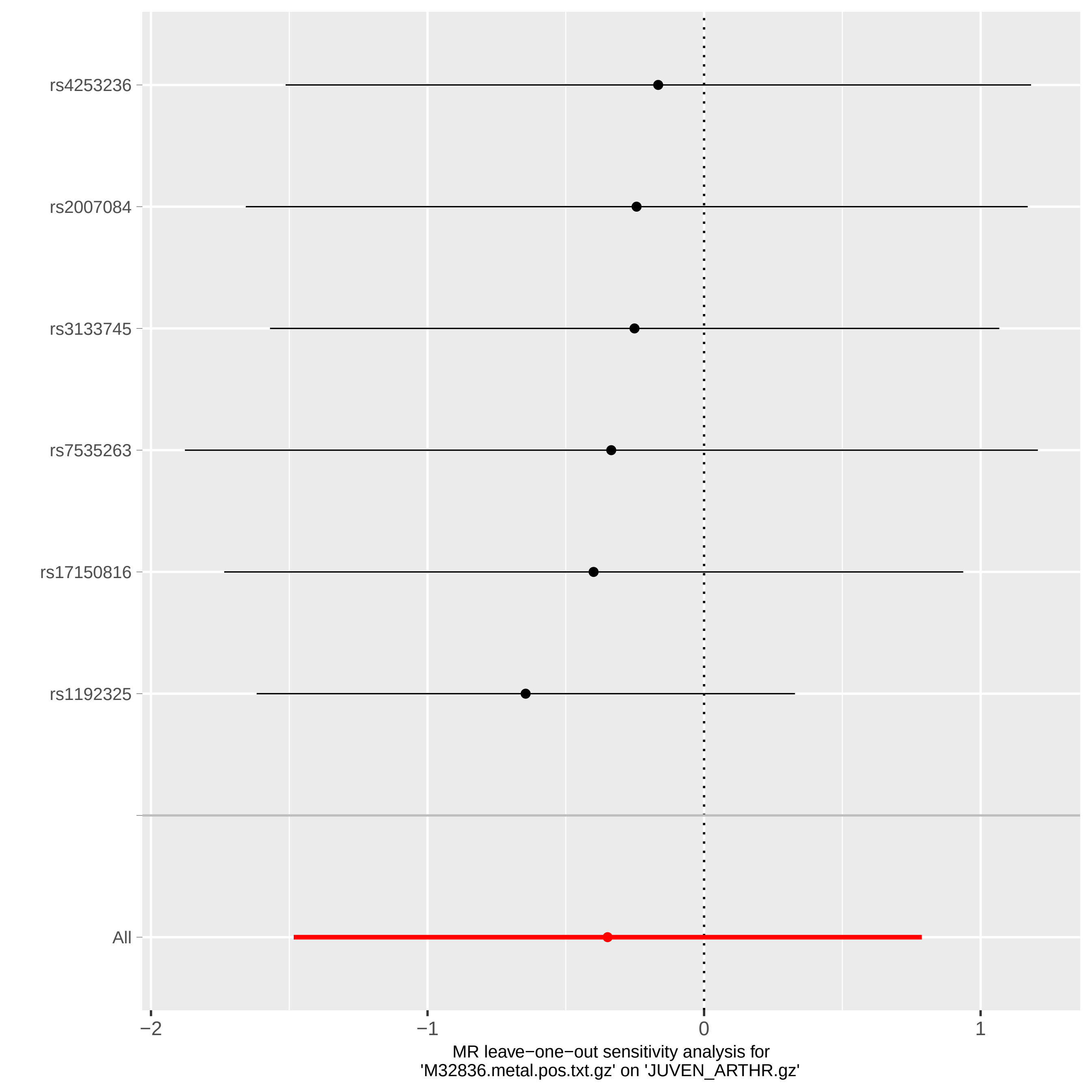

rs4253236
rs2007084
rs3133745
rs7535263
rs17150816
rs1192325
All
−2
−1
0
1
MR leave−one−out sensitivity analysis for
'M32836.metal.pos.txt.gz' on 'JUVEN_ARTHR.gz'

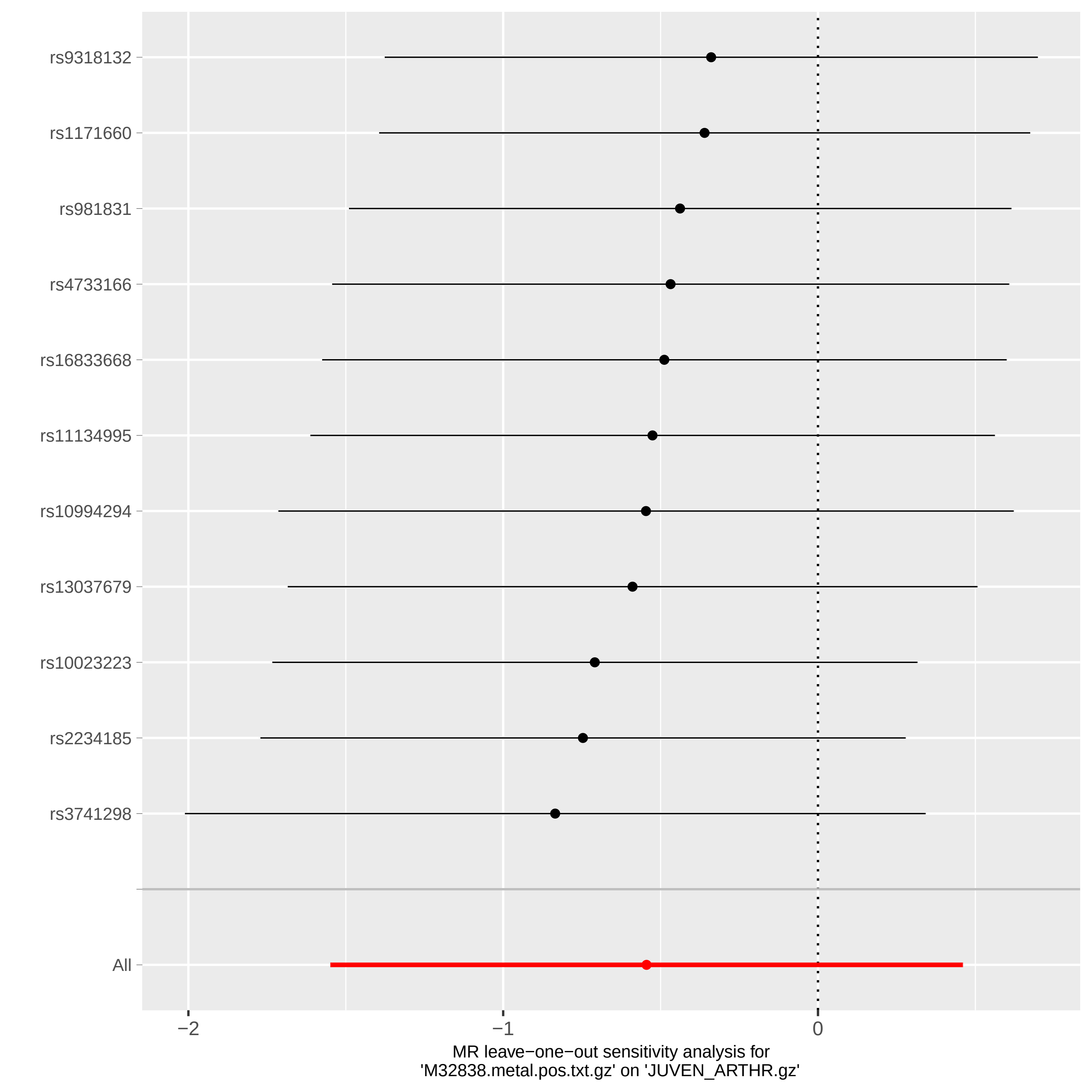
rs9318132
rs1171660
rs981831
rs4733166
rs16833668
rs11134995
rs10994294
rs13037679
rs10023223
rs2234185
rs3741298
All
−2
−1
0
MR leave−one−out sensitivity analysis for
'M32838.metal.pos.txt.gz' on 'JUVEN_ARTHR.gz'

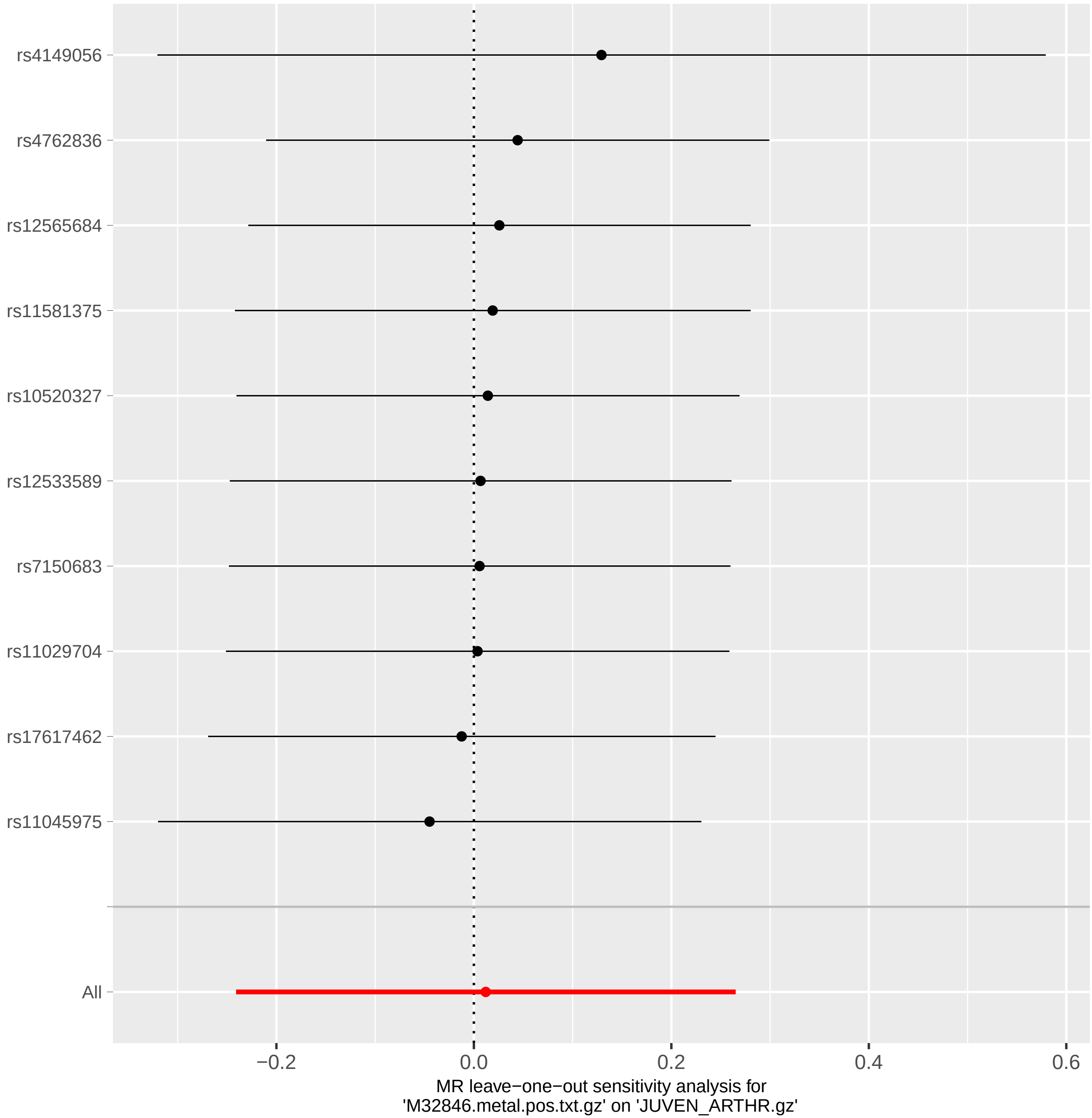
rs4149056
rs4762836
rs12565684
rs11581375
rs10520327
rs12533589
rs7150683
rs11029704
rs17617462
rs11045975
All
−0.2
0.0
0.2
0.4
0.6
MR leave−one−out sensitivity analysis for
'M32846.metal.pos.txt.gz' on 'JUVEN_ARTHR.gz'

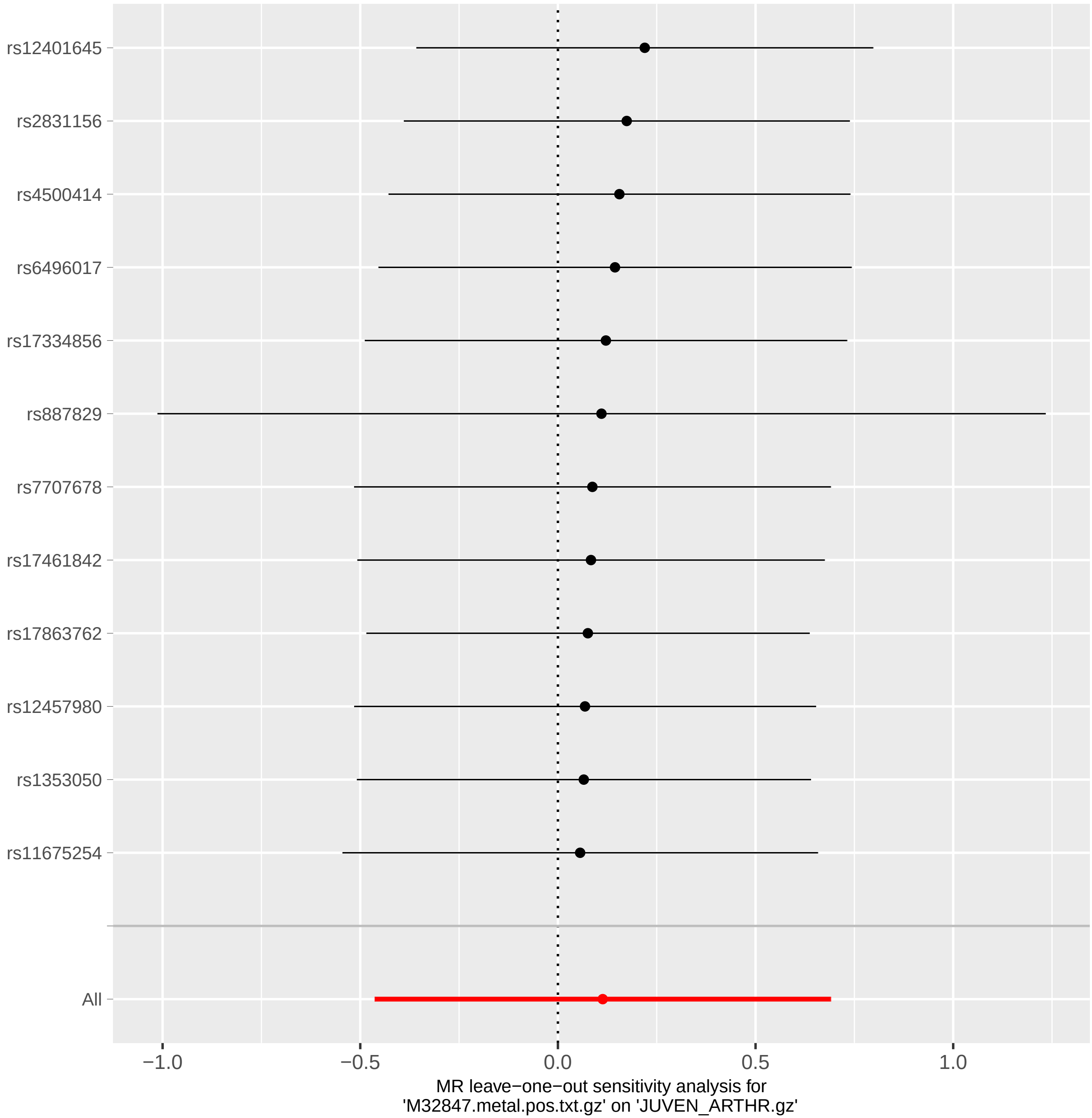
rs12401645
rs2831156
rs4500414
rs6496017
rs17334856
rs887829
rs7707678
rs17461842
rs17863762
rs12457980
rs1353050
rs11675254
All
−1.0
−0.5
0.0
0.5
1.0
MR leave−one−out sensitivity analysis for
'M32847.metal.pos.txt.gz' on 'JUVEN_ARTHR.gz'

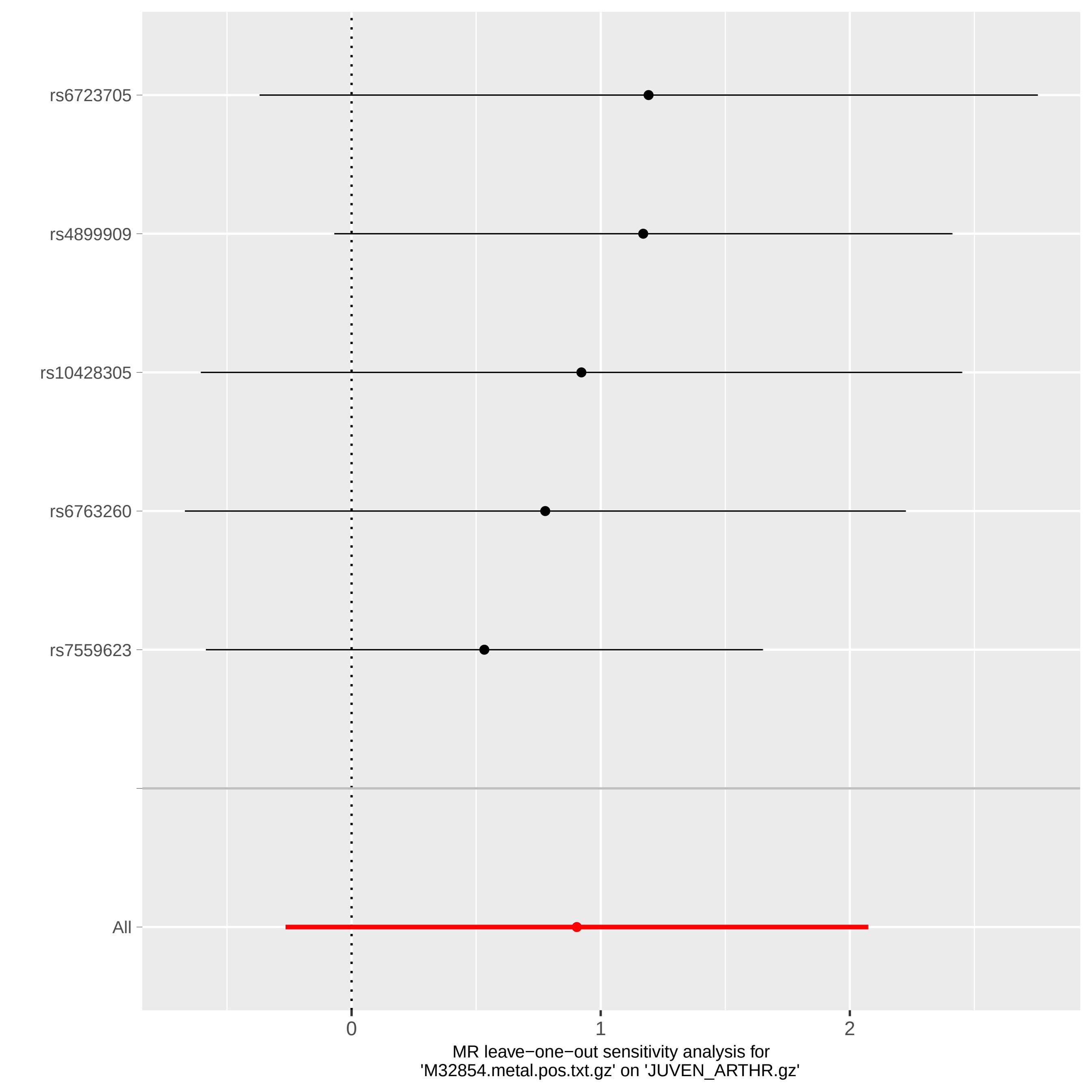

rs6723705
rs4899909
rs10428305
rs6763260
rs7559623
All
0
1
2
MR leave−one−out sensitivity analysis for
'M32854.metal.pos.txt.gz' on 'JUVEN_ARTHR.gz'

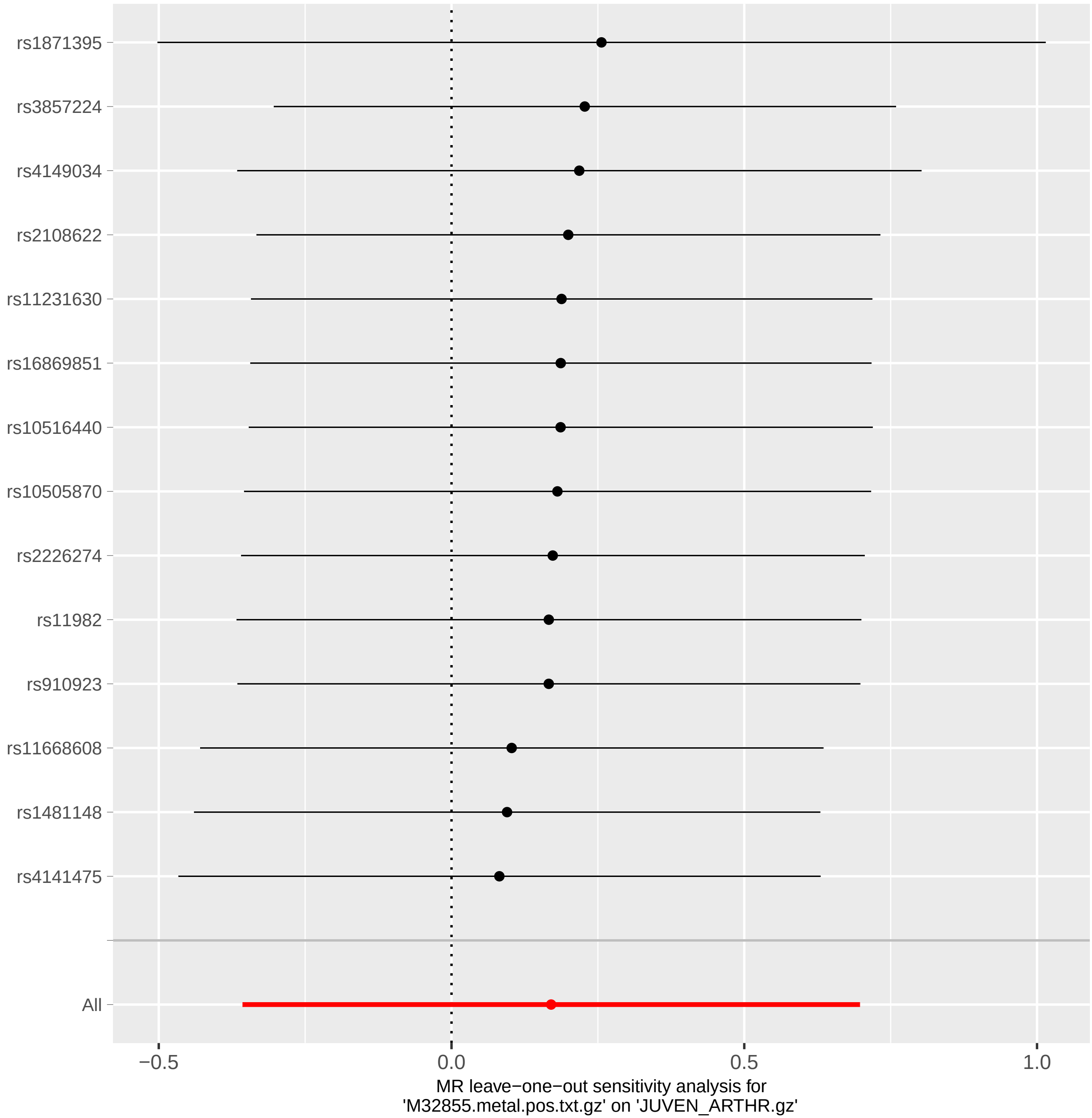
rs1871395
rs3857224
rs4149034
rs2108622
rs11231630
rs16869851
rs10516440
rs10505870
rs2226274
rs11982
rs910923
rs11668608
rs1481148
rs4141475
All
−0.5
0.0
0.5
1.0
MR leave−one−out sensitivity analysis for
'M32855.metal.pos.txt.gz' on 'JUVEN_ARTHR.gz'

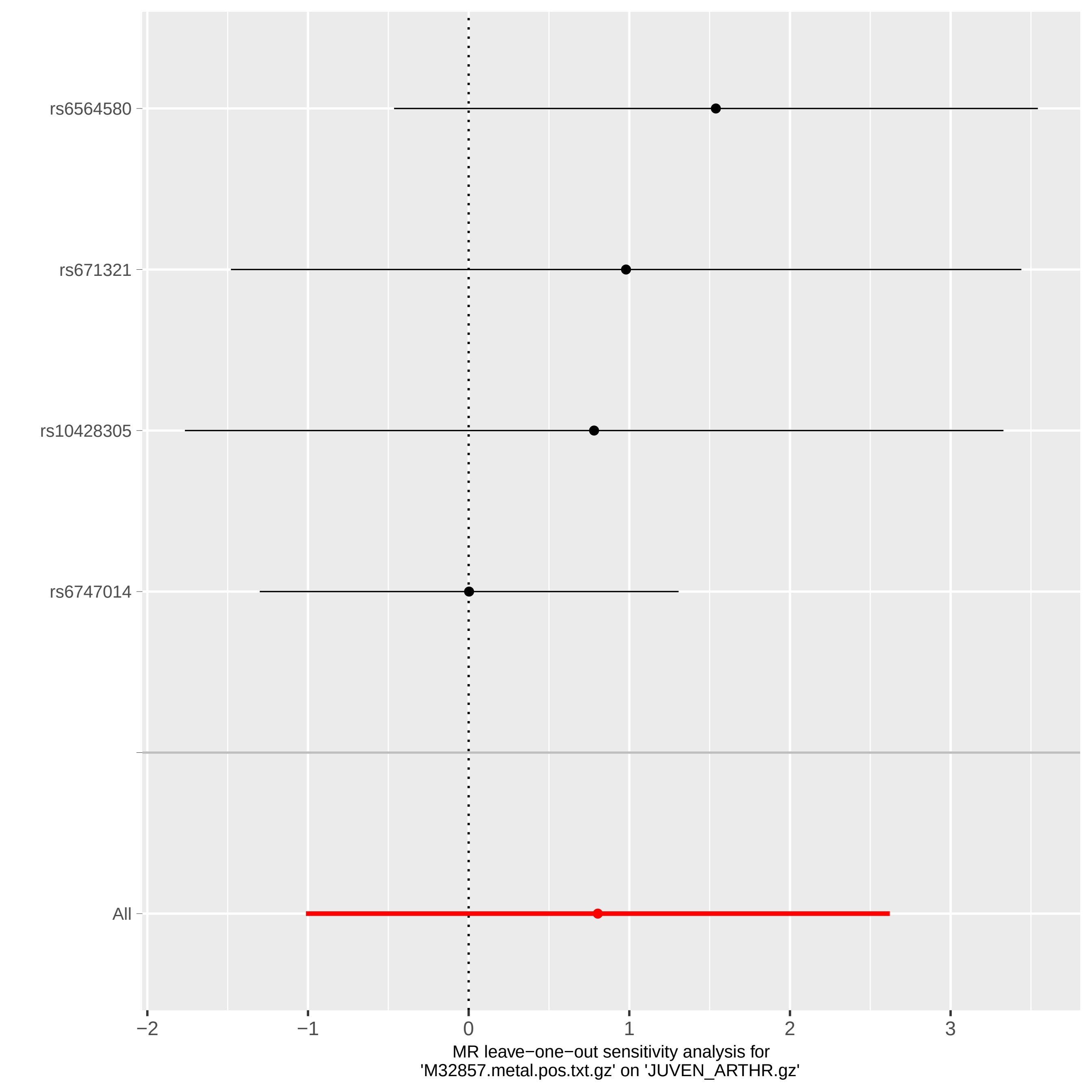

rs6564580
rs671321
rs10428305
rs6747014
All
−2
−1
0
1
2
3
MR leave−one−out sensitivity analysis for
'M32857.metal.pos.txt.gz' on 'JUVEN_ARTHR.gz'

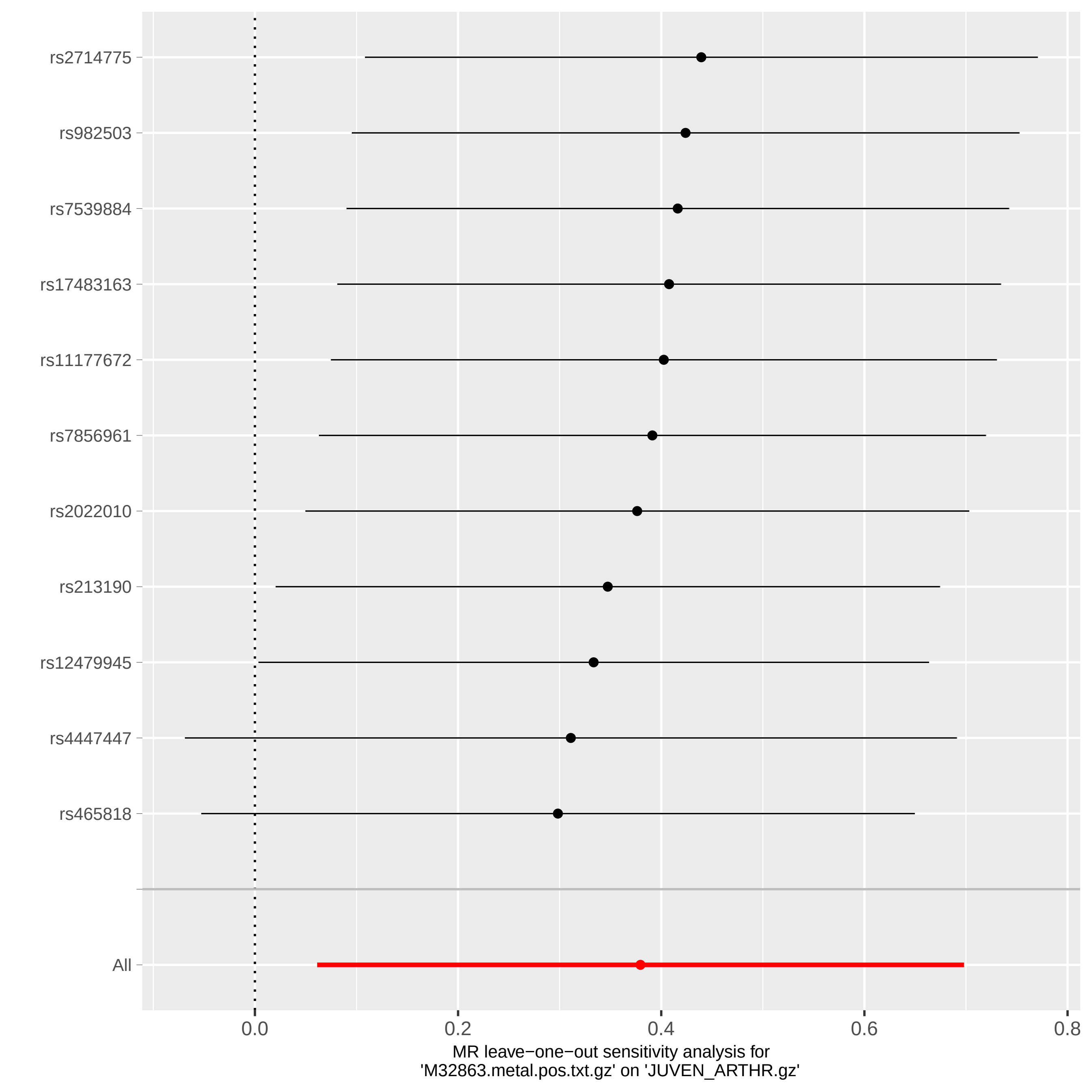

rs2714775
rs982503
rs7539884
rs17483163
rs11177672
rs7856961
rs2022010
rs213190
rs12479945
rs4447447
rs465818
All
0.0
0.2
0.4
0.6
0.8
MR leave−one−out sensitivity analysis for
'M32863.metal.pos.txt.gz' on 'JUVEN_ARTHR.gz'

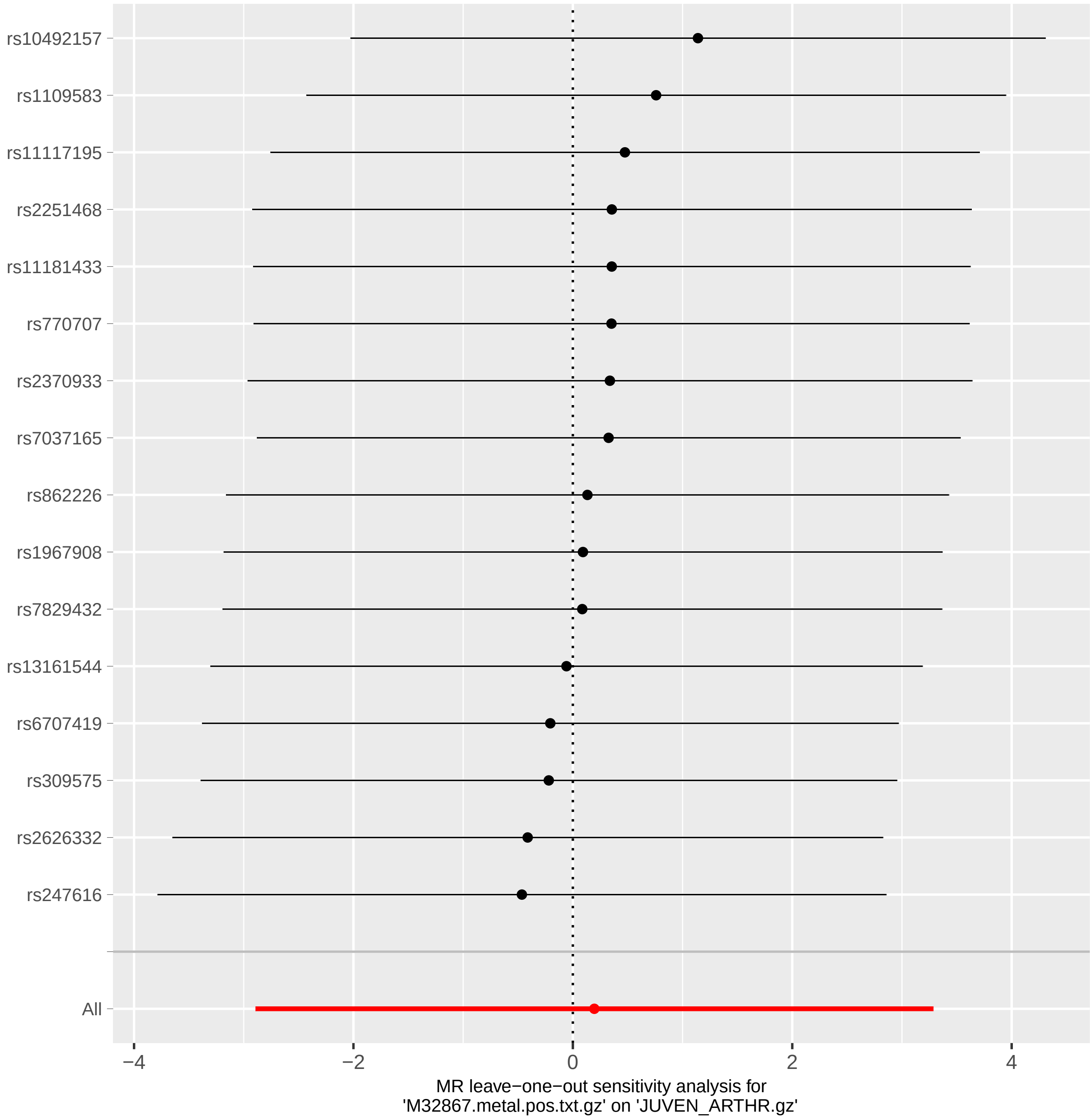
rs10492157
rs1109583
rs11117195
rs2251468
rs11181433
rs770707
rs2370933
rs7037165
rs862226
rs1967908
rs7829432
rs13161544
rs6707419
rs309575
rs2626332
rs247616
All
−4
−2
0
2
4
MR leave−one−out sensitivity analysis for
'M32867.metal.pos.txt.gz' on 'JUVEN_ARTHR.gz'

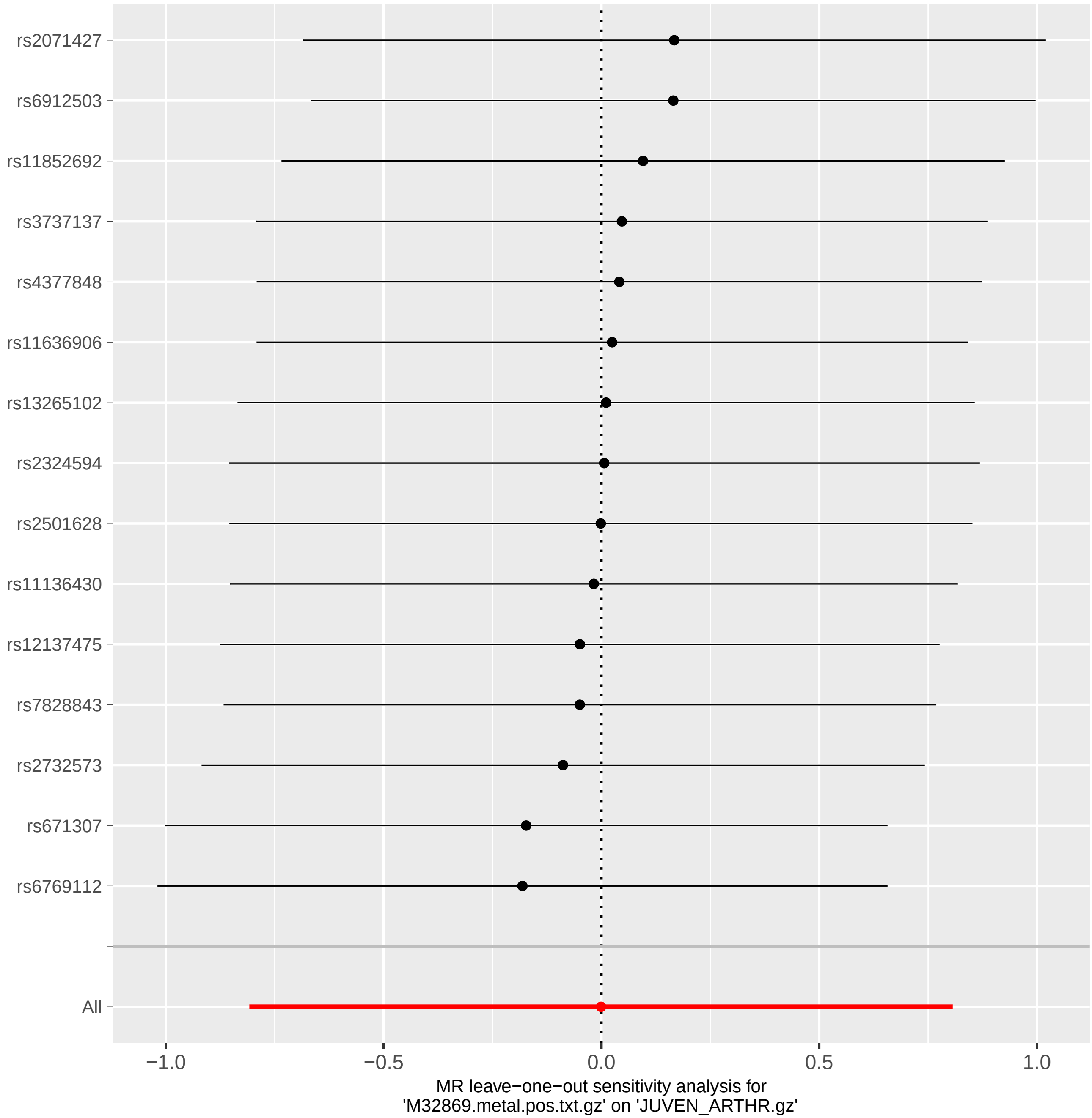
rs2071427
rs6912503
rs11852692
rs3737137
rs4377848
rs11636906
rs13265102
rs2324594
rs2501628
rs11136430
rs12137475
rs7828843
rs2732573
rs671307
rs6769112
All
−1.0
−0.5
0.0
0.5
1.0
MR leave−one−out sensitivity analysis for
'M32869.metal.pos.txt.gz' on 'JUVEN_ARTHR.gz'

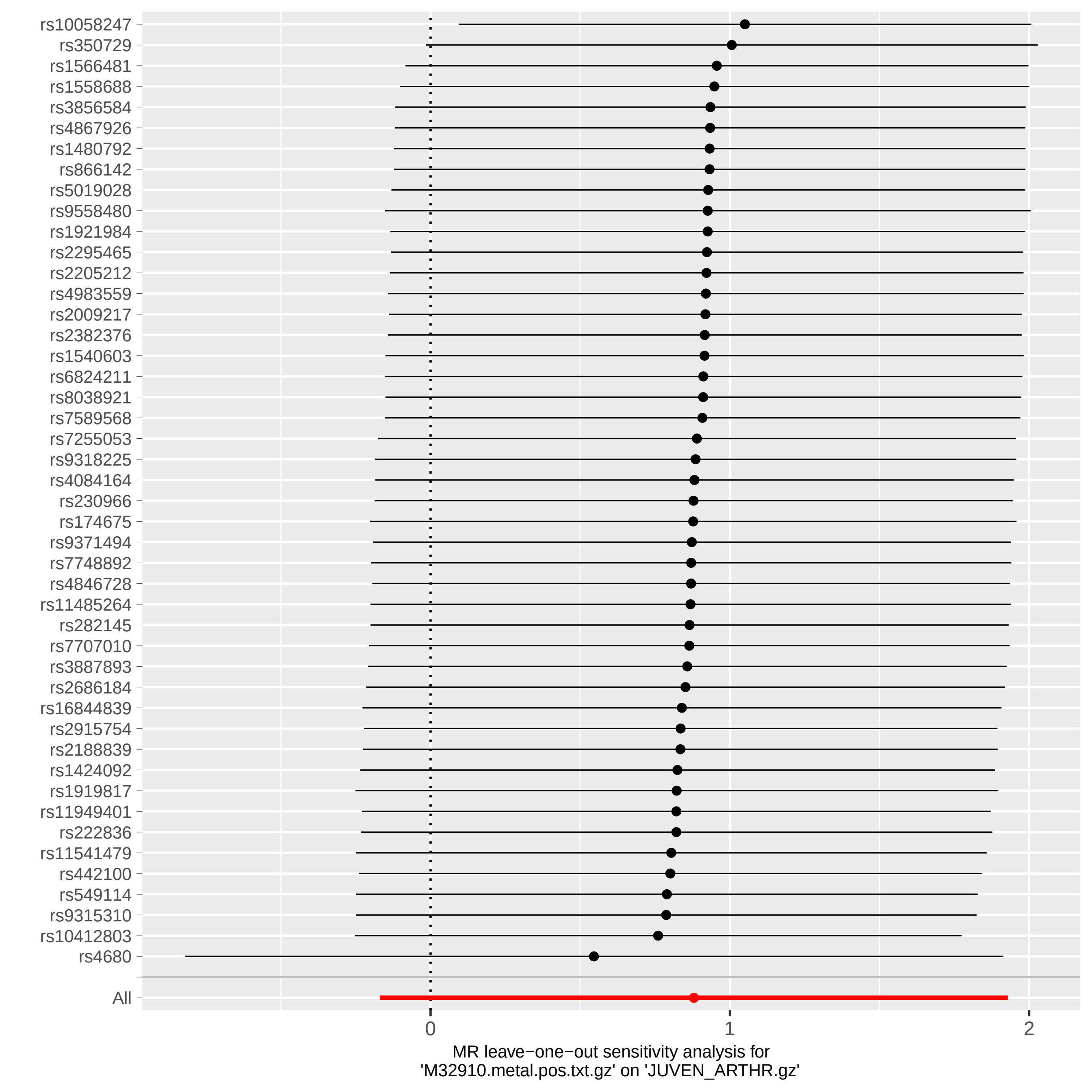
rs10058247
rs350729
rs1566481
rs1558688
rs3856584
rs4867926
rs1480792
rs866142
rs5019028
rs9558480
rs1921984
rs2295465
rs2205212
rs4983559
rs2009217
rs2382376
rs1540603
rs6824211
rs8038921
rs7589568
rs7255053
rs9318225
rs4084164
rs230966
rs174675
rs9371494
rs7748892
rs4846728
rs11485264
rs282145
rs7707010
rs3887893
rs2686184
rs16844839
rs2915754
rs2188839
rs1424092
rs1919817
rs11949401
rs222836
rs11541479
rs442100
rs549114
rs9315310
rs10412803
rs4680
All
0
1
2
MR leave−one−out sensitivity analysis for
'M32910.metal.pos.txt.gz' on 'JUVEN_ARTHR.gz'

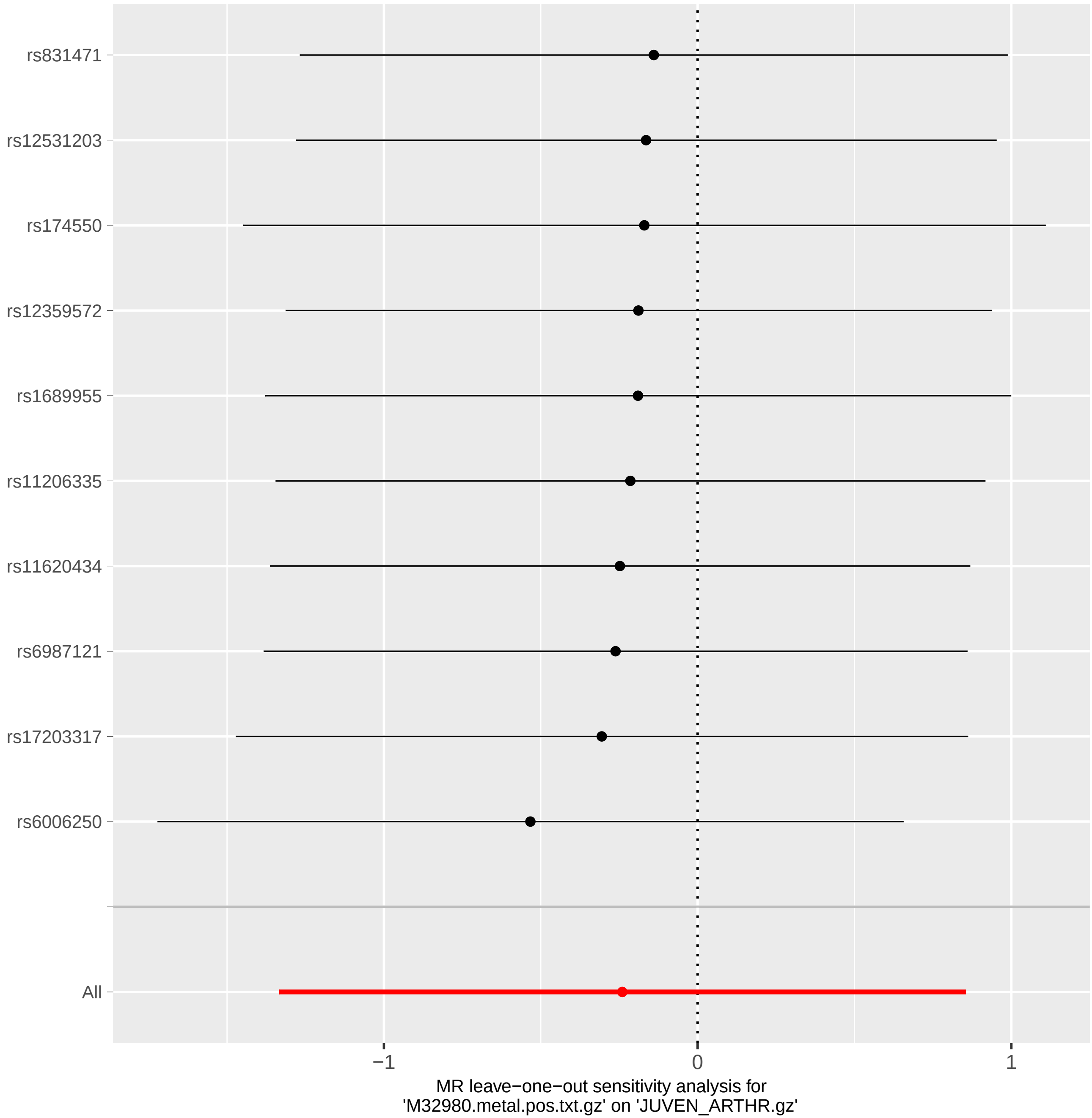
rs831471
rs12531203
rs174550
rs12359572
rs1689955
rs11206335
rs11620434
rs6987121
rs17203317
rs6006250
All
−1
0
1
MR leave−one−out sensitivity analysis for
'M32980.metal.pos.txt.gz' on 'JUVEN_ARTHR.gz'

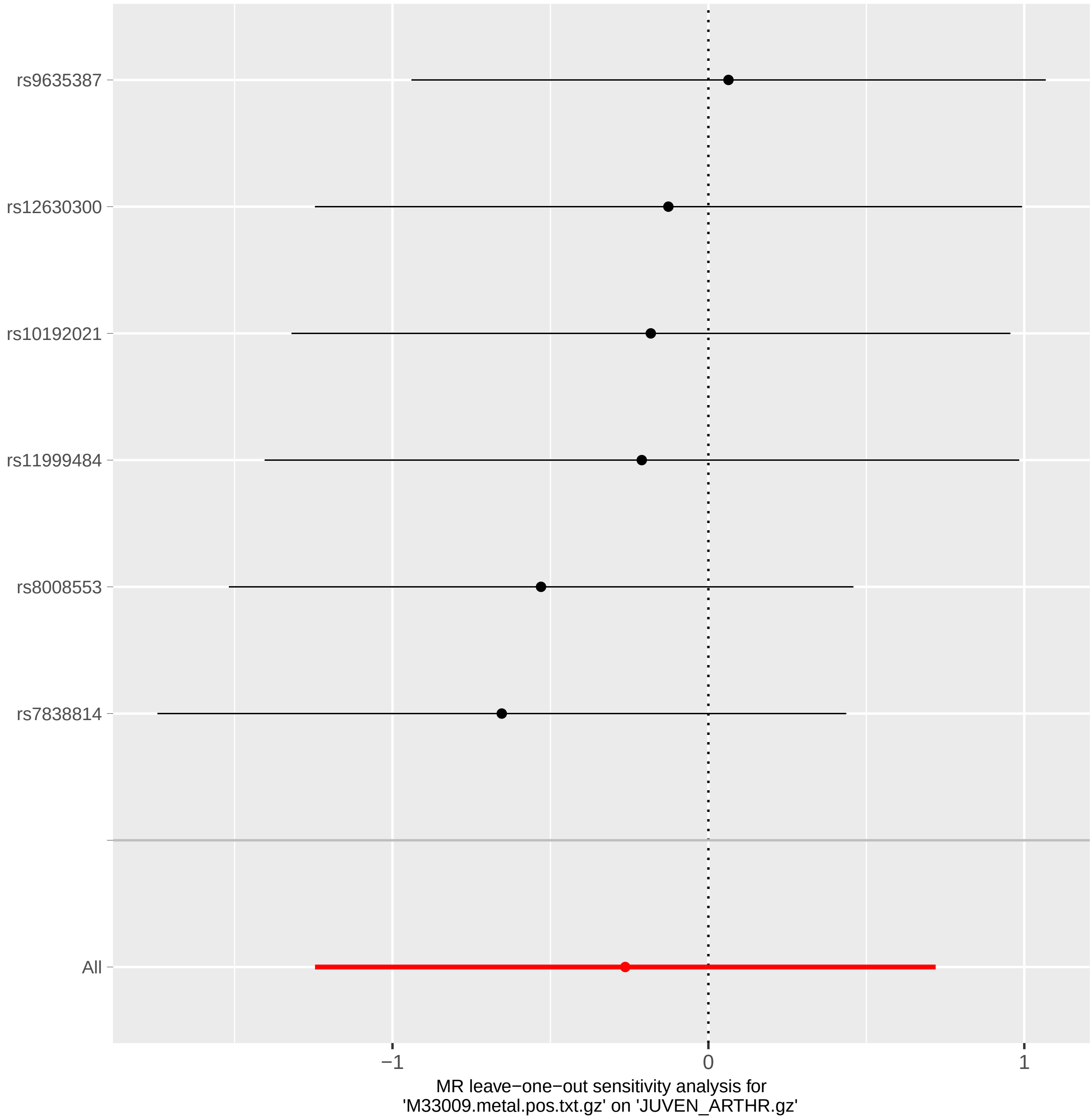
rs9635387
rs12630300
rs10192021
rs11999484
rs8008553
rs7838814
All
−1
0
1
MR leave−one−out sensitivity analysis for
'M33009.metal.pos.txt.gz' on 'JUVEN_ARTHR.gz'

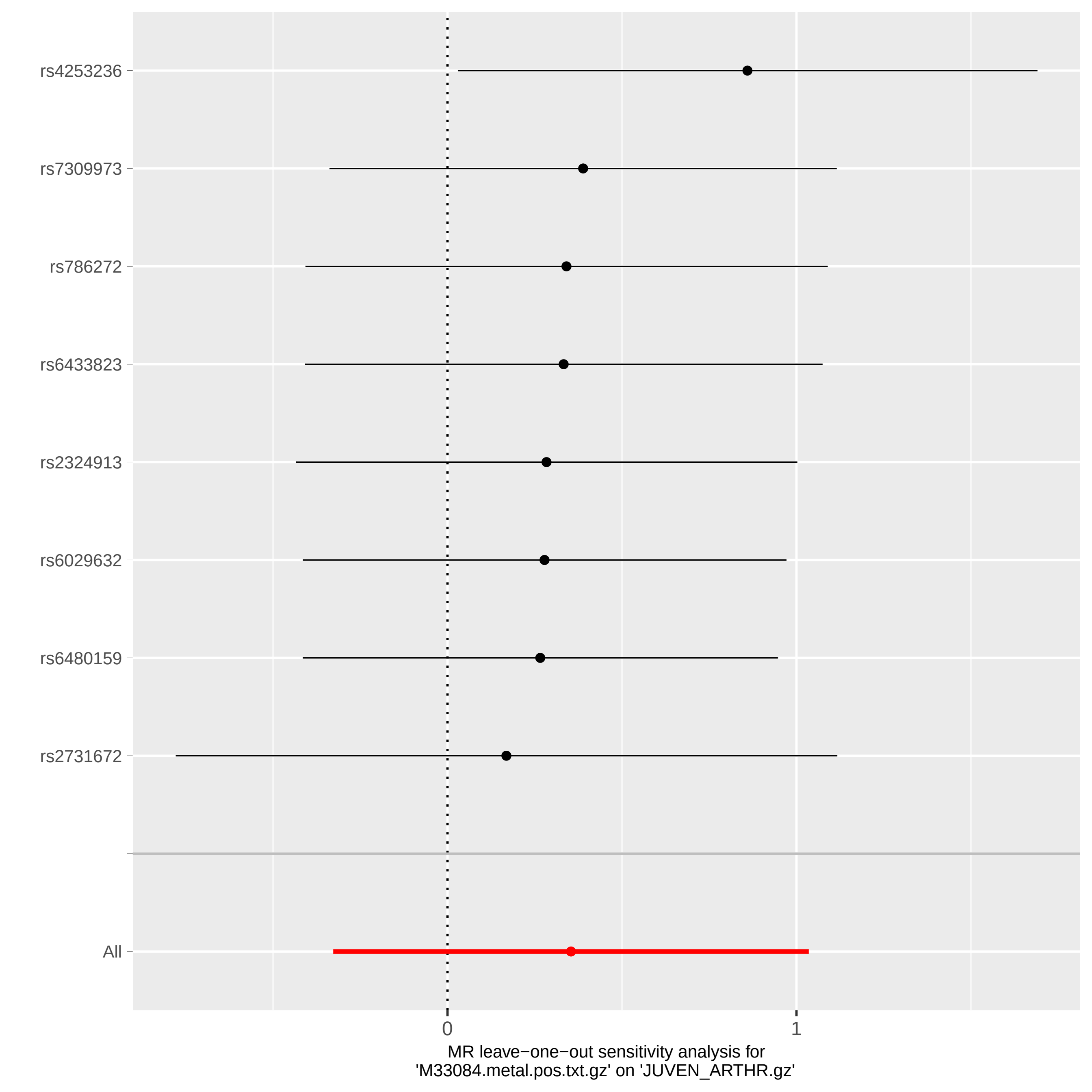

rs4253236
rs7309973
rs786272
rs6433823
rs2324913
rs6029632
rs6480159
rs2731672
All
0
1
MR leave−one−out sensitivity analysis for
'M33084.metal.pos.txt.gz' on 'JUVEN_ARTHR.gz'

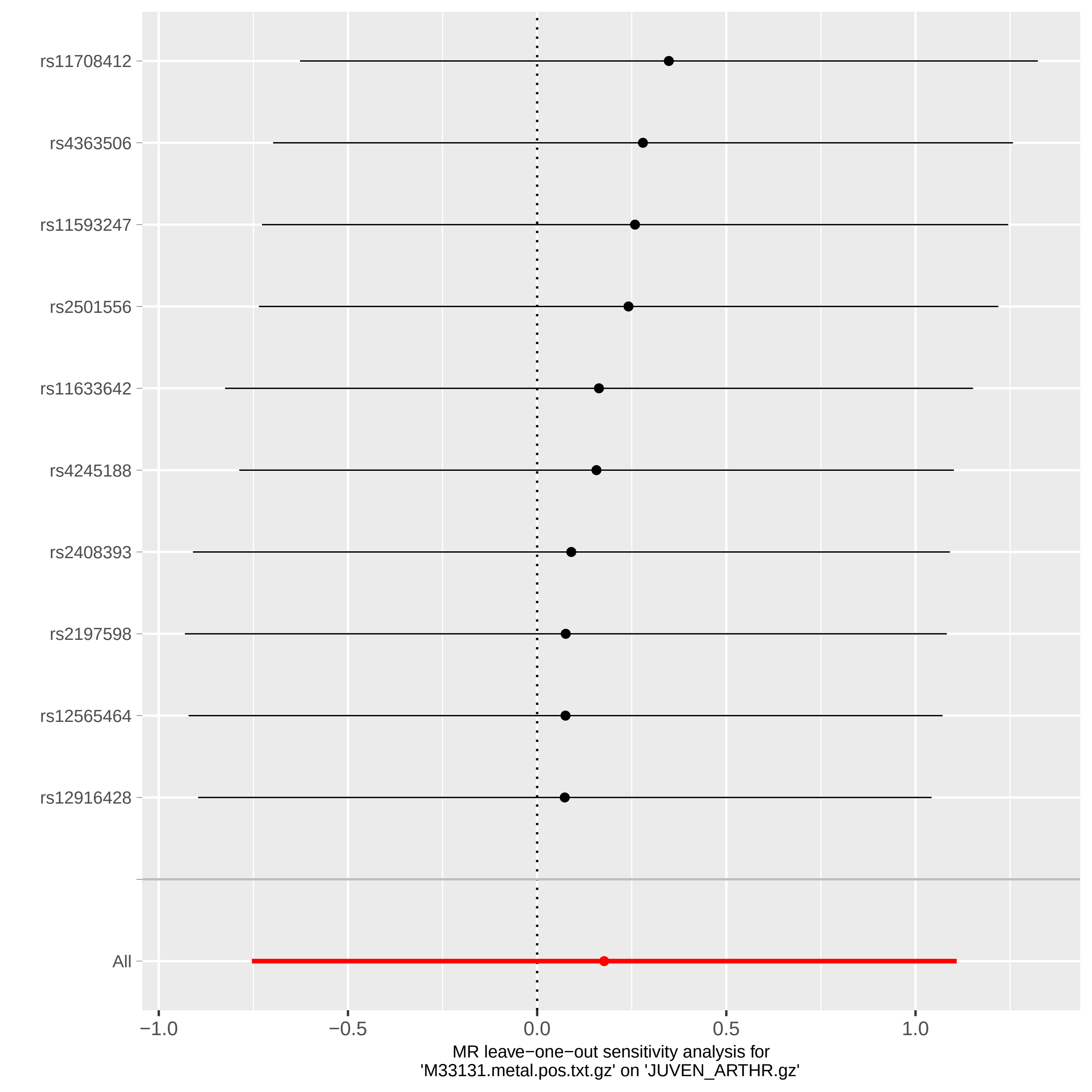
rs11708412
rs4363506
rs11593247
rs2501556
rs11633642
rs4245188
rs2408393
rs2197598
rs12565464
rs12916428
All
−1.0
−0.5
0.0
0.5
1.0
MR leave−one−out sensitivity analysis for
'M33131.metal.pos.txt.gz' on 'JUVEN_ARTHR.gz'

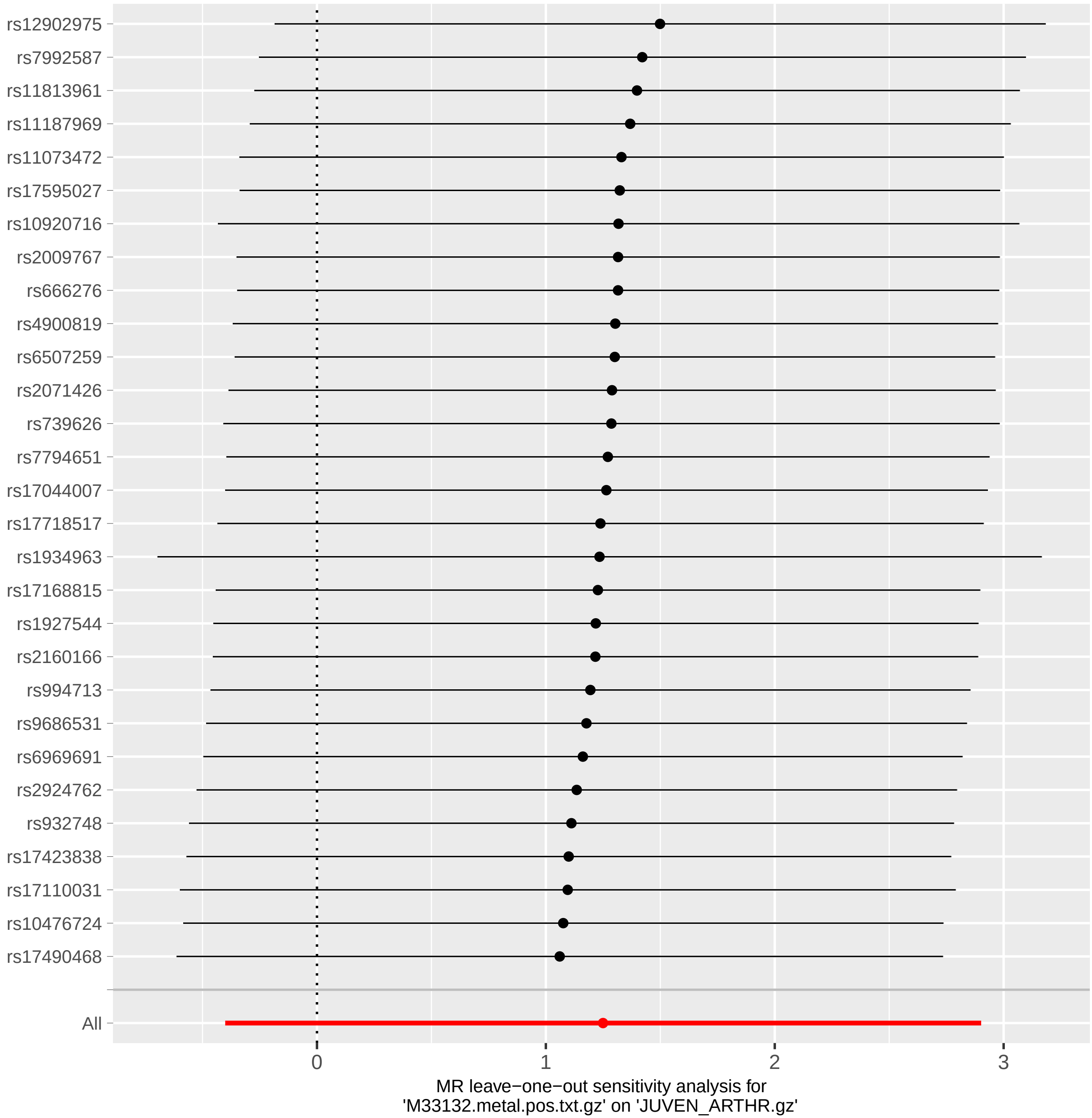

rs12902975
rs7992587
rs11813961
rs11187969
rs11073472
rs17595027
rs10920716
rs2009767
rs666276
rs4900819
rs6507259
rs2071426
rs739626
rs7794651
rs17044007
rs17718517
rs1934963
rs17168815
rs1927544
rs2160166
rs994713
rs9686531
rs6969691
rs2924762
rs932748
rs17423838
rs17110031
rs10476724
rs17490468
All
0
1
2
3
MR leave−one−out sensitivity analysis for
'M33132.metal.pos.txt.gz' on 'JUVEN_ARTHR.gz'

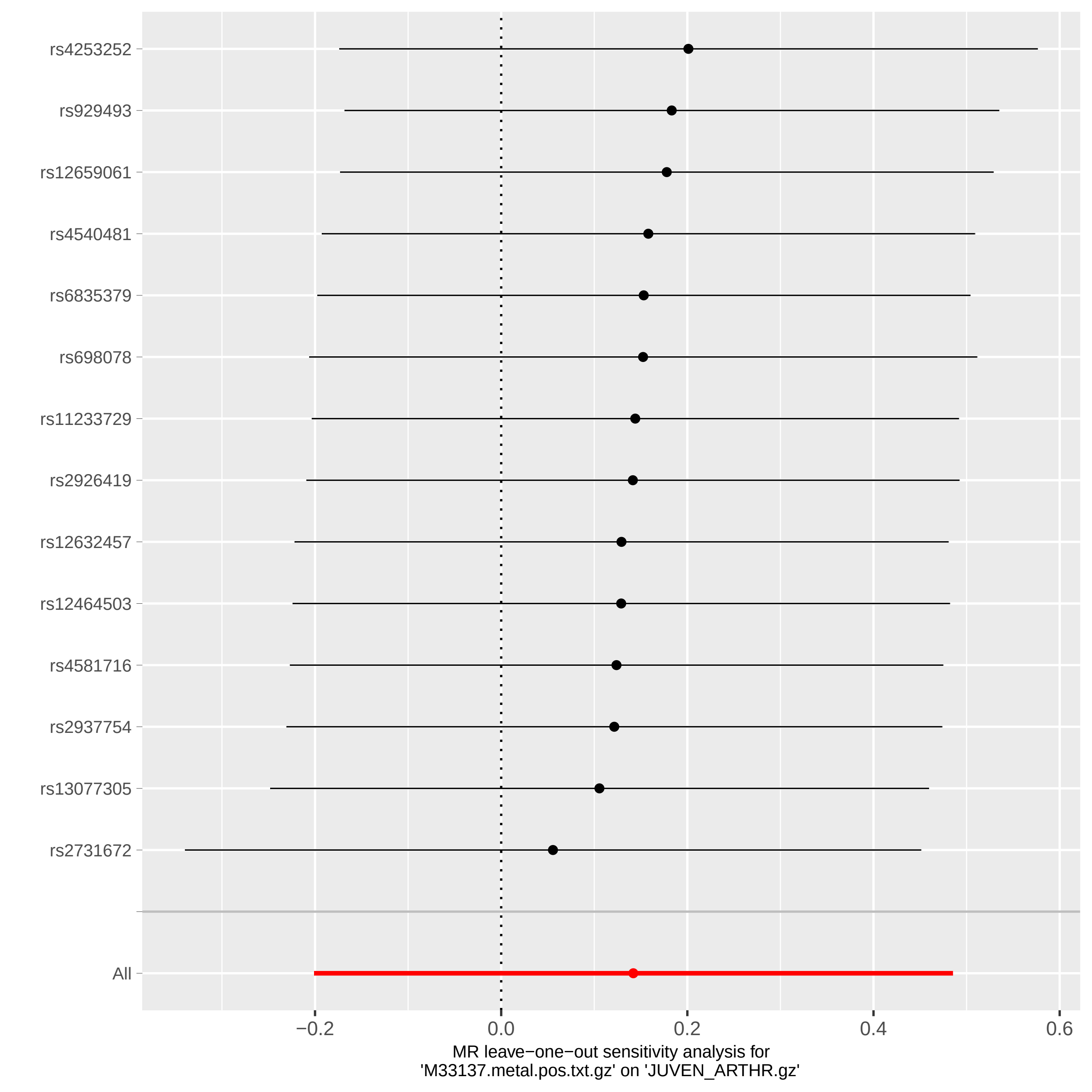
rs4253252
rs929493
rs12659061
rs4540481
rs6835379
rs698078
rs11233729
rs2926419
rs12632457
rs12464503
rs4581716
rs2937754
rs13077305
rs2731672
All
−0.2
0.0
0.2
0.4
0.6
MR leave−one−out sensitivity analysis for
'M33137.metal.pos.txt.gz' on 'JUVEN_ARTHR.gz'

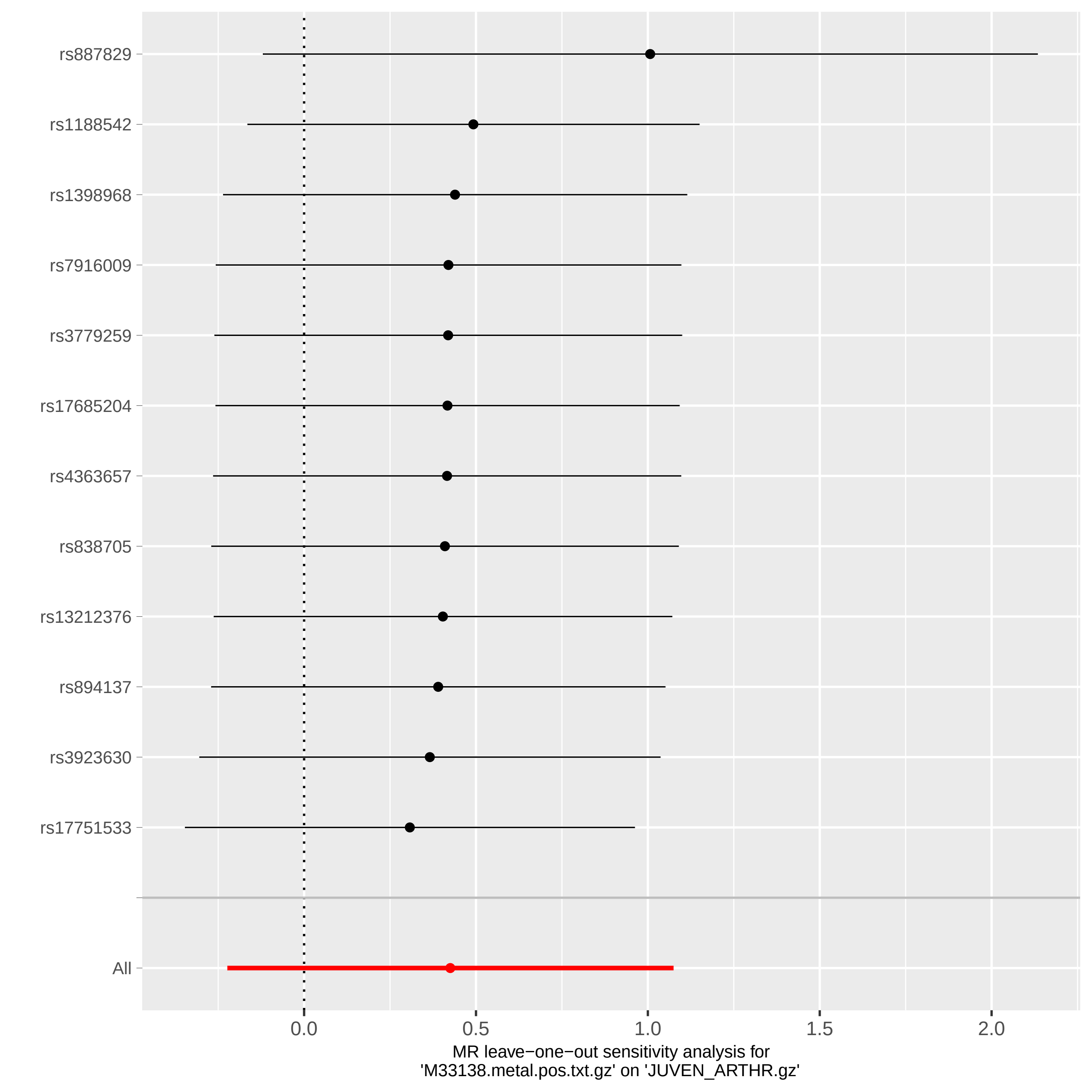

rs887829
rs1188542
rs1398968
rs7916009
rs3779259
rs17685204
rs4363657
rs838705
rs13212376
rs894137
rs3923630
rs17751533
All
0.0
0.5
1.0
1.5
2.0
MR leave−one−out sensitivity analysis for
'M33138.metal.pos.txt.gz' on 'JUVEN_ARTHR.gz'

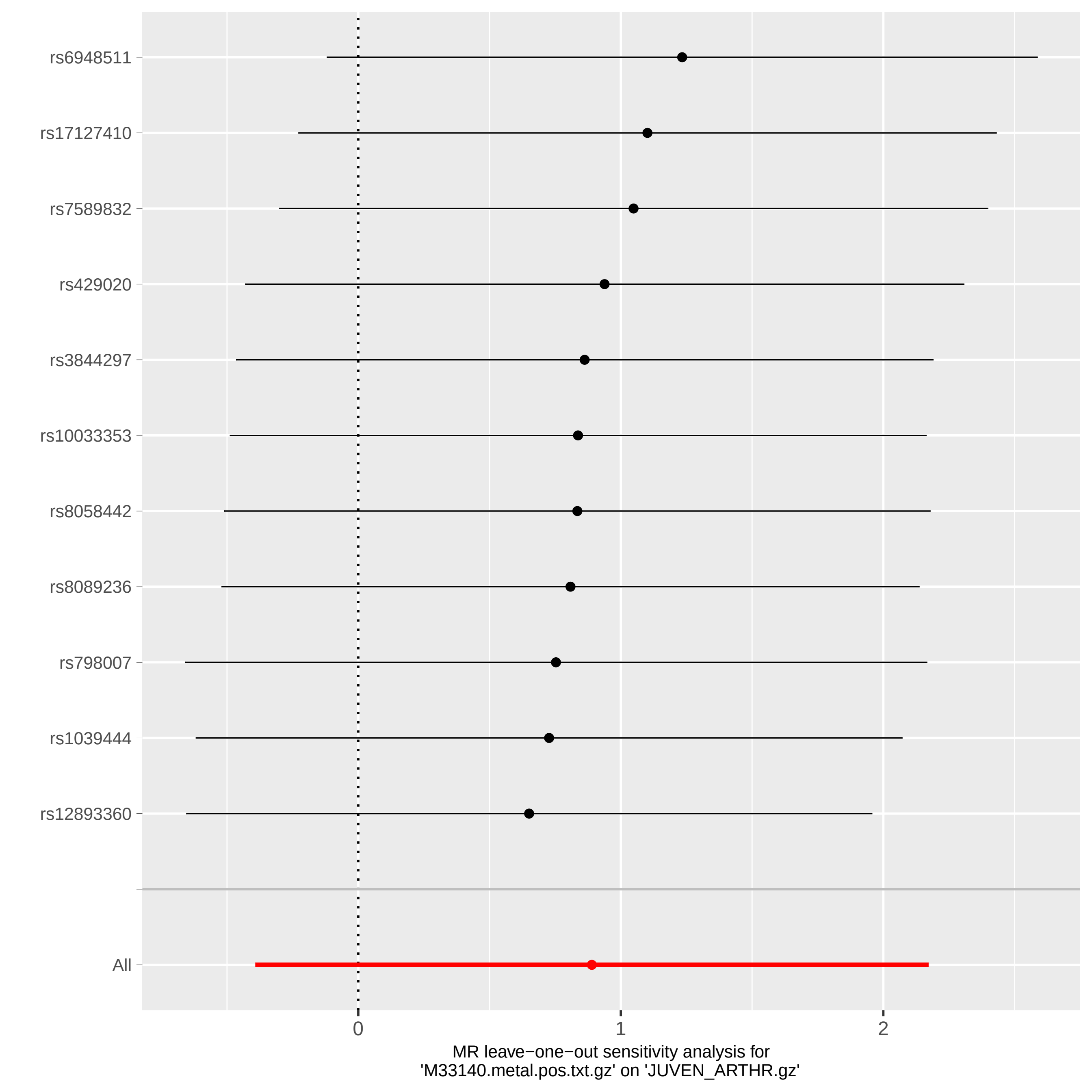
rs6948511
rs17127410
rs7589832
rs429020
rs3844297
rs10033353
rs8058442
rs8089236
rs798007
rs1039444
rs12893360
All
0
1
2
MR leave−one−out sensitivity analysis for
'M33140.metal.pos.txt.gz' on 'JUVEN_ARTHR.gz'

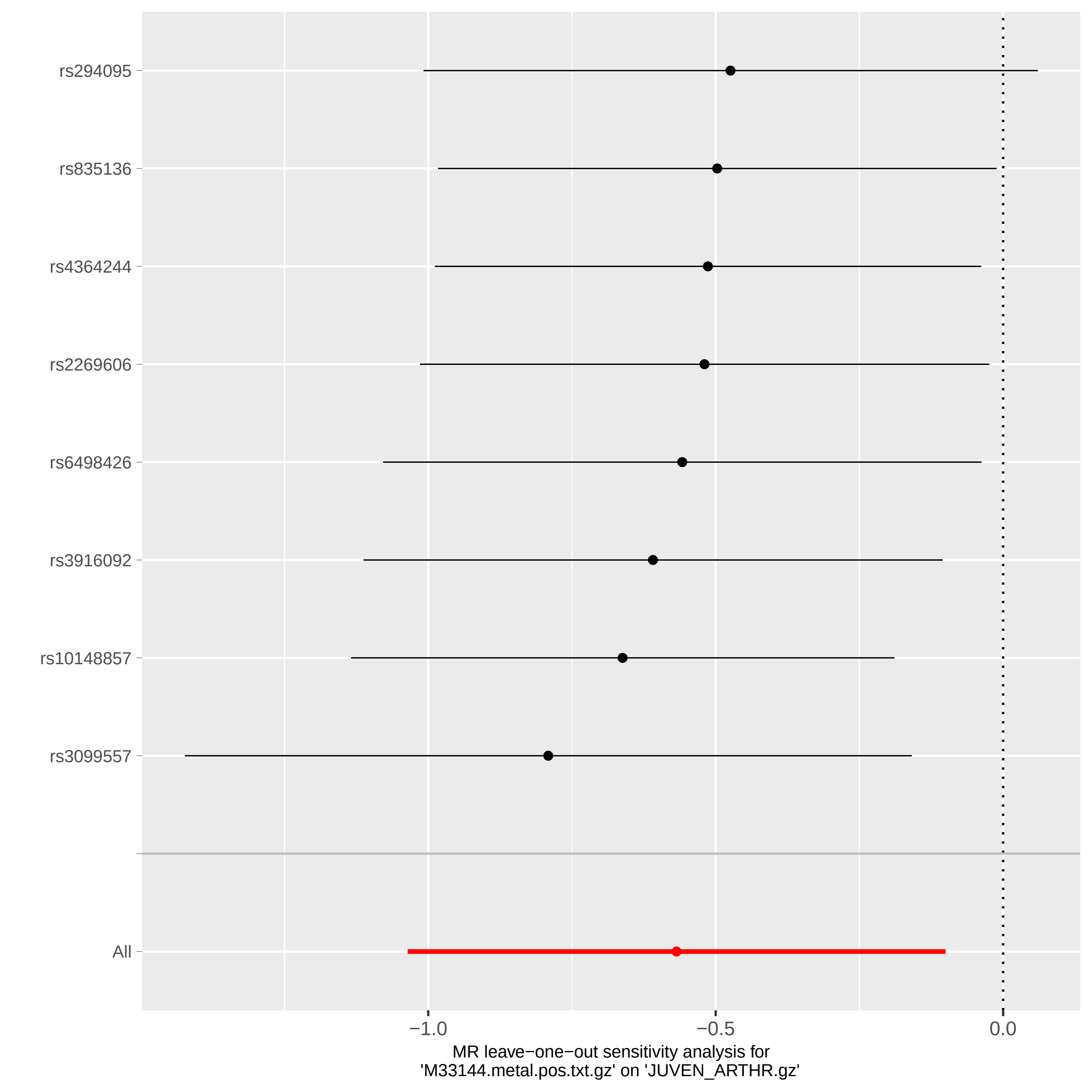
rs294095
rs835136
rs4364244
rs2269606
rs6498426
rs3916092
rs10148857
rs3099557
All
−1.0
−0.5
0.0
MR leave−one−out sensitivity analysis for
'M33144.metal.pos.txt.gz' on 'JUVEN_ARTHR.gz'

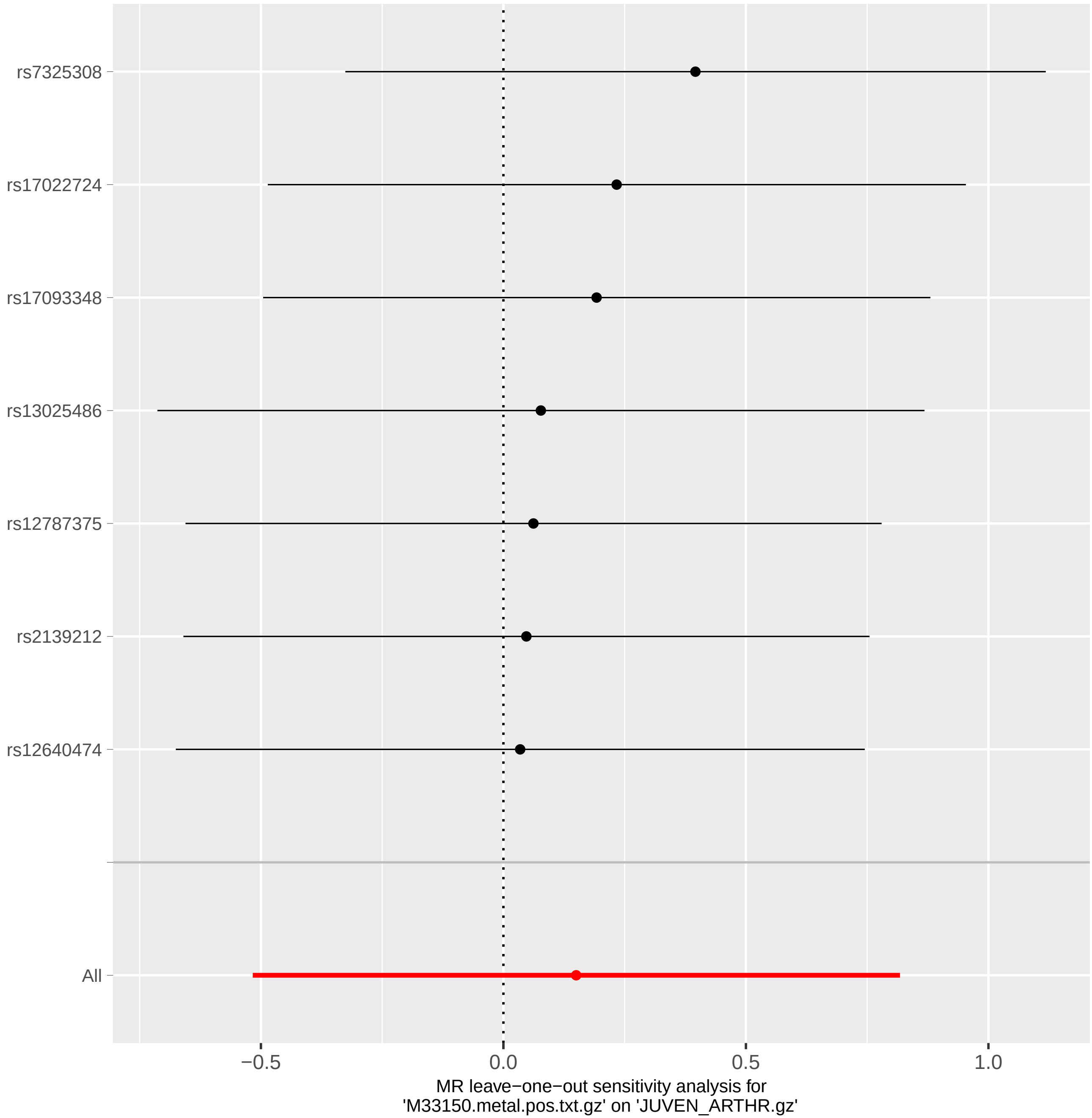
rs7325308
rs17022724
rs17093348
rs13025486
rs12787375
rs2139212
rs12640474
All
−0.5
0.0
0.5
1.0
MR leave−one−out sensitivity analysis for
'M33150.metal.pos.txt.gz' on 'JUVEN_ARTHR.gz'

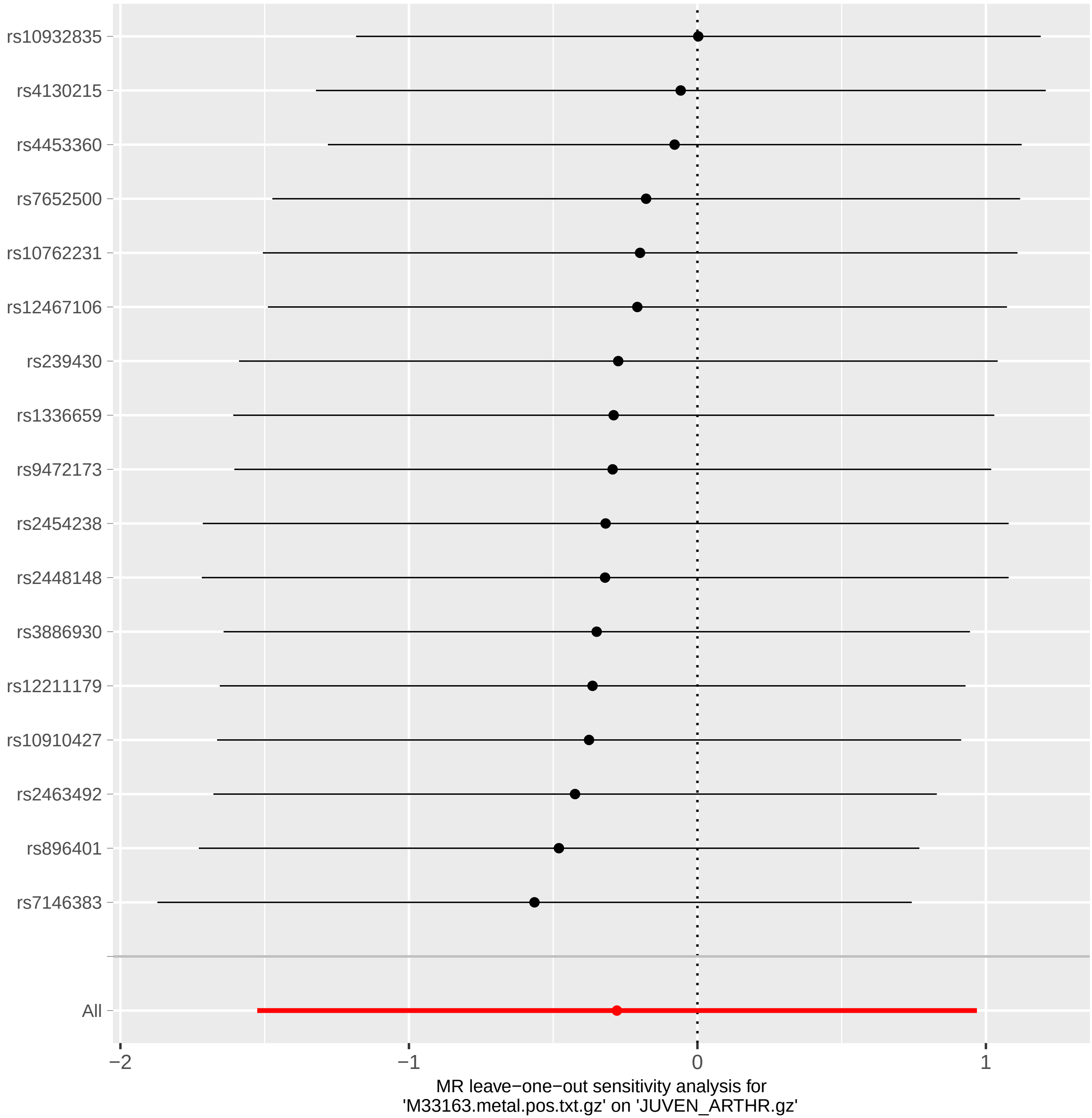
rs10932835
rs4130215
rs4453360
rs7652500
rs10762231
rs12467106
rs239430
rs1336659
rs9472173
rs2454238
rs2448148
rs3886930
rs12211179
rs10910427
rs2463492
rs896401
rs7146383
All
−2
−1
0
1
MR leave−one−out sensitivity analysis for
'M33163.metal.pos.txt.gz' on 'JUVEN_ARTHR.gz'

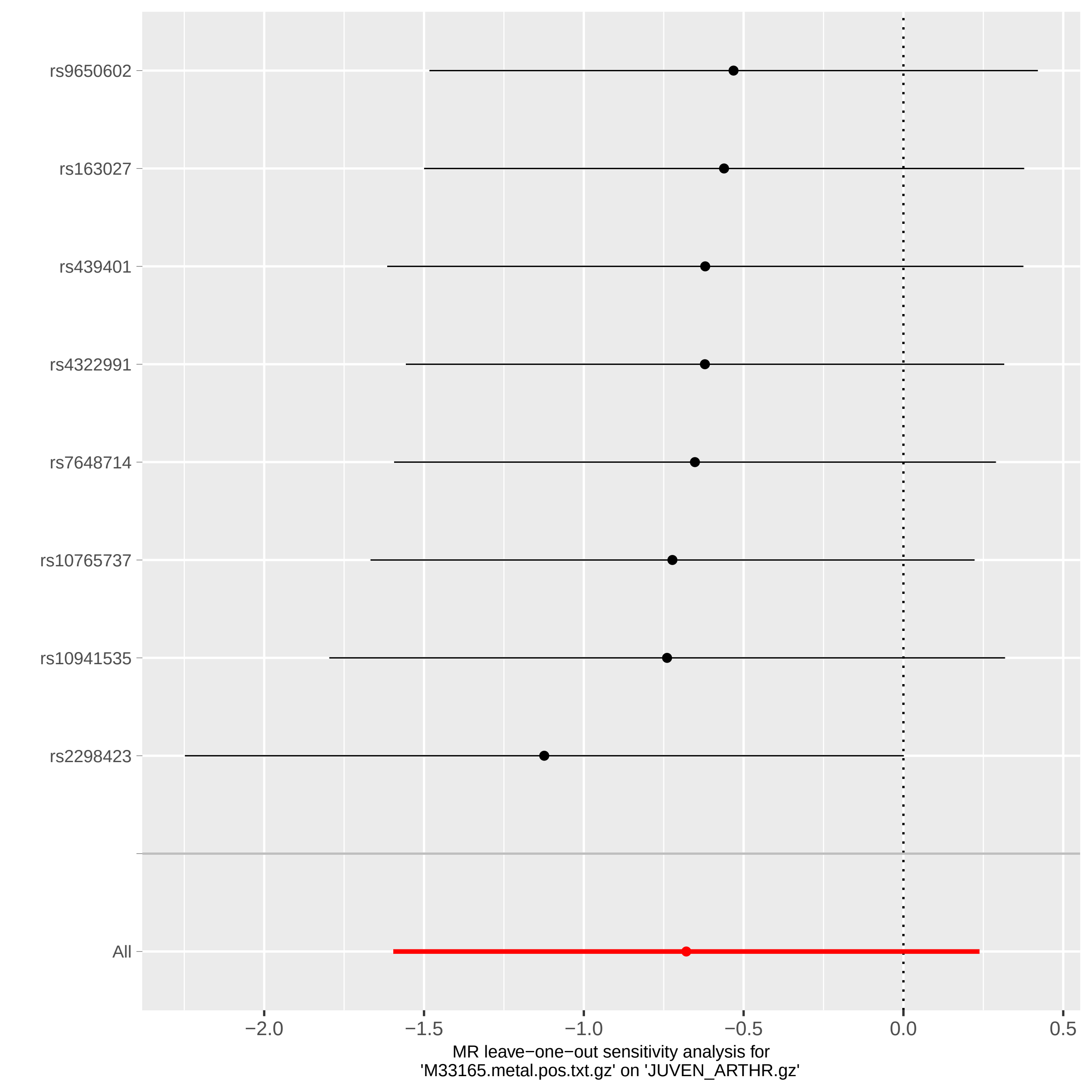
rs9650602
rs163027
rs439401
rs4322991
rs7648714
rs10765737
rs10941535
rs2298423
All
−2.0
−1.5
−1.0
−0.5
0.0
0.5
MR leave−one−out sensitivity analysis for
'M33165.metal.pos.txt.gz' on 'JUVEN_ARTHR.gz'

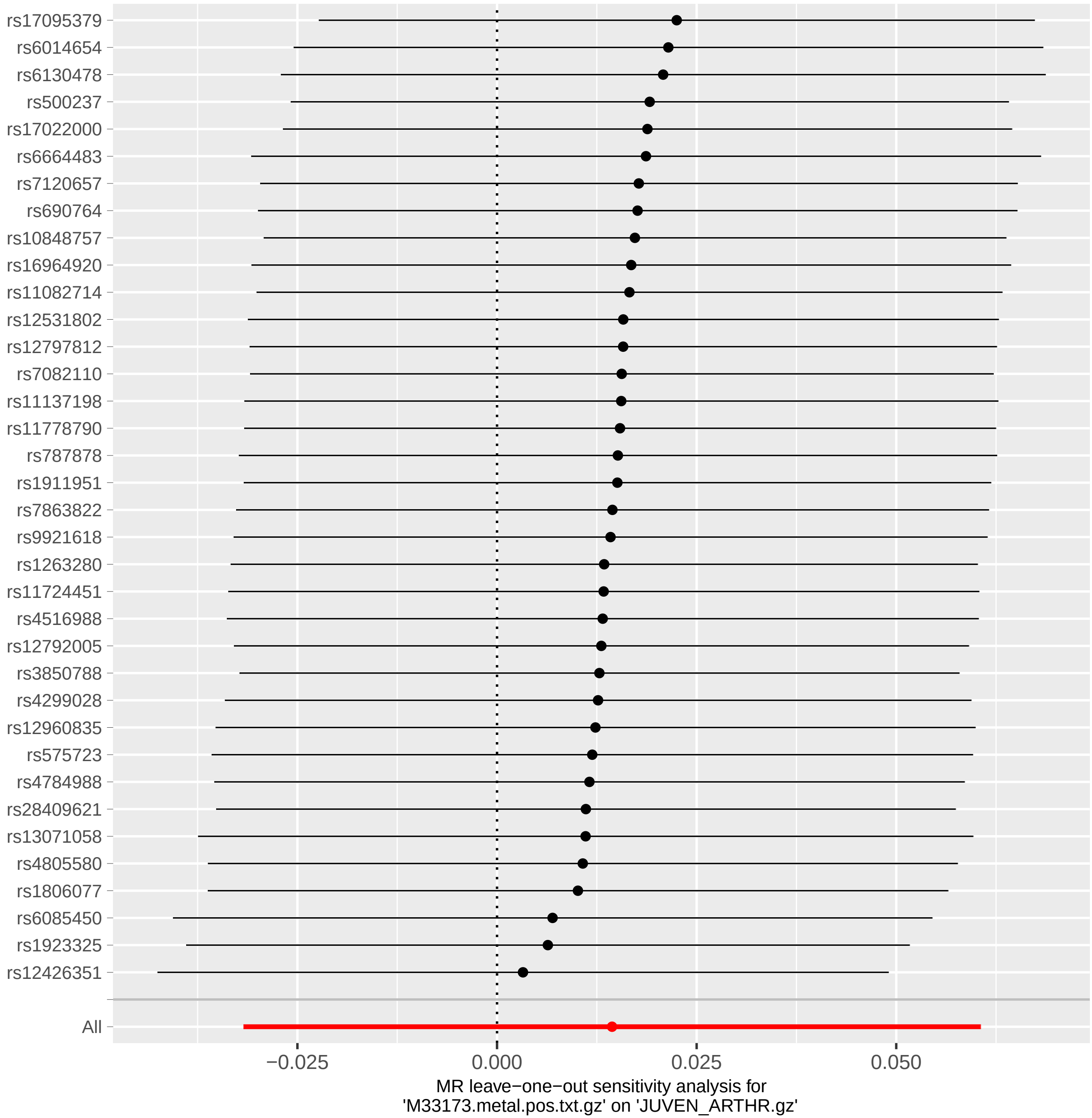
rs17095379
rs6014654
rs6130478
rs500237
rs17022000
rs6664483
rs7120657
rs690764
rs10848757
rs16964920
rs11082714
rs12531802
rs12797812
rs7082110
rs11137198
rs11778790
rs787878
rs1911951
rs7863822
rs9921618
rs1263280
rs11724451
rs4516988
rs12792005
rs3850788
rs4299028
rs12960835
rs575723
rs4784988
rs28409621
rs13071058
rs4805580
rs1806077
rs6085450
rs1923325
rs12426351
All
−0.025
0.000
0.025
0.050
MR leave−one−out sensitivity analysis for
'M33173.metal.pos.txt.gz' on 'JUVEN_ARTHR.gz'

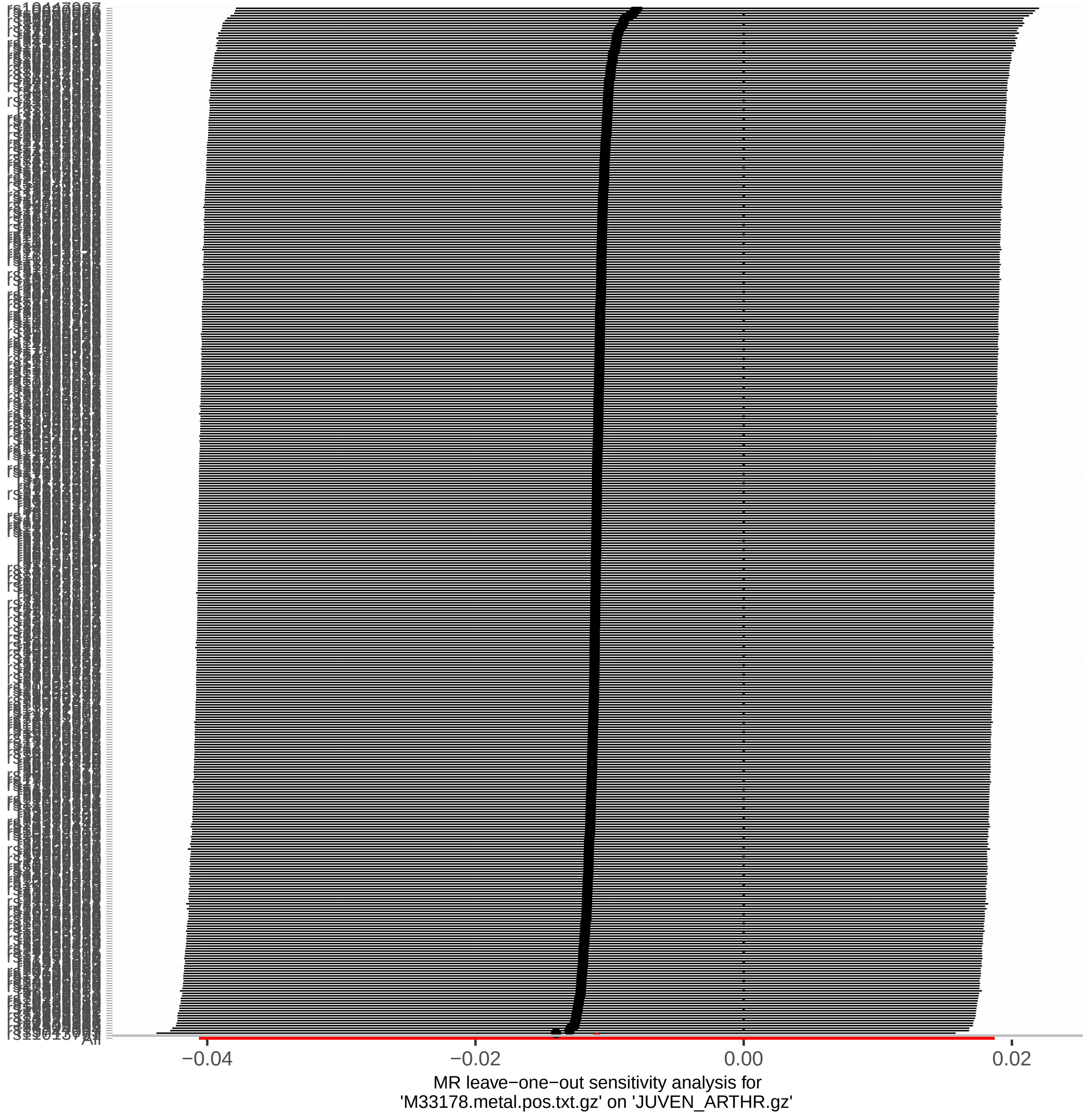

−0.04
−0.02
0.00
0.02
All
MR leave−one−out sensitivity analysis for
'M33178.metal.pos.txt.gz' on 'JUVEN_ARTHR.gz'

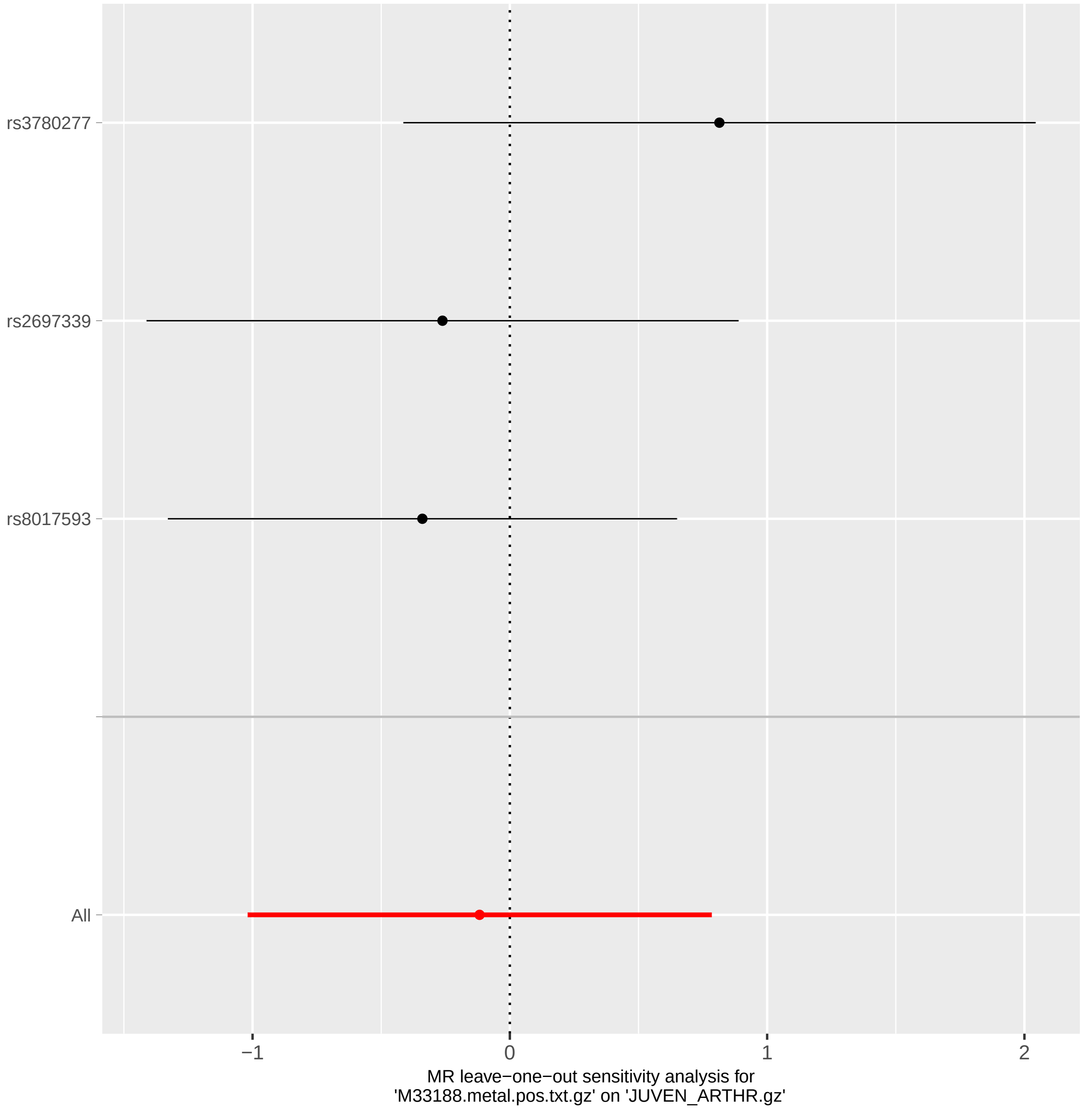
rs3780277
rs2697339
rs8017593
All
−1
0
1
2
MR leave−one−out sensitivity analysis for
'M33188.metal.pos.txt.gz' on 'JUVEN_ARTHR.gz'

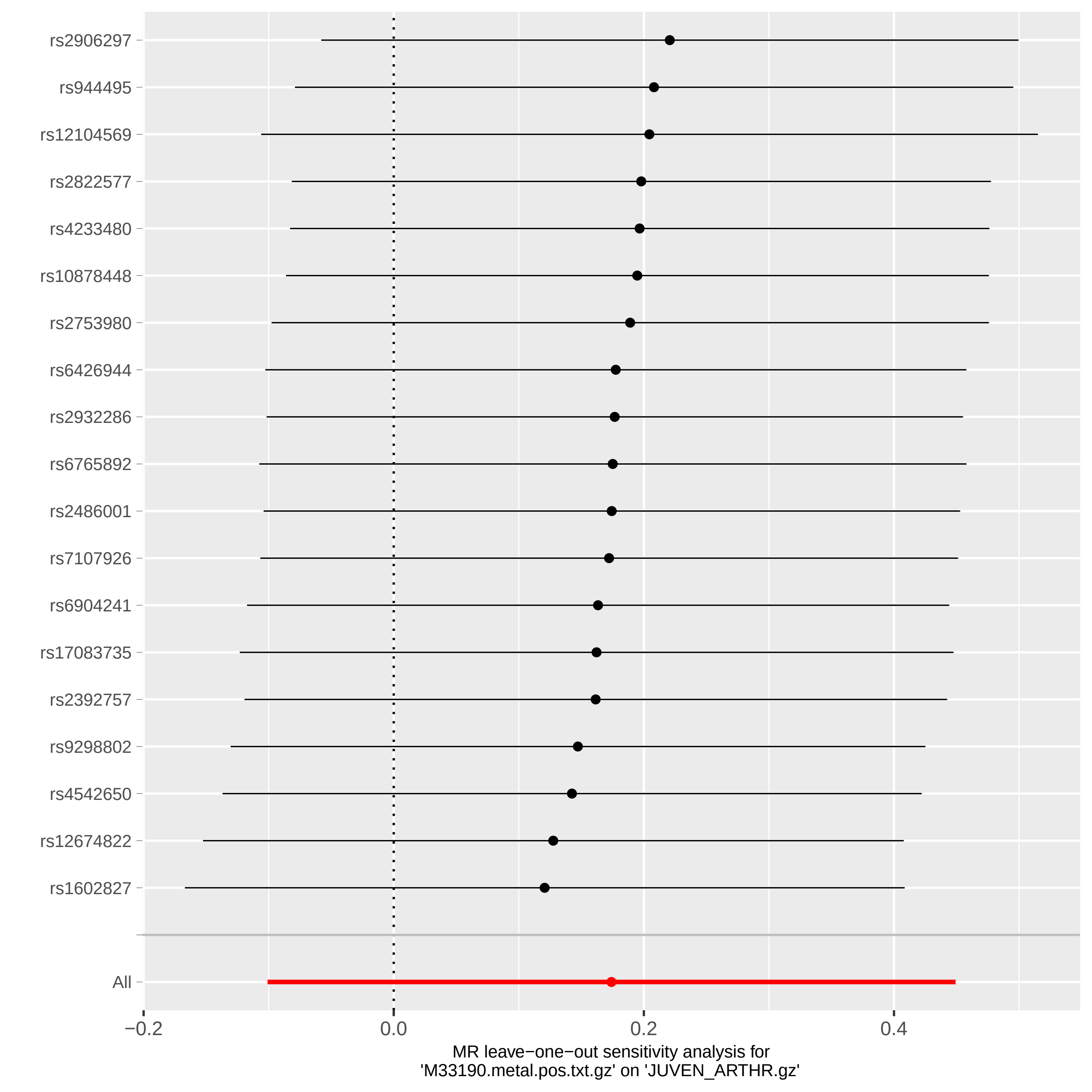
rs2906297
rs944495
rs12104569
rs2822577
rs4233480
rs10878448
rs2753980
rs6426944
rs2932286
rs6765892
rs2486001
rs7107926
rs6904241
rs17083735
rs2392757
rs9298802
rs4542650
rs12674822
rs1602827
All
−0.2
0.0
0.2
0.4
MR leave−one−out sensitivity analysis for
'M33190.metal.pos.txt.gz' on 'JUVEN_ARTHR.gz'

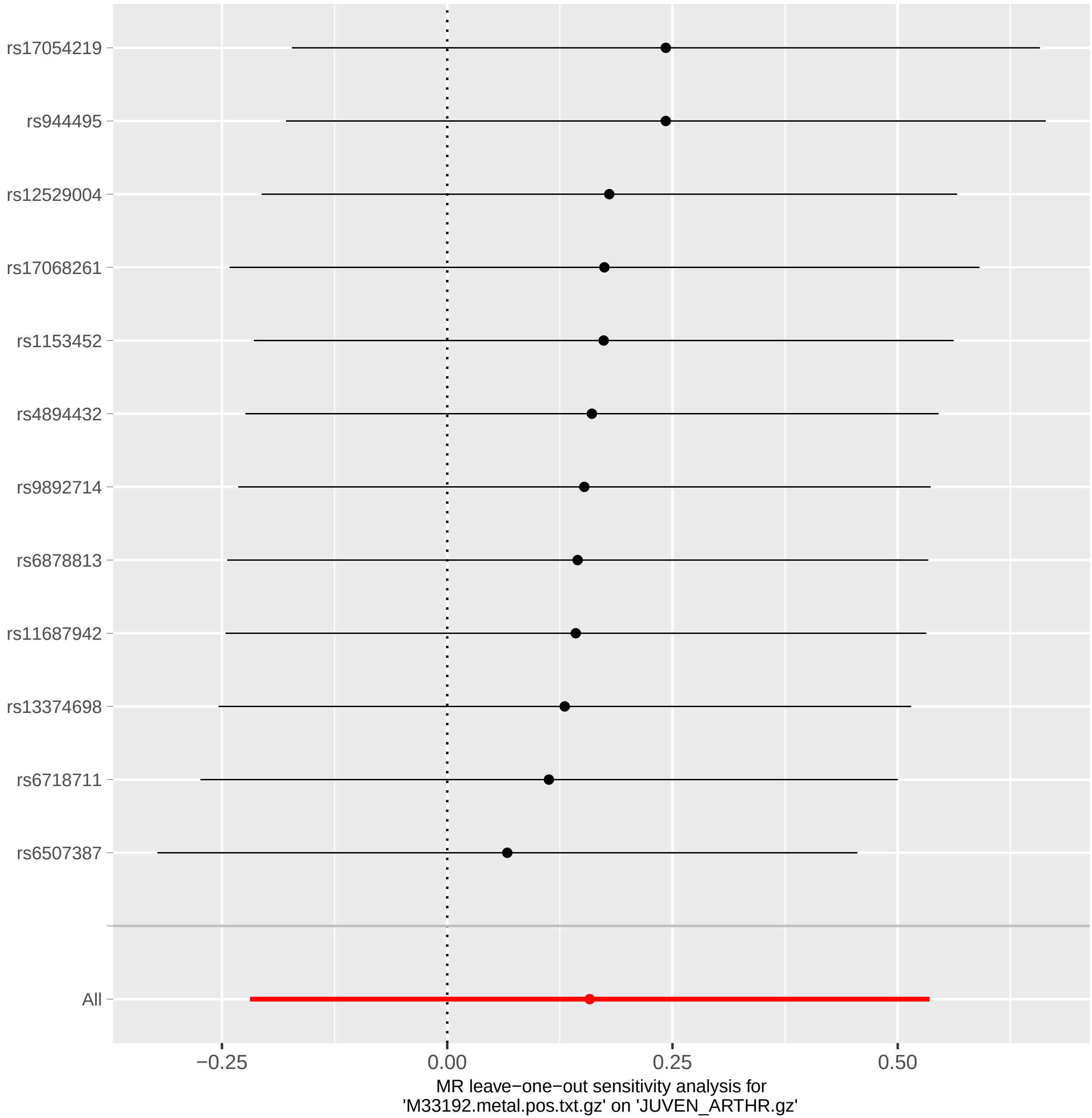

rs17054219
rs944495
rs12529004
rs17068261
rs1153452
rs4894432
rs9892714
rs6878813
rs11687942
rs13374698
rs6718711
rs6507387
All
−0.25
0.00
0.25
0.50
MR leave−one−out sensitivity analysis for
'M33192.metal.pos.txt.gz' on 'JUVEN_ARTHR.gz'

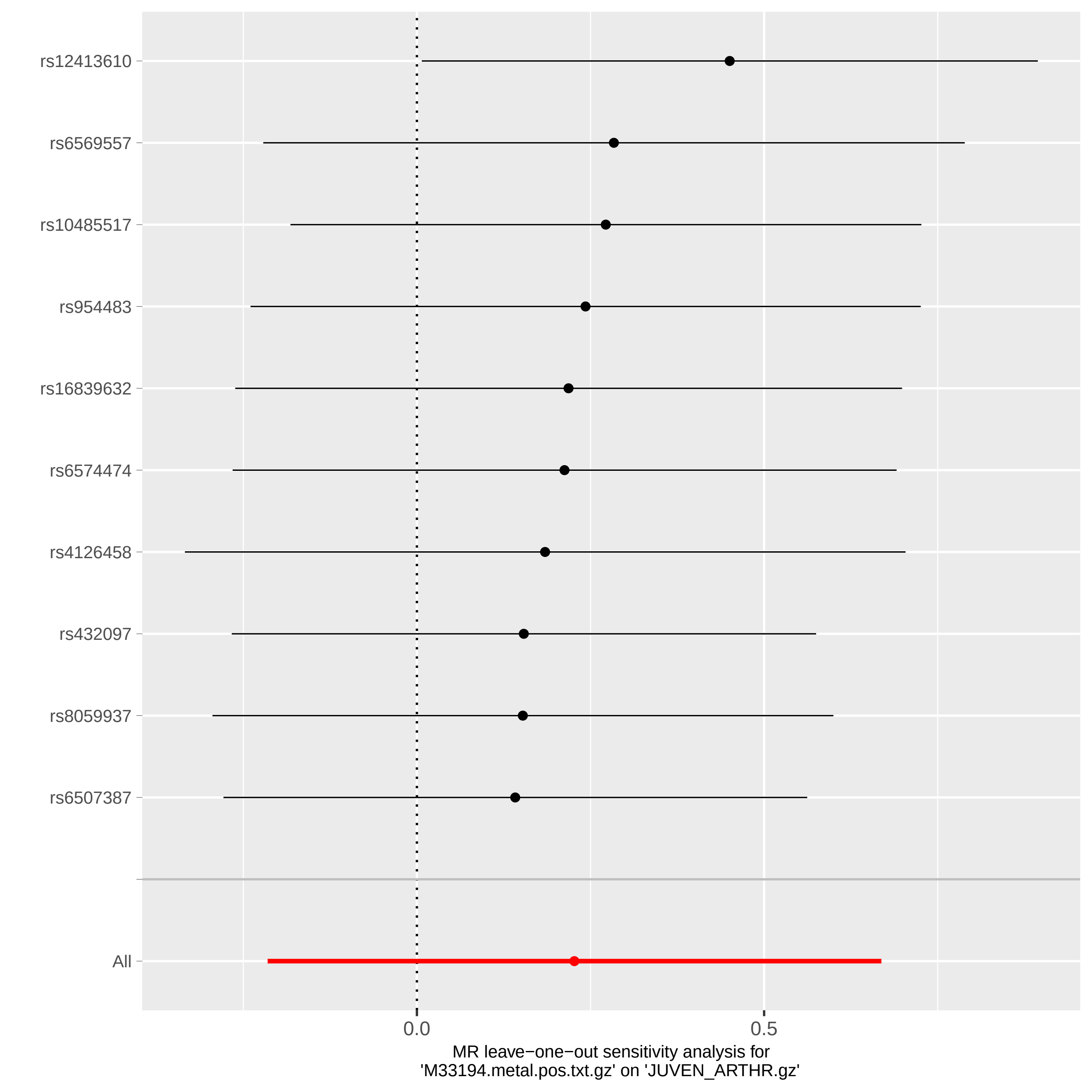

rs12413610
rs6569557
rs10485517
rs954483
rs16839632
rs6574474
rs4126458
rs432097
rs8059937
rs6507387
All
0.0
0.5
MR leave−one−out sensitivity analysis for
'M33194.metal.pos.txt.gz' on 'JUVEN_ARTHR.gz'

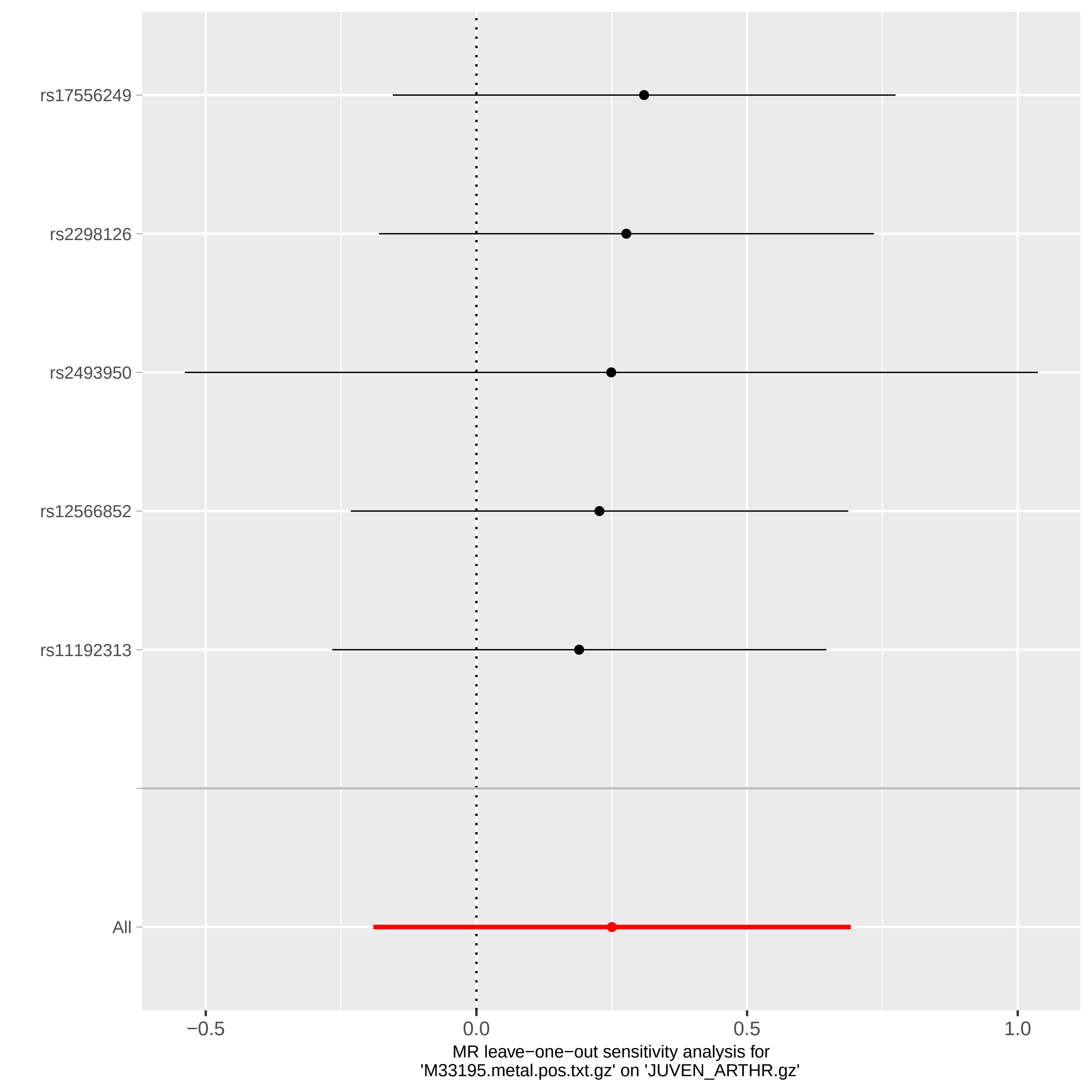

rs17556249
rs2298126
rs2493950
rs12566852
rs11192313
All
−0.5
0.0
0.5
1.0
MR leave−one−out sensitivity analysis for
'M33195.metal.pos.txt.gz' on 'JUVEN_ARTHR.gz'

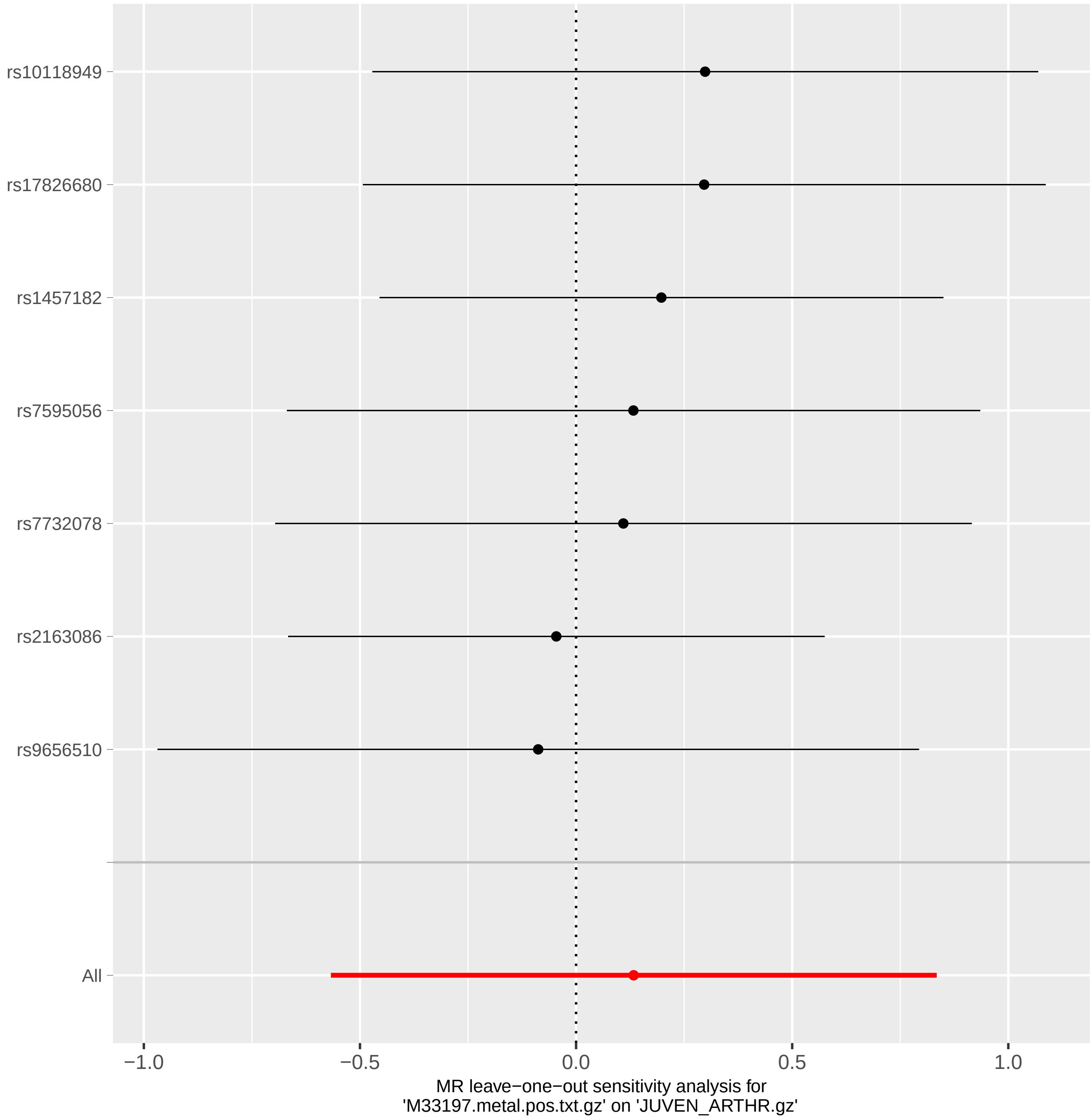
rs10118949
rs17826680
rs1457182
rs7595056
rs7732078
rs2163086
rs9656510
All
−1.0
−0.5
0.0
0.5
1.0
MR leave−one−out sensitivity analysis for
'M33197.metal.pos.txt.gz' on 'JUVEN_ARTHR.gz'

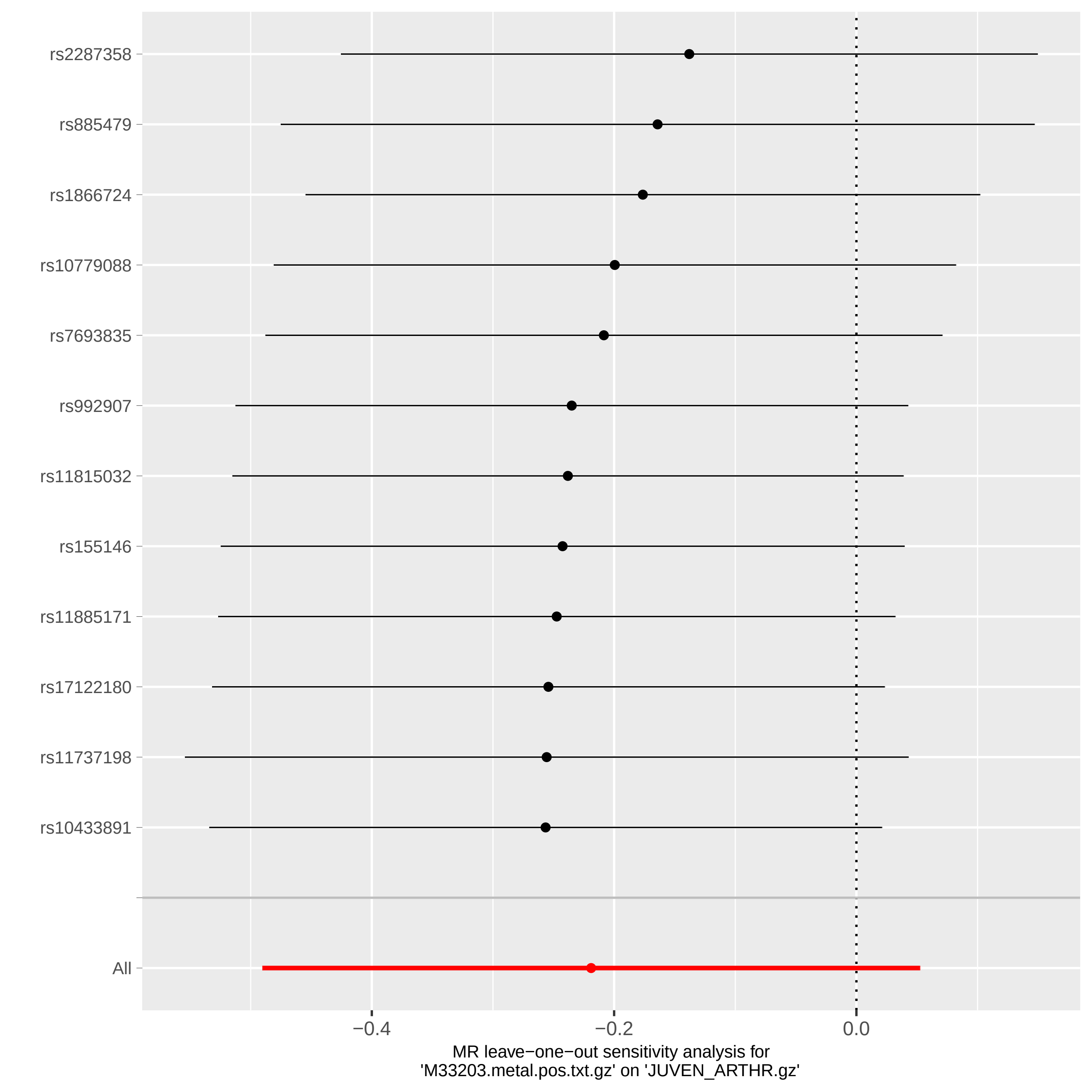
rs2287358
rs885479
rs1866724
rs10779088
rs7693835
rs992907
rs11815032
rs155146
rs11885171
rs17122180
rs11737198
rs10433891
All
−0.4
−0.2
0.0
MR leave−one−out sensitivity analysis for
'M33203.metal.pos.txt.gz' on 'JUVEN_ARTHR.gz'

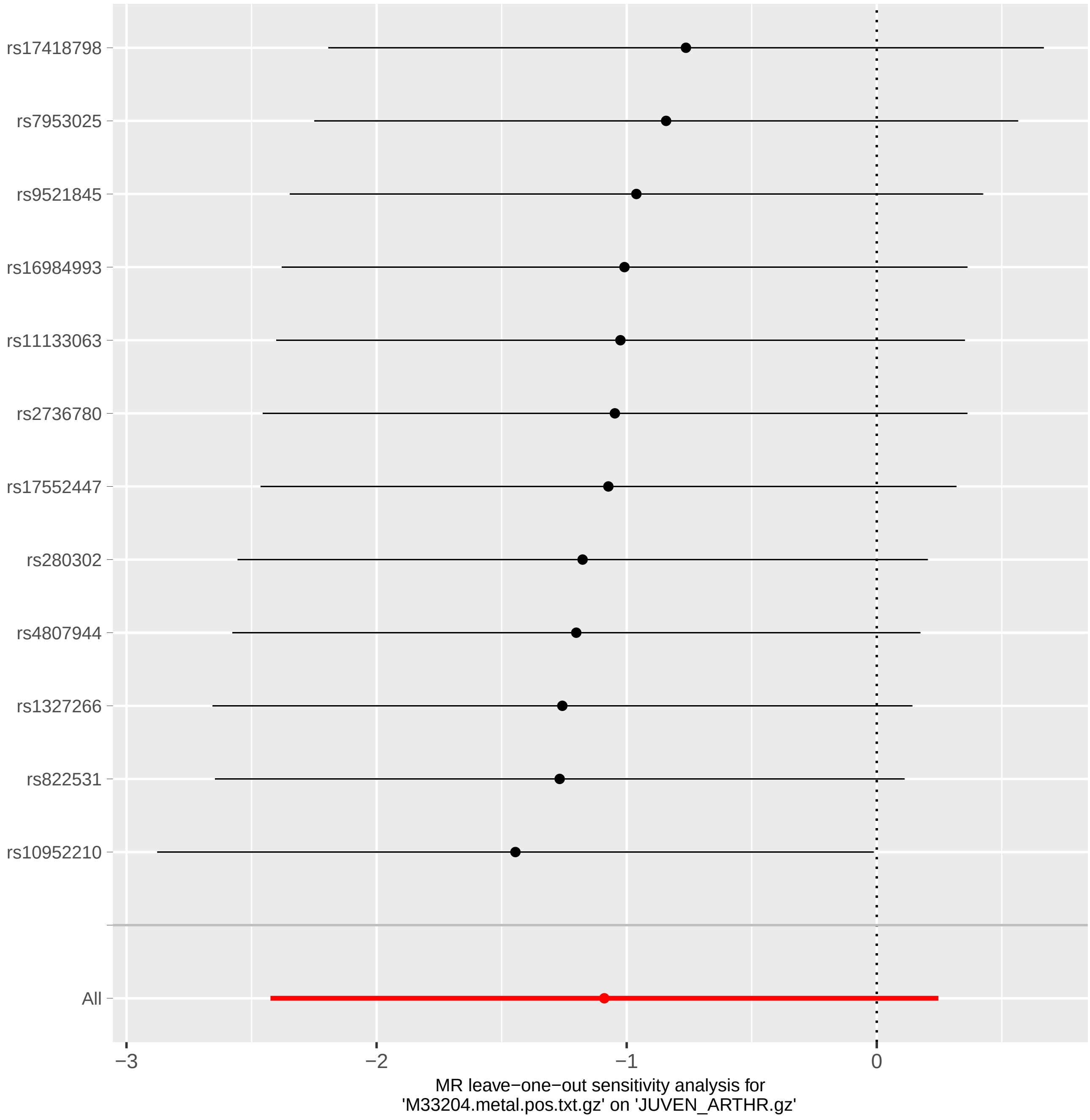
rs17418798
rs7953025
rs9521845
rs16984993
rs11133063
rs2736780
rs17552447
rs280302
rs4807944
rs1327266
rs822531
rs10952210
All
−3
−2
−1
0
MR leave−one−out sensitivity analysis for
'M33204.metal.pos.txt.gz' on 'JUVEN_ARTHR.gz'

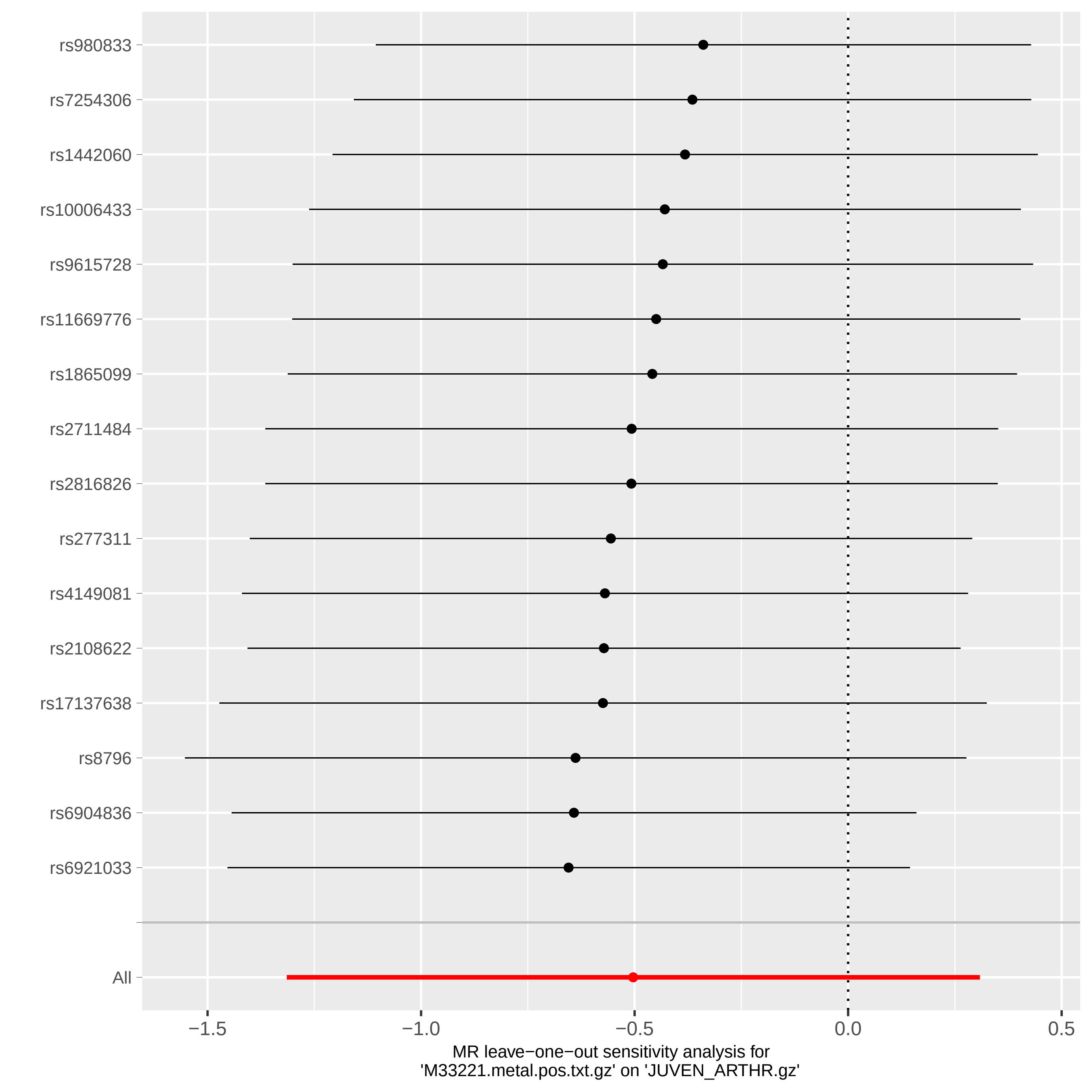

rs980833
rs7254306
rs1442060
rs10006433
rs9615728
rs11669776
rs1865099
rs2711484
rs2816826
rs277311
rs4149081
rs2108622
rs17137638
rs8796
rs6904836
rs6921033
All
−1.5
−1.0
−0.5
0.0
0.5
MR leave−one−out sensitivity analysis for
'M33221.metal.pos.txt.gz' on 'JUVEN_ARTHR.gz'

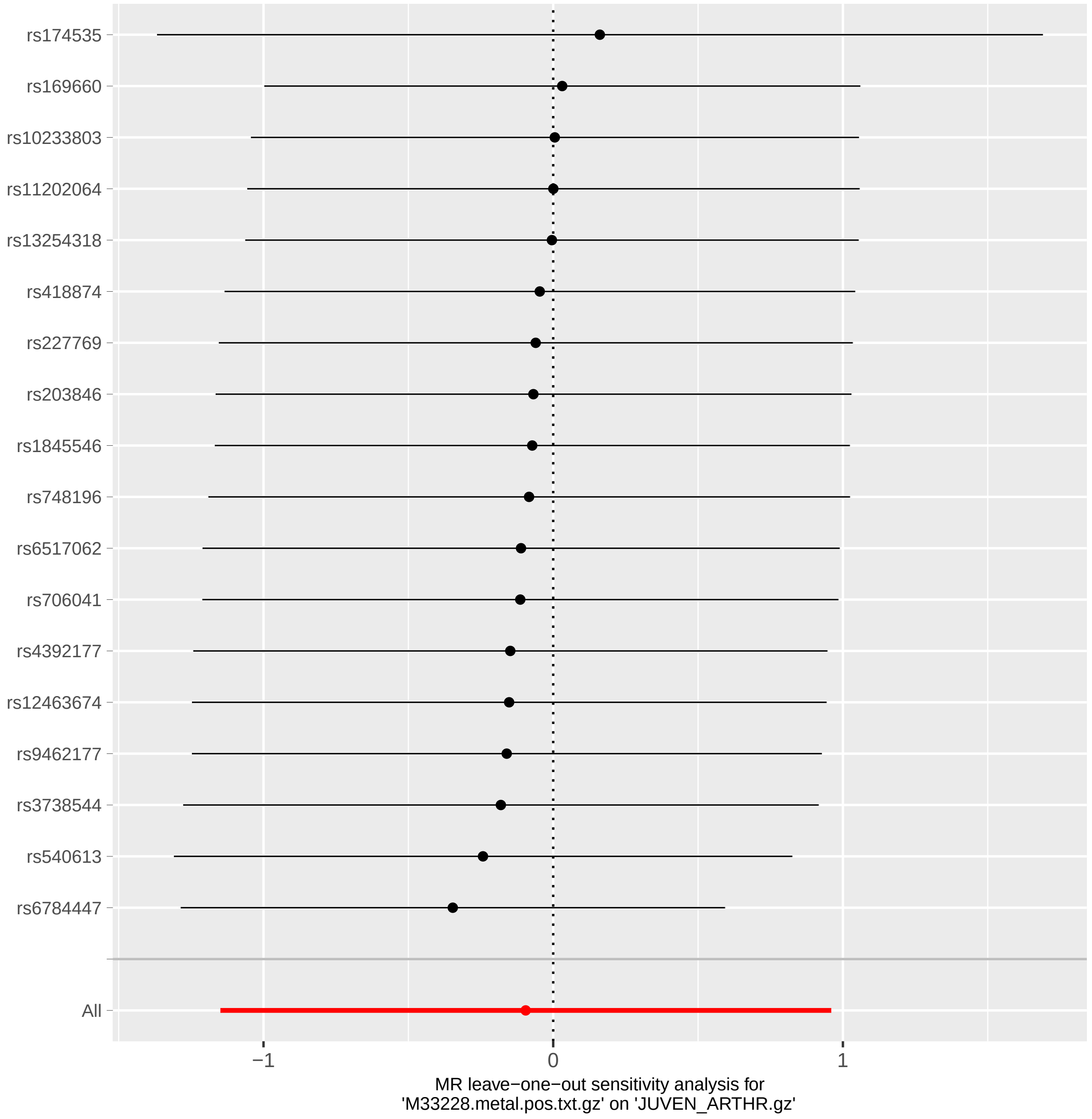
rs174535
rs169660
rs10233803
rs11202064
rs13254318
rs418874
rs227769
rs203846
rs1845546
rs748196
rs6517062
rs706041
rs4392177
rs12463674
rs9462177
rs3738544
rs540613
rs6784447
All
−1
0
1
MR leave−one−out sensitivity analysis for
'M33228.metal.pos.txt.gz' on 'JUVEN_ARTHR.gz'

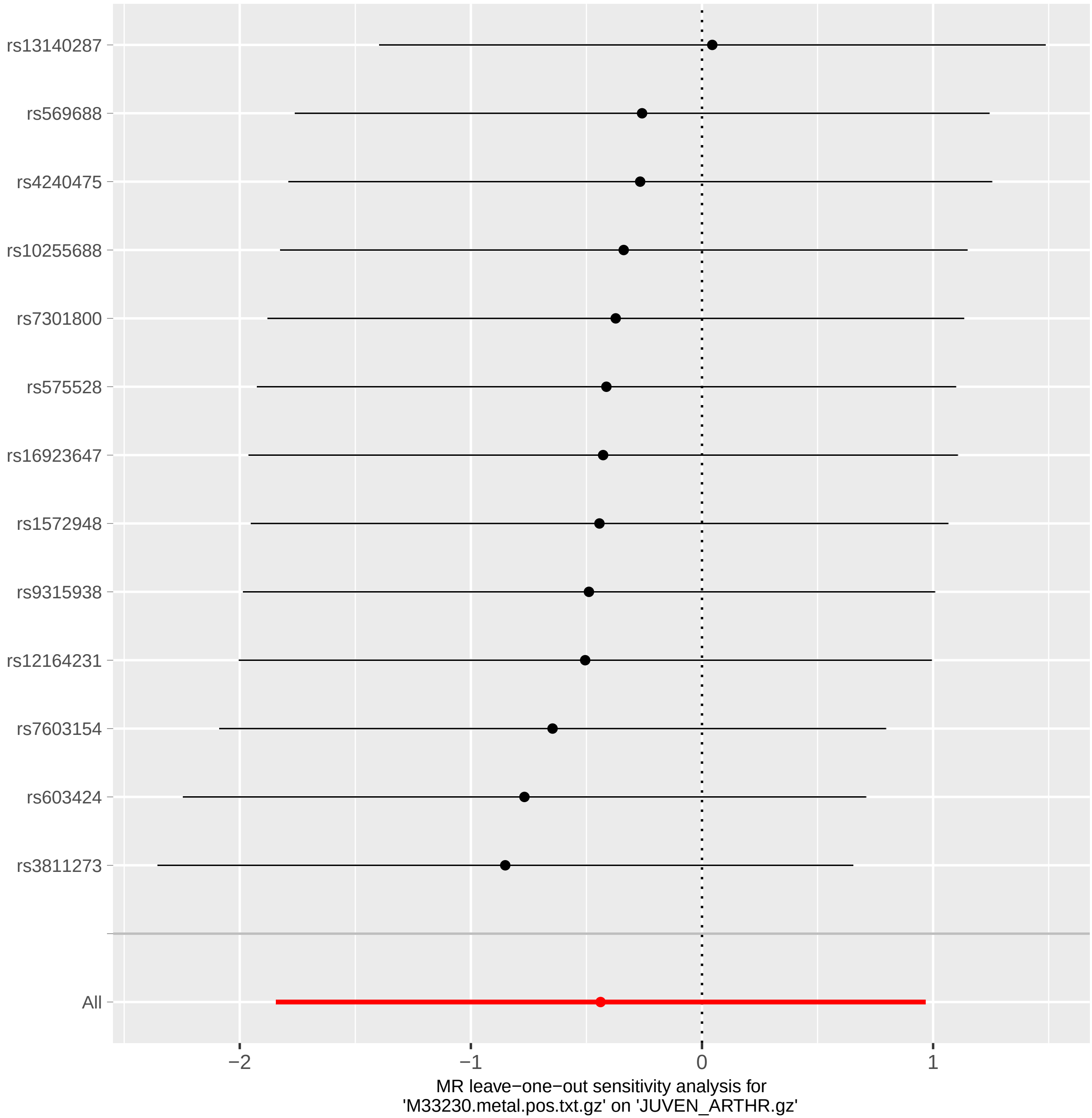

rs13140287
rs569688
rs4240475
rs10255688
rs7301800
rs575528
rs16923647
rs1572948
rs9315938
rs12164231
rs7603154
rs603424
rs3811273
All
−2
−1
0
1
MR leave−one−out sensitivity analysis for
'M33230.metal.pos.txt.gz' on 'JUVEN_ARTHR.gz'

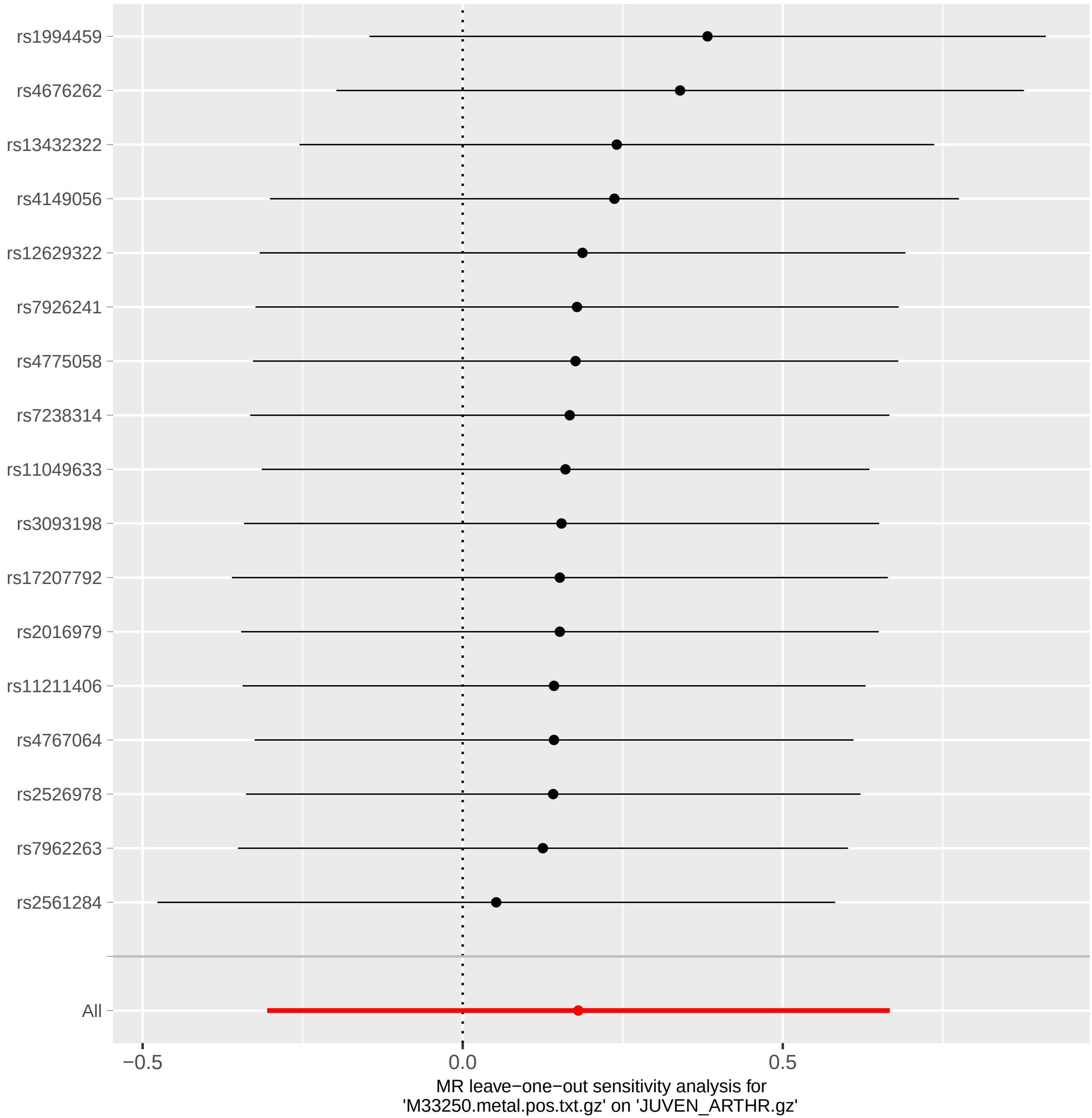
rs1994459
rs4676262
rs13432322
rs4149056
rs12629322
rs7926241
rs4775058
rs7238314
rs11049633
rs3093198
rs17207792
rs2016979
rs11211406
rs4767064
rs2526978
rs7962263
rs2561284
All
−0.5
0.0
0.5
MR leave−one−out sensitivity analysis for
'M33250.metal.pos.txt.gz' on 'JUVEN_ARTHR.gz'

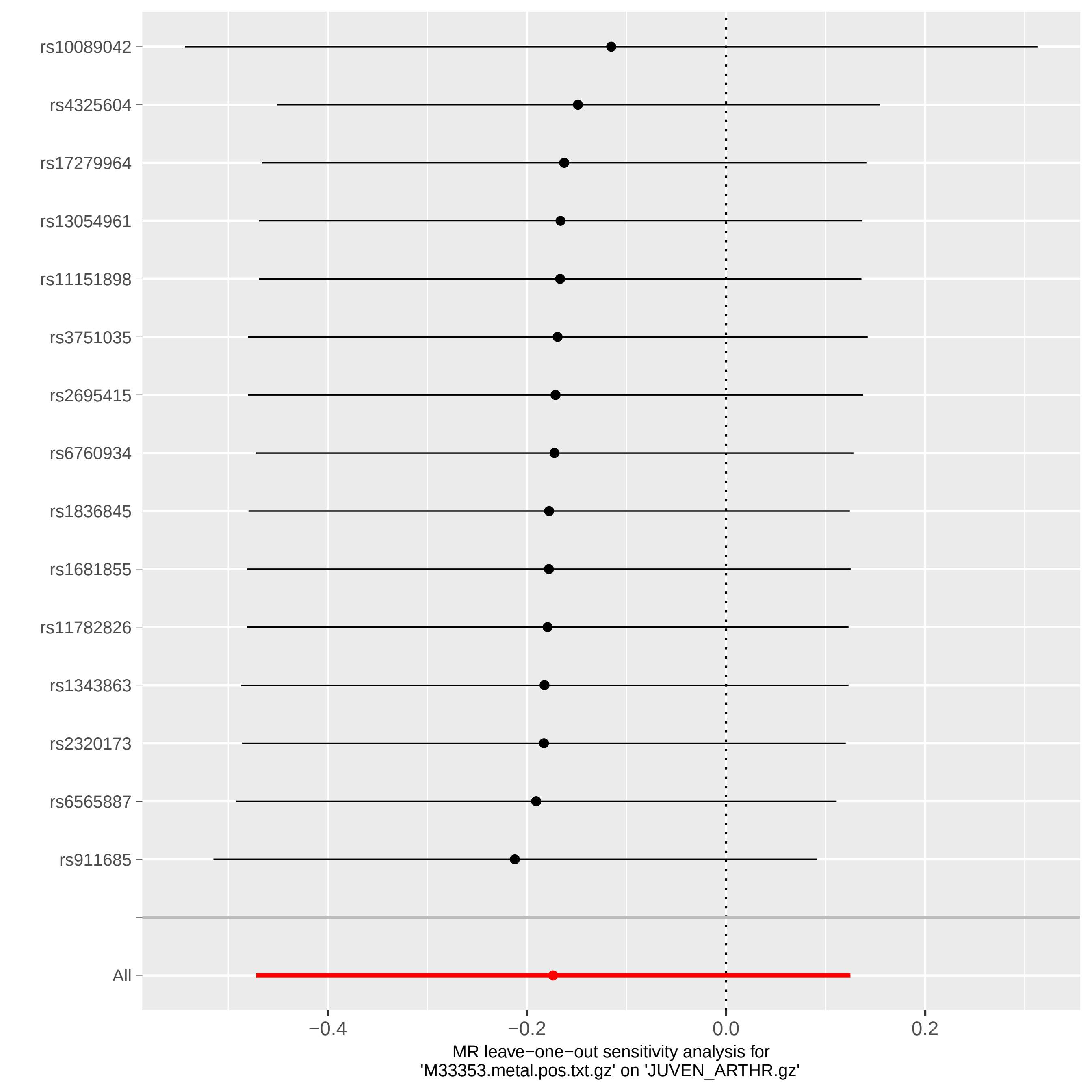
rs10089042
rs4325604
rs17279964
rs13054961
rs11151898
rs3751035
rs2695415
rs6760934
rs1836845
rs1681855
rs11782826
rs1343863
rs2320173
rs6565887
rs911685
All
−0.4
−0.2
0.0
0.2
MR leave−one−out sensitivity analysis for
'M33353.metal.pos.txt.gz' on 'JUVEN_ARTHR.gz'

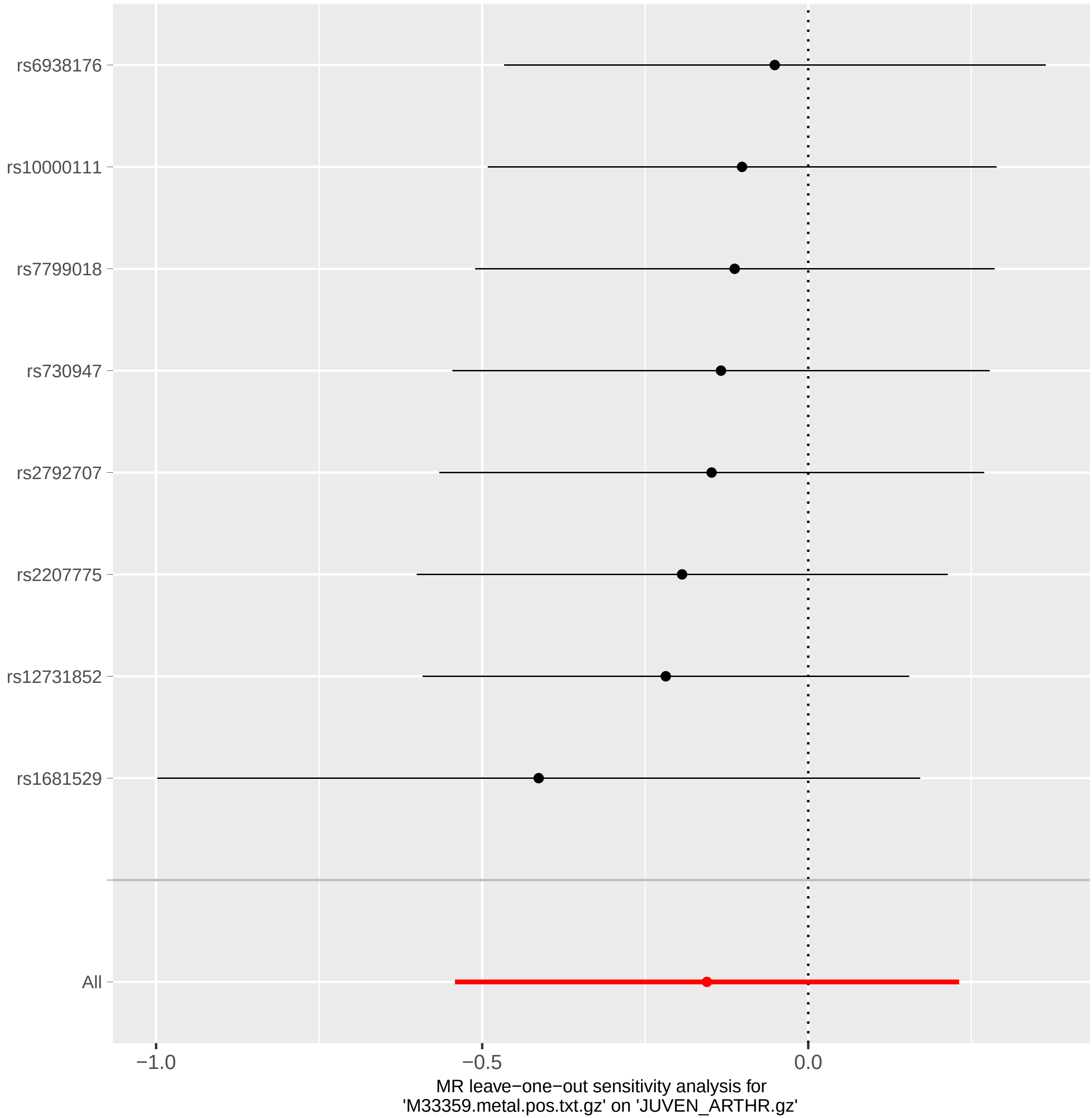
rs6938176
rs10000111
rs7799018
rs730947
rs2792707
rs2207775
rs12731852
rs1681529
All
−1.0
−0.5
0.0
MR leave−one−out sensitivity analysis for
'M33359.metal.pos.txt.gz' on 'JUVEN_ARTHR.gz'

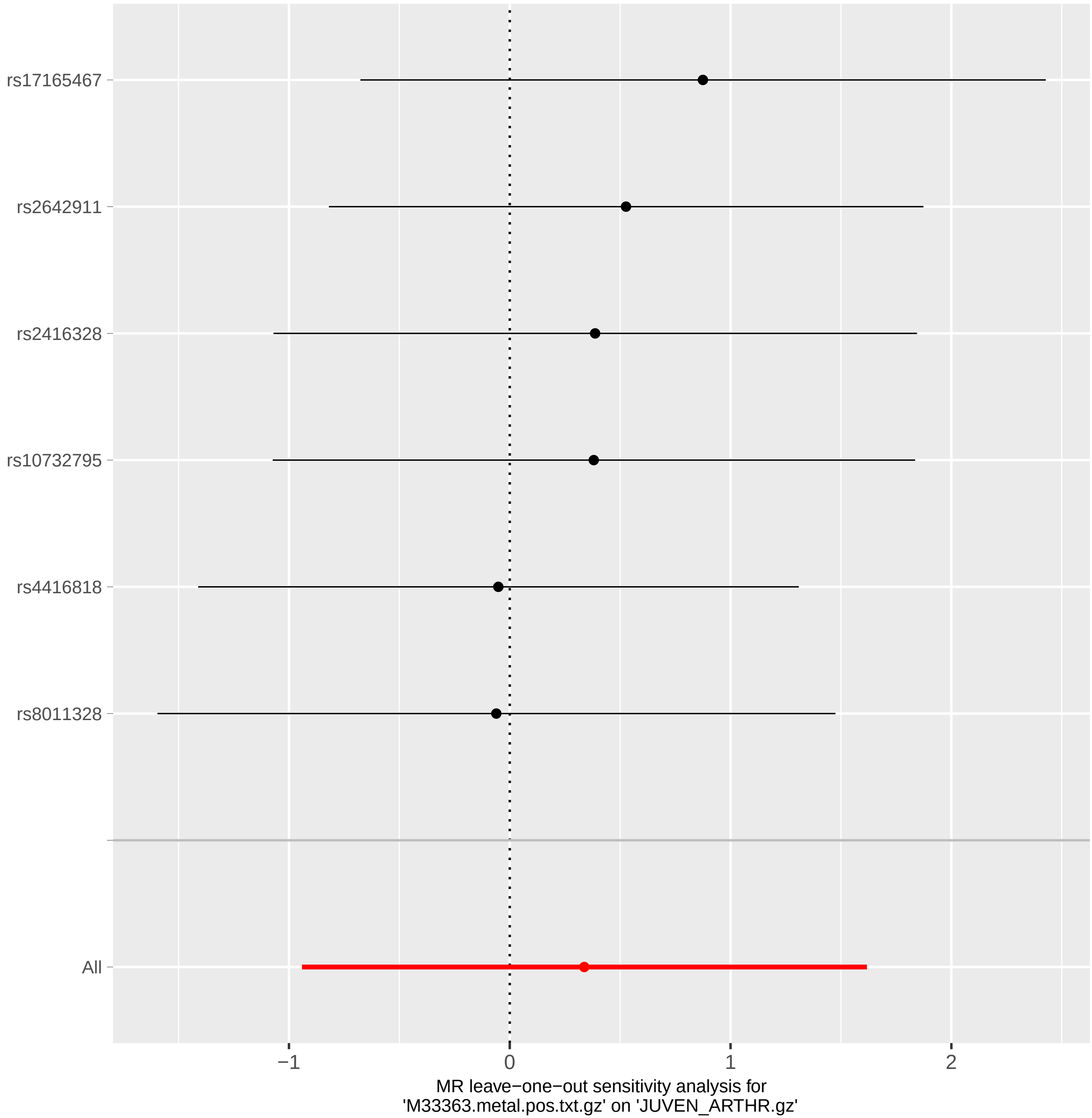
rs17165467
rs2642911
rs2416328
rs10732795
rs4416818
rs8011328
All
−1
0
1
2
MR leave−one−out sensitivity analysis for
'M33363.metal.pos.txt.gz' on 'JUVEN_ARTHR.gz'

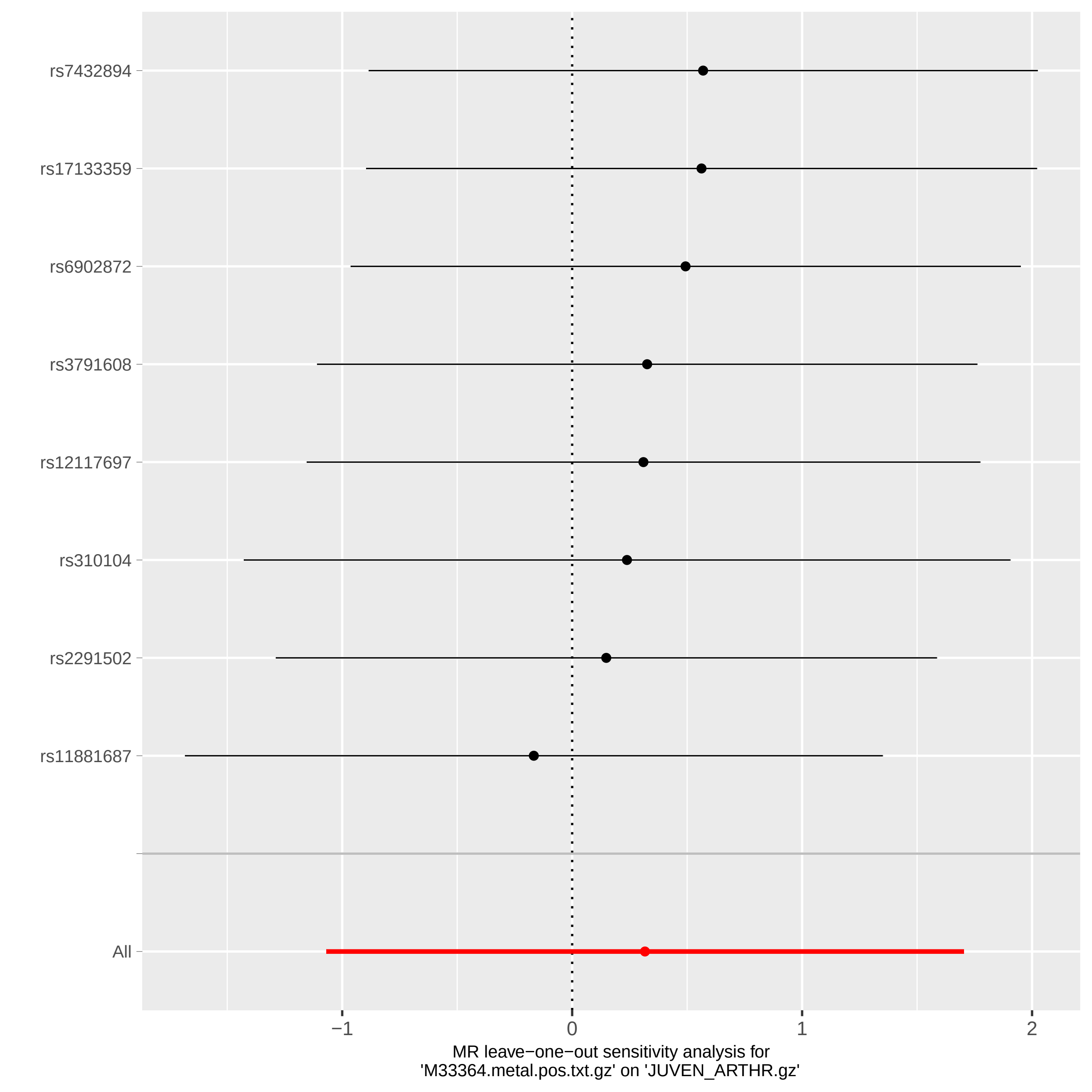
rs7432894
rs17133359
rs6902872
rs3791608
rs12117697
rs310104
rs2291502
rs11881687
All
−1
0
1
2
MR leave−one−out sensitivity analysis for
'M33364.metal.pos.txt.gz' on 'JUVEN_ARTHR.gz'

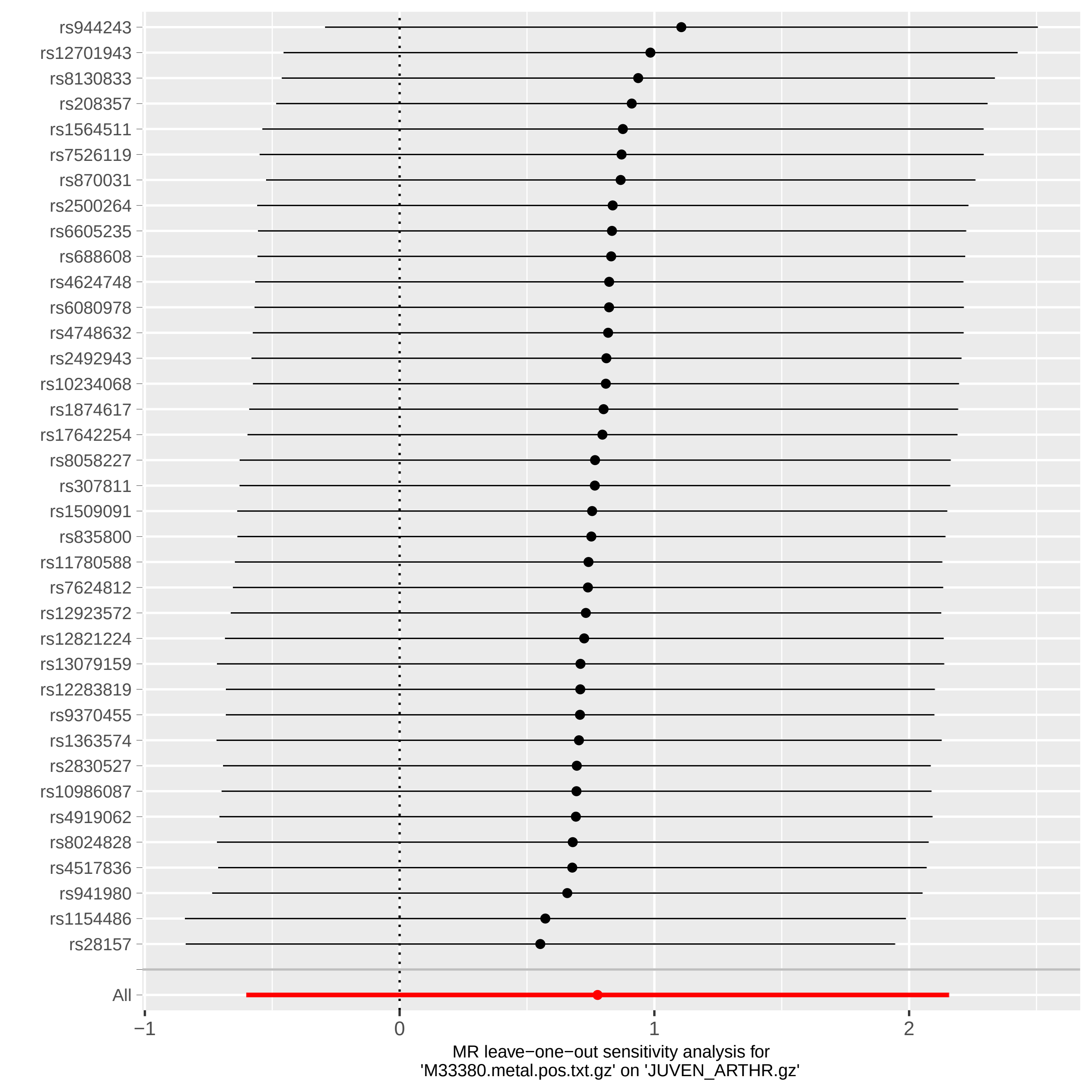
rs944243
rs12701943
rs8130833
rs208357
rs1564511
rs7526119
rs870031
rs2500264
rs6605235
rs688608
rs4624748
rs6080978
rs4748632
rs2492943
rs10234068
rs1874617
rs17642254
rs8058227
rs307811
rs1509091
rs835800
rs11780588
rs7624812
rs12923572
rs12821224
rs13079159
rs12283819
rs9370455
rs1363574
rs2830527
rs10986087
rs4919062
rs8024828
rs4517836
rs941980
rs1154486
rs28157
All
−1
0
1
2
MR leave−one−out sensitivity analysis for
'M33380.metal.pos.txt.gz' on 'JUVEN_ARTHR.gz'

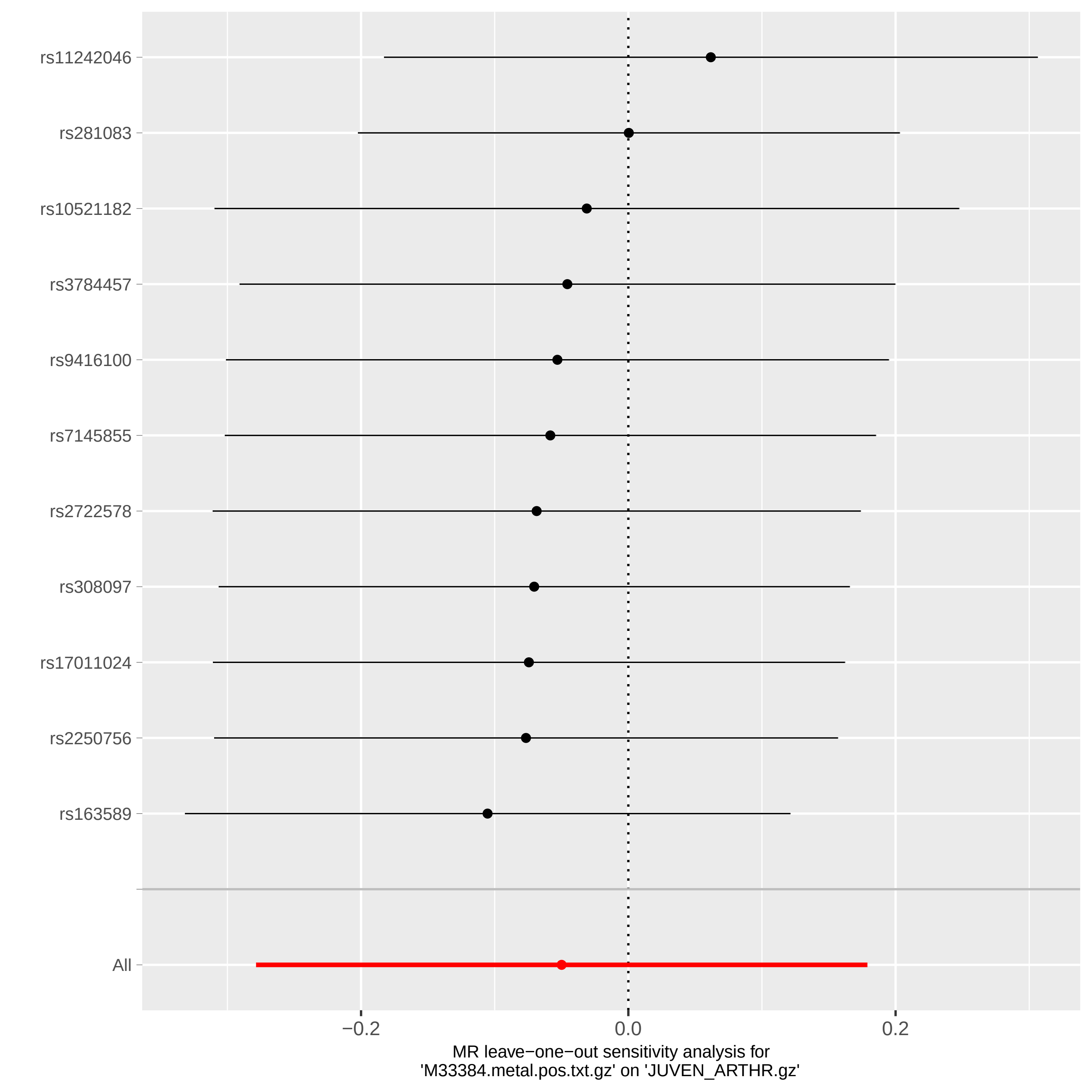

rs11242046
rs281083
rs10521182
rs3784457
rs9416100
rs7145855
rs2722578
rs308097
rs17011024
rs2250756
rs163589
All
−0.2
0.0
0.2
MR leave−one−out sensitivity analysis for
'M33384.metal.pos.txt.gz' on 'JUVEN_ARTHR.gz'

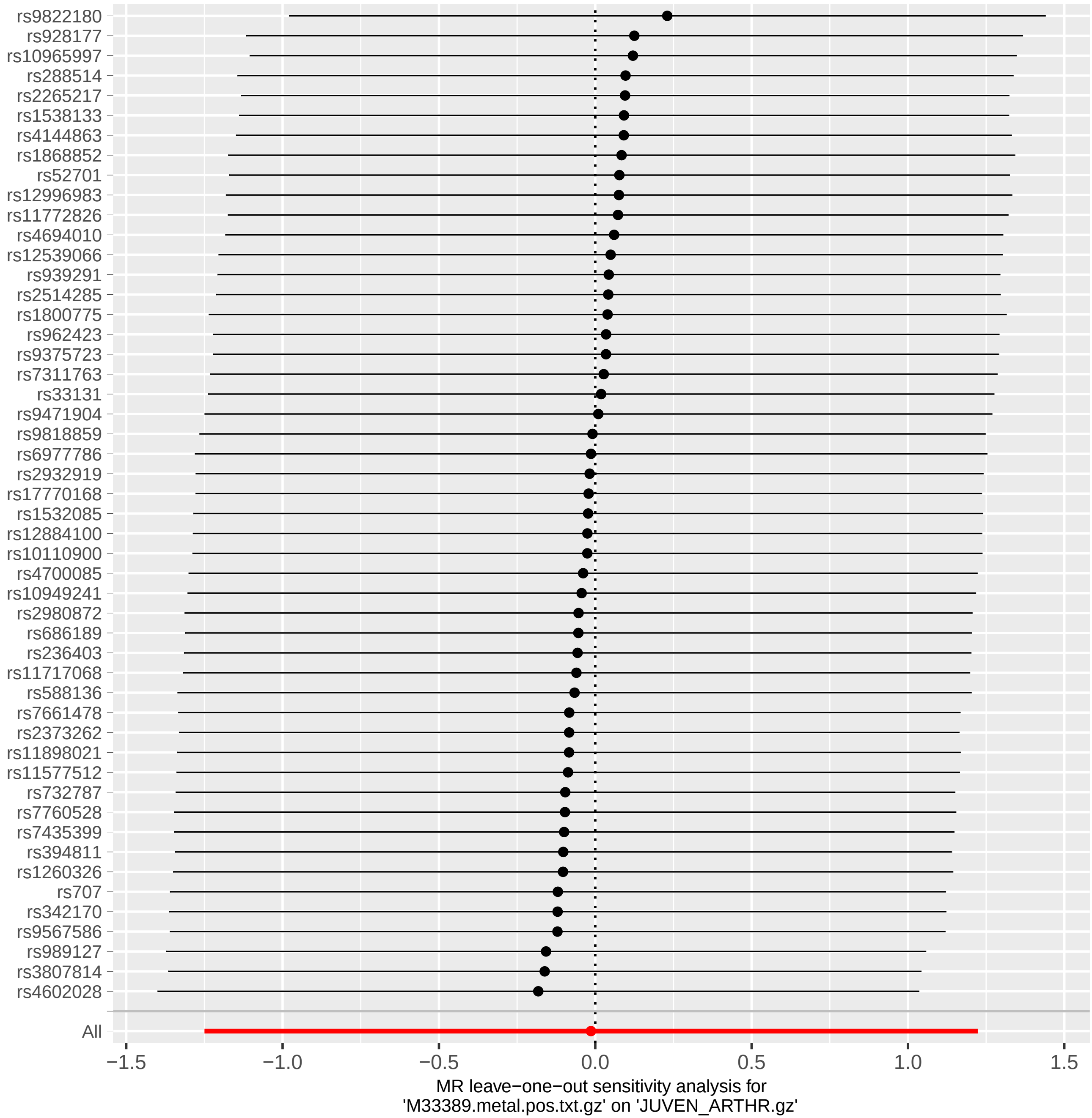
rs9822180
rs928177
rs10965997
rs288514
rs2265217
rs1538133
rs4144863
rs1868852
rs52701
rs12996983
rs11772826
rs4694010
rs12539066
rs939291
rs2514285
rs1800775
rs962423
rs9375723
rs7311763
rs33131
rs9471904
rs9818859
rs6977786
rs2932919
rs17770168
rs1532085
rs12884100
rs10110900
rs4700085
rs10949241
rs2980872
rs686189
rs236403
rs11717068
rs588136
rs7661478
rs2373262
rs11898021
rs11577512
rs732787
rs7760528
rs7435399
rs394811
rs1260326
rs707
rs342170
rs9567586
rs989127
rs3807814
rs4602028
All
−1.5
−1.0
−0.5
0.0
0.5
1.0
1.5
MR leave−one−out sensitivity analysis for
'M33389.metal.pos.txt.gz' on 'JUVEN_ARTHR.gz'

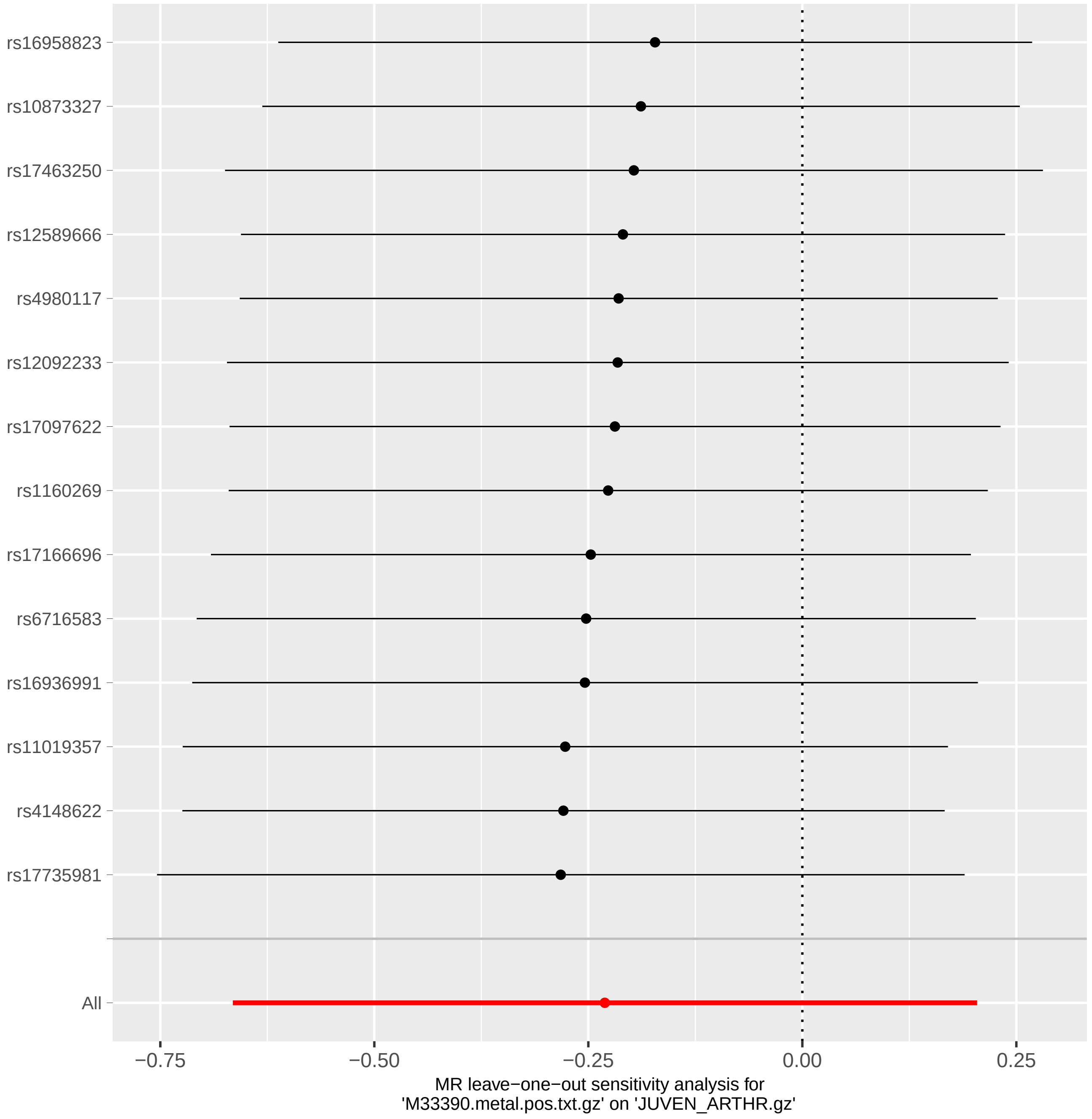
rs16958823
rs10873327
rs17463250
rs12589666
rs4980117
rs12092233
rs17097622
rs1160269
rs17166696
rs6716583
rs16936991
rs11019357
rs4148622
rs17735981
All
−0.75
−0.50
−0.25
0.00
0.25
MR leave−one−out sensitivity analysis for
'M33390.metal.pos.txt.gz' on 'JUVEN_ARTHR.gz'

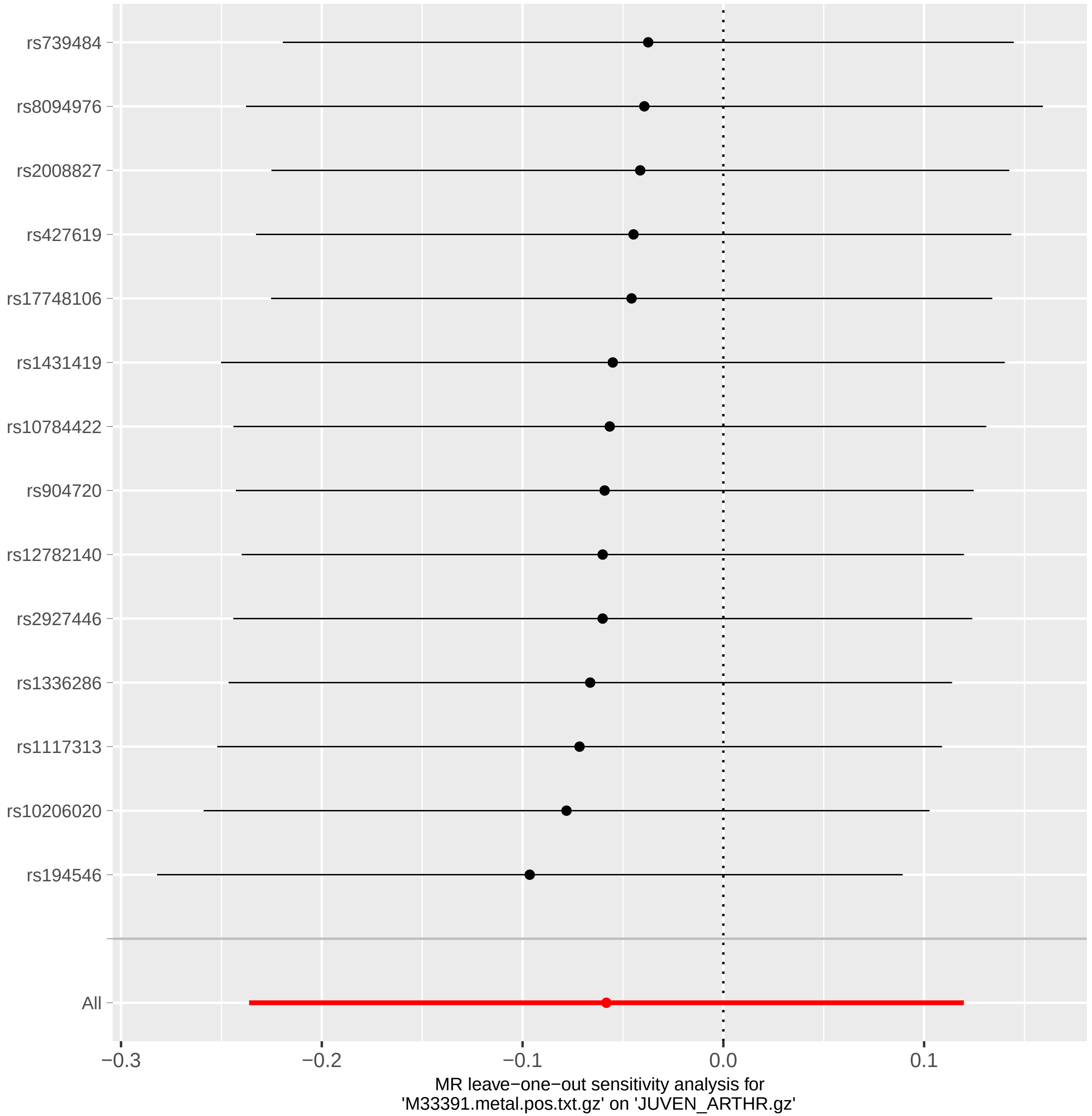
rs739484
rs8094976
rs2008827
rs427619
rs17748106
rs1431419
rs10784422
rs904720
rs12782140
rs2927446
rs1336286
rs1117313
rs10206020
rs194546
All
−0.3
−0.2
−0.1
0.0
0.1
MR leave−one−out sensitivity analysis for
'M33391.metal.pos.txt.gz' on 'JUVEN_ARTHR.gz'

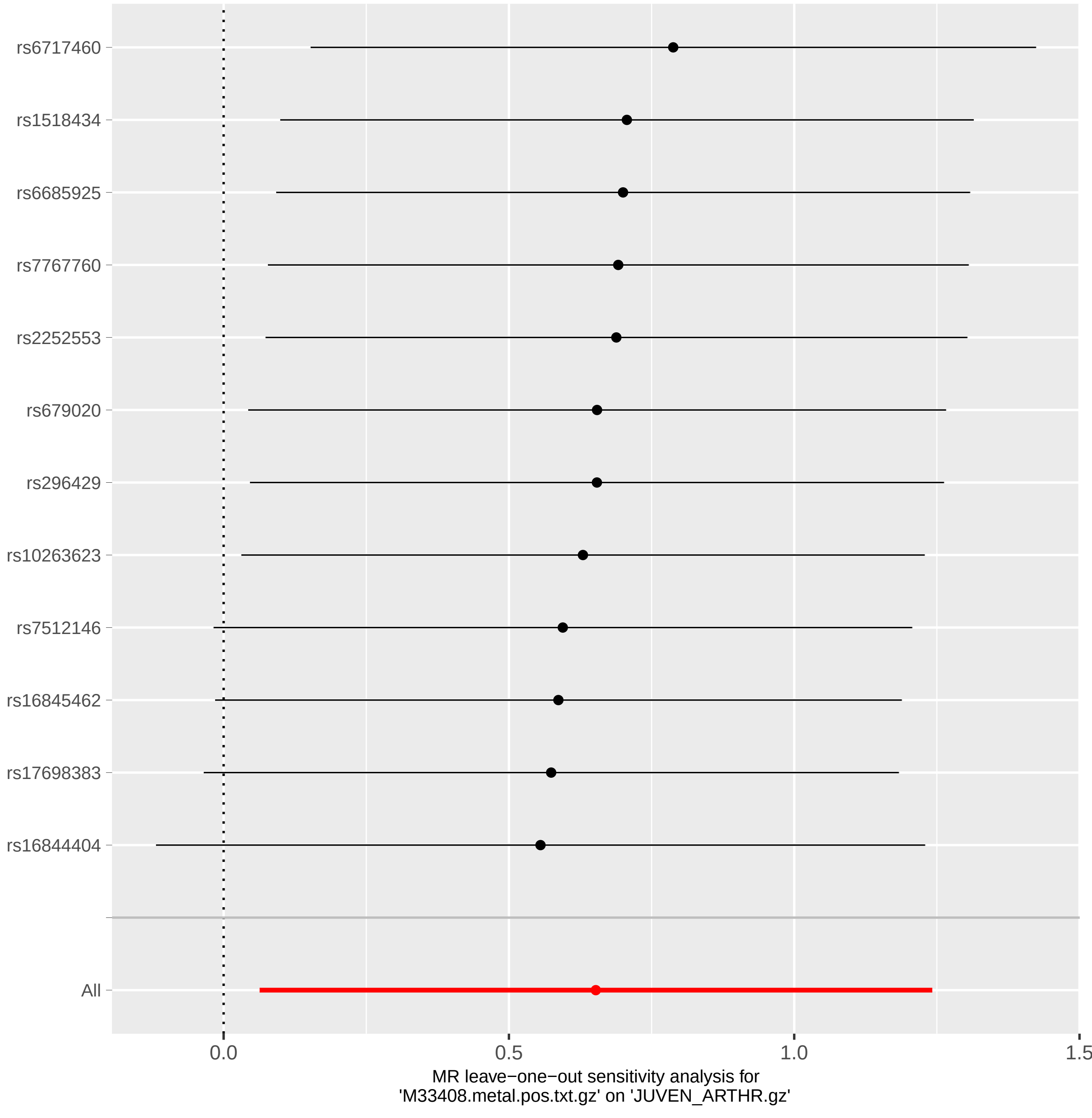
rs6717460
rs1518434
rs6685925
rs7767760
rs2252553
rs679020
rs296429
rs10263623
rs7512146
rs16845462
rs17698383
rs16844404
All
0.0
0.5
1.0
1.5
MR leave−one−out sensitivity analysis for
'M33408.metal.pos.txt.gz' on 'JUVEN_ARTHR.gz'

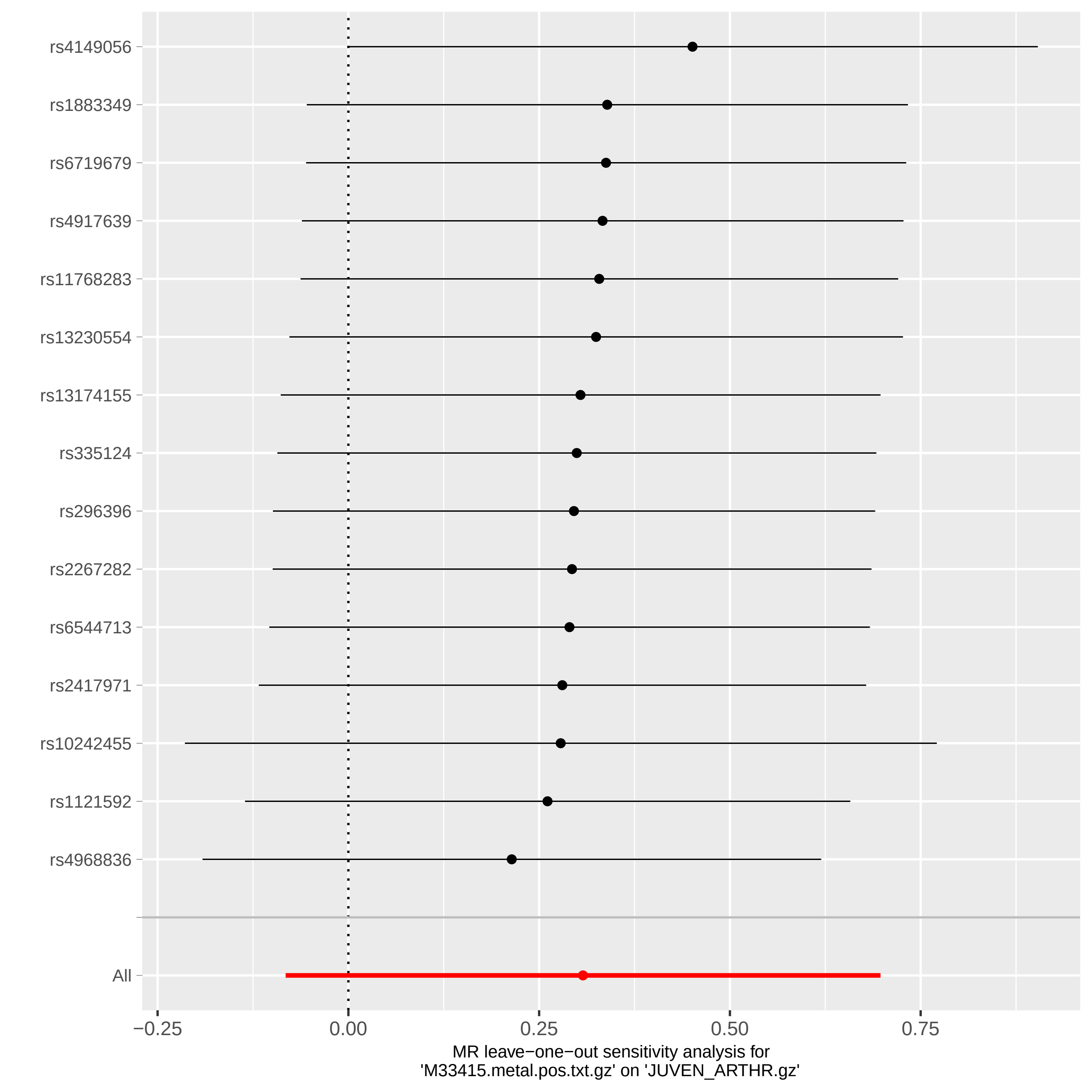
rs4149056
rs1883349
rs6719679
rs4917639
rs11768283
rs13230554
rs13174155
rs335124
rs296396
rs2267282
rs6544713
rs2417971
rs10242455
rs1121592
rs4968836
All
−0.25
0.00
0.25
0.50
0.75
MR leave−one−out sensitivity analysis for
'M33415.metal.pos.txt.gz' on 'JUVEN_ARTHR.gz'

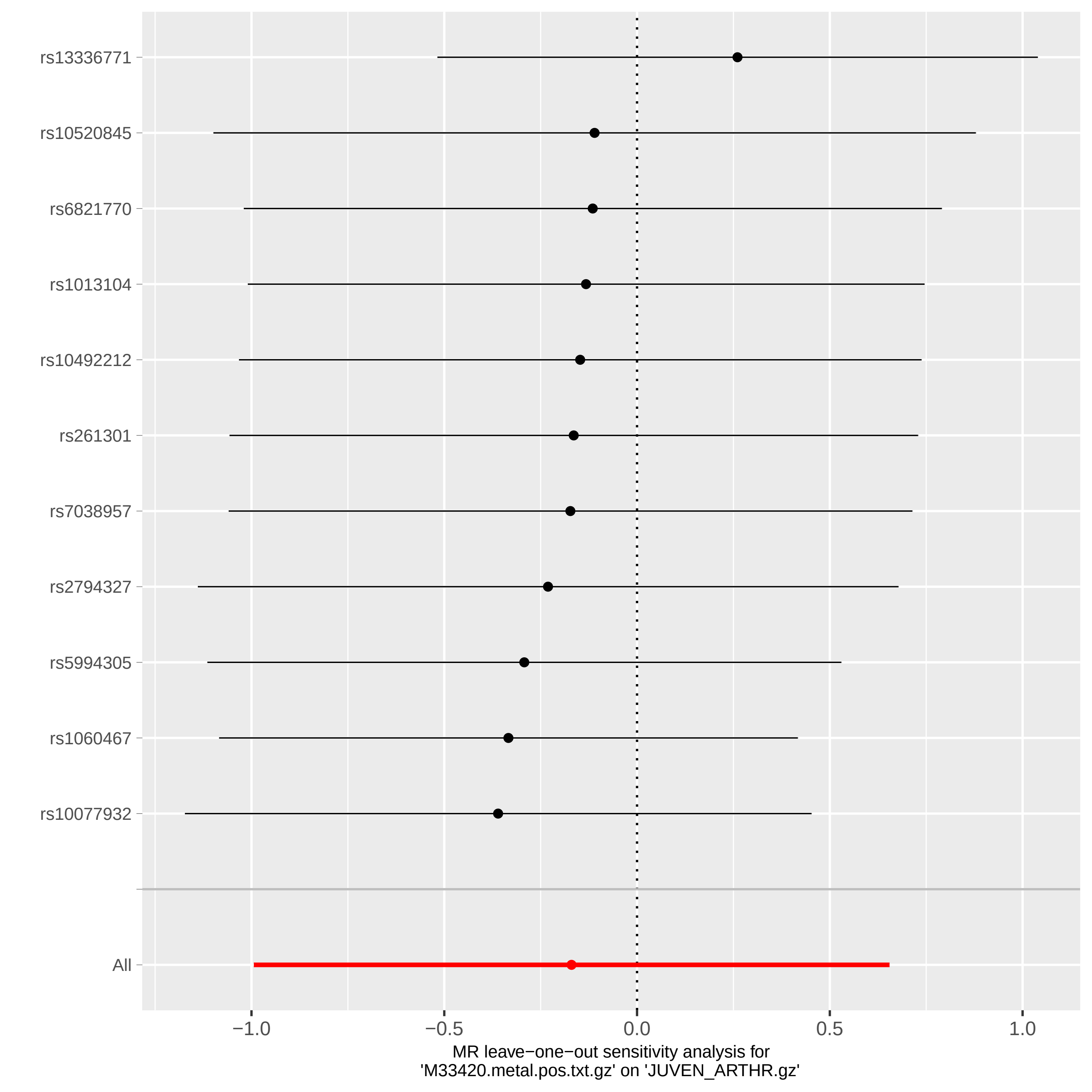
rs13336771
rs10520845
rs6821770
rs1013104
rs10492212
rs261301
rs7038957
rs2794327
rs5994305
rs1060467
rs10077932
All
−1.0
−0.5
0.0
0.5
1.0
MR leave−one−out sensitivity analysis for
'M33420.metal.pos.txt.gz' on 'JUVEN_ARTHR.gz'

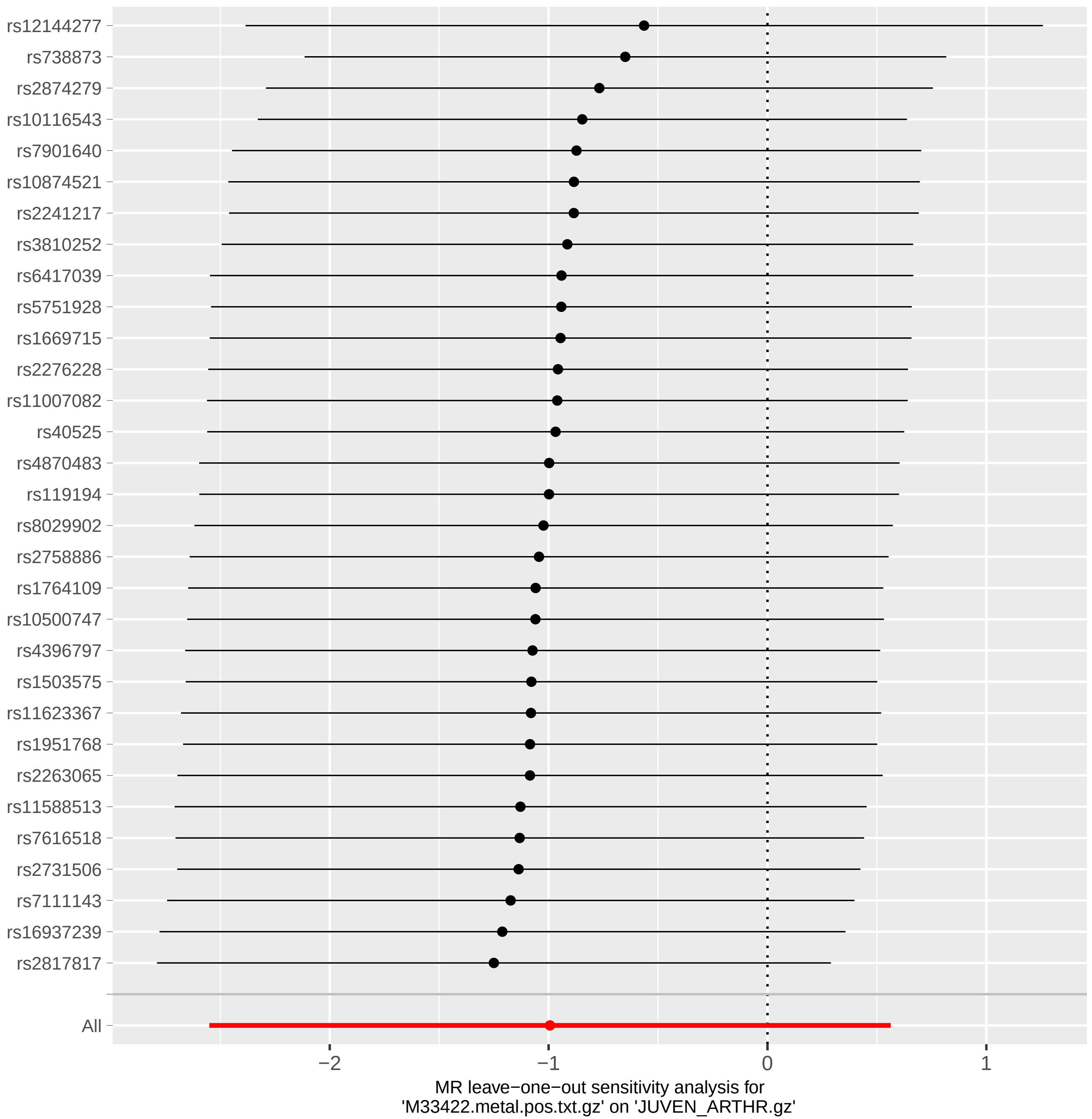

rs12144277
rs738873
rs2874279
rs10116543
rs7901640
rs10874521
rs2241217
rs3810252
rs6417039
rs5751928
rs1669715
rs2276228
rs11007082
rs40525
rs4870483
rs119194
rs8029902
rs2758886
rs1764109
rs10500747
rs4396797
rs1503575
rs11623367
rs1951768
rs2263065
rs11588513
rs7616518
rs2731506
rs7111143
rs16937239
rs2817817
All
−2
−1
0
1
MR leave−one−out sensitivity analysis for
'M33422.metal.pos.txt.gz' on 'JUVEN_ARTHR.gz'

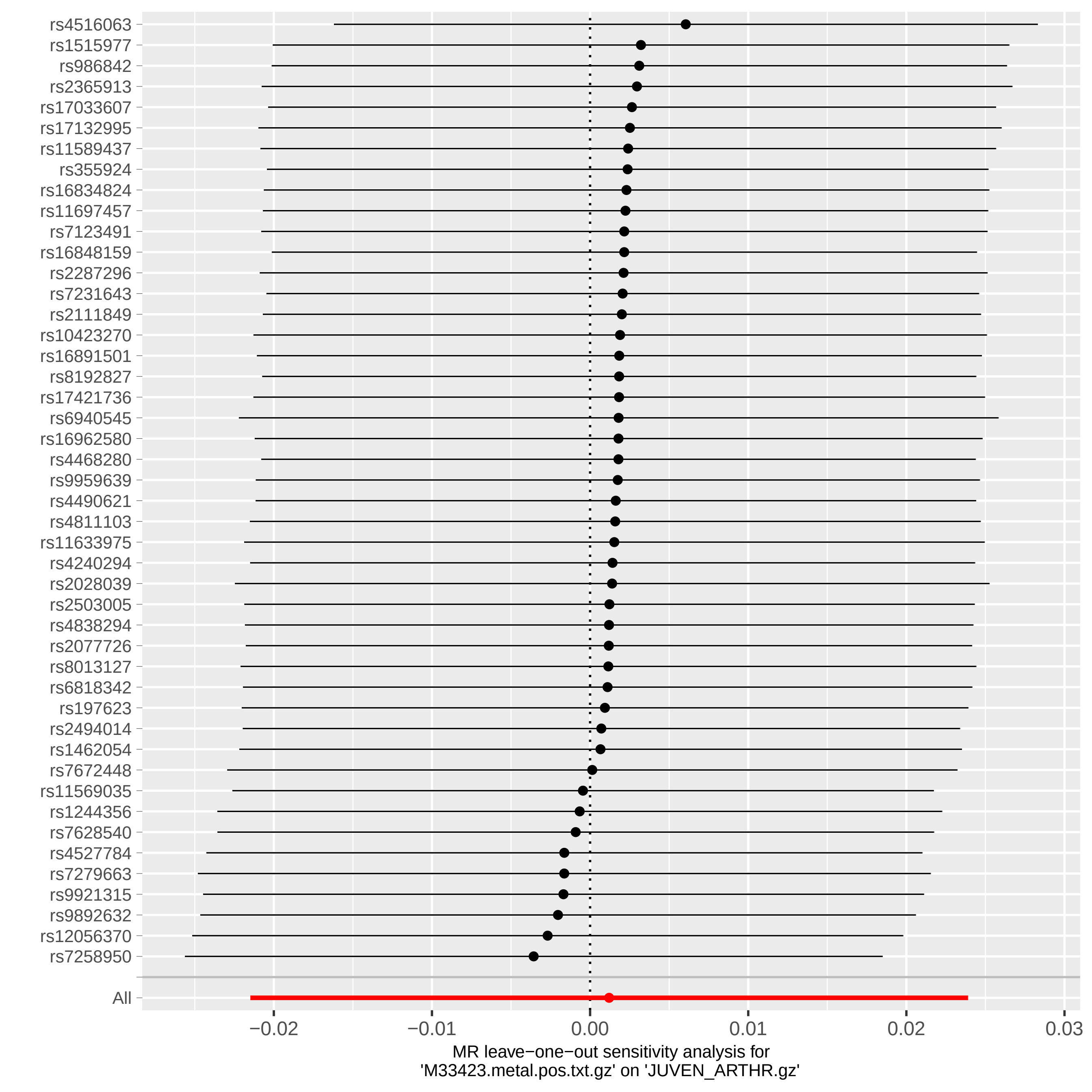
rs4516063
rs1515977
rs986842
rs2365913
rs17033607
rs17132995
rs11589437
rs355924
rs16834824
rs11697457
rs7123491
rs16848159
rs2287296
rs7231643
rs2111849
rs10423270
rs16891501
rs8192827
rs17421736
rs6940545
rs16962580
rs4468280
rs9959639
rs4490621
rs4811103
rs11633975
rs4240294
rs2028039
rs2503005
rs4838294
rs2077726
rs8013127
rs6818342
rs197623
rs2494014
rs1462054
rs7672448
rs11569035
rs1244356
rs7628540
rs4527784
rs7279663
rs9921315
rs9892632
rs12056370
rs7258950
All
−0.02
−0.01
0.00
0.01
0.02
0.03
MR leave−one−out sensitivity analysis for
'M33423.metal.pos.txt.gz' on 'JUVEN_ARTHR.gz'

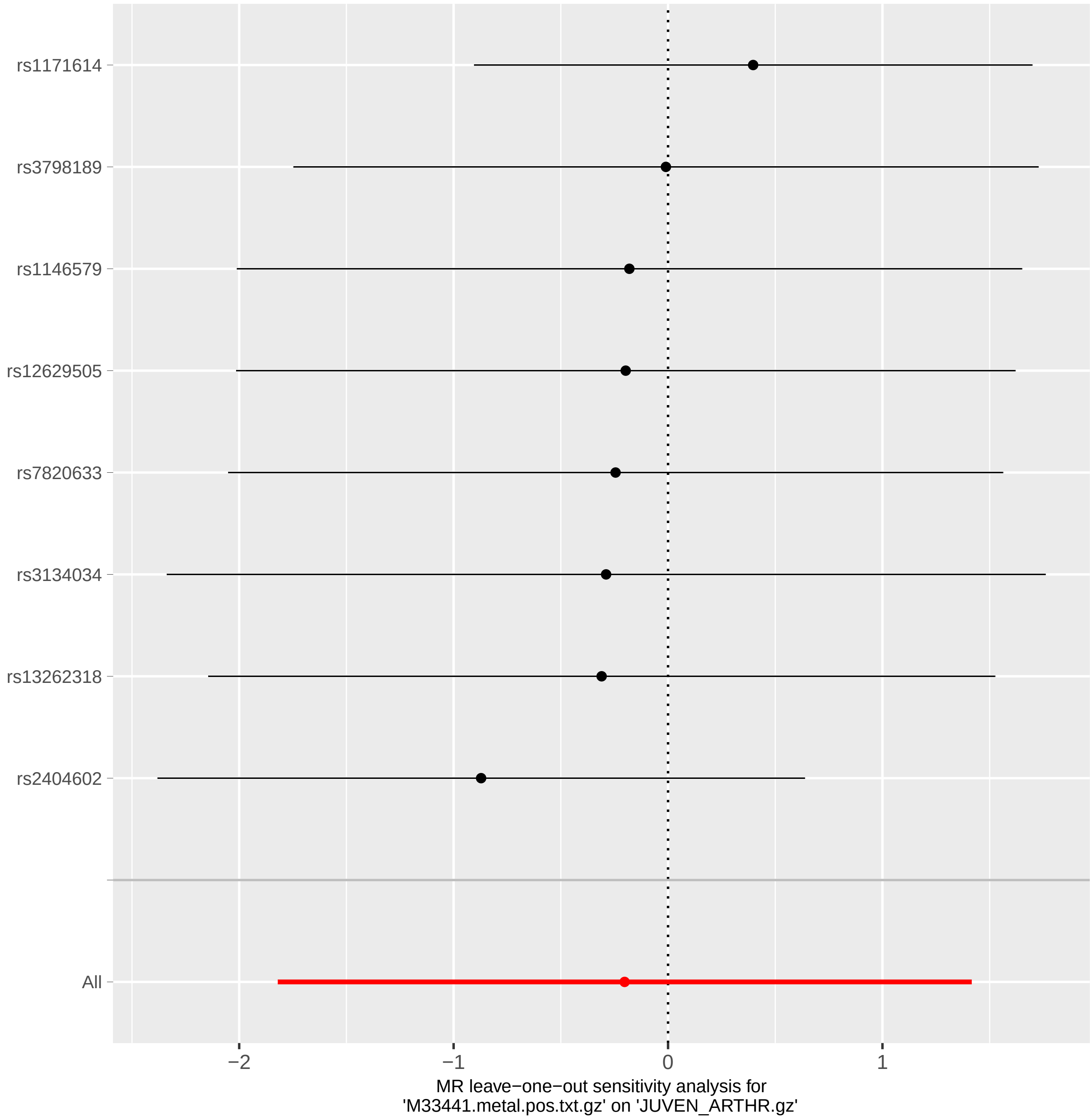
rs1171614
rs3798189
rs1146579
rs12629505
rs7820633
rs3134034
rs13262318
rs2404602
All
−2
−1
0
1
MR leave−one−out sensitivity analysis for
'M33441.metal.pos.txt.gz' on 'JUVEN_ARTHR.gz'

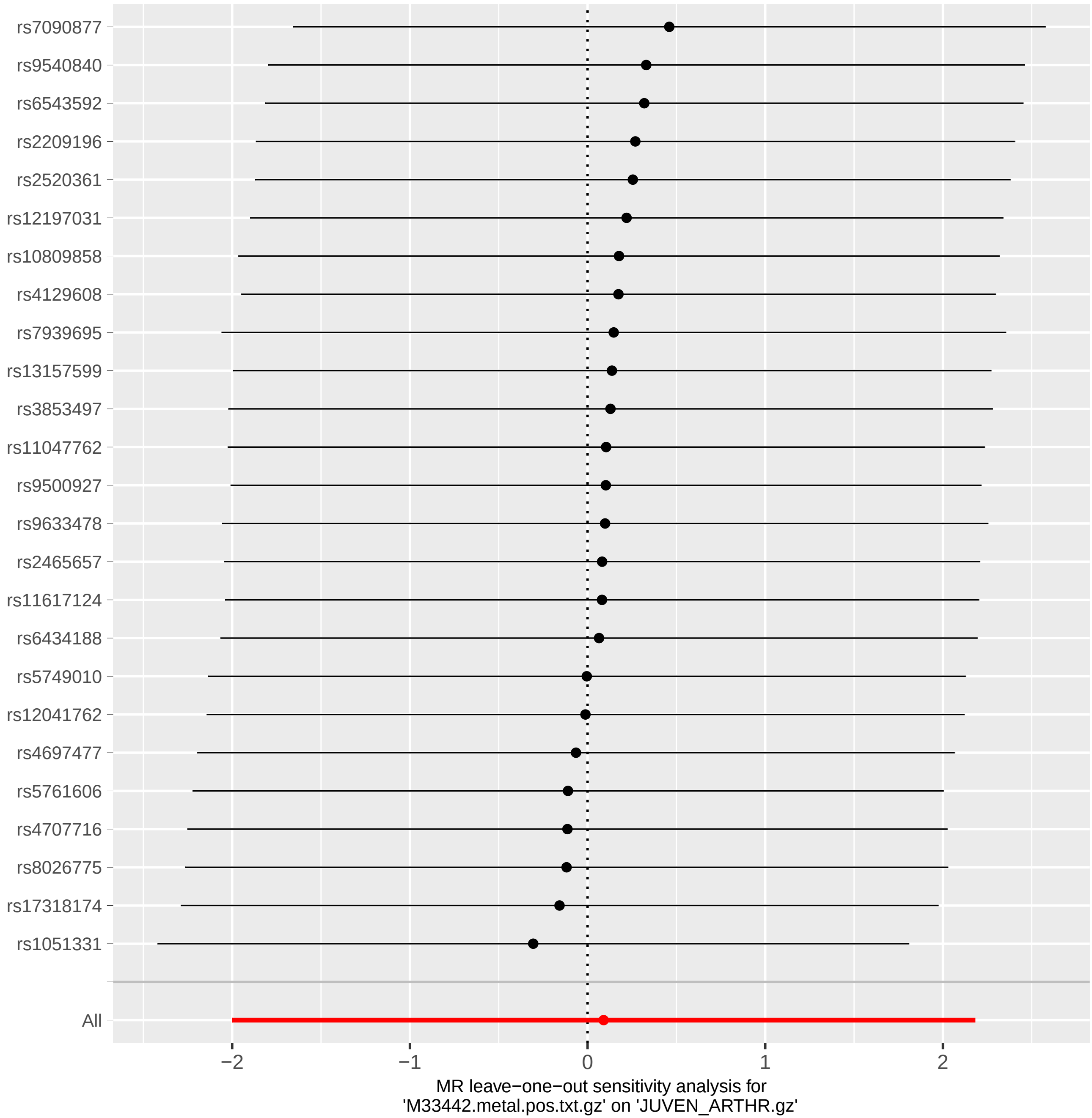
rs7090877
rs9540840
rs6543592
rs2209196
rs2520361
rs12197031
rs10809858
rs4129608
rs7939695
rs13157599
rs3853497
rs11047762
rs9500927
rs9633478
rs2465657
rs11617124
rs6434188
rs5749010
rs12041762
rs4697477
rs5761606
rs4707716
rs8026775
rs17318174
rs1051331
All
−2
−1
0
1
2
MR leave−one−out sensitivity analysis for
'M33442.metal.pos.txt.gz' on 'JUVEN_ARTHR.gz'

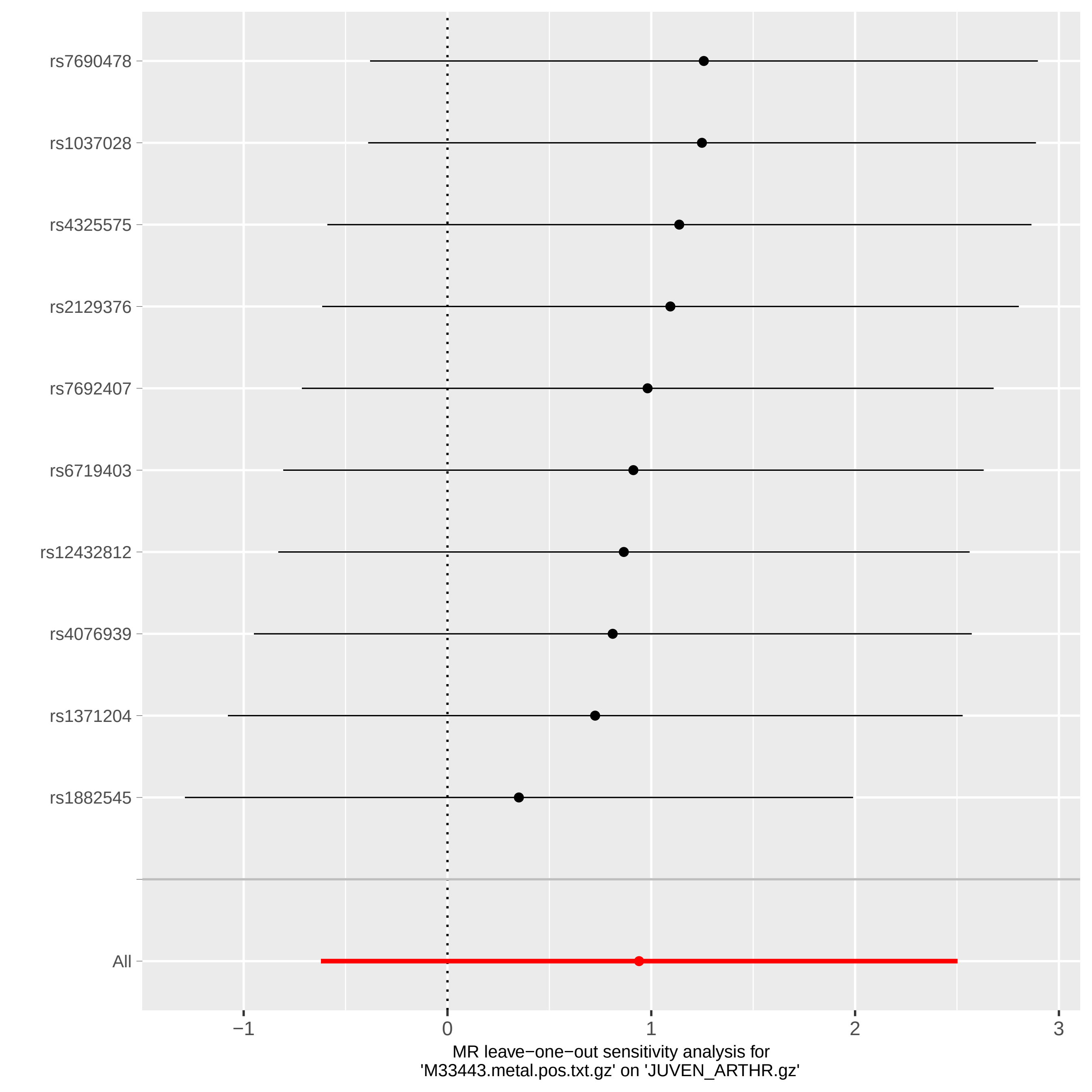
rs7690478
rs1037028
rs4325575
rs2129376
rs7692407
rs6719403
rs12432812
rs4076939
rs1371204
rs1882545
All
−1
0
1
2
3
MR leave−one−out sensitivity analysis for
'M33443.metal.pos.txt.gz' on 'JUVEN_ARTHR.gz'

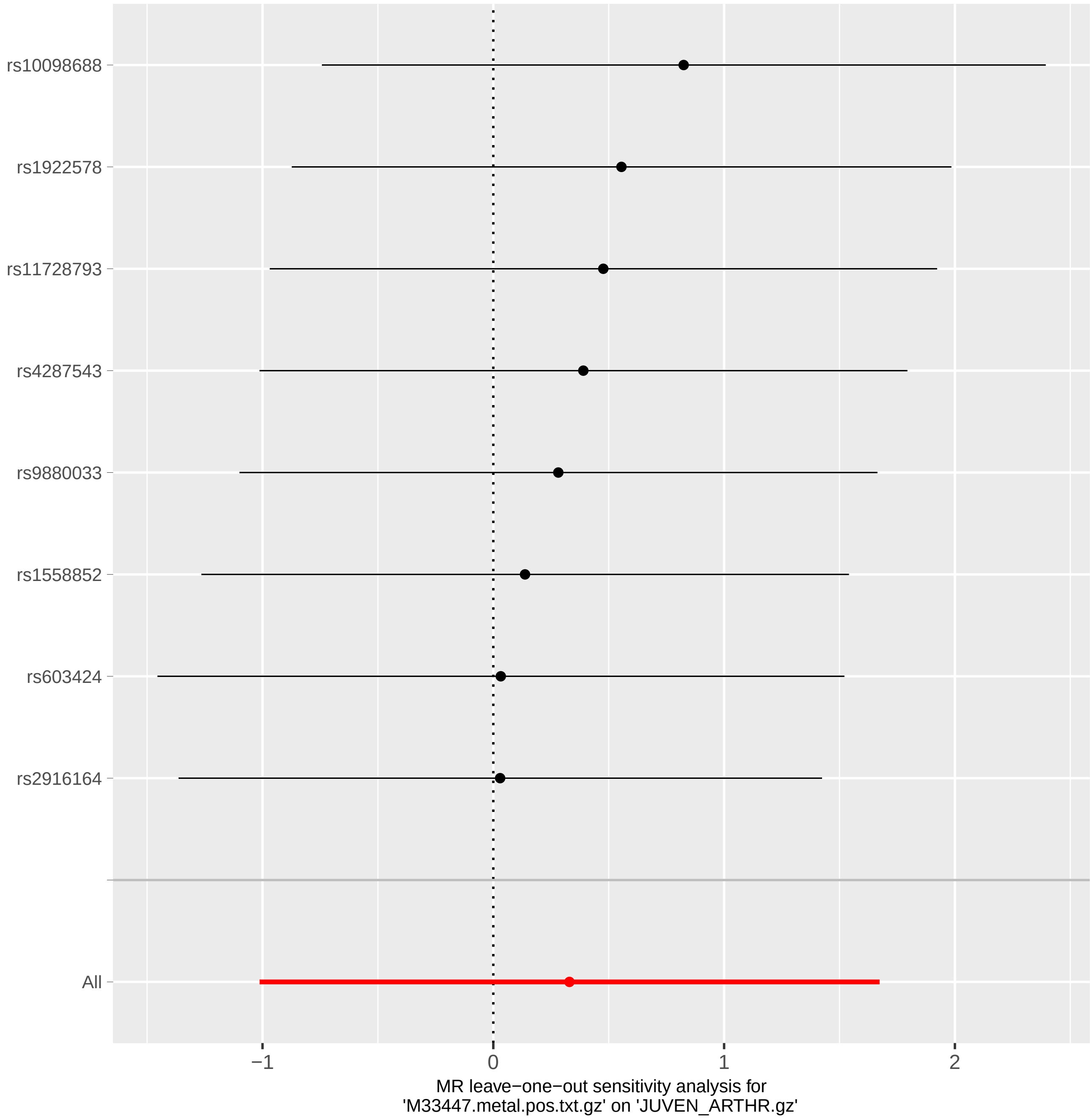
rs10098688
rs1922578
rs11728793
rs4287543
rs9880033
rs1558852
rs603424
rs2916164
All
−1
0
1
2
MR leave−one−out sensitivity analysis for
'M33447.metal.pos.txt.gz' on 'JUVEN_ARTHR.gz'

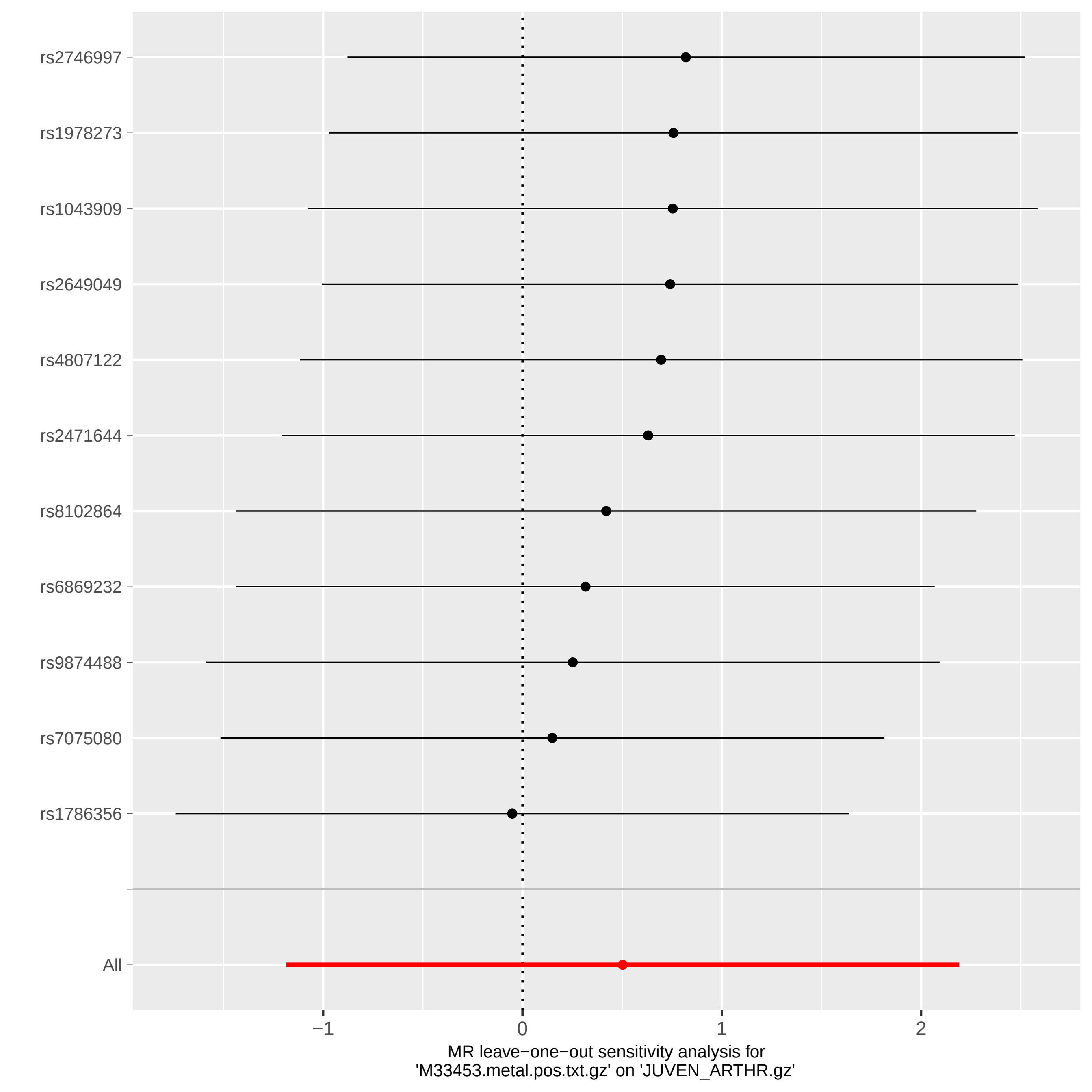
rs2746997
rs1978273
rs1043909
rs2649049
rs4807122
rs2471644
rs8102864
rs6869232
rs9874488
rs7075080
rs1786356
All
−1
0
1
2
MR leave−one−out sensitivity analysis for
'M33453.metal.pos.txt.gz' on 'JUVEN_ARTHR.gz'

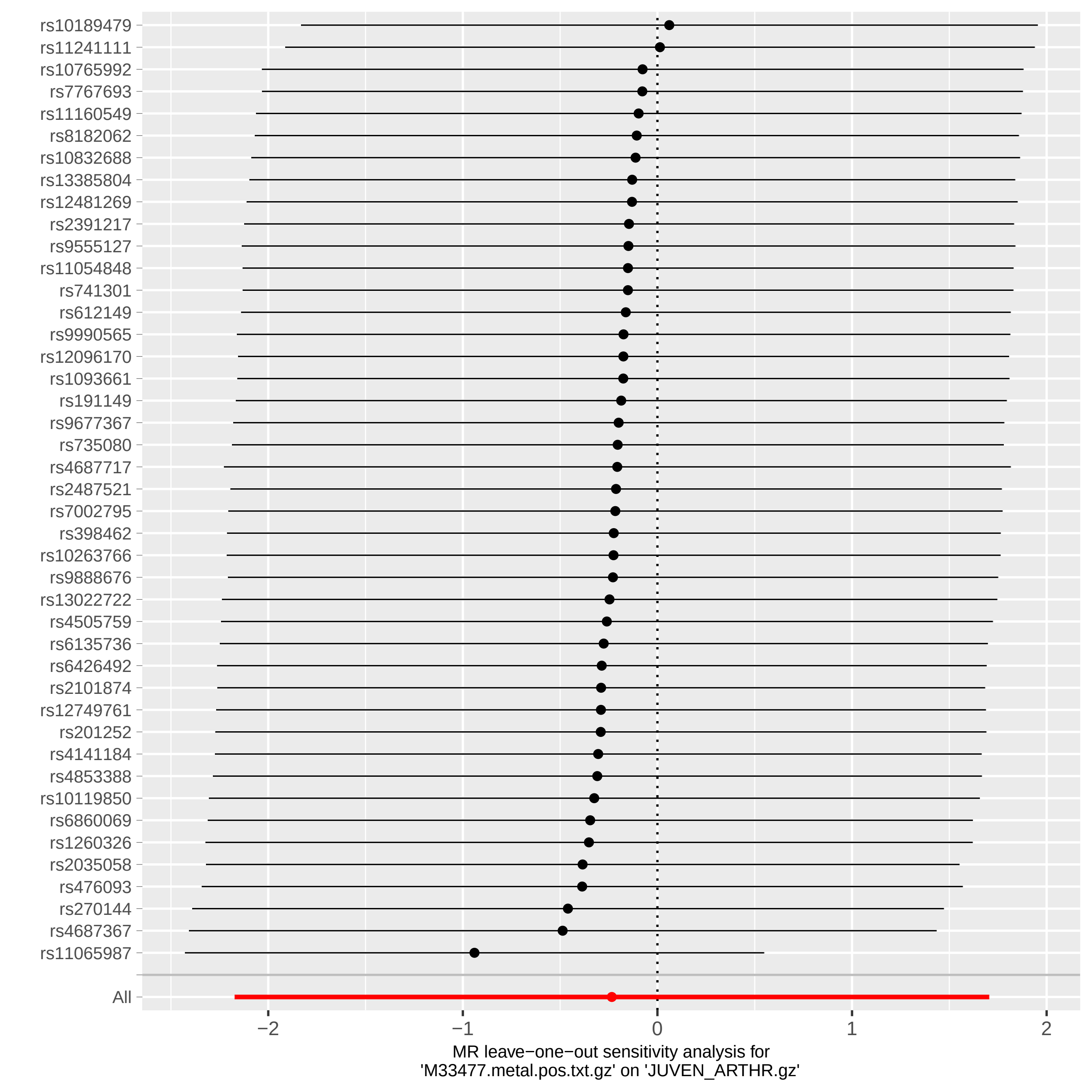
rs10189479
rs11241111
rs10765992
rs7767693
rs11160549
rs8182062
rs10832688
rs13385804
rs12481269
rs2391217
rs9555127
rs11054848
rs741301
rs612149
rs9990565
rs12096170
rs1093661
rs191149
rs9677367
rs735080
rs4687717
rs2487521
rs7002795
rs398462
rs10263766
rs9888676
rs13022722
rs4505759
rs6135736
rs6426492
rs2101874
rs12749761
rs201252
rs4141184
rs4853388
rs10119850
rs6860069
rs1260326
rs2035058
rs476093
rs270144
rs4687367
rs11065987
All
−2
−1
0
1
2
MR leave−one−out sensitivity analysis for
'M33477.metal.pos.txt.gz' on 'JUVEN_ARTHR.gz'

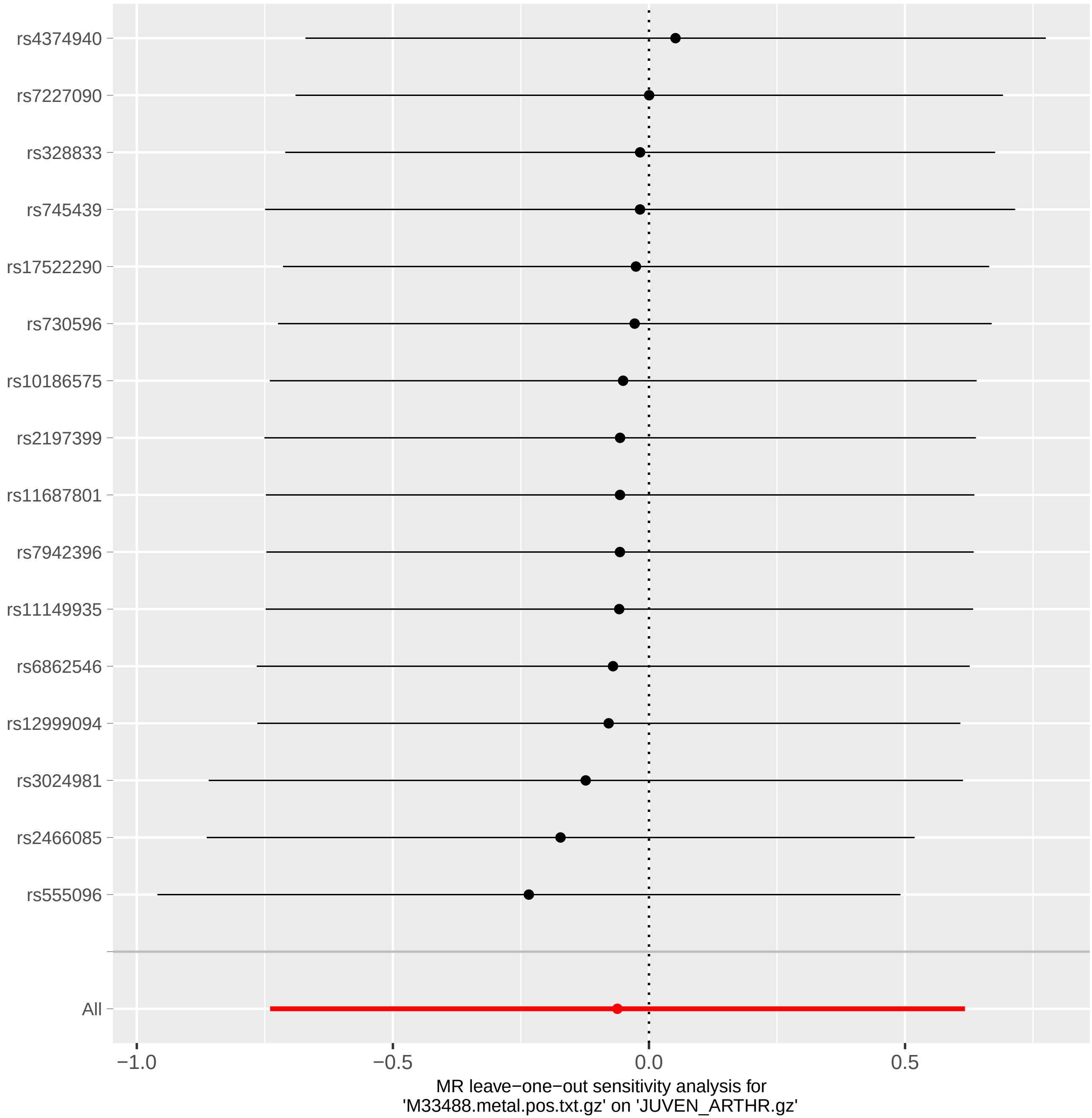
rs4374940
rs7227090
rs328833
rs745439
rs17522290
rs730596
rs10186575
rs2197399
rs11687801
rs7942396
rs11149935
rs6862546
rs12999094
rs3024981
rs2466085
rs555096
All
−1.0
−0.5
0.0
0.5
MR leave−one−out sensitivity analysis for
'M33488.metal.pos.txt.gz' on 'JUVEN_ARTHR.gz'

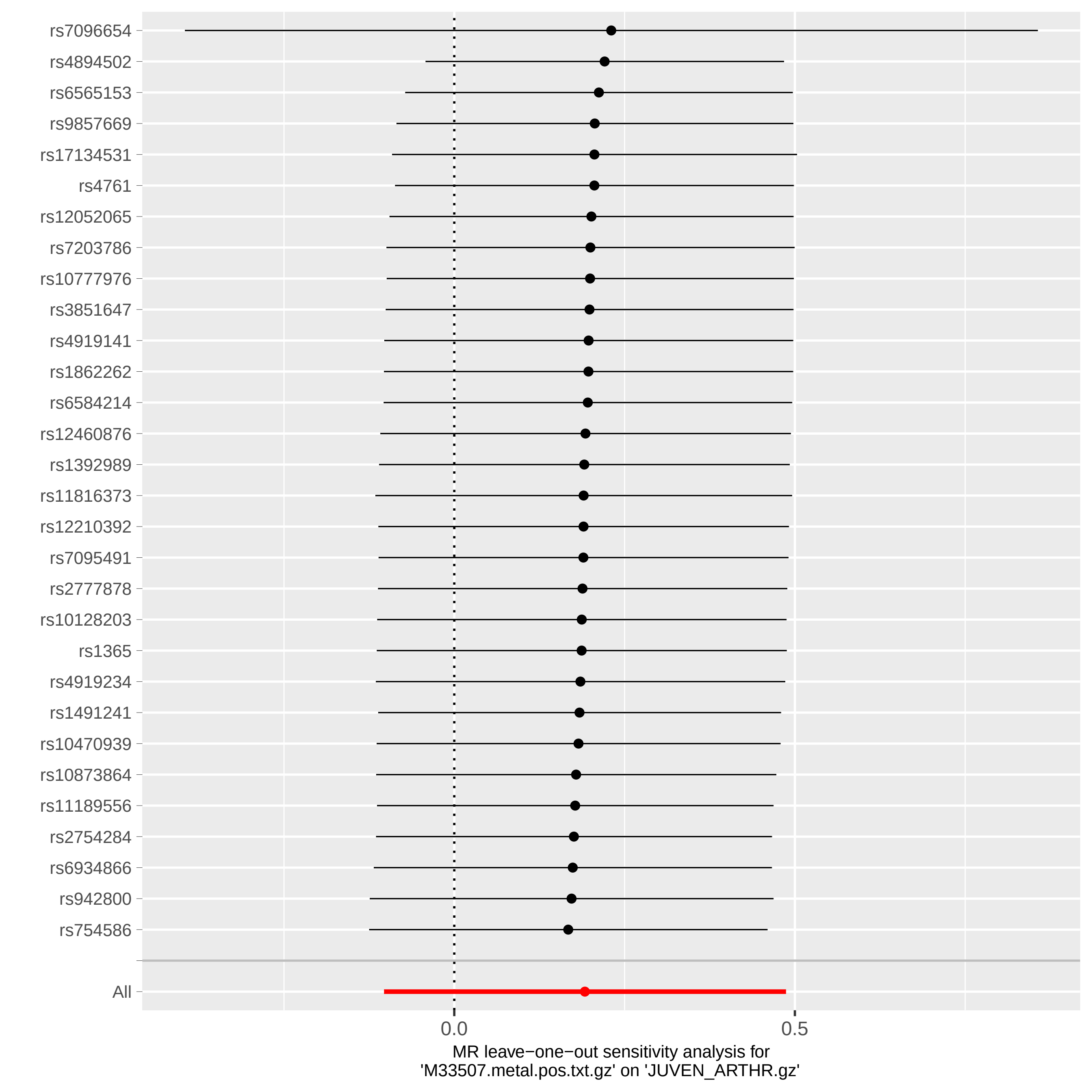
rs7096654
rs4894502
rs6565153
rs9857669
rs17134531
rs4761
rs12052065
rs7203786
rs10777976
rs3851647
rs4919141
rs1862262
rs6584214
rs12460876
rs1392989
rs11816373
rs12210392
rs7095491
rs2777878
rs10128203
rs1365
rs4919234
rs1491241
rs10470939
rs10873864
rs11189556
rs2754284
rs6934866
rs942800
rs754586
All
0.0
0.5
MR leave−one−out sensitivity analysis for
'M33507.metal.pos.txt.gz' on 'JUVEN_ARTHR.gz'

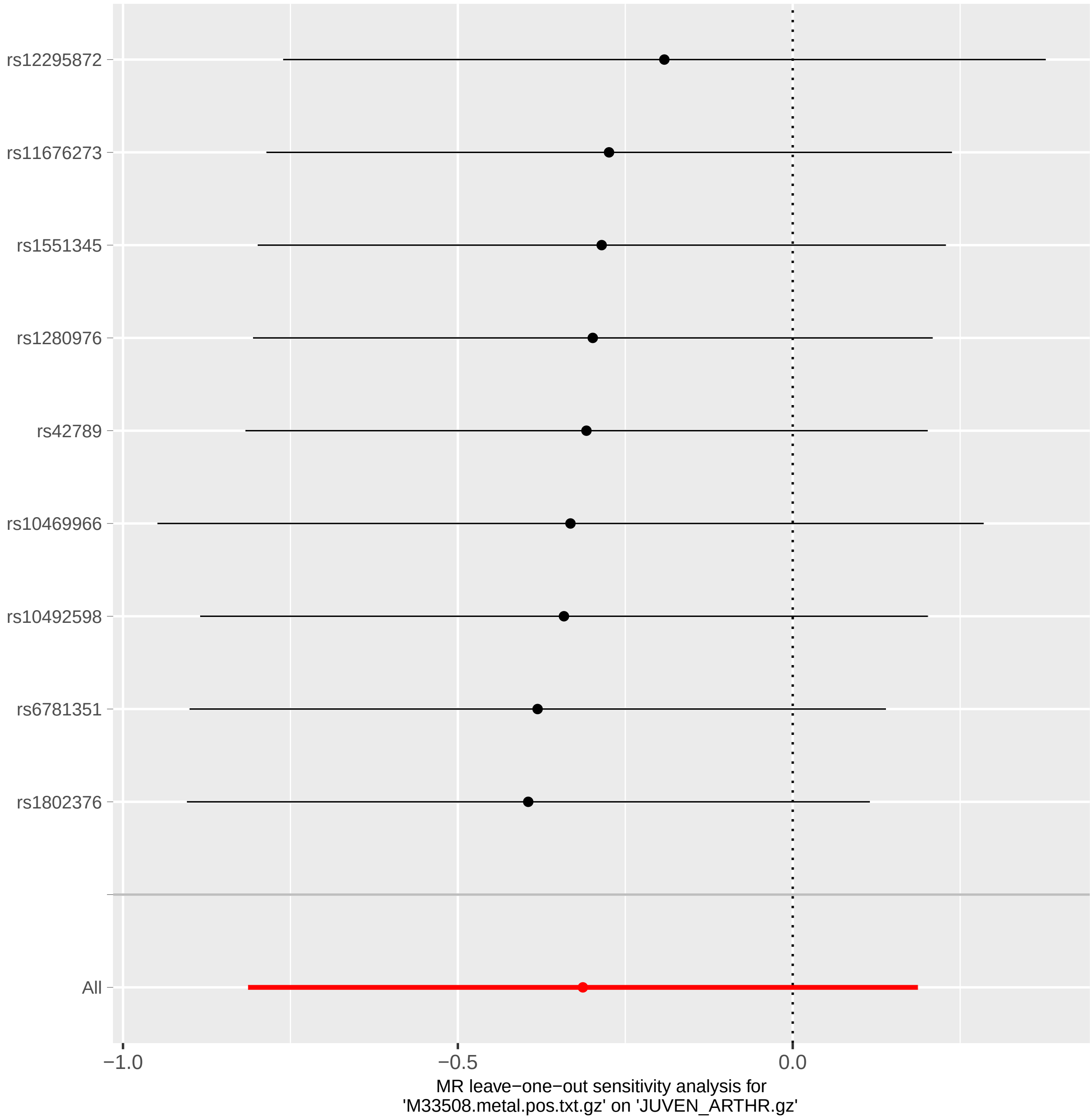

rs12295872
rs11676273
rs1551345
rs1280976
rs42789
rs10469966
rs10492598
rs6781351
rs1802376
All
−1.0
−0.5
0.0
MR leave−one−out sensitivity analysis for
'M33508.metal.pos.txt.gz' on 'JUVEN_ARTHR.gz'

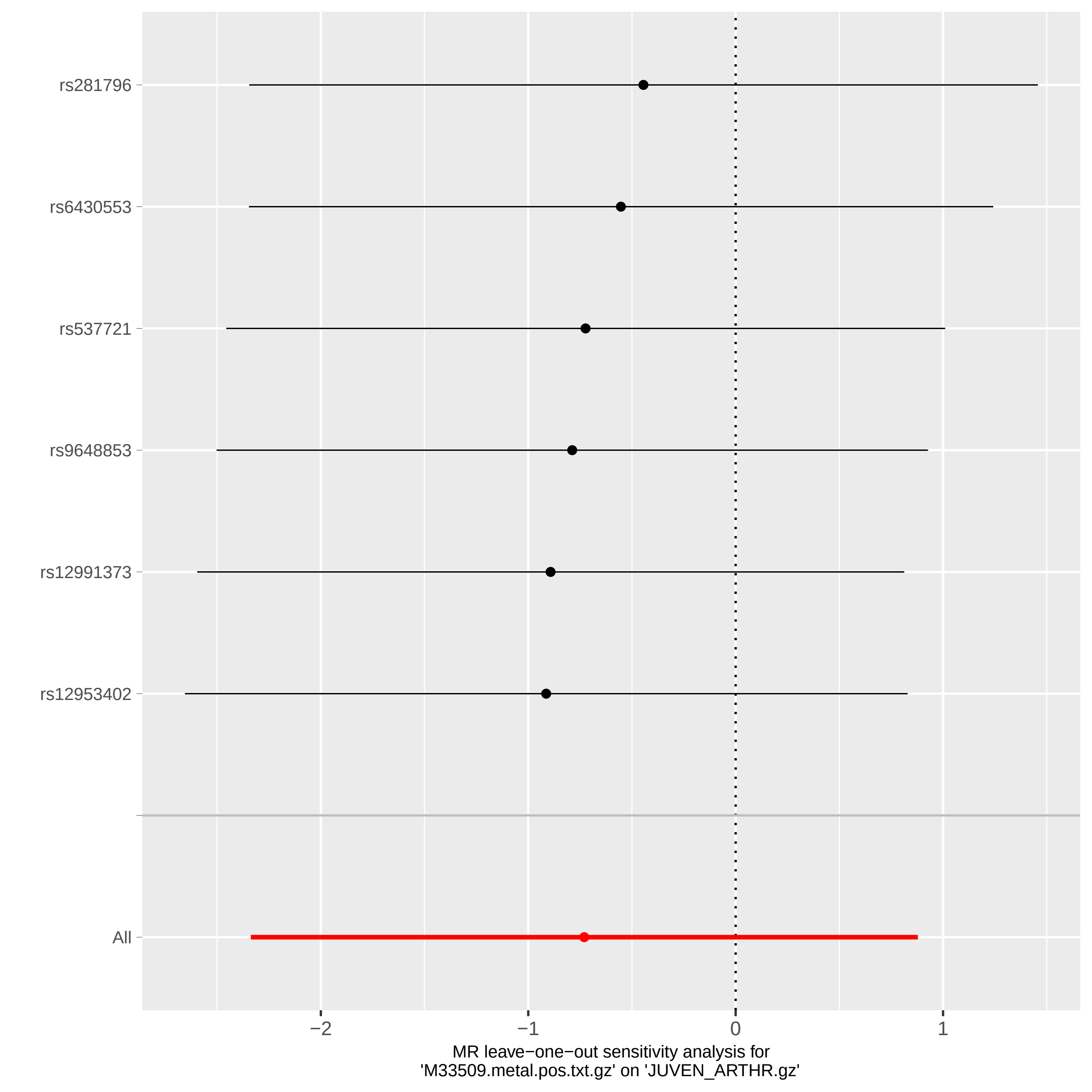

rs281796
rs6430553
rs537721
rs9648853
rs12991373
rs12953402
All
−2
−1
0
1
MR leave−one−out sensitivity analysis for
'M33509.metal.pos.txt.gz' on 'JUVEN_ARTHR.gz'

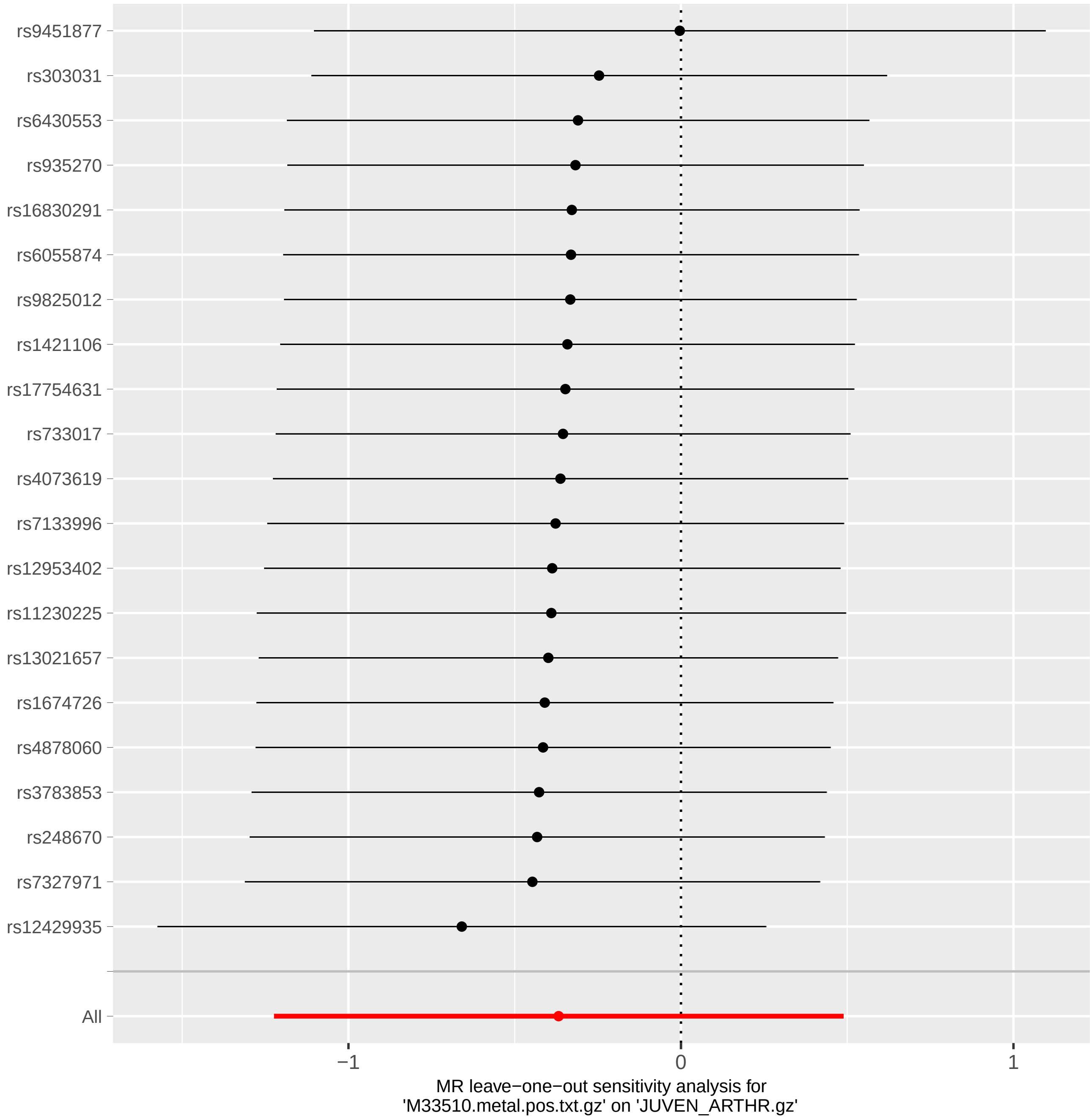

rs9451877
rs303031
rs6430553
rs935270
rs16830291
rs6055874
rs9825012
rs1421106
rs17754631
rs733017
rs4073619
rs7133996
rs12953402
rs11230225
rs13021657
rs1674726
rs4878060
rs3783853
rs248670
rs7327971
rs12429935
All
−1
0
1
MR leave−one−out sensitivity analysis for
'M33510.metal.pos.txt.gz' on 'JUVEN_ARTHR.gz'

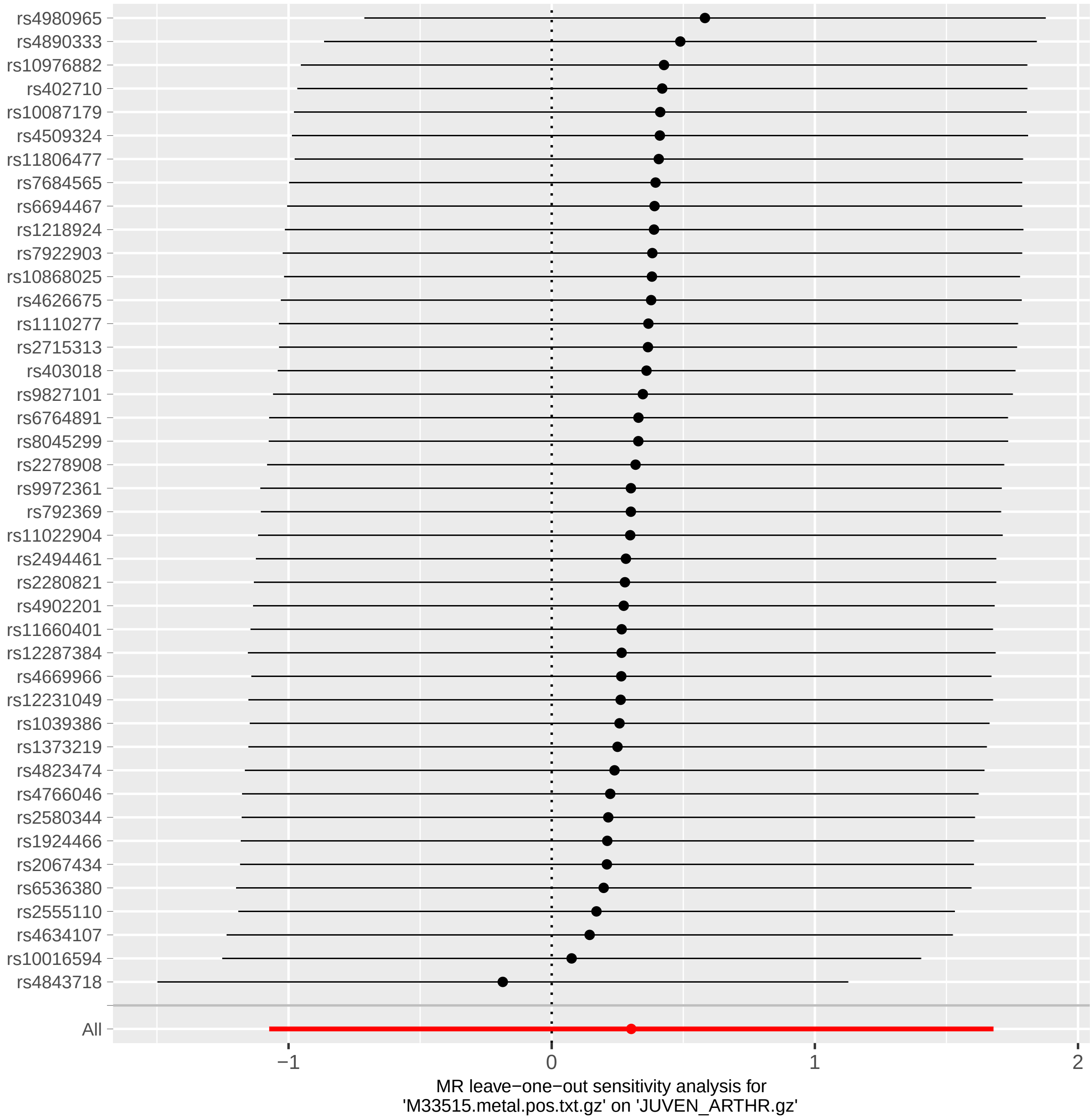
rs4980965
rs4890333
rs10976882
rs402710
rs10087179
rs4509324
rs11806477
rs7684565
rs6694467
rs1218924
rs7922903
rs10868025
rs4626675
rs1110277
rs2715313
rs403018
rs9827101
rs6764891
rs8045299
rs2278908
rs9972361
rs792369
rs11022904
rs2494461
rs2280821
rs4902201
rs11660401
rs12287384
rs4669966
rs12231049
rs1039386
rs1373219
rs4823474
rs4766046
rs2580344
rs1924466
rs2067434
rs6536380
rs2555110
rs4634107
rs10016594
rs4843718
All
−1
0
1
2
MR leave−one−out sensitivity analysis for
'M33515.metal.pos.txt.gz' on 'JUVEN_ARTHR.gz'

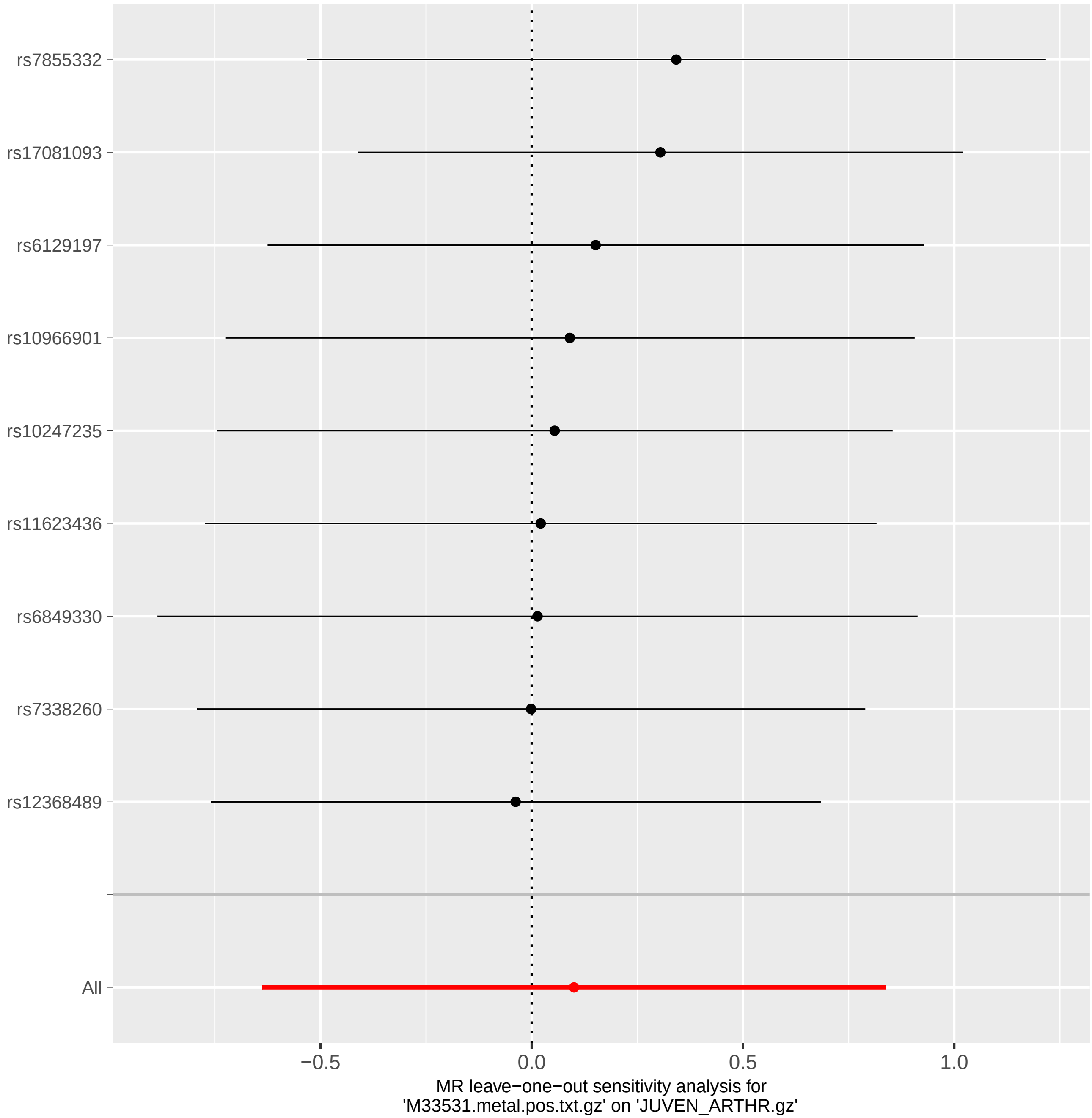

rs7855332
rs17081093
rs6129197
rs10966901
rs10247235
rs11623436
rs6849330
rs7338260
rs12368489
All
−0.5
0.0
0.5
1.0
MR leave−one−out sensitivity analysis for
'M33531.metal.pos.txt.gz' on 'JUVEN_ARTHR.gz'

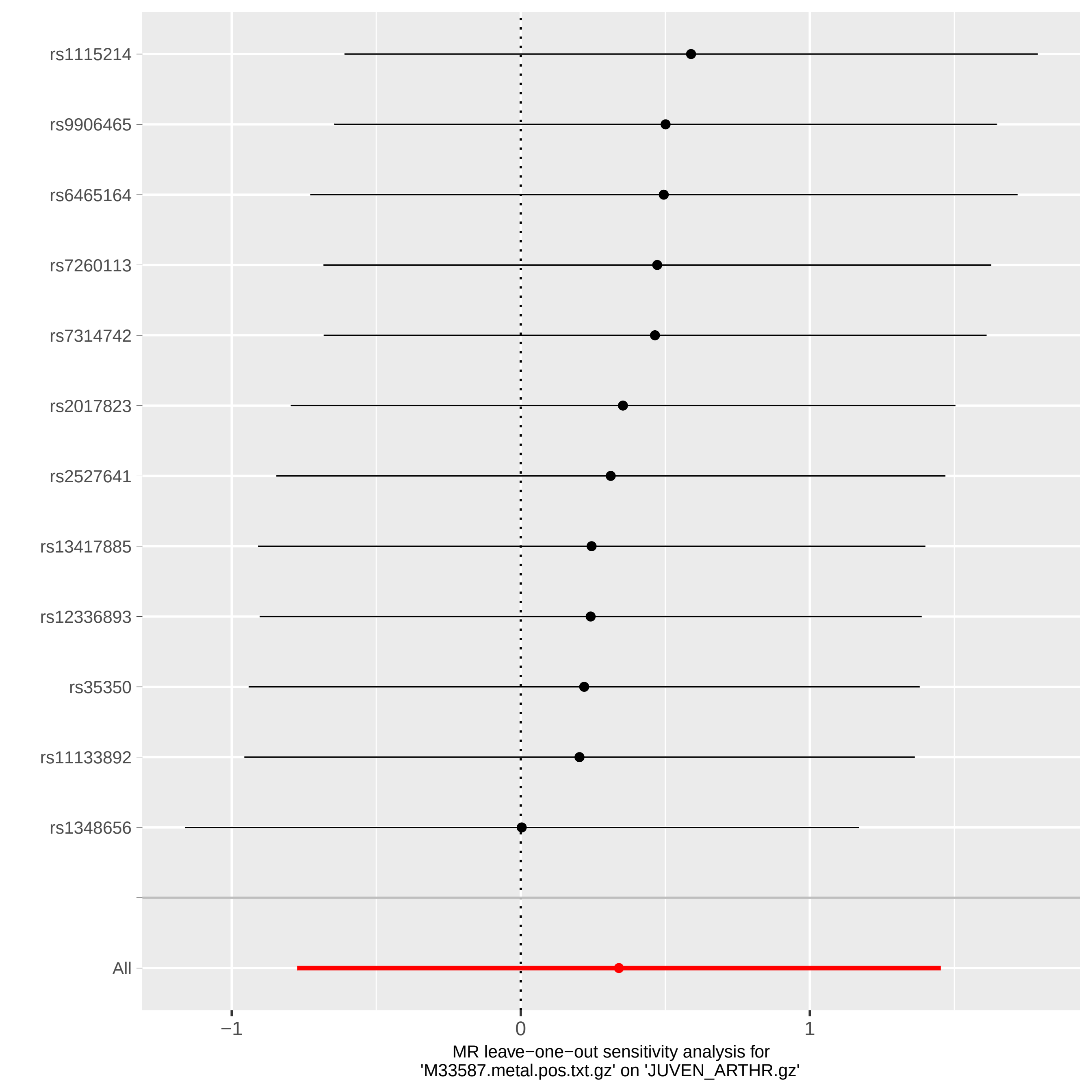
rs1115214
rs9906465
rs6465164
rs7260113
rs7314742
rs2017823
rs2527641
rs13417885
rs12336893
rs35350
rs11133892
rs1348656
All
−1
0
1
MR leave−one−out sensitivity analysis for
'M33587.metal.pos.txt.gz' on 'JUVEN_ARTHR.gz'

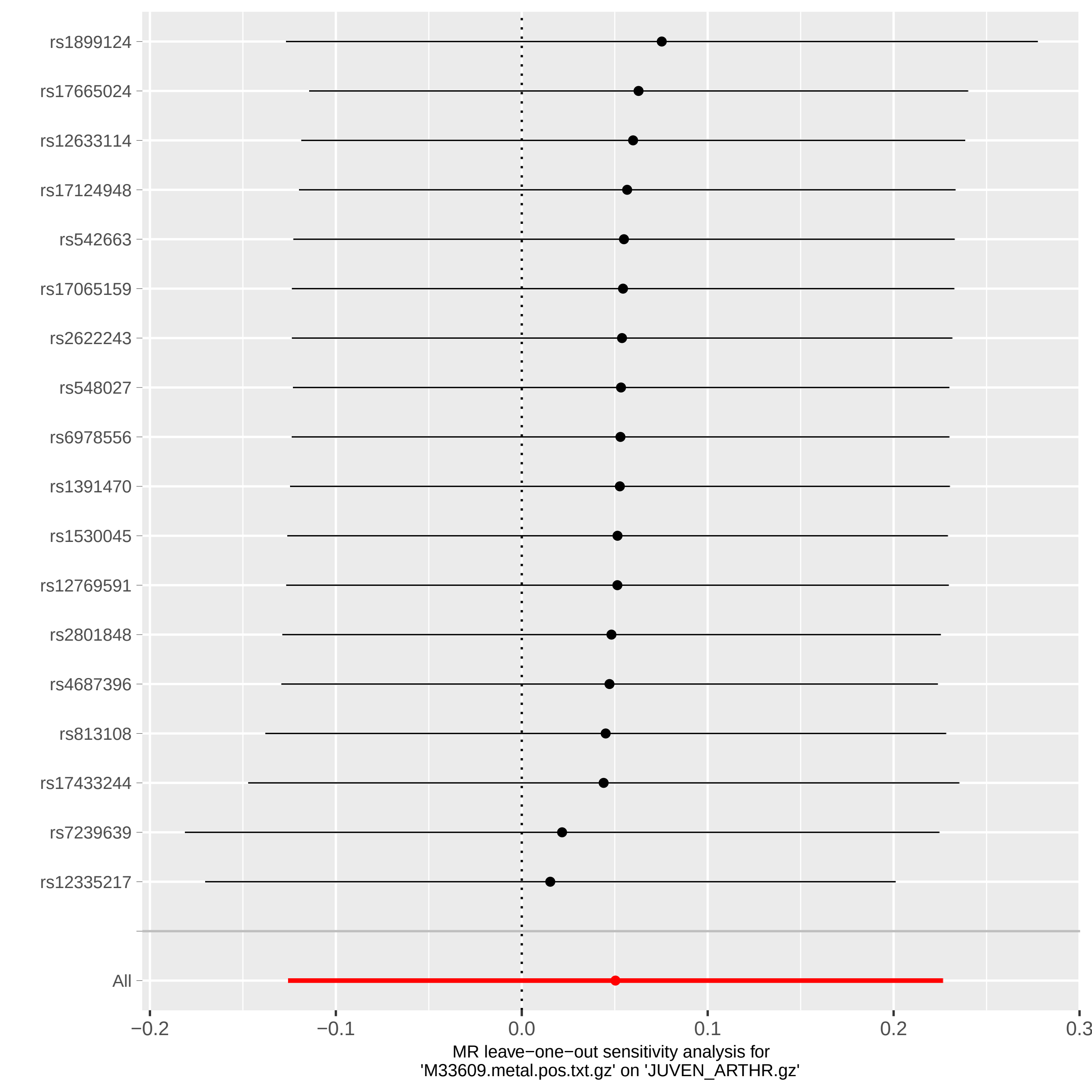
rs1899124
rs17665024
rs12633114
rs17124948
rs542663
rs17065159
rs2622243
rs548027
rs6978556
rs1391470
rs1530045
rs12769591
rs2801848
rs4687396
rs813108
rs17433244
rs7239639
rs12335217
All
−0.2
−0.1
0.0
0.1
0.2
0.3
MR leave−one−out sensitivity analysis for
'M33609.metal.pos.txt.gz' on 'JUVEN_ARTHR.gz'

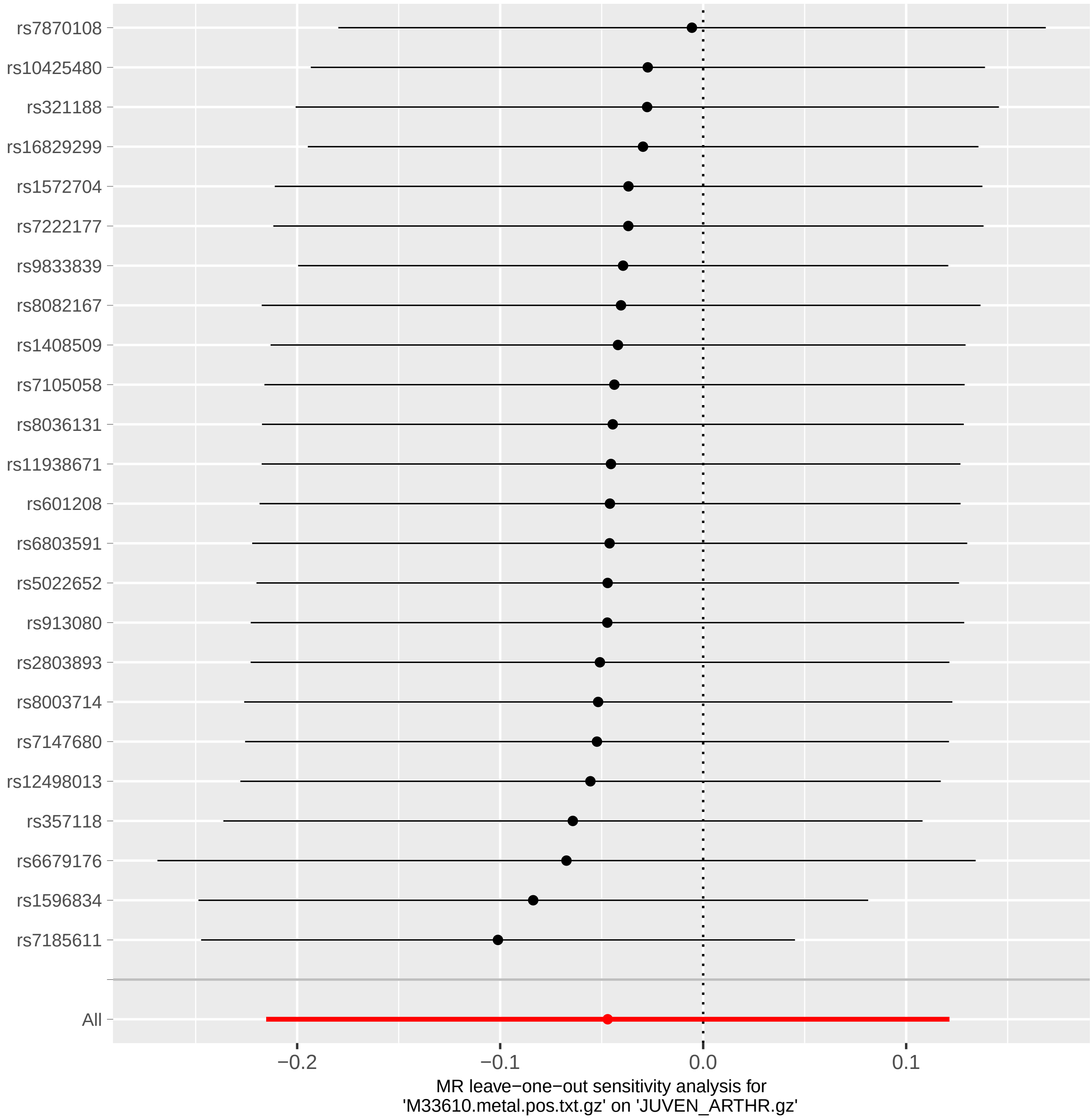
rs7870108
rs10425480
rs321188
rs16829299
rs1572704
rs7222177
rs9833839
rs8082167
rs1408509
rs7105058
rs8036131
rs11938671
rs601208
rs6803591
rs5022652
rs913080
rs2803893
rs8003714
rs7147680
rs12498013
rs357118
rs6679176
rs1596834
rs7185611
All
−0.2
−0.1
0.0
0.1
MR leave−one−out sensitivity analysis for
'M33610.metal.pos.txt.gz' on 'JUVEN_ARTHR.gz'

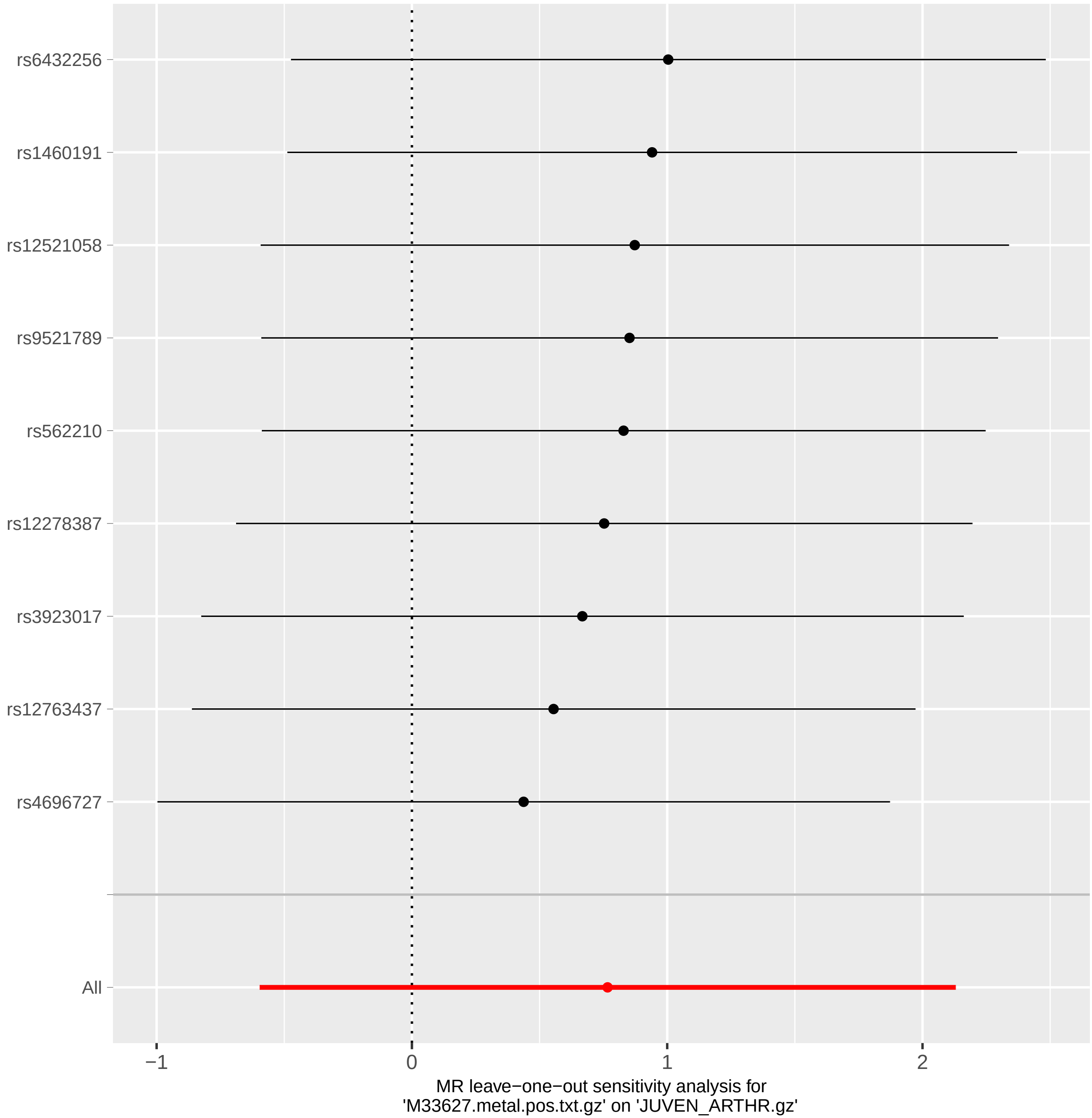

rs6432256
rs1460191
rs12521058
rs9521789
rs562210
rs12278387
rs3923017
rs12763437
rs4696727
All
−1
0
1
2
MR leave−one−out sensitivity analysis for
'M33627.metal.pos.txt.gz' on 'JUVEN_ARTHR.gz'

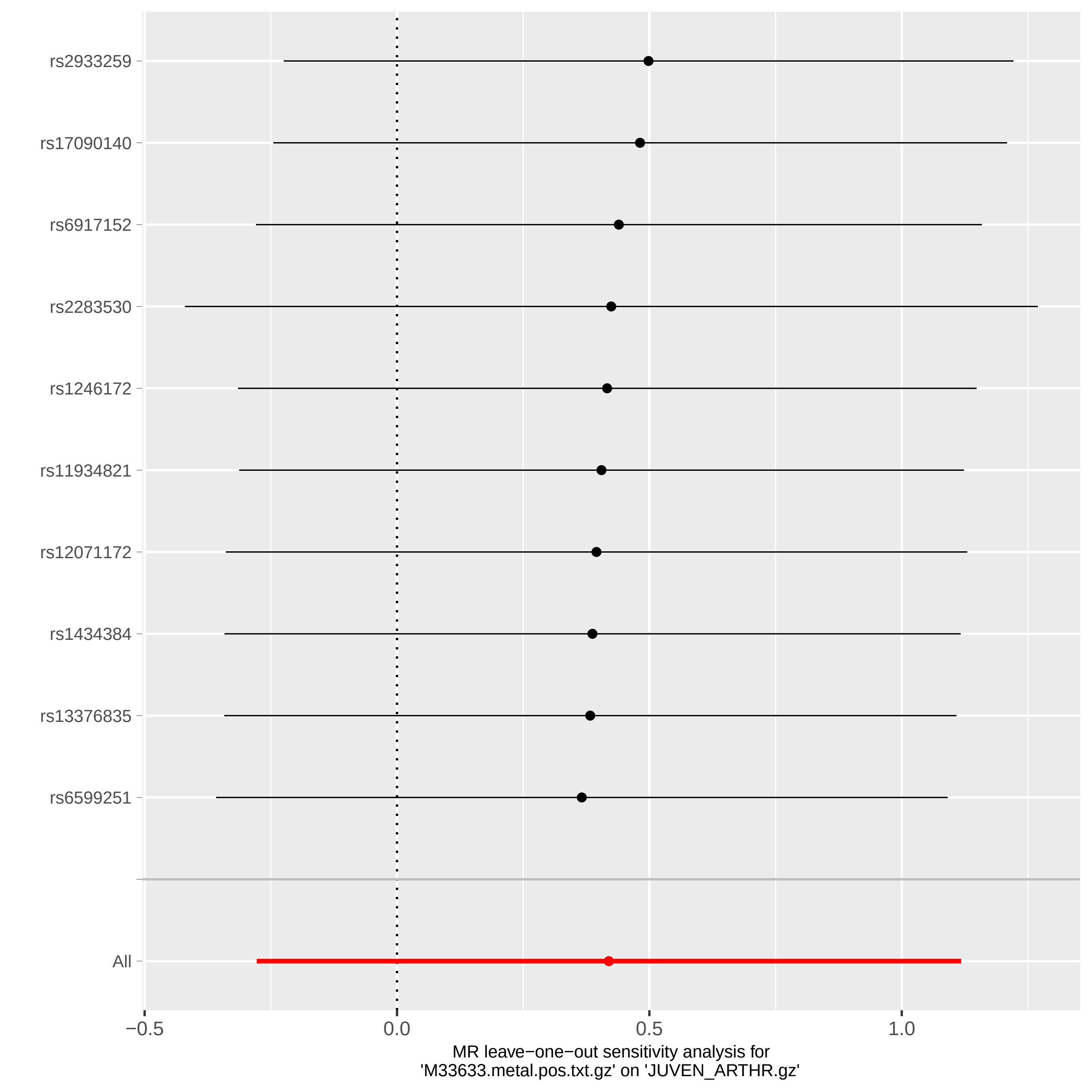

rs2933259
rs17090140
rs6917152
rs2283530
rs1246172
rs11934821
rs12071172
rs1434384
rs13376835
rs6599251
All
−0.5
0.0
0.5
1.0
MR leave−one−out sensitivity analysis for
'M33633.metal.pos.txt.gz' on 'JUVEN_ARTHR.gz'

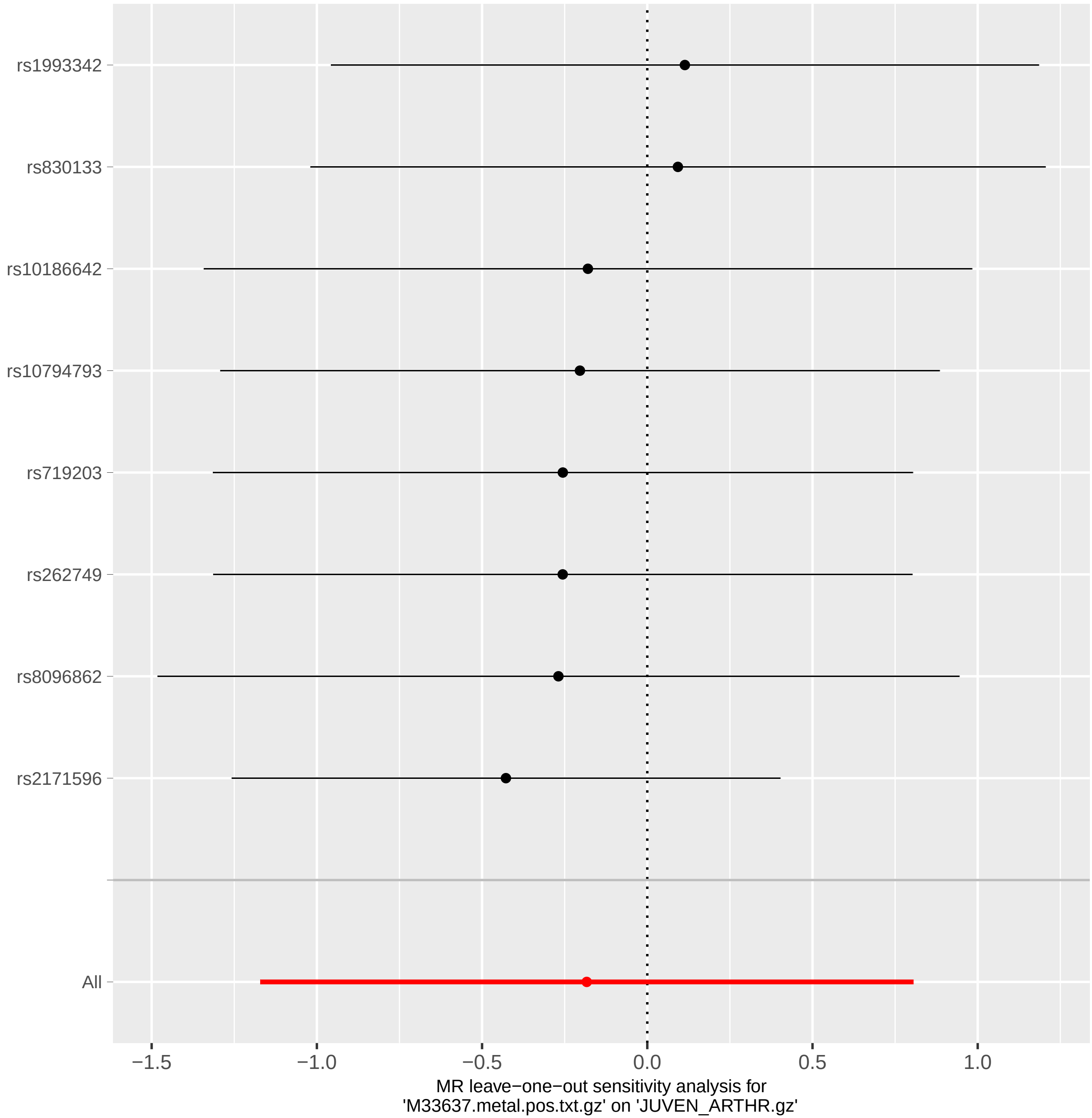
rs1993342
rs830133
rs10186642
rs10794793
rs719203
rs262749
rs8096862
rs2171596
All
−1.5
−1.0
−0.5
0.0
0.5
1.0
MR leave−one−out sensitivity analysis for
'M33637.metal.pos.txt.gz' on 'JUVEN_ARTHR.gz'

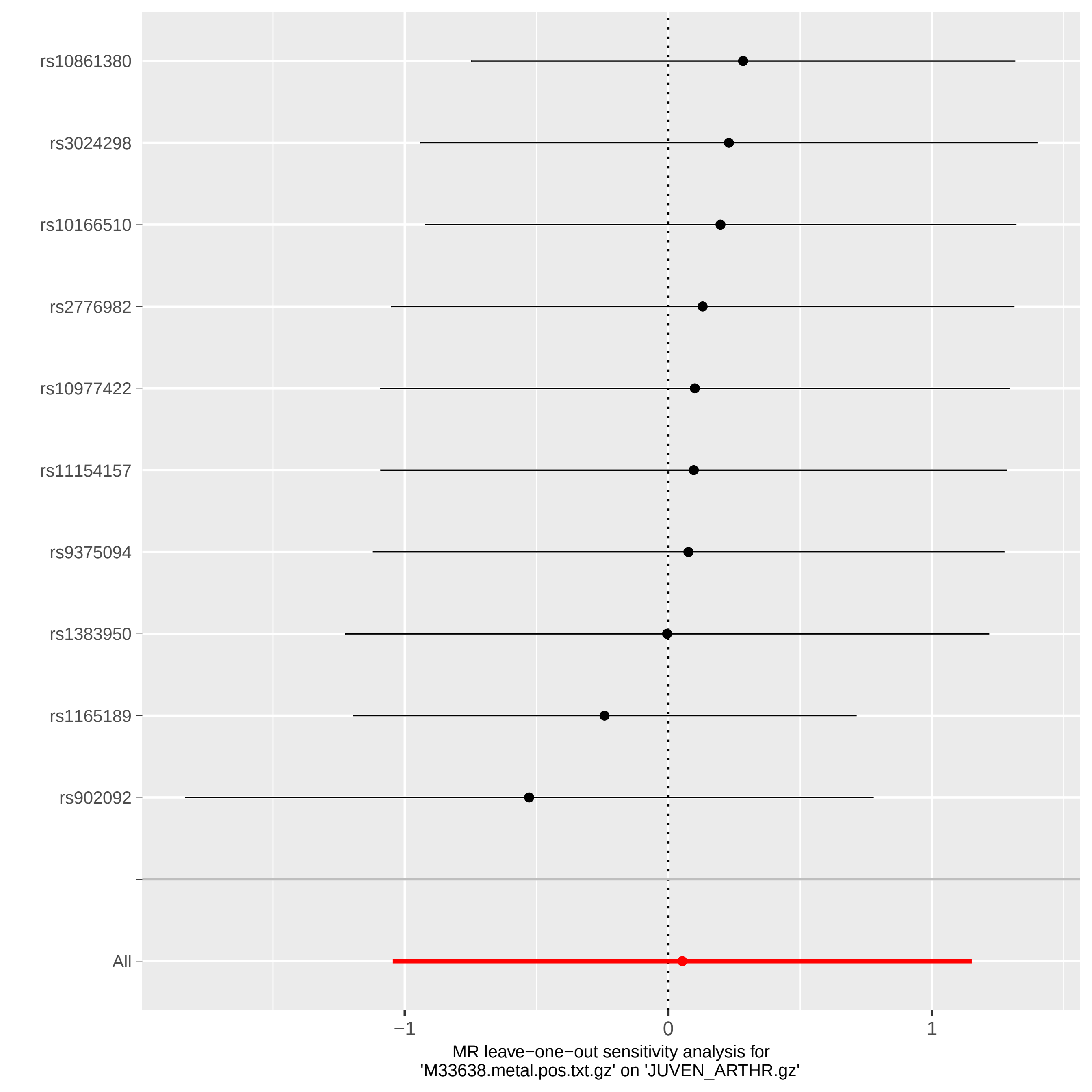

rs10861380
rs3024298
rs10166510
rs2776982
rs10977422
rs11154157
rs9375094
rs1383950
rs1165189
rs902092
All
−1
0
1
MR leave−one−out sensitivity analysis for
'M33638.metal.pos.txt.gz' on 'JUVEN_ARTHR.gz'

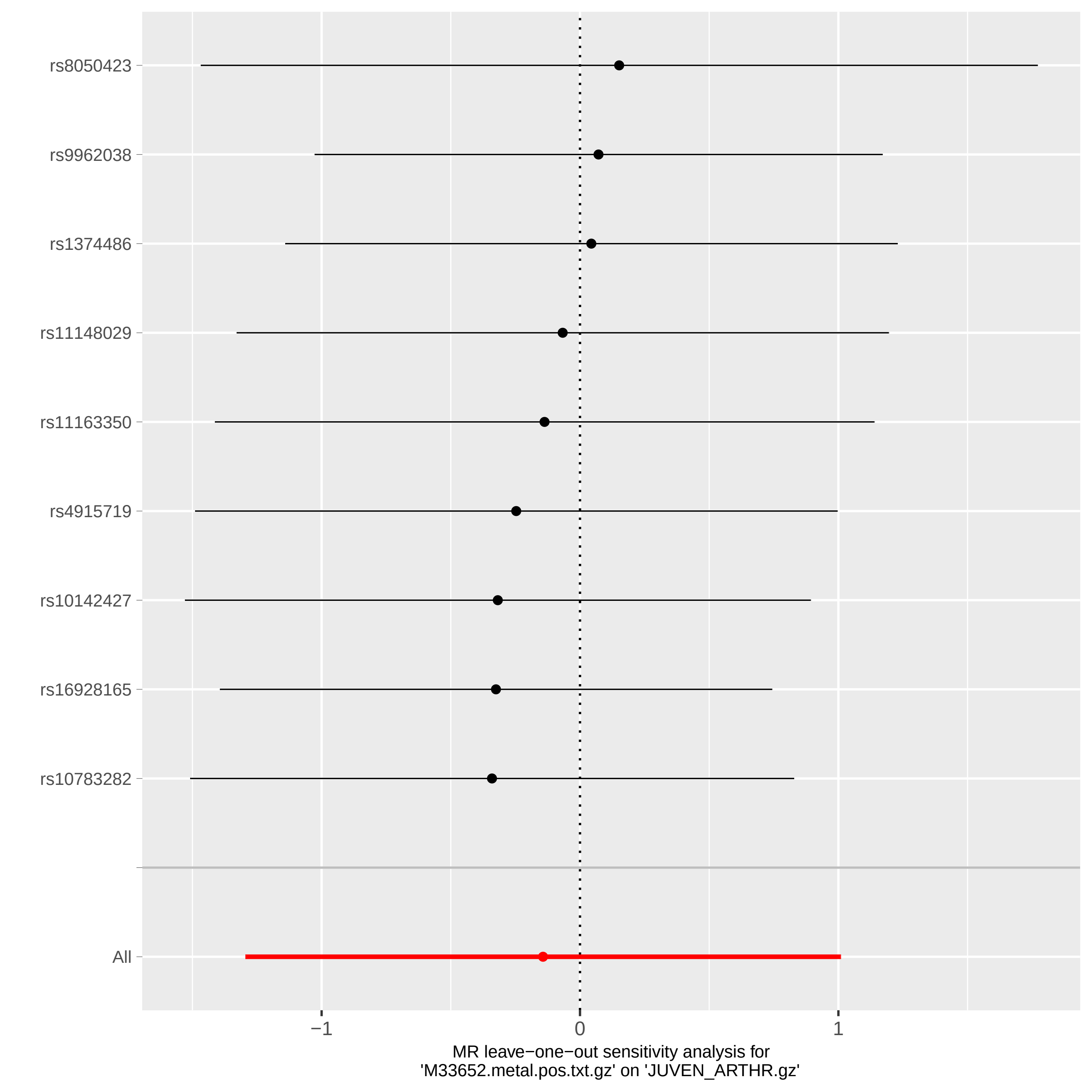
rs8050423
rs9962038
rs1374486
rs11148029
rs11163350
rs4915719
rs10142427
rs16928165
rs10783282
All
−1
0
1
MR leave−one−out sensitivity analysis for
'M33652.metal.pos.txt.gz' on 'JUVEN_ARTHR.gz'

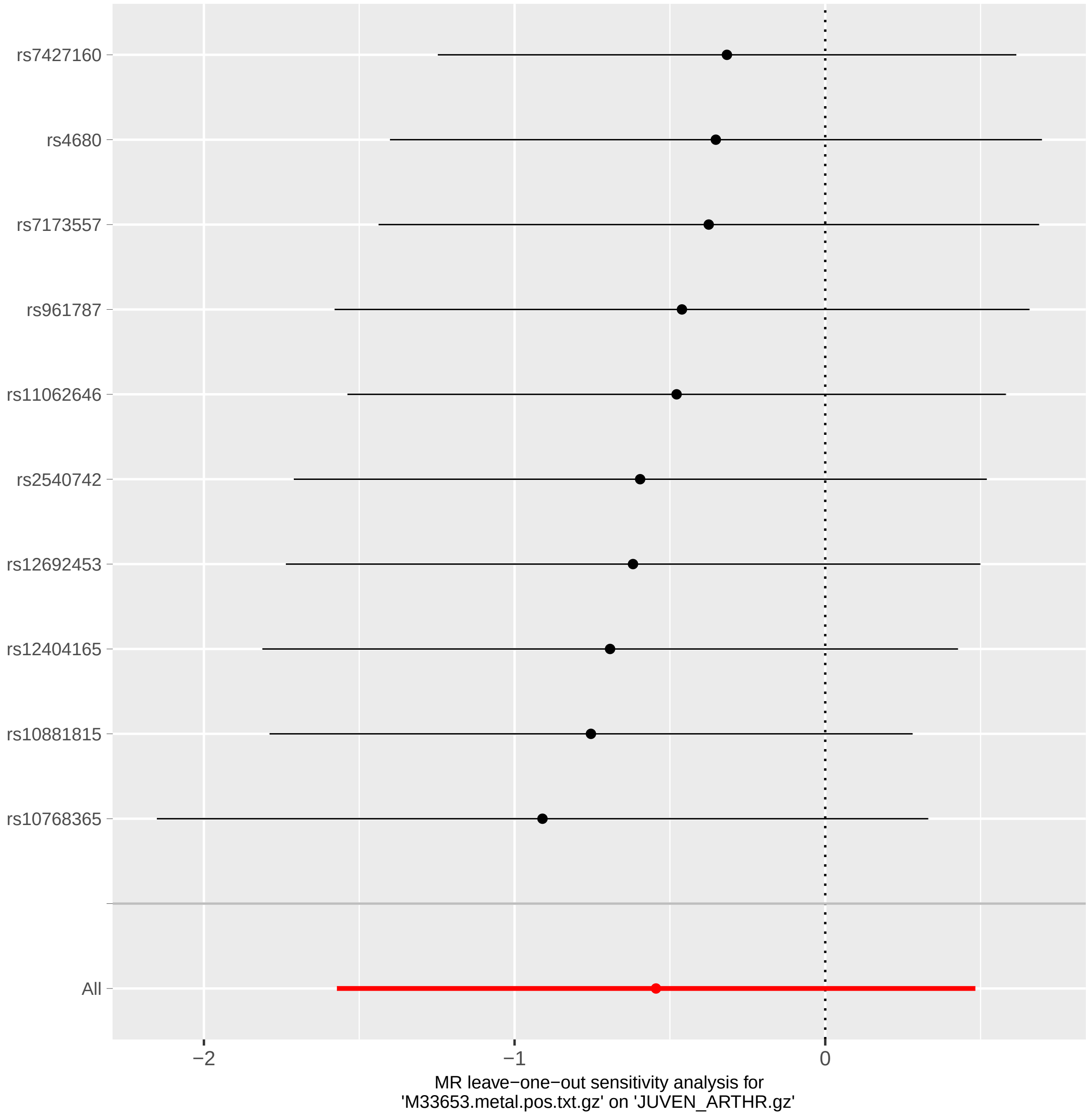
rs7427160
rs4680
rs7173557
rs961787
rs11062646
rs2540742
rs12692453
rs12404165
rs10881815
rs10768365
All
−2
−1
0
MR leave−one−out sensitivity analysis for
'M33653.metal.pos.txt.gz' on 'JUVEN_ARTHR.gz'

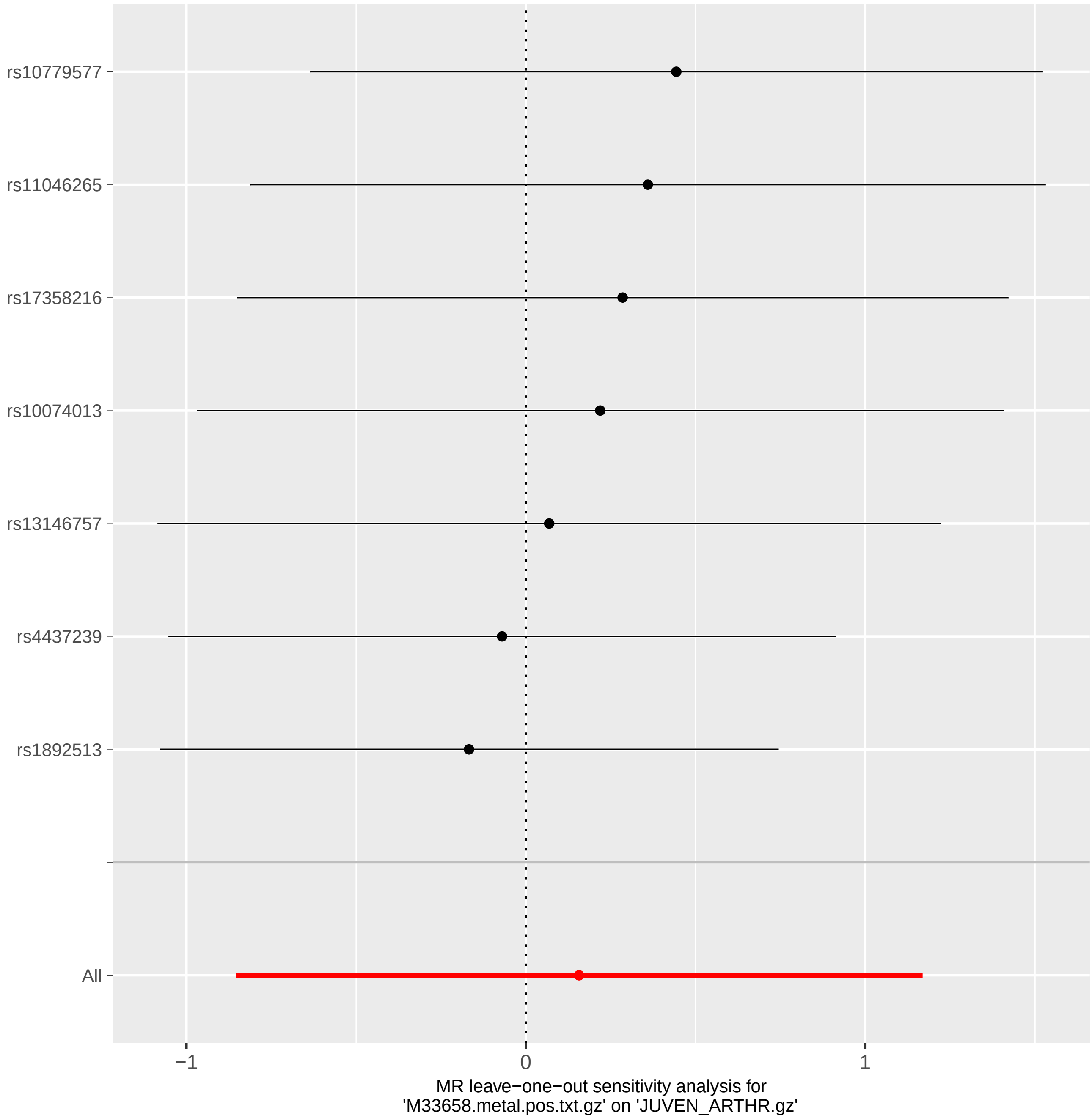

rs10779577
rs11046265
rs17358216
rs10074013
rs13146757
rs4437239
rs1892513
All
−1
0
1
MR leave−one−out sensitivity analysis for
'M33658.metal.pos.txt.gz' on 'JUVEN_ARTHR.gz'

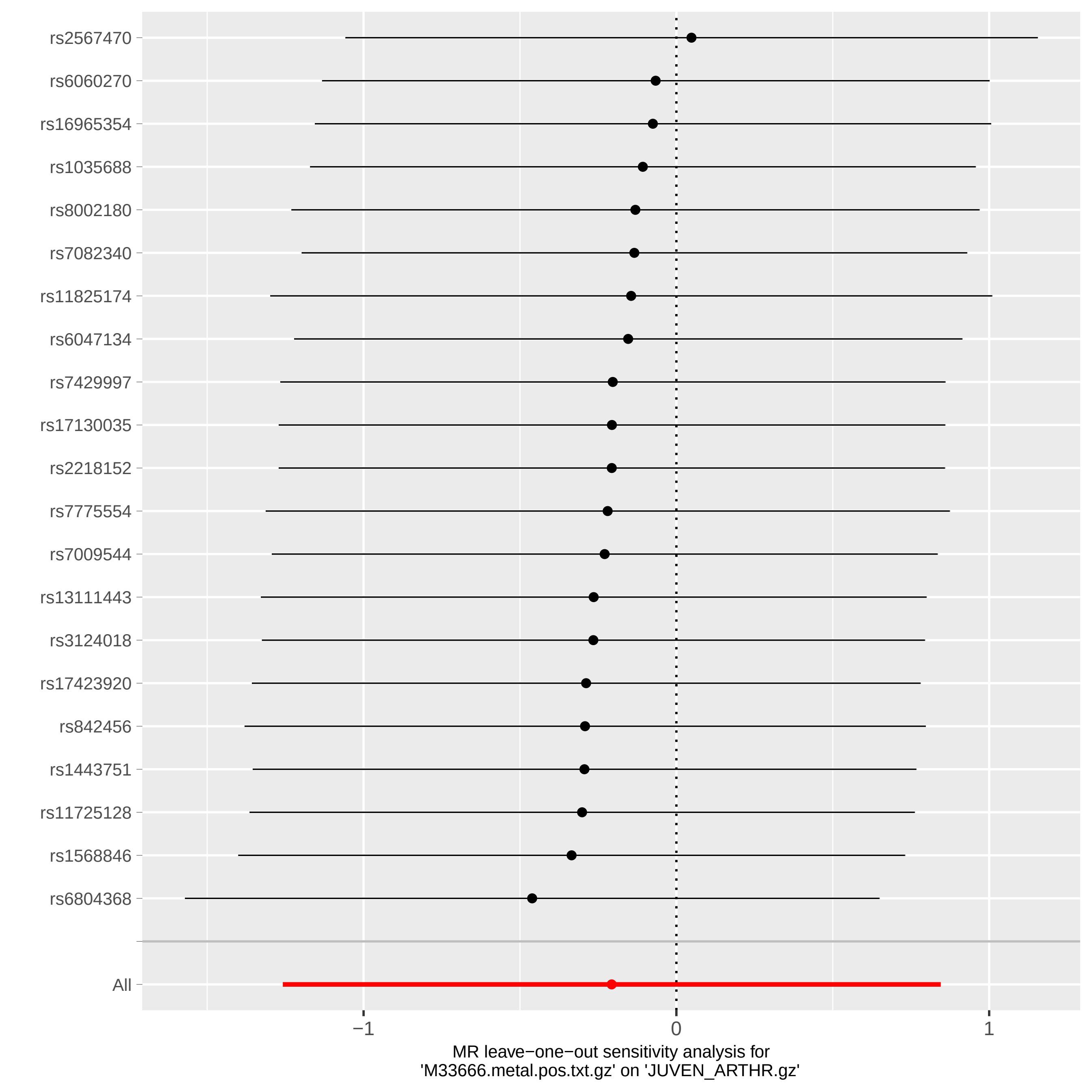

rs2567470
rs6060270
rs16965354
rs1035688
rs8002180
rs7082340
rs11825174
rs6047134
rs7429997
rs17130035
rs2218152
rs7775554
rs7009544
rs13111443
rs3124018
rs17423920
rs842456
rs1443751
rs11725128
rs1568846
rs6804368
All
−1
0
1
MR leave−one−out sensitivity analysis for
'M33666.metal.pos.txt.gz' on 'JUVEN_ARTHR.gz'

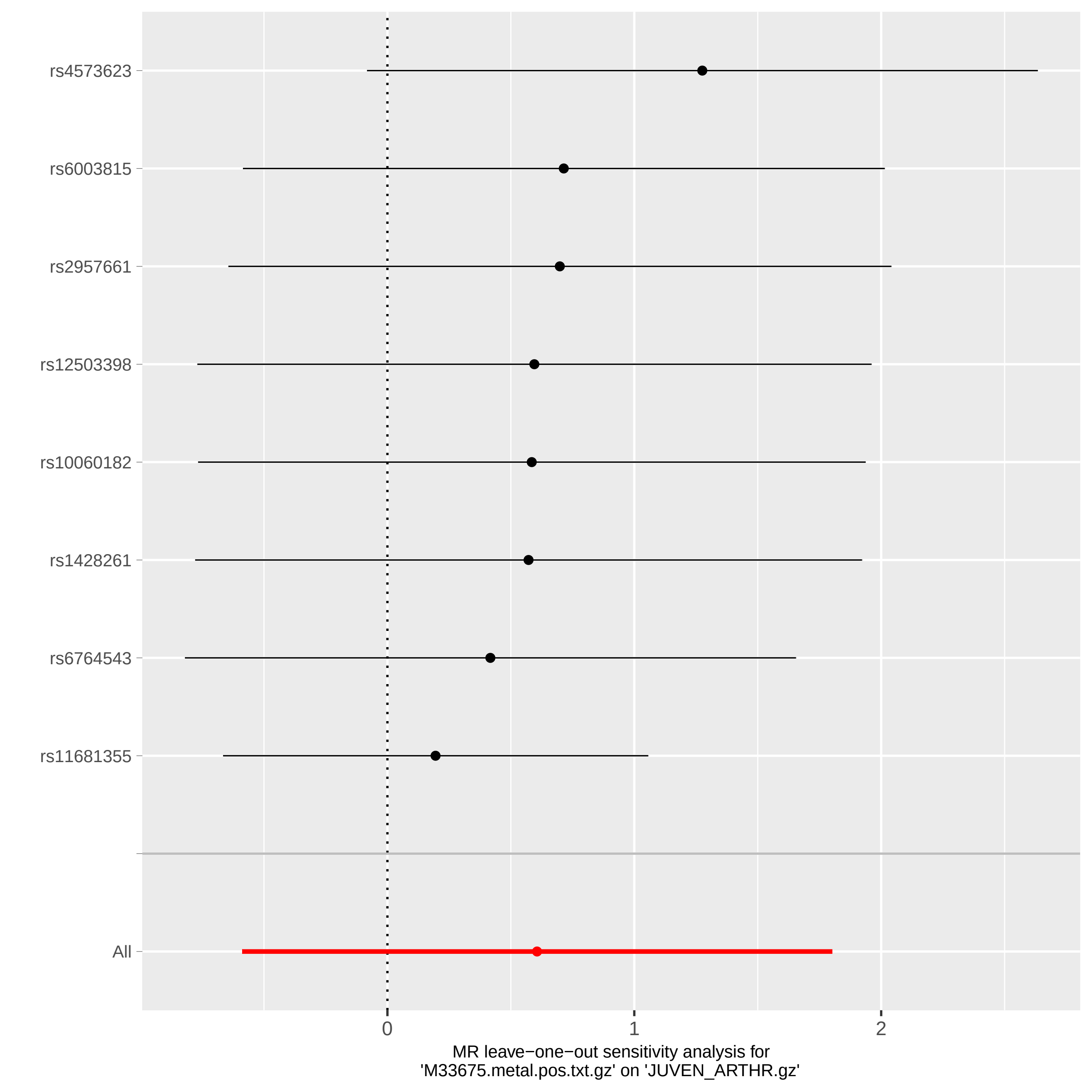
rs4573623
rs6003815
rs2957661
rs12503398
rs10060182
rs1428261
rs6764543
rs11681355
All
0
1
2
MR leave−one−out sensitivity analysis for
'M33675.metal.pos.txt.gz' on 'JUVEN_ARTHR.gz'

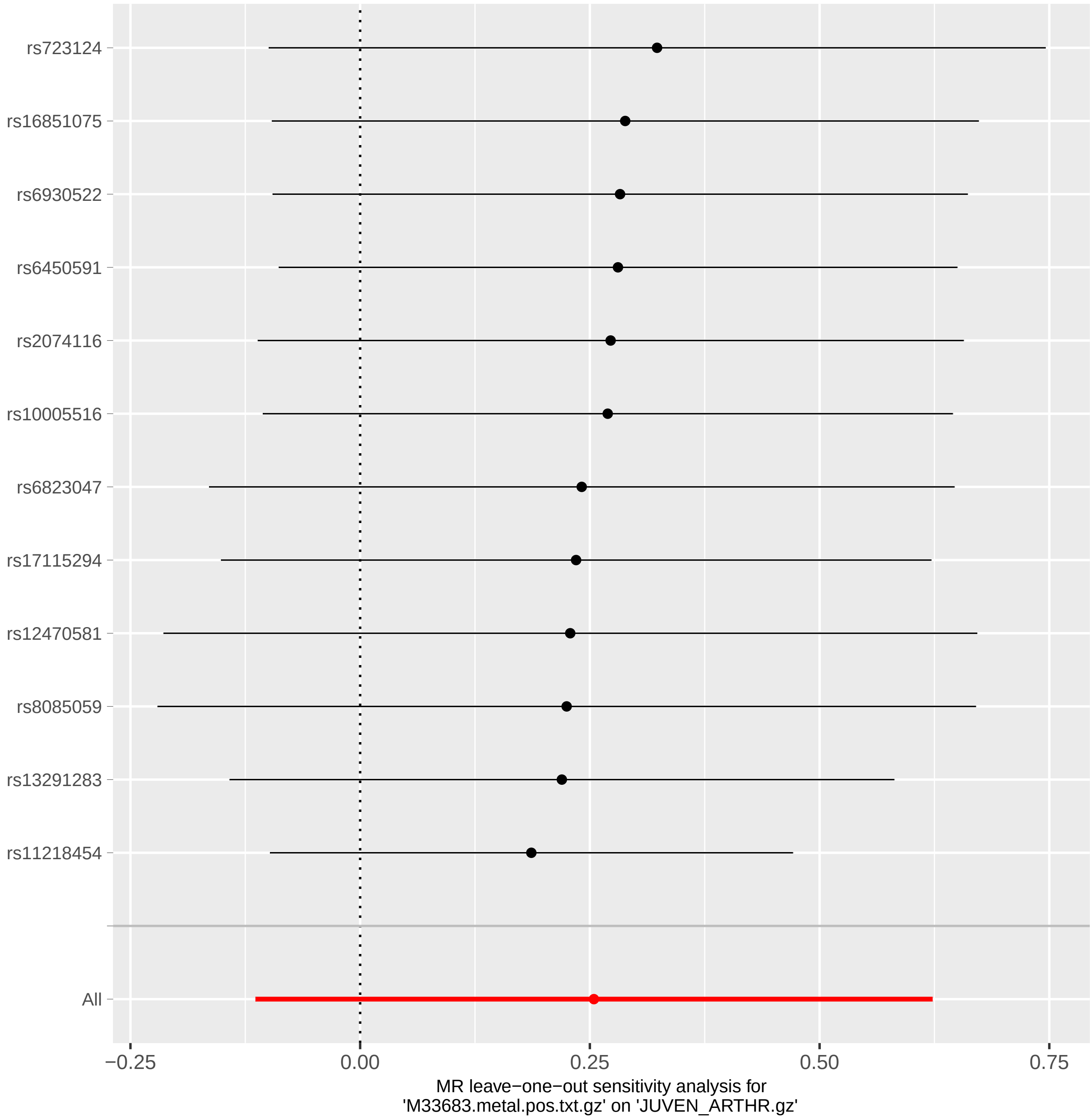

rs723124
rs16851075
rs6930522
rs6450591
rs2074116
rs10005516
rs6823047
rs17115294
rs12470581
rs8085059
rs13291283
rs11218454
All
−0.25
0.00
0.25
0.50
0.75
MR leave−one−out sensitivity analysis for
'M33683.metal.pos.txt.gz' on 'JUVEN_ARTHR.gz'

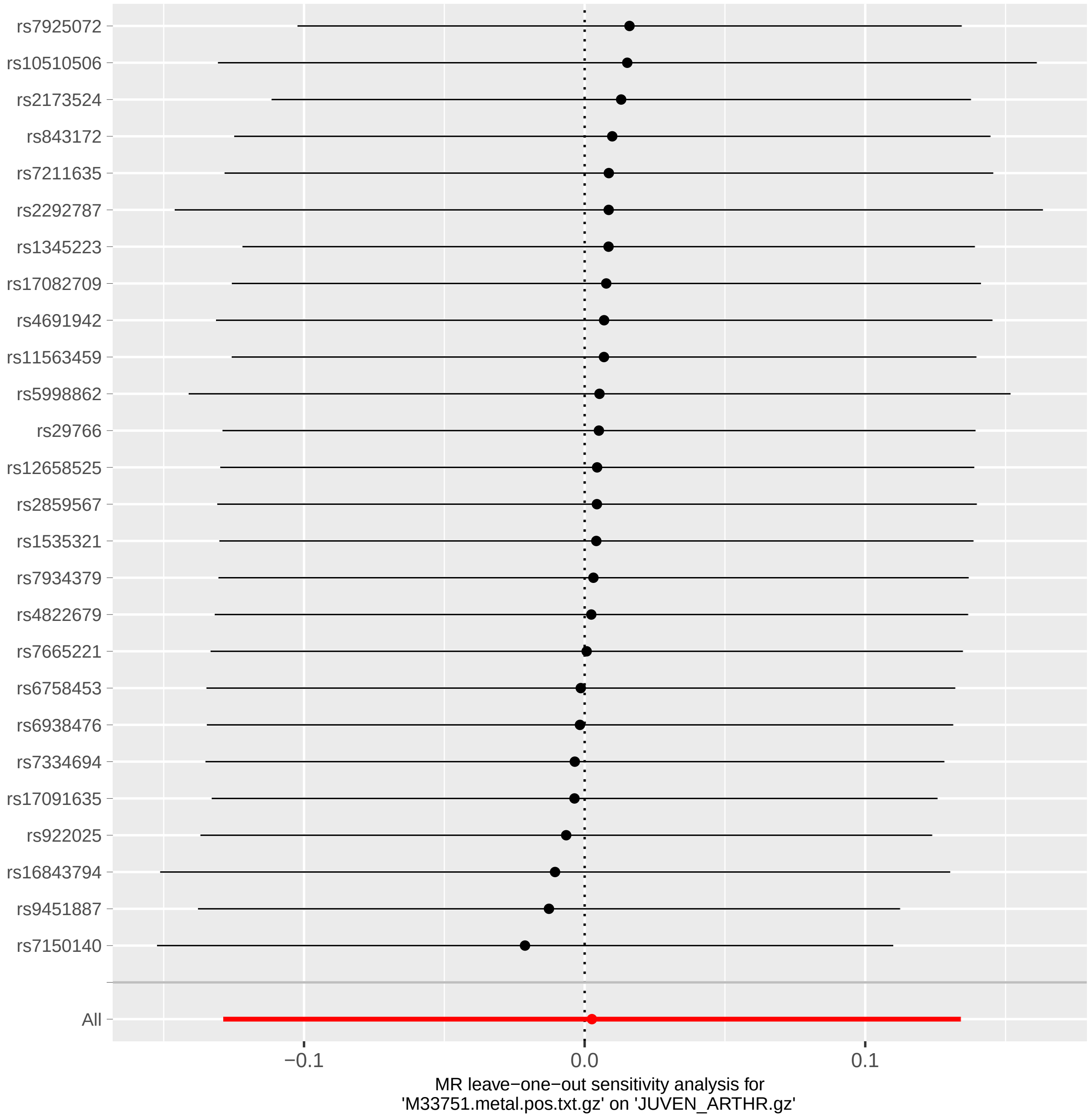
rs7925072
rs10510506
rs2173524
rs843172
rs7211635
rs2292787
rs1345223
rs17082709
rs4691942
rs11563459
rs5998862
rs29766
rs12658525
rs2859567
rs1535321
rs7934379
rs4822679
rs7665221
rs6758453
rs6938476
rs7334694
rs17091635
rs922025
rs16843794
rs9451887
rs7150140
All
−0.1
0.0
0.1
MR leave−one−out sensitivity analysis for
'M33751.metal.pos.txt.gz' on 'JUVEN_ARTHR.gz'

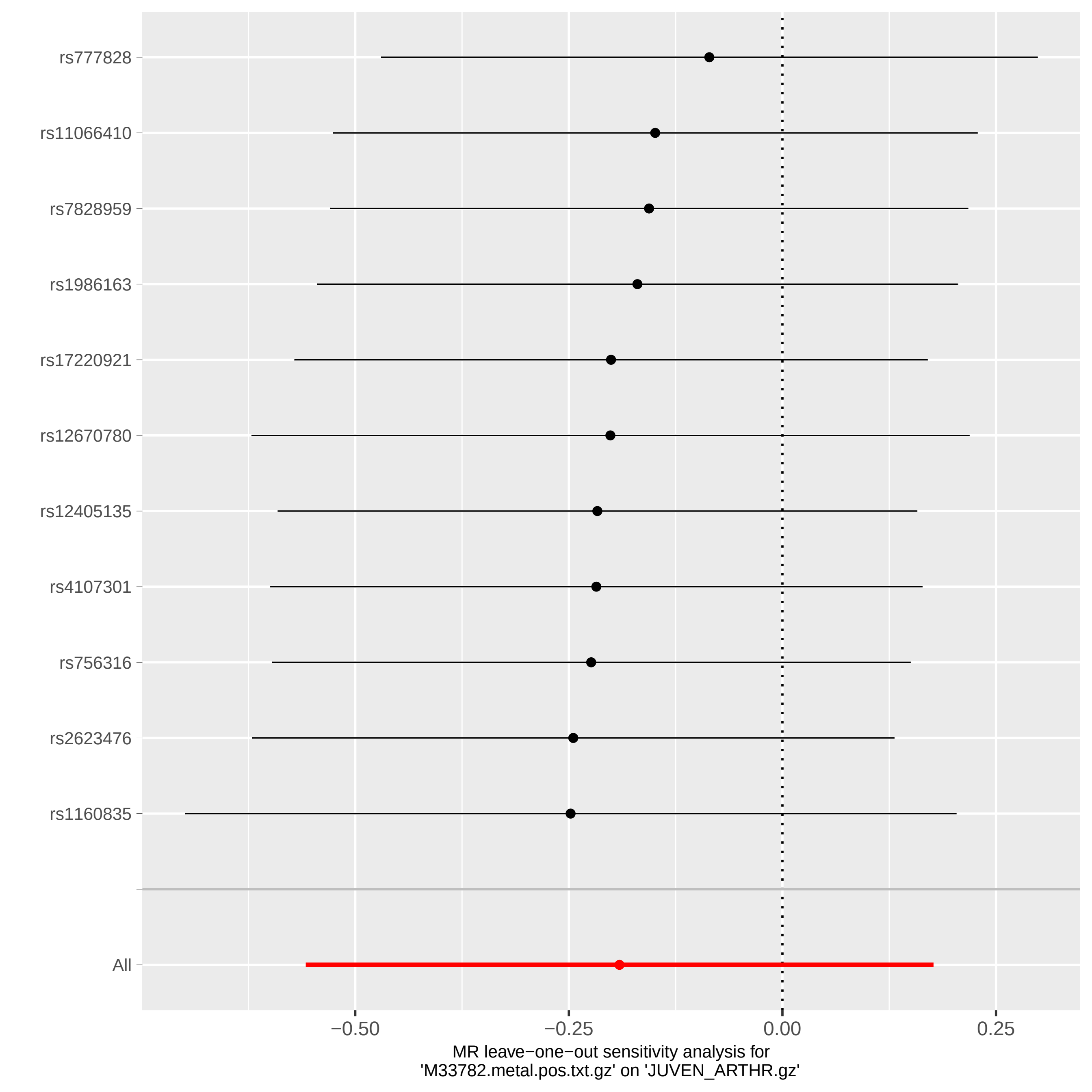

rs777828
rs11066410
rs7828959
rs1986163
rs17220921
rs12670780
rs12405135
rs4107301
rs756316
rs2623476
rs1160835
All
−0.50
−0.25
0.00
0.25
MR leave−one−out sensitivity analysis for
'M33782.metal.pos.txt.gz' on 'JUVEN_ARTHR.gz'

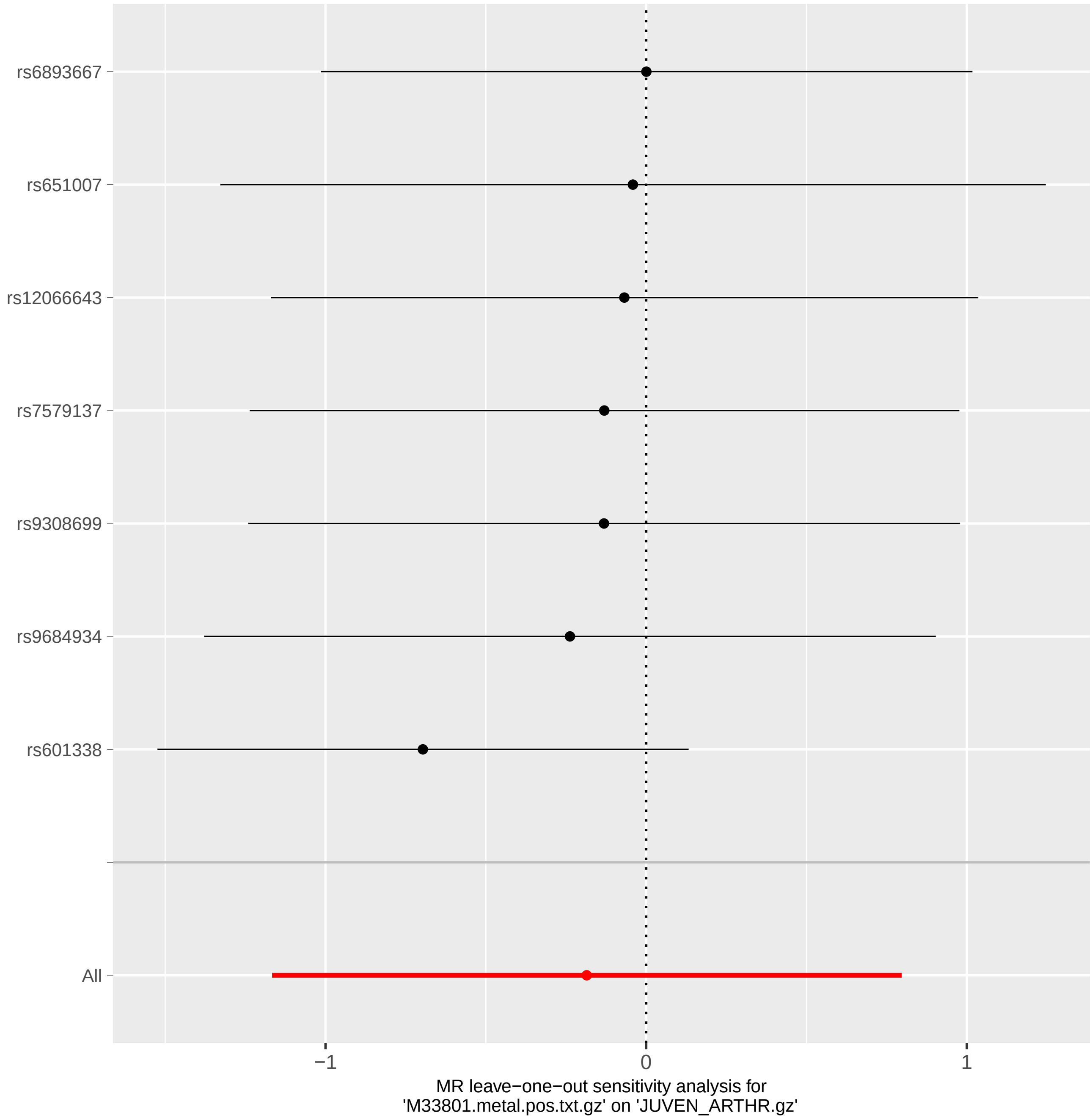
rs6893667
rs651007
rs12066643
rs7579137
rs9308699
rs9684934
rs601338
All
−1
0
1
MR leave−one−out sensitivity analysis for
'M33801.metal.pos.txt.gz' on 'JUVEN_ARTHR.gz'

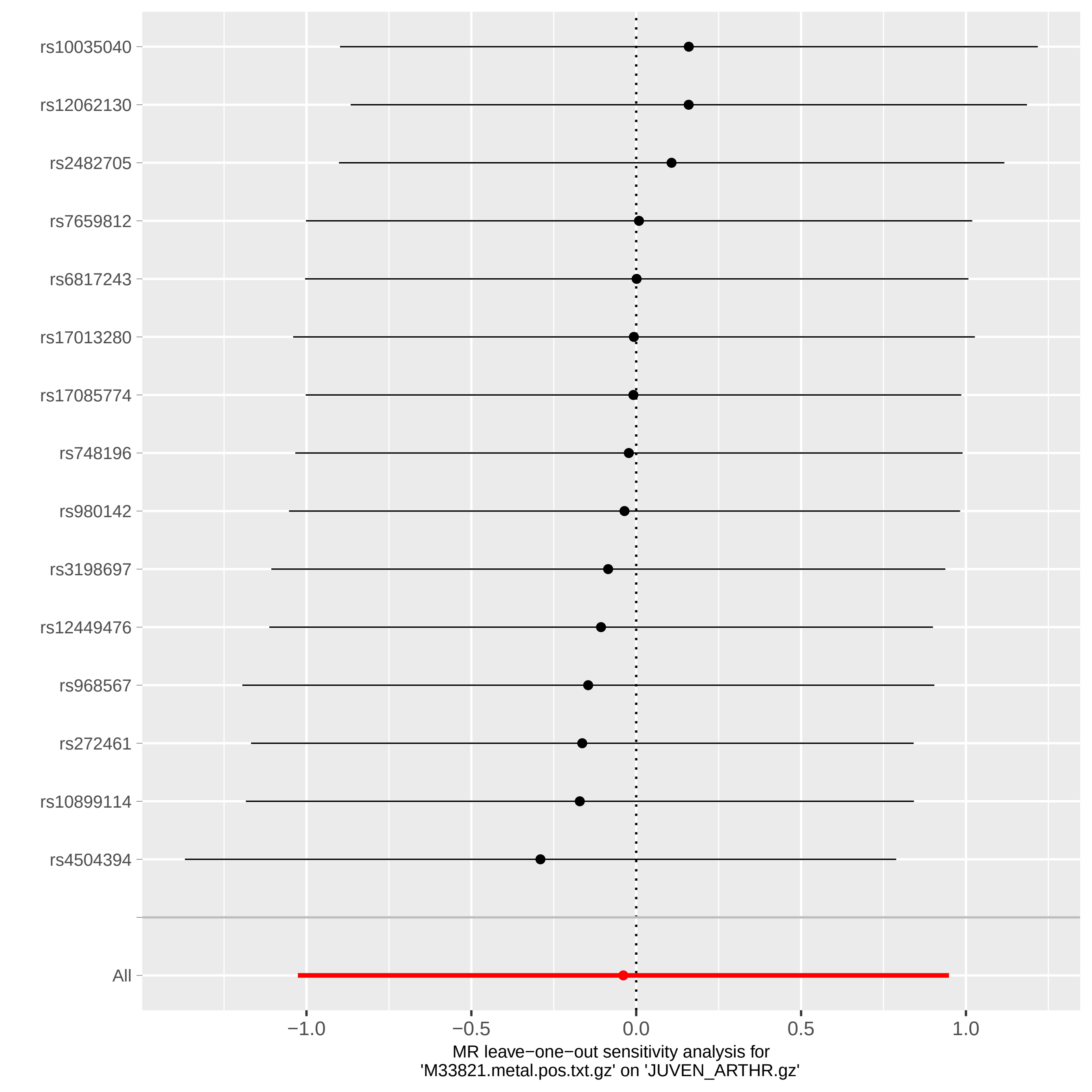
rs10035040
rs12062130
rs2482705
rs7659812
rs6817243
rs17013280
rs17085774
rs748196
rs980142
rs3198697
rs12449476
rs968567
rs272461
rs10899114
rs4504394
All
−1.0
−0.5
0.0
0.5
1.0
MR leave−one−out sensitivity analysis for
'M33821.metal.pos.txt.gz' on 'JUVEN_ARTHR.gz'

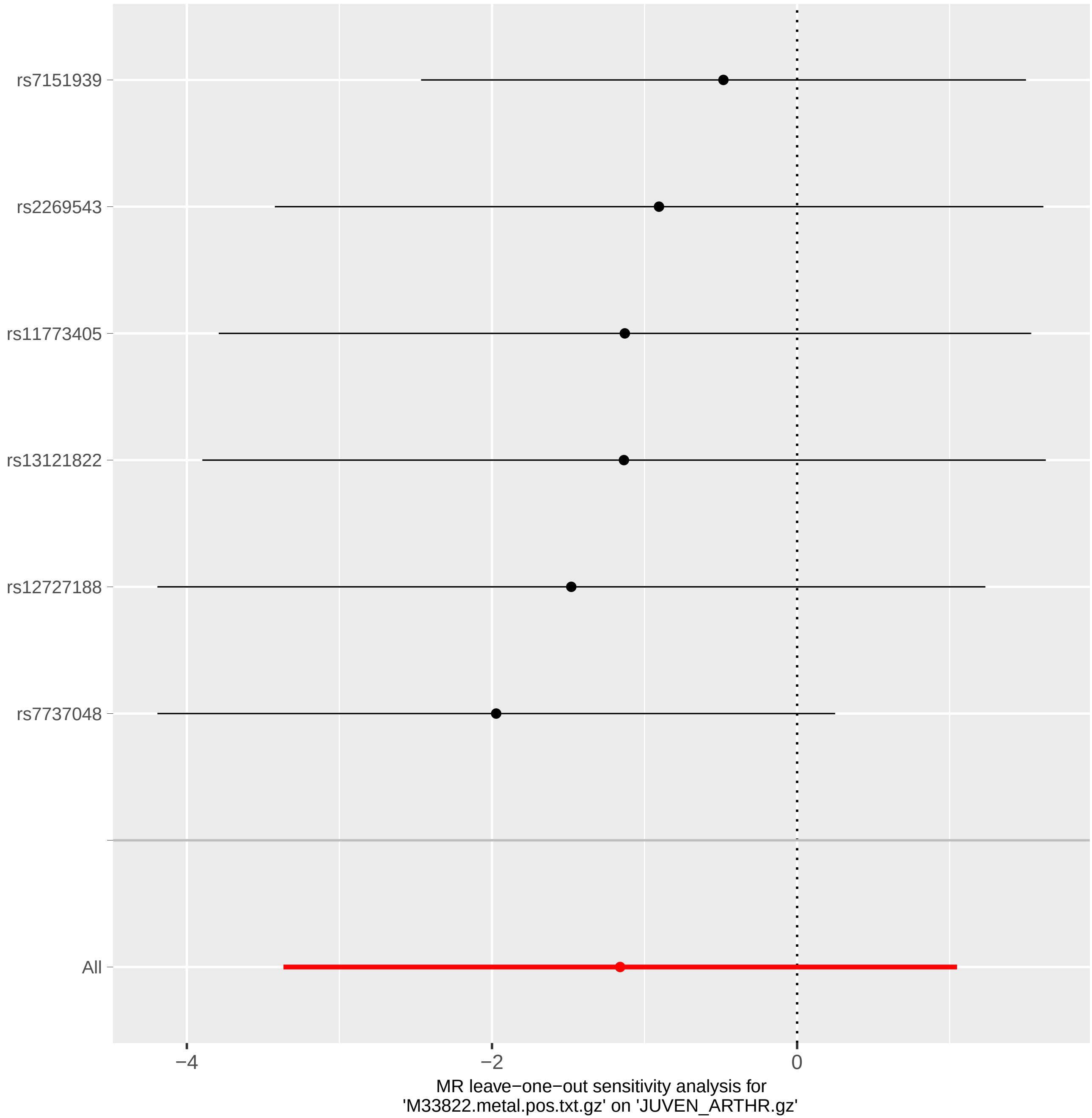

rs7151939
rs2269543
rs11773405
rs13121822
rs12727188
rs7737048
All
−4
−2
0
MR leave−one−out sensitivity analysis for
'M33822.metal.pos.txt.gz' on 'JUVEN_ARTHR.gz'

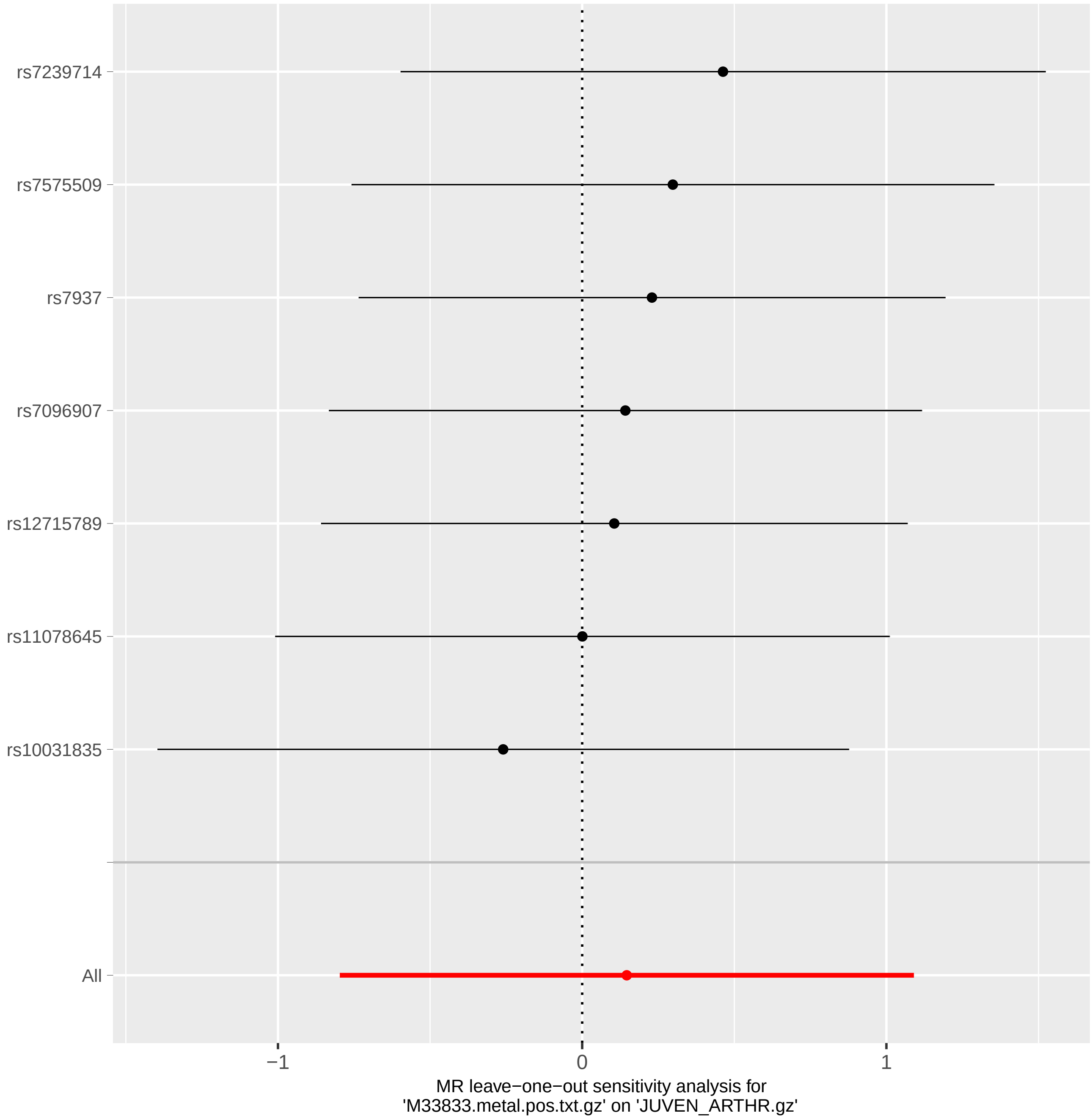
rs7239714
rs7575509
rs7937
rs7096907
rs12715789
rs11078645
rs10031835
All
−1
0
1
MR leave−one−out sensitivity analysis for
'M33833.metal.pos.txt.gz' on 'JUVEN_ARTHR.gz'

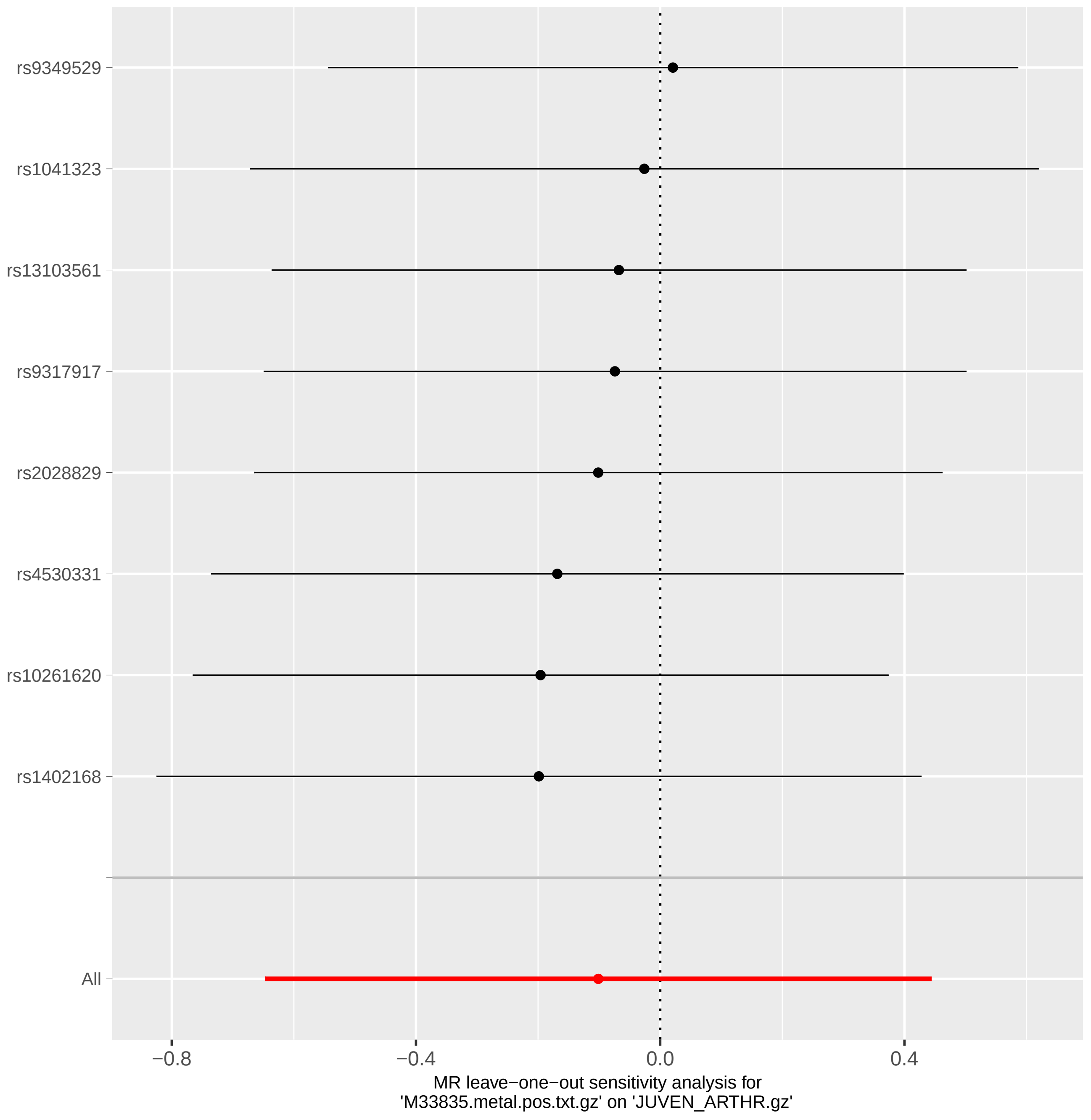

rs9349529
rs1041323
rs13103561
rs9317917
rs2028829
rs4530331
rs10261620
rs1402168
All
−0.8
−0.4
0.0
0.4
MR leave−one−out sensitivity analysis for
'M33835.metal.pos.txt.gz' on 'JUVEN_ARTHR.gz'

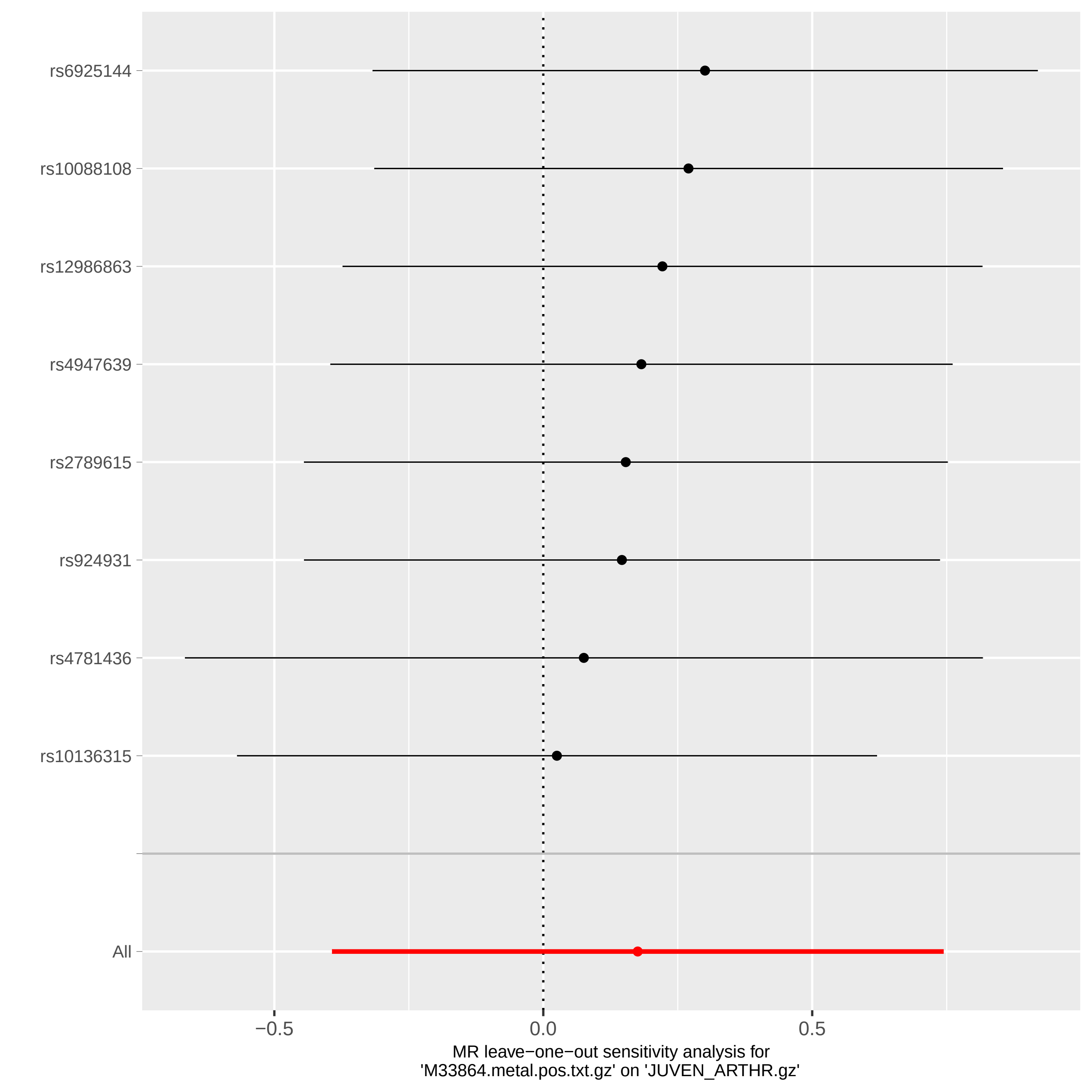
rs6925144
rs10088108
rs12986863
rs4947639
rs2789615
rs924931
rs4781436
rs10136315
All
−0.5
0.0
0.5
MR leave−one−out sensitivity analysis for
'M33864.metal.pos.txt.gz' on 'JUVEN_ARTHR.gz'

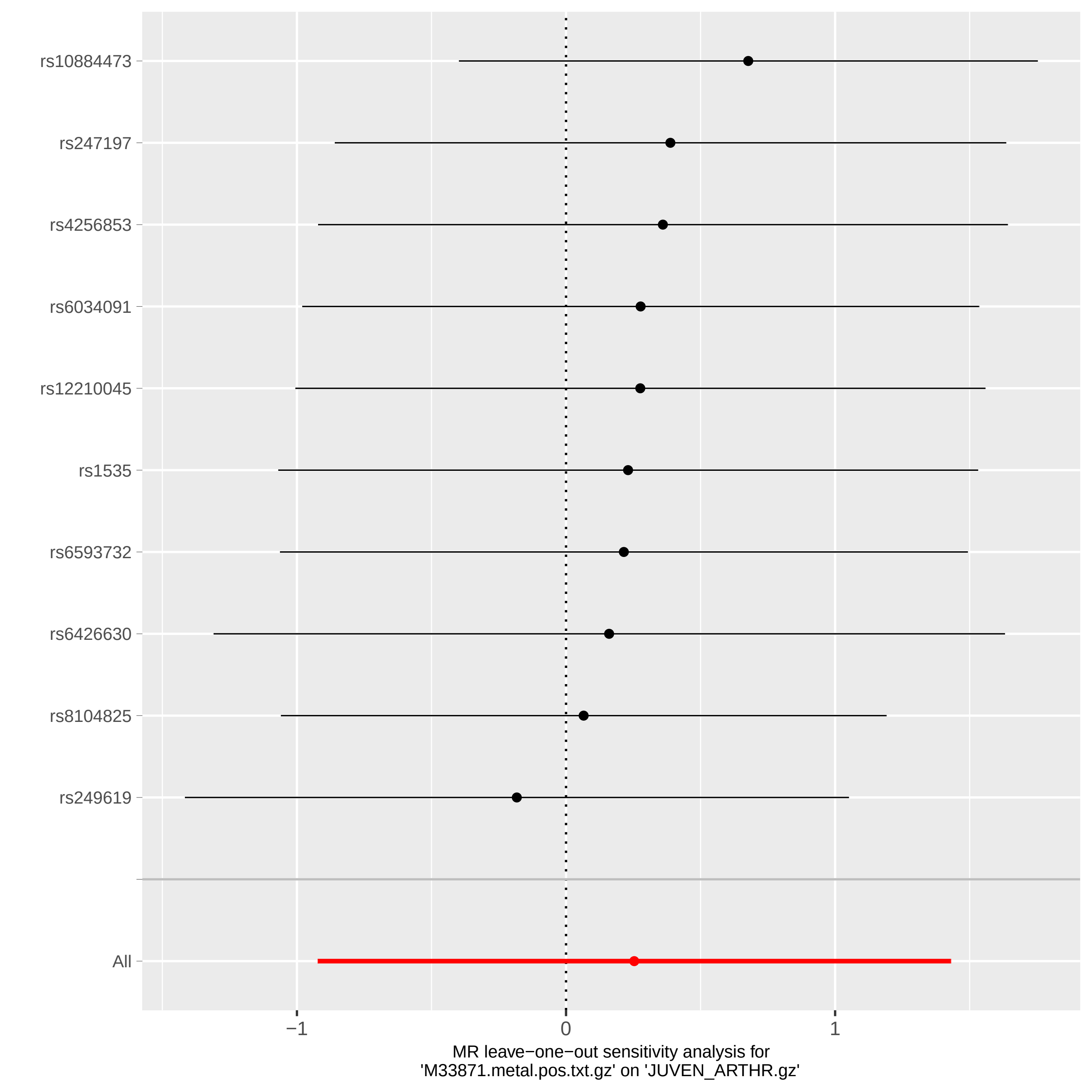
rs10884473
rs247197
rs4256853
rs6034091
rs12210045
rs1535
rs6593732
rs6426630
rs8104825
rs249619
All
−1
0
1
MR leave−one−out sensitivity analysis for
'M33871.metal.pos.txt.gz' on 'JUVEN_ARTHR.gz'

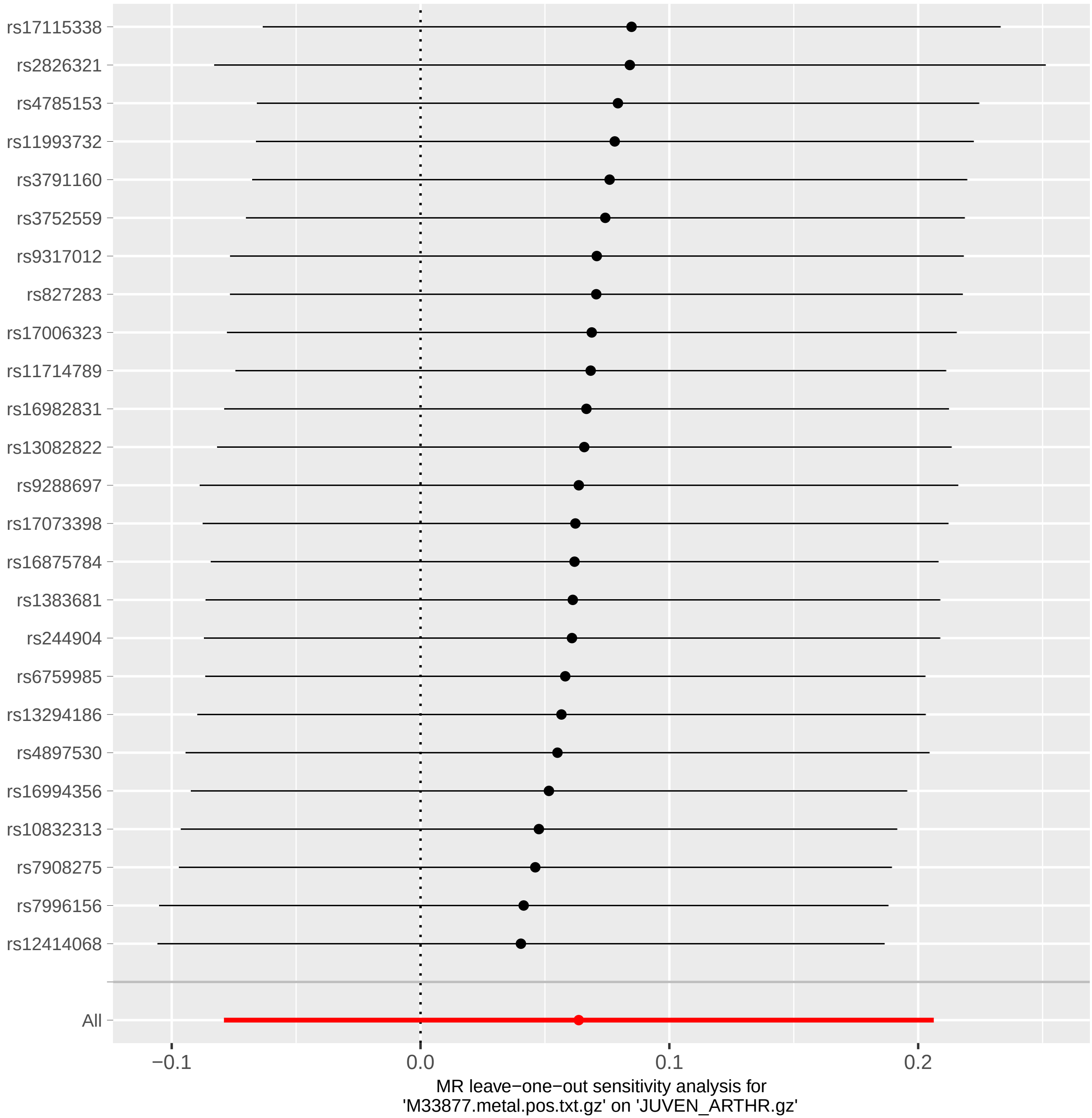
rs17115338
rs2826321
rs4785153
rs11993732
rs3791160
rs3752559
rs9317012
rs827283
rs17006323
rs11714789
rs16982831
rs13082822
rs9288697
rs17073398
rs16875784
rs1383681
rs244904
rs6759985
rs13294186
rs4897530
rs16994356
rs10832313
rs7908275
rs7996156
rs12414068
All
−0.1
0.0
0.1
0.2
MR leave−one−out sensitivity analysis for
'M33877.metal.pos.txt.gz' on 'JUVEN_ARTHR.gz'

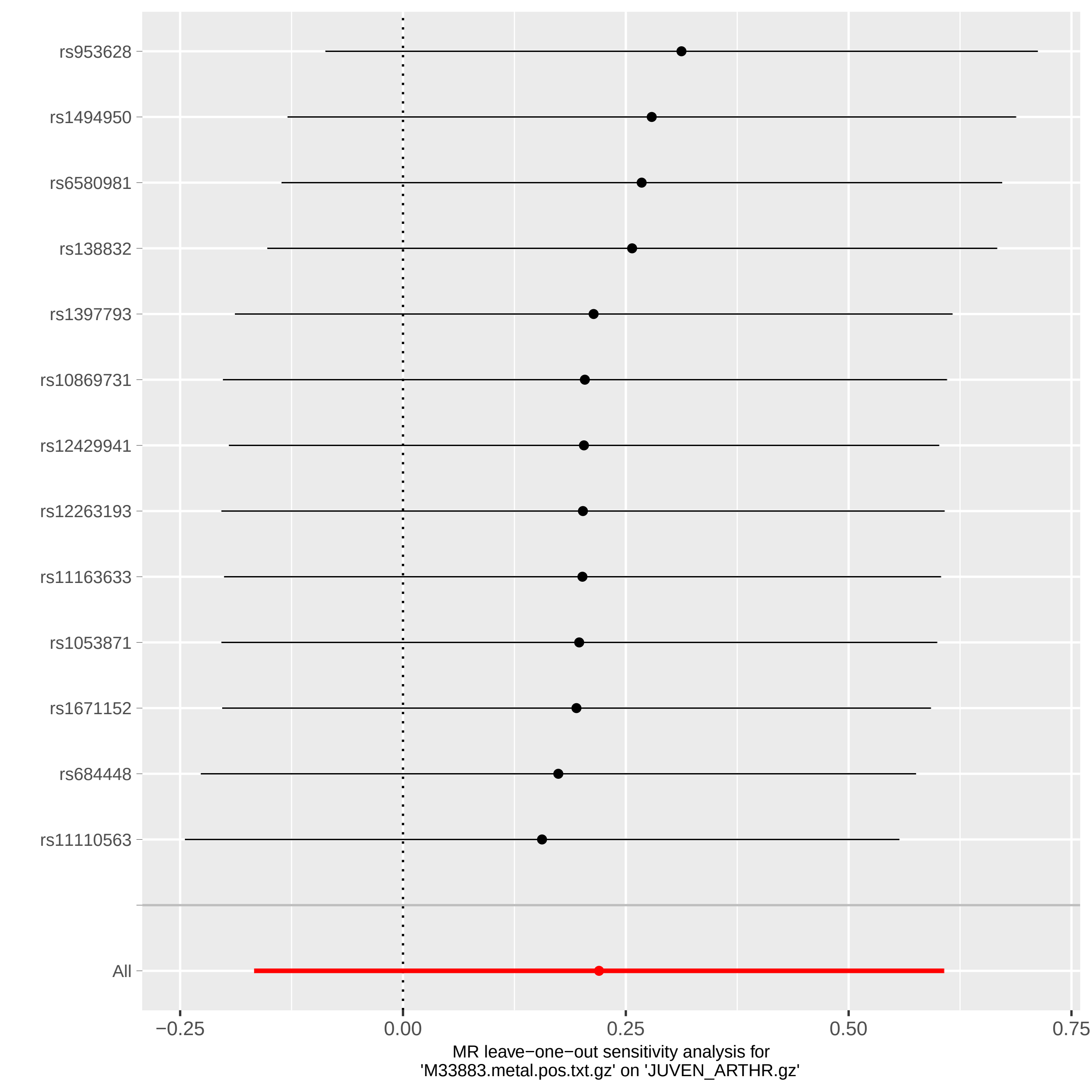
rs953628
rs1494950
rs6580981
rs138832
rs1397793
rs10869731
rs12429941
rs12263193
rs11163633
rs1053871
rs1671152
rs684448
rs11110563
All
−0.25
0.00
0.25
0.50
0.75
MR leave−one−out sensitivity analysis for
'M33883.metal.pos.txt.gz' on 'JUVEN_ARTHR.gz'

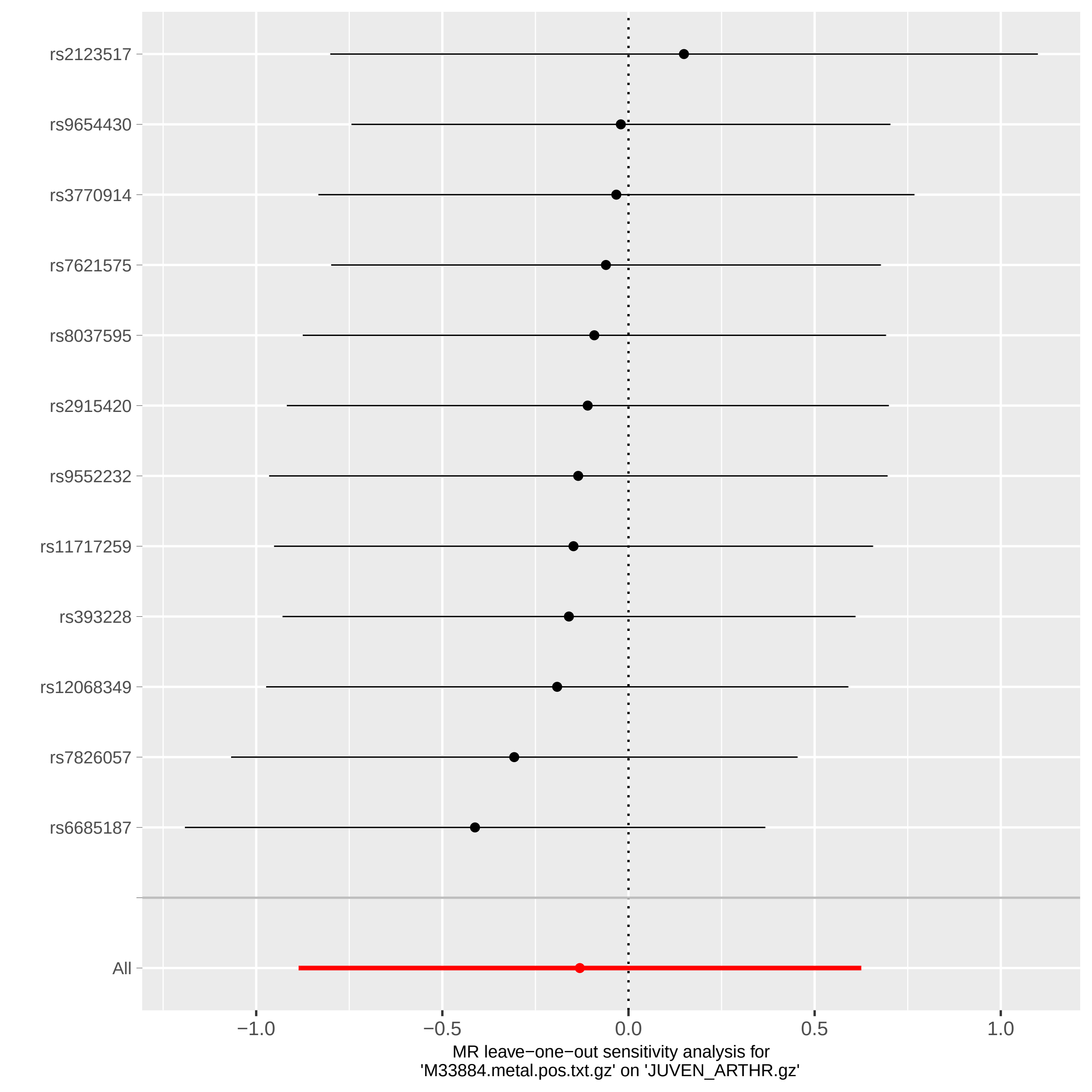
rs2123517
rs9654430
rs3770914
rs7621575
rs8037595
rs2915420
rs9552232
rs11717259
rs393228
rs12068349
rs7826057
rs6685187
All
−1.0
−0.5
0.0
0.5
1.0
MR leave−one−out sensitivity analysis for
'M33884.metal.pos.txt.gz' on 'JUVEN_ARTHR.gz'

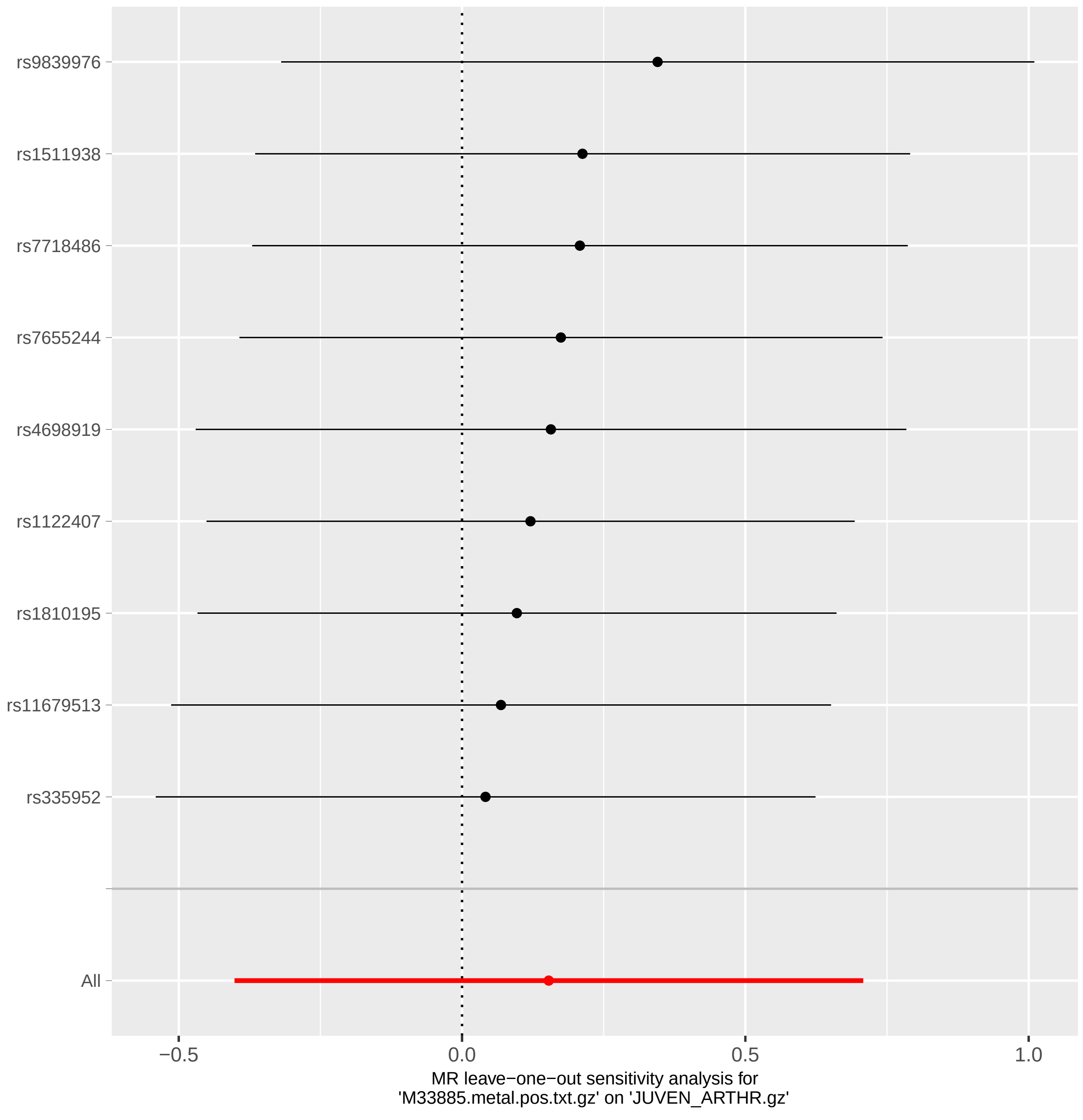
rs9839976
rs1511938
rs7718486
rs7655244
rs4698919
rs1122407
rs1810195
rs11679513
rs335952
All
−0.5
0.0
0.5
1.0
MR leave−one−out sensitivity analysis for
'M33885.metal.pos.txt.gz' on 'JUVEN_ARTHR.gz'

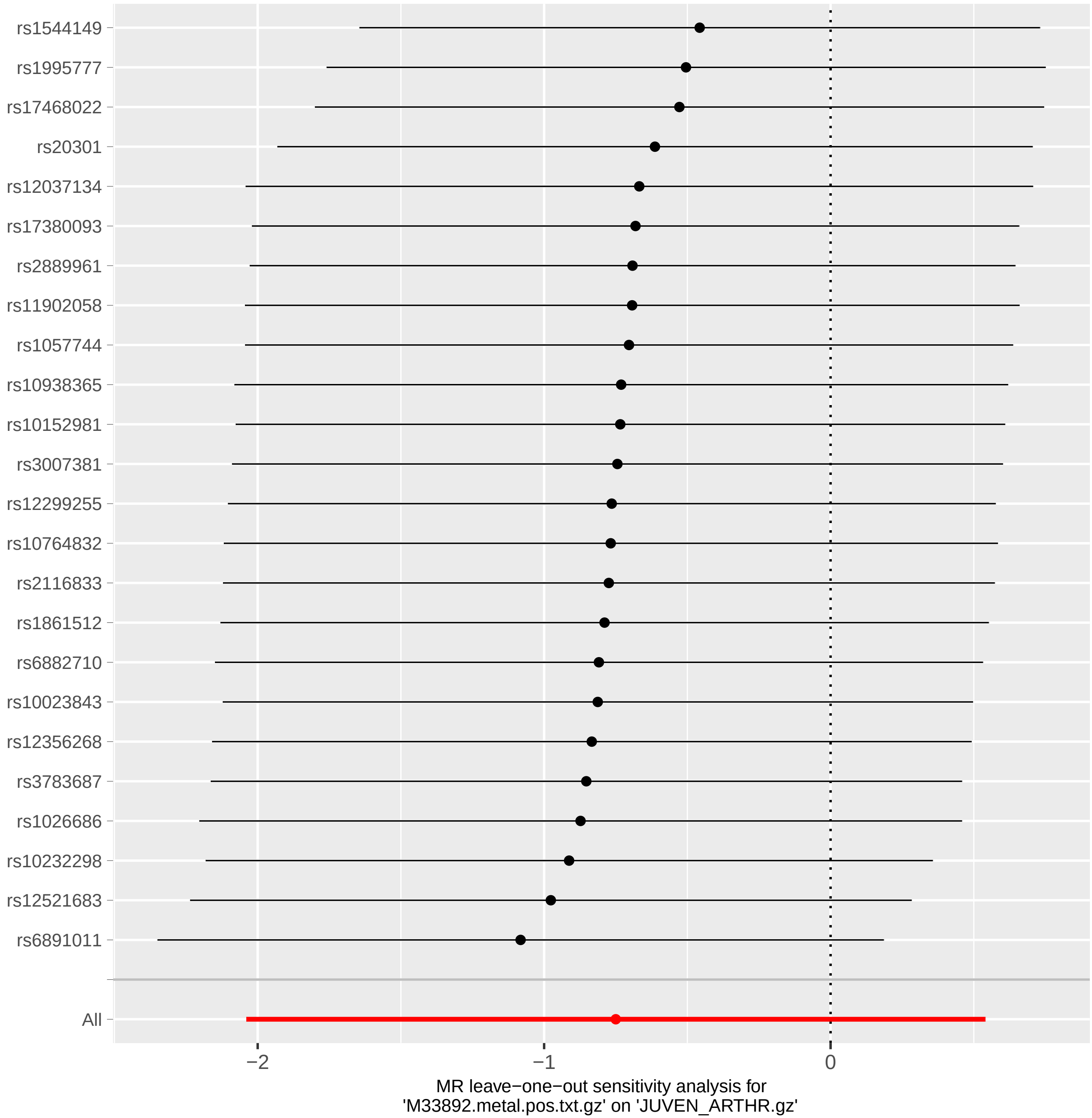
rs1544149
rs1995777
rs17468022
rs20301
rs12037134
rs17380093
rs2889961
rs11902058
rs1057744
rs10938365
rs10152981
rs3007381
rs12299255
rs10764832
rs2116833
rs1861512
rs6882710
rs10023843
rs12356268
rs3783687
rs1026686
rs10232298
rs12521683
rs6891011
All
−2
−1
0
MR leave−one−out sensitivity analysis for
'M33892.metal.pos.txt.gz' on 'JUVEN_ARTHR.gz'

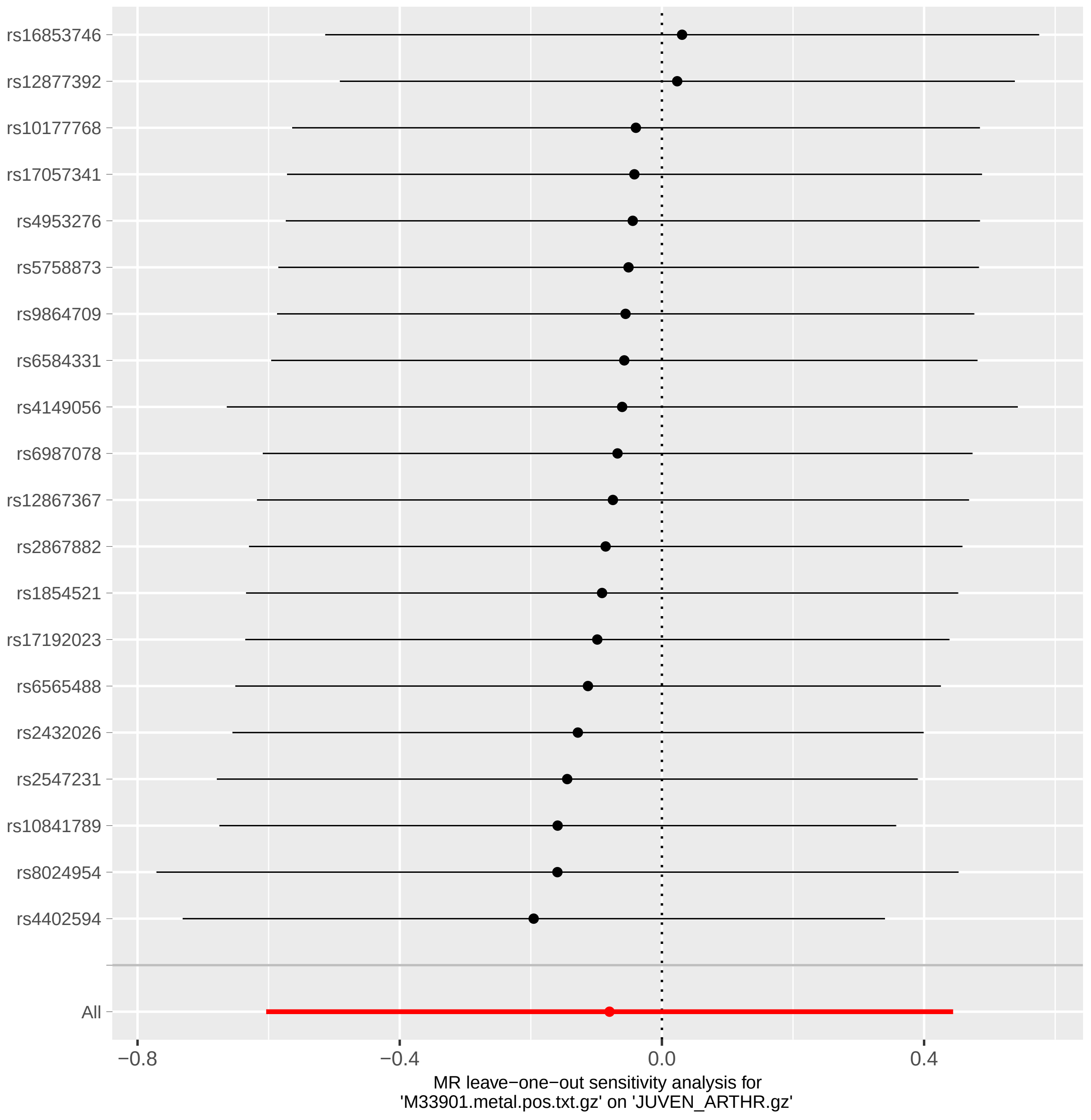
rs16853746
rs12877392
rs10177768
rs17057341
rs4953276
rs5758873
rs9864709
rs6584331
rs4149056
rs6987078
rs12867367
rs2867882
rs1854521
rs17192023
rs6565488
rs2432026
rs2547231
rs10841789
rs8024954
rs4402594
All
−0.8
−0.4
0.0
0.4
MR leave−one−out sensitivity analysis for
'M33901.metal.pos.txt.gz' on 'JUVEN_ARTHR.gz'

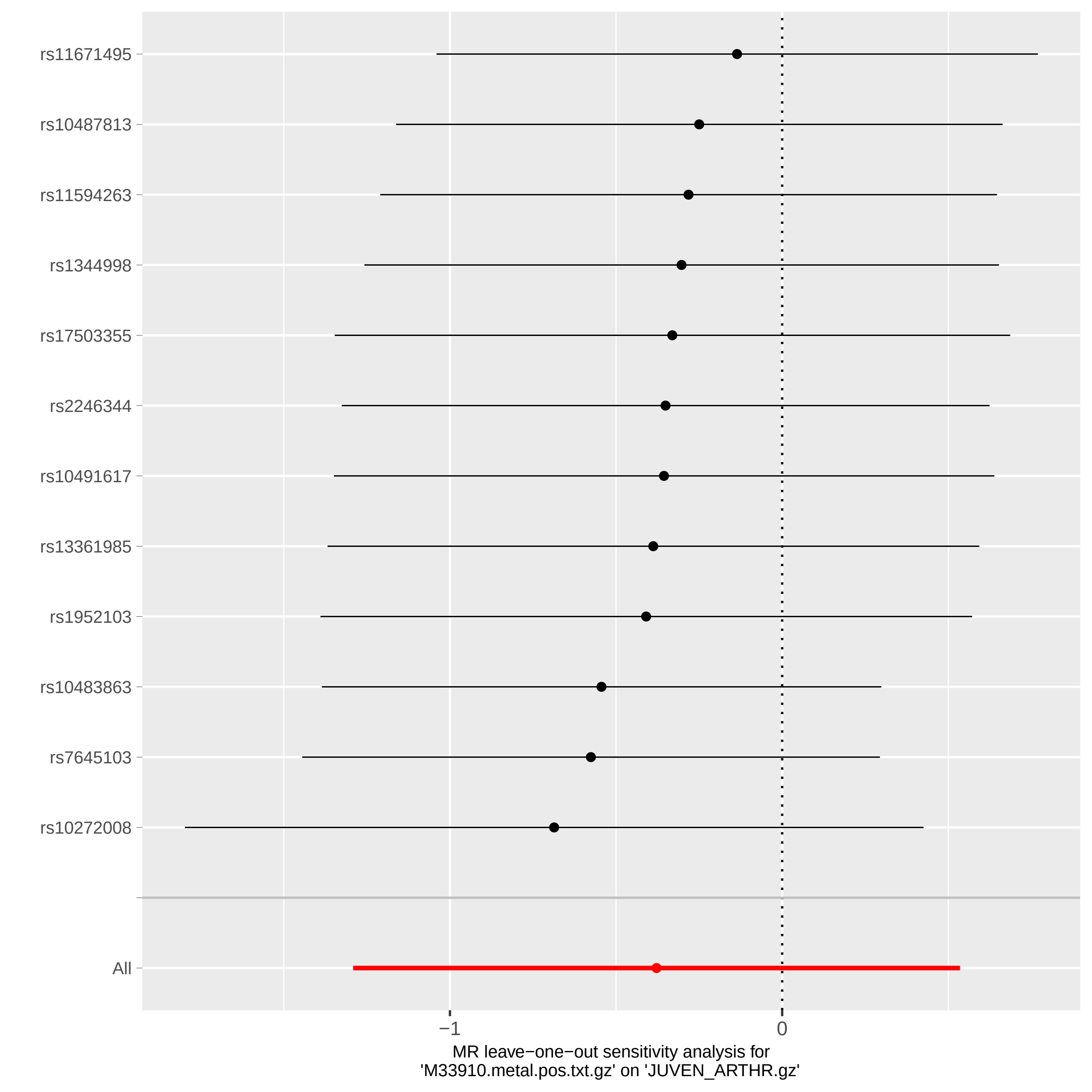

rs11671495
rs10487813
rs11594263
rs1344998
rs17503355
rs2246344
rs10491617
rs13361985
rs1952103
rs10483863
rs7645103
rs10272008
All
−1
0
MR leave−one−out sensitivity analysis for
'M33910.metal.pos.txt.gz' on 'JUVEN_ARTHR.gz'

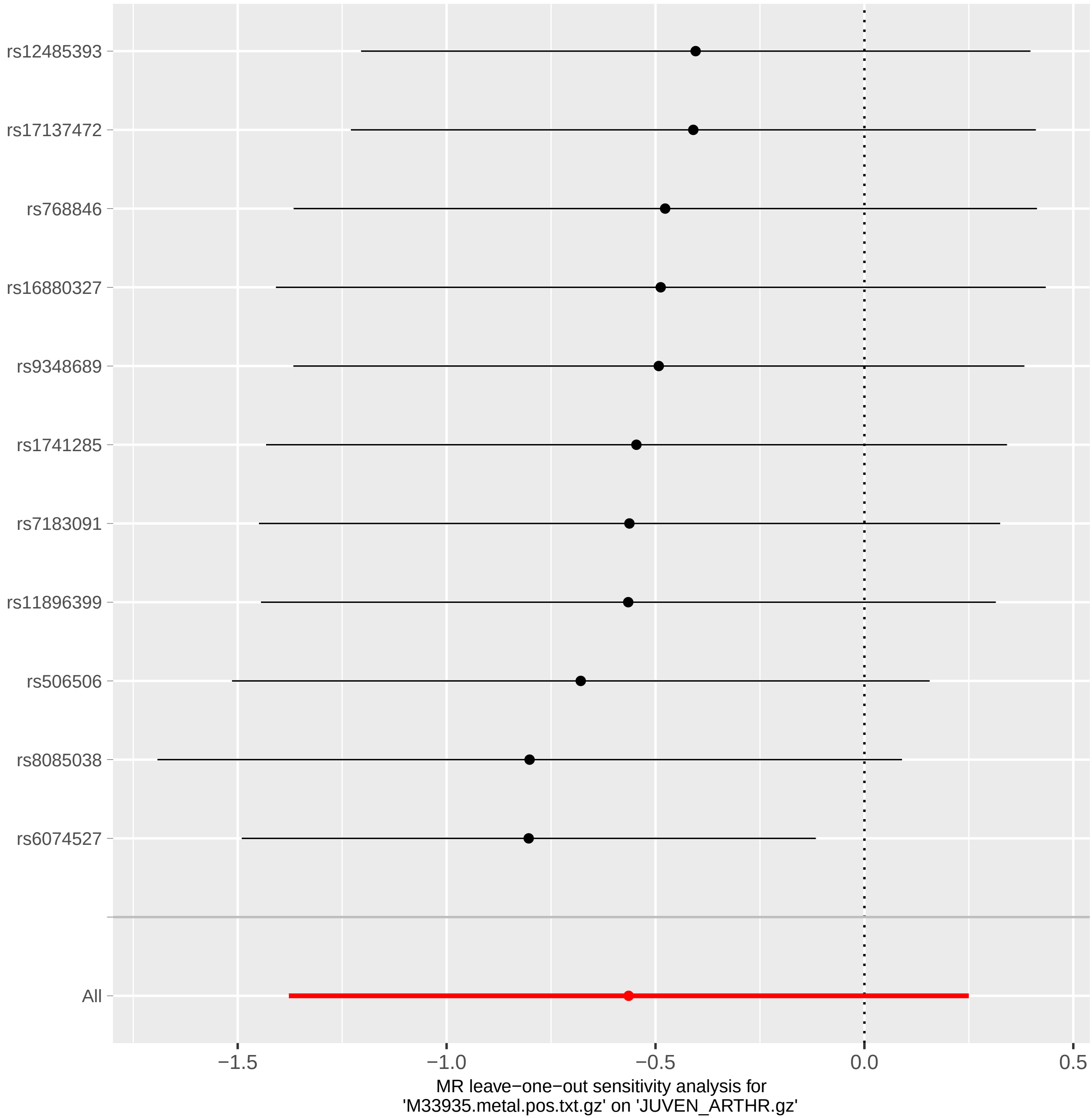

rs12485393
rs17137472
rs768846
rs16880327
rs9348689
rs1741285
rs7183091
rs11896399
rs506506
rs8085038
rs6074527
All
−1.5
−1.0
−0.5
0.0
0.5
MR leave−one−out sensitivity analysis for
'M33935.metal.pos.txt.gz' on 'JUVEN_ARTHR.gz'

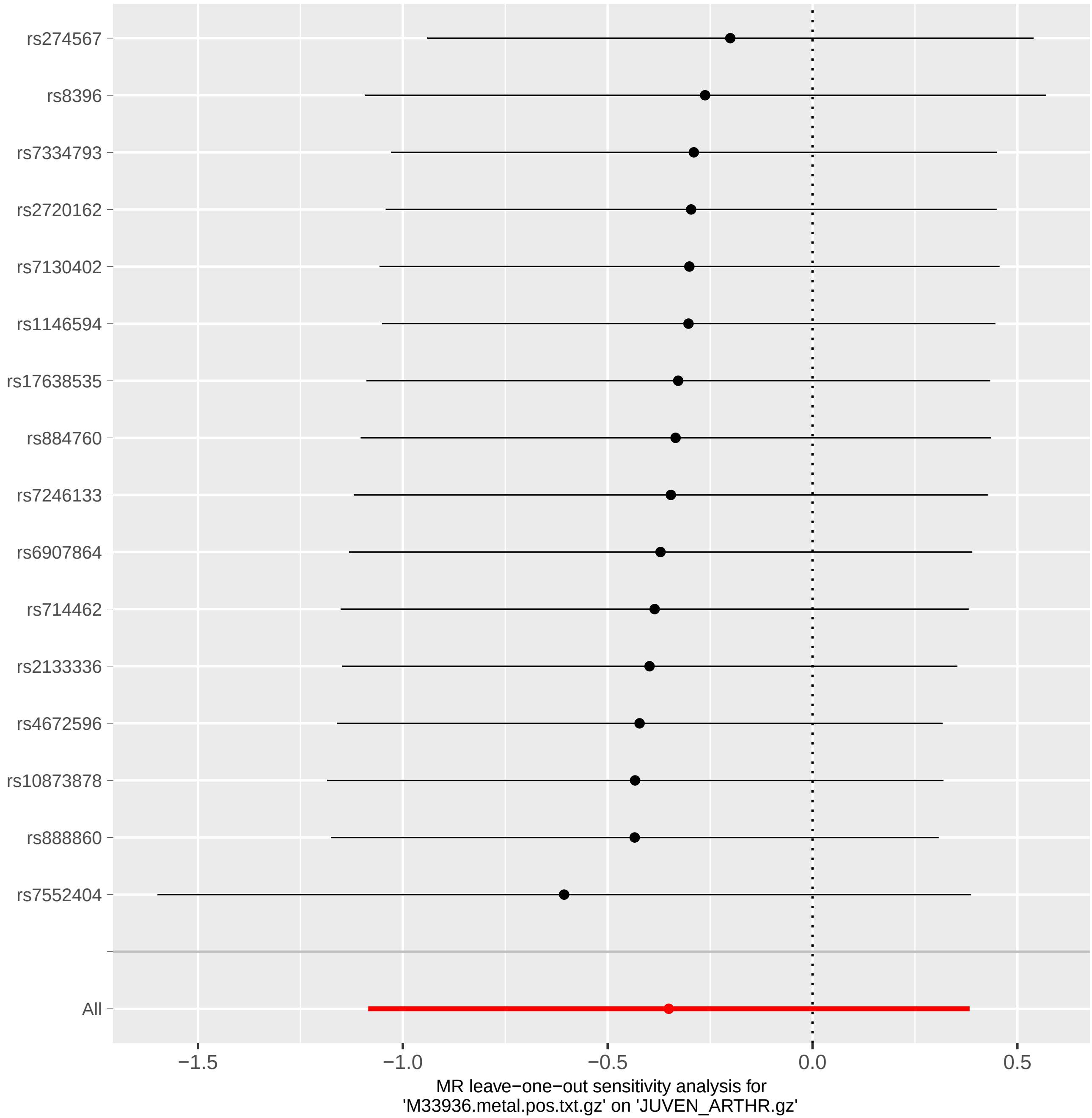

rs274567
rs8396
rs7334793
rs2720162
rs7130402
rs1146594
rs17638535
rs884760
rs7246133
rs6907864
rs714462
rs2133336
rs4672596
rs10873878
rs888860
rs7552404
All
−1.5
−1.0
−0.5
0.0
0.5
MR leave−one−out sensitivity analysis for
'M33936.metal.pos.txt.gz' on 'JUVEN_ARTHR.gz'

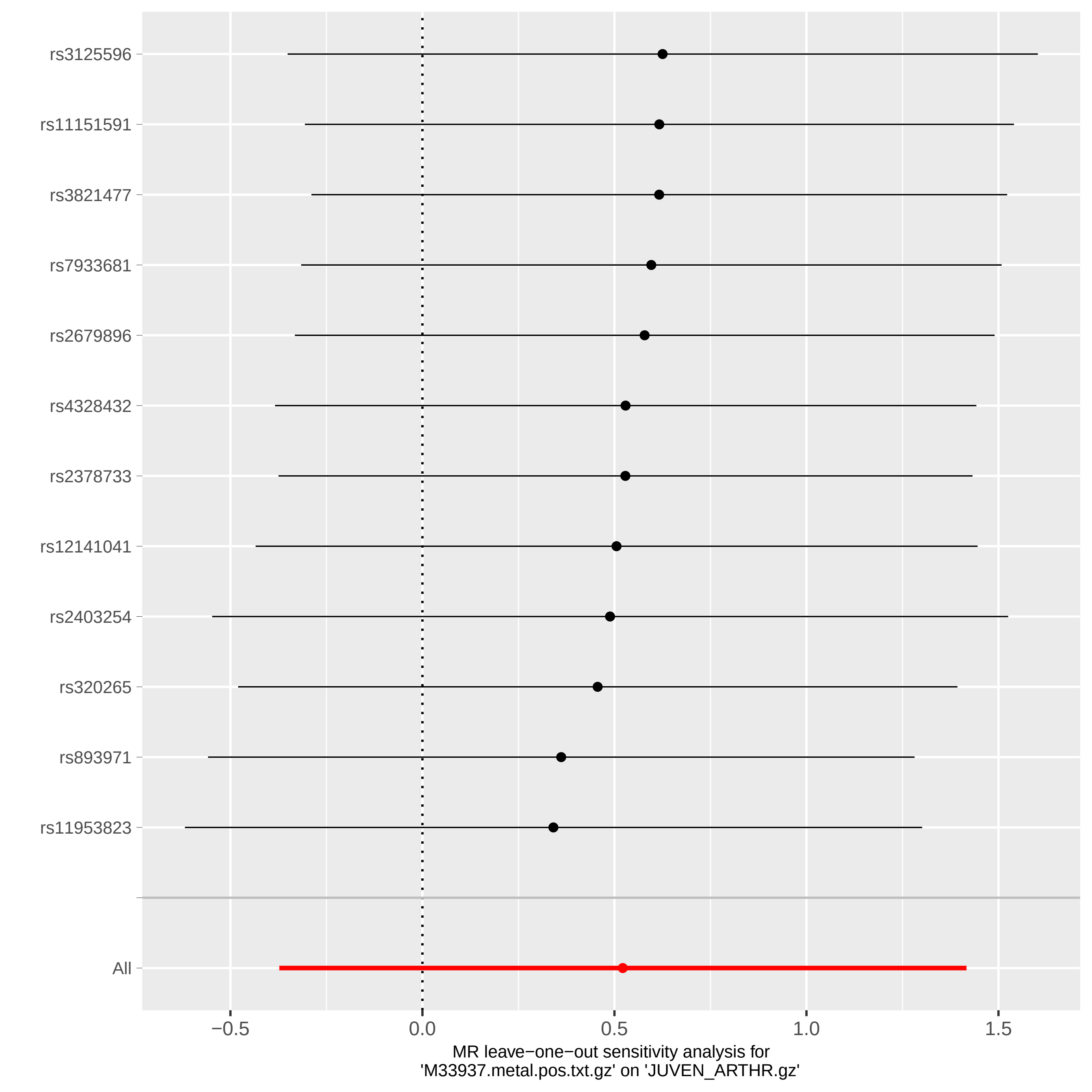

rs3125596
rs11151591
rs3821477
rs7933681
rs2679896
rs4328432
rs2378733
rs12141041
rs2403254
rs320265
rs893971
rs11953823
All
−0.5
0.0
0.5
1.0
1.5
MR leave−one−out sensitivity analysis for
'M33937.metal.pos.txt.gz' on 'JUVEN_ARTHR.gz'

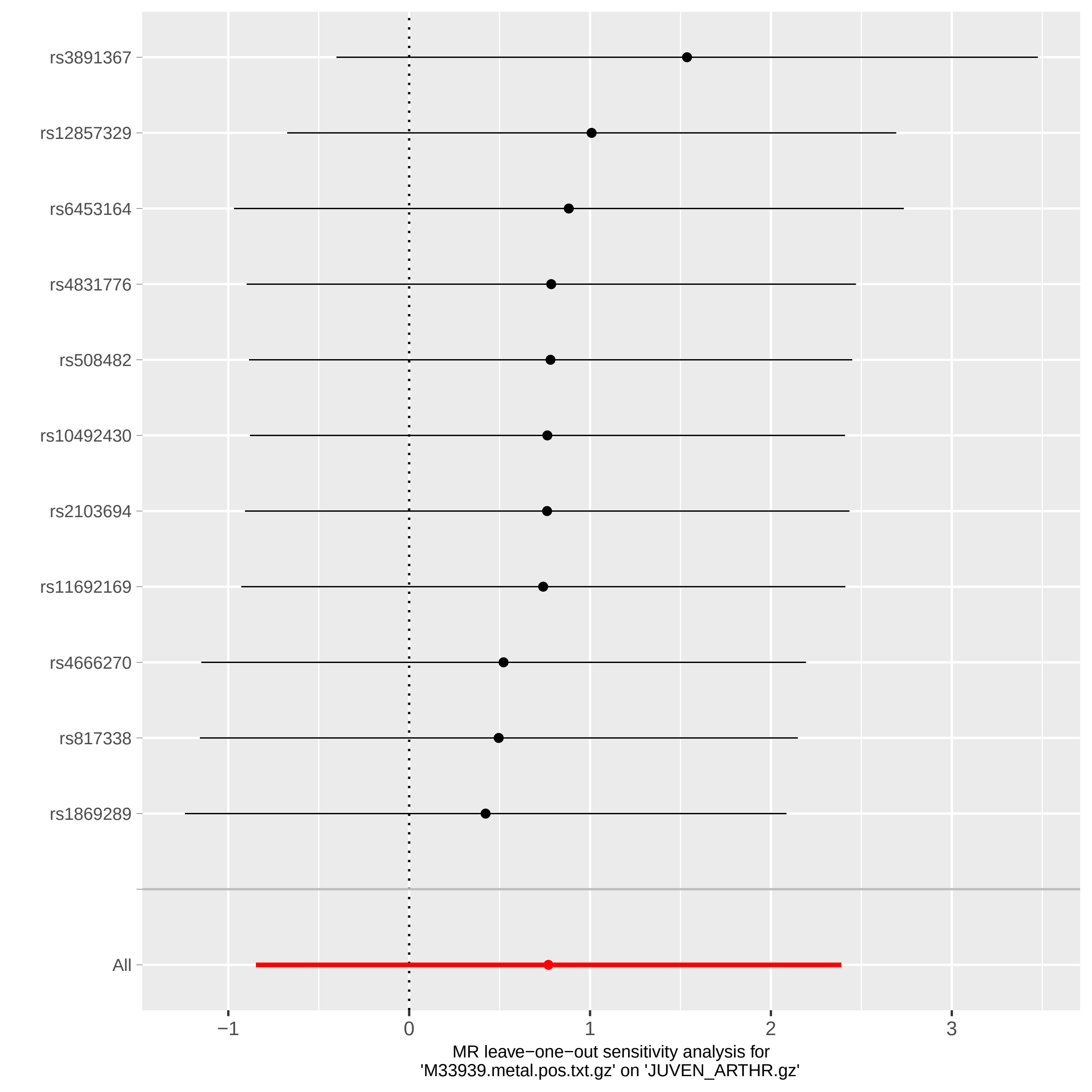

rs3891367
rs12857329
rs6453164
rs4831776
rs508482
rs10492430
rs2103694
rs11692169
rs4666270
rs817338
rs1869289
All
−1
0
1
2
3
MR leave−one−out sensitivity analysis for
'M33939.metal.pos.txt.gz' on 'JUVEN_ARTHR.gz'

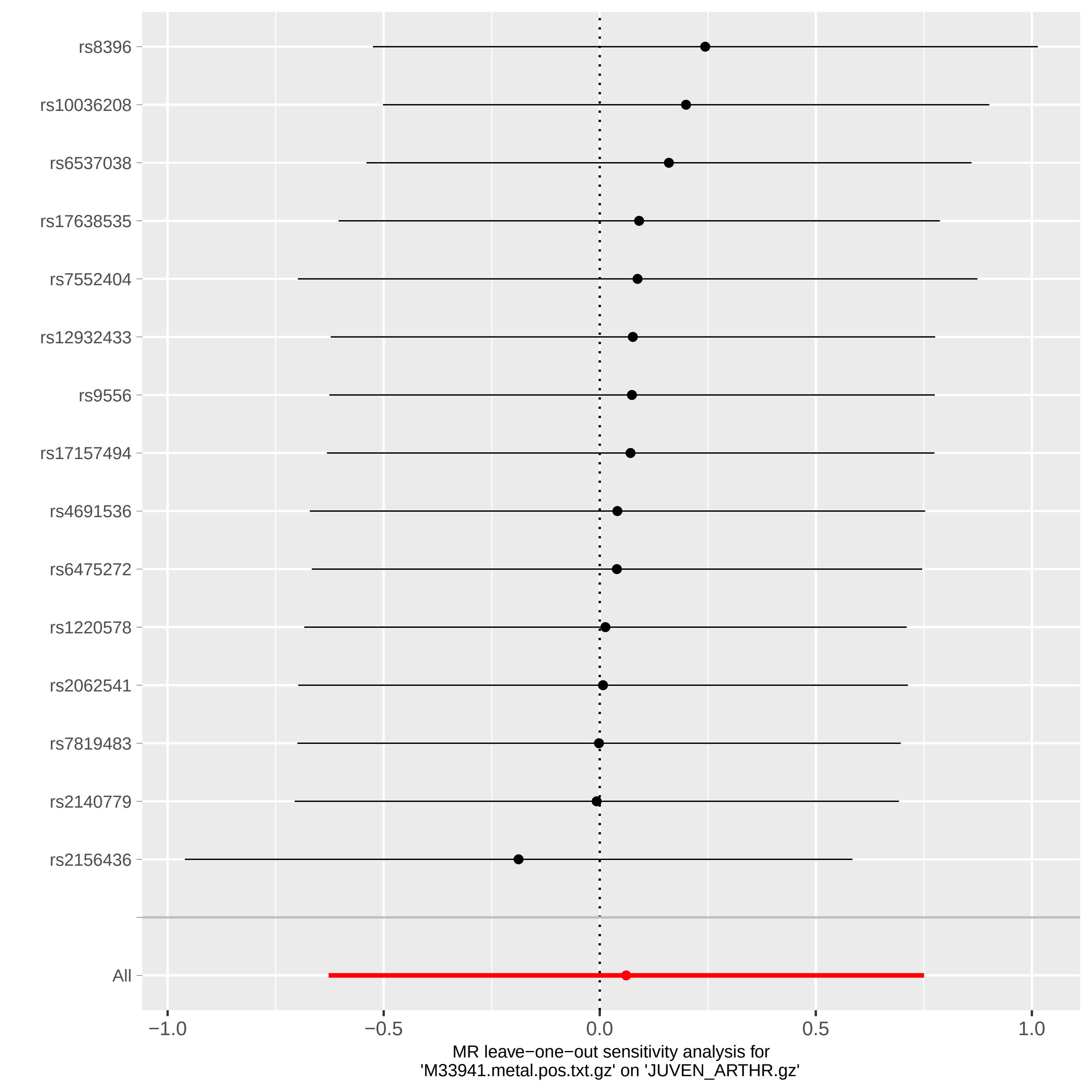
rs8396
rs10036208
rs6537038
rs17638535
rs7552404
rs12932433
rs9556
rs17157494
rs4691536
rs6475272
rs1220578
rs2062541
rs7819483
rs2140779
rs2156436
All
−1.0
−0.5
0.0
0.5
1.0
MR leave−one−out sensitivity analysis for
'M33941.metal.pos.txt.gz' on 'JUVEN_ARTHR.gz'

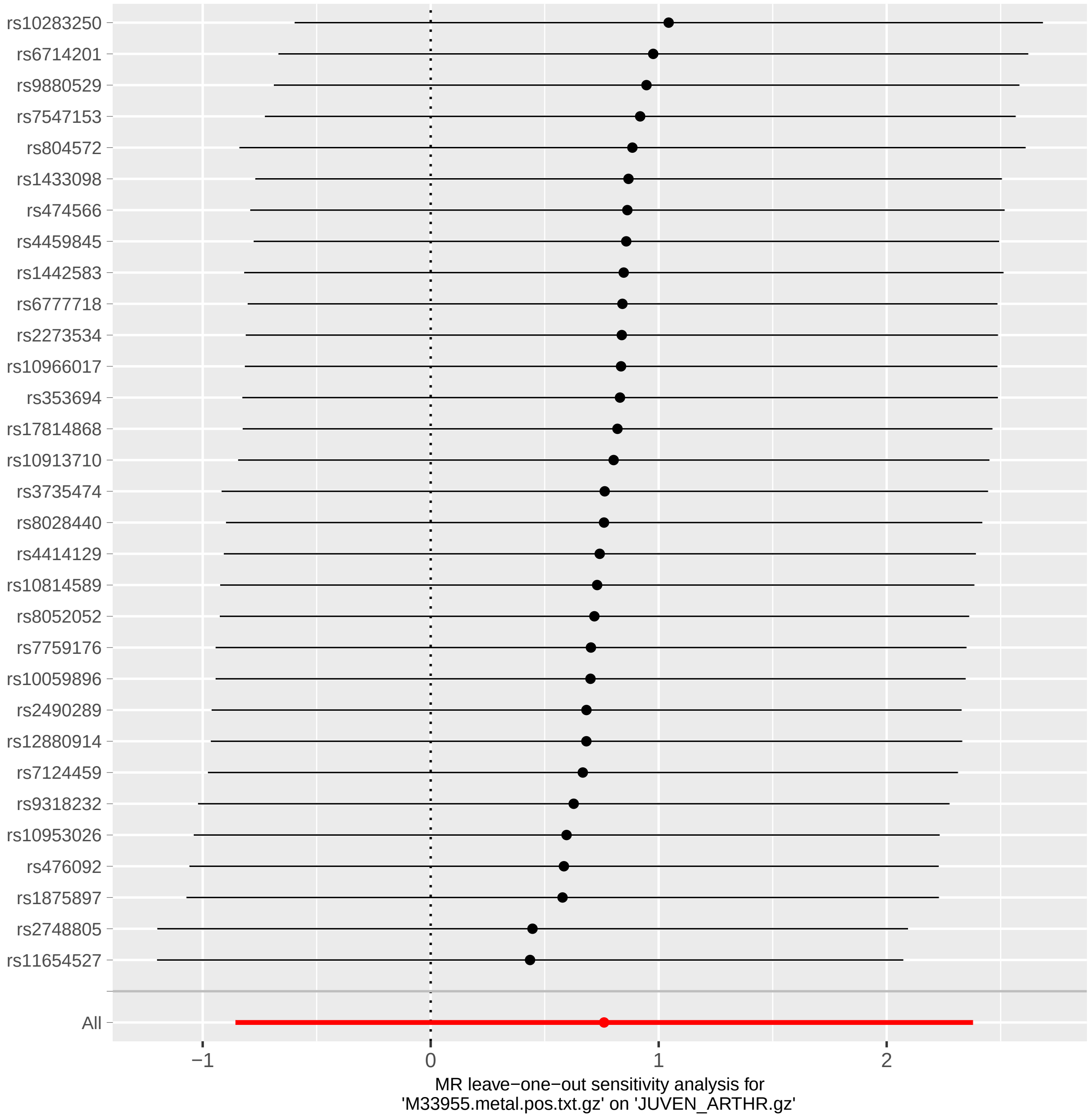
rs10283250
rs6714201
rs9880529
rs7547153
rs804572
rs1433098
rs474566
rs4459845
rs1442583
rs6777718
rs2273534
rs10966017
rs353694
rs17814868
rs10913710
rs3735474
rs8028440
rs4414129
rs10814589
rs8052052
rs7759176
rs10059896
rs2490289
rs12880914
rs7124459
rs9318232
rs10953026
rs476092
rs1875897
rs2748805
rs11654527
All
−1
0
1
2
MR leave−one−out sensitivity analysis for
'M33955.metal.pos.txt.gz' on 'JUVEN_ARTHR.gz'

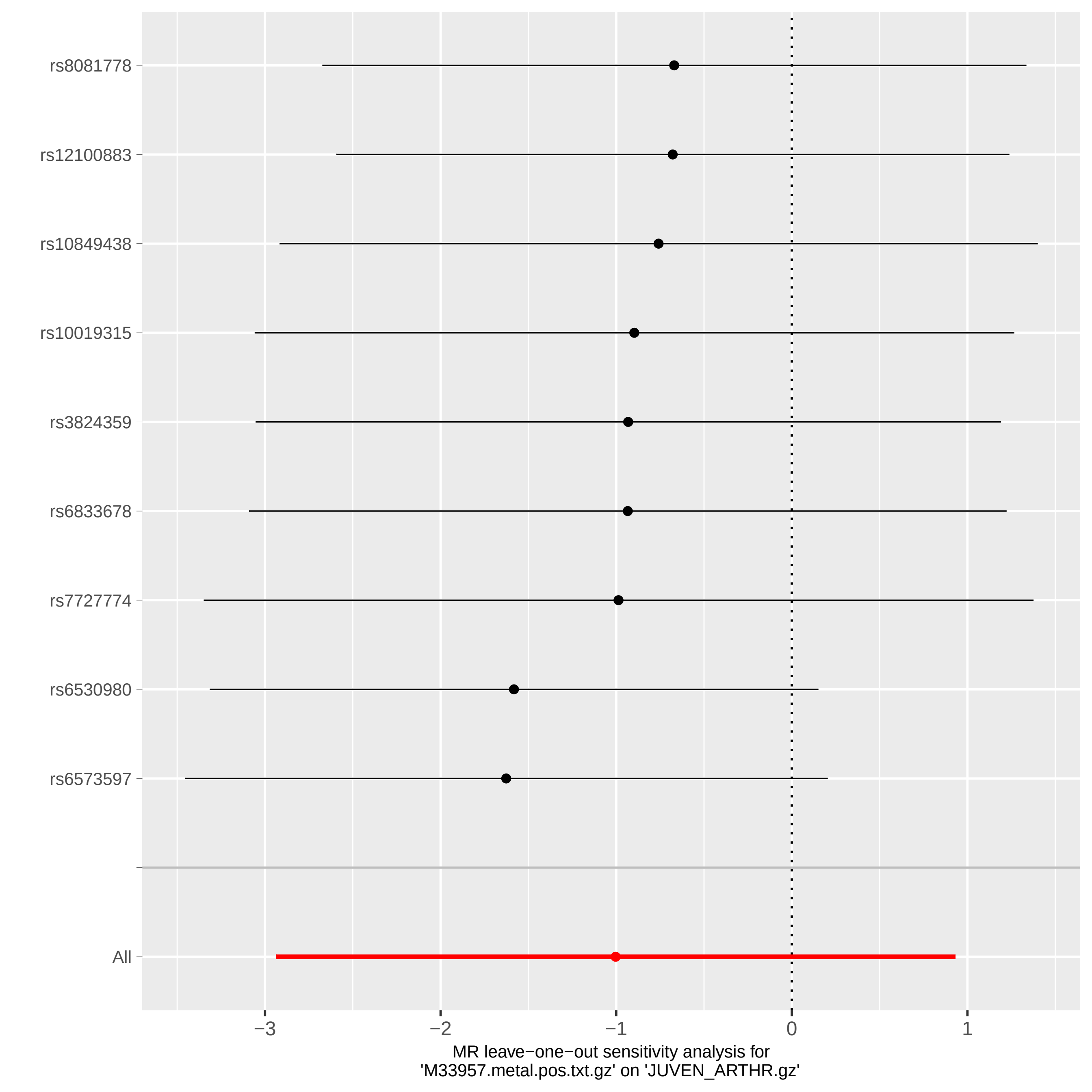
rs8081778
rs12100883
rs10849438
rs10019315
rs3824359
rs6833678
rs7727774
rs6530980
rs6573597
All
−3
−2
−1
0
1
MR leave−one−out sensitivity analysis for
'M33957.metal.pos.txt.gz' on 'JUVEN_ARTHR.gz'

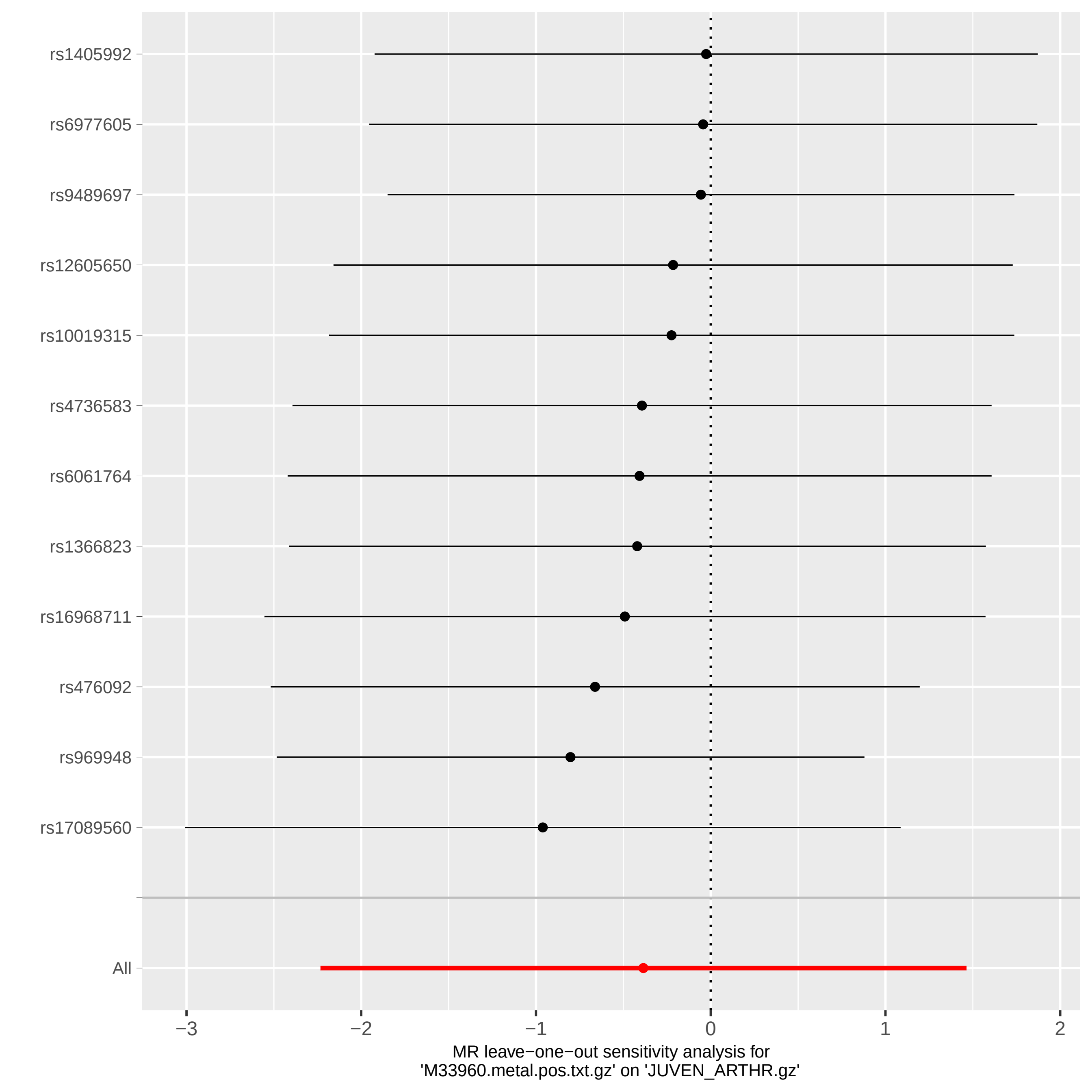

rs1405992
rs6977605
rs9489697
rs12605650
rs10019315
rs4736583
rs6061764
rs1366823
rs16968711
rs476092
rs969948
rs17089560
All
−3
−2
−1
0
1
2
MR leave−one−out sensitivity analysis for
'M33960.metal.pos.txt.gz' on 'JUVEN_ARTHR.gz'

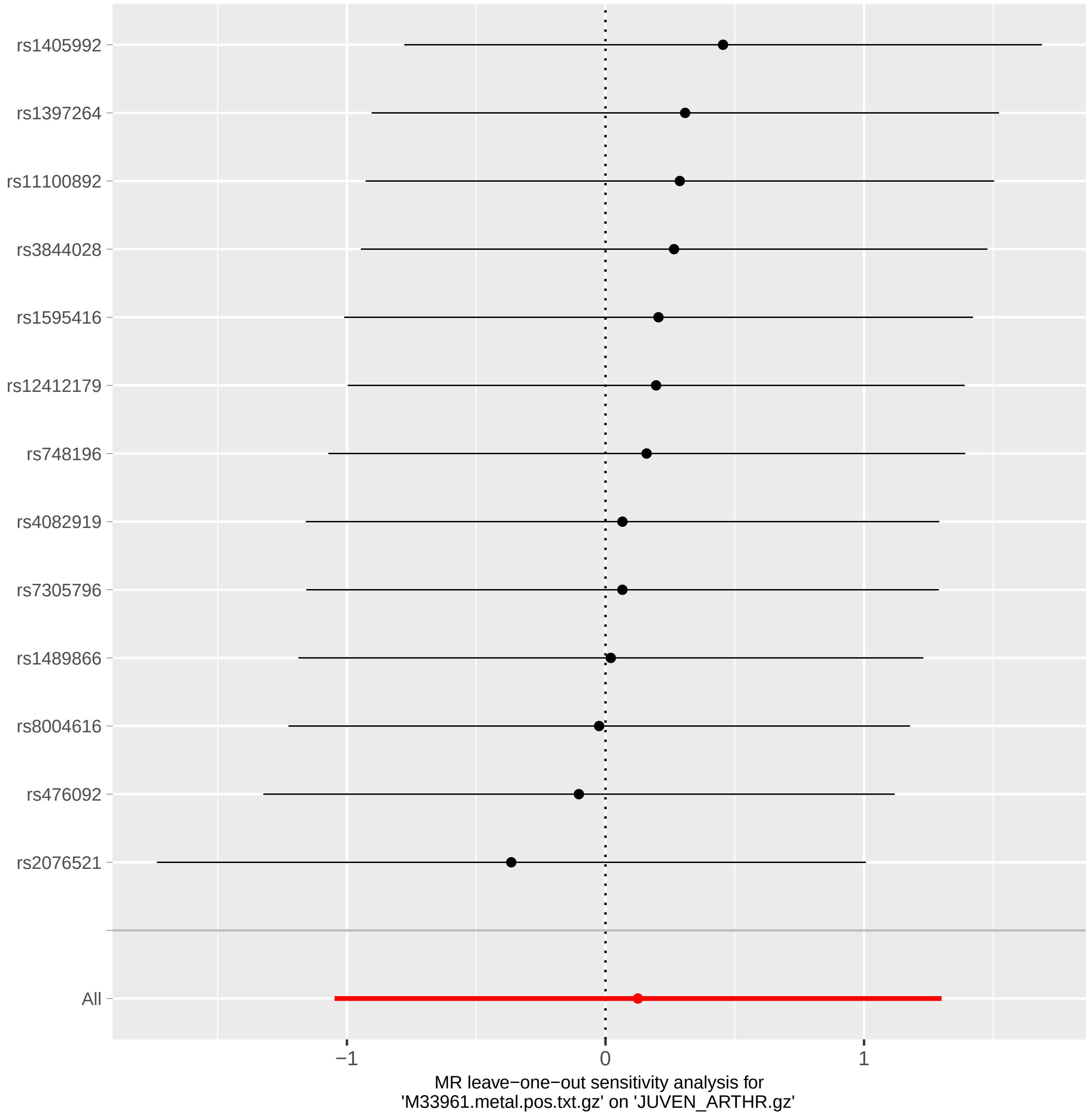
rs1405992
rs1397264
rs11100892
rs3844028
rs1595416
rs12412179
rs748196
rs4082919
rs7305796
rs1489866
rs8004616
rs476092
rs2076521
All
−1
0
1
MR leave−one−out sensitivity analysis for
'M33961.metal.pos.txt.gz' on 'JUVEN_ARTHR.gz'

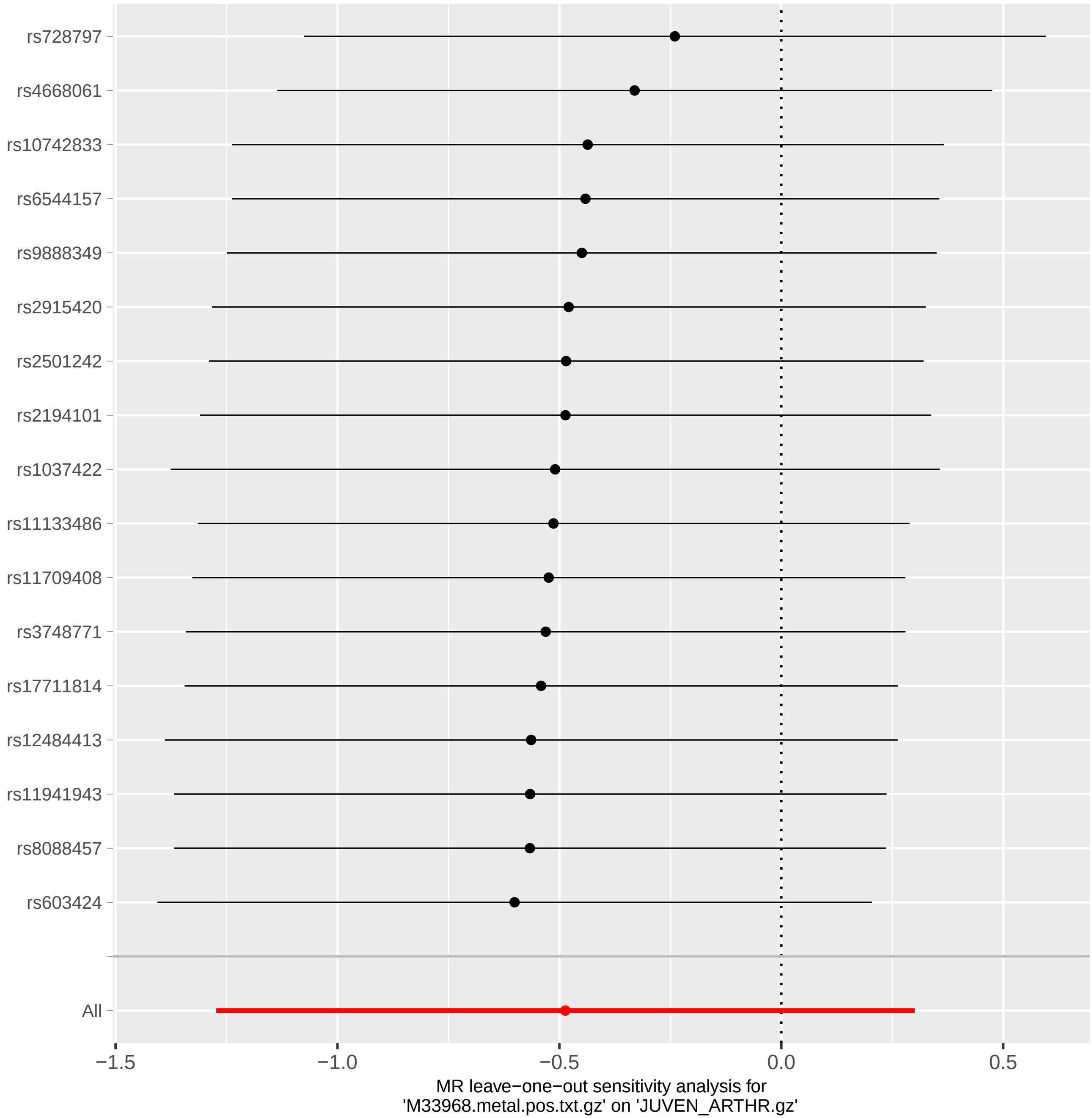
rs728797
rs4668061
rs10742833
rs6544157
rs9888349
rs2915420
rs2501242
rs2194101
rs1037422
rs11133486
rs11709408
rs3748771
rs17711814
rs12484413
rs11941943
rs8088457
rs603424
All
−1.5
−1.0
−0.5
0.0
0.5
MR leave−one−out sensitivity analysis for
'M33968.metal.pos.txt.gz' on 'JUVEN_ARTHR.gz'

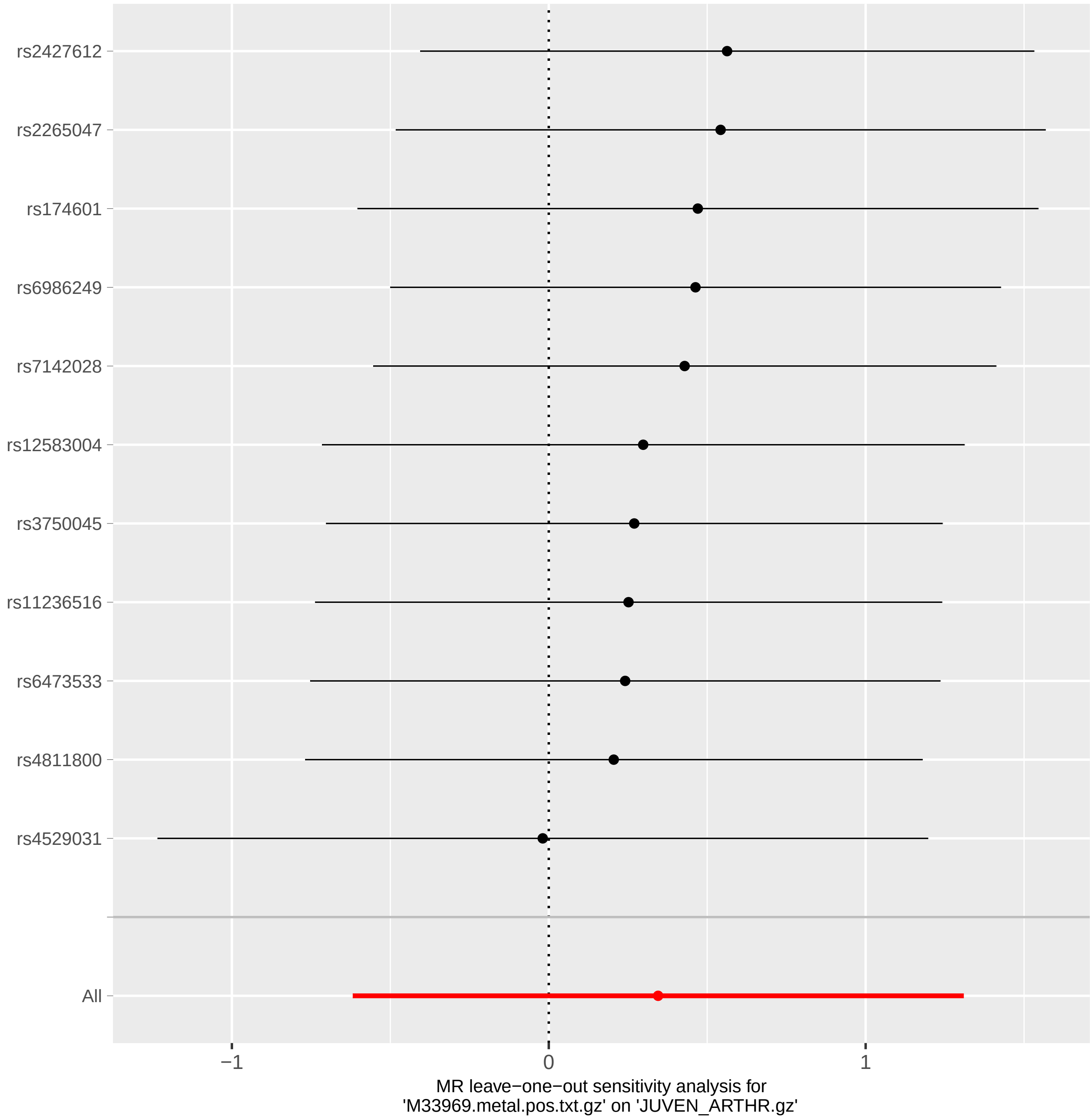

rs2427612
rs2265047
rs174601
rs6986249
rs7142028
rs12583004
rs3750045
rs11236516
rs6473533
rs4811800
rs4529031
All
−1
0
1
MR leave−one−out sensitivity analysis for
'M33969.metal.pos.txt.gz' on 'JUVEN_ARTHR.gz'

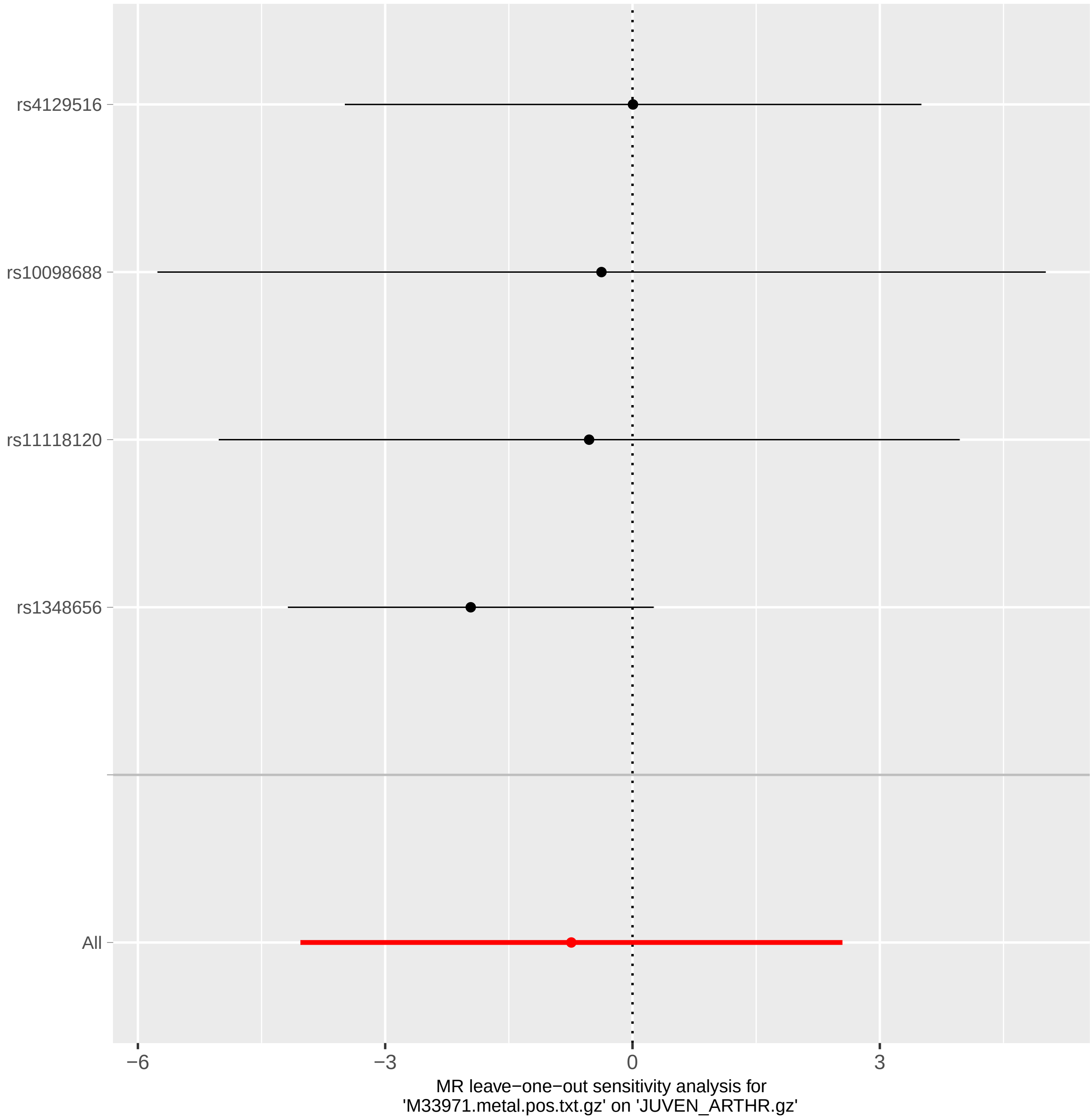

rs4129516
rs10098688
rs11118120
rs1348656
All
−6
−3
0
3
MR leave−one−out sensitivity analysis for
'M33971.metal.pos.txt.gz' on 'JUVEN_ARTHR.gz'

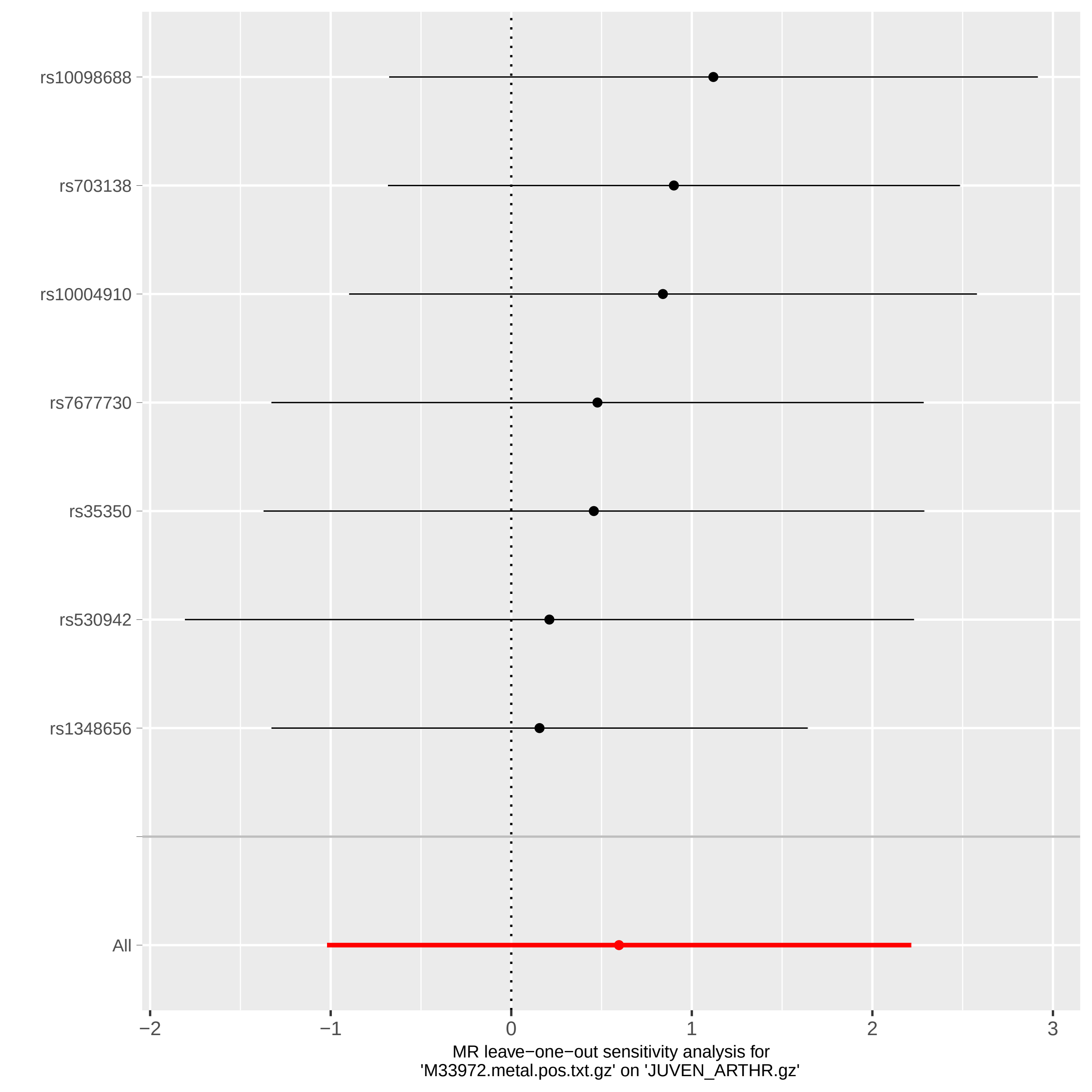

rs10098688
rs703138
rs10004910
rs7677730
rs35350
rs530942
rs1348656
All
−2
−1
0
1
2
3
MR leave−one−out sensitivity analysis for
'M33972.metal.pos.txt.gz' on 'JUVEN_ARTHR.gz'

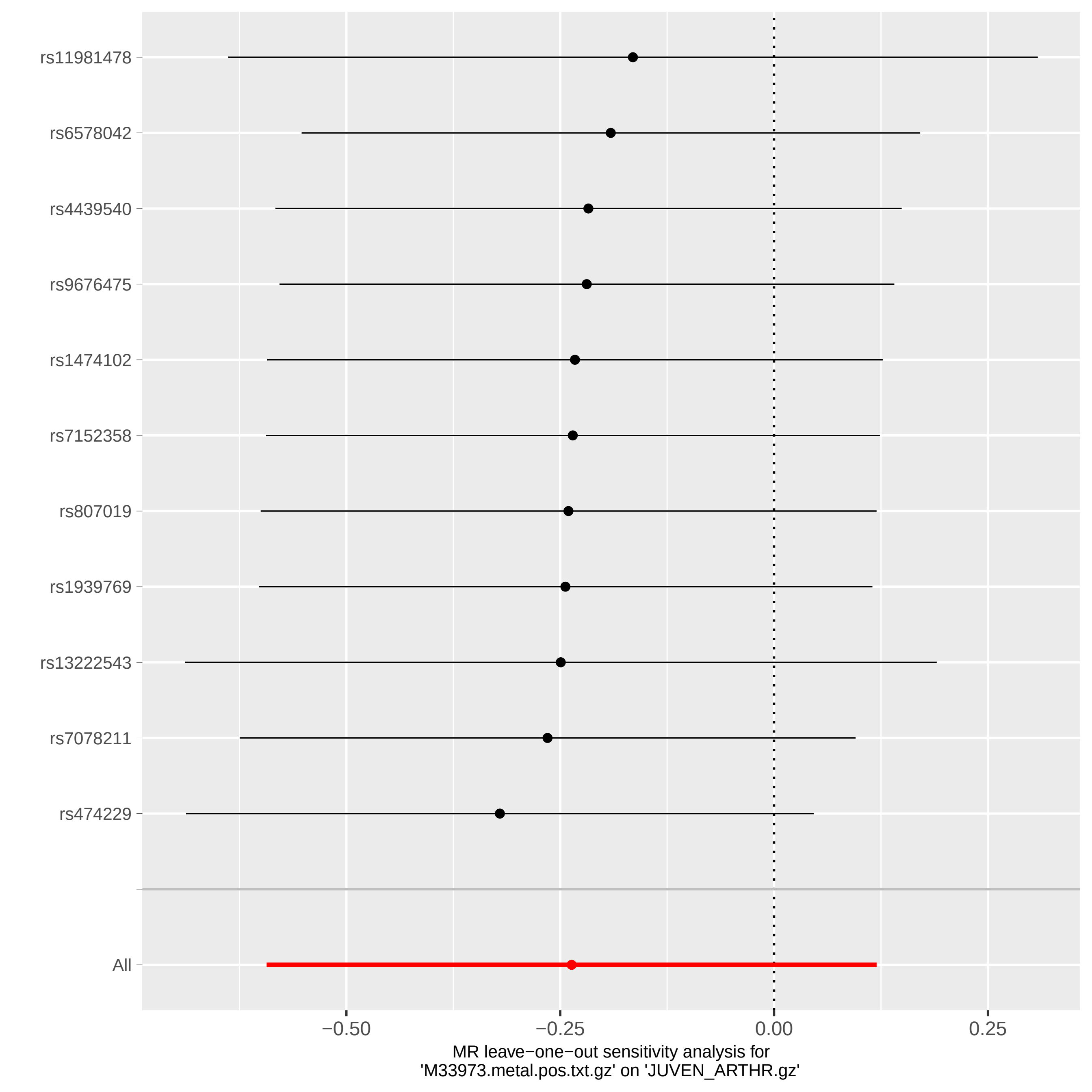

rs11981478
rs6578042
rs4439540
rs9676475
rs1474102
rs7152358
rs807019
rs1939769
rs13222543
rs7078211
rs474229
All
−0.50
−0.25
0.00
0.25
MR leave−one−out sensitivity analysis for
'M33973.metal.pos.txt.gz' on 'JUVEN_ARTHR.gz'

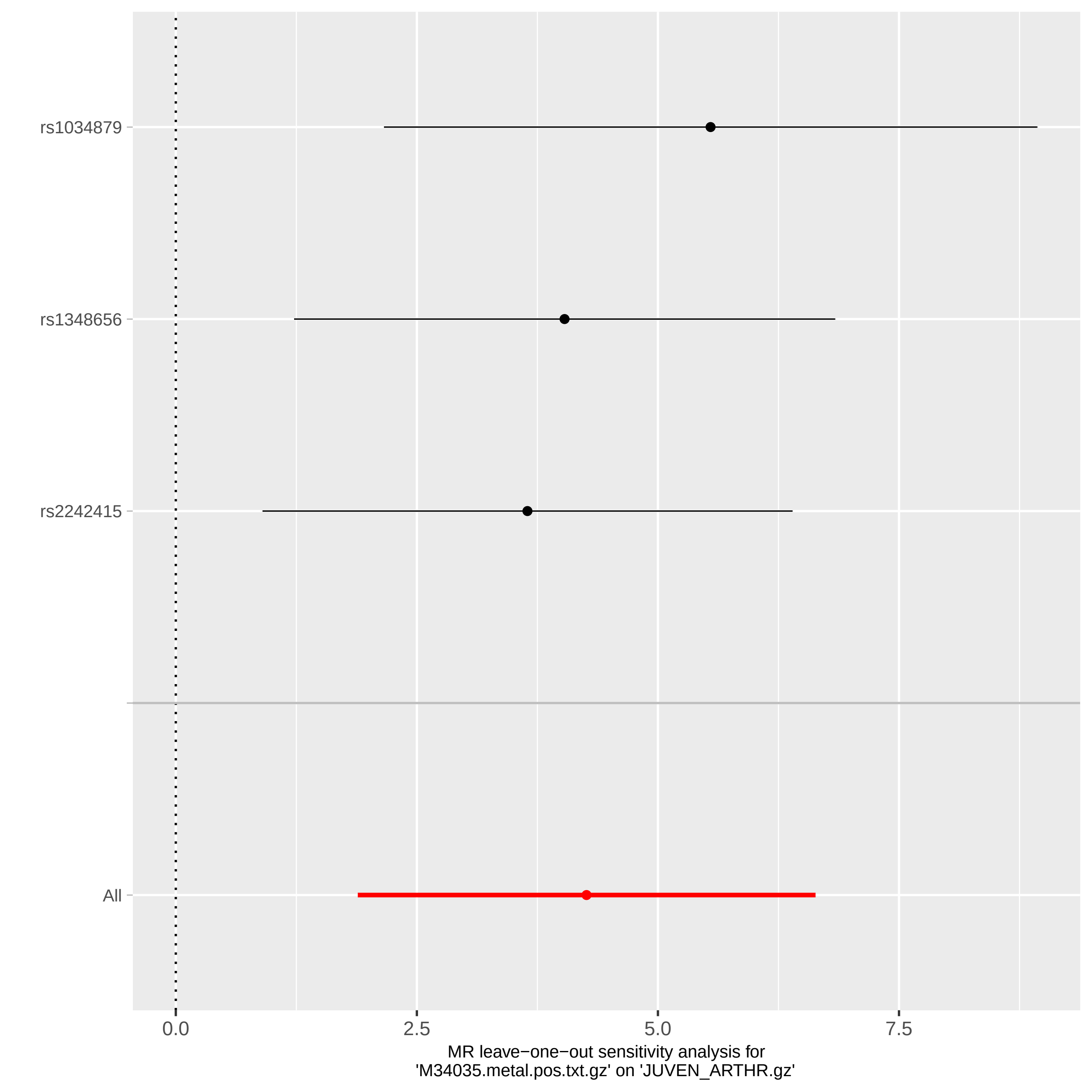
rs1034879
rs1348656
rs2242415
All
0.0
2.5
5.0
7.5
MR leave−one−out sensitivity analysis for
'M34035.metal.pos.txt.gz' on 'JUVEN_ARTHR.gz'

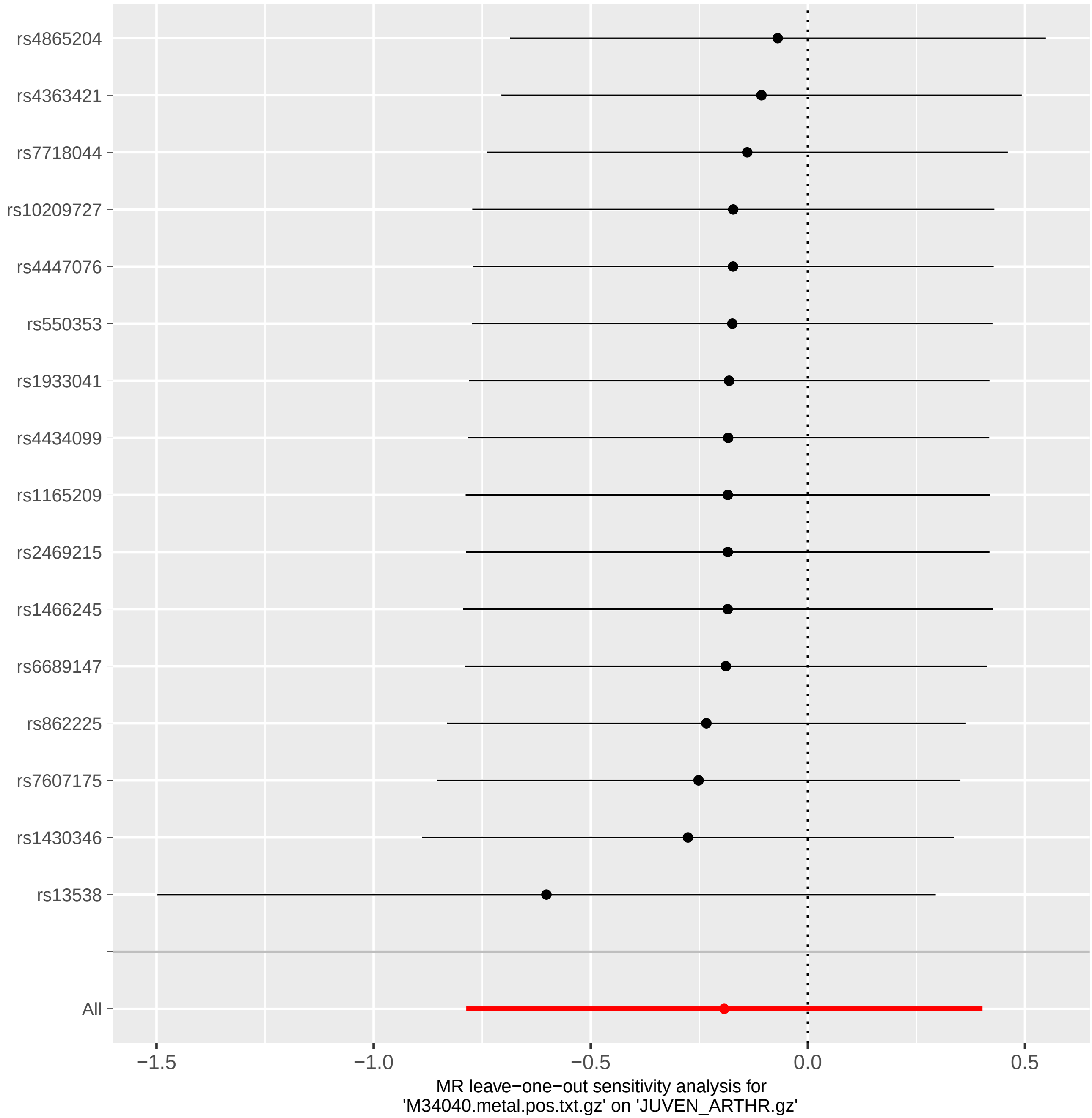

rs4865204
rs4363421
rs7718044
rs10209727
rs4447076
rs550353
rs1933041
rs4434099
rs1165209
rs2469215
rs1466245
rs6689147
rs862225
rs7607175
rs1430346
rs13538
All
−1.5
−1.0
−0.5
0.0
0.5
MR leave−one−out sensitivity analysis for
'M34040.metal.pos.txt.gz' on 'JUVEN_ARTHR.gz'

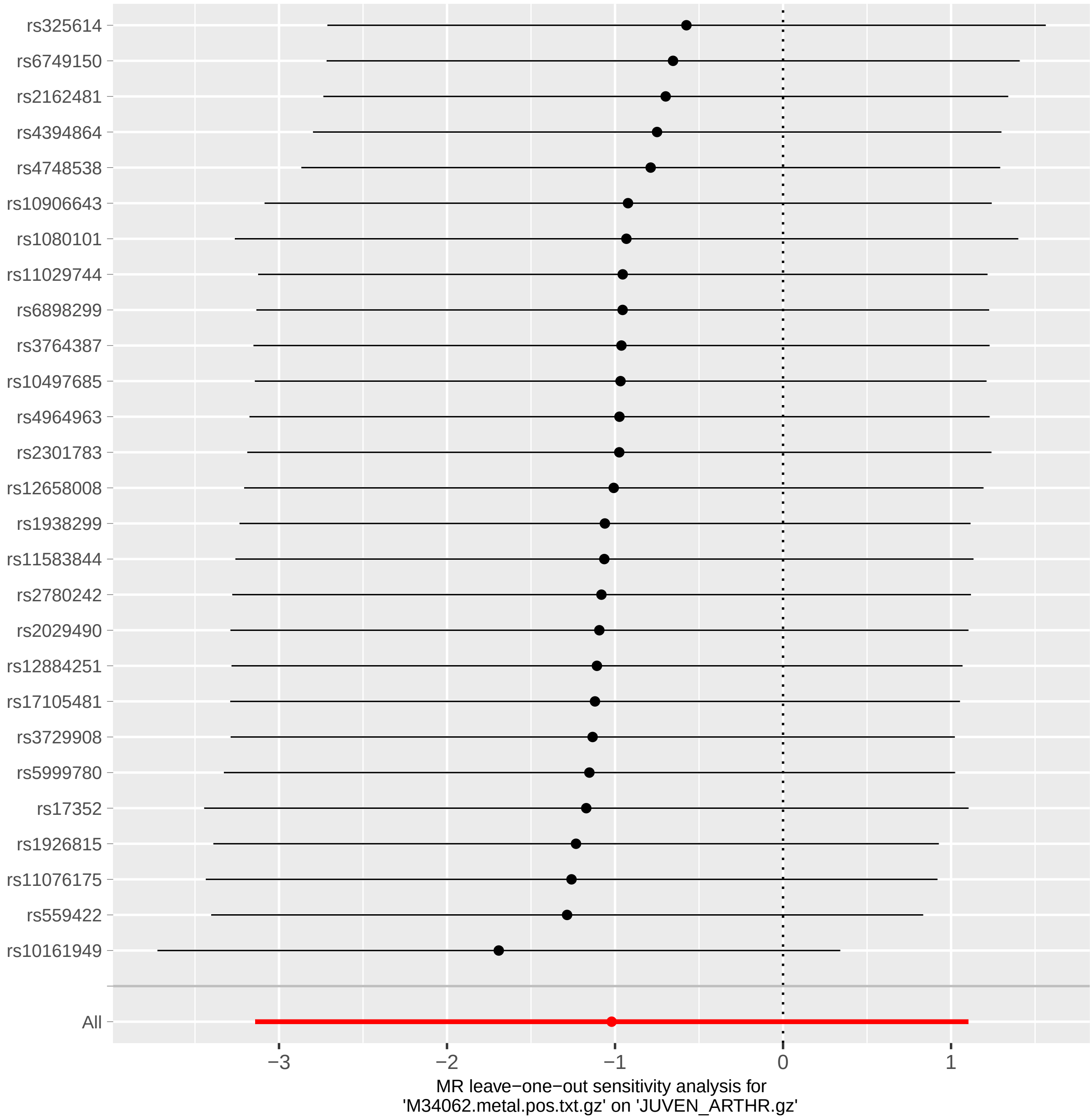

rs325614
rs6749150
rs2162481
rs4394864
rs4748538
rs10906643
rs1080101
rs11029744
rs6898299
rs3764387
rs10497685
rs4964963
rs2301783
rs12658008
rs1938299
rs11583844
rs2780242
rs2029490
rs12884251
rs17105481
rs3729908
rs5999780
rs17352
rs1926815
rs11076175
rs559422
rs10161949
All
−3
−2
−1
0
1
MR leave−one−out sensitivity analysis for
'M34062.metal.pos.txt.gz' on 'JUVEN_ARTHR.gz'

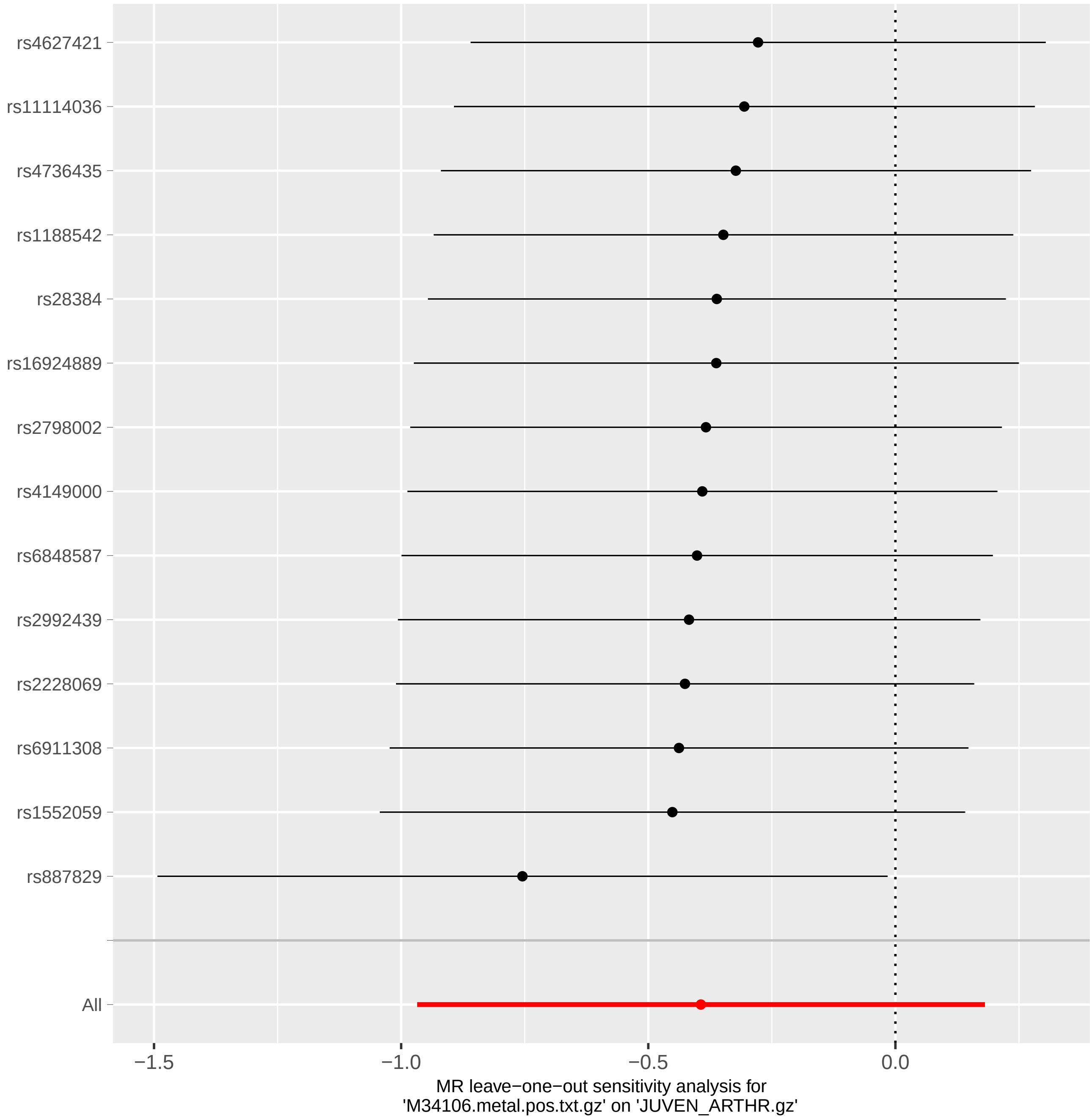

rs4627421
rs11114036
rs4736435
rs1188542
rs28384
rs16924889
rs2798002
rs4149000
rs6848587
rs2992439
rs2228069
rs6911308
rs1552059
rs887829
All
−1.5
−1.0
−0.5
0.0
MR leave−one−out sensitivity analysis for
'M34106.metal.pos.txt.gz' on 'JUVEN_ARTHR.gz'

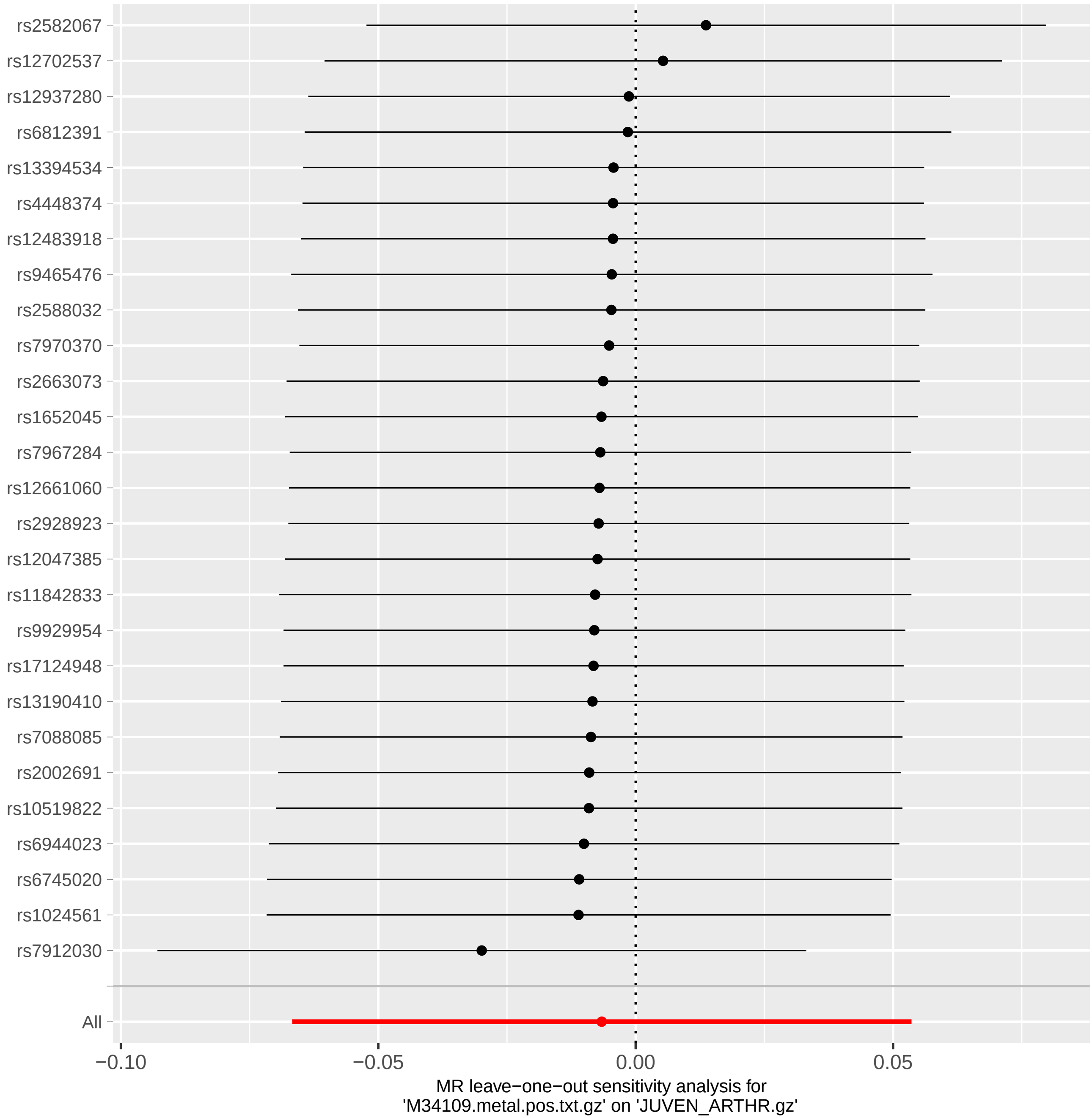
rs2582067
rs12702537
rs12937280
rs6812391
rs13394534
rs4448374
rs12483918
rs9465476
rs2588032
rs7970370
rs2663073
rs1652045
rs7967284
rs12661060
rs2928923
rs12047385
rs11842833
rs9929954
rs17124948
rs13190410
rs7088085
rs2002691
rs10519822
rs6944023
rs6745020
rs1024561
rs7912030
All
−0.10
−0.05
0.00
0.05
MR leave−one−out sensitivity analysis for
'M34109.metal.pos.txt.gz' on 'JUVEN_ARTHR.gz'

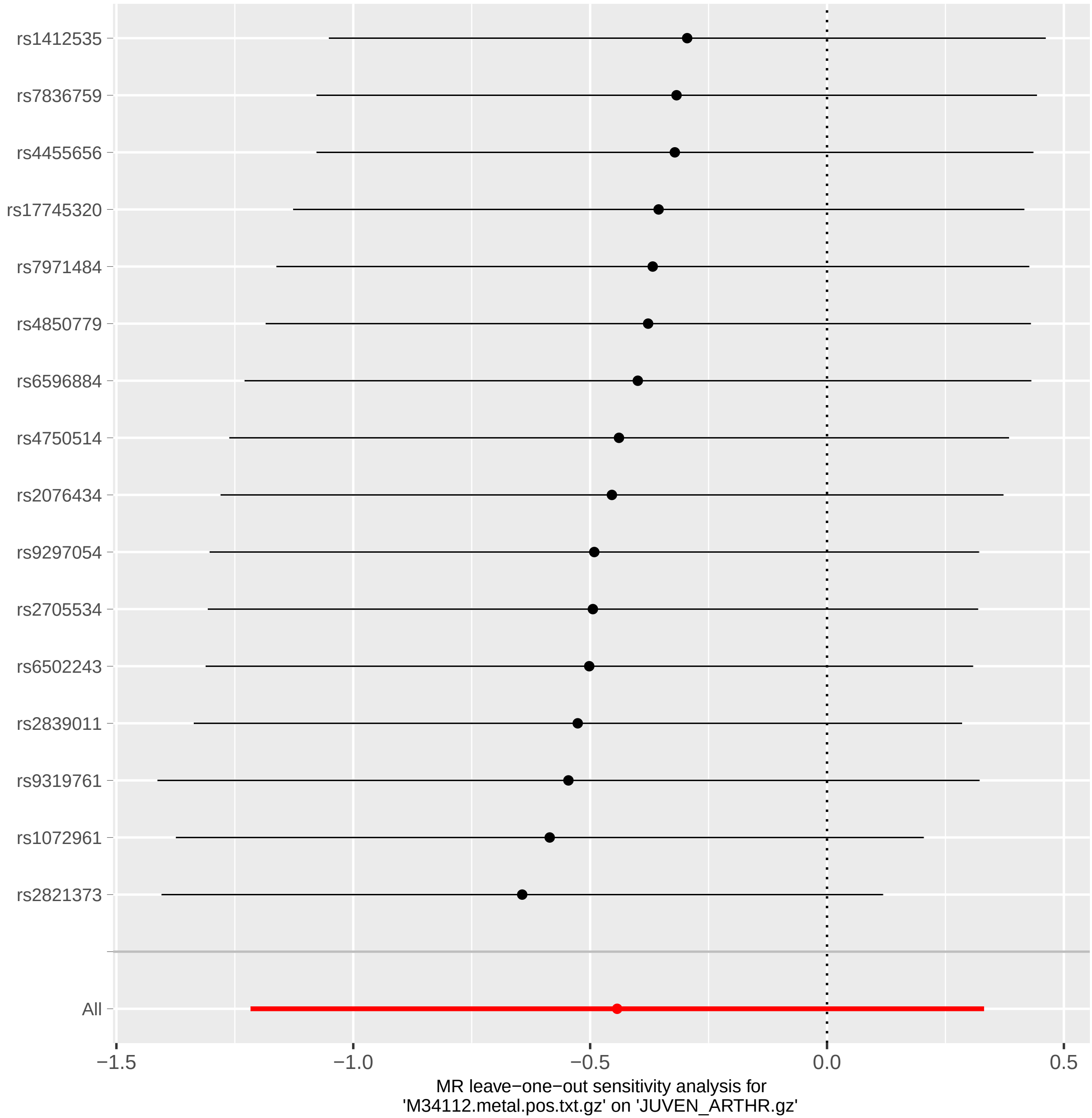
rs1412535
rs7836759
rs4455656
rs17745320
rs7971484
rs4850779
rs6596884
rs4750514
rs2076434
rs9297054
rs2705534
rs6502243
rs2839011
rs9319761
rs1072961
rs2821373
All
−1.5
−1.0
−0.5
0.0
0.5
MR leave−one−out sensitivity analysis for
'M34112.metal.pos.txt.gz' on 'JUVEN_ARTHR.gz'

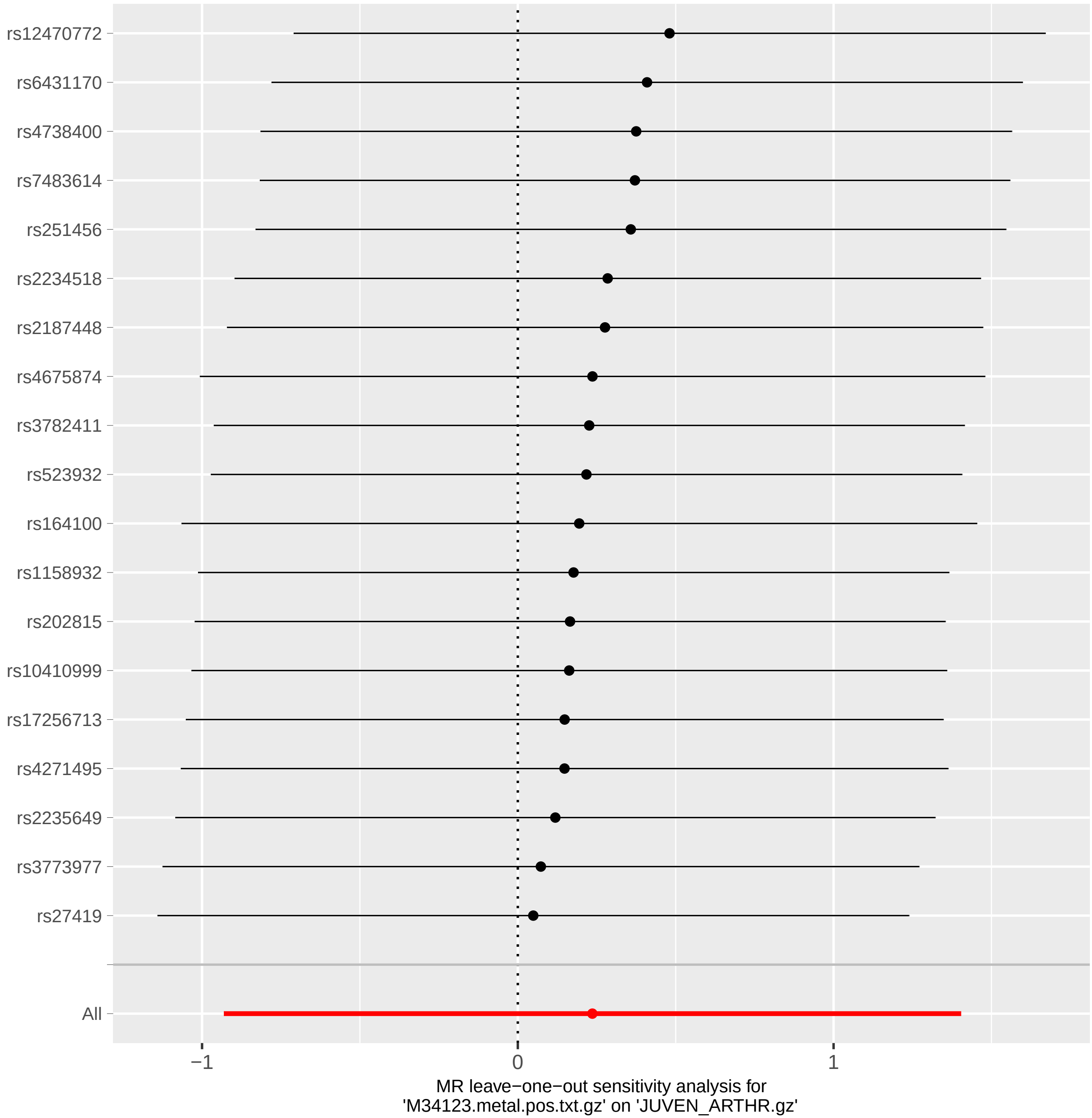
rs12470772
rs6431170
rs4738400
rs7483614
rs251456
rs2234518
rs2187448
rs4675874
rs3782411
rs523932
rs164100
rs1158932
rs202815
rs10410999
rs17256713
rs4271495
rs2235649
rs3773977
rs27419
All
−1
0
1
MR leave−one−out sensitivity analysis for
'M34123.metal.pos.txt.gz' on 'JUVEN_ARTHR.gz'

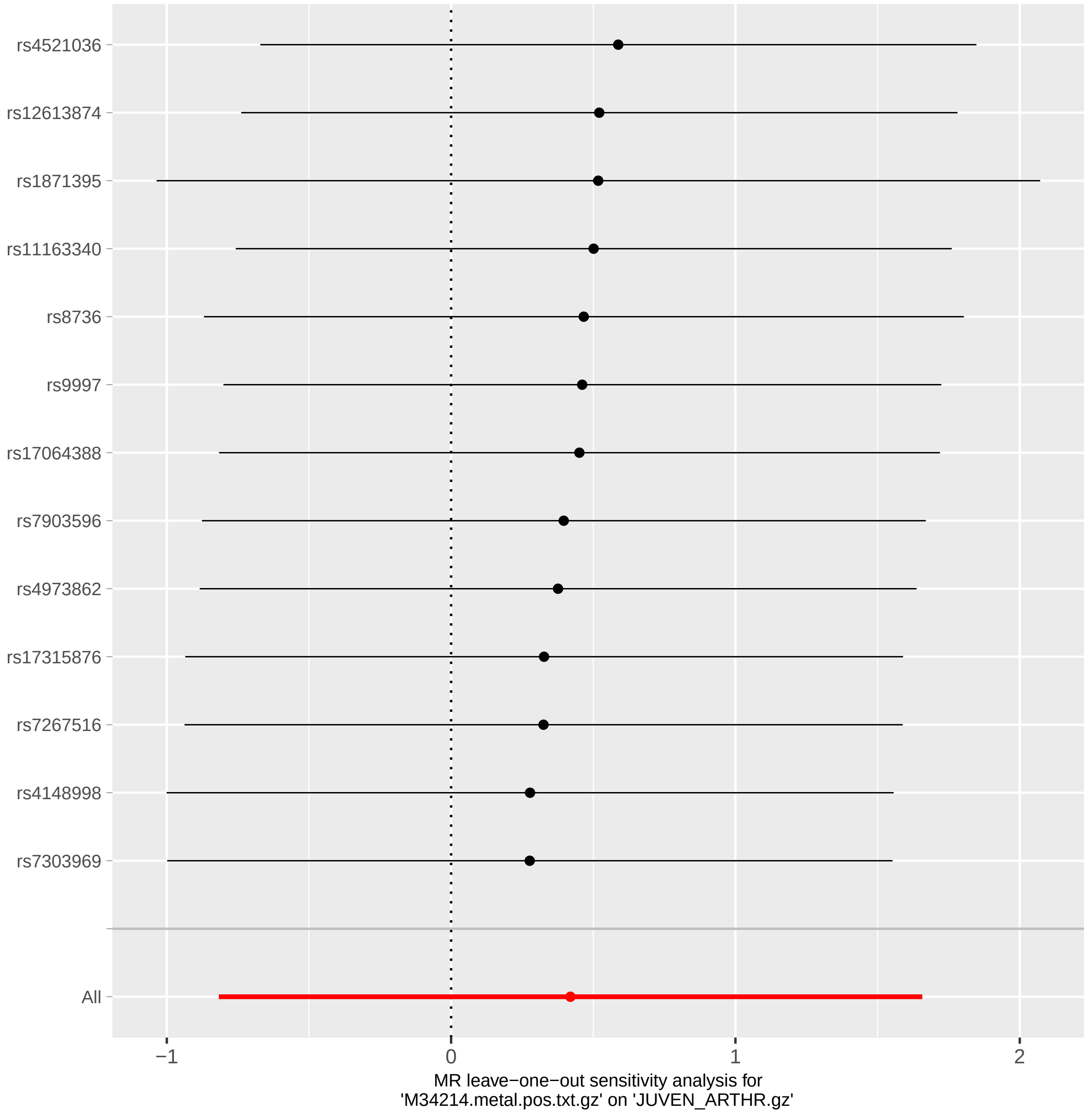

rs4521036
rs12613874
rs1871395
rs11163340
rs8736
rs9997
rs17064388
rs7903596
rs4973862
rs17315876
rs7267516
rs4148998
rs7303969
All
−1
0
1
2
MR leave−one−out sensitivity analysis for
'M34214.metal.pos.txt.gz' on 'JUVEN_ARTHR.gz'

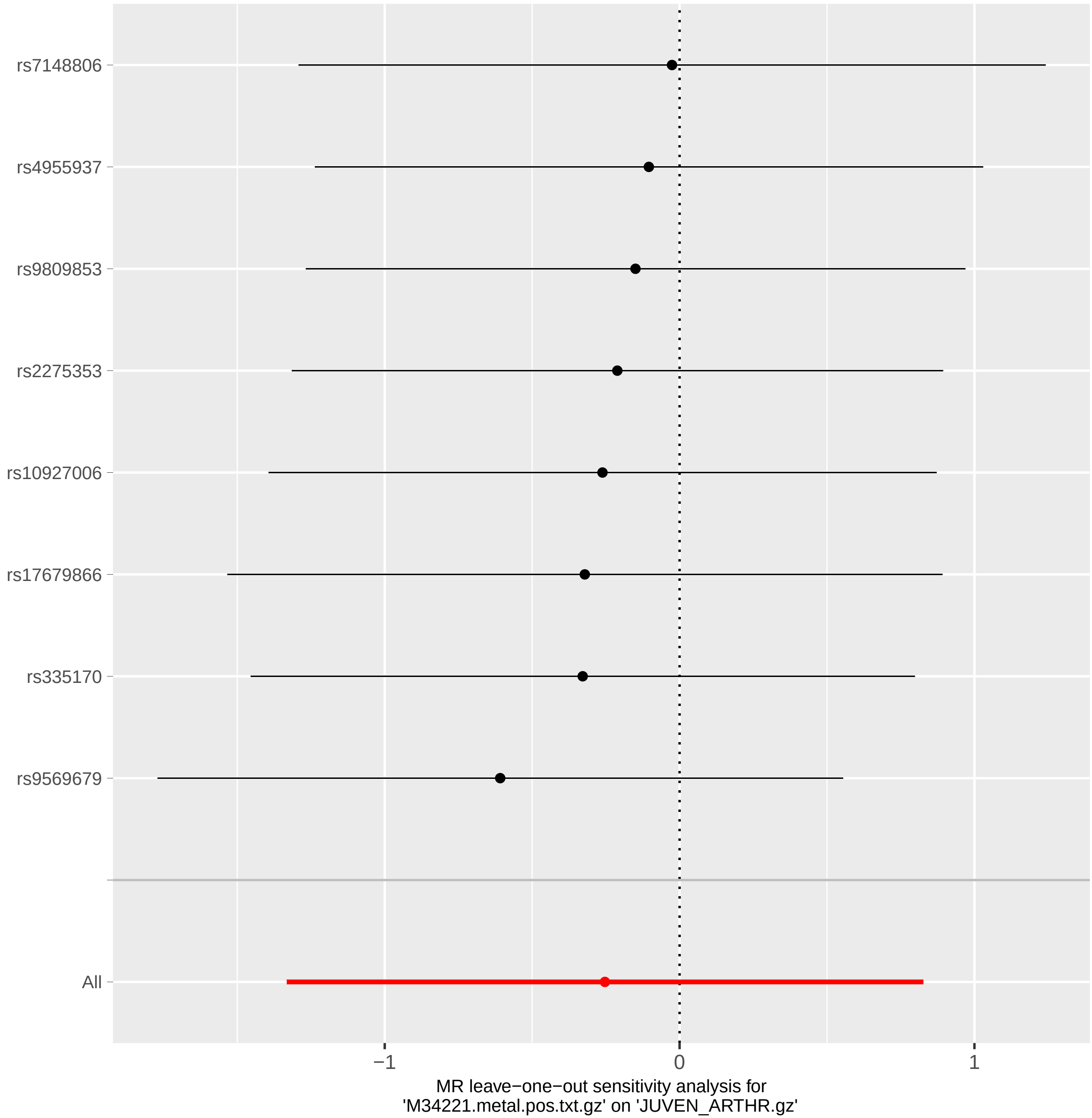
rs7148806
rs4955937
rs9809853
rs2275353
rs10927006
rs17679866
rs335170
rs9569679
All
−1
0
1
MR leave−one−out sensitivity analysis for
'M34221.metal.pos.txt.gz' on 'JUVEN_ARTHR.gz'

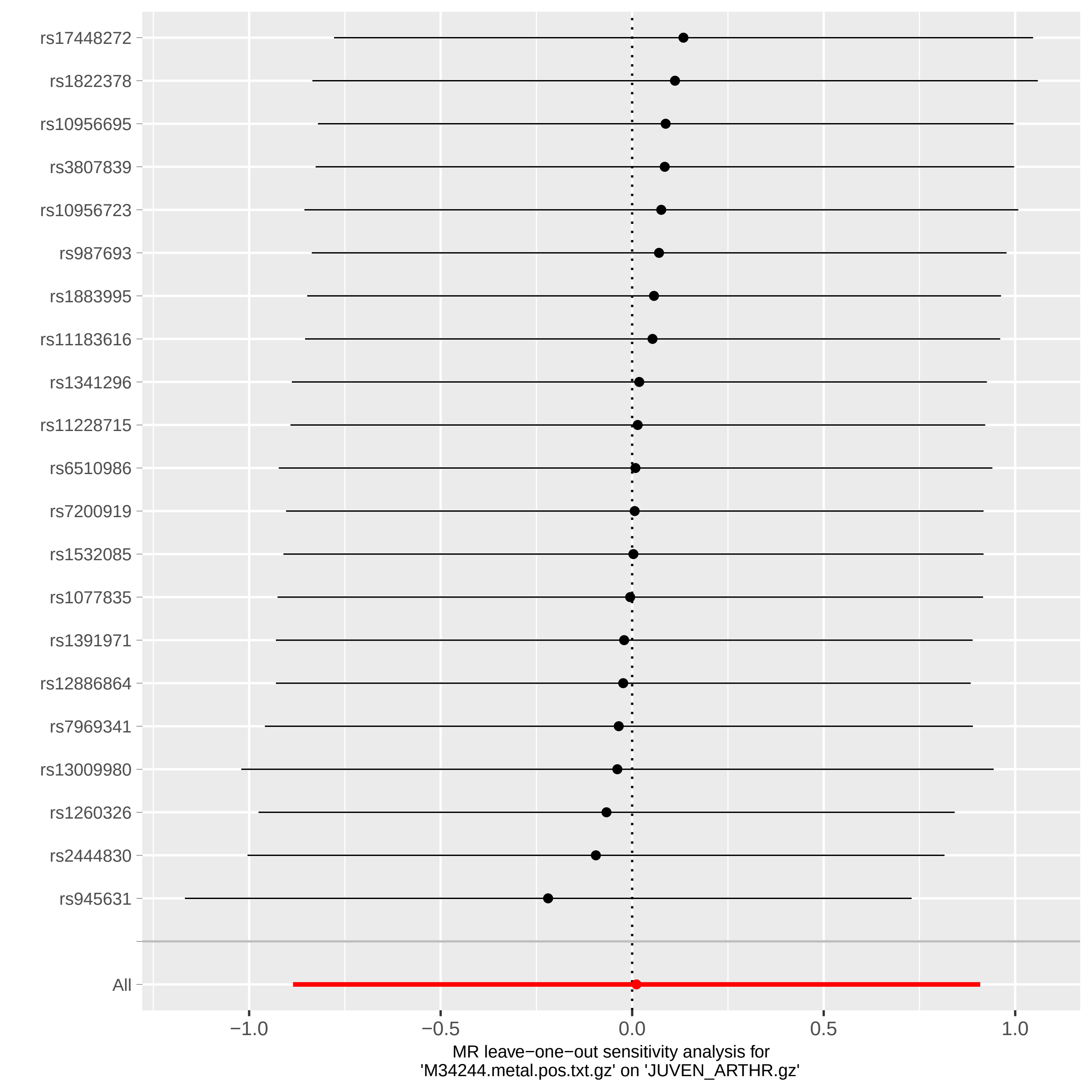
rs17448272
rs1822378
rs10956695
rs3807839
rs10956723
rs987693
rs1883995
rs11183616
rs1341296
rs11228715
rs6510986
rs7200919
rs1532085
rs1077835
rs1391971
rs12886864
rs7969341
rs13009980
rs1260326
rs2444830
rs945631
All
−1.0
−0.5
0.0
0.5
1.0
MR leave−one−out sensitivity analysis for
'M34244.metal.pos.txt.gz' on 'JUVEN_ARTHR.gz'

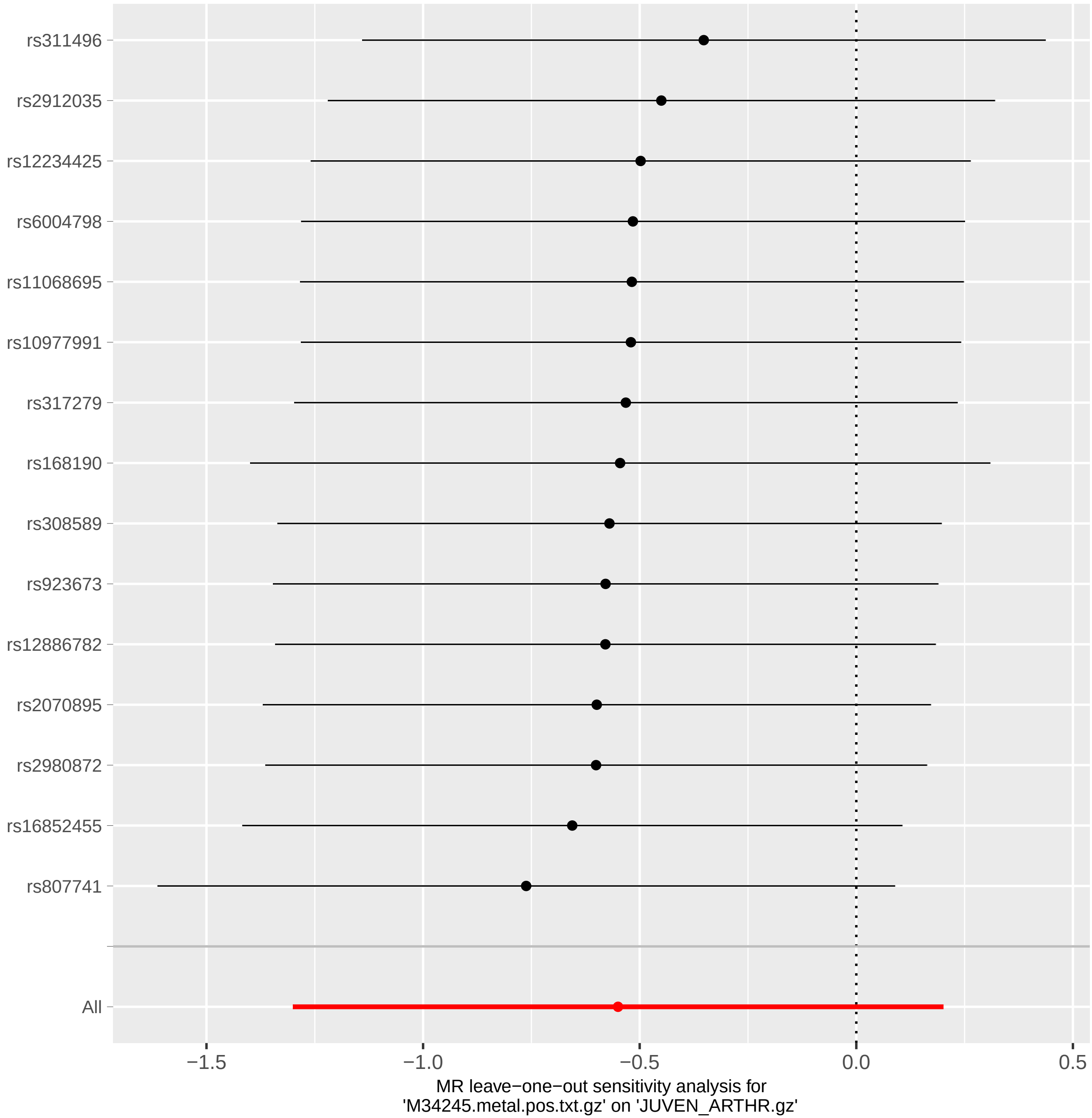
rs311496
rs2912035
rs12234425
rs6004798
rs11068695
rs10977991
rs317279
rs168190
rs308589
rs923673
rs12886782
rs2070895
rs2980872
rs16852455
rs807741
All
−1.5
−1.0
−0.5
0.0
0.5
MR leave−one−out sensitivity analysis for
'M34245.metal.pos.txt.gz' on 'JUVEN_ARTHR.gz'

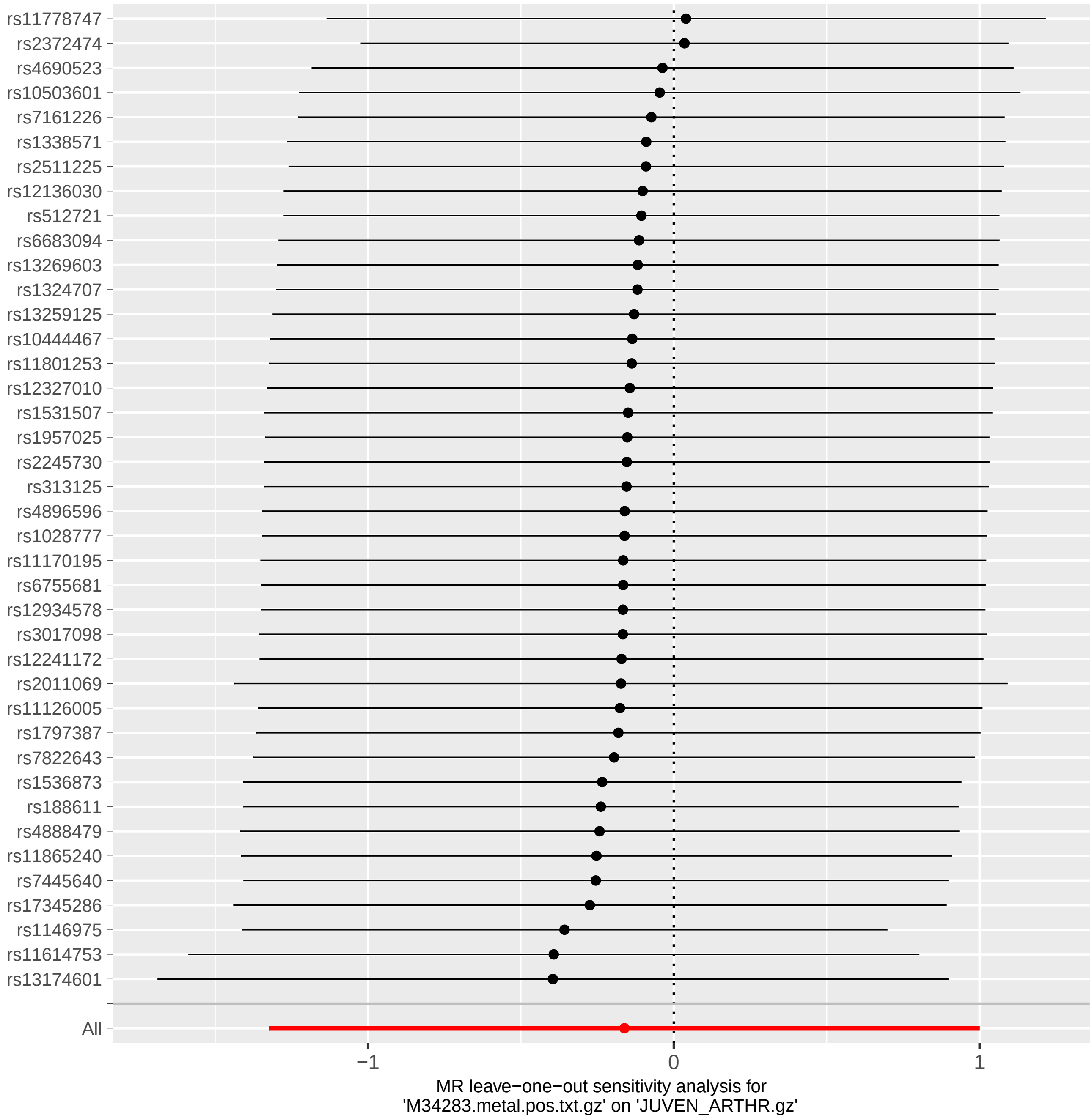
rs11778747
rs2372474
rs4690523
rs10503601
rs7161226
rs1338571
rs2511225
rs12136030
rs512721
rs6683094
rs13269603
rs1324707
rs13259125
rs10444467
rs11801253
rs12327010
rs1531507
rs1957025
rs2245730
rs313125
rs4896596
rs1028777
rs11170195
rs6755681
rs12934578
rs3017098
rs12241172
rs2011069
rs11126005
rs1797387
rs7822643
rs1536873
rs188611
rs4888479
rs11865240
rs7445640
rs17345286
rs1146975
rs11614753
rs13174601
All
−1
0
1
MR leave−one−out sensitivity analysis for
'M34283.metal.pos.txt.gz' on 'JUVEN_ARTHR.gz'

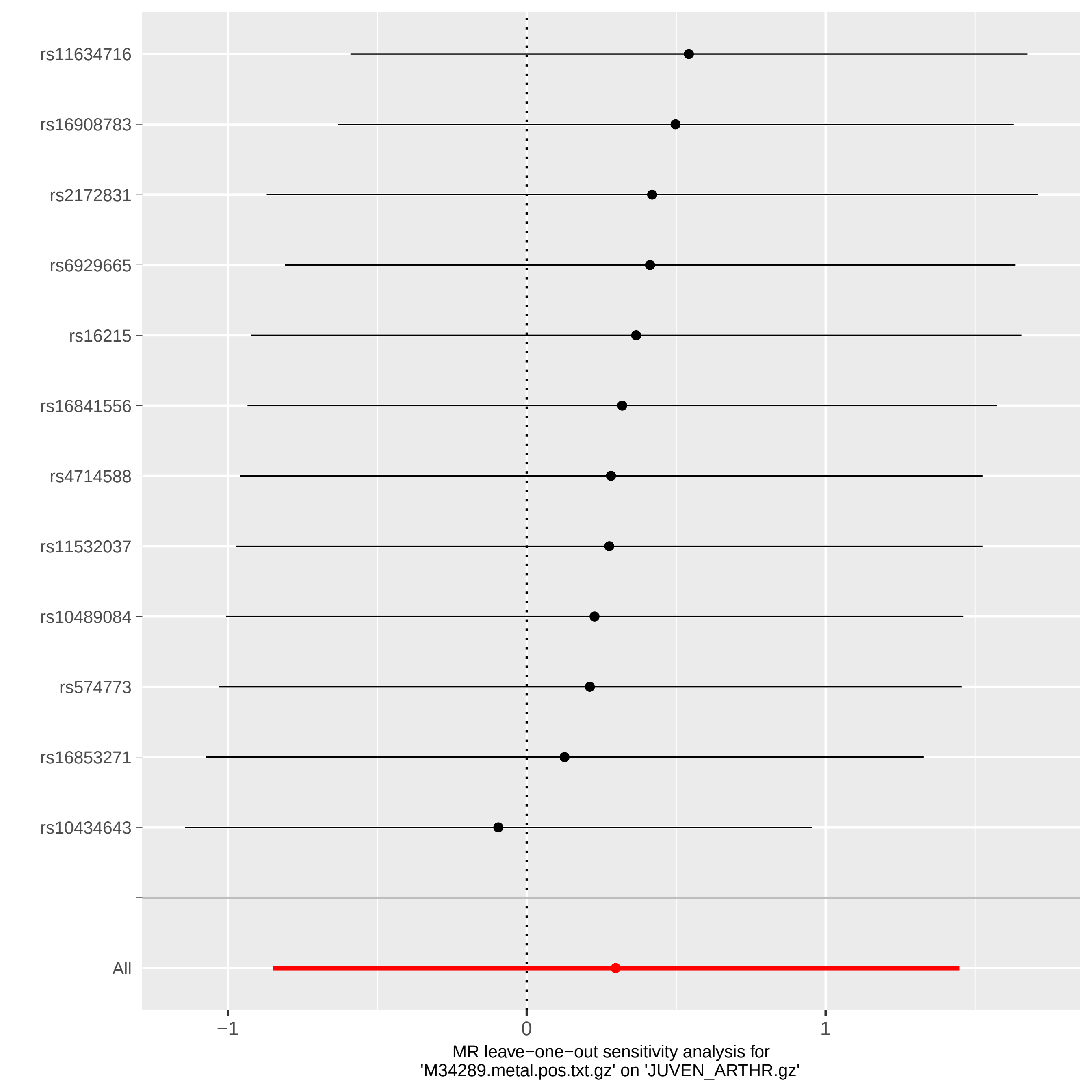
rs11634716
rs16908783
rs2172831
rs6929665
rs16215
rs16841556
rs4714588
rs11532037
rs10489084
rs574773
rs16853271
rs10434643
All
−1
0
1
MR leave−one−out sensitivity analysis for
'M34289.metal.pos.txt.gz' on 'JUVEN_ARTHR.gz'

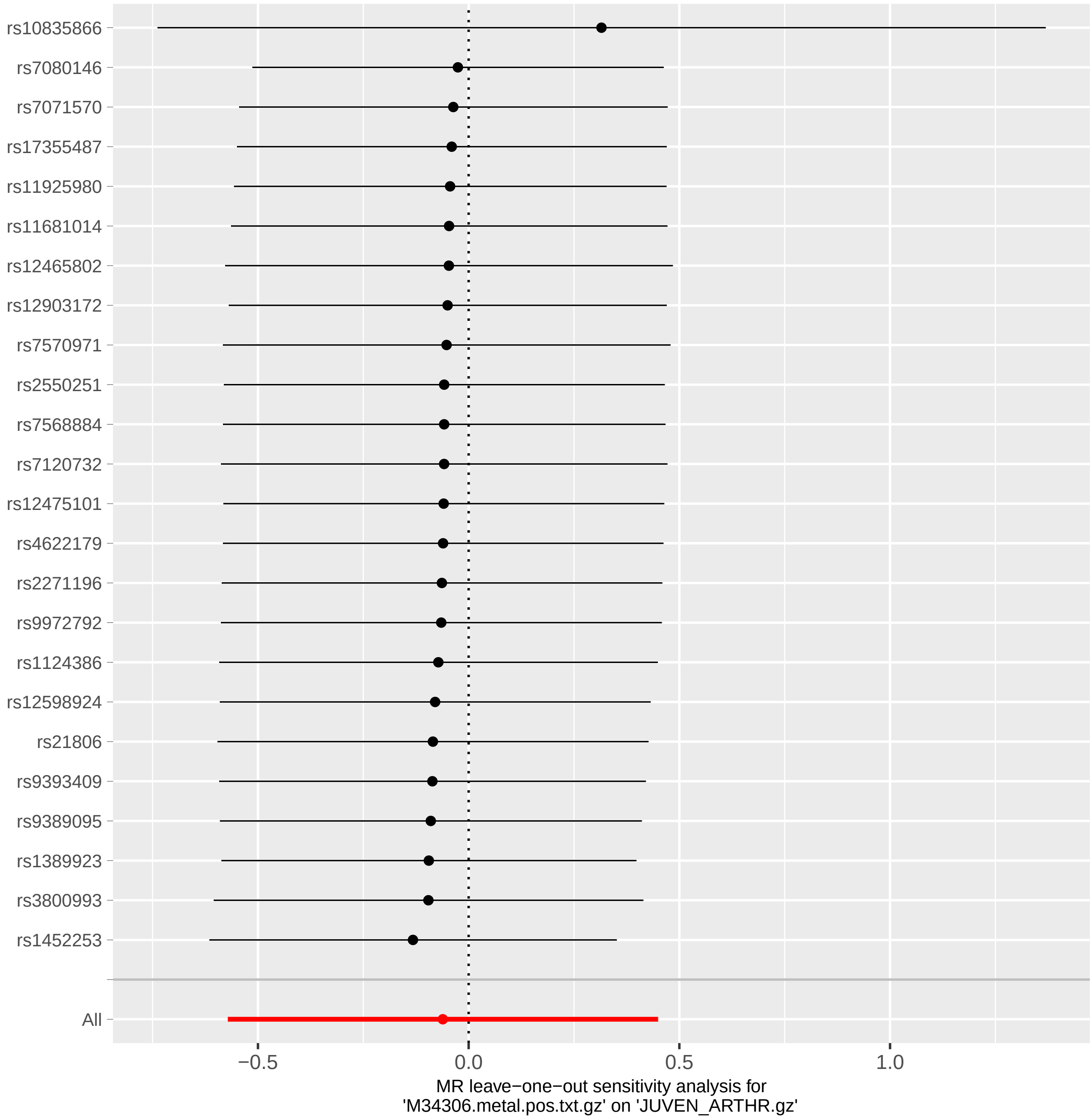

rs10835866
rs7080146
rs7071570
rs17355487
rs11925980
rs11681014
rs12465802
rs12903172
rs7570971
rs2550251
rs7568884
rs7120732
rs12475101
rs4622179
rs2271196
rs9972792
rs1124386
rs12598924
rs21806
rs9393409
rs9389095
rs1389923
rs3800993
rs1452253
All
−0.5
0.0
0.5
1.0
MR leave−one−out sensitivity analysis for
'M34306.metal.pos.txt.gz' on 'JUVEN_ARTHR.gz'

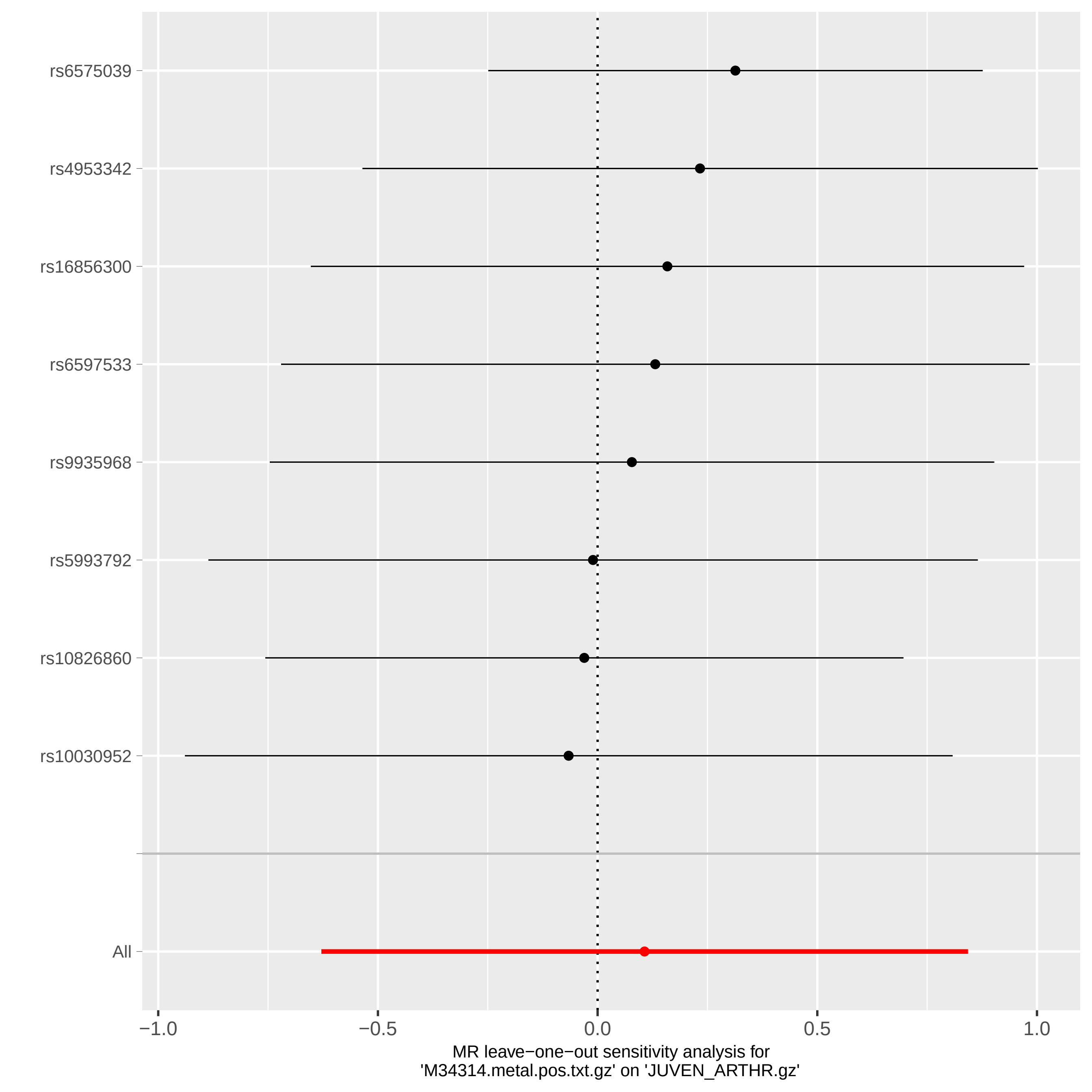

rs6575039
rs4953342
rs16856300
rs6597533
rs9935968
rs5993792
rs10826860
rs10030952
All
−1.0
−0.5
0.0
0.5
1.0
MR leave−one−out sensitivity analysis for
'M34314.metal.pos.txt.gz' on 'JUVEN_ARTHR.gz'

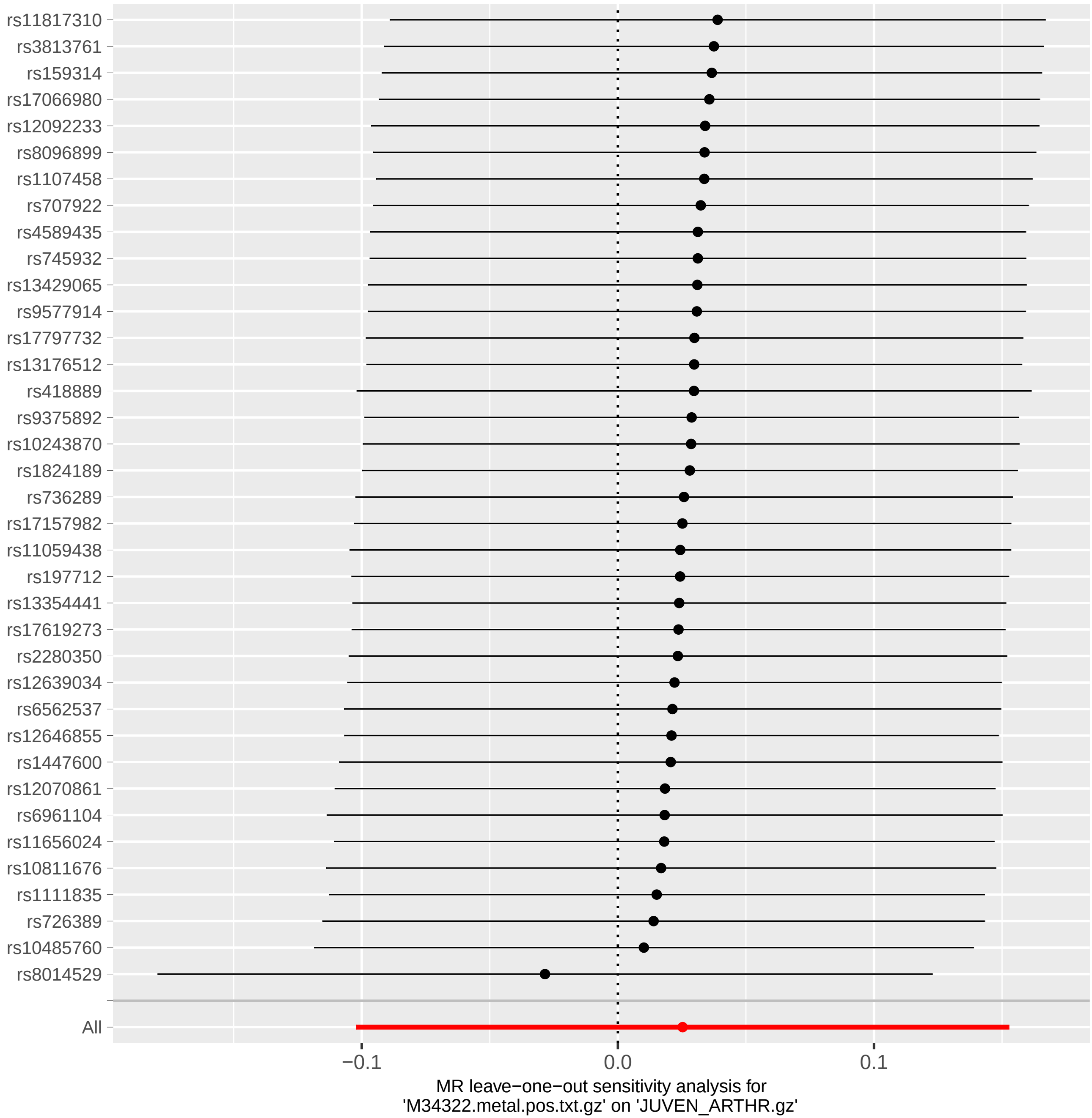
rs11817310
rs3813761
rs159314
rs17066980
rs12092233
rs8096899
rs1107458
rs707922
rs4589435
rs745932
rs13429065
rs9577914
rs17797732
rs13176512
rs418889
rs9375892
rs10243870
rs1824189
rs736289
rs17157982
rs11059438
rs197712
rs13354441
rs17619273
rs2280350
rs12639034
rs6562537
rs12646855
rs1447600
rs12070861
rs6961104
rs11656024
rs10811676
rs1111835
rs726389
rs10485760
rs8014529
All
−0.1
0.0
0.1
MR leave−one−out sensitivity analysis for
'M34322.metal.pos.txt.gz' on 'JUVEN_ARTHR.gz'

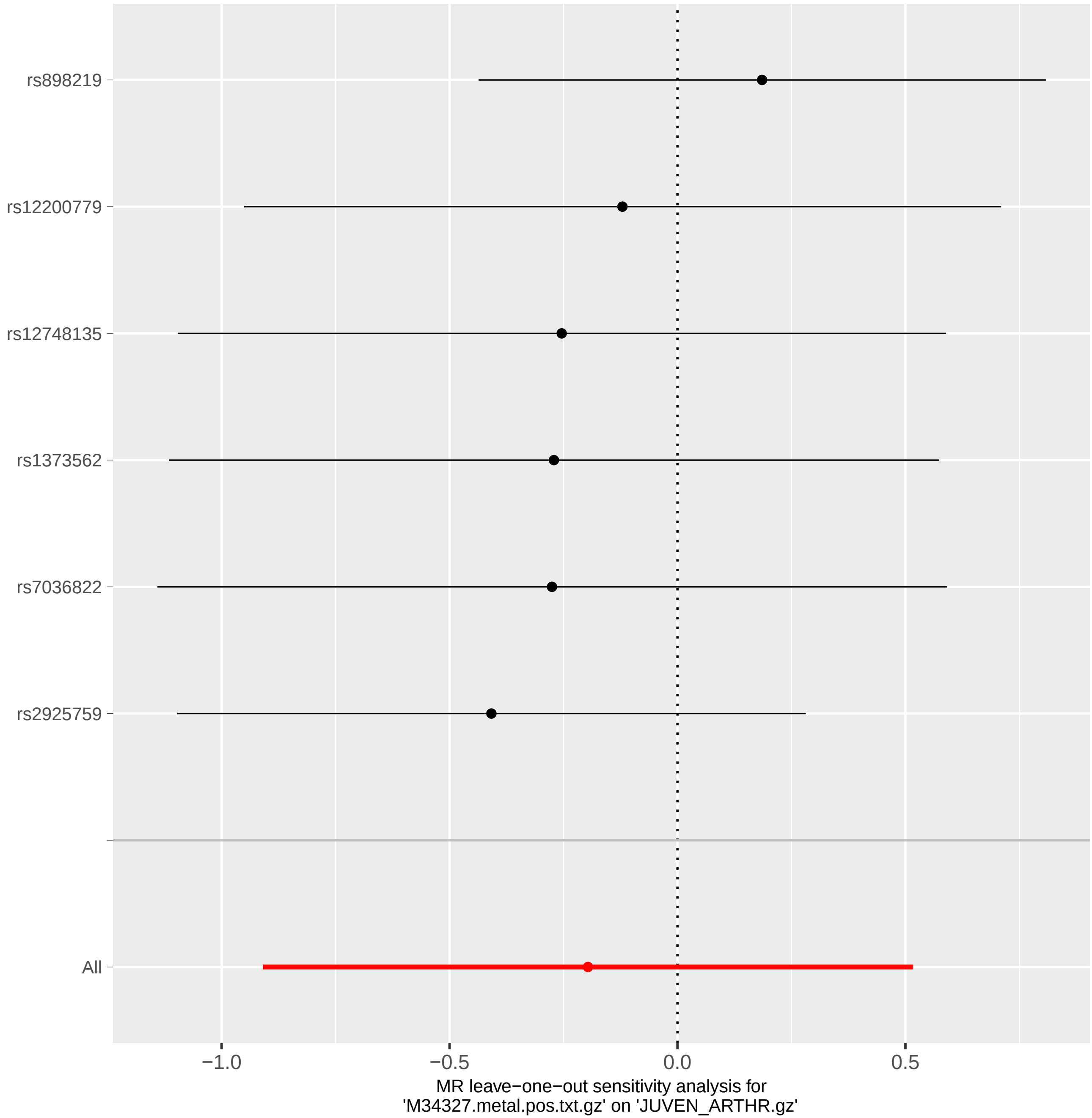
rs898219
rs12200779
rs12748135
rs1373562
rs7036822
rs2925759
All
−1.0
−0.5
0.0
0.5
MR leave−one−out sensitivity analysis for
'M34327.metal.pos.txt.gz' on 'JUVEN_ARTHR.gz'

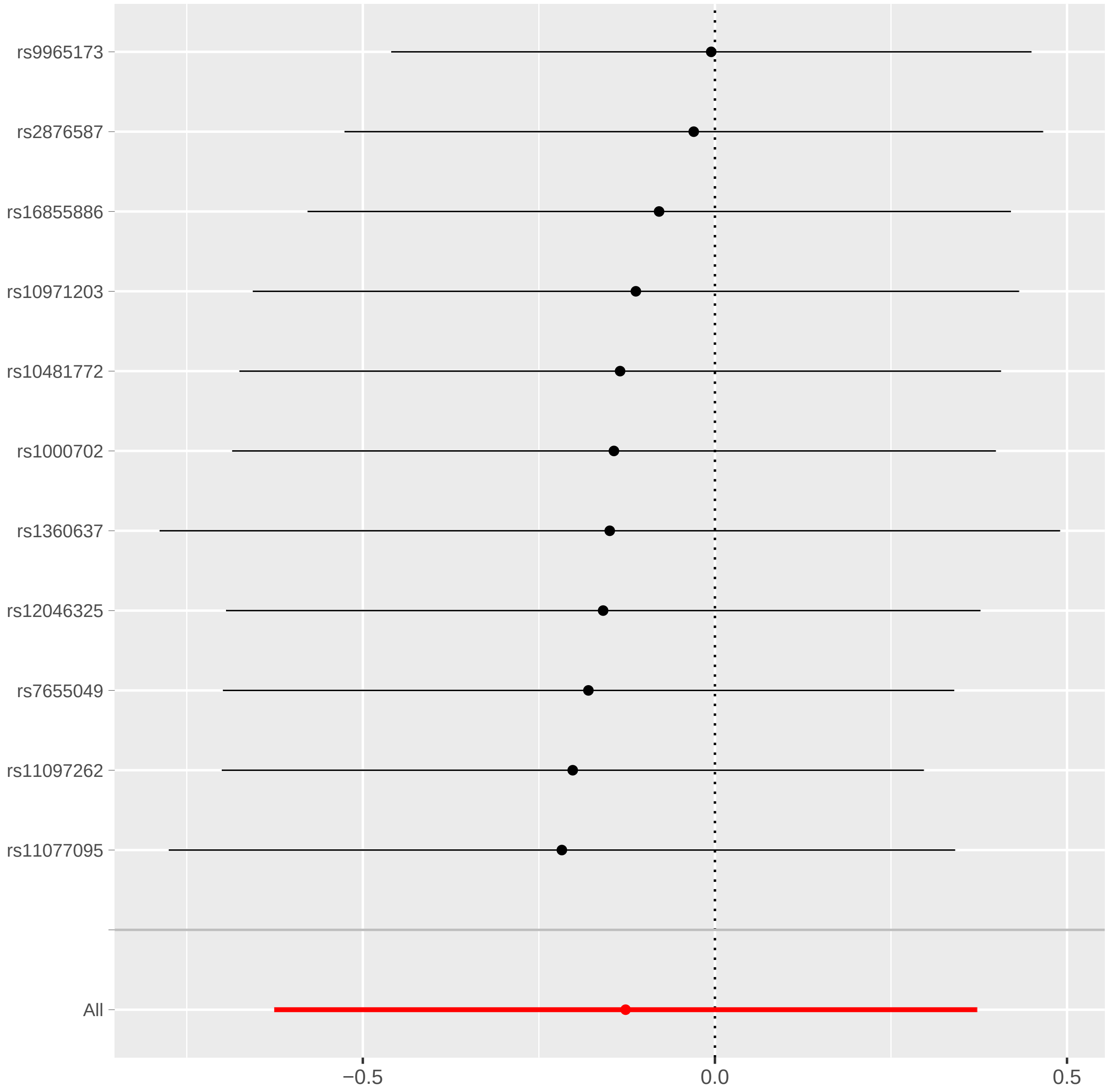

MR leave−one−out sensitivity analysis for
'M34329.metal.pos.txt.gz' on 'JUVEN_ARTHR.gz'

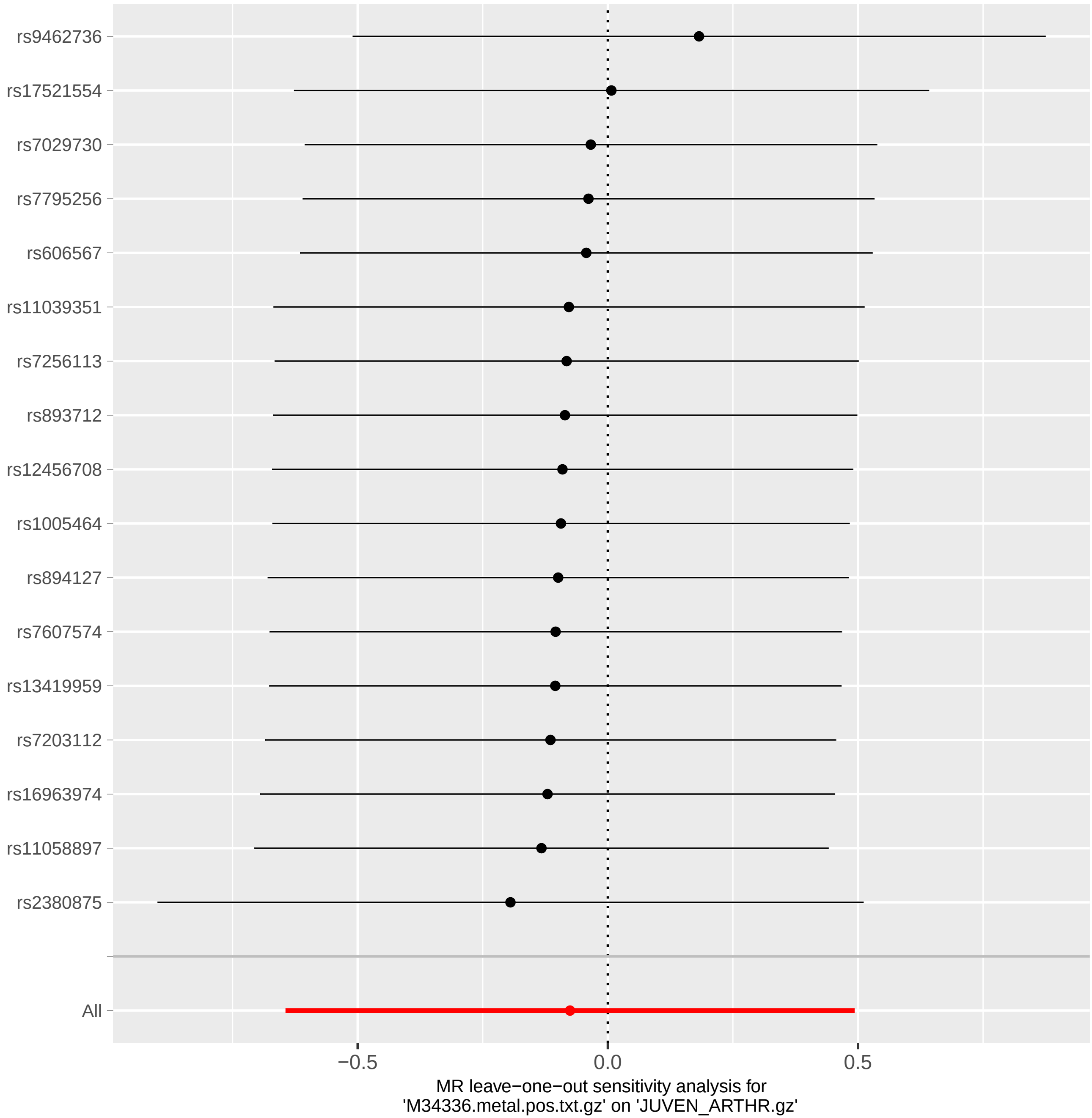

rs9462736
rs17521554
rs7029730
rs7795256
rs606567
rs11039351
rs7256113
rs893712
rs12456708
rs1005464
rs894127
rs7607574
rs13419959
rs7203112
rs16963974
rs11058897
rs2380875
All
−0.5
0.0
0.5
MR leave−one−out sensitivity analysis for
'M34336.metal.pos.txt.gz' on 'JUVEN_ARTHR.gz'

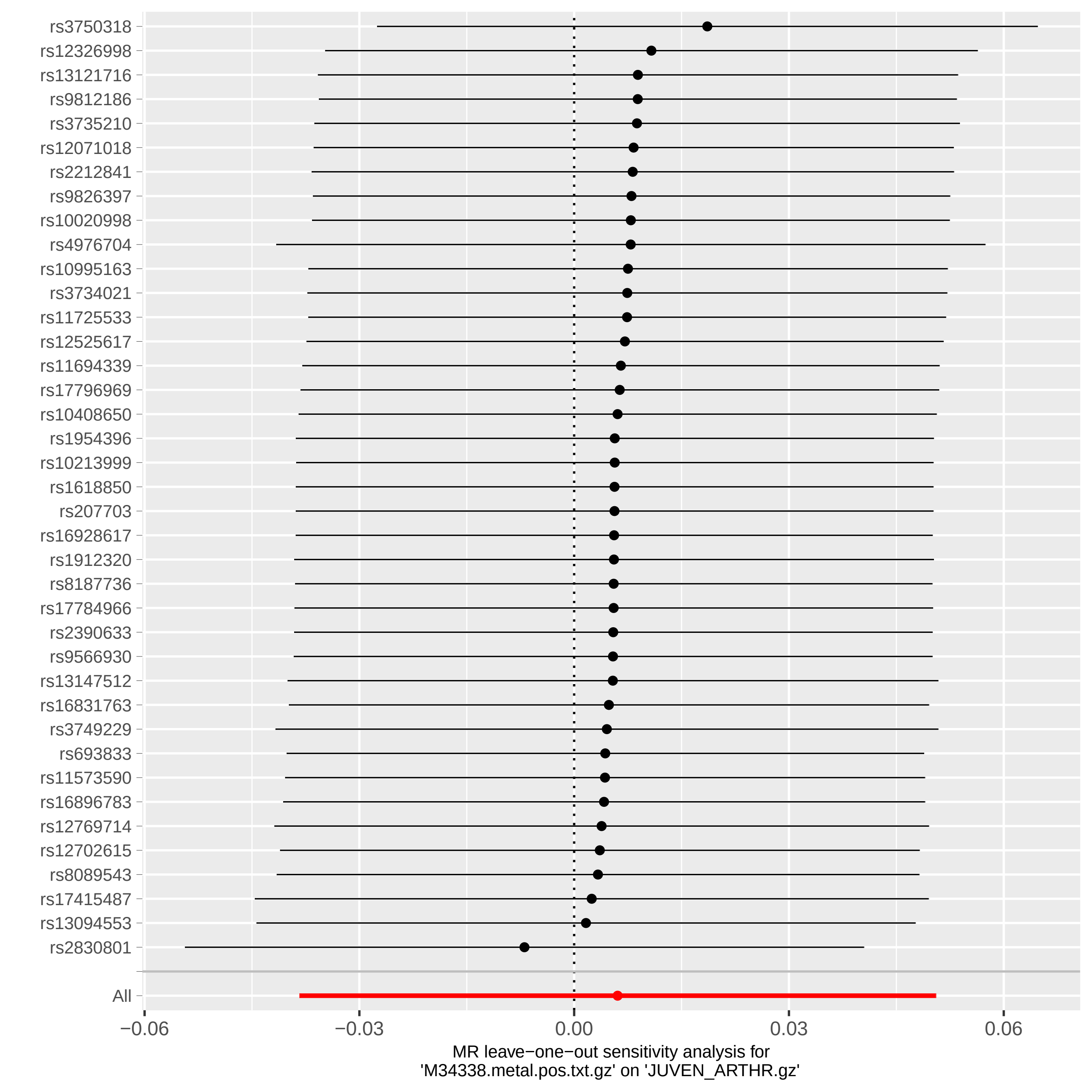

rs3750318
rs12326998
rs13121716
rs9812186
rs3735210
rs12071018
rs2212841
rs9826397
rs10020998
rs4976704
rs10995163
rs3734021
rs11725533
rs12525617
rs11694339
rs17796969
rs10408650
rs1954396
rs10213999
rs1618850
rs207703
rs16928617
rs1912320
rs8187736
rs17784966
rs2390633
rs9566930
rs13147512
rs16831763
rs3749229
rs693833
rs11573590
rs16896783
rs12769714
rs12702615
rs8089543
rs17415487
rs13094553
rs2830801
All
−0.06
−0.03
0.00
0.03
0.06
MR leave−one−out sensitivity analysis for
'M34338.metal.pos.txt.gz' on 'JUVEN_ARTHR.gz'

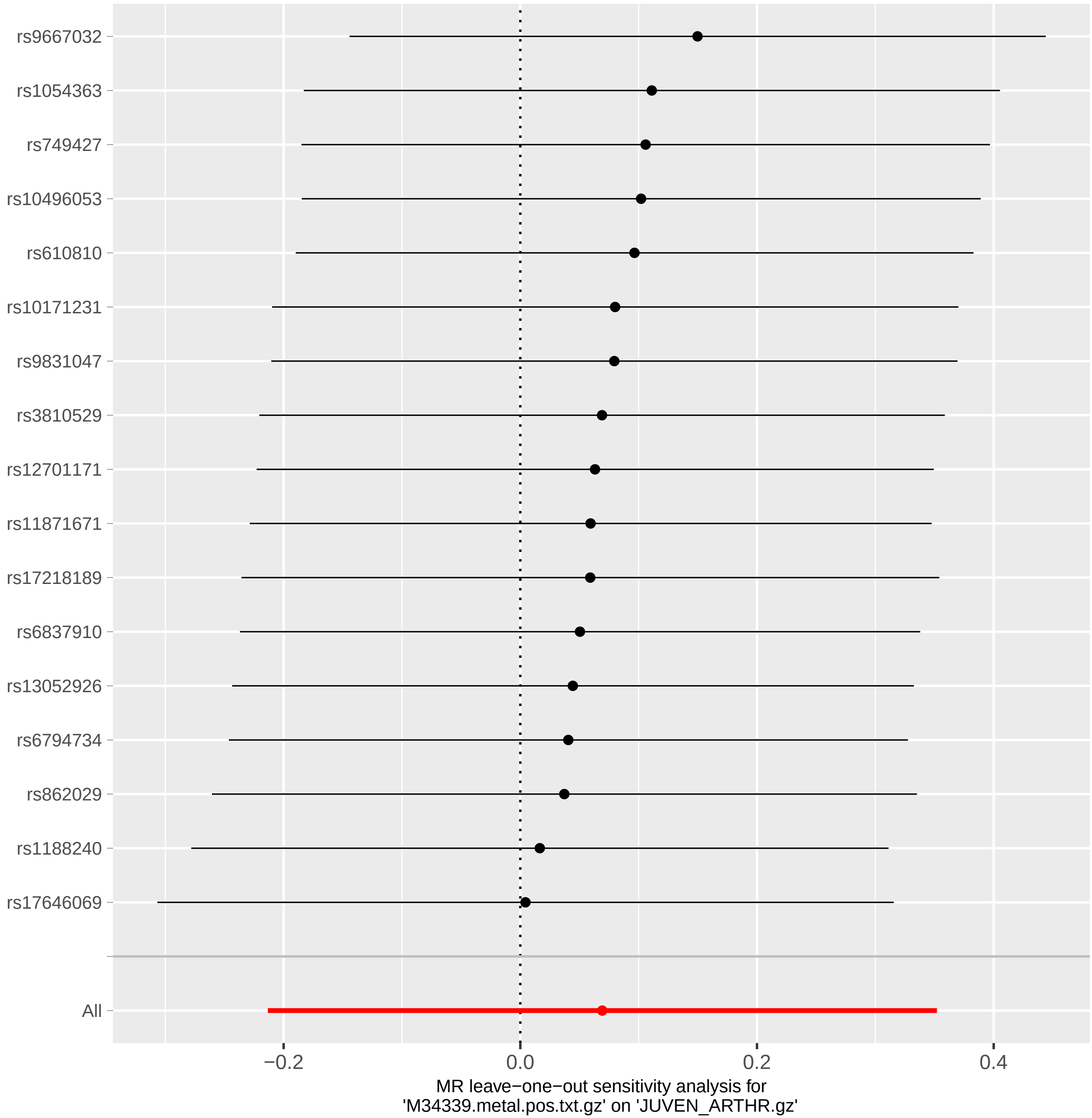
rs9667032
rs1054363
rs749427
rs10496053
rs610810
rs10171231
rs9831047
rs3810529
rs12701171
rs11871671
rs17218189
rs6837910
rs13052926
rs6794734
rs862029
rs1188240
rs17646069
All
−0.2
0.0
0.2
0.4
MR leave−one−out sensitivity analysis for
'M34339.metal.pos.txt.gz' on 'JUVEN_ARTHR.gz'

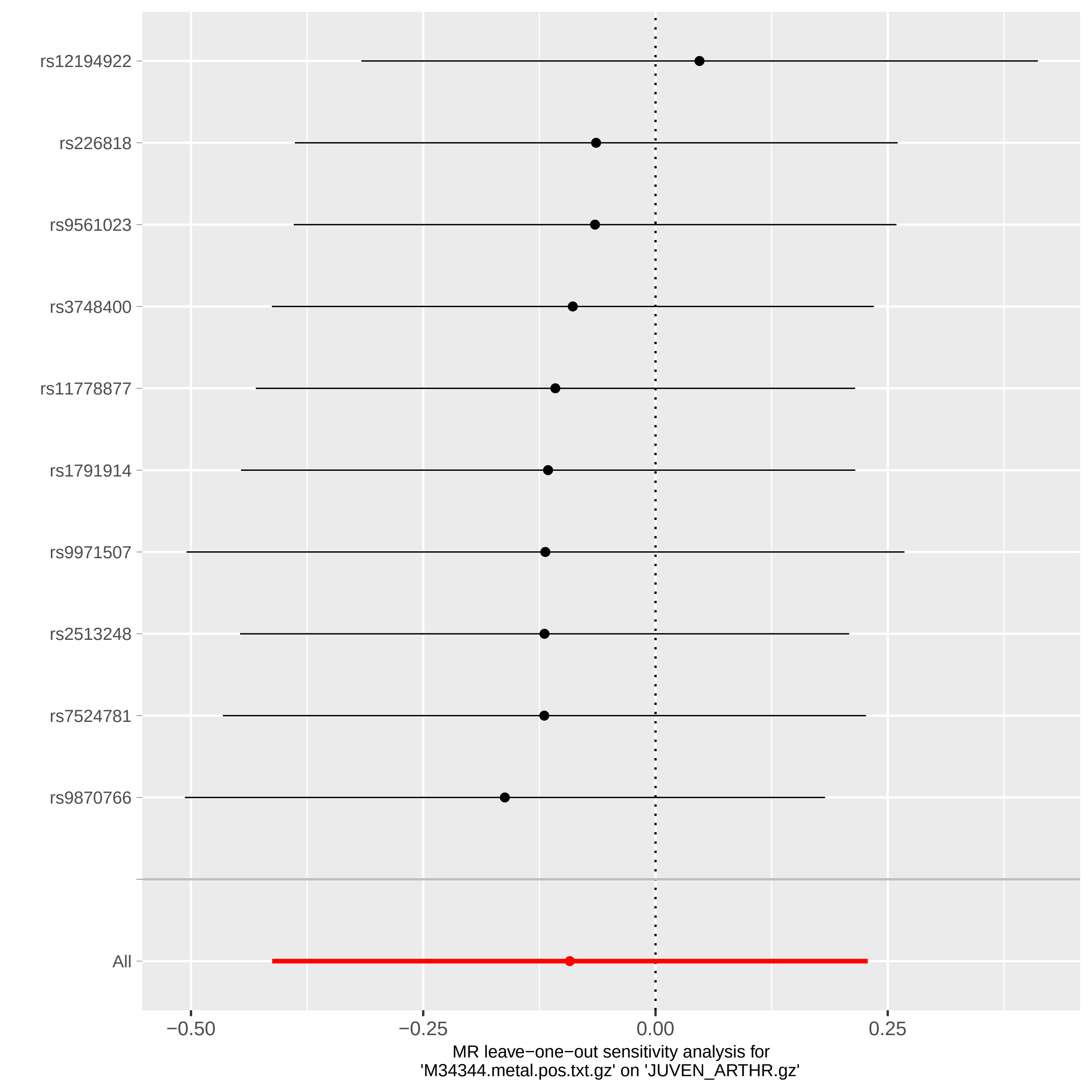
rs12194922
rs226818
rs9561023
rs3748400
rs11778877
rs1791914
rs9971507
rs2513248
rs7524781
rs9870766
All
−0.50
−0.25
0.00
0.25
MR leave−one−out sensitivity analysis for
'M34344.metal.pos.txt.gz' on 'JUVEN_ARTHR.gz'

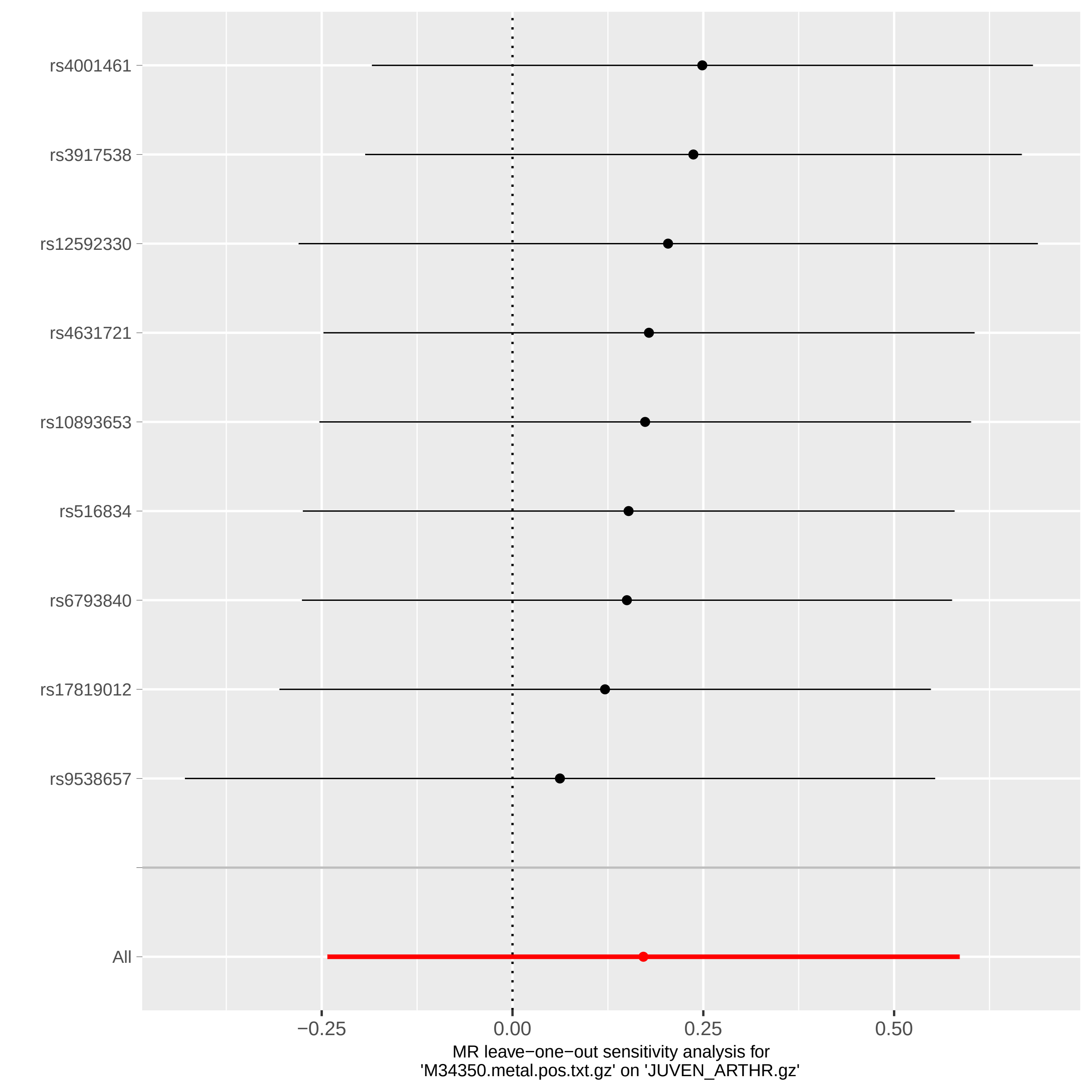
rs4001461
rs3917538
rs12592330
rs4631721
rs10893653
rs516834
rs6793840
rs17819012
rs9538657
All
−0.25
0.00
0.25
0.50
MR leave−one−out sensitivity analysis for
'M34350.metal.pos.txt.gz' on 'JUVEN_ARTHR.gz'

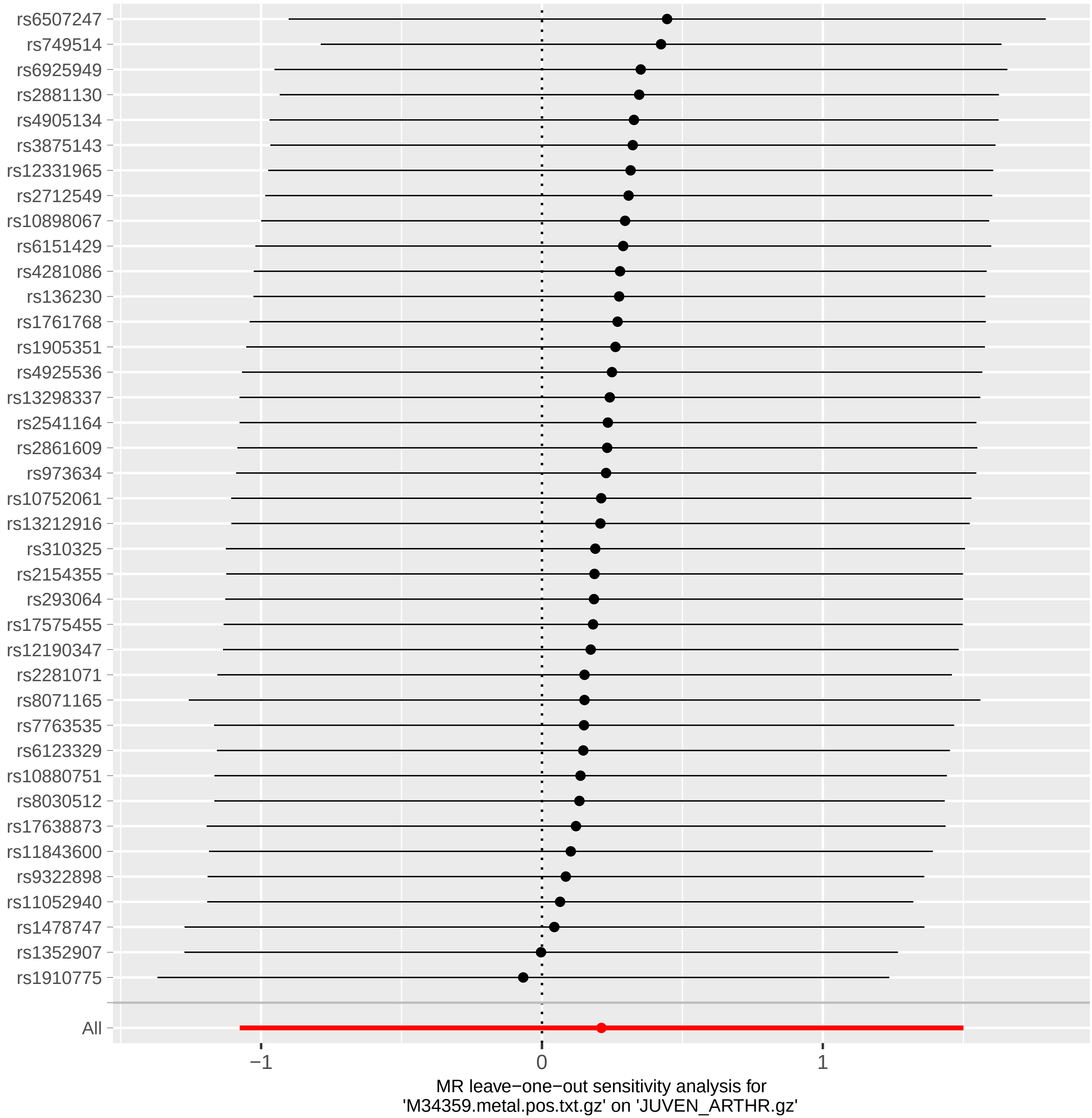
rs6507247
rs749514
rs6925949
rs2881130
rs4905134
rs3875143
rs12331965
rs2712549
rs10898067
rs6151429
rs4281086
rs136230
rs1761768
rs1905351
rs4925536
rs13298337
rs2541164
rs2861609
rs973634
rs10752061
rs13212916
rs310325
rs2154355
rs293064
rs17575455
rs12190347
rs2281071
rs8071165
rs7763535
rs6123329
rs10880751
rs8030512
rs17638873
rs11843600
rs9322898
rs11052940
rs1478747
rs1352907
rs1910775
All
−1
0
1
MR leave−one−out sensitivity analysis for
'M34359.metal.pos.txt.gz' on 'JUVEN_ARTHR.gz'

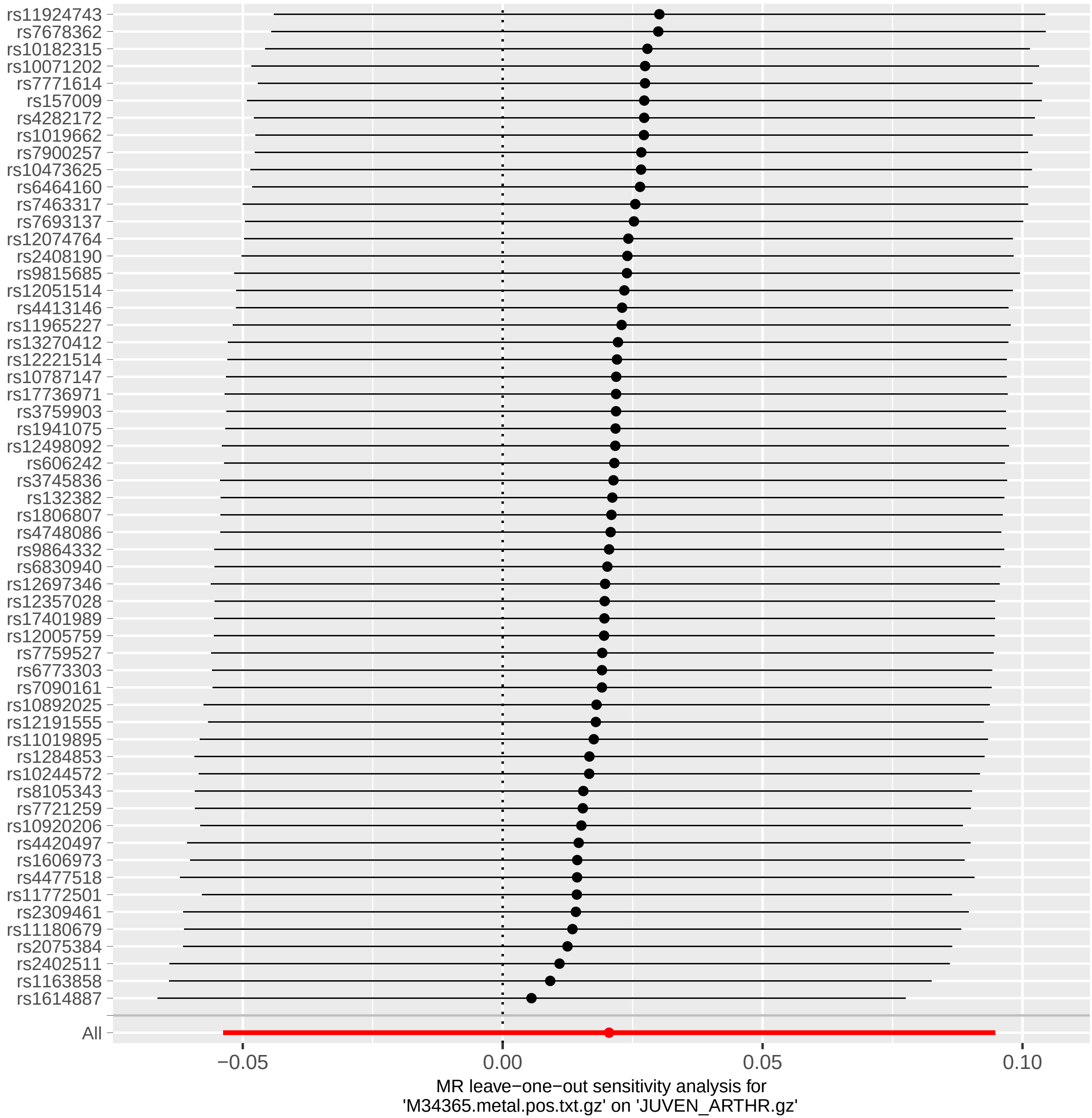
rs11924743
rs7678362
rs10182315
rs10071202
rs7771614
rs157009
rs4282172
rs1019662
rs7900257
rs10473625
rs6464160
rs7463317
rs7693137
rs12074764
rs2408190
rs9815685
rs12051514
rs4413146
rs11965227
rs13270412
rs12221514
rs10787147
rs17736971
rs3759903
rs1941075
rs12498092
rs606242
rs3745836
rs132382
rs1806807
rs4748086
rs9864332
rs6830940
rs12697346
rs12357028
rs17401989
rs12005759
rs7759527
rs6773303
rs7090161
rs10892025
rs12191555
rs11019895
rs1284853
rs10244572
rs8105343
rs7721259
rs10920206
rs4420497
rs1606973
rs4477518
rs11772501
rs2309461
rs11180679
rs2075384
rs2402511
rs1163858
rs1614887
All
−0.05
0.00
0.05
0.10
MR leave−one−out sensitivity analysis for
'M34365.metal.pos.txt.gz' on 'JUVEN_ARTHR.gz'

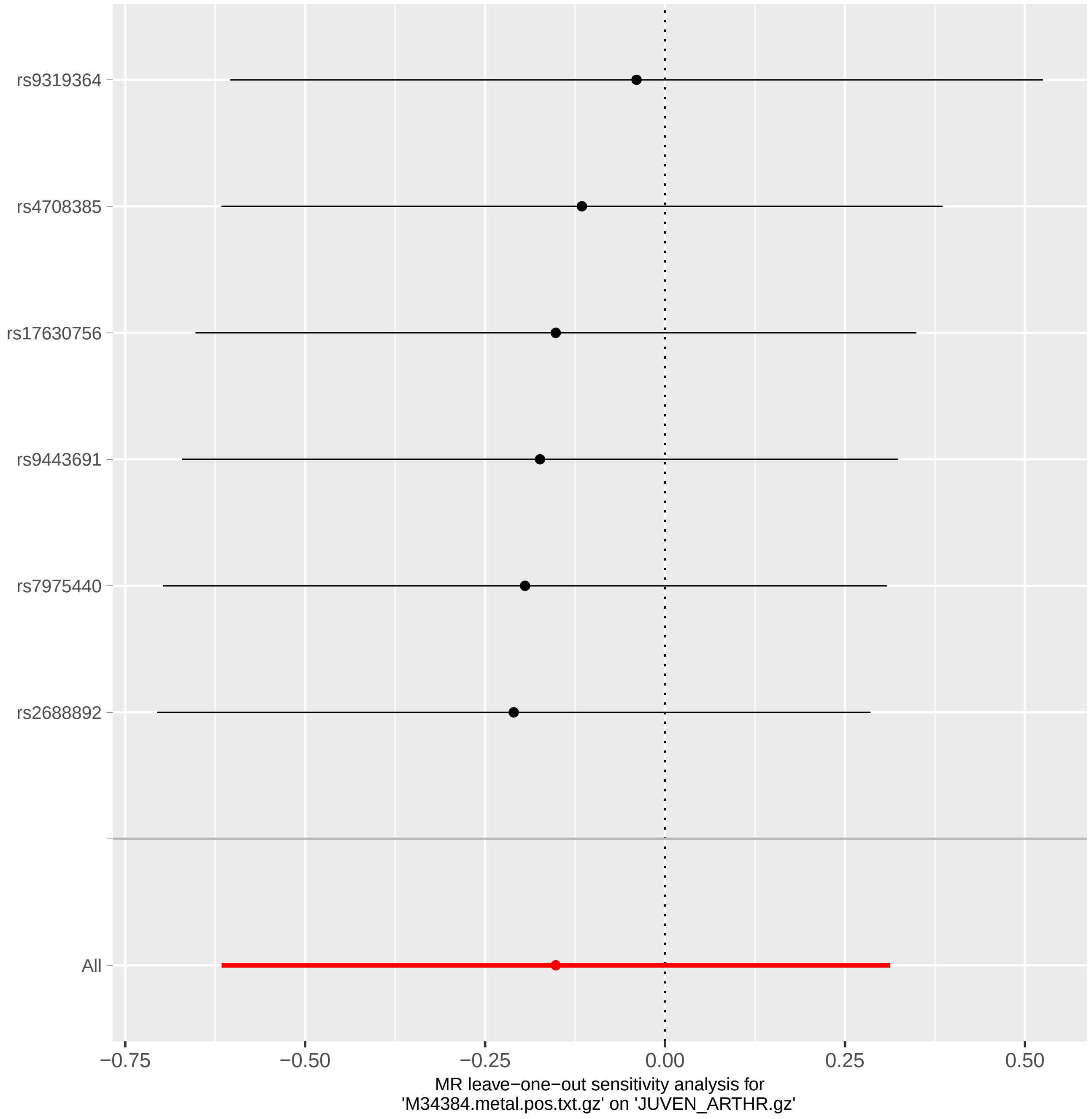
rs9319364
rs4708385
rs17630756
rs9443691
rs7975440
rs2688892
All
−0.75
−0.50
−0.25
0.00
0.25
0.50
MR leave−one−out sensitivity analysis for
'M34384.metal.pos.txt.gz' on 'JUVEN_ARTHR.gz'

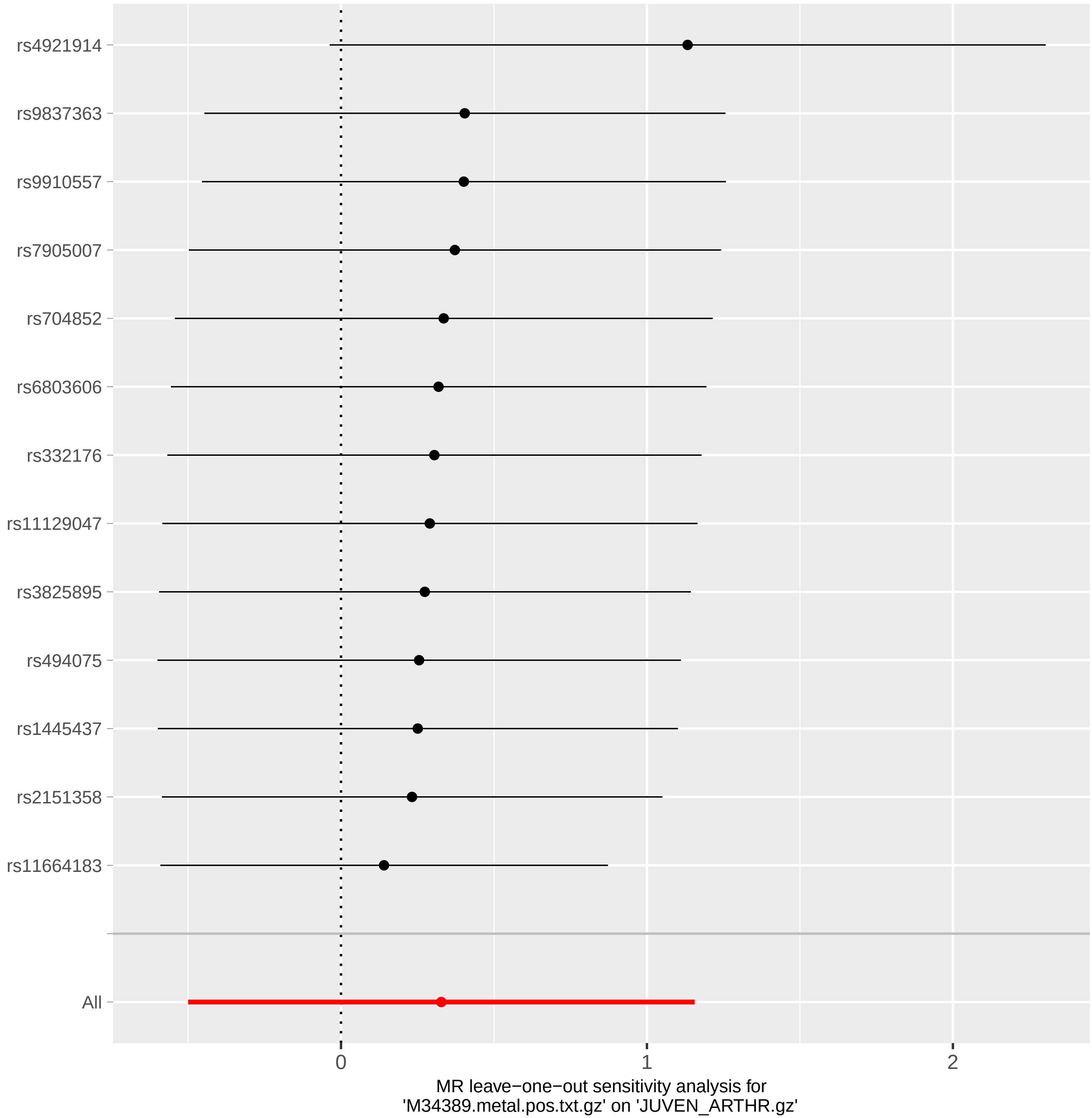
rs4921914
rs9837363
rs9910557
rs7905007
rs704852
rs6803606
rs332176
rs11129047
rs3825895
rs494075
rs1445437
rs2151358
rs11664183
All
0
1
2
MR leave−one−out sensitivity analysis for
'M34389.metal.pos.txt.gz' on 'JUVEN_ARTHR.gz'

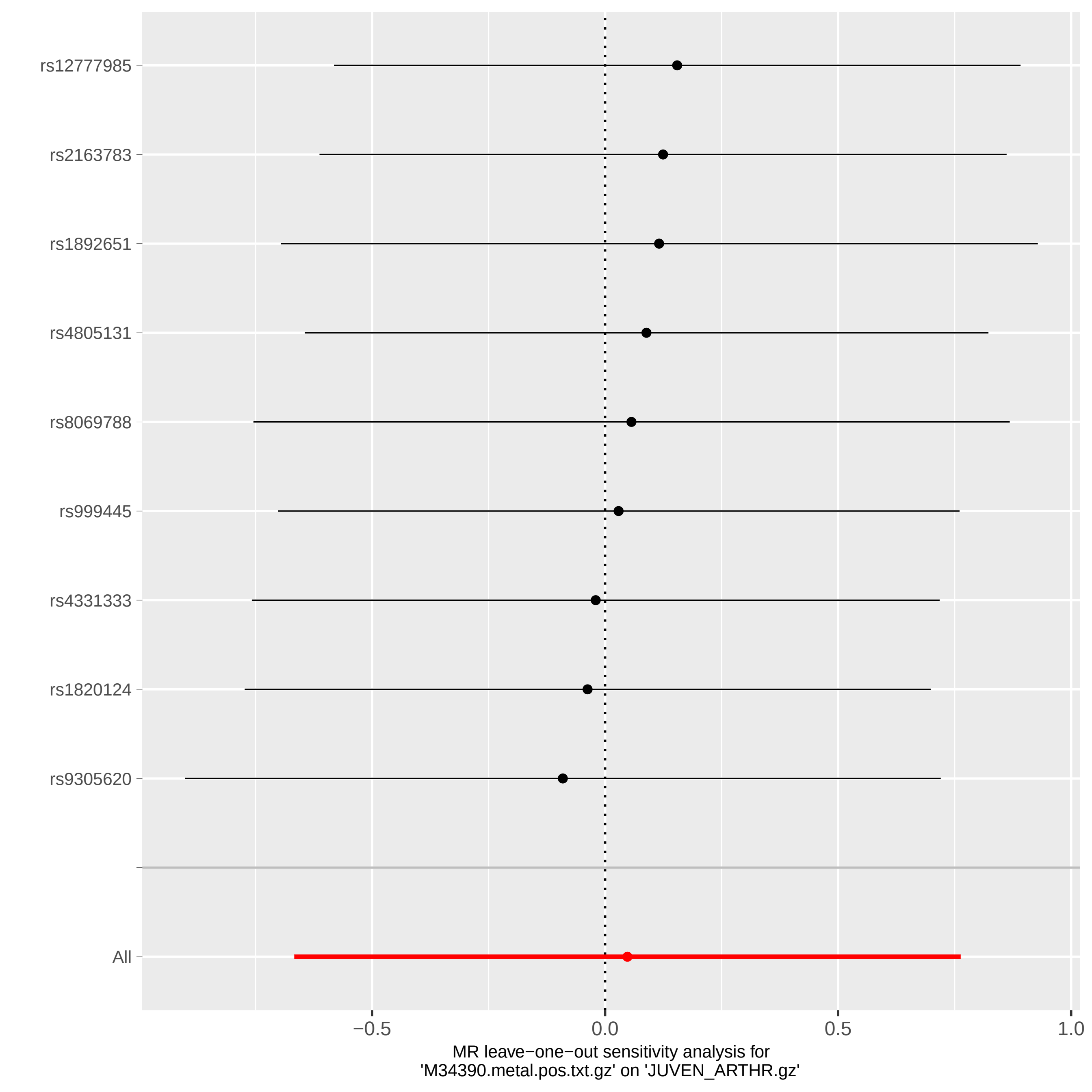

rs12777985
rs2163783
rs1892651
rs4805131
rs8069788
rs999445
rs4331333
rs1820124
rs9305620
All
−0.5
0.0
0.5
1.0
MR leave−one−out sensitivity analysis for
'M34390.metal.pos.txt.gz' on 'JUVEN_ARTHR.gz'

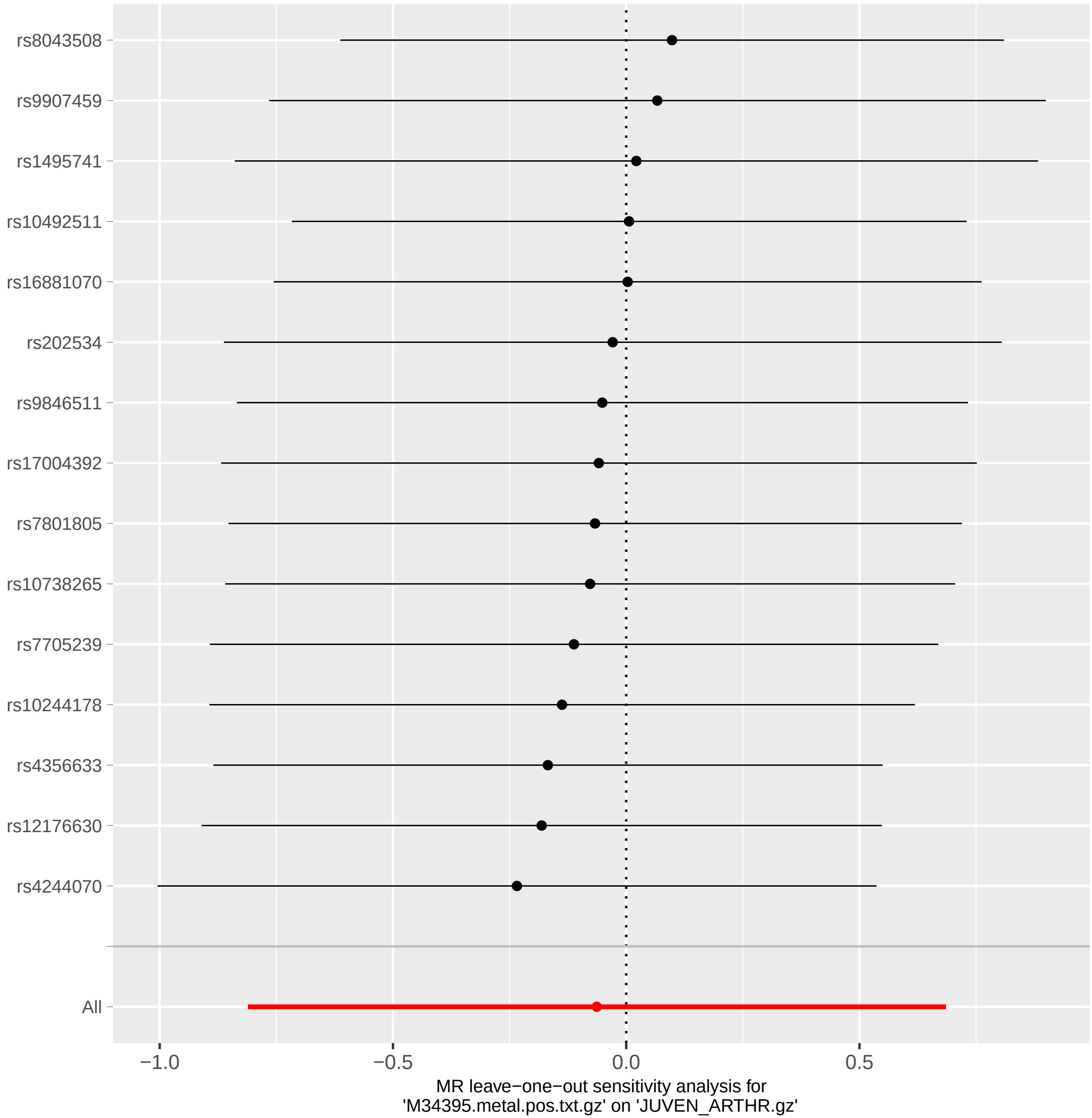
rs8043508
rs9907459
rs1495741
rs10492511
rs16881070
rs202534
rs9846511
rs17004392
rs7801805
rs10738265
rs7705239
rs10244178
rs4356633
rs12176630
rs4244070
All
−1.0
−0.5
0.0
0.5
MR leave−one−out sensitivity analysis for
'M34395.metal.pos.txt.gz' on 'JUVEN_ARTHR.gz'

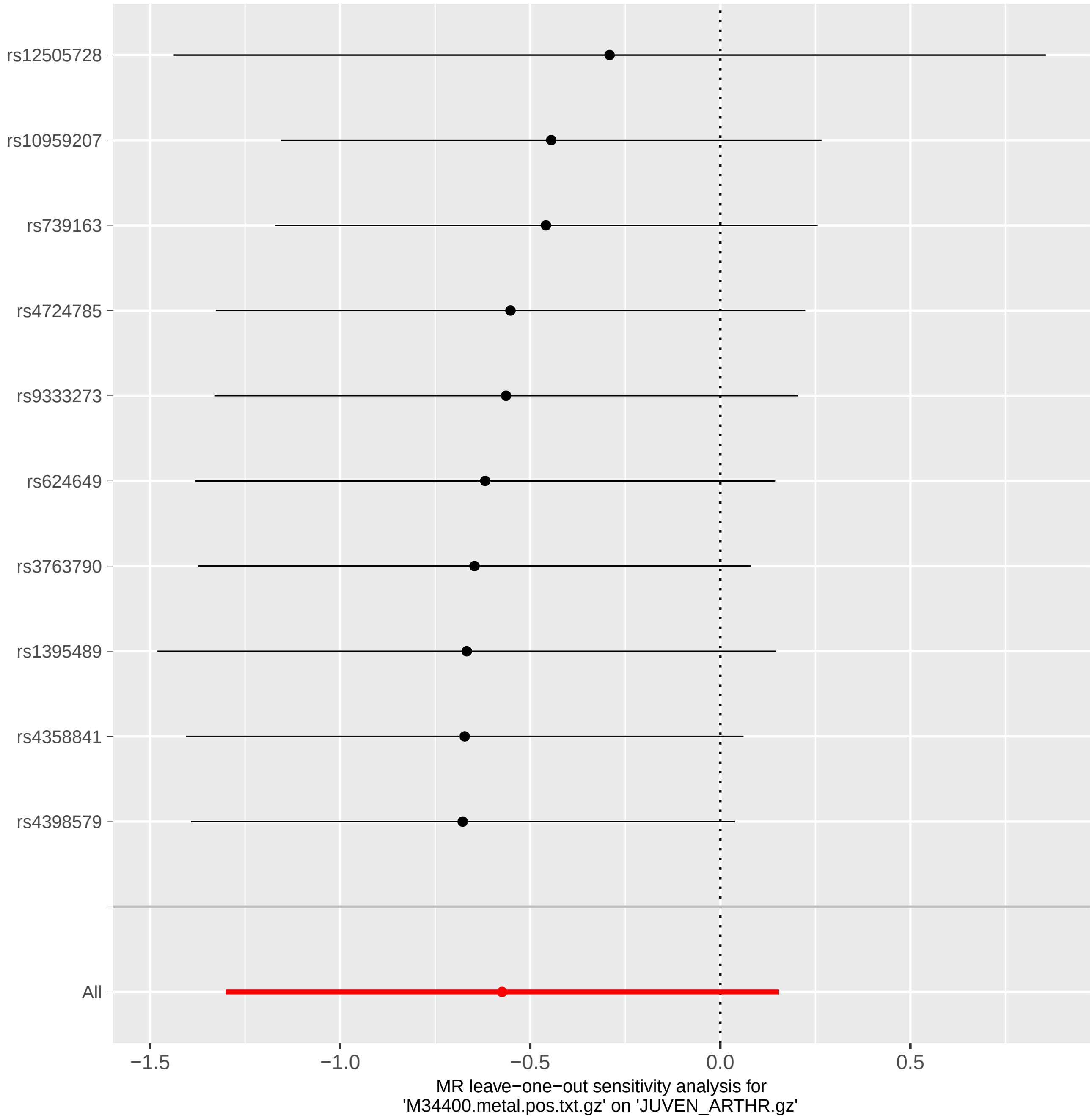
rs12505728
rs10959207
rs739163
rs4724785
rs9333273
rs624649
rs3763790
rs1395489
rs4358841
rs4398579
All
−1.5
−1.0
−0.5
0.0
0.5
MR leave−one−out sensitivity analysis for
'M34400.metal.pos.txt.gz' on 'JUVEN_ARTHR.gz'

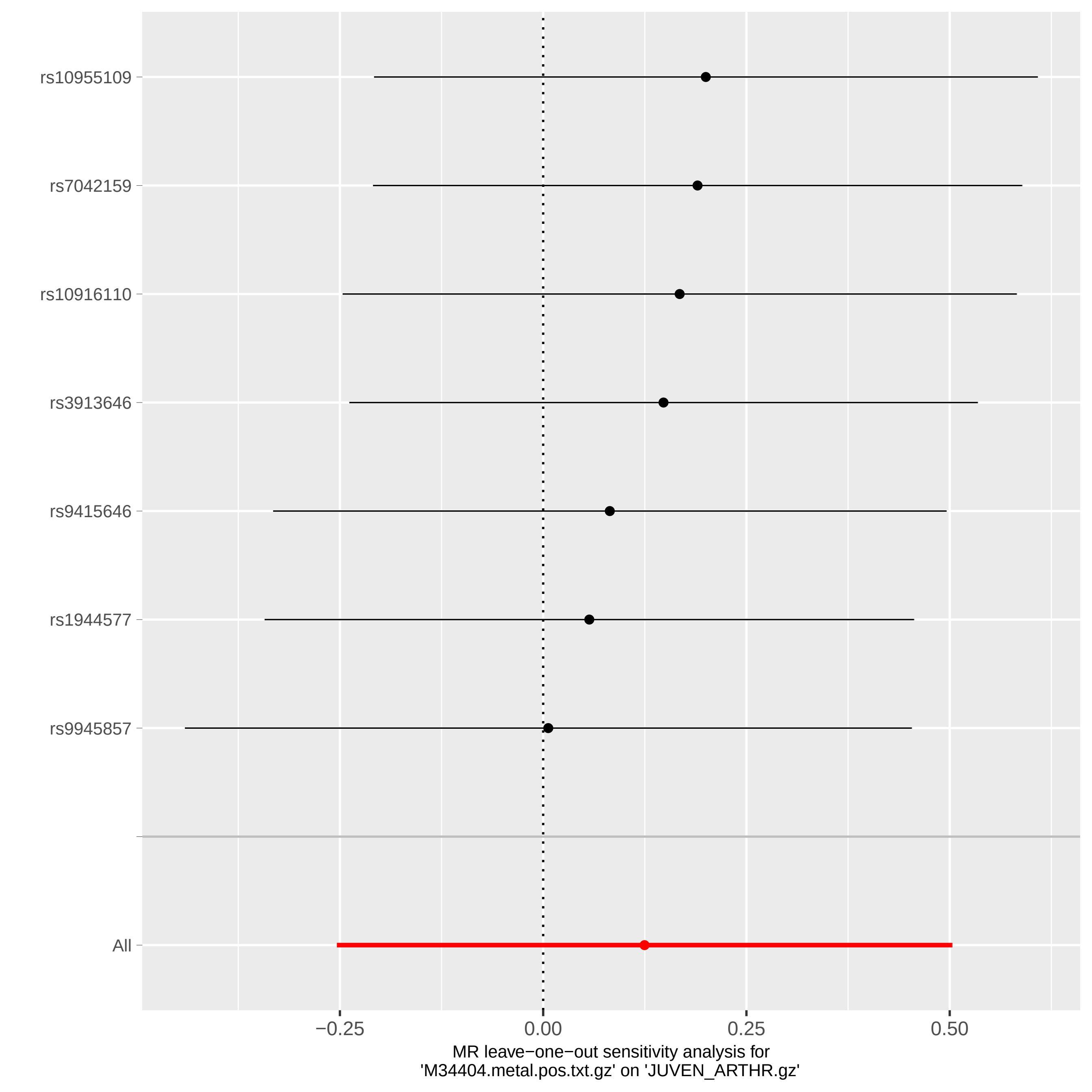
rs10955109
rs7042159
rs10916110
rs3913646
rs9415646
rs1944577
rs9945857
All
−0.25
0.00
0.25
0.50
MR leave−one−out sensitivity analysis for
'M34404.metal.pos.txt.gz' on 'JUVEN_ARTHR.gz'

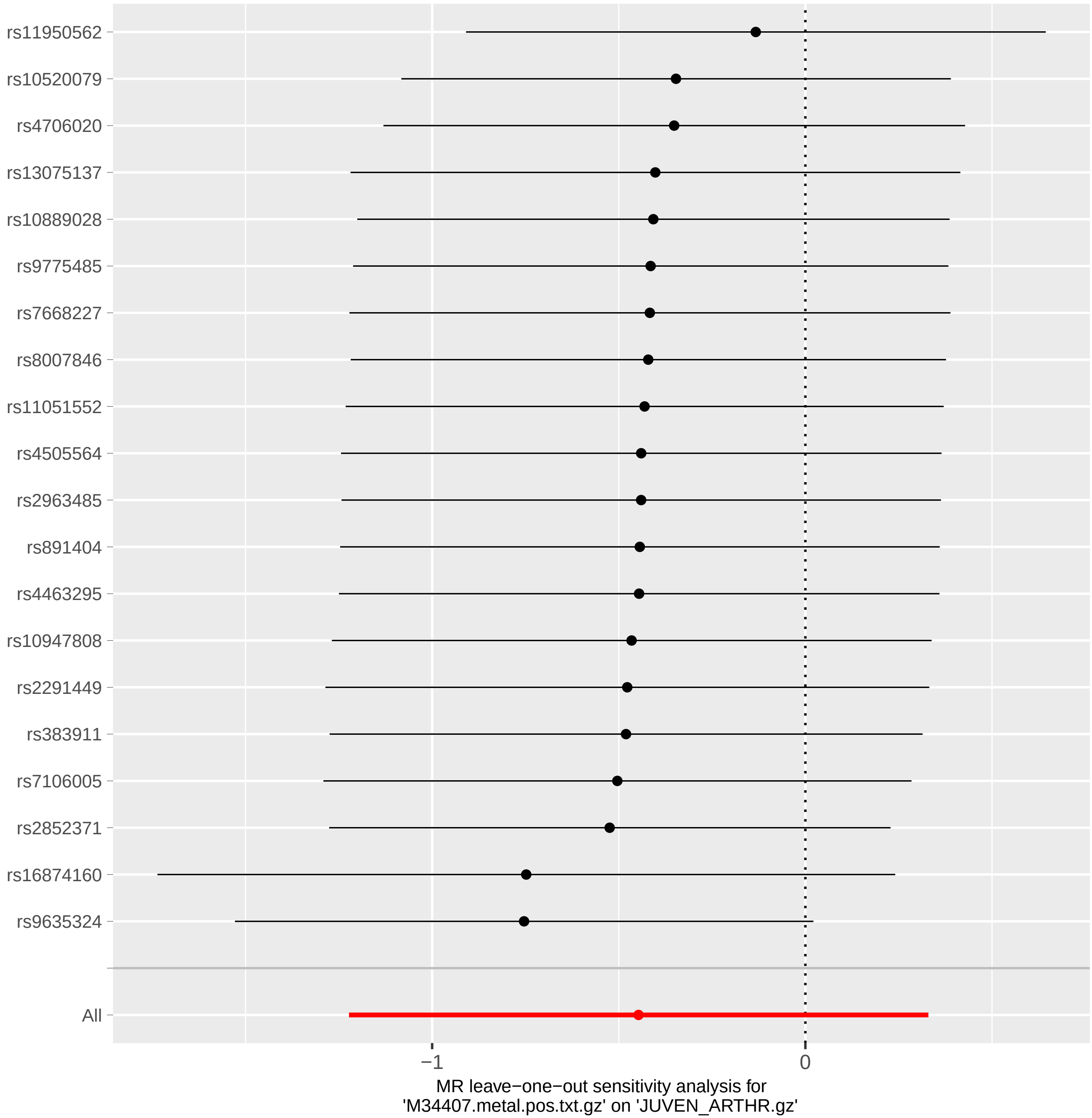

rs11950562
rs10520079
rs4706020
rs13075137
rs10889028
rs9775485
rs7668227
rs8007846
rs11051552
rs4505564
rs2963485
rs891404
rs4463295
rs10947808
rs2291449
rs383911
rs7106005
rs2852371
rs16874160
rs9635324
All
−1
0
MR leave−one−out sensitivity analysis for
'M34407.metal.pos.txt.gz' on 'JUVEN_ARTHR.gz'

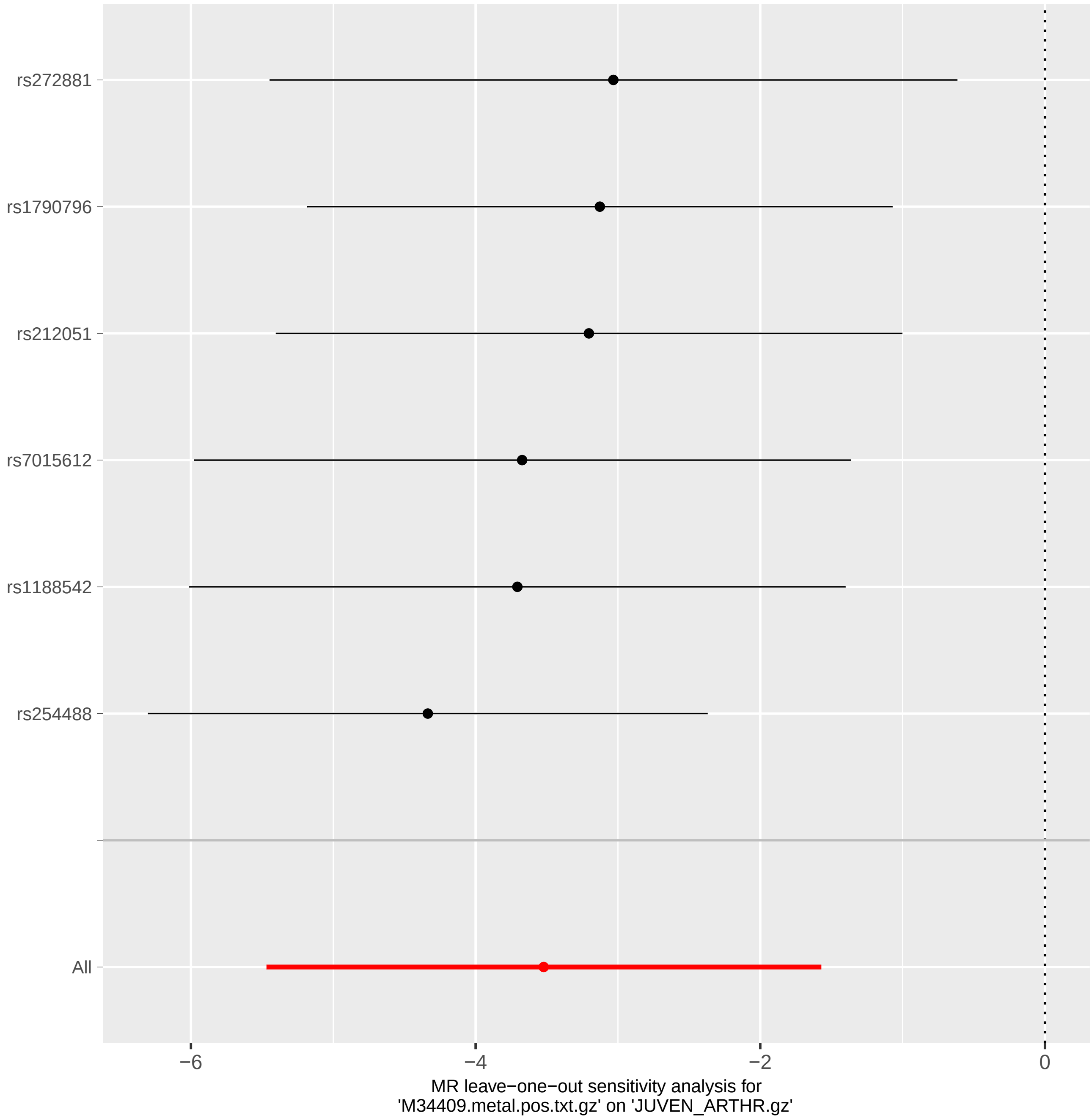
rs272881
rs1790796
rs212051
rs7015612
rs1188542
rs254488
All
−6
−4
−2
0
MR leave−one−out sensitivity analysis for
'M34409.metal.pos.txt.gz' on 'JUVEN_ARTHR.gz'

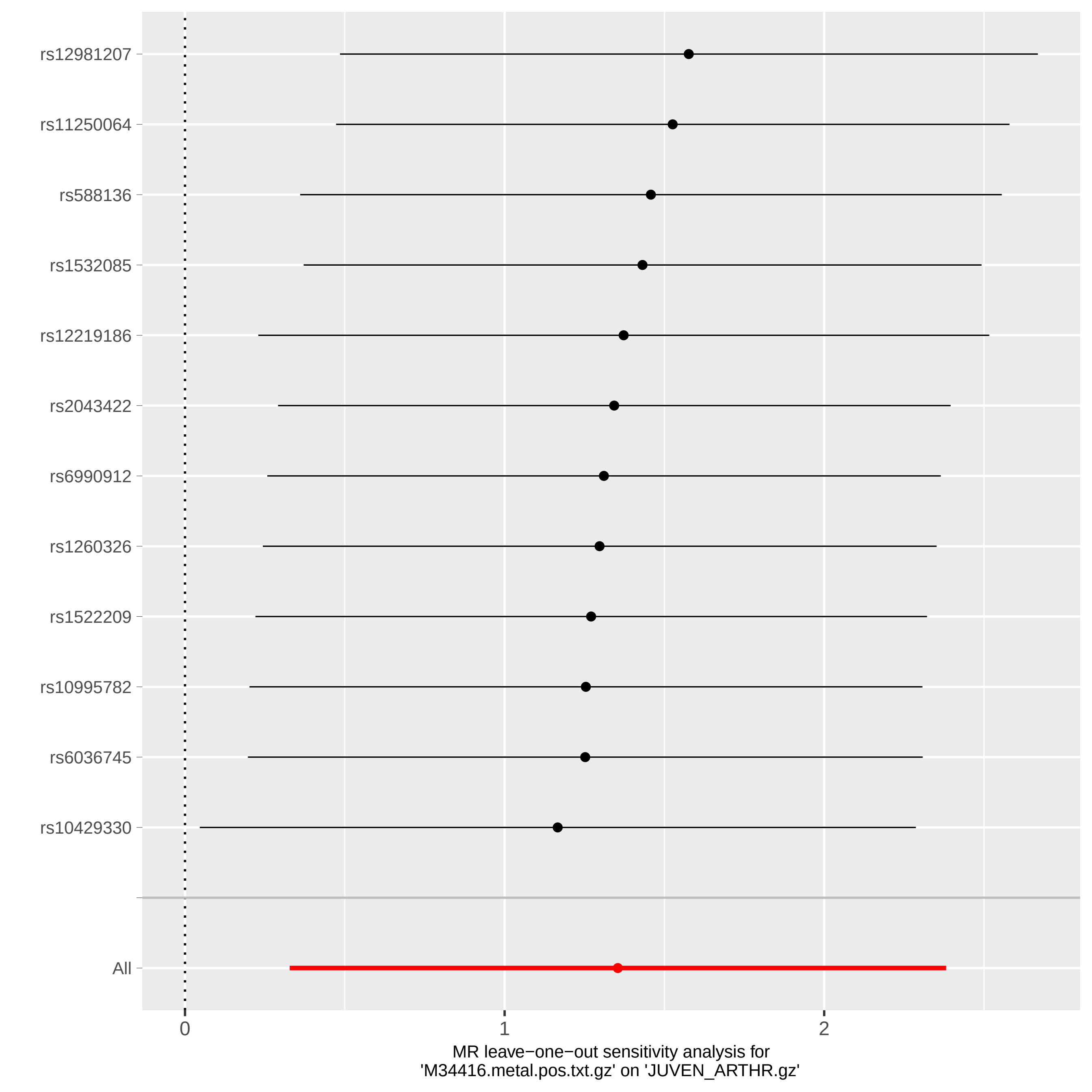
rs12981207
rs11250064
rs588136
rs1532085
rs12219186
rs2043422
rs6990912
rs1260326
rs1522209
rs10995782
rs6036745
rs10429330
All
0
1
2
MR leave−one−out sensitivity analysis for
'M34416.metal.pos.txt.gz' on 'JUVEN_ARTHR.gz'

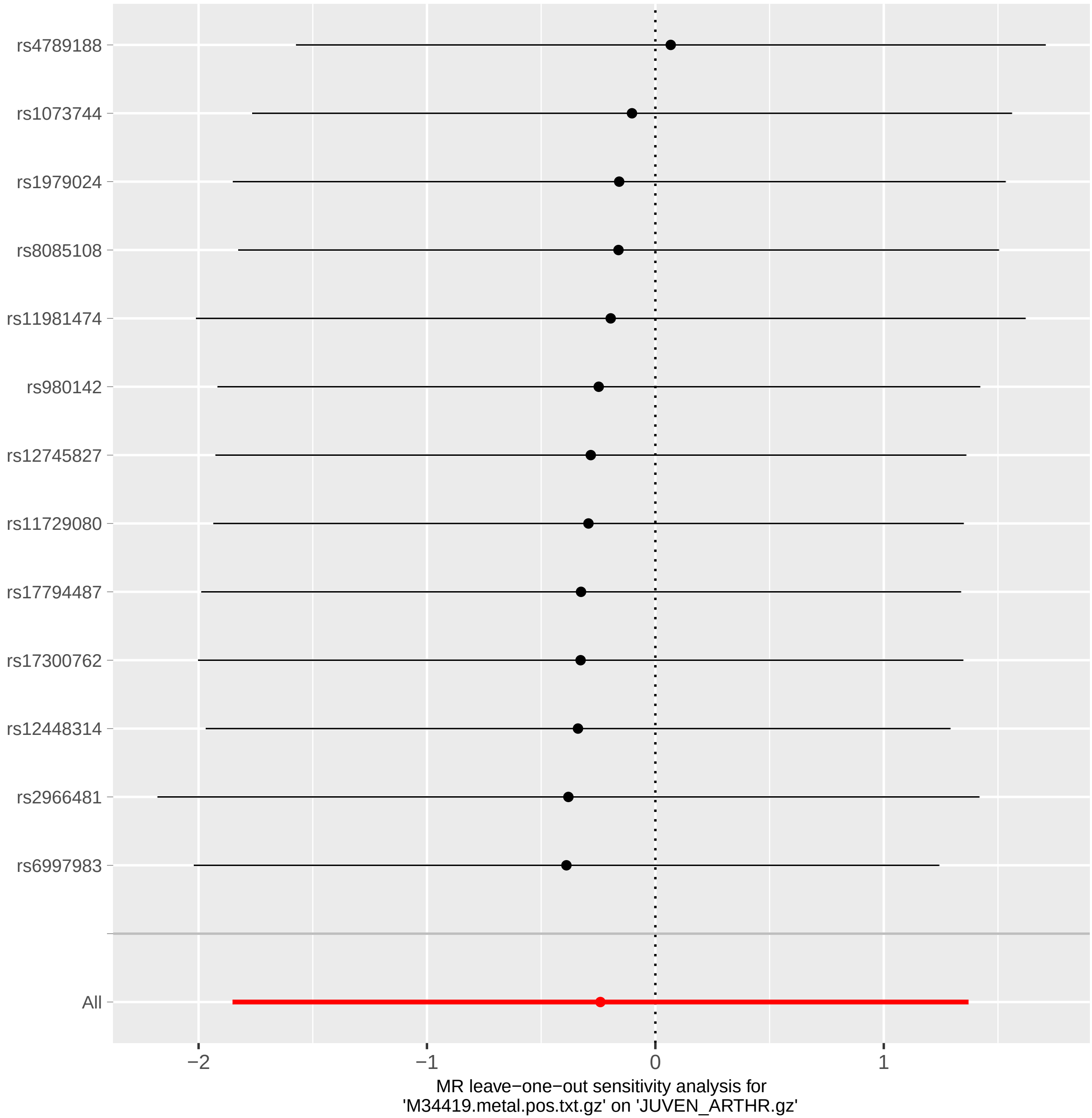

rs4789188
rs1073744
rs1979024
rs8085108
rs11981474
rs980142
rs12745827
rs11729080
rs17794487
rs17300762
rs12448314
rs2966481
rs6997983
All
−2
−1
0
1
MR leave−one−out sensitivity analysis for
'M34419.metal.pos.txt.gz' on 'JUVEN_ARTHR.gz'

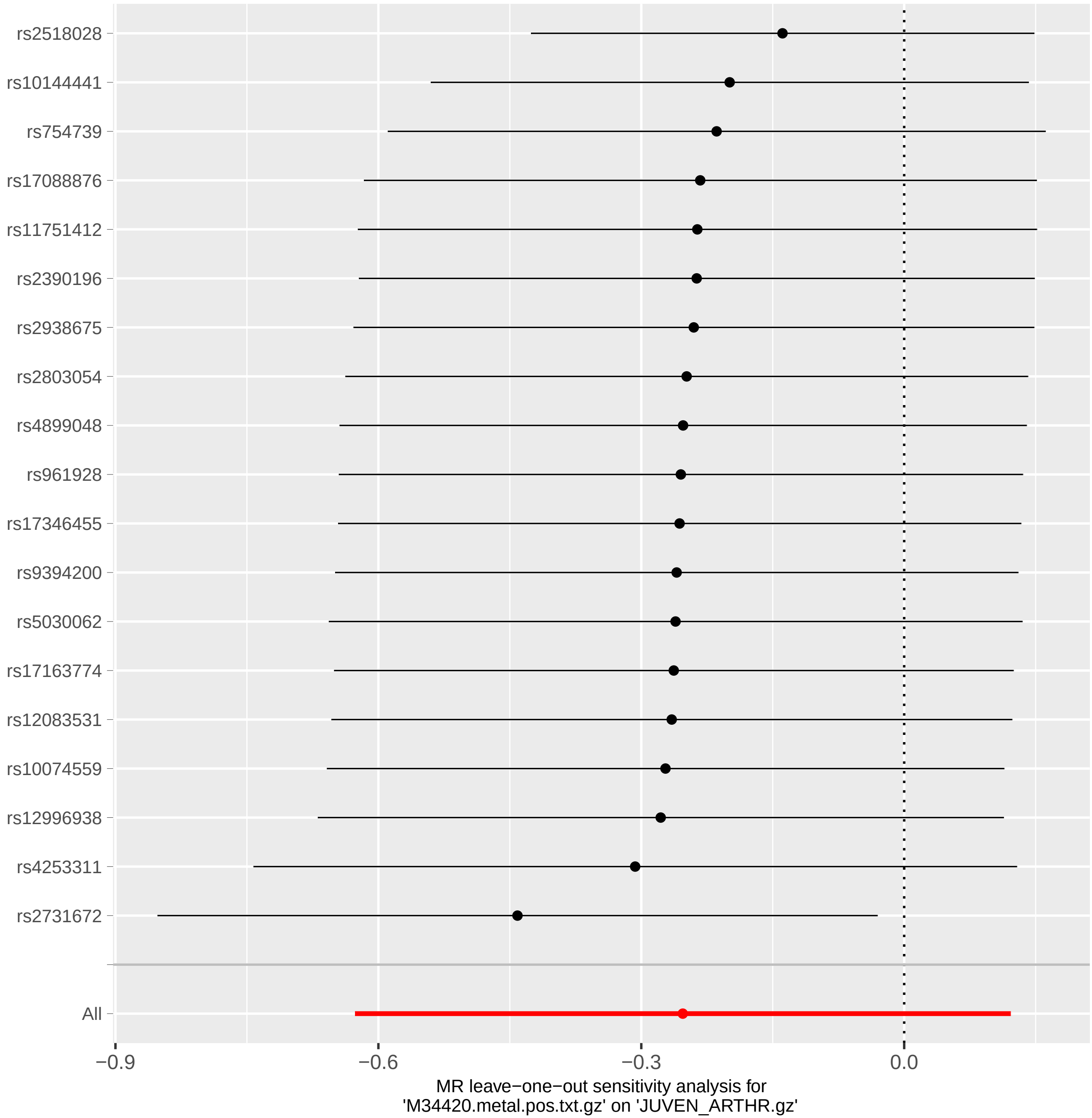
rs2518028
rs10144441
rs754739
rs17088876
rs11751412
rs2390196
rs2938675
rs2803054
rs4899048
rs961928
rs17346455
rs9394200
rs5030062
rs17163774
rs12083531
rs10074559
rs12996938
rs4253311
rs2731672
All
−0.9
−0.6
−0.3
0.0
MR leave−one−out sensitivity analysis for
'M34420.metal.pos.txt.gz' on 'JUVEN_ARTHR.gz'

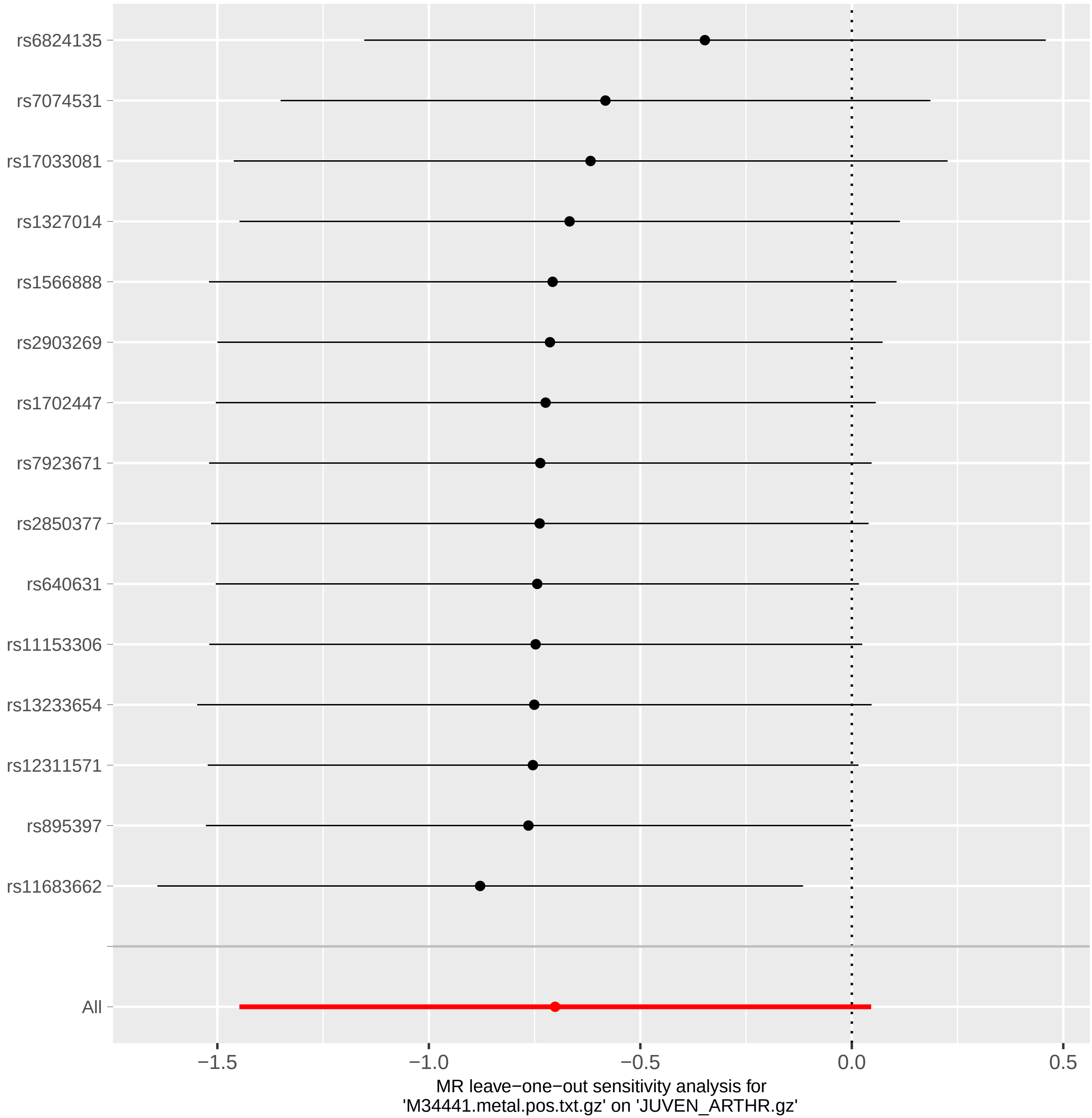

rs6824135
rs7074531
rs17033081
rs1327014
rs1566888
rs2903269
rs1702447
rs7923671
rs2850377
rs640631
rs11153306
rs13233654
rs12311571
rs895397
rs11683662
All
−1.5
−1.0
−0.5
0.0
0.5
MR leave−one−out sensitivity analysis for
'M34441.metal.pos.txt.gz' on 'JUVEN_ARTHR.gz'

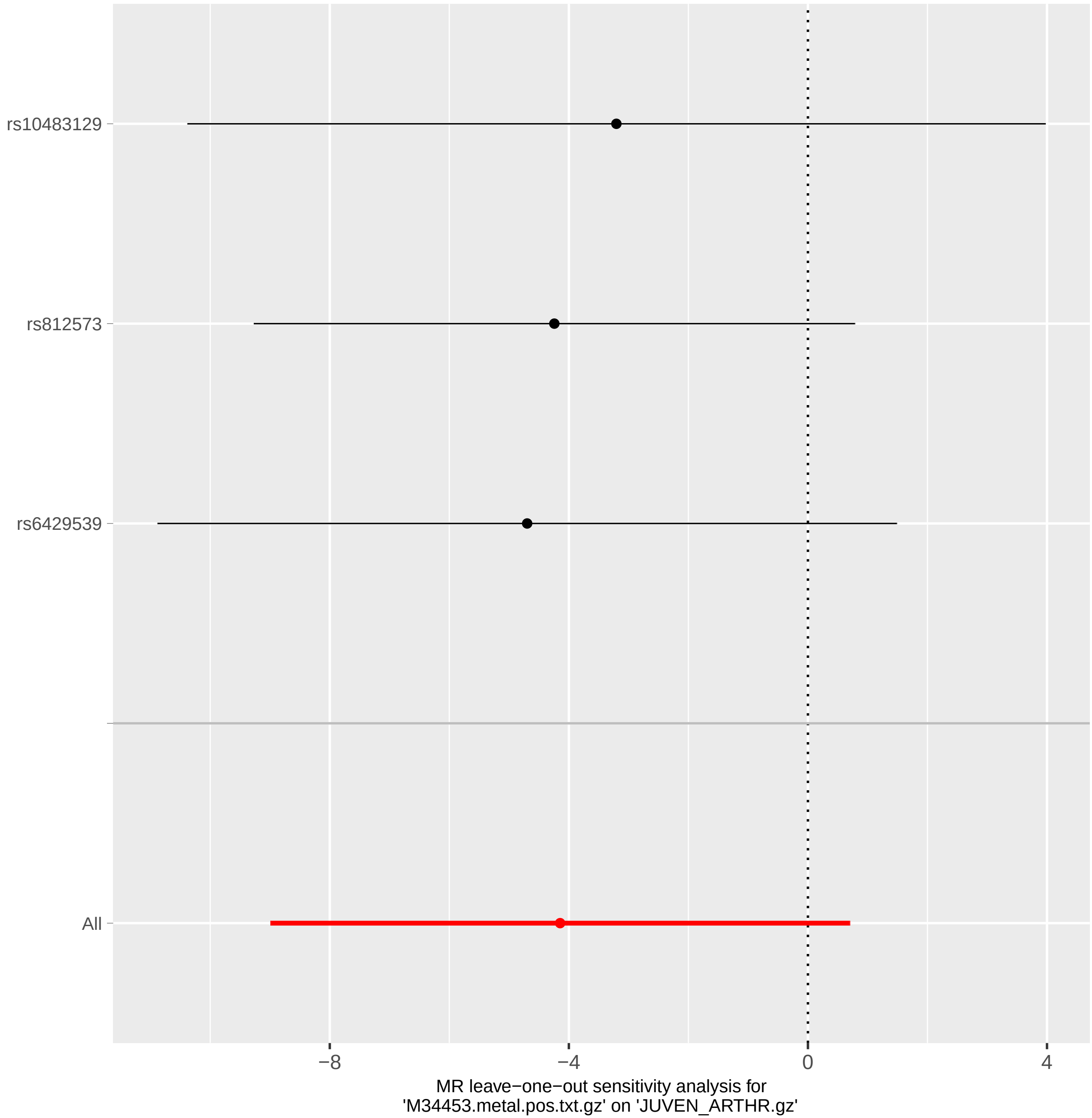
rs10483129
rs812573
rs6429539
All
−8
−4
0
4
MR leave−one−out sensitivity analysis for
'M34453.metal.pos.txt.gz' on 'JUVEN_ARTHR.gz'

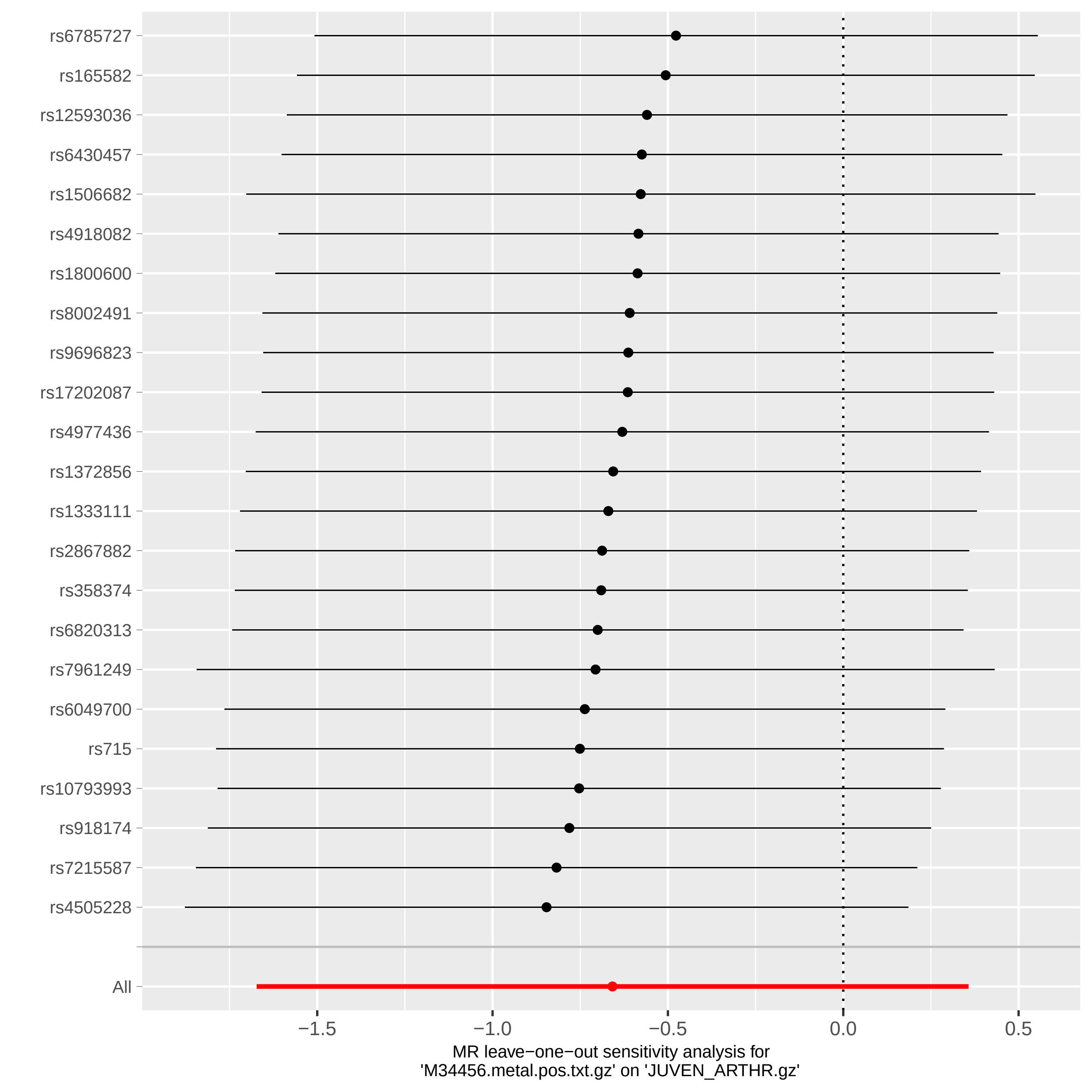
rs6785727
rs165582
rs12593036
rs6430457
rs1506682
rs4918082
rs1800600
rs8002491
rs9696823
rs17202087
rs4977436
rs1372856
rs1333111
rs2867882
rs358374
rs6820313
rs7961249
rs6049700
rs715
rs10793993
rs918174
rs7215587
rs4505228
All
−1.5
−1.0
−0.5
0.0
0.5
MR leave−one−out sensitivity analysis for
'M34456.metal.pos.txt.gz' on 'JUVEN_ARTHR.gz'

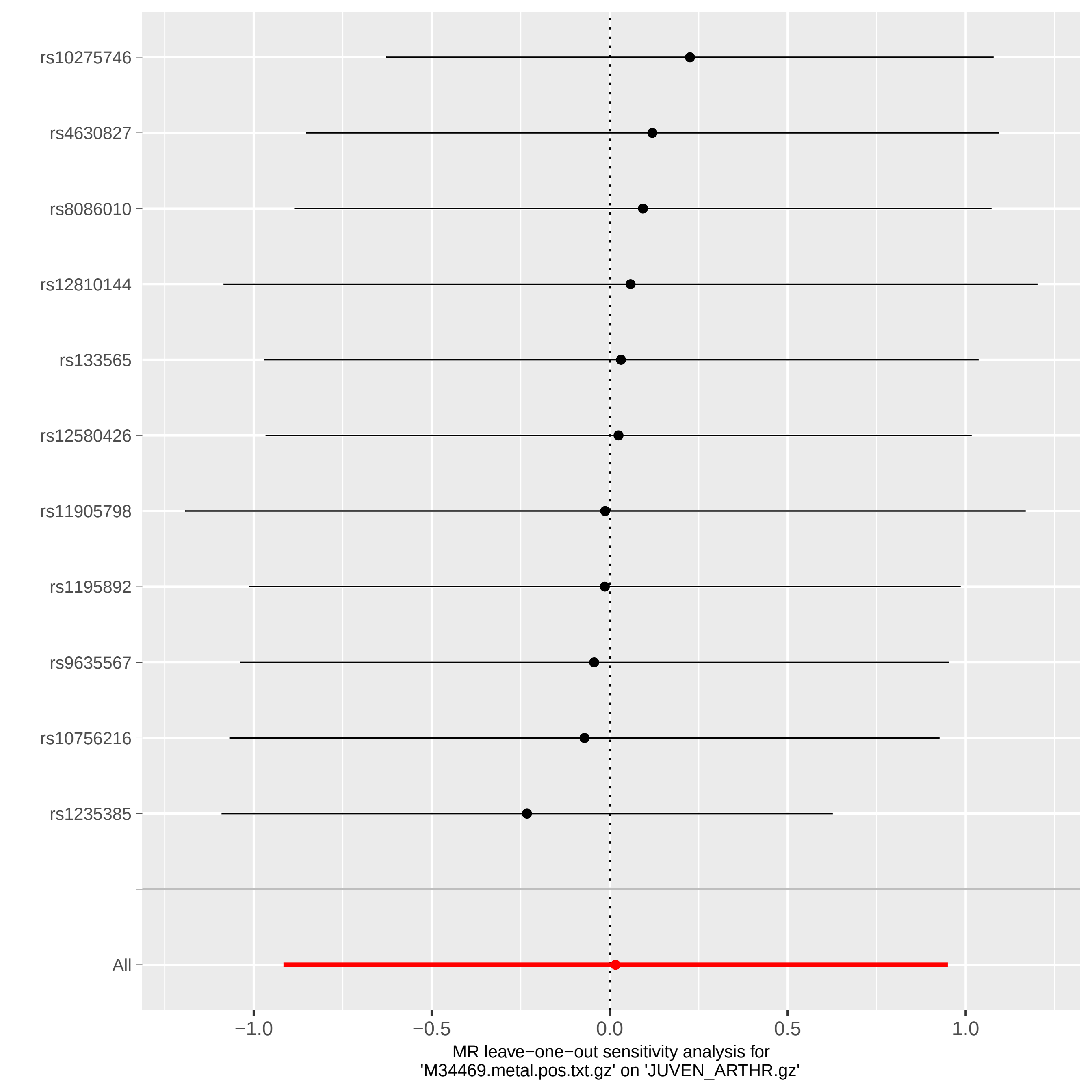

rs10275746
rs4630827
rs8086010
rs12810144
rs133565
rs12580426
rs11905798
rs1195892
rs9635567
rs10756216
rs1235385
All
−1.0
−0.5
0.0
0.5
1.0
MR leave−one−out sensitivity analysis for
'M34469.metal.pos.txt.gz' on 'JUVEN_ARTHR.gz'

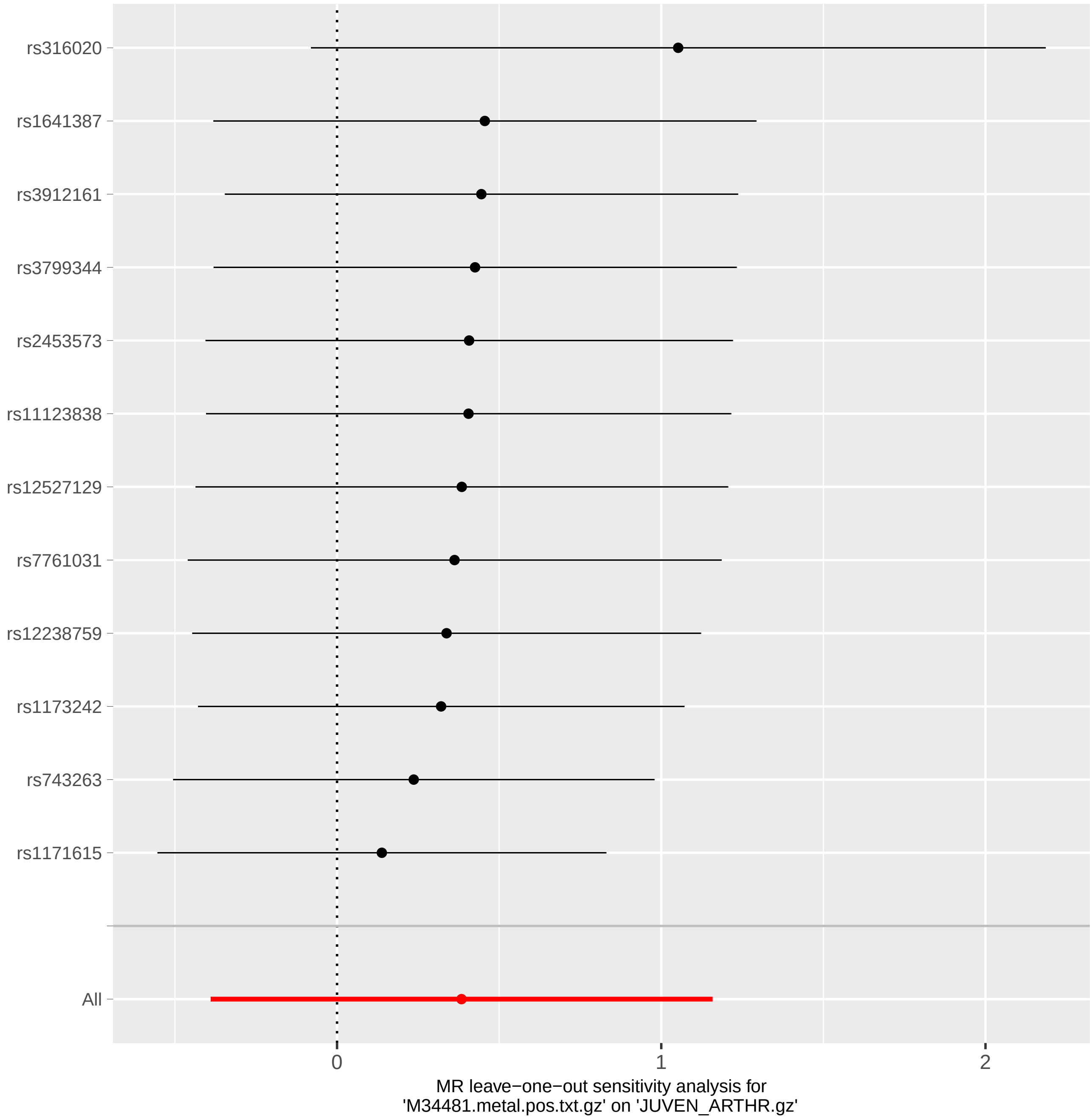
rs316020
rs1641387
rs3912161
rs3799344
rs2453573
rs11123838
rs12527129
rs7761031
rs12238759
rs1173242
rs743263
rs1171615
All
0
1
2
MR leave−one−out sensitivity analysis for
'M34481.metal.pos.txt.gz' on 'JUVEN_ARTHR.gz'

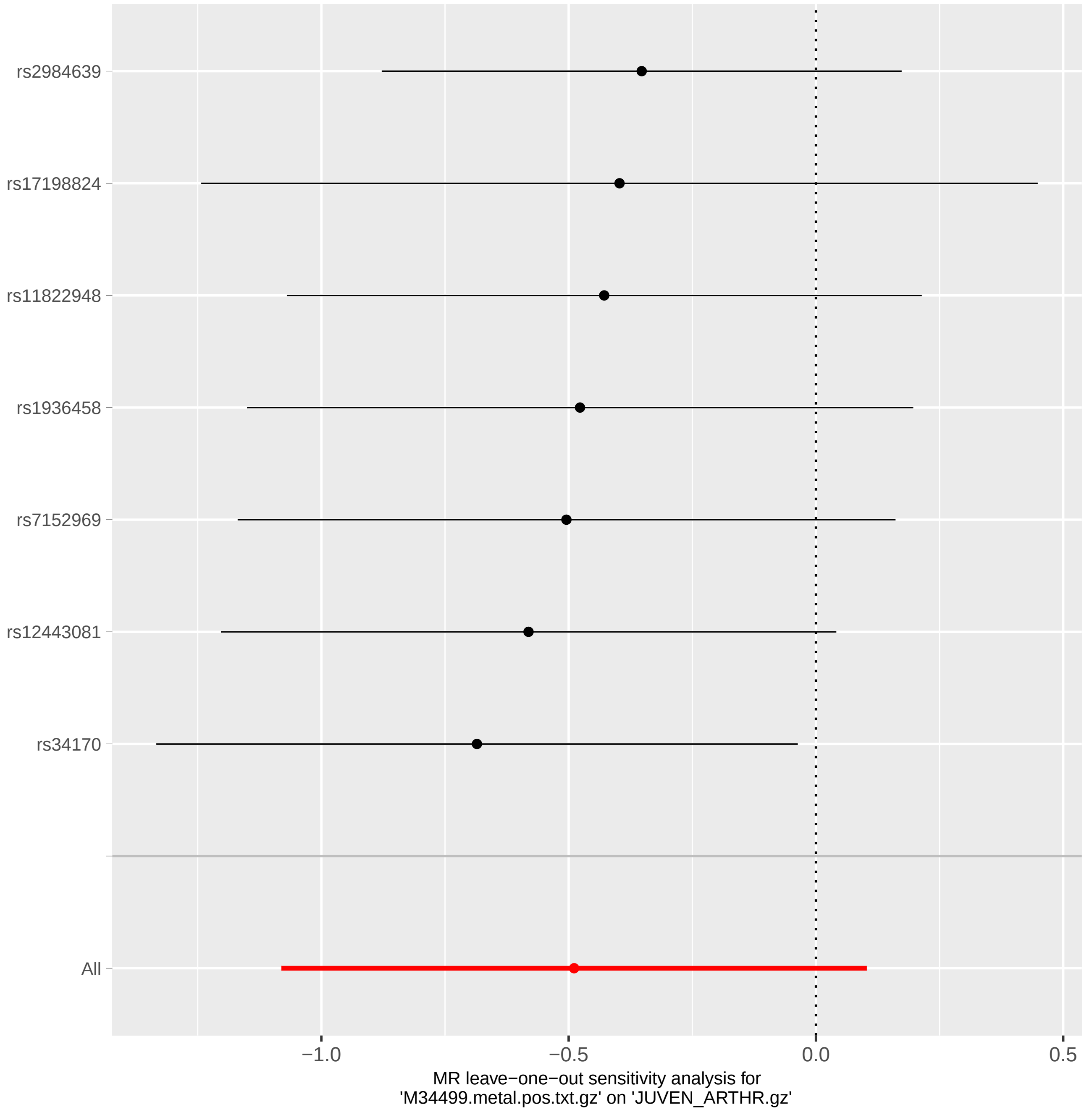

rs2984639
rs17198824
rs11822948
rs1936458
rs7152969
rs12443081
rs34170
All
−1.0
−0.5
0.0
0.5
MR leave−one−out sensitivity analysis for
'M34499.metal.pos.txt.gz' on 'JUVEN_ARTHR.gz'

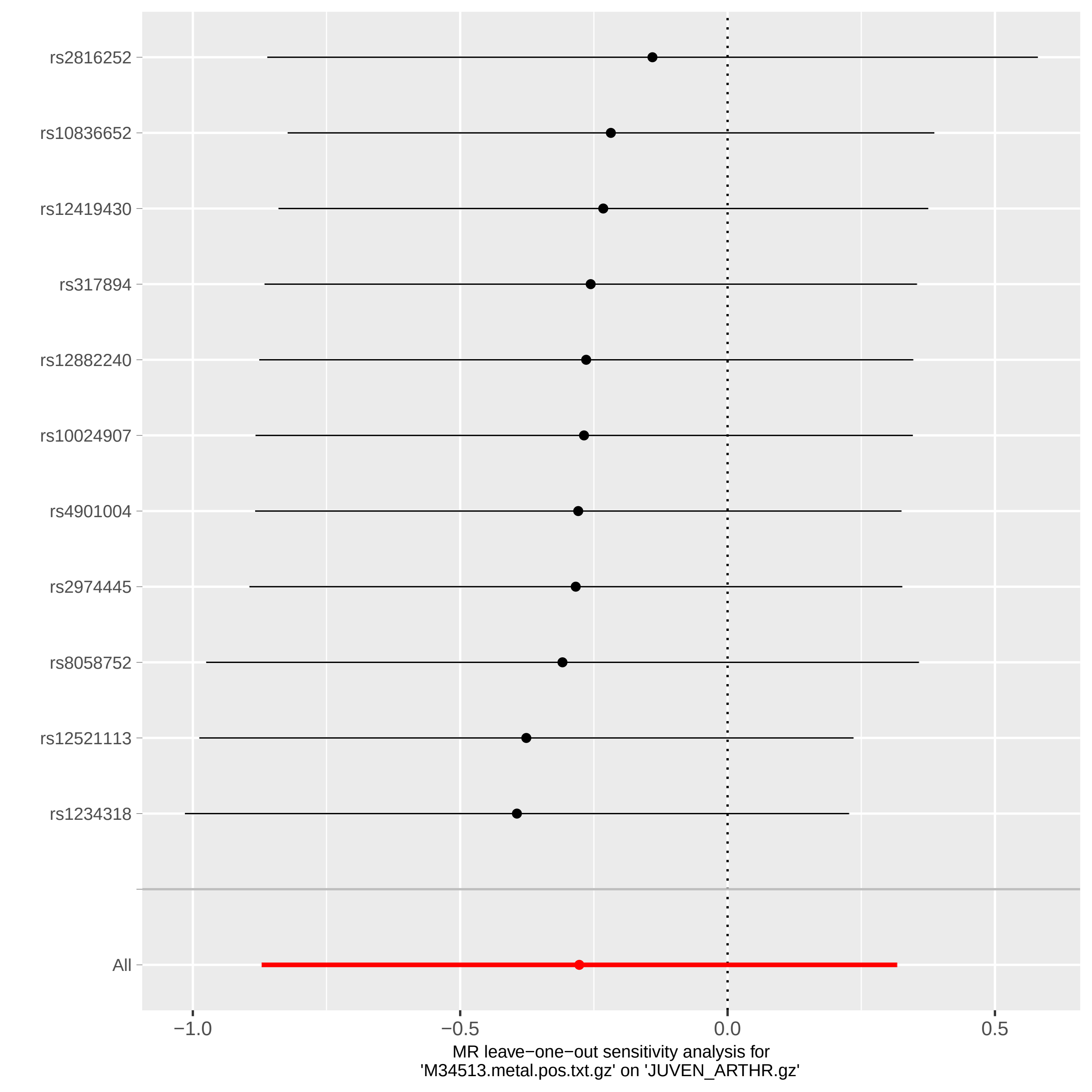

rs2816252
rs10836652
rs12419430
rs317894
rs12882240
rs10024907
rs4901004
rs2974445
rs8058752
rs12521113
rs1234318
All
−1.0
−0.5
0.0
0.5
MR leave−one−out sensitivity analysis for
'M34513.metal.pos.txt.gz' on 'JUVEN_ARTHR.gz'

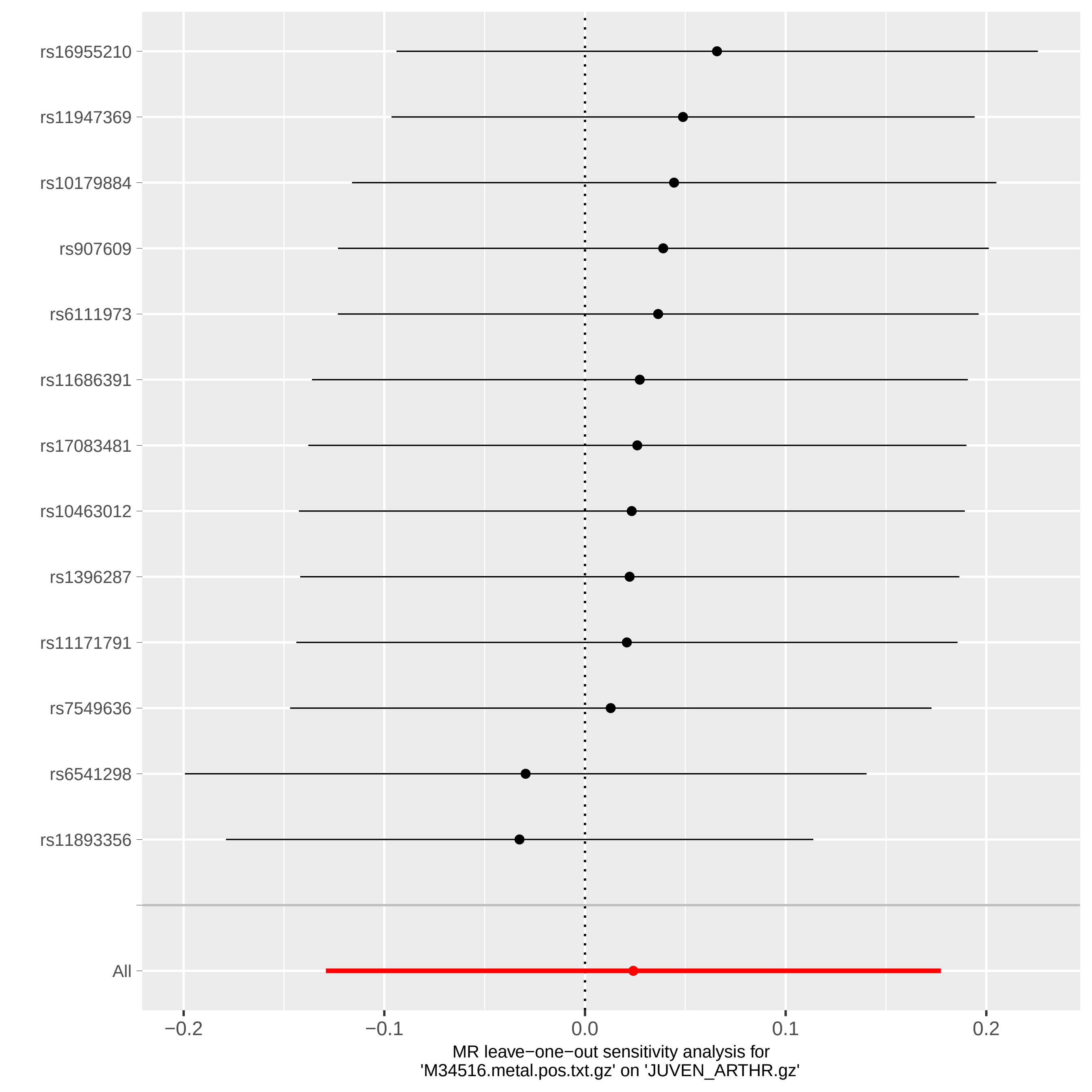
rs16955210
rs11947369
rs10179884
rs907609
rs6111973
rs11686391
rs17083481
rs10463012
rs1396287
rs11171791
rs7549636
rs6541298
rs11893356
All
−0.2
−0.1
0.0
0.1
0.2
MR leave−one−out sensitivity analysis for
'M34516.metal.pos.txt.gz' on 'JUVEN_ARTHR.gz'

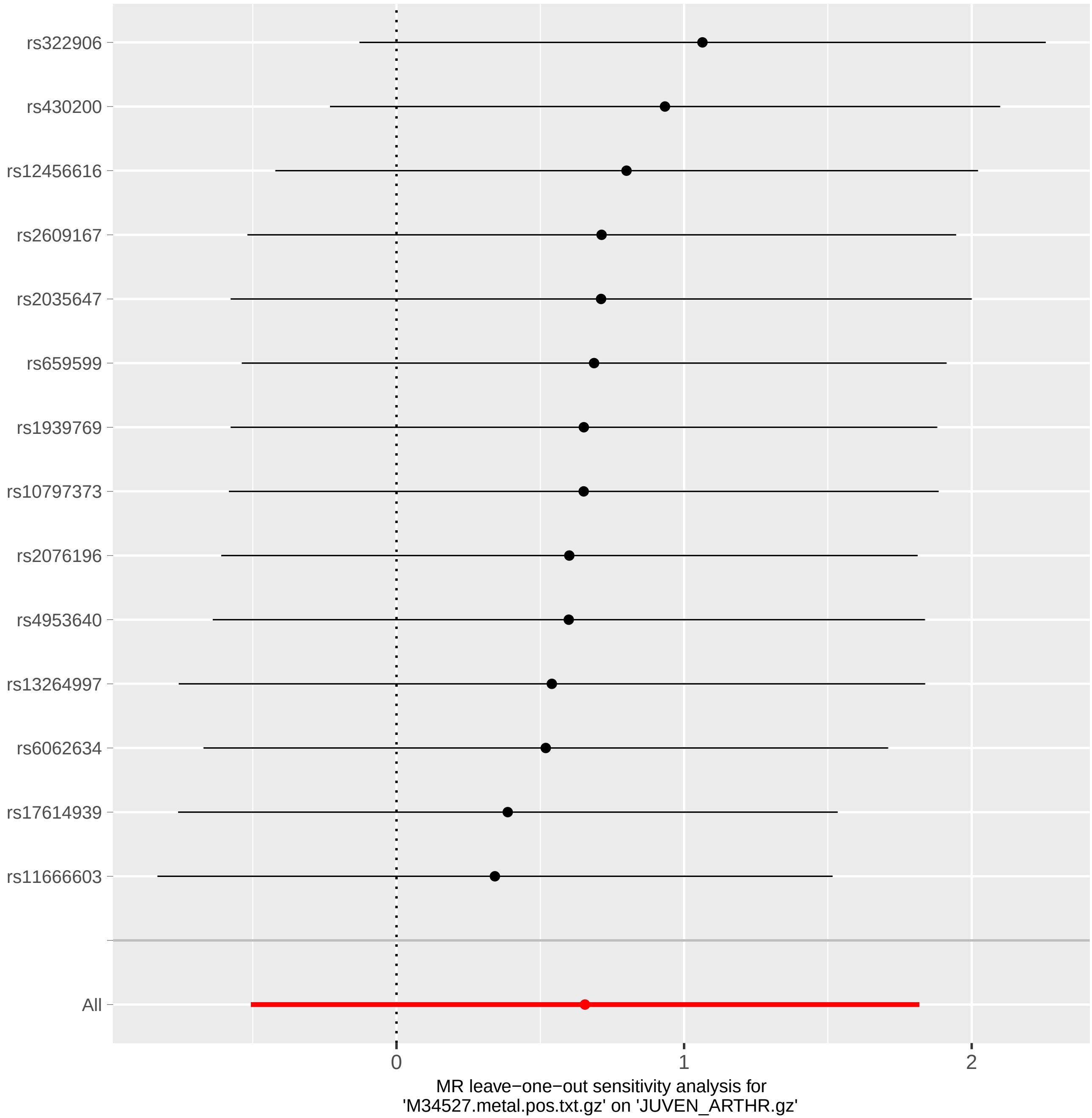
rs322906
rs430200
rs12456616
rs2609167
rs2035647
rs659599
rs1939769
rs10797373
rs2076196
rs4953640
rs13264997
rs6062634
rs17614939
rs11666603
All
0
1
2
MR leave−one−out sensitivity analysis for
'M34527.metal.pos.txt.gz' on 'JUVEN_ARTHR.gz'

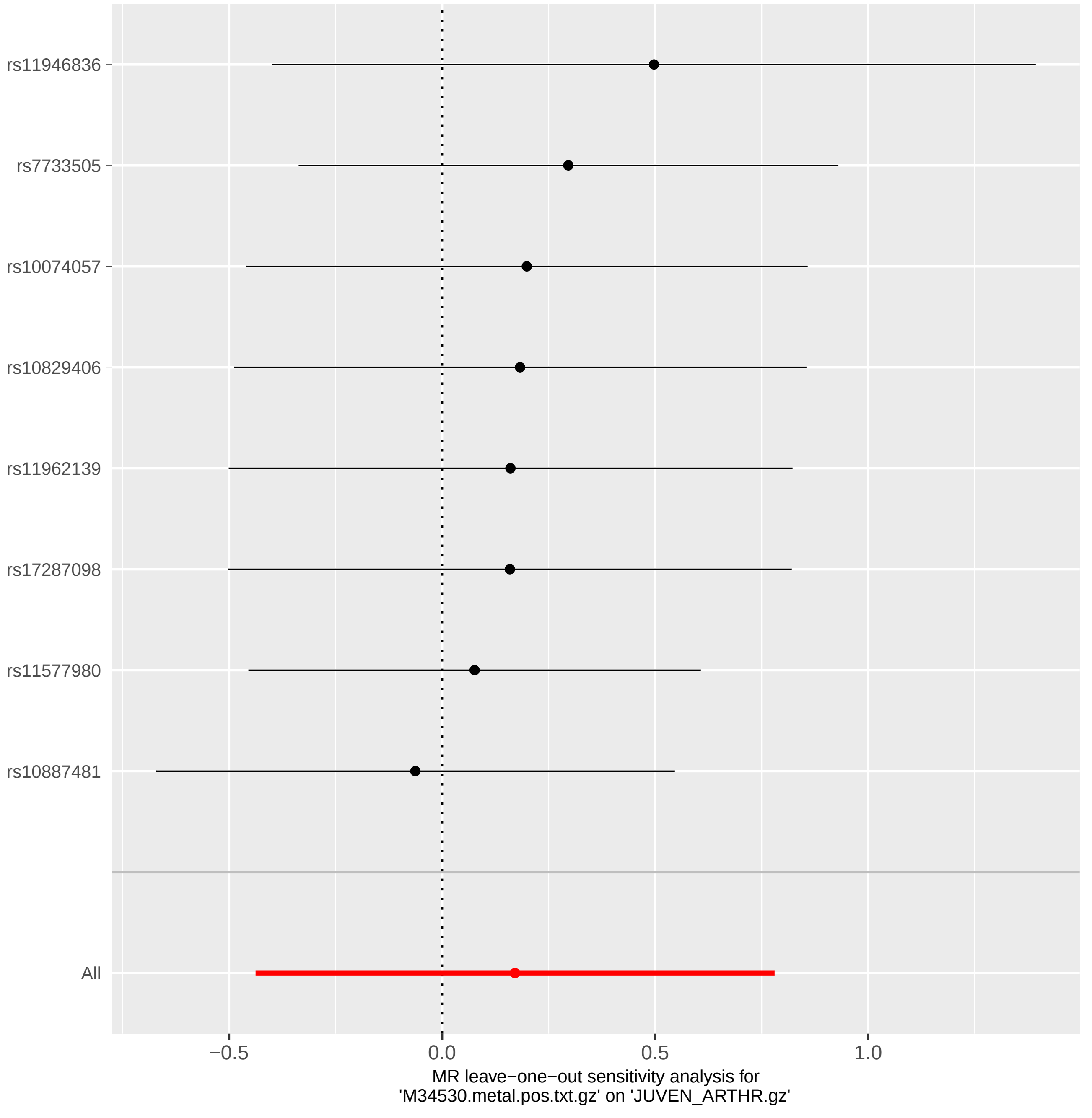

rs11946836
rs7733505
rs10074057
rs10829406
rs11962139
rs17287098
rs11577980
rs10887481
All
−0.5
0.0
0.5
1.0
MR leave−one−out sensitivity analysis for
'M34530.metal.pos.txt.gz' on 'JUVEN_ARTHR.gz'

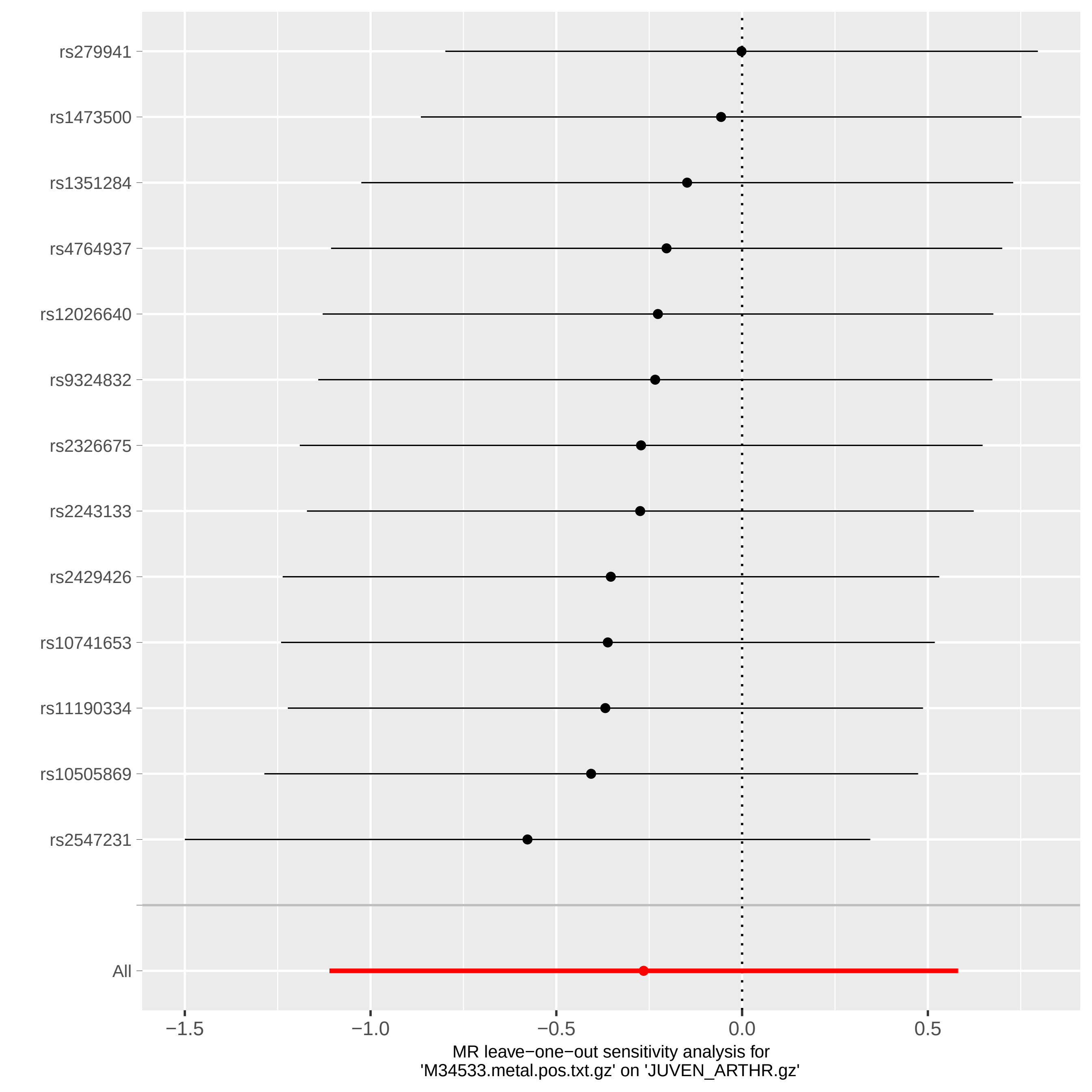

rs279941
rs1473500
rs1351284
rs4764937
rs12026640
rs9324832
rs2326675
rs2243133
rs2429426
rs10741653
rs11190334
rs10505869
rs2547231
All
−1.5
−1.0
−0.5
0.0
0.5
MR leave−one−out sensitivity analysis for
'M34533.metal.pos.txt.gz' on 'JUVEN_ARTHR.gz'

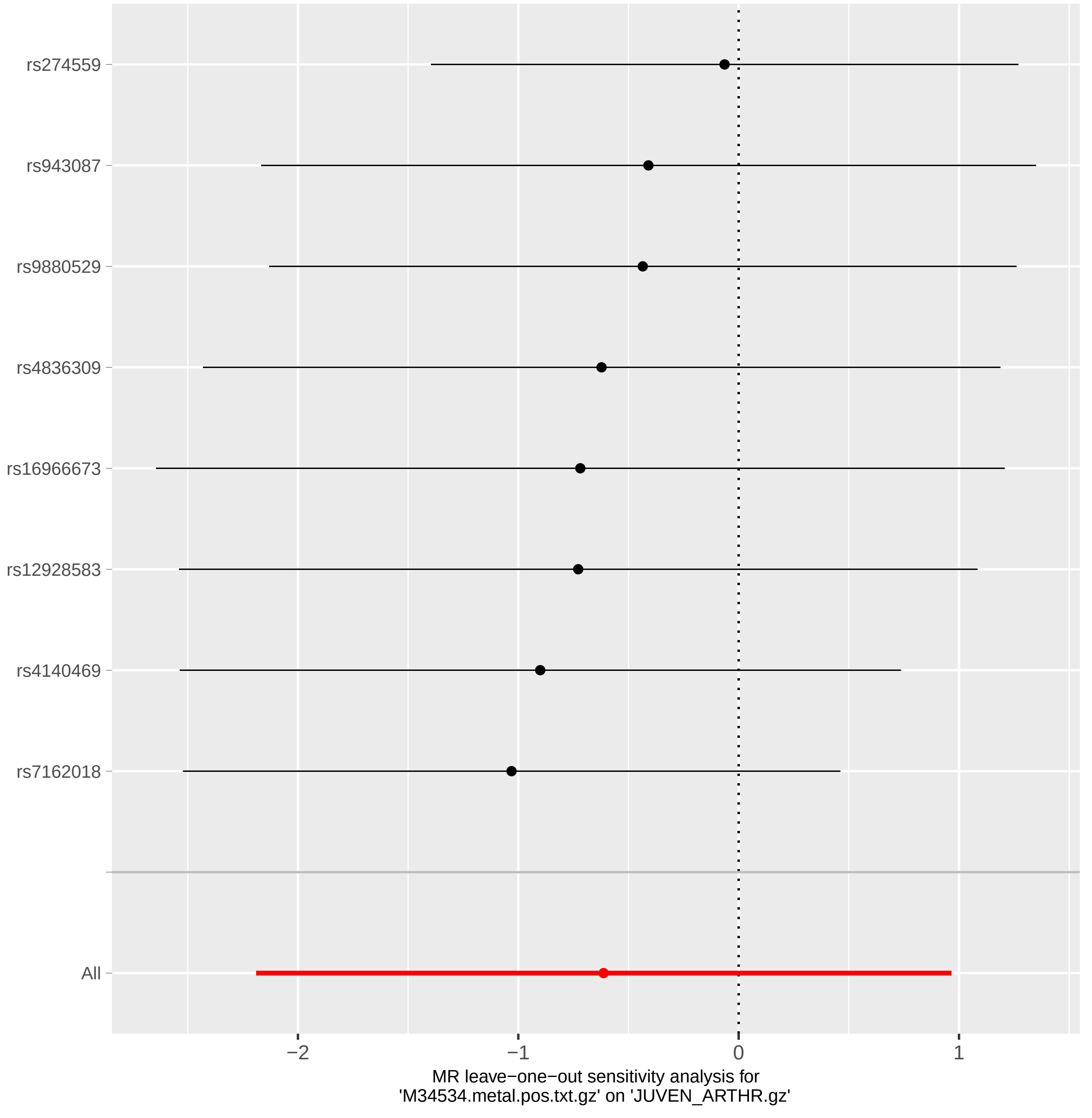

rs274559
rs943087
rs9880529
rs4836309
rs16966673
rs12928583
rs4140469
rs7162018
All
−2
−1
0
1
MR leave−one−out sensitivity analysis for
'M34534.metal.pos.txt.gz' on 'JUVEN_ARTHR.gz'

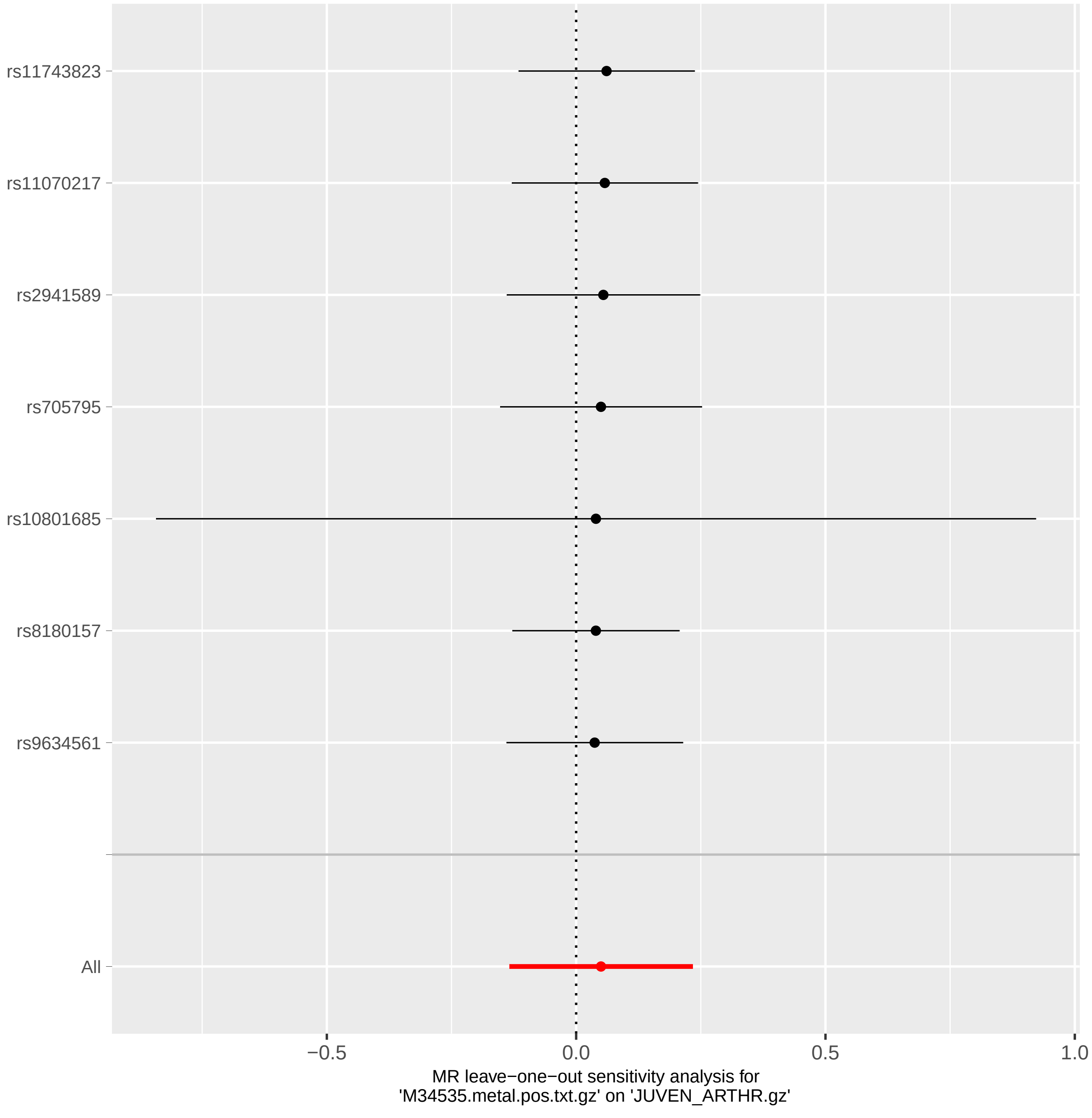
rs11743823
rs11070217
rs2941589
rs705795
rs10801685
rs8180157
rs9634561
All
−0.5
0.0
0.5
1.0
MR leave−one−out sensitivity analysis for
'M34535.metal.pos.txt.gz' on 'JUVEN_ARTHR.gz'

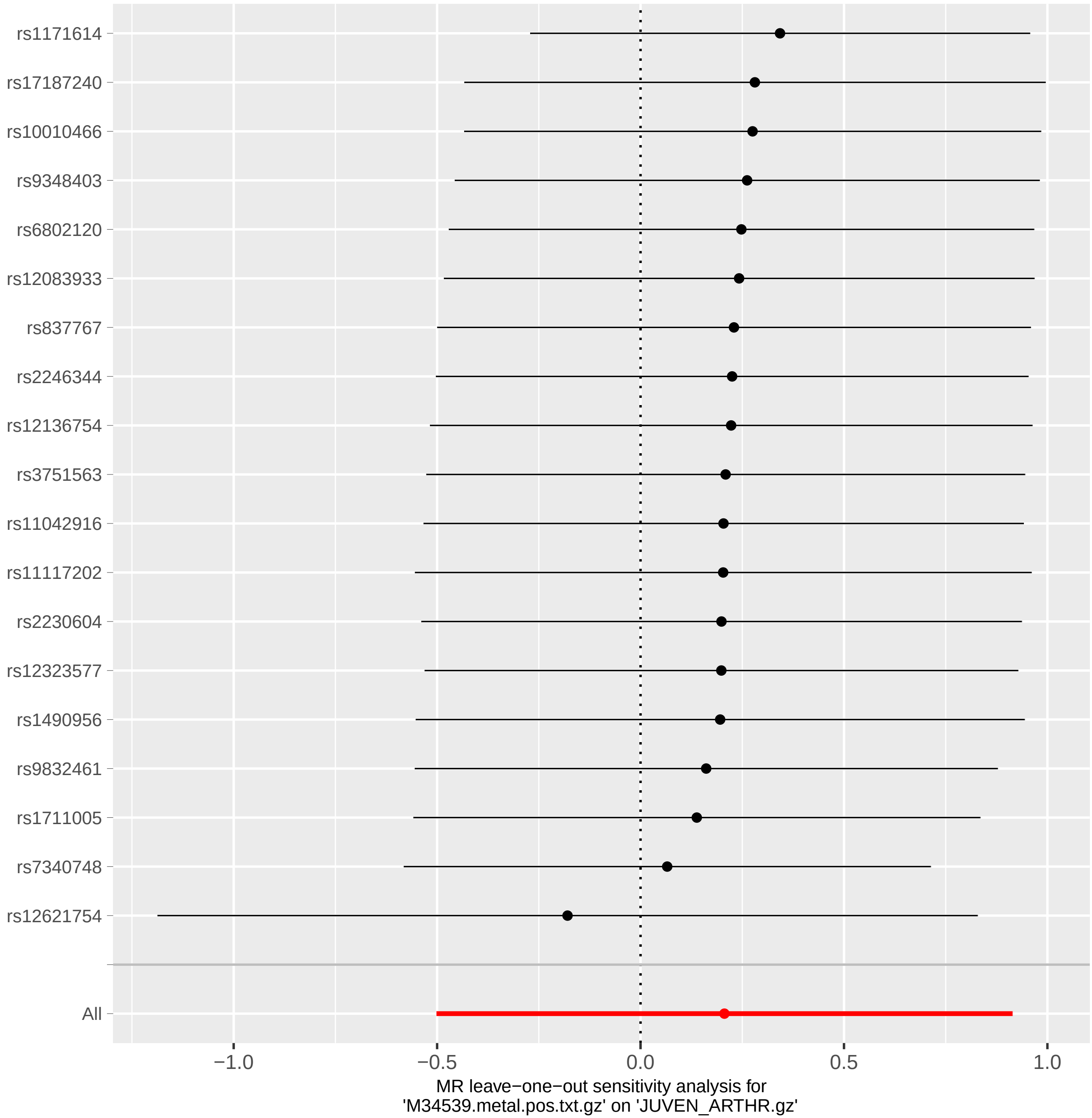
rs1171614
rs17187240
rs10010466
rs9348403
rs6802120
rs12083933
rs837767
rs2246344
rs12136754
rs3751563
rs11042916
rs11117202
rs2230604
rs12323577
rs1490956
rs9832461
rs1711005
rs7340748
rs12621754
All
−1.0
−0.5
0.0
0.5
1.0
MR leave−one−out sensitivity analysis for
'M34539.metal.pos.txt.gz' on 'JUVEN_ARTHR.gz'

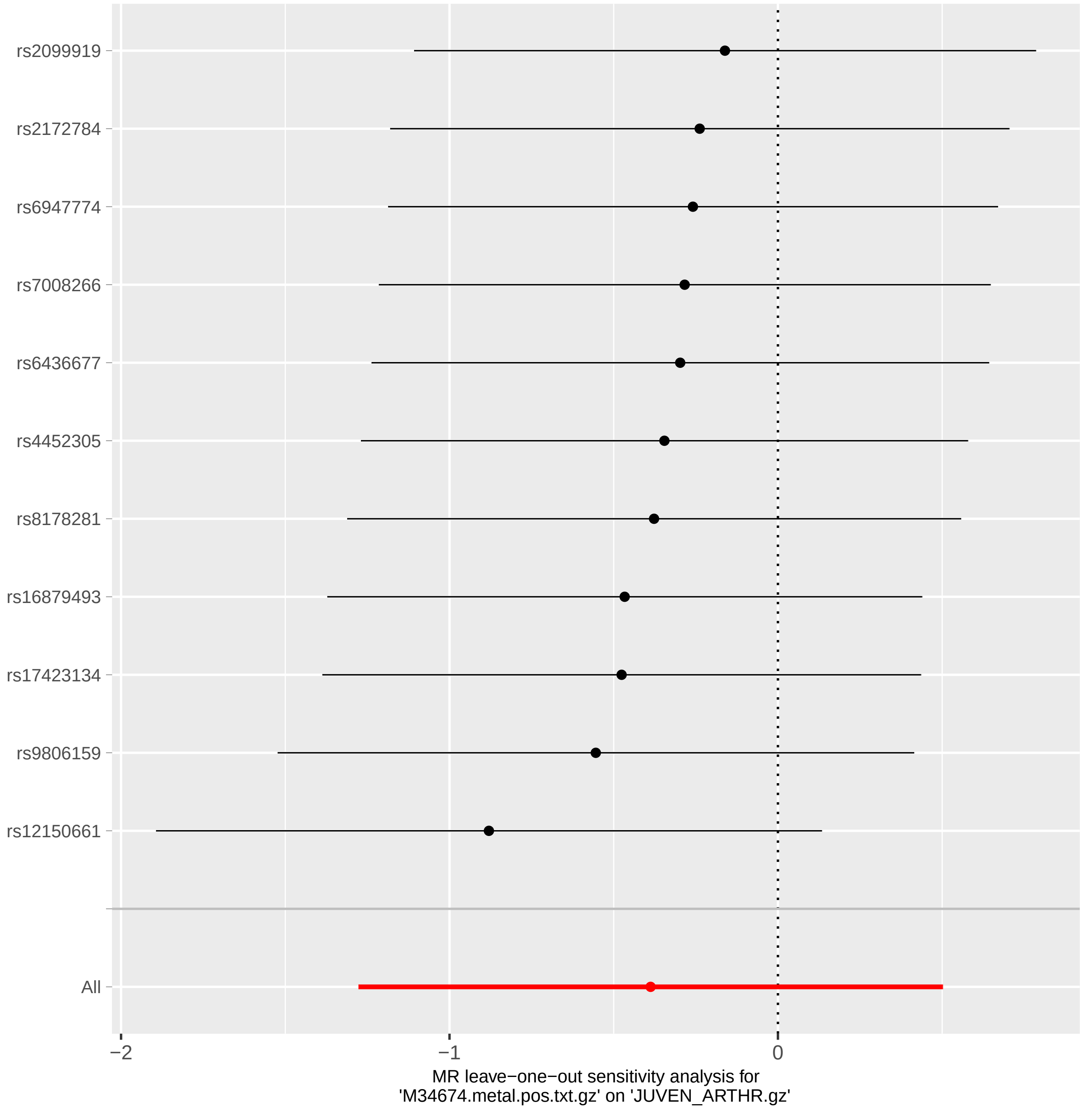
rs2099919
rs2172784
rs6947774
rs7008266
rs6436677
rs4452305
rs8178281
rs16879493
rs17423134
rs9806159
rs12150661
All
−2
−1
0
MR leave−one−out sensitivity analysis for
'M34674.metal.pos.txt.gz' on 'JUVEN_ARTHR.gz'

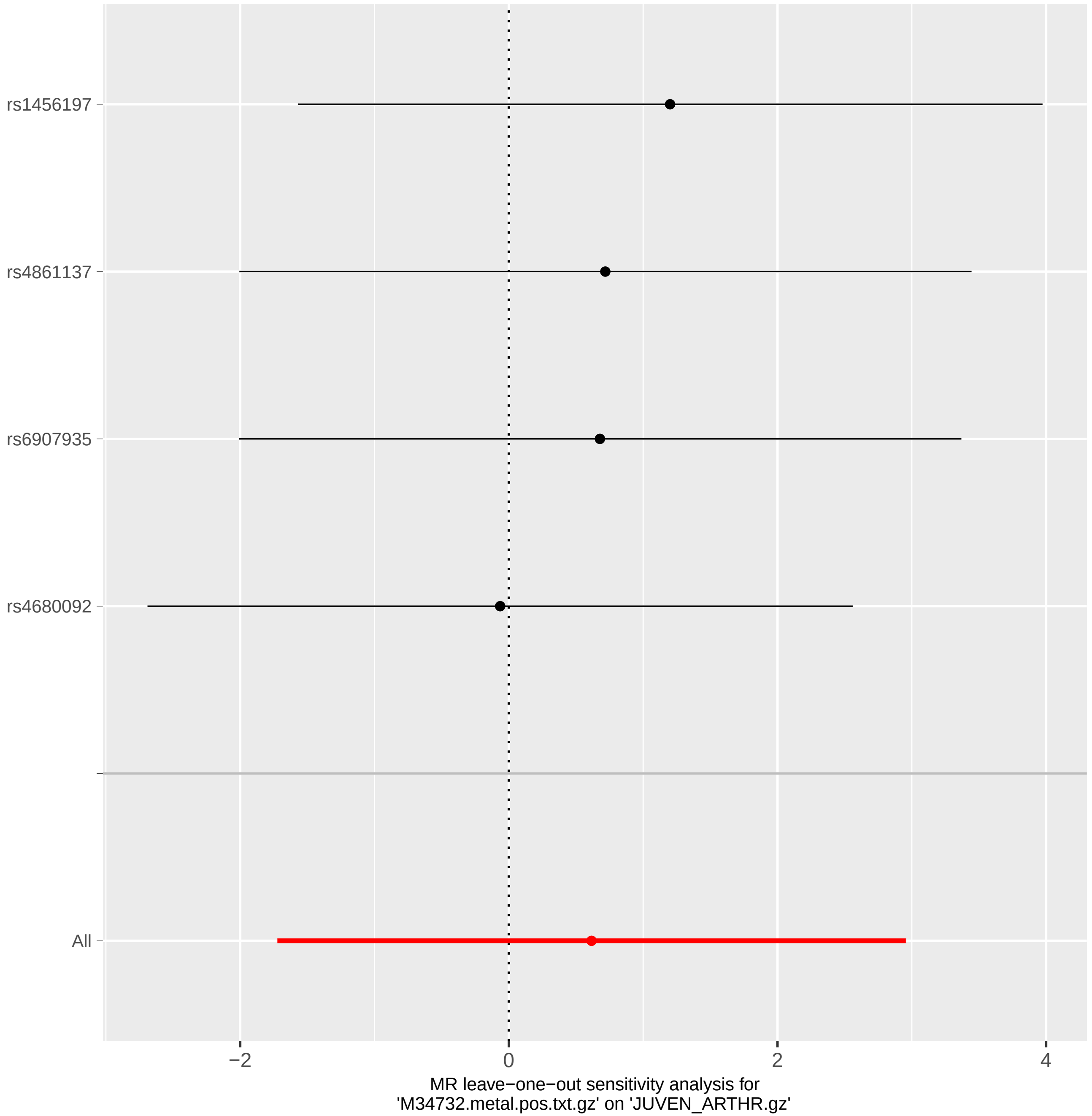
rs1456197
rs4861137
rs6907935
rs4680092
All
−2
0
2
4
MR leave−one−out sensitivity analysis for
'M34732.metal.pos.txt.gz' on 'JUVEN_ARTHR.gz'

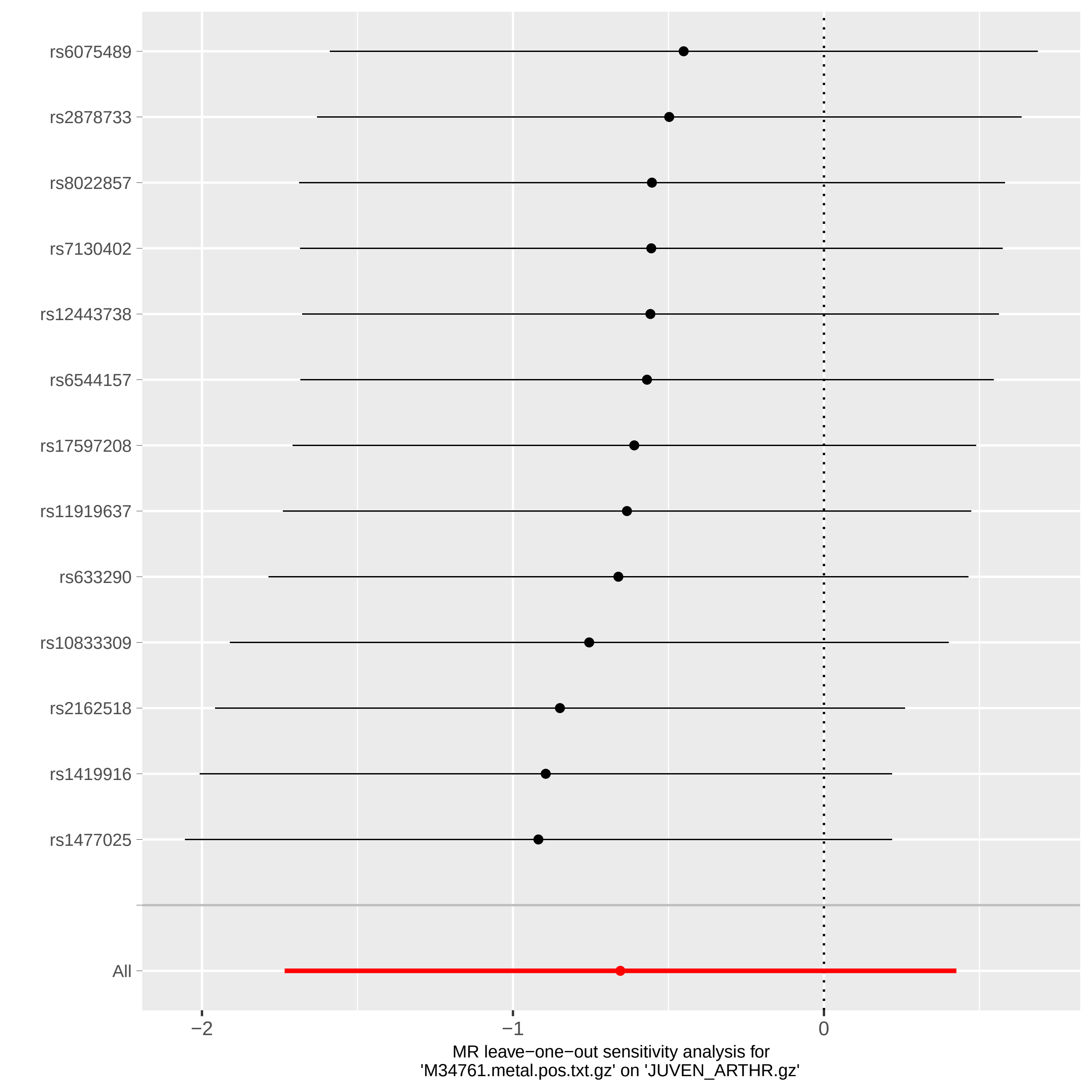
rs6075489
rs2878733
rs8022857
rs7130402
rs12443738
rs6544157
rs17597208
rs11919637
rs633290
rs10833309
rs2162518
rs1419916
rs1477025
All
−2
−1
0
MR leave−one−out sensitivity analysis for
'M34761.metal.pos.txt.gz' on 'JUVEN_ARTHR.gz'

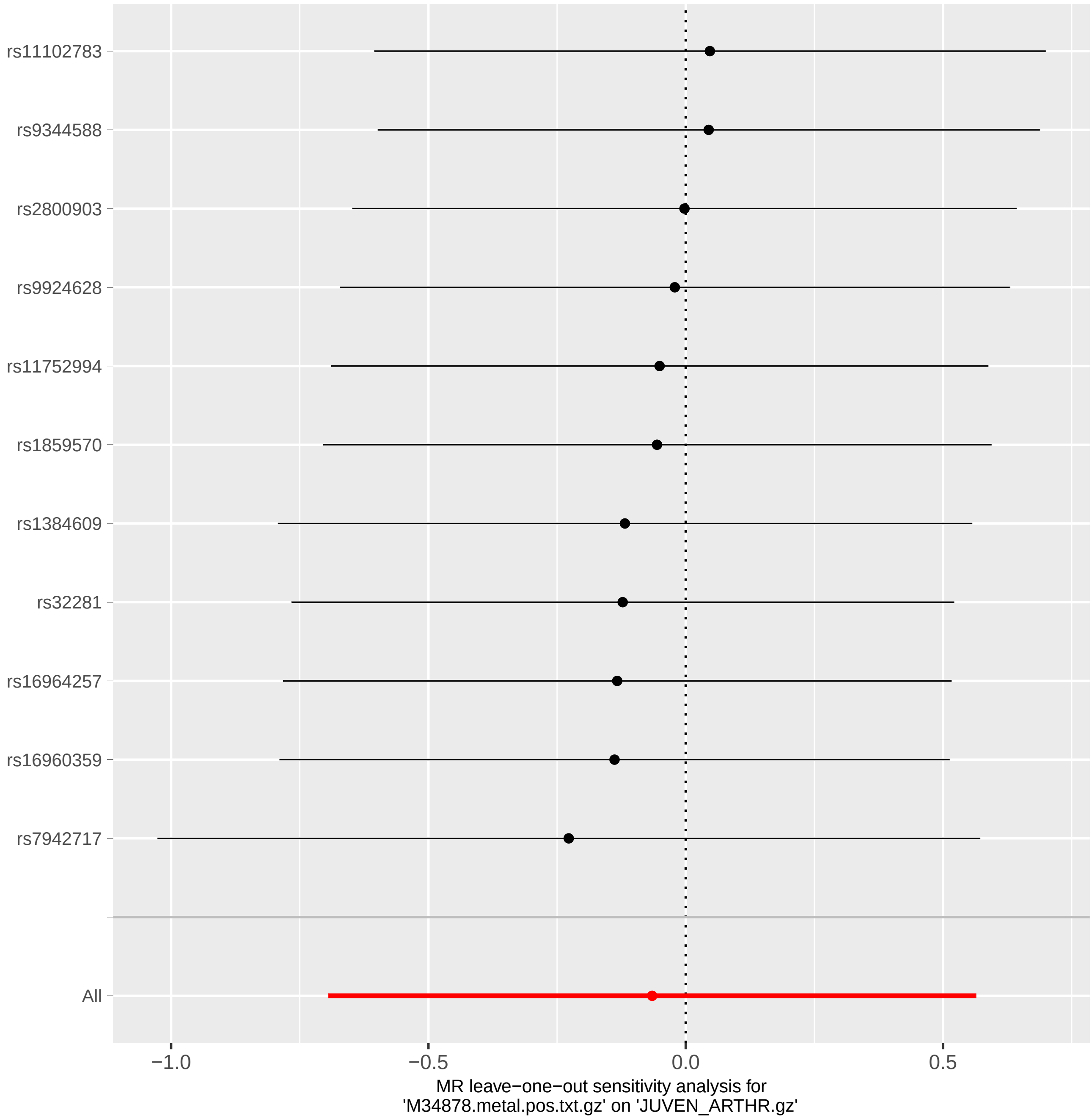

rs11102783
rs9344588
rs2800903
rs9924628
rs11752994
rs1859570
rs1384609
rs32281
rs16964257
rs16960359
rs7942717
All
−1.0
−0.5
0.0
0.5
MR leave−one−out sensitivity analysis for
'M34878.metal.pos.txt.gz' on 'JUVEN_ARTHR.gz'

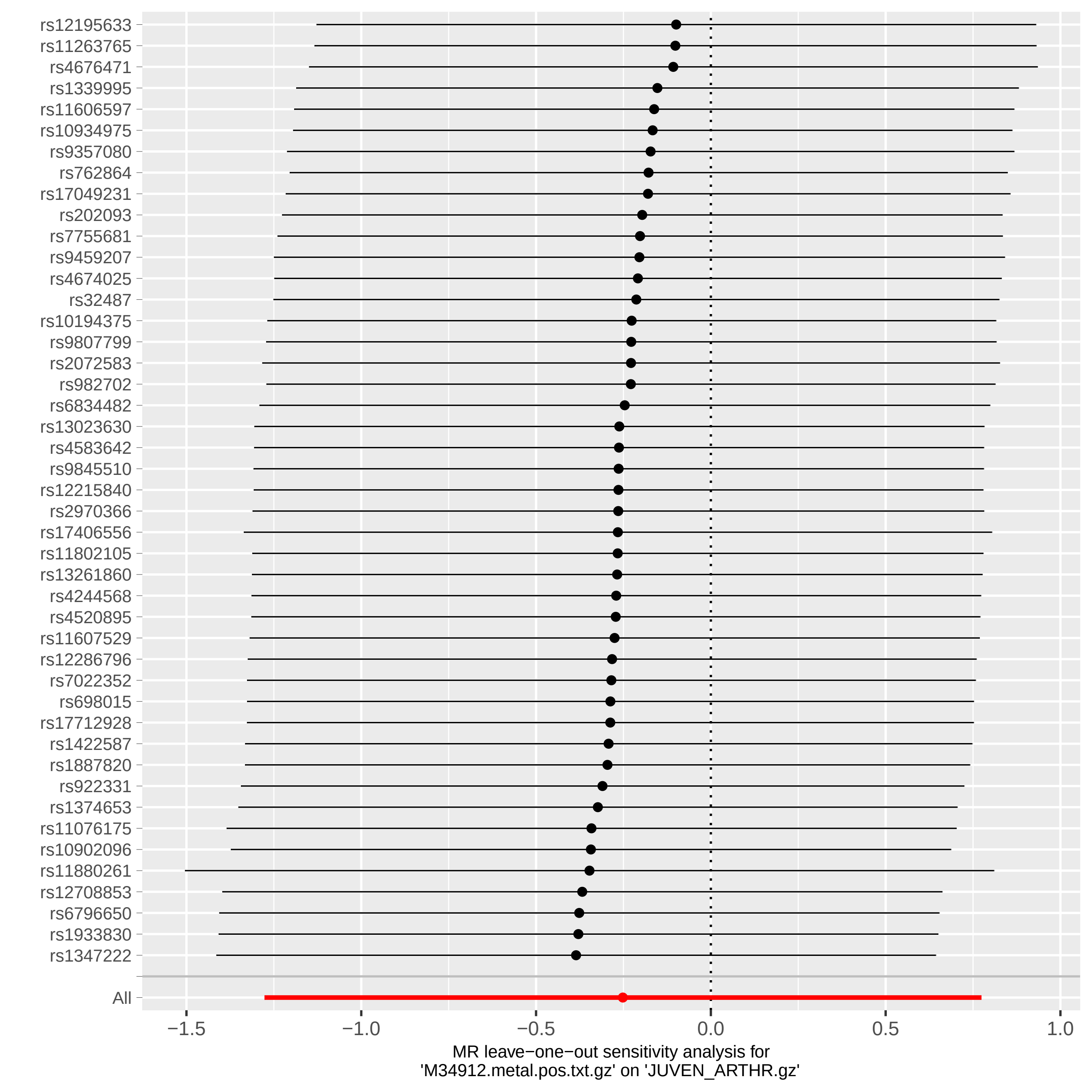
rs12195633
rs11263765
rs4676471
rs1339995
rs11606597
rs10934975
rs9357080
rs762864
rs17049231
rs202093
rs7755681
rs9459207
rs4674025
rs32487
rs10194375
rs9807799
rs2072583
rs982702
rs6834482
rs13023630
rs4583642
rs9845510
rs12215840
rs2970366
rs17406556
rs11802105
rs13261860
rs4244568
rs4520895
rs11607529
rs12286796
rs7022352
rs698015
rs17712928
rs1422587
rs1887820
rs922331
rs1374653
rs11076175
rs10902096
rs11880261
rs12708853
rs6796650
rs1933830
rs1347222
All
−1.5
−1.0
−0.5
0.0
0.5
1.0
MR leave−one−out sensitivity analysis for
'M34912.metal.pos.txt.gz' on 'JUVEN_ARTHR.gz'

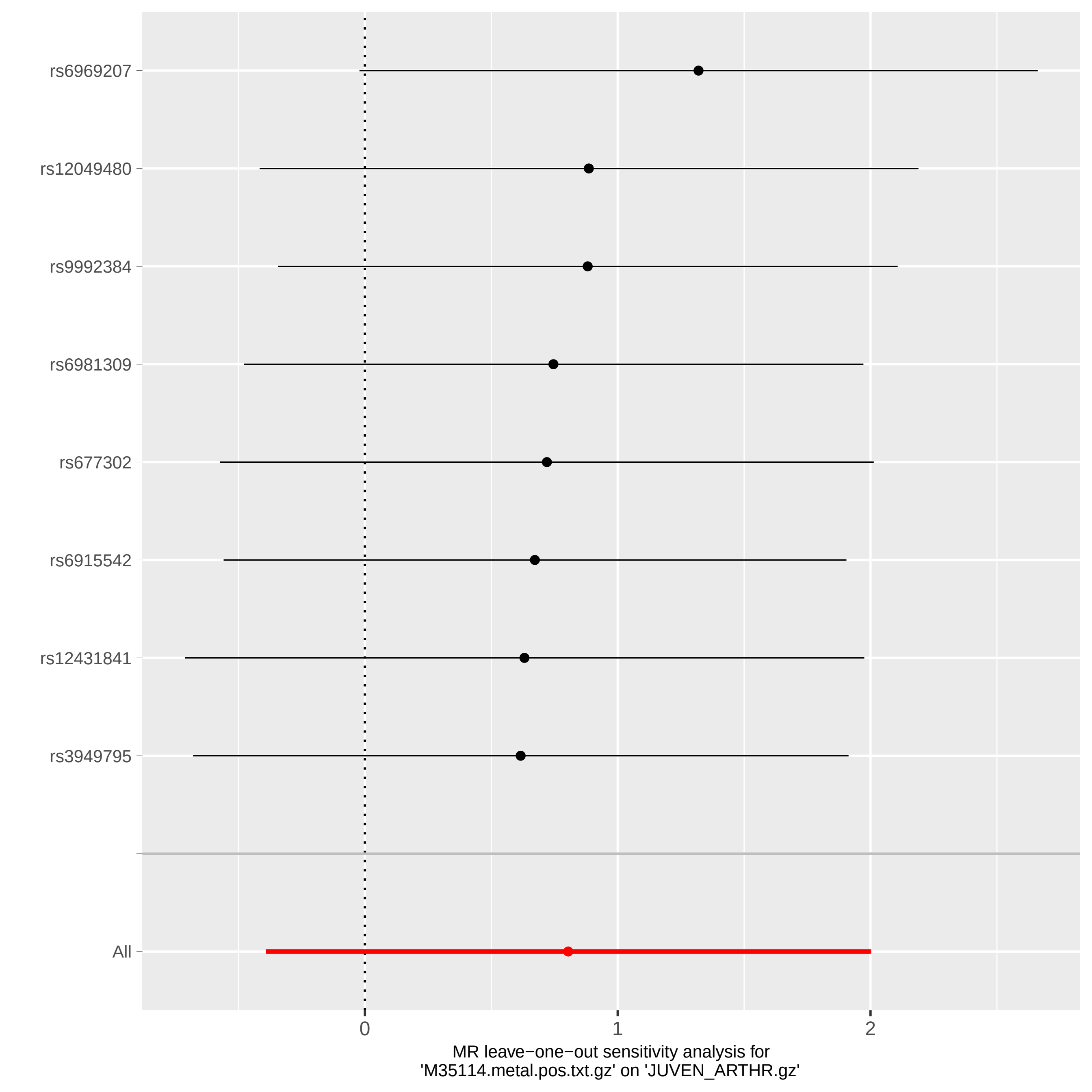
rs6969207
rs12049480
rs9992384
rs6981309
rs677302
rs6915542
rs12431841
rs3949795
All
0
1
2
MR leave−one−out sensitivity analysis for
'M35114.metal.pos.txt.gz' on 'JUVEN_ARTHR.gz'

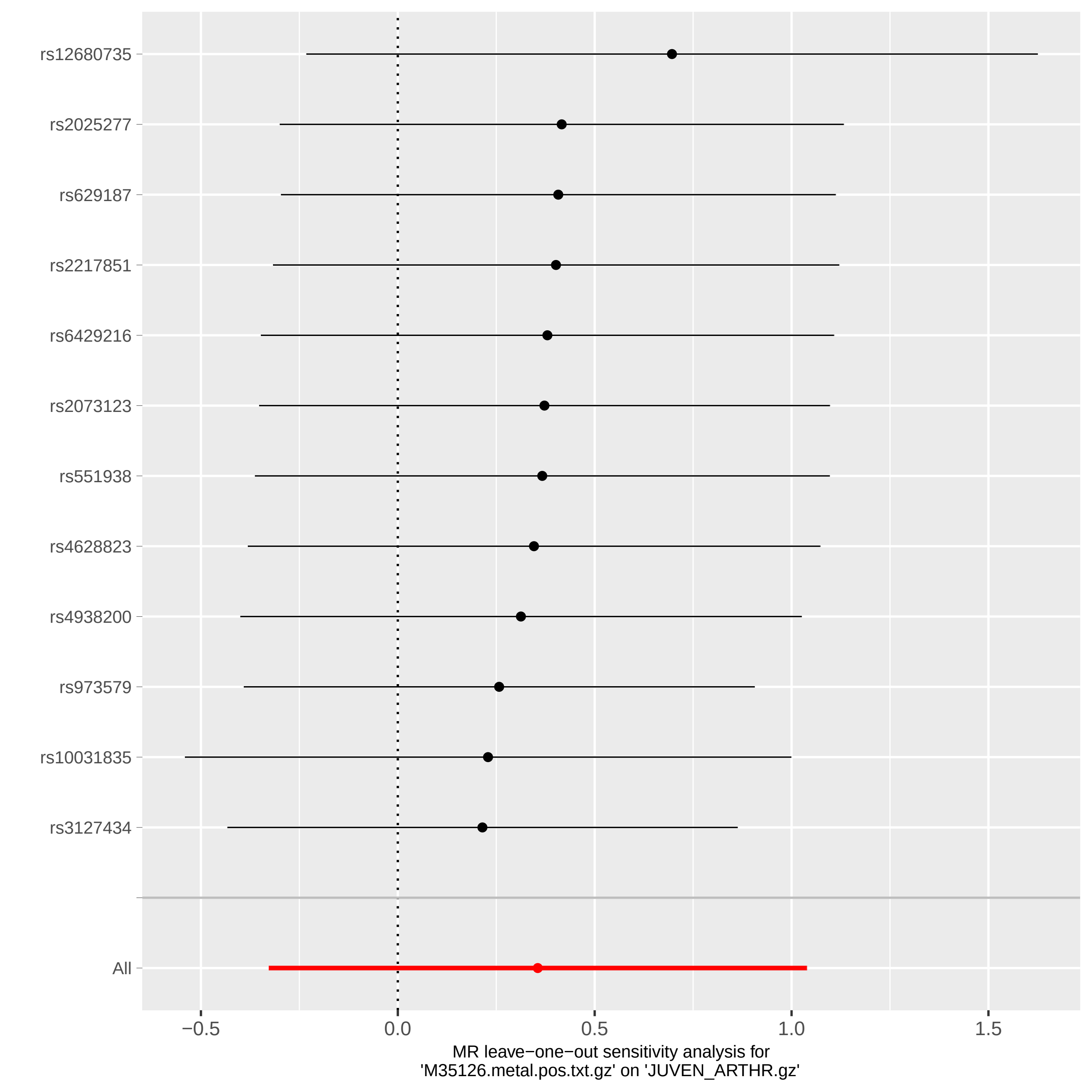
rs12680735
rs2025277
rs629187
rs2217851
rs6429216
rs2073123
rs551938
rs4628823
rs4938200
rs973579
rs10031835
rs3127434
All
−0.5
0.0
0.5
1.0
1.5
MR leave−one−out sensitivity analysis for
'M35126.metal.pos.txt.gz' on 'JUVEN_ARTHR.gz'

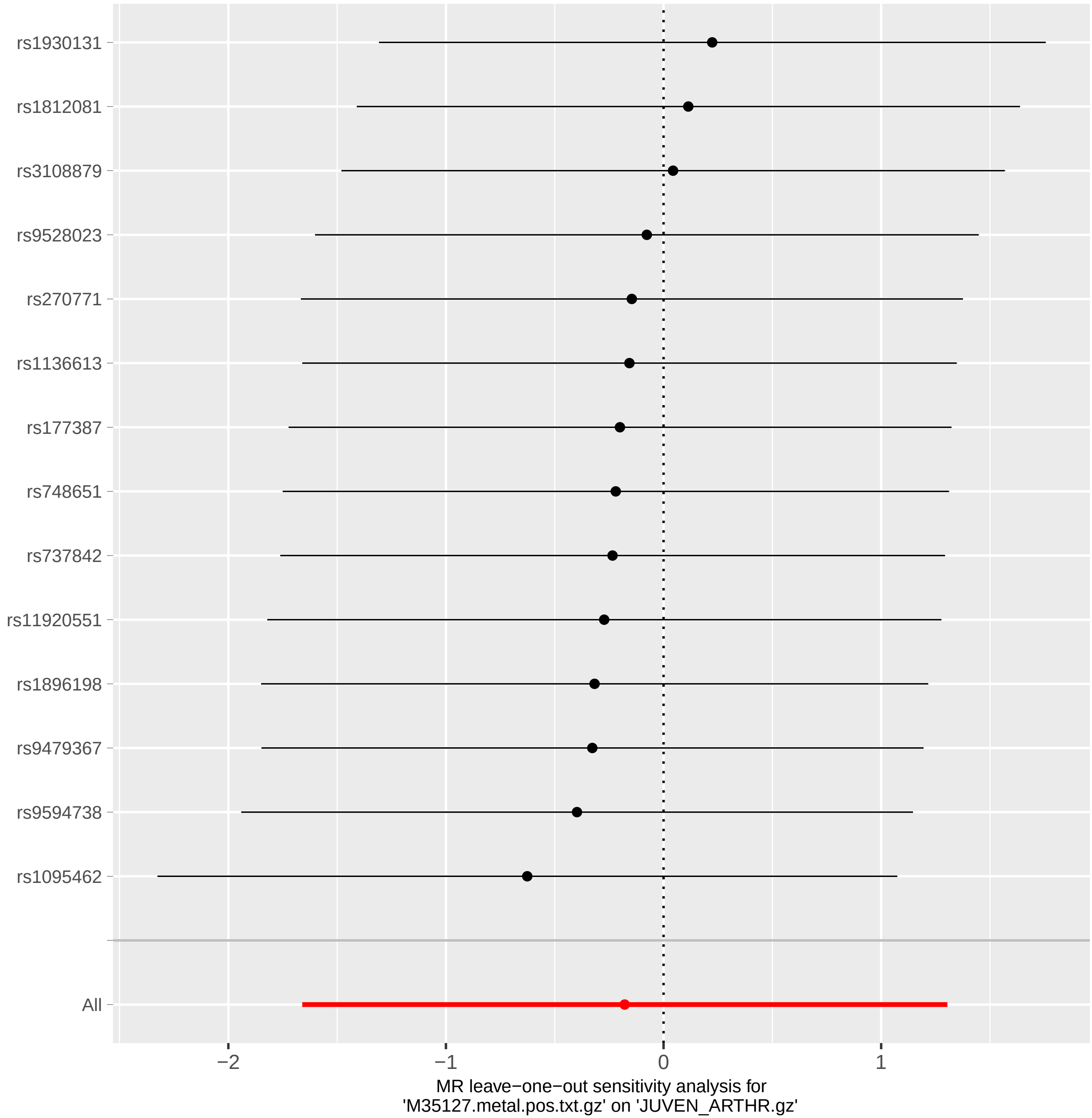
rs1930131
rs1812081
rs3108879
rs9528023
rs270771
rs1136613
rs177387
rs748651
rs737842
rs11920551
rs1896198
rs9479367
rs9594738
rs1095462
All
−2
−1
0
1
MR leave−one−out sensitivity analysis for
'M35127.metal.pos.txt.gz' on 'JUVEN_ARTHR.gz'

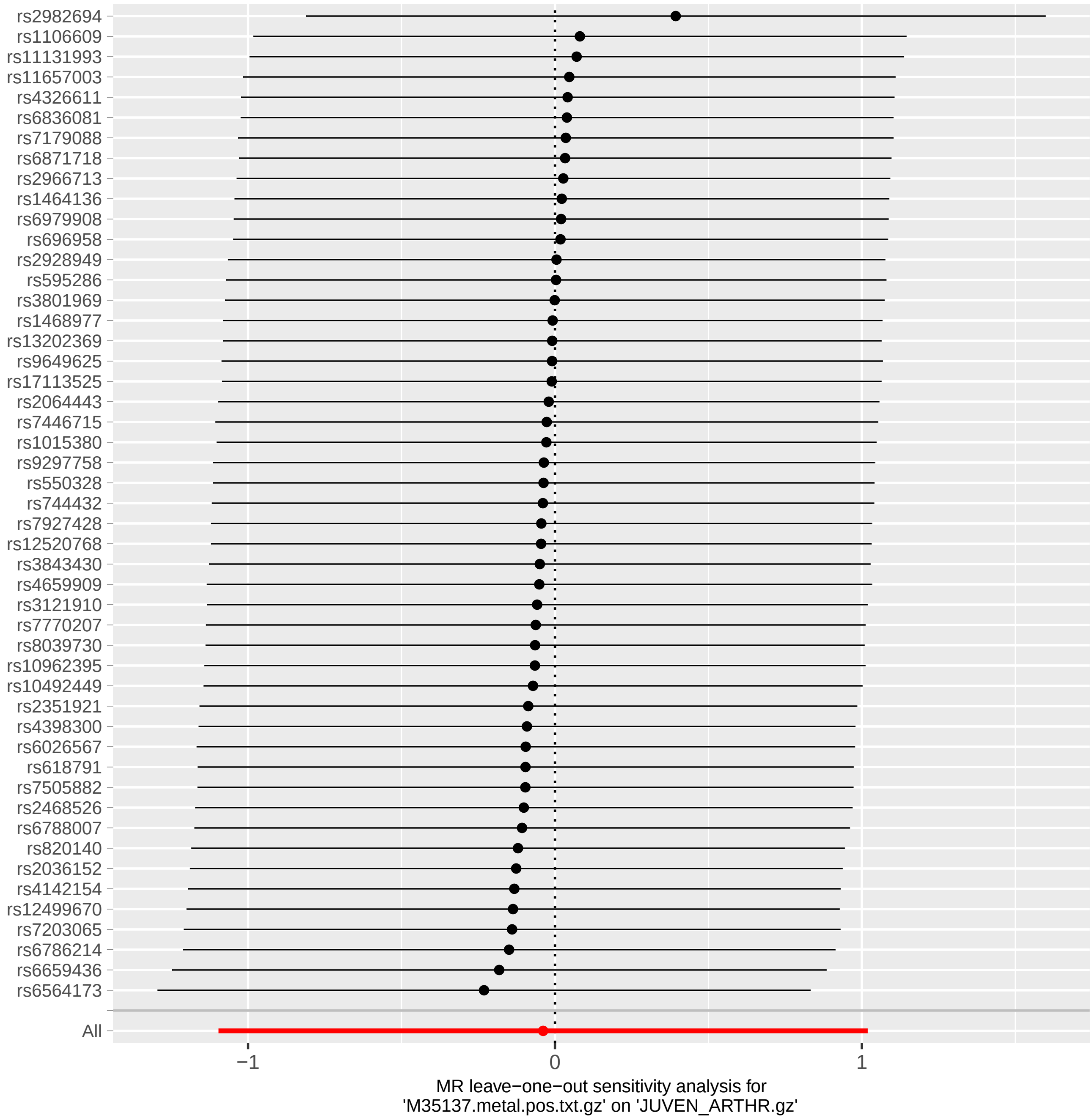

rs2982694
rs1106609
rs11131993
rs11657003
rs4326611
rs6836081
rs7179088
rs6871718
rs2966713
rs1464136
rs6979908
rs696958
rs2928949
rs595286
rs3801969
rs1468977
rs13202369
rs9649625
rs17113525
rs2064443
rs7446715
rs1015380
rs9297758
rs550328
rs744432
rs7927428
rs12520768
rs3843430
rs4659909
rs3121910
rs7770207
rs8039730
rs10962395
rs10492449
rs2351921
rs4398300
rs6026567
rs618791
rs7505882
rs2468526
rs6788007
rs820140
rs2036152
rs4142154
rs12499670
rs7203065
rs6786214
rs6659436
rs6564173
All
−1
0
1
MR leave−one−out sensitivity analysis for
'M35137.metal.pos.txt.gz' on 'JUVEN_ARTHR.gz'

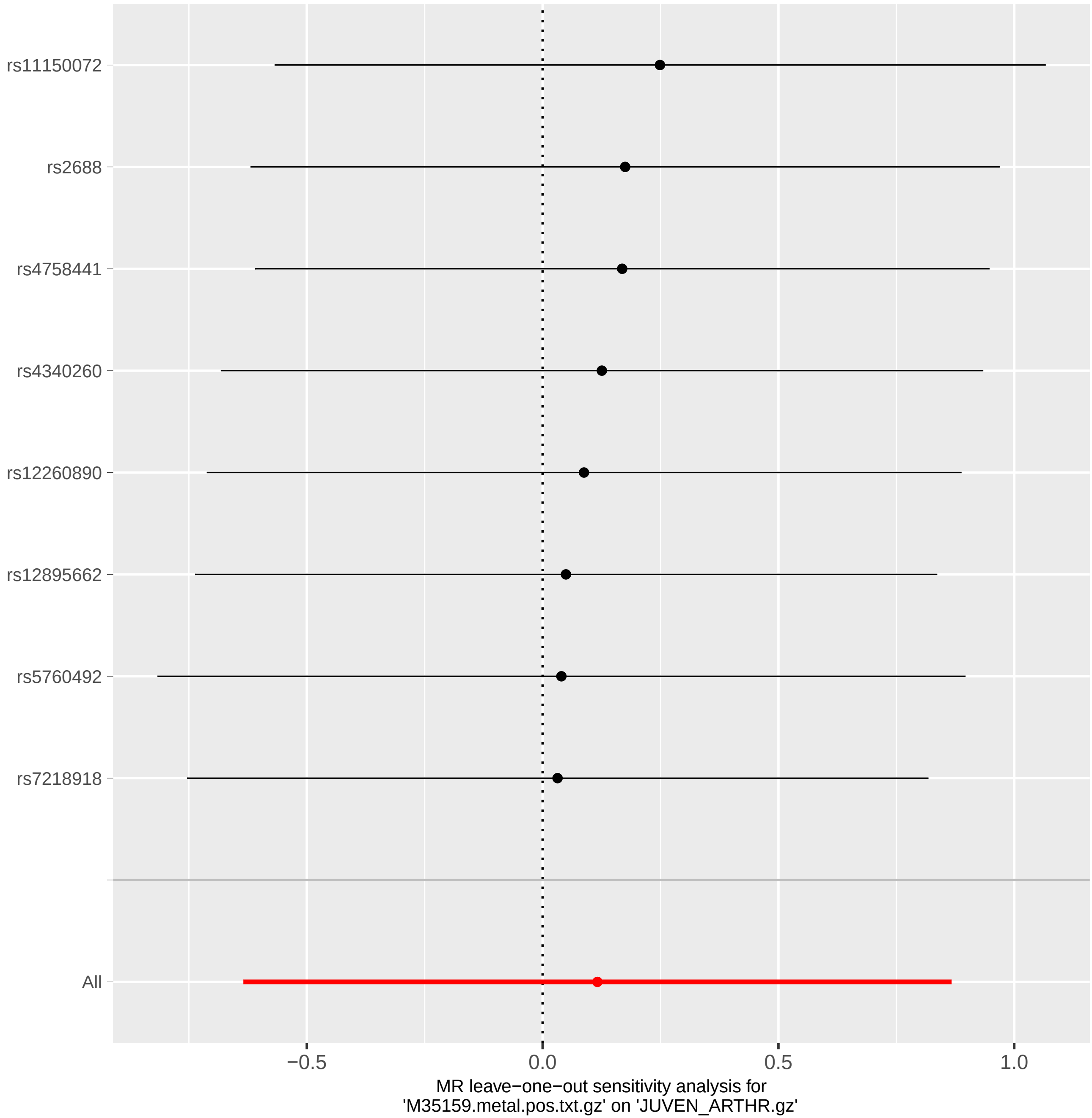
rs11150072
rs2688
rs4758441
rs4340260
rs12260890
rs12895662
rs5760492
rs7218918
All
−0.5
0.0
0.5
1.0
MR leave−one−out sensitivity analysis for
'M35159.metal.pos.txt.gz' on 'JUVEN_ARTHR.gz'

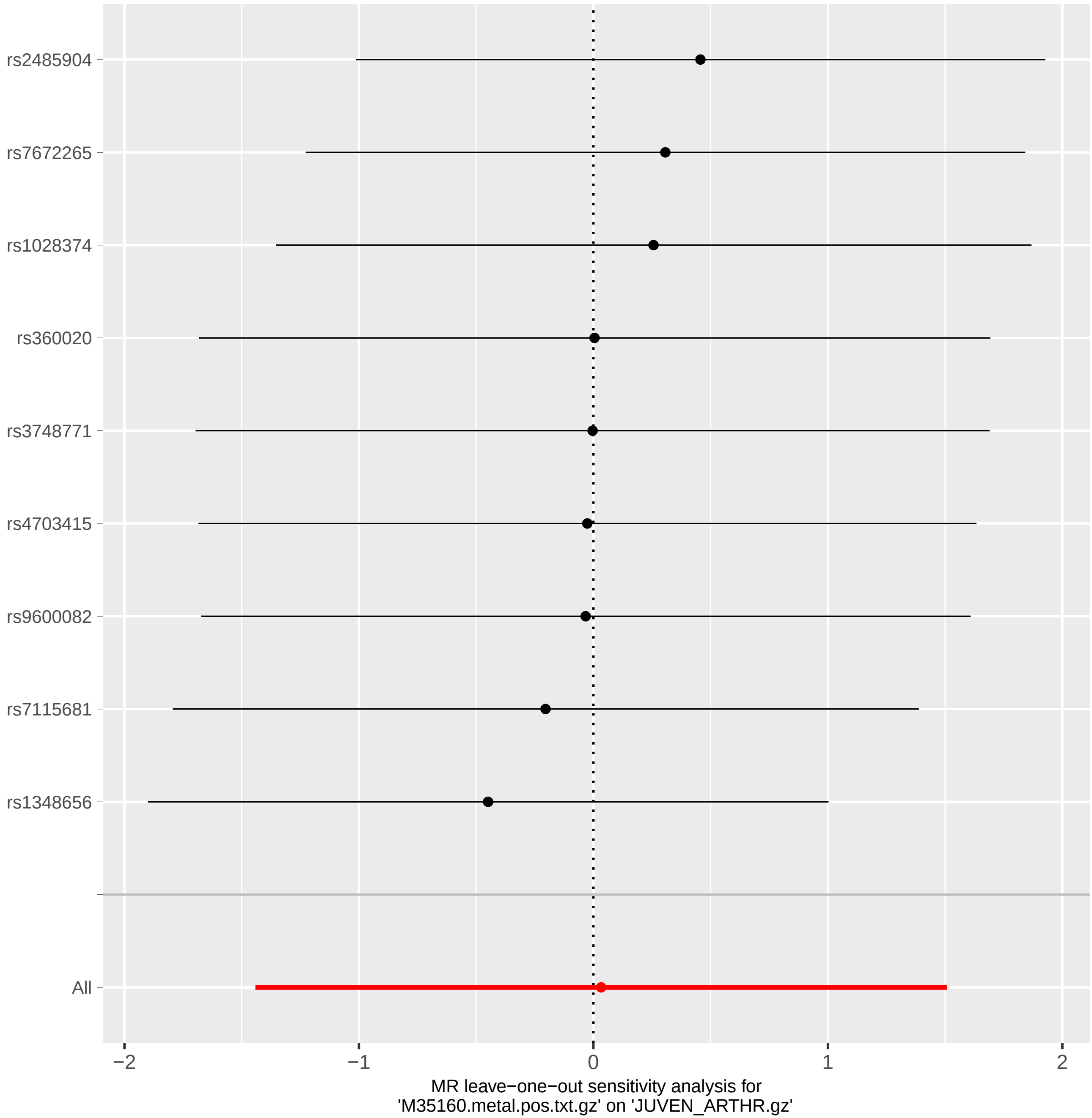
rs2485904
rs7672265
rs1028374
rs360020
rs3748771
rs4703415
rs9600082
rs7115681
rs1348656
All
−2
−1
0
1
2
MR leave−one−out sensitivity analysis for
'M35160.metal.pos.txt.gz' on 'JUVEN_ARTHR.gz'

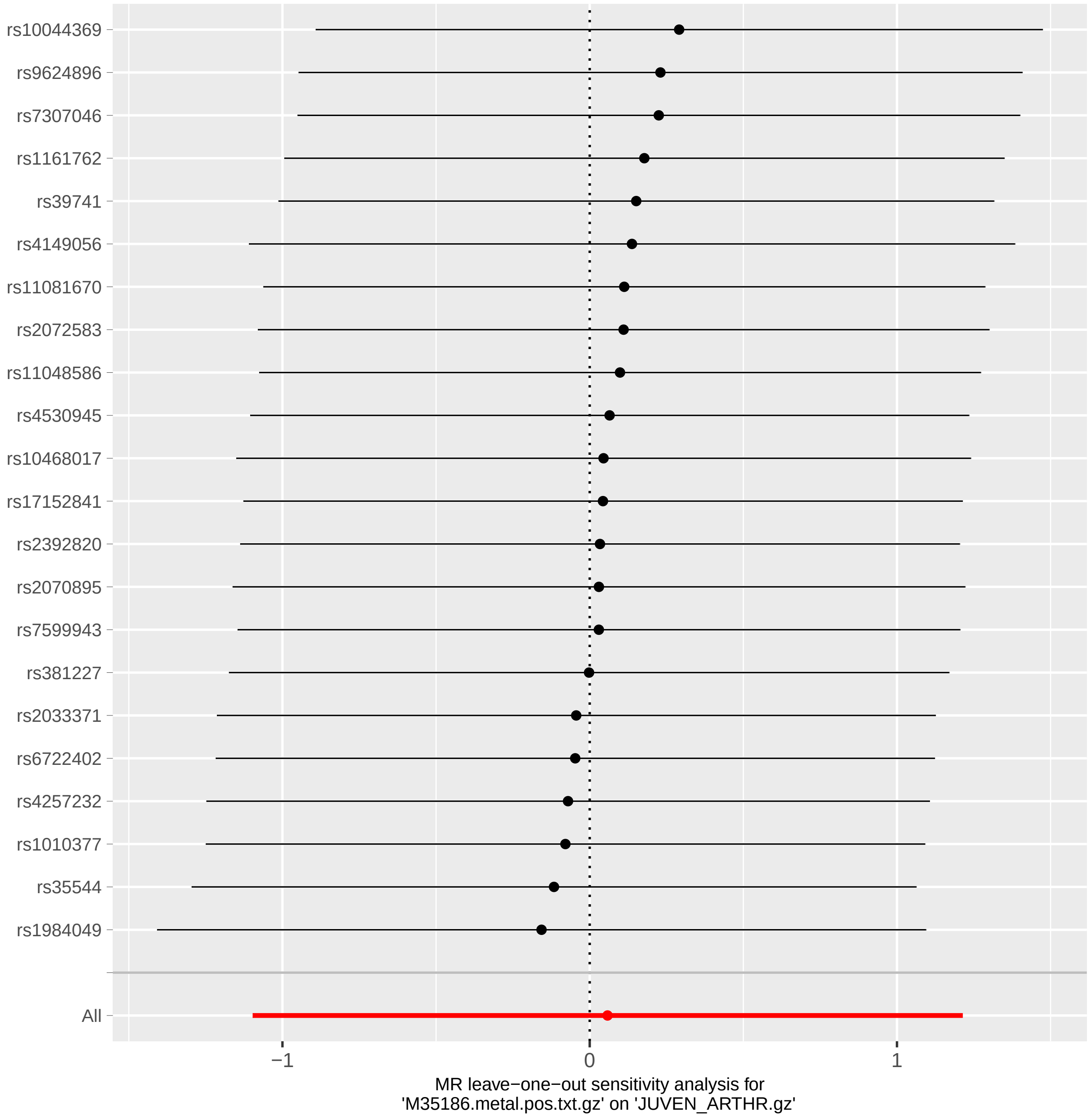
rs10044369
rs9624896
rs7307046
rs1161762
rs39741
rs4149056
rs11081670
rs2072583
rs11048586
rs4530945
rs10468017
rs17152841
rs2392820
rs2070895
rs7599943
rs381227
rs2033371
rs6722402
rs4257232
rs1010377
rs35544
rs1984049
All
−1
0
1
MR leave−one−out sensitivity analysis for
'M35186.metal.pos.txt.gz' on 'JUVEN_ARTHR.gz'

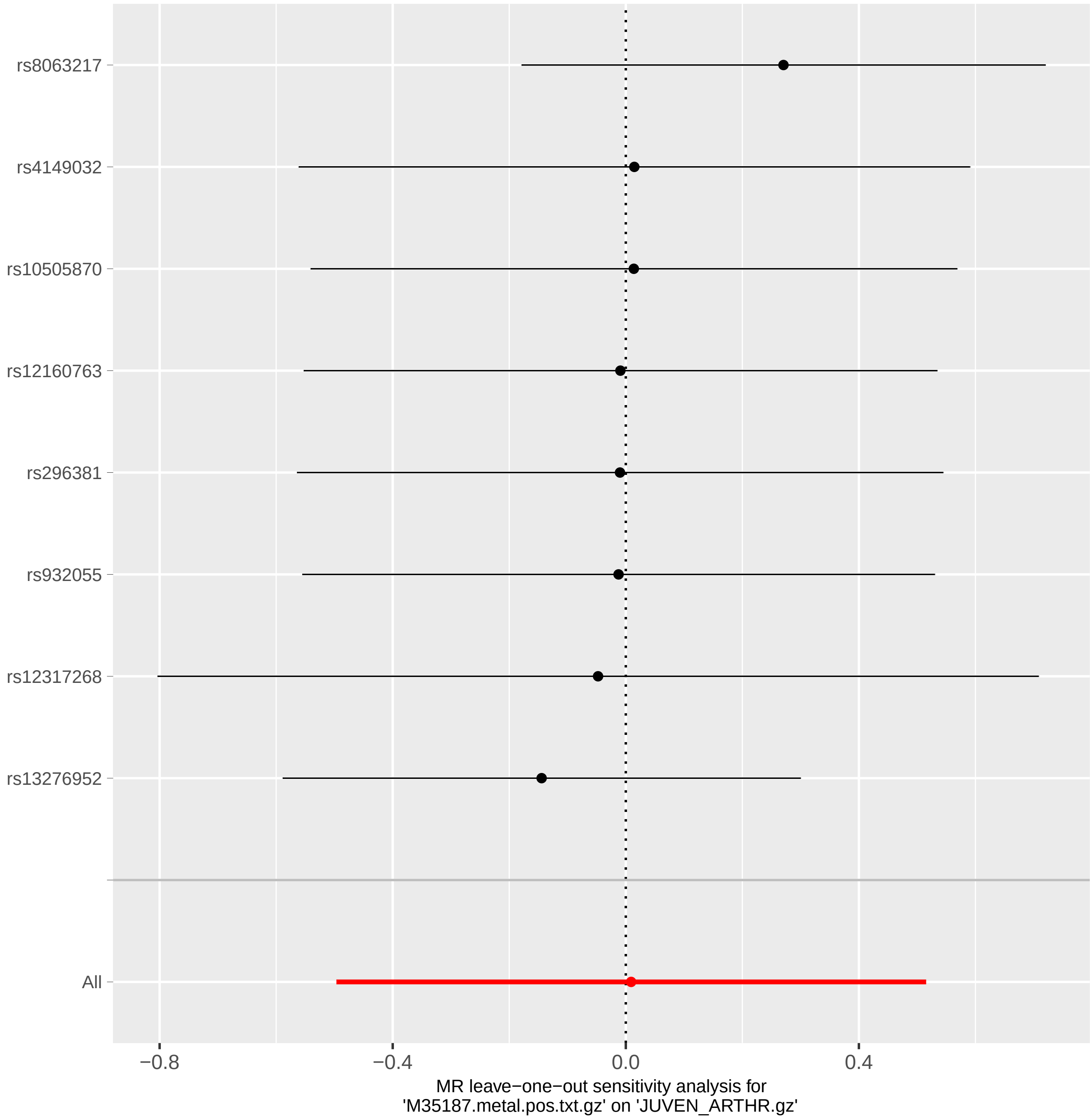
rs8063217
rs4149032
rs10505870
rs12160763
rs296381
rs932055
rs12317268
rs13276952
All
−0.8
−0.4
0.0
0.4
MR leave−one−out sensitivity analysis for
'M35187.metal.pos.txt.gz' on 'JUVEN_ARTHR.gz'

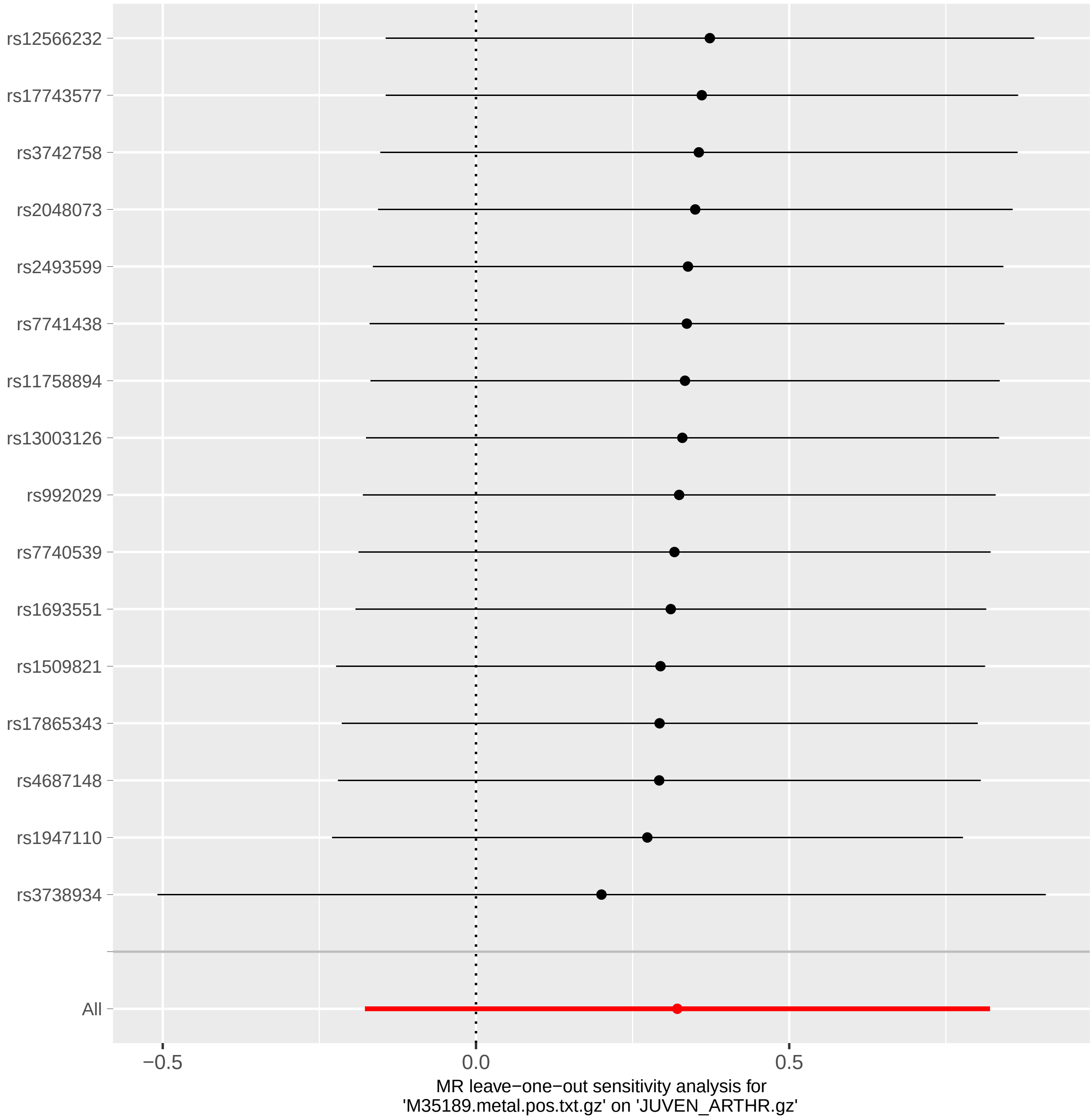

rs12566232
rs17743577
rs3742758
rs2048073
rs2493599
rs7741438
rs11758894
rs13003126
rs992029
rs7740539
rs1693551
rs1509821
rs17865343
rs4687148
rs1947110
rs3738934
All
−0.5
0.0
0.5
MR leave−one−out sensitivity analysis for
'M35189.metal.pos.txt.gz' on 'JUVEN_ARTHR.gz'

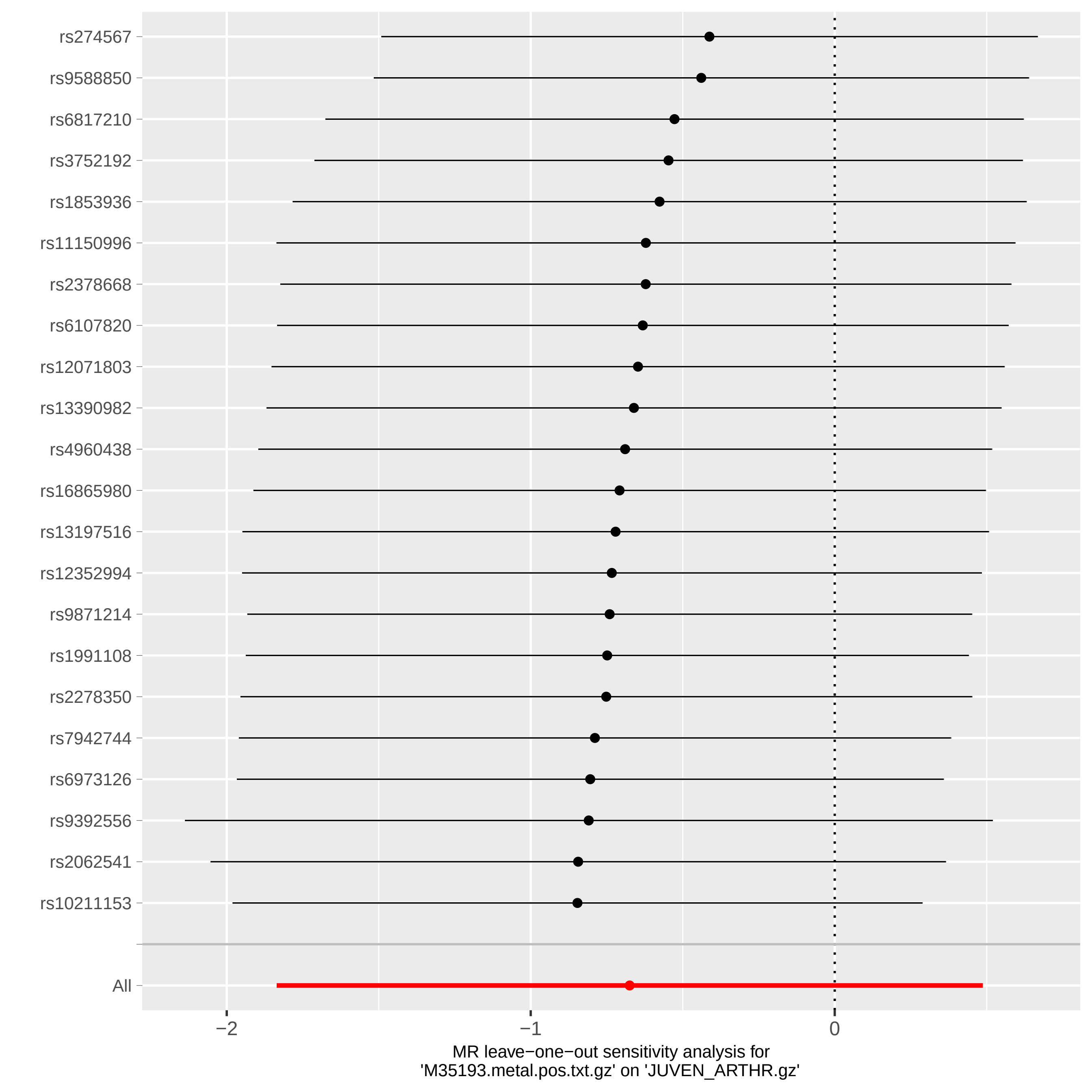

rs274567
rs9588850
rs6817210
rs3752192
rs1853936
rs11150996
rs2378668
rs6107820
rs12071803
rs13390982
rs4960438
rs16865980
rs13197516
rs12352994
rs9871214
rs1991108
rs2278350
rs7942744
rs6973126
rs9392556
rs2062541
rs10211153
All
−2
−1
0
MR leave−one−out sensitivity analysis for
'M35193.metal.pos.txt.gz' on 'JUVEN_ARTHR.gz'

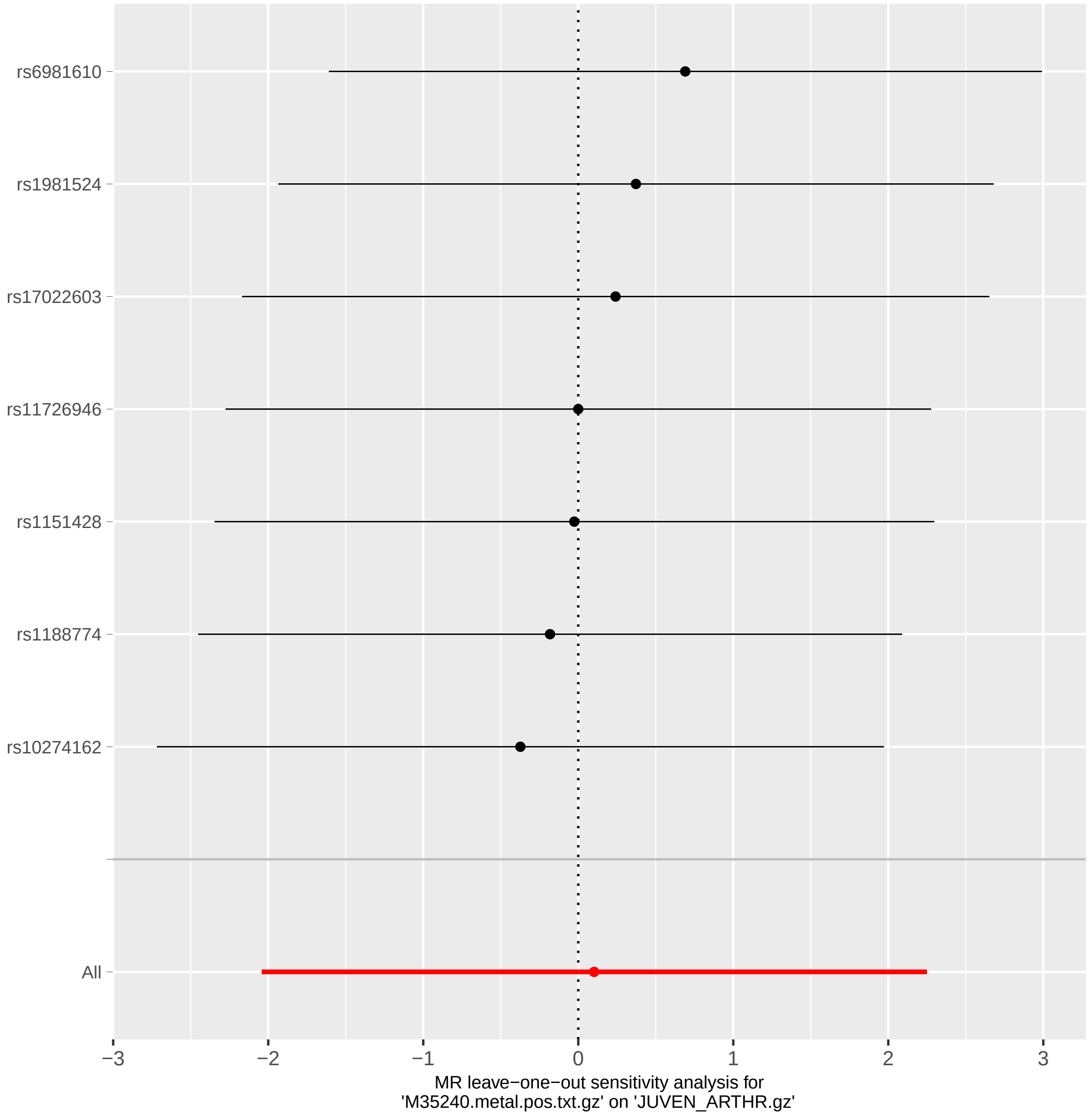

rs6981610
rs1981524
rs17022603
rs11726946
rs1151428
rs1188774
rs10274162
All
−3
−2
−1
0
1
2
3
MR leave−one−out sensitivity analysis for
'M35240.metal.pos.txt.gz' on 'JUVEN_ARTHR.gz'

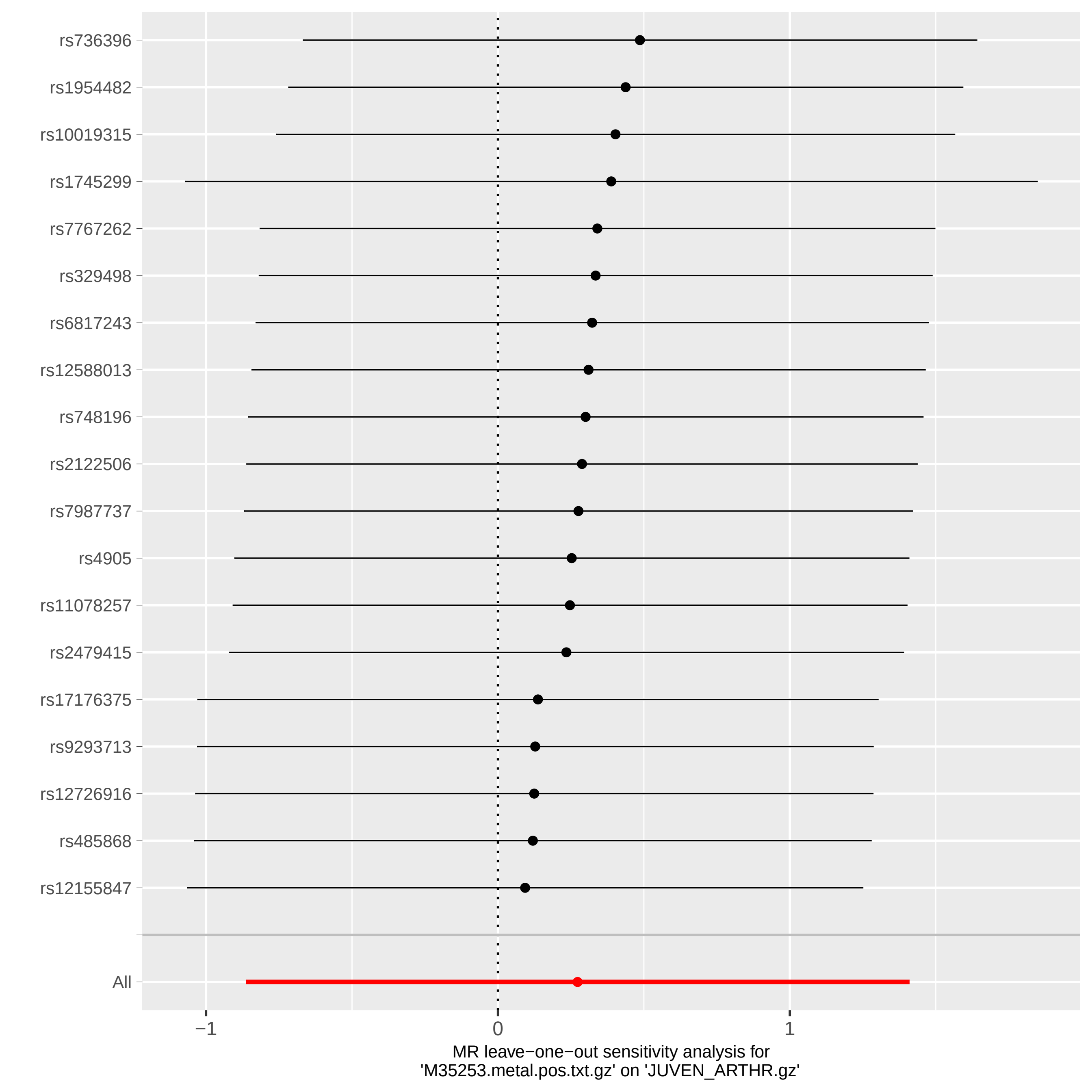

rs736396
rs1954482
rs10019315
rs1745299
rs7767262
rs329498
rs6817243
rs12588013
rs748196
rs2122506
rs7987737
rs4905
rs11078257
rs2479415
rs17176375
rs9293713
rs12726916
rs485868
rs12155847
All
−1
0
1
MR leave−one−out sensitivity analysis for
'M35253.metal.pos.txt.gz' on 'JUVEN_ARTHR.gz'

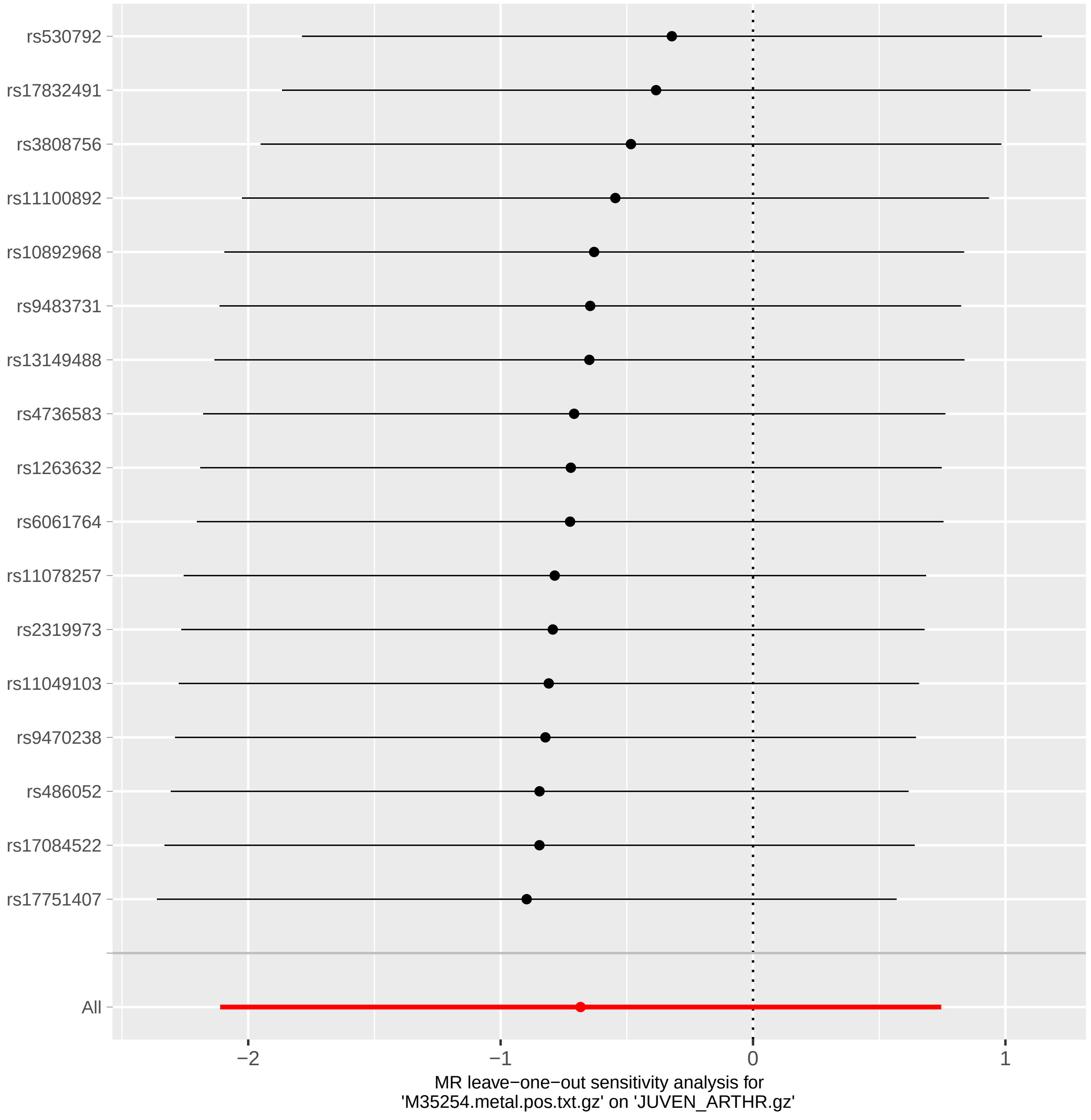
rs530792
rs17832491
rs3808756
rs11100892
rs10892968
rs9483731
rs13149488
rs4736583
rs1263632
rs6061764
rs11078257
rs2319973
rs11049103
rs9470238
rs486052
rs17084522
rs17751407
All
−2
−1
0
1
MR leave−one−out sensitivity analysis for
'M35254.metal.pos.txt.gz' on 'JUVEN_ARTHR.gz'

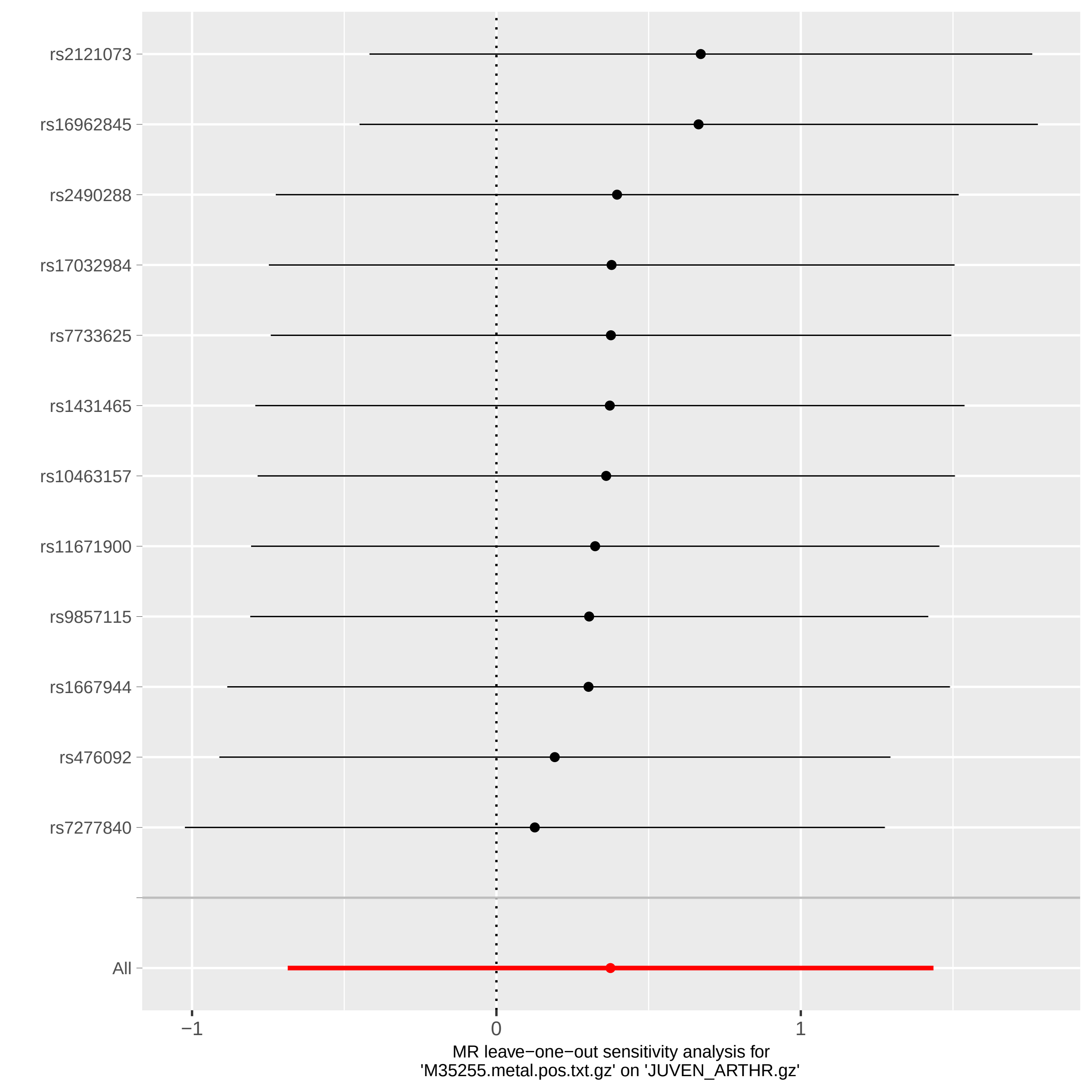
rs2121073
rs16962845
rs2490288
rs17032984
rs7733625
rs1431465
rs10463157
rs11671900
rs9857115
rs1667944
rs476092
rs7277840
All
−1
0
1
MR leave−one−out sensitivity analysis for
'M35255.metal.pos.txt.gz' on 'JUVEN_ARTHR.gz'

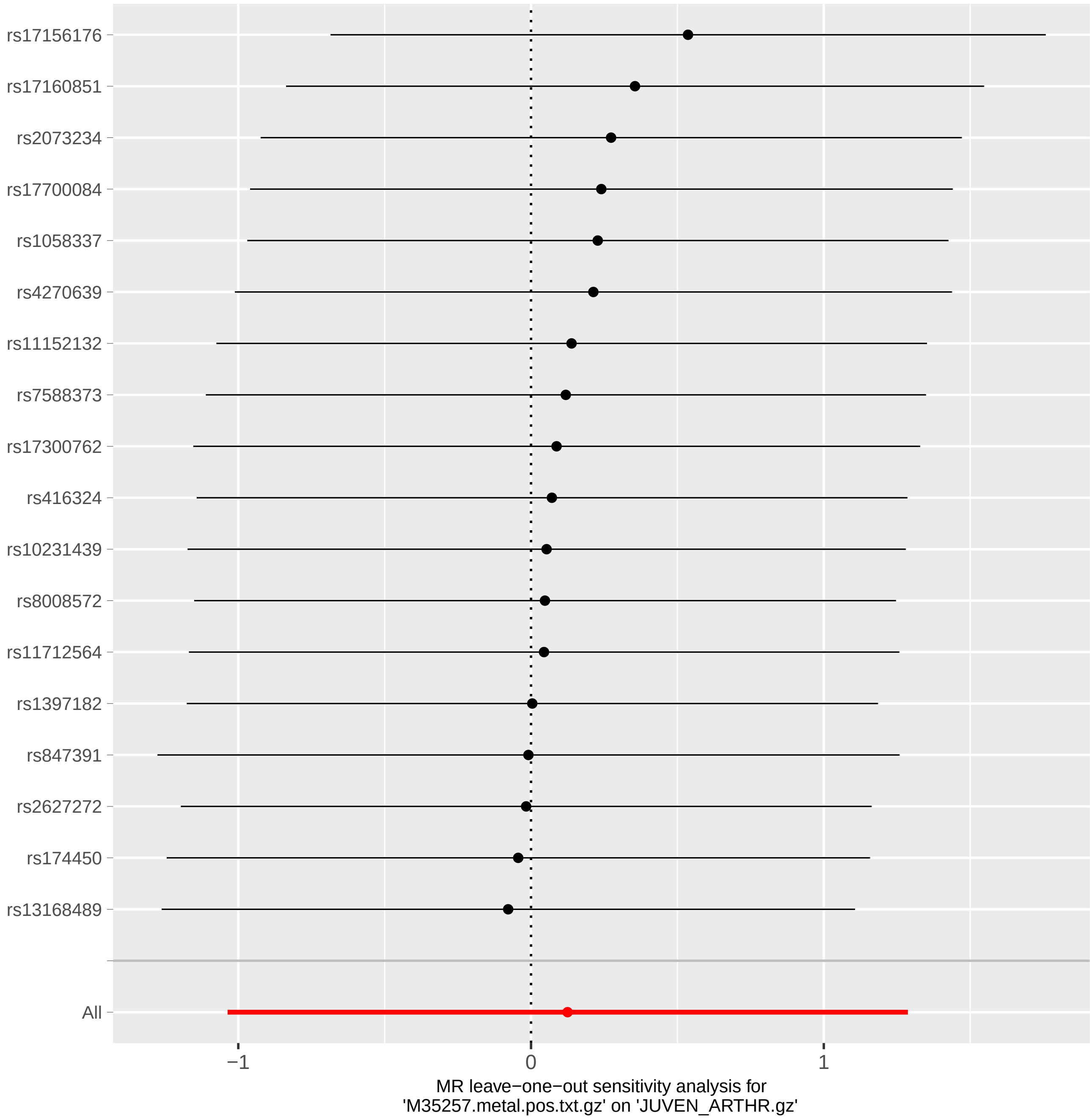
rs17156176
rs17160851
rs2073234
rs17700084
rs1058337
rs4270639
rs11152132
rs7588373
rs17300762
rs416324
rs10231439
rs8008572
rs11712564
rs1397182
rs847391
rs2627272
rs174450
rs13168489
All
−1
0
1
MR leave−one−out sensitivity analysis for
'M35257.metal.pos.txt.gz' on 'JUVEN_ARTHR.gz'

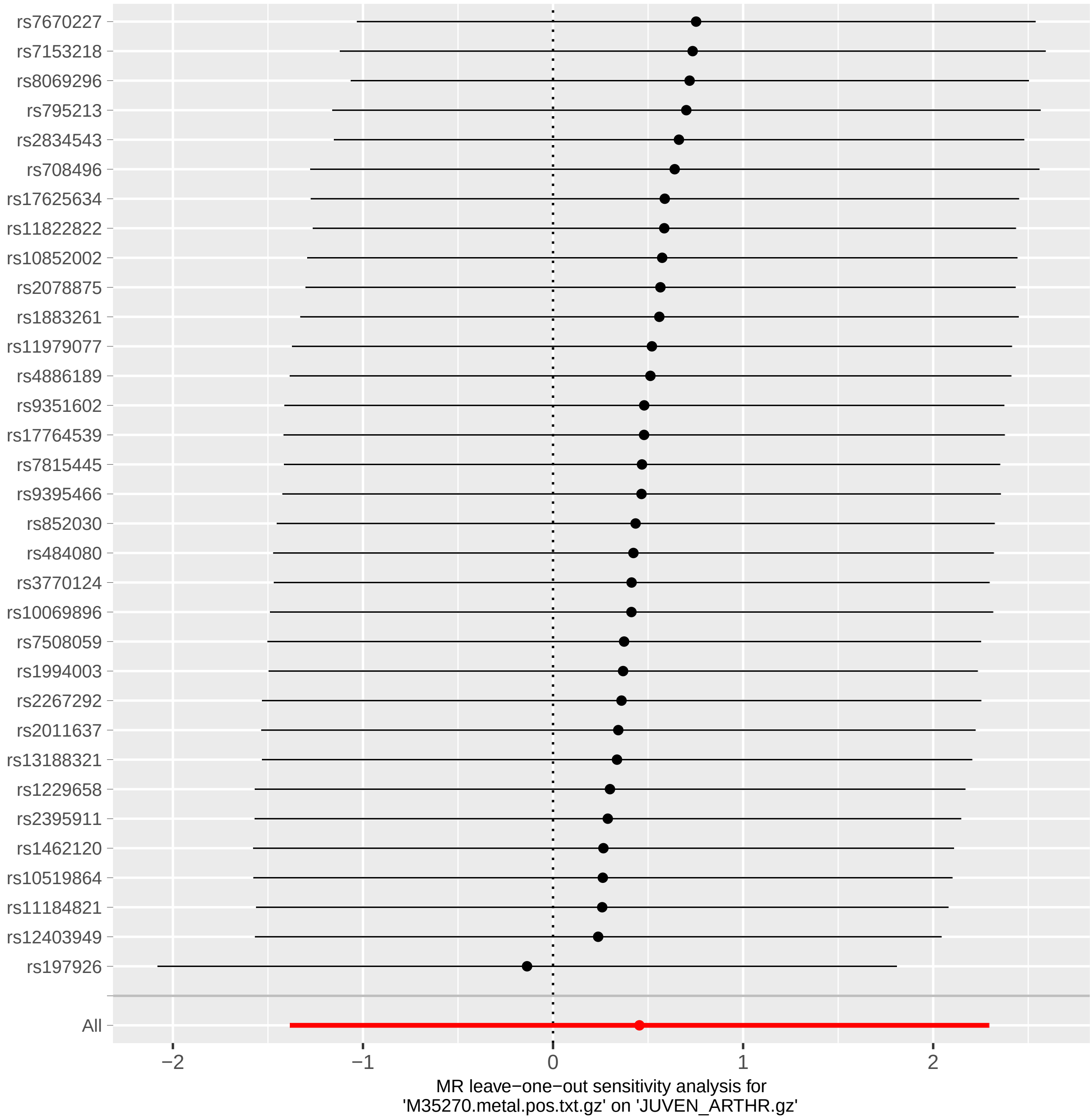
rs7670227
rs7153218
rs8069296
rs795213
rs2834543
rs708496
rs17625634
rs11822822
rs10852002
rs2078875
rs1883261
rs11979077
rs4886189
rs9351602
rs17764539
rs7815445
rs9395466
rs852030
rs484080
rs3770124
rs10069896
rs7508059
rs1994003
rs2267292
rs2011637
rs13188321
rs1229658
rs2395911
rs1462120
rs10519864
rs11184821
rs12403949
rs197926
All
−2
−1
0
1
2
MR leave−one−out sensitivity analysis for
'M35270.metal.pos.txt.gz' on 'JUVEN_ARTHR.gz'

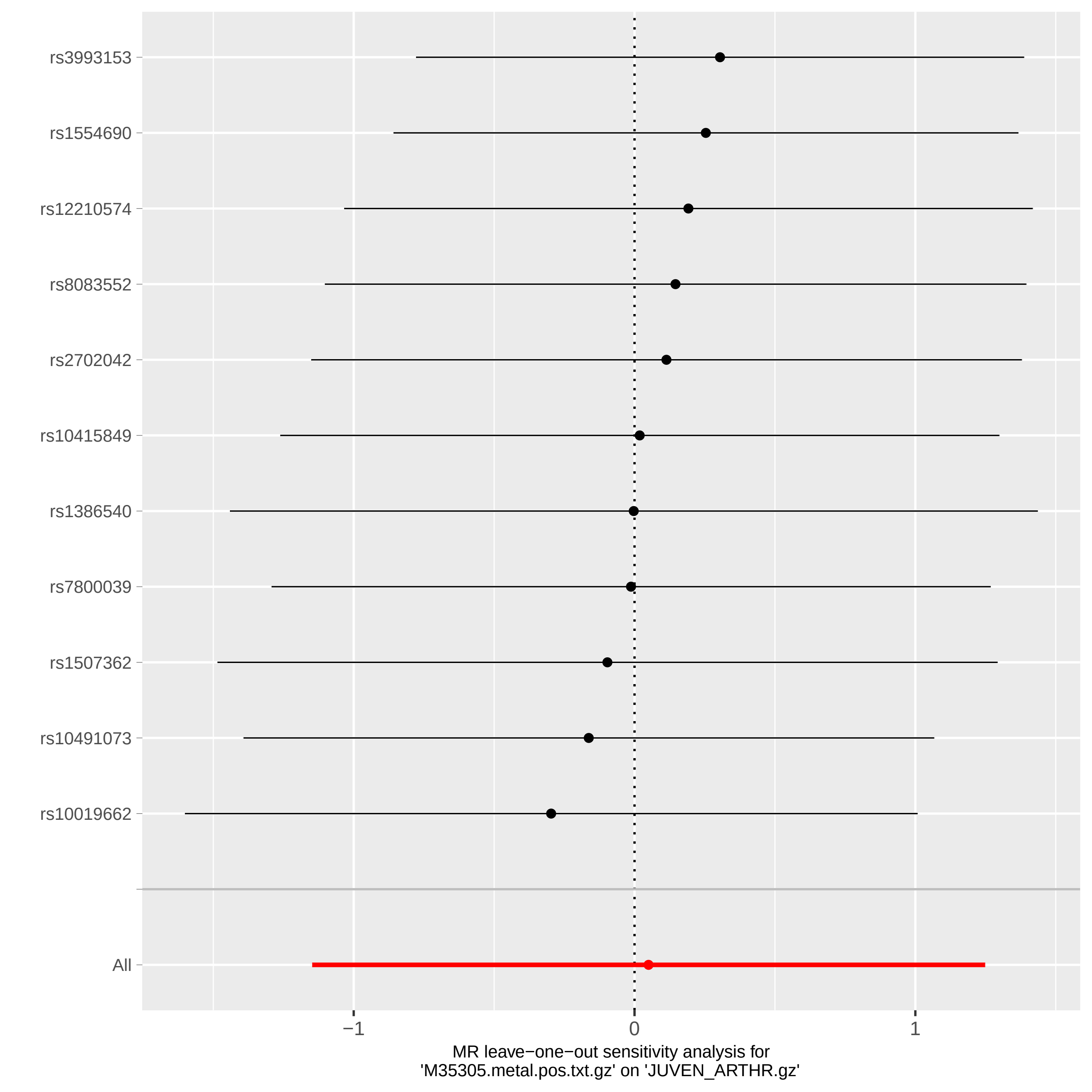

rs3993153
rs1554690
rs12210574
rs8083552
rs2702042
rs10415849
rs1386540
rs7800039
rs1507362
rs10491073
rs10019662
All
−1
0
1
MR leave−one−out sensitivity analysis for
'M35305.metal.pos.txt.gz' on 'JUVEN_ARTHR.gz'

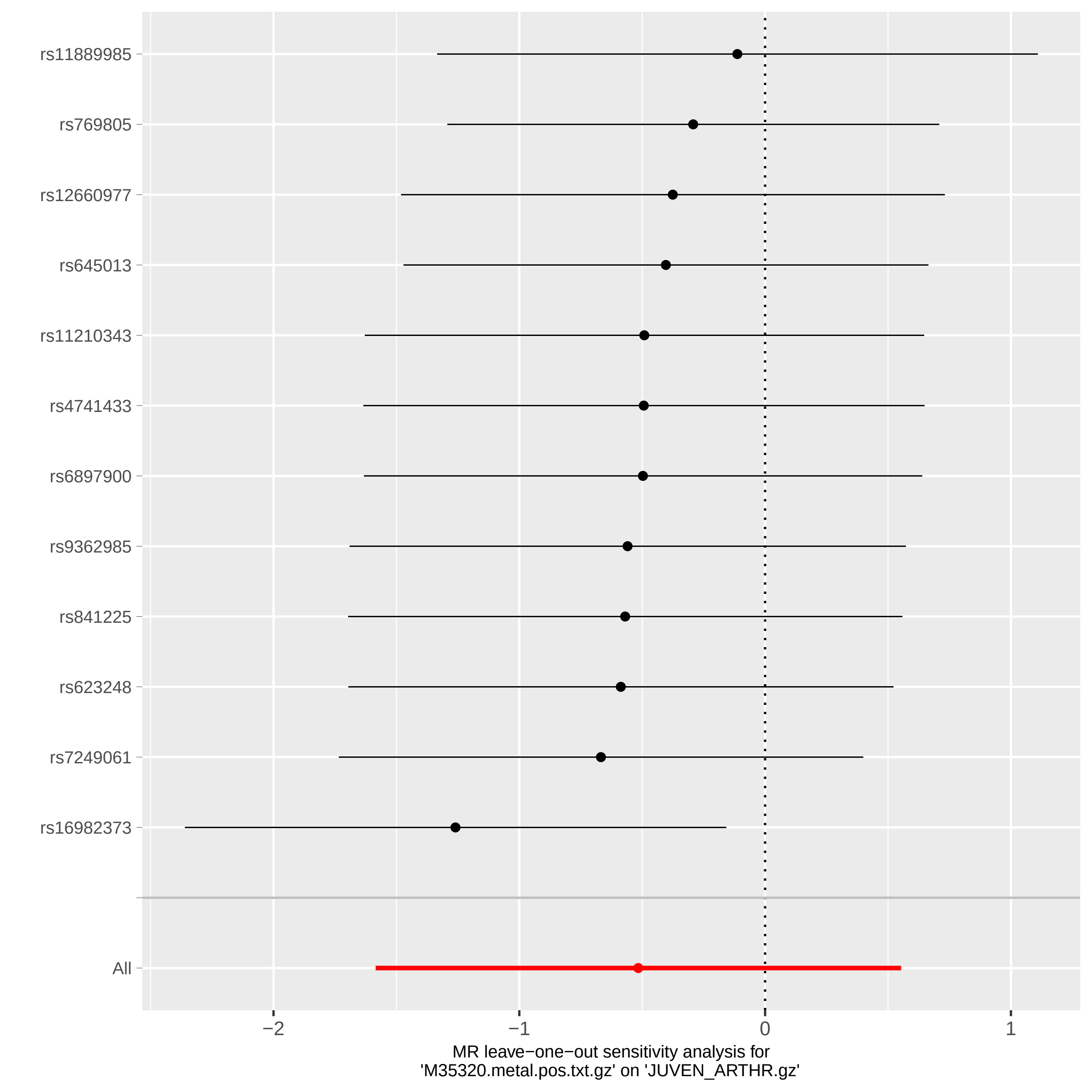

rs11889985
rs769805
rs12660977
rs645013
rs11210343
rs4741433
rs6897900
rs9362985
rs841225
rs623248
rs7249061
rs16982373
All
−2
−1
0
1
MR leave−one−out sensitivity analysis for
'M35320.metal.pos.txt.gz' on 'JUVEN_ARTHR.gz'

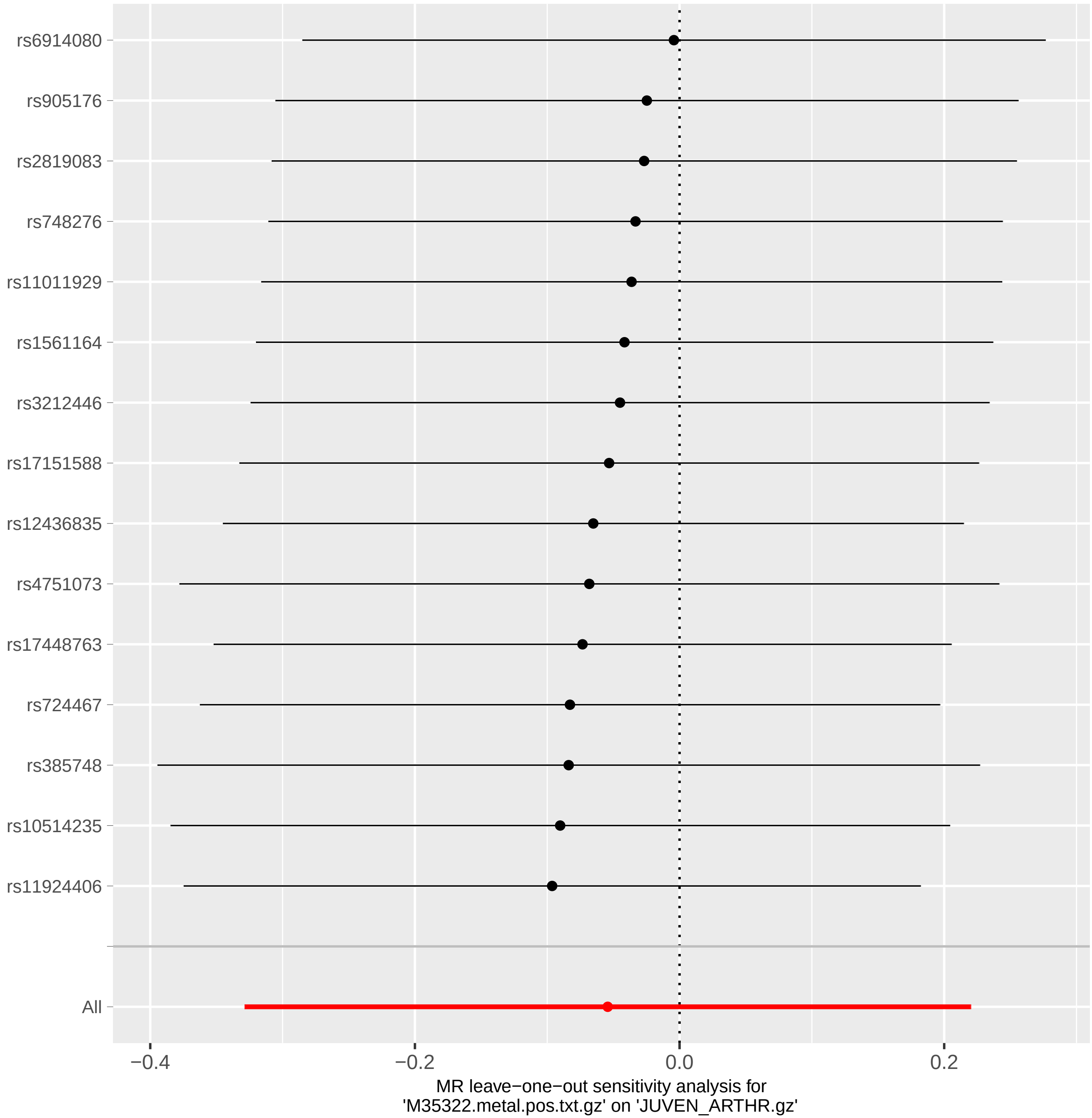
rs6914080
rs905176
rs2819083
rs748276
rs11011929
rs1561164
rs3212446
rs17151588
rs12436835
rs4751073
rs17448763
rs724467
rs385748
rs10514235
rs11924406
All
−0.4
−0.2
0.0
0.2
MR leave−one−out sensitivity analysis for
'M35322.metal.pos.txt.gz' on 'JUVEN_ARTHR.gz'

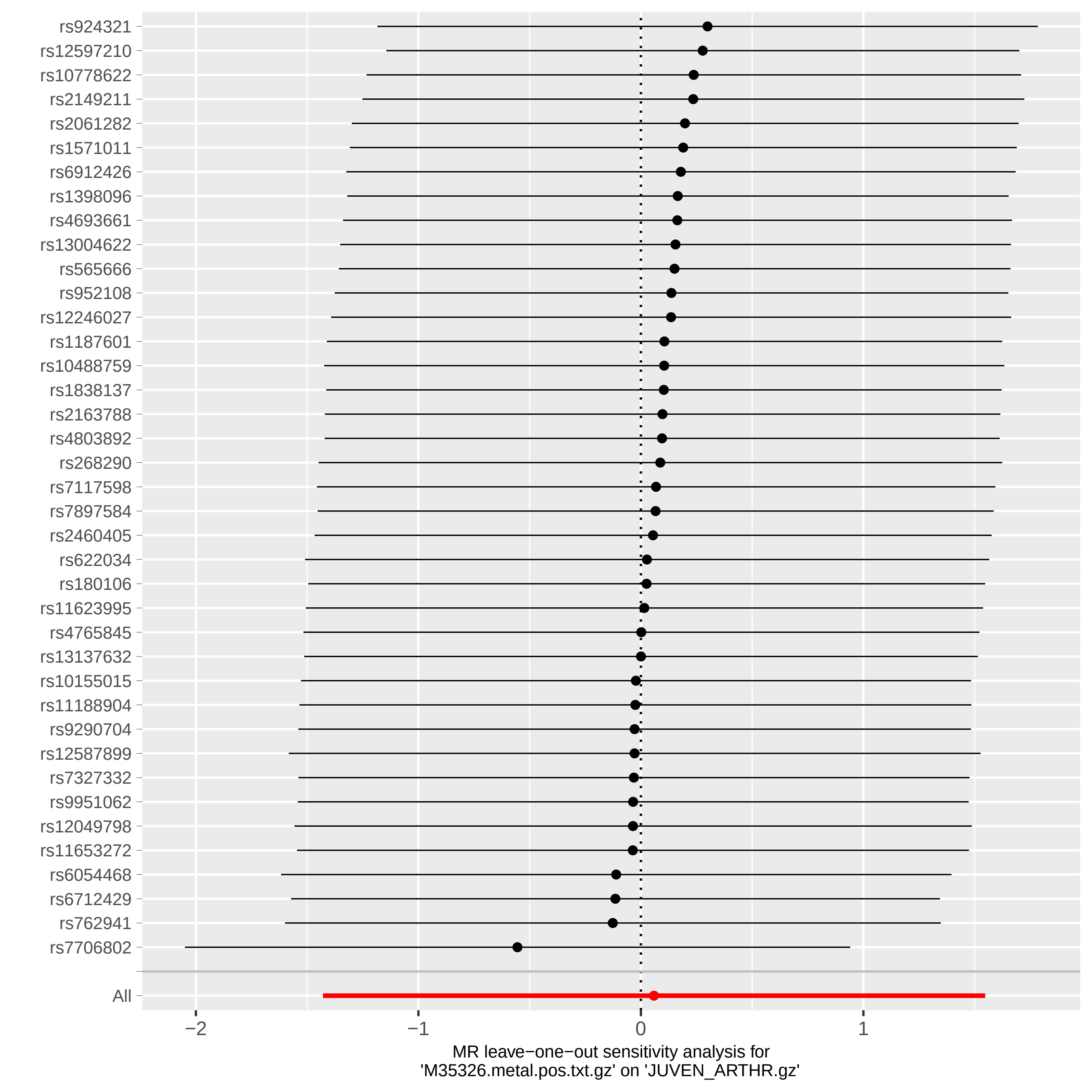
rs924321
rs12597210
rs10778622
rs2149211
rs2061282
rs1571011
rs6912426
rs1398096
rs4693661
rs13004622
rs565666
rs952108
rs12246027
rs1187601
rs10488759
rs1838137
rs2163788
rs4803892
rs268290
rs7117598
rs7897584
rs2460405
rs622034
rs180106
rs11623995
rs4765845
rs13137632
rs10155015
rs11188904
rs9290704
rs12587899
rs7327332
rs9951062
rs12049798
rs11653272
rs6054468
rs6712429
rs762941
rs7706802
All
−2
−1
0
1
MR leave−one−out sensitivity analysis for
'M35326.metal.pos.txt.gz' on 'JUVEN_ARTHR.gz'

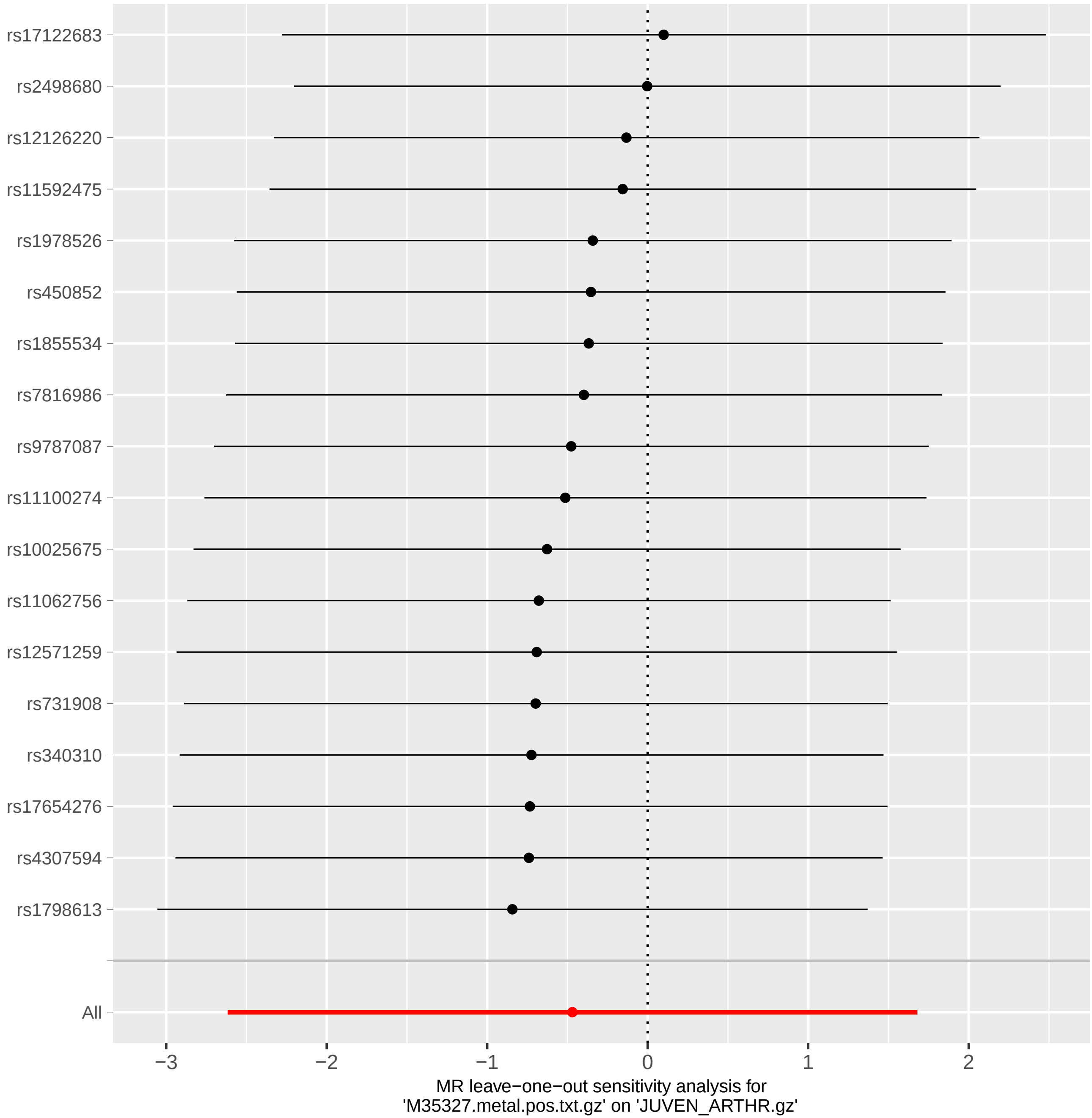
rs17122683
rs2498680
rs12126220
rs11592475
rs1978526
rs450852
rs1855534
rs7816986
rs9787087
rs11100274
rs10025675
rs11062756
rs12571259
rs731908
rs340310
rs17654276
rs4307594
rs1798613
All
−3
−2
−1
0
1
2
MR leave−one−out sensitivity analysis for
'M35327.metal.pos.txt.gz' on 'JUVEN_ARTHR.gz'

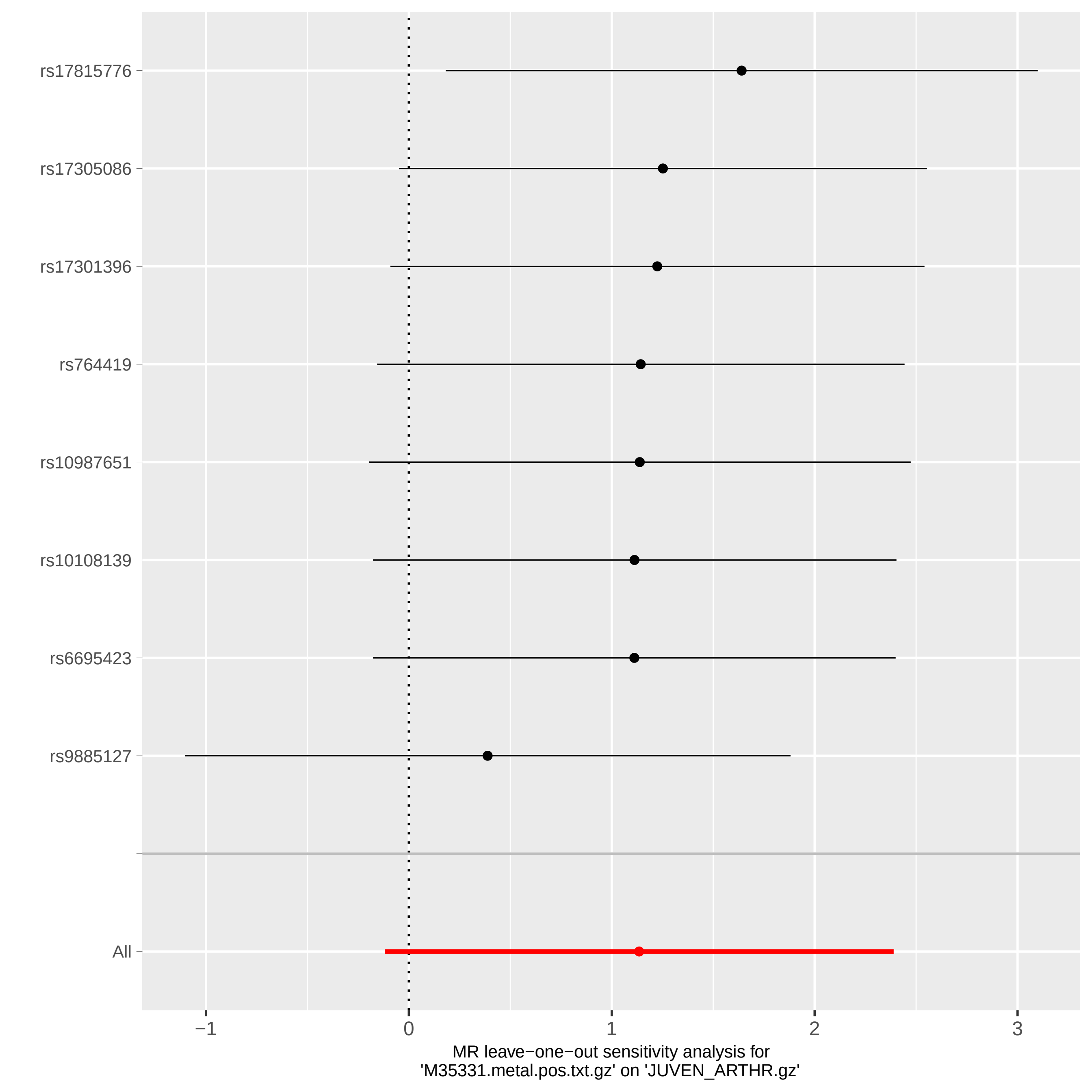

rs17815776
rs17305086
rs17301396
rs764419
rs10987651
rs10108139
rs6695423
rs9885127
All
−1
0
1
2
3
MR leave−one−out sensitivity analysis for
'M35331.metal.pos.txt.gz' on 'JUVEN_ARTHR.gz'

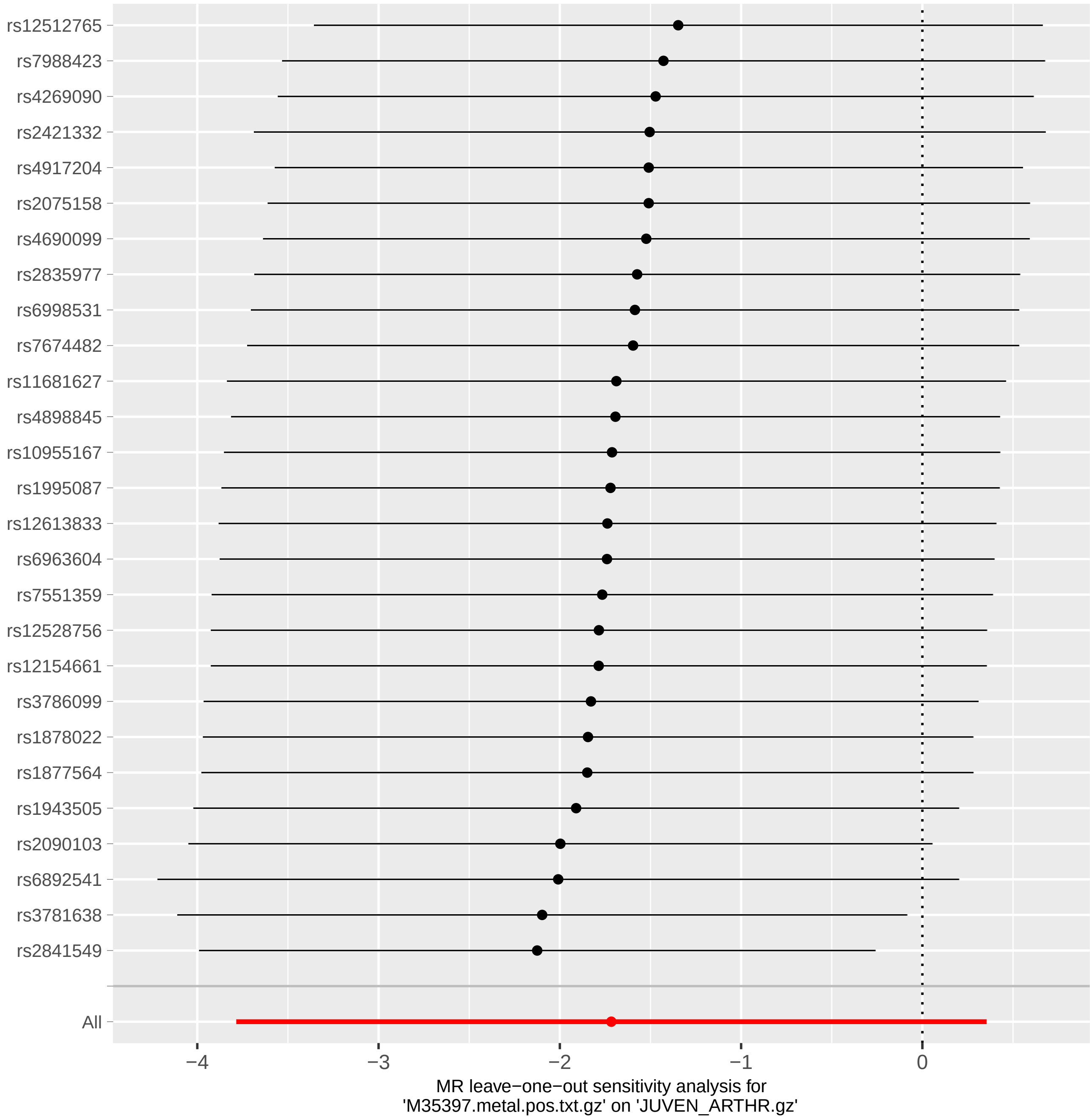
rs12512765
rs7988423
rs4269090
rs2421332
rs4917204
rs2075158
rs4690099
rs2835977
rs6998531
rs7674482
rs11681627
rs4898845
rs10955167
rs1995087
rs12613833
rs6963604
rs7551359
rs12528756
rs12154661
rs3786099
rs1878022
rs1877564
rs1943505
rs2090103
rs6892541
rs3781638
rs2841549
All
−4
−3
−2
−1
0
MR leave−one−out sensitivity analysis for
'M35397.metal.pos.txt.gz' on 'JUVEN_ARTHR.gz'

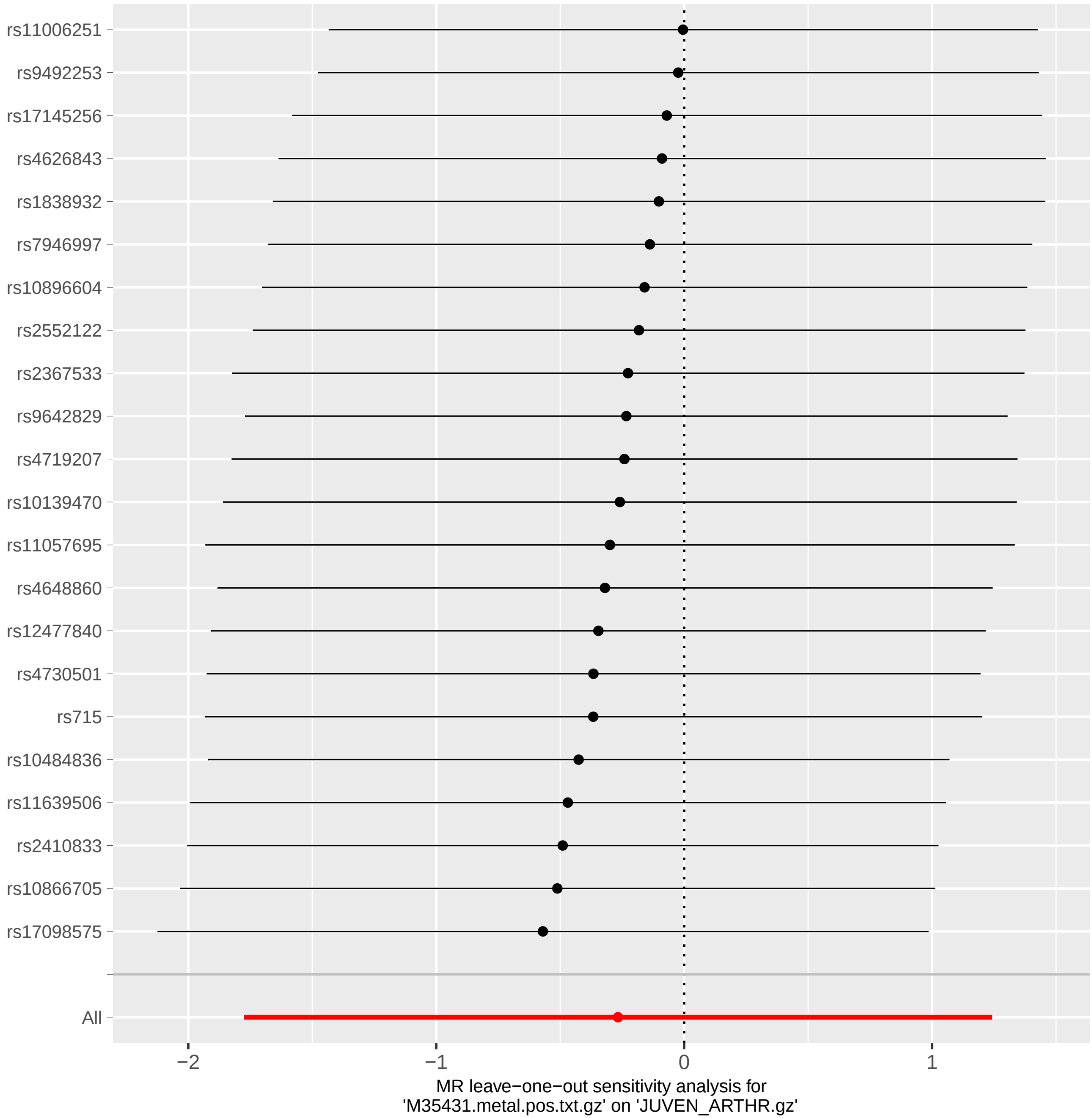
rs11006251
rs9492253
rs17145256
rs4626843
rs1838932
rs7946997
rs10896604
rs2552122
rs2367533
rs9642829
rs4719207
rs10139470
rs11057695
rs4648860
rs12477840
rs4730501
rs715
rs10484836
rs11639506
rs2410833
rs10866705
rs17098575
All
−2
−1
0
1
MR leave−one−out sensitivity analysis for
'M35431.metal.pos.txt.gz' on 'JUVEN_ARTHR.gz'

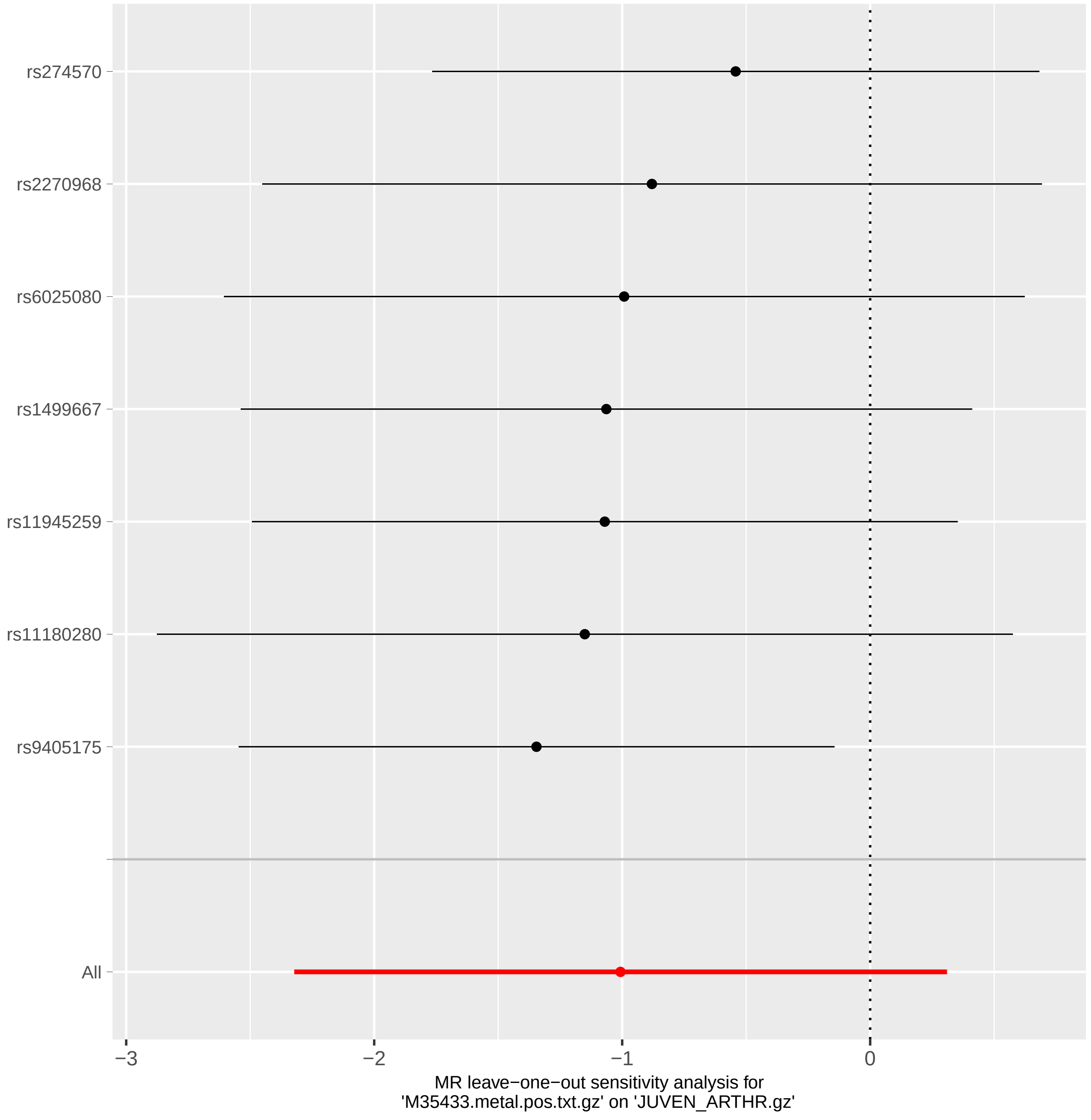
rs274570
rs2270968
rs6025080
rs1499667
rs11945259
rs11180280
rs9405175
All
−3
−2
−1
0
MR leave−one−out sensitivity analysis for
'M35433.metal.pos.txt.gz' on 'JUVEN_ARTHR.gz'

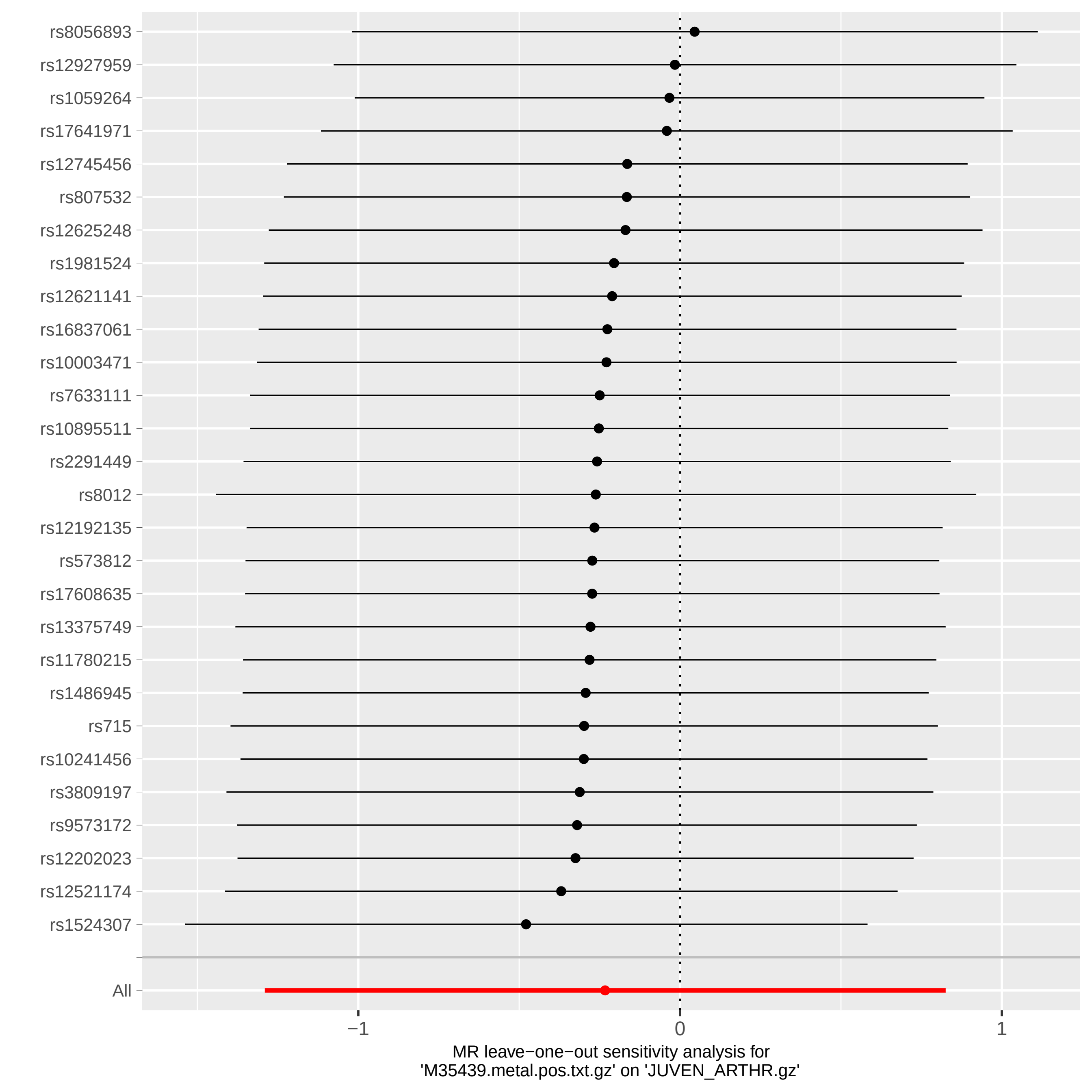
rs8056893
rs12927959
rs1059264
rs17641971
rs12745456
rs807532
rs12625248
rs1981524
rs12621141
rs16837061
rs10003471
rs7633111
rs10895511
rs2291449
rs8012
rs12192135
rs573812
rs17608635
rs13375749
rs11780215
rs1486945
rs715
rs10241456
rs3809197
rs9573172
rs12202023
rs12521174
rs1524307
All
−1
0
1
MR leave−one−out sensitivity analysis for
'M35439.metal.pos.txt.gz' on 'JUVEN_ARTHR.gz'

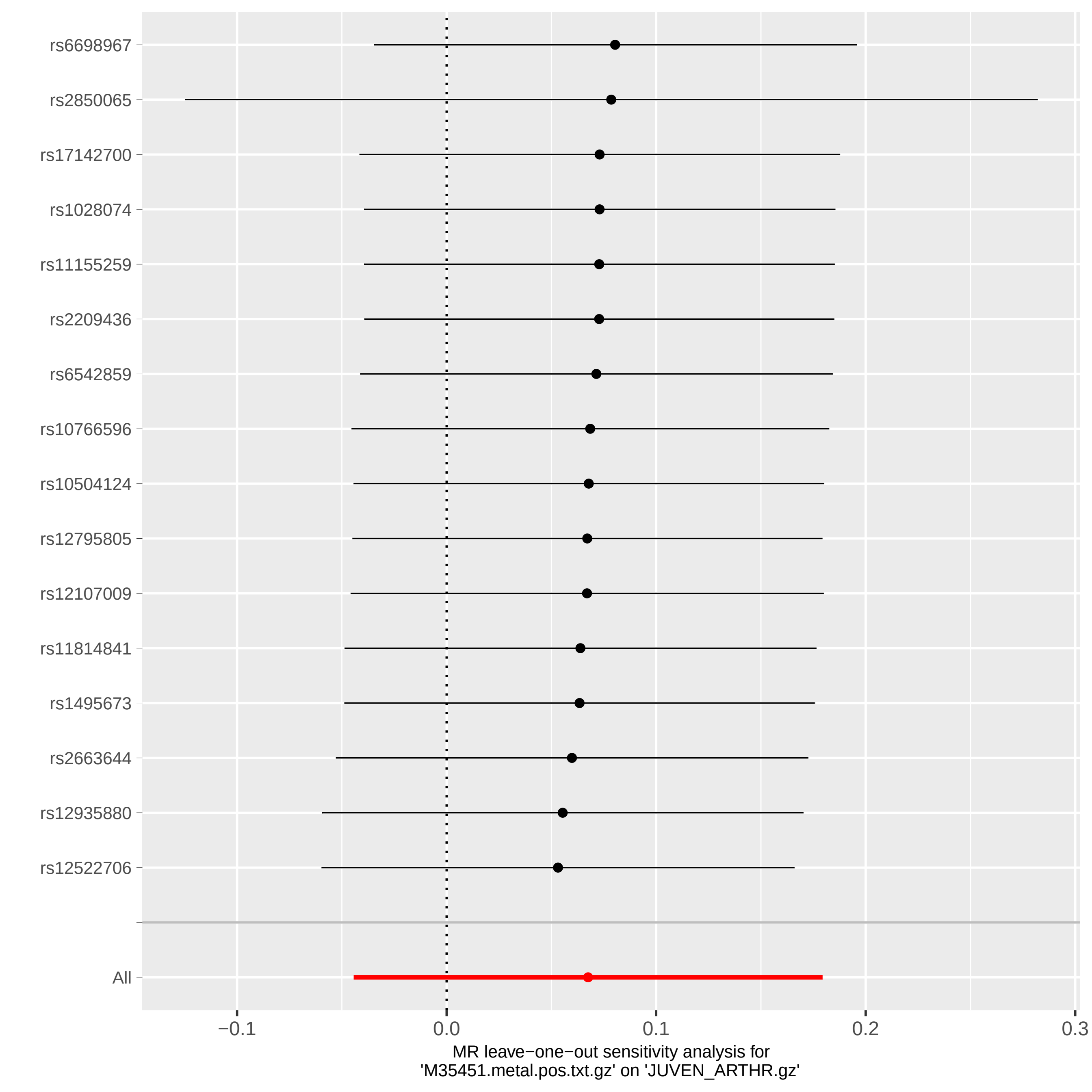
rs6698967
rs2850065
rs17142700
rs1028074
rs11155259
rs2209436
rs6542859
rs10766596
rs10504124
rs12795805
rs12107009
rs11814841
rs1495673
rs2663644
rs12935880
rs12522706
All
−0.1
0.0
0.1
0.2
0.3
MR leave−one−out sensitivity analysis for
'M35451.metal.pos.txt.gz' on 'JUVEN_ARTHR.gz'

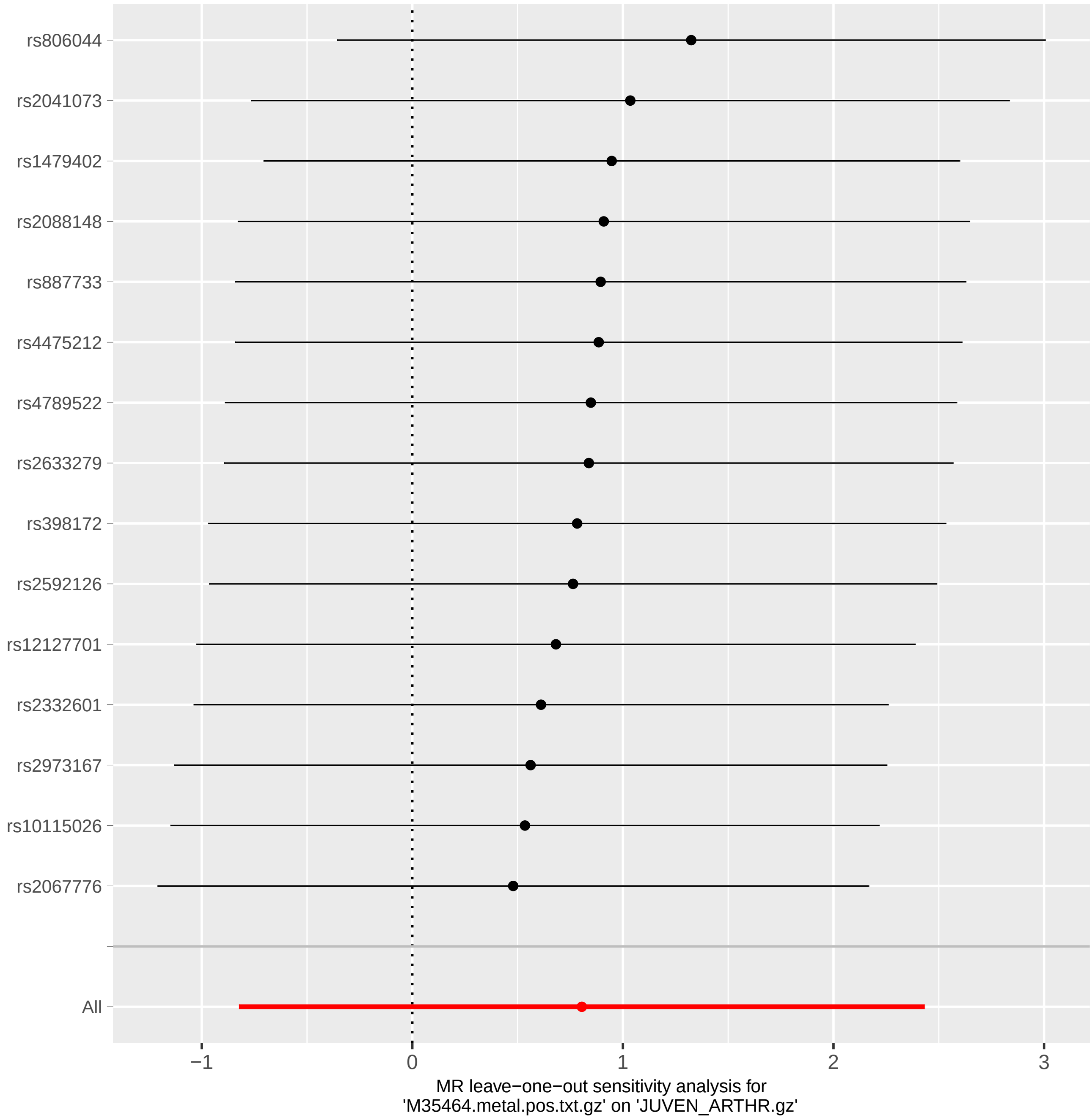

rs806044
rs2041073
rs1479402
rs2088148
rs887733
rs4475212
rs4789522
rs2633279
rs398172
rs2592126
rs12127701
rs2332601
rs2973167
rs10115026
rs2067776
All
−1
0
1
2
3
MR leave−one−out sensitivity analysis for
'M35464.metal.pos.txt.gz' on 'JUVEN_ARTHR.gz'

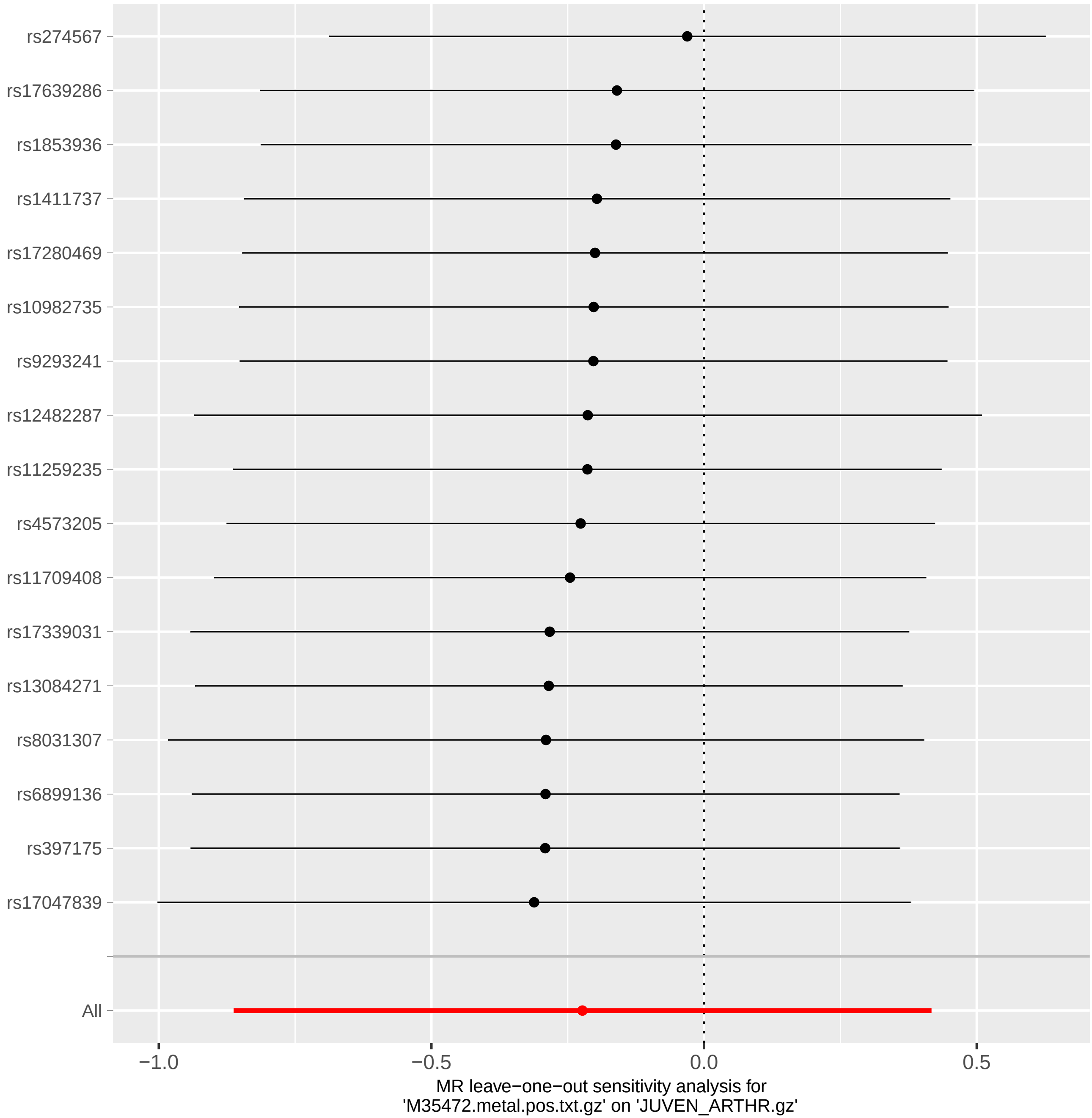

rs274567
rs17639286
rs1853936
rs1411737
rs17280469
rs10982735
rs9293241
rs12482287
rs11259235
rs4573205
rs11709408
rs17339031
rs13084271
rs8031307
rs6899136
rs397175
rs17047839
All
−1.0
−0.5
0.0
0.5
MR leave−one−out sensitivity analysis for
'M35472.metal.pos.txt.gz' on 'JUVEN_ARTHR.gz'

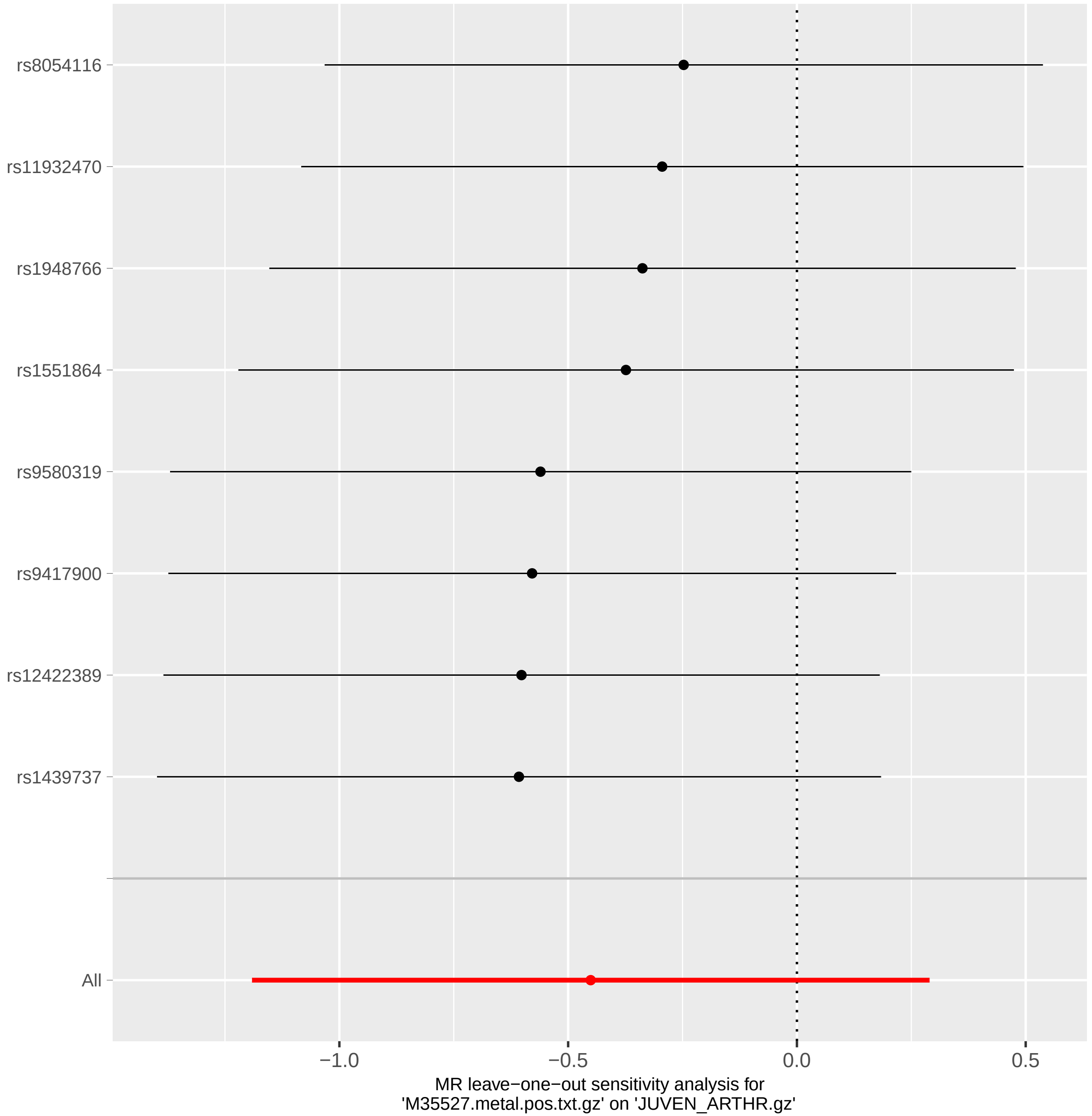
rs8054116
rs11932470
rs1948766
rs1551864
rs9580319
rs9417900
rs12422389
rs1439737
All
−1.0
−0.5
0.0
0.5
MR leave−one−out sensitivity analysis for
'M35527.metal.pos.txt.gz' on 'JUVEN_ARTHR.gz'

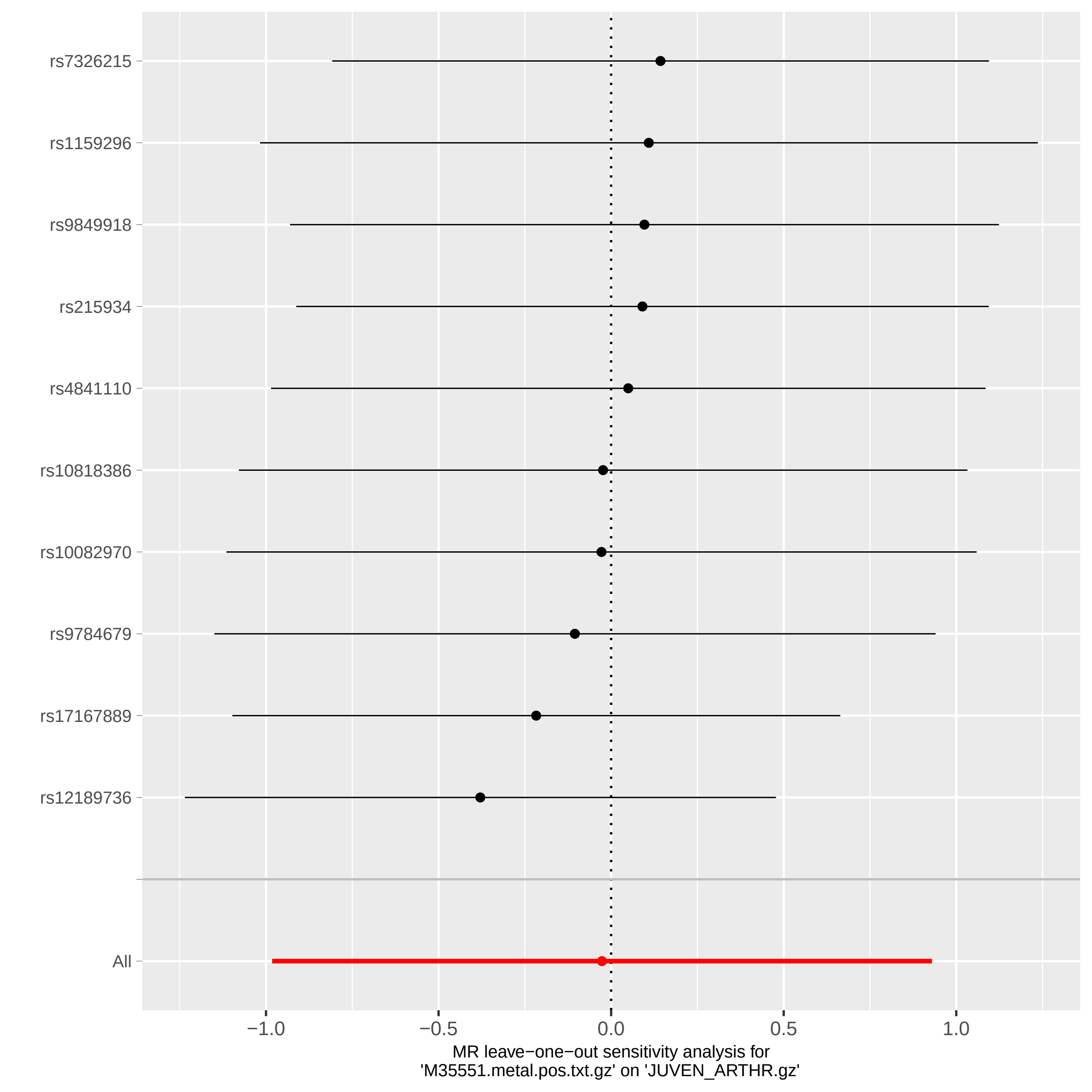
rs7326215
rs1159296
rs9849918
rs215934
rs4841110
rs10818386
rs10082970
rs9784679
rs17167889
rs12189736
All
−1.0
−0.5
0.0
0.5
1.0
MR leave−one−out sensitivity analysis for
'M35551.metal.pos.txt.gz' on 'JUVEN_ARTHR.gz'

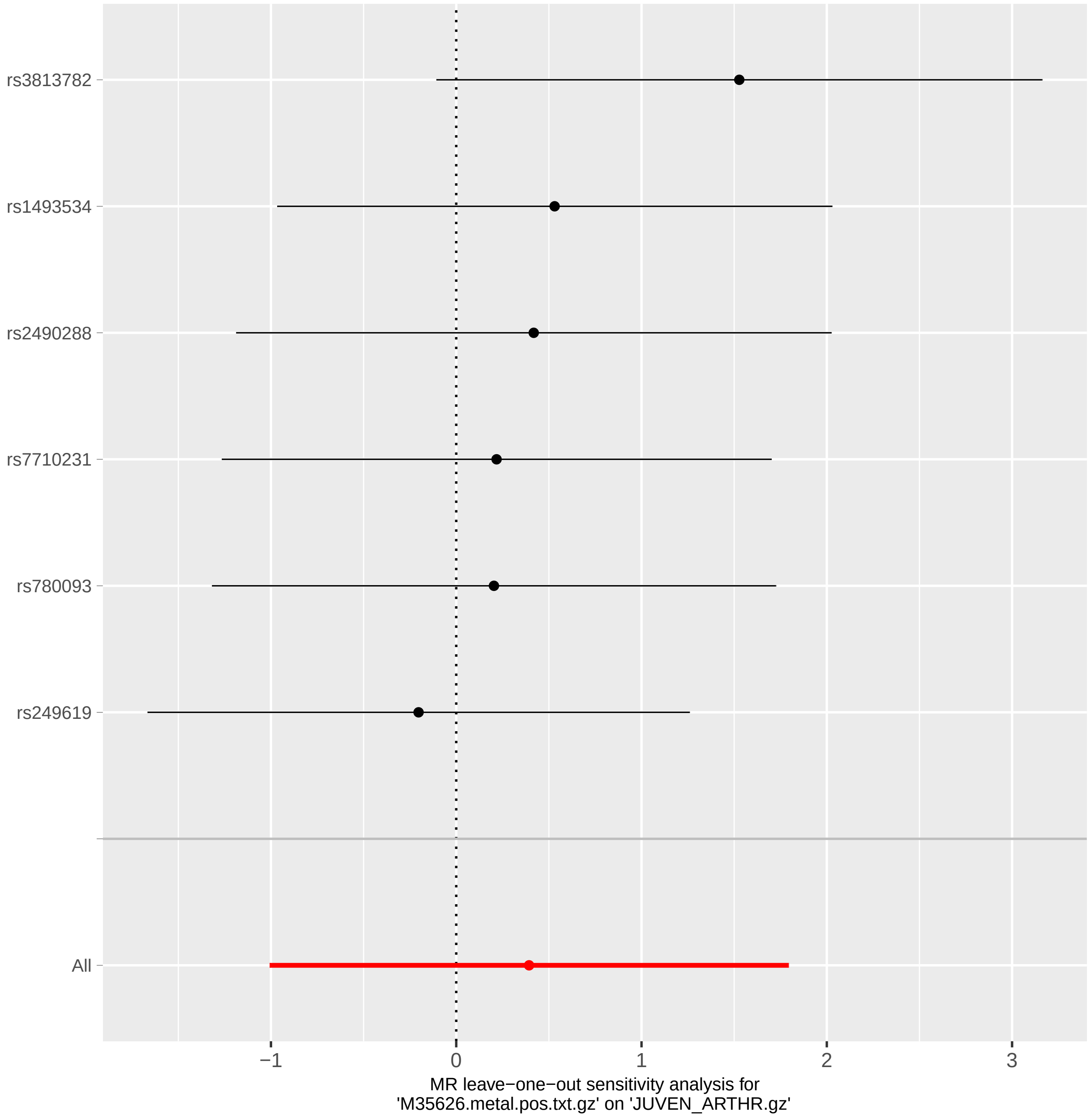
rs3813782
rs1493534
rs2490288
rs7710231
rs780093
rs249619
All
−1
0
1
2
3
MR leave−one−out sensitivity analysis for
'M35626.metal.pos.txt.gz' on 'JUVEN_ARTHR.gz'

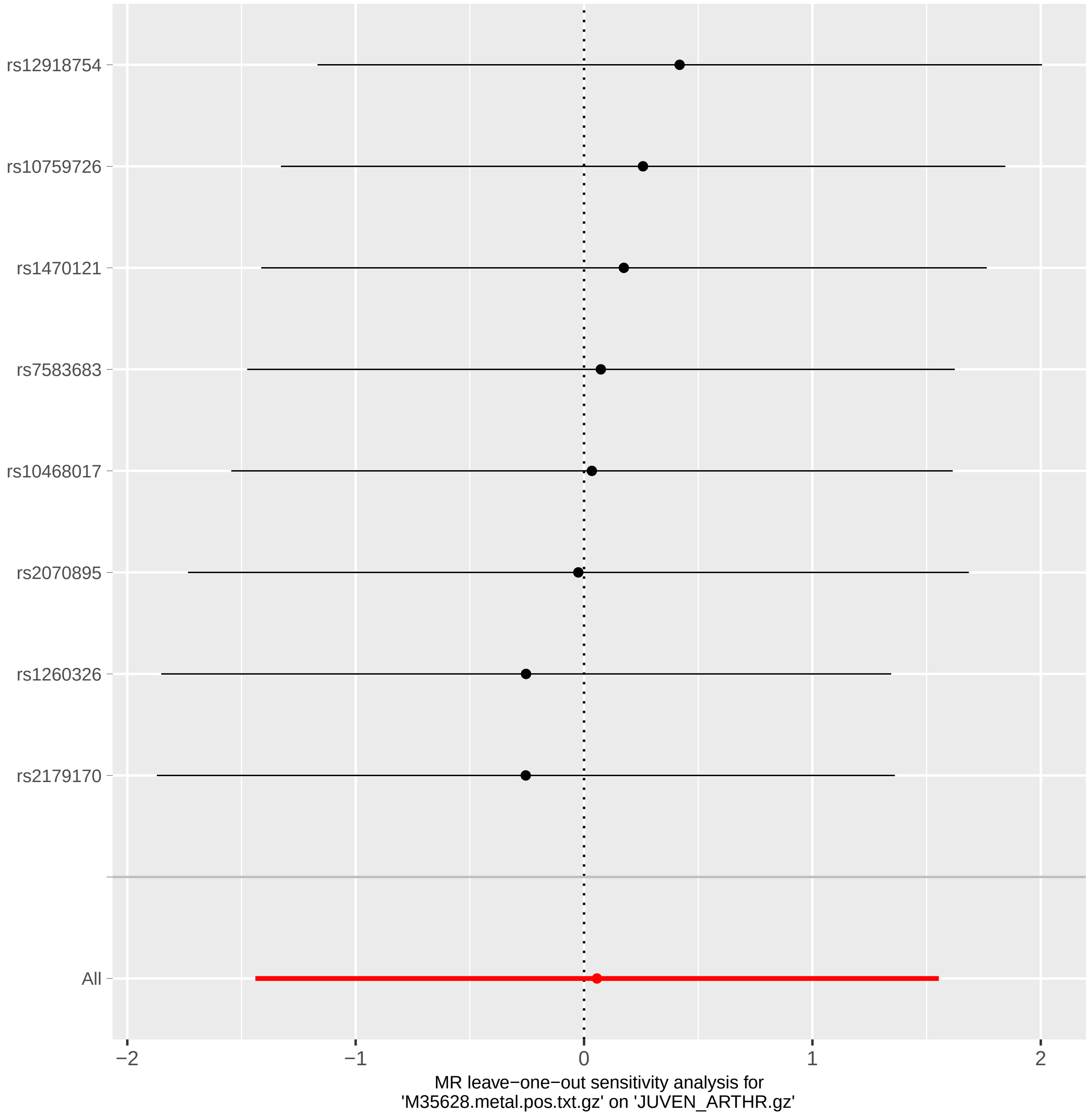
rs12918754
rs10759726
rs1470121
rs7583683
rs10468017
rs2070895
rs1260326
rs2179170
All
−2
−1
0
1
2
MR leave−one−out sensitivity analysis for
'M35628.metal.pos.txt.gz' on 'JUVEN_ARTHR.gz'

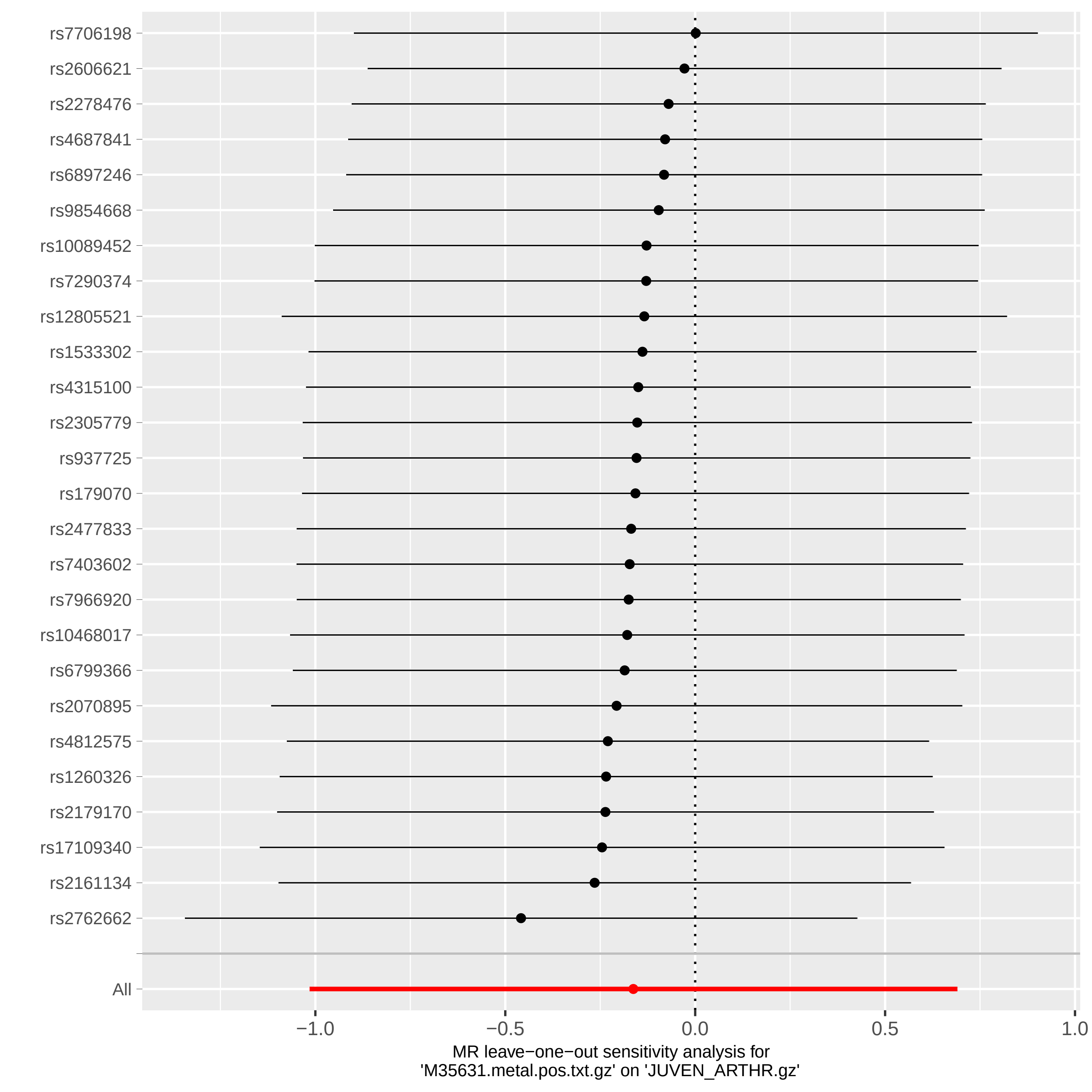

rs7706198
rs2606621
rs2278476
rs4687841
rs6897246
rs9854668
rs10089452
rs7290374
rs12805521
rs1533302
rs4315100
rs2305779
rs937725
rs179070
rs2477833
rs7403602
rs7966920
rs10468017
rs6799366
rs2070895
rs4812575
rs1260326
rs2179170
rs17109340
rs2161134
rs2762662
All
−1.0
−0.5
0.0
0.5
1.0
MR leave−one−out sensitivity analysis for
'M35631.metal.pos.txt.gz' on 'JUVEN_ARTHR.gz'

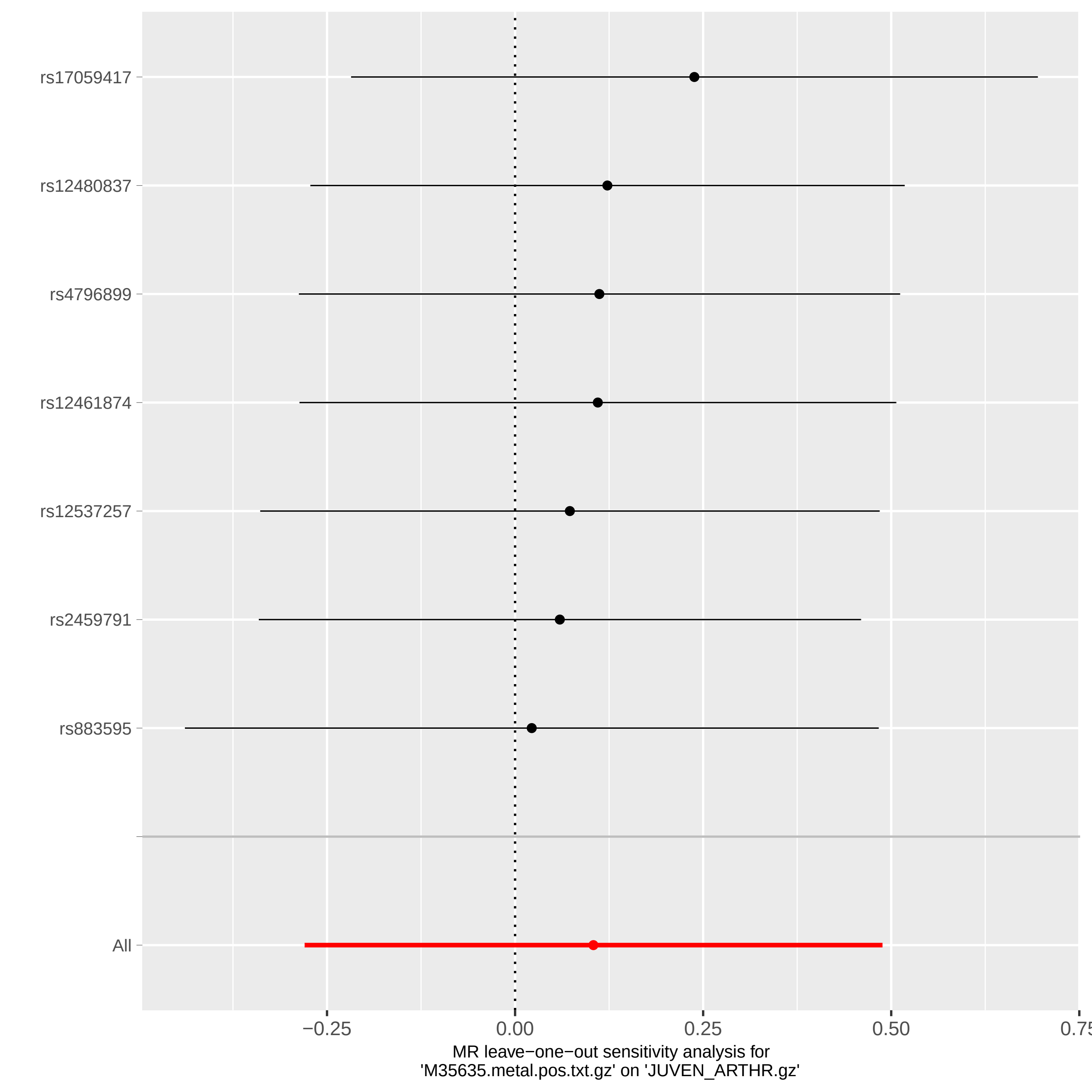
rs17059417
rs12480837
rs4796899
rs12461874
rs12537257
rs2459791
rs883595
All
−0.25
0.00
0.25
0.50
0.75
MR leave−one−out sensitivity analysis for
'M35635.metal.pos.txt.gz' on 'JUVEN_ARTHR.gz'

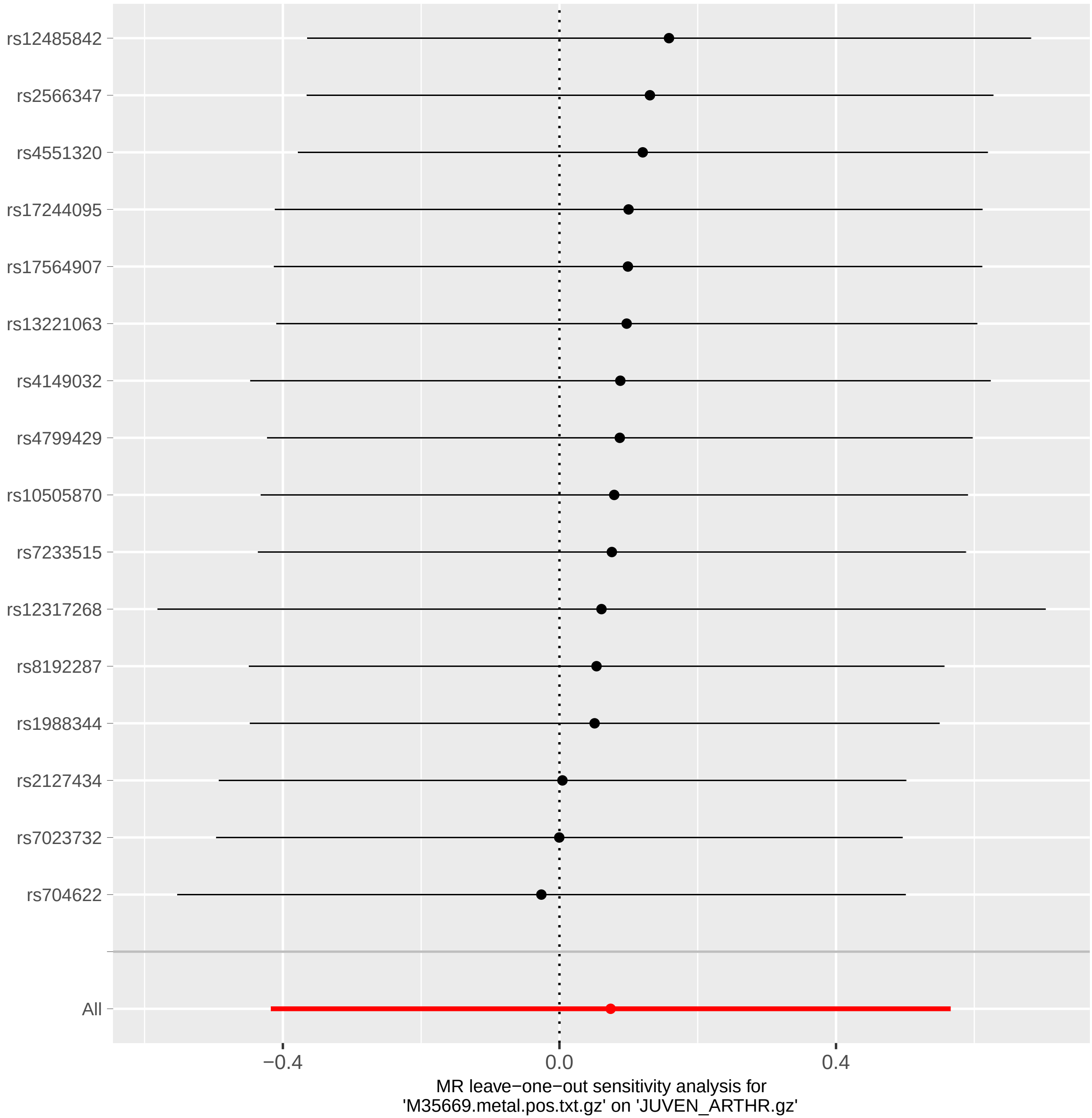
rs12485842
rs2566347
rs4551320
rs17244095
rs17564907
rs13221063
rs4149032
rs4799429
rs10505870
rs7233515
rs12317268
rs8192287
rs1988344
rs2127434
rs7023732
rs704622
All
−0.4
0.0
0.4
MR leave−one−out sensitivity analysis for
'M35669.metal.pos.txt.gz' on 'JUVEN_ARTHR.gz'

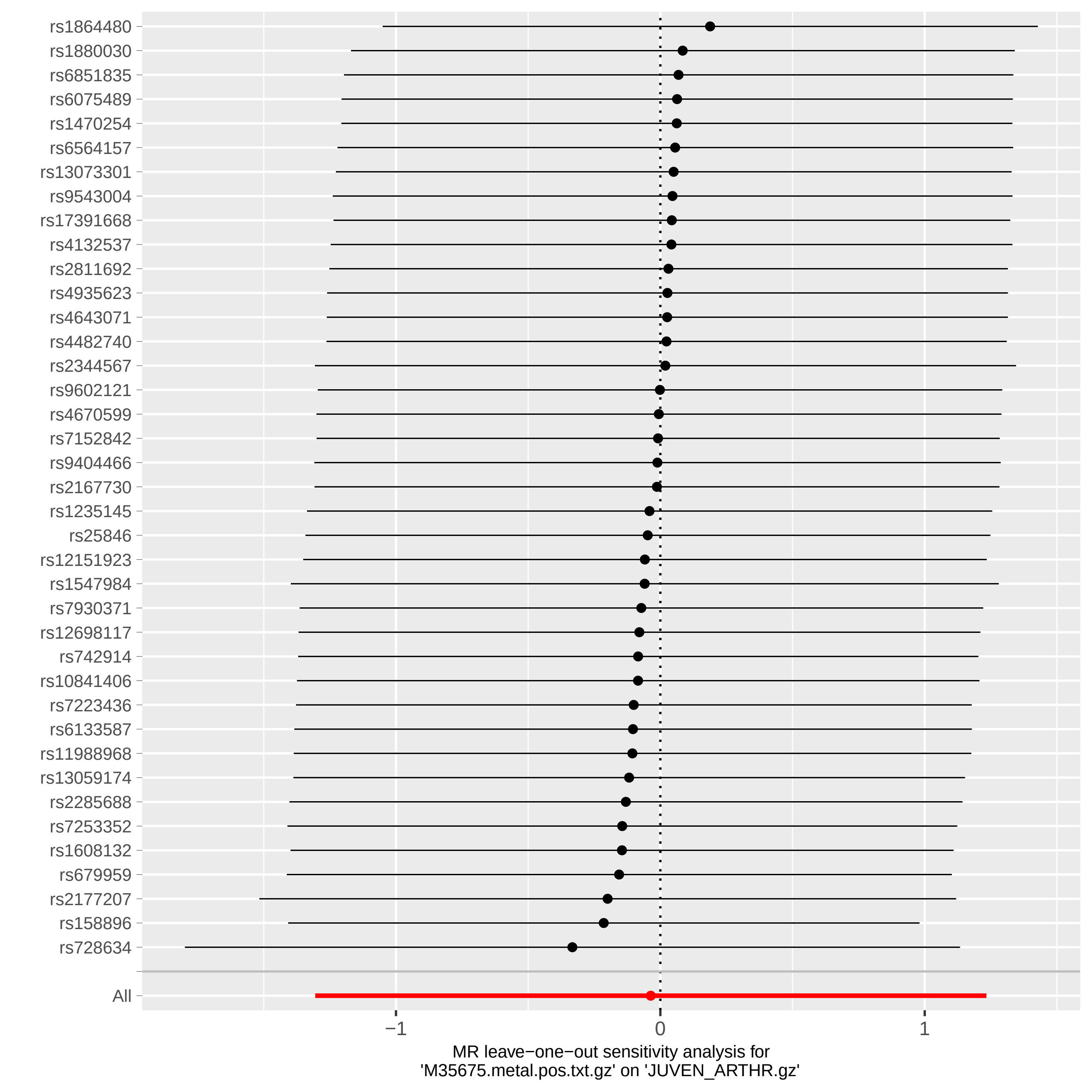

rs1864480
rs1880030
rs6851835
rs6075489
rs1470254
rs6564157
rs13073301
rs9543004
rs17391668
rs4132537
rs2811692
rs4935623
rs4643071
rs4482740
rs2344567
rs9602121
rs4670599
rs7152842
rs9404466
rs2167730
rs1235145
rs25846
rs12151923
rs1547984
rs7930371
rs12698117
rs742914
rs10841406
rs7223436
rs6133587
rs11988968
rs13059174
rs2285688
rs7253352
rs1608132
rs679959
rs2177207
rs158896
rs728634
All
−1
0
1
MR leave−one−out sensitivity analysis for
'M35675.metal.pos.txt.gz' on 'JUVEN_ARTHR.gz'

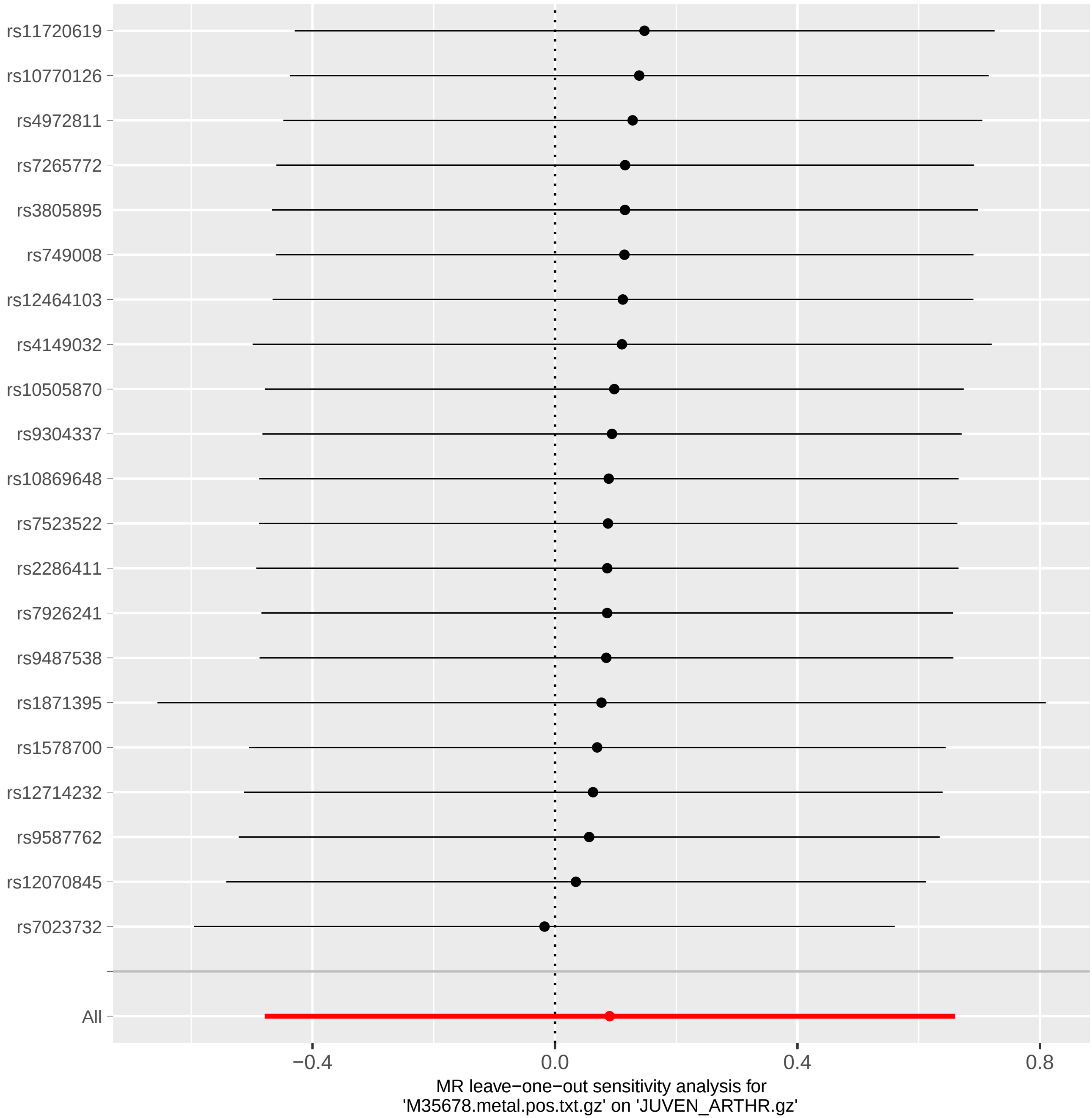
rs11720619
rs10770126
rs4972811
rs7265772
rs3805895
rs749008
rs12464103
rs4149032
rs10505870
rs9304337
rs10869648
rs7523522
rs2286411
rs7926241
rs9487538
rs1871395
rs1578700
rs12714232
rs9587762
rs12070845
rs7023732
All
−0.4
0.0
0.4
0.8
MR leave−one−out sensitivity analysis for
'M35678.metal.pos.txt.gz' on 'JUVEN_ARTHR.gz'

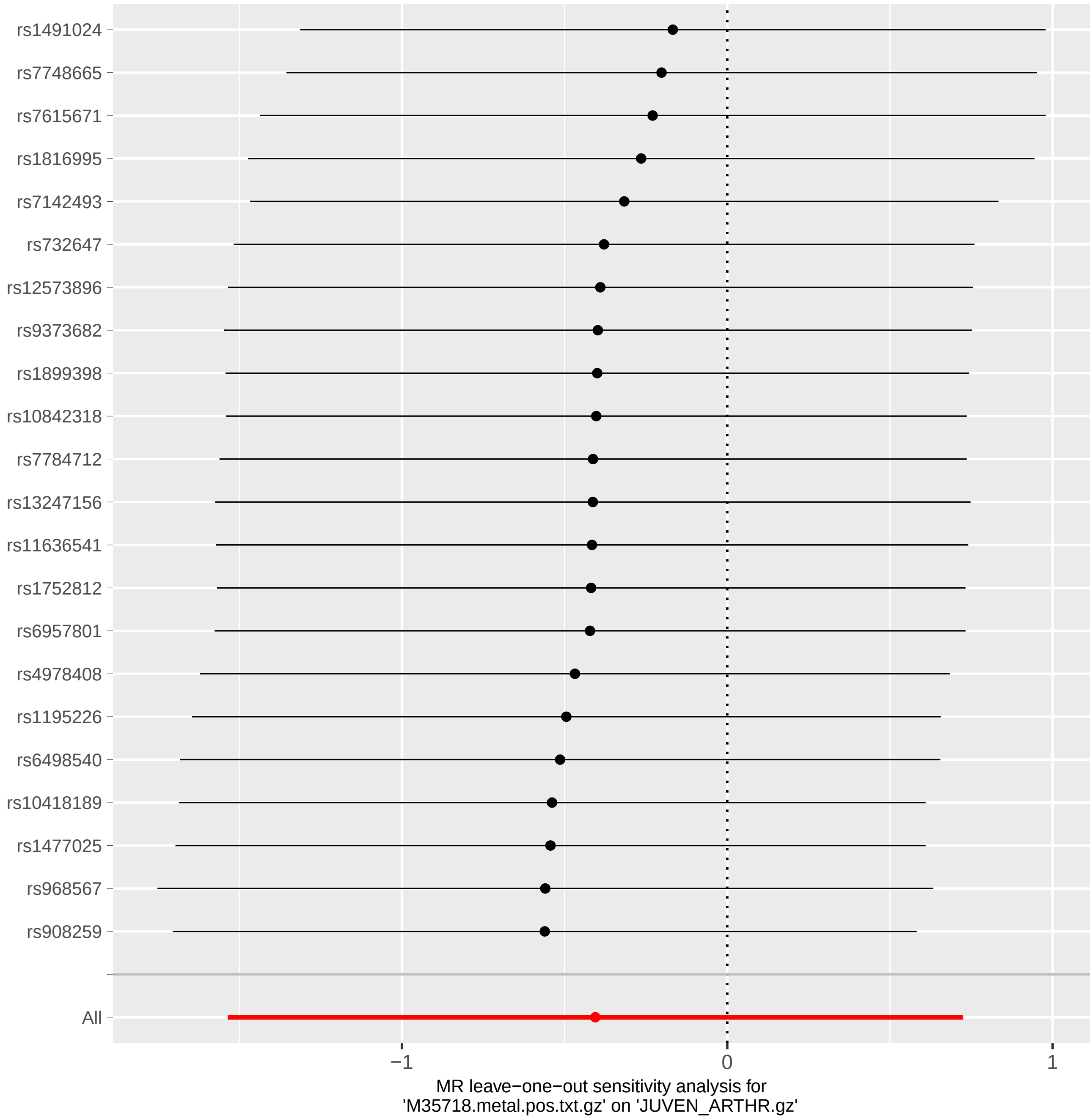
rs1491024
rs7748665
rs7615671
rs1816995
rs7142493
rs732647
rs12573896
rs9373682
rs1899398
rs10842318
rs7784712
rs13247156
rs11636541
rs1752812
rs6957801
rs4978408
rs1195226
rs6498540
rs10418189
rs1477025
rs968567
rs908259
All
−1
0
1
MR leave−one−out sensitivity analysis for
'M35718.metal.pos.txt.gz' on 'JUVEN_ARTHR.gz'

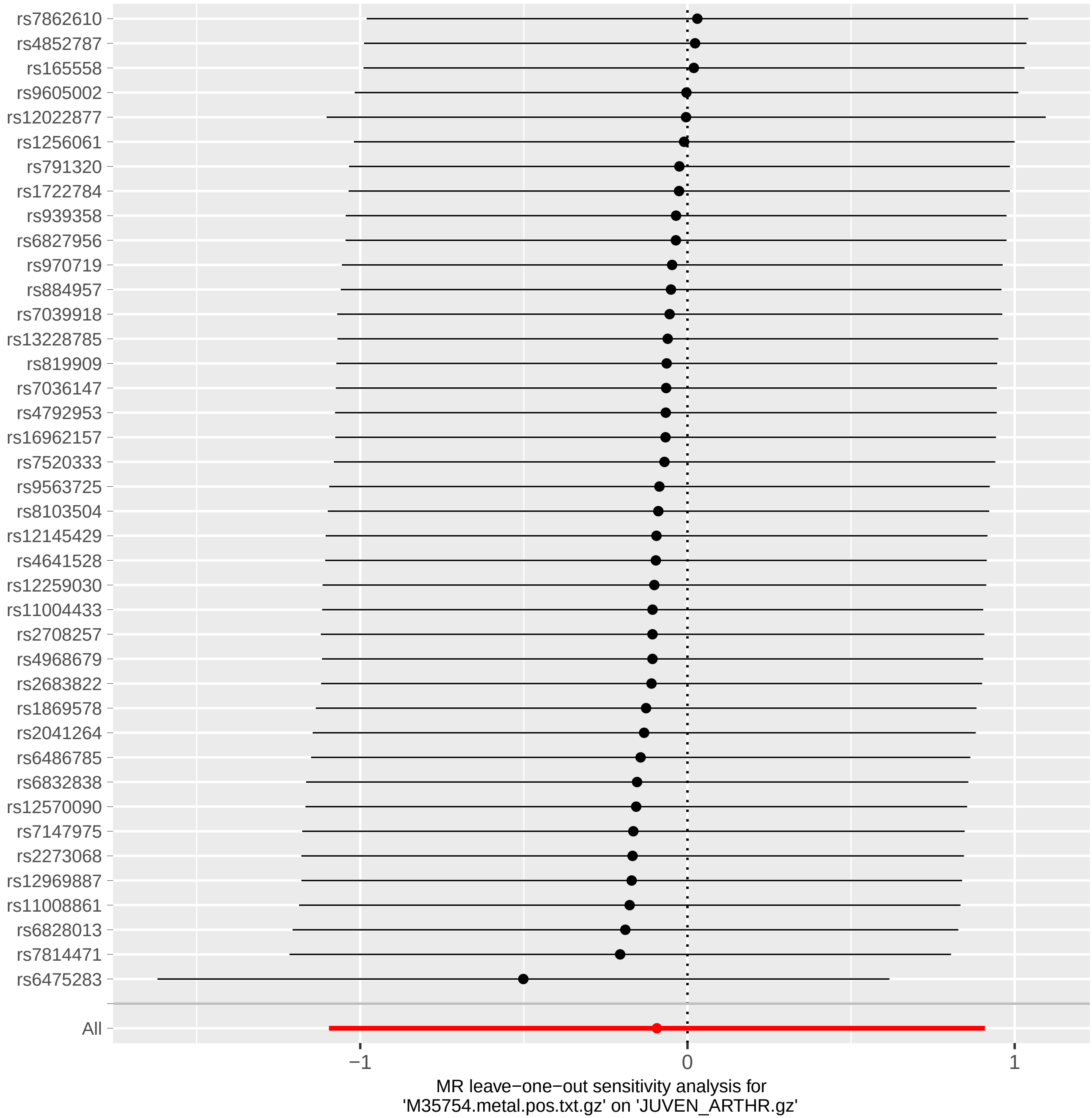
rs7862610
rs4852787
rs165558
rs9605002
rs12022877
rs1256061
rs791320
rs1722784
rs939358
rs6827956
rs970719
rs884957
rs7039918
rs13228785
rs819909
rs7036147
rs4792953
rs16962157
rs7520333
rs9563725
rs8103504
rs12145429
rs4641528
rs12259030
rs11004433
rs2708257
rs4968679
rs2683822
rs1869578
rs2041264
rs6486785
rs6832838
rs12570090
rs7147975
rs2273068
rs12969887
rs11008861
rs6828013
rs7814471
rs6475283
All
−1
0
1
MR leave−one−out sensitivity analysis for
'M35754.metal.pos.txt.gz' on 'JUVEN_ARTHR.gz'

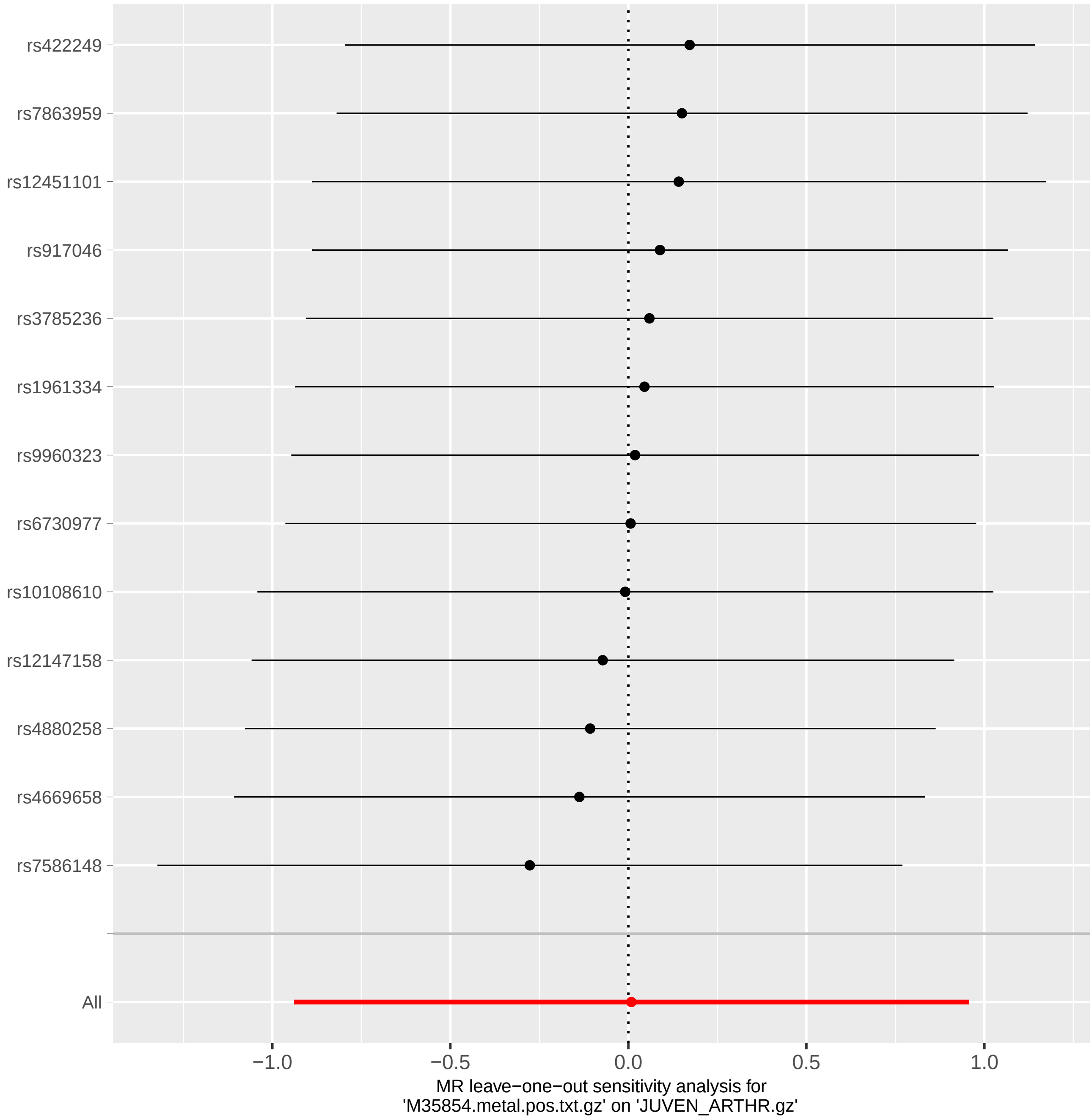
rs422249
rs7863959
rs12451101
rs917046
rs3785236
rs1961334
rs9960323
rs6730977
rs10108610
rs12147158
rs4880258
rs4669658
rs7586148
All
−1.0
−0.5
0.0
0.5
1.0
MR leave−one−out sensitivity analysis for
'M35854.metal.pos.txt.gz' on 'JUVEN_ARTHR.gz'

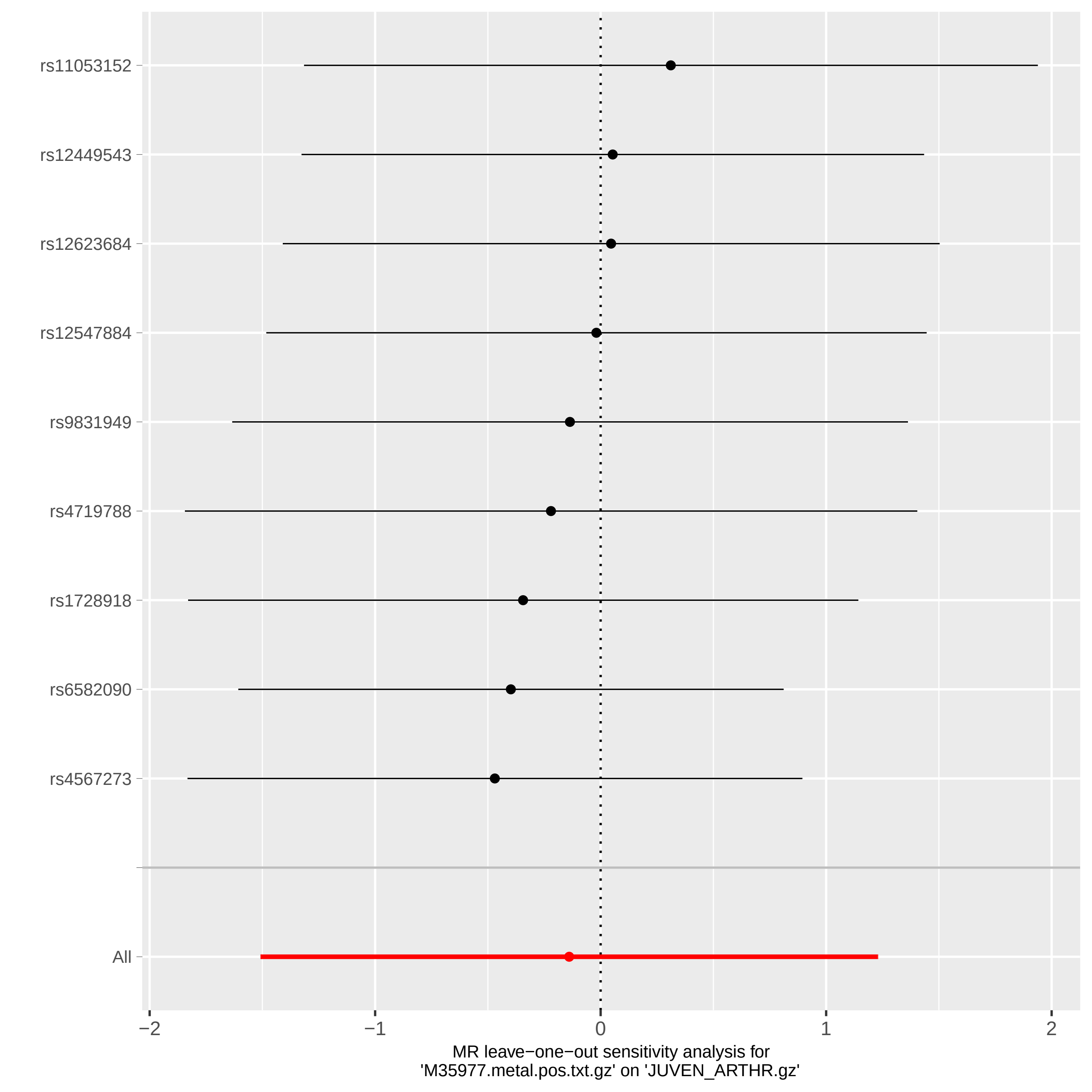
rs11053152
rs12449543
rs12623684
rs12547884
rs9831949
rs4719788
rs1728918
rs6582090
rs4567273
All
−2
−1
0
1
2
MR leave−one−out sensitivity analysis for
'M35977.metal.pos.txt.gz' on 'JUVEN_ARTHR.gz'

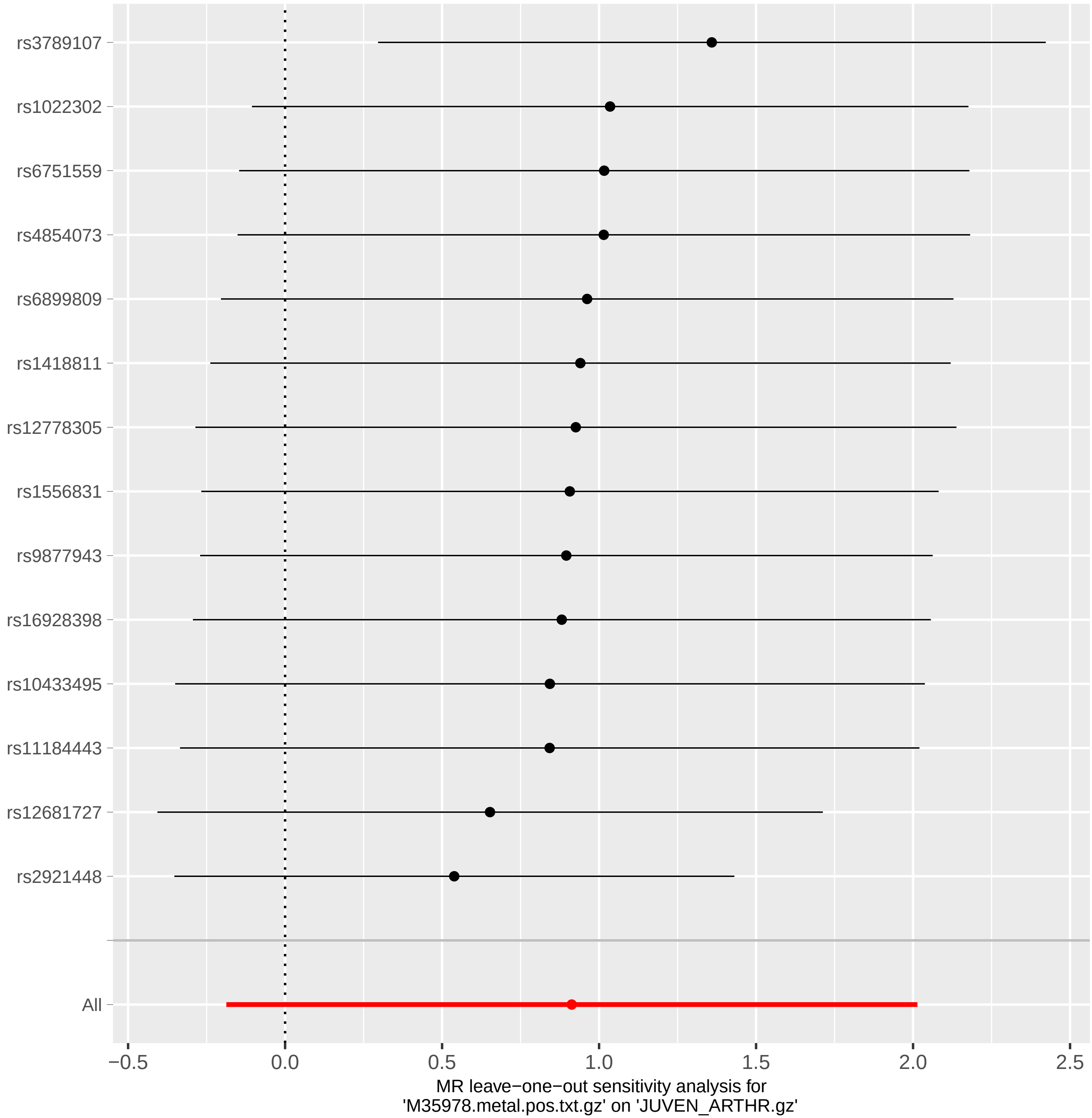

rs3789107
rs1022302
rs6751559
rs4854073
rs6899809
rs1418811
rs12778305
rs1556831
rs9877943
rs16928398
rs10433495
rs11184443
rs12681727
rs2921448
All
−0.5
0.0
0.5
1.0
1.5
2.0
2.5
MR leave−one−out sensitivity analysis for
'M35978.metal.pos.txt.gz' on 'JUVEN_ARTHR.gz'

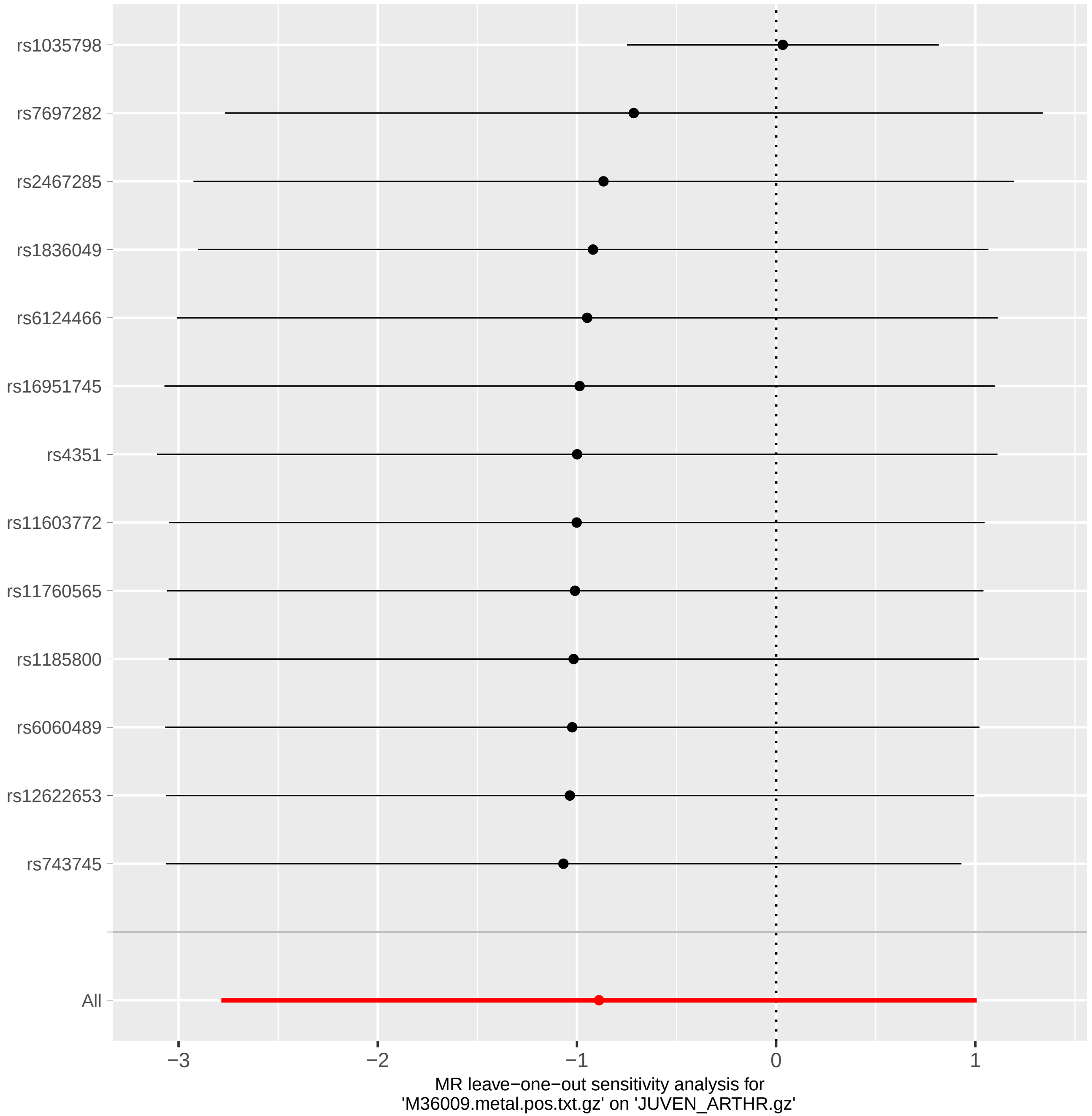
rs1035798
rs7697282
rs2467285
rs1836049
rs6124466
rs16951745
rs4351
rs11603772
rs11760565
rs1185800
rs6060489
rs12622653
rs743745
All
−3
−2
−1
0
1
MR leave−one−out sensitivity analysis for
'M36009.metal.pos.txt.gz' on 'JUVEN_ARTHR.gz'

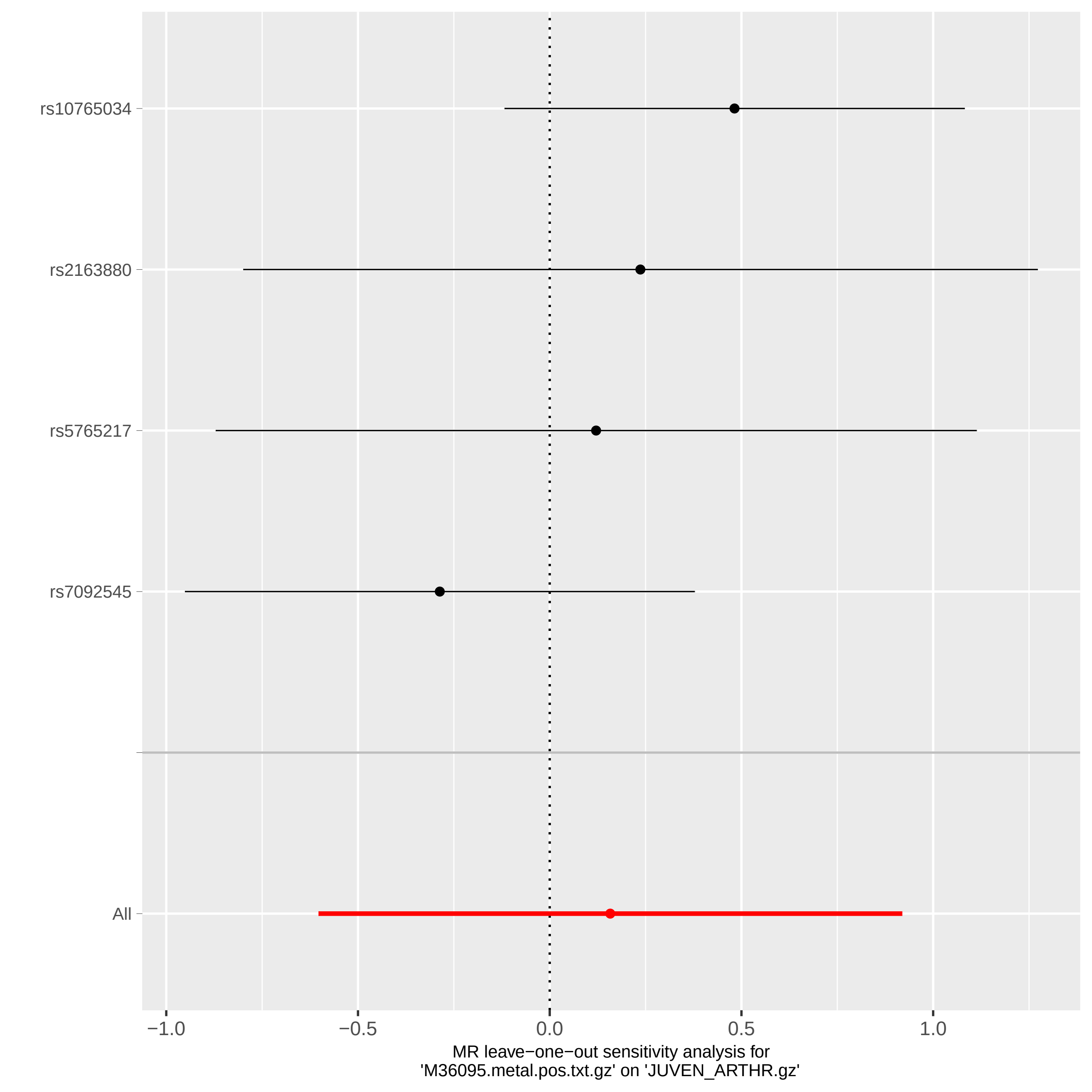
rs10765034
rs2163880
rs5765217
rs7092545
All
−1.0
−0.5
0.0
0.5
1.0
MR leave−one−out sensitivity analysis for
'M36095.metal.pos.txt.gz' on 'JUVEN_ARTHR.gz'

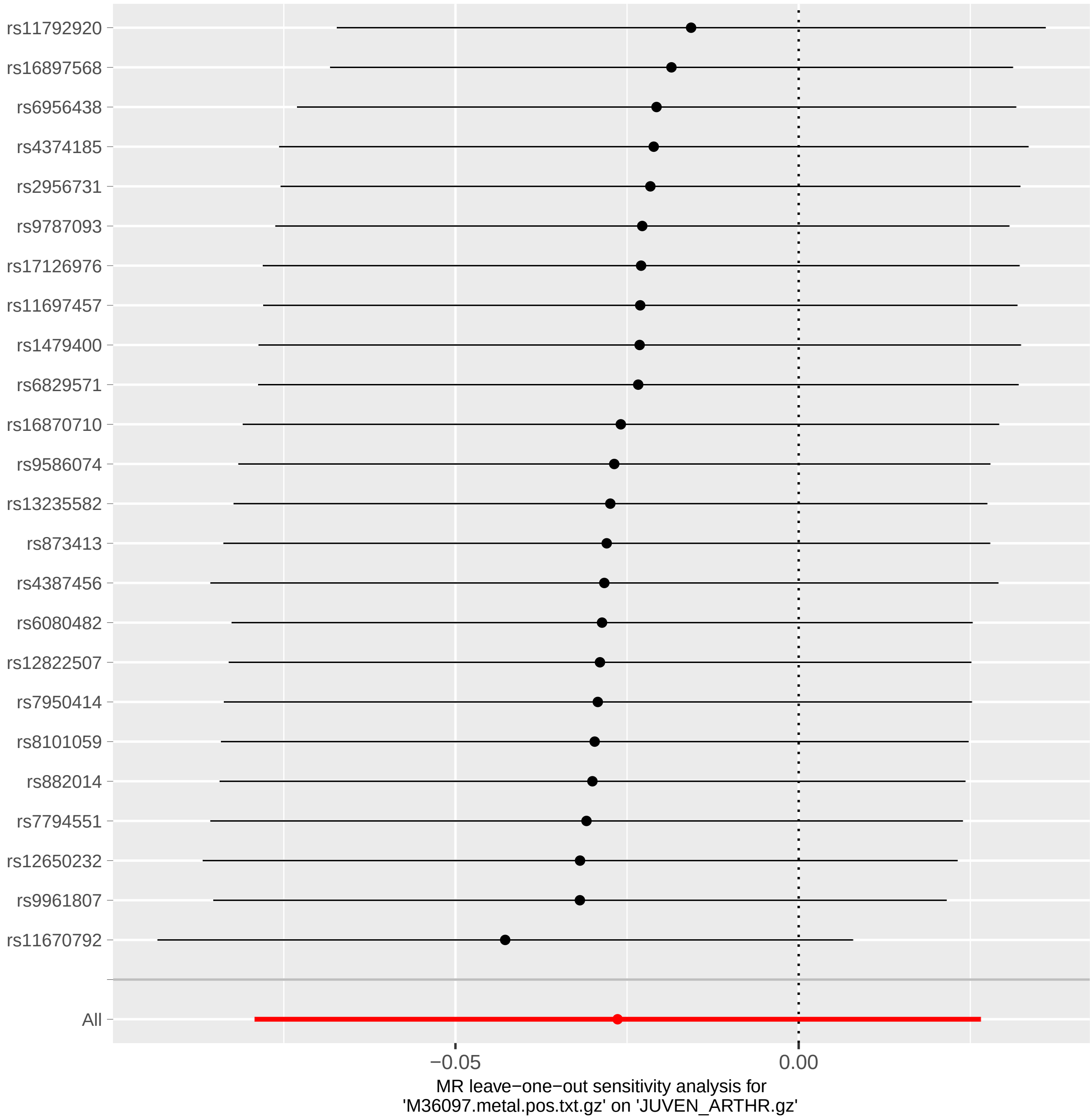
rs11792920
rs16897568
rs6956438
rs4374185
rs2956731
rs9787093
rs17126976
rs11697457
rs1479400
rs6829571
rs16870710
rs9586074
rs13235582
rs873413
rs4387456
rs6080482
rs12822507
rs7950414
rs8101059
rs882014
rs7794551
rs12650232
rs9961807
rs11670792
All
−0.05
0.00
MR leave−one−out sensitivity analysis for
'M36097.metal.pos.txt.gz' on 'JUVEN_ARTHR.gz'

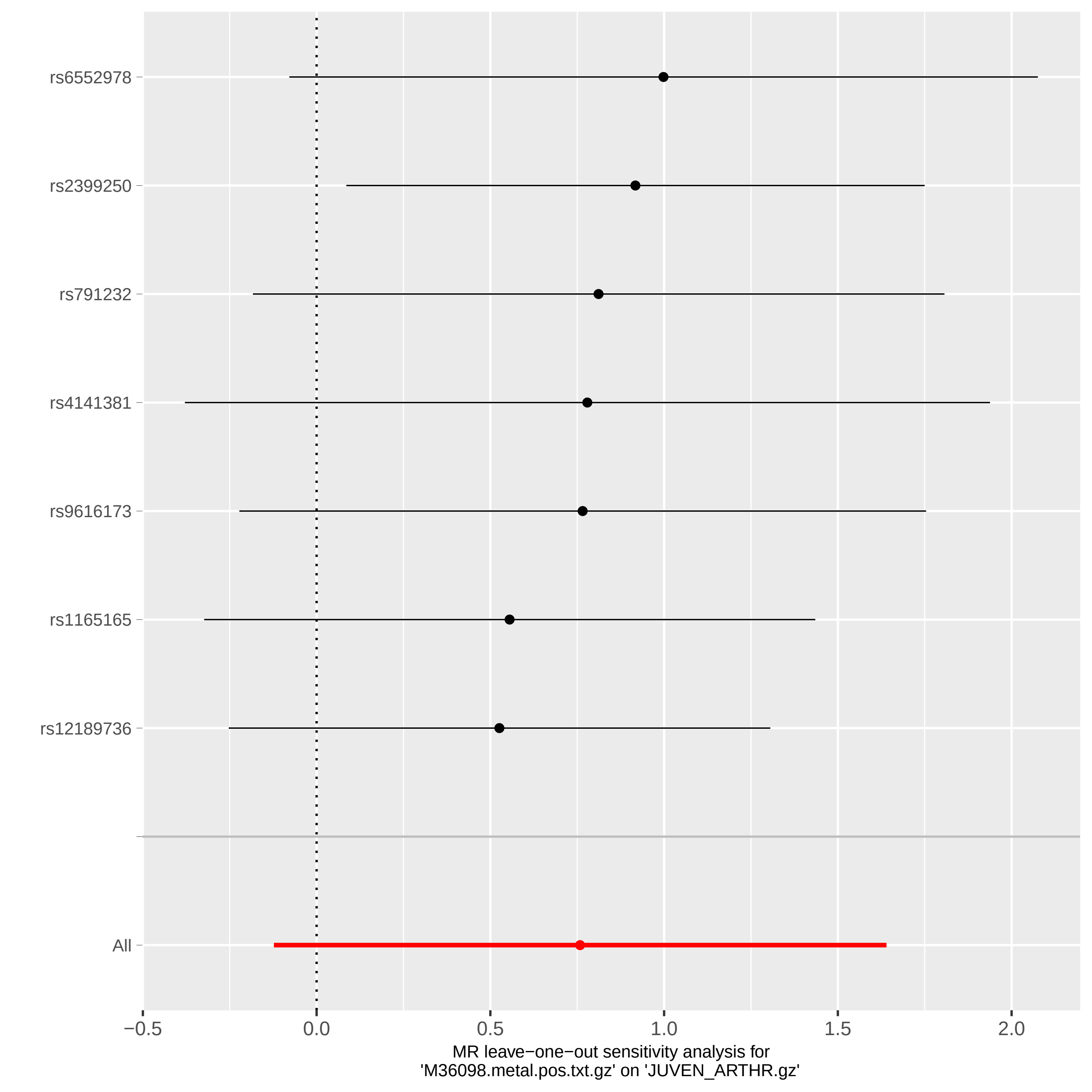
rs6552978
rs2399250
rs791232
rs4141381
rs9616173
rs1165165
rs12189736
All
−0.5
0.0
0.5
1.0
1.5
2.0
MR leave−one−out sensitivity analysis for
'M36098.metal.pos.txt.gz' on 'JUVEN_ARTHR.gz'

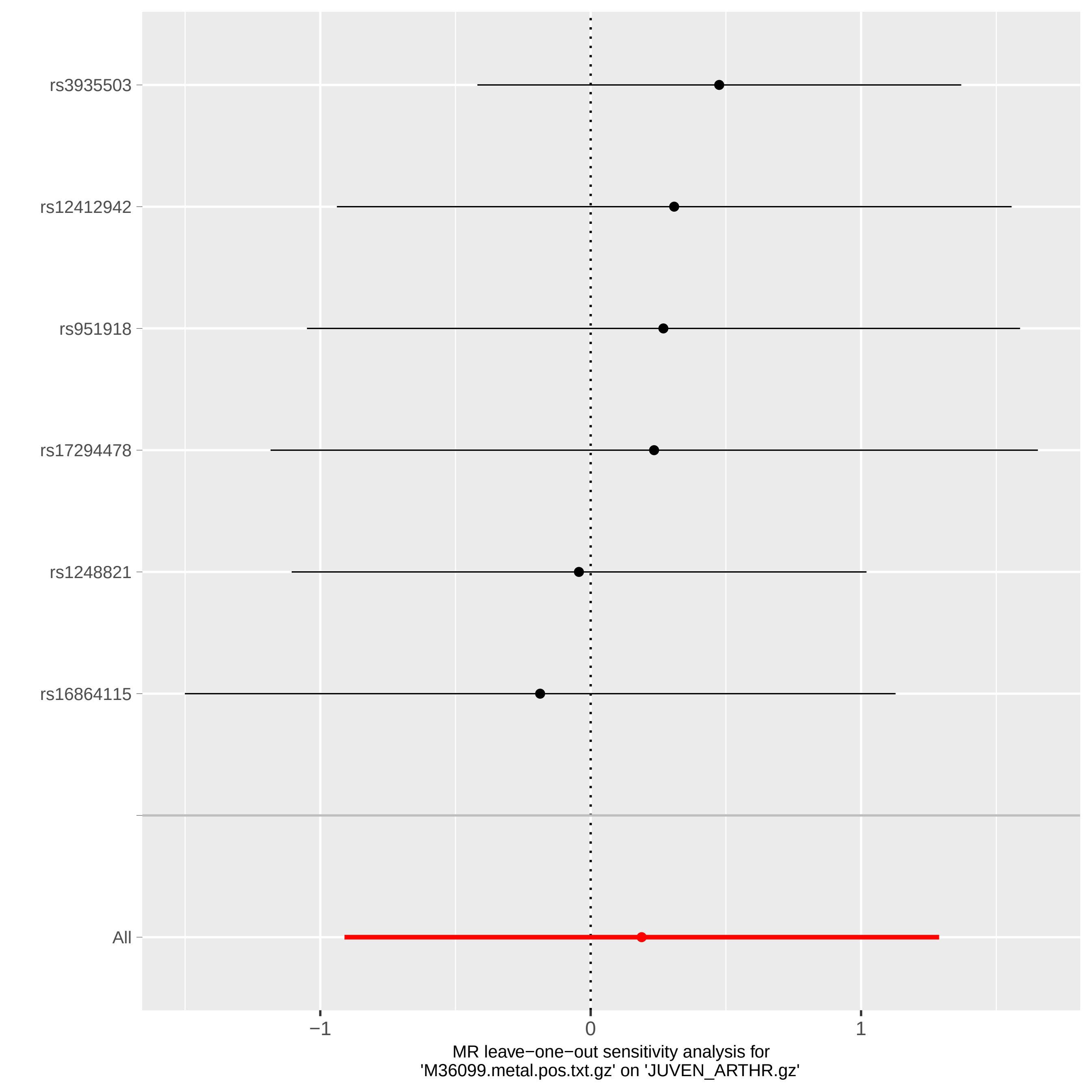

rs3935503
rs12412942
rs951918
rs17294478
rs1248821
rs16864115
All
−1
0
1
MR leave−one−out sensitivity analysis for
'M36099.metal.pos.txt.gz' on 'JUVEN_ARTHR.gz'

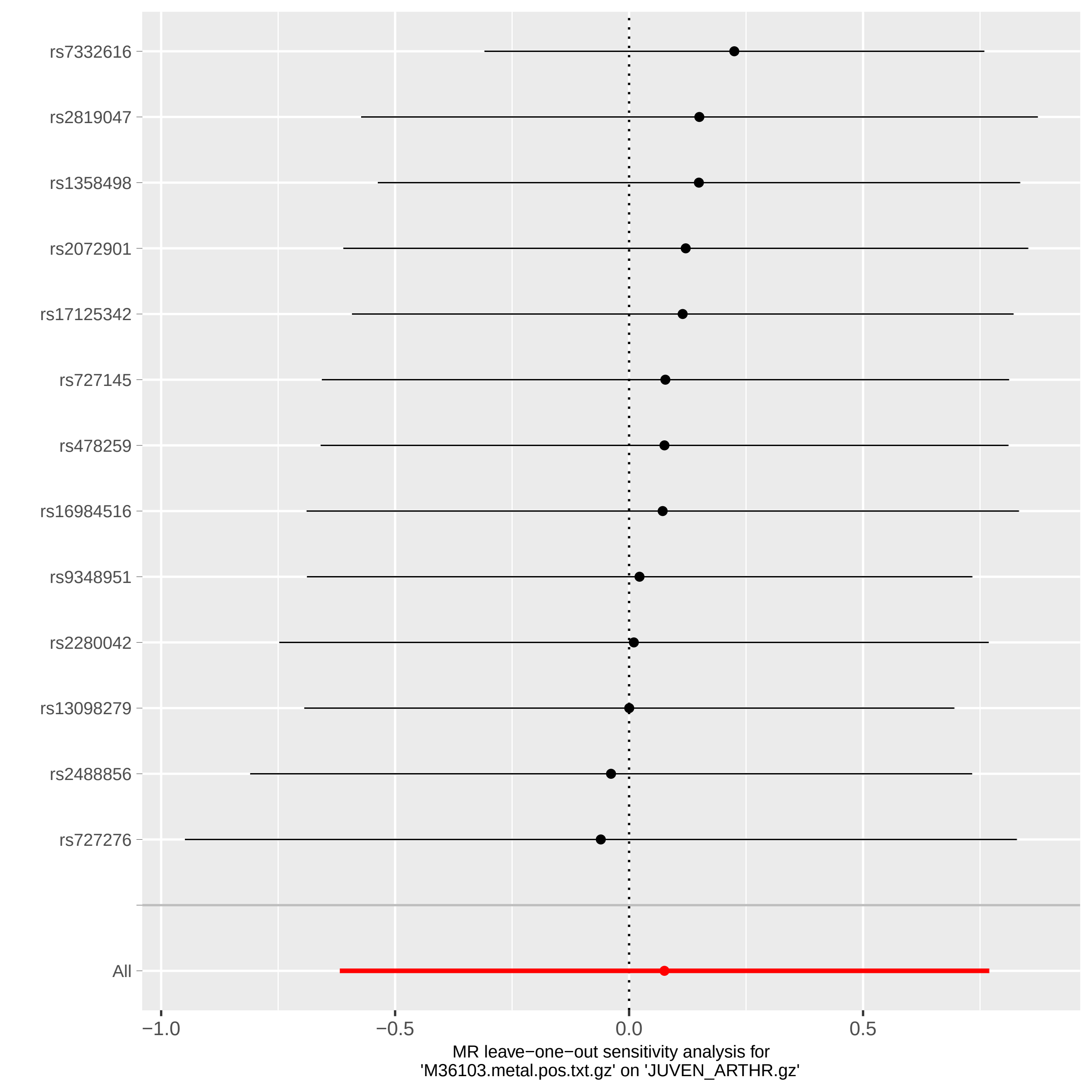
rs7332616
rs2819047
rs1358498
rs2072901
rs17125342
rs727145
rs478259
rs16984516
rs9348951
rs2280042
rs13098279
rs2488856
rs727276
All
−1.0
−0.5
0.0
0.5
MR leave−one−out sensitivity analysis for
'M36103.metal.pos.txt.gz' on 'JUVEN_ARTHR.gz'

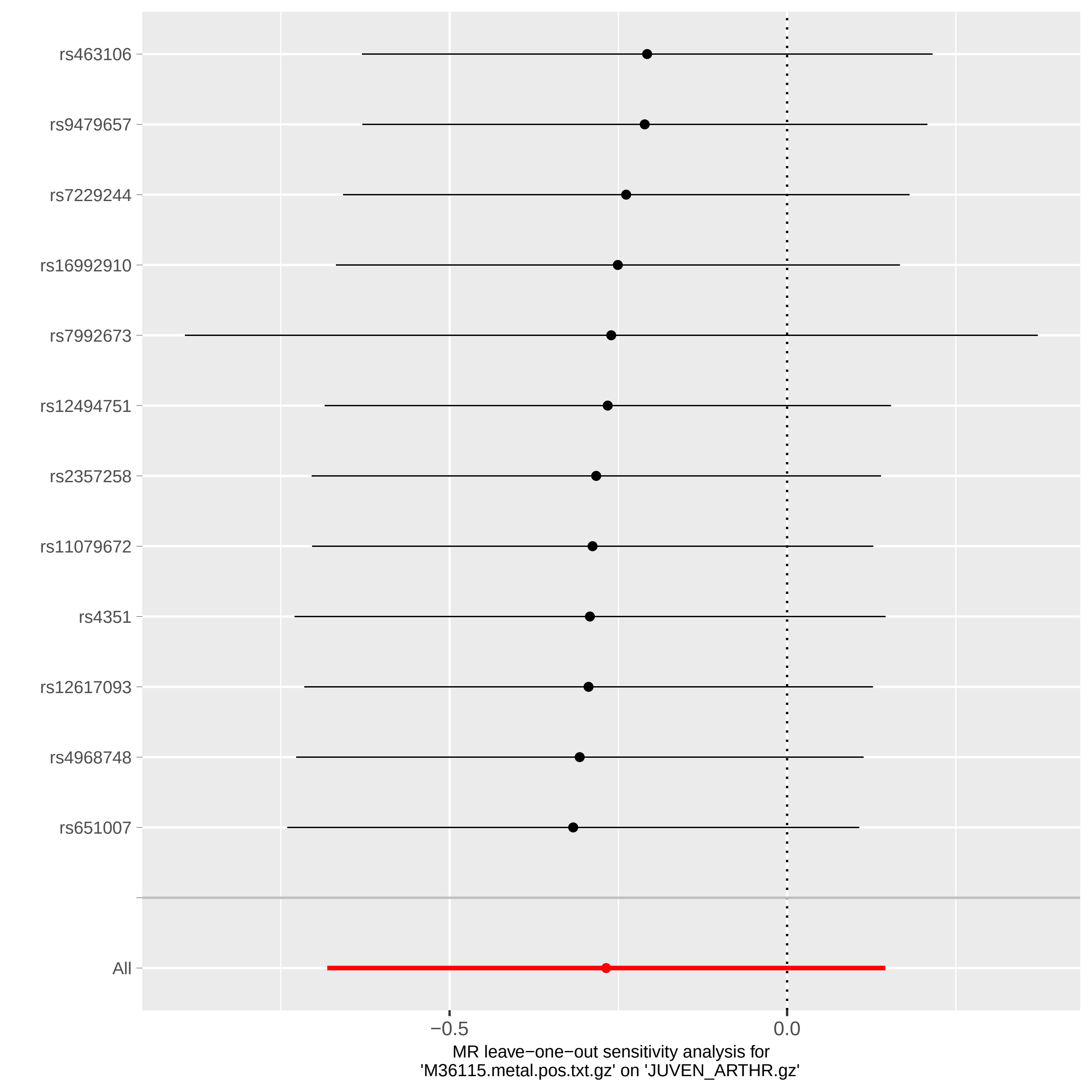
rs463106
rs9479657
rs7229244
rs16992910
rs7992673
rs12494751
rs2357258
rs11079672
rs4351
rs12617093
rs4968748
rs651007
All
−0.5
0.0
MR leave−one−out sensitivity analysis for
'M36115.metal.pos.txt.gz' on 'JUVEN_ARTHR.gz'

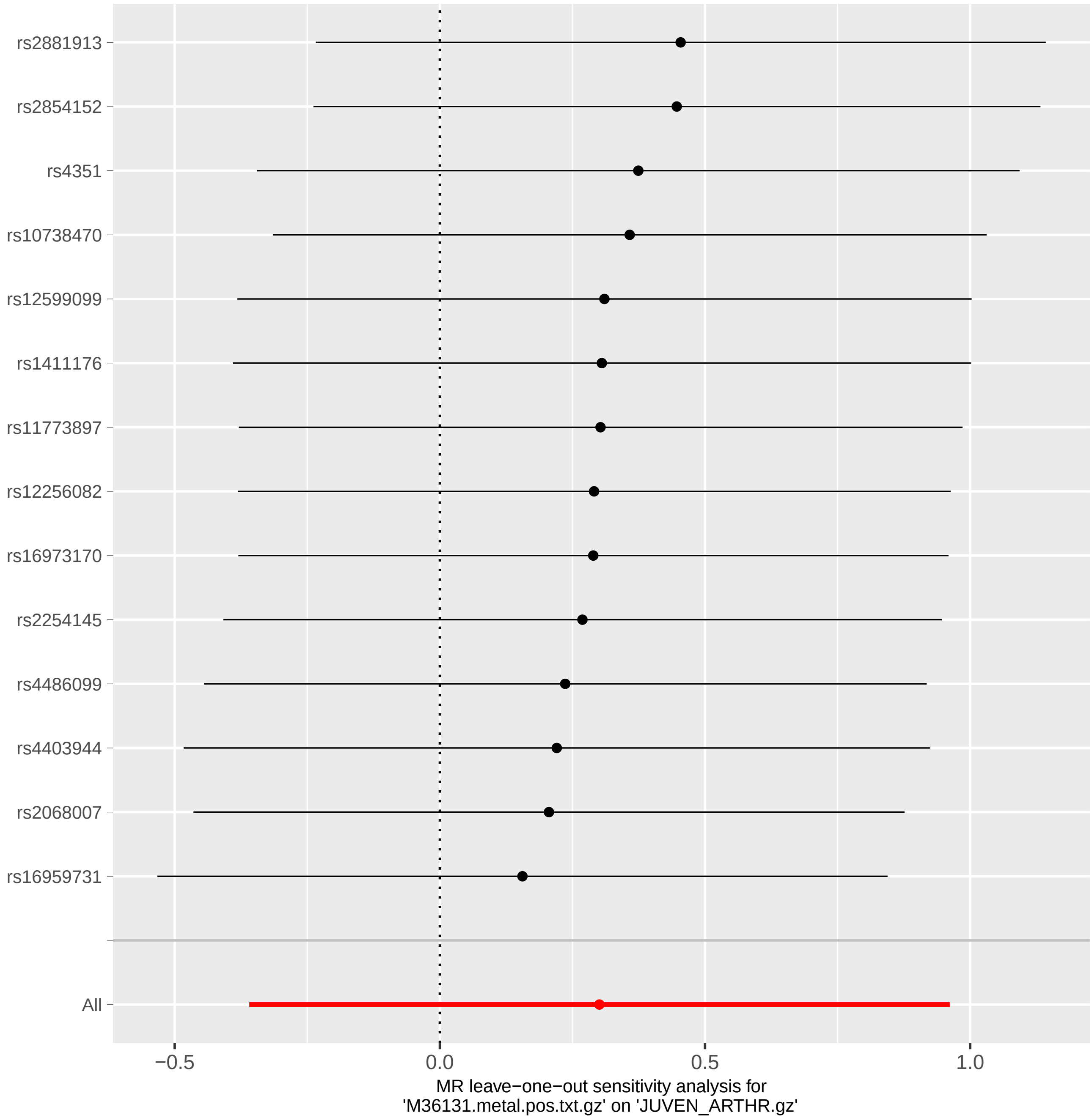

rs2881913
rs2854152
rs4351
rs10738470
rs12599099
rs1411176
rs11773897
rs12256082
rs16973170
rs2254145
rs4486099
rs4403944
rs2068007
rs16959731
All
−0.5
0.0
0.5
1.0
MR leave−one−out sensitivity analysis for
'M36131.metal.pos.txt.gz' on 'JUVEN_ARTHR.gz'

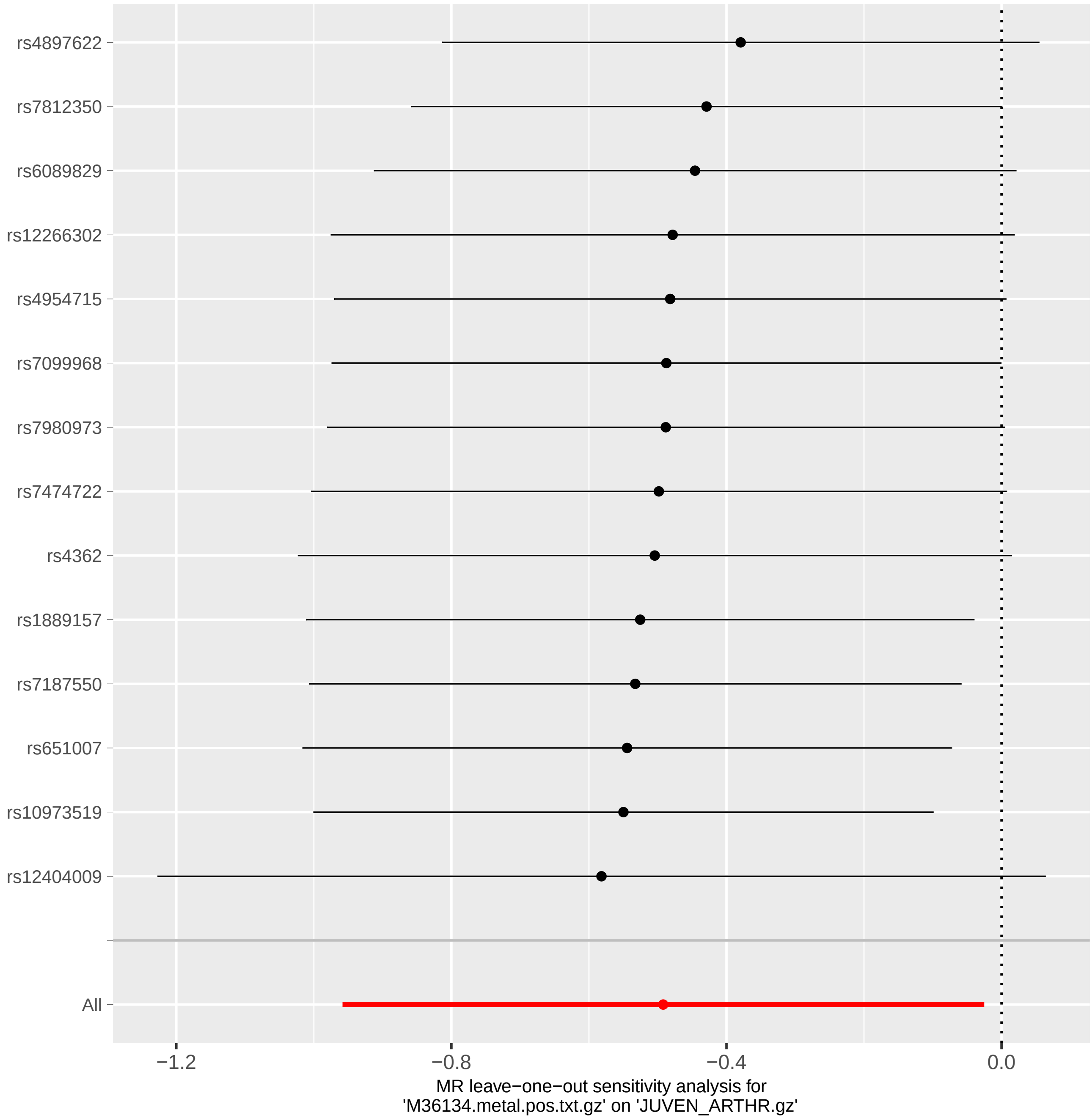
rs4897622
rs7812350
rs6089829
rs12266302
rs4954715
rs7099968
rs7980973
rs7474722
rs4362
rs1889157
rs7187550
rs651007
rs10973519
rs12404009
All
−1.2
−0.8
−0.4
0.0
MR leave−one−out sensitivity analysis for
'M36134.metal.pos.txt.gz' on 'JUVEN_ARTHR.gz'

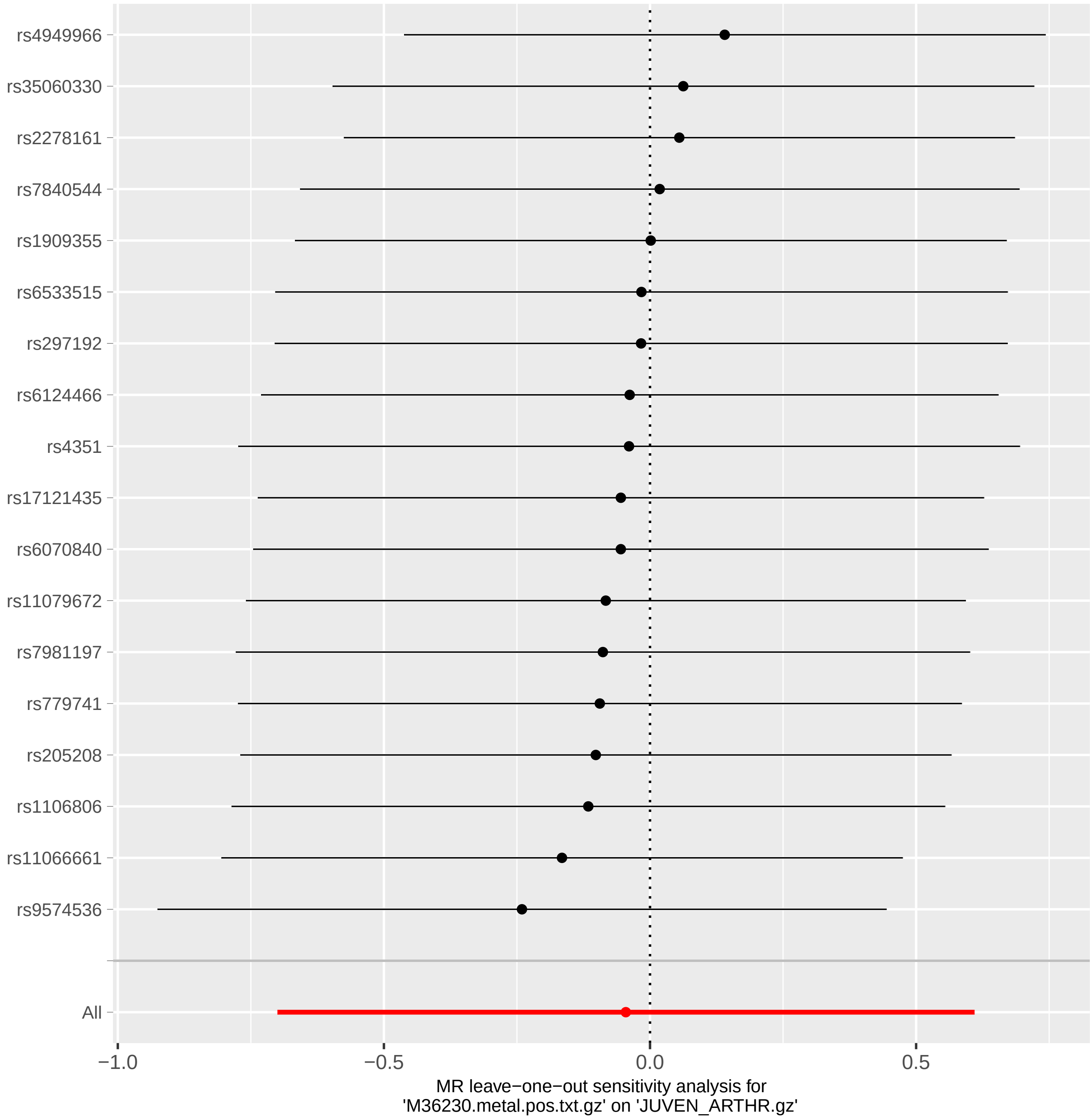

rs4949966
rs35060330
rs2278161
rs7840544
rs1909355
rs6533515
rs297192
rs6124466
rs4351
rs17121435
rs6070840
rs11079672
rs7981197
rs779741
rs205208
rs1106806
rs11066661
rs9574536
All
−1.0
−0.5
0.0
0.5
MR leave−one−out sensitivity analysis for
'M36230.metal.pos.txt.gz' on 'JUVEN_ARTHR.gz'

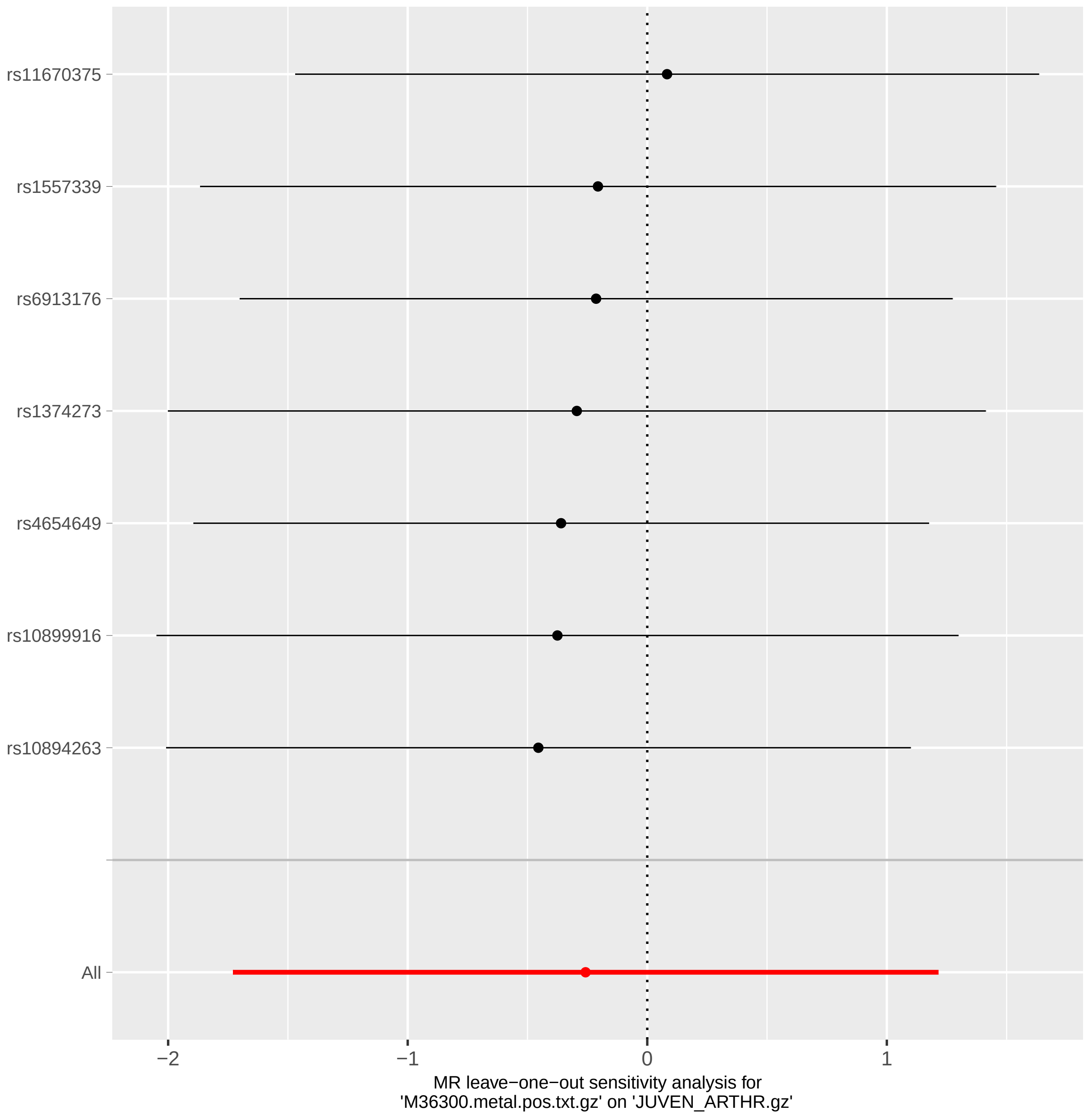

rs11670375
rs1557339
rs6913176
rs1374273
rs4654649
rs10899916
rs10894263
All
−2
−1
0
1
MR leave−one−out sensitivity analysis for
'M36300.metal.pos.txt.gz' on 'JUVEN_ARTHR.gz'

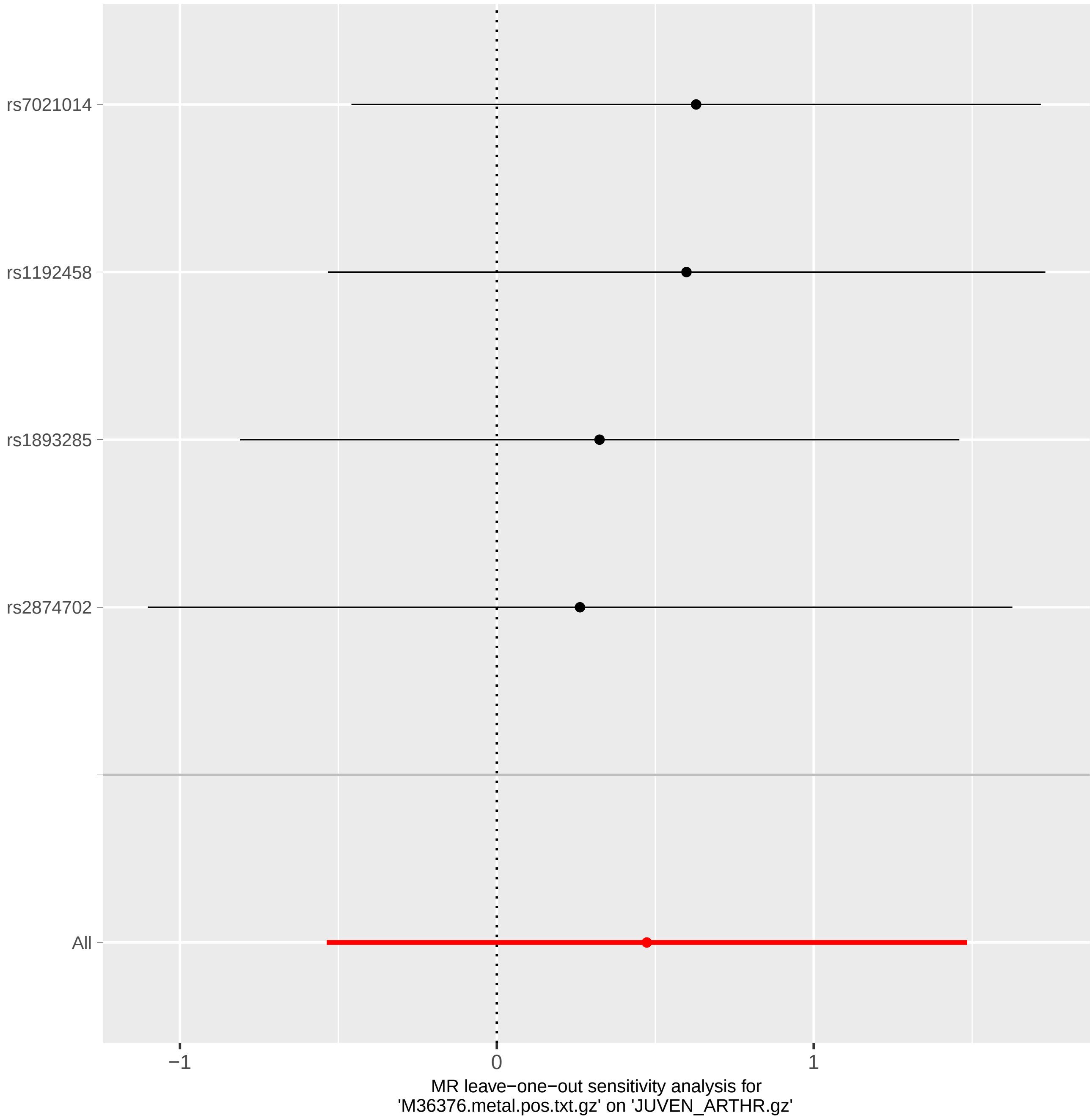
rs7021014
rs1192458
rs1893285
rs2874702
All
−1
0
1
MR leave−one−out sensitivity analysis for
'M36376.metal.pos.txt.gz' on 'JUVEN_ARTHR.gz'

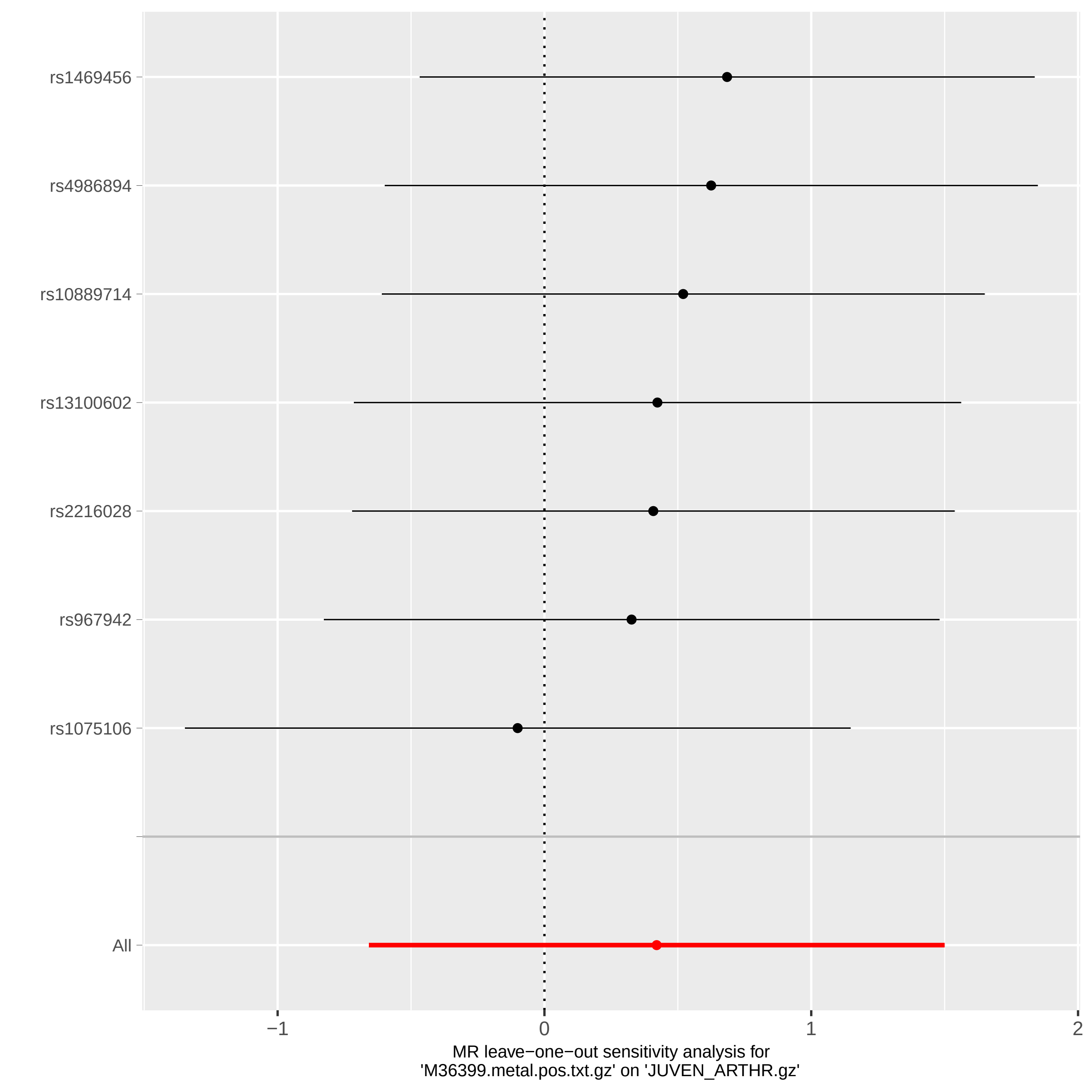
rs1469456
rs4986894
rs10889714
rs13100602
rs2216028
rs967942
rs1075106
All
−1
0
1
2
MR leave−one−out sensitivity analysis for
'M36399.metal.pos.txt.gz' on 'JUVEN_ARTHR.gz'

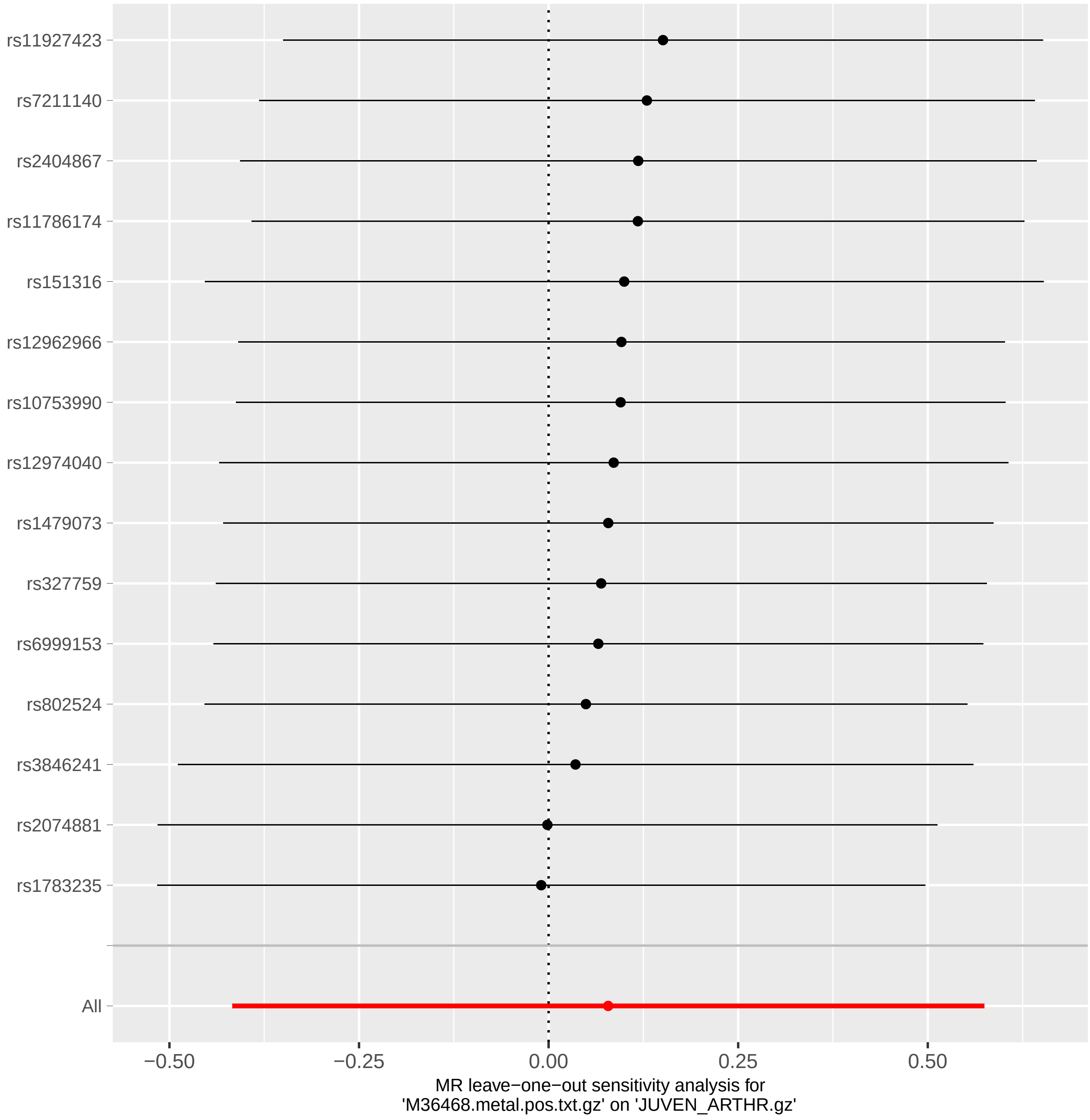

rs11927423
rs7211140
rs2404867
rs11786174
rs151316
rs12962966
rs10753990
rs12974040
rs1479073
rs327759
rs6999153
rs802524
rs3846241
rs2074881
rs1783235
All
−0.50
−0.25
0.00
0.25
0.50
MR leave−one−out sensitivity analysis for
'M36468.metal.pos.txt.gz' on 'JUVEN_ARTHR.gz'

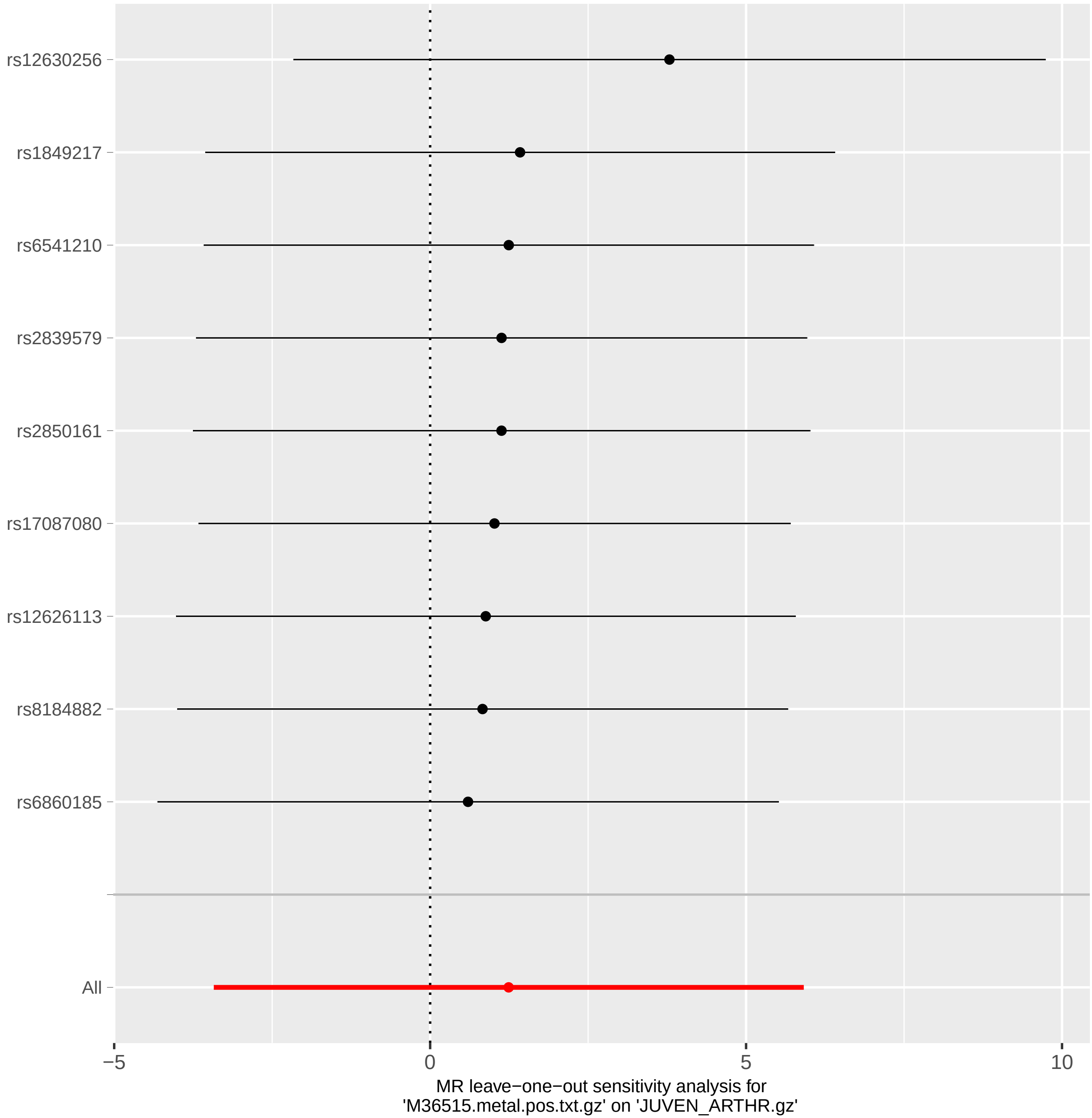
rs12630256
rs1849217
rs6541210
rs2839579
rs2850161
rs17087080
rs12626113
rs8184882
rs6860185
All
−5
0
5
10
MR leave−one−out sensitivity analysis for
'M36515.metal.pos.txt.gz' on 'JUVEN_ARTHR.gz'

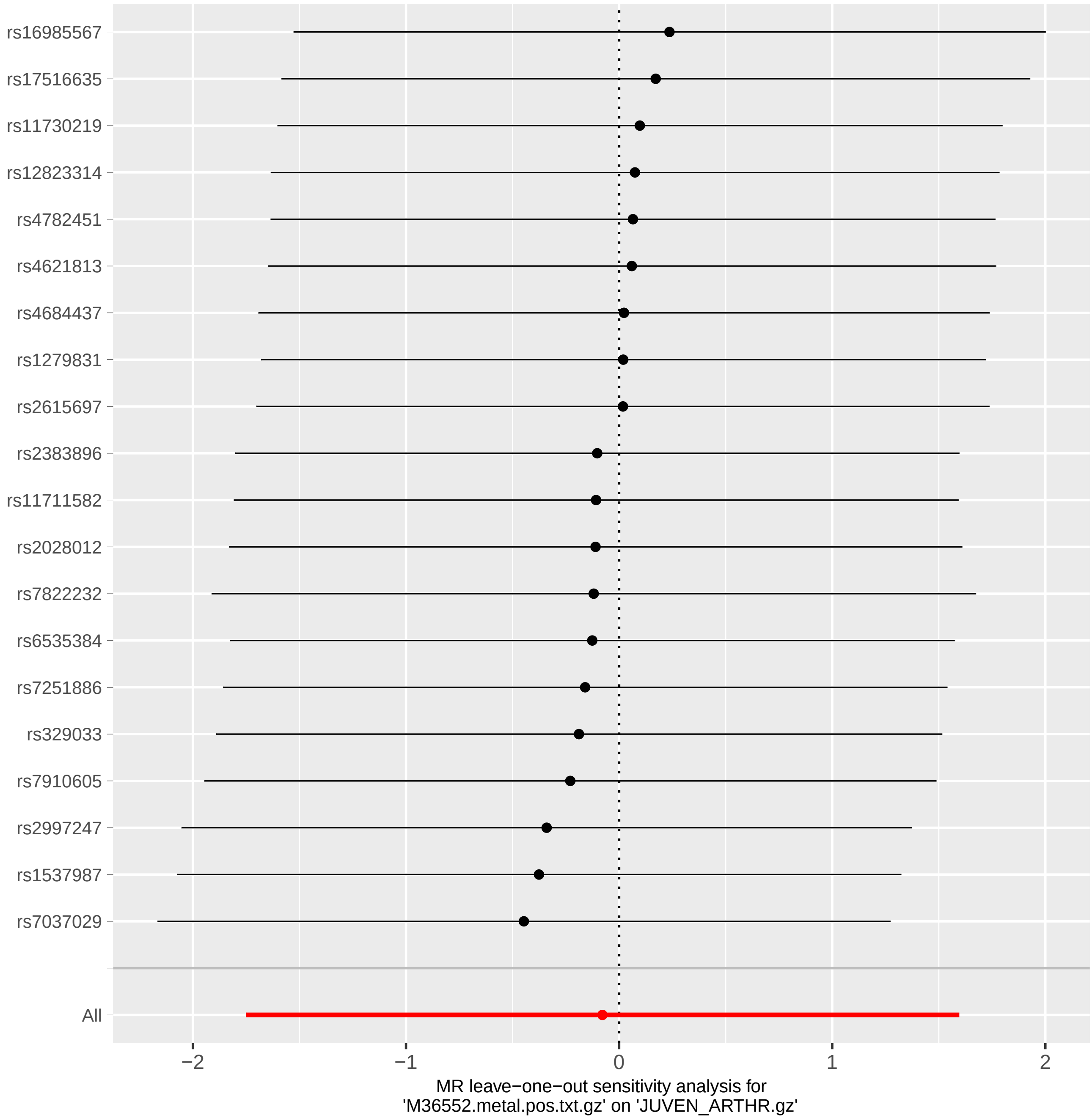

rs16985567
rs17516635
rs11730219
rs12823314
rs4782451
rs4621813
rs4684437
rs1279831
rs2615697
rs2383896
rs11711582
rs2028012
rs7822232
rs6535384
rs7251886
rs329033
rs7910605
rs2997247
rs1537987
rs7037029
All
−2
−1
0
1
2
MR leave−one−out sensitivity analysis for
'M36552.metal.pos.txt.gz' on 'JUVEN_ARTHR.gz'

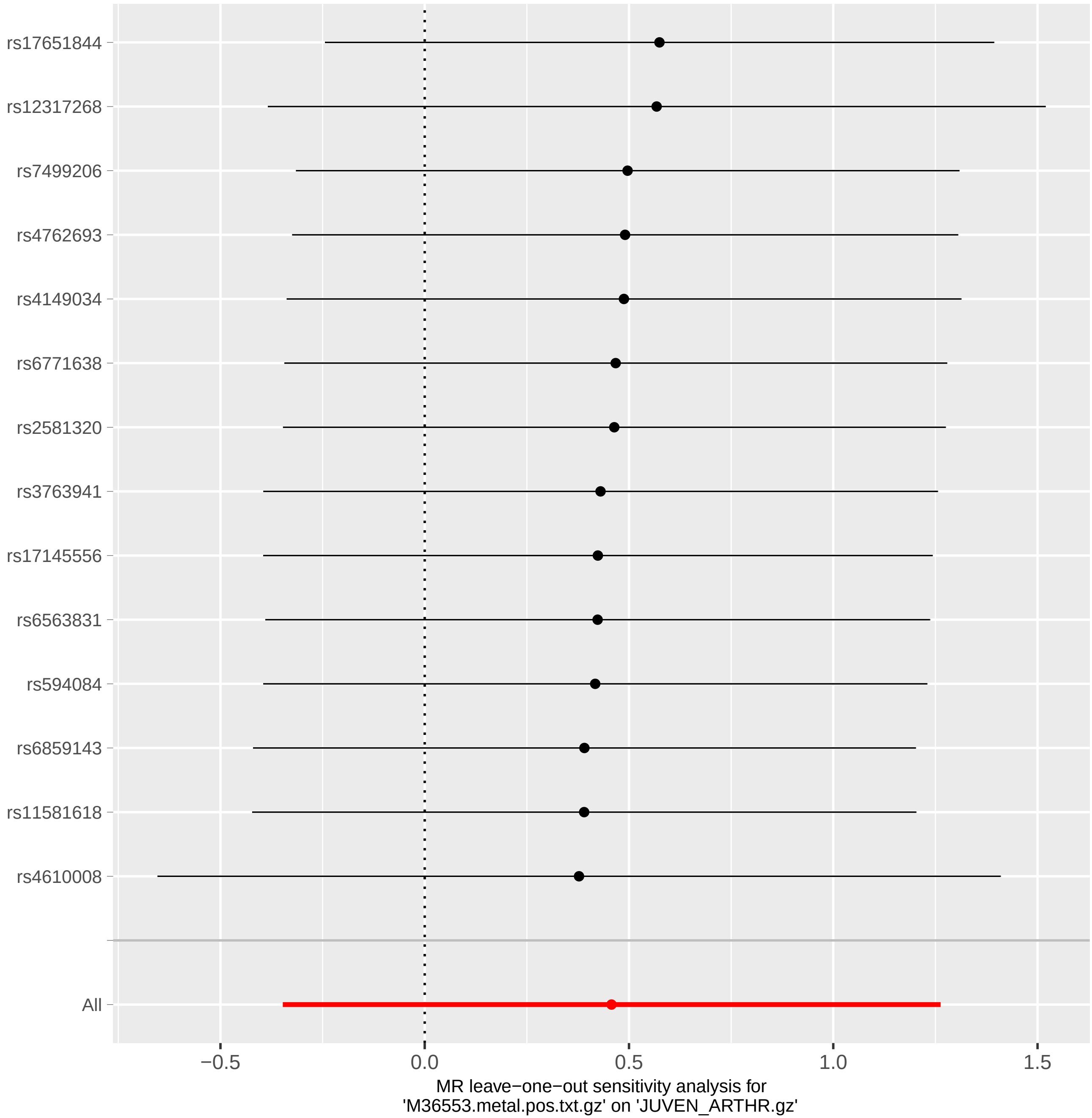
rs17651844
rs12317268
rs7499206
rs4762693
rs4149034
rs6771638
rs2581320
rs3763941
rs17145556
rs6563831
rs594084
rs6859143
rs11581618
rs4610008
All
−0.5
0.0
0.5
1.0
1.5
MR leave−one−out sensitivity analysis for
'M36553.metal.pos.txt.gz' on 'JUVEN_ARTHR.gz'

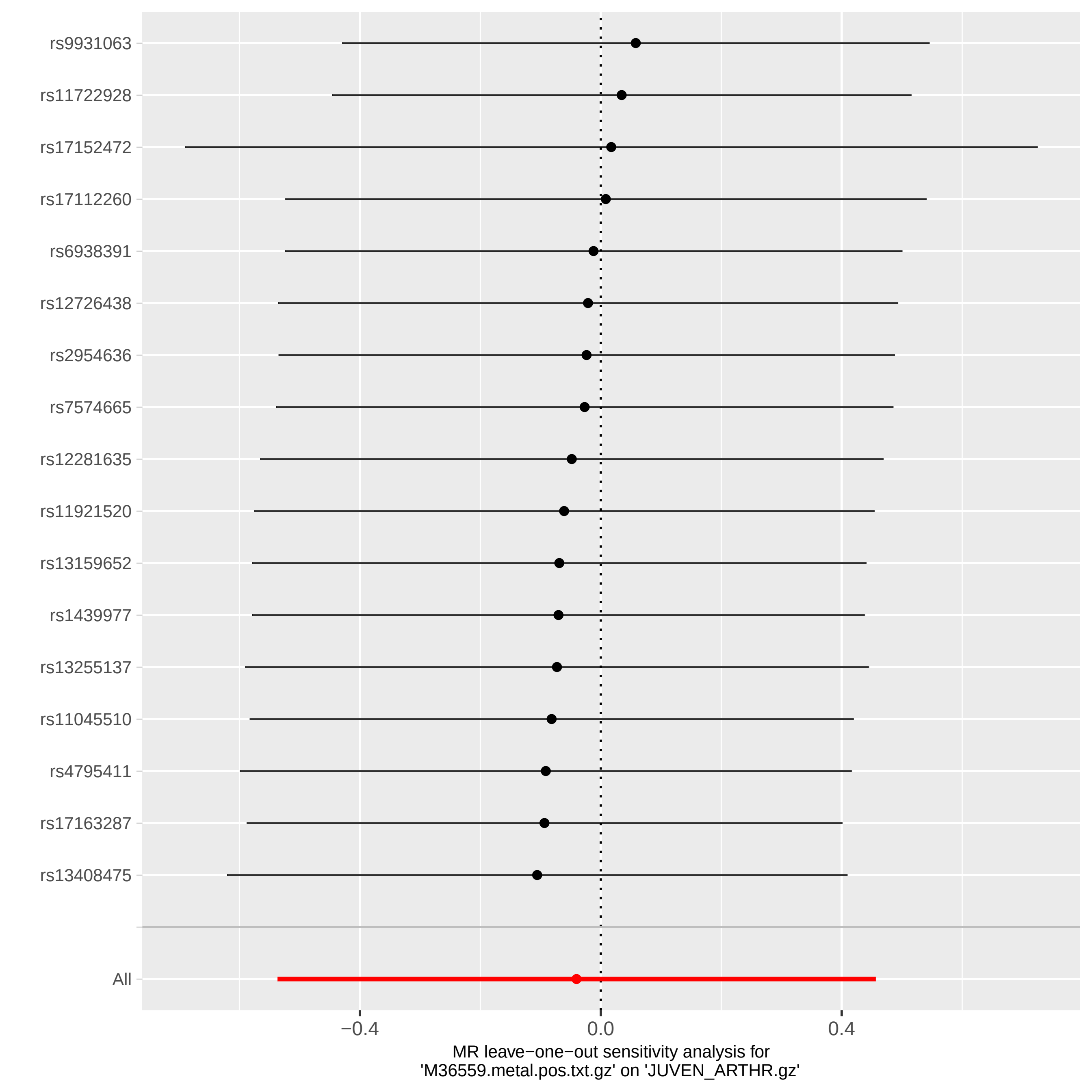
rs9931063
rs11722928
rs17152472
rs17112260
rs6938391
rs12726438
rs2954636
rs7574665
rs12281635
rs11921520
rs13159652
rs1439977
rs13255137
rs11045510
rs4795411
rs17163287
rs13408475
All
−0.4
0.0
0.4
MR leave−one−out sensitivity analysis for
'M36559.metal.pos.txt.gz' on 'JUVEN_ARTHR.gz'

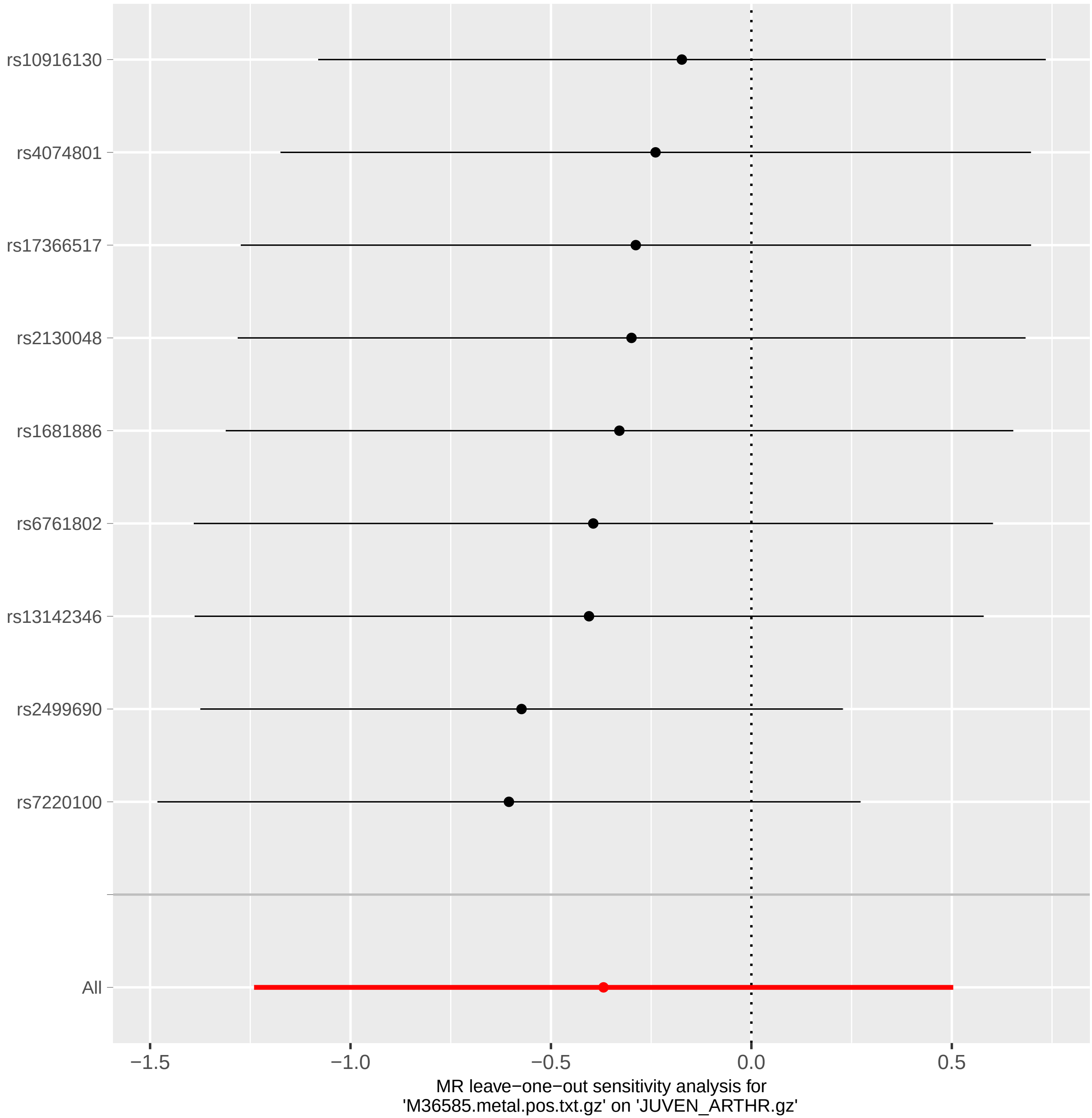
rs10916130
rs4074801
rs17366517
rs2130048
rs1681886
rs6761802
rs13142346
rs2499690
rs7220100
All
−1.5
−1.0
−0.5
0.0
0.5
MR leave−one−out sensitivity analysis for
'M36585.metal.pos.txt.gz' on 'JUVEN_ARTHR.gz'

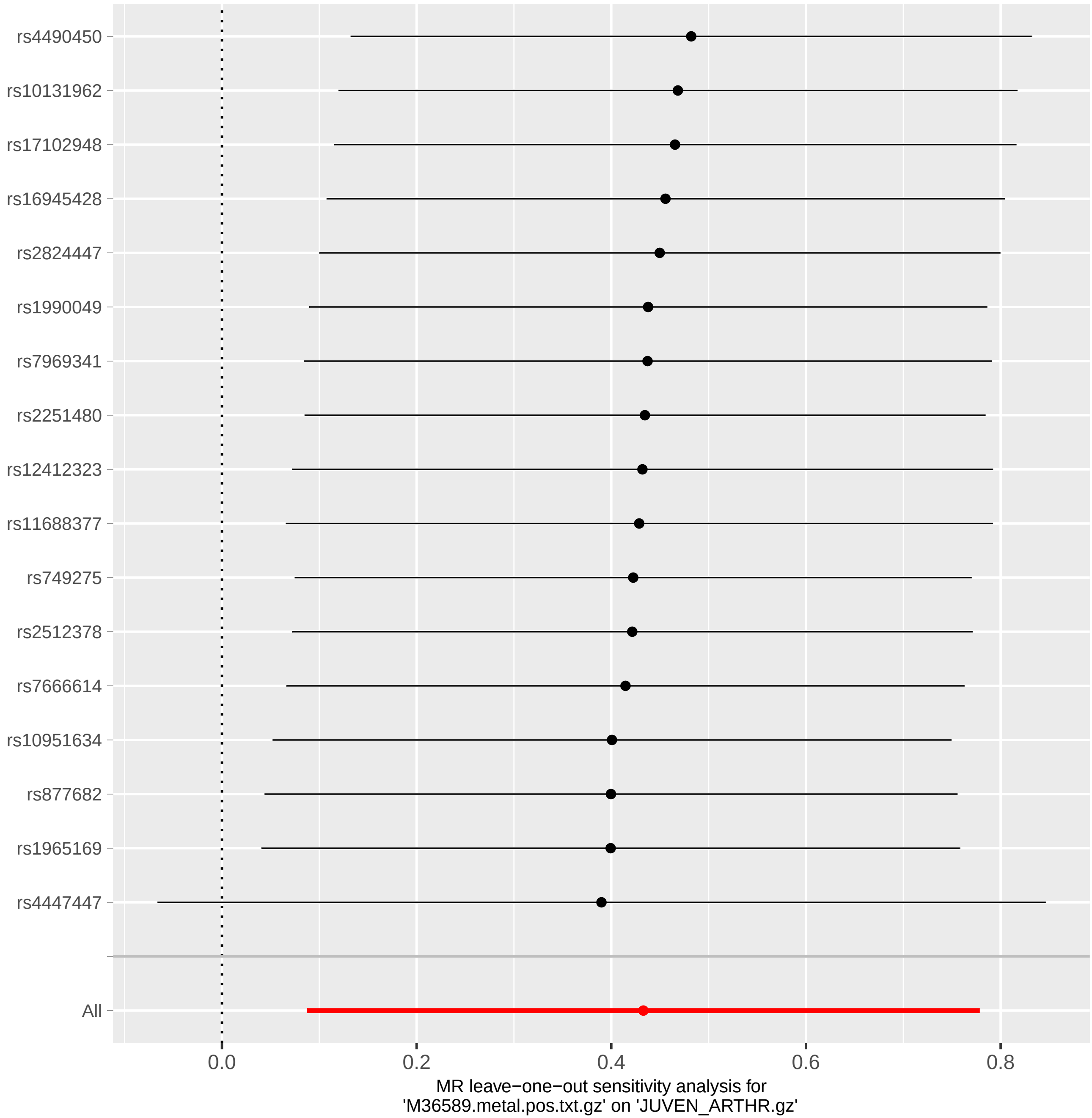

rs4490450
rs10131962
rs17102948
rs16945428
rs2824447
rs1990049
rs7969341
rs2251480
rs12412323
rs11688377
rs749275
rs2512378
rs7666614
rs10951634
rs877682
rs1965169
rs4447447
All
0.0
0.2
0.4
0.6
0.8
MR leave−one−out sensitivity analysis for
'M36589.metal.pos.txt.gz' on 'JUVEN_ARTHR.gz'

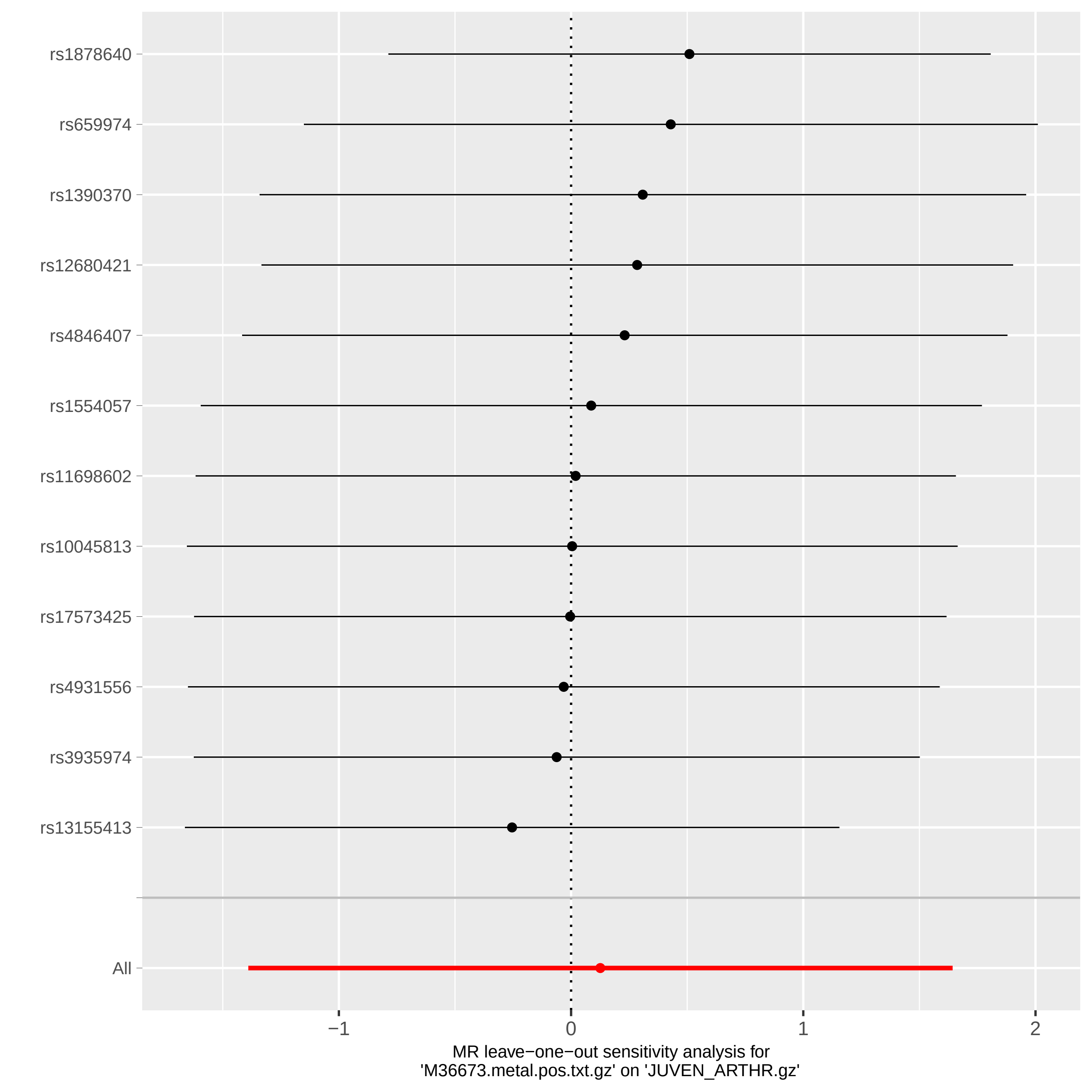
rs1878640
rs659974
rs1390370
rs12680421
rs4846407
rs1554057
rs11698602
rs10045813
rs17573425
rs4931556
rs3935974
rs13155413
All
−1
0
1
2
MR leave−one−out sensitivity analysis for
'M36673.metal.pos.txt.gz' on 'JUVEN_ARTHR.gz'

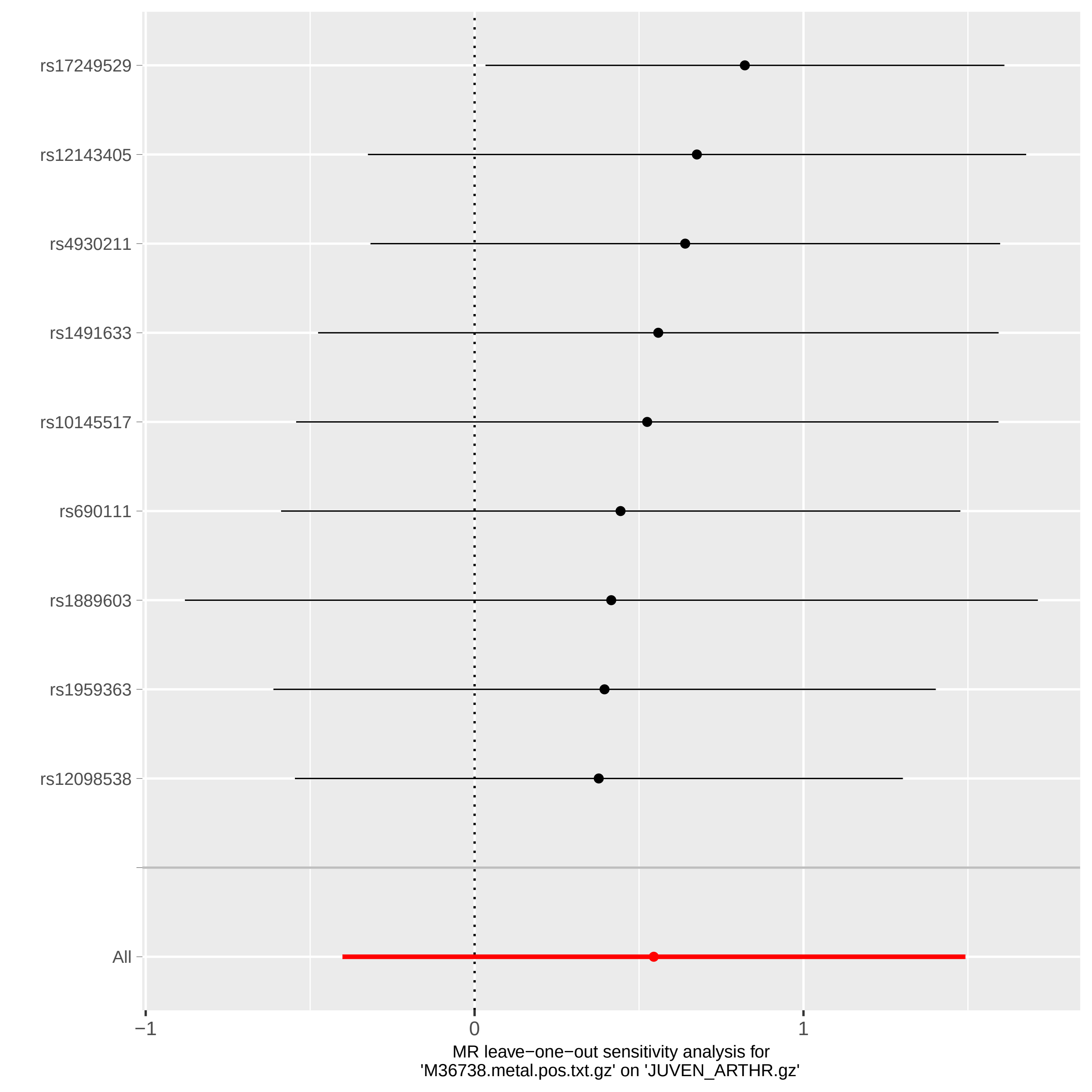

rs17249529
rs12143405
rs4930211
rs1491633
rs10145517
rs690111
rs1889603
rs1959363
rs12098538
All
−1
0
1
MR leave−one−out sensitivity analysis for
'M36738.metal.pos.txt.gz' on 'JUVEN_ARTHR.gz'

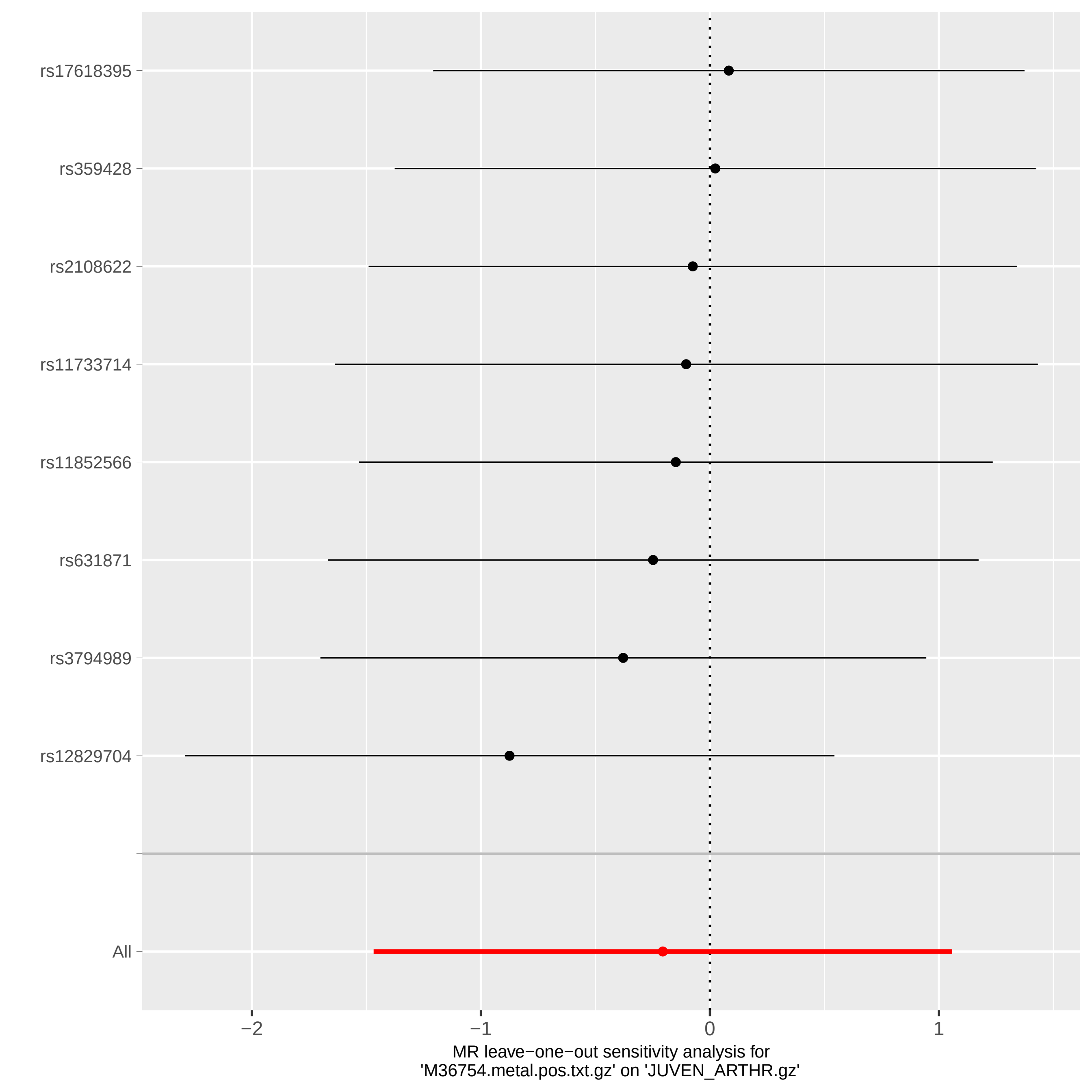

rs17618395
rs359428
rs2108622
rs11733714
rs11852566
rs631871
rs3794989
rs12829704
All
−2
−1
0
1
MR leave−one−out sensitivity analysis for
'M36754.metal.pos.txt.gz' on 'JUVEN_ARTHR.gz'

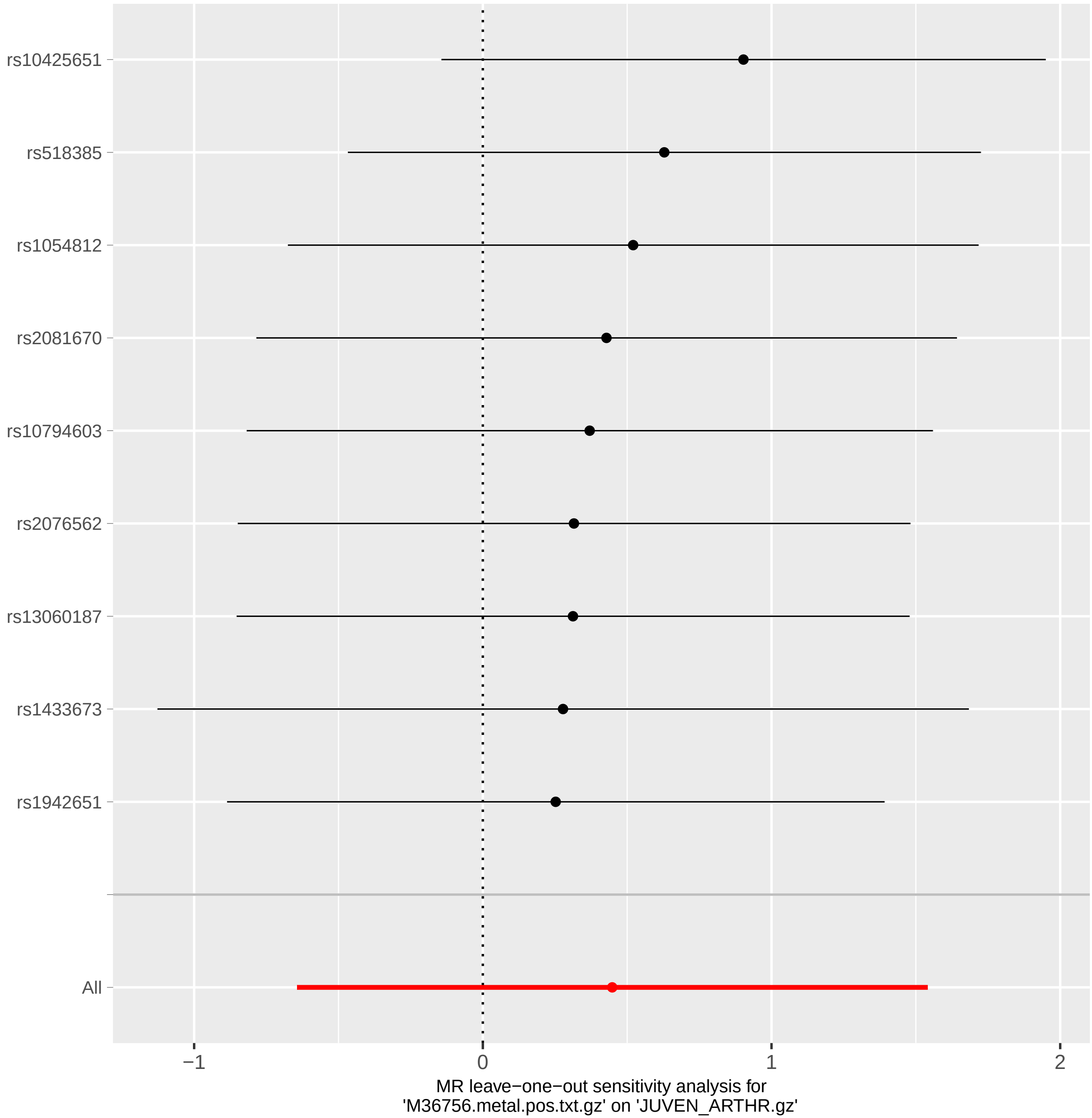
rs10425651
rs518385
rs1054812
rs2081670
rs10794603
rs2076562
rs13060187
rs1433673
rs1942651
All
−1
0
1
2
MR leave−one−out sensitivity analysis for
'M36756.metal.pos.txt.gz' on 'JUVEN_ARTHR.gz'

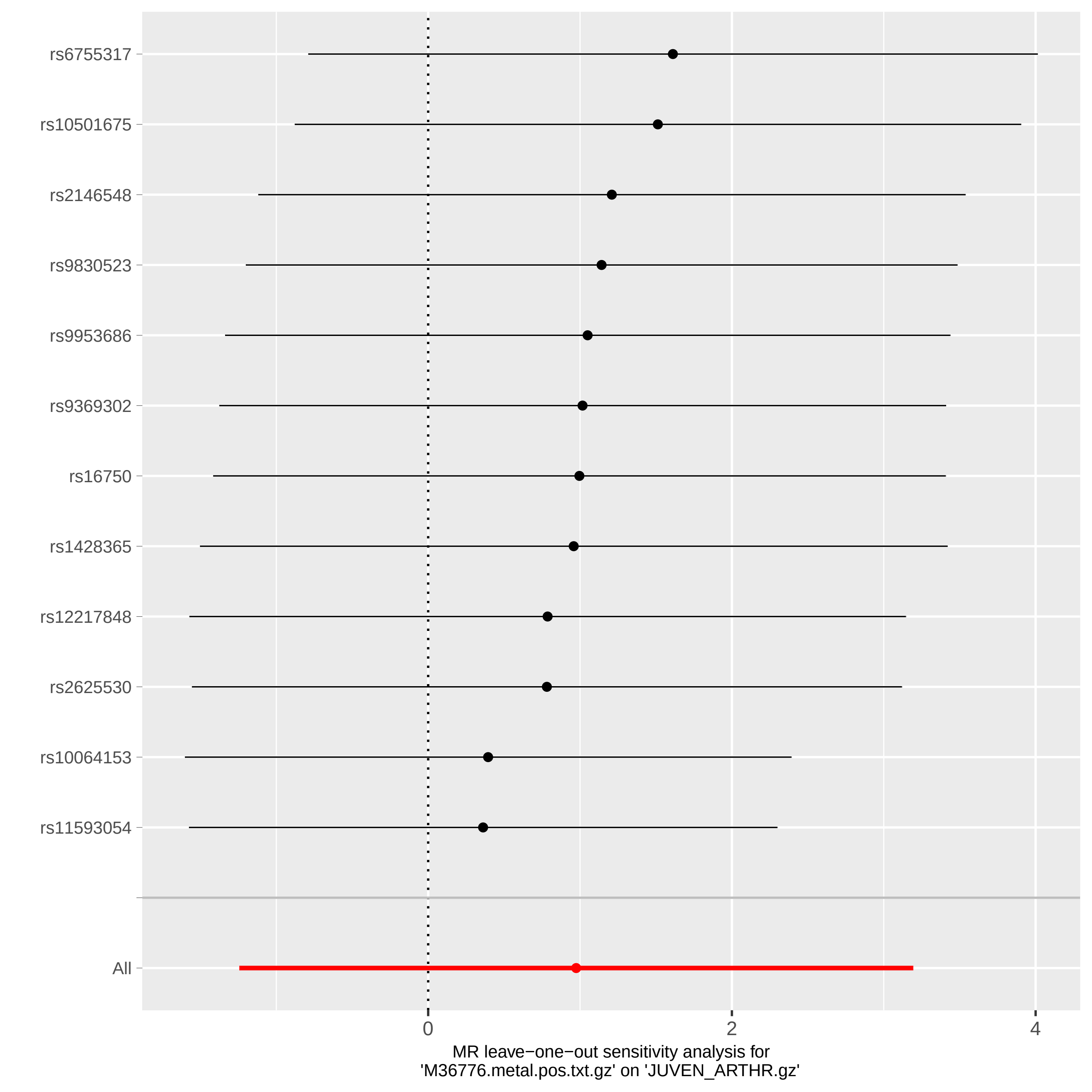
rs6755317
rs10501675
rs2146548
rs9830523
rs9953686
rs9369302
rs16750
rs1428365
rs12217848
rs2625530
rs10064153
rs11593054
All
0
2
4
MR leave−one−out sensitivity analysis for
'M36776.metal.pos.txt.gz' on 'JUVEN_ARTHR.gz'

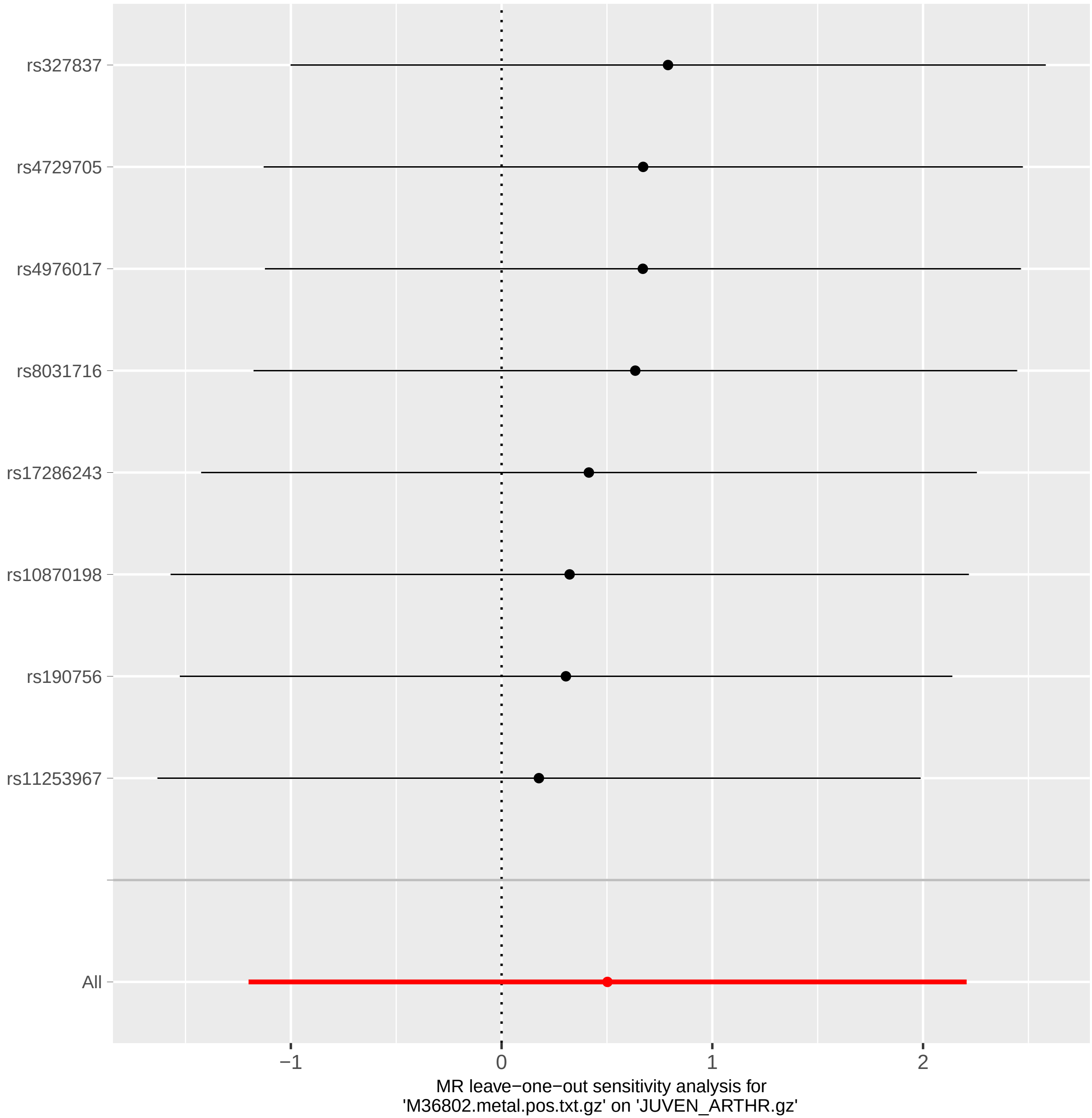
rs327837
rs4729705
rs4976017
rs8031716
rs17286243
rs10870198
rs190756
rs11253967
All
−1
0
1
2
MR leave−one−out sensitivity analysis for
'M36802.metal.pos.txt.gz' on 'JUVEN_ARTHR.gz'

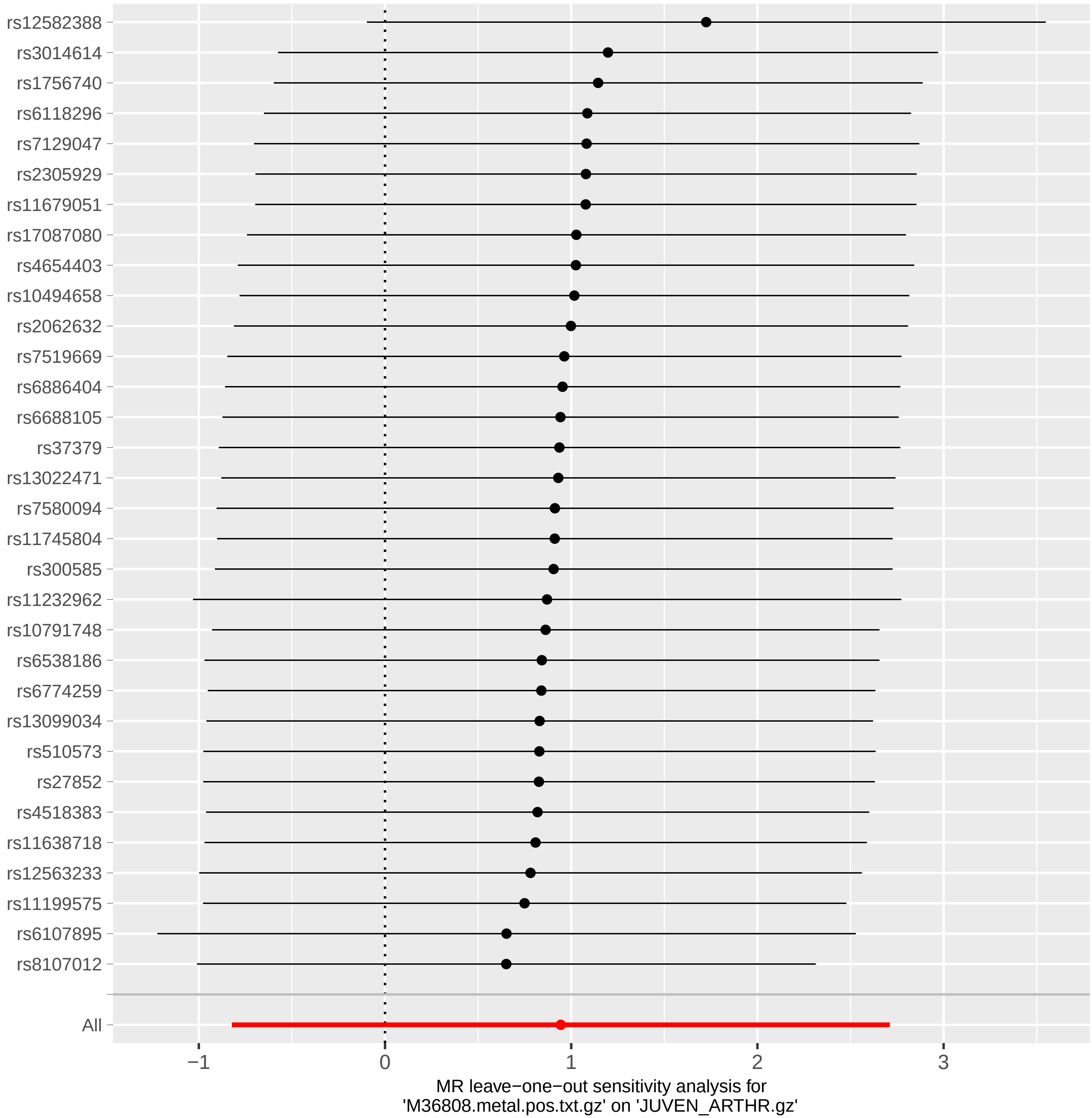
rs12582388
rs3014614
rs1756740
rs6118296
rs7129047
rs2305929
rs11679051
rs17087080
rs4654403
rs10494658
rs2062632
rs7519669
rs6886404
rs6688105
rs37379
rs13022471
rs7580094
rs11745804
rs300585
rs11232962
rs10791748
rs6538186
rs6774259
rs13099034
rs510573
rs27852
rs4518383
rs11638718
rs12563233
rs11199575
rs6107895
rs8107012
All
−1
0
1
2
3
MR leave−one−out sensitivity analysis for
'M36808.metal.pos.txt.gz' on 'JUVEN_ARTHR.gz'

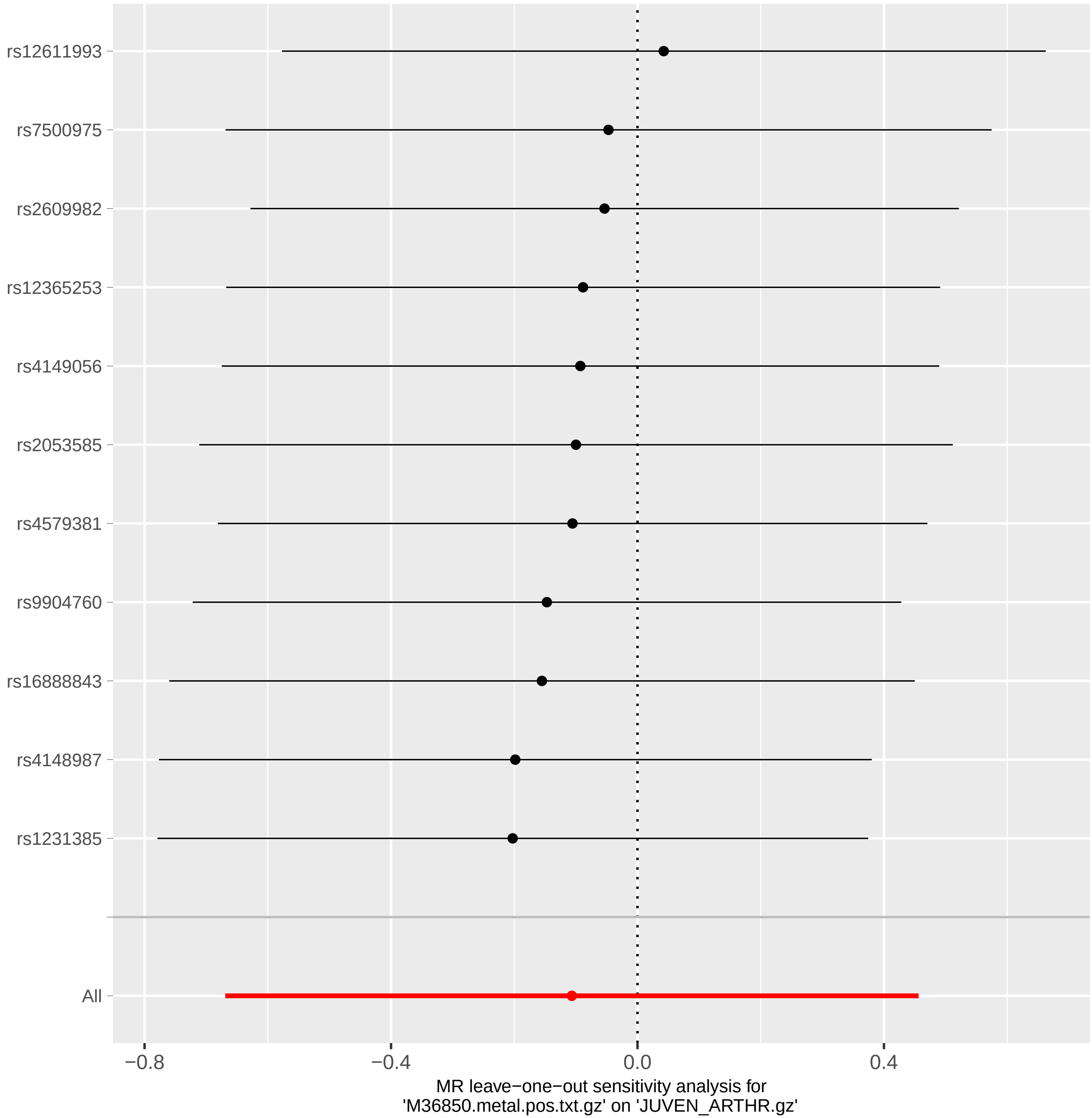
rs12611993
rs7500975
rs2609982
rs12365253
rs4149056
rs2053585
rs4579381
rs9904760
rs16888843
rs4148987
rs1231385
All
−0.8
−0.4
0.0
0.4
MR leave−one−out sensitivity analysis for
'M36850.metal.pos.txt.gz' on 'JUVEN_ARTHR.gz'

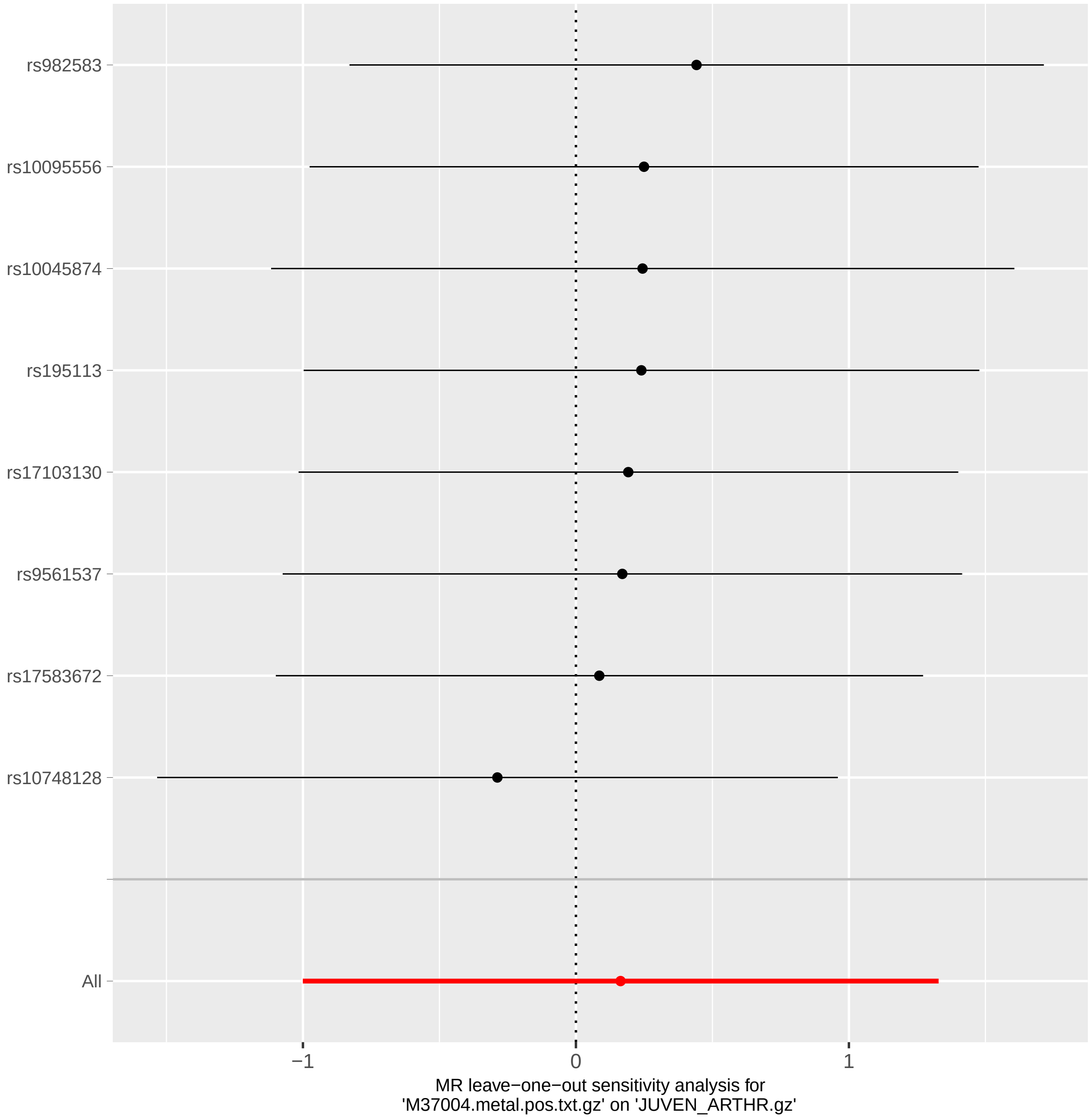

rs982583
rs10095556
rs10045874
rs195113
rs17103130
rs9561537
rs17583672
rs10748128
All
−1
0
1
MR leave−one−out sensitivity analysis for
'M37004.metal.pos.txt.gz' on 'JUVEN_ARTHR.gz'

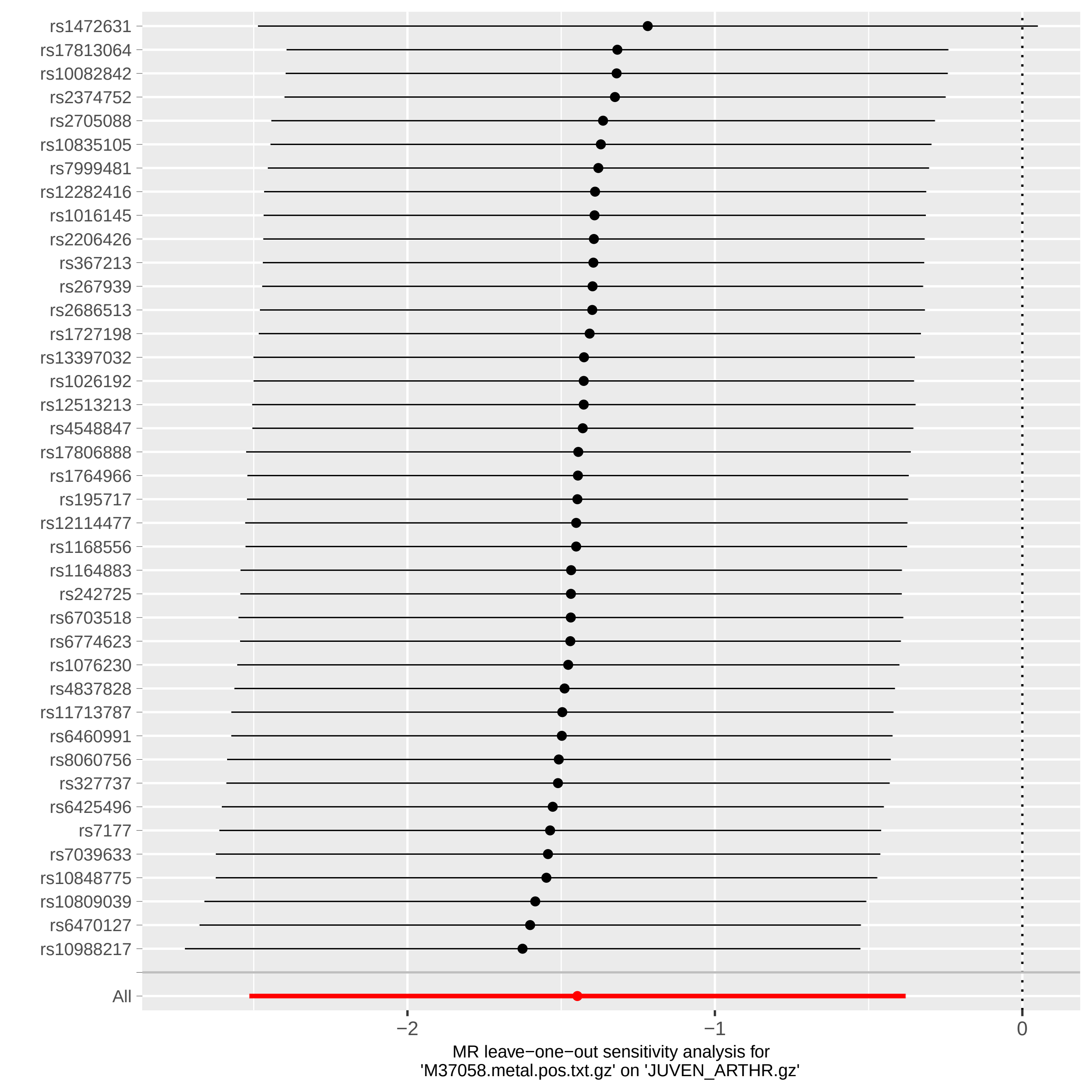

rs1472631
rs17813064
rs10082842
rs2374752
rs2705088
rs10835105
rs7999481
rs12282416
rs1016145
rs2206426
rs367213
rs267939
rs2686513
rs1727198
rs13397032
rs1026192
rs12513213
rs4548847
rs17806888
rs1764966
rs195717
rs12114477
rs1168556
rs1164883
rs242725
rs6703518
rs6774623
rs1076230
rs4837828
rs11713787
rs6460991
rs8060756
rs327737
rs6425496
rs7177
rs7039633
rs10848775
rs10809039
rs6470127
rs10988217
All
−2
−1
0
MR leave−one−out sensitivity analysis for
'M37058.metal.pos.txt.gz' on 'JUVEN_ARTHR.gz'

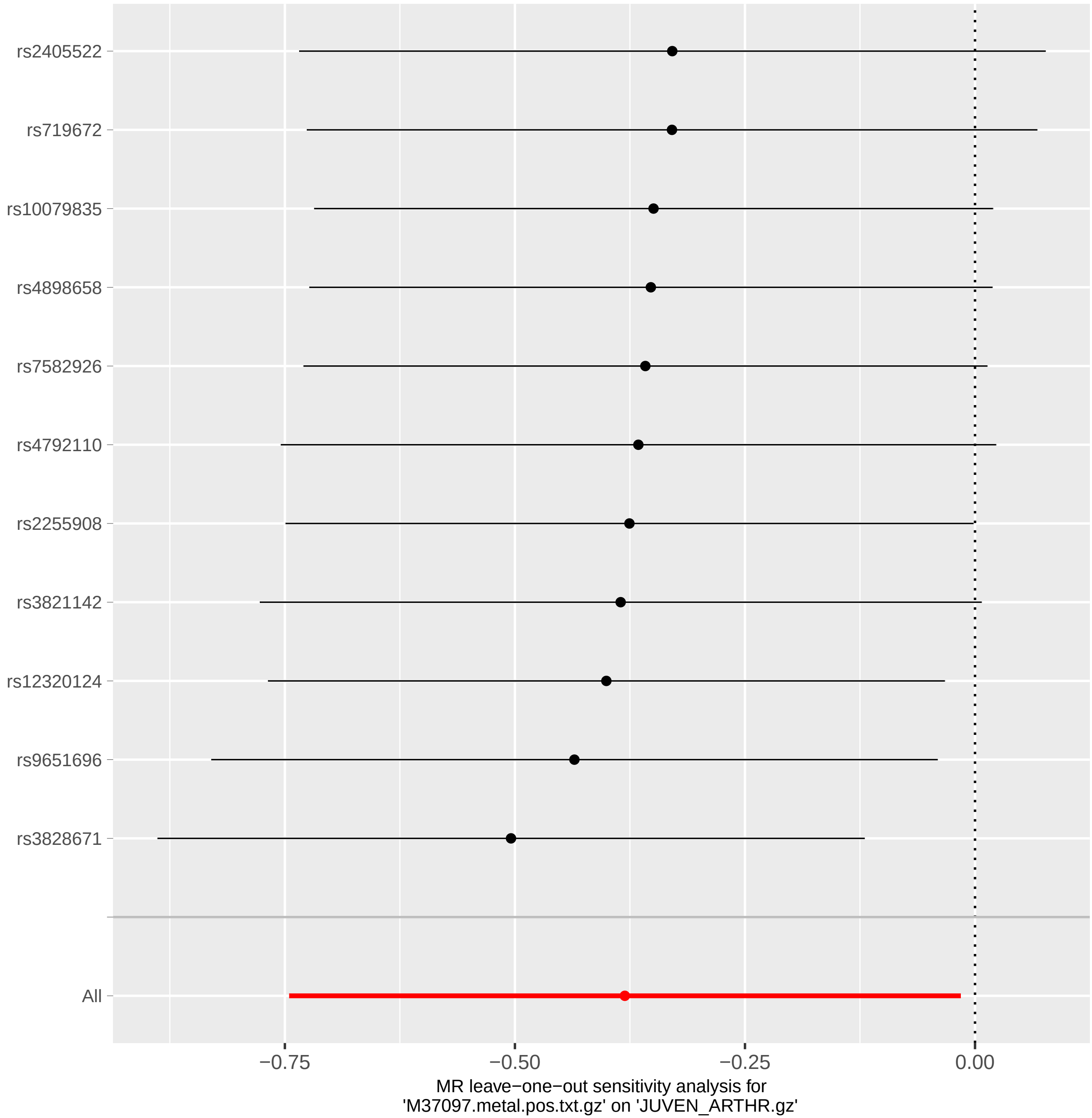

rs2405522
rs719672
rs10079835
rs4898658
rs7582926
rs4792110
rs2255908
rs3821142
rs12320124
rs9651696
rs3828671
All
−0.75
−0.50
−0.25
0.00
MR leave−one−out sensitivity analysis for
'M37097.metal.pos.txt.gz' on 'JUVEN_ARTHR.gz'

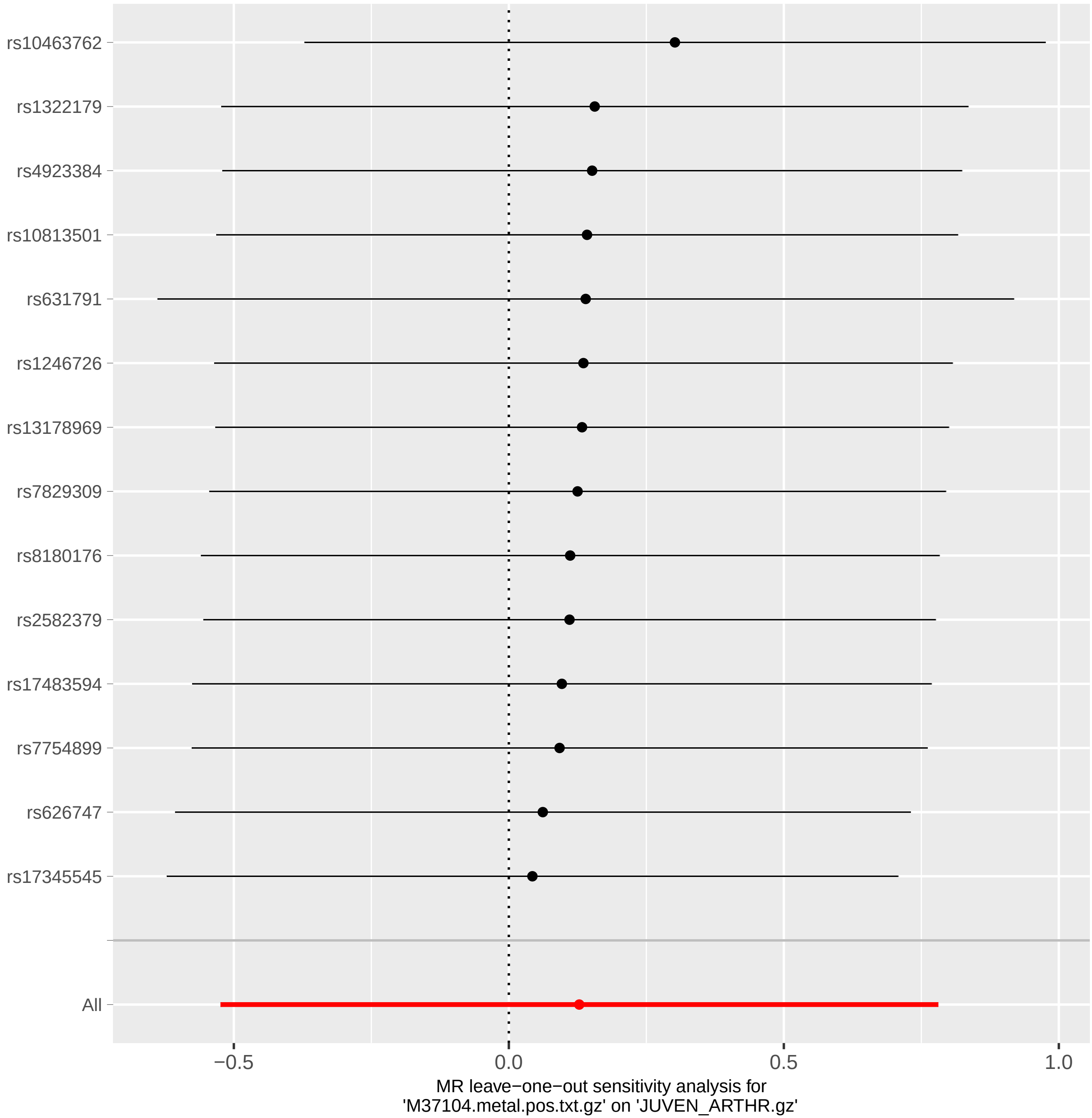
rs10463762
rs1322179
rs4923384
rs10813501
rs631791
rs1246726
rs13178969
rs7829309
rs8180176
rs2582379
rs17483594
rs7754899
rs626747
rs17345545
All
−0.5
0.0
0.5
1.0
MR leave−one−out sensitivity analysis for
'M37104.metal.pos.txt.gz' on 'JUVEN_ARTHR.gz'

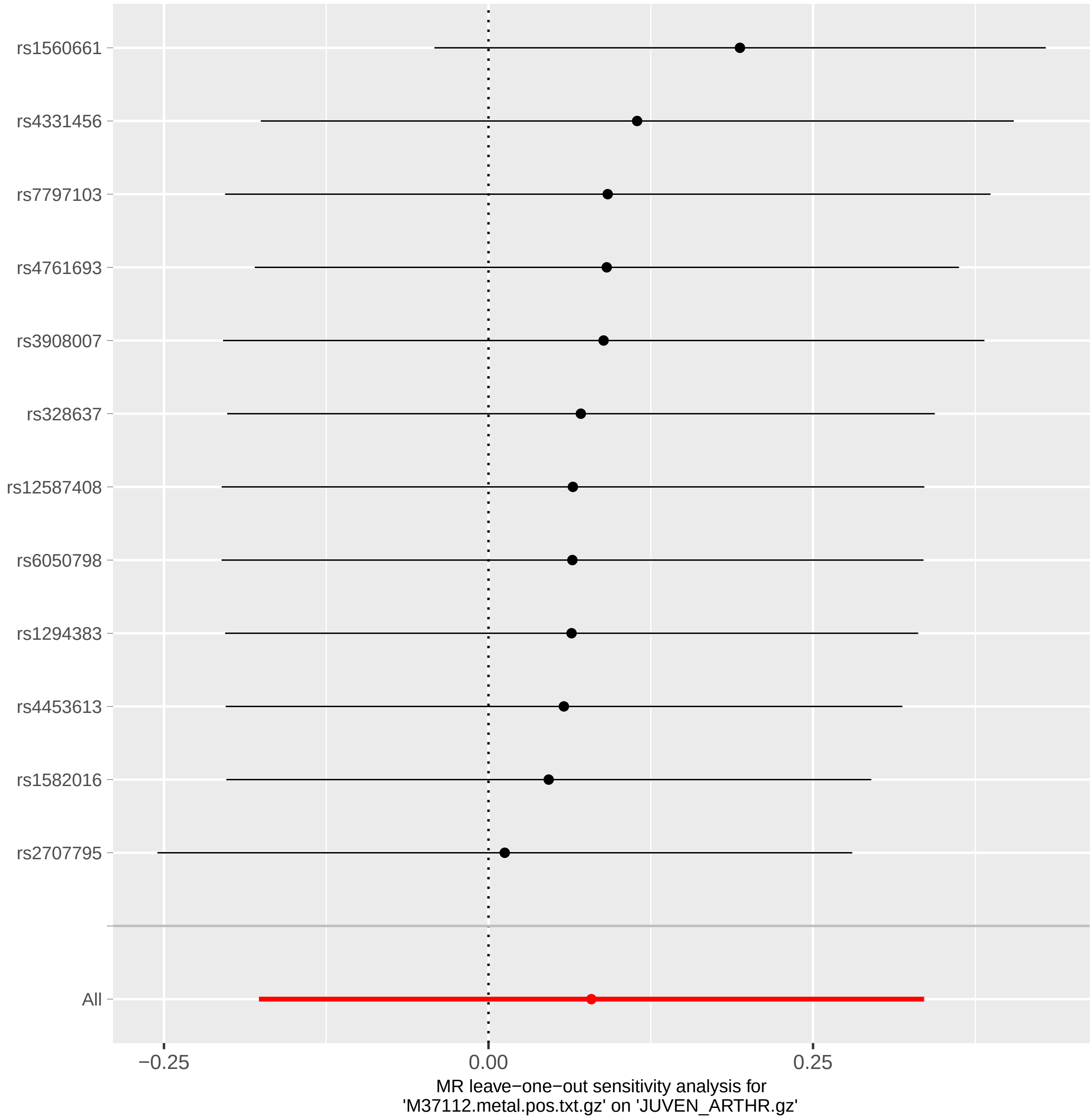

rs1560661
rs4331456
rs7797103
rs4761693
rs3908007
rs328637
rs12587408
rs6050798
rs1294383
rs4453613
rs1582016
rs2707795
All
−0.25
0.00
0.25
MR leave−one−out sensitivity analysis for
'M37112.metal.pos.txt.gz' on 'JUVEN_ARTHR.gz'

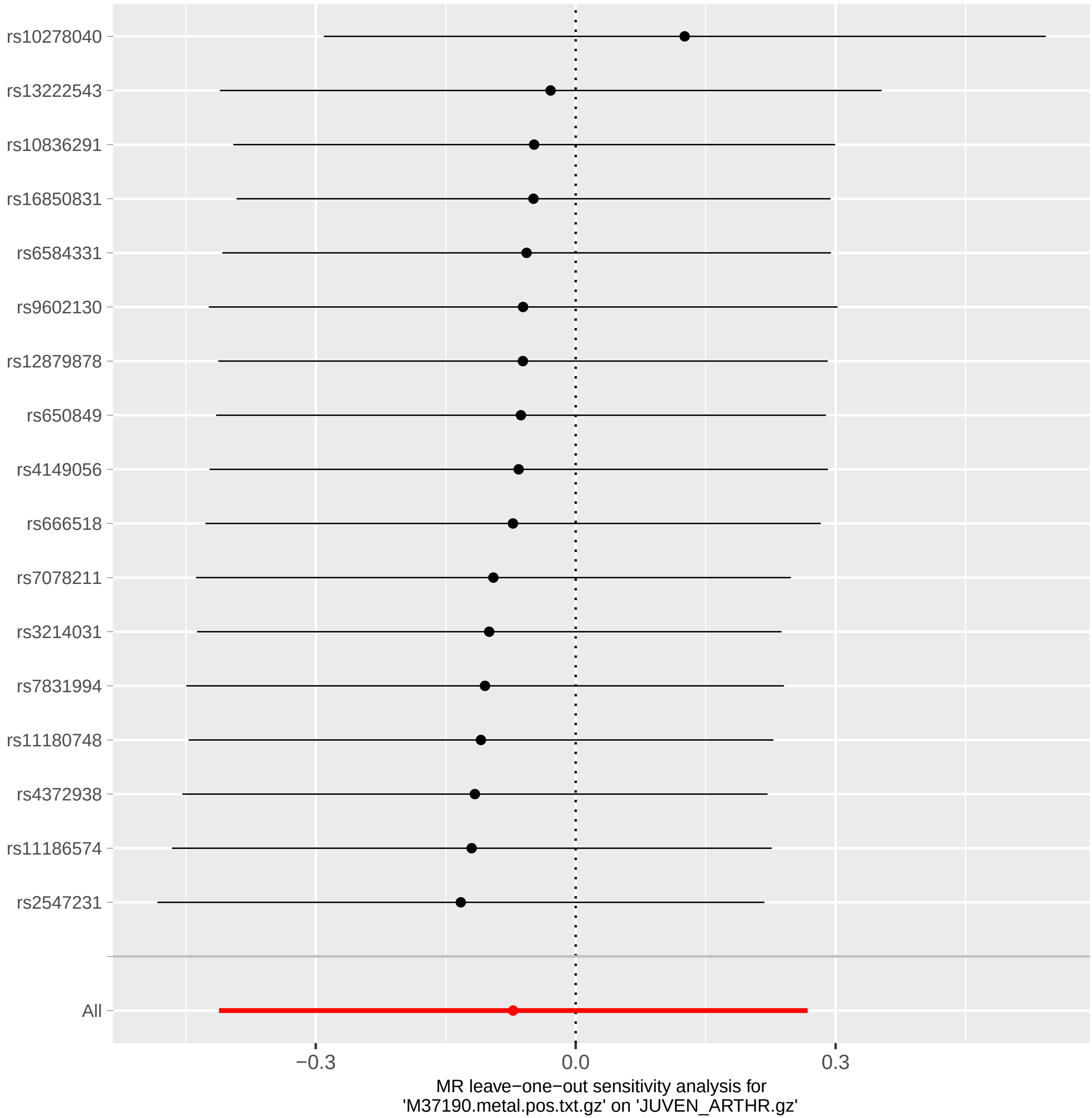

rs10278040
rs13222543
rs10836291
rs16850831
rs6584331
rs9602130
rs12879878
rs650849
rs4149056
rs666518
rs7078211
rs3214031
rs7831994
rs11180748
rs4372938
rs11186574
rs2547231
All
−0.3
0.0
0.3
MR leave−one−out sensitivity analysis for
'M37190.metal.pos.txt.gz' on 'JUVEN_ARTHR.gz'

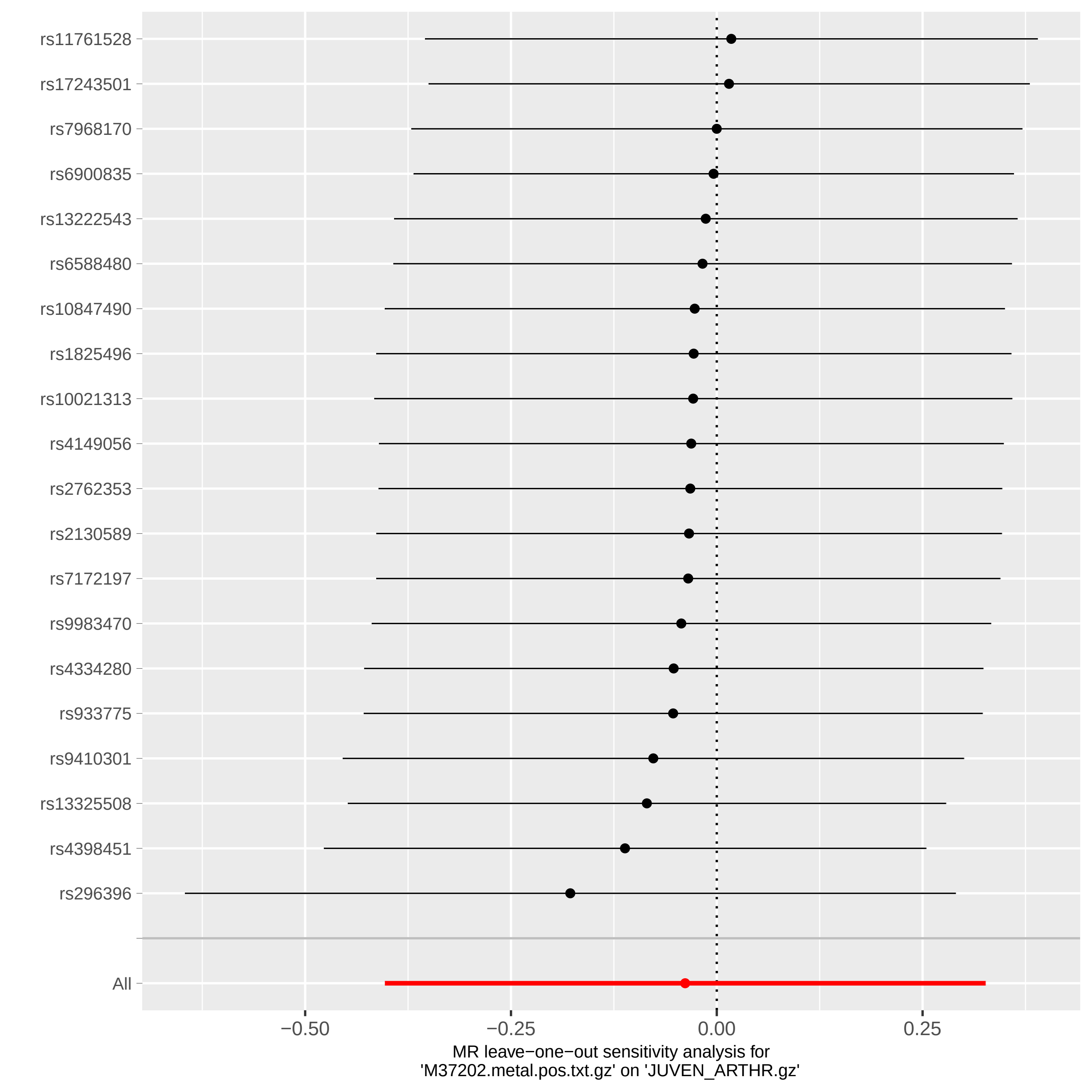
rs11761528
rs17243501
rs7968170
rs6900835
rs13222543
rs6588480
rs10847490
rs1825496
rs10021313
rs4149056
rs2762353
rs2130589
rs7172197
rs9983470
rs4334280
rs933775
rs9410301
rs13325508
rs4398451
rs296396
All
−0.50
−0.25
0.00
0.25
MR leave−one−out sensitivity analysis for
'M37202.metal.pos.txt.gz' on 'JUVEN_ARTHR.gz'

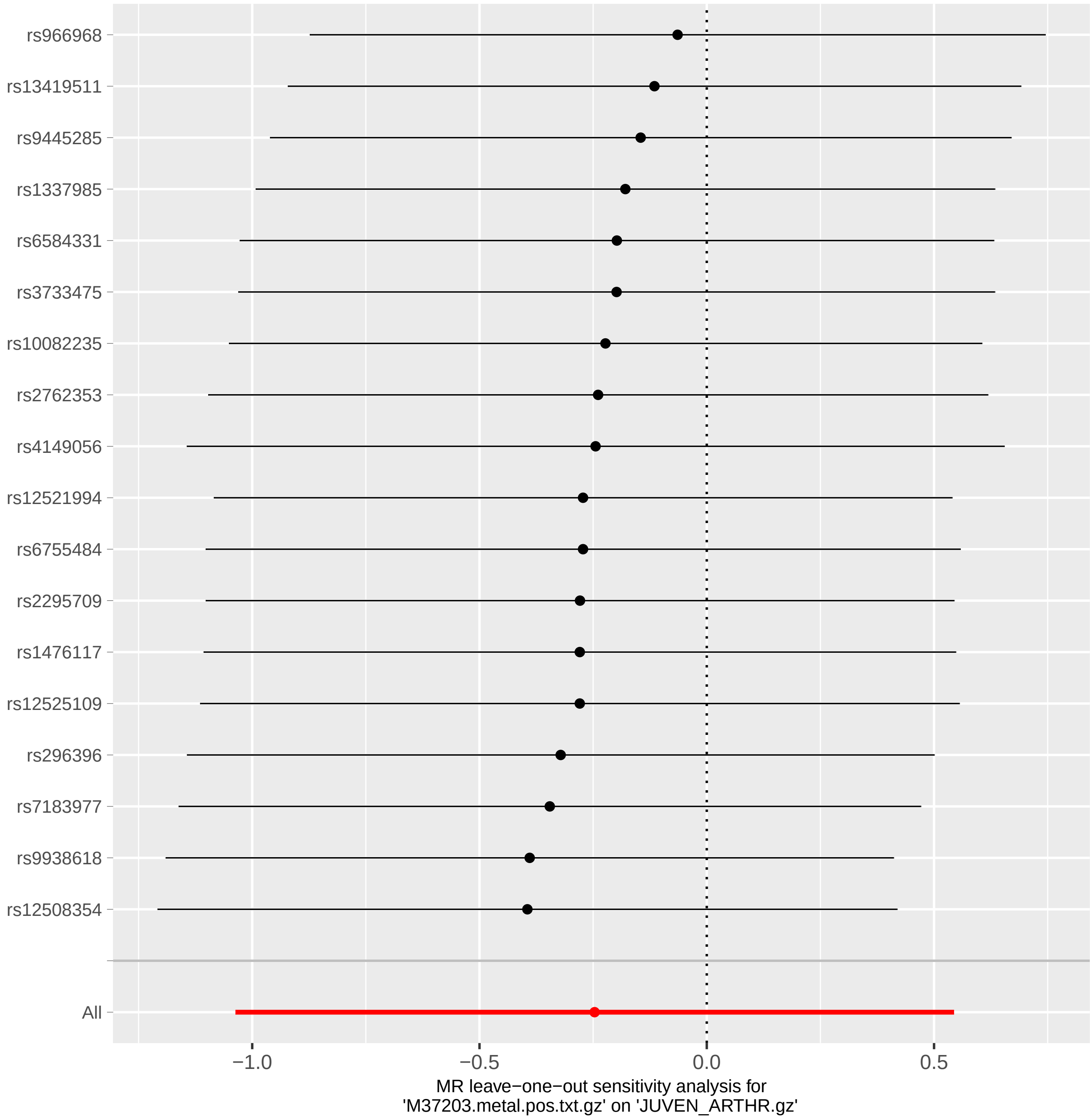
rs966968
rs13419511
rs9445285
rs1337985
rs6584331
rs3733475
rs10082235
rs2762353
rs4149056
rs12521994
rs6755484
rs2295709
rs1476117
rs12525109
rs296396
rs7183977
rs9938618
rs12508354
All
−1.0
−0.5
0.0
0.5
MR leave−one−out sensitivity analysis for
'M37203.metal.pos.txt.gz' on 'JUVEN_ARTHR.gz'

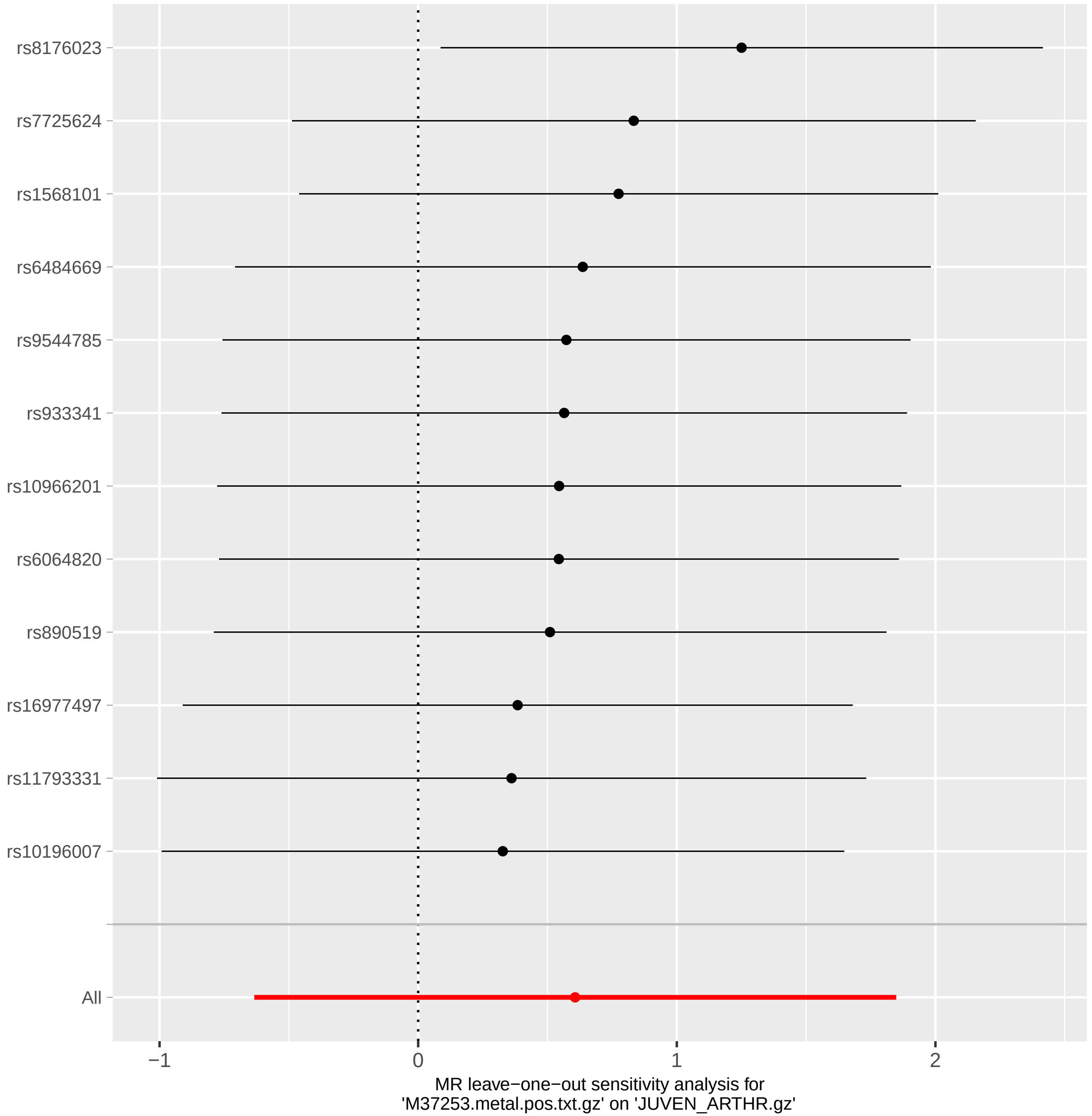
rs8176023
rs7725624
rs1568101
rs6484669
rs9544785
rs933341
rs10966201
rs6064820
rs890519
rs16977497
rs11793331
rs10196007
All
−1
0
1
2
MR leave−one−out sensitivity analysis for
'M37253.metal.pos.txt.gz' on 'JUVEN_ARTHR.gz'

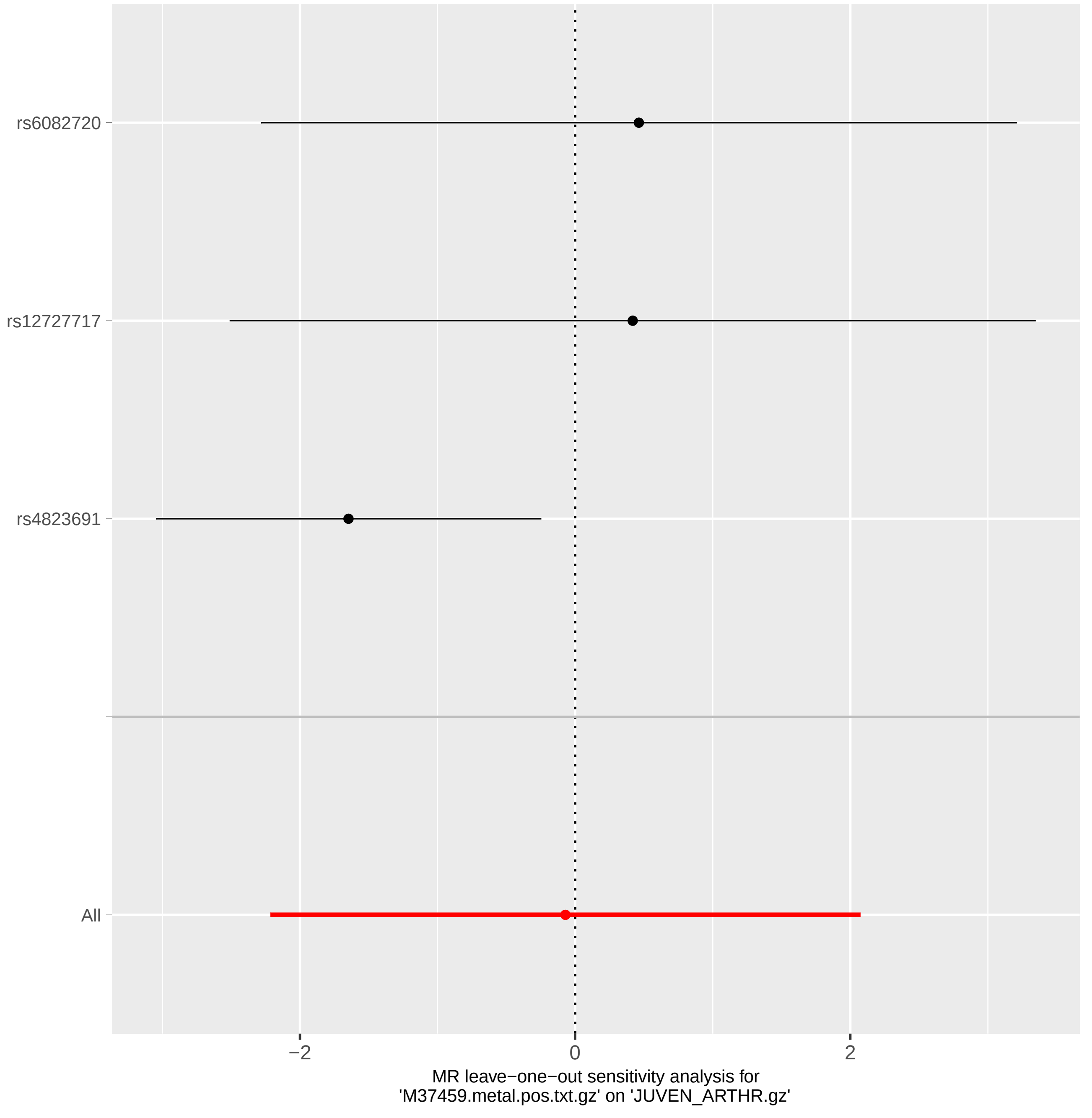
rs6082720
rs12727717
rs4823691
All
−2
0
2
MR leave−one−out sensitivity analysis for
'M37459.metal.pos.txt.gz' on 'JUVEN_ARTHR.gz'

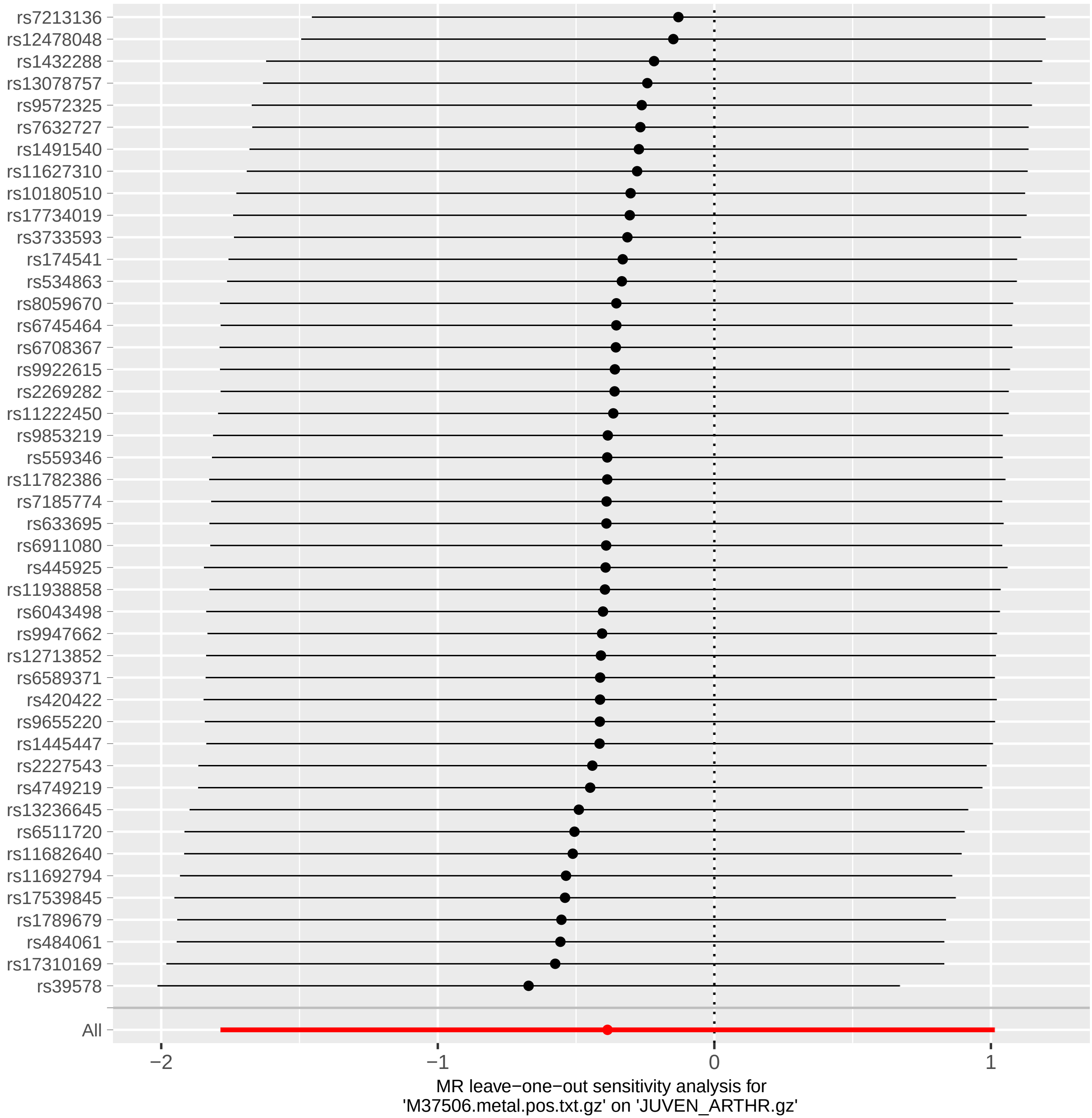
rs7213136
rs12478048
rs1432288
rs13078757
rs9572325
rs7632727
rs1491540
rs11627310
rs10180510
rs17734019
rs3733593
rs174541
rs534863
rs8059670
rs6745464
rs6708367
rs9922615
rs2269282
rs11222450
rs9853219
rs559346
rs11782386
rs7185774
rs633695
rs6911080
rs445925
rs11938858
rs6043498
rs9947662
rs12713852
rs6589371
rs420422
rs9655220
rs1445447
rs2227543
rs4749219
rs13236645
rs6511720
rs11682640
rs11692794
rs17539845
rs1789679
rs484061
rs17310169
rs39578
All
−2
−1
0
1
MR leave−one−out sensitivity analysis for
'M37506.metal.pos.txt.gz' on 'JUVEN_ARTHR.gz'

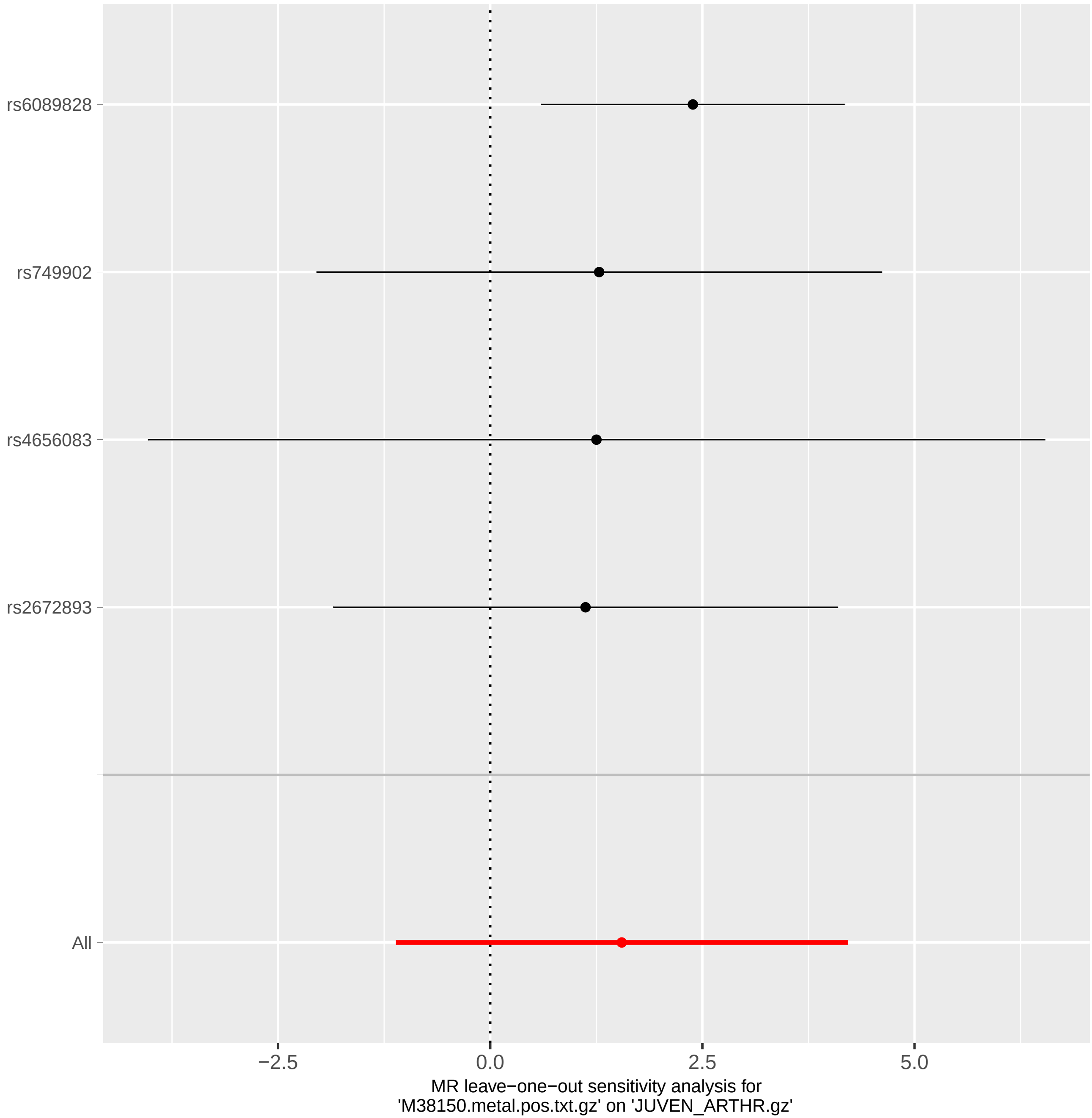
rs6089828
rs749902
rs4656083
rs2672893
All
−2.5
0.0
2.5
5.0
MR leave−one−out sensitivity analysis for
'M38150.metal.pos.txt.gz' on 'JUVEN_ARTHR.gz'

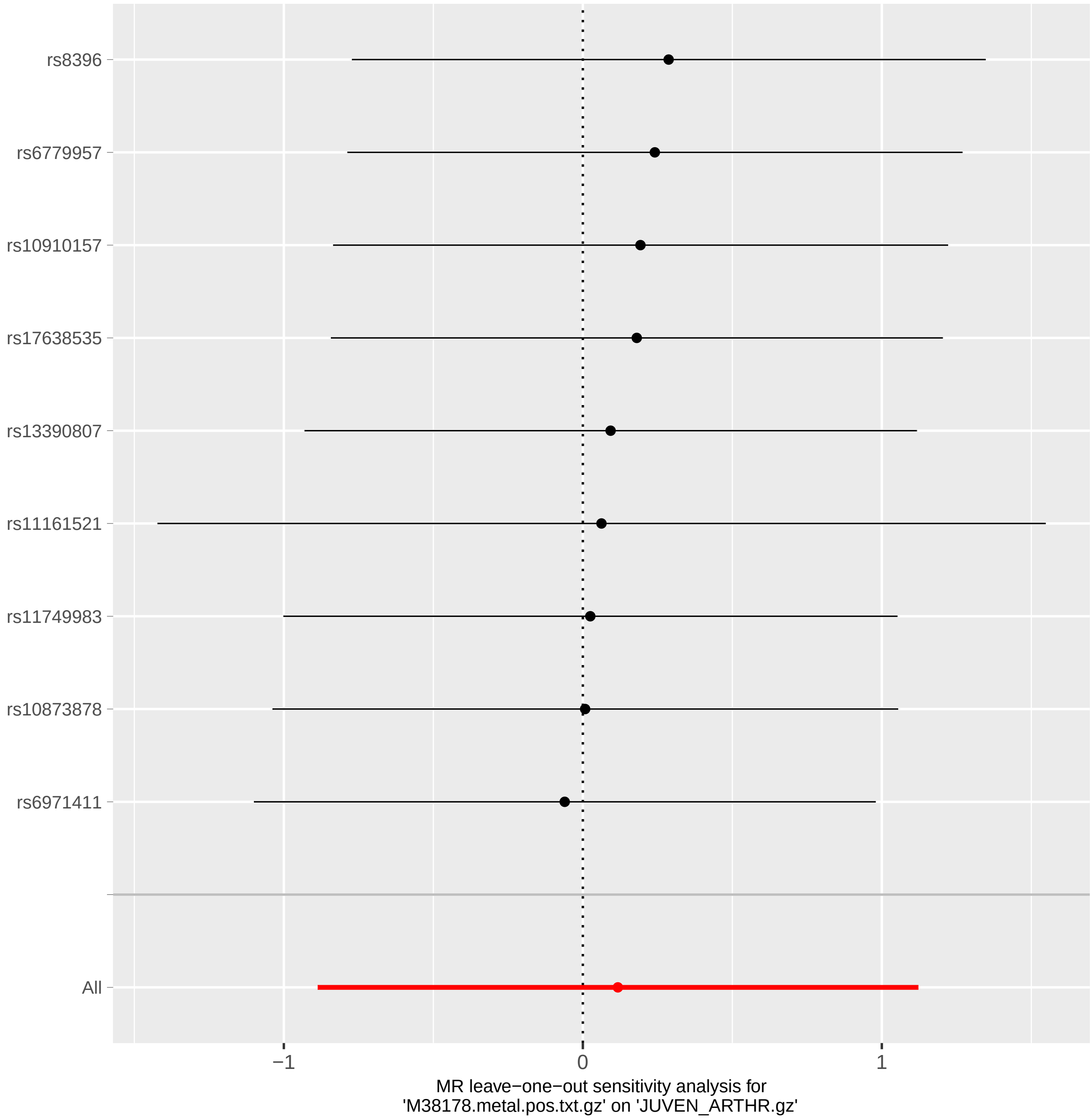
rs8396
rs6779957
rs10910157
rs17638535
rs13390807
rs11161521
rs11749983
rs10873878
rs6971411
All
−1
0
1
MR leave−one−out sensitivity analysis for
'M38178.metal.pos.txt.gz' on 'JUVEN_ARTHR.gz'

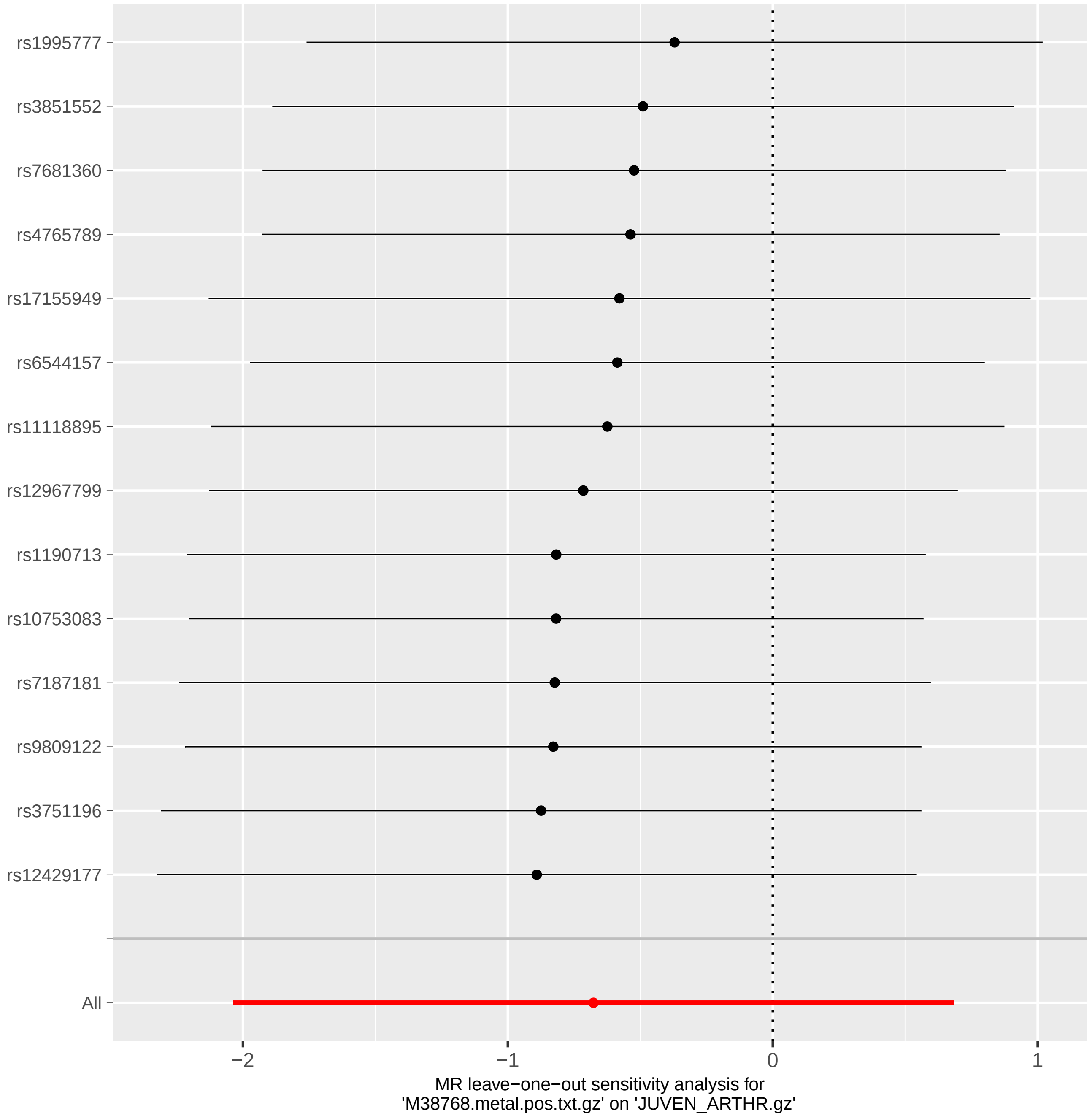
rs1995777
rs3851552
rs7681360
rs4765789
rs17155949
rs6544157
rs11118895
rs12967799
rs1190713
rs10753083
rs7187181
rs9809122
rs3751196
rs12429177
All
−2
−1
0
1
MR leave−one−out sensitivity analysis for
'M38768.metal.pos.txt.gz' on 'JUVEN_ARTHR.gz'
