# Supplementary material for: Causal relationship between serum metabolites and juvenile idiopathic arthritis: a mendelian randomization study
Source: Pediatr Rheumatol Online J. 2024 May 9;22:51. doi: 10.1186/s12969-024-00986-0 (PMC11080266; doi:10.1186/s12969-024-00986-0)
Supplement: Supplementary file 11 — Supplementary Material 11 [file 12969_2024_986_MOESM11_ESM.docx]

December 12, 2023.

Dear

Editor-in-Chief

*Pediatric Rheumatology*

Dear Editor,

We are pleased to submit an *Original Article* titled “**Causal Relationship between Serum Metabolites and Juvenile Idiopathic Arthritis: A Mendelian Randomization Study**” for publication in *Pediatric Rheumatology*.

Juvenile idiopathic arthritis (JIA) is characterized by arthritis of unknown etiology lasting more than 6 weeks with onset before the age of 16 years. Although several inflammatory, immune, and metabolic factors are associated with the pathophysiology of JIA, little is known about the genetic relationship of serum metabolites with JIA. This study assessed the causal association of 486 serum metabolites with JIA using genome-wide association study and genetic variation data. We found that five serum metabolites were causally associated with JIA, including two risk factors (kynurenine and linolenate) and three protective factors (3-dehydrocarnitine, levulinate, and X-14208 [phenylalanylserine]). Moreover, seven metabolic pathways may be associated with JIA, including pantothenate and CoA biosynthesis, aminoacyl-tRNA biosynthesis, amino acid (phenylalanine, tyrosine, and tryptophan) biosynthesis, thiamine metabolism, taurine and hypotaurine metabolism, phenylalanine metabolism, and α-linolenic acid metabolism.

We believe that this study contributes significantly to the literature by identifying risk and protective factors that can help improve screening and identify high-risk groups. Moreover, we believe that this article will be of considerable interest to the readership of your journal because these results provide a theoretical framework for developing effective JIA prevention and screening strategies..

All authors approved the final version of the manuscript and agreed to submit it to *Pediatric Rheumatology*. Further, the authors declare that there are no conflicts of interest.

This article has not been published elsewhere and is not under review by another journal. The content was carefully revised by a native English-speaking editor specialized in editing manuscripts written by non-native English-speaking scientists.

We look forward to hearing from you at your earliest convenience.

Sincerely,

Author Han Zhang

Affiliation Department of Orthopedics, Affiliated Hospital of Qingdao University, Qingdao, China

Institutional Address No. 59, Haier Road, Laoshan District, Qingdao City.

Phone: +86 18253069516

Fax: -

E-mail: zhanghan19930222@163.com
